# Supplementary material for: Effect of glycemic control and type of diabetes treatment on TB treatment outcomes among people with TB-diabetes: A systematic review (updated August 2024)
Source: PLoS One. 2025 Jul 18;20(7):e0328619. doi: 10.1371/journal.pone.0328619 (PMC12273911; doi:10.1371/journal.pone.0328619)
Supplement: S1 Annex — (DOCX) [file pone.0328619.s001.docx]

**S1 Annex. Characteristics of the studies excluded from the systematic review conducted between 1996 to August 2024*^**

| **S.No** | **Study identifier** | **T** | **P** | **E/C** | **O** |
| --- | --- | --- | --- | --- | --- |
| **1996 to 25 April 2017** | | | | | |
| 1 | Hongguage C_2015_Epidemiol Infect | Y | Y | N | Y |
| 2 | Wang CS_2009_Epidemiol Infect | Y | Y | N | Y |
| 3 | Banurekha VV_2007_IJMR | Y | Y | N | Y |
| 4 | Chang JT_2011_J Formos Med Assoc | Y | Y | N | Y |
| 5 | Kota SK_2011_Diabetes and Metabolic Syndrome Clinical Research and Reviews | Y | Y | N | Y |
| 6 | Duangrithi D_2013_International J of Clinical Practice | Y | Y | N | Y |
| 7 | Fielder JF_2002_Int J of Tub Lung Dis | Y | Y | N | Y |
| 8 | Jabbar A_2006_Eastern Mediterranean Health Journal | Y | Y | N | Y |
| 9 | Perez-Navarro LM_2015_J of Diabetes and its Complications | Y | Y | N | Y |
| 10 | Iseri AU_2010_Tuberkuloz ve ToraksDergisi | Y | Y | N | Y |
| 11 | B.E. Abdelbary_2016_Tuberculosis | Y | Y | N | Y |
| 12 | Bachti Alisjahbana_2007_Clinical Infectious Diseases | Y | Y | N | Y |
| 13 | Rani Balasubramanian_2007_Indian J of TB | Y | Y | N | Y |
| 14 | Boillant_Blanco N_2016_The J of Infectious Diseases | Y | Y | N | Y |
| 15 | Dobler CC_2012_BMJ Open | Y | Y | N | N |
| 16 | Castellanos-Joya M_2014_Plos One | Y | Y | N | Y |
| 17 | Chaudhry LA_2012_International J of Mycobacteriology | Y | Y | N | Y |
| 18 | Faurholt-Jepsen D_2012_BMC Infectious Disease | Y | Y | N | Y |
| 19 | Faurholt-Jepsen D_2013_TMIH | Y | Y | N | Y |
| 20 | Suwampimolkul G_2014_Plos One | Y | Y | N | Y |
| 21 | Gnanasan S_2012_University of Nottighamphd thesis | Y | Y | N | N |
| 22 | Johnson HD_2016_American Journal of Infectious Disease and Microbiology | Y | Y | N | Y |
| 23 | Dooley KE_2009_Am J Trop Med Hyg | Y | Y | N | Y |
| 24 | Wang JY_2015_Chest | Y | Y | N | Y |
| 25 | Lee PH_2016_Plos Medicine | Y | Y | Y | N |
| 26 | Lo HY_2016_Int J of Tub Lung Dis | Y | Y | N | Y |
| 27 | Wang JY_2013_Pharmacoepidemiology and drug safety | Y | Y | N | N |
| 28 | Workneh MH_2016_Infectious Disease of Poverty | Y | Y | N | Y |
| 29 | Oceguera DM_2016_Lung Disease and Treatment | Y | Y | N | Y |
| 30 | Orofino RDL_2012_J Bras Pneumol | Y | Y | N | Y |
| 31 | Pajankar S_2008_Oman Medical Journal | Y | Y | N | Y |
| 32 | Sahakyan S_2015_not a peer-reviewed publication | Y | Y | N | Y |
| 33 | Jimenex-Corona ME_2013_Thorax | Y | Y | N | Y |
| 34 | Shariff NM_2015_Int J of mycobacteriology | N | Y | N | Y |
| 35 | Sulaiman SAS_2013_American J of medical science | Y | Y | N | Y |
| 36 | Vellalacheruvu BN_2015_International J of Scientific and Research Publications | Y | Y | N | Y |
| 37 | Wang JY_2009_Respirology | Y | Y | N | Y |
| 38 | Yusupova S_2016_Public Health Panorama | Y | Y | N | Y |
| 39 | Siddiqui AM_2009_Journal of Taibah University Medical Sciences | Y | Y | N | Y |
| 40 | Kornfield H_2016_Chest | Y | Y | N | Y |
| 41 | Gil_Santana L_2016_Plos One | Y | Y | N | Y |
| 42 | Mukhtar F_2016_BMJ Open | Y | Y | N | Y |
| 43 | Salindri AD_2016_Open Forum Infectious Diseases | Y | Y | N | Y |
| 44 | Barss L_2016_Chest | Y | Y | N | Y |
| 45 | Wu Z_2016_J of Diabetes and its complications | Y | Y | N | Y |
| 46 | Lee EH_2017_Lung | Y | Y | N | Y |
| 47 | Perez- Navarro LM_2017_Tuberculosis | Y | Y | N | Y |
| **26 April 2017 to 31 August 2024** | | | | | |
| *48* | *Dairra_2019_JCTOMD* | *Y* | *N* | *N* | *Y* |
| *49* | *Huang_2019_ Journal of the Chinese Medical Association* | *Y* | *Y* | *Y* | *N* |
| *50* | *Song_2019_Infection and Drug Resistance* | *Y* | *Y* | *N* | *N* |
| *51* | *Adane_2023_JCTOMD* | *Y* | *Y* | *N* | *Y* |
| *52* | *Degner_2018_Clinical infectious diseases* | *Y* | *Y* | *N* | *Y* |
| *53* | *Liu_2021_American journal of respiratory and critical care medicine* | *Y* | *N* | *N* | *Y* |
| *54* | *Alkabab_2021_PloS one* | *Y* | *N* | *N* | *N* |
| *55* | *Gupte_2018_The international journal of tuberculosis and lung disease* | *Y* | *N* | *N* | *N* |
| *56* | *Byashalira_2022_Tropical medicine & international health* | *Y* | *Y* | *N* | *Y* |
| *57* | *Zhang_2021_Frontiers in public health* | *Y* | *Y* | *N* | *N* |
| *58* | *Kornfeld_2023_Diabetes research and clinical practice* | *Y* | *Y* | *N* | *Y* |
| *59* | *Mishra_2021_International Journal of Clinical Practice* | *Y* | *Y* | *Y* | *N* |
| *60* | *Tamuhla_2021_PloS one* | *Y* | *N* | *N* | *N* |
| *61* | *Viswanathan_2023_Diabetes & metabolic syndrome* | *Y* | *N* | *N* | *Y* |
| *62* | *Ruslami_2021_Diabetes research and clinical practice* | *Y* | *Y* | *N* | *Y* |
| *63* | *Pardeshi_2023_Chest* | *Y* | *Y* | *N* | *Y* |
| *64* | *George J T_2022_International Journal of Mycobacteriology* | *Y* | *Y* | *N* | *Y* |
| *65* | *Cassandra van Doorn_2022_EBioMedicine* | *Y* | *Y* | *N* | *Y* |
| *66* | *Tzanani_2022_Israel Medical Association Journal* | *N* | *Y* | *Y* | *Y* |
| *67* | *Arriaga, María B_2022_The Journal of infectious diseases* | *Y* | *Y* | *N* | *Y* |
| *68* | *Banurekha, Velayutham_2017_Indian journal of community medicine* | *Y* | *Y* | *N* | *Y* |
| *69* | *Malic_2021_The Moldovan Medical Journal* | *N* | *Y* | *Y* | *Y* |
| *70* | *Roshan_2023_South Eastern European Journal of Public Health* | *Y* | *Y* | *N* | *Y* |
| *71* | *Bezerra_2022_Jornal brasileiro de pneumologia* | *Y* | *Y* | *N* | *Y* |
| *72* | *Lee, Ye-Jin_2018_The Korean journal of internal medicine* | *Y* | *Y* | *Y* | *N* |
| *73* | *Krishnappa_2019_The Indian journal of medical research* | *Y* | *N* | *N* | *N* |
| *74* | *Barss_2019 Canadian Journal of Respiratory, Critical Care, and Sleep Medicine* | *Y* | *N* | *Y* | *Y* |
| *75* | *Sumana_2024_JFMPC_Aug 2024* | *Y* | *N* | *N* | *Y* |
| *76* | *Han shi_2024_World J Diabetes* | *N* | *Y* | *Y* | *N* |
| *77* | *Kalva_2023_Journal of public health* | *Y* | *N* | *N* | *N* |
| *78* | *Shah_2023_IJTB* | *Y* | *N* | *N* | *Y* |
| *79* | *Giri_2024_IJTB* | *Y* | *N* | *N* | *Y* |
| *80* | *Sharon_2023_BMC Health Services Research* | *Y* | *Y* | *Y* | *N* |
| *81* | *Hashiguchi_2024_PLOS Global Public Health* | *Y* | *Y* | *Y* | *N* |
| *82* | *Fanqi_2023_Frontiers in Endocrinology* | *Y* | *Y* | *Y* | *N* |
| *83* | *Sandeep_2024_IJCM* | *Y* | *N* | *N* | *Y* |
| *84* | *Rout_ 2023_Medical Journal of Dr. D.Y. Patil Vidyapeeth* | *N* | *Y* | *Y* | *Y* |
| *85* | *Kwak_2023_PLOS ONE* | *Y* | *Y* | *N* | *Y* |
| *86* | *Tong_2024_Frontiers in Medicine* | *Y* | *Y* | *Y* | *N* |
| *87* | *Rupani_2024_BMC Public Health* | *Y* | *Y* | *N* | *Y* |
| *88* | *Byashalira_2023_International Journal of Mycobacteriology* | *Y* | *Y* | *Y* | *N* |
| *89* | *Surinder_2023_Journal of Cardiovascular Disease Research* | *N* | *Y* | *N* | *N* |
| *90* | *Chung_2024_Korean J Intern Med* | *Y* | *Y* | *N* | *Y* |
| *91* | *Pardeshi_2024_Chest* | *Y* | *N* | *Y* | *Y* |
| *92* | *Fasil_2024_Journal of infection* | *N* | *N* | *N* | *Y* |
| *93* | *Adam_2023_Frontiers in Public Health* | *Y* | *N* | *N* | *Y* |

*T: Type of study = all interventional studies (randomized or non-randomized; individual or cluster randomized) and cohort studies on the topic with a control / non-exposed arm. P: participant criterion = people with TB and DM on anti-TB treatment; E/C: exposure/comparator = for research question on glycemic control, glycemic status was the exposure of interest. For the research question on insulin, the type of DM treatment was the exposure of interest. If any one of the two was present in the study, we included it; O: outcome = the study was included if any one of the primary outcomes were present; Y—yes; N—no. ^Studies in italics are from updated review from 26 April 2017 to 31 August 2024
