# Supplementary material for: Effect of glycemic control and type of diabetes treatment on TB treatment outcomes among people with TB-diabetes: A systematic review (updated August 2024)
Source: PLoS One. 2025 Jul 18;20(7):e0328619. doi: 10.1371/journal.pone.0328619 (PMC12273911; doi:10.1371/journal.pone.0328619)
Supplement: S2 Annex — (DOCX) [file pone.0328619.s002.docx]

**S2 Annex .** Characteristics of studies included in the review (1996 to 31 Aug 2024)

**Study ID: Chiang CY_2015_Plos One**

| Settings | Conducted in a programmatic setting in three teaching hospitals in North South and East Taiwan | |
| --- | --- | --- |
| Methods | Study design: Retrospective cohort  Study duration: 2005-1010  Diabetes diagnosis based on history of treatment with insulin / OHA OR ICD-9 code for DM was given at admission OR ICD-9 code was DM was given twice or more during OPD visit OR h/o DM.  HbA1c was also used for diagnosis (no mention of NGSP certification and standardized to the DCCT assay) | |
| Participants | 705 culture positive pulmonary TB DM patients of all sexes, age not reported irrespective of the HIV status diagnosed with DM within 3 months of initiation of ATT  Included TB -non DM arm with 768 patients | |
| Intervention/ Exposure | Criteria for classification of glycemic control at baseline:  HbA1c<7 – glycemic control arm  HbA1c 7-9-glycemic control – less stringent arm  HbA1c >9 - Poor glycemic control arm | |
| Outcomes | End treatment outcomes- death, treatment failure, loss to follow up | |
| Major conclusions | Poor glycemic control is associated with poor TB treatment outcome and improved glycemic control may reduce the influence of diabetes on TB. | |
| Comments | This paper does not provide direct evidence of effect of glycemic control on TB treatment outcomes among TB-DM patients. First, the comparison throughout the paper was with people without DM. Second, HbA1c values though available were used in the unadjusted analysis, but not in the adjusted analysis.  Adjusted effect of DM (reference was no DM) on TB treatment outcomes was determined before and after including DM related comorbidity (reference was no DM related comorbidity) in the multivariable model. No DM comorbidity included those without DM as well. Finding was that DM related comorbidity resulted in unfavourable TB treatment outcomes; and not DM. It was thus interpreted that hyperglycemia worsened TB treatment outcomes. | |
| **Risk of bias table** |  | |
| Representativeness of exposed cohort (truly representative/somewhat representative/selected group of users/no description of derivation of cohort) | | somewhat representative (of 705 TBDM, 195 (27.7%) info on HbA1c not available |
| Selection of unexposed cohort (drawn from same community as exposed/drawn from a different source/no description) | | same community |
| Exposure ascertainment (secure record or measurement / structured interview / written self-report/ no description) | | secure record or measurement |
| Demonstrated that outcome of interest was not present at start of study (yes/no/not clear) | | yes |
| Adjustment of confounders (age / sex / TB site / TB microbiological status / new or old TB / baseline BMI / baseline anemia / HIV status / Baseline tobacco / baseline alcohol) If yes, how many and which one? | | No (but for a different comparison) |
| Comparability of cohort / confounders Adjustment for other confounders ?mention which one? | | No (but for a different comparison) |
| Adjustment for at least four important confounders (age / sex / TB site / TB microbiological status / new or old TB / baseline BMI / baseline anemia / HIV status / Baseline tobacco / baseline alcohol) Yes / no / not clear | | No (but for a different comparison) |
| outcome assessment (independent blind / record review or linkage / self-report / no description | | record review |
| Adequacy of follow up (complete - all subjects accounted for / loss to follow up unlikely to introduce bias / loss to follow up likely to introduce bias / no statement | | Complete |

**Study ID: Mi F_2013_TMIH**

| Settings | Conducted in a programmatic setting in two TB clinics in Guangzhou Chest Hospital, South China | |
| --- | --- | --- |
| Methods | Study design: Retrospective cohort study  Study duration: September 2011 to June 2012  Diagnosis of DM was done if patient had two FBG measures ≥ 126 mg/dl, one at TB clinic and one at DM clinic | |
| Participants | 189 pulmonary/ extra pulmonary TBDM patients of all age groups, both sexes, HIV status not reported  Included TB non DM arm with 1400 patients | |
| Intervention/ Exposure | Criteria for classification of glycemic control (baseline, 2 months and 6 months)  Glycemic control – good: FBG<7.0 mmol/l  Glycemic control –poor: FBG 7.0- 10 mmol/l  Glycemic control- bad: FBG >10.0 mmol/l | |
| Outcomes | TB treatment outcomes – smear non conversion at 2 months, with favourable outcomes defined as cure and treatment completed and unfavourable outcomes defined as loss-to-follow-up, dead, failure and transfer-out | |
| Major conclusions | **End IP unfavourable outcome- sputum smear positive for acid-fast bacilli at 2 months:**  Comp1:good control vs poor; RR=2.33 (0.8,6.75)  Comp2:good vs bad; RR=1.55 (0.46,5.17)  Comp 3: good v/s poor plus bad; RR=1.97 (0.71,5.47)  (good control is reference)  **End treatment unfavourable outcome- failure**  Comp1:good control vs poor; RR=0.88(0.24,3.20)  Comp2:good vs bad; RR=0.97(0.26,4.69)  Comp 3: good vs poor plus bad; RR=0.91 (0.28, 2.94)  (good control is reference)  Diabetes mellitus in new smear-positive people with PTB was associated with failure to sputum smear convert at 2 months and adverse treatment outcomes of loss-to-follow-up and failure. | |
| Comments | They had glycemic control value at baseline, 2 mon and 6 mon. FBG cut offs (7, 7-10,>10mmol/l) used to define levels of glycemic control did not match with what we have specified in the protocol (7.2mmol/l, 7.2-8.99,>=9)  Though started with 185 people with TB-DM, data on end IP and end treatment outcomes were available for 86 and 77 respectively. And the percentages reported are not based on original cohort but on data availability on outcomes.  Unadjusted crude RRs was based on 2 month FBG and end IP outcome; and 6 month FBG and treatment failed at 6 months (which is cross sectional data). The analysis was cross-section despite a cohort design. Also, the summary measure (RR) used was appropriate for the design; but not for the cross-sectional analysis. | |
| **Risk of bias table** |  | |
| Representativeness of exposed cohort (truly representative/somewhat representative/selected group of users/no description of derivation of cohort) | | no description of derivation of TB DM glycemic control/uncontrolled cohort |
| Selection of unexposed cohort (drawn from same community as exposed/drawn from a different source/no description) | | same community |
| Exposure ascertainment (secure record or measurement / structured interview / written self-report/ no description) | | secure record or measurement |
| Demonstrated that outcome of interest was not present at start of study (yes/no/not clear) | | Yes |
| Adjustment of confounders (age / sex / TB site / TB microbiological status / new or old TB / baseline BMI / baseline anemia / HIV status / Baseline tobacco / baseline alcohol) If yes, how many and which one? | | No |
| Comparability of cohort / confounders Adjustment for other confounders ?mention which one? | | No |
| Adjustment for at least four important confounders (age / sex / TB site / TB microbiological status / new or old TB / baseline BMI / baseline anemia / HIV status / Baseline tobacco / baseline alcohol) Yes / no / not clear | | No |
| outcome assessment (independent blind / record review or linkage / self-report / no description | | record review |
| Adequacy of follow up (complete - all subjects accounted for / loss to follow up unlikely to introduce bias / loss to follow up likely to introduce bias / no statement | | incomplete follow up likely to introduce bias |

**Study ID: Magee MJ_2013_International J of Infectious Diseases**

| Settings | Conducted in a programmatic setting (as a pilot) at primary/secondary level in Lima, Peru | |
| --- | --- | --- |
| Methods | Study design: Retrospective/ prospective cohort- time of data collection unclear  Study duration: Jan2005-May2008  On DM treatment OR all those who reported DM were confirmed by fasting plasma glucose ≥7.0 mmol/l (126 mg/dl) OR 2-h plasma glucose/random blood glucose ≥ 11.1 mmol/l (200 mg/dl) OR ≥ glycosylated hemoglobin (HbA1c) 7%. (no mention of NGSP certification and standardized to the DCCT assay) | |
| Participants | Selected group of TB patients screened for high risk to become MDR TB (people with presumptive MDR-TB)  Included TB non DM arm of 1485 patients and 186 TB DM patients (pulmonary and EP), new and retreatment aged >=15 years of either sex, regardless of HIV status with DM | |
| Intervention/ Exposure | Based on documentation of control in records/ FBG below median/ FBG<136. Exact criteria not specified  Details on glucose lowering treatment available  OHA only- 56  Insulin only- 16  Both- 26 | |
| Outcomes | End treatment- Failed, default or died  Time to smear conversion- aHR= 2.2(1.1-4.1) where uncontrolled group is reference. This was faster in those with no history of TB previous TB treatment. Frequent DM care also had faster culture conversion but not significant. | |
| Major conclusions | Diabetes was common in a cohort of TB patients at high risk for drug-resistant TB. Despite prevalent multidrug-resistant TB among TB–DM patients, the majority had a favorable TB treatment outcome. | |
| Comments | Complete TB treatment outcome data were available for 136 (73.1%) TB–DM patients. Analysis done among these 176 was satisfactory (use of aHR). They had baseline and during TB treatment information of the following: glycemic control through HbA1c level, level of diabetes care (scored between 0 and 4) and diabetes medical treatment details. Though TB end-treatment outcomes have been described among TB-DM patients, there was no mention of effect of glycemic control and DM care on TB end-treatment outcomes. Though DM control was objectively measured, it was mentioned that DM control status was based on records (details not clear). | |
| **Risk of bias table** |  | |
| Representativeness of exposed cohort (truly representative/somewhat representative/selected group of users/no description of derivation of cohort) | | selected group of TB-DM (presumptive MDR) and only those 70% with TB outcome data included in the analysis |
| Selection of unexposed cohort (drawn from same community as exposed/drawn from a different source/no description) | | same |
| Exposure ascertainment (secure record or measurement / structured interview / written self-report/ no description) | | secure record |
| Demonstrated that outcome of interest was not present at start of study (yes/no/not clear) | | yes |
| Adjustment of confounders (age / sex / TB site / TB microbiological status / new or old TB / baseline BMI / baseline anemia / HIV status / Baseline tobacco / baseline alcohol) If yes, how many and which one? | | Yes, two, previous treatment, drug resistance |
| Comparability of cohort / confounders Adjustment for other confounders ?mention which one? | | Yes |
| Adjustment for at least four important confounders (age / sex / TB site / TB microbiological status / new or old TB / baseline BMI / baseline anemia / HIV status / Baseline tobacco / baseline alcohol) Yes / no / not clear | | No |
| outcome assessment (independent blind / record review or linkage / self-report / no description | | record review |
| Adequacy of follow up (complete - all subjects accounted for / loss to follow up unlikely to introduce bias / loss to follow up likely to introduce bias / no statement | | Incomplete follow up likely to introduce bias |

**Study ID: Nandakumar KV_2013_Plos One**

| Settings | Conducted in a programmatic setting, Primary / Secondary level peripheral health institutions in Malappuram district, Kerala, India | |
| --- | --- | --- |
| Methods | Study design: retrospective cohort  Study duration: April 2010-Sep2011  Diabetes diagnosis: h/o of DM/DM treatment OR FBG≥126 OR RBS≥200 at baseline | |
| Participants | 667 TB DM patients, new or retreatment, pulmonary or extrapulmonary, aged >=15 years, belonging to either sex, irrespective of HIV status | |
| Intervention/ Exposure | Criteria for glycemic control: assessed three times, at least one month apart and at least one in CP. Those with all three values available were classified as ‘known’ glycemic control. Those with all three value less than cut off was 'controlled'. Criteria for glycemic control too strict.  Glycemic cut offs: FBS<100, PPBS/RBS<140 | |
| Outcomes | Sputum non conversion at 2 months : RR=1.14(0.74, 1.75)  End treatment unfavourable outcomes- death, treatment failed, loss to follow up, not evaluated: RR=2.0(0.97, 4.13)  MDR | |
| Major conclusions | Could not confirm an adverse association between DM or its control during treatment and the course of response to TB treatment.DM screening in TB cases and recording of DM care needs to be improved to enable more conclusive evidence. | |
| Comments | Large number of people with ‘unknown’ glycemic control as three FBS values were required for a known status.  Very strict operational definition for ‘known’ glycemic status and ‘control’ glycemic status among those with ‘known’ glycemic status.  Analysis was appropriate | |
| **Risk of bias table** |  | |
| Representativeness of exposed cohort (truly representative/somewhat representative/selected group of users/no description of derivation of cohort) | | somewhat representative (of 667 TBDM, 427 (64%), exposure status was unknown based on their definition) |
| Selection of unexposed cohort (drawn from same community as exposed/drawn from a different source/no description) | | same |
| Exposure ascertainment (secure record or measurement / structured interview / written self-report/ no description) | | secure record. Due to the low cut-off (not in line with recommendations) and glycemic control criteria, we are not sure if glycemic status groups were representative |
| Demonstrated that outcome of interest was not present at start of study (yes/no/not clear) | | yes |
| Adjustment of confounders (age / sex / TB site / TB microbiological status / new or old TB / baseline BMI / baseline anemia / HIV status / Baseline tobacco / baseline alcohol) If yes, how many and which one? | | no |
| Comparability of cohort / confounders Adjustment for other confounders ?mention which one? | | no |
| Adjustment for at least four important confounders (age / sex / TB site / TB microbiological status / new or old TB / baseline BMI / baseline anemia / HIV status / Baseline tobacco / baseline alcohol) Yes / no / not clear | | no |
| outcome assessment (independent blind / record review or linkage / self-report / no description | | record review |
| Adequacy of follow up (complete - all subjects accounted for / loss to follow up unlikely to introduce bias / loss to follow up likely to introduce bias / no statement | | complete |

**Study ID: Park SW_2012_Eur J ClinMicrobiol Infect Dis**

| Settings | Study in a clinical setting in two tertiary hospitals Chung-Ang University Hospital and Yong San Hospital, South Korea | |
| --- | --- | --- |
| Methods | Study design: Retrospective cohort  Study duration: Jan 2005- Dec 2009  Diabetes diagnosis based on h/o of DM/DM treatment OR FBG≥126 OR RBS≥200 at baseline | |
| Participants | New pulmonary TB patients of whom were 124 TB DM patients and 368 Tb non DM patients aged >=15 years of either sex who were HIV negative | |
| Intervention/ Exposure | Criteria for glycemic control assessment at baseline  Glycemic control: HbA1c<7  Poor glycemic control HbA1c>=7 | |
| Outcomes | End IP outcome- culture non conversion 2months | |
| Major conclusions | Uncontrolled diabetics seem to have more cavities, higher positive smear rates and lack of culture conversion after two months of therapy. Therefore, TB people with uncontrolled diabetes should be carefully managed and treated. | |
| Comments | Comparison has been made with non-DM as reference. Above information is also not reliable as many did not have info on culture conversion. Crude numbers were extracted in those with and without glycemic control. | |
| **Risk of bias table** |  | |
| Representativeness of exposed cohort (truly representative/somewhat representative/selected group of users/no description of derivation of cohort) | | selected group of users |
| Selection of unexposed cohort (drawn from same community as exposed/drawn from a different source/no description) | | same |
| Exposure ascertainment (secure record or measurement / structured interview / written self-report/ no description) | | secure record |
| Demonstrated that outcome of interest was not present at start of study (yes/no/not clear) | | yes |
| Adjustment of confounders (age / sex / TB site / TB microbiological status / new or old TB / baseline BMI / baseline anemia / HIV status / Baseline tobacco / baseline alcohol) If yes, how many and which one? | | No |
| Comparability of cohort / confounders Adjustment for other confounders ?mention which one? | | no |
| Adjustment for at least four important confounders (age / sex / TB site / TB microbiological status / new or old TB / baseline BMI / baseline anemia / HIV status / Baseline tobacco / baseline alcohol) Yes / no / not clear | | no |
| outcome assessment (independent blind / record review or linkage / self-report / no description | | record review |
| Adequacy of follow up (complete - all subjects accounted for / loss to follow up unlikely to introduce bias / loss to follow up likely to introduce bias / no statement | | Incomplete follow up likely to introduce bias |

**Study ID: Tabarsi P_2014_Journal of Diabetes and Metabolic Disorder**

| Settings | Inpatients in a clinical setting in National Research Institution of Tuberculosis and Lung diseases, Tehran, Iran | |
| --- | --- | --- |
| Methods | Study design: Cohort prospective  Study duration: May 2012-May 2013  Diagnosis of DM based on HbA1c at baseline and 3months. Cut off <6.5% (no mention of NGSP certification and standardized to the DCCT assay)  Normal- normal- No DM  Normal- elevated- uncertain DM  Elevated – Normal- uncertain DM  Elevated – Elevated- DM | |
| Participants | New pulmonary TB patients aged >15 years, of either sex, irrespective of HIV status | |
| Intervention/ Exposure | No attempt to define glycemic control groups | |
| Outcomes | End treatment unfavourable outcomes- death, treatment failed, loss to follow up | |
| Major conclusions | There were changes in HbA1c during the first three-months of anti-TB treatment, but these were not associated with differences in TB treatment outcomes. Transient hyperglycemia should be considered in TB patients and needs to be taken into account in planning care and management. | |
| Comments | Though this study did not exactly fit into our research question(s), we could extract the data that we required. ‘Elevated-normal’ and ‘elevated-elevated’ were included as the study participants relevant to our review. | |
| **Risk of bias table** |  | |
| Representativeness of exposed cohort (truly representative/somewhat representative/selected group of users/no description of derivation of cohort) | | somewhat representative |
| Selection of unexposed cohort (drawn from same community as exposed/drawn from a different source/no description) | | same |
| Exposure ascertainment (secure record or measurement / structured interview / written self-report/ no description) | | secure record or measurement |
| Demonstrated that outcome of interest was not present at start of study (yes/no/not clear) | | yes |
| Adjustment of confounders (age / sex / TB site / TB microbiological status / new or old TB / baseline BMI / baseline anemia / HIV status / Baseline tobacco / baseline alcohol) If yes, how many and which one? | | no |
| Comparability of cohort / confounders Adjustment for other confounders ?mention which one? | | no |
| Adjustment for at least four important confounders (age / sex / TB site / TB microbiological status / new or old TB / baseline BMI / baseline anemia / HIV status / Baseline tobacco / baseline alcohol) Yes / no / not clear | | no |
| outcome assessment (independent blind / record review or linkage / self-report / no description | | record review |
| Adequacy of follow up (complete - all subjects accounted for / loss to follow up unlikely to introduce bias / loss to follow up likely to introduce bias / no statement | | complete |

**Study ID: Viswanathan V_2014_Journal of Diabetes and its complications**

| Settings | Outpatients in a programmatic setting in Five TUs from three districts in TN, India | |
| --- | --- | --- |
| Methods | Study design: retrospective cohort  Study duration: Jan 2011- Mar 2011  Diabetes was diagnosed at baseline based on previous h/o DM OR FPG and 2hPG used but exact criteria not clear  Details of Glucose lowering treatment also elicited | |
| Participants | New pulmonary TB patients, 96 TB DM and 148 Tb non DM, aged >=18 years, of either sex whose HIV status is not reported | |
| Intervention/ Exposure | OHA only n=53, insulin onlyn=18, both n=3, none | |
| Outcomes | End treatment unfavourable outcomes individually death, treatment failed, loss to follow up, not evaluated, MDR Tb | |
| Major conclusions | delayed sputum conversion and high failure rates in TB treatment outcome were common in new smear-positive pulmonary TB people with diabetes. | |
| Comments | Though information on DM treatment was available, sufficient numbers of TB-DM and sufficient numbers of outcomes were not present for us to make any meaningful conclusions | |
| **Risk of bias table** |  | |
| Representativeness of exposed cohort (truly representative/somewhat representative/selected group of users/no description of derivation of cohort) | | somewhat representative |
| Selection of unexposed cohort (drawn from same community as exposed/drawn from a different source/no description) | | same |
| Exposure ascertainment (secure record or measurement / structured interview / written self-report/ no description) | | self-report |
| Demonstrated that outcome of interest was not present at start of study (yes/no/not clear) | | yes |
| Adjustment of confounders (age / sex / TB site / TB microbiological status / new or old TB / baseline BMI / baseline anemia / HIV status / Baseline tobacco / baseline alcohol) If yes, how many and which one? | | no |
| Comparability of cohort / confounders Adjustment for other confounders ?mention which one? | | no |
| Adjustment for at least four important confounders (age / sex / TB site / TB microbiological status / new or old TB / baseline BMI / baseline anemia / HIV status / Baseline tobacco / baseline alcohol) Yes / no / not clear | | no |
| outcome assessment (independent blind / record review or linkage / self-report / no description | | record review |
| Adequacy of follow up (complete - all subjects accounted for / loss to follow up unlikely to introduce bias / loss to follow up likely to introduce bias / no statement | | incomplete follow up unlikely to introduce bias |

**Study ID: Yoon YS_2017_Thorax**

| Settings | Done in a clinical setting in 10 referral hospitals South Korea | |
| --- | --- | --- |
| Methods | Study design: Prospective cohort  Study duration: Sep2012- Sep2014  Diabetes diagnosis based on history or HbA1c≥6.5%  (no mention of NGSP certification and standardized to the DCCT assay) | |
| Participants | New pulmonary TB patients157 TB DM and 504 TB non DM patients, aged 18-75 years of either sex, excluding those with HIV positive | |
| Intervention/ Exposure | Criteria for classifying glycemic control at baseline  Glycemic control: HbA1c<7  Glycemic control-less stringent- HbA1c 7-8.99  Poor glycemic control: HbA1c>=9 | |
| Outcomes | End IP unfavourable- culture non conversion at 2 months  End treatment unfavourable: death, failure, default | |
| Major conclusions | Uncontrolled diabetes is an independent risk factor for poor treatment response in PTB | |
| Comments | The HbA1c cut offs were not as per our protocol. Though mentioned three subgroups of glycemic controls based on HbA1c, they eventually used <7 and≥ 7 at baseline for glycemic control (two subgroups). For adjusted analysis of effect of glycemic control on end IP and end treatment outcomes, reference used was people without DM. OR was used as the summary measure instead of RR. However, we were able to extract the crude numbers for treatment outcomes among the subgroups of glycemic control and people with TB-DM. | |
| **Risk of bias table** |  | |
| Representativeness of exposed cohort (truly representative/somewhat representative/selected group of users/no description of derivation of cohort) | | Some what representative |
| Selection of unexposed cohort (drawn from same community as exposed/drawn from a different source/no description) | | same |
| Exposure ascertainment (secure record or measurement / structured interview / written self-report/ no description) | | secure record |
| Demonstrated that outcome of interest was not present at start of study (yes/no/not clear) | | yes |
| Adjustment of confounders (age / sex / TB site / TB microbiological status / new or old TB / baseline BMI / baseline anemia / HIV status / Baseline tobacco / baseline alcohol) If yes, how many and which one? | | yes but for a different comparison (comparison was between controllled / uncontrolled and non-DM |
| Comparability of cohort / confounders Adjustment for other confounders ?mention which one? | | yes but for a different comparison (comparison was between controllled / uncontrolled and non-DM |
| Adjustment for at least four important confounders (age / sex / TB site / TB microbiological status / new or old TB / baseline BMI / baseline anemia / HIV status / Baseline tobacco / baseline alcohol) Yes / no / not clear | | yes but for a different comparison (comparison was between controllled / uncontrolled and non-DM |
| outcome assessment (independent blind / record review or linkage / self-report / no description | | record review |
| Adequacy of follow up (complete - all subjects accounted for / loss to follow up unlikely to introduce bias / loss to follow up likely to introduce bias / no statement | | incomplete follow up likely to introduce bias |

**Study ID: Mahishale V_2017_Iran J MS**

| Settings | Programmatic setting in departments of Pulmonary Medicine, Internal Medicine, Diabetes and Endocrine Department and DOTS centre (TB unit) at a tertiary care hospital, Belgaum, Karnataka | |
| --- | --- | --- |
| Methods | Study design: Prospective cohort  Study duration: Jan 2012- Dec 2014  DM was diagnosed if baseline FBS was more than 126 mg/dl or PPBS more than 200 mg/dl | |
| Participants | 675 new pulmonary TB patients belonging to either sexes, age group unspecified, excluding known HIV positive | |
| Intervention/ Exposure | Glycemic control was defined as baseline  Poor glycemic control- HbA1c>=7%  Optimal glycemic control- HbA1c<7%  (no mention of NGSP certification and standardized to the DCCT assay) | |
| Outcomes | End IP- Smear positivity at 2 months  End treatment- death, default, treatment failed, MDR TB, recurrence | |
| Major conclusions | Poor glycemic control is associated with an increased risk of advanced and more severe TB disease in the form of lung cavitations, positive sputum smear, and slower smear conversion. It has a profound negative effect on treatment completion, cure, and relapse rates in people with pulmonary tuberculosis. | |
| Comments | Used OR instead of RR. Glycemic control was based on baseline HbA1c. Additional HbA1c measurements during treatment would have been helpful | |
| **Risk of bias table** |  | |
| Representativeness of exposed cohort (truly representative/somewhat representative/selected group of users/no description of derivation of cohort) | | truly |
| Selection of unexposed cohort (drawn from same community as exposed/drawn from a different source/no description) | | same |
| Exposure ascertainment (secure record or measurement / structured interview / written self-report/ no description) | | secure record/ measurement |
| Demonstrated that outcome of interest was not present at start of study (yes/no/not clear) | | yes |
| Adjustment of confounders (age / sex / TB site / TB microbiological status / new or old TB / baseline BMI / baseline anemia / HIV status / Baseline tobacco / baseline alcohol) If yes, how many and which one? | | Yes  age, duration of DM, nutritional status (BMI), socioeconomic status, smoking, comorbidities, extent of lung disease, lung cavitations, and bacteriology |
| Comparability of cohort / confounders Adjustment for other confounders ? mention which one? | | Yes |
| Adjustment for at least four important confounders (age / sex / TB site / TB microbiological status / new or old TB / baseline BMI / baseline anemia / HIV status / Baseline tobacco / baseline alcohol) Yes / no / not clear | | Yes |
| outcome assessment (independent blind / record review or linkage / self-report / no description | | record review |
| Adequacy of follow up (complete - all subjects accounted for / loss to follow up unlikely to introduce bias / loss to follow up likely to introduce bias / no statement | | Loss to follow up unlikely to introduce bias |

***Study ID: Kornfeld H_2020_Clinical infectious diseases****

| *Settings* | *Private, tertiary, outpatient, clinical setting in urban Chennai, Tamil Nadu, India* | |
| --- | --- | --- |
| *Methods* | *Study design: Prospective cohort*  *Study duration: Jan 2014-June 2018* | |
| *Participants* | *256 TB DM patients [94 (new DM)/162 (already had DM)], culture positive, adult, new pulmonary TB patients, 25-60 years, all sexes* | |
| *Intervention/ Exposure* | *Criteria for classification of glycemic control at baseline used among all TB patients (this information is not available among the 256 TB-DM patients):*  *HbA1c < 8% – Glycemic control*  *HbA1c ≥8 % - Suboptimal / Poor/no glycemic control arm* | |
| *Outcomes* | *Baseline TB severity, sputum conversion and treatment outcomes (cure, failure, death, loss to follow-up) were compared between groups with respect to glycemic control status and BMI* | |
| *Major conclusions* | *The study reported poorly controlled diabetes (HbA1c ≥8 %) combined with low BMI was unexpectedly associated with better TB treatment outcomes.* | |
| *Comments* | *This paper stratifies TB patients (n=389) based on HbA1c 8% cut off and BMI 18.5 kg/m^2^ cut off into four categories at baseline (TB treatment initiation). The outcomes of these patients were assessed at six months and compared after adjusting for age, sex, height, household income, smoking and alcohol consumption. This paper does not provide direct evidence of the effect of glycemic control on TB treatment outcomes among TB-DM patients. This paper offers the impact of glycemic status and BMI on TB treatment outcomes among TB patients.*  *The study provides limited clarity as ‘when’ these patients were enrolled after notification. Lack of clarity about the denominator from where and which date ranges of notification the patients were enrolled and potential delay in enrolment (from notification) could result in selection bias and not getting ‘true’ baseline HbA1C. During treatment lost to follow up were excluded (extent known) from the analysis and then cure rate was calculated (both crude and adjusted analysis). Those not evaluated (extent unknown) for treatment outcomes were excluded from the analysis. Ideally as per WHO, these should have been reported and included in the denominator and reported as ‘not cured/unfavourable outcome’ in the numerator. Patients without evaluable data were also excluded. Operational definition for ‘evaluable data’ is not mentioned as well.*  *Additionally, analysis wise relative risk should have been used in line with the study design (cohort study). The odds ratio is statistically more robust when compared to relative risk. Here in statistically significant adjusted OR values for cure as the outcome, the lower limit of 95% confidence interval is close to one (null value). If relative risk was used, there is a possibility that these results may not be statistically significant. Height should not be included in the adjusted analysis as BMI categorized using 18.5 cut off was a key exposure variable height is part of BMI derivation*  *On request, authors provided* *unadjusted and adjusted relative risk (for both primary objectives) after adjusting for age, sex, body mass index, glycemic status at baseline / use of insulin during treatment (as applicable based on primary objective), household income, smoking, alcohol consumption. They included only TB-DM patients with loss to follow up included in the analysis. However, patients without evaluable data remained excluded (extent unknown).* | |
| ***Risk of bias table*** |  | |
| *Representativeness of exposed cohort (truly representative/somewhat representative/selected group of users/no description of derivation of cohort)* | | *No/ limited description of derivation of cohort* |
| *Selection of unexposed cohort (drawn from same community as exposed/drawn from a different source/no description)* | | *same community* |
| *Exposure ascertainment (secure record or measurement / structured interview / written self-report/ no description)* | | *secure record or measurement* |
| *Demonstrated that outcome of interest was not present at start of study (yes/no/not clear)* | | *yes* |
| *Adjustment of confounders (age / sex / TB site / TB microbiological status / new or old TB / baseline BMI / baseline anemia / HIV status / Baseline tobacco / baseline alcohol) If yes, how many and which one?* | | *Yes (age, sex, body mass index, glycemic control status at baseline or insulin use depending on the primary objective, household income, smoking, alcohol consumption)* |
| *Comparability of cohort / confounders Adjustment for other confounders? Mention which one?* | | *Yes (age, sex, body mass index, glycemic control status at baseline or insulin use depending on the primary objective, household income, smoking, alcohol consumption)* |
| *Adjustment for at least four important confounders (age / sex / TB site / TB microbiological status / new or old TB / baseline BMI / baseline anemia / HIV status / Baseline tobacco / baseline alcohol) Yes / no / not clear* | | *Yes* |
| *Outcome assessment (independent blind / record review or linkage / self-report / no description* | | *record review* |
| *Adequacy of follow up (complete - all subjects accounted for / loss to follow up unlikely to introduce bias / loss to follow up likely to introduce bias / no statement* | | *Patients without evaluable (extent unknown) excluded from the calculation of cure rate (crude and adjusted analysis) which not in line WHO recommendation. Likely to introduce bias* |

***Study ID: Udaykumar P_2022_Clinical Epidemiology and Global Health****

| *Settings* | *Outpatient, primary/secondary/district, programmatic, 5 TU units from Dakshina district of Karnataka, India* | |
| --- | --- | --- |
| *Methods* | *Study design: cohort study*  *Study duration: July 2017 to Dec 2019*  *If FBS greater than 126 mg/dl the patients were labelled as diabetes* | |
| *Participants* | *102 TB DM patients, adult (18-65 yrs), both sexes, both pulmonary and extrapulmonary, microbiologically confirmed* | |
| *Intervention/ Exposure* | *OHA only arm- control (n=32)*  *Insulin only arm (n=11)*  *Insulin and OHA arm (n=59)*  *Unknown (n-=2)*  *At initiation, three months and end of treatment*  *HbA1C diabetic range (as stated by authors, cut off not known)*  *HbA1C normal (as stated by authors, cut off not known)*  *Systematic diabetic monitoring was done in one geographical area (n=52) whereas in other areas (n=50) monitoring was not done* | |
| *Outcomes* | *TB treatment outcome (negative results, treatment success, lost to follow up)* | |
| *Major conclusions* | *Sample size was not sufficient to answer the primary objective that was to assess the effect of monitoring of Diabetes treatment on unfavourable treatment outcomes among patients with TB-DM. Same applies to our objective of interest for which data on HbA1C was there at three time points during the treatment* | |
| *Comments* | *We could not infer valuable results from the study owing to its limited sample size* | |
| ***Risk of bias table*** |  | |
| *Representativeness of exposed cohort (truly representative/somewhat representative/selected group of users/no description of derivation of cohort)* | | *Somewhat representative* |
| *Selection of unexposed cohort (drawn from same community as exposed/drawn from a different source/no description)* | | *same community* |
| *Exposure ascertainment (secure record or measurement / structured interview / written self-report/ no description)* | | *secure record or measurement* |
| *Demonstrated that outcome of interest was not present at start of study (yes/no/not clear)* | | *Yes* |
| *Adjustment of confounders (age / sex / TB site / TB microbiological status / new or old TB / baseline BMI / baseline anemia / HIV status / Baseline tobacco / baseline alcohol) If yes, how many and which one?* | | *No* |
| *Comparability of cohort / confounders Adjustment for other confounders ?mention which one?* | | *No* |
| *Adjustment for at least four important confounders (age / sex / TB site / TB microbiological status / new or old TB / baseline BMI / baseline anemia / HIV status / Baseline tobacco / baseline alcohol) Yes / no / not clear* | | *No* |
| *Outcome assessment (independent blind / record review or linkage / self-report / no description* | | *record review* |
| *Adequacy of follow up (complete - all subjects accounted for / loss to follow up unlikely to introduce bias / loss to follow up likely to introduce bias / no statement* | | *Study participant loss to follow-up likely to introduce bias, especially considering they were not reported as unfavorable outcome, likely to introduce bias.* |

***Study ID: Baltas I_2023_The international journal of tuberculosis and lung disease****

| *Settings* | *Outpatient, tertiary, clinical, public and all new TB cases in the United Kingdom are notified to a central register by each treatment centre, which is mandatory. All TB patients notified by Northwick Park Hospital between 1 January 2016 and 31 December 2020* | |
| --- | --- | --- |
| *Methods* | *Study design: Retrospective cohort*  *Study duration: 1st Jan 2016-31st Dec 2020*  *A single glycated haemoglobin (HbA1c) was recorded if available from 3 months before the date of TB treatment initiation until the date of treatment cessation. If multiple measurements were available, the result chronologically closest to the TB diagnosis was used* | |
| *Participants* | *126 TB DM pts, 123 TB Ty2 DM pts, 105 TB Ty2 DM on Rx, 3 had no assessable outcome, Hence, 102 TB DM pts. Both new and retreatment cases,*  *>15 yrs, both sexes, included HIV patients, both pulmonary and extrapulmonary cases, both microbiologically confirmed and clinically confirmed* | |
| *Intervention/ Exposure* | *Diabetes diagnosing criteria HbA1c ≥48mmol/mol (corresponds to 6.5%)*  *Poorly controlled DM- HbA1c ≥64 mmol/mol (corresponds to 8%)*  *Measured during baseline, during IP, during CP* | |
| *Outcomes* | *Unfavourable TB treatment outcomes (‘‘treatment failed’’, ‘‘died’’ or ‘‘loss to follow-up)* | |
| *Major conclusions* | *Among TB-DM, treatment outcomes by glycemic control status not provided. On author request among TB-DM group, TB treatment outcomes by DM treatment have been provided. In OHA (only) group, 10.5% (9/86) had unfavourable outcomes. In OHA plus insulin group, 6.3% (1/16) had an unfavourable outcome* | |
| *Comments* | *Lack of data and low sample size prevents any meaningful inferences from the data. Single HbA1C measure could be anytime from three months before TB diagnosis to treatment outcome (potential for lack of temporality in some instances)* | |
| ***Risk of bias table*** |  | |
| *Representativeness of exposed cohort (truly representative/somewhat representative/selected group of users/no description of derivation of cohort)* | | *Somewhat representative* |
| *Selection of unexposed cohort (drawn from same community as exposed/drawn from a different source/no description)* | | *same* |
| *Exposure ascertainment (secure record or measurement / structured interview / written self-report/ no description)* | | *secure record or measurement* |
| *Demonstrated that outcome of interest was not present at start of study (yes/no/not clear)* | | *Yes* |
| *Adjustment of confounders (age / sex / TB site / TB microbiological status / new or old TB / baseline BMI / baseline anemia / HIV status / Baseline tobacco / baseline alcohol) If yes, how many and which one?* | | *No* |
| *Comparability of cohort / confounders Adjustment for other confounders ?mention which one?* | | *No* |
| *Adjustment for at least four important confounders (age / sex / TB site / TB microbiological status / new or old TB / baseline BMI / baseline anemia / HIV status / Baseline tobacco / baseline alcohol) Yes / no / not clear* | | *No* |
| *Outcome assessment (independent blind / record review or linkage / self-report / no description* | | *record review* |
| *Adequacy of follow up (complete - all subjects accounted for / loss to follow up unlikely to introduce bias / loss to follow up likely to introduce bias / no statement* | | *Study participants lost to follow-up likely to introduce bias.* |

***Study ID: Kim KH_March 2024_Respirology***

| *Settings* | *Outpatient, tertiary, clinical, enrolled patients from 18 university affiliated hospitals (COSMOTB subgroup analysis)* | |
| --- | --- | --- |
| *Methods* | *Study design: Prospective cohort*  *Study duration: 2019-2021*  *At baseline, diagnosis of DM was based on,*  *(1) random glucose level ≥200 mg/dL, (2) HbA1c ≥6.5% or (3) previous DM history* | |
| *Participants* | *328 TB DM patients from COSMO TB subgroup analysis*  *Age >/=19 yrs*  *Predominantly male, HIV status unknown, pulmonary TB, microbiologically and clinically confirmed TB* | |
| *Intervention/ Exposure* | 1. ***At follow up:***   *Glycemic control arm-RBG <180 [212/328,64.6%]*  *Glycemic control, not strict arm- NA*  *No glycemic control arm-RBG ≥180[116/328, 35.4%]*  *Number unfavorable outcomes-not reported, aOR given.*  *Association described- Controlled and uncontrolled DM (poor glycemic control during follow up / during Tb treatment) with unfavorable outcomes and all-cause mortality*  **Info on treated and untreated TB DM pts is given. No info on specific treatment details.* | |
| *Outcomes* | 1. ***At follow up***   *Uncontrolled DM (poor glycemic control) not significantly associated with unfavorable outcomes*  *aOR=1.29, 95% CI (0.67-2.35)*  *Uncontrolled DM (poor glycemic control) not significantly associated with all-cause mortality*  *aOR 1.37, 95% CI(0.63-3.92)* | |
| *Major conclusions* | *The study did not find any significant association between uncontrolled DM during follow up (during TB treatment) and unfavorable outcome, all-cause mortality* | |
| *Comments* | *Random blood glucose is not a reliable test for glycemic control. The number/proportion of patients with uncontrolled TB DM (poor glycemic control during follow up) having unfavorable outcomes is not mentioned.*  *Adjusted analysis done. No adjustment done for insulin use.*  *The number of TB patients on DM treatment and untreated DM are mentioned. However, the number of patients specifically on insulin/OHA is not mentioned.* | |
| ***Risk of bias table*** |  | |
| *Representativeness of exposed cohort (truly representative/somewhat representative/selected group of users/no description of derivation of cohort)* | | *Not representative* |
| *Selection of unexposed cohort (drawn from same community as exposed/drawn from a different source/no description)* | | *Not representative* |
| *Exposure ascertainment (secure record or measurement / structured interview / written self-report/ no description)* | | *Secure record/measurement* |
| *Demonstrated that outcome of interest was not present at start of study (yes/no/not clear)* | | *Yes* |
| *Adjustment of confounders (age / sex / TB site / TB microbiological status / new or old TB / baseline BMI / baseline anemia / HIV status / Baseline tobacco / baseline alcohol) If yes, how many and which one?* | | *Yes, seven, [Diabetes, age, BMI, malignancy, prior TB history, No TB related symptoms, initial severe disease]* |
| *Comparability of cohort / confounders Adjustment for other confounders? Mention which one?* | | *Yes [Diabetes, age, BMI, malignancy, prior TB history, No TB related symptoms, initial severe disease]* |
| *Adjustment for at least four important confounders (age / sex / TB site / TB microbiological status / new or old TB / baseline BMI / baseline anemia / HIV status / Baseline tobacco / baseline alcohol) Yes / no / not clear* | | *Yes* |
| *Outcome assessment (independent blind / record review or linkage / self-report / no description* | | *Record review* |
| *Adequacy of follow up (complete - all subjects accounted for / loss to follow up unlikely to introduce bias / loss to follow up likely to introduce bias / no statement* | | *All subjects accounted for* |

***Study ID: J Mistry_April 2024_Asian J Pharm Clin Res***

| *Settings* | *OPD and IPD, tertiary teaching, clinical, patients who come to OPD and IPD of respiratory medicine at tertiary health-care centers have symptoms of tuberculosis* | |
| --- | --- | --- |
| *Methods* | *Study design: Prospective cohort*  *Study duration: March 2021 to September 2022*  *DM diagnosing criteria not mentioned* | |
| *Participants* | *65 TB DM patients. Age and gender not mentioned, both pulmonary and extra-pulmonary TB, microbiologically confirmed, clinically diagnosed* | |
| *Intervention/ Exposure* | ***At baseline:***  *Glycemic control arm- HbA1c <7% [1/65]*  *Glycemic control, not strict arm- HbA1c 7-7.9% [2/65]*  *No glycemic control arm- HbA1c ≥8% [62/65]*  *Association described: Poor glycemic control and Tb treatment outcome*  *Adjusted for age*  ***Info on glucose-lowering treatment given***  *OHA only arm [43/65]*  *Insulin (+/- OHA) arm [22/65]*  *Association described: DM treatment and TB treatment outcomes*  *Adjusted for age* | |
| *Outcomes* | *Those on glycemic control arm- 0 had unfavorable outcome*  *Those on glycemic control, not strict arm- 0 had unfavorable outcome*  *Those on no glycemic control arm- 8 had unfavorable outcome*  *Those on OHA only arm- 3 had unfavorable outcome*  *Those on insulin (+/-OHA)- 5 had unfavorable outcome* | |
| *Major conclusions* | *Patients of ATT with poor glycemic control had a higher percentage of sputum positivity. This study recommends the management of glycemic levels in patients receiving ATT+ADD for better outcomes.* | |
| *Comments* | *The study provides limited clarity on ‘when’ enrolment happened after notification (baseline/follow up). Low sample size to infer the findings and generalize the results. No data on patient characteristics. No adjusted analysis.* | |
| ***Risk of bias table*** |  | |
| *Representativeness of exposed cohort (truly representative/somewhat representative/selected group of users/no description of derivation of cohort)* | | *Somewhat (low SS)* |
| *Selection of unexposed cohort (drawn from same community as exposed/drawn from a different source/no description)* | | *Same* |
| *Exposure ascertainment (secure record or measurement / structured interview / written self-report/ no description)* | | *Secure record/measurement* |
| *Demonstrated that outcome of interest was not present at start of study (yes/no/not clear)* | | *Yes* |
| *Adjustment of confounders (age / sex / TB site / TB microbiological status / new or old TB / baseline BMI / baseline anemia / HIV status / Baseline tobacco / baseline alcohol) If yes, how many and which one?* | | *Adjusted only for age* |
| *Comparability of cohort / confounders Adjustment for other confounders ?mention which one?* | | *No* |
| *Adjustment for at least four important confounders (age / sex / TB site / TB microbiological status / new or old TB / baseline BMI / baseline anemia / HIV status / Baseline tobacco / baseline alcohol) Yes / no / not clear* | | *No, only age* |
| *Outcome assessment (independent blind / record review or linkage / self-report / no description* | | *Record review* |
| *Adequacy of follow up (complete - all subjects accounted for / loss to follow up unlikely to introduce bias / loss to follow up likely to introduce bias / no statement* | | *loss to follow up likely to introduce bias* |

**Studies in italics are from updated review from 26 April 2017 to 31 August 2024*
