# Supplementary material for: Effect of glycemic control and type of diabetes treatment on TB treatment outcomes among people with TB-diabetes: A systematic review (updated August 2024)
Source: PLoS One. 2025 Jul 18;20(7):e0328619. doi: 10.1371/journal.pone.0328619 (PMC12273911; doi:10.1371/journal.pone.0328619)
Supplement: S3 Annex — (DOCX) [file pone.0328619.s003.docx]

**S3 Annex .** Characteristics of ongoing studies (1996-31 Aug 2024)

1. **Study ID: ChiCTR-IPR-15006395**

| Title | The influence and mechanism of vitamin D3 supplementation on the treatment outcomes of tuberculosis patients of different glucose tolerance |
| --- | --- |
| Principal Investigator / contacts | Wang Qiuzhen, Qingdao University, China  [kevin_1971@126.com](mailto:kevin_1971@126.com) |
| Weblink | <http://www.chictr.org.cn/showproj.aspx?proj=10964> |
| Enrolment status | Unknown status |
| Study design | Randomized parallel controlled trial |
| Country | China |
| Participants | Pulmonary TB patients with DM |
| Eligibility criteria | Inclusion Criteria:  new diagnosed tuberculosis, sputum smear positive, aged >=18, stable address  Exclusion Criteria:  HIV positive, tumor, pregnant or lactase women, injured lately, adjusted calcium concentration >2.65mmol/L |
| Published | Not known (No response for the paper from authors over email)  Exposure does not match with our review criteria |

1. **Study ID: ChiCTR-TRC-12002546**

| Title | The Effect and Mechanism of Retinol and Vitamin A Supplementation in people with diabetes and pulmonary tuberculosis |
| --- | --- |
| Principal Investigator / contacts | Wang Qiuzhen, Qingdao University, China  [kevin_1971@126.com](mailto:kevin_1971@126.com) Aiguo Ma, Qingdao University, China  [maiguo@public.qd.sd.cn](mailto:maiguo@public.qd.sd.cn) |
| Weblink | <http://www.chictr.org.cn/showproj.aspx?proj=7005> |
| Enrolment status | Completed |
| Study design | Randomized parallel controlled trial |
| Country | China |
| Participants | Pulmonary TB people with DM |
| Eligibility criteria | Inclusion Criteria:  The people with pulmonary tuberculosis and diabetes, aged 18~75, diagnosed by the golden criteria of pulmonary tuberculosis and diabetes; no vitamin or mineral supplement one month before the screening.  Exclusion Criteria:  No severe complications of diabetes including diabetic eye diseases, renal disease and foot disease, etc; pregnancy or lactation women; cancer; coronary heart disease; recent suffered trauma or underwent surgery |
| Published | Not known (No response for the paper from authors over email)  Exposure does not match with our review criteria |

1. **Study ID: ChiCTR-TRC-10001032**

| Title | The Effect of retinol and vitamin D Supplementation on nutritional state and Treatment Outcome of people with Pulmonary Tuberculosis and diabetes mellitus in China |
| --- | --- |
| Principal Investigator / contacts | Wang Qiuzhen, Qingdao University, China  [kevin_1971@126.com](mailto:kevin_1971@126.com) Aiguo Ma, Qingdao University, China  maiguo@public.qd.sd.cn |
| Weblink | <http://www.chictr.org.cn/showproj.aspx?proj=8506> |
| Enrolment status | Completed |
| Study design | Randomized parallel controlled trial |
| Country | China |
| Participants | Pulmonary TB patients with DM |
| Eligibility criteria | Inclusion Criteria:  1.patient who plan to stay in local place for 2 years; 2. three sputum specimens positive for acid-fast bacilli by direct microscopy and culture; 3. clinical and radiologic signs consistent with pulmonary tuberculosis; 4. no history of previous antituberculosis treatment.  Exclusion Criteria:  1. drug resistance at baseline or during the follow up; 2. extrapulmonary tuberculosis; 3. pregnancy; 4. lactation; 5. use of corticosteroids or supplements containing vitamin A or vitamin D during the previous month; 6. moderate to severe injury or surgery during the previous month; 7. chronic renal failure, liver disease or heart failure. |
| Published | Not known (No response for the paper from authors over email)  Exposure does not match with our review criteria |

1. **Study ID: NCT02169570**

| Title | Effect of Supplementary Vitamin D in Patients With Diabetes Mellitus and Pulmonary Tuberculosis (EVIDENT Study): a Randomized, Double Blind, Controlled Trial |
| --- | --- |
| Principal Investigator / contacts | Nadia - Shah, MAS  [nadia.shah@live.com](mailto:nadia.shah@live.com)  Saadiyah Rao, MSc  [dr.saadrao@gamil.com](mailto:dr.saadrao@gamil.com) |
| Weblink | <https://clinicaltrials.gov/ct2/show/record/NCT02169570> |
| Enrolment status | Unknown status |
| Study design | Interventional  Allocation: Randomized  Intervention Model: Parallel Assignment  Masking: Double Blind (Participant, Care Provider, Outcomes Assessor)  Primary Purpose: Treatment |
| Country | Pakistan |
| Participants | Pulmonary TB patients with type 2 DM |
| Eligibility criteria | Inclusion Criteria:  Age 30 to 60 years  Patients having both TB and type 2 DM  Patients consenting to participate  No history of previous ATT  Plane to have ATT and DM treatment  Exclusion Criteria:  Age less than 30 years or greater than 60 years  Pregnant women  Patients having either TB or type 2 DM  Patients refuse to participate  Patients having extra-pulmonary TB or Multi-drug resistant MDR TB or relapse cases  Patients having hepatic or renal diseases or HIV infection  Patients having hypo- or hyper-parathyroidism  Patients on corticosteroids or immunosuppressive or thiazides diuretics or any other drugs known to interfere with vitamin D levels |
| Published | Not known (No response for the paper from authors over email)  Exposure does not match with our review criteria |

1. **Study ID: NCT02106039**

| Title | Concurrent Tuberculosis and Diabetes: Clinical Monitoring, and Microbiological and Immunological Effects of Diabetes During Tuberculosis Treatment |
| --- | --- |
| Principal Investigator / contacts | Hazel Dockrell, Prof  [Hazel.Dockrell@lshtm.ac.uk](mailto:Hazel.Dockrell@lshtm.ac.uk)  Reinout van Crevel, MD, PhD  [reinout.vancrevel@radboudumc.nl](mailto:reinout.vancrevel@radboudumc.nl) |
| Weblink | <https://clinicaltrials.gov/ct2/show/record/NCT02106039> |
| Enrolment status | Completed |
| Study design | Interventional  Allocation: Randomized  Intervention Model: Parallel Assignment  Masking: No masking  Primary Purpose: Treatment |
| Country | Indonesia, Peru, Romania |
| Participants | Pulmonary TB patients with DM |
| Eligibility criteria | Inclusion Criteria:  adult (> 18 years old) diabetes mellitus patients  diagnosed as having active pulmonary TB  willing to join the study  Exclusion Criteria:  under TB treatment more than 72 hours  steroid-induced or gestational diabetes |
| Experimental arm  Control arm | Intensive monitoring: more intensive monitoring strategy of blood glucose and clinical review  Standard monitoring: glucose monitoring following the prevailing practice at each site |
| Outcome | Better diabetes control in diabetes people with tuberculosis under treatment [Time Frame: Up to 6 months during TB treatment ]  Diabetes control is determined by HbA1c level which will be measured at month 3 and 6 of TB treatment. |
| Published | One of the paper published under this trial is  **The effect of a structured clinical algorithm on glycemic control in patients with combined tuberculosis and diabetes in Indonesia: A randomized trial**  We included this study in Tiab screening. Excluded during full text screening.  The authors have outcome data, which they are analysing, that is not yet published (reply from authors) |

***Updated review:***

1. ***Study ID: ISRCTN16347615***

| *Title* | *The effect of malnutrition and diabetes on outcomes for drug-resistant and drug sensitive patients starting anti-tuberculosis treatment* |
| --- | --- |
| *Principal Investigator / contact* | *Sharon    Cox*  [*sharon.cox@lshtm.ac.u*](mailto:sharon.cox@lshtm.ac.u)  *Shuichi    Suzuki*  *suzuki_shuichi@nagasaki-u.ac.jp* |
| *Weblink* | [*https://www.isrctn.com/ISRCTN16347615*](https://www.isrctn.com/ISRCTN16347615) |
| *Enrolment status* | *Completed* |
| *Study design* | *Observational prospective cohort study* |
| *Country* | *Philippines* |
| *Participants* | *Pulmonary TB patients with DM* |
| *Eligibility criteria* | *Inclusion Criteria:*  *Aged 18 or more (adults) who are initiating a new TB treatment regimen*  *Exclusion Criteria:*  *1. Pregnant woman 2. Plan to move away from the study site or do not give consent to participate 3. Started the current ATT regimen more than 5 days before enrolment 4. Currently imprisoned 5. Severe medical or psychiatric disorder which in the opinion of the local investigators might interfere with the ability to give true informed consent or to adhere to the study requirements 6. Taking part in any investigational product trials related to TB and/or lung disease or diabetes.* |
| *Exposure* | *Diabetes – primary exposure will be diabetes present at any one time point during treatment (HbA1c >= 6.5% or on recognized drug treatment for diabetes), secondary will be HbA1C >= 7% at 2 or more time points or on recognized drug treatment for diabetes.*  *Exploratory analysis will assess the effect of degree of hyperglycemia during treatment on TB treatment outcomes* |
| *Outcome* | *Adverse TB treatment outcome at end of study defined as death, loss to follow-up, default (two or more consecutive months of interrupted treatment) or treatment failure.*  *Adverse treatment outcome in the secondary outcome also includes relapse/ recurrent active TB diagnosed clinically or bacteriologically confirmed within 2 years of completing treatment.* |
| *Published* | ***Patterns of non-communicable comorbidities at start of tuberculosis treatment in three regions of the Philippines: The St-ATT cohort (2021)-*** *we excluded this paper in Tiab screening*  ***How can tuberculosis services better support patients with a diabetes co-morbidity? A mixed methods study in the Philippines-*** *we included this paper. Excluded during full text screening.*  ***Glycemic control during TB treatment among Filipinos: The Starting Anti-Tuberculosis Treatment Cohort Study (pub 2024 May)-****included this paper . Excluded during full text screening*  *The authors have data that is not yet published. (reply by authors)*  *Based on the preliminary data provided by the authors, we could conclude that both the analysis of our interest was restricted by sample size.*  *The authors do have some data on drug use during treatment that is yet to be finalized.* |

1. ***Study ID: TCTR20190128004***

| *Title* | *Effect of metformin on sputum smear conversion in pulmonary tuberculosis with type 2 diabetes mellitus** |
| --- | --- |
| *Principal Investigator / contacts* | *Kanyanut Kasiansin*  [*k.kasiansin@gmail.com*](mailto:k.kasiansin@gmail.com) |
| *Weblink* | [*https://www.thaiclinicaltrials.org/show/TCTR20190128004*](https://www.thaiclinicaltrials.org/show/TCTR20190128004) |
| *Enrolment status* | *Pending (Not yet recruiting)* |
| *Study design* | *Observational* |
| *Country* | *Thailand* |
| *Participants* | *Pulmonary TB patients with DM* |
| *Eligibility criteria* | *Inclusion Criteria:*  *Patients who are newly diagnosed pulmonary tuberculosis*  *Patients who diagnosed type 2 diabetes mellitus*  *Exclusion Criteria:*  *There is no exclusion criteria in this study* |
| *Exposure* | *Patients who are newly diagnosed pulmonary tuberculosis and diagnosed type 2 diabetes mellitus which are exposed to metformin along period of study, Patients who are newly diagnosed pulmonary tuberculosis and diagnosed type 2 diabetes mellitus which are not exposed to metformin along period of study* |
| *Outcome* | *Sputum smear (AFB) conversion at the end of 2, 4, 8 and 12 weeks after anti-TB treatment event rate*  *Doses of metformin that are associated with sputum smear conversion at the end of 2, 4, 8 and 12 weeks after anti-TB treatment event rate of sputum smear conversion at each dose, adverse events, hospital admission from any causes, rate of multi-drug resistant tuberculosis.* |

**we hope to get effect of DM treatment on TB treatment outcomes from this study*

1. ***Study ID: TCTR20190128004***

| *Title* | *Study on optimizing blood glucose intervention strategy for improving treatment outcome of patients with drug-susceptible pulmonary tuberculosis and diabetes** |
| --- | --- |
| *Principal Investigator / contacts* | *Peize Zhang*  *82880246@qq.com* |
| *Weblink* | <https://www.chictr.org.cn/showproj.html?proj=230648> |
| *Enrolment status* | *Pending* |
| *Study design* | *Interventional study (single arm)* |
| *Country* | *China* |
| *Participants* | *Pulmonary TB patients with DM* |
| *Eligibility criteria* | *Inclusion Criteria:*  *Male or female age>18 years old; Confirmed drug-susceptible pulmonary tuberculosis through molecular and/or phenotypic drug susceptible test;  Confirmed type 2 diabetes according to guideline; Voluntarily participate in this study, agree to wear a continuous glucose monitoring system for a total of 2 months during the strengthening period, and regular follow up until anti-TB treatment completion*  *Exclusion Criteria:*  *Individuals with severe skin allergies or other conditions who are unable to wear a continuous glucose monitoring system; Drug-resistant tuberculosis patients; Other situations evaluated by the researcher and deemed unsuitable for participation in this study* |
| *Exposure* | *Experimental group: Wearing CGM* |
| *Outcome* | *The relationship between TIR and sputum negative conversion at the end of 2 months and the treatment outcome of pulmonary tuberculosis*  *The incidence of adverse events* |

**we hope to get effect of glycemic control status on TB treatment outcomes from this study.*

*Studies in italics are from updated review (26 April 2017 to 31 Aug 2024)*
