# Supplementary material for: Effect of glycemic control and type of diabetes treatment on TB treatment outcomes among people with TB-diabetes: A systematic review (updated August 2024)
Source: PLoS One. 2025 Jul 18;20(7):e0328619. doi: 10.1371/journal.pone.0328619 (PMC12273911; doi:10.1371/journal.pone.0328619)

**Search 1: Effect of glycemic control on TB treatment outcomes among TB-DM patients(RESEARCH QUESTION 1)**

| Search | Query | Items found |
| --- | --- | --- |
| 1 | Search tuberculosis |  |
| 2 | Search "tuberculosis"[MeSH Terms] |  |
| 3 | Search "treatment outcome$" |  |
| 4 | Search "Treatment Outcome"[MeSH Terms] |  |
| 5 | Search treatment |  |
| 6 | Search outcome |  |
| 7 | Search "diabetes mellitus"[MeSH Terms] |  |
| 8 | Search DM |  |
| 9 | Search diabetes |  |
| 10 | Search mellitus |  |
| 11 | 1 OR 2 |  |
| 12 | 3 OR 4 OR 5 0R 6 |  |
| 13 | 7 OR 8 OR 9 OR 10 |  |
| 14 | 11 AND 12 AND 13 |  |
| 15 | Search “cohort studies”[MeSH Terms] |  |
| 16 | Search cohort |  |
| 17 | 15 OR 16 |  |
| 18 | 14 AND 17 Filters: Humans |  |
| 19 | ("trial$"[All Fields] OR ("randomized controlled trial"[Publication Type] OR "randomized controlled trials as topic"[MeSH Terms] OR "randomized controlled trial"[All Fields])) |  |
| 20 | 14 AND 19 Filters: Humans |  |

**Search 2: Effect of glucose lowering treatment on TB treatment outcomes among TB-DM patients(RESEARCH QUESTION 2)**

The search will consist of a merge of the below 3 searches:

Search 1:

1_: ((TUBERCULOSES OR (KOCHS ADJ DISEASE$1)

OR TUBERCULAR OR MTB OR ANTITUBERCULAR)

OR (KOCH ADJ S ADJ DISEASE$1) OR

TUBERCULOSIS).TI,AB.

2_: (LYXUMIA OR SEMAGLUTIDE OR BYDUREON OR

DULAGLUTIDE OR VICTOZA OR ALBIGLUTIDE

OR BYETTA OR TASPOGLUTIDE OR

LIXISENATIDE OR LIRAGLUTIDE OR

EXENATIDE OR EXENDIN-4).TI.

3_: (((((GLPI OR GLP1 OR INCRETIN$1) OR

(GLP ADJ "1")) OR (GLP ADJ I)) OR

(GLUCAGON ADJ LIKE ADJ PEPTIDE ADJ

"1")) OR (GLUCAGON ADJ LIKE ADJ PEPTIDE

ADJ I)).TI.

4_: ((NN5401 OR NN1250 OR RYZODEG OR

TRESIBA OR IDEGASP OR NN304 OR LEVEMIR

OR IDEG OR DEGLUDEC OR LANTUS OR

DETEMIR) OR (LISPRO NEAR PROTAMINE NEAR

SUSPENSION) OR GLARGINE).TI.

5_: ((APIDRA OR NOVOLOG OR NOVORAPID OR

HUMALOG OR GLULISINE OR ASPART OR

LISPRO) OR ((LONG-ACTING OR (LONG ADJ

ACTING) OR BASAL) ADJ INSULIN)).TI.

6_: (((((RAPID-ACTING OR FAST-ACTING OR

SHORT-ACTING OR PRANDIAL OR BOLUS) OR

(FAST ADJ ACTING)) OR (RAPID ADJ

ACTING)) OR (SHORT ADJ ACTING)) ADJ

INSULIN).TI.

7_: ((((((NOVOMIX OR (NOVOLOG ADJ MIX)) OR

(HUMALOG ADJ MIX)) OR ((BIPHASIC ADJ

INSULIN) ADJ ASPART)) OR ((BIPHASIC ADJ

INSULIN) ADJ LISPRO)) OR (((DUAL-ACTING

OR PREMIX$ OR BIPHASIC) OR (DUAL ADJ

ACTING)) ADJ INSULIN)) OR (INSULIN ADJ

ANALOG$)).TI.

8_: ((((ULTRAPHANE OR VELASULIN OR BIOHULIN

OR PROTAPHAN$ OR VELOSULIN OR

INSULATARD OR NOVOLIN OR ACTRAPID) OR

((NEUTRAL ADJ PROTAMINE ADJ HAGEDORN)

NEAR2 INSULIN)) OR (NPH NEAR2 INSULIN))

OR (HUMAN ADJ INSULIN)).TI.

9_: ((PENMIX OR NOVOLET OR ACTRAPHANE OR

ULTRATARD OR MIXTARD OR NOVOLIN OR

MONOTARD) OR ((INTERMEDIATE ADJ ACTING)

ADJ1 INSULIN)).TI.

10_: (AFREZZA OR AERX OR EXUBERA).TI.

11_: (SGLT2 ADJ INHIBITOR$1) OR ((((SODIUM

ADJ GLUCOSE) ADJ (COTRANSPORTER OR (CO

ADJ TRANSPORTER))) ADJ "2" ADJ

INHIBITOR$1).TI.)

12_: (SUGLAT OR IVOKANA OR LIPAGLYN OR

FORXIGA OR SAROGLITAZAR OR

LUSEOGLIFLOZIN OR ERTUGLIFLOZIN OR

TOFOGLIFLOZIN OR REMOGLIFLOZIN OR

SERGLIFLOZIN OR IPRAGLIFLOZIN OR

EMPAGLIFLOZIN OR CANAGLIFLOZIN OR

DAPAGLIFLOZIN).TI.

13_: (((ALEGLITAZAR OR TESAGLITAZAR OR

MURAGLITAZAR) OR ((PEROXISOME ADJ

PROLIFERATOR) ADJ ACTIVATED ADJ

RECEPTOR ADJ AGONIST$1)) OR (PPAR ADJ

AGONIST$1)).TI.

14_: ((VOGLIB OR GLYSET OR GLUCOBAY OR

VOGLIBOSE OR MIGLITOL OR ACARBOSE) OR

((ALPHA ADJ GLUCOSIDASE) ADJ

INHIBITOR$1)).TI.

15_: (GLUFAST OR STARLIX OR MEGLITINIDES OR

MITIGLINIDE OR NATEGLINIDE).TI.

16_: (METFORMIN NEAR5 DIABETES).TI.

17_: (BIGUANIDE$1 OR THIAZOLIDINEDIONE$1 OR

SULFONYLUREA$1).TI.

18_: (RESULIN OR ROMOZIN OR NOSCAL OR

RIVOGLITAZONE OR REZULIN OR ACTOS OR

AVANDIA OR TROGLITAZONE OR PIOGLITAZONE

OR ROSIGLITAZONE).TI.

19_: (GLUCIDORAL OR DYMELOR OR METAHEXAMIDE

OR ORINASE OR GLUCOTROL OR DIAMICRON OR

CARBUTAMIDE OR TOLAZAMIDE OR

ACETOHEXAMIDE OR CHLORPROPAMIDE OR

GLIPIZIDE OR GLICLAZIDE OR

TOLBUTAMIDE).TI.

20_: ((DEAMELIN ADJ S) OR GLYCLOPYRAMIDE OR

GLYNASE OR MICRONASE OR GLURENORM OR

EUGLUCON OR AMARYL OR DAONIL OR

GLISOXEPIDE OR DIABETA OR GLIQUIDONE OR

GLIMEPIRIDE OR GLYBURIDE OR

GLIBENCLAMIDE).TI.

21_: (((SESTRINE OR EUREPA OR GLUCONORM OR

NOVONORM OR PRANDIN OR REPAGLINIDE) OR

(GLUCO ADJ NORM)) OR (NOVO ADJ

NORM)).TI.

22_: ((((GLIPTIN$1 OR (DPP ADJ (IV OR "4")

ADJ I)) OR (DPP ADJ (IV OR "4") ADJ

INHIBITOR$1)) OR ((DIPEPTIDYL ADJ

PEPTIDASE) ADJ (IV OR "4") ADJ

INHIBITOR$1)) OR ((DIPEPTIDYL ADJ

PEPTIDASE) ADJ (IV OR "4") ADJ I)).TI.

23_: (VIPIDIA OR SYR-472 OR TRELAGLIPTIN OR

OMARIGLIPTIN OR TRAZENTA OR TRAJENTA OR

TRADJENTA OR BI-1356 OR NESINA OR

ONGLYZA OR JANUVIA OR ALOGLIPTIN OR

LINAGLIPTIN OR SAXAGLIPTIN OR

SITAGLIPTIN).TI.

24_: (BESKOA OR SUINY OR ZEMIGLO OR TENELIA

OR GEMIGLIPTIN OR TENELIGLIPTIN OR

GALVUS OR ANAGLIPTIN OR

VILDAGLIPTIN).TI.

25_: 9 OR 10 OR 12 OR 6 OR 13 OR 24 OR 15 OR

21 OR 11 OR 16 OR 22 OR 23 OR 7 OR 4 OR

2 OR 14 OR 5 OR 8 OR 20 OR 19 OR 17 OR

3 OR 18

26_: 25 AND 1

Search 2:

1_: ((TUBERCULOSES OR (KOCHS ADJ DISEASE$1)

OR TUBERCULAR OR MTB OR ANTITUBERCULAR)

OR (KOCH ADJ S ADJ DISEASE$1) OR

TUBERCULOSIS).TI,AB.

2_: LYXUMIA OR SEMAGLUTIDE OR BYDUREON OR

DULAGLUTIDE OR VICTOZA OR ALBIGLUTIDE

OR BYETTA OR TASPOGLUTIDE OR

LIXISENATIDE OR LIRAGLUTIDE OR

EXENATIDE OR EXENDIN-4

3_: ((((GLPI OR GLP1 OR INCRETIN$1) OR (GLP

ADJ "1")) OR (GLP ADJ I)) OR (GLUCAGON

ADJ LIKE ADJ PEPTIDE ADJ "1")) OR

(GLUCAGON ADJ LIKE ADJ PEPTIDE ADJ I)

4_: (NN5401 OR NN1250 OR RYZODEG OR TRESIBA

OR IDEGASP OR NN304 OR LEVEMIR OR IDEG

OR DEGLUDEC OR LANTUS OR DETEMIR) OR

(LISPRO NEAR PROTAMINE NEAR SUSPENSION)

OR GLARGINE

5_: (APIDRA OR NOVOLOG OR NOVORAPID OR

HUMALOG OR GLULISINE OR ASPART OR

LISPRO) OR ((LONG-ACTING OR (LONG ADJ

ACTING) OR BASAL) ADJ INSULIN)

6_: ((((RAPID-ACTING OR FAST-ACTING OR

SHORT-ACTING OR PRANDIAL OR BOLUS) OR

(FAST ADJ ACTING)) OR (RAPID ADJ

ACTING)) OR (SHORT ADJ ACTING)) ADJ

INSULIN

7_: (((((NOVOMIX OR (NOVOLOG ADJ MIX)) OR

(HUMALOG ADJ MIX)) OR ((BIPHASIC ADJ

INSULIN) ADJ ASPART)) OR ((BIPHASIC ADJ

INSULIN) ADJ LISPRO)) OR (((DUAL-ACTING

OR PREMIX$ OR BIPHASIC) OR (DUAL ADJ

ACTING)) ADJ INSULIN)) OR (INSULIN ADJ

ANALOG$)

8_: (((ULTRAPHANE OR VELASULIN OR BIOHULIN

OR PROTAPHAN$ OR VELOSULIN OR

INSULATARD OR NOVOLIN OR ACTRAPID) OR

((NEUTRAL ADJ PROTAMINE ADJ HAGEDORN)

NEAR2 INSULIN)) OR (NPH NEAR2 INSULIN))

OR (HUMAN ADJ INSULIN)

9_: (PENMIX OR NOVOLET OR ACTRAPHANE OR

ULTRATARD OR MIXTARD OR NOVOLIN OR

MONOTARD) OR ((INTERMEDIATE ADJ ACTING)

ADJ1 INSULIN)

10_: AFREZZA OR AERX OR EXUBERA

11_: (SGLT2 ADJ INHIBITOR$1) OR (((SODIUM

ADJ GLUCOSE) ADJ (COTRANSPORTER OR (CO

ADJ TRANSPORTER))) ADJ "2" ADJ

INHIBITOR$1)

12_: SUGLAT OR IVOKANA OR LIPAGLYN OR

FORXIGA OR SAROGLITAZAR OR

LUSEOGLIFLOZIN OR ERTUGLIFLOZIN OR

TOFOGLIFLOZIN OR REMOGLIFLOZIN OR

SERGLIFLOZIN OR IPRAGLIFLOZIN OR

EMPAGLIFLOZIN OR CANAGLIFLOZIN OR

DAPAGLIFLOZIN

13_: ((ALEGLITAZAR OR TESAGLITAZAR OR

MURAGLITAZAR) OR ((PEROXISOME ADJ

PROLIFERATOR) ADJ ACTIVATED ADJ

RECEPTOR ADJ AGONIST$1)) OR (PPAR ADJ

AGONIST$1)

14_: (VOGLIB OR GLYSET OR GLUCOBAY OR

VOGLIBOSE OR MIGLITOL OR ACARBOSE) OR

((ALPHA ADJ GLUCOSIDASE) ADJ

INHIBITOR$1)

15_: GLUFAST OR STARLIX OR MEGLITINIDES OR

MITIGLINIDE OR NATEGLINIDE

16_: METFORMIN NEAR5 DIABETES

17_: BIGUANIDE$1 OR THIAZOLIDINEDIONE$1 OR

SULFONYLUREA$1

18_: RESULIN OR ROMOZIN OR NOSCAL OR

RIVOGLITAZONE OR REZULIN OR ACTOS OR

AVANDIA OR TROGLITAZONE OR PIOGLITAZONE

OR ROSIGLITAZONE

19_: GLUCIDORAL OR DYMELOR OR METAHEXAMIDE

OR ORINASE OR GLUCOTROL OR DIAMICRON OR

CARBUTAMIDE OR TOLAZAMIDE OR

ACETOHEXAMIDE OR CHLORPROPAMIDE OR

GLIPIZIDE OR GLICLAZIDE OR TOLBUTAMIDE

20_: (DEAMELIN ADJ S) OR GLYCLOPYRAMIDE OR

GLYNASE OR MICRONASE OR GLURENORM OR

EUGLUCON OR AMARYL OR DAONIL OR

GLISOXEPIDE OR DIABETA OR GLIQUIDONE OR

GLIMEPIRIDE OR GLYBURIDE OR

GLIBENCLAMIDE

21_: ((SESTRINE OR EUREPA OR GLUCONORM OR

NOVONORM OR PRANDIN OR REPAGLINIDE) OR

(GLUCO ADJ NORM)) OR (NOVO ADJ NORM)

22_: (((GLIPTIN$1 OR (DPP ADJ (IV OR "4")

ADJ I)) OR (DPP ADJ (IV OR "4") ADJ

INHIBITOR$1)) OR ((DIPEPTIDYL ADJ

PEPTIDASE) ADJ (IV OR "4") ADJ

INHIBITOR$1)) OR ((DIPEPTIDYL ADJ

PEPTIDASE) ADJ (IV OR "4") ADJ I)

23_: VIPIDIA OR SYR-472 OR TRELAGLIPTIN OR

OMARIGLIPTIN OR TRAZENTA OR TRAJENTA OR

TRADJENTA OR BI-1356 OR NESINA OR

ONGLYZA OR JANUVIA OR ALOGLIPTIN OR

LINAGLIPTIN OR SAXAGLIPTIN OR

SITAGLIPTIN

24_: BESKOA OR SUINY OR ZEMIGLO OR TENELIA

OR GEMIGLIPTIN OR TENELIGLIPTIN OR

GALVUS OR ANAGLIPTIN OR VILDAGLIPTIN

25_: 10 OR 11 OR 9 OR 16 OR 6 OR 12 OR 13 OR

24 OR 15 OR 21 OR 22 OR 7 OR 23 OR 4 OR

14 OR 2 OR 8 OR 5 OR 19 OR 20 OR 17 OR

3 OR 18

26_: (BENEFIT$1 OR IMPROVE$1 OR OUTCOME$1 OR

RESULT$1 OR EFFECT$1) NEAR10 (TAKING OR

MEDICAT$3 OR CARE OR TREATMENT$1 OR

CONTROL$3 OR THERAP$3)

27_: 26 NEAR20 ((TUBERCULOSES OR (KOCHS ADJ

DISEASE$1) OR TUBERCULAR OR MTB OR

ANTITUBERCULAR) OR (KOCH ADJ S ADJ

DISEASE$1) OR TUBERCULOSIS OR DIABETES)

28_: ((TUBERCULOSES OR (KOCHS ADJ DISEASE$1)

OR TUBERCULAR OR MTB OR ANTITUBERCULAR)

OR (KOCH ADJ S ADJ DISEASE$1) OR

TUBERCULOSIS OR DIABETES) NEAR10

(CONVERGENCE OR EPIDEMIC$1 OR

PREVALENCE$1 OR INCIDENCE$1 OR

EPIDEMIOLOGY OR RISK$1)

29_: (27 OR 28) AND 1 AND 25

Search 3:

1_: ((TUBERCULOSES OR (KOCHS ADJ DISEASE$1)

OR MTB) OR (KOCH ADJ S ADJ DISEASE$1)

OR TUBERCULOSIS).TI,AB.

2_: (LYXUMIA OR SEMAGLUTIDE OR BYDUREON OR

DULAGLUTIDE OR VICTOZA OR ALBIGLUTIDE

OR BYETTA OR TASPOGLUTIDE OR

LIXISENATIDE OR LIRAGLUTIDE OR

EXENATIDE OR EXENDIN-4).TI,AB.

3_: (((((GLPI OR GLP1 OR INCRETIN$1) OR

(GLP ADJ "1")) OR (GLP ADJ I)) OR

(GLUCAGON ADJ LIKE ADJ PEPTIDE ADJ

"1")) OR (GLUCAGON ADJ LIKE ADJ PEPTIDE

ADJ I)).TI,AB.

4_: (NN5401 OR NN1250 OR RYZODEG OR TRESIBA

OR IDEGASP OR NN304 OR LEVEMIR OR IDEG

OR DEGLUDEC OR LANTUS OR DETEMIR) OR

(LISPRO NEAR PROTAMINE NEAR SUSPENSION)

OR GLARGINE

5_: (APIDRA OR NOVOLOG OR NOVORAPID OR

HUMALOG OR GLULISINE OR ASPART OR

LISPRO) OR ((LONG-ACTING OR (LONG ADJ

ACTING) OR BASAL) ADJ INSULIN)

6_: ((((RAPID-ACTING OR FAST-ACTING OR

SHORT-ACTING OR PRANDIAL OR BOLUS) OR

(FAST ADJ ACTING)) OR (RAPID ADJ

ACTING)) OR (SHORT ADJ ACTING)) ADJ

INSULIN

7_: (((((NOVOMIX OR (NOVOLOG ADJ MIX)) OR

(HUMALOG ADJ MIX)) OR ((BIPHASIC ADJ

INSULIN) ADJ ASPART)) OR ((BIPHASIC ADJ

INSULIN) ADJ LISPRO)) OR (((DUAL-ACTING

OR PREMIX$ OR BIPHASIC) OR (DUAL ADJ

ACTING)) ADJ INSULIN)) OR (INSULIN ADJ

ANALOG$)

8_: (((ULTRAPHANE OR VELASULIN OR BIOHULIN

OR PROTAPHAN$ OR VELOSULIN OR

INSULATARD OR NOVOLIN OR ACTRAPID) OR

((NEUTRAL ADJ PROTAMINE ADJ HAGEDORN)

NEAR2 INSULIN)) OR (NPH NEAR2 INSULIN))

OR (HUMAN ADJ INSULIN)

9_: (PENMIX OR NOVOLET OR ACTRAPHANE OR

ULTRATARD OR MIXTARD OR NOVOLIN OR

MONOTARD) OR ((INTERMEDIATE ADJ ACTING)

ADJ1 INSULIN)

10_: AFREZZA OR AERX OR EXUBERA

11_: (SGLT2 ADJ INHIBITOR$1) OR ((((SODIUM

ADJ GLUCOSE) ADJ (COTRANSPORTER OR (CO

ADJ TRANSPORTER))) ADJ "2" ADJ

INHIBITOR$1).TI,AB.)

12_: (SUGLAT OR IVOKANA OR LIPAGLYN OR

FORXIGA OR SAROGLITAZAR OR

LUSEOGLIFLOZIN OR ERTUGLIFLOZIN OR

TOFOGLIFLOZIN OR REMOGLIFLOZIN OR

SERGLIFLOZIN OR IPRAGLIFLOZIN OR

EMPAGLIFLOZIN OR CANAGLIFLOZIN OR

DAPAGLIFLOZIN).TI,AB.

13_: (((ALEGLITAZAR OR TESAGLITAZAR OR

MURAGLITAZAR) OR ((PEROXISOME ADJ

PROLIFERATOR) ADJ ACTIVATED ADJ

RECEPTOR ADJ AGONIST$1)) OR (PPAR ADJ

AGONIST$1)).TI,AB.

14_: ((VOGLIB OR GLYSET OR GLUCOBAY OR

VOGLIBOSE OR MIGLITOL OR ACARBOSE) OR

((ALPHA ADJ GLUCOSIDASE) ADJ

INHIBITOR$1)).TI,AB.

15_: GLUFAST OR STARLIX OR MEGLITINIDES OR

MITIGLINIDE OR NATEGLINIDE

16_: (METFORMIN NEAR5 DIABETES).TI,AB.

17_: (BIGUANIDE$1 OR THIAZOLIDINEDIONE$1 OR

SULFONYLUREA$1).TI,AB.

18_: (RESULIN OR ROMOZIN OR NOSCAL OR

RIVOGLITAZONE OR REZULIN OR ACTOS OR

AVANDIA OR TROGLITAZONE OR PIOGLITAZONE

OR ROSIGLITAZONE).TI,AB.

19_: (GLUCIDORAL OR DYMELOR OR METAHEXAMIDE

OR ORINASE OR GLUCOTROL OR DIAMICRON OR

CARBUTAMIDE OR TOLAZAMIDE OR

ACETOHEXAMIDE OR CHLORPROPAMIDE OR

GLIPIZIDE OR GLICLAZIDE OR

TOLBUTAMIDE).TI,AB.

20_: ((DEAMELIN ADJ S) OR GLYCLOPYRAMIDE OR

GLYNASE OR MICRONASE OR GLURENORM OR

EUGLUCON OR AMARYL OR DAONIL OR

GLISOXEPIDE OR DIABETA OR GLIQUIDONE OR

GLIMEPIRIDE OR GLYBURIDE OR

GLIBENCLAMIDE).TI,AB.

21_: (((SESTRINE OR EUREPA OR GLUCONORM OR

NOVONORM OR PRANDIN OR REPAGLINIDE) OR

(GLUCO ADJ NORM)) OR (NOVO ADJ

NORM)).TI,AB.

22_: ((((GLIPTIN$1 OR (DPP ADJ (IV OR "4")

ADJ I)) OR (DPP ADJ (IV OR "4") ADJ

INHIBITOR$1)) OR ((DIPEPTIDYL ADJ

PEPTIDASE) ADJ (IV OR "4") ADJ

INHIBITOR$1)) OR ((DIPEPTIDYL ADJ

PEPTIDASE) ADJ (IV OR "4") ADJ

I)).TI,AB.

23_: (VIPIDIA OR SYR-472 OR TRELAGLIPTIN OR

OMARIGLIPTIN OR TRAZENTA OR TRAJENTA OR

TRADJENTA OR BI-1356 OR NESINA OR

ONGLYZA OR JANUVIA OR ALOGLIPTIN OR

LINAGLIPTIN OR SAXAGLIPTIN OR

SITAGLIPTIN).TI,AB.

24_: (BESKOA OR SUINY OR ZEMIGLO OR TENELIA

OR GEMIGLIPTIN OR TENELIGLIPTIN OR

GALVUS OR ANAGLIPTIN OR

VILDAGLIPTIN).TI,AB.

25_: 11 OR 10 OR 9 OR 12 OR 13 OR 16 OR 24

OR 21 OR 6 OR 22 OR 15 OR 23 OR 7 OR 14

OR 2 OR 19 OR 4 OR 17 OR 8 OR 5 OR 20

OR 18 OR 3

26_: (TUBERCULOSIS OR DIABETES) NEAR10

(CONVERGENCE OR EPIDEMIC$1 OR

PREVALENCE$1 OR INCIDENCE$1 OR

EPIDEMIOLOGY OR RISK$1)

27_: 26 AND 1 AND 25


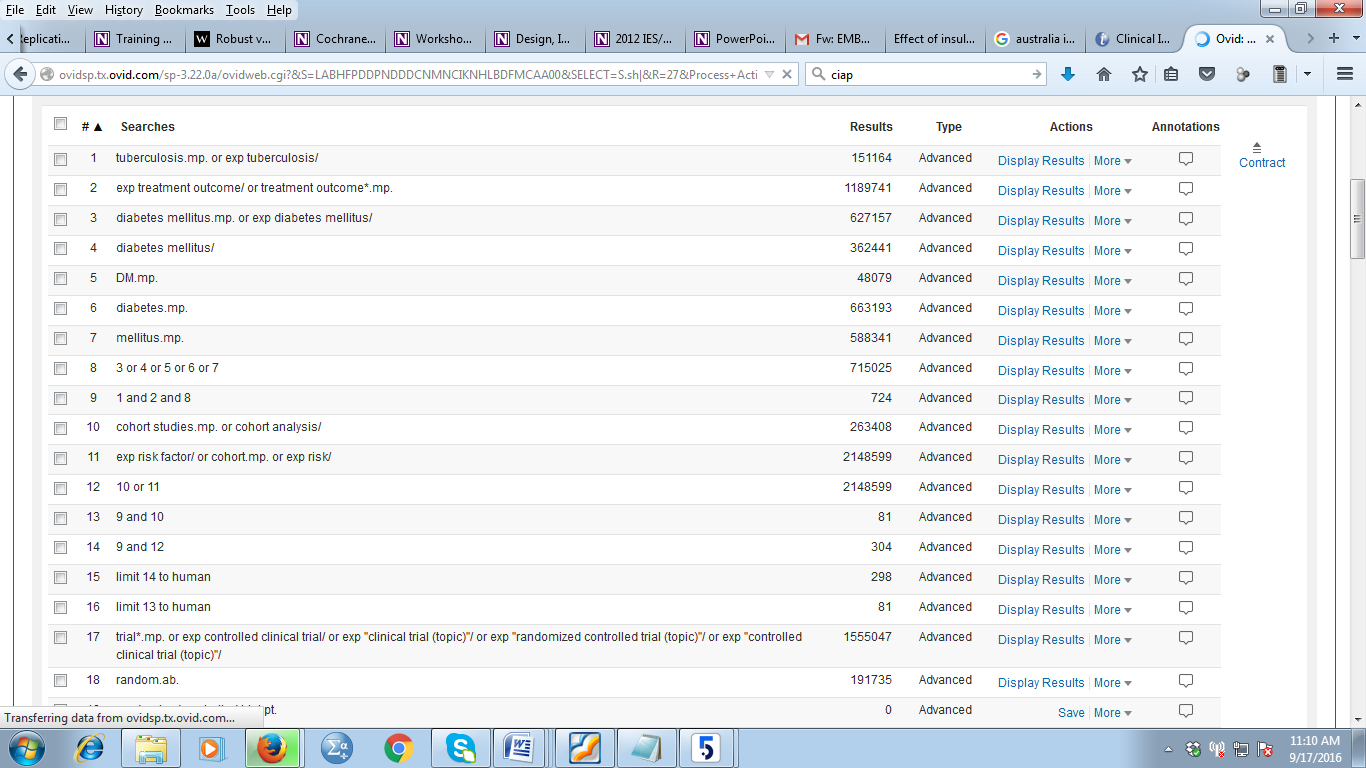


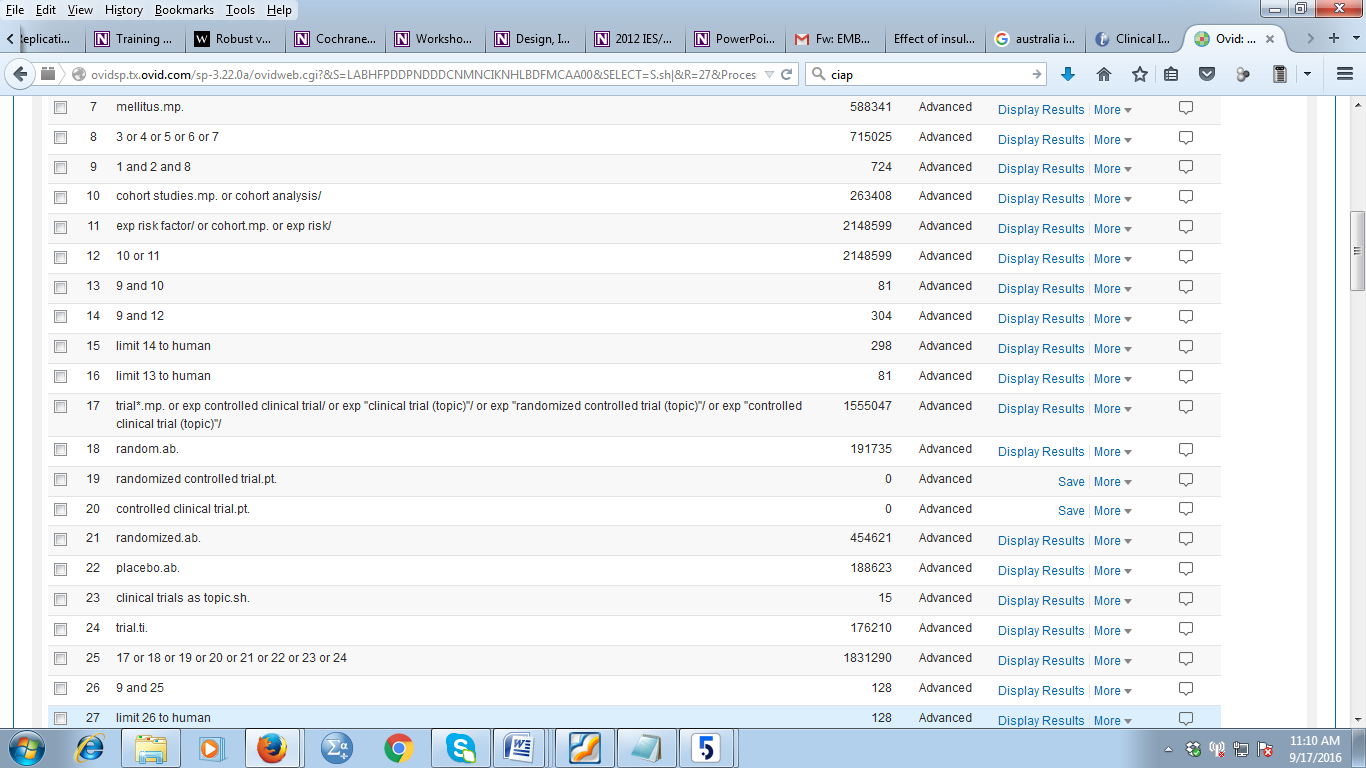

Supplement: S1 Appendix — (ZIP) [file pone.0328619.s004.zip › S1 appendix_old/EMBASE/Embase search strategy.docx]
