# Supplementary material for: Effect of glycemic control and type of diabetes treatment on TB treatment outcomes among people with TB-diabetes: A systematic review (updated August 2024)
Source: PLoS One. 2025 Jul 18;20(7):e0328619. doi: 10.1371/journal.pone.0328619 (PMC12273911; doi:10.1371/journal.pone.0328619)

| 1 | tuberculosis.mp. or exp tuberculosis/ | 291433 | Advanced | [Display Results](http://ovidsp.tx.ovid.com.abc.cardiff.ac.uk/sp-3.24.1b/ovidweb.cgi?&S=GABDFPGMPDDDBANGNCHKPCOBJDKGAA00&SELECT=S.sh%7c&R=1&Process+Action=display)  [More](http://ovidsp.tx.ovid.com.abc.cardiff.ac.uk/sp-3.24.1b/ovidweb.cgi) |  |  |
| --- | --- | --- | --- | --- | --- | --- |
|  | 2 | exp treatment outcome/ or treatment outcome*.mp. | 1289437 | Advanced | [Display Results](http://ovidsp.tx.ovid.com.abc.cardiff.ac.uk/sp-3.24.1b/ovidweb.cgi?&S=GABDFPGMPDDDBANGNCHKPCOBJDKGAA00&SELECT=S.sh%7c&R=2&Process+Action=display)  [More](http://ovidsp.tx.ovid.com.abc.cardiff.ac.uk/sp-3.24.1b/ovidweb.cgi) |  |
|  | 3 | diabetes mellitus.mp. or exp diabetes mellitus/ | 880018 | Advanced | [Display Results](http://ovidsp.tx.ovid.com.abc.cardiff.ac.uk/sp-3.24.1b/ovidweb.cgi?&S=GABDFPGMPDDDBANGNCHKPCOBJDKGAA00&SELECT=S.sh%7c&R=3&Process+Action=display)  [More](http://ovidsp.tx.ovid.com.abc.cardiff.ac.uk/sp-3.24.1b/ovidweb.cgi) |  |
|  | 4 | diabetes mellitus/ | 599265 | Advanced | [Display Results](http://ovidsp.tx.ovid.com.abc.cardiff.ac.uk/sp-3.24.1b/ovidweb.cgi?&S=GABDFPGMPDDDBANGNCHKPCOBJDKGAA00&SELECT=S.sh%7c&R=4&Process+Action=display)  [More](http://ovidsp.tx.ovid.com.abc.cardiff.ac.uk/sp-3.24.1b/ovidweb.cgi) |  |
|  | 5 | DM.mp. | 234038 | Advanced | [Display Results](http://ovidsp.tx.ovid.com.abc.cardiff.ac.uk/sp-3.24.1b/ovidweb.cgi?&S=GABDFPGMPDDDBANGNCHKPCOBJDKGAA00&SELECT=S.sh%7c&R=5&Process+Action=display)  [More](http://ovidsp.tx.ovid.com.abc.cardiff.ac.uk/sp-3.24.1b/ovidweb.cgi) |  |
|  | 6 | diabetes.mp. | 891272 | Advanced | [Display Results](http://ovidsp.tx.ovid.com.abc.cardiff.ac.uk/sp-3.24.1b/ovidweb.cgi?&S=GABDFPGMPDDDBANGNCHKPCOBJDKGAA00&SELECT=S.sh%7c&R=6&Process+Action=display)  [More](http://ovidsp.tx.ovid.com.abc.cardiff.ac.uk/sp-3.24.1b/ovidweb.cgi) |  |
|  | 7 | mellitus.mp. | 827628 | Advanced | [Display Results](http://ovidsp.tx.ovid.com.abc.cardiff.ac.uk/sp-3.24.1b/ovidweb.cgi?&S=GABDFPGMPDDDBANGNCHKPCOBJDKGAA00&SELECT=S.sh%7c&R=7&Process+Action=display)  [More](http://ovidsp.tx.ovid.com.abc.cardiff.ac.uk/sp-3.24.1b/ovidweb.cgi) |  |
|  | 8 | 3 or 4 or 5 or 6 or 7 | 1125394 | Advanced | [Display Results](http://ovidsp.tx.ovid.com.abc.cardiff.ac.uk/sp-3.24.1b/ovidweb.cgi?&S=GABDFPGMPDDDBANGNCHKPCOBJDKGAA00&SELECT=S.sh%7c&R=8&Process+Action=display)  [More](http://ovidsp.tx.ovid.com.abc.cardiff.ac.uk/sp-3.24.1b/ovidweb.cgi) |  |
|  | 9 | 1 and 2 and 8 | 1560 | Advanced | [Display Results](http://ovidsp.tx.ovid.com.abc.cardiff.ac.uk/sp-3.24.1b/ovidweb.cgi?&S=GABDFPGMPDDDBANGNCHKPCOBJDKGAA00&SELECT=S.sh%7c&R=9&Process+Action=display)  [More](http://ovidsp.tx.ovid.com.abc.cardiff.ac.uk/sp-3.24.1b/ovidweb.cgi) |  |
|  | 10 | cohort studies.mp. or cohort analysis/ | 334934 | Advanced | [Display Results](http://ovidsp.tx.ovid.com.abc.cardiff.ac.uk/sp-3.24.1b/ovidweb.cgi?&S=GABDFPGMPDDDBANGNCHKPCOBJDKGAA00&SELECT=S.sh%7c&R=10&Process+Action=display)  [More](http://ovidsp.tx.ovid.com.abc.cardiff.ac.uk/sp-3.24.1b/ovidweb.cgi) |  |
|  | 11 | exp risk factor/ or cohort.mp. or exp risk/ | 2843815 | Advanced | [Display Results](http://ovidsp.tx.ovid.com.abc.cardiff.ac.uk/sp-3.24.1b/ovidweb.cgi?&S=GABDFPGMPDDDBANGNCHKPCOBJDKGAA00&SELECT=S.sh%7c&R=11&Process+Action=display)  [More](http://ovidsp.tx.ovid.com.abc.cardiff.ac.uk/sp-3.24.1b/ovidweb.cgi) |  |
|  | 12 | 10 or 11 | 2843815 | Advanced | [Display Results](http://ovidsp.tx.ovid.com.abc.cardiff.ac.uk/sp-3.24.1b/ovidweb.cgi?&S=GABDFPGMPDDDBANGNCHKPCOBJDKGAA00&SELECT=S.sh%7c&R=12&Process+Action=display)  [More](http://ovidsp.tx.ovid.com.abc.cardiff.ac.uk/sp-3.24.1b/ovidweb.cgi) |  |
|  | 13 | 9 and 10 | 161 | Advanced | [Display Results](http://ovidsp.tx.ovid.com.abc.cardiff.ac.uk/sp-3.24.1b/ovidweb.cgi?&S=GABDFPGMPDDDBANGNCHKPCOBJDKGAA00&SELECT=S.sh%7c&R=13&Process+Action=display)  [More](http://ovidsp.tx.ovid.com.abc.cardiff.ac.uk/sp-3.24.1b/ovidweb.cgi) |  |
|  | 14 | 9 and 12 | 652 | Advanced | [Display Results](http://ovidsp.tx.ovid.com.abc.cardiff.ac.uk/sp-3.24.1b/ovidweb.cgi?&S=GABDFPGMPDDDBANGNCHKPCOBJDKGAA00&SELECT=S.sh%7c&R=14&Process+Action=display)  [More](http://ovidsp.tx.ovid.com.abc.cardiff.ac.uk/sp-3.24.1b/ovidweb.cgi) |  |
|  | 15 | limit 14 to human | 642 | Advanced | [Display Results](http://ovidsp.tx.ovid.com.abc.cardiff.ac.uk/sp-3.24.1b/ovidweb.cgi?&S=GABDFPGMPDDDBANGNCHKPCOBJDKGAA00&SELECT=S.sh%7c&R=15&Process+Action=display)  [More](http://ovidsp.tx.ovid.com.abc.cardiff.ac.uk/sp-3.24.1b/ovidweb.cgi) |  |
|  | 16 | limit 13 to human | 160 | Advanced | [Display Results](http://ovidsp.tx.ovid.com.abc.cardiff.ac.uk/sp-3.24.1b/ovidweb.cgi?&S=GABDFPGMPDDDBANGNCHKPCOBJDKGAA00&SELECT=S.sh%7c&R=16&Process+Action=display)  [More](http://ovidsp.tx.ovid.com.abc.cardiff.ac.uk/sp-3.24.1b/ovidweb.cgi) |  |
|  | 17 | trial*.mp. or exp controlled clinical trial/ or exp "clinical trial (topic)"/ or exp "randomized controlled trial (topic)"/ or exp "controlled clinical trial (topic)"/ | 2044040 | Advanced | [Display Results](http://ovidsp.tx.ovid.com.abc.cardiff.ac.uk/sp-3.24.1b/ovidweb.cgi?&S=GABDFPGMPDDDBANGNCHKPCOBJDKGAA00&SELECT=S.sh%7c&R=17&Process+Action=display)  [More](http://ovidsp.tx.ovid.com.abc.cardiff.ac.uk/sp-3.24.1b/ovidweb.cgi) |  |
|  | 18 | random.ab. | 253017 | Advanced | [Display Results](http://ovidsp.tx.ovid.com.abc.cardiff.ac.uk/sp-3.24.1b/ovidweb.cgi?&S=GABDFPGMPDDDBANGNCHKPCOBJDKGAA00&SELECT=S.sh%7c&R=18&Process+Action=display)  [More](http://ovidsp.tx.ovid.com.abc.cardiff.ac.uk/sp-3.24.1b/ovidweb.cgi) |  |
|  | 19 | randomized controlled trial.pt. | 0 | Advanced | [Save](http://ovidsp.tx.ovid.com.abc.cardiff.ac.uk/sp-3.24.1b/ovidweb.cgi?&S=GABDFPGMPDDDBANGNCHKPCOBJDKGAA00&SELECT=S.sh%7c&R=19&Process+Action=save)  [More](http://ovidsp.tx.ovid.com.abc.cardiff.ac.uk/sp-3.24.1b/ovidweb.cgi) |  |
|  | 20 | controlled clinical trial.pt. | 0 | Advanced | [Save](http://ovidsp.tx.ovid.com.abc.cardiff.ac.uk/sp-3.24.1b/ovidweb.cgi?&S=GABDFPGMPDDDBANGNCHKPCOBJDKGAA00&SELECT=S.sh%7c&R=20&Process+Action=save)  [More](http://ovidsp.tx.ovid.com.abc.cardiff.ac.uk/sp-3.24.1b/ovidweb.cgi) |  |
|  | 21 | randomized.ab. | 540645 | Advanced | [Display Results](http://ovidsp.tx.ovid.com.abc.cardiff.ac.uk/sp-3.24.1b/ovidweb.cgi?&S=GABDFPGMPDDDBANGNCHKPCOBJDKGAA00&SELECT=S.sh%7c&R=21&Process+Action=display)  [More](http://ovidsp.tx.ovid.com.abc.cardiff.ac.uk/sp-3.24.1b/ovidweb.cgi) |  |
|  | 22 | placebo.ab. | 252710 | Advanced | [Display Results](http://ovidsp.tx.ovid.com.abc.cardiff.ac.uk/sp-3.24.1b/ovidweb.cgi?&S=GABDFPGMPDDDBANGNCHKPCOBJDKGAA00&SELECT=S.sh%7c&R=22&Process+Action=display)  [More](http://ovidsp.tx.ovid.com.abc.cardiff.ac.uk/sp-3.24.1b/ovidweb.cgi) |  |
|  | 23 | clinical trials as topic.sh. | 2 | Advanced | [Display Results](http://ovidsp.tx.ovid.com.abc.cardiff.ac.uk/sp-3.24.1b/ovidweb.cgi?&S=GABDFPGMPDDDBANGNCHKPCOBJDKGAA00&SELECT=S.sh%7c&R=23&Process+Action=display)  [More](http://ovidsp.tx.ovid.com.abc.cardiff.ac.uk/sp-3.24.1b/ovidweb.cgi) |  |
|  | 24 | trial.ti. | 233523 | Advanced | [Display Results](http://ovidsp.tx.ovid.com.abc.cardiff.ac.uk/sp-3.24.1b/ovidweb.cgi?&S=GABDFPGMPDDDBANGNCHKPCOBJDKGAA00&SELECT=S.sh%7c&R=24&Process+Action=display)  [More](http://ovidsp.tx.ovid.com.abc.cardiff.ac.uk/sp-3.24.1b/ovidweb.cgi) |  |
|  | 25 | 17 or 18 or 19 or 20 or 21 or 22 or 23 or 24 | 2406494 | Advanced | [Display Results](http://ovidsp.tx.ovid.com.abc.cardiff.ac.uk/sp-3.24.1b/ovidweb.cgi?&S=GABDFPGMPDDDBANGNCHKPCOBJDKGAA00&SELECT=S.sh%7c&R=25&Process+Action=display)  [More](http://ovidsp.tx.ovid.com.abc.cardiff.ac.uk/sp-3.24.1b/ovidweb.cgi) |  |
|  | 26 | 9 and 25 | 443 | Advanced | [Display Results](http://ovidsp.tx.ovid.com.abc.cardiff.ac.uk/sp-3.24.1b/ovidweb.cgi?&S=GABDFPGMPDDDBANGNCHKPCOBJDKGAA00&SELECT=S.sh%7c&R=26&Process+Action=display)  [More](http://ovidsp.tx.ovid.com.abc.cardiff.ac.uk/sp-3.24.1b/ovidweb.cgi) |  |
|  | 27 | limit 26 to human | 442 | Advanced | [Display Results](http://ovidsp.tx.ovid.com.abc.cardiff.ac.uk/sp-3.24.1b/ovidweb.cgi?&S=GABDFPGMPDDDBANGNCHKPCOBJDKGAA00&SELECT=S.sh%7c&R=27&Process+Action=display)  [More](http://ovidsp.tx.ovid.com.abc.cardiff.ac.uk/sp-3.24.1b/ovidweb.cgi) |  |
|  | 28 | limit 16 to yr="2016 - 2017" | 19 | Advanced | [Display Results](http://ovidsp.tx.ovid.com.abc.cardiff.ac.uk/sp-3.24.1b/ovidweb.cgi?&S=GABDFPGMPDDDBANGNCHKPCOBJDKGAA00&SELECT=S.sh%7c&R=28&Process+Action=display)  [More](http://ovidsp.tx.ovid.com.abc.cardiff.ac.uk/sp-3.24.1b/ovidweb.cgi) |  |
|  | 29 | limit 15 to yr="2016 -Current" | 61 | Advanced | [Display Results](http://ovidsp.tx.ovid.com.abc.cardiff.ac.uk/sp-3.24.1b/ovidweb.cgi?&S=GABDFPGMPDDDBANGNCHKPCOBJDKGAA00&SELECT=S.sh%7c&R=29&Process+Action=display)  [More](http://ovidsp.tx.ovid.com.abc.cardiff.ac.uk/sp-3.24.1b/ovidweb.cgi) |  |
|  | 30 | limit 16 to yr="2016 -Current" | 19 | Advanced | [Display Results](http://ovidsp.tx.ovid.com.abc.cardiff.ac.uk/sp-3.24.1b/ovidweb.cgi?&S=GABDFPGMPDDDBANGNCHKPCOBJDKGAA00&SELECT=S.sh%7c&R=30&Process+Action=display)  [More](http://ovidsp.tx.ovid.com.abc.cardiff.ac.uk/sp-3.24.1b/ovidweb.cgi) |  |
|  | 31 | limit 27 to yr="2016 -Current" | 50 | Advanced | [Display Results](http://ovidsp.tx.ovid.com.abc.cardiff.ac.uk/sp-3.24.1b/ovidweb.cgi?&S=GABDFPGMPDDDBANGNCHKPCOBJDKGAA00&SELECT=S.sh%7c&R=31&Process+Action=display)  [More](http://ovidsp.tx.ovid.com.abc.cardiff.ac.uk/sp-3.24.1b/ovidweb.cgi) |  |
|  | | |  |  |  |  |


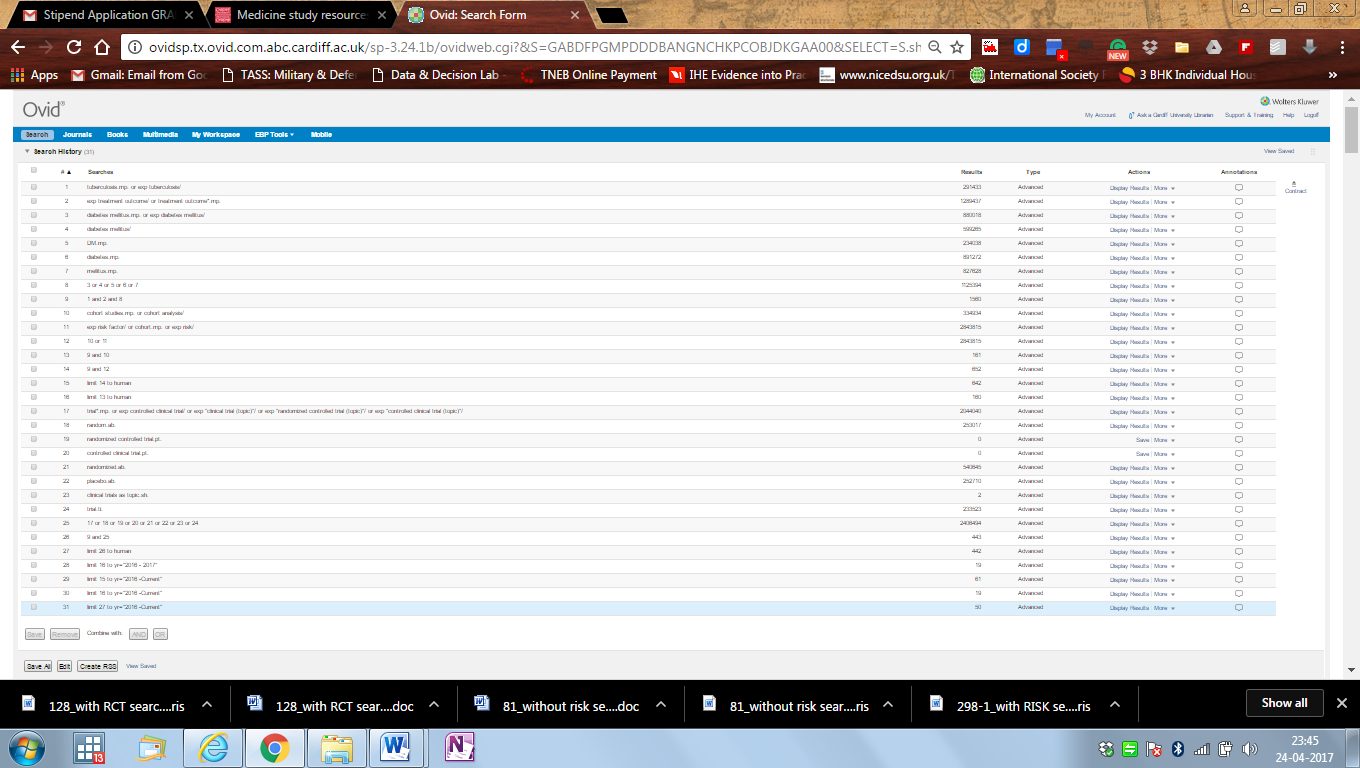

Supplement: S1 Appendix — (ZIP) [file pone.0328619.s004.zip › S1 appendix_old/EMBASE/update Sept 2016 to current 24_04_2017/search method_24_04_2017_tbDM.docx]
