# Supplementary material for: Effect of glycemic control and type of diabetes treatment on TB treatment outcomes among people with TB-diabetes: A systematic review (updated August 2024)
Source: PLoS One. 2025 Jul 18;20(7):e0328619. doi: 10.1371/journal.pone.0328619 (PMC12273911; doi:10.1371/journal.pone.0328619)
Supplement: S1 Appendix — (ZIP) [file pone.0328619.s004.zip › S1 appendix_old/PubMed/~WRL1691.tmp]

TB-DM SR - RCTs

(tuberculosis OR tuberculosis[MeSH]) AND (treatment outcome[MeSH] OR "treatment outcome*" OR treatment outcome) AND (mellitus OR diabetes OR DM OR diabetes mellitus[MeSH] OR diabetes mellitus) AND ("trial$"[All Fields] OR "randomized controlled trial"[Publication Type] OR "randomized controlled trials as topic"[MeSH Terms] OR "randomized controlled trial"[All Fields]) AND Human[MeSH]

TB-DM Sr – Cohort studies

(tuberculosis OR tuberculosis[MeSH]) AND (treatment outcome[MeSH] OR "treatment outcome*" OR treatment outcome) AND (mellitus OR diabetes OR DM OR diabetes mellitus[MeSH] OR diabetes mellitus) AND ("risk factors"[MeSH Terms] OR risk factor[Text Word] OR cohort OR risk[MeSH] OR cohort studies OR cohort study OR cohort analysis) AND Human[MeSH]
