# Supplementary material for: Effect of glycemic control and type of diabetes treatment on TB treatment outcomes among people with TB-diabetes: A systematic review (updated August 2024)
Source: PLoS One. 2025 Jul 18;20(7):e0328619. doi: 10.1371/journal.pone.0328619 (PMC12273911; doi:10.1371/journal.pone.0328619)

**Effect of glycemic control on TB treatment outcomes among TB-DM patients (RCT)**

| Search | Query | Items found (Search conducted on 1^st^ Sept 2023) |
| --- | --- | --- |
| 1 | Search tuberculosis | 394883 |
| 2 | Search "tuberculosis"[MeSH Terms] | 299398 |
| 3 | Search "treatment outcome$" | 1927067 |
| 4 | Search "Treatment Outcome"[MeSH Terms] | 2304859 |
| 5 | Search treatment | 8725179 |
| 6 | Search outcome | 33667078 |
| 7 | Search "diabetes mellitus"[MeSH Terms] | 1273396 |
| 8 | Search DM | 113480 |
| 9 | Search diabetes | 1575410 |
| 10 | Search mellitus | 1201070 |
| 11 | 1 OR 2 | 394883 |
| 12 | 3 OR 4 OR 5 0R 6 | 10220076 |
| 13 | 7 OR 8 OR 9 OR 10 | 1629839 |
| 14 | 11 AND 12 AND 13 | 6621 |
| 15 | Search “cohort studies”[MeSH Terms] | 1040284 |
| 16 | Search cohort | 1596292 |
| 17 | 15 OR 16 | 1040284 |
| 18 | 14 AND 17 Filters: Humans | 773 |
| 19 | trial | 2626031 |
| 20 | Randomized controlled trial | 0 |
| 21 | "randomized controlled trials as topic" | 260351 |
| 22 | ("randomized controlled trial"[Publication Type] OR "randomized controlled trials as topic"[MeSH Terms] OR "randomized controlled trial" | 1127292 |
| 23 | 19 OR 20 OR 21 OR 22 | 2626031 |
| 24 | 14 AND 23 | 710 |
| 25 | #24 AND humans /de | 696 |
| 26 | #25 AND 2017 OR 2018 or 2019 or 2020 or 2021 or 2022 or 2023 | 300 |
| 27 | #24 And human | 696 |
| 28 | #24 AND human AND26-4-2017 NOT 22-08-2022 | 310 |
|  |  |  |
|  |  |  |


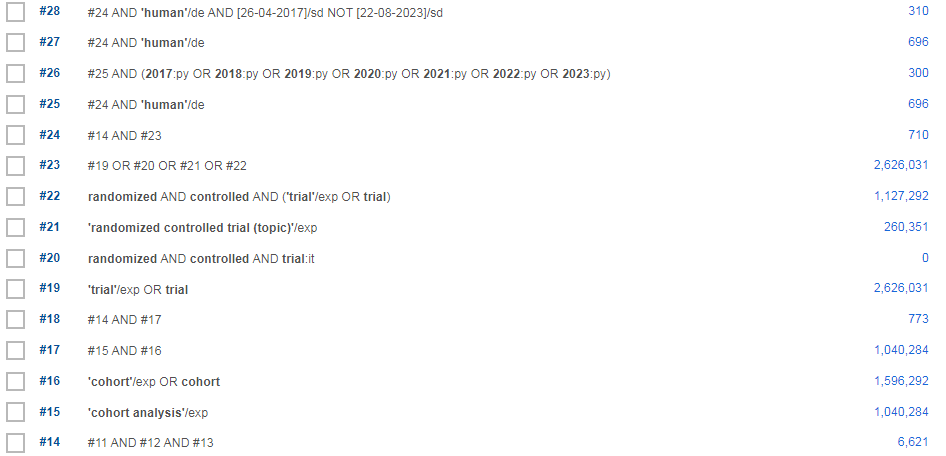


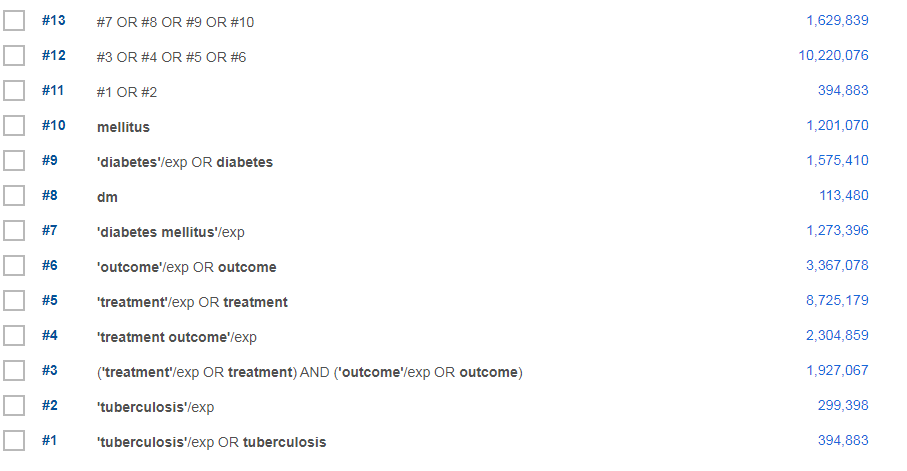


**Effect of glycemic control on TB treatment outcomes among TB-DM patients (COHORT)**

| Search | Query | Items found (Search conducted on 25 Sept 2023) |
| --- | --- | --- |
| 1 | Search tuberculosis | 395693 |
| 2 | Search "tuberculosis"[MeSH Terms] | 299979 |
| 3 | Search "treatment outcome$" | 1099242 |
| 4 | Search "Treatment Outcome"[MeSH Terms] | 2316393 |
| 5 | Search treatment | 8754226 |
| 6 | Search outcome | 3381311 |
| 7 | Search "diabetes mellitus"[MeSH Terms] | 1278789 |
| 8 | Search DM | 114003 |
| 9 | Search diabetes | 1511640 |
| 10 | Search mellitus | 1206103 |
| 11 | 1 OR 2 | 395693 |
| 12 | 3 OR 4 OR 5 0R 6 | 10256527 |
| 13 | 7 OR 8 OR 9 OR 10 | 1636637 |
| 14 | 11 AND 12 AND 13 | 6651 |
| 15 | Search “cohort studies”[MeSH Terms] | 1048475 |
| 16 | Search cohort | 1606036 |
| 17 | 15 OR 16 | 1606036 |
| 18 | 14 AND 17 | 1606036 |
| 19 | 18 AND ‘human’/de | 947 |
|  |  |  |
| 20 | #19 AND (2017:py OR 2018:py OR 2019:py OR 2020:py OR 2021:py OR 2022:py OR 2023:py) | 662 |


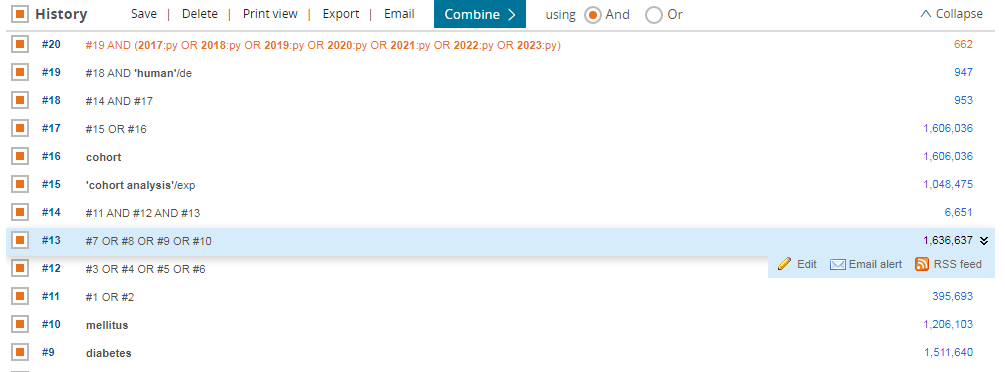


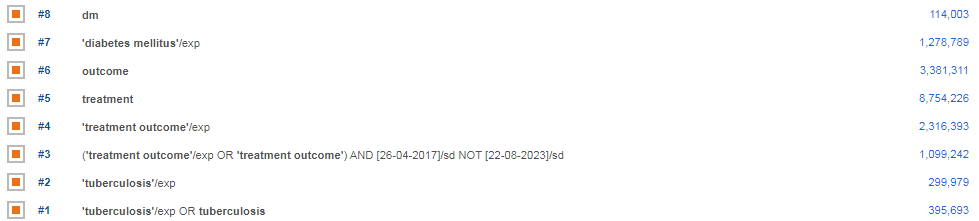


- Cochrane Review


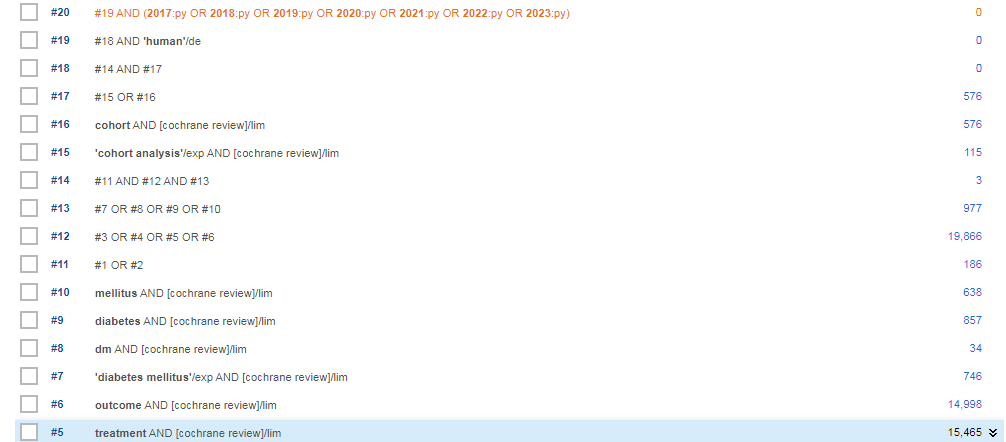


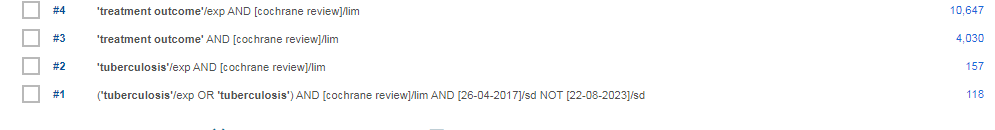

Supplement: S1A Appendix — (ZIP) [file pone.0328619.s005.zip › S1A appendix/S1A_2017-21 Aug 2023/Embase/Search results obj 1 (RCT Cohort).docx]
