# Supplementary material for: Effect of glycemic control and type of diabetes treatment on TB treatment outcomes among people with TB-diabetes: A systematic review (updated August 2024)
Source: PLoS One. 2025 Jul 18;20(7):e0328619. doi: 10.1371/journal.pone.0328619 (PMC12273911; doi:10.1371/journal.pone.0328619)

**Effect of glucose lowering treatment on TB treatment outcomes among TB-DM patients**

Search 1

| #26 | #1 AND #25 |  | 83 | 2023-09-14 | 2023-09-14 |  |
| --- | --- | --- | --- | --- | --- | --- |
|  | #25 | #2 OR #3 OR #4 OR #5 OR #6 OR #7 OR #8 OR #9 OR #10 OR #11 OR #12 OR #13 OR #14 OR #15 OR #16 OR #17 OR #18 OR #19 OR #20 OR #21 OR #22 OR #23 OR #24  Show full |  | 56,867 | 2023-09-14 | 2023-09-14 |
|  | #24 | (beskoa OR 'suiny'/exp OR suiny OR zemiglo OR 'tenelia'/exp OR tenelia OR 'gemigliptin'/exp OR gemigliptin OR 'teneligliptin'/exp OR teneligliptin OR 'galvus'/exp OR galvus OR 'anagliptin'/exp OR anagliptin OR vildagliptin:ti) AND [26-04-2017]/sd NOT [22-08-2023]/sd  Collapse |  | 2,499 | 2023-09-14 | 2023-09-14 |
|  | #23 | ((vipidia OR syr) AND 472 OR 'trelagliptin'/exp OR trelagliptin OR 'omarigliptin'/exp OR omarigliptin OR trazenta OR 'trajenta'/exp OR trajenta OR 'tradjenta'/exp OR tradjenta OR 'bi1356'/exp OR bi1356 OR 'nesina'/exp OR nesina OR 'onglyza'/exp OR onglyza OR 'januvia'/exp OR januvia OR 'alogliptin'/exp OR alogliptin OR 'linagliptin'/exp OR linagliptin OR 'saxagliptin'/exp OR saxagliptin OR 'metformin plus sitagliptin':ti) AND [26-04-2017]/sd NOT [22-08-2023]/sd  Collapse |  | 7,378 | 2023-09-14 | 2023-09-14 |
|  | #22 | (((((((('gliptin'/exp OR gliptin OR 'dpp'/exp OR dpp) AND adj AND iv OR '4') AND adj AND i OR 'dpp'/exp OR dpp) AND adj AND iv OR '4') AND adj AND inhibitor$1 OR dipeptidyl) AND ('peptidase'/exp OR peptidase) AND adj AND iv OR '4') AND adj AND inhibitor$1 OR dipeptidyl) AND ('peptidase'/exp OR peptidase) AND adj AND iv OR '4') AND adj AND i:ti AND [26-04-2017]/sd NOT [22-08-2023]/sd  Show full |  | 33 | 2023-09-14 | 2023-09-14 |
|  | #21 | (('sestrine'/exp OR sestrine OR eurepa OR 'gluconorm'/exp OR gluconorm OR 'novonorm'/exp OR novonorm OR 'prandin'/exp OR prandin OR 'repaglinide'/exp OR repaglinide OR gluco) AND adj AND ('norm'/exp OR norm) OR 'novo'/exp OR novo) AND adj AND norm:ti AND [26-04-2017]/sd NOT [22-08-2023]/sd  Collapse |  | 0 | 2023-09-14 | 2023-09-14 |
|  | #20 | ((deamelin AND adj AND ('s'/exp OR s) OR glyclopyramide OR 'glynase'/exp OR glynase OR 'micronase'/exp OR micronase OR 'glurenorm'/exp OR glurenorm OR 'euglucon'/exp OR euglucon OR 'amaryl'/exp OR amaryl OR 'daonil'/exp OR daonil OR 'glisoxepide'/exp OR glisoxepide OR 'diabeta'/exp OR diabeta OR 'gliquidone'/exp OR gliquidone OR 'glimepirid'/exp OR glimepirid) AND e OR 'glyburide'/exp OR glyburide OR glibenclamide:ti) AND [26-04-2017]/sd NOT [22-08-2023]/sd  Collapse |  | 6,420 | 2023-09-14 | 2023-09-14 |
|  | #19 | ('glucidoral'/exp OR glucidoral OR 'dymelor'/exp OR dymelor OR 'metahexamide'/exp OR metahexamide OR 'orinase'/exp OR orinase OR 'glucotrol'/exp OR glucotrol OR 'diamicron'/exp OR diamicron OR 'carbutamide'/exp OR carbutamide OR 'tolazamide'/exp OR tolazamide OR 'acetohexamide'/exp OR acetohexamide OR 'chlorpropamide'/exp OR chlorpropamide OR 'glipizide'/exp OR glipizide OR 'gliclazide'/exp OR gliclazide OR 'tolbutamide therapy':ti) AND [26-04-2017]/sd NOT [22-08-2023]/sd  Collapse |  | 4,159 | 2023-09-14 | 2023-09-14 |
|  | #18 | (resulin OR 'romozin'/exp OR romozin OR 'noscal'/exp OR noscal OR 'rivoglitazone'/exp OR rivoglitazone OR 'rezulin'/exp OR rezulin OR 'actos'/exp OR actos OR 'avandia'/exp OR avandia OR 'troglitazone'/exp OR troglitazone OR 'pioglitazone'/exp OR pioglitazone OR rosiglitazone:ti) AND [26-04-2017]/sd NOT [22-08-2023]/sd  Collapse |  | 9,892 | 2023-09-14 | 2023-09-14 |
|  | #17 | (biguanideor AND ('thiazolidinedione'/exp OR thiazolidinedione) OR sulfonylurea:ti) AND [26-04-2017]/sd NOT [22-08-2023]/sd |  | 437 | 2023-09-14 | 2023-09-14 |
|  | #16 | ('metformin'/exp OR metformin) AND near AND diabetes:ti AND [26-04-2017]/sd NOT [22-08-2023]/sd |  | 99 | 2023-09-14 | 2023-09-14 |
|  | #15 | ('glufast'/exp OR glufast OR 'starlix'/exp OR starlix OR meglitinides OR 'mitiglinide'/exp OR mitiglinide OR nateglinide:ti) AND [26-04-2017]/sd NOT [22-08-2023]/sd  Show full |  | 675 | 2023-09-14 | 2023-09-14 |
|  | #14 | (voglib OR 'glyset'/exp OR glyset OR 'glucobay'/exp OR glucobay OR 'voglibose'/exp OR voglibose OR 'miglitol'/exp OR miglitol OR 'acarbose'/exp OR acarbose OR 'alpha'/exp OR alpha) AND ('glucosidase'/exp OR glucosidase) AND adj AND inhibitor$1:ti AND [26-04-2017]/sd NOT [22-08-2023]/sd  Collapse |  | 0 | 2023-09-14 | 2023-09-14 |
|  | #13 | (('aleglitazar'/exp OR aleglitazar OR 'tesaglitazar'/exp OR tesaglitazar OR 'muraglitazar'/exp OR muraglitazar OR 'peroxisome'/exp OR peroxisome) AND proliferator AND activated AND ('receptor'/exp OR receptor) AND adj AND agoni1 OR 'ppar'/exp OR ppar) AND adj AND agonist:ti AND [26-04-2017]/sd NOT [22-08-2023]/sd  Collapse |  | 0 | 2023-09-14 | 2023-09-14 |
|  | #12 | ('suglat'/exp OR suglat OR ivokana OR 'lipaglyn'/exp OR lipaglyn OR 'forxiga'/exp OR forxiga OR 'saroglitazar'/exp OR saroglitazar OR 'luseogliflozin'/exp OR luseogliflozin OR 'ertugliflozin'/exp OR ertugliflozin OR 'tofogliflozin'/exp OR tofogliflozin OR 'remogliflozin'/exp OR remogliflozin OR 'sergliflozin'/exp OR sergliflozin OR 'ipragliflozin'/exp OR ipragliflozin OR 'empagliflozin'/exp OR empagliflozin OR 'canagliflozin'/exp OR canagliflozin OR dapagliflozin:ti) AND [26-04-2017]/sd NOT [22-08-2023]/sd  Collapse |  | 13,605 | 2023-09-14 | 2023-09-14 |
|  | #11 | ((sglt2 AND adj AND ('inhibitor'/exp OR inhibitor) OR 'sodium'/exp OR sodium) AND ('glucose'/exp OR glucose) AND adj AND ('cotransporter'/exp OR cotransporter) OR co) AND ('transporter'/exp OR transporter) AND ('2'/exp OR '2') AND adj AND inhibitor:ti AND [26-04-2017]/sd NOT [22-08-2023]/sd  Collapse |  | 1 | 2023-09-14 | 2023-09-14 |
|  | #10 | ('afrezza'/exp OR afrezza OR 'aerx'/exp OR aerx OR exubera:ti) AND [26-04-2017]/sd NOT [22-08-2023]/sd |  | 9,778 | 2023-09-14 | 2023-09-14 |
|  | #9 | (penmix OR novolet OR 'actraphane'/exp OR actraphane OR 'ultratard'/exp OR ultratard OR 'mixtard'/exp OR mixtard OR 'novolin'/exp OR novolin OR 'monotard'/exp OR monotard OR ntermediate) AND adj AND acting AND adj1 AND insulin:ti AND [26-04-2017]/sd NOT [22-08-2023]/sd  Collapse |  | 0 | 2023-09-14 | 2023-09-14 |
|  | #8 | (((ultraphane OR 'velasulin'/exp OR velasulin OR 'biohulin'/exp OR biohulin OR 'protaphan'/exp OR protaphan OR 'velosulin'/exp OR velosulin OR 'insulatard'/exp OR insulatard OR 'novolin'/exp OR novolin OR 'actrapid'/exp OR actrapid OR 'neutral'/exp OR neutral) AND ('protamine'/exp OR protamine) AND adj AND agedorn AND near2 AND ('insulin'/exp OR insulin) OR 'nph'/exp OR nph) AND near2 AND ('insulin'/exp OR insulin) OR 'human'/exp OR human) AND adj AND insulin:ti AND [26-04-2017]/sd NOT [22-08-2023]/sd  Collapse |  | 28 | 2023-09-14 | 2023-09-14 |
|  | #7 | ((((((('novomix'/exp OR novomix OR 'novolog'/exp OR novolog) AND adj AND mix OR 'humalog'/exp OR humalog) AND adj AND mix OR biphasic) AND ('insulin'/exp OR insulin) AND adj AND ('aspart'/exp OR aspart) OR biphasic) AND ('insulin'/exp OR insulin) AND adj AND ('lispro'/exp OR lispro) OR dual) AND acting OR premix$ OR biphasic OR dual) AND acting AND adj AND ('insulin'/exp OR insulin) OR nsulin) AND adj AND analog:ti AND [26-04-2017]/sd NOT [22-08-2023]/sd  Collapse |  | 0 | 2023-09-14 | 2023-09-14 |
|  | #6 | (((((('rapid'/exp OR rapid) AND acting OR fast) AND acting OR short) AND acting OR prandial OR 'bolus'/exp OR bolus OR fast) AND adj AND acting OR 'rapid'/exp OR rapid) AND adj AND acting OR short) AND acting AND adj AND insulin:ti AND [26-04-2017]/sd NOT [22-08-2023]/sd  Collapse |  | 1 | 2023-09-14 | 2023-09-14 |
|  | #5 | (('apidra'/exp OR apidra OR 'novolog'/exp OR novolog OR 'novorapid'/exp OR novorapid OR 'humalog'/exp OR humalog OR 'glulisine'/exp OR glulisine OR 'aspart'/exp OR aspart OR 'lispro'/exp OR lispro OR 'long acting' OR long) AND adj AND acting OR basal) AND adj AND insulin:ti AND [26-04-2017]/sd NOT [22-08-2023]/sd  Collapse |  | 2 | 2023-09-14 | 2023-09-14 |
|  | #4 | (('nn5401'/exp OR nn5401 OR 'nn1250'/exp OR nn1250 OR 'ryzodeg'/exp OR ryzodeg OR 'tresiba'/exp OR tresiba OR 'idegasp'/exp OR idegasp OR 'nn304'/exp OR nn304 OR 'levemir'/exp OR levemir OR ideg OR 'degludec'/exp OR degludec OR 'lantus'/exp OR lantus OR 'detemir'/exp OR detemir OR 'lispro'/exp OR lispro) AND ('rotamine'/exp OR rotamine) AND near AND ('suspension'/exp OR suspension) OR 'glargine':ti) AND [26-04-2017]/sd NOT [22-08-2023]/sd  Collapse |  | 1,300 | 2023-09-14 | 2023-09-14 |
|  | #3 | ((((glpi OR glp1 OR 'incretin'/exp OR incretin OR glp) AND adj OR glp) AND adj AND i OR 'glucagon'/exp OR glucagon) AND like AND ('peptide'/exp OR peptide) AND adj OR 'glucagon'/exp OR glucagon) AND like AND adj AND ('peptide'/exp OR peptide) AND adj:ti  Collapse |  | 0 | 2023-09-14 | 2023-09-14 |
|  | #2 | ('lyxumia'/exp OR lyxumia OR 'semaglutide'/exp OR semaglutide OR 'bydureon'/exp OR bydureon OR 'dulaglutide'/exp OR dulaglutide OR 'victoza'/exp OR victoza OR 'albiglutide'/exp OR albiglutide OR 'byetta'/exp OR byetta OR 'taspoglutide'/exp OR taspoglutide OR 'lixisenatide'/exp OR lixisenatide OR 'liraglutide'/exp OR liraglutide OR 'exenatide'/exp OR exenatide OR exendin:ti) AND [26-04-2017]/sd NOT [22-08-2023]/sd  Collapse |  | 13,587 | 2023-09-14 | 2023-09-14 |
|  | #1 | (((tuberculoses OR kochs) AND adj AND ('disease'/exp OR disease) OR tubercular OR mtb OR antitubercular OR koch) AND ('s'/exp OR s) AND adj AND ('disease'/exp OR disease) OR tuberculosis:ti) AND [26-04-2017]/sd NOT [22-08-2023]/sd  Collapse |  | 38,081 | 2023-09-14 | 2023-09-14 |


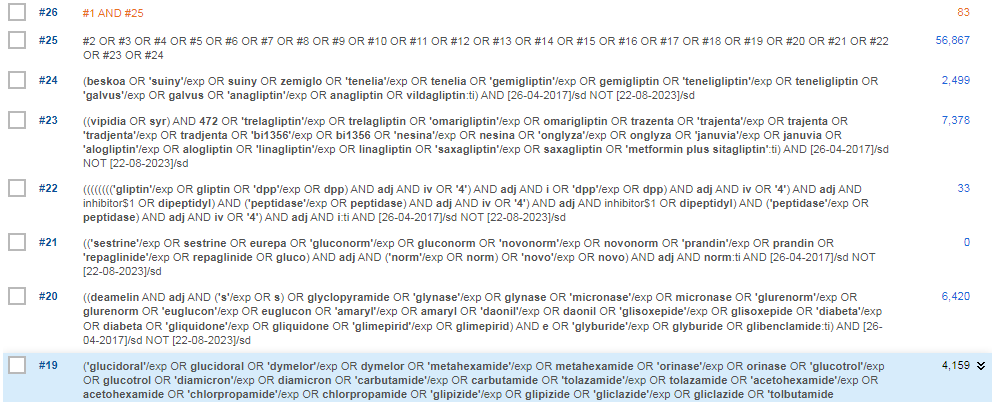

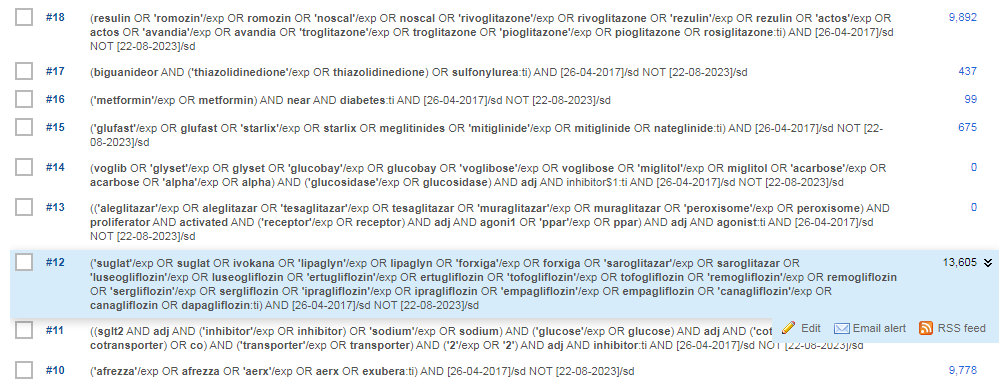


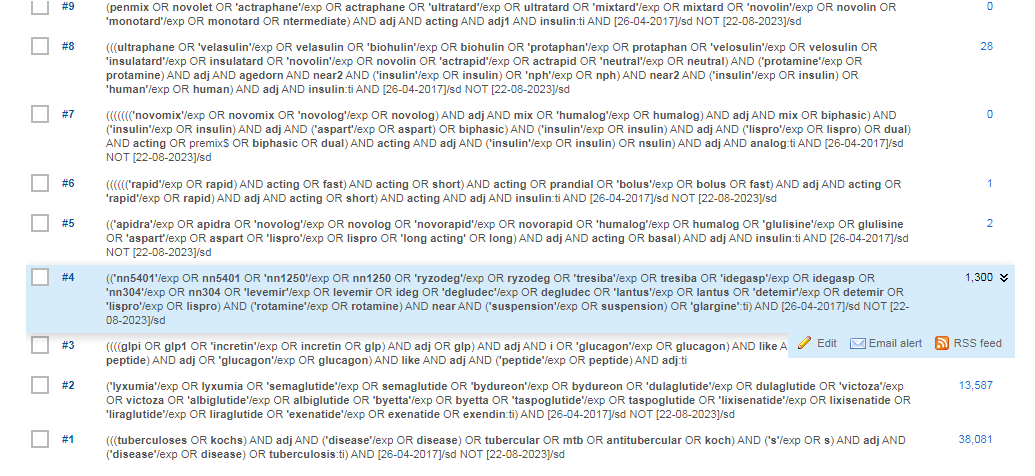


Search 2

| #30 | #1 AND #25 | search2 :Effect of glucose lowering treatment on TB treatment outcomes | 165 | 2023-09-14 | 2023-09-14 |  |
| --- | --- | --- | --- | --- | --- | --- |
|  | #29 | #27 OR #28 | search2 :Effect of glucose lowering treatment on TB treatment outcomes | 0 | 2023-09-14 | 2023-09-14 |
|  | #28 | (tuberculoses OR (kochs AND adj AND disease$1) OR tubercular OR mtb OR antitubercular OR (koch AND s AND adj AND disease$1) OR tuberculosis OR diabetes) AND near10 AND (convergence OR epidemic$1 OR prevalence$1 OR ncidence$1 OR epidemiology OR risk$1) AND [26-04-2017]/sd NOT [22-08-2023]/sd  Show full | search2 :Effect of glucose lowering treatment on TB treatment outcomes | 0 | 2023-09-14 | 2023-09-14 |
|  | #27 | 26 AND near20 AND (tuberculoses OR (kochs AND adj AND disease$1) OR tubercular OR mtb OR antitubercular OR (koch AND s AND adj AND disease$1) OR tuberculosis OR diabetes) AND [26-04-2017]/sd NOT [22-08-2023]/sd  Show full | search2 :Effect of glucose lowering treatment on TB treatment outcomes | 0 | 2023-09-14 | 2023-09-14 |
|  | #26 | (benefit$1 OR improve$1 OR outcome$1 OR result$1 OR effect$1) AND near10 AND (taking OR medicat$3 OR care OR treatment$1 OR control$3 OR therap$3) AND [26-04-2017]/sd NOT [22-08-2023]/sd  Show full | search2 :Effect of glucose lowering treatment on TB treatment outcomes | 0 | 2023-09-14 | 2023-09-14 |
|  | #25 | #2 OR #3 OR #4 OR #5 OR #6 OR #7 OR #8 OR #9 OR #10 OR #11 OR #12 OR #13 OR #14 OR #15 OR #16 OR #17 OR #18 OR #19 OR #20 OR #21 OR #22 OR #23 OR #24  Show full | search2 :Effect of glucose lowering treatment on TB treatment outcomes | 173,133 | 2023-09-14 | 2023-09-14 |
|  | #24 | (beskoa OR 'suiny'/exp OR suiny OR zemiglo OR 'tenelia'/exp OR tenelia OR 'gemigliptin'/exp OR gemigliptin OR 'teneligliptin'/exp OR teneligliptin OR 'galvus'/exp OR galvus OR 'anagliptin'/exp OR anagliptin OR 'vildagliptin'/exp OR vildagliptin) AND [26-04-2017]/sd NOT [22-08-2023]/sd  Show full | search2 :Effect of glucose lowering treatment on TB treatment outcomes | 2,596 | 2023-09-14 | 2023-09-14 |
|  | #23 | ('vipidia'/exp OR vipidia OR 'syr 472'/exp OR 'syr 472' OR 'trelagliptin'/exp OR trelagliptin OR 'omarigliptin'/exp OR omarigliptin OR trazenta OR 'trajenta'/exp OR trajenta OR 'tradjenta'/exp OR tradjenta OR 'bi 1356'/exp OR 'bi 1356' OR 'nesina'/exp OR nesina OR 'onglyza'/exp OR onglyza OR 'januvia'/exp OR januvia OR 'alogliptin'/exp OR alogliptin OR 'linagliptin'/exp OR linagliptin OR 'saxagliptin'/exp OR saxagliptin OR 'sitagliptin'/exp OR sitagliptin) AND [26-04-2017]/sd NOT [22-08-2023]/sd  Show full | search2 :Effect of glucose lowering treatment on TB treatment outcomes | 7,642 | 2023-09-14 | 2023-09-14 |
|  | #22 | (gliptin$1 OR (('dpp'/exp OR dpp) AND (iv OR '4') AND adj AND i) OR (('dpp'/exp OR dpp) AND (iv OR '4') AND adj AND inhibitor$1) OR (dipeptidyl AND ('peptidase'/exp OR peptidase) AND (iv OR '4') AND adj AND inhibitor$1) OR (dipeptidyl AND ('peptidase'/exp OR peptidase) AND (iv OR '4') AND adj AND i)) AND [26-04-2017]/sd NOT [22-08-2023]/sd  Show full | search2 :Effect of glucose lowering treatment on TB treatment outcomes | 5 | 2023-09-14 | 2023-09-14 |
|  | #21 | ('sestrine'/exp OR sestrine OR eurepa OR 'gluconorm'/exp OR gluconorm OR 'novonorm'/exp OR novonorm OR 'prandin'/exp OR prandin OR 'repaglinide'/exp OR repaglinide OR (gluco AND adj AND ('norm'/exp OR norm)) OR (('novo'/exp OR novo) AND adj AND ('norm'/exp OR norm))) AND [26-04-2017]/sd NOT [22-08-2023]/sd  Show full | search2 :Effect of glucose lowering treatment on TB treatment outcomes | 1,185 | 2023-09-14 | 2023-09-14 |
|  | #20 | (deamelin AND adj AND ('s'/exp OR s) OR glyclopyramide OR 'glynase'/exp OR glynase OR 'micronase'/exp OR micronase OR 'glurenorm'/exp OR glurenorm OR 'euglucon'/exp OR euglucon OR 'amaryl'/exp OR amaryl OR 'daonil'/exp OR daonil OR 'glisoxepide'/exp OR glisoxepide OR 'diabeta'/exp OR diabeta OR 'gliquidone'/exp OR gliquidone OR 'glimepiride'/exp OR glimepiride OR 'glyburide'/exp OR glyburide OR 'glibenclamide'/exp OR glibenclamide) AND [26-04-2017]/sd NOT [22-08-2023]/sd  Show full | search2 :Effect of glucose lowering treatment on TB treatment outcomes | 8,427 | 2023-09-14 | 2023-09-14 |
|  | #19 | ('glucidoral'/exp OR glucidoral OR 'dymelor'/exp OR dymelor OR 'metahexamide'/exp OR metahexamide OR 'orinase'/exp OR orinase OR 'glucotrol'/exp OR glucotrol OR 'diamicron'/exp OR diamicron OR 'carbutamide'/exp OR carbutamide OR 'tolazamide'/exp OR tolazamide OR 'acetohexamide'/exp OR acetohexamide OR 'chlorpropamide'/exp OR chlorpropamide OR 'glipizide'/exp OR glipizide OR 'gliclazide'/exp OR gliclazide OR 'tolbutamide'/exp OR tolbutamide) AND [26-04-2017]/sd NOT [22-08-2023]/sd  Show full | search2 :Effect of glucose lowering treatment on TB treatment outcomes | 4,211 | 2023-09-14 | 2023-09-14 |
|  | #18 | (resulin OR 'romozin'/exp OR romozin OR 'noscal'/exp OR noscal OR 'rivoglitazone'/exp OR rivoglitazone OR 'rezulin'/exp OR rezulin OR 'actos'/exp OR actos OR 'avandia'/exp OR avandia OR 'troglitazone'/exp OR troglitazone OR 'pioglitazone'/exp OR pioglitazone OR 'rosiglitazone'/exp OR rosiglitazone) AND [26-04-2017]/sd NOT [22-08-2023]/sd  Show full | search2 :Effect of glucose lowering treatment on TB treatment outcomes | 10,064 | 2023-09-14 | 2023-09-14 |
|  | #17 | (biguanide$1 OR thiazolidinedione$1 OR sulfonylurea$1) AND [26-04-2017]/sd NOT [22-08-2023]/sd | search2 :Effect of glucose lowering treatment on TB treatment outcomes | 3 | 2023-09-14 | 2023-09-14 |
|  | #16 | ('metformin'/exp OR metformin) AND near AND ('diabetes'/exp OR diabetes) AND [26-04-2017]/sd NOT [22-08-2023]/sd | search2 :Effect of glucose lowering treatment on TB treatment outcomes | 249 | 2023-09-14 | 2023-09-14 |
|  | #15 | ('glufast'/exp OR glufast OR 'starlix'/exp OR starlix OR meglitinides OR 'mitiglinide'/exp OR mitiglinide OR 'nateglinide'/exp OR nateglinide) AND [26-04-2017]/sd NOT [22-08-2023]/sd  Show full | search2 :Effect of glucose lowering treatment on TB treatment outcomes | 681 | 2023-09-14 | 2023-09-14 |
|  | #14 | voglib OR 'glyset'/exp OR glyset OR 'glucobay'/exp OR glucobay OR 'voglibose'/exp OR voglibose OR 'miglitol'/exp OR miglitol OR 'acarbose'/exp OR acarbose OR (('alpha'/exp OR alpha) AND ('glucosidase'/exp OR glucosidase) AND adj AND inhibitor$1)  Show full | search2 :Effect of glucose lowering treatment on TB treatment outcomes | 12,851 | 2023-09-14 | 2023-09-14 |
|  | #13 | ('aleglitazar'/exp OR aleglitazar OR 'tesaglitazar'/exp OR tesaglitazar OR 'muraglitazar'/exp OR muraglitazar OR (('peroxisome'/exp OR peroxisome) AND proliferator AND activated AND ('receptor'/exp OR receptor) AND adj AND agonist$1) OR (('ppar'/exp OR ppar) AND adj AND agonist$1)) AND [26-04-2017]/sd NOT [22-08-2023]/sd  Show full | search2 :Effect of glucose lowering treatment on TB treatment outcomes | 120 | 2023-09-14 | 2023-09-14 |
|  | #12 | ('suglat'/exp OR suglat OR ivokana OR 'lipaglyn'/exp OR lipaglyn OR 'forxiga'/exp OR forxiga OR 'saroglitazar'/exp OR saroglitazar OR 'luseogliflozin'/exp OR luseogliflozin OR 'ertugliflozin'/exp OR ertugliflozin OR 'tofogliflozin'/exp OR tofogliflozin OR 'remogliflozin'/exp OR remogliflozin OR 'sergliflozin'/exp OR sergliflozin OR 'ipragliflozin'/exp OR ipragliflozin OR 'empagliflozin'/exp OR empagliflozin OR 'canagliflozin'/exp OR canagliflozin OR 'dapagliflozin'/exp OR dapagliflozin) AND [26-04-2017]/sd NOT [22-08-2023]/sd  Show full | search2 :Effect of glucose lowering treatment on TB treatment outcomes | 13,711 | 2023-09-14 | 2023-09-14 |
|  | #11 | (sglt2 AND adj AND inhibitor$1 OR (('sodium'/exp OR sodium) AND ('glucose'/exp OR glucose) AND ('cotransporter'/exp OR cotransporter OR (co AND adj AND ('transporter'/exp OR transporter))) AND ('2'/exp OR '2') AND adj AND inhibitor$1)) AND [26-04-2017]/sd NOT [22-08-2023]/sd  Show full | search2 :Effect of glucose lowering treatment on TB treatment outcomes | 0 | 2023-09-14 | 2023-09-14 |
|  | #10 | ('afrezza'/exp OR afrezza OR 'aerx'/exp OR aerx OR 'exubera'/exp OR exubera) AND [26-04-2017]/sd NOT [22-08-2023]/sd | search2 :Effect of glucose lowering treatment on TB treatment outcomes | 9,779 | 2023-09-14 | 2023-09-14 |
|  | #9 | (penmix OR novolet OR 'actraphane'/exp OR actraphane OR 'ultratard'/exp OR ultratard OR 'mixtard'/exp OR mixtard OR 'novolin'/exp OR novolin OR 'monotard'/exp OR monotard OR (('intermediate'/exp OR intermediate) AND adj AND acting AND adj1 AND ('insulin'/exp OR insulin))) AND [26-04-2017]/sd NOT [22-08-2023]/sd  Show full | search2 :Effect of glucose lowering treatment on TB treatment outcomes | 108,580 | 2023-09-14 | 2023-09-14 |
|  | #8 | (ultraphane OR 'velasulin'/exp OR velasulin OR 'biohulin'/exp OR biohulin OR protaphan$ OR 'velosulin'/exp OR velosulin OR 'insulatard'/exp OR insulatard OR 'novolin'/exp OR novolin OR 'actrapid'/exp OR actrapid OR (('neutral'/exp OR neutral) AND ('protamine'/exp OR protamine) AND adj AND ('hagedorn'/exp OR hagedorn) AND near2 AND ('insulin'/exp OR insulin)) OR (('nph'/exp OR nph) AND near2 AND ('insulin'/exp OR insulin)) OR (('human'/exp OR human) AND adj AND ('insulin'/exp OR insulin))) AND [26-04-2017]/sd NOT [22-08-2023]/sd  Show full | search2 :Effect of glucose lowering treatment on TB treatment outcomes | 109,505 | 2023-09-14 | 2023-09-14 |
|  | #7 | ('novomix'/exp OR novomix OR (('novolog'/exp OR novolog) AND adj AND mix) OR (('humalog'/exp OR humalog) AND adj AND mix) OR (biphasic AND ('insulin'/exp OR insulin) AND adj AND ('aspart'/exp OR aspart)) OR (biphasic AND ('insulin'/exp OR insulin) AND adj AND ('lispro'/exp OR lispro)) OR (('dual cting' OR premix$ OR biphasic OR (dual AND adj AND acting)) AND adj AND ('insulin'/exp OR insulin)) OR (('insulin'/exp OR insulin) AND adj AND analog$)) AND [26-04-2017]/sd NOT [22-08-2023]/sd  Show full | search2 :Effect of glucose lowering treatment on TB treatment outcomes | 2,188 | 2023-09-14 | 2023-09-14 |
|  | #6 | ('rapid acting' OR 'fast acting' OR 'short acting' OR prandial OR 'bolus'/exp OR bolus OR (fast AND adj AND acting) OR (('rapid'/exp OR rapid) AND adj AND acting) OR (short AND adj AND acting)) AND adj AND ('insulin'/exp OR insulin) AND [26-04-2017]/sd NOT [22-08-2023]/sd  Show full | search2 :Effect of glucose lowering treatment on TB treatment outcomes | 4 | 2023-09-14 | 2023-09-14 |
|  | #5 | ('apidra'/exp OR apidra OR 'novolog'/exp OR novolog OR 'novorapid'/exp OR novorapid OR 'humalog'/exp OR humalog OR 'glulisine'/exp OR glulisine OR 'aspart'/exp OR aspart OR 'lispro'/exp OR lispro OR (('long acting' OR (long AND adj AND acting) OR basal) AND adj AND ('insulin'/exp OR insulin))) AND [26-04-2017]/sd NOT [22-08-2023]/sd  Show full | search2 :Effect of glucose lowering treatment on TB treatment outcomes | 3,905 | 2023-09-14 | 2023-09-14 |
|  | #4 | ('nn5401'/exp OR nn5401 OR 'nn1250'/exp OR nn1250 OR 'ryzodeg'/exp OR ryzodeg OR 'tresiba'/exp OR tresiba OR 'idegasp'/exp OR idegasp OR 'nn304'/exp OR nn304 OR 'levemir'/exp OR levemir OR ideg OR 'degludec'/exp OR degludec OR 'lantus'/exp OR lantus OR 'detemir'/exp OR detemir OR (('lispro'/exp OR lispro) AND ('rotamine'/exp OR rotamine) AND near AND ('suspension'/exp OR suspension)) OR 'glargine'/exp OR glargine) AND [26-04-2017]/sd NOT [22-08-2023]/sd  Show full | search2 :Effect of glucose lowering treatment on TB treatment outcomes | 6,853 | 2023-09-14 | 2023-09-14 |
|  | #3 | (glpi OR glp1 OR incretin$1 OR (glp AND adj AND ('1'/exp OR '1')) OR (glp AND adj AND i) OR (('glucagon'/exp OR glucagon) AND like AND ('peptide'/exp OR peptide) AND adj AND ('1'/exp OR '1')) OR (('glucagon'/exp OR glucagon) AND like AND ('peptide'/exp OR peptide) AND adj AND i)) AND [26-04-2017]/sd NOT [22-08-2023]/sd  Show full | search2 :Effect of glucose lowering treatment on TB treatment outcomes | 12,442 | 2023-09-14 | 2023-09-14 |
|  | #2 | ('lyxumia'/exp OR lyxumia OR 'semaglutide'/exp OR semaglutide OR 'bydureon'/exp OR bydureon OR 'dulaglutide'/exp OR dulaglutide OR 'victoza'/exp OR victoza OR 'albiglutide'/exp OR albiglutide OR 'byetta'/exp OR byetta OR 'taspoglutide'/exp OR taspoglutide OR 'lixisenatide'/exp OR lixisenatide OR 'liraglutide'/exp OR liraglutide OR 'exenatide'/exp OR exenatide OR 'exendin 4'/exp OR 'exendin 4') AND [26-04-2017]/sd NOT [22-08-2023]/sd  Show full | search2 :Effect of glucose lowering treatment on TB treatment outcomes | 13,617 | 2023-09-14 | 2023-09-14 |
|  | #1 | (((tuberculoses OR kochs) AND adj AND ('disease'/exp OR disease) OR tubercular OR mtb OR antitubercular OR koch) AND ('s'/exp OR s) AND adj AND ('disease'/exp OR disease) OR tuberculosis:ti) AND [26-04-2017]/sd NOT [22-08-2023]/sd  Show full | search2 :Effect of glucose lowering treatment on TB treatment outcomes | 38,081 | 2023-09-14 | 2023-09-14 |


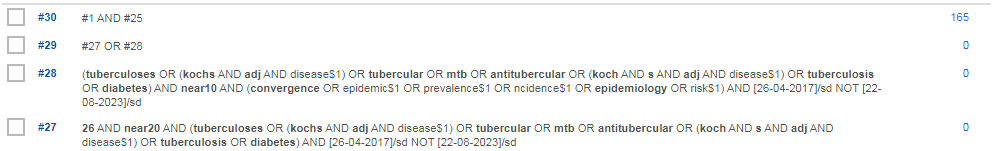


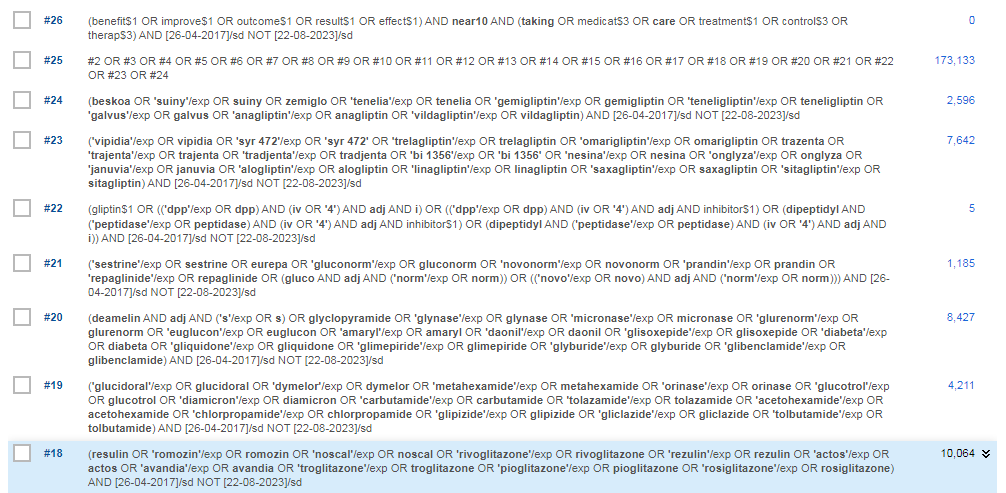


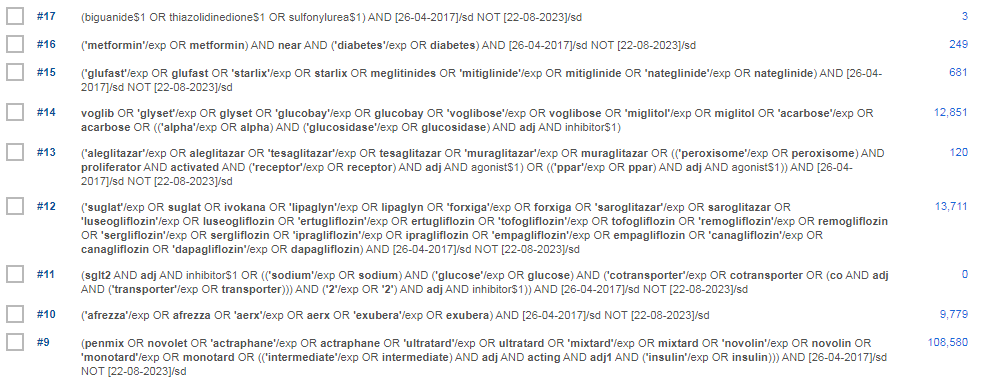
Bottom of Form


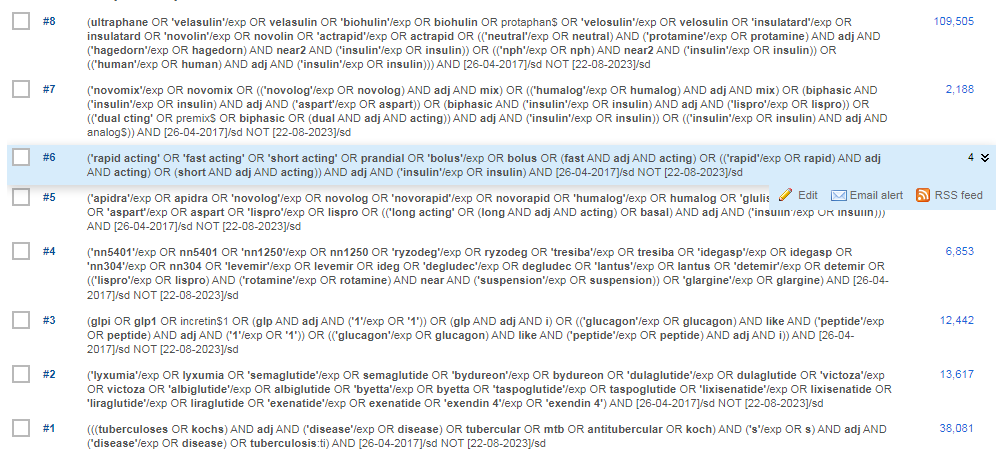


Search 3

Search Top of Form

**#27**

#1 AND #25 AND #26

[67](https://www.embase.com/)

**#26**

((**'tuberculosis'**/exp OR **tuberculosis** OR **'diabetes'**/exp OR **diabetes**) AND **near10** AND (**'convergence'**/exp OR **convergence**) OR **'epidemic'**/exp OR **epidemic** OR **'prevalence'**/exp OR **prevalence** OR **'incidence'**/exp OR **incidence** OR **'epidemiology'**/exp OR **epidemiology** OR **risk**:ti) AND [26-04-2017]/sd NOT [22-08-2023]/sd

[2,736,501](https://www.embase.com/)

**#25**

#2 OR #3 OR #4 OR #5 OR #6 OR #7 OR #8 OR #9 OR #10 OR #11 OR #12 OR #13 OR #14 OR #15 OR #16 OR #17 OR #18 OR #19 OR #20 OR #21 OR #22 OR #23 OR #24

[459,466](https://www.embase.com/)

**#24**

(**beskoa** OR **'suiny'**/exp OR **suiny** OR **zemiglo** OR **'tenelia'**/exp OR **tenelia** OR **'gemigliptin'**/exp OR **gemigliptin** OR **'teneligliptin'**/exp OR **teneligliptin** OR **'galvus'**/exp OR **galvus** OR **'anagliptin'**/exp OR **anagliptin** OR **vildagliptin**:ti) AND [26-04-2017]/sd NOT [22-08-2023]/sd

[2,499](https://www.embase.com/)

**#23**

((**'vipidia'**/exp OR **vipidia** OR **syr**) AND **472** OR **'trelagliptin'**/exp OR **trelagliptin** OR **'omarigliptin'**/exp OR **omarigliptin** OR **trazenta** OR **'trajenta'**/exp OR **trajenta** OR **'tradjenta'**/exp OR **tradjenta** OR **'bi 1356'**/exp OR **'bi 1356'** OR **'nesina'**/exp OR **nesina** OR **'onglyza'**/exp OR **onglyza** OR **'januvia'**/exp OR **januvia** OR **'alogliptin'**/exp OR **alogliptin** OR **'linagliptin'**/exp OR **linagliptin** OR **'saxagliptin'**/exp OR **saxagliptin** OR **sitagliptin**:ti) AND [26-04-2017]/sd NOT [22-08-2023]/sd

[7,424](https://www.embase.com/)

**#22**

(((((((**gliptinor** AND (**'dpp'**/exp OR **dpp**) AND **adj** AND **iv** OR **'4'**) AND **adj** AND **i** OR **'dpp'**/exp OR **dpp**) AND **adj** AND **iv** OR **'4'**) AND **adj** AND (**'inhibitor'**/exp OR **inhibitor**) OR **dipeptidyl**) AND (**'peptidase'**/exp OR **peptidase**) AND **adj** AND **iv** OR **'4'**) AND **adj** AND (**'inhibitor'**/exp OR **inhibitor**) OR **dipeptidyl**) AND (**'peptidase'**/exp OR **peptidase**) AND **adj** AND **iv** OR **'4'**) AND **adj**:ti AND [26-04-2017]/sd NOT [22-08-2023]/sd

[26](https://www.embase.com/)

**#21**

((**'sestrine'**/exp OR **sestrine** OR **eurepa** OR **'gluconorm'**/exp OR **gluconorm** OR **'novonorm'**/exp OR **novonorm** OR **'prandin'**/exp OR **prandin** OR **'repaglinide'**/exp OR **repaglinide** OR **gluco**) AND **adj** AND (**'norm'**/exp OR **norm**) OR **'novo'**/exp OR **novo**) AND **adj** AND **norm**:ti AND [26-04-2017]/sd NOT [22-08-2023]/sd

[0](https://www.embase.com/)

**#20**

(**deamelin** AND **adj** AND (**'s'**/exp OR **s**) OR **glyclopyramide** OR **'glynase'**/exp OR **glynase** OR **'micronase'**/exp OR **micronase** OR **'glurenorm'**/exp OR **glurenorm** OR **'euglucon'**/exp OR **euglucon** OR **'amaryl'**/exp OR **amaryl** OR **'daonil'**/exp OR **daonil** OR **'glisoxepide'**/exp OR **glisoxepide** OR **'diabeta'**/exp OR **diabeta** OR **'gliquidone'**/exp OR **gliquidone** OR **'glimepiride'**/exp OR **glimepiride** OR **'glyburide'**/exp OR **glyburide** OR **glibenclamide**:ti) AND [26-04-2017]/sd NOT [22-08-2023]/sd

[8,189](https://www.embase.com/)

**#19**

(**'glucidoral'**/exp OR **glucidoral** OR **'dymelor'**/exp OR **dymelor** OR **'metahexamide'**/exp OR **metahexamide** OR **'orinase'**/exp OR **orinase** OR **'glucotrol'**/exp OR **glucotrol** OR **'diamicron'**/exp OR **diamicron** OR **'carbutamide'**/exp OR **carbutamide** OR **'tolazamide'**/exp OR **tolazamide** OR **'acetohexamide'**/exp OR **acetohexamide** OR **'chlorpropamide'**/exp OR **chlorpropamide** OR **'glipizide'**/exp OR **glipizide** OR **'gliclazide'**/exp OR **gliclazide** OR **tolbutamide**:ti) AND [26-04-2017]/sd NOT [22-08-2023]/sd

[4,159](https://www.embase.com/)

**#18**

(**resulin** OR **'romozin'**/exp OR **romozin** OR **'noscal'**/exp OR **noscal** OR **'rivoglitazone'**/exp OR **rivoglitazone** OR **'rezulin'**/exp OR **rezulin** OR **'actos'**/exp OR **actos** OR **'avandia'**/exp OR **avandia** OR **'troglitazone'**/exp OR **troglitazone** OR **'pioglitazone'**/exp OR **pioglitazone** OR **rosiglitazone**:ti) AND [26-04-2017]/sd NOT [22-08-2023]/sd

[9,892](https://www.embase.com/)

**#17**

(**'biguanide'**/exp OR **biguanide** OR **'thiazolidinedione'**/exp OR **thiazolidinedione** OR **sulfonylurea**:ti) AND [26-04-2017]/sd NOT [22-08-2023]/sd

[6,913](https://www.embase.com/)

**#16**

(**'metformin'**/exp OR **metformin**) AND **near** AND (**'diabetes'**/exp OR **diabetes**) AND [26-04-2017]/sd NOT [22-08-2023]/sd

[249](https://www.embase.com/)

**#15**

(**'glufast'**/exp OR **glufast** OR **'starlix'**/exp OR **starlix** OR **meglitinides** OR **'mitiglinide'**/exp OR **mitiglinide** OR **'nateglinide'**/exp OR **nateglinide**) AND [26-04-2017]/sd NOT [22-08-2023]/sd

[681](https://www.embase.com/)

**#14**

(**voglib** OR **'glyset'**/exp OR **glyset** OR **'glucobay'**/exp OR **glucobay** OR **'voglibose'**/exp OR **voglibose** OR **'miglitol'**/exp OR **miglitol** OR **'acarbose'**/exp OR **acarbose** OR **'alpha'**/exp OR **alpha**) AND (**'glucosidase'**/exp OR **glucosidase**) AND **adj** AND **inhibitor**:ti AND [26-04-2017]/sd NOT [22-08-2023]/sd

[0](https://www.embase.com/)

**#13**

((**'aleglitazar'**/exp OR **aleglitazar** OR **'tesaglitazar'**/exp OR **tesaglitazar** OR **'muraglitazar'**/exp OR **muraglitazar** OR **'peroxisome'**/exp OR **peroxisome**) AND **proliferator** AND **activated** AND (**'receptor'**/exp OR **receptor**) AND **adj** AND (**'agonist'**/exp OR **agonist**) OR **'ppar'**/exp OR **ppar**) AND **adj** AND **agonist**:ti AND [26-04-2017]/sd NOT [22-08-2023]/sd

[0](https://www.embase.com/)

**#12**

(**'suglat'**/exp OR **suglat** OR **ivokana** OR **'lipaglyn'**/exp OR **lipaglyn** OR **'forxiga'**/exp OR **forxiga** OR **'saroglitazar'**/exp OR **saroglitazar** OR **'luseogliflozin'**/exp OR **luseogliflozin** OR **'ertugliflozin'**/exp OR **ertugliflozin** OR **'tofogliflozin'**/exp OR **tofogliflozin** OR **'remogliflozin'**/exp OR **remogliflozin** OR **'sergliflozin'**/exp OR **sergliflozin** OR **'ipragliflozin'**/exp OR **ipragliflozin** OR **'empagliflozin'**/exp OR **empagliflozin** OR **'canagliflozin'**/exp OR **canagliflozin** OR **dapagliflozin**:ti) AND [26-04-2017]/sd NOT [22-08-2023]/sd

[13,605](https://www.embase.com/)

**#11**

((**sglt2** AND **adj** AND (**'inhibitor'**/exp OR **inhibitor**) OR **'sodium'**/exp OR **sodium**) AND (**'glucose'**/exp OR **glucose**) AND **adj** AND (**'cotransporter'**/exp OR **cotransporter**) OR **co**) AND (**'transporter'**/exp OR **transporter**) AND (**'2'**/exp OR **'2'**) AND **adj** AND **inhibitor**:ti AND [26-04-2017]/sd NOT [22-08-2023]/sd

[1](https://www.embase.com/)

**#10**

(**'afrezza'**/exp OR **afrezza** OR **'aerx'**/exp OR **aerx** OR **'exubera'**/exp OR **exubera**) AND [26-04-2017]/sd NOT [22-08-2023]/sd

[9,779](https://www.embase.com/)

**#9**

**penmix** OR **novolet** OR **'actraphane'**/exp OR **actraphane** OR **'ultratard'**/exp OR **ultratard** OR **'mixtard'**/exp OR **mixtard** OR **'novolin'**/exp OR **novolin** OR **'monotard'**/exp OR **monotard** OR ((**'intermediate'**/exp OR **intermediate**) AND **adj** AND **acting** AND **adj1** AND (**'insulin'**/exp OR **insulin**))

[408,499](https://www.embase.com/)

**#8**

(**ultraphane** OR **'velasulin'**/exp OR **velasulin** OR **'biohulin'**/exp OR **biohulin** OR protaphan$ OR **'velosulin'**/exp OR **velosulin** OR **'insulatard'**/exp OR **insulatard** OR **'novolin'**/exp OR **novolin** OR **'actrapid'**/exp OR **actrapid** OR ((**'neutral'**/exp OR **neutral**) AND (**'protamine'**/exp OR **protamine**) AND **adj** AND (**'hagedorn'**/exp OR **hagedorn**) AND **near2** AND (**'insulin'**/exp OR **insulin**)) OR ((**'nph'**/exp OR **nph**) AND **near2** AND (**'insulin'**/exp OR **insulin**)) OR ((**'human'**/exp OR **human**) AND **adj** AND (**'insulin'**/exp OR **insulin**))) AND [26-04-2017]/sd NOT [22-08-2023]/sd

[109,505](https://www.embase.com/)

**#7**

(**'novomix'**/exp OR **novomix** OR ((**'novolog'**/exp OR **novolog**) AND **adj** AND **mix**) OR ((**'humalog'**/exp OR **humalog**) AND **adj** AND **mix**) OR (**biphasic** AND (**'insulin'**/exp OR **insulin**) AND **adj** AND (**'aspart'**/exp OR **aspart**)) OR (**biphasic** AND (**'insulin'**/exp OR **insulin**) AND **adj** AND (**'lispro'**/exp OR **lispro**)) OR ((**'dual acting'** OR premix$ OR **biphasic** OR (**dual** AND **adj** AND **acting**)) AND **adj** AND (**'insulin'**/exp OR **insulin**)) OR ((**'insulin'**/exp OR **insulin**) AND **adj** AND analog$)) AND [26-04-2017]/sd NOT [22-08-2023]/sd

[2,188](https://www.embase.com/)

**#6**

(**'rapid acting'** OR **'fast acting'** OR **'short acting'** OR **prandial** OR **'bolus'**/exp OR **bolus** OR (**fast** AND **adj** AND **acting**) OR ((**'rapid'**/exp OR **rapid**) AND **adj** AND **acting**) OR (**short** AND **adj** AND **acting**)) AND **adj** AND (**'insulin'**/exp OR **insulin**) AND [26-04-2017]/sd NOT [22-08-2023]/sd

[4](https://www.embase.com/)

**#5**

(**'apidra'**/exp OR **apidra** OR **'novolog'**/exp OR **novolog** OR **'novorapid'**/exp OR **novorapid** OR **'humalog'**/exp OR **humalog** OR **'glulisine'**/exp OR **glulisine** OR **'aspart'**/exp OR **aspart** OR **'lispro'**/exp OR **lispro** OR ((**'long acting'** OR (**long** AND **adj** AND **acting**) OR **basal**) AND **adj** AND (**'insulin'**/exp OR **insulin**))) AND [26-04-2017]/sd NOT [22-08-2023]/sd

[3,905](https://www.embase.com/)

**#4**

(**'nn5401'**/exp OR **nn5401** OR **'nn1250'**/exp OR **nn1250** OR **'ryzodeg'**/exp OR **ryzodeg** OR **'tresiba'**/exp OR **tresiba** OR **'idegasp'**/exp OR **idegasp** OR **'nn304'**/exp OR **nn304** OR **'levemir'**/exp OR **levemir** OR **ideg** OR **'degludec'**/exp OR **degludec** OR **'lantus'**/exp OR **lantus** OR **'detemir'**/exp OR **detemir** OR ((**'lispro'**/exp OR **lispro**) AND (**'protamine'**/exp OR **protamine**) AND **near** AND (**'suspension'**/exp OR **suspension**)) OR **'glargine'**/exp OR **glargine**) AND [26-04-2017]/sd NOT [22-08-2023]/sd

[6,853](https://www.embase.com/)

**#3**

((((**glpi** OR **glp1** OR **'incretin'**/exp OR **incretin** OR **glp**) AND **adj** OR **glp**) AND **adj** OR **'glucagon'**/exp OR **glucagon**) AND **like** AND (**'peptide'**/exp OR **peptide**) AND **adj** OR **'glucagon'**/exp OR **glucagon**) AND **like** AND **adj** AND (**'peptide'**/exp OR **peptide**) AND **adj**:ti AND [26-04-2017]/sd NOT [22-08-2023]/sd

[0](https://www.embase.com/)

**#2**

(**'lyxumia'**/exp OR **lyxumia** OR **'semaglutide'**/exp OR **semaglutide** OR **'bydureon'**/exp OR **bydureon** OR **'dulaglutide'**/exp OR **dulaglutide** OR **'victoza'**/exp OR **victoza** OR **'albiglutide'**/exp OR **albiglutide** OR **'byetta'**/exp OR **byetta** OR **'taspoglutide'**/exp OR **taspoglutide** OR **'lixisenatide'**/exp OR **lixisenatide** OR **'liraglutide'**/exp OR **liraglutide** OR **'exenatide'**/exp OR **exenatide** OR **'exendin 3'**:ti) AND [26-04-2017]/sd NOT [22-08-2023]/sd

[13,495](https://www.embase.com/)

**#1**

(((**tuberculoses** OR **kochs**) AND **adj** AND (**'disease'**/exp OR **disease**) OR **mtb** OR **koch**) AND (**'s'**/exp OR **s**) AND **adj** AND (**'disease'**/exp OR **disease**) OR **tuberculosis**:ti) AND [26-04-2017]/sd NOT [22-08-2023]/sd

[38,081](https://www.embase.com/)

Bottom of Form


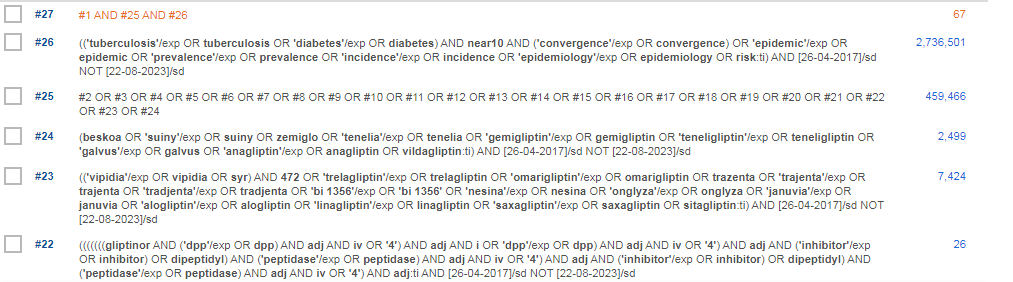


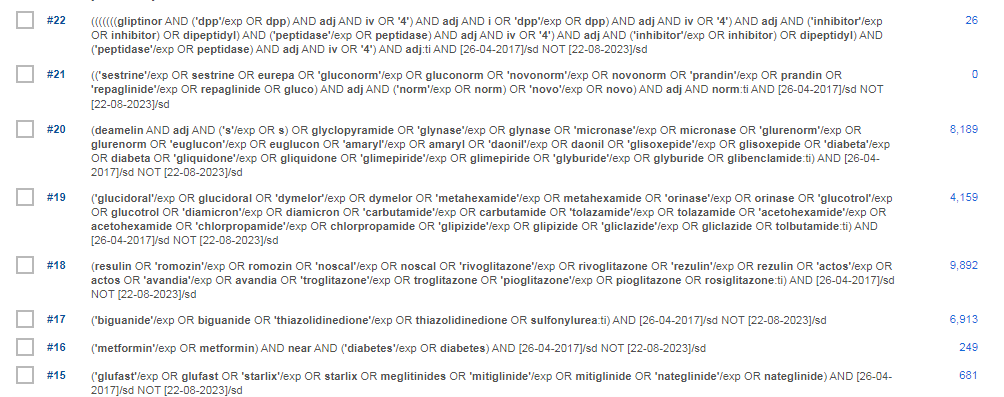


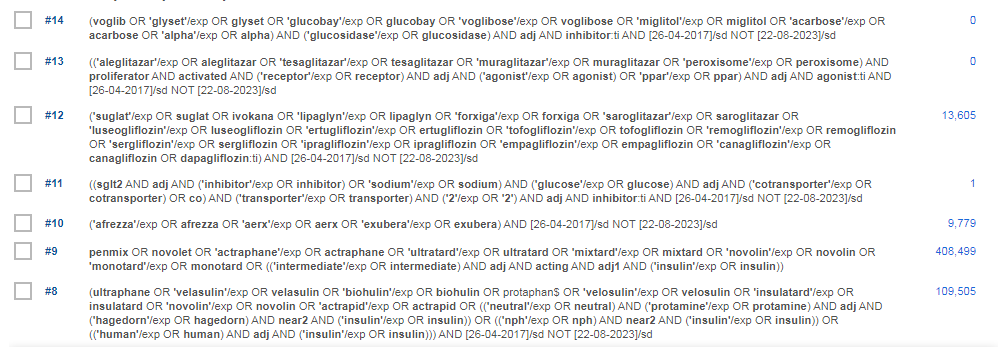


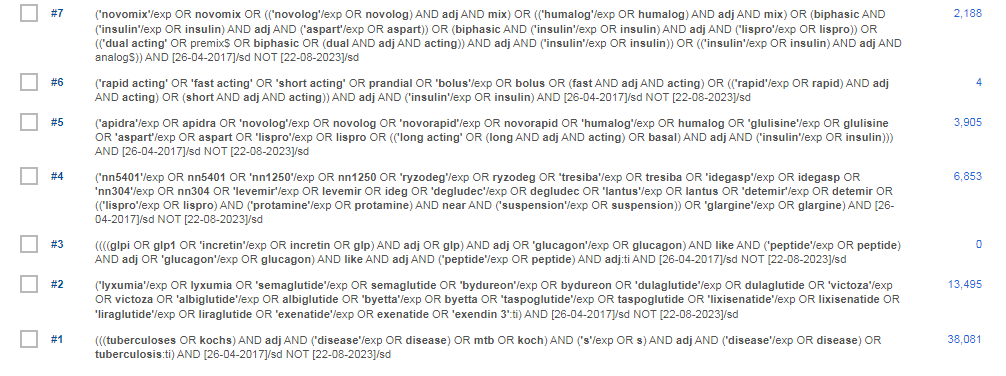

Supplement: S1A Appendix — (ZIP) [file pone.0328619.s005.zip › S1A appendix/S1A_2017-21 Aug 2023/Embase/Search results obj 2 (search 1-3).docx]
