# Supplementary material for: Effect of glycemic control and type of diabetes treatment on TB treatment outcomes among people with TB-diabetes: A systematic review (updated August 2024)
Source: PLoS One. 2025 Jul 18;20(7):e0328619. doi: 10.1371/journal.pone.0328619 (PMC12273911; doi:10.1371/journal.pone.0328619)
Supplement: S1A Appendix — (ZIP) [file pone.0328619.s005.zip › S1A appendix/S1A_2017-21 Aug 2023/Google Scholar Search terms.docx]

**GOOGLE SCHOLAR**

**TB-DM Sr – Cohort studies**

Tuberculosis AND "treatment outcome" AND "Diabetes Mellitus" AND (cohort OR cohort studies OR cohort study OR cohort analysis) 2017-2023

**TB-DM SR - trial**

(((Tuberculosis AND "treatment outcome") AND "Diabetes Mellitus") AND (trial OR RCT)) 2017-2023
