# Supplementary material for: Effect of glycemic control and type of diabetes treatment on TB treatment outcomes among people with TB-diabetes: A systematic review (updated August 2024)
Source: PLoS One. 2025 Jul 18;20(7):e0328619. doi: 10.1371/journal.pone.0328619 (PMC12273911; doi:10.1371/journal.pone.0328619)
Supplement: S1A Appendix — (ZIP) [file pone.0328619.s005.zip › S1A appendix/S1A_2017-21 Aug 2023/Pubmed/Obj 1 search results.docx]

| Search | Query | Items found (Search conducted on 13 Sept 2023) |
| --- | --- | --- |
| 1 | Search tuberculosis | 54221 |
| 2 | Search "tuberculosis"[MeSH] | 25826 |
| 3 | Search "treatment outcome" | 364574 |
| 4 | Search Treatment outcome | 507877 |
| 5 | Search treatment | 3458784 |
| 6 | Search outcome | 1333497 |
| 7 | Search DM | 35791 |
| 8 | Search "diabetes mellitus"[MeSH Terms] | 133718 |
| 9 | Search mellitus | 166042 |
| 10 | Search diabetes | 327881 |
| 11 | Risk Factor (Mesh) | 257510 |
| 12 | Risk factor (text word) | 112609 |
| 13 | Cohort | 501212 |
| 14 | Risk (mesh) | 348320 |
| 15 | Cohort studies | 882214 |
| 16 | Cohort study | 983563 |
| 17 | Cohort analysis | 939063 |
| 18 | 1 OR 2 | 54221 |
| 19 | 3 OR 4 OR 5 0R 6 | 3861884 |
| 20 | 7 OR 8 OR 9 OR 10 | 3480450 |
| 21 | 11 OR 12 OR 13 OR 14 | 829261 |
| 22 | 15 OR 16 OR 17 | 997414 |
| 23 | 21 OR 22 | 1294908 |
| 24 | 18 AND 19 AND 20 AND 23 | 609 |
| 25 | Human (Mesh) | 4565405 |
| 26 | #24 AND #25 | 532 |

**Search 1: Effect of glycemic control on TB treatment outcomes among TB-DM patients (COHORT)**

| **Search** | **Actions** | **Details** | **Query** | **Results** | **Time** |
| --- | --- | --- | --- | --- | --- |
| #26 |  |  | Search: **#24 AND #25** Filters: **from 2017/4/26 - 2023/8/21**  (((("tuberculosi"[All Fields] OR "tuberculosis"[MeSH Terms] OR "tuberculosis"[All Fields] OR "tuberculoses"[All Fields] OR "tuberculosis s"[All Fields]) AND 2017/04/26:2023/08/21[Date - Publication]) OR ("tuberculosis"[MeSH Terms] AND 2017/04/26:2023/08/21[Date - Publication])) AND 2017/04/26:2023/08/21[Date - Publication] AND ((("treatment outcome"[MeSH Terms] AND 2017/04/26:2023/08/21[Date - Publication]) OR (("treatment outcome"[MeSH Terms] OR ("treatment"[All Fields] AND "outcome"[All Fields]) OR "treatment outcome"[All Fields]) AND 2017/04/26:2023/08/21[Date - Publication]) OR (("therapeutics"[MeSH Terms] OR "therapeutics"[All Fields] OR "treatments"[All Fields] OR "therapy"[MeSH Subheading] OR "therapy"[All Fields] OR "treatment"[All Fields] OR "treatment s"[All Fields]) AND 2017/04/26:2023/08/21[Date - Publication]) OR (("outcome"[All Fields] OR "outcomes"[All Fields]) AND 2017/04/26:2023/08/21[Date - Publication])) AND 2017/04/26:2023/08/21[Date - Publication]) AND (((("dyn med"[Journal] OR "dis mon"[Journal] OR "dis manag"[Journal] OR "dm"[All Fields]) AND 2017/04/26:2023/08/21[Date - Publication]) OR ("diabetes mellitus"[MeSH Terms] AND 2017/04/26:2023/08/21[Date - Publication]) OR ("mellitus"[All Fields] AND 2017/04/26:2023/08/21[Date - Publication]) OR (("diabete"[All Fields] OR "diabetes mellitus"[MeSH Terms] OR ("diabetes"[All Fields] AND "mellitus"[All Fields]) OR "diabetes mellitus"[All Fields] OR "diabetes"[All Fields] OR "diabetes insipidus"[MeSH Terms] OR ("diabetes"[All Fields] AND "insipidus"[All Fields]) OR "diabetes insipidus"[All Fields] OR "diabetic"[All Fields] OR "diabetics"[All Fields] OR "diabets"[All Fields]) AND 2017/04/26:2023/08/21[Date - Publication])) AND 2017/04/26:2023/08/21[Date - Publication]) AND ((((("risk factors"[MeSH Terms] AND 2017/04/26:2023/08/21[Date - Publication]) OR ("risk factor"[Text Word] AND 2017/04/26:2023/08/21[Date - Publication]) OR (("cohort"[All Fields] OR "cohort s"[All Fields] OR "cohorte"[All Fields] OR "cohorts"[All Fields]) AND 2017/04/26:2023/08/21[Date - Publication]) OR ("risk"[MeSH Terms] AND 2017/04/26:2023/08/21[Date - Publication])) AND 2017/04/26:2023/08/21[Date - Publication]) OR (((("cohort studies"[MeSH Terms] OR ("cohort"[All Fields] AND "studies"[All Fields]) OR "cohort studies"[All Fields]) AND 2017/04/26:2023/08/21[Date - Publication]) OR (("cohort studies"[MeSH Terms] OR ("cohort"[All Fields] AND "studies"[All Fields]) OR "cohort studies"[All Fields] OR ("cohort"[All Fields] AND "study"[All Fields]) OR "cohort study"[All Fields]) AND 2017/04/26:2023/08/21[Date - Publication]) OR (("cohort studies"[MeSH Terms] OR ("cohort"[All Fields] AND "studies"[All Fields]) OR "cohort studies"[All Fields] OR ("cohort"[All Fields] AND "analysis"[All Fields]) OR "cohort analysis"[All Fields]) AND 2017/04/26:2023/08/21[Date - Publication])) AND 2017/04/26:2023/08/21[Date - Publication])) AND 2017/04/26:2023/08/21[Date - Publication]) AND 2017/04/26:2023/08/21[Date - Publication] AND ("humans"[MeSH Terms] AND 2017/04/26:2023/08/21[Date - Publication])) AND (2017/4/26:2023/8/21[pdat])  **Translations**  **Tuberculosis:** "tuberculosi"[All Fields] OR "tuberculosis"[MeSH Terms] OR "tuberculosis"[All Fields] OR "tuberculoses"[All Fields] OR "tuberculosis's"[All Fields]  **tuberculosis [mesh]:** "tuberculosis"[MeSH Terms]  **treatment outcome [Mesh]:** "treatment outcome"[MeSH Terms]  **Treatment outcome:** "treatment outcome"[MeSH Terms] OR ("treatment"[All Fields] AND "outcome"[All Fields]) OR "treatment outcome"[All Fields]  **Treatment:** "therapeutics"[MeSH Terms] OR "therapeutics"[All Fields] OR "treatments"[All Fields] OR "therapy"[Subheading] OR "therapy"[All Fields] OR "treatment"[All Fields] OR "treatment's"[All Fields]  **outcome:** "outcome"[All Fields] OR "outcomes"[All Fields]  **DM:** "Dyn Med"[Journal:__jid101152197] OR "Dis Mon"[Journal:__jid0370657] OR "Dis Manag"[Journal:__jid9802539] OR "dm"[All Fields]  **Diabetes mellitus [Mesh]:** "diabetes mellitus"[MeSH Terms]  **Diabetes:** "diabete"[All Fields] OR "diabetes mellitus"[MeSH Terms] OR ("diabetes"[All Fields] AND "mellitus"[All Fields]) OR "diabetes mellitus"[All Fields] OR "diabetes"[All Fields] OR "diabetes insipidus"[MeSH Terms] OR ("diabetes"[All Fields] AND "insipidus"[All Fields]) OR "diabetes insipidus"[All Fields] OR "diabetic"[All Fields] OR "diabetics"[All Fields] OR "diabets"[All Fields]  **risk factors [mesh]:** "risk factors"[MeSH Terms]  **cohort:** "cohort"[All Fields] OR "cohort's"[All Fields] OR "cohorte"[All Fields] OR "cohorts"[All Fields]  **risk [Mesh]:** "risk"[MeSH Terms]  **cohort studies:** "cohort studies"[MeSH Terms] OR ("cohort"[All Fields] AND "studies"[All Fields]) OR "cohort studies"[All Fields]  **cohort study:** "cohort studies"[MeSH Terms] OR ("cohort"[All Fields] AND "studies"[All Fields]) OR "cohort studies"[All Fields] OR ("cohort"[All Fields] AND "study"[All Fields]) OR "cohort study"[All Fields]  **cohort analysis:** "cohort studies"[MeSH Terms] OR ("cohort"[All Fields] AND "studies"[All Fields]) OR "cohort studies"[All Fields] OR ("cohort"[All Fields] AND "analysis"[All Fields]) OR "cohort analysis"[All Fields]  **Human [Mesh]:** "humans"[MeSH Terms] | [532](https://pubmed.ncbi.nlm.nih.gov/?term=%2324+AND+%2325&sort=&filter=dates.2017%2F4%2F26-2023%2F8%2F21&size=200) | 01:02:10 |
| #25 |  |  | Search: **Human [Mesh]** Filters: **from 2017/4/26 - 2023/8/21**  ("humans"[MeSH Terms]) AND (2017/4/26:2023/8/21[pdat])  **Translations**  **Human [Mesh]:** "humans"[MeSH Terms] | [4,565,405](https://pubmed.ncbi.nlm.nih.gov/?term=Human+%5BMesh%5D&sort=&filter=dates.2017%2F4%2F26-2023%2F8%2F21&size=200) | 01:01:39 |
| #24 |  |  | Search: **#18 AND #19 AND #20 AND #23** Filters: **from 2017/4/26 - 2023/8/21**  (((("tuberculosi"[All Fields] OR "tuberculosis"[MeSH Terms] OR "tuberculosis"[All Fields] OR "tuberculoses"[All Fields] OR "tuberculosis s"[All Fields]) AND 2017/04/26:2023/08/21[Date - Publication]) OR ("tuberculosis"[MeSH Terms] AND 2017/04/26:2023/08/21[Date - Publication])) AND 2017/04/26:2023/08/21[Date - Publication] AND ((("treatment outcome"[MeSH Terms] AND 2017/04/26:2023/08/21[Date - Publication]) OR (("treatment outcome"[MeSH Terms] OR ("treatment"[All Fields] AND "outcome"[All Fields]) OR "treatment outcome"[All Fields]) AND 2017/04/26:2023/08/21[Date - Publication]) OR (("therapeutics"[MeSH Terms] OR "therapeutics"[All Fields] OR "treatments"[All Fields] OR "therapy"[MeSH Subheading] OR "therapy"[All Fields] OR "treatment"[All Fields] OR "treatment s"[All Fields]) AND 2017/04/26:2023/08/21[Date - Publication]) OR (("outcome"[All Fields] OR "outcomes"[All Fields]) AND 2017/04/26:2023/08/21[Date - Publication])) AND 2017/04/26:2023/08/21[Date - Publication]) AND (((("dyn med"[Journal] OR "dis mon"[Journal] OR "dis manag"[Journal] OR "dm"[All Fields]) AND 2017/04/26:2023/08/21[Date - Publication]) OR ("diabetes mellitus"[MeSH Terms] AND 2017/04/26:2023/08/21[Date - Publication]) OR ("mellitus"[All Fields] AND 2017/04/26:2023/08/21[Date - Publication]) OR (("diabete"[All Fields] OR "diabetes mellitus"[MeSH Terms] OR ("diabetes"[All Fields] AND "mellitus"[All Fields]) OR "diabetes mellitus"[All Fields] OR "diabetes"[All Fields] OR "diabetes insipidus"[MeSH Terms] OR ("diabetes"[All Fields] AND "insipidus"[All Fields]) OR "diabetes insipidus"[All Fields] OR "diabetic"[All Fields] OR "diabetics"[All Fields] OR "diabets"[All Fields]) AND 2017/04/26:2023/08/21[Date - Publication])) AND 2017/04/26:2023/08/21[Date - Publication]) AND ((((("risk factors"[MeSH Terms] AND 2017/04/26:2023/08/21[Date - Publication]) OR ("risk factor"[Text Word] AND 2017/04/26:2023/08/21[Date - Publication]) OR (("cohort"[All Fields] OR "cohort s"[All Fields] OR "cohorte"[All Fields] OR "cohorts"[All Fields]) AND 2017/04/26:2023/08/21[Date - Publication]) OR ("risk"[MeSH Terms] AND 2017/04/26:2023/08/21[Date - Publication])) AND 2017/04/26:2023/08/21[Date - Publication]) OR (((("cohort studies"[MeSH Terms] OR ("cohort"[All Fields] AND "studies"[All Fields]) OR "cohort studies"[All Fields]) AND 2017/04/26:2023/08/21[Date - Publication]) OR (("cohort studies"[MeSH Terms] OR ("cohort"[All Fields] AND "studies"[All Fields]) OR "cohort studies"[All Fields] OR ("cohort"[All Fields] AND "study"[All Fields]) OR "cohort study"[All Fields]) AND 2017/04/26:2023/08/21[Date - Publication]) OR (("cohort studies"[MeSH Terms] OR ("cohort"[All Fields] AND "studies"[All Fields]) OR "cohort studies"[All Fields] OR ("cohort"[All Fields] AND "analysis"[All Fields]) OR "cohort analysis"[All Fields]) AND 2017/04/26:2023/08/21[Date - Publication])) AND 2017/04/26:2023/08/21[Date - Publication])) AND 2017/04/26:2023/08/21[Date - Publication])) AND (2017/4/26:2023/8/21[pdat])  **Translations**  **Tuberculosis:** "tuberculosi"[All Fields] OR "tuberculosis"[MeSH Terms] OR "tuberculosis"[All Fields] OR "tuberculoses"[All Fields] OR "tuberculosis's"[All Fields]  **tuberculosis [mesh]:** "tuberculosis"[MeSH Terms]  **treatment outcome [Mesh]:** "treatment outcome"[MeSH Terms]  **Treatment outcome:** "treatment outcome"[MeSH Terms] OR ("treatment"[All Fields] AND "outcome"[All Fields]) OR "treatment outcome"[All Fields]  **Treatment:** "therapeutics"[MeSH Terms] OR "therapeutics"[All Fields] OR "treatments"[All Fields] OR "therapy"[Subheading] OR "therapy"[All Fields] OR "treatment"[All Fields] OR "treatment's"[All Fields]  **outcome:** "outcome"[All Fields] OR "outcomes"[All Fields]  **DM:** "Dyn Med"[Journal:__jid101152197] OR "Dis Mon"[Journal:__jid0370657] OR "Dis Manag"[Journal:__jid9802539] OR "dm"[All Fields]  **Diabetes mellitus [Mesh]:** "diabetes mellitus"[MeSH Terms]  **Diabetes:** "diabete"[All Fields] OR "diabetes mellitus"[MeSH Terms] OR ("diabetes"[All Fields] AND "mellitus"[All Fields]) OR "diabetes mellitus"[All Fields] OR "diabetes"[All Fields] OR "diabetes insipidus"[MeSH Terms] OR ("diabetes"[All Fields] AND "insipidus"[All Fields]) OR "diabetes insipidus"[All Fields] OR "diabetic"[All Fields] OR "diabetics"[All Fields] OR "diabets"[All Fields]  **risk factors [mesh]:** "risk factors"[MeSH Terms]  **cohort:** "cohort"[All Fields] OR "cohort's"[All Fields] OR "cohorte"[All Fields] OR "cohorts"[All Fields]  **risk [Mesh]:** "risk"[MeSH Terms]  **cohort studies:** "cohort studies"[MeSH Terms] OR ("cohort"[All Fields] AND "studies"[All Fields]) OR "cohort studies"[All Fields]  **cohort study:** "cohort studies"[MeSH Terms] OR ("cohort"[All Fields] AND "studies"[All Fields]) OR "cohort studies"[All Fields] OR ("cohort"[All Fields] AND "study"[All Fields]) OR "cohort study"[All Fields]  **cohort analysis:** "cohort studies"[MeSH Terms] OR ("cohort"[All Fields] AND "studies"[All Fields]) OR "cohort studies"[All Fields] OR ("cohort"[All Fields] AND "analysis"[All Fields]) OR "cohort analysis"[All Fields] | [609](https://pubmed.ncbi.nlm.nih.gov/?term=%2318+AND+%2319+AND+%2320+AND+%2323&sort=&filter=dates.2017%2F4%2F26-2023%2F8%2F21&size=200) | 00:59:59 |
| #23 |  |  | Search: **#21 OR #22** Filters: **from 2017/4/26 - 2023/8/21**  (((("risk factors"[MeSH Terms] AND 2017/04/26:2023/08/21[Date - Publication]) OR ("risk factor"[Text Word] AND 2017/04/26:2023/08/21[Date - Publication]) OR (("cohort"[All Fields] OR "cohort s"[All Fields] OR "cohorte"[All Fields] OR "cohorts"[All Fields]) AND 2017/04/26:2023/08/21[Date - Publication]) OR ("risk"[MeSH Terms] AND 2017/04/26:2023/08/21[Date - Publication])) AND 2017/04/26:2023/08/21[Date - Publication]) OR (((("cohort studies"[MeSH Terms] OR ("cohort"[All Fields] AND "studies"[All Fields]) OR "cohort studies"[All Fields]) AND 2017/04/26:2023/08/21[Date - Publication]) OR (("cohort studies"[MeSH Terms] OR ("cohort"[All Fields] AND "studies"[All Fields]) OR "cohort studies"[All Fields] OR ("cohort"[All Fields] AND "study"[All Fields]) OR "cohort study"[All Fields]) AND 2017/04/26:2023/08/21[Date - Publication]) OR (("cohort studies"[MeSH Terms] OR ("cohort"[All Fields] AND "studies"[All Fields]) OR "cohort studies"[All Fields] OR ("cohort"[All Fields] AND "analysis"[All Fields]) OR "cohort analysis"[All Fields]) AND 2017/04/26:2023/08/21[Date - Publication])) AND 2017/04/26:2023/08/21[Date - Publication])) AND (2017/4/26:2023/8/21[pdat])  **Translations**  **risk factors [mesh]:** "risk factors"[MeSH Terms]  **cohort:** "cohort"[All Fields] OR "cohort's"[All Fields] OR "cohorte"[All Fields] OR "cohorts"[All Fields]  **risk [Mesh]:** "risk"[MeSH Terms]  **cohort studies:** "cohort studies"[MeSH Terms] OR ("cohort"[All Fields] AND "studies"[All Fields]) OR "cohort studies"[All Fields]  **cohort study:** "cohort studies"[MeSH Terms] OR ("cohort"[All Fields] AND "studies"[All Fields]) OR "cohort studies"[All Fields] OR ("cohort"[All Fields] AND "study"[All Fields]) OR "cohort study"[All Fields]  **cohort analysis:** "cohort studies"[MeSH Terms] OR ("cohort"[All Fields] AND "studies"[All Fields]) OR "cohort studies"[All Fields] OR ("cohort"[All Fields] AND "analysis"[All Fields]) OR "cohort analysis"[All Fields] | [1,294,908](https://pubmed.ncbi.nlm.nih.gov/?term=%2321+OR+%2322&sort=&filter=dates.2017%2F4%2F26-2023%2F8%2F21&size=200) | 00:59:13 |
| #22 |  |  | Search: **#15 OR #16 OR #17** Filters: **from 2017/4/26 - 2023/8/21**  ((("cohort studies"[MeSH Terms] OR ("cohort"[All Fields] AND "studies"[All Fields]) OR "cohort studies"[All Fields]) AND 2017/04/26:2023/08/21[Date - Publication]) OR (("cohort studies"[MeSH Terms] OR ("cohort"[All Fields] AND "studies"[All Fields]) OR "cohort studies"[All Fields] OR ("cohort"[All Fields] AND "study"[All Fields]) OR "cohort study"[All Fields]) AND 2017/04/26:2023/08/21[Date - Publication]) OR (("cohort studies"[MeSH Terms] OR ("cohort"[All Fields] AND "studies"[All Fields]) OR "cohort studies"[All Fields] OR ("cohort"[All Fields] AND "analysis"[All Fields]) OR "cohort analysis"[All Fields]) AND 2017/04/26:2023/08/21[Date - Publication])) AND (2017/4/26:2023/8/21[pdat])  **Translations**  **cohort studies:** "cohort studies"[MeSH Terms] OR ("cohort"[All Fields] AND "studies"[All Fields]) OR "cohort studies"[All Fields]  **cohort study:** "cohort studies"[MeSH Terms] OR ("cohort"[All Fields] AND "studies"[All Fields]) OR "cohort studies"[All Fields] OR ("cohort"[All Fields] AND "study"[All Fields]) OR "cohort study"[All Fields]  **cohort analysis:** "cohort studies"[MeSH Terms] OR ("cohort"[All Fields] AND "studies"[All Fields]) OR "cohort studies"[All Fields] OR ("cohort"[All Fields] AND "analysis"[All Fields]) OR "cohort analysis"[All Fields] | [997,414](https://pubmed.ncbi.nlm.nih.gov/?term=%2315+OR+%2316+OR+%2317&sort=&filter=dates.2017%2F4%2F26-2023%2F8%2F21&size=200) | 00:58:51 |
| #21 |  |  | Search: **#11 OR #12 OR #13 OR #14** Filters: **from 2017/4/26 - 2023/8/21**  (("risk factors"[MeSH Terms] AND 2017/04/26:2023/08/21[Date - Publication]) OR ("risk factor"[Text Word] AND 2017/04/26:2023/08/21[Date - Publication]) OR (("cohort"[All Fields] OR "cohort s"[All Fields] OR "cohorte"[All Fields] OR "cohorts"[All Fields]) AND 2017/04/26:2023/08/21[Date - Publication]) OR ("risk"[MeSH Terms] AND 2017/04/26:2023/08/21[Date - Publication])) AND (2017/4/26:2023/8/21[pdat])  **Translations**  **risk factors [mesh]:** "risk factors"[MeSH Terms]  **cohort:** "cohort"[All Fields] OR "cohort's"[All Fields] OR "cohorte"[All Fields] OR "cohorts"[All Fields]  **risk [Mesh]:** "risk"[MeSH Terms] | [829,261](https://pubmed.ncbi.nlm.nih.gov/?term=%2311+OR+%2312+OR+%2313+OR+%2314&sort=&filter=dates.2017%2F4%2F26-2023%2F8%2F21&size=200) | 00:58:14 |
| #20 |  |  | Search: **#7 OR #8 OR #9 OR #10** Filters: **from 2017/4/26 - 2023/8/21**  ((("dyn med"[Journal] OR "dis mon"[Journal] OR "dis manag"[Journal] OR "dm"[All Fields]) AND 2017/04/26:2023/08/21[Date - Publication]) OR ("diabetes mellitus"[MeSH Terms] AND 2017/04/26:2023/08/21[Date - Publication]) OR ("mellitus"[All Fields] AND 2017/04/26:2023/08/21[Date - Publication]) OR (("diabete"[All Fields] OR "diabetes mellitus"[MeSH Terms] OR ("diabetes"[All Fields] AND "mellitus"[All Fields]) OR "diabetes mellitus"[All Fields] OR "diabetes"[All Fields] OR "diabetes insipidus"[MeSH Terms] OR ("diabetes"[All Fields] AND "insipidus"[All Fields]) OR "diabetes insipidus"[All Fields] OR "diabetic"[All Fields] OR "diabetics"[All Fields] OR "diabets"[All Fields]) AND 2017/04/26:2023/08/21[Date - Publication])) AND (2017/4/26:2023/8/21[pdat])  **Translations**  **DM:** "Dyn Med"[Journal:__jid101152197] OR "Dis Mon"[Journal:__jid0370657] OR "Dis Manag"[Journal:__jid9802539] OR "dm"[All Fields]  **Diabetes mellitus [Mesh]:** "diabetes mellitus"[MeSH Terms]  **Diabetes:** "diabete"[All Fields] OR "diabetes mellitus"[MeSH Terms] OR ("diabetes"[All Fields] AND "mellitus"[All Fields]) OR "diabetes mellitus"[All Fields] OR "diabetes"[All Fields] OR "diabetes insipidus"[MeSH Terms] OR ("diabetes"[All Fields] AND "insipidus"[All Fields]) OR "diabetes insipidus"[All Fields] OR "diabetic"[All Fields] OR "diabetics"[All Fields] OR "diabets"[All Fields] | [348,050](https://pubmed.ncbi.nlm.nih.gov/?term=%237+OR+%238+OR+%239+OR+%2310&sort=&filter=dates.2017%2F4%2F26-2023%2F8%2F21&size=200) | 00:57:33 |
| #19 |  |  | Search: **#3 OR #4 OR #5 OR #6** Filters: **from 2017/4/26 - 2023/8/21**  (("treatment outcome"[MeSH Terms] AND 2017/04/26:2023/08/21[Date - Publication]) OR (("treatment outcome"[MeSH Terms] OR ("treatment"[All Fields] AND "outcome"[All Fields]) OR "treatment outcome"[All Fields]) AND 2017/04/26:2023/08/21[Date - Publication]) OR (("therapeutics"[MeSH Terms] OR "therapeutics"[All Fields] OR "treatments"[All Fields] OR "therapy"[MeSH Subheading] OR "therapy"[All Fields] OR "treatment"[All Fields] OR "treatment s"[All Fields]) AND 2017/04/26:2023/08/21[Date - Publication]) OR (("outcome"[All Fields] OR "outcomes"[All Fields]) AND 2017/04/26:2023/08/21[Date - Publication])) AND (2017/4/26:2023/8/21[pdat])  **Translations**  **treatment outcome [Mesh]:** "treatment outcome"[MeSH Terms]  **Treatment outcome:** "treatment outcome"[MeSH Terms] OR ("treatment"[All Fields] AND "outcome"[All Fields]) OR "treatment outcome"[All Fields]  **Treatment:** "therapeutics"[MeSH Terms] OR "therapeutics"[All Fields] OR "treatments"[All Fields] OR "therapy"[Subheading] OR "therapy"[All Fields] OR "treatment"[All Fields] OR "treatment's"[All Fields]  **outcome:** "outcome"[All Fields] OR "outcomes"[All Fields] | [3,861,884](https://pubmed.ncbi.nlm.nih.gov/?term=%233+OR+%234+OR+%235+OR+%236&sort=&filter=dates.2017%2F4%2F26-2023%2F8%2F21&size=200) | 00:56:48 |
| #18 |  |  | Search: **#1 OR #2** Filters: **from 2017/4/26 - 2023/8/21**  ((("tuberculosi"[All Fields] OR "tuberculosis"[MeSH Terms] OR "tuberculosis"[All Fields] OR "tuberculoses"[All Fields] OR "tuberculosis s"[All Fields]) AND 2017/04/26:2023/08/21[Date - Publication]) OR ("tuberculosis"[MeSH Terms] AND 2017/04/26:2023/08/21[Date - Publication])) AND (2017/4/26:2023/8/21[pdat])  **Translations**  **Tuberculosis:** "tuberculosi"[All Fields] OR "tuberculosis"[MeSH Terms] OR "tuberculosis"[All Fields] OR "tuberculoses"[All Fields] OR "tuberculosis's"[All Fields]  **tuberculosis [mesh]:** "tuberculosis"[MeSH Terms] | [54,221](https://pubmed.ncbi.nlm.nih.gov/?term=%231+OR+%232&sort=&filter=dates.2017%2F4%2F26-2023%2F8%2F21&size=200) | 00:56:06 |
| #17 |  |  | Search: **cohort analysis** Filters: **from 2017/4/26 - 2023/8/21**  ("cohort studies"[MeSH Terms] OR ("cohort"[All Fields] AND "studies"[All Fields]) OR "cohort studies"[All Fields] OR ("cohort"[All Fields] AND "analysis"[All Fields]) OR "cohort analysis"[All Fields]) AND (2017/4/26:2023/8/21[pdat])  **Translations**  **cohort analysis:** "cohort studies"[MeSH Terms] OR ("cohort"[All Fields] AND "studies"[All Fields]) OR "cohort studies"[All Fields] OR ("cohort"[All Fields] AND "analysis"[All Fields]) OR "cohort analysis"[All Fields] | [939,063](https://pubmed.ncbi.nlm.nih.gov/?term=cohort+analysis&sort=&filter=dates.2017%2F4%2F26-2023%2F8%2F21&size=200) | 00:55:06 |
| #16 |  |  | Search: **cohort study** Filters: **from 2017/4/26 - 2023/8/21**  ("cohort studies"[MeSH Terms] OR ("cohort"[All Fields] AND "studies"[All Fields]) OR "cohort studies"[All Fields] OR ("cohort"[All Fields] AND "study"[All Fields]) OR "cohort study"[All Fields]) AND (2017/4/26:2023/8/21[pdat])  **Translations**  **cohort study:** "cohort studies"[MeSH Terms] OR ("cohort"[All Fields] AND "studies"[All Fields]) OR "cohort studies"[All Fields] OR ("cohort"[All Fields] AND "study"[All Fields]) OR "cohort study"[All Fields] | [983,563](https://pubmed.ncbi.nlm.nih.gov/?term=cohort+study&sort=&filter=dates.2017%2F4%2F26-2023%2F8%2F21&size=200) | 00:54:48 |
| #15 |  |  | Search: **cohort studies** Filters: **from 2017/4/26 - 2023/8/21**  ("cohort studies"[MeSH Terms] OR ("cohort"[All Fields] AND "studies"[All Fields]) OR "cohort studies"[All Fields]) AND (2017/4/26:2023/8/21[pdat])  **Translations**  **cohort studies:** "cohort studies"[MeSH Terms] OR ("cohort"[All Fields] AND "studies"[All Fields]) OR "cohort studies"[All Fields] | [882,214](https://pubmed.ncbi.nlm.nih.gov/?term=cohort+studies&sort=&filter=dates.2017%2F4%2F26-2023%2F8%2F21&size=200) | 00:54:30 |
| #14 |  |  | Search: **risk [Mesh]** Filters: **from 2017/4/26 - 2023/8/21**  ("risk"[MeSH Terms]) AND (2017/4/26:2023/8/21[pdat])  **Translations**  **risk [Mesh]:** "risk"[MeSH Terms] | [348,320](https://pubmed.ncbi.nlm.nih.gov/?term=risk+%5BMesh%5D&sort=&filter=dates.2017%2F4%2F26-2023%2F8%2F21&size=200) | 00:54:07 |
| #13 |  |  | Search: **cohort** Filters: **from 2017/4/26 - 2023/8/21**  ("cohort"[All Fields] OR "cohort s"[All Fields] OR "cohorte"[All Fields] OR "cohorts"[All Fields]) AND (2017/4/26:2023/8/21[pdat])  **Translations**  **cohort:** "cohort"[All Fields] OR "cohort's"[All Fields] OR "cohorte"[All Fields] OR "cohorts"[All Fields] | [501,212](https://pubmed.ncbi.nlm.nih.gov/?term=cohort&sort=&filter=dates.2017%2F4%2F26-2023%2F8%2F21&size=200) | 00:53:24 |
| #12 |  |  | Search: **risk factor[Text Word]** Filters: **from 2017/4/26 - 2023/8/21**  ("risk factor"[Text Word]) AND (2017/4/26:2023/8/21[pdat]) | [112,609](https://pubmed.ncbi.nlm.nih.gov/?term=risk+factor%5BText+Word%5D&sort=&filter=dates.2017%2F4%2F26-2023%2F8%2F21&size=200) | 00:53:05 |
| #11 |  |  | Search: **risk factors [mesh]** Filters: **from 2017/4/26 - 2023/8/21**  ("risk factors"[MeSH Terms]) AND (2017/4/26:2023/8/21[pdat])  **Translations**  **risk factors [mesh]:** "risk factors"[MeSH Terms] | [257,510](https://pubmed.ncbi.nlm.nih.gov/?term=risk+factors+%5Bmesh%5D&filter=dates.2017%2F4%2F26-2023%2F8%2F21&size=200&sort=relevance) | 00:51:30 |
| #10 |  |  | Search: **Diabetes** Filters: **from 2017/4/26 - 2023/8/21**  ("diabete"[All Fields] OR "diabetes mellitus"[MeSH Terms] OR ("diabetes"[All Fields] AND "mellitus"[All Fields]) OR "diabetes mellitus"[All Fields] OR "diabetes"[All Fields] OR "diabetes insipidus"[MeSH Terms] OR ("diabetes"[All Fields] AND "insipidus"[All Fields]) OR "diabetes insipidus"[All Fields] OR "diabetic"[All Fields] OR "diabetics"[All Fields] OR "diabets"[All Fields]) AND (2017/4/26:2023/8/21[pdat])  **Translations**  **Diabetes:** "diabete"[All Fields] OR "diabetes mellitus"[MeSH Terms] OR ("diabetes"[All Fields] AND "mellitus"[All Fields]) OR "diabetes mellitus"[All Fields] OR "diabetes"[All Fields] OR "diabetes insipidus"[MeSH Terms] OR ("diabetes"[All Fields] AND "insipidus"[All Fields]) OR "diabetes insipidus"[All Fields] OR "diabetic"[All Fields] OR "diabetics"[All Fields] OR "diabets"[All Fields] | [327,881](https://pubmed.ncbi.nlm.nih.gov/?term=Diabetes&filter=dates.2017%2F4%2F26-2023%2F8%2F21&size=200&sort=relevance) | 00:51:05 |
| #9 |  |  | Search: **mellitus** Filters: **from 2017/4/26 - 2023/8/21**  ("mellitus"[All Fields]) AND (2017/4/26:2023/8/21[pdat]) | [166,042](https://pubmed.ncbi.nlm.nih.gov/?term=mellitus&sort=&filter=dates.2017%2F4%2F26-2023%2F8%2F21&size=200) | 00:50:44 |
| #8 |  |  | Search: **Diabetes mellitus [Mesh]** Filters: **from 2017/4/26 - 2023/8/21**  ("diabetes mellitus"[MeSH Terms]) AND (2017/4/26:2023/8/21[pdat])  **Translations**  **Diabetes mellitus [Mesh]:** "diabetes mellitus"[MeSH Terms] | [133,718](https://pubmed.ncbi.nlm.nih.gov/?term=Diabetes+mellitus+%5BMesh%5D&filter=dates.2017%2F4%2F26-2023%2F8%2F21&size=200&sort=relevance) | 00:50:23 |
| #7 |  |  | Search: **DM** Filters: **from 2017/4/26 - 2023/8/21**  ("dyn med"[Journal] OR "dis mon"[Journal] OR "dis manag"[Journal] OR "dm"[All Fields]) AND (2017/4/26:2023/8/21[pdat])  **Translations**  **DM:** "Dyn Med"[Journal:__jid101152197] OR "Dis Mon"[Journal:__jid0370657] OR "Dis Manag"[Journal:__jid9802539] OR "dm"[All Fields] | [35,791](https://pubmed.ncbi.nlm.nih.gov/?term=DM&sort=&filter=dates.2017%2F4%2F26-2023%2F8%2F21&size=200) | 00:49:54 |
| #6 |  |  | Search: **outcome** Filters: **from 2017/4/26 - 2023/8/21**  ("outcome"[All Fields] OR "outcomes"[All Fields]) AND (2017/4/26:2023/8/21[pdat])  **Translations**  **outcome:** "outcome"[All Fields] OR "outcomes"[All Fields] | [1,333,500](https://pubmed.ncbi.nlm.nih.gov/?term=outcome&sort=&filter=dates.2017%2F4%2F26-2023%2F8%2F21&size=200) | 00:49:38 |
| #5 |  |  | Search: **Treatment** Filters: **from 2017/4/26 - 2023/8/21**  ("therapeutics"[MeSH Terms] OR "therapeutics"[All Fields] OR "treatments"[All Fields] OR "therapy"[MeSH Subheading] OR "therapy"[All Fields] OR "treatment"[All Fields] OR "treatment s"[All Fields]) AND (2017/4/26:2023/8/21[pdat])  **Translations**  **Treatment:** "therapeutics"[MeSH Terms] OR "therapeutics"[All Fields] OR "treatments"[All Fields] OR "therapy"[Subheading] OR "therapy"[All Fields] OR "treatment"[All Fields] OR "treatment's"[All Fields] | [3,458,784](https://pubmed.ncbi.nlm.nih.gov/?term=Treatment&sort=&filter=dates.2017%2F4%2F26-2023%2F8%2F21&size=200) | 00:49:13 |
| #4 |  |  | Search: **Treatment outcome** Filters: **from 2017/4/26 - 2023/8/21**  ("treatment outcome"[MeSH Terms] OR ("treatment"[All Fields] AND "outcome"[All Fields]) OR "treatment outcome"[All Fields]) AND (2017/4/26:2023/8/21[pdat])  **Translations**  **Treatment outcome:** "treatment outcome"[MeSH Terms] OR ("treatment"[All Fields] AND "outcome"[All Fields]) OR "treatment outcome"[All Fields] | [507,877](https://pubmed.ncbi.nlm.nih.gov/?term=Treatment+outcome&sort=&filter=dates.2017%2F4%2F26-2023%2F8%2F21&size=200) | 00:48:45 |
| #3 |  |  | Search: **treatment outcome [Mesh]** Filters: **from 2017/4/26 - 2023/8/21**  ("treatment outcome"[MeSH Terms]) AND (2017/4/26:2023/8/21[pdat])  **Translations**  **treatment outcome [Mesh]:** "treatment outcome"[MeSH Terms] | [364,574](https://pubmed.ncbi.nlm.nih.gov/?term=treatment+outcome+%5BMesh%5D&sort=&filter=dates.2017%2F4%2F26-2023%2F8%2F21&size=200) | 00:48:18 |
| #2 |  |  | Search: **tuberculosis [mesh]** Filters: **from 2017/4/26 - 2023/8/21**  ("tuberculosis"[MeSH Terms]) AND (2017/4/26:2023/8/21[pdat])  **Translations**  **tuberculosis [mesh]:** "tuberculosis"[MeSH Terms] | [25,826](https://pubmed.ncbi.nlm.nih.gov/?term=tuberculosis+%5Bmesh%5D&sort=&filter=dates.2017%2F4%2F26-2023%2F8%2F21&size=200) | 00:47:41 |
| #1 |  |  | Search: **Tuberculosis** Filters: **from 2017/4/26 - 2023/8/21**  ("tuberculosi"[All Fields] OR "tuberculosis"[MeSH Terms] OR "tuberculosis"[All Fields] OR "tuberculoses"[All Fields] OR "tuberculosis s"[All Fields]) AND (2017/4/26:2023/8/21[pdat])  **Translations**  **Tuberculosis:** "tuberculosi"[All Fields] OR "tuberculosis"[MeSH Terms] OR "tuberculosis"[All Fields] OR "tuberculoses"[All Fields] OR "tuberculosis's"[All Fields] | [54,221](https://pubmed.ncbi.nlm.nih.gov/?term=Tuberculosis&sort=&filter=dates.2017%2F4%2F26-2023%2F8%2F21&size=200) | 00:46:47 |

**Search 2: Effect of glycemic control on TB treatment outcomes among TB-DM (RCT)**

| Search | Query | Items found (Search conducted on 13 Sept 2023) |
| --- | --- | --- |
| 1 | Search tuberculosis | 54221 |
| 2 | Search "tuberculosis"[MeSH Terms] | 25826 |
| 3 | Search "treatment outcome"[Mesh] | 364574 |
| 4 | Search "Treatment Outcome" | 507877 |
| 5 | Search treatment | 3458784 |
| 6 | Search outcome | 1333500 |
| 7 | Search DM | 35791 |
| 8 | Search "diabetes mellitus"[MeSH Terms] | 133718 |
| 9 | Search mellitus | 166042 |
| 10 | Search diabetes | 327881 |
| 11 | Trial (all fields) | 650776 |
| 12 | Randomized controlled trial (publication type) | 149173 |
| 13 | Randomized controlled trial as topic (mesh) | 52187 |
| 14 | Randomized controlled trial (all fields) | 229506 |
| 15 | 1 OR 2 | 54221 |
| 16 | 3 OR 4 OR 5 0R 6 | 3861884 |
| 17 | 7 OR 8 OR 9 OR 10 | 318050 |
| 18 | 11 OR 12 OR 13 OR 14 | 650775 |
| 19 | 15 AND 16 AND 17 AND 18 | 166 |
| 20 | Human (mesh) | 4565405 |
| 21 | #19 AND #20 | 128 |

| **Search** | **Actions** | **Details** | **Query** | **Results** | **Time** |
| --- | --- | --- | --- | --- | --- |
| #21 |  |  | Search: **#19 and #20** Filters: **from 2017/4/26 - 2023/8/21**  (((("tuberculosi"[All Fields] OR "tuberculosis"[MeSH Terms] OR "tuberculosis"[All Fields] OR "tuberculoses"[All Fields] OR "tuberculosis s"[All Fields]) AND 2017/04/26:2023/08/21[Date - Publication]) OR ("tuberculosis"[MeSH Terms] AND 2017/04/26:2023/08/21[Date - Publication])) AND 2017/04/26:2023/08/21[Date - Publication] AND ((("treatment outcome"[MeSH Terms] AND 2017/04/26:2023/08/21[Date - Publication]) OR (("treatment outcome"[MeSH Terms] OR ("treatment"[All Fields] AND "outcome"[All Fields]) OR "treatment outcome"[All Fields]) AND 2017/04/26:2023/08/21[Date - Publication]) OR (("therapeutics"[MeSH Terms] OR "therapeutics"[All Fields] OR "treatments"[All Fields] OR "therapy"[MeSH Subheading] OR "therapy"[All Fields] OR "treatment"[All Fields] OR "treatment s"[All Fields]) AND 2017/04/26:2023/08/21[Date - Publication]) OR (("outcome"[All Fields] OR "outcomes"[All Fields]) AND 2017/04/26:2023/08/21[Date - Publication])) AND 2017/04/26:2023/08/21[Date - Publication]) AND (((("dyn med"[Journal] OR "dis mon"[Journal] OR "dis manag"[Journal] OR "dm"[All Fields]) AND 2017/04/26:2023/08/21[Date - Publication]) OR ("diabetes mellitus"[MeSH Terms] AND 2017/04/26:2023/08/21[Date - Publication]) OR ("MELLITUS"[All Fields] AND 2017/04/26:2023/08/21[Date - Publication]) OR (("diabete"[All Fields] OR "diabetes mellitus"[MeSH Terms] OR ("diabetes"[All Fields] AND "MELLITUS"[All Fields]) OR "diabetes mellitus"[All Fields] OR "diabetes"[All Fields] OR "diabetes insipidus"[MeSH Terms] OR ("diabetes"[All Fields] AND "insipidus"[All Fields]) OR "diabetes insipidus"[All Fields] OR "diabetic"[All Fields] OR "diabetics"[All Fields] OR "diabets"[All Fields]) AND 2017/04/26:2023/08/21[Date - Publication])) AND 2017/04/26:2023/08/21[Date - Publication]) AND (((("clinical trials as topic"[MeSH Terms] OR ("clinical"[All Fields] AND "trials"[All Fields] AND "topic"[All Fields]) OR "clinical trials as topic"[All Fields] OR "TRIAL"[All Fields] OR "trial s"[All Fields] OR "trialed"[All Fields] OR "trialing"[All Fields] OR "trials"[All Fields]) AND 2017/04/26:2023/08/21[Date - Publication]) OR ("randomized controlled trial"[Publication Type] AND 2017/04/26:2023/08/21[Date - Publication]) OR ("randomized controlled trials as topic"[MeSH Terms] AND 2017/04/26:2023/08/21[Date - Publication]) OR (("randomized controlled trial"[Publication Type] OR "randomized controlled trials as topic"[MeSH Terms] OR "randomized controlled trial"[All Fields] OR "randomised controlled trial"[All Fields]) AND 2017/04/26:2023/08/21[Date - Publication])) AND 2017/04/26:2023/08/21[Date - Publication]) AND 2017/04/26:2023/08/21[Date - Publication] AND ("humans"[MeSH Terms] AND 2017/04/26:2023/08/21[Date - Publication])) AND (2017/4/26:2023/8/21[pdat])  **Translations**  **TUBERCULOSIS:** "tuberculosi"[All Fields] OR "tuberculosis"[MeSH Terms] OR "tuberculosis"[All Fields] OR "tuberculoses"[All Fields] OR "tuberculosis's"[All Fields]  **tUBERCULOSIS [MESH]:** "tuberculosis"[MeSH Terms]  **TREATMENT OUTCOME [MESH]:** "treatment outcome"[MeSH Terms]  **TREATMENT OUTCOME:** "treatment outcome"[MeSH Terms] OR ("treatment"[All Fields] AND "outcome"[All Fields]) OR "treatment outcome"[All Fields]  **TREATMENT:** "therapeutics"[MeSH Terms] OR "therapeutics"[All Fields] OR "treatments"[All Fields] OR "therapy"[Subheading] OR "therapy"[All Fields] OR "treatment"[All Fields] OR "treatment's"[All Fields]  **OUTCOME:** "outcome"[All Fields] OR "outcomes"[All Fields]  **DM:** "Dyn Med"[Journal:__jid101152197] OR "Dis Mon"[Journal:__jid0370657] OR "Dis Manag"[Journal:__jid9802539] OR "dm"[All Fields]  **DIABETES MELLITUS [MESH]:** "diabetes mellitus"[MeSH Terms]  **DIABETES:** "diabete"[All Fields] OR "diabetes mellitus"[MeSH Terms] OR ("diabetes"[All Fields] AND "mellitus"[All Fields]) OR "diabetes mellitus"[All Fields] OR "diabetes"[All Fields] OR "diabetes insipidus"[MeSH Terms] OR ("diabetes"[All Fields] AND "insipidus"[All Fields]) OR "diabetes insipidus"[All Fields] OR "diabetic"[All Fields] OR "diabetics"[All Fields] OR "diabets"[All Fields]  **TRIAL:** "clinical trials as topic"[MeSH Terms] OR ("clinical"[All Fields] AND "trials"[All Fields] AND "topic"[All Fields]) OR "clinical trials as topic"[All Fields] OR "trial"[All Fields] OR "trial's"[All Fields] OR "trialed"[All Fields] OR "trialing"[All Fields] OR "trials"[All Fields]  **RANDOMIZED CONTROLLED TRIALS [MESH]:** "randomized controlled trials as topic"[MeSH Terms]  **RANDOMIZED CONTROLLED TRIAL:** "randomized controlled trial"[Publication Type] OR "randomized controlled trials as topic"[MeSH Terms] OR "randomized controlled trial"[All Fields] OR "randomised controlled trial"[All Fields]  **HUMANS [MESH]:** "humans"[MeSH Terms] | [128](https://pubmed.ncbi.nlm.nih.gov/?term=%2319+and+%2320&sort=&filter=dates.2017%2F4%2F26-2023%2F8%2F21&size=200) | 02:30:39 |
| #20 |  |  | Search: **HUMANS [MESH]** Filters: **from 2017/4/26 - 2023/8/21**  ("humans"[MeSH Terms]) AND (2017/4/26:2023/8/21[pdat])  **Translations**  **HUMANS [MESH]:** "humans"[MeSH Terms] | [4,565,405](https://pubmed.ncbi.nlm.nih.gov/?term=HUMANS+%5BMESH%5D&sort=&filter=dates.2017%2F4%2F26-2023%2F8%2F21&size=200) | 02:28:27 |
| #19 |  |  | Search: **#15 AND #16 AND #17 AND #18** Filters: **from 2017/4/26 - 2023/8/21**  (((("tuberculosi"[All Fields] OR "tuberculosis"[MeSH Terms] OR "tuberculosis"[All Fields] OR "tuberculoses"[All Fields] OR "tuberculosis s"[All Fields]) AND 2017/04/26:2023/08/21[Date - Publication]) OR ("tuberculosis"[MeSH Terms] AND 2017/04/26:2023/08/21[Date - Publication])) AND 2017/04/26:2023/08/21[Date - Publication] AND ((("treatment outcome"[MeSH Terms] AND 2017/04/26:2023/08/21[Date - Publication]) OR (("treatment outcome"[MeSH Terms] OR ("treatment"[All Fields] AND "outcome"[All Fields]) OR "treatment outcome"[All Fields]) AND 2017/04/26:2023/08/21[Date - Publication]) OR (("therapeutics"[MeSH Terms] OR "therapeutics"[All Fields] OR "treatments"[All Fields] OR "therapy"[MeSH Subheading] OR "therapy"[All Fields] OR "treatment"[All Fields] OR "treatment s"[All Fields]) AND 2017/04/26:2023/08/21[Date - Publication]) OR (("outcome"[All Fields] OR "outcomes"[All Fields]) AND 2017/04/26:2023/08/21[Date - Publication])) AND 2017/04/26:2023/08/21[Date - Publication]) AND (((("dyn med"[Journal] OR "dis mon"[Journal] OR "dis manag"[Journal] OR "dm"[All Fields]) AND 2017/04/26:2023/08/21[Date - Publication]) OR ("diabetes mellitus"[MeSH Terms] AND 2017/04/26:2023/08/21[Date - Publication]) OR ("MELLITUS"[All Fields] AND 2017/04/26:2023/08/21[Date - Publication]) OR (("diabete"[All Fields] OR "diabetes mellitus"[MeSH Terms] OR ("diabetes"[All Fields] AND "MELLITUS"[All Fields]) OR "diabetes mellitus"[All Fields] OR "diabetes"[All Fields] OR "diabetes insipidus"[MeSH Terms] OR ("diabetes"[All Fields] AND "insipidus"[All Fields]) OR "diabetes insipidus"[All Fields] OR "diabetic"[All Fields] OR "diabetics"[All Fields] OR "diabets"[All Fields]) AND 2017/04/26:2023/08/21[Date - Publication])) AND 2017/04/26:2023/08/21[Date - Publication]) AND (((("clinical trials as topic"[MeSH Terms] OR ("clinical"[All Fields] AND "trials"[All Fields] AND "topic"[All Fields]) OR "clinical trials as topic"[All Fields] OR "TRIAL"[All Fields] OR "trial s"[All Fields] OR "trialed"[All Fields] OR "trialing"[All Fields] OR "trials"[All Fields]) AND 2017/04/26:2023/08/21[Date - Publication]) OR ("randomized controlled trial"[Publication Type] AND 2017/04/26:2023/08/21[Date - Publication]) OR ("randomized controlled trials as topic"[MeSH Terms] AND 2017/04/26:2023/08/21[Date - Publication]) OR (("randomized controlled trial"[Publication Type] OR "randomized controlled trials as topic"[MeSH Terms] OR "randomized controlled trial"[All Fields] OR "randomised controlled trial"[All Fields]) AND 2017/04/26:2023/08/21[Date - Publication])) AND 2017/04/26:2023/08/21[Date - Publication])) AND (2017/4/26:2023/8/21[pdat])  **Translations**  **TUBERCULOSIS:** "tuberculosi"[All Fields] OR "tuberculosis"[MeSH Terms] OR "tuberculosis"[All Fields] OR "tuberculoses"[All Fields] OR "tuberculosis's"[All Fields]  **tUBERCULOSIS [MESH]:** "tuberculosis"[MeSH Terms]  **TREATMENT OUTCOME [MESH]:** "treatment outcome"[MeSH Terms]  **TREATMENT OUTCOME:** "treatment outcome"[MeSH Terms] OR ("treatment"[All Fields] AND "outcome"[All Fields]) OR "treatment outcome"[All Fields]  **TREATMENT:** "therapeutics"[MeSH Terms] OR "therapeutics"[All Fields] OR "treatments"[All Fields] OR "therapy"[Subheading] OR "therapy"[All Fields] OR "treatment"[All Fields] OR "treatment's"[All Fields]  **OUTCOME:** "outcome"[All Fields] OR "outcomes"[All Fields]  **DM:** "Dyn Med"[Journal:__jid101152197] OR "Dis Mon"[Journal:__jid0370657] OR "Dis Manag"[Journal:__jid9802539] OR "dm"[All Fields]  **DIABETES MELLITUS [MESH]:** "diabetes mellitus"[MeSH Terms]  **DIABETES:** "diabete"[All Fields] OR "diabetes mellitus"[MeSH Terms] OR ("diabetes"[All Fields] AND "mellitus"[All Fields]) OR "diabetes mellitus"[All Fields] OR "diabetes"[All Fields] OR "diabetes insipidus"[MeSH Terms] OR ("diabetes"[All Fields] AND "insipidus"[All Fields]) OR "diabetes insipidus"[All Fields] OR "diabetic"[All Fields] OR "diabetics"[All Fields] OR "diabets"[All Fields]  **TRIAL:** "clinical trials as topic"[MeSH Terms] OR ("clinical"[All Fields] AND "trials"[All Fields] AND "topic"[All Fields]) OR "clinical trials as topic"[All Fields] OR "trial"[All Fields] OR "trial's"[All Fields] OR "trialed"[All Fields] OR "trialing"[All Fields] OR "trials"[All Fields]  **RANDOMIZED CONTROLLED TRIALS [MESH]:** "randomized controlled trials as topic"[MeSH Terms]  **RANDOMIZED CONTROLLED TRIAL:** "randomized controlled trial"[Publication Type] OR "randomized controlled trials as topic"[MeSH Terms] OR "randomized controlled trial"[All Fields] OR "randomised controlled trial"[All Fields] | [166](https://pubmed.ncbi.nlm.nih.gov/?term=%2315+AND+%2316+AND+%2317+AND+%2318&sort=&filter=dates.2017%2F4%2F26-2023%2F8%2F21&size=200) | 02:28:02 |
| #18 |  |  | Search: **#11 OR #12 OR #13 OR #14** Filters: **from 2017/4/26 - 2023/8/21**  ((("clinical trials as topic"[MeSH Terms] OR ("clinical"[All Fields] AND "trials"[All Fields] AND "topic"[All Fields]) OR "clinical trials as topic"[All Fields] OR "TRIAL"[All Fields] OR "trial s"[All Fields] OR "trialed"[All Fields] OR "trialing"[All Fields] OR "trials"[All Fields]) AND 2017/04/26:2023/08/21[Date - Publication]) OR ("randomized controlled trial"[Publication Type] AND 2017/04/26:2023/08/21[Date - Publication]) OR ("randomized controlled trials as topic"[MeSH Terms] AND 2017/04/26:2023/08/21[Date - Publication]) OR (("randomized controlled trial"[Publication Type] OR "randomized controlled trials as topic"[MeSH Terms] OR "randomized controlled trial"[All Fields] OR "randomised controlled trial"[All Fields]) AND 2017/04/26:2023/08/21[Date - Publication])) AND (2017/4/26:2023/8/21[pdat])  **Translations**  **TRIAL:** "clinical trials as topic"[MeSH Terms] OR ("clinical"[All Fields] AND "trials"[All Fields] AND "topic"[All Fields]) OR "clinical trials as topic"[All Fields] OR "trial"[All Fields] OR "trial's"[All Fields] OR "trialed"[All Fields] OR "trialing"[All Fields] OR "trials"[All Fields]  **RANDOMIZED CONTROLLED TRIALS [MESH]:** "randomized controlled trials as topic"[MeSH Terms]  **RANDOMIZED CONTROLLED TRIAL:** "randomized controlled trial"[Publication Type] OR "randomized controlled trials as topic"[MeSH Terms] OR "randomized controlled trial"[All Fields] OR "randomised controlled trial"[All Fields] | [650,777](https://pubmed.ncbi.nlm.nih.gov/?term=%2311+OR+%2312+OR+%2313+OR+%2314&sort=&filter=dates.2017%2F4%2F26-2023%2F8%2F21&size=200) | 02:27:26 |
| #17 |  |  | Search: **#7 OR #8 OR #9 OR #10** Filters: **from 2017/4/26 - 2023/8/21**  ((("dyn med"[Journal] OR "dis mon"[Journal] OR "dis manag"[Journal] OR "dm"[All Fields]) AND 2017/04/26:2023/08/21[Date - Publication]) OR ("diabetes mellitus"[MeSH Terms] AND 2017/04/26:2023/08/21[Date - Publication]) OR ("MELLITUS"[All Fields] AND 2017/04/26:2023/08/21[Date - Publication]) OR (("diabete"[All Fields] OR "diabetes mellitus"[MeSH Terms] OR ("diabetes"[All Fields] AND "MELLITUS"[All Fields]) OR "diabetes mellitus"[All Fields] OR "diabetes"[All Fields] OR "diabetes insipidus"[MeSH Terms] OR ("diabetes"[All Fields] AND "insipidus"[All Fields]) OR "diabetes insipidus"[All Fields] OR "diabetic"[All Fields] OR "diabetics"[All Fields] OR "diabets"[All Fields]) AND 2017/04/26:2023/08/21[Date - Publication])) AND (2017/4/26:2023/8/21[pdat])  **Translations**  **DM:** "Dyn Med"[Journal:__jid101152197] OR "Dis Mon"[Journal:__jid0370657] OR "Dis Manag"[Journal:__jid9802539] OR "dm"[All Fields]  **DIABETES MELLITUS [MESH]:** "diabetes mellitus"[MeSH Terms]  **DIABETES:** "diabete"[All Fields] OR "diabetes mellitus"[MeSH Terms] OR ("diabetes"[All Fields] AND "mellitus"[All Fields]) OR "diabetes mellitus"[All Fields] OR "diabetes"[All Fields] OR "diabetes insipidus"[MeSH Terms] OR ("diabetes"[All Fields] AND "insipidus"[All Fields]) OR "diabetes insipidus"[All Fields] OR "diabetic"[All Fields] OR "diabetics"[All Fields] OR "diabets"[All Fields] | [348,050](https://pubmed.ncbi.nlm.nih.gov/?term=%237+OR+%238+OR+%239+OR+%2310&sort=&filter=dates.2017%2F4%2F26-2023%2F8%2F21&size=200) | 02:26:28 |
| #16 |  |  | Search: **#3 OR #4 OR #5 OR #6** Filters: **from 2017/4/26 - 2023/8/21**  (("treatment outcome"[MeSH Terms] AND 2017/04/26:2023/08/21[Date - Publication]) OR (("treatment outcome"[MeSH Terms] OR ("treatment"[All Fields] AND "outcome"[All Fields]) OR "treatment outcome"[All Fields]) AND 2017/04/26:2023/08/21[Date - Publication]) OR (("therapeutics"[MeSH Terms] OR "therapeutics"[All Fields] OR "treatments"[All Fields] OR "therapy"[MeSH Subheading] OR "therapy"[All Fields] OR "treatment"[All Fields] OR "treatment s"[All Fields]) AND 2017/04/26:2023/08/21[Date - Publication]) OR (("outcome"[All Fields] OR "outcomes"[All Fields]) AND 2017/04/26:2023/08/21[Date - Publication])) AND (2017/4/26:2023/8/21[pdat])  **Translations**  **TREATMENT OUTCOME [MESH]:** "treatment outcome"[MeSH Terms]  **TREATMENT OUTCOME:** "treatment outcome"[MeSH Terms] OR ("treatment"[All Fields] AND "outcome"[All Fields]) OR "treatment outcome"[All Fields]  **TREATMENT:** "therapeutics"[MeSH Terms] OR "therapeutics"[All Fields] OR "treatments"[All Fields] OR "therapy"[Subheading] OR "therapy"[All Fields] OR "treatment"[All Fields] OR "treatment's"[All Fields]  **OUTCOME:** "outcome"[All Fields] OR "outcomes"[All Fields] | [3,861,884](https://pubmed.ncbi.nlm.nih.gov/?term=%233+OR+%234+OR+%235+OR+%236&sort=&filter=dates.2017%2F4%2F26-2023%2F8%2F21&size=200) | 02:24:39 |
| #15 |  |  | Search: **#1 OR #2** Filters: **from 2017/4/26 - 2023/8/21**  ((("tuberculosi"[All Fields] OR "tuberculosis"[MeSH Terms] OR "tuberculosis"[All Fields] OR "tuberculoses"[All Fields] OR "tuberculosis s"[All Fields]) AND 2017/04/26:2023/08/21[Date - Publication]) OR ("tuberculosis"[MeSH Terms] AND 2017/04/26:2023/08/21[Date - Publication])) AND (2017/4/26:2023/8/21[pdat])  **Translations**  **TUBERCULOSIS:** "tuberculosi"[All Fields] OR "tuberculosis"[MeSH Terms] OR "tuberculosis"[All Fields] OR "tuberculoses"[All Fields] OR "tuberculosis's"[All Fields]  **tUBERCULOSIS [MESH]:** "tuberculosis"[MeSH Terms] | [54,221](https://pubmed.ncbi.nlm.nih.gov/?term=%231+OR+%232&sort=&filter=dates.2017%2F4%2F26-2023%2F8%2F21&size=200) | 02:24:07 |
| #14 |  |  | Search: **RANDOMIZED CONTROLLED TRIAL** Filters: **from 2017/4/26 - 2023/8/21**  ("randomized controlled trial"[Publication Type] OR "randomized controlled trials as topic"[MeSH Terms] OR "randomized controlled trial"[All Fields] OR "randomised controlled trial"[All Fields]) AND (2017/4/26:2023/8/21[pdat])  **Translations**  **RANDOMIZED CONTROLLED TRIAL:** "randomized controlled trial"[Publication Type] OR "randomized controlled trials as topic"[MeSH Terms] OR "randomized controlled trial"[All Fields] OR "randomised controlled trial"[All Fields] | [229,506](https://pubmed.ncbi.nlm.nih.gov/?term=RANDOMIZED+CONTROLLED+TRIAL&sort=&filter=dates.2017%2F4%2F26-2023%2F8%2F21&size=200) | 02:21:52 |
| #13 |  |  | Search: **RANDOMIZED CONTROLLED TRIALS [MESH]** Filters: **from 2017/4/26 - 2023/8/21**  ("randomized controlled trials as topic"[MeSH Terms]) AND (2017/4/26:2023/8/21[pdat])  **Translations**  **RANDOMIZED CONTROLLED TRIALS [MESH]:** "randomized controlled trials as topic"[MeSH Terms] | [52,187](https://pubmed.ncbi.nlm.nih.gov/?term=RANDOMIZED+CONTROLLED+TRIALS+%5BMESH%5D&sort=&filter=dates.2017%2F4%2F26-2023%2F8%2F21&size=200) | 02:18:23 |
| #12 |  |  | Search: **RANDOMIZED CONTROLLED TRIAL[Publication Type]** Filters: **from 2017/4/26 - 2023/8/21**  ("randomized controlled trial"[Publication Type]) AND (2017/4/26:2023/8/21[pdat]) | [149,173](https://pubmed.ncbi.nlm.nih.gov/?term=RANDOMIZED+CONTROLLED+TRIAL%5BPublication+Type%5D&sort=&filter=dates.2017%2F4%2F26-2023%2F8%2F21&size=200) | 02:16:53 |
| #11 |  |  | Search: **TRIAL** Filters: **from 2017/4/26 - 2023/8/21**  ("clinical trials as topic"[MeSH Terms] OR ("clinical"[All Fields] AND "trials"[All Fields] AND "topic"[All Fields]) OR "clinical trials as topic"[All Fields] OR "trial"[All Fields] OR "trial s"[All Fields] OR "trialed"[All Fields] OR "trialing"[All Fields] OR "trials"[All Fields]) AND (2017/4/26:2023/8/21[pdat])  **Translations**  **TRIAL:** "clinical trials as topic"[MeSH Terms] OR ("clinical"[All Fields] AND "trials"[All Fields] AND "topic"[All Fields]) OR "clinical trials as topic"[All Fields] OR "trial"[All Fields] OR "trial's"[All Fields] OR "trialed"[All Fields] OR "trialing"[All Fields] OR "trials"[All Fields] | [650,776](https://pubmed.ncbi.nlm.nih.gov/?term=TRIAL&filter=dates.2017%2F4%2F26-2023%2F8%2F21&size=200&sort=relevance) | 02:16:04 |
| #10 |  |  | Search: **DIABETES** Filters: **from 2017/4/26 - 2023/8/21**  ("diabete"[All Fields] OR "diabetes mellitus"[MeSH Terms] OR ("diabetes"[All Fields] AND "mellitus"[All Fields]) OR "diabetes mellitus"[All Fields] OR "diabetes"[All Fields] OR "diabetes insipidus"[MeSH Terms] OR ("diabetes"[All Fields] AND "insipidus"[All Fields]) OR "diabetes insipidus"[All Fields] OR "diabetic"[All Fields] OR "diabetics"[All Fields] OR "diabets"[All Fields]) AND (2017/4/26:2023/8/21[pdat])  **Translations**  **DIABETES:** "diabete"[All Fields] OR "diabetes mellitus"[MeSH Terms] OR ("diabetes"[All Fields] AND "mellitus"[All Fields]) OR "diabetes mellitus"[All Fields] OR "diabetes"[All Fields] OR "diabetes insipidus"[MeSH Terms] OR ("diabetes"[All Fields] AND "insipidus"[All Fields]) OR "diabetes insipidus"[All Fields] OR "diabetic"[All Fields] OR "diabetics"[All Fields] OR "diabets"[All Fields] | [327,881](https://pubmed.ncbi.nlm.nih.gov/?term=DIABETES&filter=dates.2017%2F4%2F26-2023%2F8%2F21&size=200&sort=relevance) | 02:15:54 |
| #9 |  |  | Search: **MELLITUS** Filters: **from 2017/4/26 - 2023/8/21**  ("MELLITUS"[All Fields]) AND (2017/4/26:2023/8/21[pdat]) | [166,042](https://pubmed.ncbi.nlm.nih.gov/?term=MELLITUS&filter=dates.2017%2F4%2F26-2023%2F8%2F21&size=200&sort=relevance) | 02:15:45 |
| #8 |  |  | Search: **DIABETES MELLITUS [MESH]** Filters: **from 2017/4/26 - 2023/8/21**  ("diabetes mellitus"[MeSH Terms]) AND (2017/4/26:2023/8/21[pdat])  **Translations**  **DIABETES MELLITUS [MESH]:** "diabetes mellitus"[MeSH Terms] | [133,718](https://pubmed.ncbi.nlm.nih.gov/?term=DIABETES+MELLITUS+%5BMESH%5D&filter=dates.2017%2F4%2F26-2023%2F8%2F21&size=200&sort=relevance) | 02:15:32 |
| #7 |  |  | Search: **DM** Filters: **from 2017/4/26 - 2023/8/21**  ("dyn med"[Journal] OR "dis mon"[Journal] OR "dis manag"[Journal] OR "dm"[All Fields]) AND (2017/4/26:2023/8/21[pdat])  **Translations**  **DM:** "Dyn Med"[Journal:__jid101152197] OR "Dis Mon"[Journal:__jid0370657] OR "Dis Manag"[Journal:__jid9802539] OR "dm"[All Fields] | [35,791](https://pubmed.ncbi.nlm.nih.gov/?term=DM&filter=dates.2017%2F4%2F26-2023%2F8%2F21&size=200&sort=relevance) | 02:15:16 |
| #6 |  |  | Search: **OUTCOME** Filters: **from 2017/4/26 - 2023/8/21**  ("outcome"[All Fields] OR "outcomes"[All Fields]) AND (2017/4/26:2023/8/21[pdat])  **Translations**  **OUTCOME:** "outcome"[All Fields] OR "outcomes"[All Fields] | [1,333,500](https://pubmed.ncbi.nlm.nih.gov/?term=OUTCOME&filter=dates.2017%2F4%2F26-2023%2F8%2F21&size=200&sort=relevance) | 02:15:07 |
| #5 |  |  | Search: **TREATMENT** Filters: **from 2017/4/26 - 2023/8/21**  ("therapeutics"[MeSH Terms] OR "therapeutics"[All Fields] OR "treatments"[All Fields] OR "therapy"[MeSH Subheading] OR "therapy"[All Fields] OR "treatment"[All Fields] OR "treatment s"[All Fields]) AND (2017/4/26:2023/8/21[pdat])  **Translations**  **TREATMENT:** "therapeutics"[MeSH Terms] OR "therapeutics"[All Fields] OR "treatments"[All Fields] OR "therapy"[Subheading] OR "therapy"[All Fields] OR "treatment"[All Fields] OR "treatment's"[All Fields] | [3,458,784](https://pubmed.ncbi.nlm.nih.gov/?term=TREATMENT&filter=dates.2017%2F4%2F26-2023%2F8%2F21&size=200&sort=relevance) | 02:14:54 |
| #4 |  |  | Search: **TREATMENT OUTCOME** Filters: **from 2017/4/26 - 2023/8/21**  ("treatment outcome"[MeSH Terms] OR ("treatment"[All Fields] AND "outcome"[All Fields]) OR "treatment outcome"[All Fields]) AND (2017/4/26:2023/8/21[pdat])  **Translations**  **TREATMENT OUTCOME:** "treatment outcome"[MeSH Terms] OR ("treatment"[All Fields] AND "outcome"[All Fields]) OR "treatment outcome"[All Fields] | [507,877](https://pubmed.ncbi.nlm.nih.gov/?term=TREATMENT+OUTCOME+&filter=dates.2017%2F4%2F26-2023%2F8%2F21&size=200&sort=relevance) | 02:14:42 |
| #3 |  |  | Search: **TREATMENT OUTCOME [MESH]** Filters: **from 2017/4/26 - 2023/8/21**  ("treatment outcome"[MeSH Terms]) AND (2017/4/26:2023/8/21[pdat])  **Translations**  **TREATMENT OUTCOME [MESH]:** "treatment outcome"[MeSH Terms] | [364,574](https://pubmed.ncbi.nlm.nih.gov/?term=TREATMENT+OUTCOME+%5BMESH%5D&filter=dates.2017%2F4%2F26-2023%2F8%2F21&size=200&sort=relevance) | 02:14:22 |
| #2 |  |  | Search: **tUBERCULOSIS [MESH]** Filters: **from 2017/4/26 - 2023/8/21**  ("tuberculosis"[MeSH Terms]) AND (2017/4/26:2023/8/21[pdat])  **Translations**  **tUBERCULOSIS [MESH]:** "tuberculosis"[MeSH Terms] | [25,826](https://pubmed.ncbi.nlm.nih.gov/?term=tUBERCULOSIS+%5BMESH%5D&filter=dates.2017%2F4%2F26-2023%2F8%2F21&size=200&sort=relevance) | 02:14:04 |
| #1 |  |  | Search: **TUBERCULOSIS** Filters: **from 2017/4/26 - 2023/8/21**  ("tuberculosi"[All Fields] OR "tuberculosis"[MeSH Terms] OR "tuberculosis"[All Fields] OR "tuberculoses"[All Fields] OR "tuberculosis s"[All Fields]) AND (2017/4/26:2023/8/21[pdat])  **Translations**  **TUBERCULOSIS:** "tuberculosi"[All Fields] OR "tuberculosis"[MeSH Terms] OR "tuberculosis"[All Fields] OR "tuberculoses"[All Fields] OR "tuberculosis's"[All Fields] | [54,221](https://pubmed.ncbi.nlm.nih.gov/?term=TUBERCULOSIS&sort=&filter=dates.2017%2F4%2F26-2023%2F8%2F21&size=200) | 02:13:47 |
