# Supplementary material for: Effect of glycemic control and type of diabetes treatment on TB treatment outcomes among people with TB-diabetes: A systematic review (updated August 2024)
Source: PLoS One. 2025 Jul 18;20(7):e0328619. doi: 10.1371/journal.pone.0328619 (PMC12273911; doi:10.1371/journal.pone.0328619)
Supplement: S1A Appendix — (ZIP) [file pone.0328619.s005.zip › S1A appendix/S1A_2017-21 Aug 2023/Pubmed/Obj 1 search strategy.docx]

**PUBMED
TB-DM SR - RCTs (for both research questions)**

(tuberculosis OR tuberculosis[MeSH]) AND (treatment outcome[MeSH] OR "treatment outcome*" OR treatment outcome) AND (mellitus OR diabetes OR DM OR diabetes mellitus[MeSH] OR diabetes mellitus) AND ("trial$"[All Fields] OR "randomized controlled trial"[Publication Type] OR "randomized controlled trials as topic"[MeSH Terms] OR "randomized controlled trial"[All Fields]) AND Human[MeSH]

**TB-DM Sr – Cohort studies (for both research questions)**

(tuberculosis OR tuberculosis[MeSH]) AND (treatment outcome[MeSH] OR "treatment outcome*" OR treatment outcome) AND (mellitus OR diabetes OR DM OR diabetes mellitus[MeSH] OR diabetes mellitus) AND ("risk factors"[MeSH Terms] OR risk factor[Text Word] OR cohort OR risk[MeSH] OR cohort studies OR cohort study OR cohort analysis) AND Human[MeSH]
