# Supplementary material for: Effect of glycemic control and type of diabetes treatment on TB treatment outcomes among people with TB-diabetes: A systematic review (updated August 2024)
Source: PLoS One. 2025 Jul 18;20(7):e0328619. doi: 10.1371/journal.pone.0328619 (PMC12273911; doi:10.1371/journal.pone.0328619)
Supplement: S1A Appendix — (ZIP) [file pone.0328619.s005.zip › S1A appendix/S1A_2017-21 Aug 2023/Pubmed/Obj 2 search strategy 3 results.docx]

**Search 2: Effect of glucose lowering treatment on TB treatment outcomes among TB-DM patients(RESEARCH QUESTION 1) 2017/4/26 - 2023/8/21**

The search will consist of a merge of the below 3 searches:

Search 3:

| **Search** | **Actions** | **Details** | **Query** | **Results** | **Time** |
| --- | --- | --- | --- | --- | --- |
| #27 |  |  | Search: **#1 AND #25** | [91](https://pubmed.ncbi.nlm.nih.gov/?term=%231+AND+%2325&sort=) | 01:58:56 |
| #26 |  |  | Search: **(TUBERCULOSIS OR DIABETES) NEAR10 (CONVERGENCE OR EPIDEMIC OR PREVALENCE OR INCIDENCE OR EPIDEMIOLOGY OR RISK) - Schema: all** | 0 | 01:57:33 |
| #25 |  |  | Search: **#11 OR #10 OR #9 OR #12 OR #13 OR #16 OR #24 OR #21 OR #6 OR #22 OR #15 OR #23 OR #7 OR #14 OR #2 OR #19 OR #4 OR #17 OR #8 OR #5 OR #20 OR #18 OR #3** | [25,184](https://pubmed.ncbi.nlm.nih.gov/?term=%2311+OR+%2310+OR+%239+OR+%2312+OR+%2313+OR+%2316+OR+%2324+OR+%2321+OR+%236+OR+%2322+OR+%2315+OR+%2323+OR+%237+OR+%2314+OR+%232+OR+%2319+OR+%234+OR+%2317+OR+%238+OR+%235+OR+%2320+OR+%2318+OR+%233%0D%0A&sort=) | 01:36:43 |
| #24 |  |  | Search: **(BESKOA[Title] OR SUINY[Title] OR ZEMIGLO[Title] OR TENELIA[Title] OR GEMIGLIPTIN[Title] OR TENELIGLIPTIN[Title] OR GALVUS[Title] OR ANAGLIPTIN[Title] OR VILDAGLIPTIN[Title])** Filters: **from 2017/4/26 - 2023/8/21** | [402](https://pubmed.ncbi.nlm.nih.gov/?term=%28BESKOA%5BTitle%5D+OR+SUINY%5BTitle%5D+OR+ZEMIGLO%5BTitle%5D+OR+TENELIA%5BTitle%5D+OR+GEMIGLIPTIN%5BTitle%5D+OR+TENELIGLIPTIN%5BTitle%5D+OR+GALVUS%5BTitle%5D+OR+ANAGLIPTIN%5BTitle%5D+OR+VILDAGLIPTIN%5BTitle%5D%29&sort=&filter=dates.2017%2F4%2F26-2023%2F8%2F21) | 01:35:06 |
| #23 |  |  | Search: **(VIPIDIA[Title] OR SYR-472[Title] OR TRELAGLIPTIN[Title] OR OMARIGLIPTIN[Title] OR TRAZENTA[Title] OR TRAJENTA[Title] OR TRADJENTA[Title] OR BI-1356[Title] OR NESINA[Title] OR ONGLYZA[Title] OR JANUVIA[Title] OR ALOGLIPTIN[Title] OR LINAGLIPTIN[Title] OR SAXAGLIPTIN[Title] OR SITAGLIPTIN[Title])** Filters: **from 2017/4/26 - 2023/8/21** | [1,200](https://pubmed.ncbi.nlm.nih.gov/?term=%28VIPIDIA%5BTitle%5D+OR+SYR-472%5BTitle%5D+OR+TRELAGLIPTIN%5BTitle%5D+OR+OMARIGLIPTIN%5BTitle%5D+OR+TRAZENTA%5BTitle%5D+OR+TRAJENTA%5BTitle%5D+OR+TRADJENTA%5BTitle%5D+OR+BI-1356%5BTitle%5D+OR+NESINA%5BTitle%5D+OR+ONGLYZA%5BTitle%5D+OR+JANUVIA%5BTitle%5D+OR+ALOGLIPTIN%5BTitle%5D+OR+LINAGLIPTIN%5BTitle%5D+OR+SAXAGLIPTIN%5BTitle%5D+OR+SITAGLIPTIN%5BTitle%5D%29&sort=&filter=dates.2017%2F4%2F26-2023%2F8%2F21) | 01:34:03 |
| #22 |  |  | Search: **((((GLIPTIN$1[Title] OR (DPP ADJ (IV[Title] OR "4"[Title]) ADJ I)) OR (DPP ADJ (IV[Title] OR "4"[Title]) ADJ INHIBITOR)) OR ((DIPEPTIDYL ADJ PEPTIDASE) ADJ (IV[Title] OR "4"[Title]) ADJ INHIBITOR)) OR ((DIPEPTIDYL ADJ PEPTIDASE) ADJ (IV[Title] OR "4") ADJ I)).[Title]** Filters: **from 2017/4/26 - 2023/8/21** | [270](https://pubmed.ncbi.nlm.nih.gov/?term=%28%28%28%28GLIPTIN%241%5BTitle%5D+OR+%28DPP+ADJ+%28IV%5BTitle%5D+OR+%224%22%5BTitle%5D%29++++++++++++++++++++ADJ+I%29%29+OR+%28DPP+ADJ+%28IV%5BTitle%5D+OR+%224%22%5BTitle%5D%29+ADJ+++++++++++INHIBITOR%29%29+OR+%28%28DIPEPTIDYL+ADJ+++++++++++PEPTIDASE%29+ADJ+%28IV%5BTitle%5D+OR+%224%22%5BTitle%5D%29+ADJ+++++++++++INHIBITOR%29%29+OR+%28%28DIPEPTIDYL+ADJ+++++++++++PEPTIDASE%29+ADJ+%28IV%5BTitle%5D+OR+%224%22%29+ADJ+++++++++++I%29%29.%5BTitle%5D&sort=&filter=dates.2017%2F4%2F26-2023%2F8%2F21) | 01:33:34 |
| #21 |  |  | Search: **(((SESTRINE[Title] OR EUREPA[Title] OR GLUCONORM[Title] OR NOVONORM[Title] OR PRANDIN[Title] OR REPAGLINIDE[Title]) OR (GLUCO ADJ NORM[Title])) OR (NOVO ADJ NORM)).[Title]** Filters: **from 2017/4/26 - 2023/8/21** | [87](https://pubmed.ncbi.nlm.nih.gov/?term=%28%28%28SESTRINE%5BTitle%5D+OR+EUREPA%5BTitle%5D+OR+GLUCONORM%5BTitle%5D+OR+NOVONORM%5BTitle%5D+OR+PRANDIN%5BTitle%5D+OR+REPAGLINIDE%5BTitle%5D%29+OR+%28GLUCO+ADJ+NORM%5BTitle%5D%29%29+OR+%28NOVO+ADJ+++++++++++NORM%29%29.%5BTitle%5D&sort=&filter=dates.2017%2F4%2F26-2023%2F8%2F21) | 01:32:49 |
| #20 |  |  | Search: **((DEAMELIN ADJ S[Title]) OR GLYCLOPYRAMIDE[Title] OR GLYNASE[Title] OR MICRONASE[Title] OR GLURENORM[Title] OR EUGLUCON[Title] OR AMARYL[Title] OR DAONIL[Title] OR GLISOXEPIDE[Title] OR DIABETA[Title] OR GLIQUIDONE[Title] OR GLIMEPIRIDE[Title] OR GLYBURIDE[Title] OR GLIBENCLAMIDE).[Title]** Filters: **from 2017/4/26 - 2023/8/21** | [1,826](https://pubmed.ncbi.nlm.nih.gov/?term=%28%28DEAMELIN+ADJ+S%5BTitle%5D%29+OR+GLYCLOPYRAMIDE%5BTitle%5D+OR+GLYNASE%5BTitle%5D+OR+MICRONASE%5BTitle%5D+OR+GLURENORM%5BTitle%5D+OR+EUGLUCON%5BTitle%5D+OR+AMARYL%5BTitle%5D+OR+DAONIL%5BTitle%5D+OR+GLISOXEPIDE%5BTitle%5D+OR+DIABETA%5BTitle%5D+OR+GLIQUIDONE%5BTitle%5D+OR+GLIMEPIRIDE%5BTitle%5D+OR+GLYBURIDE%5BTitle%5D+OR+GLIBENCLAMIDE%29.%5BTitle%5D&sort=&filter=dates.2017%2F4%2F26-2023%2F8%2F21) | 01:32:23 |
| #19 |  |  | Search: **(GLUCIDORAL[Title] OR DYMELOR[Title] OR METAHEXAMIDE[Title] OR ORINASE[Title] OR GLUCOTROL[Title] OR DIAMICRON[Title] OR CARBUTAMIDE[Title] OR TOLAZAMIDE[Title] OR ACETOHEXAMIDE[Title] OR CHLORPROPAMIDE[Title] OR GLIPIZIDE[Title] OR GLICLAZIDE[Title] OR TOLBUTAMIDE[Title])** Filters: **from 2017/4/26 - 2023/8/21** | [215](https://pubmed.ncbi.nlm.nih.gov/?term=%28GLUCIDORAL%5BTitle%5D+OR+DYMELOR%5BTitle%5D+OR+METAHEXAMIDE%5BTitle%5D+OR+ORINASE%5BTitle%5D+OR+GLUCOTROL%5BTitle%5D+OR+DIAMICRON%5BTitle%5D+OR+CARBUTAMIDE%5BTitle%5D+OR+TOLAZAMIDE%5BTitle%5D+OR+ACETOHEXAMIDE%5BTitle%5D+OR+CHLORPROPAMIDE%5BTitle%5D+OR+GLIPIZIDE%5BTitle%5D+OR+GLICLAZIDE%5BTitle%5D+OR+TOLBUTAMIDE%5BTitle%5D%29&sort=&filter=dates.2017%2F4%2F26-2023%2F8%2F21) | 01:31:54 |
| #18 |  |  | Search: **(RESULIN[Title] OR ROMOZIN[Title] OR NOSCAL[Title] OR RIVOGLITAZONE[Title] OR REZULIN[Title] OR ACTOS[Title] OR AVANDIA[Title] OR TROGLITAZONE[Title] OR PIOGLITAZONE[Title] OR ROSIGLITAZONE).[Title]** Filters: **from 2017/4/26 - 2023/8/21** | [1,879](https://pubmed.ncbi.nlm.nih.gov/?term=%28RESULIN%5BTitle%5D+OR+ROMOZIN%5BTitle%5D+OR+NOSCAL%5BTitle%5D+OR+RIVOGLITAZONE%5BTitle%5D+OR+REZULIN%5BTitle%5D+OR+ACTOS%5BTitle%5D+OR+AVANDIA%5BTitle%5D+OR+TROGLITAZONE%5BTitle%5D+OR+PIOGLITAZONE%5BTitle%5D+OR+ROSIGLITAZONE%29.%5BTitle%5D&sort=&filter=dates.2017%2F4%2F26-2023%2F8%2F21) | 01:31:29 |
| #17 |  |  | Search: **(BIGUANIDE[Title] OR THIAZOLIDINEDIONE[Title] OR SULFONYLUREA).[Title]** Filters: **from 2017/4/26 - 2023/8/21** | [3,831](https://pubmed.ncbi.nlm.nih.gov/?term=%28BIGUANIDE%5BTitle%5D+OR+THIAZOLIDINEDIONE%5BTitle%5D+OR+SULFONYLUREA%29.%5BTitle%5D&sort=&filter=dates.2017%2F4%2F26-2023%2F8%2F21) | 01:30:47 |
| #16 |  |  | Search: **(METFORMIN NEAR5 DIABETES).[Title]** Filters: **from 2017/4/26 - 2023/8/21** | [4,096](https://pubmed.ncbi.nlm.nih.gov/?term=%28METFORMIN+NEAR5+DIABETES%29.%5BTitle%5D&sort=&filter=dates.2017%2F4%2F26-2023%2F8%2F21) | 01:29:58 |
| #15 |  |  | Search: **GLUFAST[Title] OR STARLIX[Title] OR MEGLITINIDES[Title] OR MITIGLINIDE[Title] OR NATEGLINIDE[Title]** Filters: **from 2017/4/26 - 2023/8/21** | [48](https://pubmed.ncbi.nlm.nih.gov/?term=GLUFAST%5BTitle%5D+OR+STARLIX%5BTitle%5D+OR+MEGLITINIDES%5BTitle%5D+OR+MITIGLINIDE%5BTitle%5D+OR+NATEGLINIDE%5BTitle%5D&sort=&filter=dates.2017%2F4%2F26-2023%2F8%2F21) | 01:29:07 |
| #14 |  |  | Search: **((VOGLIB[Title] OR GLYSET[Title] OR GLUCOBAY[Title] OR VOGLIBOSE[Title] OR MIGLITOL[Title] OR ACARBOSE[Title]) OR ((ALPHA ADJ GLUCOSIDASE) ADJ INHIBITOR)).[Title]** Filters: **from 2017/4/26 - 2023/8/21** | [224](https://pubmed.ncbi.nlm.nih.gov/?term=%28%28VOGLIB%5BTitle%5D+OR+GLYSET%5BTitle%5D+OR+GLUCOBAY%5BTitle%5D+OR+VOGLIBOSE%5BTitle%5D+OR+MIGLITOL%5BTitle%5D+OR+ACARBOSE%5BTitle%5D%29+OR+%28%28ALPHA+ADJ+GLUCOSIDASE%29+ADJ+++++++++++INHIBITOR%29%29.%5BTitle%5D&sort=&filter=dates.2017%2F4%2F26-2023%2F8%2F21) | 01:28:27 |
| #13 |  |  | Search: **(((ALEGLITAZAR[Title] OR TESAGLITAZAR[Title] OR MURAGLITAZAR[Title]) OR ((PEROXISOME ADJ PROLIFERATOR[Title]) ADJ ACTIVATED ADJ RECEPTOR ADJ AGONIST)) OR (PPAR ADJ AGONIST)).[Title]** Filters: **from 2017/4/26 - 2023/8/21** | [11](https://pubmed.ncbi.nlm.nih.gov/?term=%28%28%28ALEGLITAZAR%5BTitle%5D+OR+TESAGLITAZAR%5BTitle%5D+OR+MURAGLITAZAR%5BTitle%5D%29+OR+%28%28PEROXISOME+ADJ+++++++++++PROLIFERATOR%5BTitle%5D%29+ADJ+ACTIVATED+ADJ+++++++++++RECEPTOR+ADJ+AGONIST%29%29+OR+%28PPAR+ADJ+++++++++++AGONIST%29%29.%5BTitle%5D&sort=&filter=dates.2017%2F4%2F26-2023%2F8%2F21) | 01:27:52 |
| #12 |  |  | Search: **(SUGLAT[Title] OR IVOKANA[Title] OR LIPAGLYN[Title] OR FORXIGA[Title] OR SAROGLITAZAR[Title] OR LUSEOGLIFLOZIN[Title] OR ERTUGLIFLOZIN[Title] OR TOFOGLIFLOZIN[Title] OR REMOGLIFLOZIN[Title] OR SERGLIFLOZIN[Title] OR IPRAGLIFLOZIN[Title] OR EMPAGLIFLOZIN[Title] OR CANAGLIFLOZIN[Title] OR DAPAGLIFLOZIN).[Title]** Filters: **from 2017/4/26 - 2023/8/21** | [4,188](https://pubmed.ncbi.nlm.nih.gov/?term=%28SUGLAT%5BTitle%5D+OR+IVOKANA%5BTitle%5D+OR+LIPAGLYN%5BTitle%5D+OR+FORXIGA%5BTitle%5D+OR+SAROGLITAZAR%5BTitle%5D+OR+LUSEOGLIFLOZIN%5BTitle%5D+OR+ERTUGLIFLOZIN%5BTitle%5D+OR+TOFOGLIFLOZIN%5BTitle%5D+OR+REMOGLIFLOZIN%5BTitle%5D+OR+SERGLIFLOZIN%5BTitle%5D+OR+IPRAGLIFLOZIN%5BTitle%5D+OR+EMPAGLIFLOZIN%5BTitle%5D+OR+CANAGLIFLOZIN%5BTitle%5D+OR+DAPAGLIFLOZIN%29.%5BTitle%5D&sort=&filter=dates.2017%2F4%2F26-2023%2F8%2F21) | 01:27:13 |
| #11 |  |  | Search: **(SGLT2 ADJ INHIBITOR 1) OR ((((SODIUM ADJ GLUCOSE) ADJ (COTRANSPORTER OR (CO ADJ TRANSPORTER))) ADJ "2" ADJ INHIBITOR 1).** Filters: **from 2017/4/26 - 2023/8/21** | [3](https://pubmed.ncbi.nlm.nih.gov/?term=%28SGLT2+ADJ+INHIBITOR+1%29+OR+%28%28%28%28SODIUM++++++++++++++++++++ADJ+GLUCOSE%29+ADJ+%28COTRANSPORTER+OR+%28CO+ADJ+TRANSPORTER%29%29%29+ADJ+%222%22+ADJ+++++++++++INHIBITOR+1%29.&sort=&filter=dates.2017%2F4%2F26-2023%2F8%2F21) | 01:26:34 |
| #10 |  |  | Search: **AFREZZA[Title] OR AERX[Title] OR EXUBERA[Title]** Filters: **from 2017/4/26 - 2023/8/21** | [5](https://pubmed.ncbi.nlm.nih.gov/?term=AFREZZA%5BTitle%5D+OR+AERX%5BTitle%5D+OR+EXUBERA%5BTitle%5D&sort=&filter=dates.2017%2F4%2F26-2023%2F8%2F21) | 01:25:08 |
| #9 |  |  | Search: **(penix[Title] OR novoleta[Title] OR ACTRAPHANE[Title] OR ULTRATARD[Title] OR MIXTARD[Title] OR novo in[Title] OR MONOTARD[Title]) OR ((INTERMEDIATE ADJ ACTING[Title]) ADJ1 INSULIN)** Filters: **from 2017/4/26 - 2023/8/21** | [22](https://pubmed.ncbi.nlm.nih.gov/?term=%28PENMIX%5BTitle%5D+OR+NOVOLET%5BTitle%5D+OR+ACTRAPHANE%5BTitle%5D+OR+ULTRATARD%5BTitle%5D+OR+MIXTARD%5BTitle%5D+OR+NOVOLIN%5BTitle%5D+OR+MONOTARD%5BTitle%5D%29+OR+%28%28INTERMEDIATE+ADJ+ACTING%5BTitle%5D%29+++++++++++ADJ1+INSULIN%29&sort=&filter=dates.2017%2F4%2F26-2023%2F8%2F21) | 01:24:26 |
| #8 |  |  | Search: **(((ULTRAPHANE[Title] OR VELASULIN[Title] OR BIOHULIN[Title] OR PROTAPHAN [Title] OR VELOSULIN[Title] OR INSULATARD[Title] OR NOVOLIN[Title] OR ACTRAPID[Title]) OR ((NEUTRAL ADJ PROTAMINE ADJ HAGEDORN[Title]) NEAR2 INSULIN)) OR (NPH NEAR2 INSULIN[Title])) OR (HUMAN ADJ INSULIN[Title])** Filters: **from 2017/4/26 - 2023/8/21** | [9](https://pubmed.ncbi.nlm.nih.gov/?term=%28%28%28ULTRAPHANE%5BTitle%5D+OR+VELASULIN%5BTitle%5D+OR+BIOHULIN%5BTitle%5D+OR+PROTAPHAN+%5BTitle%5D+OR+VELOSULIN%5BTitle%5D+OR+INSULATARD%5BTitle%5D+OR+NOVOLIN%5BTitle%5D+OR+ACTRAPID%5BTitle%5D%29+OR+%28%28NEUTRAL+ADJ+PROTAMINE+ADJ+HAGEDORN%5BTitle%5D%29+++++++++++NEAR2+INSULIN%29%29+OR+%28NPH+NEAR2+INSULIN%5BTitle%5D%29%29+OR+%28HUMAN+ADJ+INSULIN%5BTitle%5D%29&sort=&filter=dates.2017%2F4%2F26-2023%2F8%2F21) | 01:23:49 |
| #7 |  |  | Search: **(((((NOVOMIX[Title] OR (NOVOLOG ADJ MIX[Title])) OR (HUMALOG ADJ MIX[Title])) OR ((BIPHASIC ADJ INSULIN[Title]) ADJ ASPART)) OR ((BIPHASIC ADJ INSULIN[Title]) ADJ LISPRO)) OR (((DUAL-ACTING[Title] OR PREMIX [Title] OR BIPHASIC[Title]) OR (DUAL ADJ ACTING[Title])) ADJ INSULIN)) OR (INSULIN ADJ ANALOG [Title])** Filters: **from 2017/4/26 - 2023/8/21** | [5](https://pubmed.ncbi.nlm.nih.gov/?term=%28%28%28%28%28NOVOMIX%5BTitle%5D+OR+%28NOVOLOG+ADJ+MIX%5BTitle%5D%29%29+OR+%28HUMALOG+ADJ+MIX%5BTitle%5D%29%29+OR+%28%28BIPHASIC+ADJ+++++++++++INSULIN%5BTitle%5D%29+ADJ+ASPART%29%29+OR+%28%28BIPHASIC+ADJ+++++++++++INSULIN%5BTitle%5D%29+ADJ+LISPRO%29%29+OR+%28%28%28DUAL-ACTING%5BTitle%5D+OR+PREMIX+%5BTitle%5D+OR+BIPHASIC%5BTitle%5D%29+OR+%28DUAL+ADJ+++++++++++ACTING%5BTitle%5D%29%29+ADJ+INSULIN%29%29+OR+%28INSULIN+ADJ+++++++++++ANALOG+%5BTitle%5D%29&sort=&filter=dates.2017%2F4%2F26-2023%2F8%2F21) | 01:21:17 |
| #6 |  |  | Search: **((((RAPID-ACTING[Title] OR FAST-ACTING[Title] OR SHORT-ACTING[Title] OR PRANDIAL[Title] OR BOLUS[Title]) OR (FAST ADJ ACTING[Title])) OR (RAPID ADJ ACTING[Title])) OR (SHORT ADJ ACTING)) and insulin[Title]** Filters: **from 2017/4/26 - 2023/8/21** | [17](https://pubmed.ncbi.nlm.nih.gov/?term=%28%28%28%28RAPID-ACTING%5BTitle%5D+OR+FAST-ACTING%5BTitle%5D+OR+SHORT-ACTING%5BTitle%5D+OR+PRANDIAL%5BTitle%5D+OR+BOLUS%5BTitle%5D%29+OR+%28FAST+ADJ+ACTING%5BTitle%5D%29%29+OR+%28RAPID+ADJ+++++++++++ACTING%5BTitle%5D%29%29+OR+%28SHORT+ADJ+ACTING%29%29+ADJ+++++++++++INSULIN%5BTitle%5D&sort=&filter=dates.2017%2F4%2F26-2023%2F8%2F21) | 01:20:09 |
| #5 |  |  | Search: **(APIDRA[Title] OR NOVOLOG[Title] OR NOVORAPID[Title] OR HUMALOG[Title] OR GLULISINE[Title] OR ASPART[Title] OR LISPRO[Title]) OR ((LONG-ACTING[Title] OR (LONG ADJ ACTING[Title]) OR BASAL[Title]) ADJ INSULIN)** Filters: **from 2017/4/26 - 2023/8/21** | [309](https://pubmed.ncbi.nlm.nih.gov/?term=%28APIDRA%5BTitle%5D+OR+NOVOLOG%5BTitle%5D+OR+NOVORAPID%5BTitle%5D+OR+HUMALOG%5BTitle%5D+OR+GLULISINE%5BTitle%5D+OR+ASPART%5BTitle%5D+OR+LISPRO%5BTitle%5D%29+OR+%28%28LONG-ACTING%5BTitle%5D+OR+%28LONG+ADJ+++++++++++ACTING%5BTitle%5D%29+OR+BASAL%5BTitle%5D%29+ADJ+INSULIN%29&sort=&filter=dates.2017%2F4%2F26-2023%2F8%2F21) | 01:19:40 |
| #4 |  |  | Search: **(NN5401[Title] OR NN1250[Title] OR RYZODEG[Title] OR TRESIBA[Title] OR IDEGASP[Title] OR NN304[Title] OR LEVEMIR[Title] OR IDEG[Title] OR DEGLUDEC[Title] OR LANTUS[Title] OR DETEMIR[Title]) OR (LISPRO NEAR PROTAMINE NEAR SUSPENSION[Title]) OR GLARGINE[Title]** Filters: **from 2017/4/26 - 2023/8/21** | [815](https://pubmed.ncbi.nlm.nih.gov/?term=%28NN5401%5BTitle%5D+OR+NN1250%5BTitle%5D+OR+RYZODEG%5BTitle%5D+OR+TRESIBA%5BTitle%5D+OR+IDEGASP%5BTitle%5D+OR+NN304%5BTitle%5D+OR+LEVEMIR%5BTitle%5D+OR+IDEG%5BTitle%5D+OR+DEGLUDEC%5BTitle%5D+OR+LANTUS%5BTitle%5D+OR+DETEMIR%5BTitle%5D%29+OR+%28LISPRO+NEAR+PROTAMINE+NEAR+SUSPENSION%5BTitle%5D%29+OR+GLARGINE%5BTitle%5D&sort=&filter=dates.2017%2F4%2F26-2023%2F8%2F21) | 01:17:50 |
| #3 |  |  | Search: **(((((GLPI[Title] OR GLP1[Title] OR INCRETIN [Title]) OR (GLP ADJ "1"[Title])) OR (GLP ADJ I[Title])) OR (GLUCAGON ADJ LIKE ADJ PEPTIDE ADJ "1"[Title])) OR (GLUCAGON ADJ LIKE ADJ PEPTIDE ADJ I)).[Title]** Filters: **from 2017/4/26 - 2023/8/21** | [2,110](https://pubmed.ncbi.nlm.nih.gov/?term=%28%28%28%28%28GLPI%5BTitle%5D+OR+GLP1%5BTitle%5D+OR+INCRETIN+%5BTitle%5D%29+OR+%28GLP+ADJ+%221%22%5BTitle%5D%29%29+OR+%28GLP+ADJ+I%5BTitle%5D%29%29+OR+%28GLUCAGON+ADJ+LIKE+ADJ+PEPTIDE+ADJ+++++++++++%221%22%5BTitle%5D%29%29+OR+%28GLUCAGON+ADJ+LIKE+ADJ+PEPTIDE+++++++++++ADJ+I%29%29.%5BTitle%5D&sort=&filter=dates.2017%2F4%2F26-2023%2F8%2F21) | 01:17:09 |
| #2 |  |  | Search: **(LYXUMIA[Title] OR SEMAGLUTIDE[Title] OR BYDUREON[Title] OR DULAGLUTIDE[Title] OR VICTOZA[Title] OR ALBIGLUTIDE[Title] OR BYETTA[Title] OR TASPOGLUTIDE[Title] OR LIXISENATIDE[Title] OR LIRAGLUTIDE[Title] OR EXENATIDE[Title] OR EXENDIN-4).[Title]** Filters: **from 2017/4/26 - 2023/8/21** | [3,647](https://pubmed.ncbi.nlm.nih.gov/?term=%28LYXUMIA%5BTitle%5D+OR+SEMAGLUTIDE%5BTitle%5D+OR+BYDUREON%5BTitle%5D+OR+DULAGLUTIDE%5BTitle%5D+OR+VICTOZA%5BTitle%5D+OR+ALBIGLUTIDE%5BTitle%5D+OR+BYETTA%5BTitle%5D+OR+TASPOGLUTIDE%5BTitle%5D+OR+LIXISENATIDE%5BTitle%5D+OR+LIRAGLUTIDE%5BTitle%5D+OR+EXENATIDE%5BTitle%5D+OR+EXENDIN-4%29.%5BTitle%5D&sort=&filter=dates.2017%2F4%2F26-2023%2F8%2F21) | 01:16:37 |
| #1 |  |  | Search: **((TUBERCULOSES[Title] OR (KOCHS ADJ DISEASE[Title]) OR MTB[Title]) OR (KOCH ADJ S ADJ DISEASE[Title]) OR TUBERCULOSIS).[Title]** Filters: **from 2017/4/26 - 2023/8/21** | [54,259](https://pubmed.ncbi.nlm.nih.gov/?term=%28%28TUBERCULOSES%5BTitle%5D+OR+%28KOCHS+ADJ+DISEASE%5BTitle%5D%29+OR+MTB%5BTitle%5D%29+OR+%28KOCH+ADJ+S+ADJ+DISEASE%5BTitle%5D%29+OR+TUBERCULOSIS%29.%5BTitle%5D&sort=&filter=dates.2017%2F4%2F26-2023%2F8%2F21) | 01:16:04 |
