# Supplementary material for: Effect of glycemic control and type of diabetes treatment on TB treatment outcomes among people with TB-diabetes: A systematic review (updated August 2024)
Source: PLoS One. 2025 Jul 18;20(7):e0328619. doi: 10.1371/journal.pone.0328619 (PMC12273911; doi:10.1371/journal.pone.0328619)
Supplement: S1 Appendix — (ZIP) [file pone.0328619.s004.zip › S1 appendix_old/EMBASE/128_with RCT search results no 27.doc]

Database: Embase <1996 to 2016 September 16>

Search Strategy:

--------------------------------------------------------------------------------

1	tuberculosis.mp. or exp tuberculosis/ (151164)

2	exp treatment outcome/ or treatment outcome*.mp. (1189741)

3	diabetes mellitus.mp. or exp diabetes mellitus/ (627157)

4	diabetes mellitus/ (362441)

5	DM.mp. (48079)

6	diabetes.mp. (663193)

7	mellitus.mp. (588341)

8	3 or 4 or 5 or 6 or 7 (715025)

9	1 and 2 and 8 (724)

10	cohort studies.mp. or cohort analysis/ (263408)

11	exp risk factor/ or cohort.mp. or exp risk/ (2148599)

12	10 or 11 (2148599)

13	9 and 10 (81)

14	9 and 12 (304)

15	limit 14 to human (298)

16	limit 13 to human (81)

17	trial*.mp. or exp controlled clinical trial/ or exp "clinical trial (topic)"/ or exp "randomized controlled trial (topic)"/ or exp "controlled clinical trial (topic)"/ (1555047)

18	random.ab. (191735)

19	randomized controlled trial.pt. (0)

20	controlled clinical trial.pt. (0)

21	randomized.ab. (454621)

22	placebo.ab. (188623)

23	clinical trials as topic.sh. (15)

24	trial.ti. (176210)

25	17 or 18 or 19 or 20 or 21 or 22 or 23 or 24 (1831290)

26	9 and 25 (128)

27	limit 26 to human (128)

***************************

1.

Long-term study of infliximab for psoriasis in daily practice: drug survival depends on combined treatment, obesity and infusion reactions.

Spertino J., Lopez-Ferrer A., Vilarrasa E., Puig L.

Journal of the European Academy of Dermatology and Venereology. 28 (11) (pp 1514-1521), 2014. Date of Publication: 01 Nov 2014.

AN: 2014775069

Background: Infiximab has been shown to be highly effective in phase III clinical trials, but limited information is available regarding drug survival and maintenance of efficacy beyond 1 year in real-life clinical setting. Objectives: To analyse the efficacy and safety of infliximab in a large number of patients with a long follow-up and to identify clinical factors associated with long-term drug survival. Methods: A retrospective review of patients with moderate-to-severe psoriasis treated with infliximab from March 2004 to August 2012 at a tertiary dermatology centre was carried out. Results: In total, 63 treatment courses with infliximab were administered to 56 patients. The mean duration of treatment was 31.6 months. The only significant positive predictor of drug survival was combination treatment [hazard ratio (HR) vs. monotherapy 2.90, 95% confidence interval (CI) 1.42-5.92]. Significant negative predictors of drug survival were obesity (HR 0.40, 95% CI 0.19-0.87) and infusion reactions (HR 0.40, 95% CI 0.19-0.87). Infusion reactions occurred in 13 (23%) of our patients and were a reason for discontinuation of treatment in 5. Conclusions: This retrospective review of a cohort of patients with moderate-to-severe psoriasis treated with infliximab in daily practice shows that the PASI75 response rates at 24 and 52 weeks of treatment are similar to those of the pivotal studies, but 37 courses of treatment (59%) had to be discontinued after a median of 12 months. The major cause for discontinuation was loss of response, in 18 cases. Combination treatment, obesity and infusion reactions were found to be predictors of drug survival.

Institution

(Spertino, Lopez-Ferrer, Vilarrasa, Puig) Department of Dermatology, Hospital de la Santa Creu i Sant Pau, Universitat Autonoma de Barcelona, Barcelona, Catalonia, Spain

Publisher

Blackwell Publishing Ltd

Emtree Heading

adult; aged; aminotransferase blood level; antibody titer; arthralgia/si [Side Effect]; arthralgia/dt [Drug Therapy]; backache/si [Side Effect]; bacterial skin disease/si [Side Effect]; basal cell carcinoma/si [Side Effect]; biological therapy; body mass; body temperature; chemoprophylaxis; chest tightness/si [Side Effect]; cholesteatoma/si [Side Effect]; clinical evaluation; *combination drug therapy; controlled study; diabetes mellitus/si [Side Effect]; dizziness/si [Side Effect]; *drug efficacy; *drug safety; drug treatment failure; drug withdrawal; dyspnea/si [Side Effect]; face erythema/si [Side Effect]; female; fever/si [Side Effect]; fever/dt [Drug Therapy]; fibromyalgia/si [Side Effect]; flushing; follow up; gastroenteritis/si [Side Effect]; headache/si [Side Effect]; headache/si [Side Effect]; heart palpitation/si [Side Effect]; human; hypertension/si [Side Effect]; hypertransaminasemia/si [Side Effect]; hypotension/si [Side Effect]; *infusion related reaction/si [Side Effect]; knee prosthesis; large cell lung carcinoma/si [Side Effect]; latent tuberculosis/di [Diagnosis]; latent tuberculosis/pc [Prevention]; latent tuberculosis/si [Side Effect]; latent tuberculosis/dt [Drug Therapy]; leukoplakia/si [Side Effect]; *long term survival; lung tuberculosis/di [Diagnosis]; lung tuberculosis/pc [Prevention]; lung tuberculosis/dt [Drug Therapy]; lupus like syndrome/si [Side Effect]; major clinical study; male; medical record review; monotherapy; myalgia/si [Side Effect]; myalgia/dt [Drug Therapy]; Mycobacterium tuberculosis test kit; nausea/si [Side Effect]; *obesity; otitis/si [Side Effect]; paraproteinemia/si [Side Effect]; phototherapy; priority journal; prosthesis infection/co [Complication]; pruritus/si [Side Effect]; *psoriasis; *psoriasis vulgaris/th [Therapy]; *psoriasis vulgaris/dt [Drug Therapy]; retrospective study; review; rigor/si [Side Effect]; side effect/si [Side Effect]; squamous cell carcinoma/si [Side Effect]; stridor/si [Side Effect]; survival rate; survival time; sweating; systemic therapy; systolic blood pressure; thorax pain/si [Side Effect]; treatment duration; tuberculin test; ultraviolet A radiation; upper respiratory tract infection/si [Side Effect]; urticaria/si [Side Effect]; vagina candidiasis/si [Side Effect]; viral skin disease/si [Side Effect]; wheezing/si [Side Effect]; adalimumab/dt [Drug Therapy]; aminotransferase/ec [Endogenous Compound]; antinuclear antibody/ec [Endogenous Compound]; cyclosporin A/ae [Adverse Drug Reaction]; cyclosporin A/cb [Drug Combination]; cyclosporin A/cm [Drug Comparison]; cyclosporin A/dt [Drug Therapy]; efalizumab/dt [Drug Therapy]; etanercept/dt [Drug Therapy]; etretin/ae [Adverse Drug Reaction]; etretin/cb [Drug Combination]; etretin/cm [Drug Comparison]; etretin/dt [Drug Therapy]; *infliximab/ae [Adverse Drug Reaction]; *infliximab/cb [Drug Combination]; *infliximab/cm [Drug Comparison]; *infliximab/dt [Drug Therapy]; isoniazid/dt [Drug Therapy]; methotrexate/ae [Adverse Drug Reaction]; methotrexate/cb [Drug Combination]; methotrexate/cm [Drug Comparison]; methotrexate/dt [Drug Therapy]; nonsteroid antiinflammatory agent/dt [Drug Therapy]; psoralen; ustekinumab/dt [Drug Therapy].

Link to the Ovid Full Text or citation:

Click here for full text options

2.

Optimizing treatment outcome of first-line anti-tuberculosis drugs: the role of therapeutic drug monitoring.

Verbeeck R.K., Gunther G., Kibuule D., Hunter C., Rennie T.W.

European Journal of Clinical Pharmacology. 72 (8) (pp 905-916), 2016. Date of Publication: 01 Aug 2016.

AN: 20160456385

Introduction: Tuberculosis (TB) remains one of the world's deadliest communicable diseases. Although cure rates of the standard four-drug (rifampicin, isoniazid, pyrazinamide, ethambutol) treatment schedule can be as high as 95-98 % under clinical trial conditions, success rates may be much lower in less well resourced countries. Unsuccessful treatment with these first-line anti-TB drugs may lead to the development of multidrug resistant and extensively drug resistant TB. The intrinsic interindividual variability in the pharmacokinetics (PK) of the first-line anti-TB drugs is further exacerbated by co-morbidities such as HIV infection and diabetes. Methods: Therapeutic drug monitoring has been proposed in an attempt to optimize treatment outcome and reduce the development of drug resistance. Several studies have shown that maximum plasma concentrations (Cmax), especially of rifampicin and isoniazid, are well below the proposed target Cmax concentrations in a substantial fraction of patients being treated with the standard four-drug treatment schedule, even though treatment's success rate in these studies was typically at least 85 %. Discussion: The proposed target Cmax concentrations are based on the concentrations of these agents achieved in healthy volunteers and patients receiving the standard doses. Estimation of Cmax based on one or two sampling times may not have the necessary accuracy since absorption rate, especially for rifampicin, may be highly variable. In addition, minimum inhibitory concentration (MIC) variability should be taken into account to set clinically meaningful susceptibility breakpoints. Clearly, there is a need to better define the key target PK and pharmacodynamic (PD) parameters for therapeutic drug monitoring (TDM) of the first-line anti-TB drugs to be efficacious, Cmax (or area under the curve (AUC)) and Cmax/MIC (or AUC/MIC). Conclusion: Although TDM of first-line anti-TB drugs has been successfully used in a limited number of specialized centers to improve treatment outcome in slow responders, a better characterization of the target PK and/or PK/PD parameters is in our opinion necessary to make it cost-effective.

Institution

(Verbeeck, Kibuule, Hunter, Rennie) Faculty of Health Sciences, University of Namibia, Windhoek, Namibia (Gunther) Katutura State Hospital, Windhoek, Namibia

(Gunther) Leibniz Center for Medicine and Biosciences, Borstel, Germany

Publisher

Springer Verlag

Emtree Heading

area under the curve; childhood disease; clinical trial (topic); comorbidity; diabetes mellitus; drug blood level; *drug monitoring; drug penetration; drug quality; drug sensitivity; human; Human immunodeficiency virus infection; kidney failure; liver failure; *lung tuberculosis/dt [Drug Therapy]; meta analysis (topic); minimum inhibitory concentration; mixed infection; nonhuman; pharmacodynamics; pharmacogenetics; priority journal; randomized controlled trial (topic); review; systematic review (topic); *treatment outcome; *ethambutol/dt [Drug Therapy]; *ethambutol/cr [Drug Concentration]; *ethambutol/pk [Pharmacokinetics]; *isoniazid/dt [Drug Therapy]; *isoniazid/cr [Drug Concentration]; *isoniazid/pk [Pharmacokinetics]; *pyrazinamide/dt [Drug Therapy]; *pyrazinamide/cr [Drug Concentration]; *pyrazinamide/pk [Pharmacokinetics]; *rifampicin/dt [Drug Therapy]; *rifampicin/cr [Drug Concentration]; *rifampicin/pk [Pharmacokinetics].

Link to the Ovid Full Text or citation:

Click here for full text options

3.

Health and ethical consequences of outsourcing pivotal clinical trials to Latin America: A cross-sectional, descriptive study.

Homedes N., Ugalde A.

PLoS ONE. 11 (6) (no pagination), 2016. Article Number: e0157756. Date of Publication: June 2016.

AN: 20160489947

Introduction: The implications of conducting clinical trials in low and middle income countries on the financial accessibility and safety of the pharmaceutical products available in those markets have not been studied. Regulatory practices and ethical declarations lead to the commercialization of the new products, referred to as New Molecular Entities (NMEs), in the countries where tested as soon as they are approved in high surveillance countries. Patients and patients' associations use the Latin American courts to access new and expensive treatments, regardless of their safety profile and therapeutic value. Design and Objectives: Cross-sectional, descriptive study. To determine the therapeutic value and safety profile of the NMEs approved by the Food and Drug Administration (FDA) in 2011 and 2012 that had been tested in Latin America, and the implications of their market approval for the pharmaceutical budgets in the countries where tested. Setting: Latin America. Measures: To assess the therapeutic value and safety of the NMEs commercialized in the different countries we used f independent drug bulletins. The prices of the NMEs for the consumers were obtained from the pharmaceutical price observatories of the countries were the medicines had been tested. If the price was not available in the observatories, it was obtained from pharmaceutical distributors. We used the countries' minimum wage and per capita income to calculate the financial accessibility of a course of treatment with the NMEs. Results: We found that 33 NMEs approved by the FDA in 2011 and 2012 have been tested in Latin America. Of these, 26 had been evaluated by independent drug bulletins and only five were found to add some value to a subset of patients and had significant side-effects. The pharmaceutical prices were very high, varied widely across countries and were unrelated to the countries' income per capita or minimum wage. Conclusion: The implementation of clinical trials in Latin America results in the commercialization of medicines with questionable safety profiles and limited therapeutic value, putting patients at risk and causing budgetary strains in pharmaceutical budgets.

Institution

(Homedes) Management Policy and Community Health, School of Public Health, University of Texas, El Paso, TX, United States (Homedes) Department of International Health, Georgetown University, Washington, DC, United States

(Ugalde) Department of Sociology, University of Texas, Austin, TX, United States

Publisher

Public Library of Science

Emtree Heading

acute coronary syndrome/dt [Drug Therapy]; age related macular degeneration/dt [Drug Therapy]; Argentina; article; atrial fibrillation/dt [Drug Therapy]; breast cancer/dt [Drug Therapy]; castration resistant prostate cancer/dt [Drug Therapy]; Chile; chronic hepatitis C/dt [Drug Therapy]; chronic myeloid leukemia/dt [Drug Therapy]; chronic obstructive lung disease/dt [Drug Therapy]; Colombia; controlled clinical trial; controlled study; cross sectional study; Cushing syndrome/dt [Drug Therapy]; deep vein thrombosis/dt [Drug Therapy]; drug approval; drug cost; drug efficacy; drug safety; *financial management; government regulation; human; Human immunodeficiency virus infection/dt [Drug Therapy]; kidney graft rejection/dt [Drug Therapy]; lung embolism/dt [Drug Therapy]; *medical ethics; melanoma/dt [Drug Therapy]; metastatic colorectal cancer/dt [Drug Therapy]; multidrug resistant tuberculosis/dt [Drug Therapy]; non insulin dependent diabetes mellitus/dt [Drug Therapy]; non small cell lung cancer/dt [Drug Therapy]; outcome assessment; overall survival; Philadelphia 1 chromosome/dt [Drug Therapy]; progression free survival; risk assessment; risk benefit analysis; seizure/dt [Drug Therapy]; *South and Central America; systemic lupus erythematosus/dt [Drug Therapy]; thyroid medullary carcinoma/dt [Drug Therapy]; aclidinium bromide/ct [Clinical Trial]; aclidinium bromide/pe [Pharmacoeconomics]; aclidinium bromide/dt [Drug Therapy]; aflibercept/ct [Clinical Trial]; aflibercept/pe [Pharmacoeconomics]; aflibercept/dt [Drug Therapy]; apixaban/ct [Clinical Trial]; apixaban/pe [Pharmacoeconomics]; apixaban/dt [Drug Therapy]; bedaquiline/ct [Clinical Trial]; bedaquiline/pe [Pharmacoeconomics]; bedaquiline/dt [Drug Therapy]; belatacept/ct [Clinical Trial]; belatacept/pe [Pharmacoeconomics]; belatacept/dt [Drug Therapy]; belimumab/ct [Clinical Trial]; belimumab/pe [Pharmacoeconomics]; belimumab/dt [Drug Therapy]; bosutinib/ct [Clinical Trial]; bosutinib/pe [Pharmacoeconomics]; bosutinib/dt [Drug Therapy]; cabozantinib/ct [Clinical Trial]; cabozantinib/pe [Pharmacoeconomics]; cabozantinib/dt [Drug Therapy]; cobicistat/ct [Clinical Trial]; cobicistat/pe [Pharmacoeconomics]; cobicistat/dt [Drug Therapy]; crizotinib/ct [Clinical Trial]; crizotinib/pe [Pharmacoeconomics]; crizotinib/dt [Drug Therapy]; elvitegravir/ct [Clinical Trial]; elvitegravir/pe [Pharmacoeconomics]; elvitegravir/dt [Drug Therapy]; emtricitabine/ct [Clinical Trial]; emtricitabine/pe [Pharmacoeconomics]; emtricitabine/dt [Drug Therapy]; enzalutamide/ct [Clinical Trial]; enzalutamide/pe [Pharmacoeconomics]; enzalutamide/dt [Drug Therapy]; indacaterol/ct [Clinical Trial]; indacaterol/pe [Pharmacoeconomics]; indacaterol/dt [Drug Therapy]; ipilimumab/ct [Clinical Trial]; ipilimumab/pe [Pharmacoeconomics]; ipilimumab/dt [Drug Therapy]; linagliptin/ct [Clinical Trial]; linagliptin/pe [Pharmacoeconomics]; linagliptin/dt [Drug Therapy]; pasireotide/ct [Clinical Trial]; pasireotide/pe [Pharmacoeconomics]; pasireotide/dt [Drug Therapy]; perampanel/ct [Clinical Trial]; perampanel/pe [Pharmacoeconomics]; perampanel/dt [Drug Therapy]; pertuzumab/ct [Clinical Trial]; pertuzumab/pe [Pharmacoeconomics]; pertuzumab/dt [Drug Therapy]; regorafenib/ct [Clinical Trial]; regorafenib/pe [Pharmacoeconomics]; regorafenib/dt [Drug Therapy]; rilpivirine/ct [Clinical Trial]; rilpivirine/pe [Pharmacoeconomics]; rilpivirine/dt [Drug Therapy]; rivaroxaban/ct [Clinical Trial]; rivaroxaban/pe [Pharmacoeconomics]; rivaroxaban/dt [Drug Therapy]; roflumilast/ct [Clinical Trial]; roflumilast/pe [Pharmacoeconomics]; roflumilast/dt [Drug Therapy]; telaprevir/ct [Clinical Trial]; telaprevir/pe [Pharmacoeconomics]; telaprevir/dt [Drug Therapy]; tenofovir/ct [Clinical Trial]; tenofovir/pe [Pharmacoeconomics]; tenofovir/dt [Drug Therapy]; ticagrelor/ct [Clinical Trial]; ticagrelor/pe [Pharmacoeconomics]; ticagrelor/dt [Drug Therapy]; vandetanib/ct [Clinical Trial]; vandetanib/pe [Pharmacoeconomics]; vandetanib/dt [Drug Therapy].

Drug Trade Names and Manufacturers

Almirall, Astellas, Astra Zeneca, Bayer, Bms, Boehringer, Discovery, Eisai, Exelixis, Forest, Genentech, Genzyme, Gilead, Glaxo SmithKline, Hoffmann La Roche, Janssen, Labaz, Novartis, Pfizer, Rafo, Sobi, Takeda, Teva, Vertex, arcapta neohaler: Novartis, aubagio: Genzyme, benlysta: Glaxo SmithKline, bosulif: Pfizer, brilinta: Astra Zeneca, caprelsa: Astra Zeneca, cometriq: Exelixis, cometriq: Sobi, daliresp: Forest, daxas: Takeda, edarbi: Takeda, edurant: Janssen, eklaire genuari: Almirall, elelyso: Pfizer, elicuis: Bms, eliquis: Bms, eylea: Bayer, eylia: Bayer, fycompa: Eisai, granix: Teva, incivek: Janssen, incivek: Vertex, inlyta: Pfizer, neutroval: Teva, nulojix: Bms, onbrize: Novartis, perjeta: Genentech, perjeta: Hoffmann La Roche, potiga: Glaxo SmithKline, signifor: Novartis, sirturo: Janssen, stivarga: Bayer, stribild: Gilead, surfaxin: Discovery, tradjenta: Boehringer, tudorza pressair: Forest, uplyso: Pfizer, xalkori: Pfizer, xarelto: Bayer, xarelto: Janssen, xeljan: Pfizer, xtandi: Rafo, xtandi: Astellas, yerboy: Bms, yervoy: Bms, zaltrap: Labaz

Link to the Ovid Full Text or citation:

Click here for full text options

4.

Management of Pemphigus Vulgaris.

Cholera M., Chainani-Wu N.

Advances in Therapy. 33 (6) (pp 910-958), 2016. Date of Publication: 01 Jun 2016.

AN: 20160442538

Introduction: Pemphigus vulgaris (PV) is a chronic, autoimmune, vesiculobullous disease. As a result of the relative rarity of PV, published randomized controlled trials (RCTs) are limited, which makes it difficult to evaluate the efficacy of different treatment regimens in this disease. This also precludes conduct of a meta-analysis. Methods: English-language publications describing treatment outcomes of patients with PV were identified by searches of electronic databases through May 2015, and additionally by review of the bibliography of these publications. A total of 89 papers, which included 21 case reports, 47 case series, 8 RCTs, and 13 observational studies, were identified. The findings from these publications, including information on disease course and prognosis, medications used, treatment responses, and side effects, are summarized in the tables and text of this review. Results: Prior to availability of corticosteroid therapy, PV had a high fatality rate. Early publications from the 1970s reported high-dose, prolonged corticosteroid use and significant associated side effects. Later reports described use of corticosteroids along with steroid-sparing adjuvants, which allows a reduction in the total dose of corticosteroids and a reduction in observed mortality and morbidity. For the majority of patients in these reports, a long-term course on medications lasting about 5-10 years was observed; however, subgroups of patients requiring shorter courses or needing longer-term therapy have also been described. Early diagnosis of PV and early initiation of treatment were prognostic factors. In recent publications, commonly used initial regimens include corticosteroids in combination with mycophenolate or azathioprine; whereas, for patients with inadequate response to these regimens, adjuvants such as intravenous immunoglobulin (IVIg) or rituximab are used. Conclusion: The review findings emphasize the importance of early diagnosis, early initiation of treatment, and use of steroid-sparing adjuvants to allow a reduced total dose and duration on corticosteroids. Also highlighted is the need for more RCTs.

Institution

(Cholera, Chainani-Wu) Private Practice in Oral Medicine Clinic, 100 West El Camino Real Suite 76, Mountain View, CA 94040, United States

Publisher

Springer Healthcare

Emtree Heading

abdominal discomfort/si [Side Effect]; acidosis/si [Side Effect]; acute psychosis/si [Side Effect]; adjuvant therapy; adrenal suppression; alopecia/si [Side Effect]; alopecia/si [Side Effect]; anemia/si [Side Effect]; angioneurotic edema/si [Side Effect]; arthralgia/si [Side Effect]; bacterial infection/si [Side Effect]; body fat; bone marrow depression/si [Side Effect]; bronchopneumonia/si [Side Effect]; candidiasis/si [Side Effect]; cataract/si [Side Effect]; chill/si [Side Effect]; chronic drug administration; clinical evaluation; constipation/si [Side Effect]; corticosteroid therapy; coughing/si [Side Effect]; Cushingoid syndrome/si [Side Effect]; depression/si [Side Effect]; diabetes mellitus/si [Side Effect]; diarrhea/si [Side Effect]; drug dose reduction; drug mechanism; drug megadose; drug use; early diagnosis; electrolyte disturbance/si [Side Effect]; eye disease/si [Side Effect]; fatigue/si [Side Effect]; fever/si [Side Effect]; follow up; gastrointestinal symptom/si [Side Effect]; gingivitis/si [Side Effect]; glucose blood level; headache/si [Side Effect]; heart palpitation/si [Side Effect]; hepatitis C/si [Side Effect]; herpes simplex/si [Side Effect]; human; hyperglycemia/si [Side Effect]; hyperkalemia/si [Side Effect]; hyperlipidemia/si [Side Effect]; hypertension/si [Side Effect]; hypocalcemia/si [Side Effect]; immunoadsorption; immunotherapy; infusion related reaction/si [Side Effect]; insomnia/si [Side Effect]; lethargy/si [Side Effect]; leukopenia/si [Side Effect]; liver function test; liver toxicity/si [Side Effect]; lymphocytopenia/si [Side Effect]; meta analysis; mortality; mortality rate; muscle weakness/si [Side Effect]; myalgia/si [Side Effect]; mycosis/si [Side Effect]; myopathy/si [Side Effect]; nausea/si [Side Effect]; neutropenia/si [Side Effect]; osteoporosis/si [Side Effect]; outcome assessment; pain/si [Side Effect]; pancytopenia/si [Side Effect]; *pemphigus vulgaris/di [Diagnosis]; *pemphigus vulgaris/th [Therapy]; *pemphigus vulgaris/dt [Drug Therapy]; phlebitis/si [Side Effect]; plasmapheresis; prognosis; pyoderma/si [Side Effect]; randomized controlled trial (topic); rash/si [Side Effect]; remission; review; sepsis/si [Side Effect]; septicemia/si [Side Effect]; side effect/si [Side Effect]; systematic review; thrombocytopenia/si [Side Effect]; treatment duration; treatment response; tuberculosis/si [Side Effect]; virus infection/si [Side Effect]; weight change; weight gain; azathioprine/ae [Adverse Drug Reaction]; azathioprine/cb [Drug Combination]; azathioprine/dt [Drug Therapy]; azathioprine/ct [Clinical Trial]; azathioprine/pd [Pharmacology]; corticosteroid/ae [Adverse Drug Reaction]; corticosteroid/cb [Drug Combination]; corticosteroid/dt [Drug Therapy]; corticosteroid/ct [Clinical Trial]; corticosteroid/do [Drug Dose]; corticosteroid/to [Drug Toxicity]; cyclophosphamide/dt [Drug Therapy]; cyclosporin/dt [Drug Therapy]; dapsone/dt [Drug Therapy]; glucose/dt [Drug Therapy]; glucose/ec [Endogenous Compound]; gold salt/dt [Drug Therapy]; immunoglobulin/ae [Adverse Drug Reaction]; immunoglobulin/dt [Drug Therapy]; immunoglobulin/iv [Intravenous Drug Administration]; immunoglobulin/ct [Clinical Trial]; immunoglobulin/pd [Pharmacology]; levamisole/dt [Drug Therapy]; methotrexate/ae [Adverse Drug Reaction]; methotrexate/dt [Drug Therapy]; methotrexate/ct [Clinical Trial]; methotrexate/pd [Pharmacology]; mycophenolate mofetil/ae [Adverse Drug Reaction]; mycophenolate mofetil/cb [Drug Combination]; mycophenolate mofetil/dt [Drug Therapy]; mycophenolate mofetil/ct [Clinical Trial]; mycophenolate mofetil/pd [Pharmacology]; nicotinamide/dt [Drug Therapy]; prednisolone/dt [Drug Therapy]; prednisone/dt [Drug Therapy]; rituximab/ae [Adverse Drug Reaction]; rituximab/dt [Drug Therapy]; rituximab/ct [Clinical Trial]; rituximab/pd [Pharmacology]; steroid/dt [Drug Therapy]; tetracycline/dt [Drug Therapy].

Link to the Ovid Full Text or citation:

Click here for full text options

5.

Comparison of low-dose intravenous cyclophosphamide with oral mycophenolate mofetil in the treatment of lupus nephritis.

Rathi M., Goyal A., Jaryal A., Sharma A., Gupta P.K., Ramachandran R., Kumar V., Kohli H.S., Sakhuja V., Jha V., Gupta K.L.

Kidney International. 89 (1) (pp 235-242), 2016. Date of Publication: 01 Jan 2016.

AN: 2015468734

No previous study has compared mycophenolate mofetil (MMF) with low-dose cyclophosphamide (CYC) in the treatment of lupus nephritis (LN). To do so, we recruited patients with LN (class III, IV, or V) and randomized them to receive either low-dose CYC or oral MMF. Those with crescentic LN, a serum creatinine over 265 mumol/l, and neurological or pulmonary lupus were excluded. MMF was prescribed at daily doses of 1.5-3 g for 24 weeks, while CYC was administered as six fortnightly infusions of 500 mg each. All patients received three methylprednisolone injections, followed by oral corticosteroids. Maintenance therapy with azathioprine and low-dose corticosteroid was started at end of induction therapy. The primary end point was treatment response at 24 weeks, while secondary end points were complete remission, Systemic Lupus Erythematosus Disease Activity Index and adverse events. Of the 173 patients recruited, 100 were equally randomized to receive either CYC or MMF. Baseline characteristics were similar, except for higher 24 h proteinuria in the CYC group. At 24 weeks, 37 patients in each group achieved the primary end point. The complete remission rate was 50% in CYC and 54% in MMF group. Gastrointestinal symptoms were significantly more frequent in patients receiving MMF (52 vs. 4%). However, other adverse events were similar. Thus, low-dose intravenous CYC is comparable in safety and efficacy to oral MMF in the induction treatment of less severe LN.

Institution

(Rathi, Goyal, Jaryal, Ramachandran, Kumar, Kohli, Jha, Gupta) Department of Nephrology, Post Graduate Institute of Medical Education and Research, Chandigarh 160 012, India (Sharma) Department of Internal Medicine, Rheumatology Wing, Post Graduate Institute of Medical Education and Research, Chandigarh, India

(Gupta) Department of Biostatistics, Post Graduate Institute of Medical Education and Research, Chandigarh, India

(Sakhuja) Department of Nephrology and Renal Transplant Surgery, Max Super Speciality Hospital, Mohali, India

Publisher

Elsevier

Emtree Heading

abscess/si [Side Effect]; adult; alopecia/si [Side Effect]; amenorrhea/si [Side Effect]; article; breast abscess/si [Side Effect]; cerebrovascular accident/si [Side Effect]; controlled study; creatinine blood level; cytopenia/si [Side Effect]; deep vein thrombosis/si [Side Effect]; deep vein thrombosis/si [Side Effect]; diabetes mellitus/si [Side Effect]; drug cost; drug efficacy; drug fatality/si [Side Effect]; drug safety; drug withdrawal; esophagus candidiasis/si [Side Effect]; female; gastrointestinal symptom/si [Side Effect]; glomerulus filtration rate; gluteal abscess/si [Side Effect]; headache/si [Side Effect]; health economics; herpes zoster/si [Side Effect]; human; hypertransaminasemia/si [Side Effect]; leg ulcer/si [Side Effect]; low drug dose; lung hemorrhage/si [Side Effect]; lung tuberculosis/si [Side Effect]; *lupus erythematosus nephritis/co [Complication]; *lupus erythematosus nephritis/dm [Disease Management]; *lupus erythematosus nephritis/dt [Drug Therapy]; major clinical study; male; menorrhagia/si [Side Effect]; open study; outcome assessment; parallel design; parotitis/si [Side Effect]; pneumonia/si [Side Effect]; priority journal; prospective study; proteinuria; psychosis/si [Side Effect]; randomized controlled trial; remission; seizure/si [Side Effect]; shock/si [Side Effect]; side effect/si [Side Effect]; SLEDAI; stomach ulcer/si [Side Effect]; treatment duration; treatment response; urinary tract infection/si [Side Effect]; azathioprine/dt [Drug Therapy]; corticosteroid/po [Oral Drug Administration]; corticosteroid/do [Drug Dose]; corticosteroid/dt [Drug Therapy]; creatinine/ec [Endogenous Compound]; *cyclophosphamide/ct [Clinical Trial]; *cyclophosphamide/do [Drug Dose]; *cyclophosphamide/pe [Pharmacoeconomics]; *cyclophosphamide/ae [Adverse Drug Reaction]; *cyclophosphamide/cm [Drug Comparison]; *cyclophosphamide/iv [Intravenous Drug Administration]; *cyclophosphamide/dt [Drug Therapy]; methylprednisolone/dt [Drug Therapy]; *mycophenolate mofetil/ct [Clinical Trial]; *mycophenolate mofetil/pe [Pharmacoeconomics]; *mycophenolate mofetil/ae [Adverse Drug Reaction]; *mycophenolate mofetil/cm [Drug Comparison]; *mycophenolate mofetil/po [Oral Drug Administration]; *mycophenolate mofetil/dt [Drug Therapy].

Link to the Ovid Full Text or citation:

Click here for full text options

6.

Treatment effect analysis of the standard regimen and the optimized regimen for retreatment pulmonary tuberculosis complicated with diabetes.

Du J., Gao W., Ma Y., Zhong Q., Liang X., Fu Y., Ji B., Xie L., Huang X., Ge Q., Han X., Tian X., Shu W., Li L.

Zhonghua jie he he hu xi za zhi = Zhonghua jiehe he huxi zazhi = Chinese journal of tuberculosis and respiratory diseases. 38 (12) (pp 886-891), 2015. Date of Publication: 01 Dec 2015.

AN: 26850888

OBJECTIVE: To analyze the therapeutic effects of the standard regimen and the optimized regimen in retreatment pulmonary tuberculosis complicated with diabetes mellitus (DM). METHODS: In a multi-center cohort study, patients with smear positive retreatment pulmonary tuberculosis (TB) with DM and those without DM [excluding multi-drug resistance (MDR), extensively drug-resistant (XDR) and non-tuberculosis Mycobacterium pulmonary disease(NTM)] were enrolled. There were a total of 178 cases, including 60 smear positive retreatment TB patients with DM and 118 without DM, who were randomly divided into 4 groups: Optimized group 1 [individualized treatment in 30 DM cases, 29 males, age (48 +/- 11)], retreatment group 1 [standard retreatment regimen in 30 DM cases, 28 males, age(48 +/- 10)], Optimized group 2[individual regimen in 57 non-DM cases, 37 males, age (41 +/- 14)], and retreatment group 2 [standard retreatment regimen in 61 non-DM cases, 49 males, age (43 +/- 13)]. Patients in the optimized group were treated with optimized individualized regimen based on DST result, with 3-4 sensitive drugs in the regimen, while those in the retreatment group were treated with national standard retreatment regimen. The therapeutic effect of different groups were recorded and the related factors of treatment outcome were analyzed with Chi-square test and multi-factor analysis.

RESULTS: The treatment success rates of the optimized group 1 and the retreatment group 1 were 83.3%(25/30) and 60.0%(18/30), respectively, and the difference was statistically significant (chi(2)=4.02, P=0.045<0.05). The treatment failure rate of the optimized group 1 (6.7%, 2/30) and the retreatment group 1(30.0%, 9/30) was statistically different (chi(2)=5.46, P=0.02<0.05). The outcome difference between the optimized group 2 and the retreatment group 2 showed no statistical significance. Multi-factor analysis showed that treatment regimen, DM, gender and drug resistance were the significant factors related with treatment outcome. The probability of treatment success using the individualized treatment regimen was 2.7 times higher than that using the standard regimen (P=0.025). The risk of treatment failure of the drug resistance cases was 2.8 times higher than that of the drug sensitive cases (P=0.038). The probability of treatment success in DM cases was 0.4 times that in non-DM cases (P<0.05).

CONCLUSION: The outcome of the optimized regimen group was better than that of the standard regimen group, and retreatment TB patients complicated with DM faced a higher risk of treatment failure, which should receive more attention.

Institution

(Du, Gao, Ma, Zhong, Liang, Fu, Ji, Xie, Huang, Ge, Han, Tian, Shu) Beijing Chest Hospital, Capital Medical University, Beijing Tuberculosis and Thoracic Tumor Research Institute, Beijing 101149, China	(Li) Email:

Emtree Heading

adult; cohort analysis; controlled study; diabetes mellitus; female; human; *lung tuberculosis; male; middle aged; randomized controlled trial; retreatment; treatment outcome.

Link to the Ovid Full Text or citation:

Click here for full text options

7.

The effectiveness of mobile phone text messaging in improving medication adherence for patients with chronic diseases: A systematic review.

Sarabi R.E., Sadoughi F., Orak R.J., Bahaadinbeigy K.

Iranian Red Crescent Medical Journal. 18 (5) (no pagination), 2016. Article Number: e25183. Date of Publication: May 2016.

AN: 20160385224

Context: Medication non-adherence is a commonly observed problem in the self-administration of treatment, regardless of the disease type. Text messaging reminders, as electronic reminders, provide an opportunity to improve medication adherence. In this study, we aimed to provide evidence addressing the question of whether text message reminders were effective in improving patients' adherence to medication. Evidence Acquisition: We carried out a systematic literature search, using the five electronic bibliographic databases: PubMed, Embase, PsycINFO, CINAHL, and the Cochrane central register of controlled trials. Studies were included on the basis of whether they examined the benefits and effects of short-message service (SMS) interventions on medication adherence. Results: The results of this systematic review indicated that text messaging interventions have improved patients' medication adherence rate (85%, 29.34). Included in the review, those who had problems with adherence, or those whom text messaging was most helpful had HIV, asthma, diabetes, schizophrenia and heart disease (73.5%). The period of intervention varied from 1 week to 14 months. The most common study design was randomized controlled trials (RCTs) (66%) carried out in the developed countries. Conclusions: This study demonstrated the potential of mobile phone text messaging for medication non-adherence problem solving.

Institution

(Sarabi, Sadoughi, Orak) Health Information Management Department, School of Management and Medical Information Sciences, Iran University of Medical Sciences, Tehran, Iran, Islamic Republic of (Bahaadinbeigy) Medical Informatics Research Center, Institute of Futures Studies in Health, Kerman University of Medical Sciences, Kerman, Iran, Islamic Republic of

Publisher

Kowsar Medical Publishing Company

Emtree Heading

allergy; asthma; *chronic disease; clinical effectiveness; diabetes mellitus; health care cost; health care quality; heart disease; hospitalization; human; intervention study; lupus vulgaris; *medication compliance; meta analysis; *mobile phone; mortality; outcome assessment; review; schizophrenia; sickle cell anemia; systematic review; *text messaging; tuberculosis.

Link to the Ovid Full Text or citation:

Click here for full text options

8.

Pemphigus in North-Western Yemen: A therapeutic study of 75 cases.

Khatri M.L.

Indian Journal of Dermatology, Venereology and Leprology. 82 (3) (pp 359), 2016. Date of Publication: May-June 2016.

AN: 20160342209

Background: The incidence of pemphigus, though not documented, seems to be quite high in Yemen. There is no universal consensus on the treatment of this disease. Aims: The aim was to evaluate the efficacy and side effects of different therapeutic regimens used in patients of pemphigus in North-Western Yemen. Patients and Methods: Seventy-five Yemeni patients (39 males and 36 females) were included. Diagnosis was based on clinical features, histopathology and the Tzanck test. Results of treatment with these different therapeutic regimens were compared: (1) dexamethasone-cyclophosphamide pulse (DCP), (2) dexamethasone pulse with oral azathioprine, (3) oral prednisolone with azathioprine, (4) oral prednisolone with oral cyclophosphamide, and (5) prednisolone monotherapy. Results: Pemphigus vulgaris (PV) was diagnosed in 46 patients, pemphigus foliaceus (PF) in 23, pemphigus vegetans (PVEG) in 5 and pemphigus herpetiformis (PH) in one. Among the 16 patients who received regular DCP therapy, 13 were in remission for 6 months to 11 years without medications (phase 4). Remission without pharmacotherapy could not be achieved with the other regimens and steroid-induced side-effects appeared to be more than with DCP. Limitations: Immunofluorescence was not available to confirm the diagnosis of pemphigus. Randomization was not done. Conclusion: The DCP regimen seemed to be superior to the other regimens used.

Institution

(Khatri) Department of Dermatology, Saudi Hospital, Hajjah, Yemen (Khatri) Saudi Hospital, Hajjah, P.O. Box: 2757, Sana'a, Yemen

Publisher

Medknow Publications (B9, Kanara Business Centre, off Link Road, Ghatkopar (E), Mumbai 400 075, India)

Emtree Heading

adjuvant therapy; adolescent; adult; aged; alopecia/si [Side Effect]; amenorrhea/si [Side Effect]; article; chickenpox/si [Side Effect]; child; clinical feature; clinical trial; Cushingoid syndrome/si [Side Effect]; diabetes mellitus/si [Side Effect]; diarrhea/si [Side Effect]; disease severity; drug dose increase; drug efficacy; *drug pulse therapy; drug safety; drug tolerability; drug withdrawal; epilepsy/si [Side Effect]; epistaxis/si [Side Effect]; female; follow up; genital candidiasis/si [Side Effect]; herpes simplex/si [Side Effect]; herpes zoster/si [Side Effect]; hiccup/si [Side Effect]; histopathology; human; hyperbilirubinemia; hypertension/si [Side Effect]; hypertransaminasemia/si [Side Effect]; leukopenia/si [Side Effect]; major clinical study; male; outcome assessment; *pemphigus/dt [Drug Therapy]; *pemphigus foliaceus/dt [Drug Therapy]; *pemphigus herpetiformis; pemphigus herpetiformis/dt [Drug Therapy]; *pemphigus vegetans; pemphigus vegetans/dt [Drug Therapy]; *pemphigus vulgaris/dt [Drug Therapy]; prospective study; recurrent disease; remission; thrush/si [Side Effect]; tuberculosis/si [Side Effect]; Yemen; *azathioprine/ae [Adverse Drug Reaction]; *azathioprine/cb [Drug Combination]; *azathioprine/dt [Drug Therapy]; *azathioprine/po [Oral Drug Administration]; *azathioprine/ct [Clinical Trial]; *cyclophosphamide/po [Oral Drug Administration]; *cyclophosphamide/ae [Adverse Drug Reaction]; *cyclophosphamide/cb [Drug Combination]; *cyclophosphamide/dt [Drug Therapy]; *cyclophosphamide/ct [Clinical Trial]; *dexamethasone/ae [Adverse Drug Reaction]; *dexamethasone/cb [Drug Combination]; *dexamethasone/dt [Drug Therapy]; *dexamethasone/iv [Intravenous Drug Administration]; *dexamethasone/ct [Clinical Trial]; immunoglobulin/iv [Intravenous Drug Administration]; immunoglobulin/dt [Drug Therapy]; *prednisolone/po [Oral Drug Administration]; *prednisolone/ae [Adverse Drug Reaction]; *prednisolone/cb [Drug Combination]; *prednisolone/dt [Drug Therapy]; *prednisolone/ct [Clinical Trial].

Link to the Ovid Full Text or citation:

Click here for full text options

9.

Comparison of Efficacy and Safety of Ciclosporin to Prednisolone in the Treatment of Erythema Nodosum Leprosum: Two Randomised, Double Blind, Controlled Pilot Studies in Ethiopia.

Lambert S.M., Nigusse S.D., Alembo D.T., Walker S.L., Nicholls P.G., Idriss M.H., Yamuah L.K., Lockwood D.N.J.

PLoS Neglected Tropical Diseases. 10 (2) (no pagination), 2016. Article Number: e0004149. Date of Publication: 26 Feb 2016.

AN: 20160184554

Background: Erythema Nodosum Leprosum (ENL) is a serious complication of leprosy. It is normally treated with high dose steroids, but its recurrent nature leads to prolonged steroid usage and associated side effects. There is little evidence on the efficacy of alternative treatments for ENL, especially for patients who have become steroid resistant or have steroid side effects. These two pilot studies compare the efficacy and side effect profile of ciclosporin plus prednisolone against prednisolone alone in the treatment of patients with either new ENL or chronic and recurrent ENL. Methods and Results: Thirteen patients with new ENL and twenty patients with chronic ENL were recruited into two double-blinded randomised controlled trials. Patients were randomised to receive ciclosporin and prednisolone or prednisolone treatment only. Patients with acute ENL had a delay of 16 weeks in the occurrence of ENL flare-up episode, with less severe flare-ups and decreased requirements for additional prednisolone. Patients with chronic ENL on ciclosporin had the first episode of ENL flare-up 4 weeks earlier than those on prednisolone, as well as more severe ENL flare-ups requiring 2.5 times more additional prednisolone. Adverse events attributable to prednisolone were more common that those attributable to ciclosporin. Conclusions: This is the first clinical trial on ENL management set in the African context, and also the first trial in leprosy to use patients' assessment of outcomes. Patients on ciclosporin showed promising results in the management of acute ENL in this small pilot study. But ciclosporin, did not appear to have a significant steroid-sparing effects in patients with chronic ENL, which may have been due to the prolonged use of steroids in these patients in combination with a too rapid decrease of steroids in patients given ciclosporin. Further research is needed to determine whether the promising results of ciclosporin in acute ENL can be reproduced on a larger scale.

Institution

(Lambert, Walker, Lockwood) Department of Clinical Research, London School of Hygiene and Tropical Medicine, London, United Kingdom (Nigusse, Alembo, Idriss) All Africa Leprosy Rehabilitation and Training (ALERT) Center, Addis Ababa, Ethiopia

(Nicholls) School of Health Sciences, University of Southampton, Southampton, United Kingdom

(Yamuah) Data Management, Armauer Hansen Research Institute (AHRI), Addis Ababa, Ethiopia

Publisher

Public Library of Science

Emtree Heading

acne/si [Side Effect]; adult; article; blood cell count; bone pain; cataract/si [Side Effect]; clinical assessment; comparative study; controlled study; diabetes mellitus/si [Side Effect]; disease severity; double blind procedure; drug efficacy; drug safety; drug tolerability; *erythema nodosum leprosum/co [Complication]; *erythema nodosum leprosum/dm [Disease Management]; *erythema nodosum leprosum/dt [Drug Therapy]; erythrocyte sedimentation rate; Ethiopia; female; glaucoma/si [Side Effect]; hirsutism/si [Side Effect]; human; human tissue; hypertension/si [Side Effect]; *leprosy; male; moon face/si [Side Effect]; mycosis/si [Side Effect]; neuritis; peptic ulcer/si [Side Effect]; psychosis/si [Side Effect]; quality of life; randomized controlled trial; skin biopsy; stomach pain; strongyloidiasis/pc [Prevention]; strongyloidiasis/dt [Drug Therapy]; treatment outcome; tuberculosis/si [Side Effect]; urinalysis; albendazole/dt [Drug Therapy]; *cyclosporin A/ct [Clinical Trial]; *cyclosporin A/cb [Drug Combination]; *cyclosporin A/cm [Drug Comparison]; *cyclosporin A/dt [Drug Therapy]; *cyclosporin A/ae [Adverse Drug Reaction]; placebo; *prednisolone/ct [Clinical Trial]; *prednisolone/cb [Drug Combination]; *prednisolone/cm [Drug Comparison]; *prednisolone/dt [Drug Therapy]; *prednisolone/ae [Adverse Drug Reaction].

Link to the Ovid Full Text or citation:

Click here for full text options

10.

Does enhanced diabetes management reduce the risk and improve the outcome of tuberculosis?.

Lo H.-Y., Yang S.-L., Lin H.-H., Bai K.-J., Lee J.-J., Lee T.-I., Chiang C.-Y.

International Journal of Tuberculosis and Lung Disease. 20 (3) (pp 376-382), 2016. Date of Publication: 01 Mar 2016.

AN: 20160148052

SETTING: The Bureau of National Health Insurance (NHI) has implemented a pay-for-performance (p4p) programme for diabetes mellitus (DM) in Taiwan. OBJECTIVE : To investigate whether patients with DM enrolled in the p4p programme (DM-p4p) are less likely to develop tuberculosis (TB) and whether they have a better outcome than patients with DMnot enrolled in the p4p programme (DM-non-p4p) if they do develop TB. DESIGN: A random sample of 79 471 DM-p4p, 100 000 DM-non-p4p and 100 000 non-diabetic patients (non-DM) was obtained from the 2008-2009 NHI database, and the patients were matched with the National TB Registry to determine whether they had developed TB by the end of 2010. RESULTS : The average annual incidence of TB was respectively 259.9 (95%CI 230.2-293.4), 137.5 (95%CI 116.4-162.5) and 74.1 (95%CI 59.0-93.0) per 100 000 population among DM-non-p4p, DM-p4p and non-DM patients. The relative risk of death over treatment success was 1.79 (95%CI 1.05-3.04) among DM-non-p4p and 1.69 (95%CI 0.84-3.40) among non-DM patients, relative to DM-p4p patients. CONCLUSIONS : Enhanced case management of DM reduced risk and improved outcomes of TB among patients with DM.

Institution

(Lo, Yang) Taiwan Centers for Disease Control, Ministry of Health and Welfare, Taipei, Taiwan (Republic of China) (Lin) Institute of Epidemiology and Preventive Medicine, National Taiwan University, Taipei, Taiwan (Republic of China)

(Bai) School of Respiratory Therapy, College of Medicine, Taipei Medical University, Taipei, Taiwan (Republic of China)

(Bai, Chiang) Division of Pulmonary Medicine, Department of Internal Medicine, Wan Fang Hospital, Taipei Medical University, 111 Hsin-Long Road, Taipei, Taiwan (Republic of China)

(Lee) Department of Internal Medicine, Tzu Chi General Hospital and Tzu Chi University, Hualien, Taiwan (Republic of China)

(Lee) Division of Endocrinology and Metabolism, Department of Internal Medicine, Wan Fang Hospital, Taipei Medical University, Taipei, Taiwan (Republic of China)

(Lee) Department of General Medicine, School of Medicine, College of Medicine, Taipei Medical University, Taipei, Taiwan (Republic of China)

(Chiang) Department of Internal Medicine, School of Medicine, College of Medicine, Taipei Medical University, Taipei, Taiwan (Republic of China)

(Chiang) International Union Against Tuberculosis and Lung Disease, Paris, France

Publisher

International Union against Tubercul. and Lung Dis.

Emtree Heading

adult; aged; article; case management; cohort analysis; controlled study; *diabetes mellitus; female; *health insurance; human; incidence; major clinical study; male; mortality; *pay-for-performance; priority journal; *risk reduction; treatment outcome; *tuberculosis; very elderly.

Link to the Ovid Full Text or citation:

Click here for full text options

11.

Results of the implementation of a pilot model for the bidirectional screening and joint management of patients with pulmonary tuberculosis and diabetes mellitus in Mexico.

Castellanos-Joya M., Delgado-Sanchez G., Ferreyra-Reyes L., Cruz-Hervert P., Ferreira-Guerrero E., Ortiz-Solis G., Jimenez M.I., Salazar L.L., Montero-Campos R., Mongua-Rodriguez N., Baez-Saldana R., Bobadilla-del-Valle M., Gonzalez-Roldan J.F., Ponce-de-Leon A., Sifuentes-Osornio J., Garcia-Garcia L.

PloS one. 9 (9) (pp e106961), 2014. Date of Publication: 2014.

AN: 25229236

BACKGROUND: Recently, the World Health Organisation and the International Union Against Tuberculosis and Lung Disease published a Collaborative Framework for the Care and Control of Tuberculosis (TB) and Diabetes (DM) (CFTB/DM) proposing bidirectional screening and joint management. OBJECTIVE: To evaluate the feasibility and effectiveness of the CFTB/DM in Mexico.

DESIGN: Prospective observational cohort.

SETTING: 15 primary care units in 5 states in Mexico.

PARTICIPANTS: Patients aged >20 years diagnosed with DM or pulmonary TB who sought care at participating clinics.

INTERVENTION: The WHO/Union CFTB/DM was adapted and implemented according to official Mexican guidelines. We recruited participants from July 2012 to April 2013 and followed up until March 2014. Bidirectional screening was performed. Patients diagnosed with TB and DM were invited to receive TB treatment under joint management.

MAIN OUTCOME MEASURES: Diagnoses of TB among DM, of DM among TB, and treatment outcomes among patients with DM and TB.

RESULTS: Of 783 DM patients, 11 (1.4%) were unaware of their TB. Of 361 TB patients, 16 (4.4%) were unaware of their DM. 95 TB/DM patients accepted to be treated under joint management, of whom 85 (89.5%) successfully completed treatment. Multiple linear regression analysis with change in HbA1c and random capillary glucose as dependent variables revealed significant decrease with time (regression coefficients (beta) = -0.660, (95% confidence interval (CI), -0.96 to -0.35); and beta = -1.889 (95% CI, -2.77 to -1.01, respectively)) adjusting by sex, age and having been treated for a previous TB episode. Patients treated under joint management were more likely to experience treatment success than patients treated under routine DM and TB programs as compared to historical (adjusted OR (aOR), 2.8, 95%CI 1.28-6.13) and same period (aOR 2.37, 95% CI 1.13-4.96) comparison groups.

CONCLUSIONS: Joint management of TB and DM is feasible and appears to improve clinical outcomes.

Institution

(Castellanos-Joya) Direccion del Programa de Micobacteriosis, Centro Nacional de Programas Preventivos y Control de Enfermedades, Mexico, Distrito Federal, Mexico (Delgado-Sanchez) Centro de Investigacion sobre Enfermedades Infecciosas, Instituto Nacional de Salud Publica, Cuernavaca, Morelos, Mexico

(Ferreyra-Reyes) Centro de Investigacion sobre Enfermedades Infecciosas, Instituto Nacional de Salud Publica, Cuernavaca, Morelos, Mexico

(Cruz-Hervert) Centro de Investigacion sobre Enfermedades Infecciosas, Instituto Nacional de Salud Publica, Cuernavaca, Morelos, Mexico

(Ferreira-Guerrero) Centro de Investigacion sobre Enfermedades Infecciosas, Instituto Nacional de Salud Publica, Cuernavaca, Morelos, Mexico

(Ortiz-Solis) Direccion del Programa de Salud en el Adulto y en el Anciano, Centro Nacional de Programas Preventivos y Control de Enfermedades, Mexico, Distrito Federal, Mexico

(Jimenez) Direccion del Programa de Micobacteriosis, Centro Nacional de Programas Preventivos y Control de Enfermedades, Mexico, Distrito Federal, Mexico

(Salazar) Direccion del Programa de Micobacteriosis, Centro Nacional de Programas Preventivos y Control de Enfermedades, Mexico, Distrito Federal, Mexico

(Montero-Campos) Centro de Investigacion sobre Enfermedades Infecciosas, Instituto Nacional de Salud Publica, Cuernavaca, Morelos, Mexico

(Mongua-Rodriguez) Centro de Investigacion sobre Enfermedades Infecciosas, Instituto Nacional de Salud Publica, Cuernavaca, Morelos, Mexico

(Baez-Saldana) Centro de Investigacion sobre Enfermedades Infecciosas, Instituto Nacional de Salud Publica, Cuernavaca, Morelos, Mexico

(Bobadilla-del-Valle) Laboratorio de Microbiologia, Instituto Nacional de Ciencias Medicas y de Nutricion "Salvador Zubiran", Mexico, Distrito Federal, Mexico

(Gonzalez-Roldan) Direccion General, Centro Nacional de Programas Preventivos y Control de Enfermedades, Mexico, Distrito Federal, Mexico

(Ponce-de-Leon) Laboratorio de Microbiologia, Instituto Nacional de Ciencias Medicas y de Nutricion "Salvador Zubiran", Mexico, Distrito Federal, Mexico

(Sifuentes-Osornio) Direccion Medica, Instituto Nacional de Ciencias Medicas y de Nutricion "Salvador Zubiran", Mexico, Distrito Federal, Mexico

(Garcia-Garcia) Centro de Investigacion sobre Enfermedades Infecciosas, Instituto Nacional de Salud Publica, Cuernavaca, Morelos, Mexico

Emtree Heading

aged; diabetes mellitus/di [Diagnosis]; disease management; human; Mexico; middle aged; prospective study; Tuberculosis, Pulmonary/di [Diagnosis].

Link to the Ovid Full Text or citation:

Click here for full text options

12.

Progress in Medicine: Experts Take Stock.

Garvey C., McBride T., Nevin L., Parthasarathy S., Peiperl L., Ross A., Simpson P., Beck A., Birney E., Graeber M., Tumwine J., Hay P., Ahn H.S., Patel A., du Cros P., von Seidlein L., Wareham N., Low N.

PLoS Medicine. 12 (12) (no pagination), 2015. Article Number: e1001933. Date of Publication: 2015.

AN: 20160031519

Institution

Public Library of Science, San Francisco, CA, United States (Beck) Department of Pathology, Harvard Medical School, Beth Israel Deaconess Medical Center, Boston, MA, United States

(Birney) European Bioinformatics Institute, Hinxton, United Kingdom

(Graeber) Brain Tumour Research, University of Sydney, Sydney, Australia

(Tumwine) Paediatrics and Child Health, School of Medicine, Makerere University, College of Health Sciences, Kampala, Uganda

(Hay) School of Medicine, University of Western Sydney, Sydney, Australia

(Ahn) Department of Preventive Medicine, College of Medicine University, Seoul, South Korea

(Patel) The George Institute for Global Health, University of Sydney, Sydney, Australia

(du Cros) Manson Unit, Medecins Sans Frontieres (MSF), London, United Kingdom

(von Seidlein) Mahidol-Oxford Tropical Medicine Research Unit (MORU), Mahidol University, Bangkok, Thailand

(Wareham) MRC Epidemiology Unit, University of Cambridge, Cambridge, United Kingdom

(Low) Institute of Social and Preventive Medicine, University of Bern, Bern, Switzerland

Publisher

Public Library of Science

Emtree Heading

Alzheimer disease; cancer screening; diabetes mellitus; editorial; genomics; health care personnel; human; Human immunodeficiency virus infection; Lewy body; malaria; medical education; *medicine; mental health; mental health care; molecular biology; neoplasm; outcome assessment; Parkinson disease; personalized medicine; randomized controlled trial (topic); sexually transmitted disease; tuberculosis; vascular disease.

Link to the Ovid Full Text or citation:

Click here for full text options

13.

Progressive Disseminated Histoplasmosis and HIV/AIDS: a Dermatological Perspective.

Chang P., Meaux T.

Current Fungal Infection Reports. 9 (4) (pp 213-219), 2015. Date of Publication: 01 Dec 2015.

AN: 2015463852

Progressive disseminated histoplasmosis (PDH) occurs as a result of internal spread of Histoplasma capsulatum from the lungs to organs rich in monocytes. This typically occurs in immunosuppressed patients, such as those with HIV/AIDS. Skin involvement is found in 10 % of all patients with PDH in the USA and in up to 25 % of those with AIDS. Crusted papular or plaque-like lesions are most common, with others being nodular, pustular, ulcerated, vegetative, acneiform, or wart-like. Lesions may be caused by the fungus itself or an immune response to the infection, such as erythema nodosum or erythema multiform. Histopathology demonstrates necrosis of the superficial dermal vessels. The differential diagnosis is broad and varies depending on the location and morphology of the lesion(s). Culture is the gold standard for diagnosis, and first-line treatment is amphotericin B for 1-2 weeks at a dose of 3 mg/kg/day.

Institution

(Chang, Meaux) Dermatology Service Social Security General Hospital-IGSS, 9. Street 7-55 Zone 9, Guatemala City 01009, Guatemala (Meaux) Dermatology Elective, Hospital General de Enfermedades IGSS, 9. Street 7-55 Zone 9, Guatemala City 01009, Guatemala

Publisher

Current Medicine Group LLC 1

Emtree Heading

*acquired immune deficiency syndrome/pc [Prevention]; *acquired immune deficiency syndrome/dt [Drug Therapy]; Africa; age; Ajellomyces capsulatus; alcoholism; Argentina; blastomycosis; Brazil; cancer radiotherapy; candidiasis/di [Diagnosis]; cellulitis/di [Diagnosis]; coccidioidomycosis; Colombia; complement fixation test; coughing; Crohn disease/di [Diagnosis]; cryptococcosis/di [Diagnosis]; cytosol; diabetes mellitus; disease association; drug tolerability; erythema multiforme; erythema nodosum/di [Diagnosis]; erythroderma/di [Diagnosis]; fever; fine needle aspiration biopsy; fungus culture; Germany; giant cell granuloma; gold standard; highly active antiretroviral therapy; histiocyte; histopathology; *histoplasmosis/co [Complication]; *histoplasmosis/di [Diagnosis]; *histoplasmosis/ep [Epidemiology]; *histoplasmosis/pc [Prevention]; *histoplasmosis/dt [Drug Therapy]; human; Human immunodeficiency virus infected patient; immunocompromised patient; immunodiffusion; immunosuppressive treatment; incidence; Italy; kidney transplantation; leishmaniasis/di [Diagnosis]; lung lavage; lymphocyte; lymphocytopenia; lymphoma/di [Diagnosis]; microscopy; molluscum contagiosum/di [Diagnosis]; multiplex polymerase chain reaction; Mycobacterium haemophilum; neutrophil; nonhuman; penicilliosis/di [Diagnosis]; physical examination; prevalence; primary cutaneous histoplasmosis; *progressive disseminated histoplasmosis/co [Complication]; *progressive disseminated histoplasmosis/di [Diagnosis]; *progressive disseminated histoplasmosis/ep [Epidemiology]; *progressive disseminated histoplasmosis/pc [Prevention]; *progressive disseminated histoplasmosis/dt [Drug Therapy]; pyoderma gangrenosum/di [Diagnosis]; randomized controlled trial(topic); relapse; review; risk factor; rosacea/di [Diagnosis]; sensitivity and specificity; Sjoegren syndrome; smear; squamous cell carcinoma/di [Diagnosis]; syphilis/di [Diagnosis]; systemic mycosis/di [Diagnosis]; treatment outcome; Trichosporon; Trichosporon inkin; tuberculosis/di [Diagnosis]; United States; weight reduction; amphotericin B/ct [Clinical Trial]; amphotericin B/dt [Drug Therapy]; amphotericin B deoxycholate/ct [Clinical Trial]; amphotericin B deoxycholate/dt [Drug Therapy]; C reactive protein/ec [Endogenous Compound]; fluconazole/ct [Clinical Trial]; fluconazole/dt [Drug Therapy]; integrase inhibitor/dt [Drug Therapy]; itraconazole/ct [Clinical Trial]; itraconazole/dt [Drug Therapy]; methenamine; nonnucleoside reverse transcriptase inhibitor/dt [Drug Therapy]; posaconazole/ct [Clinical Trial]; posaconazole/dt [Drug Therapy]; proteinase inhibitor/dt [Drug Therapy]; RNA directed DNA polymerase inhibitor/dt [Drug Therapy]; voriconazole/ct [Clinical Trial]; voriconazole/dt [Drug Therapy].

Link to the Ovid Full Text or citation:

Click here for full text options

14.

Long-term outcomes of NSAID-induced small intestinal injury assessed by capsule endoscopy in Korea: A nationwide multicenter retrospective study.

Shim K.-N., Song E.M., Jeen Y.T., Kim J.-O., Jeon S.R., Chang D.K., Song H.J., Lim Y.J., Kim J.S., Ye B.D., Park C.H., Jeon S.W., Cheon J.H., Lee K.J., Kim J.H., Jang B.I., Moon J.S., Chun H.J., Choi M.-G.

Gut and Liver. 9 (6) (pp 727-733), 2015. Date of Publication: November 2015.

AN: 2015506633

Background/Aims: We evaluated the long-term outcome and clinical course of patients of nonsteroidal anti-inflammatory drug (NSAID)-induced small intestinal injury by performing capsule endoscopy (CE). Methods: A multicenter retrospective study was conducted using data collected from the CE nationwide database registry, which has been established since 2002. Results: A total of 140 patients (87 males; mean age, 60.6+/-14.8 years) from the CE nationwide database registry (n=2,885) were diagnosed with NSAID-induced small intestinal injury and enrolled in our study. Forty-nine patients (35.0%) presented with a history of aspirin use and an additional 49 (35.0%) were taking NSAIDs without aspirin. The most prominent findings after performing CE were multiple ulcerations (n=82, 58.6%) and erosions or aphthae (n=32, 22.9%). During the follow-up period (mean, 15.9+/-19.0 months; range, 0 to 106 months), NSAID-induced small intestinal injury only recurred in six patients (4.3%). Older age and hypertension were positive predictive factors for recurrence. Conclusions: These results suggest that the recurrence of NSAID-induced small bowel injury was not frequent in the presence of conservative treatment. Therefore, the initial diagnosis using CE and the medication history are important.

Institution

(Shim, Song) Department of Internal Medicine, Ewha Womans University School of Medicine, Seoul, South Korea (Jeen, Chun) Department of Internal Medicine, Korea University College of Medicine, 73 Inchon-ro, Seongbuk-gu, Seoul 02841, South Korea

(Kim, Jeon) Department of Internal Medicine, Soonchunhyang University College of Medicine, Seoul, South Korea

(Chang) Department of Internal Medicine, Samsung Medical Center, Sungkyunkwan University School of Medicine, Seoul, South Korea

(Song) Department of Internal Medicine, Jeju National University College of Medicine, Jeju, South Korea

(Lim) Department of Internal Medicine, Dongguk University College of Medicine, Goyang, South Korea

(Kim, Choi) Department of Internal Medicine, Catholic University, Korea College of Medicine, Seoul, South Korea

(Ye) Department of Gastroenterology, University of Ulsan College of Medicine, Seoul, South Korea

(Park) Department of Internal Medicine, Hallym University College of Medicine, Seoul, South Korea

(Jeon) Department of Internal Medicine, Kyungpook National University School of Medicine, Daegu, South Korea

(Cheon) Department of Internal Medicine, Yonsei University College of Medicine, Seoul, South Korea

(Lee) Department of Gastroenterology, Ajou University School of Medicine, Suwon, South Korea

(Kim) Department of Gastroenterology, Inje University College of Medicine, Busan, South Korea

(Jang) Department of Internal Medicine, Yeungnam University College of Medicine, Daegu, South Korea

(Moon) Department of Internal Medicine, Inje University College of Medicine, Seoul, South Korea

Publisher

Joe Bok Chung

Emtree Heading

abdominal pain/si [Side Effect]; adult; age; aged; albumin blood level; anemia; angiodysplasia/di [Diagnosis]; arthritis/dt [Drug Therapy]; article; Behcet disease/di [Diagnosis]; capsule endoscope; *capsule endoscopy; cardiovascular disease/dt [Drug Therapy]; cerebrovascular disease/dt [Drug Therapy]; chronic kidney disease/dt [Drug Therapy]; clinical feature; computer program; diabetes mellitus/dt [Drug Therapy]; diagnostic error; Dieulafoy disease/di [Diagnosis]; disease association; disease course; *drug induced disease/si [Side Effect]; drug withdrawal; enteritis/di [Diagnosis]; erosion/di [Diagnosis]; erythema/si [Side Effect]; female; follow up; gastrointestinal hemorrhage/si [Side Effect]; gastrointestinal stromal tumor/di [Diagnosis]; human; hypertension/dt [Drug Therapy]; idiopathic ulceration/di [Diagnosis]; ileal erosion/di [Diagnosis]; *intestine injury/si [Side Effect]; *intestine injury/dt [Drug Therapy]; intestine polyp/si [Side Effect]; intestine stenosis/si [Side Effect]; ischemia/di [Diagnosis]; ischemic enteritis/di [Diagnosis]; Korea; *long term care; major clinical study; male; melena/si [Side Effect]; middle aged; mucosal erythema/si [Side Effect]; multicenter study (topic); non invasive procedure; patient history of therapy; prescription; rectum hemorrhage/si [Side Effect]; recurrent disease; retrospective study; small intestine tumor/si [Side Effect]; treatment duration; treatment outcome; treatment response; tuberculosis/di [Diagnosis]; acetylsalicylic acid/ae [Adverse Drug Reaction]; acetylsalicylic acid/dt [Drug Therapy]; acetylsalicylic acid/ct [Clinical Trial]; albumin/ec [Endogenous Compound]; misoprostol/dt [Drug Therapy]; *nonsteroid antiinflammatory agent/ae [Adverse Drug Reaction]; *nonsteroid antiinflammatory agent/dt [Drug Therapy]; proton pump inhibitor/dt [Drug Therapy]; rebamipide/dt [Drug Therapy].

Link to the Ovid Full Text or citation:

Click here for full text options

15.

Hookworm infection among patients with pulmonary tuberculosis: Impact of co-infection on the therapeutic failure of pulmonary tuberculosis.

Hasanain A.F.A., Zayed A.A.A.H., Mahdy R.E., Nafee A.M.A., Attia R.A.M.H., Mohamed A.O.

International Journal of Mycobacteriology. 4 (4) (pp 318-322), 2015. Date of Publication: December 2015.

AN: 2015445105

Objective/background: The aim of this study is to determine the rate of hookworm infection among patients with pulmonary tuberculosis (TB) and to find out if there is a relation between hookworm infection and the therapeutic failure of pulmonary TB. Methods: We carried out a prospective, hospital-based study. The study included 231 naive patients with pulmonary TB, consecutively. Patients were evaluated at the 4th month of therapy for persistence of Mycobacterium tuberculosis infection. All patients had clinical evaluation, laboratory investigations (including sputum culture and stool microscopic examination), and imaging studies (abdominal ultrasonography and chest radiography). Results: The study population mean age was 42.7. +/-. 13.9. years old with 26.8% of them 40. years old or more. Out of 231 patients, 133 (57.6%) were men. Therapeutic failure rate of pulmonary TB was 29.4%. Hookworm infection was diagnosed among 16.5% of patients and 27.7% had diabetes mellitus (DM). Using multivariate analysis, it was found that age of 40. years or more (odds ratio [OR] 8.4; 95% confidence interval [CI] 1.7-41.3; p=. .009), hookworm infection (OR 7.6; 95% CI 1.2-49.9; p=. .034), and DM (OR 5.9; 1.2-28; p=. .027) were independently associated with therapeutic failure of pulmonary TB among the study population with pulmonary TB. Conclusion: In conclusion, the rate of therapeutic failure of pulmonary TB is high. Besides older age and DM, hookworm infection can reduce the therapeutic response of pulmonary TB. Screening for and control of DM and hookworm infection among patients with pulmonary TB may improve their therapeutic response.

Institution

(Hasanain) Department of Tropical Medicine and Gastroenterology, Faculty of Medicine, Assiut University, Assiut, Egypt (Zayed) Department of Chest Diseases, Faculty of Medicine, Assiut University, Assiut, Egypt

(Mahdy) Department of Internal Medicine, Faculty of Medicine, Assiut University, Assiut, Egypt

(Nafee) Department of Microbiology and Immunology, Faculty of Medicine, Assiut University, Assiut, Egypt

(Attia) Department of Parasitology, Faculty of Medicine, Assiut University, Assiut, Egypt

(Mohamed) Department of Clinical Pathology, Faculty of Medicine, Assiut University, Assiut, Egypt

Publisher

Elsevier Ltd

Emtree Heading

adult; aging; article; clinical trial; diabetes mellitus; *drug treatment failure; echography; feces analysis; female; *hookworm infection; human; *lung tuberculosis/dt [Drug Therapy]; major clinical study; male; microscopy; *mixed infection; priority journal; prospective study; sputum culture; thorax radiography; *ethambutol/ct [Clinical Trial]; *ethambutol/dt [Drug Therapy]; *isoniazid/ct [Clinical Trial]; *isoniazid/dt [Drug Therapy]; *pyrazinamide/ct [Clinical Trial]; *pyrazinamide/dt [Drug Therapy]; *rifampicin/ct [Clinical Trial]; *rifampicin/dt [Drug Therapy].

Link to the Ovid Full Text or citation:

Click here for full text options

16.

Safety of biologic therapies for the treatment of juvenile idiopathic arthritis.

Horneff G.

Expert Opinion on Drug Safety. 14 (7) (pp 1111-1126), 2015. Date of Publication: 2015.

AN: 2015148100

Introduction: The introduction of biological therapies opened a new era of treatment of juvenile idiopathic arthritis. After 15 years of experience with the first biologics for treatment of pediatric rheumatic disease, long-term safety effects are of great interest.Areas covered: This review summarizes published knowledge about safety aspects from clinical trials as well as from biologic registries in juvenile idiopathic arthritis patients. Beside infusion and injection reactions, the occurrence and aggravation of infections, the occurrence of a second autoimmune diseases, including uveitis, psoriasis, chronic inflammatory bowel disease, multiple sclerosis, diabetes mellitus, as well as cytopenias and the development of malignancies are major concerns regarding treatment with biologics.Expert opinion: The safety profiles of approved biologics, the TNF-alpha inhibitors etanercept and adalimumab, and the IL-6-inhibitor tocilizumab are highly acceptable. This conclusion is not easily expandable to the IL-1 inhibitor canakinumab as well as the T-cell-activation-inhibitor abatacept due to lack of experience; however, both have showed an excellent safety profile so far. An increase in knowledge about risk profiles in national and international collaborations, with national as well as international registries, is necessary.

Institution

(Horneff) Centre for Paediatric Rheumatology, Department of Paediatrics, Asklepios Clinic Sankt Augustin, Arnold-Janssen-Str. 29, Sankt Augustin 53757, Germany

Publisher

Informa Healthcare

Emtree Heading

abdominal pain/si [Side Effect]; acute lymphoblastic leukemia/si [Side Effect]; anaphylaxis/si [Side Effect]; appendicitis/si [Side Effect]; aseptic meningitis/si [Side Effect]; autoimmune disease/si [Side Effect]; bacterial infection/si [Side Effect]; bacterial superinfection/si [Side Effect]; *biological therapy; bronchitis/si [Side Effect]; candidiasis/si [Side Effect]; cellulitis/si [Side Effect]; cervical spine dislocation/si [Side Effect]; chickenpox/si [Side Effect]; concussion/si [Side Effect]; conjunctivitis/si [Side Effect]; coughing/si [Side Effect]; coxitis/si [Side Effect]; Crohn disease/si [Side Effect]; cytopenia/si [Side Effect]; demyelinating disease/si [Side Effect]; dental caries/si [Side Effect]; drug efficacy; drug fatality/si [Side Effect]; drug fever/si [Side Effect]; drug induced headache/si [Side Effect]; *drug safety; epigastric pain/si [Side Effect]; flu like syndrome/si [Side Effect]; gastroenteritis/si [Side Effect]; gastrointestinal hemorrhage/si [Side Effect]; herpes simplex/si [Side Effect]; herpes zoster/si [Side Effect]; Hodgkin disease/si [Side Effect]; human; hypertransaminasemia/si [Side Effect]; infection/si [Side Effect]; infusion related reaction/si [Side Effect]; injection site pain/si [Side Effect]; injection site reaction/si [Side Effect]; insulin dependent diabetes mellitus/si [Side Effect]; *juvenile rheumatoid arthritis/dt [Drug Therapy]; lung tuberculosis/si [Side Effect]; lupus like syndrome/si [Side Effect]; macrophage activation syndrome/si [Side Effect]; meningococcemia/si [Side Effect]; monotherapy; multiple sclerosis/si [Side Effect]; musculoskeletal chest pain/si [Side Effect]; neoplasm/si [Side Effect]; nonhodgkin lymphoma/si [Side Effect]; nonhuman; opportunistic infection/si [Side Effect]; otitis/si [Side Effect]; peritonitis/si [Side Effect]; pharyngitis/si [Side Effect]; phase 1 clinical trial (topic); phase 2 clinical trial (topic); pneumonia/si [Side Effect]; psychosis/si [Side Effect]; pulmonary hypertension/si [Side Effect]; pulmonary veno-occlusive disease/si [Side Effect]; pyrexia idiopathica/si [Side Effect]; pyrexia idiopathica/si [Side Effect]; randomized controlled trial(topic); review; rhinopharyngitis/si [Side Effect]; rhinorrhea/si [Side Effect]; sepsis/si [Side Effect]; septic shock/si [Side Effect]; side effect/si [Side Effect]; skin infection/si [Side Effect]; soft tissue infection/si [Side Effect]; surgical infection/si [Side Effect]; T lymphocyte activation; thyroid cancer/si [Side Effect]; treatment outcome; treatment response; tuberculosis/si [Side Effect]; ulcerative colitis/si [Side Effect]; upper respiratory tract infection/si [Side Effect]; uveitis/si [Side Effect]; vasculitis/si [Side Effect]; viral gastroenteritis/si [Side Effect]; viral upper respiratory tract infection/si [Side Effect]; abatacept/ae [Adverse Drug Reaction]; abatacept/dt [Drug Therapy]; abatacept/pd [Pharmacology]; adalimumab/ae [Adverse Drug Reaction]; adalimumab/dt [Drug Therapy]; adalimumab/ct [Clinical Trial]; *antirheumatic agent/ae [Adverse Drug Reaction]; *antirheumatic agent/dt [Drug Therapy]; *biological product/ae [Adverse Drug Reaction]; *biological product/dt [Drug Therapy]; canakinumab/ae [Adverse Drug Reaction]; canakinumab/dt [Drug Therapy]; etanercept/ae [Adverse Drug Reaction]; etanercept/dt [Drug Therapy]; etanercept/ct [Clinical Trial]; infliximab/ae [Adverse Drug Reaction]; infliximab/dt [Drug Therapy]; infliximab/ct [Clinical Trial]; lymphotoxin/ec [Endogenous Compound]; methotrexate/dt [Drug Therapy]; tocilizumab/ae [Adverse Drug Reaction]; tocilizumab/dt [Drug Therapy]; tocilizumab/ct [Clinical Trial]; tumor necrosis factor alpha/ec [Endogenous Compound].

Link to the Ovid Full Text or citation:

Click here for full text options

17.

Smoking on treatment outcomes among tuberculosis patients.

Khan A.H., Israr M., Khan A., Aftab R.A., Khan T.M.

American Journal of the Medical Sciences. 349 (6) (pp 505-509), 2015. Date of Publication: 06 Jun 2015.

AN: 2015105159

Background: Smoking is a risk factor not only for the development of cancer and coronary heart disease but also for tuberculosis (TB). The aim of this study was to determine the prevalence of smoking in patients with TB, identify demographic and clinical characteristics associated with smoking and to evaluate TB treatment outcomes in the smokers. Method: A retrospective cohort study of patients with TB was conducted at Khyber Teaching Hospital, Peshawar, Pakistan. All patients with pulmonary and extrapulmonary TB and those coinfected with HIV, hepatitis or diabetes mellitus were included in the study. The patients were categorized into smokers and nonsmokers. Treatment outcomes were evaluated by smear testing at the end of the treatment. Results: Of 472 enrolled subjects, 68 (14.4%) were smokers. The prevalence of smoking among male and female patients with TB was 11.8% and 2.5%, respectively. Univariate analysis indicated that the gender, age group and marital status of patients with TB were associated with smoking. The results indicated that patient gender (P 0.05), age: 15 to 24 years (P 0.05) and age >55 years (P 0.004) were risk factors associated with smoking among TB patients. Of the 68 smokers with TB, the treatment outcomes among 54 patients (79.4%) were unsuccessful. The treatment outcomes was statistically significantly associated with smoking (odds ratio: 2.58, P 0.004). Conclusions: Findings from the current study proved smoking to be one of the main factors associated with the occurrence of TB and significantly reducing the outcomes of TB therapy.

Institution

(Khan, Aftab) Department of Clinical Pharmacy, School of Pharmaceutical Sciences, Universiti Sains Malaysia, Penang 11800, Malaysia (Israr) Department of Respiratory Diseases, District TB Office Bannu, Bannu, Pakistan

(Khan) Medical B Unit, Khyber Teaching Hospital, Peshawar, Pakistan

(Khan) School of Pharmacy, Monash University, Selangor Darul Ehsan, Malaysia

Publisher

Lippincott Williams and Wilkins

Emtree Heading

adolescent; adult; article; clinical feature; controlled clinical trial; controlled study; demography; diabetes mellitus; *extrapulmonary tuberculosis/dt [Drug Therapy]; female; groups by age; hepatitis; human; Human immunodeficiency virus infection; *lung tuberculosis/dt [Drug Therapy]; major clinical study; male; marriage; mixed infection; prevalence; retrospective study; risk factor; *smoking; sputum smear; *treatment outcome; *tuberculostatic agent/dt [Drug Therapy].

Link to the Ovid Full Text or citation:

Click here for full text options

18.

Nonopioid Medications for Pain.

Tauben D.

Physical Medicine and Rehabilitation Clinics of North America. 26 (2) (pp 219-248), 2015. Date of Publication: 01 May 2015.

AN: 2015049328

Evidence of nonopioid analgesic effectiveness exceeds that for long-term opioids in chronic noncancer pain (CNCP), most with lower risk. Non-drug therapies such as cognitive behavioral therapy and physical activation are safer and also effective. Nonsteroidal antiinflammatory drugs are useful for inflammatory and nociceptive pain, share renal and variable gastrointestinal, bleeding and cardiovascular side effects. Antidepressants with noradrenergic activity (such as tricyclics and seroton-norepinephrine reuptake inhibitors) and neuromodulating anticonvulsant drugs (gabapentinoids and sodium-channel blockers) are proven to be effective for neuropathic and centralized pain. Ketamine and cannabinoids are other studied analgesics but have a less well-proven role in CNCP.

Institution

(Tauben) Division of Pain Medicine, Department of Anesthesiology and Pain Medicine, UW Center for Pain Relief, University of Washington, 1959 Northeast Pacific Street, Box 356540, Seattle, WA 98195, United States (Tauben) Division of General Internal Medicine, Department of Medicine, UW Center for Pain Relief, University of Washington, 1959 Northeast Pacific Street, Box 356540, Seattle, WA 98195, United States

Publisher

W.B. Saunders

Emtree Heading

acupuncture; alcoholic beverage; *analgesia; analgesic activity; analgesic nephropathy/si [Side Effect]; anticholinergic syndrome/si [Side Effect]; application site pain/si [Side Effect]; application site reaction/si [Side Effect]; arthritis/dt [Drug Therapy]; asthma/si [Side Effect]; balance disorder/si [Side Effect]; cannabis addiction/si [Side Effect]; cardiovascular disease; cardiovascular risk; chronic bronchitis/si [Side Effect]; *chronic pain/dm [Disease Management]; *chronic pain/et [Etiology]; *chronic pain/th [Therapy]; *chronic pain/dt [Drug Therapy]; clinical trial(topic); cognitive defect/si [Side Effect]; cognitive therapy; comparative effectiveness; constipation/si [Side Effect]; cost control; diabetic nephropathy/dt [Drug Therapy]; diabetic neuropathy/dt [Drug Therapy]; dose kidney function relation; drug absorption; drug alcohol interaction; drug bioavailability; drug choice; drug classification; drug contraindication; drug dose reduction; drug efficacy; drug hypersensitivity/si [Side Effect]; drug mechanism; drug megadose; drug misuse; drug monitoring; drug potency; drug potentiation; drug safety; drug selectivity; drug tolerability; drug withdrawal; edema/si [Side Effect]; endometriosis/dt [Drug Therapy]; enthesopathy/dt [Drug Therapy]; evidence based medicine; face pain/dt [Drug Therapy]; fever/dt [Drug Therapy]; fibromyalgia/dt [Drug Therapy]; fracture nonunion/si [Side Effect]; glucose intolerance/si [Side Effect]; hallucination/si [Side Effect]; headache/dt [Drug Therapy]; health care cost; human; hyperpyrexia/si [Side Effect]; hypnosis; inflammatory pain/dt [Drug Therapy]; insomnia/th [Therapy]; insomnia/dt [Drug Therapy]; interstitial nephritis/si [Side Effect]; intraocular hypertension/si [Side Effect]; kidney papilla necrosis/si [Side Effect]; kinesiotherapy; knee osteoarthritis/dt [Drug Therapy]; liver failure/si [Side Effect]; low drug dose; lupus vulgaris/dt [Drug Therapy]; maximum plasma concentration; medical decision making; memory disorder/si [Side Effect]; migraine/dt [Drug Therapy]; multiple sclerosis/dt [Drug Therapy]; muscle spasm/dt [Drug Therapy]; myositis/dt [Drug Therapy]; nausea/si [Side Effect]; nephrotoxicity/si [Side Effect]; nephrotoxicity/si [Side Effect]; neuritis/dt [Drug Therapy]; neurolysis/si [Side Effect]; neuromodulation; neuropathic pain/dt [Drug Therapy]; nociceptive pain/dt [Drug Therapy]; nonhuman; orthostatic hypotension/si [Side Effect]; osteoarthritis/dt [Drug Therapy]; outcome assessment; pain assessment; pathophysiology; patient satisfaction; pelvis pain syndrome/dt [Drug Therapy]; peripheral neuropathy/dt [Drug Therapy]; pharmacodynamics; physical activity; polypharmacy; postherpetic neuralgia/dt [Drug Therapy]; postoperative nausea and vomiting/dt [Drug Therapy]; prescription; priority journal; prostate hypertrophy/si [Side Effect]; psychosocial care; QT prolongation/si [Side Effect]; quality of life; radiculopathy/dt [Drug Therapy]; randomized controlled trial(topic); review; Reye syndrome/si [Side Effect]; rhabdomyolysis/si [Side Effect]; rheumatic disease/dt [Drug Therapy]; rheumatic polymyalgia/dt [Drug Therapy]; rheumatoid arthritis/dt [Drug Therapy]; risk benefit analysis; sedation; sensitization; sexual dysfunction/si [Side Effect]; side effect/si [Side Effect]; sleep quality; sodium retention; spondyloarthropathy/dt [Drug Therapy]; stiff man syndrome/dt [Drug Therapy]; tendinitis/dt [Drug Therapy]; tension headache/dt [Drug Therapy]; trigeminus neuralgia/dt [Drug Therapy]; urine retention/si [Side Effect]; xerostomia/si [Side Effect]; yoga; acetylsalicylic acid/do [Drug Dose]; acetylsalicylic acid/ae [Adverse Drug Reaction]; acetylsalicylic acid/cb [Drug Combination]; anticonvulsive agent/ae [Adverse Drug Reaction]; anticonvulsive agent/dt [Drug Therapy]; antidepressant agent/pd [Pharmacology]; antidepressant agent/it [Drug Interaction]; antidepressant agent/dt [Drug Therapy]; baclofen/po [Oral Drug Administration]; baclofen/ae [Adverse Drug Reaction]; baclofen/dt [Drug Therapy]; benzodiazepine derivative/dt [Drug Therapy]; calcium channel blocking agent/dt [Drug Therapy]; cannabis/ct [Clinical Trial]; cannabis/ae [Adverse Drug Reaction]; cannabis/dt [Drug Therapy]; capsaicin/td [Transdermal Drug Administration]; capsaicin/pd [Pharmacology]; capsaicin/do [Drug Dose]; capsaicin/ae [Adverse Drug Reaction]; capsaicin/dt [Drug Therapy]; carbamazepine/pd [Pharmacology]; carbamazepine/dt [Drug Therapy]; corticosteroid/ae [Adverse Drug Reaction]; corticosteroid/ei [Epidural Drug Administration]; corticosteroid/dt [Drug Therapy]; diclofenac/td [Transdermal Drug Administration]; diclofenac/pk [Pharmacokinetics]; diclofenac/ae [Adverse Drug Reaction]; diclofenac/dt [Drug Therapy]; diphenhydramine/dt [Drug Therapy]; ketamine/po [Oral Drug Administration]; ketamine/td [Transdermal Drug Administration]; ketamine/pd [Pharmacology]; ketamine/ae [Adverse Drug Reaction]; ketamine/bd [Buccal Drug Administration]; ketamine/dt [Drug Therapy]; lidocaine/td [Transdermal Drug Administration]; lidocaine/dt [Drug Therapy]; methylprednisolone/po [Oral Drug Administration]; methylprednisolone/do [Drug Dose]; methylprednisolone/dt [Drug Therapy]; muscle relaxant agent/dt [Drug Therapy]; nonsteroid antiinflammatory agent/pd [Pharmacology]; nonsteroid antiinflammatory agent/ae [Adverse Drug Reaction]; nonsteroid antiinflammatory agent/cb [Drug Combination]; nonsteroid antiinflammatory agent/it [Drug Interaction]; nonsteroid antiinflammatory agent/iv [Intravenous Drug Administration]; nonsteroid antiinflammatory agent/po [Oral Drug Administration]; nonsteroid antiinflammatory agent/td [Transdermal Drug Administration]; nonsteroid antiinflammatory agent/dt [Drug Therapy]; paracetamol/pd [Pharmacology]; paracetamol/ae [Adverse Drug Reaction]; paracetamol/dt [Drug Therapy]; phenacetin/ae [Adverse Drug Reaction]; phenacetin/cb [Drug Combination]; phenacetin/dt [Drug Therapy]; phenytoin/it [Drug Interaction]; prednisone/po [Oral Drug Administration]; prednisone/do [Drug Dose]; prednisone/dt [Drug Therapy]; serotonin noradrenalin reuptake inhibitor/ct [Clinical Trial]; serotonin noradrenalin reuptake inhibitor/pd [Pharmacology]; serotonin noradrenalin reuptake inhibitor/ae [Adverse Drug Reaction]; serotonin noradrenalin reuptake inhibitor/dt [Drug Therapy]; serotonin uptake inhibitor/ct [Clinical Trial]; serotonin uptake inhibitor/pd [Pharmacology]; serotonin uptake inhibitor/dt [Drug Therapy]; sodium channel blocking agent/dt [Drug Therapy]; spasmolytic agent/dt [Drug Therapy]; tizanidine/pd [Pharmacology]; tizanidine/ae [Adverse Drug Reaction]; tizanidine/dt [Drug Therapy]; tramadol/pd [Pharmacology]; tramadol/dt [Drug Therapy]; tricyclic antidepressant agent/ct [Clinical Trial]; tricyclic antidepressant agent/pd [Pharmacology]; tricyclic antidepressant agent/ae [Adverse Drug Reaction]; tricyclic antidepressant agent/dt [Drug Therapy]; unindexed drug; warfarin/it [Drug Interaction].

Drug Trade Names and Manufacturers

pennsaid

Link to the Ovid Full Text or citation:

Click here for full text options

19.

Sofosbuvir compassionate use program for patients with severe recurrent hepatitis C after liver transplantation.

Forns X., Charlton M., Denning J., Mchutchison J.G., Symonds W.T., Brainard D., Brandt-Sarif T., Chang P., Kivett V., Castells L., Prieto M., Fontana R.J., Baumert T.F., Coilly A., Londono M.C., Habersetzer F.

Hepatology. 61 (5) (pp 1485-1494), 2015. Date of Publication: 01 May 2015.

AN: 2015901720

Recurrent hepatitis C virus (HCV) infection after liver transplantation (LT) is associated with accelerated progression of liver disease, frequently leading to graft loss and early death. Existing treatment options for severe recurrent HCV infection are limited by suboptimal efficacy, poor tolerability, and numerous drug interactions. We provided sofosbuvir (SOF) and ribavirin (RBV) on a compassionate-use basis to patients with severe recurrent hepatitis C, including those with fibrosing cholestatic hepatitis (FCH) and decompensated cirrhosis who had a life expectancy of 1 year or less. All patients were to receive 24-48 weeks of SOF plus RBV. Investigators could add pegylated interferon to the regimen at their discretion. Data from the first 104 patients who completed or prematurely discontinued treatment by January 1, 2014 are presented. Of the 104 patients analyzed, 52 had an early severe recurrence (diagnosed <12 months after LT) and 52 had cirrhosis (diagnosed >12 months after LT). Twelve patients who underwent retransplantation were excluded from our efficacy analysis. Of the 92 patients assessed, 54 (59%) achieved sustained virological response (SVR) at 12 weeks after the end of treatment, with a higher rate (73%; 35 of 48) in patients with early severe recurrence. Of the 103 patients assessed for clinical outcome, 59 (57%) reported clinical improvement at the last study visit, 23 (22%) were unchanged, 3 (3%) had a worsened clinical status, and 13 (13%) died. Overall, 123 serious adverse events (SAEs) occurred in 49 patients (47%). SAEs associated with hepatic decompensation were the most frequent, with 26 SAEs occurring in 19 patients (18%). Conclusion: SOF and RBV provide high rates of SVR in patients with severe recurrent HCV, including patients with early severe recurrence, FCH, and cirrhosis.

Institution

(Forns, Londono) Liver Unit, IDIBAPS, CIBEREHD, Hospital Clinic, University of Barcelona, Barcelona, Spain (Charlton) Intermountain Transplant Center, Murray, UT, United States

(Denning, Mchutchison, Symonds, Brainard, Brandt-Sarif, Chang, Kivett) Gilead Sciences, Inc, Foster City, CA, United States

(Castells) Liver Unit-Internal Medicine Department, CIBEREHD, Hospital Universitari Vall Hebron, Barcelona, Spain

(Prieto) Hepatology Unit, CIBEREHD, Hospital Universitari i Politecnic La Fe, Valencia, Spain

(Fontana) Department of Internal Medicine, University of Michigan, Ann Arbor, MI, United States

(Baumert, Habersetzer) Hopitaux Universitaires de Strasbourg, Inserm U 1110, Strasbourg, France

(Coilly) Centre Hepato-Bilaire, Hopital Paul Brousse, Inserm UMR-S785, Villejuif, France

Publisher

John Wiley and Sons Inc. (P.O.Box 18667, Newark NJ 07191-8667, United States)

Emtree Heading

acute kidney failure/si [Side Effect]; add on therapy; adolescent; adult; aged; alanine aminotransferase blood level; anemia/si [Side Effect]; aplasia/si [Side Effect]; aplastic anemia/si [Side Effect]; article; ascites/si [Side Effect]; aspartate aminotransferase blood level; autoimmune hemolytic anemia/si [Side Effect]; bacterial peritonitis/si [Side Effect]; bone marrow depression/si [Side Effect]; cholestatic hepatitis; chronic kidney failure/si [Side Effect]; clinical trial; *compassionate use; cytomegalovirus infection/si [Side Effect]; decompensated liver cirrhosis; deep vein thrombosis/si [Side Effect]; diabetes mellitus/si [Side Effect]; drug efficacy; drug safety; drug withdrawal; female; follow up; hemophagocytic syndrome/si [Side Effect]; *hepatitis C/co [Complication]; *hepatitis C/su [Surgery]; *hepatitis C/dt [Drug Therapy]; human; infection/si [Side Effect]; international normalized ratio; kidney dysfunction/si [Side Effect]; kidney failure/si [Side Effect]; kidney infection/si [Side Effect]; life expectancy; *liver transplantation; major clinical study; male; neutropenia/si [Side Effect]; outcome assessment; pancytopenia/si [Side Effect]; pleura empyema/si [Side Effect]; pneumonia/si [Side Effect]; postoperative infection/dt [Drug Therapy]; priority journal; pseudomembranous colitis/si [Side Effect]; *recurrent infection/co [Complication]; *recurrent infection/dt [Drug Therapy]; related/si [Side Effect]; retransplantation; sepsis/si [Side Effect]; single drug dose; Staphylococcus infection/si [Side Effect]; treatment duration; treatment response; tuberculosis/si [Side Effect]; alanine aminotransferase/ec [Endogenous Compound]; albumin/ec [Endogenous Compound]; aspartate aminotransferase/ec [Endogenous Compound]; bilirubin/ec [Endogenous Compound]; peginterferon/ae [Adverse Drug Reaction]; peginterferon/cb [Drug Combination]; peginterferon/dt [Drug Therapy]; *ribavirin/ae [Adverse Drug Reaction]; *ribavirin/cb [Drug Combination]; *ribavirin/dt [Drug Therapy]; *ribavirin/ct [Clinical Trial]; *sofosbuvir/ae [Adverse Drug Reaction]; *sofosbuvir/cb [Drug Combination]; *sofosbuvir/dt [Drug Therapy]; *sofosbuvir/ct [Clinical Trial].

Link to the Ovid Full Text or citation:

Click here for full text options

20.

Predictors of pulmonary tuberculosis treatment outcomes in South Korea: a prospective cohort study, 2005-2012.

Choi H., Lee M., Chen R.Y., Kim Y., Yoon S., Joh J.S., Park S.K., Dodd L.E., Lee J., Song T., Cai Y., Goldfeder L.C., Via L.E., Carroll M.W., Barry C.E., Cho S.-N.

BMC infectious diseases. 14 (pp 360), 2014. Date of Publication: 2014.

AN: 24990578

BACKGROUND: Tuberculosis remains an important health concern in many countries. The aim of this study was to identify predictors of unfavorable outcomes at the end of treatment (EOT) and at the end of study (EOS; 40 months after EOT) in South Korea. METHODS: New or previously treated tuberculosis patients were recruited into a prospective observational cohort study at two hospitals in South Korea. To identify predictors of unfavorable outcomes at EOT and EOS, logistic regression analysis was performed.

RESULTS: The proportion of multidrug-resistant tuberculosis (MDR-TB) was 8.2% in new cases and 57.9% in previously treated cases. Of new cases, 68.6% were cured, as were 40.7% of previously treated cases. At EOT, diabetes, >3 previous TB episodes, >1 significant regimen change, and MDR-TB were significantly associated with treatment failure or death. At EOS, age >35, body-mass index (BMI) <18.5, diabetes, and MDR-TB were significantly associated with treatment failure, death, or relapse. Among cases that were cured at EOT, age >50 and a BMI <18.5 were associated with subsequent death or relapse during follow-up to EOS. Treatment interruption was associated with service sector employees or laborers, bilateral lesions on chest X-ray, and previous treatment failure or treatment interruption history.

CONCLUSIONS: Risk factors for poor treatment outcomes at EOT and EOS include both patient factors (diabetes status, age, BMI) and disease factors (history of multiple previous treatment episodes, MDR-TB). In this longitudinal, observational cohort study, diabetes mellitus and MDR-TB were risk factors for poor treatment outcomes and relapse. Measures to help ensure that the first tuberculosis treatment episode is also the last one may improve treatment outcomes.

TRIAL REGISTRATION: ClinicalTrials.gov ID: NCT00341601.

Institution

(Cho) International Tuberculosis Research Center, Changwon, Republic of Korea. raycho@yonsei.kr

Emtree Heading

adult; cohort analysis; female; human; male; middle aged; prospective study; risk factor; South Korea; treatment failure; treatment outcome; Tuberculosis, Multidrug-Resistant/dt [Drug Therapy]; Tuberculosis, Multidrug-Resistant/ep [Epidemiology]; Tuberculosis, Pulmonary/dt [Drug Therapy]; Tuberculosis, Pulmonary/ep [Epidemiology]; tuberculostatic agent/dt [Drug Therapy].

Link to the Ovid Full Text or citation:

Click here for full text options

21.

Review of evidence for measuring drug concentrations of first-line antitubercular agents in adults.

Wilby K.J., Ensom M.H., Marra F.

Clinical pharmacokinetics. 53 (10) (pp 873-890), 2014. Date of Publication: 01 Oct 2014.

AN: 25172553

Measurement of drug concentrations and performing therapeutic drug monitoring (TDM) are widely used to optimize efficacy and safety of many commonly used drugs today. Although TDM of first-line antitubercular drugs is used during the treatment of tuberculosis, the extent of any benefit achieved is currently unknown. This review summarizes the available literature describing TDM of first-line treatment agents in patients with tuberculosis and describes clinical associations with achievement of target drug concentrations, including data from special populations. A literature review was conducted for articles describing drug concentration and TDM outcomes for first-line tuberculosis agents in adults. A total of 40 studies were included in the review. Studies were a mixture of controlled trials, observational studies, cross-sectional studies, and case reports. The majority of the studies showed standard dosing does not consistently achieve target concentrations for the first-line antitubercular drugs; however, the clinical implications of this finding are still unclear. Presence of HIV and diabetes mellitus appeared to indicate achievement of lower than target concentrations and this warrants further study in prospective studies. Current published data neither prove nor disprove the utility of TDM for general tuberculosis populations but evidence does not currently support routine measurement of drug concentrations.

Institution

(Wilby, Ensom, Marra) College of Pharmacy, Qatar University, PO Box 2713, Doha, Qatar, kjw@qu.edu.qa

Emtree Heading

adult; *blood; diabetes mellitus; *drug monitoring; human; Human immunodeficiency virus infection; treatment outcome; tuberculosis/dt [Drug Therapy]; tuberculostatic agent.

Link to the Ovid Full Text or citation:

Click here for full text options

22.

Does Alcohol consumption during multidrug-resistant tuberculosis treatment affect outcome?. A population-based study in Kerala, India.

Duraisamy K., Mrithyunjayan S., Ghosh S., Nair S.A., Balakrishnan S., Subramoniapillai J., Oeltmann J.E., Moonan P.K., Kumar A.M.

Annals of the American Thoracic Society. 11 (5) (pp 712-718), 2014. Date of Publication: 01 Jun 2014.

AN: 24735096

RATIONALE: India reports the largest number of multidrug-resistant tuberculosis cases in the world; yet, no longitudinal study has assessed factors related to treatment outcomes under programmatic conditions in the public sector. OBJECTIVES: To describe demographic, clinical, and risk characteristics associated with treatment outcomes for all patients with multidrug-resistant tuberculosis registered in the Revised National Tuberculosis Control Programme, Kerala State, India from January 1, 2009 to June 30, 2010.

METHODS: Cox regression methods were used to calculate adjusted hazard ratios with 95% confidence intervals (CIs) to assess factors associated with an unsuccessful treatment outcome.

MEASUREMENTS AND MAIN RESULTS: Of 179 patients with multidrug-resistant tuberculosis registered, 112 (63%) had successful treatment outcomes (77 bacteriologically cured, 35 treatment completed) and 67 (37%) had unsuccessful treatment outcomes (30 died, 26 defaulted, 9 failed treatment, 1 stopped treatment because of drug-related adverse events, and 1 developed extensively drug-resistant tuberculosis). The hazard for unsuccessful outcome was significantly higher among patients who consumed alcohol during treatment (adjusted hazard ratio, 4.3; 95% CI, 1.1-17.6) than those who did not. Persons who consumed alcohol during treatment, on average, missed 18 more intensive-phase doses (95% CI, 13-22) than those who did not. Although many patients had diabetes (33%), were ever smokers (39%), or had low body mass index (47%), these factors were not associated with outcome.

CONCLUSION: Overall treatment success was greater than global and national averages; however, outcomes among patients consuming alcohol remained poor. Integration of care for multidrug-resistant tuberculosis and alcoholism should be considered to improve treatment adherence and outcomes.

Institution

(Duraisamy, Mrithyunjayan, Ghosh, Nair, Balakrishnan, Subramoniapillai, Oeltmann, Moonan, Kumar) 1 Department of Tuberculosis, WHO Country Office for India, New Delhi, India

Emtree Heading

adult; clinical trial; drinking behavior; female; follow up; *health survey; human; incidence; India; isolation and purification; male; microbiology; middle aged; multicenter study; Mycobacterium tuberculosis; retrospective study; *risk assessment; risk factor; sputum; survival rate; treatment outcome; trends; Tuberculosis, Multidrug-Resistant/dt [Drug Therapy]; Tuberculosis, Multidrug-Resistant/ep [Epidemiology]; tuberculostatic agent/dt [Drug Therapy].

Link to the Ovid Full Text or citation:

Click here for full text options

23.

Review of Evidence for Measuring Drug Concentrations of First-Line Antitubercular Agents in Adults.

Wilby K.J., Ensom M.H.H., Marra F.

Clinical Pharmacokinetics. 53 (10) (pp 873-890), 2014. Date of Publication: 23 Sep 2014.

AN: 2014983133

Measurement of drug concentrations and performing therapeutic drug monitoring (TDM) are widely used to optimize efficacy and safety of many commonly used drugs today. Although TDM of first-line antitubercular drugs is used during the treatment of tuberculosis, the extent of any benefit achieved is currently unknown. This review summarizes the available literature describing TDM of first-line treatment agents in patients with tuberculosis and describes clinical associations with achievement of target drug concentrations, including data from special populations. A literature review was conducted for articles describing drug concentration and TDM outcomes for first-line tuberculosis agents in adults. A total of 40 studies were included in the review. Studies were a mixture of controlled trials, observational studies, cross-sectional studies, and case reports. The majority of the studies showed standard dosing does not consistently achieve target concentrations for the first-line antitubercular drugs; however, the clinical implications of this finding are still unclear. Presence of HIV and diabetes mellitus appeared to indicate achievement of lower than target concentrations and this warrants further study in prospective studies. Current published data neither prove nor disprove the utility of TDM for general tuberculosis populations but evidence does not currently support routine measurement of drug concentrations.

Institution

(Wilby) College of Pharmacy, Qatar University, PO Box 2713, Doha, Qatar (Ensom, Marra) University of British Columbia, 2405 Wesbrook Mall, Vancouver, BC V6T 1Z3, Canada

Publisher

Springer International Publishing

Emtree Heading

case report; comorbidity; controlled clinical trial (topic); cross-sectional study; diabetes mellitus; drug blood level; *drug monitoring; human; Human immunodeficiency virus infection; meta analysis; observational study; outcome assessment; randomized controlled trial (topic); review; systematic review; time to maximum plasma concentration; tuberculosis/dt [Drug Therapy]; ethambutol/dt [Drug Therapy]; ethambutol/cr [Drug Concentration]; ethambutol/pk [Pharmacokinetics]; isoniazid/dt [Drug Therapy]; isoniazid/cr [Drug Concentration]; isoniazid/pk [Pharmacokinetics]; isoniazid plus rifampicin/dt [Drug Therapy]; isoniazid plus rifampicin/cr [Drug Concentration]; isoniazid plus rifampicin/pk [Pharmacokinetics]; moxifloxacin/dt [Drug Therapy]; moxifloxacin/cr [Drug Concentration]; moxifloxacin/pk [Pharmacokinetics]; pyrazinamide/dt [Drug Therapy]; pyrazinamide/cr [Drug Concentration]; pyrazinamide/pk [Pharmacokinetics]; rifabutin/dt [Drug Therapy]; rifabutin/cr [Drug Concentration]; rifabutin/pk [Pharmacokinetics]; rifampicin/dt [Drug Therapy]; rifampicin/cr [Drug Concentration]; rifampicin/pk [Pharmacokinetics]; *tuberculostatic agent/dt [Drug Therapy]; *tuberculostatic agent/ct [Clinical Trial]; *tuberculostatic agent/cr [Drug Concentration]; *tuberculostatic agent/pk [Pharmacokinetics].

Link to the Ovid Full Text or citation:

Click here for full text options

24.

Results of the implementation of a pilot model for the bidirectional screening and joint management of patients with pulmonary Tuberculosis and Diabetes mellitus in Mexico.

Castellanos-Joya M., Delgado-Sanchez G., Ferreyra-Reyes L., Cruz-Hervert P., Ferreira-Guerrero E., Ortiz-Solis G., Jimenez M.I., Salazar L.L., Montero-Campos R., Mongua-Rodriguez N., Baez-Saldana R., Bobadilla-Del-Valle M., Gonza Lez-Roldan J.F., Ponce-De-Leon A., Sifuentes-Osornio J., Garcia-Garcia L.

PLoS ONE. 9 (9) (no pagination), 2014. Article Number: e106961. Date of Publication: 17 Sep 2014.

AN: 2014804910

Objective: To evaluate the feasibility and effectiveness of the CFTB/DM in Mexico. Design. Prospective observational cohort. Setting. 15 primary care units in 5 states in Mexico. Participants: Patients aged >20 years diagnosed with DM or pulmonary TB who sought care at participating clinics. Intervention: The WHO/Union CFTB/DM was adapted and implemented according to official Mexican guidelines. We recruited participants from July 2012 to April 2013 and followed up until March 2014. Bidirectional screening was performed. Patients diagnosed with TB and DM were invited to receive TB treatment under joint management. Main outcome measures. Diagnoses of TB among DM, of DM among TB, and treatment outcomes among patients with DM and TB. Background: Recently, the World Health Organisation and the International Union Against Tuberculosis and Lung Disease published a Collaborative Framework for the Care and Control of Tuberculosis (TB) and Diabetes (DM) (CFTB/DM) proposing bidirectional screening and joint management.

Results: Of 783 DM patients, 11 (1.4%) were unaware of their TB. Of 361 TB patients, 16 (4.4%) were unaware of their DM. 95 TB/DM patients accepted to be treated under joint management, of whom 85 (89.5%) successfully completed treatment. Multiple linear regression analysis with change in HbA1c and random capillary glucose as dependent variables revealed significant decrease with time (regression coefficients (beta) =20.660, (95% confidence interval (CI), 20.96 to 20.35); and beta =21.889 (95% CI, 22.77 to 21.01, respectively)) adjusting by sex, age and having been treated for a previous TB episode. Patients treated under joint management were more likely to experience treatment success than patients treated under routine DM and TB programs as compared to historical (adjusted OR (aOR), 2.8, 95%CI 1.28-6.13) and same period (aOR 2.37, 95% CI 1.13-4.96) comparison groups.

Conclusions: Joint management of TB and DM is feasible and appears to improve clinical outcomes.

Institution

(Castellanos-Joya, Jimenez, Salazar) Direccion Del Programa de Micobacteriosis, Centro Nacional de Programas Preventivos y Control de Enfermedades, Mexico, Distrito Federal, Mexico (Delgado-Sanchez, Ferreyra-Reyes, Cruz-Hervert, Ferreira-Guerrero, Montero-Campos, Mongua-Rodriguez, Baez-Saldana, Garcia-Garcia) Centro de Investigacion Sobre Enfermedades Infecciosas, Instituto Nacional de Salud Publica, Cuernavaca, Morelos, Mexico

(Ortiz-Solis) Direccion Del Programa de Salud en El Adulto y en El Anciano, Centro Nacional de Programas Preventivos y Control de Enfermedades, Mexico, Distrito Federal, Mexico

(Bobadilla-Del-Valle, Ponce-De-Leon) Laboratorio de Microbiologia, Instituto Nacional de Ciencias Medicas y de Nutricion Salvador Zubira N, Mexico, Distrito Federal, Mexico

(Gonza Lez-Roldan) Direccion General, Centro Nacional de Programas Preventivos y Control de Enfermedades, Mexico, Distrito Federal, Mexico

(Sifuentes-Osornio) Direccion Medica, Instituto Nacional de Ciencias Medicas y de Nutricion Salvador Zubiran, Mexico, Distrito Federal, Mexico

Publisher

Public Library of Science

Emtree Heading

adult; age; aged; article; *bidirectional screening; clinical effectiveness; cohort analysis; Collaborative Framework for the Care and Control of Tuberculosis and Diabetes; control strategy; controlled study; *diabetes mellitus/di [Diagnosis]; *diabetes mellitus/ep [Epidemiology]; *diabetes mellitus/pc [Prevention]; *diabetes mellitus/dt [Drug Therapy]; disease control; feasibility study; female; follow up; glucose blood level; *health care planning; hemoglobin blood level; human; human cell; *lung tuberculosis/di [Diagnosis]; *lung tuberculosis/ep [Epidemiology]; *lung tuberculosis/pc [Prevention]; *lung tuberculosis/dt [Drug Therapy]; major clinical study; male; Mexico; multiple linear regression analysis; observational study; *patient care; physical activity; pilot study; prospective study; screening; sex difference; glucose/ec [Endogenous Compound]; hemoglobin A1c/ec [Endogenous Compound]; insulin/dt [Drug Therapy]; isoniazid/dt [Drug Therapy]; oral antidiabetic agent/po [Oral Drug Administration]; oral antidiabetic agent/dt [Drug Therapy]; rifampicin/dt [Drug Therapy].

Link to the Ovid Full Text or citation:

Click here for full text options

25.

Update on Janus Kinase Antagonists in Inflammatory Bowel Disease.

Boland B.S., Sandborn W.J., Chang J.T.

Gastroenterology Clinics of North America. 43 (3) (pp 603-617), 2014. Date of Publication: September 2014.

AN: 2014534256

Janus kinase (JAK) inhibitors have emerged as a novel orally administered small-molecule therapy for the treatment of ulcerative colitis and possibly Crohn disease. These molecules are designed to selectively target the activity of specific JAKs and to offer a targeted mechanism of action without risk of immunogenicity. Based on data from clinical trials in rheumatoid arthritis and phase 2 studies in inflammatory bowel disease, tofacitinib and other JAK inhibitors are likely to become a new form of medical therapy for the treatment of inflammatory bowel disease. © 2014 Elsevier Inc.

Institution

(Boland, Sandborn, Chang) Division of Gastroenterology, Department of Medicine, Inflammatory Bowel Disease Center, University of California San Diego, 9500 Gilman Drive, La Jolla, CA 92093, United States (Boland, Sandborn, Chang) Digestive Diseases Research Development Center, University of California San Diego, 9500 Gilman Drive, La Jolla, CA 92093, United States

Publisher

W.B. Saunders

Emtree Heading

abdominal pain/si [Side Effect]; autoimmune disease/dt [Drug Therapy]; bone marrow suppression; bronchiectasis/si [Side Effect]; bronchitis/si [Side Effect]; cancer risk; cardiovascular disease/si [Side Effect]; cellulitis/si [Side Effect]; cholesterol blood level; colitis/dt [Drug Therapy]; congestive heart failure/si [Side Effect]; Crohn disease/dt [Drug Therapy]; diabetic foot/si [Side Effect]; disease exacerbation/si [Side Effect]; drug dose comparison; drug efficacy; drug fatality/si [Side Effect]; drug mechanism; drug safety; drug tolerability; drug withdrawal; dyslipidemia/si [Side Effect]; enzyme analysis; enzyme deficiency; enzyme inhibition; genetic association; graft rejection/dt [Drug Therapy]; herpes zoster/si [Side Effect]; human; infection/si [Side Effect]; infection risk; *inflammatory bowel disease/dt [Drug Therapy]; influenza/si [Side Effect]; lipoprotein blood level; liver abscess/si [Side Effect]; liver toxicity/si [Side Effect]; lung tuberculosis/si [Side Effect]; lymphoma/si [Side Effect]; malignant neoplastic disease/si [Side Effect]; multicenter study (topic); myelofibrosis/dt [Drug Therapy]; nausea/si [Side Effect]; nephrotoxicity/si [Side Effect]; neutropenia/si [Side Effect]; neutrophil count; nonhuman; opportunistic infection/si [Side Effect]; outcome assessment; perianal abscess/si [Side Effect]; phase 1 clinical trial (topic); phase 2 clinical trial(topic); phase 3 clinical trial (topic); pneumonia/si [Side Effect]; protein expression; protein family; psoriasis/dt [Drug Therapy]; pulmonary cryptococcosis/si [Side Effect]; randomized controlled trial (topic); review; rheumatoid arthritis/dt [Drug Therapy]; rhinopharyngitis/si [Side Effect]; sepsis/si [Side Effect]; side effect/si [Side Effect]; signal transduction; single nucleotide polymorphism; solid tumor/si [Side Effect]; treatment duration; tuberculous pleurisy/si [Side Effect]; ulcerative colitis/dt [Drug Therapy]; vomiting/si [Side Effect]; vulva abscess/si [Side Effect]; vulva disease/si [Side Effect]; adalimumab/cb [Drug Combination]; adalimumab/cm [Drug Comparison]; adalimumab/dt [Drug Therapy]; baricitinib/dt [Drug Therapy]; baricitinib/pd [Pharmacology]; certolizumab pegol/ct [Clinical Trial]; certolizumab pegol/dt [Drug Therapy]; cytokine receptor/ec [Endogenous Compound]; cytokine receptor 1/ec [Endogenous Compound]; cytokine receptor 2/ec [Endogenous Compound]; decernotinib/ct [Clinical Trial]; decernotinib/dt [Drug Therapy]; decernotinib/pd [Pharmacology]; disease modifying antirheumatic drug/cb [Drug Combination]; disease modifying antirheumatic drug/dt [Drug Therapy]; glpg 0634/ct [Clinical Trial]; glpg 0634/dt [Drug Therapy]; glpg 0634/pd [Pharmacology]; glpg 0974/ct [Clinical Trial]; glpg 0974/dt [Drug Therapy]; glpg 0974/po [Oral Drug Administration]; glpg 0974/pd [Pharmacology]; high density lipoprotein/ec [Endogenous Compound]; Janus kinase 1/ec [Endogenous Compound]; Janus kinase 2/ec [Endogenous Compound]; Janus kinase 3/ec [Endogenous Compound]; *Janus kinase inhibitor/dt [Drug Therapy]; jnj 54781532/ct [Clinical Trial]; jnj 54781532/dt [Drug Therapy]; jnj 54781532/pd [Pharmacology]; low density lipoprotein cholesterol/ec [Endogenous Compound]; methotrexate/cb [Drug Combination]; methotrexate/dt [Drug Therapy]; peficitinib/pd [Pharmacology]; placebo; ruxolitinib/ct [Clinical Trial]; ruxolitinib/cm [Drug Comparison]; ruxolitinib/dt [Drug Therapy]; ruxolitinib/pd [Pharmacology]; STAT protein/ec [Endogenous Compound]; tofacitinib/ae [Adverse Drug Reaction]; tofacitinib/ct [Clinical Trial]; tofacitinib/cb [Drug Combination]; tofacitinib/cm [Drug Comparison]; tofacitinib/do [Drug Dose]; tofacitinib/dt [Drug Therapy]; tofacitinib/pd [Pharmacology]; unclassified drug.

Drug Trade Names and Manufacturers

AbbVie, Astellas, Galapagos, Incyte, Janssen, Lilly, Vertex, asp 015k: Astellas, cp 690550, glpg 0634: AbbVie, glpg 0634: Galapagos, glpg 0974: Galapagos, incb 028050, jnj 54781532: Janssen, vx 509: Vertex

Link to the Ovid Full Text or citation:

Click here for full text options

26.

Is there an effect of glucose lowering treatment on incidence and prognosis of tuberculosis? A systematic review.

Jorgensen M.E., Faurholt-Jepsen D.

Current Diabetes Reports. 14 (7) (no pagination), 2014. Article Number: 505. Date of Publication: July 2014.

AN: 2014443596

The association between diabetes and incidence of tuberculosis is well established, and observational studies have shown poor treatment outcome in tuberculosis related to hyperglycemia. The WHO recommends screening for diabetes among all patients with tuberculosis and optimized glycemic control aiming at improving tuberculosis outcome. However, no intervention studies support this notion. Patients with tuberculosis are often vulnerable with high degree of comorbidity, and, therefore, at high risk of adverse effects of intensive glucose control. Controlled intervention studies of the effect of glucose lowering treatment on tuberculosis outcomes are clearly warranted to justify screening for- and tight control of diabetes. © 2014 Springer Science+Business Media New York.

Institution

(Jorgensen) Steno Diabetes Center, Niels Steensensvej 2, DK-2820 Gentofte, Denmark (Faurholt-Jepsen) Department of Nutrition, Exercise, and Sports, University of Copenhagen, Frederiksberg, Denmark

(Faurholt-Jepsen) Department of Infectious Diseases, Rigshospitalet, Copenhagen, Denmark

Publisher

Current Medicine Group LLC 1

Emtree Heading

area under the curve; article; clinical trial (topic); comorbidity; diabetes control; *diabetes mellitus/dt [Drug Therapy]; diabetic patient; disease association; drug bioavailability; drug efficacy; drug potentiation; drug safety; drug treatment failure; Embase; glucose blood level; glycemic control; high risk patient; human; *hyperglycemia; incidence; insulin resistance; Medical Subject Headings; Medline; multidrug resistant tuberculosis; observational study; prevalence; prognosis; screening test; sputum culture; systematic review; thorax radiography; treatment outcome; *tuberculosis; antidiabetic agent/dt [Drug Therapy]; ethambutol; *glucose/ec [Endogenous Compound]; insulin; isoniazid; placebo; pyrazinamide; rifampicin; saxagliptin.

Link to the Ovid Full Text or citation:

Click here for full text options

27.

Tobacco smoking, alcohol drinking, diabetes, low body mass index and the risk of self-reported symptoms of active tuberculosis: Individual Participant Data (IPD) meta-analyses of 72,684 individuals in 14 high tuberculosis burden countries.

Patra J., Jha P., Rehm J., Suraweera W.

PLoS ONE. 9 (5) (no pagination), 2014. Article Number: e96433. Date of Publication: 02 May 2014.

AN: 2014330403

Background: The effects of multiple exposures on active tuberculosis (TB) are largely undetermined. We sought to establish a dose-response relationship for smoking, drinking, and body mass index (BMI) and to investigate the independent and joint effects of these and diabetes on the risk of self-reported symptoms of active TB disease. Methods and Findings: We analyzed 14 national studies in 14 high TB-burden countries using self-reports of blood in cough/phlegm and cough lasting > = 3 weeks in the last year as the measures of symptoms of active TB. The random effect estimates of the relative risks (RR) between active TB and smoking, drinking, diabetes, and BMI<18.5 kg/m 2 were reported for each gender. Floating absolute risks were used to examine dyads of exposure. Adjusted for age and education, the risks of active TB were significantly associated with diabetes and BMI<18.5 kg/m2 in both sexes, with ever drinking in men and with ever smoking in women. Stronger dose-response relationships were seen in women than in men for smoking amount, smoking duration and drinking amount but BMI<18.5 kg/m2 showed a stronger dose-response relationship in men. In men, the risks from joint exposures were statistically significant for diabetics with BMI<18.5 kg/m2 (RR = 6.4), diabetics who smoked (RR = 3.8), and diabetics who drank alcohol (RR = 3.2). The risks from joint risk factors were generally larger in women than in men, with statistically significant risks for diabetics with BMI<18.5 kg/m2 (RR = 10.0), diabetics who smoked (RR = 5.4) and women with BMI<18.5 kg/m2 who smoked (RR = 5.0). These risk factors account for 61% of male and 34% of female estimated TB incidents in these 14 countries. Conclusions: Tobacco, alcohol, diabetes, and low BMI are significant individual risk factors but in combination are associated with triple or quadruple the risk of development of recent active TB. These risk factors might help to explain the wide variation in TB across countries. © 2014 Patra et al.

Institution

(Patra, Jha, Suraweera) Centre for Global Health Research, St. Michael's Hospital, Toronto, ON, Canada (Patra, Jha, Rehm) Dalla Lana School of Public Health, University of Toronto, Toronto, ON, Canada

(Rehm) Centre for Addiction and Mental Health, Toronto, ON, Canada

Publisher

Public Library of Science

Emtree Heading

adult; article; *body mass; controlled study; coughing; *diabetes mellitus; disease association; disease duration; dose response; *drinking behavior; female; geographic distribution; human; major clinical study; male; middle aged; outcomes research; pathogenesis; risk factor; self report; sex difference; *smoking; smoking habit; *tuberculosis/et [Etiology]; young adult.

Link to the Ovid Full Text or citation:

Click here for full text options

28.

Effects of early versus delayed initiation of antiretroviral treatment on clinical outcomes of HIV-1 infection: Results from the phase 3 HPTN 052 randomised controlled trial.

Grinsztejn B., Hosseinipour M.C., Ribaudo H.J., Swindells S., Eron J., Chen Y.Q., Wang L., Ou S.-S., Anderson M., McCauley M., Gamble T., Kumarasamy N., Hakim J.G., Kumwenda J., Pilotto J.H.S., Godbole S.V., Chariyalertsak S., De Melo M.G., Mayer K.H., Eshleman S.H., Piwowar-Manning E., Makhema J., Mills L.A., Panchia R., Sanne I., Gallant J., Hoffman I., Taha T.E., Nielsen-Saines K., Celentano D., Essex M., Havlir D., Cohen M.S.

The Lancet Infectious Diseases. 14 (4) (pp 281-290), 2014. Date of Publication: April 2014.

AN: 2014203771

Background: Use of antiretroviral treatment for HIV-1 infection has decreased AIDS-related morbidity and mortality and prevents sexual transmission of HIV-1. However, the best time to initiate antiretroviral treatment to reduce progression of HIV-1 infection or non-AIDS clinical events is unknown. We reported previously that early antiretroviral treatment reduced HIV-1 transmission by 96%. We aimed to compare the effects of early and delayed initiation of antiretroviral treatment on clinical outcomes. Methods: The HPTN 052 trial is a randomised controlled trial done at 13 sites in nine countries. We enrolled HIV-1-serodiscordant couples to the study and randomly allocated them to either early or delayed antiretroviral treatment by use of permuted block randomisation, stratified by site. Random assignment was unblinded. The HIV-1-infected member of every couple initiated antiretroviral treatment either on entry into the study (early treatment group) or after a decline in CD4 count or with onset of an AIDS-related illness (delayed treatment group). Primary events were AIDS clinical events (WHO stage 4 HIV-1 disease, tuberculosis, and severe bacterial infections) and the following serious medical conditions unrelated to AIDS: serious cardiovascular or vascular disease, serious liver disease, end-stage renal disease, new-onset diabetes mellitus, and non-AIDS malignant disease. Analysis was by intention-to-treat. This trial is registered with ClinicalTrials.gov, number NCT00074581. Findings: 1763 people with HIV-1 infection and a serodiscordant partner were enrolled in the study; 886 were assigned early antiretroviral treatment and 877 to the delayed treatment group (two individuals were excluded from this group after randomisation). Median CD4 counts at randomisation were 442 (IQR 373-522) cells per muL in patients assigned to the early treatment group and 428 (357-522) cells per muL in those allocated delayed antiretroviral treatment. In the delayed group, antiretroviral treatment was initiated at a median CD4 count of 230 (IQR 197-249) cells per muL. Primary clinical events were reported in 57 individuals assigned to early treatment initiation versus 77 people allocated to delayed antiretroviral treatment (hazard ratio 0.73, 95% CI 0.52-1.03; p=0.074). New-onset AIDS events were recorded in 40 participants assigned to early antiretroviral treatment versus 61 allocated delayed initiation (0.64, 0.43-0.96; p=0.031), tuberculosis developed in 17 versus 34 patients, respectively (0.49, 0.28-0.89, p=0.018), and primary non-AIDS events were rare (12 in the early group vs nine with delayed treatment). In total, 498 primary and secondary outcomes occurred in the early treatment group (incidence 24.9 per 100 person-years, 95% CI 22.5-27.5) versus 585 in the delayed treatment group (29.2 per 100 person-years, 26.5-32.1; p=0.025). 26 people died, 11 who were allocated to early antiretroviral treatment and 15 who were assigned to the delayed treatment group. Interpretation: Early initiation of antiretroviral treatment delayed the time to AIDS events and decreased the incidence of primary and secondary outcomes. The clinical benefits recorded, combined with the striking reduction in HIV-1 transmission risk previously reported, provides strong support for earlier initiation of antiretroviral treatment. Funding: US National Institute of Allergy and Infectious Diseases. © 2014 Elsevier Ltd.

Institution

(Chen, Wang, Ou, Anderson) Fred Hutchinson Cancer Research Center, Seattle, WA, United States (Taha, Celentano) Johns Hopkins Bloomberg School of Public Health, Baltimore, MD, United States

(Chariyalertsak) Research Institute for Health Sciences, Chiang Mai University, Chaing Mai, Thailand

(Hosseinipour, Eron, Hoffman, Cohen) University of North Carolina School of Medicine, Chapel Hill, NC, United States

(Eshleman, Piwowar-Manning, Gallant) Johns Hopkins University School of Medicine, Baltimore, MD, United States

(Ribaudo, Essex) Harvard School of Public Health, Boston, MA, United States

(Gamble) FHI 360, Durham, NC, United States

(Godbole) National AIDS Research Institute (ICMR), Pune, India

(De Melo) Hospital Nossa Senhora da Conceicao, Porto Alegre RS, Brazil

(Grinsztejn) Instituto de Pesquisa Clinica Evandro Chagas, Fiocruz, Rio de Janeiro, Brazil

(Hakim) University of Zimbabwe, Harare, Zimbabwe

(Havlir) University of California, San Francisco, CA, United States

(Hosseinipour) UNC Project-Malawi, Lilongwe, Malawi

(Kumarasamy) Y R Gaitonade Center for AIDS Research and Education, Chennai, India

(Kumwenda) College of Medicine-Johns Hopkins Project, Lilongwe, Malawi

(Makhema) Botswana Harvard AIDS Institute, Gabarone, Botswana

(Mayer) Fenway Institute, Boston, MA, United States

(McCauley) FHI 360, Washington, DC, United States

(Mills) KEMRI-CDC, Kisumu, Kenya

(Nielsen-Saines) David Geffen UCLA School of Medicine, Los Angeles, CA, United States

(Panchia, Sanne) Faculty of Health Sciences, University of the Witwatersrand, Johannesburg, South Africa

(Pilotto) Hospital Geral de Nova Iguacu and Laboratorio de AIDS e Imunologia Molecular-IOC/Fiocruz, Rio de Janeiro, Brazil

(Swindells) University of Nebraska Medical Center, Omaha, NE, United States

Publisher

Lancet Publishing Group

Emtree Heading

adult; *antiviral therapy; article; bacterial infection; cardiovascular disease; CD4 lymphocyte count; chronic kidney failure; controlled study; diabetes mellitus; disease course; *early intervention; end stage renal disease; female; follow up; hepatitis B; human; *Human immunodeficiency virus 1 infection/dt [Drug Therapy]; hypertension; hypertransaminasemia; intention to treat analysis; lactic acidosis; lipodystrophy; liver disease; major clinical study; malaria; male; multicenter study; peripheral neuropathy; phase 3 clinical trial; priority journal; randomized controlled trial; *therapy delay; thrombocytopenia; time to treatment; treatment duration; treatment outcome; tuberculosis; vascular disease; virus load; *antivirus agent/dt [Drug Therapy]; cotrimoxazole/dt [Drug Therapy]; efavirenz/cb [Drug Combination]; efavirenz/dt [Drug Therapy]; hemoglobin/ec [Endogenous Compound]; isoniazid/dt [Drug Therapy]; lamivudine/cb [Drug Combination]; lamivudine/dt [Drug Therapy]; zidovudine/cb [Drug Combination]; zidovudine/dt [Drug Therapy].

Link to the Ovid Full Text or citation:

Click here for full text options

29.

Risk factors for poor treatment outcomes in patients with MDR-TB and XDR-TB in China: retrospective multi-center investigation.

Tang S., Tan S., Yao L., Li F., Li L., Guo X., Liu Y., Hao X., Li Y., Ding X., Zhang Z., Tong L., Huang J.

PloS one. 8 (12) (pp e82943), 2013. Date of Publication: 2013.

AN: 24349402

BACKGROUND: The treatment of patients with MDR- and XDR-TB is usually more complex, toxic and costly and less effective than treatment of other forms of TB. However, there is little information available on risk factors for poor outcomes in patients with MDR- and XDR-TB in China. METHODOLOGY/PRINCIPAL FINDINGS: We retrospectively analyzed the clinical records of HIV-negative TB Patients with culture-proven MDR- or XDR-TB who were registered from July 2006 to June 2011 at five large-scale Tuberculosis Specialized Hospitals in China. Among 1662 HIV-seronegative TB cases which were culture-positive for M. tuberculosis complex and had positive sputum-smear microscopy results, 965 cases (58.1%) were DR-TB, and 586 cases (35.3%) were classified as having MDR-TB, accounting for 60.7% of DR-TB. 169 cases (10.2%) were XDR-TB, accounting for 17.5% of DR-TB, 28.8% of MDR-TB. The MDR-TB patients were divided into XDR-TB group (n=169) and other MDR-TB group (non-XDR MDR-TB) (n=417). In total, 240 patients (40.95%) had treatment success, and 346 (59.05%) had poor treatment outcomes. The treatment success rate in other MDR-TB group was 52.2%, significantly higher than that in the XDR-TB group (13%, P<0.001). In multivariate logistic regression analysis, poor outcomes were associated with duration of previous anti-TB treatment of more than one year (OR, 0.077; 95% CI, 0.011-0.499, P<0.001), a BMI less than 18.5 kg/m(2) (OR, 2.185; 95% CI, 1.372-3.478, P<0.001), XDR (OR, 13.368; 95% CI, 6.745-26.497, P<0.001), retreatment (OR, 0.171; 95% CI, 0.093-0.314, P<0.001), diabetes (OR, 0.305; 95% CI, 0.140-0.663, P=0.003), tumor (OR, 0.095; 95% CI, 0.011-0.795, P=0.03), decreased albumin (OR, 0.181; 95% CI, 0.118-0.295, P<0.001), cavitation (OR, 0.175; 95% CI, 0.108-0.286, P<0.001).

CONCLUSIONS/SIGNIFICANCE: The patients with MDR-TB and XDR-TB have poor treatment outcomes in China.The presence of extensive drug resistance, low BMI, hypoalbuminemia, comorbidity, cavitary disease and previous anti-TB treatment are independent prognostic factors for poor outcome in patients with MDR-TB.

Institution

(Tang) Department of Respiratory Medicine, The First Affiliated Hospital of Soochow University, Suzhou, China ; Tuberculosis Center for Diagnosis and Treatment, Shanghai Pulmonary Hospital, Tongji University School of Medicine, Shanghai, China (Tan) Department of Tuberculosis, Guangzhou Chest Hospital, State Key Laboratory of Respiratory Disease, Guangzhou, China

(Yao) Tuberculosis Center for Diagnosis and Treatment, Shanghai Pulmonary Hospital, Tongji University School of Medicine, Shanghai, China

(Li) Hangzhou Red Cross Hospital, Hangzhou, China

(Li) Tianjin Haihe Hospital, Tianjin, China

(Guo) Henan Infectious Hospital, Zhengzhou, China

(Liu) Tuberculosis Center for Diagnosis and Treatment, Shanghai Pulmonary Hospital, Tongji University School of Medicine, Shanghai, China

(Hao) Tuberculosis Center for Diagnosis and Treatment, Shanghai Pulmonary Hospital, Tongji University School of Medicine, Shanghai, China

(Li) Department of Tuberculosis, Guangzhou Chest Hospital, State Key Laboratory of Respiratory Disease, Guangzhou, China

(Ding) Department of Tuberculosis, Guangzhou Chest Hospital, State Key Laboratory of Respiratory Disease, Guangzhou, China

(Zhang) Tuberculosis Center for Diagnosis and Treatment, Shanghai Pulmonary Hospital, Tongji University School of Medicine, Shanghai, China

(Tong) Department of Tuberculosis, Guangzhou Chest Hospital, State Key Laboratory of Respiratory Disease, Guangzhou, China

(Huang) Department of Respiratory Medicine, The First Affiliated Hospital of Soochow University, Suzhou, China

Emtree Heading

adolescent; adult; aged; China; clinical trial; epidemiology; human; *microbiology; middle aged; *mortality; multicenter study; *Mycobacterium tuberculosis; retrospective study; sputum; Tuberculosis, Multidrug-Resistant/dt [Drug Therapy]; very elderly.

Link to the Ovid Full Text or citation:

Click here for full text options

30.

The double burden.

Faurholt-Jepsen D.

Danish medical journal. 60 (7) (pp B4673), 2013. Date of Publication: 01 Jul 2013.

AN: 23809978

One third of the world's population is latently infected with Mycobacterium tuberculosis, and with the lifestyle changes succeeding the on-going urbanization, populations already burdened by tuberculosis are experiencing a dramatic increase in chronic diseases, with diabetes being a serious challenge. Tuberculosis and diabetes are not only becoming co-existing diseases. In fact, the diseases interact, and there is evidence to suggest that especially diabetes disease increases the susceptibility for developing active tuberculosis disease. Furthermore, it is plausible that tuberculosis leads to, either transient or permanent, impairment of the glucose metabolism, which ultimately will turn into diabetes. A number of studies from the Americas, Europe, Asia, and, most lately, from sub-Saharan Africa have reported strong association between tuberculosis and diabetes; on average, the estimated risk of active tuberculosis is thrice as high among people with diabetes. The study from sub-Saharan Africa was conducted in Tanzania and is the basis of this thesis. Based on available evidence on the association between tuberculosis and diabetes, the primary aim of the study was to assess the role of diabetes for tuberculosis risk, manifestations, treatment outcomes and survival in a Tanzanian population of tuberculosis patients and non-tuberculosis neighbourhood controls. The study was conducted in Mwanza City in northern Tanzania, with a population exceeding half a million inhabitants, with tuberculosis and HIV being common infections in the region, but with little knowledge about the prevalence of diabetes. We recruited newly diagnosed pulmonary tuberculosis patients from spring 2006 and continuously till the fall 2009, with all participating in a nutritional intervention running in parallel with the medical tuberculosis treatment. All participants underwent diabetes and HIV testing as well as a series of measurements such as anthropometric, clinical and paraclinical parameters. The population was followed up during treatment (2 and 5 months) to assess treatment outcome as well as after one year to assess their survival status. Based on data from 1,250 tuberculosis patients and 350 neighbourhood controls, we found that 38 and 21%, respectively, had impaired glycaemia, and that the prevalence of diabetes was 17 and 9% among tuberculosis patients and controls, respectively. This difference in prevalence between patients and controls was equivalent to an adjusted odds ratio of more than four, indicating a strong association between tuberculosis and diabetes. Furthermore, we found that diabetes was associated with tuberculosis among both participants with or without HIV co-infection. Despite the strong association, diabetes had only moderate clinical implications when the tuberculosis patients initiated the tuberculosis treatment; the patients with diabetes co-morbidity had a minor elevation in the immune response and more frequently reported to have fever. Furthermore, diabetes did not seem to delay time to sputum conversion during treatment. Nevertheless, diabetes co-morbidity led to impaired treatment outcome with slower recovery of weight and haemoglobin and a more than four times higher mortality rate within the initial phase of tuberculosis treatment. In conclusion, in the African region, the double burden of tuberculosis and diabetes is becoming a major health problem. Although the tuberculosis incidence has stabilized during the last decade, the increasing incidence of diabetes will possibly interfere with tuberculosis control and may, consequently, make the tuberculosis incidence increase again. Future research strategies should focus on enhanced diagnostic tools to identify tuberculosis patients with diabetes co-morbidity, and on the role of disease-disease, drug-disease and drug-drug interactions between tuberculosis and diabetes diseases and treatments.

Institution

(Faurholt-Jepsen) Department of Human Nutrition, University of Copenhagen, Rolighedsvej 30, 1958 Frederiksberg C, Denmark

Emtree Heading

adolescent; adult; aged; case control study; clinical trial; complication; cross-sectional study; female; follow up; human; Human immunodeficiency virus infection; male; middle aged; mortality; non insulin dependent diabetes mellitus; prospective study; risk factor; statistical model; survival; Tanzania; treatment outcome; Tuberculosis, Pulmonary/di [Diagnosis]; Tuberculosis, Pulmonary/dt [Drug Therapy]; very elderly; young adult; tuberculostatic agent/dt [Drug Therapy].

Link to the Ovid Full Text or citation:

Click here for full text options

31.

Impact of diabetes on treatment outcomes and long-term survival in multidrug-resistant tuberculosis.

Kang Y.A., Kim S.Y., Jo K.-W., Kim H.J., Park S.-K., Kim T.-H., Kim E.K., Lee K.M., Lee S.S., Park J.S., Koh W.-J., Kim D.Y., Shim T.S.

Respiration. 86 (6) (pp 472-478), 2013. Date of Publication: 2013.

AN: 2015947462

Background: Few studies have investigated the impact of diabetes mellitus (DM), a globally increasing metabolic disease, on treatment outcomes and long-term survival in patients with multidrug-resistant forms of tuberculosis (MDR-TB). Objectives: We analyzed outcomes in a large cohort to assess the impact of DM on treatment outcomes of patients with MDR-TB. Methods: MDR-TB patients newly diagnosed or retreated between 2000 and 2002 and followed for 8-11 years were retrospectively analyzed with respect to the effect of DM as a comorbidity on their treatment outcome and long-term survival. Results: Of 1,407 patients with MDR-TB, 239 (17.0%) had coexisting DM. The mean age and body mass index were higher in MDR-TB patients with DM [MDR-TBDM(+)] than in those without DM [MDR-TBDM(-)]. Patients with MDR-TB and a comorbidity of DM had a significantly lower treatment success rate than those without a history of DM (36.0 vs. 47.2%, p = 0.002). In addition, DM was the negative predictor for MDR-TB treatment success in multivariate analyses [odds ratio 0.51, 95% confidence interval (CI) 0.26-0.99]. Mean survival times were also lower in MDR-TBDM(+) than in MDR-TBDM(-) patients (102 vs. 114 months, p = 0.001), with DM as a significant predictor of poor long-term survival in multivariate analyses (hazard ratio 1.59, 95% CI 1.01-2.50). Conclusions: Among MDR-TB patients, DM was a relatively common comorbidity. In patients undergoing treatment for MDR-TB and followed for 8-11 years, it was found to be independently associated with an increased risk of both treatment failure and death.

Institution

(Kang, Kim) Department of Internal Medicine, Yonsei University College of Medicine, Asan Medical Center, Seoul, South Korea (Jo, Shim) Division of Pulmonary and Critical Care Medicine, University of Ulsan College of Medicine, Asan Medical Center, 388-1 Pungnap-dong, Songpa-gu, Seoul, South Korea

(Kim) Department of Epidemiology, Korean Institute of Tuberculosis, Sungkyunkwan University School of Medicine, Seoul, South Korea

(Kim) Department of Internal Medicine, Hanyang University College of Medicine, Sungkyunkwan University School of Medicine, Seoul, South Korea

(Koh) Department of Medicine, Samsung Medical Center, Sungkyunkwan University School of Medicine, Seoul, South Korea

(Park) Clinical Research Center, National Masan Hospital, Masan, South Korea

(Kim) Department of Internal Medicine, College of Medicine, Pochon CHA University, Seongnam, South Korea

(Lee) Department of Internal Medicine, Chungbuk National University College of Medicine, Chungbuk National University Hospital, Cheongju, South Korea

(Lee) Department of Internal Medicine, Inje University School of Medicine, Ilsan Paik Hospital, Goyang, South Korea

(Park) Department of Internal Medicine, Dankook University College of Medicine, Dankook University Hospital, Cheonan, South Korea

(Kim) Department of Thoracic Surgery, National Mokpo Hospital, Mokpo, South Korea

Publisher

S. Karger AG

Emtree Heading

acid fast bacterium; adult; aged; article; bacterium culture; body mass; chronic kidney disease; chronic lung disease; cohort analysis; comorbidity; connective tissue disease; controlled study; *diabetes mellitus; diabetic patient; drug sensitivity; extensively drug resistant tuberculosis; female; human; hyperthyroidism; hypothyroidism; Korean (people); long term survival; major clinical study; male; malnutrition; mental disease; *multidrug resistant tuberculosis; outcome assessment; postgastrectomy syndrome; priority journal; randomized controlled trial; retrospective study; short course therapy; survival time; thorax radiography; treatment duration.

Link to the Ovid Full Text or citation:

Click here for full text options

32.

Targeting of memory T cells with alefacept in new-onset type 1 diabetes (T1DAL study): 12 month results of a randomised, double-blind, placebo-controlled phase 2 trial.

Rigby M.R., DiMeglio L.A., Rendell M.S., Felner E.I., Dostou J.M., Gitelman S.E., Patel C.M., Griffin K.J., Tsalikian E., Gottlieb P.A., Greenbaum C.J., Sherry N.A., Moore W.V., Monzavi R., Willi S.M., Raskin P., Moran A., Russell W.E., Pinckney A., Keyes-Elstein L., Howell M., Aggarwal S., Lim N., Phippard D., Nepom G.T., McNamara J., Ehlers M.R.

The Lancet Diabetes and Endocrinology. 1 (4) (pp 284-294), 2013. Date of Publication: December 2013.

AN: 2013721484

Background: Type 1 diabetes results from autoimmune targeting of the pancreatic beta cells, likely mediated by effector memory T (Tem) cells. CD2, a T cell surface protein highly expressed on Tem cells, is targeted by the fusion protein alefacept, depleting Tem cells and central memory T (Tcm) cells. We postulated that alefacept would arrest autoimmunity and preserve residual beta cells in patients newly diagnosed with type 1 diabetes. Methods: The T1DAL study is a phase 2, double-blind, placebo-controlled trial in patients with type 1 diabetes, aged 12-35 years who, within 100 days of diagnosis, were enrolled at 14 US sites. Patients were randomly assigned (2:1) to receive alefacept (two 12-week courses of 15 mg intramuscularly per week, separated by a 12-week pause) or a placebo. Randomisation was stratified by site, and was computer-generated with permuted blocks of three patients per block. All participants and site personnel were masked to treatment assignment. The primary endpoint was the change from baseline in mean 2 h C-peptide area under the curve (AUC) at 12 months. Secondary endpoints at 12 months were the change from baseline in the 4 h C-peptide AUC, insulin use, major hypoglycaemic events, and HbA1c concentrations. This trial is registered with ClinicalTrials.gov, number NCT00965458. Findings: Of 73 patients assessed for eligibility, 33 were randomly assigned to receive alefacept and 16 to receive placebo. The mean 2 h C-peptide AUC at 12 months increased by 0.015 nmol/L (95% CI -0.080 to 0.110) in the alefacept group and decreased by 0.115 nmol/L (-0.278 to 0.047) in the placebo group, and the difference between groups was not significant (p=0.065). However, key secondary endpoints were met: the mean 4 h C-peptide AUC was significantly higher (mean increase of 0.015 nmol/L [95% CI -0.076 to 0.106] vs decrease of -0.156 nmol/L [-0.305 to -0.006]; p=0.019), and daily insulin use (0.48 units per kg per day for placebo vs 0.36 units per kg per day for alefacept; p=0.02) and the rate of hypoglycaemic events (mean of 10.9 events per person per year for alefacept vs 17.3 events for placebo; p<0.0001) was significantly lower at 12 months in the alefacept group than in the placebo group. Mean HbA1c concentrations at week 52 were not different between treatment groups (p=0.75). So far, no serious adverse events were reported and all patients had at least one adverse event. In the alefacept group, 29 (88%) participants had an adverse event related to study drug versus 15 (94%) participants in the placebo group. In the alefacept group, 14 (42%) participants had grade 3 or 4 adverse events compared with nine (56%) participants in the placebo group; no deaths occurred. Interpretation: Although the primary outcome was not met, at 12 months, alefacept preserved the 4 h C-peptide AUC, lowered insulin use, and reduced hypoglycaemic events, suggesting efficacy. Safety and tolerability were similar in the alefacept and placebo groups. Alefacept could be useful to preserve beta-cell function in patients with new-onset type 1 diabetes. Funding: US National Institutes of Health and the Juvenile Diabetes Research Foundation. © 2013 Elsevier Ltd.

Institution

(Rigby, DiMeglio) Indiana University, Riley Hospital for Children at Indiana University Health, Indianapolis, IN, United States (Rendell) Creighton Diabetes Center, Omaha, NE, United States

(Felner) Emory University, Atlanta, GA, United States

(Dostou) University of North Carolina, Durham, NC, United States

(Gitelman) University of California, San Francisco, CA, United States

(Patel, Griffin) University of Arizona, Tucson, AZ, United States

(Tsalikian) University of Iowa, Iowa City, Iowa, United States

(Gottlieb) Barbara Davis Center, University of Colorado, Aurora, CO, United States

(Greenbaum, Nepom) Benaroya Research Institute, Seattle, WA, United States

(Sherry) Massachusetts General Hospital, Boston, MA, United States

(Moore) Children's Mercy Hospital, Kansas City, MO, United States

(Monzavi) Children's Hospital Los Angeles, Los Angeles, CA, United States

(Willi) Children's Hospital of Philadelphia, Philadelphia, PA, United States

(Raskin) University of Texas, Southwestern Medical Center, Dallas, TX, United States

(Moran) University of Minnesota, Minneapolis, MN, United States

(Russell) Vanderbilt University Medical Center, Nashville, Tennessee, United States

(Pinckney, Keyes-Elstein) Rho Federal Systems Division, Chapel Hill, NC, United States

(Howell, Aggarwal, Lim, Phippard) Immune Tolerance Network, Bethesda, MD, United States

(McNamara) National Institutes of Allergy and Infectious Diseases, Bethesda, MD, United States

(Ehlers) Immune Tolerance Network, San Francisco, CA, United States

Publisher

Lancet Publishing Group (Langford Lane, Kidlington, Oxford OX5 1GB, United Kingdom)

Emtree Heading

adolescent; adult; allergic reaction/si [Side Effect]; area under the curve; article; autoimmunity; cell function; child; clinical article; controlled study; cytomegalovirus infection/si [Side Effect]; double blind procedure; *drug targeting; drug use; effector cell; Epstein Barr virus infection/si [Side Effect]; female; hemoglobin blood level; human; hypoglycemia/si [Side Effect]; *immunotherapy; injection site reaction/si [Side Effect]; *insulin dependent diabetes mellitus/dt [Drug Therapy]; insulin synthesis; insulin treatment; liver injury/si [Side Effect]; lymphocytopenia/si [Side Effect]; male; *memory T lymphocyte; multicenter study; outcome assessment; pancreas islet beta cell; phase 2 clinical trial; priority journal; protein blood level; randomized controlled trial; school child; treatment duration; tuberculosis/si [Side Effect]; *alefacept/ae [Adverse Drug Reaction]; *alefacept/ct [Clinical Trial]; *alefacept/dt [Drug Therapy]; *alefacept/im [Intramuscular Drug Administration]; C peptide/ec [Endogenous Compound]; hemoglobin A1c/ec [Endogenous Compound]; insulin/dt [Drug Therapy].

Link to the Ovid Full Text or citation:

Click here for full text options

33.

Adalimumab for the treatment of psoriasis in real life: A retrospective cohort of 119 patients at a single Spanish centre.

Lopez-Ferrer A., Vilarrasa E., Gich I.J., Puig L.

British Journal of Dermatology. 169 (5) (pp 1141-1147), 2013. Date of Publication: November 2013.

AN: 2013696878

Background Patients with moderate-to-severe psoriasis treated with adalimumab in daily clinical practice are different from those in clinical trials, and outcomes may differ in different geographical settings. Objectives To analyse the efficacy, retention of treatment and adverse events in a cohort of such patients at a referral centre in Barcelona, Spain. Methods Data from a cohort of 119 consecutive patients treated between January 2008 and March 2013 were retrospectively collected. Drug survival was analysed by the Kaplan-Meier method with log-rank test and Cox regression. Results The mean duration of treatment was 25 months (median 22, range 2-60). The 75% improvement in Psoriasis Area and Severity Index (PASI 75) response rates at 16 weeks, 6 months and 1 year of treatment were 64%, 58% and 53%, respectively (intention-to-treat analysis). The corresponding PASI 90 values were 49%, 52% and 50%. Biologic-naive patients (41%) had significantly higher PASI 75 and PASI 90 response rates at 6 months and 1 year. On multivariate analysis, only PASI 90 response at 6 months was significantly associated with treatment retention (P = 0.0009), with a hazard ratio of 7.3 (95% confidence interval 2.3-23.6). Forty-eight adverse events (AEs) occurred in 29 patients, and were serious in eight (0.032 events per patient-year). Paradoxical flares of psoriasis or arthritis were seen in five patients. Infections accounted for seven serious AEs, and were the reason for discontinuation in two patients. Conclusions PASI 90 response at 6 months was the only independent variable predicting drug survival on multivariate analysis. Infections, including de novo infection by Mycobacterium tuberculosis, accounted for seven serious AEs. What's already known about this topic? There are few reports on the use of adalimumab for the treatment of moderate-to-severe psoriasis in clinical practice according to the European Medicines Agency. Psoriasis Area and Severity Index (PASI) 75% response rates at 16 weeks and 6 months were approximately 60% in a previously published U.K. series. Male sex and the presence of arthritis have been associated with decreased drug survival in one study. What does this study add? Biologic-naive status and efficacy parameters denoting a good or excellent response appear to be associated with a higher probability of drug survival. Combination treatment increased PASI response rates at 6 months and might provide an explanation for the relatively high rate of PASI 90 responders in our cohort. Infections, including de novo infection by Mycobacterium tuberculosis, accounted for most serious adverse events, and paradoxical flares of psoriasis and psoriatic arthritis were relatively frequent. © 2013 British Association of Dermatologists.

Institution

(Lopez-Ferrer, Vilarrasa, Puig) Department of Dermatology, Hospital de la Santa Creu i Sant Pau, Universitat Autonoma de Barcelona, Sant Antoni Maria Claret 167, 08025 Barcelona, Catalonia, Spain (Gich) Department of Clinical Epidemiology and Public Health, Hospital de la Santa Creu i Sant Pau, Universitat Autonoma de Barcelona, Sant Antoni Maria Claret 167, 08025 Barcelona, Catalonia, Spain

Publisher

Blackwell Publishing Ltd (9600 Garsington Road, Oxford OX4 2XG, United Kingdom)

Emtree Heading

abscess/si [Side Effect]; adult; aged; alcoholism; article; bronchitis/si [Side Effect]; cohort analysis; comparative study; controlled study; diabetes mellitus; disease course; drug efficacy; drug safety; drug withdrawal; dyslipidemia; female; furunculosis/si [Side Effect]; gastroenteritis/si [Side Effect]; heart infarction/si [Side Effect]; herpes simplex/si [Side Effect]; human; hyperglycemia/si [Side Effect]; hypertension/si [Side Effect]; hypertriglyceridemia/si [Side Effect]; injection site reaction/si [Side Effect]; intention to treat analysis; latent tuberculosis; leukopenia/si [Side Effect]; major clinical study; male; medical record review; miliary tuberculosis/si [Side Effect]; muscle cramp/si [Side Effect]; nonalcoholic fatty liver; obesity; open study; paronychia/si [Side Effect]; patient monitoring; patient referral; pneumonia/si [Side Effect]; priority journal; prostatitis/si [Side Effect]; pruritus/si [Side Effect]; *psoriasis/dt [Drug Therapy]; Psoriasis Area and Severity Index; psoriasis vulgaris; psoriatic arthritis/di [Diagnosis]; psoriatic arthritis/dt [Drug Therapy]; retrospective study; scabies/si [Side Effect]; Spain; survival; thrombocytopenia/si [Side Effect]; thrush/si [Side Effect]; treatment duration; treatment outcome; treatment response; tuberculosis/dt [Drug Therapy]; urinary tract infection/si [Side Effect]; vasculitis/si [Side Effect]; *adalimumab/ae [Adverse Drug Reaction]; *adalimumab/ct [Clinical Trial]; *adalimumab/cb [Drug Combination]; *adalimumab/dt [Drug Therapy]; *adalimumab/pd [Pharmacology]; cyclosporin A/cb [Drug Combination]; cyclosporin A/dt [Drug Therapy]; etretin/cb [Drug Combination]; etretin/dt [Drug Therapy]; infliximab/dt [Drug Therapy]; leflunomide/dt [Drug Therapy]; methotrexate/cb [Drug Combination]; methotrexate/dt [Drug Therapy]; tuberculostatic agent/dt [Drug Therapy].

Link to the Ovid Full Text or citation:

Click here for full text options

34.

DiaPep277 and immune intervention for treatment of type 1 diabetes.

Schloot N.C., Cohen I.R.

Clinical Immunology. 149 (3) (pp 307-316), 2013. Date of Publication: December 2013.

AN: 2013694793

Type 1 diabetes is a chronic immune-mediated disease resulting in destruction of insulin-producing beta-cells. Several studies have been performed aiming to halt disease progression after diagnosis; to reduce the increased diabetes risk in islet-autoantibody positive subjects; and to prevent the onset of beta-cell autoimmunity in subjects genetically at risk but without autoantibodies. Whereas secondary prevention trials failed, trials in newly diagnosed patients have shown partial success in preserving C-peptide. These studies target T-cells and inflammation and make use of antigen-specific immune modulation or stem cell approaches. However, thus far no immune-based therapeutic regimen has cured type 1 diabetes after its clinical onset or has stabilized the decline of C-peptide to achieve the status of an approved drug. This review summarizes immune intervention trials and the current knowledge of DiaPep277 peptide as a form of immune intervention in type 1 diabetes. © 2013 Elsevier Inc.

Institution

(Schloot) Institute for Clinical Diabetology, German Diabetes Center, Leibniz-Center for Diabetes Research, Heinrich-Heine University, Dusseldorf, Germany (Cohen) Department of Immunology, Weizmann Institute of Science, Herzl 234, Rehovot 76100, Israel

Publisher

Academic Press Inc. (1250 Sixth Avenue, San Diego, California CA 92101, United States)

Emtree Heading

adaptive immunity; antiinflammatory activity; area under the curve; autoimmunity; B lymphocyte; blood sampling; CD8+ T lymphocyte; cell destruction; cell function; cell loss; cellular immunity; cytokine production; cytokine release; cytokine response; diabetes control; diabetic patient; down regulation; drug effect; drug efficacy; drug megadose; drug safety; drug tolerability; enzyme linked immunospot assay; Epstein Barr virus infection/si [Side Effect]; first degree relative; flu like syndrome/si [Side Effect]; follow up; genotype; glycemic control; gold standard; HLA system; human; hyperglycemia; hypoglycemia; immune status; immunocompetent cell; immunoreactivity; immunosuppressive treatment; in vitro study; *insulin dependent diabetes mellitus/di [Diagnosis]; *insulin dependent diabetes mellitus/dt [Drug Therapy]; *insulin dependent diabetes mellitus/et [Etiology]; *insulin dependent diabetes mellitus/pc [Prevention]; insulin release; insulin treatment; intervention study; long term care; lymphocyte proliferation; meta analysis; multicenter study (topic); Mycobacterium tuberculosis; non insulin dependent diabetes mellitus/dt [Drug Therapy]; nonhuman; observational study; outcome assessment; pancreas islet cell; phase 2 clinical trial (topic); phase 3 clinical trial (topic); prevention study; priority journal; randomized controlled trial (topic); rash/si [Side Effect]; rehabilitation; repeated drug dose; review; rheumatoid arthritis/dt [Drug Therapy]; secondary prevention; test meal; therapy effect; treatment duration; vital sign; *antiinflammatory agent/dt [Drug Therapy]; *antiinflammatory agent/pd [Pharmacology]; *antiinflammatory agent/sc [Subcutaneous Drug Administration]; atorvastatin/dt [Drug Therapy]; C peptide/ec [Endogenous Compound]; C reactive protein/ec [Endogenous Compound]; chaperonin 60; diapep 277; *diapep277/dt [Drug Therapy]; *diapep277/pd [Pharmacology]; *diapep277/sc [Subcutaneous Drug Administration]; etanercept/dt [Drug Therapy]; gamma interferon/ec [Endogenous Compound]; glutamate decarboxylase/sc [Subcutaneous Drug Administration]; hemoglobin A1c; insulin/dt [Drug Therapy]; insulin lispro; interleukin 1; interleukin 10/ec [Endogenous Compound]; interleukin 13/ec [Endogenous Compound]; interleukin 4/ec [Endogenous Compound]; interleukin 5/ec [Endogenous Compound]; mannitol; otelixizumab/ae [Adverse Drug Reaction]; otelixizumab/iv [Intravenous Drug Administration]; *peptide/dt [Drug Therapy]; *peptide/pd [Pharmacology]; *peptide/sc [Subcutaneous Drug Administration]; placebo; proinsulin/im [Intramuscular Drug Administration]; recombinant alpha interferon/dt [Drug Therapy]; recombinant alpha interferon/po [Oral Drug Administration]; recombinant interleukin 1 receptor blocking agent/dt [Drug Therapy]; recombinant interleukin 1 receptor blocking agent/pd [Pharmacology]; rituximab; streptozocin; teplizumab; tetanus toxoid; tumor necrosis factor alpha; unclassified drug.

Drug Trade Names and Manufacturers

diapep 277, kineret

Link to the Ovid Full Text or citation:

Click here for full text options

35.

An official American thoracic society/European respiratory society statement: Key concepts and advances in pulmonary rehabilitation.

Spruit M.A., Singh S.J., Garvey C., Zu Wallack R., Nici L., Rochester C., Hill K., Holland A.E., Lareau S.C., Man W.D.-C., Pitta F., Sewell L., Raskin J., Bourbeau J., Crouch R., Franssen F.M.E., Casaburi R., Vercoulen J.H., Vogiatzis I., Gosselink R., Clini E.M., Effing T.W., Maltais F., Van Der Palen J., Troosters T., Janssen D.J.A., Collins E., Garcia-Aymerich J., Brooks D., Fahy B.F., Puhan M.A., Hoogendoorn M., Garrod R., Schols A.M.W.J., Carlin B., Benzo R., Meek P., Morgan M., Rutten-Van Molken M.P.M.H., Ries A.L., Make B., Goldstein R.S., Dowson C.A., Brozek J.L., Donner C.F., Wouters E.F.M.

American Journal of Respiratory and Critical Care Medicine. 188 (8) (pp e13-e64), 2013. Date of Publication: 15 Oct 2013.

AN: 2013677207

Background: Pulmonary rehabilitation is recognized as a core component of themanagement of individuals with chronic respiratory disease. Since the 2006 American Thoracic Society (ATS)/European Respiratory Society (ERS) Statement on Pulmonary Rehabilitation, there has been considerable growth in our knowledge of its efficacy and scope. Purpose: The purpose of this Statement is to update the 2006 document, including a new definition of pulmonary rehabilitation and highlighting key concepts and major advances in the field. Methods: A multidisciplinary committee of experts representing the ATS Pulmonary Rehabilitation Assembly and the ERS Scientific Group 01.02, "Rehabilitation and Chronic Care," determined the overall scope of this update through group consensus. Focused literature reviews in key topic areas were conducted by committee members with relevant clinicaland scientific expertise.The final content of this Statement was agreed on by all members. Results: An updated definition of pulmonary rehabilitation is proposed. New data are presented on the science and application of pulmonary rehabilitation, including its effectiveness in acutely ill individuals with chronic obstructive pulmonary disease, and in individuals with other chronic respiratory diseases.The important role of pulmonary rehabilitation in chronic disease management is highlighted. In addition, the role of health behavior change in optimizing and maintaining benefits is discussed. Conclusions: The considerable growth in the science and application of pulmonary rehabilitation since 2006 adds further support for its efficacy in a wide range of individuals with chronic respiratory disease. Copyright © 2013 by the American Thoracic Society.

Publisher

American Thoracic Society (61 Broadway 4th Floor, New York NY 10006 - 2755, United States)

Emtree Heading

acute respiratory failure/rh [Rehabilitation]; acute respiratory failure/th [Therapy]; ADL disability; adult respiratory distress syndrome/rh [Rehabilitation]; adult respiratory distress syndrome/th [Therapy]; allergic pneumonitis/rh [Rehabilitation]; allergic pneumonitis/th [Therapy]; anemia; ankylosing spondylitis/rh [Rehabilitation]; ankylosing spondylitis/th [Therapy]; anxiety disorder; article; asthma/rh [Rehabilitation]; asthma/th [Therapy]; behavior change; bicycle ergometry; bronchiectasis/rh [Rehabilitation]; bronchiectasis/th [Therapy]; bronchiolitis obliterans/rh [Rehabilitation]; bronchiolitis obliterans/th [Therapy]; cardiopulmonary exercise test; cardiovascular disease/th [Therapy]; cardiovascular mortality; Charlson Comorbidity Index; chronic obstructive lung disease/dt [Drug Therapy]; chronic obstructive lung disease/rt [Radiotherapy]; chronic obstructive lung disease/rh [Rehabilitation]; Chronic Respiratory Questionnaire; *chronic respiratory tract disease/dm [Disease Management]; *chronic respiratory tract disease/dt [Drug Therapy]; *chronic respiratory tract disease/rh [Rehabilitation]; cognitive defect; comorbidity; congestive heart failure/rh [Rehabilitation]; congestive heart failure/th [Therapy]; connective tissue disease/rh [Rehabilitation]; connective tissue disease/th [Therapy]; coronary artery disease; cost effectiveness analysis; cystic fibrosis/rh [Rehabilitation]; cystic fibrosis/th [Therapy]; depression; diabetes mellitus; dysphagia; dyspnea; endurance training; fatigue; fibrosing alveolitis/rh [Rehabilitation]; fibrosing alveolitis/th [Therapy]; functional status; gastroesophageal reflux; health care cost; health care utilization; heart arrhythmia; heart rehabilitation; human; hyperlipidemia; hypertension; hypoxemia; infection; inspiratory capacity; interstitial lung disease/rh [Rehabilitation]; interstitial lung disease/th [Therapy]; ischemic heart disease; kidney failure; kyphoscoliosis/rh [Rehabilitation]; kyphoscoliosis/th [Therapy]; lung cancer; lung resection; lung sarcoidosis/rh [Rehabilitation]; lung sarcoidosis/th [Therapy]; lung transplantation; lung vascular resistance; lung ventilation; lymphangiomyoma/rh [Rehabilitation]; lymphangiomyoma/th [Therapy]; muscle exercise; musculoskeletal disease/th [Therapy]; neuromuscular electrical stimulation; nutritional deficiency; obesity/th [Therapy]; osteoarthritis; osteoporosis; outcome assessment; oxygen therapy; patient assessment; patient compliance; patient referral; pedometer; pedometry; peripheral vascular disease/th [Therapy]; physical activity; priority journal; psychosocial disorder; pulmonary hypertension/rh [Rehabilitation]; pulmonary hypertension/th [Therapy]; *pulmonary rehabilitation; quality adjusted life year; quality of life; randomized controlled trial (topic); resistance training; self concept; Short Form 12; sleep disordered breathing; St. George Respiratory Questionnaire; systematic review; telehealth; telemonitoring; thorax wall defect/rh [Rehabilitation]; thorax wall defect/th [Therapy]; treadmill exercise; treatment indication; tuberculosis/rh [Rehabilitation]; tuberculosis/th [Therapy]; bronchodilating agent/dt [Drug Therapy].

Link to the Ovid Full Text or citation:

Click here for full text options

36.

Treatment optimization: An outline for future success.

Flexner C., Plumley B., Brown Ripin D.H.

Current Opinion in HIV and AIDS. 8 (6) (pp 523-527), 2013. Date of Publication: November 2013.

AN: 2013669462

PURPOSE OF REVIEW: In this issue of Current Opinion, the Guest Editors and their colleagues provide a comprehensive overview of current activities aimed at optimizing global HIV treatment. In this introduction, we outline current goals and approaches that will be described in more detail elsewhere in this issue. RECENT FINDINGS: Two recent conferences, the first and second Conference on Antiretroviral Drug Optimization (CADO), brought together experts from academia, governments, foundations, the pharmaceutical industry, and community activists to develop a global HIV-treatment research agenda for the coming decade focused on better therapies and how to make them accessible to a broader population of people living with HIV. Important recommendations included a focus on more efficient process chemistry for antiretroviral drugs, investigation of antiretroviral dose reduction as a possible optimization strategy, recognition of the increasing importance of concurrent infections and comorbidities especially tuberculosis and aging-related diseases, and identifying a highly effective and affordable nontoxic, once-daily fixed-dose combination regimen for first-line treatment. SUMMARY: HIV treatment optimization is a process intended to enhance the long-term efficacy, adherence, tolerability, safety, convenience, and affordability of combination ART. The ultimate goal of this process is to expand access to well tolerated and effective lifetime treatment to all those in need. © 2013 Wolters Kluwer Health.

Institution

(Flexner) Johns Hopkins University School of Medicine and Bloomberg School of Public Health, Osler 527, 600 N, Wolfe Street, Baltimore, MD 21287-5554, United States (Plumley) Pangaea Global AIDS Foundation, Oakland, CA, United States

(Brown Ripin) Clinton Health Access Initiative, Boston, MA, United States

Publisher

Lippincott Williams and Wilkins (250 Waterloo Road, London SE1 8RD, United Kingdom)

Emtree Heading

absence of side effects/si [Side Effect]; *antiviral therapy; cardiovascular disease; comorbidity; concurrent infection; diabetes mellitus; drug bioavailability; drug cost; drug efficacy; drug safety; drug tolerability; drug treatment failure; drug withdrawal; hepatitis B/si [Side Effect]; human; Human immunodeficiency virus; *Human immunodeficiency virus infection/dt [Drug Therapy]; *Human immunodeficiency virus infection/pc [Prevention]; hypertension; low drug dose; medication compliance; mixed infection; patient compliance; phase 3 clinical trial (topic); priority journal; recurrent infection/si [Side Effect]; review; risk reduction; tuberculosis/dt [Drug Therapy]; unspecified side effect/si [Side Effect]; *anti human immunodeficiency virus agent/dt [Drug Therapy]; *anti human immunodeficiency virus agent/pe [Pharmacoeconomics]; cobicistat/cb [Drug Combination]; cobicistat/dt [Drug Therapy]; darunavir/cb [Drug Combination]; darunavir/cm [Drug Comparison]; darunavir/dt [Drug Therapy]; dolutegravir/ct [Clinical Trial]; dolutegravir/cb [Drug Combination]; dolutegravir/dt [Drug Therapy]; efavirenz/cb [Drug Combination]; efavirenz/dt [Drug Therapy]; emtricitabine/ae [Adverse Drug Reaction]; emtricitabine/cb [Drug Combination]; emtricitabine/dt [Drug Therapy]; Human immunodeficiency virus proteinase inhibitor/cm [Drug Comparison]; Human immunodeficiency virus proteinase inhibitor/dt [Drug Therapy]; lamivudine/ae [Adverse Drug Reaction]; lamivudine/cb [Drug Combination]; lamivudine/dt [Drug Therapy]; raltegravir/ae [Adverse Drug Reaction]; raltegravir/dt [Drug Therapy]; rifampicin/dt [Drug Therapy]; ritonavir/cb [Drug Combination]; ritonavir/dt [Drug Therapy]; tenofovir/cb [Drug Combination]; tenofovir/dt [Drug Therapy]; tenofovir/po [Oral Drug Administration]; tenofovir alafenamide/ct [Clinical Trial]; tenofovir alafenamide/cb [Drug Combination]; tenofovir alafenamide/dt [Drug Therapy]; tenofovir disoproxil/dt [Drug Therapy].

Link to the Ovid Full Text or citation:

Click here for full text options

37.

Pediatric uveitis: New and future treatments.

Mehta P.J., Alexander J.L., Sen H.N.

Current Opinion in Ophthalmology. 24 (5) (pp 453-462), 2013. Date of Publication: September 2013.

AN: 2013550461

PURPOSE OF REVIEW: Pediatric uveitis is relatively uncommon, accounting for only 5-10% of all patients with uveitis. However, owing to high prevalence of complications and devastating outcomes, its lifetime burden can be significant. RECENT FINDINGS: Immunomodulatory therapy has been associated with better outcomes in noninfectious pediatric uveitis. However, effective treatments are limited by medication-related complications, including multiorgan toxicities and systemic side effects. SUMMARY: We review the current therapies available to treat pediatric uveitis, discuss novel and future therapies, and provide clinical recommendations utilizing these new agents. The consideration for treatment regimens in noninfectious pediatric uveitis is multifactorial. Understanding past, present, and future technology will aid in treatment of a complex and refractory disease. © 2013 Wolters Kluwer Health Lippincott Williams & Wilkins.

Institution

(Mehta) National Eye Institute, National Institutes of Health, Bethesda, MD, United States (Alexander) Children's National Medical Center, Bethesda, MD, United States

(Sen) Uveitis and Ocular Immunology Fellowship Program, National Eye Institute, National Institutes of Health, 10 Center Drive, Bethesda, MD 20892, United States

Publisher

Lippincott Williams and Wilkins (530 Walnut Street,P O Box 327, Philadelphia PA 19106-3621, United States)

Emtree Heading

acne/si [Side Effect]; allergic reaction/si [Side Effect]; alopecia/si [Side Effect]; arthralgia/si [Side Effect]; bone marrow suppression/si [Side Effect]; cataract/si [Side Effect]; clinical trial; demyelinating disease/si [Side Effect]; diabetes mellitus/si [Side Effect]; diarrhea/si [Side Effect]; fever/si [Side Effect]; gastrointestinal symptom/si [Side Effect]; gum hyperplasia,/si [Side Effect]; hair loss/si [Side Effect]; heart failure/si [Side Effect]; hemorrhagic cystitis/si [Side Effect]; hepatitis/si [Side Effect]; hirsutism/si [Side Effect]; human; hypercholesterolemia/si [Side Effect]; hypertension/si [Side Effect]; hypertriglyceridemia/si [Side Effect]; immunomodulation; immunosuppressive treatment; infection/si [Side Effect]; infusion related reaction/si [Side Effect]; injection site reaction/si [Side Effect]; intraocular pressure; kidney function; liver toxicity/si [Side Effect]; low drug dose; lymphoma/si [Side Effect]; lymphoproliferative disease/si [Side Effect]; multiple organ failure/co [Complication]; nausea/si [Side Effect]; nephrotoxicity/si [Side Effect]; neurotoxicity/si [Side Effect]; neutropenia/si [Side Effect]; nonhuman; osteoporosis/si [Side Effect]; outcome assessment; pediatrics; peripheral edema/si [Side Effect]; priority journal; recommended drug dose; review; side effect/si [Side Effect]; skin manifestation/si [Side Effect]; stomatitis/si [Side Effect]; stunting/si [Side Effect]; thrombocytopenia/si [Side Effect]; tremor/si [Side Effect]; tuberculosis/si [Side Effect]; *uveitis/dt [Drug Therapy]; *uveitis/su [Surgery]; weight gain; abatacept/ae [Adverse Drug Reaction]; abatacept/ct [Clinical Trial]; abatacept/dt [Drug Therapy]; abatacept/pd [Pharmacology]; adalimumab/ae [Adverse Drug Reaction]; adalimumab/ct [Clinical Trial]; adalimumab/dt [Drug Therapy]; adalimumab/pd [Pharmacology]; azathioprine/ae [Adverse Drug Reaction]; azathioprine/ct [Clinical Trial]; azathioprine/dt [Drug Therapy]; azathioprine/pd [Pharmacology]; canakinumab/ae [Adverse Drug Reaction]; canakinumab/ct [Clinical Trial]; canakinumab/dt [Drug Therapy]; canakinumab/pd [Pharmacology]; certolizumab pegol/ct [Clinical Trial]; certolizumab pegol/dt [Drug Therapy]; certolizumab pegol/pd [Pharmacology]; chlorambucil/ae [Adverse Drug Reaction]; chlorambucil/ct [Clinical Trial]; chlorambucil/dt [Drug Therapy]; chlorambucil/pd [Pharmacology]; cyclophosphamide/ae [Adverse Drug Reaction]; cyclophosphamide/ct [Clinical Trial]; cyclophosphamide/dt [Drug Therapy]; cyclophosphamide/pd [Pharmacology]; cyclosporin A/ae [Adverse Drug Reaction]; cyclosporin A/ct [Clinical Trial]; cyclosporin A/do [Drug Dose]; cyclosporin A/dt [Drug Therapy]; cyclosporin A/pd [Pharmacology]; daclizumab/ae [Adverse Drug Reaction]; daclizumab/ct [Clinical Trial]; daclizumab/dt [Drug Therapy]; daclizumab/pd [Pharmacology]; dexamethasone/ae [Adverse Drug Reaction]; dexamethasone/ct [Clinical Trial]; dexamethasone/dt [Drug Therapy]; dexamethasone/pd [Pharmacology]; etanercept/ct [Clinical Trial]; etanercept/dt [Drug Therapy]; etanercept/pd [Pharmacology]; fluocinolone acetonide/ae [Adverse Drug Reaction]; fluocinolone acetonide/ct [Clinical Trial]; fluocinolone acetonide/dt [Drug Therapy]; fluocinolone acetonide/po [Oral Drug Administration]; fluocinolone acetonide/pd [Pharmacology]; golimumab/ct [Clinical Trial]; golimumab/dt [Drug Therapy]; golimumab/pd [Pharmacology]; infliximab/ae [Adverse Drug Reaction]; infliximab/ct [Clinical Trial]; infliximab/dt [Drug Therapy]; infliximab/iv [Intravenous Drug Administration]; infliximab/pd [Pharmacology]; methotrexate/ae [Adverse Drug Reaction]; methotrexate/ct [Clinical Trial]; methotrexate/dt [Drug Therapy]; methotrexate/pd [Pharmacology]; mycophenolic acid 2 morpholinoethyl ester/ae [Adverse Drug Reaction]; mycophenolic acid 2 morpholinoethyl ester/ct [Clinical Trial]; mycophenolic acid 2 morpholinoethyl ester/dt [Drug Therapy]; mycophenolic acid 2 morpholinoethyl ester/pd [Pharmacology]; prednisone/ae [Adverse Drug Reaction]; prednisone/ct [Clinical Trial]; prednisone/dt [Drug Therapy]; prednisone/pd [Pharmacology]; rapamycin/ae [Adverse Drug Reaction]; rapamycin/ct [Clinical Trial]; rapamycin/dt [Drug Therapy]; rapamycin/pd [Pharmacology]; recombinant interleukin 1 receptor blocking agent/ae [Adverse Drug Reaction]; recombinant interleukin 1 receptor blocking agent/ct [Clinical Trial]; recombinant interleukin 1 receptor blocking agent/dt [Drug Therapy]; recombinant interleukin 1 receptor blocking agent/pd [Pharmacology]; rituximab/ae [Adverse Drug Reaction]; rituximab/ct [Clinical Trial]; rituximab/dt [Drug Therapy]; rituximab/pd [Pharmacology]; tacrolimus/ae [Adverse Drug Reaction]; tacrolimus/ct [Clinical Trial]; tacrolimus/dt [Drug Therapy]; tacrolimus/pd [Pharmacology]; tocilizumab/ae [Adverse Drug Reaction]; tocilizumab/ct [Clinical Trial]; tocilizumab/dt [Drug Therapy]; tocilizumab/pd [Pharmacology]; voclosporin/ae [Adverse Drug Reaction]; voclosporin/ct [Clinical Trial]; voclosporin/dt [Drug Therapy]; voclosporin/pd [Pharmacology].

Drug Trade Names and Manufacturers

Abbott [United States], Allergan [United States], Amgen [United States], Astellas [United States], Bristol Myers Squibb, Dava [United States], Genentech, Glaxo SmithKline, Hoffmann La Roche [United States], Novartis, Teva [United States], actemra: Genentech, cellcept, cimzia, cytoxan: Bristol Myers Squibb, deltasone, enbrel, fk 506, gengraf: Abbott, humira: Abbott [United States], ilaris: Novartis, imuran, kineret: Amgen [United States], leukeran: Glaxo SmithKline, neoral: Novartis, orencia: Bristol Myers Squibb, ozurdex: Allergan [United States], prograf: Astellas [United States], rapamune, remicade, retisert, rheumatrex: Dava [United States], rituxan: Genentech, sandimmune: Novartis, simponi, trexall: Teva [United States], zenapax: Hoffmann La Roche [United States]

Link to the Ovid Full Text or citation:

Click here for full text options

38.

Acupuncture for allergic rhinitis: Active and sham acupuncture both seem to work.

Lim C.E.D., Cheng N.C.L.

Focus on Alternative and Complementary Therapies. 18 (3) (pp 136-137), 2013. Date of Publication: September 2013.

AN: 2013505008

Institution

(Lim, Cheng) SWS Clinical School, University of New South Wales, Sydney, Australia

Publisher

Wiley-Blackwell (350 Main Street, Malden MA 02148, United States)

Emtree Heading

*acupuncture; *allergic rhinitis/th [Therapy]; controlled study; human; hypertension; insulin dependent diabetes mellitus; kidney dysfunction; lung tuberculosis; major clinical study; multicenter study; nose obstruction; note; outcome assessment; perennial rhinitis; questionnaire; randomized controlled trial; rhinitis; rhinorrhea; sham procedure; skin allergy; sneezing; teratoma.

Link to the Ovid Full Text or citation:

Click here for full text options

39.

Beyond pregnancy - The neglected burden of mortality in young women of reproductive age in Bangladesh: A prospective cohort study.

Labrique A.B., Sikder S.S., Wu L., Rashid M., Ali H., Ullah B., Shamim A.A., Mehra S., Klemm R., Banu H., West Jr. K.P., Christian P.

BJOG: An International Journal of Obstetrics and Gynaecology. 120 (9) (pp 1085-1089), 2013. Date of Publication: August 2013.

AN: 2013441665

Objective To describe proportionate mortality and causes of death unrelated to pregnancy. Design Prospective cohort study. Setting Rural northwest Bangladesh. Population A cohort of 133 617 married women of reproductive age. Methods Verbal autopsies were conducted for women who died whilst under surveillance in the cohort trial. Physician-assigned causes of death based on verbal autopsies were used to categorise deaths. Main outcome measures The proportion of deaths due to non-communicable diseases, infectious diseases, injury or pregnancy. Results Of the 1107 deaths occurring among women between 2001 and 2007, 48% were attributed to non-communicable diseases, 22% to pregnancy, 17% to infections, 9% to injury and 4% to other causes. Conclusions Although focus on pregnancy-related mortality remains important, more attention is warranted on non-communicable diseases among women of reproductive age. © 2013 The Authors BJOG An International Journal of Obstetrics and Gynaecology © 2013 RCOG.

Institution

(Labrique, Sikder, Wu, Mehra, Klemm, West Jr., Christian) Department of International Health, Johns Hopkins Bloomberg School of Public Health, 615 N. Wolfe St., Baltimore, MD 21205, United States (Rashid, Ali, Shamim) JiVitA Maternal and Child Health Research Project Gaibandha, Poschim Para, Bangladesh

(Ullah) Maternal and Neonatal Health Initiative, United Nations Population Fund Narail, Aladatpur, Bangladesh

(Banu) Department of Gynecology, Institute of Child and Mother Health Matuail, Dhaka, Bangladesh

Publisher

Blackwell Publishing Ltd (9600 Garsington Road, Oxford OX4 2XG, United Kingdom)

Emtree Heading

adult; anaphylactic shock; anemia; anorexia nervosa; asthma; autopsy; Bangladesh; bleeding; cause of death; cerebrovascular accident; cohort analysis; dehydration; diabetes mellitus; diarrhea; diphtheria; epilepsy; female; gastrointestinal disease; heart failure; heart infarction; homicide; human; infection; injury; ischemic heart disease; kidney disease; lung embolism; major clinical study; malaria; malnutrition; married woman; meningitis; *mortality; neoplasm; non communicable disease; outcome assessment; pericardial effusion; *pregnancy; priority journal; prospective study; rabies; respiratory tract infection; review; rheumatic disease; sepsis; suicide; tetanus; tonsillitis; tuberculosis; typhoid fever.

Link to the Ovid Full Text or citation:

Click here for full text options

40.

Immunotherapeutic treatment of autoimmune diabetes.

Kim J.H., Jin S.-M., Kim H.S., Kim K.-A., Lee M.-S.

Critical Reviews in Immunology. 33 (3) (pp 245-281), 2013. Date of Publication: 2013.

AN: 2013362619

Type 1 diabetes is a prototypic, organ-specific autoimmune disease. Diverse antigen-specific immunotherapy using insulin or glutamic acid decarboxylase peptides and other immunotherapies, such as antibodies, fusion proteins, cytokines, regulatory T cells, small-molecule inhibitors, nonspecific immune modulators, or dietary modifications, have been attempted in human type 1 diabetes. Some of these immunotherapies delay the onset of diabetes or reduce insulin requirements or blood glucose level in patients with established type 1 diabetes. However, most of these immunotherapies failed to induce complete remission of established type 1 diabetes, which could be due to 1) technical difficulties in the achievement of immune tolerance to diabetic autoantigens or in the inhibition of autoimmune responses to those antigens that can be applied to human patients without significant adverse effects, and 2) markedly reduced b-cell mass at the time of disease onset that should be replenished. This review focuses on the immunological aspects of the disease and its treatment, and data from previous or ongoing human clinical trials using immune-logical measures, and recent results from immunological studies employing animal models are discussed. © 2013 by Begell House, Inc.

Institution

(Kim, Jin, Lee) Department of Medicine, Samsung Medical Center, Sungkyunkwan University School of Medicine, Kyunggi-do 410-773, South Korea (Kim) Graduate School, University of Ulsan, Kyunggi-do 410-773, South Korea

(Kim) Department of Medicine, Dongguk University International Hospital, Dongguk University School of Medicine, Kyunggi-do 410-773, South Korea

Publisher

Begell House Inc. (50 Cross Highway, Redding CT 06886, United States)

Emtree Heading

antiinflammatory activity; article; autologous hematopoietic stem cell transplantation; blood glucose monitoring; cytokine production; cytokine release syndrome/si [Side Effect]; diet supplementation; diet therapy; drug effect; drug efficacy; drug safety; Epstein Barr virus infection/si [Side Effect]; fever/si [Side Effect]; headache/si [Side Effect]; human; immune response; immunological tolerance; immunomodulation; *immunotherapy; infection/si [Side Effect]; *insulin dependent diabetes mellitus/dt [Drug Therapy]; *insulin dependent diabetes mellitus/pc [Prevention]; *insulin dependent diabetes mellitus/th [Therapy]; insulin release; low drug dose; lymphocytopenia/si [Side Effect]; mesenchymal stem cell; mesenchymal stem cell transplantation; myalgia/si [Side Effect]; nonhuman; opportunistic infection/si [Side Effect]; pancreas islet beta cell; phase 2 clinical trial (topic); phase 3 clinical trial (topic); priority journal; randomized controlled trial (topic); rash/si [Side Effect]; regulatory T lymphocyte; serum sickness/si [Side Effect]; treatment outcome; treatment response; tuberculosis/si [Side Effect]; abatacept/ct [Clinical Trial]; abatacept/dt [Drug Therapy]; abatacept/iv [Intravenous Drug Administration]; abatacept/pd [Pharmacology]; alpha 1 antitrypsin/dt [Drug Therapy]; alpha 1 antitrypsin/pd [Pharmacology]; BCG vaccine/dt [Drug Therapy]; bht 3021/ct [Clinical Trial]; bht 3021/dt [Drug Therapy]; canakinumab/ct [Clinical Trial]; canakinumab/dt [Drug Therapy]; diamyd; diapep277/ct [Clinical Trial]; diapep277/dt [Drug Therapy]; dipeptidyl peptidase IV inhibitor/dt [Drug Therapy]; DNA vaccine/ct [Clinical Trial]; DNA vaccine/dt [Drug Therapy]; etanercept/ae [Adverse Drug Reaction]; etanercept/ct [Clinical Trial]; etanercept/dt [Drug Therapy]; etanercept/pd [Pharmacology]; glucagon like peptide 1 receptor agonist/dt [Drug Therapy]; glutamate decarboxylase 65/ct [Clinical Trial]; glutamate decarboxylase 65/dt [Drug Therapy]; glutamate decarboxylase 65/sc [Subcutaneous Drug Administration]; granulocyte colony stimulating factor/ct [Clinical Trial]; granulocyte colony stimulating factor/cb [Drug Combination]; granulocyte colony stimulating factor/dt [Drug Therapy]; immunosuppressive agent/dt [Drug Therapy]; insulin/ct [Clinical Trial]; insulin/dt [Drug Therapy]; insulin/na [Intranasal Drug Administration]; insulin/po [Oral Drug Administration]; interleukin 2/ct [Clinical Trial]; interleukin 2/cb [Drug Combination]; interleukin 2/dt [Drug Therapy]; lansoprazole/ct [Clinical Trial]; lansoprazole/cb [Drug Combination]; lansoprazole/dt [Drug Therapy]; nbi 6024/ct [Clinical Trial]; nbi 6024/dt [Drug Therapy]; nicotinamide/ct [Clinical Trial]; nicotinamide/dt [Drug Therapy]; otelixizumab/ae [Adverse Drug Reaction]; otelixizumab/ct [Clinical Trial]; otelixizumab/dt [Drug Therapy]; otelixizumab/pd [Pharmacology]; placebo; proinsulin/dt [Drug Therapy]; proinsulin/ip [Intraperitoneal Drug Administration]; rapamycin/ct [Clinical Trial]; rapamycin/cb [Drug Combination]; rapamycin/dt [Drug Therapy]; recombinant alpha interferon/ct [Clinical Trial]; recombinant alpha interferon/dt [Drug Therapy]; recombinant alpha interferon/po [Oral Drug Administration]; recombinant alpha interferon/pd [Pharmacology]; recombinant interleukin 1 receptor blocking agent/ct [Clinical Trial]; recombinant interleukin 1 receptor blocking agent/dt [Drug Therapy]; rituximab/ct [Clinical Trial]; rituximab/dt [Drug Therapy]; rituximab/iv [Intravenous Drug Administration]; rituximab/pd [Pharmacology]; sitagliptin/ct [Clinical Trial]; sitagliptin/cb [Drug Combination]; sitagliptin/dt [Drug Therapy]; synthetic peptide/ct [Clinical Trial]; synthetic peptide/dt [Drug Therapy]; teplizumab/ae [Adverse Drug Reaction]; teplizumab/ct [Clinical Trial]; teplizumab/dt [Drug Therapy]; teplizumab/pd [Pharmacology]; thymocyte antibody/ae [Adverse Drug Reaction]; thymocyte antibody/ct [Clinical Trial]; thymocyte antibody/cb [Drug Combination]; thymocyte antibody/dt [Drug Therapy]; unclassified drug; unindexed drug.

Drug Trade Names and Manufacturers

aralast, bht 3021, diamyd, diapep277, nbi 6024

Link to the Ovid Full Text or citation:

Click here for full text options

41.

Oral paricalcitol reduces the prevalence of posttransplant hyperparathyroidism: Results of an open label randomized trial.

Amer H., Griffin M.D., Stegall M.D., Cosio F.G., Park W.D., Kremers W.K., Heilman R.L., Mazur M.J., Hamawi K., Larson T.S., Kumar R.

American Journal of Transplantation. 13 (6) (pp 1576-1585), 2013. Date of Publication: June 2013.

AN: 2013348987

Postkidney transplant hyperparathyroidism is a significant problem. Vitamin D receptor agonists are known to suppress parathyroid hormone (PTH) secretion. We examined the effect of oral paricalcitol on posttransplant secondary hyperparathyroidism by conducting an open label randomized trial in which 100 incident kidney transplant recipients were randomized 1:1 to receive oral paricalcitol, 2 mug per day, for the first year posttransplant or no additional therapy. Serial measurements of serum PTH, calcium and bone alkaline phosphatase, 24-h urine calcium and bone density were performed. The primary endpoint was the frequency of hyperparathyroidism 1-year posttransplant. Eighty-seven patients completed the trial. One-year posttransplant, 29% of paricalcitol-treated subjects had hyperparathyroidism compared with 63% of untreated patients (p = 0.0005). Calcium supplementation was discontinued in two control and 15 treatment patients due to mild hypercalcemia or hypercalcuria. Paricalcitol was discontinued in four patients due to hypercalcuria/ hypercalcemia and in one for preference. Two subjects required decreasing the dose of paricalcitol to 1 mug daily. Hypercalcemia was asymptomatic and reversible. Incidence of acute rejection, BK nephropathy and renal function at 1 year were similar between groups. Moderate renal allograft fibrosis was reduced in treated patients. Oral paricalcitol is effective in decreasing posttransplant hyperparathyroidism and may have beneficial effects on renal allograft histology. Paricalcitol decreases the prevalence of post-kidney transplant hyperparathyroidism and may have beneficial effects on renal allograft histology. © 2013 The American Society of Transplantation and the American Society of Transplant Surgeons.

Institution

(Amer, Cosio, Larson, Kumar) Division of Nephrology and Hypertension, Department of Medicine, Mayo Clinic, Rochester, MN, United States (Griffin) Regenerative Medicine Institute, National University of Ireland, Galway, Ireland

(Stegall) Division of Transplant Surgery, Department of Surgery, Mayo Clinic, Rochester, MN, United States

(Park, Kremers) Department of Health Sciences Research, Mayo Clinic, Rochester, MN, United States

(Heilman, Mazur, Hamawi) Department of Transplantation, Mayo Clinic, Scottsdale, AZ, United States

Publisher

Blackwell Publishing Ltd (9600 Garsington Road, Oxford OX4 2XG, United Kingdom)

Emtree Heading

acute graft rejection/co [Complication]; adult; afibrinogenemia/si [Side Effect]; article; bacteremia/si [Side Effect]; bone density; breast cancer/si [Side Effect]; bronchitis/si [Side Effect]; calcium excretion; cellulitis/si [Side Effect]; Clostridium difficile infection/si [Side Effect]; controlled study; creatinine clearance; cytomegalovirus infection/si [Side Effect]; depression/si [Side Effect]; diabetes mellitus/si [Side Effect]; diarrhea/si [Side Effect]; drug dose increase; drug dose reduction; drug efficacy; drug safety; drug withdrawal; falling; female; fever/si [Side Effect]; fracture/si [Side Effect]; gastroenteritis/si [Side Effect]; glomerulus filtration rate; gout/si [Side Effect]; graft recipient; headache/si [Side Effect]; heart infarction/si [Side Effect]; herpes zoster/si [Side Effect]; histopathology; human; human tissue; hypercalcemia/si [Side Effect]; hypercalciuria/si [Side Effect]; hyperkalemia/si [Side Effect]; hypomagnesemia/si [Side Effect]; immunosuppressive treatment; kidney disease/dt [Drug Therapy]; kidney disease/si [Side Effect]; kidney function; kidney transplantation; leg pain/si [Side Effect]; liver cirrhosis/si [Side Effect]; major clinical study; malaise/si [Side Effect]; male; micturition; nausea and vomiting/si [Side Effect]; neutropenia/si [Side Effect]; paranoia/si [Side Effect]; parathyroidectomy; pneumonia/si [Side Effect]; polyomavirus nephropathy/dt [Drug Therapy]; polyomavirus nephropathy/si [Side Effect]; posttransplant diabetes mellitus/si [Side Effect]; *posttransplant hyperparathyroidism/co [Complication]; *posttransplant hyperparathyroidism/dt [Drug Therapy]; *posttransplant hyperparathyroidism/su [Surgery]; prevalence; priority journal; pyelonephritis/si [Side Effect]; radicular pain/si [Side Effect]; randomized controlled trial; *secondary hyperparathyroidism/co [Complication]; *secondary hyperparathyroidism/dt [Drug Therapy]; *secondary hyperparathyroidism/su [Surgery]; seizure/si [Side Effect]; side effect/si [Side Effect]; single drug dose; sinusitis/si [Side Effect]; standing; treatment outcome; tuberculosis/si [Side Effect]; upper respiratory tract infection/si [Side Effect]; ureter obstruction/si [Side Effect]; urinary excretion; urinary tract infection/si [Side Effect]; urolithiasis/si [Side Effect]; wound infection/si [Side Effect]; alemtuzumab; alkaline phosphatase bone isoenzyme/ec [Endogenous Compound]; calcium/ec [Endogenous Compound]; calcium carbonate; cidofovir/dt [Drug Therapy]; methylprednisolone; mycophenolic acid 2 morpholinoethyl ester; parathyroid hormone/ec [Endogenous Compound]; *paricalcitol/ae [Adverse Drug Reaction]; *paricalcitol/ct [Clinical Trial]; *paricalcitol/do [Drug Dose]; *paricalcitol/dt [Drug Therapy]; *paricalcitol/po [Oral Drug Administration]; tacrolimus.

Link to the Ovid Full Text or citation:

Click here for full text options

42.

The biological and clinical activity of anti-malarial drugs in autoimmune disorders.

Taherian E., Rao A., Malemud C.J., Askari A.D.

Current Rheumatology Reviews. 9 (1) (pp 45-62), 2013. Date of Publication: 2013.

AN: 2013358663

Chloroquine and hydroxychloroquine are 4-aminoquinoline compounds commonly employed as anti-malarial drugs. Chloroquine and its synthetic analogue, hydroxychloroquine also belong to the disease-modifying anti-rheumatic drug class because these drugs are immunosuppressive. The immunosuppressive activity of chloroquine and hydroxychloroquine is likely to account for their capacity to reduce T-cell and B-cell hyperactivity as well as pro-inflammatory cytokine gene expression. This review evaluated experimental and clinical trials results as well as clinical response data relative to the use of chloroquine and/or hydroxychloroquine as first-line medical therapies in systemic lupus erythematosus, rheumatoid arthritis, primary Sjogren's syndrome, the anti-phospholipid syndrome and in the treatment of sarcoidosis. A primary outcomes measure in these clinical trials was the extent to which chloroquine and/or hydroxychloroquine reduced disease progression or exacerbations and/or the use and dosage of corticosteroids. The relative efficacy of chloroquine and hydroxychloroquine in modifying the clinical course of these autoimmune disorders is balanced against evidence that these drugs induce adverse effects which may reduce their use and effectiveness in the therapy of autoimmune disorders. © 2013 Bentham Science Publishers.

Institution

(Taherian, Rao, Malemud, Askari) Department of Medicine, Division of Rheumatic Diseases, Case Western Reserve University School of Medicine and University Hospitals Case Medical Center, Cleveland, OH 44106, United States (Malemud) Department of Anatomy, Case Western Reserve University School of Medicine, University Hospitals Case Medical Center, Cleveland, OH 44106, United States

Publisher

Bentham Science Publishers B.V. (P.O. Box 294, Bussum 1400 AG, Netherlands)

Emtree Heading

anticoagulation; antigen antibody complex; *antimalarial activity; *antiphospholipid syndrome; Apgar score; arterial stiffness; artery thrombosis/co [Complication]; artery thrombosis/pc [Prevention]; arthralgia; atherosclerosis; atomic force microscopy; autologous mixed lymphocyte reaction; birth weight; body mass; bone density; bone erosion; burning sensation; cardiomyopathy; cardiovascular risk; cell surface; cholesterol blood level; clinical practice; cohort analysis; coronary artery disease; cross-sectional study; deep vein thrombosis/pc [Prevention]; diabetes mellitus; disease activity; disease course; disease exacerbation; drug dose titration; drug efficacy; dyslipidemia; echography; ex vivo study; fat intake; follow up; glucose tolerance; heart left ventricle filling pressure; heart muscle biopsy; human; hypercalcemia; hypertension; in vitro study; in vivo study; insulin release; insulin sensitivity; joint function; kidney failure; kidney injury; lean body weight; lung embolism/pc [Prevention]; lung function; lung infiltrate; lung lesion; lupus erythematosus nephritis/dt [Drug Therapy]; lupus erythematosus nephritis/pc [Prevention]; lymphadenopathy; morning stiffness; mouth mucosa; myalgia; Mycobacterium tuberculosis; myopathy; North America; open study; outcome assessment; overall survival; phospholipid bilayer; pigment epithelium; pneumonia; postmenopause; postoperative period; pregnancy; pregnancy outcome; priority journal; propensity score; prospective study; pulse wave; randomized controlled trial (topic); remission; retinopathy/dt [Drug Therapy]; retrospective study; review; *rheumatoid arthritis/dt [Drug Therapy]; rheumatology; risk factor; sarcoidosis/dt [Drug Therapy]; sarcoidosis/et [Etiology]; *Sjoegren syndrome; skin defect; skin pigmentation; SLEDAI; *systemic lupus erythematosus/di [Diagnosis]; *systemic lupus erythematosus/dt [Drug Therapy]; *systemic lupus erythematosus/et [Etiology]; thorax radiography; thrombocyte aggregation; tissue injury; transthoracic echocardiography; world health organization; xerostomia; azathioprine; CD8 antigen/ec [Endogenous Compound]; chlorambucil; *chloroquine/dt [Drug Therapy]; cholesterol/ec [Endogenous Compound]; colchicine; cyclosporin; *hydroxychloroquine/cb [Drug Combination]; *hydroxychloroquine/dt [Drug Therapy]; *hydroxychloroquine/po [Oral Drug Administration]; interleukin 1/ec [Endogenous Compound]; interleukin 15/ec [Endogenous Compound]; interleukin 18/ec [Endogenous Compound]; interleukin 2/ec [Endogenous Compound]; interleukin 6/ec [Endogenous Compound]; interleukin 8/ec [Endogenous Compound]; leflunomide; lipocortin 5; low density lipoprotein/ec [Endogenous Compound]; methotrexate/cb [Drug Combination]; mycophenolic acid 2 morpholinoethyl ester/dt [Drug Therapy]; placebo; prednisone; quinoprox; salazosulfapyridine/cb [Drug Combination]; triacylglycerol/ec [Endogenous Compound]; tumor necrosis factor alpha/ec [Endogenous Compound]; unclassified drug.

Drug Trade Names and Manufacturers

plaquenil, quinoprox

Link to the Ovid Full Text or citation:

Click here for full text options

43.

Rationale and design of a randomized controlled trial of the effect of retinol and vitamin D supplementation on treatment in active pulmonary tuberculosis patients with diabetes.

Wang Q., Ma A., Bygbjerg I.C., Han X., Liu Y., Zhao S., Cai J.

BMC Infectious Diseases. 13 (1) (no pagination), 2013. Article Number: 104. Date of Publication: 26 Feb 2013.

AN: 2013166562

Background: The association between pulmonary tuberculosis (PTB) and diabetes mellitus (DM) has been previously attracted much attention. Diabetes alters immunity to tuberculosis, leading to more frequent treatment failure in TB patients with DM. Moreover, TB and DM often coincide with micronutrients deficiencies, such as retinol and vitamin D, which are especially important to immunity of the body and may influence pancreas beta-cell function. However, the effects of retinol and vitamin D supplementation in active TB patients with diabetes on treatment outcomes, immune and nutrition state are still uncertain. We are conducting a randomized controlled trial of vitamin A and/or D in active PTB patients with DM in a network of 4 TB treatment clinics to determine whether the supplementation could improve the outcome in the patients.Methods/design: This is a 2x2 factorial trial. We plan to enroll 400 active PTB patients with DM, and randomize them to VA (2000 IU daily retinol); VD (400 IU daily cholecalciferol); VAD (2000 IU daily retinol plus 400 IU cholecalciferol) or control (placebo) group. Our primary outcome measure is the efficacy of anti-tuberculosis treatment and ameliorating of glucose metabolism, and the secondary outcome measure being immune and nutrition status of the subjects. Of the first 37 subjects enrolled: 8 have been randomized to VA, 10 to VD, 9 to VAD and 10 to control. To date, the sample is 97.3% Han Chinese and 91.9% female. The average fasting plasma glucose level is 12.19 mmol/L.Discussion: This paper describes the design and rationale of a randomized clinical trial comparing VA and/or VD supplementation to active pulmonary TB patients with DM. Our trial will allow rigorous evaluation of the efficacy of the supplementation to active TB and DM therapy for improving clinical outcomes and immunological condition. This detailed description of trial methodology can serve as a template for the development of future treatment scheme for active TB patient with DM.Trial registration: ChiCTR-TRC-12002546. © 2013 Wang et al; licensee BioMed Central Ltd.

Institution

(Wang, Ma, Cai) The Institute of Human Nutrition, Medical College of Qingdao University, 38 Dengzhou Road, Qingdao 266021, China (Bygbjerg) Department of International Health, University of Copenhagen, Copenhagen DK-1014, Denmark

(Han) Microbiology teaching and research division, Medical College of Qingdao University, 308 Ningxia Road, Qingdao 266021, China

(Liu) Clinical department, Qingdao Chest Hospital, 896 Chongqing Middle Road, Qingdao 266000, China

(Zhao) Clinical department, Lingyi Chest Hospital, East Fenghuang Avenue, Linyi 276000, China

Publisher

BioMed Central Ltd. (Floor 6, 236 Gray's Inn Road, London WC1X 8HB, United Kingdom)

Emtree Heading

adult; anorexia/si [Side Effect]; article; Chinese; controlled study; *diabetes mellitus/dt [Drug Therapy]; drug efficacy; drug fever/si [Side Effect]; drug induced headache/si [Side Effect]; fatigue/si [Side Effect]; female; glucose blood level; glucose metabolism; Han Chinese; human; immune status; insomnia/si [Side Effect]; irritability; *lung tuberculosis/dt [Drug Therapy]; major clinical study; male; nausea/si [Side Effect]; nutritional status; randomized controlled trial; sex ratio; side effect/si [Side Effect]; treatment outcome; *vitamin supplementation; vomiting/si [Side Effect]; *colecalciferol/ae [Adverse Drug Reaction]; *colecalciferol/ct [Clinical Trial]; *colecalciferol/cb [Drug Combination]; *colecalciferol/cm [Drug Comparison]; *colecalciferol/dt [Drug Therapy]; glucose/ec [Endogenous Compound]; placebo; *retinol/ae [Adverse Drug Reaction]; *retinol/ct [Clinical Trial]; *retinol/cb [Drug Combination]; *retinol/cm [Drug Comparison]; *retinol/dt [Drug Therapy].

Link to the Ovid Full Text or citation:

Click here for full text options

44.

Effects of hemodiafiltration on uremic inflammation.

Leurs P., Lindholm B., Stenvinkel P.

Blood Purification. 35 (SUPPL. 1) (pp 11-17), 2013. Date of Publication: February 2013.

AN: 2013148032

Chronic inflammation is highly prevalent among end-stage renal disease patients and is linked to, and thought to contribute to, the high morbidity and mortality in this patient population. Hemodiafiltration (HDF) may potentially reduce inflammatory stimuli induced by the bioincompatibility of conventional hemodialysis (HD) systems. In addition, HDF may more efficiently remove inflammatory mediators. This brief review shows that the circulating levels of various markers of systemic inflammation in general are somewhat reduced in patients treated by HDF as compared to HD. On the other hand, according to the current literature, this favorable small impact on inflammation biomarkers does not seem to translate into better clinical outcomes. It is possible that this is due to inadequate design, inadequate number of investigated patients and too short duration of previous studies. The results of larger, better-designed ongoing prospective studies may hopefully clarify this. Copyright © 2013 S. Karger AG, Basel.

Institution

(Stenvinkel) Divisions of Renal Medicine, K56 Karolinska University Hospital at Huddinge, SE-141 86 Stockholm, Sweden (Leurs, Lindholm) Baxter Novum, Department of Clinical Science, Intervention and Technology, Stockholm, Sweden

Publisher

S. Karger AG (Allschwilerstrasse 10, P.O. Box, Basel CH-4009, Switzerland)

Emtree Heading

biocompatibility; body mass; calcium metabolism; Chlamydophila pneumoniae; chronic kidney disease; conference paper; device infection; diabetes mellitus; dialysate; dialysis membrane; endothelial progenitor cell; epigenetics; heart failure; Helicobacter pylori; hematocrit; *hemodiafiltration; hemodialysis; heredity; human; *inflammation/co [Complication]; *inflammation/et [Etiology]; *inflammation/th [Therapy]; kidney failure/co [Complication]; kidney graft rejection; lean body weight; mononuclear cell; morbidity; mortality; nonhuman; obesity; outcome assessment; oxidative stress; periodontal disease; peritonitis; phosphate metabolism; pneumonia; priority journal; randomized controlled trial (topic); sedentary lifestyle; septicemia; tuberculosis; *uremic inflammation/co [Complication]; *uremic inflammation/et [Etiology]; *uremic inflammation/th [Therapy]; urinary tract infection; vagus nerve stimulation; albumin/ec [Endogenous Compound]; beta 2 microglobulin/ec [Endogenous Compound]; *biological marker/ec [Endogenous Compound]; C reactive protein/ec [Endogenous Compound]; CD14 antigen/ec [Endogenous Compound]; CD16 antigen/ec [Endogenous Compound]; erythropoietin/ec [Endogenous Compound]; ferritin/ec [Endogenous Compound]; heme oxygenase 1/ec [Endogenous Compound]; hemoglobin/ec [Endogenous Compound]; indican/ec [Endogenous Compound]; inducible nitric oxide synthase/ec [Endogenous Compound]; interleukin 1/ec [Endogenous Compound]; interleukin 1 receptor blocking agent/ec [Endogenous Compound]; interleukin 1beta/ec [Endogenous Compound]; interleukin 6/ec [Endogenous Compound]; interleukin 8/ec [Endogenous Compound]; kelch like ECH associated protein 1/ec [Endogenous Compound]; leptin/ec [Endogenous Compound]; oxidized low density lipoprotein/ec [Endogenous Compound]; parathyroid hormone/ec [Endogenous Compound]; pentosidine/ec [Endogenous Compound]; pentraxin 3/ec [Endogenous Compound]; phosphate/ec [Endogenous Compound]; reactive oxygen metabolite/ec [Endogenous Compound]; superoxide dismutase/ec [Endogenous Compound]; transferrin/ec [Endogenous Compound]; tumor necrosis factor/ec [Endogenous Compound]; tumor necrosis factor alpha/ec [Endogenous Compound]; unindexed drug.

Link to the Ovid Full Text or citation:

Click here for full text options

45.

Tofacitinib or adalimumab versus placebo in rheumatoid arthritis.

Van Vollenhoven R.F., Fleischmann R., Cohen S., Lee E.B., Meijide J.A.G., Wagner S., Forejtova S., Zwillich S.H., Gruben D., Koncz T., Wallenstein G.V., Krishnaswami S., Bradley J.D., Wilkinson B.

New England Journal of Medicine. 367 (6) (pp 508-519), 2012. Date of Publication: 09 Aug 2012.

AN: 2012464365

BACKGROUND: Tofacitinib (CP-690,550) is a novel oral Janus kinase inhibitor that is being investigated for the treatment of rheumatoid arthritis. METHODS: In this 12-month, phase 3 trial, 717 patients who were receiving stable doses of methotrexate were randomly assigned to 5 mg of tofacitinib twice daily, 10 mg of tofacitinib twice daily, 40 mg of adalimumab once every 2 weeks, or placebo. At month 3, patients in the placebo group who did not have a 20% reduction from baseline in the number of swollen and tender joints were switched in a blinded fashion to either 5 mg or 10 mg of tofacitinib twice daily; at month 6, all patients still receiving placebo were switched to tofacitinib in a blinded fashion. The three primary outcome measures were a 20% improvement at month 6 in the American College of Rheumatology scale (ACR 20); the change from baseline to month 3 in the score on the Health Assessment Questionnaire-Disability Index (HAQ-DI) (which ranges from 0 to 3, with higher scores indicating greater disability); and the percentage of patients at month 6 who had a Disease Activity Score for 28-joint counts based on the erythrocyte sedimentation rate (DAS28-4[ESR]) of less than 2.6 (with scores ranging from 0 to 9.4 and higher scores indicating greater disease activity). RESULTS: At month 6, ACR 20 response rates were higher among patients receiving 5 mg or 10 mg of tofacitinib (51.5% and 52.6%, respectively) and among those receiving adalimumab (47.2%) than among those receiving placebo (28.3%) (P<0.001 for all comparisons). There were also greater reductions in the HAQ-DI score at month 3 and higher percentages of patients with a DAS28-4(ESR) below 2.6 at month 6 in the active-treatment groups than in the placebo group. Adverse events occurred more frequently with tofacitinib than with placebo, and pulmonary tuberculosis developed in two patients in the 10-mg tofacitinib group. Tofacitinib was associated with an increase in both low-density and high-density lipoprotein cholesterol levels and with reductions in neutrophil counts. CONCLUSIONS: In patients with rheumatoid arthritis receiving background methotrexate, tofacitinib was significantly superior to placebo and was numerically similar to adalimumab in efficacy. (Funded by Pfizer; ORAL Standard ClinicalTrials.gov number, NCT00853385.) Copyright © 2012 Massachusetts Medical Society.

Institution

(Van Vollenhoven) Karolinska Institute, Stockholm, Sweden (Fleischmann, Cohen) Metroplex Clinical Research Center, Dallas, United States

(Lee) Seoul National University, Seoul, South Korea

(Meijide) Hospital Nuestra Senora de la Esperanza, Santiago de Compostela, Spain

(Wagner) Internistische Schwerpunktpraxis fur Rheumatologie, Halle, Germany

(Wagner) Department of Medicine 2, University of Leipzig, Leipzig, Germany

(Forejtova) Revmatologicky Ustav No. 5, Prague, Czech Republic

(Zwillich, Gruben, Wallenstein, Krishnaswami, Bradley, Wilkinson) Pfizer Inc., Bldg. 98, MS 8260-2517, 445 Eastern Point Rd., Groton, CT 06340, United States

(Koncz) Pfizer, New York, NY, United States

Publisher

Massachussetts Medical Society (860 Winter Street, Waltham MA 02451-1413, United States)

Emtree Heading

abdominal wall hernia/si [Side Effect]; acute cholecystitis/si [Side Effect]; acute heart infarction/si [Side Effect]; anus polyp/si [Side Effect]; article; autoimmune thyroiditis/si [Side Effect]; blood disease/si [Side Effect]; bone marrow depression/si [Side Effect]; breast abscess/si [Side Effect]; bursitis/si [Side Effect]; cellulitis/si [Side Effect]; cholecystitis/si [Side Effect]; cholelithiasis/si [Side Effect]; chronic obstructive lung disease/si [Side Effect]; complete heart block/si [Side Effect]; congestive heart failure/si [Side Effect]; connective tissue disease/si [Side Effect]; controlled study; creatinine blood level; deep vein thrombosis/si [Side Effect]; diabetes mellitus/si [Side Effect]; disease activity; disease duration; drug efficacy; drug response; drug safety; ear disease/si [Side Effect]; empyema/si [Side Effect]; endocrine disease/si [Side Effect]; erysipelas/si [Side Effect]; erythrocyte sedimentation rate; eye disease/si [Side Effect]; femur fracture/si [Side Effect]; fever/si [Side Effect]; fibula fracture/si [Side Effect]; gallbladder empyema/si [Side Effect]; gastroenteritis/si [Side Effect]; gastrointestinal disease/si [Side Effect]; genital system disease/si [Side Effect]; Health Assessment Questionnaire; hearing impairment/si [Side Effect]; heart arrest/si [Side Effect]; heart disease/si [Side Effect]; heart muscle ischemia/si [Side Effect]; hematemesis/si [Side Effect]; hepatobiliary disease/si [Side Effect]; herpes zoster/si [Side Effect]; human; humerus fracture/si [Side Effect]; ileus/si [Side Effect]; immune response; immunomodulation; inner ear disease/si [Side Effect]; interstitial lung disease/si [Side Effect]; intestine perforation/si [Side Effect]; joint dislocation/si [Side Effect]; limb fracture/si [Side Effect]; liver disease/si [Side Effect]; lung abscess/si [Side Effect]; lung non small cell cancer/si [Side Effect]; lung sarcoidosis/si [Side Effect]; lymphatic system disease/si [Side Effect]; major clinical study; melena/si [Side Effect]; metabolic disorder/si [Side Effect]; metrorrhagia/si [Side Effect]; musculoskeletal disease/si [Side Effect]; neuroma/si [Side Effect]; neutropenia/si [Side Effect]; nutritional disorder/si [Side Effect]; osteomyelitis/si [Side Effect]; outcome assessment; ovary cyst/si [Side Effect]; ovary inflammation/si [Side Effect]; ovary torsion/si [Side Effect]; peptic ulcer bleeding/si [Side Effect]; phase 3 clinical trial; pneumonia/si [Side Effect]; polyp/si [Side Effect]; priority journal; prurigo/si [Side Effect]; randomized controlled trial; retina detachment/si [Side Effect]; *rheumatoid arthritis/dt [Drug Therapy]; *rheumatoid arthritis/si [Side Effect]; salivary gland calculus/si [Side Effect]; salivary gland disease/si [Side Effect]; septic shock/si [Side Effect]; sialoadenitis/si [Side Effect]; side effect/si [Side Effect]; spondylolisthesis/si [Side Effect]; tendon disease/si [Side Effect]; tendon rupture/si [Side Effect]; thorax pain/si [Side Effect]; tibia fracture/si [Side Effect]; uterine cervix disease/si [Side Effect]; vascular disease/si [Side Effect]; vertigo/si [Side Effect]; *adalimumab/ae [Adverse Drug Reaction]; *adalimumab/dt [Drug Therapy]; methotrexate/dt [Drug Therapy]; placebo; *tofacitinib/ae [Adverse Drug Reaction]; *tofacitinib/dt [Drug Therapy].

Drug Trade Names and Manufacturers

cp 690550

Link to the Ovid Full Text or citation:

Click here for full text options

46.

Belatacept post kidney transplantation.

Bajjoka I., Makowski C., Churchill D., Abouljoud M.

Journal of Pharmacy Technology. 28 (3) (pp 106-114), 2012. Date of Publication: May-June 2012.

AN: 2012459433

Objective: To review the use of belatacept as an alternative to calcineurin inhibitor-based regimens for maintenance immunosuppression in renal transplant recipients. Data Sources: To provide an extensive overview of the pharmacology, pharmacokinetics, efficacy, and safety of belatacept, a MEDLINE/PubMed search (1980-December 2011) was performed for all articles evaluating belatacept's properties and patient outcomes, as well as abstracts from recent meetings, using key words belatacept, pharmacology, efficacy, pharmacokinetics, and safety. Study Selection/Data Extraction: Phase 2 and 3 studies in humans describing use, adverse reactions, pharmacology, pharmacokinetics, efficacy, and safety of belatacept were identified and reviewed. Other articles were identified through PubMed. Data Synthesis: Belatacept, a costimulation blocker, is a biologic recombinant fusion protein that has been shown to prevent acute cellular rejection in kidney transplant recipients and preserve renal function. It was recently approved by the FDA as an antirejection immunosuppressant agent for use in kidney transplant recipients. It is the first biologic agent used for maintenance immunosuppression. It acts as an antagonist to CD80 and CD86 receptors located on the surface of antigen presenting cells, thereby blocking CD28 T-cell activation and, thus, preventing acute rejection. In comparison with patients receiving other current therapies, patients on belatacept have demonstrated superior renal function with comparable outcomes in patient and graft survival. Conclusions: Belatacept has potential for use as an alternative to current maintenance immunosuppression regimens, with potentially fewer adverse effects.

Institution

(Bajjoka, Abouljoud) Henry Ford Transplant Institute, Detroit, MI, United States (Makowski) Eugene Applebaum School of Pharmacy, Wayne State University, Detroit, United States

(Churchill) School of Medicine, Wayne State University, United States

Publisher

Harvey Whitney Books Company (8044 Montgomery Road, Suite 415, Cincinnati OH 45236, United States)

Emtree Heading

acute graft rejection/th [Therapy]; apoptosis; binding affinity; cell proliferation; chronic allograft nephropathy/si [Side Effect]; creatinine blood level; cytokine production; cytomegalovirus infection/si [Side Effect]; diabetes mellitus/si [Side Effect]; disease severity; drug accumulation; drug clearance; drug dose reduction; drug efficacy; drug elimination; drug exposure; drug half life; drug potency; drug safety; dyslipidemia/si [Side Effect]; follow up; food and drug administration; glomerulus filtration rate; graft failure; graft recipient; health care personnel; human; hypertension/si [Side Effect]; immune response; immunosuppressive treatment; in vitro study; infusion related reaction/si [Side Effect]; intensive care; kidney donor; kidney function; kidney graft; *kidney transplantation; long term care; lung mycosis/si [Side Effect]; lymphoproliferative disease/th [Therapy]; malignant neoplastic disease/si [Side Effect]; patient counseling; pharmacokinetics; pharmacology; phase 1 clinical trial (topic); phase 2 clinical trial (topic); phase 3 clinical trial (topic); plasma concentration-time curve; polyomavirus infection/si [Side Effect]; posttransplant lymphoproliferative disease/si [Side Effect]; protein expression; randomized controlled trial (topic); review; side effect/si [Side Effect]; treatment duration; treatment outcome; triacylglycerol blood level; tuberculosis/si [Side Effect]; B7 antigen/ec [Endogenous Compound]; *belatacept/ae [Adverse Drug Reaction]; *belatacept/cb [Drug Combination]; *belatacept/cm [Drug Comparison]; *belatacept/iv [Intravenous Drug Administration]; *belatacept/pk [Pharmacokinetics]; *belatacept/pd [Pharmacology]; calcineurin inhibitor; CD28 antigen/ec [Endogenous Compound]; CD3 antigen/ec [Endogenous Compound]; CD86 antigen/ec [Endogenous Compound]; corticosteroid; creatinine/ec [Endogenous Compound]; *cyclosporin/ae [Adverse Drug Reaction]; *cyclosporin/cm [Drug Comparison]; cytochrome P450 3A4/ec [Endogenous Compound]; cytotoxic T lymphocyte antigen 4/ec [Endogenous Compound]; mycophenolic acid; mycophenolic acid 2 morpholinoethyl ester/cb [Drug Combination]; OKT 3; rapamycin/cb [Drug Combination]; tacrolimus; thymocyte antibody.

Link to the Ovid Full Text or citation:

Click here for full text options

47.

Pulmonary aspergilloma. Clinical aspects and surgical treatment outcome.

Passera E., Rizzi A., Robustellini M., Rossi G., Della Pona C., Massera F., Rocco G.

Thoracic Surgery Clinics. 22 (3) (pp 345-361), 2012. Date of Publication: August 2012.

AN: 2012412920

Aspergillomas are fungal balls within lung cavities. The natural history is variable. Hemoptysis is a dangerous sequela. Medical therapy is ineffective because of the lack of a lesion blood supply. Randomized trials are lacking. Surgery should be the treatment of choice in cases of hemoptysis, and even in asymptomatic patients, if lung function is not severely compromised. Cavernostomy and cavernoplasty may be options for high-risk patients. Percutaneous therapy should be reserved for patients who are not fit for surgery. Bronchial artery embolization is appropriate for symptomatic patients not suitable for surgery. Embolization could be considered a preoperative and temporary strategy. © 2012 Elsevier Inc.

Institution

(Passera, Rizzi) Division of Thoracic Surgery, Humanitas Gavazzeni Hospital, via Gavazzeni, 21, 24123 Bergamo, Italy (Robustellini, Della Pona) Division of Thoracic Surgery, E. Morelli Hospital, via Zubiani, 33, 23039 Sondalo, Italy

(Rossi) Division of Thoracic Surgery, Valduce Hospital, via Alighieri, 11, 22100 Como, Italy

(Massera) Division of Thoracic Surgery, Maggiore della Carita Hospital, Corso Mazzini, 18, 28100 Novara, Italy

(Rocco) Division of Thoracic Surgery, Department of Thoracic Surgery and Oncology, National Cancer Institute, Pascale Foundation, Via Semmola 81, 80131 Naples, Italy

Publisher

W.B. Saunders (Independence Square West, Philadelphia PA 19106-3399, United States)

Emtree Heading

acute leukemia; allergic bronchopulmonary aspergillosis/co [Complication]; allergic pneumonitis/co [Complication]; anatomy; artificial embolism; *aspergilloma/di [Diagnosis]; *aspergilloma/dt [Drug Therapy]; *aspergilloma/ep [Epidemiology]; *aspergilloma/et [Etiology]; *aspergilloma/pc [Prevention]; *aspergilloma/su [Surgery]; *aspergilloma/th [Therapy]; Aspergillus; bacterial infection/co [Complication]; bleeding/co [Complication]; bleeding/pc [Prevention]; bleeding/th [Therapy]; bronchitis/co [Complication]; bronchopleural fistula/co [Complication]; cancer chemotherapy; cancer patient; cancer radiotherapy; cavernoplasty; cavernostomy; chronic inflammation/co [Complication]; chronic obstructive lung disease/co [Complication]; computer assisted tomography; coughing; death; decortication; diabetes mellitus; dyspnea; empyema/co [Complication]; external beam radiotherapy; extrinsic asthma/co [Complication]; fever; graft recipient; hemoptysis; human; immune deficiency/co [Complication]; immunosuppressive treatment; intensive care unit; invasive aspergillosis/co [Complication]; lung cancer/co [Complication]; lung fibrosis/co [Complication]; lung lobectomy; lung resection; lung tuberculosis/di [Diagnosis]; morbidity; mortality; pathophysiology; pleuropneumonectomy; pneumonia/co [Complication]; pneumothorax; postoperative complication/co [Complication]; priority journal; pulmonary aspiration/co [Complication]; respiratory failure/co [Complication]; retrospective study; review; segmentectomy; sputum; thorax pain; thorax surgery; tracheobronchitis/co [Complication]; treatment outcome; video assisted thoracoscopic surgery; wedge resection; weight reduction; acetylcysteine/ct [Clinical Trial]; acetylcysteine/dt [Drug Therapy]; amphotericin B/ct [Clinical Trial]; amphotericin B/dt [Drug Therapy]; corticosteroid/dt [Drug Therapy]; miconazole/ct [Clinical Trial]; miconazole/dt [Drug Therapy]; natamycin/ct [Clinical Trial]; natamycin/dt [Drug Therapy]; nystatin/ct [Clinical Trial]; nystatin/dt [Drug Therapy]; sodium iodide/ct [Clinical Trial]; sodium iodide/dt [Drug Therapy]; tuberculostatic agent/dt [Drug Therapy].

Link to the Ovid Full Text or citation:

Click here for full text options

48.

Clinical trials in rheumatoid arthritis: A status report from the ClinicalTrials.gov website.

Paul J.R., Ranganathan P.

Rheumatology International. 32 (6) (pp 1831-1835), 2012. Date of Publication: June 2012.

AN: 2012414345

The aims of this study are to describe the characteristics of clinical trials in rheumatoid arthritis (RA) listed in ClinicalTrials.gov and examine existing trends in study design, funding sources, outcomes, and drugs under investigation. We conducted a survey of ongoing clinical trials in RA registered in the ClinicalTrials.gov website. We used the advanced search option and applied the following inclusion criteria, "rheumatoid arthritis", "open studies", "interventional", and "adults 18 years or older". Of 127 eligible trials, 53.5% of the studies were either phase 3 or 4, and 40.2% were phase 1, 2, and 2/3. Twothirds of the trials were randomized (70.9%), and over half were, in addition, double-blinded (53.5%) and placebo-controlled (53.5%). Universities were listed as the primary sponsor for 18.9% of the trials and pharmaceutical industry for 73.2%. Majority of the trials were multi-center studies (93%) conducted outside the United States (54.3%). The most frequently used endpoint was drug efficacy (54.3%) followed by drug safety (25.2%). Most industry-funded trials were open for less than 12 months, whereas most university- funded trials were open for more than 24 months (58% each). Biologic therapies were the focus of most trials in the registry (78.5%). Randomized, double-blinded, placebo- controlled, phase 3 and 4 trials form the majority of ongoing clinical trials in RA. The preponderance of industry funding of RA trials and the short duration of such trials are troubling trends which need to be addressed. © Springer-Verlag 2011.

Institution

(Paul) Department of Medicine, St. Luke's Hospital, St. Louis, MO, United States (Ranganathan) Division of Rheumatology, Washington University, School of Medicine, 660 S. Euclid, St Louis, MO 63110, United States

Publisher

Springer Verlag (Tiergartenstrasse 17, Heidelberg D-69121, Germany)

Emtree Heading

article; biological therapy; *clinical trial (topic); diabetes mellitus/dt [Drug Therapy]; disease activity; double blind procedure; drug approval; drug efficacy; drug indication; drug industry; drug safety; drug surveillance program; drug targeting; food and drug administration; government; heart infarction/si [Side Effect]; human; molecularly targeted therapy; multicenter study (topic); open study; outcome assessment; phase 1 clinical trial (topic); phase 2 clinical trial (topic); phase 3 clinical trial (topic); phase 4 clinical trial (topic); priority journal; randomized controlled trial (topic); register; *rheumatoid arthritis/dt [Drug Therapy]; single blind procedure; stroke/si [Side Effect]; treatment response; tuberculosis/si [Side Effect]; university; adenosine receptor blocking agent/ct [Clinical Trial]; adenosine receptor blocking agent/dt [Drug Therapy]; *antirheumatic agent/ct [Clinical Trial]; *antirheumatic agent/dt [Drug Therapy]; *antirheumatic agent/pk [Pharmacokinetics]; *antirheumatic agent/pd [Pharmacology]; biological product/ct [Clinical Trial]; biological product/cb [Drug Combination]; biological product/dt [Drug Therapy]; CD20 antigen; CD28 antigen; CD3 antigen; curcumin/ct [Clinical Trial]; curcumin/dt [Drug Therapy]; *disease modifying antirheumatic drug/ct [Clinical Trial]; *disease modifying antirheumatic drug/dt [Drug Therapy]; etanercept/ae [Adverse Drug Reaction]; etanercept/ct [Clinical Trial]; etanercept/dt [Drug Therapy]; granulocyte macrophage colony stimulating factor; infliximab/ae [Adverse Drug Reaction]; infliximab/ct [Clinical Trial]; infliximab/dt [Drug Therapy]; interleukin 17; *Janus kinase 3 inhibitor/ct [Clinical Trial]; *Janus kinase 3 inhibitor/dt [Drug Therapy]; methotrexate/ct [Clinical Trial]; methotrexate/cb [Drug Combination]; methotrexate/dt [Drug Therapy]; *monoclonal antibody/ct [Clinical Trial]; *monoclonal antibody/dt [Drug Therapy]; *nonsteroid antiinflammatory agent/ct [Clinical Trial]; *nonsteroid antiinflammatory agent/dt [Drug Therapy]; placebo; protein tyrosine kinase inhibitor/ct [Clinical Trial]; protein tyrosine kinase inhibitor/dt [Drug Therapy]; rosiglitazone/ae [Adverse Drug Reaction]; rosiglitazone/dt [Drug Therapy]; *tumor necrosis factor inhibitor/ct [Clinical Trial]; *tumor necrosis factor inhibitor/dt [Drug Therapy]; vitamin D/ct [Clinical Trial]; vitamin D/dt [Drug Therapy].

Link to the Ovid Full Text or citation:

Click here for full text options

49.

All you need to read in the other general journals.

Anonymous

BMJ (Online). 344 (7861) (no pagination), 2012. Article Number: e4055. Date of Publication: 16 Jun 2012.

AN: 2012345872

Publisher

BMJ Publishing Group (Tavistock Square, London WC1H 9JR, United Kingdom)

Emtree Heading

add on therapy; antibiotic resistance; article; bleeding/si [Side Effect]; *brain cancer; brain hemorrhage/si [Side Effect]; cancer incidence; cancer risk; cardiovascular disease/dt [Drug Therapy]; cardiovascular disease/pc [Prevention]; cardiovascular surgery; *cognitive therapy; *computer assisted tomography; depression/th [Therapy]; drug tolerability; drug withdrawal; gastrointestinal hemorrhage/si [Side Effect]; glucose blood level; glycemic control; health care quality; human; hypoglycemia/si [Side Effect]; intermethod comparison; *leukemia; lifestyle modification; low drug dose; medical decision making; multicenter study (topic); *multidrug resistant tuberculosis/dr [Drug Resistance]; nausea/si [Side Effect]; *non insulin dependent diabetes mellitus/dt [Drug Therapy]; *non insulin dependent diabetes mellitus/pc [Prevention]; *non insulin dependent diabetes mellitus/th [Therapy]; open study; pancreatitis/si [Side Effect]; patient compliance; prediction; priority journal; psychotherapy; radiation dose; randomized controlled trial (topic); remission; risk assessment; risk benefit analysis; side effect/si [Side Effect]; surgical mortality; survival rate; *telemedicine; treatment outcome; unnecessary procedure; weight gain; weight reduction; *acetylsalicylic acid/ae [Adverse Drug Reaction]; *acetylsalicylic acid/do [Drug Dose]; *acetylsalicylic acid/dt [Drug Therapy]; bedaquiline; delamanid; ethambutol; *exendin 4/ae [Adverse Drug Reaction]; *exendin 4/ct [Clinical Trial]; *exendin 4/cm [Drug Comparison]; *exendin 4/dt [Drug Therapy]; *glimepiride/ae [Adverse Drug Reaction]; *glimepiride/ct [Clinical Trial]; *glimepiride/cm [Drug Comparison]; *glimepiride/dt [Drug Therapy]; glucose/ec [Endogenous Compound]; hemoglobin A1c/ec [Endogenous Compound]; isoniazid; kanamycin; *metformin/dt [Drug Therapy]; ofloxacin; rifampicin; streptomycin; *troponin T/ec [Endogenous Compound].

Link to the Ovid Full Text or citation:

Click here for full text options

50.

Predictors of mortality in staphylococcus aureus bacteremia.

van Hal S.J., Jensen S.O., Vaska V.L., Espedido B.A., Paterson D.L., Gosbell I.B.

Clinical Microbiology Reviews. 25 (2) (pp 362-386), 2012. Date of Publication: April 2012.

AN: 2012205805

Staphylococcus aureus bacteremia (SAB) is an important infection with an incidence rate ranging from 20 to 50 cases/100,000 population per year. Between 10% and 30% of these patients will die from SAB. Comparatively, this accounts for a greater number of deaths than for AIDS, tuberculosis, and viral hepatitis combined. Multiple factors influence outcomes for SAB patients. The most consistent predictor of mortality is age, with older patients being twice as likely to die. Except for the presence of comorbidities, the impacts of other host factors, including gender, ethnicity, socioeconomic status, and immune status, are unclear. Pathogen-host interactions, especially the presence of shock and the source of SAB, are strong predictors of outcomes. Although antibiotic resistance may be associated with increased mortality, questions remain as to whether this reflects pathogen-specific factors or poorer responses to antibiotic therapy, namely, vancomycin. Optimal management relies on starting appropriate antibiotics in a timely fashion, resulting in improved outcomes for certain patient subgroups. The roles of surgery and infectious disease consultations require further study. Although the rate of mortality from SAB is declining, it remains high. Future international collaborative studies are required to tease out the relative contributions of various factors to mortality, which would enable the optimization of SAB management and patient outcomes. © 2012, American Society for Microbiology. All Rights Reserved.

Institution

(van Hal, Gosbell) Department of Microbiology and Infectious Diseases, Sydney South West Pathology Service-Liverpool, South Western Sydney Local Health Network, Sydney, NSW, Australia (van Hal, Jensen, Espedido, Gosbell) Antibiotic Resistance and Mobile Elements Group, Microbiology and Infectious Diseases Unit, School of Medicine, University of Western Sydney, Sydney, NSW, Australia

(Jensen, Espedido, Gosbell) Ingham Institute of Applied Medical Research, Sydney, NSW, Australia

(Vaska, Paterson) The University of Queensland, UQ Centre for Clinical Research (UQCCR), Herston, QLD, Australia

Publisher

American Society for Microbiology (1752 N Street N.W., Washington DC 20036-2904, United States)

Emtree Heading

acquired immune deficiency syndrome; alcoholism; bacterial endocarditis; bactericidal activity; bone and joint infections/ep [Epidemiology]; Caucasian; cellular immunity; central nervous system infection/ep [Epidemiology]; chronic kidney failure; cohort analysis; confidence interval; congestive heart failure; crowding; device infection/co [Complication]; diabetes mellitus; drug fatality/si [Side Effect]; ethnicity; gender; hematologic malignancy; host pathogen interaction; human; humoral immunity; immune deficiency/cn [Congenital Disorder]; immune status; in vitro study; innate immunity; intensive care unit; liver cirrhosis; meta analysis (topic); methicillin resistant Staphylococcus aureus; methicillin resistant Staphylococcus aureus infection/dt [Drug Therapy]; mortality; multidrug resistance; multivariate analysis; neutropenia; newborn intensive care; penicillin resistance; phagocyte; postoperative complication/co [Complication]; prospective study; prostate cancer; prosthetic valve endocarditis/co [Complication]; prosthetic valve endocarditis/et [Etiology]; randomized controlled trial (topic); retrospective study; review; risk; sepsis; shock; social status; socioeconomics; soft tissue infection/co [Complication]; *staphylococcal bacteremia/dr [Drug Resistance]; *staphylococcal bacteremia/ep [Epidemiology]; *staphylococcal bacteremia/et [Etiology]; Staphylococcus infection/dt [Drug Therapy]; surgical infection/co [Complication]; survival prediction; systematic review (topic); treatment failure; tuberculosis; United States; unspecified side effect/si [Side Effect]; virus hepatitis; cefazolin/dt [Drug Therapy]; cloxacillin/dt [Drug Therapy]; cotrimoxazole/cm [Drug Comparison]; cotrimoxazole/dt [Drug Therapy]; exotoxin; flucloxacillin; gentamicin/ae [Adverse Drug Reaction]; levofloxacin; linezolid/ae [Adverse Drug Reaction]; linezolid/cm [Drug Comparison]; meticillin; rifampicin/pk [Pharmacokinetics]; vancomycin/cm [Drug Comparison]; vancomycin/dt [Drug Therapy].

Link to the Ovid Full Text or citation:

Click here for full text options

51.

A pilot study on area under curve of mycophenolic acid as a guide for its optimal use in renal transplant recipients.

Sarangi S.C., Reeta K.H., Agarwal S.K., Kaleekal T., Guleria S., Gupta Y.K.

Indian Journal of Medical Research. 135 (1) (pp 84-91), 2012. Date of Publication: January 2012.

AN: 2012137350

Background & objectives: The immunosuppressants administered to renal transplant subjects are usually monitored therapeutically to prevent graft rejection and drug toxicity. Mycophenolic acid (MPA) is an immunosuppressant. The present prospective study was undertaken to establish the utility of plasma level monitoring of MPA and to correlate it with clinical outcomes in renal transplant receipients. Methods: MPA plasma level at 2, 4 and 9 h and the area under concentration-time curve (AUC) were estimated using high performance liquid chromatography in 24 renal transplant recipients receiving immunosuppressant MPA plus tacrolimus and steroid. Results: There was wide inter-individual variation in MPA plasma level and the AUC. The incidences of gastrointestinal adverse drug events (diarrhoea and acidity) were significantly more in the high MPA AUC patients. Though biopsy proven acute rejection was not found, of the six subjects with lower MPA AUC (<30 mg.h/l), three were clinically diagnosed to develop tacrolimus nephrotoxicity. The Gastrointestinal Symptom Rating Scale (GSRS) and Gastrointestinal Quality of Life Index (GIQLI) scores represented better health related quality of life in lower MPA AUC than in the higher MPA AUC (>60 mg.h/l). Interpretation & conclusions: The present findings suggest the MPA AUC of 30 - 60 mg.h/l in the maintenance stage of renal transplant patients to have optimum clinical benefit and relegated adverse events profile indicating the usefulness of AUC of MPA with limited sampling strategy in optimizing its use.

Institution

(Sarangi, Reeta, Kaleekal, Gupta) Department of Pharmacology, All India Institute of Medical Sciences, New Delhi, India (Agarwal) Department of Nephrology, All India Institute of Medical Sciences, New Delhi, India

(Guleria) Department of Surgery, All India Institute of Medical Sciences, New Delhi, India

Publisher

Indian Council of Medical Research (Ansari Nagar, P.O. Box 4911, New Delhi 110029, India)

Emtree Heading

adult; anemia/si [Side Effect]; area under the curve; article; blood analysis; cellulitis/si [Side Effect]; chickenpox/si [Side Effect]; controlled clinical trial; controlled study; cytomegalovirus infection/si [Side Effect]; dehydration/si [Side Effect]; diabetes mellitus/si [Side Effect]; diarrhea/si [Side Effect]; drug monitoring; drug safety; drug withdrawal; female; fever/si [Side Effect]; Gastrointestinal Quality of Life Index; giardiasis/si [Side Effect]; graft recipient; hepatitis B/si [Side Effect]; high performance liquid chromatography; human; human tissue; immunosuppressive treatment; kidney biopsy; kidney graft rejection/dt [Drug Therapy]; kidney graft rejection/pc [Prevention]; *kidney transplantation; leukopenia/si [Side Effect]; lower respiratory tract infection/si [Side Effect]; major clinical study; male; nephrotoxicity/si [Side Effect]; otitis media/si [Side Effect]; polyomavirus infection/si [Side Effect]; rating scale; sepsis/si [Side Effect]; septicemia/si [Side Effect]; side effect/si [Side Effect]; stomach pH; thrombocytopenia/si [Side Effect]; treatment outcome; tuberculosis/si [Side Effect]; unspecified side effect/si [Side Effect]; urinary tract infection/si [Side Effect]; vomiting/si [Side Effect]; wound infection/si [Side Effect]; *mycophenolic acid/ae [Adverse Drug Reaction]; *mycophenolic acid/ct [Clinical Trial]; *mycophenolic acid/cb [Drug Combination]; *mycophenolic acid/cr [Drug Concentration]; *mycophenolic acid/dt [Drug Therapy]; *mycophenolic acid/pk [Pharmacokinetics]; steroid/cb [Drug Combination]; steroid/dt [Drug Therapy]; tacrolimus/ae [Adverse Drug Reaction]; tacrolimus/cb [Drug Combination]; tacrolimus/dt [Drug Therapy].

Link to the Ovid Full Text or citation:

Click here for full text options

52.

Three-year outcomes from BENEFIT-EXT: A phase III study of belatacept versus cyclosporine in recipients of extended criteria donor kidneys.

Pestana J.O.M., Grinyo J.M., Vanrenterghem Y., Becker T., Campistol J.M., Florman S., Garcia V.D., Kamar N., Lang P., Manfro R.C., Massari P., Rial M.D.C., Schnitzler M.A., Vitko S., Duan T., Block A., Harler M.B., Durrbach A.

American Journal of Transplantation. 12 (3) (pp 630-639), 2012. Date of Publication: March 2012.

AN: 2012123125

Recipients of extended-criteria donor (ECD) kidneys have poorer long-term outcomes compared to standard-criteria donor kidney recipients. We report 3-year outcomes from a randomized, phase III study in recipients of de novo ECD kidneys (n = 543) assigned (1:1:1) to either a more intensive (MI) or less intensive (LI) belatacept regimen, or cyclosporine. Three hundred twenty-three patients completed treatment by year 3. Patient survival with a functioning graft was comparable between groups (80% in MI, 82% in LI, 80% in cyclosporine). Mean calculated GFR (cGFR) was 11 mL/min higher in belatacept-treated versus cyclosporine-treated patients (42.7 in MI, 42.2 in LI, 31.5 mL/min in cyclosporine). More cyclosporine-treated patients (44%) progressed to GFR <30 mL/min (chronic kidney disease [CKD] stage 4/5) than belatacept-treated patients (27-30%). Acute rejection rates were similar between groups. Posttransplant lymphoproliferative disorder (PTLD) occurrence was higher in belatacept-treated patients (two in MI, three in LI), most of which occurred during the first 18 months; four additional cases (3 in LI, 1 in cyclosporine) occurred after 3 years. Tuberculosis was reported in two MI, four LI and no cyclosporine patients. In conclusion, at 3 years after transplantation, immunosuppression with belatacept resulted in similar patient survival, graft survival and acute rejection, with better renal function compared with cyclosporine. As previously reported, PTLD and tuberculosis were the principal safety findings associated with belatacept in this study population. Three-year results from the BENEFIT-EXT study confirm the durability of the renal function benefits of belatacept over time compared to cyclosporine, while providing comparable 3-year patient/graft survival and balancing risks associated with belatacept in recipients of extended criteria donor kidneys. © copyright 2012 The American Society of Transplantation and the American Society of Transplant Surgeons.

Institution

(Pestana) Department of Medicine, Division of Nephrology, Hospital Do Rim e Hipertensao Unifesp, Sao Paulo, Brazil (Grinyo) Division of Nephrology, University Hospital, Bellvitge, Barcelona, Spain

(Vanrenterghem) Department of Nephrology, University Hospital Leuven, Leuven, Belgium

(Becker) Department of Abdominal and Transplantation Surgery, Medizinische Hochschule Hannover, Hannover, Germany

(Campistol) Renal Transplantation Unit, Hospital Clinic i Provincial, University of Barcelona, Barcelona, Spain

(Florman) Recanati/Miller Transplantation Institute, Mount Sinai Medical Center, New York, NY, United States

(Garcia) Kidney and Pancreas Transplant Unit, Hospital Dom Vicente Scherer, Porto Alegre, Brazil

(Kamar) Department of Nephrology, Dialysis and Organ Transplantation, INSERM U858, IFR 31, CHU Rangueil, Toulouse Cedex, France

(Lang) Nephrology Department, AP-HP, Hospital Henri Mondor, IFRNT, INSERM U955, Universite Paris-Est, Creteil, France

(Manfro) Division of Nephrology, Renal Transplant Unit, Hospital de Clinicas de Porto Alegre, Porto Alegre, Brazil

(Massari) Renal Transplant Program, Hospital Privado Centro Medico de Cordoba, Cordoba, Argentina

(Rial) Kidney Transplant Unit, Instituto de Nefrologia, Buenos Aires, Argentina

(Schnitzler) St. Louis University Center for Outcomes Research, St. Louis University, St. Louis, MO, United States

(Vitko) Department of Nephrology, Institute for Clinical and Experimental Medicine, Prague, Czech Republic

(Duan) Global Biometric Sciences, Bristol-Myers Squibb, Hopewell, NJ, United States

(Block, Harler) Global Clinical Research, Bristol-Myers Squibb, Princeton, NJ, United States

(Durrbach) Department of Nephrology, Bicetre Hopital, Kremlin Bicetre, IFRNT, Universite Paris Sud, France

(Becker) Klinik fur Allgemeine und Thoraxchirurgie, Universitatsklinikum Schleswig-Holstein, Kiel, Germany

Publisher

Blackwell Publishing Ltd (9600 Garsington Road, Oxford OX4 2XG, United Kingdom)

Emtree Heading

acute graft rejection/dt [Drug Therapy]; adult; anemia/si [Side Effect]; article; basal cell carcinoma/si [Side Effect]; cancer incidence; candidiasis/si [Side Effect]; cause of death; chronic kidney disease/su [Surgery]; constipation/si [Side Effect]; cytomegalovirus infection/si [Side Effect]; diabetes mellitus; diarrhea/si [Side Effect]; drug dose reduction; drug efficacy; drug safety; drug withdrawal; fever/si [Side Effect]; genital herpes/si [Side Effect]; glomerulus filtration rate; graft dysfunction/si [Side Effect]; graft survival; herpes simplex/si [Side Effect]; herpes virus infection/si [Side Effect]; herpes zoster/si [Side Effect]; human; hyperlipidemia/dt [Drug Therapy]; hypertension/dt [Drug Therapy]; hypertension/si [Side Effect]; *immunosuppressive treatment; infection/co [Complication]; infection rate; Kaposi sarcoma/si [Side Effect]; kidney function; *kidney graft rejection/co [Complication]; *kidney graft rejection/dt [Drug Therapy]; *kidney graft rejection/pc [Prevention]; *kidney transplantation; leukopenia/si [Side Effect]; lymphoproliferative disease/si [Side Effect]; major clinical study; multicenter study; mycosis/si [Side Effect]; nausea/si [Side Effect]; onychomycosis/si [Side Effect]; organ transplantation; outcomes research; patient compliance; peripheral edema/si [Side Effect]; phase 3 clinical trial; polyomavirus infection/si [Side Effect]; posttransplant lymphoproliferative disorder; priority journal; prostate cancer/si [Side Effect]; randomization; renal protection; rhinopharyngitis/si [Side Effect]; skin carcinoma/si [Side Effect]; squamous cell carcinoma/si [Side Effect]; survival rate; thrush/si [Side Effect]; tinea versicolor/si [Side Effect]; treatment outcome; tuberculosis/si [Side Effect]; urinary tract infection/si [Side Effect]; virus infection/si [Side Effect]; vomiting/si [Side Effect]; antihypertensive agent/dt [Drug Therapy]; antilipemic agent/dt [Drug Therapy]; basiliximab/dt [Drug Therapy]; basiliximab/iv [Intravenous Drug Administration]; *belatacept/ae [Adverse Drug Reaction]; *belatacept/ct [Clinical Trial]; *belatacept/cm [Drug Comparison]; *belatacept/dt [Drug Therapy]; *belatacept/pd [Pharmacology]; corticosteroid/do [Drug Dose]; corticosteroid/dt [Drug Therapy]; *cyclosporin/ae [Adverse Drug Reaction]; *cyclosporin/ct [Clinical Trial]; *cyclosporin/cm [Drug Comparison]; *cyclosporin/cr [Drug Concentration]; *cyclosporin/dt [Drug Therapy]; immunosuppressive agent/dt [Drug Therapy]; mycophenolic acid 2 morpholinoethyl ester/dt [Drug Therapy].

Link to the Ovid Full Text or citation:

Click here for full text options

53.

Has nanotechnology led to improved therapeutic outcomes?.

Bosselmann S., Williams III R.O.

Drug Development and Industrial Pharmacy. 38 (2) (pp 158-170), 2012. Date of Publication: February 2012.

AN: 2011706406

Nanoparticulate drug delivery systems provide new opportunities for solving issues associated with problematic drugs or disease states and have, therefore, created great expectations in the field of drug delivery. This review focuses on the potential benefits of nanoparticles compared with other conventional delivery systems. Several nanoparticulate drug delivery systems have already been marketed or are currently under development and are presented in this review. Results from clinical trials demonstrate that nanoparticulate formulations generally enable superior therapeutic outcomes than compared with standard formulations. Therefore, the implementation of nanotechnology in drug delivery represents an important advance with substantial potential to improve therapeutic effectiveness and increase patient's quality of life.

Institution

(Bosselmann, Williams III) Division of Pharmaceutics, University of Texas at Austin, Austin, TX, United States

Publisher

Informa Healthcare (69-77 Paul Street, London EC2A 4LQ, United Kingdom)

Emtree Heading

acquired immune deficiency syndrome/dt [Drug Therapy]; anorexia/dt [Drug Therapy]; area under the curve; Aspergillus fumigatus; asthma/dt [Drug Therapy]; breast cancer/dt [Drug Therapy]; cachexia/dt [Drug Therapy]; cancer chemotherapy; cancer resistance; chemotherapy induced emesis/dt [Drug Therapy]; chemotherapy induced emesis/pc [Prevention]; cholesterol blood level; comparative study; drug absorption; drug bioavailability; drug clearance; *drug delivery system; drug dosage form; drug dose reduction; drug efficacy; drug elimination; drug formulation; drug hypersensitivity/pc [Prevention]; drug marketing; drug safety; drug solubility; drug targeting; drug uptake; dyslipidemia/dt [Drug Therapy]; epileptic state/dt [Drug Therapy]; first pass effect; food drug interaction; heart arrest/dt [Drug Therapy]; human; hypercholesterolemia/dt [Drug Therapy]; hyperlipidemia/dt [Drug Therapy]; hypertriglyceridemia/dt [Drug Therapy]; insulin dependent diabetes mellitus/dt [Drug Therapy]; ischemic heart disease/dt [Drug Therapy]; kidney graft rejection/dt [Drug Therapy]; kidney graft rejection/pc [Prevention]; lipoprotein blood level; lung alveolus macrophage; lung aspergillosis/dt [Drug Therapy]; lung aspergillosis/pc [Prevention]; lung cancer/dt [Drug Therapy]; lung mycosis/dt [Drug Therapy]; lung mycosis/pc [Prevention]; lung tuberculosis/dt [Drug Therapy]; multidrug resistance; Mycobacterium tuberculosis; *nanotechnology; nausea/dt [Drug Therapy]; nausea/pc [Prevention]; nebulization; nonhuman; outcome assessment; particle size; patient compliance; phase 1 clinical trial (topic); phase 3 clinical trial (topic); powder inhaler; quality of life; randomized controlled trial (topic); review; single drug dose; steroid therapy; therapy effect; thermodynamics; triacylglycerol blood level; unspecified side effect/si [Side Effect]; vascular tumor/dt [Drug Therapy]; albumin; antineoplastic agent/dt [Drug Therapy]; antineoplastic agent/pr [Pharmaceutics]; aprepitant/ae [Adverse Drug Reaction]; aprepitant/ct [Clinical Trial]; aprepitant/cb [Drug Combination]; aprepitant/dt [Drug Therapy]; budesonide/dt [Drug Therapy]; budesonide/pr [Pharmaceutics]; budesonide/pk [Pharmacokinetics]; budesonide/pd [Pharmacology]; cremophor; dexamethasone/cb [Drug Combination]; dexamethasone/dt [Drug Therapy]; doxorubicin/dt [Drug Therapy]; doxorubicin/pr [Pharmaceutics]; doxorubicin/pk [Pharmacokinetics]; doxorubicin/pd [Pharmacology]; fenofibrate/cr [Drug Concentration]; fenofibrate/do [Drug Dose]; fenofibrate/dt [Drug Therapy]; fenofibrate/pr [Pharmaceutics]; fenofibrate/pk [Pharmacokinetics]; fluorouracil/dt [Drug Therapy]; fluorouracil/ih [Inhalational Drug Administration]; fluorouracil/pr [Pharmaceutics]; fluorouracil/pk [Pharmacokinetics]; fluorouracil/pd [Pharmacology]; high density lipoprotein cholesterol/ec [Endogenous Compound]; insulin/dt [Drug Therapy]; insulin/tr [Intratracheal Drug Administration]; insulin/pr [Pharmaceutics]; insulin/pd [Pharmacology]; insulin/sc [Subcutaneous Drug Administration]; isoniazid/cb [Drug Combination]; isoniazid/dt [Drug Therapy]; isoniazid/po [Oral Drug Administration]; isoniazid/pr [Pharmaceutics]; isoniazid/pk [Pharmacokinetics]; itraconazole/cr [Drug Concentration]; itraconazole/dt [Drug Therapy]; itraconazole/po [Oral Drug Administration]; itraconazole/pr [Pharmaceutics]; low density lipoprotein/ec [Endogenous Compound]; megestrol acetate/ct [Clinical Trial]; megestrol acetate/do [Drug Dose]; megestrol acetate/dt [Drug Therapy]; megestrol acetate/po [Oral Drug Administration]; megestrol acetate/pr [Pharmaceutics]; megestrol acetate/pk [Pharmacokinetics]; nanobudesonide/dt [Drug Therapy]; nanobudesonide/pr [Pharmaceutics]; nanobudesonide/pk [Pharmacokinetics]; nanobudesonide/pd [Pharmacology]; nanocrystal; *nanoparticle/ct [Clinical Trial]; *nanoparticle/cr [Drug Concentration]; *nanoparticle/dt [Drug Therapy]; *nanoparticle/to [Drug Toxicity]; *nanoparticle/iv [Intravenous Drug Administration]; *nanoparticle/po [Oral Drug Administration]; *nanoparticle/pr [Pharmaceutics]; *nanoparticle/pe [Pharmacoeconomics]; *nanoparticle/pk [Pharmacokinetics]; *nanoparticle/pd [Pharmacology]; paclitaxel/ct [Clinical Trial]; paclitaxel/cr [Drug Concentration]; paclitaxel/dt [Drug Therapy]; paclitaxel/iv [Intravenous Drug Administration]; paclitaxel/pr [Pharmaceutics]; paclitaxel/pk [Pharmacokinetics]; paclitaxel/pd [Pharmacology]; polyglactin; pyrazinamide/cb [Drug Combination]; pyrazinamide/dt [Drug Therapy]; pyrazinamide/po [Oral Drug Administration]; pyrazinamide/pr [Pharmaceutics]; pyrazinamide/pk [Pharmacokinetics]; rapamycin/ct [Clinical Trial]; rapamycin/dt [Drug Therapy]; rapamycin/po [Oral Drug Administration]; rapamycin/pr [Pharmaceutics]; rapamycin/pk [Pharmacokinetics]; rifampicin/cb [Drug Combination]; rifampicin/dt [Drug Therapy]; rifampicin/po [Oral Drug Administration]; rifampicin/pr [Pharmaceutics]; rifampicin/pk [Pharmacokinetics]; serotonin 3 antagonist/cb [Drug Combination]; serotonin 3 antagonist/dt [Drug Therapy]; solid lipid nanoparticle/pr [Pharmaceutics]; solid lipid nanoparticle/pd [Pharmacology]; triacylglycerol/ec [Endogenous Compound]; unclassified drug.

Drug Trade Names and Manufacturers

abraxane, emend, megace, rapamune, resmycin, taxol, tricor

Link to the Ovid Full Text or citation:

Click here for full text options

54.

Mycobacterium vaccae as adjuvant therapy to anti-tuberculosis chemotherapy in never-treated tuberculosis patients: a meta-analysis.

Yang X.Y., Chen Q.F., Li Y.P., Wu S.M.

PloS one. 6 (9) (pp e23826), 2011. Date of Publication: 2011.

AN: 21909406

To evaluate the effectiveness and safety of heat-killed M. vaccae added to chemotherapy of never-treated tuberculosis (TB) patients. The databases of Medline, Embase, Biosis, Cochrane Central Register of Controlled Trials, SCI, CBM, VIP and CNKI were searched. Randomized controlled trials (RCT) and Controlled clinical trials (CCT) comparing M. vaccae with or without a placebo-control injection as adjuvant therapy in the chemotherapy of never-treated TB patients were included. Two reviewers independently performed data extraction and quality assessment. Data were analyzed using RevMan 5.0 software by The Cochrane Collaboration. Fifty four studies were included. At the end of the follow-up period, Pooled RR (Risk Ratio) and its 95% CI of sputum smear conversion rate were 1.07 (1.04, 1.10) in TB patients without complications, 1.17 (0.92, 1.49) in TB patients with diabetes mellitus, 1.02 (0.94, 1.10) in TB patients with hepatitis B, and 1.46 (0.21, 10.06) in TB patients with pneumosilicosis. In elderly TB patients the RR was 1.22 (1.13, 1.32). Analysis of each time point during the follow-up period showed that M. vaccae could help to improve the removal of acid-fast bacilli from the sputum, and promote improvement of radiological focal lesions and cavity closure. Compared with the control group, the differences in levels of immunological indicators of Th1 such as IL-2 and TNF-alpha were not statistical significant (P = 0.65 and 0.31 respectively), and neither was that of IL-6 produced by Th2 (P = 0.52). An effect of M. vaccae of prevention of liver damage was found in TB patients with hepatitis B (RR 0.20 and 95% CI (0.12, 0.33). No systemic adverse events were reported. Added to chemotherapy, M. vaccae is helpful in the treatment of never-treated TB patients in terms of improving both sputum conversion and X-ray appearances.

Institution

(Yang) Chinese Evidence-Based Medicine Center, West China Hospital, Sichuan University, Chengdu, China.

Emtree Heading

adolescent; adult; aged; article; follow up; health care quality; hepatitis B/co [Complication]; human; immunology; liver; meta analysis; microbiology; middle aged; multimodality cancer therapy; *Mycobacterium; pathology; sputum; treatment outcome; *tuberculosis/co [Complication]; *tuberculosis/dt [Drug Therapy]; *BCG vaccine; *tuberculostatic agent/dt [Drug Therapy].

Link to the Ovid Full Text or citation:

Click here for full text options

55.

Vaccines of the future.

Nossal G.J.V.

Vaccine. 29 (SUPPL. 4) (pp D111-D115), 2011. Date of Publication: 30 Dec 2011.

AN: 2012204396

Vaccines of the future can be divided into three broad groups, namely those of the near future (<10 years); the medium-term future (10-19 years); and the long-term future (20-50 years).For the near future, there is some "low hanging fruit" which is clearly on the horizon, such as a Vi-conjugate vaccine for typhoid or a protein-based vaccine for Neisseria meningitidis serogroup B. Just slightly more distant will be vaccines for shigellosis and a common protein vaccine for Streptococcus pneumoniae. Also in this group, but not as far advanced, will be a vaccine for Group A streptococcus. I place vaccines for the "big three", malaria, tuberculosis and HIV/AIDS in the medium term basket. The sporozoite malaria vaccine RTS-S is closest, but surely a definitive malaria vaccine will also require antigens from other stages of the life cycle. A tuberculosis vaccine will be either a re-engineered BCG; or a molecular vaccine with several protein antigens; or one based on prime-boost strategies. What will delay this is the high cost of clinical trials. For HIV/AIDS, the partial success of the Sanofi-Pasteur prime-boost vaccine has given some hope. I still place much faith in antibody-based vaccines and especially on mimotopes of the env transitional state assumed after initial CD4 binding. Monoclonal antibodies are also leading us in interesting directions. Longer term, the vaccine approach will be successful for autoimmune diseases, e.g. juvenile diabetes and coeliac disease. Cancer vaccines are also briefly surveyed. Adjunct issues needing to be addressed include more extensive combinations; alternate delivery systems; and more intelligently designed adjuvants based on knowledge of the innate immune system. © 2011.

Institution

(Nossal) Department of Pathology, The University of Melbourne, Australia

Publisher

Elsevier Ltd (Langford Lane, Kidlington, Oxford OX5 1GB, United Kingdom)

Emtree Heading

*acquired immune deficiency syndrome/dt [Drug Therapy]; advanced cancer/dt [Drug Therapy]; antigen binding; article; autoimmune disease; B cell lymphoma/dt [Drug Therapy]; cancer adjuvant therapy; cancer immunotherapy; celiac disease; clinical practice; colon cancer/dt [Drug Therapy]; drug industry; drug protein binding; human; *Human immunodeficiency virus infection/dt [Drug Therapy]; innate immunity; insulin dependent diabetes mellitus; juvenile diabetes mellitus; *malaria/dt [Drug Therapy]; measles/dt [Drug Therapy]; melanoma/dt [Drug Therapy]; mumps/dt [Drug Therapy]; pharmaceutical engineering; phase 2 clinical trial (topic); phase 3 clinical trial (topic); Plasmodium malariae; priority journal; rubella/dt [Drug Therapy]; sporozoite; treatment outcome; *tuberculosis/dt [Drug Therapy]; tuberculous meningitis/dt [Drug Therapy]; vaccine production; af 03; antineoplastic agent/dt [Drug Therapy]; as 03; as 04; *BCG vaccine/dt [Drug Therapy]; cancer vaccine/ct [Clinical Trial]; cancer vaccine/dt [Drug Therapy]; CD4 antigen/ec [Endogenous Compound]; cytotoxic T lymphocyte antigen 4 antibody/dt [Drug Therapy]; *Human immunodeficiency virus vaccine/dt [Drug Therapy]; *malaria vaccine/dt [Drug Therapy]; measles mumps rubella vaccine/dt [Drug Therapy]; measles vaccine/dt [Drug Therapy]; measles vaccine/ih [Inhalational Drug Administration]; measles vaccine/na [Intranasal Drug Administration]; mifamurtide; monoclonal antibody/pd [Pharmacology]; sporozoite vaccine/dt [Drug Therapy]; unclassified drug.

Drug Trade Names and Manufacturers

Glaxo SmithKline, Novartis, Sanofi Pasteur, af 03: Sanofi Pasteur, as 03: Glaxo SmithKline, as 04: Glaxo SmithKline, mf 59: Novartis

Link to the Ovid Full Text or citation:

Click here for full text options

56.

Pitavastatin: Novel effects on lipid parameters.

Chapman M.J.

Atherosclerosis Supplements. 12 (3) (pp 277-284), 2011. Date of Publication: November 2011.

AN: 2011675734

Atherogenic dyslipidemia is characterised by high levels of triglycerides, low levels of high-density lipoprotein-cholesterol (HDL-C), and moderate to marked elevations in low-density lipoprotein-cholesterol (LDL-C) concentrations; such dyslipidemia is further characterised by high apolipoprotein B (apoB): apolipoprotein A1 (apoA1) ratios. Numerous clinical trials have demonstrated that statins are effective in lowering LDL-C and reducing cardiovascular (CV) risk in people with dyslipidemia. However, the most effective treatments should target all of the key atherogenic features, rather than LDL-C alone. Pitavastatin is a new member of the statin class whose distinct pharmacological features translate into a broad spectrum of action on both apoB-containing and apoA1-containing lipoprotein components of the atherogenic lipid profile. The efficacy and safety of this statin has been demonstrated by a large clinical development programme conducted both in Japanese and Caucasian populations. Phase III and IV studies in a wide range of patients with primary hypercholesterolemia or combined dyslipidemia showed that 12 weeks' treatment with pitavastatin l-4 mg was well tolerated, significantly improved lipid profiles (including LDL-C, TG, and HDL-C levels) and increased the EAS-/NCEP ATP Ill-recommended LDL-C target attainment rate to a similar or greater degree as comparable doses of atorvastatin, simvastatin, or pravastatin. Results were similar across all patient groups and were generally sustained after 52 weeks of treatment. However, whereas the effects of atorvastatin and simvastatin on HDL-C levels remained constant over the long term, pitavastatin-treated patients experienced progressive and maintained elevations in HDL-C, ultimately increasing by up to 14.3% vs. initial baseline. In this context, it is significant that the in vitro studies of Yamashita et al. [J Atheroscler Thromb 2010;17:436-51] have shown pitavastatin to be distinguished by its potent stimulation of apoA1 production in hepatocyte-like cells. These findings suggest that pitavastatin may be highly efficacious in raising levels of lipid-poor apoA1 particles, which are known to be highly active in ABCA1-mediated cellular cholesterol efflux, an observation which is pertinent to the excessive accumulation of cholesterol in macrophage foam cells of the atherosclerotic plaque. Indeed, the intravascular remodelling and maturation of lipid-poor apoA1 particles is known to drive flux of apoA1, cholesterol and phospholipid through the HDL pathway. It is equally relevant that pitavastatin therapy has been shown to be efficacious in markedly reducing coronary atheroma volume in acute coronary syndrome patients in the JAPAN-ACS trial, a therapeutic effect which may be linked to its impact on apoA1/HDL metabolism and function. Overall, Phase III and IV studies demonstrate that pitavastatin 1-4 mg is well tolerated, attenuates the atherogenic lipid profile and increases LDL-C target attainment rates with a similar or greater efficacy to comparable doses of atorvastatin, simvastatin and pravastatin. Furthermore, pitavastatin may be particularly beneficial in high-risk patients with elevated concentrations of TG-rich lipoproteins and low levels of HDL-C, and in whom the atheroprotective function of HDL particles is typically defective; significantly, such patients typically exhibit persistent, residual cardiometabolic risk even when LDL-C is at goal. In this context, it is relevant that such patient groups cover a wide spectrum of metabolic diseases, including metabolic syndrome, type 2 diabetes, coronary disease, familial and non-familial forms of hypercholesterolemia, auto-immune diseases such as rheumatoid arthritis and lupus, renal disease and some forms of hepatic insufficiency. © 2011 Elsevier Ireland Ltd.

Institution

(Chapman) Institut National de la Sante et de la Recherche Medicale (INSERM) UMR-S939, Pavillon Benjamin Delessert, Hopital de la Pitie, 83 Blud l'Hopital, 75651 Paris Cedex 13, France

Publisher

Elsevier Ireland Ltd (P.O. Box 85, Limerick, Ireland)

Emtree Heading

acute coronary syndrome; article; atheroma; atherosclerosis; atherosclerotic plaque; autoimmune disease; cardiovascular risk; Caucasian; coronary artery disease; drug dose titration; drug efficacy; drug mechanism; drug tolerability; *dyslipidemia/dt [Drug Therapy]; high risk population; human; *hypercholesterolemia/dt [Drug Therapy]; in vitro study; Japanese; kidney disease; liver failure; lupus vulgaris; macrophage; metabolic syndrome X; myopathy/si [Side Effect]; myositis/si [Side Effect]; non insulin dependent diabetes mellitus; phase 3 clinical trial (topic); phase 4 clinical trial (topic); priority journal; rhabdomyolysis/si [Side Effect]; rheumatoid arthritis; treatment outcome; apolipoprotein A1/ec [Endogenous Compound]; apolipoprotein B/ec [Endogenous Compound]; atorvastatin/cm [Drug Comparison]; atorvastatin/dt [Drug Therapy]; cholesterol/ec [Endogenous Compound]; high density lipoprotein cholesterol/ec [Endogenous Compound]; hydroxymethylglutaryl coenzyme A reductase inhibitor/pd [Pharmacology]; *lipid/ec [Endogenous Compound]; low density lipoprotein cholesterol/ec [Endogenous Compound]; phospholipid/ec [Endogenous Compound]; *pitavastatin/ae [Adverse Drug Reaction]; *pitavastatin/ct [Clinical Trial]; *pitavastatin/cm [Drug Comparison]; *pitavastatin/dt [Drug Therapy]; *pitavastatin/pd [Pharmacology]; pravastatin/ae [Adverse Drug Reaction]; pravastatin/cm [Drug Comparison]; pravastatin/dt [Drug Therapy]; simvastatin/cm [Drug Comparison]; simvastatin/dt [Drug Therapy]; triacylglycerol/ec [Endogenous Compound].

Link to the Ovid Full Text or citation:

Click here for full text options

57.

Alemtuzumab preconditioning allows steroid-calcineurin inhibitor-free regimen in live-donor kidney transplant.

Refaie A.F., Mahmoud K.M., Ismail A.M., Sheashaa H.A., Kamal A.I., Ghoneim M.A.

Experimental and Clinical Transplantation. 9 (5) (pp 295-301), 2011. Date of Publication: October 2011.

AN: 2011565333

Objectives: This prospective study was designed to develop a steroid and calcineurin inhibitor-free regimen for kidney transplants using alemtuzumab. Materials and Methods: A single dose of alemtuzumab (30 mg) was given preoperatively. Phase 1: Twenty-one patients were randomized into 2 groups; the tacrolimus (n=11) and the sirolimus groups (n=10). Steroids were given for 5 days. Azathioprine (1 mg/kg) was added when white blood cells > 4000 cells/cm3. Mean follow-up was 48 +/- 2.8 and 48.2 +/- 1.6 months for the tacrolimus and sirolimus groups. Phase 2: Twenty patients were included and the study design was modified. Tacrolimus was given for 2 months, and was replaced by sirolimus thereafter. The mean follow-up was 28.3 +/- 2.1 months. Results: Phase 1: Acute rejection episodes were encountered in 5 patients of the tacrolimus versus 2 cases in the sirolimus group (P =.44). Antibodymediated rejection was diagnosed in 2 recipients in each group. Four patients were switched from sirolimus to tacrolimus owing to resistant rejection, significant proteinuria, persistent thrombocytopenia, lymphocele, and urinary leakage. One patient was shifted from tacrolimus to sirolimus owing to Kaposi sarcoma. Glomerular filtration rate was significantly higher in the sirolimus group. Currently, 14 patients (8 tacrolimus, and 6 sirolimus) are steroid-free. One patient died from the tacrolimus group owing to fulminant hepatitis. Two grafts were lost in the sirolimus group versus 1 graft in the tacrolimus group Phase 2: Five patients developed successfully treated borderline changes with no antibodymediated rejection. Mean serum creatinine was 114.9 +/- 17.7 mumol/L. Currently, 17 patients are steroidfree and 15 of them are calcineurin inhibitor-free as well. In this phase, only 1 patient died with a functioning graft. Conclusions: This clinical trial provides a good insight into a potentially effective steroid and calcineurin inhibitor-free protocol with the use of alemtuzumab induction in combination with sirolimus. © Baskent University 2011.

Institution

(Refaie, Mahmoud, Sheashaa, Kamal) Departments of Nephrology, Departments of Urology and Nephrology Center, Mansoura 35516, Egypt (Ismail) Departments of Immunology, Departments of Urology and Nephrology Center, Mansoura 35516, Egypt

(Ghoneim) Departments of Urology and Nephrology Center, Mansoura 35516, Egypt

Publisher

Baskent University (26 Austin Avenue,Baglica Kampusu, P.O. Box 337,Ankara 06530, Turkey)

Emtree Heading

adult; antibody response; article; clinical article; clinical protocol; controlled study; creatinine blood level; cytomegalovirus infection/dt [Drug Therapy]; cytomegalovirus infection/si [Side Effect]; death; diabetes mellitus/co [Complication]; diabetes mellitus/si [Side Effect]; drug blood level; drug dose reduction; drug efficacy; drug pulse therapy; drug substitution; drug tolerability; drug withdrawal; female; follow up; glomerulus filtration rate; graft failure/co [Complication]; graft recipient; graft survival; hepatitis; hepatitis B/co [Complication]; hepatitis B/dt [Drug Therapy]; herpes zoster/dt [Drug Therapy]; herpes zoster/si [Side Effect]; human; human tissue; *immunosuppressive treatment; Kaposi sarcoma/co [Complication]; Kaposi sarcoma/dt [Drug Therapy]; kidney biopsy; kidney donor; kidney graft rejection/co [Complication]; kidney graft rejection/dr [Drug Resistance]; kidney graft rejection/dt [Drug Therapy]; kidney graft rejection/pc [Prevention]; kidney graft rejection/th [Therapy]; *kidney transplantation; leukocyte count; *living donor; lung tuberculosis/co [Complication]; lung tuberculosis/dt [Drug Therapy]; lymphocele/co [Complication]; lymphocyte depletion; male; multiple cycle treatment; nephrotoxicity/si [Side Effect]; outcome assessment; phase 1 clinical trial; phase 2 clinical trial; plasmapheresis; preoperative treatment; prospective study; proteinuria/si [Side Effect]; Pseudomonas pneumonia/dt [Drug Therapy]; Pseudomonas pneumonia/si [Side Effect]; randomized controlled trial; single drug dose; thrombocytopenia; treatment duration; urine incontinence/co [Complication]; aciclovir/dt [Drug Therapy]; aciclovir/iv [Intravenous Drug Administration]; aciclovir/po [Oral Drug Administration]; *alemtuzumab/ct [Clinical Trial]; *alemtuzumab/do [Drug Dose]; *alemtuzumab/dt [Drug Therapy]; *alemtuzumab/iv [Intravenous Drug Administration]; antibiotic agent/dt [Drug Therapy]; azathioprine/cb [Drug Combination]; azathioprine/dt [Drug Therapy]; creatinine/ec [Endogenous Compound]; entecavir/cb [Drug Combination]; entecavir/dt [Drug Therapy]; ganciclovir/dt [Drug Therapy]; ganciclovir/iv [Intravenous Drug Administration]; ganciclovir/po [Oral Drug Administration]; immunoglobulin/dt [Drug Therapy]; immunoglobulin/iv [Intravenous Drug Administration]; lamivudine/cb [Drug Combination]; lamivudine/dt [Drug Therapy]; methylprednisolone/dt [Drug Therapy]; methylprednisolone/iv [Intravenous Drug Administration]; mycophenolic acid 2 morpholinoethyl ester/cb [Drug Combination]; mycophenolic acid 2 morpholinoethyl ester/dt [Drug Therapy]; paclitaxel/cb [Drug Combination]; paclitaxel/dt [Drug Therapy]; paclitaxel/iv [Intravenous Drug Administration]; *rapamycin/ae [Adverse Drug Reaction]; *rapamycin/ct [Clinical Trial]; *rapamycin/cb [Drug Combination]; *rapamycin/cm [Drug Comparison]; *rapamycin/cr [Drug Concentration]; *rapamycin/dt [Drug Therapy]; rituximab/dt [Drug Therapy]; steroid/cb [Drug Combination]; steroid/dt [Drug Therapy]; *tacrolimus/ae [Adverse Drug Reaction]; *tacrolimus/ct [Clinical Trial]; *tacrolimus/cb [Drug Combination]; *tacrolimus/cm [Drug Comparison]; *tacrolimus/cr [Drug Concentration]; *tacrolimus/dt [Drug Therapy]; tuberculostatic agent/dt [Drug Therapy]; vincristine/cb [Drug Combination]; vincristine/dt [Drug Therapy].

Drug Trade Names and Manufacturers

Ilex Oncology [United Kingdom], mabcampath: Ilex Oncology [United Kingdom]

Link to the Ovid Full Text or citation:

Click here for full text options

58.

Effect of age on presentation with diabetes: Comparison of nondiabetic patients with new smear-positive pulmonary tuberculosis patients.

Rawat J., Sindhwani G., Biswas D.

Lung India. 28 (3) (pp 187-190), 2011. Date of Publication: July-September 2011.

AN: 2011500012

Background: Diabetes mellitus (DM) has been reported to modify the presenting features of pulmonary tuberculosis (PTB), but data regarding the effect of diabetes on the presentation of PTB are highly variable. Objective: To determine whether DM alters the demographic, clinical, and radiological manifestations of tuberculosis and whether the effect of diabetes varies with the age group of PTB patients. Materials and Methods : This prospective observational study was conducted on new smear-positive PTB patients with DM (PTB-DM group) and non-diabetic PTB patients (PTB group). Patients of both groups were again divided into six age groups (15-29, 30-39, 40-49, 50-59, 60-69, and >70 rears) to analyze and compare the impact of age on clinicoradiological presentations of PTB. Results: Patients in the PTB-DM group were significantly older (53.34 14.06 year) in comparison to their nondiabetic counterparts (PTB group) (44.35 18.14 year) (P < 0.001). The former group also had a lower male:female ratio, although the difference was not statistically significant (1.16:1 vs. 2.05:1, P = 0.101). Tuberculin positivity was significantly higher in the PTB group, compared with patients in the PTB-DM group (P < 0.004). The proportion of patients with lower lung field involvement (P = 0.003) and cavitations (P = 0.005) was also higher in the former group compared with the latter. Conclusion: Diabetic patients with tuberculosis were relatively older, had lower tuberculin positivity, and higher proportion of lower lung field involvement and cavitation in comparison to nondiabetic patients.

Institution

(Rawat, Sindhwani) Department of Pulmonary Medicine, Himalayan Institute of Medical Sciences, Dehradun, Uttarakhand 248140, India (Biswas) Department of Microbiology, Himalayan Institute of Medical Sciences, Dehradun, Uttarakhand, India

Publisher

Medknow Publications and Media Pvt. Ltd (B9, Kanara Business Centre, off Link Road, Ghatkopar (E), Mumbai 400 075, India)

Emtree Heading

adolescent; adult; *age distribution; aged; article; comparative study; controlled clinical trial; controlled study; coughing; *diabetes mellitus; diabetic patient; female; fever; hemoptysis; human; *lung tuberculosis/di [Diagnosis]; major clinical study; male; observational study; outcome assessment; prospective study; sex difference; thorax radiography; tuberculin test.

Link to the Ovid Full Text or citation:

Click here for full text options

59.

Mycobacterium vaccae as adjuvant therapy to anti-tuberculosis chemotherapy in never-treated tuberculosis patients: A meta-analysis.

Yang X.-Y., Chen Q.-F., Li Y.-P., Wu S.-M.

PLoS ONE. 6 (9) (no pagination), 2011. Article Number: e23826. Date of Publication: 2011.

AN: 2011498754

Objective: To evaluate the effectiveness and safety of heat-killed M.vaccae added to chemotherapy of never-treated tuberculosis (TB) patients. Methods: The databases of Medline, Embase, Biosis, Cochrane Central Register of Controlled Trials, SCI, CBM, VIP and CNKI were searched. Randomized controlled trials (RCT) and Controlled clinical trials (CCT) comparing M.vaccae with or without a placebo-control injection as adjuvant therapy in the chemotherapy of never-treated TB patients were included. Two reviewers independently performed data extraction and quality assessment. Data were analyzed using RevMan 5.0 software by The Cochrane Collaboration. Results: Fifty four studies were included. At the end of the follow-up period, Pooled RR (Risk Ratio) and its 95% CI of sputum smear conversion rate were 1.07 (1.04, 1.10) in TB patients without complications, 1.17 (0.92, 1.49) in TB patients with diabetes mellitus, 1.02 (0.94, 1.10) in TB patients with hepatitis B, and 1.46 (0.21, 10.06) in TB patients with pneumosilicosis. In elderly TB patients the RR was 1.22 (1.13, 1.32). Analysis of each time point during the follow-up period showed that M.vaccae could help to improve the removal of acid-fast bacilli from the sputum, and promote improvement of radiological focal lesions and cavity closure. Compared with the control group, the differences in levels of immunological indicators of Th1 such as IL-2 and TNF-alpha were not statistical significant (P = 0.65 and 0.31 respectively), and neither was that of IL-6 produced by Th2 (P = 0.52). An effect of M.vaccae of prevention of liver damage was found in TB patients with hepatitis B (RR 0.20 and 95% CI (0.12, 0.33). No systemic adverse events were reported. Conclusion: Added to chemotherapy, M.vaccae is helpful in the treatment of never-treated TB patients in terms of improving both sputum conversion and X-ray appearances. © 2011 Yang et al.

Institution

(Yang, Chen, Li) Chinese Evidence-Based Medicine Center/The Chinese Cochrane Center, West China Hospital, Sichuan University, Chengdu, China (Wu) West China Medical School, Sichuan University, Chengdu, China

Publisher

Public Library of Science (185 Berry Street, Suite 1300, San Francisco CA 94107, United States)

Emtree Heading

acid fast bacterium; *adjuvant therapy; adult; aged; aging; antibacterial activity; arm disease/si [Side Effect]; controlled clinical trial; controlled study; cytokine production; data analysis software; data extraction; diabetes mellitus; drug efficacy; drug fever/si [Side Effect]; drug safety; drug tolerability; hepatitis B; human; immunotherapy; injection site induration/si [Side Effect]; injection site pain/si [Side Effect]; liver injury/dt [Drug Therapy]; liver injury/pc [Prevention]; major clinical study; meta analysis; Mycobacterium vaccae; nonhuman; outcomes research; review; silicosis; single drug dose; sputum smear; systematic review; systemic disease/si [Side Effect]; Th1 cell; Th2 cell; thorax radiography; *tuberculosis/dt [Drug Therapy]; interleukin 2/ec [Endogenous Compound]; interleukin 6/ec [Endogenous Compound]; isoniazid/cb [Drug Combination]; isoniazid/dt [Drug Therapy]; *Mycobacterium vaccae vaccine/ae [Adverse Drug Reaction]; *Mycobacterium vaccae vaccine/ct [Clinical Trial]; *Mycobacterium vaccae vaccine/dt [Drug Therapy]; *Mycobacterium vaccine/ae [Adverse Drug Reaction]; *Mycobacterium vaccine/ct [Clinical Trial]; *Mycobacterium vaccine/dt [Drug Therapy]; placebo; pyrazinamide/cb [Drug Combination]; pyrazinamide/dt [Drug Therapy]; rifampicin/cb [Drug Combination]; rifampicin/dt [Drug Therapy]; streptomycin/cb [Drug Combination]; streptomycin/dt [Drug Therapy]; tumor necrosis factor alpha/ec [Endogenous Compound]; unclassified drug.

Link to the Ovid Full Text or citation:

Click here for full text options

60.

Drug repositioning: Re-investigating existing drugs for new therapeutic indications.

Padhy B.M., Gupta Y.K.

Journal of Postgraduate Medicine. 57 (2) (pp 153-160), 2011. Date of Publication: April-June 2011.

AN: 2011479478

Drug discovery and development is an expensive, time-consuming, and risky enterprise. In order to accelerate the drug development process with reduced risk of failure and relatively lower costs, pharmaceutical companies have adopted drug repositioning as an alternative. This strategy involves exploration of drugs that have already been approved for treatment of other diseases and/or whose targets have already been discovered. Various techniques including data mining, bioinformatics, and usage of novel screening platforms have been used for identification and screening of potential repositioning candidates. However, challenges in clinical trials and intellectual property issues may be encountered during the repositioning process. Nevertheless, such initiatives not only add value to the portfolio of pharmaceutical companies but also provide an opportunity for academia and government laboratories to develop new and innovative uses of existing drugs for infectious and neglected diseases, especially in emerging countries like India.

Institution

(Padhy) Department of Pharmacology, Hamdard Institute of Medical Sciences and Research, New Delhi, India (Gupta) Department of Pharmacology, All India Institute of Medical Sciences, New Delhi, India

Publisher

Medknow Publications and Media Pvt. Ltd (B9, Kanara Business Centre, off Link Road, Ghatkopar (E), Mumbai 400 075, India)

Emtree Heading

acne/dt [Drug Therapy]; acquired immune deficiency syndrome/dt [Drug Therapy]; acute heart infarction; African trypanosomiasis/dt [Drug Therapy]; alcoholism/dt [Drug Therapy]; amyotrophic lateral sclerosis/dt [Drug Therapy]; angina pectoris/dt [Drug Therapy]; angiogenesis; anthelmintic activity; attention deficit disorder/dt [Drug Therapy]; autoimmune disease; bacterial infection/dt [Drug Therapy]; bioinformatics; breast cancer/dt [Drug Therapy]; cachexia; Caenorhabditis elegans; cell assay; clinical trial (topic); computer program; data base; data mining; depression/dt [Drug Therapy]; developed country; developing country; diabetes mellitus/dt [Drug Therapy]; Drosophila melanogaster; drug control; drug cost; drug efficacy; drug identification; drug manufacture; drug marketing; drug mechanism; drug quality; *drug repositioning; drug safety; drug screening; drug treatment failure; epilepsy/dt [Drug Therapy]; erectile dysfunction/dt [Drug Therapy]; erythema nodosum leprosum/dt [Drug Therapy]; Europe; ex vivo study; eye disease; eyelash; fibromyalgia/dt [Drug Therapy]; food and drug administration; genomics; glaucoma/dt [Drug Therapy]; gout/dt [Drug Therapy]; government; hematopoietic stem cell; high throughput screening; hirsutism/dt [Drug Therapy]; human; Human immunodeficiency virus; Human immunodeficiency virus infection/dt [Drug Therapy]; hypertension/dt [Drug Therapy]; in vivo study; India; infection; inflammation/dt [Drug Therapy]; influenza/dt [Drug Therapy]; leishmaniasis/dt [Drug Therapy]; leprosy/dt [Drug Therapy]; library; lifestyle; lymphatic filariasis; malaria/dt [Drug Therapy]; male type alopecia/dt [Drug Therapy]; melanoma/dt [Drug Therapy]; migraine/dt [Drug Therapy]; migraine/pc [Prevention]; morbidity; morning sickness/dt [Drug Therapy]; mortality; multiple myeloma/dt [Drug Therapy]; mycosis/dt [Drug Therapy]; neoplasm/dt [Drug Therapy]; neurologic disease; neuropathic pain/dt [Drug Therapy]; nonhuman; obesity; osteoarthritis; osteoporosis/dt [Drug Therapy]; pain/dt [Drug Therapy]; Parkinson disease/dt [Drug Therapy]; patent; pediatrics; pericarditis/dt [Drug Therapy]; pharmacogenetics; phase 1 clinical trial (topic); phenotype; Plasmodium falciparum; Plasmodium vivax; pregnant woman; promyelocytic leukemia/dt [Drug Therapy]; proteomics; psoriasis/dt [Drug Therapy]; recurrent disease/dt [Drug Therapy]; restless legs syndrome/dt [Drug Therapy]; review; rheumatoid arthritis/dt [Drug Therapy]; risk; smoking cessation; stress incontinence/dt [Drug Therapy]; *treatment indication; tropical disease; tuberculosis/dt [Drug Therapy]; United States; visceral leishmaniasis/dt [Drug Therapy]; yaws; acetylsalicylic acid/dt [Drug Therapy]; amantadine/dt [Drug Therapy]; amfebutamone/dt [Drug Therapy]; amphotericin/dt [Drug Therapy]; atomoxetine/dt [Drug Therapy]; auranofin/dt [Drug Therapy]; bimatoprost/dt [Drug Therapy]; bromocriptine/dt [Drug Therapy]; ceftriaxone/dt [Drug Therapy]; clofazimine/dt [Drug Therapy]; colchicine/dt [Drug Therapy]; dapsone/dt [Drug Therapy]; disulfiram/dt [Drug Therapy]; duloxetine/dt [Drug Therapy]; eflornithine/dt [Drug Therapy]; gabapentin/dt [Drug Therapy]; methotrexate/dt [Drug Therapy]; milnacipran/dt [Drug Therapy]; miltefosine/dt [Drug Therapy]; minocycline/dt [Drug Therapy]; minoxidil/dt [Drug Therapy]; plerixafor/dt [Drug Therapy]; propranolol/dt [Drug Therapy]; raloxifene/ct [Clinical Trial]; raloxifene/dt [Drug Therapy]; retinoic acid/dt [Drug Therapy]; ropinirole/dt [Drug Therapy]; sildenafil/ct [Clinical Trial]; sildenafil/dt [Drug Therapy]; thalidomide/dt [Drug Therapy]; unindexed drug; zidovudine/dt [Drug Therapy].

Drug Trade Names and Manufacturers

aspirin

Link to the Ovid Full Text or citation:

Click here for full text options

61.

Alemtuzumab induction in deceased donor kidney transplantation.

Shin M., Song S.H., Kim J.M., Kwon C.H., Joh J.W., Lee S.-K., Kim S.-J.

Transplantation Proceedings. 43 (6) (pp 2365-2378), 2011. Date of Publication: July-August 2011.

AN: 2011450223

Purpose: Alemtuzumab (Campath-1H), a humanized monoclonal antibody directed against CD52, is a lymphocyte-depleting agent currently being evaluated as an induction agent in solid organ transplantation. This study analyzed the clinical outcomes and effects on peripheral blood lymphocyte subset counts in adult deceased donor renal transplant recipients who received an alemtuzumab-based induction protocol. Methods: Eleven kidney alone or simultaneous pancreas-kidney transplant recipients received 20 mg alemtuzumab on postoperative days 0 and 1, followed by calcineurin inhibitorbased maintenance immunosuppression after postoperative day 5. We collected 1-year data including recipient and donor demographic features, renal function and adverse events including endocrine impact, incidence of acute rejection episodes, infections or malignancies as well as hematologic and late immunologic parameters for correlation with patient or graft survival. Results: Mean HLA mismatch was 3.6 and 8/11 deceased donors were of the extended criteria type. Only 2 (18%) recipients displayed delayed graft function with a failure of the serum creatinine to decrease by 25% on the first day; however, their long-term outcomes were similar to other nonaffected patients. Serious adverse events were absent; there was no hyperlipidemia or new-onset diabetes. We failed to observe an acute rejection. The 3 (27%) recipients with infectious complications experienced pericardial tuberculosis, urinary tract infection, or invasive pulmonary aspergillosis. Two (18%) cases of posttransplantation lymphoproliferative disease were diagnosed in this study during the follow-up. Overall patient and graft survival rates were both 91%. Conclusion: This study demonstrated that preconditioning with antibody-depletion using alemtuzumab was efficient with satisfactory patient and graft survivals at 1 year. Alemtuzumab induction was safe even for recipients of extended criteria donor renal transplantation. © 2011 by Elsevier Inc. All rights reserved.

Institution

(Shin, Song, Kim, Kwon, Joh, Lee, Kim) Department of Surgery, Samsung Medical Center, Sungkyunkwan University School of Medicine, #50 Ilwon-dong, Kangnam-ku, 135-710, Seoul, South Korea

Publisher

Elsevier USA (6277 Sea Harbor Drive, Orlando FL 32862 8239, United States)

Emtree Heading

acute graft rejection; adult; article; bacterial pneumonia/si [Side Effect]; clinical article; clinical trial; colitis/si [Side Effect]; creatinine blood level; delayed graft function; demography; drug substitution; drug withdrawal; female; follow up; graft recipient; graft survival; HLA matching; human; immunosuppressive treatment; kidney function; kidney pancreas transplantation; *kidney transplantation; leukopenia/si [Side Effect]; liver failure/si [Side Effect]; low drug dose; lung aspergillosis/si [Side Effect]; lymphocele/co [Complication]; lymphocyte count; lymphocytopenia/si [Side Effect]; lymphoproliferative disease/co [Complication]; maintenance therapy; male; neutropenia/si [Side Effect]; peripheral lymphocyte; postoperative care; postoperative hemorrhage/co [Complication]; postoperative infection/co [Complication]; priority journal; surgical wound/co [Complication]; treatment outcome; tuberculous pericarditis/si [Side Effect]; ureter obstruction/co [Complication]; urinary tract infection/si [Side Effect]; *alemtuzumab/ae [Adverse Drug Reaction]; *alemtuzumab/ct [Clinical Trial]; *alemtuzumab/iv [Intravenous Drug Administration]; calcineurin inhibitor; creatinine/ec [Endogenous Compound]; cyclosporin A/cb [Drug Combination]; cyclosporin A/po [Oral Drug Administration]; methylprednisolone/cb [Drug Combination]; methylprednisolone/iv [Intravenous Drug Administration]; methylprednisolone/po [Oral Drug Administration]; mycophenolic acid; mycophenolic acid 2 morpholinoethyl ester/cb [Drug Combination]; tacrobell; tacrolimus/cb [Drug Combination]; unclassified drug.

Drug Trade Names and Manufacturers

chongkundang, Hoffmann La Roche [Switzerland], Novartis [Switzerland], cellcept: Hoffmann La Roche [Switzerland], myfortic: Novartis, neoral: Novartis [Switzerland], tacrobell: chongkundang

Link to the Ovid Full Text or citation:

Click here for full text options

62.

Teplizumab for treatment of type 1 diabetes (Protege study): 1-year results from a randomised, placebo-controlled trial.

Sherry N., Hagopian W., Ludvigsson J., Jain S.M., Wahlen J., Ferry Jr. R.J., Bode B., Aronoff S., Holland C., Carlin D., King K.L., Wilder R.L., Pillemer S., Bonvini E., Johnson S., Stein K.E., Koenig S., Herold K.C., Daifotis A.G.

The Lancet. 378 (9790) (pp 487-497), 2011. Date of Publication: August 6-12, 2011.

AN: 2011436035

Findings of small studies have suggested that short treatments with anti-CD3 monoclonal antibodies that are mutated to reduce Fc receptor binding preserve beta-cell function and decrease insulin needs in patients with recent-onset type 1 diabetes. In this phase 3 trial, we assessed the safety and efficacy of one such antibody, teplizumab. In this 2-year trial, patients aged 8-35 years who had been diagnosed with type 1 diabetes for 12 weeks or fewer were enrolled and treated at 83 clinical centres in North America, Europe, Israel, and India. Participants were allocated (2:1:1:1 ratio) by an interactive telephone system, according to computer-generated block randomisation, to receive one of three regimens of teplizumab infusions (14-day full dose, 14-day low dose, or 6-day full dose) or placebo at baseline and at 26 weeks. The Protege study is still underway, and patients and study staff remain masked through to study closure. The primary composite outcome was the percentage of patients with insulin use of less than 0.5 U/kg per day and glycated haemoglobin A1c (HbA1C) of less than 6.5 at 1 year. Analyses included all patients who received at least one dose of study drug. This trial is registered with ClinicalTrials.gov, number NCT00385697. 763 patients were screened, of whom 516 were randomised to receive 14-day full-dose teplizumab (n=209), 14-day low-dose teplizumab (n=102), 6-day full-dose teplizumab (n=106), or placebo (n=99). Two patients in the 14-day full-dose group and one patient in the placebo group did not start treatment, so 513 patients were eligible for efficacy analyses. The primary outcome did not differ between groups at 1 year: 19.8 (41/207) in the 14-day full-dose group; 13.7 (14/102) in the 14-day low-dose group; 20.8 (22/106) in the 6-day full-dose group; and 20.4 (20/98) in the placebo group. 5 (19/415) of patients in the teplizumab groups were not taking insulin at 1 year, compared with no patients in the placebo group at 1 year (p=0.03). Across the four study groups, similar proportions of patients had adverse events (414/417 [99] in the teplizumab groups vs 98/99 [99] in the placebo group) and serious adverse events (42/417 [10] vs 9/99 [9]). The most common clinical adverse event in the teplizumab groups was rash (220/417 [53] vs 20/99 [20] in the placebo group). Findings of exploratory analyses suggest that future studies of immunotherapeutic intervention with teplizumab might have increased success in prevention of a decline in beta-cell function (measured by C-peptide) and provision of glycaemic control at reduced doses of insulin if they target patients early after diagnosis of diabetes and children. MacroGenics, the Juvenile Diabetes Research Foundation, and Eli Lilly. © 2011 Elsevier Ltd.

Institution

(Sherry) Massachusetts General Hospital for Children, 175 Cambridge Street, Boston, MA 02114, United States (Hagopian) Pacific Northwest Diabetes Research Institute, Seattle, WA, United States

(Ludvigsson) Department of Clinical and Experimental Medicine, Faculty of Health Sciences, Linkoping University, Linkoping, Sweden

(Jain) TOTALL Diabetes Hormone Research Institute, Indore, Madhya Pradesh, India

(Wahlen) Endocrine Research Specialists, Ogden, UT, United States

(Ferry Jr.) Division of Pediatric Endocrinology and Metabolism, Le Bonheur Children's Hospital, University of Tennessee Health Science Center, Memphis, TN, United States

(Bode) Atlanta Diabetes Associates, Atlanta, GA, United States

(Aronoff) Research Institute of Dallas, Dallas, TX, United States

(Holland, Carlin, King, Bonvini, Johnson, Stein, Koenig, Daifotis) MacroGenics, Rockville, MD, United States

(Wilder) PAREXEL International, Durham, NC, United States

(Pillemer) American Biopharma Corporation, Gaithersburg, MD, United States

(Herold) Yale University, New Haven, CT, United States

Publisher

Elsevier Limited (32 Jamestown Road, London NW1 7BY, United Kingdom)

Emtree Heading

abdominal pain/si [Side Effect]; abscess/si [Side Effect]; adolescent; adult; alkaline phosphatase blood level; anal abscess/si [Side Effect]; anemia/si [Side Effect]; appendicitis/si [Side Effect]; area under the curve; article; bicarbonate blood level; bronchitis/si [Side Effect]; celiac disease/si [Side Effect]; cellulitis/si [Side Effect]; child; chill/si [Side Effect]; controlled study; cornea erosion/si [Side Effect]; cytokine release syndrome/si [Side Effect]; dehydration/si [Side Effect]; depression/si [Side Effect]; diabetes mellitus/si [Side Effect]; diabetic ketoacidosis/si [Side Effect]; dose response; drug dose comparison; drug dose escalation; drug efficacy; drug eruption/si [Side Effect]; drug fever/si [Side Effect]; drug safety; epididymitis/si [Side Effect]; fatigue/si [Side Effect]; female; fever/si [Side Effect]; gastritis/si [Side Effect]; gastroenteritis/si [Side Effect]; headache/si [Side Effect]; hepatobiliary disease/si [Side Effect]; human; hyperglycemia/si [Side Effect]; hyperkalemia/si [Side Effect]; hypersensitivity/si [Side Effect]; hypoalbuminemia/si [Side Effect]; hypocalcemia/si [Side Effect]; hypoglycemic coma/si [Side Effect]; hypoglycemic seizure/si [Side Effect]; hypokalemia/si [Side Effect]; hyponatremia/si [Side Effect]; infection/si [Side Effect]; injury/si [Side Effect]; insulin dependent diabetes mellitus/dt [Drug Therapy]; intestine obstruction/si [Side Effect]; intoxication/si [Side Effect]; leukocyte count; leukopenia/si [Side Effect]; low drug dose; lung tuberculosis/si [Side Effect]; lymphocyte count; lymphocytopenia/si [Side Effect]; major clinical study; male; melanoma/si [Side Effect]; metastasis/si [Side Effect]; multicenter study; multiple cycle treatment; musculoskeletal chest pain/si [Side Effect]; musculoskeletal disease/si [Side Effect]; nausea/si [Side Effect]; neutropenia/si [Side Effect]; neutrophil count; oropharyngeal pain/si [Side Effect]; pain/si [Side Effect]; paronychia/si [Side Effect]; phase 3 clinical trial; pneumonia/si [Side Effect]; priority journal; proteinuria/si [Side Effect]; pruritus/si [Side Effect]; randomized controlled trial; rash/si [Side Effect]; rhinopharyngitis/si [Side Effect]; school child; screening test; seizure/si [Side Effect]; side effect/si [Side Effect]; sodium blood level; thorax pain/si [Side Effect]; thrombocyte count; thrombocytopenia/si [Side Effect]; treatment outcome; tuberculosis/si [Side Effect]; unconsciousness/si [Side Effect]; upper extremity deep vein thrombosis/si [Side Effect]; upper respiratory tract infection/si [Side Effect]; viral gastritis/si [Side Effect]; viral gastroenteritis/si [Side Effect]; vomiting/si [Side Effect]; hemoglobin A1c/ec [Endogenous Compound]; insulin/dt [Drug Therapy]; placebo; *teplizumab/ae [Adverse Drug Reaction]; *teplizumab/ct [Clinical Trial]; *teplizumab/do [Drug Dose]; *teplizumab/dt [Drug Therapy]; *teplizumab/iv [Intravenous Drug Administration].

Link to the Ovid Full Text or citation:

Click here for full text options

63.

Intralesional infliximab in noninfectious cutaneous granulomas: Three cases of necrobiosis lipoidica.

Barde C., Laffitte E., Campanelli A., Saurat J.-H., Thielen A.-M.

Dermatology. 222 (3) (pp 213-216), 2011. Date of Publication: June 2011.

AN: 2011367730

Background: Necrobiosis lipoidica is a rare granulomatous noninfectious skin disease. Treatment of this chronic debilitating disease is of importance because ulceration of the plaques may induce important psychological and physical morbidity. Objective: Infliximab, an anti-TNF- alpha chimeric monoclonal antibody used intravenously and intralesionally for other extradermatological granulomatous diseases including Crohn's disease and sarcoidosis, was administered by intradermal injection in necrobiosis lipoidica. The aim of this study was to evaluate the efficacy and safety profile of a locally delivered drug compared to its systemic use. Patients and Methods: Weekly injections of intralesional infliximab for 3 weeks were followed by a 1-week treatment interruption. This treatment schedule was repeated thrice. Results: Two patients who benefitted from complete treatment experienced almost complete remission for up to 18 months. The third patient, who had treatment interruptions, showed partial improvement. No serious side effects were noticed, although the injections caused pain. Conclusions: This is the first report about the ef-ficacy and safety of a therapy consisting of intralesional injections of infliximab for a granulomatous skin disease. Although this approach was clearly effective for necrobiosis lipoidica, the disease recurred several months after treatment interruption, raising the question of the need for maintenance therapy. Further controlled long-term trials are thus necessary. © 2011 S. Karger AG, Basel.

Institution

(Barde, Laffitte, Campanelli, Thielen) Department of Clinical Specialities, Dermatology, University Hospital of Geneva, Switzerland (Saurat) Swiss Center for Applied Human Toxicology, University Medical Center, Geneva, Switzerland

(Barde) Hopital Universitaire de Geneve, 4, rue Perret-Gentil, CH-1211 Geneva 14, Switzerland

Publisher

S. Karger AG (Allschwilerstrasse 10, P.O. Box, Basel CH-4009, Switzerland)

Emtree Heading

adult; aged; allograft; article; autograft; case report; debridement; drug efficacy; drug safety; fatigue/si [Side Effect]; female; human; injection site pain/si [Side Effect]; insulin dependent diabetes mellitus; lymphatic drainage; miliary tuberculosis/si [Side Effect]; multiple cycle treatment; *necrobiosis lipoidica/di [Diagnosis]; *necrobiosis lipoidica/dt [Drug Therapy]; *necrobiosis lipoidica/su [Surgery]; *necrobiosis lipoidica/th [Therapy]; priority journal; PUVA; remission; skin graft; streptococcal pharyngitis/dt [Drug Therapy]; treatment duration; treatment failure; treatment outcome; treatment response; wound care; adalimumab/dt [Drug Therapy]; adalimumab/sc [Subcutaneous Drug Administration]; antibiotic agent/dt [Drug Therapy]; antibiotic agent/iv [Intravenous Drug Administration]; antibiotic agent/po [Oral Drug Administration]; calcineurin inhibitor/dt [Drug Therapy]; corticosteroid/dt [Drug Therapy]; corticosteroid/il [Intralesional Drug Administration]; corticosteroid/tp [Topical Drug Administration]; cyclosporin/dt [Drug Therapy]; dapsone/dt [Drug Therapy]; etanercept/ae [Adverse Drug Reaction]; etanercept/dt [Drug Therapy]; etanercept/il [Intralesional Drug Administration]; etanercept/sc [Subcutaneous Drug Administration]; hydroxychloroquine/dt [Drug Therapy]; *infliximab/ae [Adverse Drug Reaction]; *infliximab/dt [Drug Therapy]; *infliximab/il [Intralesional Drug Administration]; *infliximab/iv [Intravenous Drug Administration]; penicillin G/dt [Drug Therapy]; penicillin G/po [Oral Drug Administration]; pentoxifylline/dt [Drug Therapy]; prednisone/dt [Drug Therapy]; prednisone/po [Oral Drug Administration]; retinoid/dt [Drug Therapy]; steroid/dt [Drug Therapy]; steroid/il [Intralesional Drug Administration]; steroid/tp [Topical Drug Administration]; tacrolimus/dt [Drug Therapy].

Link to the Ovid Full Text or citation:

Click here for full text options

64.

Adding saxagliptin to extended-release metformin vs. uptitrating metformin dosage.

Fonseca V., Zhu T., Karyekar C., Hirshberg B.

Diabetes, Obesity and Metabolism. 14 (4) (pp 365-371), 2012. Date of Publication: April 2012.

AN: 2012141595

Aim: To investigate whether patients taking metformin for type 2 diabetes mellitus (T2DM) have improved glycaemic control without compromising tolerability by adding an agent with a complementary mechanism of action vs. uptitrating metformin. Methods: Adults with T2DM and glycated haemoglobin (HbA1c) between 7.0 and 10.5% receiving metformin extended release (XR) 1500 mg/day for >8 weeks were randomized to receive saxagliptin 5 mg added to metformin XR 1500 mg (n = 138) or metformin XR uptitrated to 2000 mg/day (n = 144). Endpoints were change from baseline to week 18 in HbA1c (primary), 120-min postprandial glucose (PPG), fasting plasma glucose (FPG) and the proportion of patients achieving HbA1c <7%. Results: At week 18, the adjusted mean reduction from baseline HbA1c was -0.88% for saxagliptin + metformin XR and -0.35% for uptitrated metformin XR (difference, -0.52%; p < 0.0001). For 120-min PPG and FPG, differences in adjusted mean change from baseline between saxagliptin + metformin XR and uptitrated metformin XR were -1.3 mmol/l (-23.32 mg/dl) (p = 0.0013) and -0.73 mmol/l (-13.18 mg/dl) (p = 0.0030), respectively. More patients achieved HbA1c <7.0% with saxagliptin + metformin XR than with uptitrated metformin XR (37.2 vs. 26.1%; p = 0.0459). The proportions of patients experiencing any adverse events (AEs) were generally similar between groups; neither group showed any notable difference in hypoglycaemia or gastrointestinal AEs. Conclusion: Adding saxagliptin to metformin XR provided superior glycaemic control compared with uptitrating metformin XR without the emergence of additional safety concerns (Clinical Trials.gov registration: NCT00960076). © 2011 Blackwell Publishing Ltd.

Institution

(Fonseca) Department of Medicine, Tulane University Health Sciences Center, New Orleans, LA, United States (Zhu, Hirshberg) Clinical Development, AstraZeneca LP, Wilmington, DE, United States

(Karyekar) Global Clinical Research, Bristol-Myers Squibb, Princeton, NJ, United States

Publisher

Blackwell Publishing Ltd (9600 Garsington Road, Oxford OX4 2XG, United Kingdom)

Emtree Heading

abdominal pain/si [Side Effect]; add on therapy; adult; arteriosclerosis/si [Side Effect]; article; combination chemotherapy; comparative effectiveness; controlled study; coronary artery atherosclerosis/si [Side Effect]; diabetic patient; diarrhea/si [Side Effect]; double blind procedure; drug dose titration; drug efficacy; drug formulation; drug safety; drug tolerability; drug withdrawal; duodenum perforation/si [Side Effect]; female; gastrointestinal symptom/si [Side Effect]; glucose blood level; glycemic control; heart infarction/si [Side Effect]; hemoglobin blood level; human; hyperglycemia/si [Side Effect]; hypoglycemia/si [Side Effect]; insomnia/si [Side Effect]; lung tuberculosis/si [Side Effect]; male; monotherapy; multicenter study; nausea/si [Side Effect]; nephrotic syndrome/si [Side Effect]; *non insulin dependent diabetes mellitus/dt [Drug Therapy]; phase 3 clinical trial; randomized controlled trial; rhinopharyngitis/si [Side Effect]; treatment outcome; urinary tract infection/si [Side Effect]; glucose/ec [Endogenous Compound]; hemoglobin A1c/ec [Endogenous Compound]; *metformin/ae [Adverse Drug Reaction]; *metformin/ct [Clinical Trial]; *metformin/cb [Drug Combination]; *metformin/cm [Drug Comparison]; *metformin/dt [Drug Therapy]; *metformin/pr [Pharmaceutics]; *saxagliptin/ae [Adverse Drug Reaction]; *saxagliptin/ct [Clinical Trial]; *saxagliptin/cb [Drug Combination]; *saxagliptin/cm [Drug Comparison]; *saxagliptin/dt [Drug Therapy].

Drug Trade Names and Manufacturers

Bristol Myers Squibb [United States], glucophage: Bristol Myers Squibb [United States], onglyza: Bristol Myers Squibb [United States]

Link to the Ovid Full Text or citation:

Click here for full text options

65.

Geographic differences in time to culture conversion in liquid media: Tuberculosis trials Consortium Study 28. Culture conversion is delayed in Africa.

Mac Kenzie W.R., Heilig C.M., Bozeman L., Johnson J.L., Muzanye G., Dunbar D., Jost Jr. K.C., Diem L., Metchock B., Eisenach K., Dorman S., Goldberg S.

PLoS ONE. 6 (4) (no pagination), 2011. Article Number: e18358. Date of Publication: 2011.

AN: 2011208234

Background: Tuberculosis Trials Consortium Study 28, was a double blind, randomized, placebo-controlled, phase 2 clinical trial examining smear positive pulmonary Mycobacterium tuberculosis. Over the course of intensive phase therapy, patients from African sites had substantially delayed and lower rates of culture conversion to negative in liquid media compared to non-African patients. We explored potential explanations of this finding. Methods: In TBTC Study 28, protocol-correct patients (n = 328) provided spot sputum specimens for M. tuberculosis culture in liquid media, at baseline and weeks 2, 4, 6 and 8 of study therapy. We compared sputum culture conversion for African and non-African patients stratified by four baseline measures of disease severity: AFB smear quantification, extent of disease on chest radiograph, cavity size and the number of days to detection of M. tuberculosis in liquid media using the Kaplan-Meier product-limit method. We evaluated specimen processing and culture procedures used at 29 study laboratories serving 27 sites. Results: African TB patients had more extensive disease at enrollment than non-African patients. However, African patients with the least disease by the 4 measures of disease severity had conversion rates on liquid media that were substantially lower than conversion rates in non-African patients with the greatest extent of disease. HIV infection, smoking and diabetes did not explain delayed conversion in Africa. Some inter-site variation in laboratory processing and culture procedures within accepted practice for clinical diagnostic laboratories was found. Conclusions: Compared with patients from non-African sites, African patients being treated for TB had delayed sputum culture conversion and lower sputum conversion rates in liquid media that were not explained by baseline severity of disease, HIV status, age, smoking, diabetes or race. Further investigation is warranted into whether modest variation in laboratory processes substantially influences the efficacy outcomes of phase 2 TB treatment trials or if other factors (e.g., nutrition, host response) are involved. Trial Registration: ClinicalTrials.gov NCT00144417.

Institution

(Mac Kenzie, Heilig, Bozeman, Goldberg) Tuberculosis Trials Consortium, Division of Tuberculosis Elimination, Centers for Disease Control, Atlanta, GA, United States (Johnson) Tuberculosis Research Unit, Case Western Reserve University School of Medicine, Cleveland, OH, United States

(Muzanye) Uganda-Case Western Reserve University Research Collaboration, Makerere University, Mulago Hospital, Kampala, Uganda, United States

(Dunbar, Jost Jr.) Mycobacteriology Mycology Laboratory, Texas Department of State Health Services, Austin, TX, United States

(Diem, Metchock) Laboratory Branch, Division of Tuberculosis Elimination, Centers for Disease Control, Atlanta, GA, United States

(Eisenach) University of Arkansas for Medical Sciences, Little Rock, AR, United States

(Dorman) Johns Hopkins University School of Medicine, Baltimore, MD, United States

Publisher

Public Library of Science (185 Berry Street, Suite 1300, San Francisco CA 94107, United States)

Emtree Heading

acid fast bacterium; Africa; article; *bacterium culture; bacterium detection; cigarette smoking; controlled study; diabetes mellitus; disease severity; geography; human; Human immunodeficiency virus infection; laboratory test; liquid culture; major clinical study; Mycobacterium tuberculosis; race difference; sputum culture; sputum smear; thorax radiography; treatment outcome; *tuberculosis/dt [Drug Therapy]; isoniazid/dt [Drug Therapy]; pyrazinamide/dt [Drug Therapy]; quinoline derived antiinfective agent/dt [Drug Therapy]; rifampicin/dt [Drug Therapy].

Link to the Ovid Full Text or citation:

Click here for full text options

66.

Adalimumab for the treatment of moderate to severe hidradenitis suppurativa: A parallel randomized trial.

Kimball A.B., Kerdel F., Adams D., Mrowietz U., Gelfand J.M., Gniadecki R., Prens E.P., Schlessinger J., Zouboulis C.C., van der Zee H.H., Rosenfeld M., Mulani P., Gu Y., Paulson S., Okun M., Jemec G.B.E.

Annals of Internal Medicine. 157 (12) (pp 846-855), 2012. Date of Publication: 18 Dec 2012.

AN: 2012743138

Background: Hidradenitis suppurativa (HS) is a chronic, painful skin disease characterized by abscesses, nodules, and draining fistulas in the axilla and groin of young adults. Objective: To evaluate the efficacy and safety of adalimumab, an anti-tumor necrosis factor-alphaantibody, in patients with moderate to severe HS. Design: Phase 2, parallel, randomized, placebo-controlled trial consisting of a blinded 16-week period (period 1) and an open-label 36-week period (period 2). All study personnel, investigators, and patients remained blinded to treatment group throughout the study. (ClinicalTrials.gov: NCT00918255) Setting: 26 academic and private practice medical centers in the United States and Europe. Patients: 154 adult patients with moderate to severe HS who were unresponsive or intolerant to oral antibiotics. Intervention: Patients were assigned in a 1:1:1 ratio to adalimumab, 40 mg/wk; adalimumab, 40 mg every other week (EOW); or placebo. All patients received adalimumab, 40 mg EOW, at the beginning of period 2 but switched to weekly dosing if the response was suboptimal (HS Physician's Global Assessment [PGA] score of moderate or worse) at weeks 28 or 31. Measurements: The primary outcome measure (clinical response) was the proportion of patients achieving an HS-PGA score of clear, minimal, or mild with at least a 2-grade improvement relative to baseline at week 16. Results: At week 16, 3.9% of placebo patients (2 of 51), 9.6% of EOW patients (5 of 52), and 17.6% of weekly patients (9 of 51) achieved clinical response (EOW vs. placebo strata-adjusted difference, 5.6% [95% CI,- 4.0% to 15.3%]; P = 0.25; weekly vs. placebo strata-adjusted difference, 13.7% [CI, 1.7% to 25.7%]; P = 0.025). Serious adverse event rates were 3.9%, 5.8%, and 7.8% for placebo, EOW, and weekly patients, respectively (EOW vs. placebo difference, 1.8% [CI,- 6.4% to 10.1%]; weekly vs. placebo difference, 3.9% [CI,- 5.2% to 13.0%]). Significantly greater improvements in patient-reported outcomes and pain were seen in the weekly dosing group than in the placebo group. A decrease in response was seen after the switch from weekly to EOW dosing in period 2. Limitations: Weeks 16 to 52 of the study were open-label. The study was not powered to assess the risk for known serious adverse effects of adalimumab, such as tuberculosis, other serious infections, and demyelinating disorders. Conclusion: Adalimumab dosed once per week alleviates moderate to severe HS. Primary Funding Source: Abbott Laboratories. © 2012 American College of Physicians.

Institution

(Kimball) Clinical Unit for Research Trials and Outcomes in Skin, Massachusetts General Hospital, Harvard Medical School, 50 Staniford Street, No. 246, Boston, MA 02114, United States (Kerdel) Florida Academic Dermatology Centers, 1400 NW 12 Avenue, Suite 4, Miami, FL 33136, United States

(Adams) Penn State Milton S. Hershey Medical Center, Department of Dermatology, 500 University Drive, Hershey, PA 17033, United States

(Mrowietz) Department of Dermatology, University Medical Center Schleswig-Holstein, Schittenhelmstrase 7, 24105 Kiel, Germany

(Gelfand) University of Pennsylvania, 1471 Penn Tower, 1 Convention Avenue, Philadelphia, PA 19104, United States

(Gniadecki) Bispebjerg Hospital, Department of Dermatology, Bispebjerg bakke 23, 2400 Copenhagen, Denmark

(Prens) Erasmus Medical Center, Dr. Molewaterplein 50, 3015 GE Rotterdam, Netherlands

(Schlessinger, van der Zee) Skin Specialists P.C., 2802 Oak View Drive, Suite 100, Omaha, NE 68144, United States

(Zouboulis) Departments of Dermatology, Venereology, Allergology, and Immunology, Dessau Medical Center, Auenweg 38, 06847 Dessau, Germany

(Rosenfeld, Mulani, Gu, Paulson, Okun) Abbott Laboratories, 100 Abbott Park Road, Abbott Park, IL 60064, United States

(Jemec) University of Copenhagen, Department of Neurology, Psychiatry, and Sensory Sciences, Roskilde Sygehus, Kogevej 7-13, 4000 Roskilde, Denmark

Publisher

American College of Physicians (190 N. Indenpence Mall West, Philadelphia PA 19106-1572, United States)

Emtree Heading

abscess drainage; adult; anemia/si [Side Effect]; anus fissure/si [Side Effect]; arthralgia/si [Side Effect]; arthritis/si [Side Effect]; article; backache/si [Side Effect]; bacteremia/si [Side Effect]; bacterial infection/si [Side Effect]; cellulitis/si [Side Effect]; coccidioidomycosis/si [Side Effect]; colitis/si [Side Effect]; controlled study; coughing/si [Side Effect]; diabetes mellitus/si [Side Effect]; diarrhea/si [Side Effect]; disease activity; dosage schedule comparison; double blind procedure; drug dose escalation; drug efficacy; drug fatality/si [Side Effect]; drug safety; drug withdrawal; Escherichia coli infection/si [Side Effect]; fatigue/si [Side Effect]; female; folliculitis/si [Side Effect]; gastroenteritis/si [Side Effect]; gastroesophageal reflux/si [Side Effect]; genital tract infection/si [Side Effect]; goiter/si [Side Effect]; headache/si [Side Effect]; hidradenitis/si [Side Effect]; human; hypesthesia/si [Side Effect]; incision; influenza/si [Side Effect]; interstitial lung disease/si [Side Effect]; larynx tumor/si [Side Effect]; major clinical study; male; multicenter study; nausea/si [Side Effect]; noncardiac chest pain/si [Side Effect]; oropharynx pain/si [Side Effect]; outcome assessment; pain/si [Side Effect]; parallel design; paresthesia/si [Side Effect]; penile swelling/si [Side Effect]; phase 2 clinical trial; physician global assessment; pilonidal sinus/si [Side Effect]; priority journal; pruritus/si [Side Effect]; pus/si [Side Effect]; pustular psoriasis/si [Side Effect]; pustular rash/si [Side Effect]; randomized controlled trial; rash/si [Side Effect]; rhinopharyngitis/si [Side Effect]; scoring system; scrotal swelling/si [Side Effect]; sinusitis/si [Side Effect]; *suppurative hidradenitis/dt [Drug Therapy]; *suppurative hidradenitis/si [Side Effect]; *suppurative hidradenitis/su [Surgery]; treatment response; tuberculosis/si [Side Effect]; upper respiratory tract infection/si [Side Effect]; virus infection/si [Side Effect]; vomiting/si [Side Effect]; *adalimumab/ae [Adverse Drug Reaction]; *adalimumab/ct [Clinical Trial]; *adalimumab/do [Drug Dose]; *adalimumab/dt [Drug Therapy]; placebo; steroid/dt [Drug Therapy]; steroid/il [Intralesional Drug Administration].

Link to the Ovid Full Text or citation:

Click here for full text options

67.

The PREDICTIVETM Study: A multinational, prospective observational study to evaluate the safety and efficacy of insulin detemir treatment in patients with type 1 and 2 diabetes-data from the Japan cohort.

Namba M., Kaku K., Yoshioka N., Yamada Y., Watada H., Ueki K., Terauchi Y., Tobe K., Araki E., Shuto Y., Kadowaki T.

Diabetology International. 3 (1) (pp 11-20), 2012. Date of Publication: March 2012.

AN: 2012706617

The PREDICTIVETM Study of Japan was an open-label, non-randomized 52-week observational study to evaluate the safety and efficacy of insulin detemir when used in routine clinical practice. Patients with either type 1 or 2 diabetes in whom insulin detemir was to be prescribed but without a previous history of using insulin detemir were eligible to participate. The primary endpoint was an incidence of serious adverse drug reaction (SADR) for the entire observational period. Data were collected at baseline, 12, 26, and 52 weeks. A total of 3,519 patients were enrolled in 540 institutions between 2008 and 2009. Thirty patients (0. 9%) reported 32 SADRs. The rate of total hypoglycemic episodes was 1. 51 episodes/patient-year in patients with type 1 diabetes and 0. 23 episodes/patient-year in patients with type 2 diabetes. The rate of total hypoglycemic episodes did not increase during the observational period. Significant reductions were observed at 52 weeks in HbA1c levels (0. 51% in type 1 diabetes, P < 0. 001; 0. 80% in type 2 diabetes, P < 0. 001), fasting glucose level (reduction of 22. 1 mg/dl in type 1 diabetes, P < 0. 001; 25. 6 mg/dl in type 2 diabetes, P < 0. 001), and intrapatient fasting glucose variability (coefficient of variance; reduction of 11. 4% in type 1 diabetes, P < 0. 001; 4. 1% for type 2 diabetes, P < 0. 001). Our results from the 52-week follow-up data of 3,345 Japanese patients with diabetes demonstrated that insulin detemir contributed to a lower incidence of SADRs (<1%) and suggested that treatment with detemir could provide better glycemic control without increasing hypoglycemic episodes. © 2011 The Japan Diabetes Society.

Institution

(Namba) Division of Diabetes and Metabolism, Department of Internal Medicine, Hyogo College of Medicine, Mukogawa-cho 1-1, Nishinomiya, Hyogo, 663-8501, Japan (Kaku) Division of Diabetes, Endocrinology and Metabolism, Department of Internal Medicine, Kawasaki Medical School, Kurashiki, Okayama, Japan

(Yoshioka) Department of Division of Diabetes and Metabolism, Sapporo Medical Center NTT East Corporation, Sapporo, Hokkaido, Japan

(Yamada) Department of Endocrinology, Diabetes and Geriatric Medicine, Akita University Graduate School of Medicine, Akita, Japan

(Watada) Division of Metabolism and Endocrinology, Department of Internal Medicine, Juntendo University, Tokyo, Japan

(Ueki, Kadowaki) Department of Metabolic Diseases, Graduate School of Medicine, University of Tokyo, Tokyo, Japan

(Terauchi) Division of Endocrinology and Metabolism, Yokohama City University Graduate School of Medicine, Yokohama, Kanagawa, Japan

(Tobe) Department of Internal Medicine, Faculty of Medicine, University of Toyama, Toyama, Japan

(Araki) Department of Metabolic Medicine, Faculty of Life Sciences, Kumamoto University, Kumamoto, Japan

(Shuto) Safety Management Department, Novo Nordisk Pharma Ltd., Tokyo, Japan

Publisher

Springer Japan (1-11-11 Kudan-kita, Chiyoda-ku, No. 2 Funato Bldg., Tokyo 102-0073, Japan)

Emtree Heading

abdominal pain/si [Side Effect]; acute heart failure/si [Side Effect]; acute heart infarction/si [Side Effect]; acute pancreatitis/si [Side Effect]; acute pyelonephritis/si [Side Effect]; adult; angina pectoris/si [Side Effect]; article; aspiration pneumonia/si [Side Effect]; bile duct carcinoma/si [Side Effect]; bile duct stone/si [Side Effect]; bloating/si [Side Effect]; brain hemorrhage/si [Side Effect]; brain infarction/si [Side Effect]; cardiopulmonary arrest/si [Side Effect]; carotid artery obstruction/si [Side Effect]; carpal tunnel syndrome/si [Side Effect]; cataract/si [Side Effect]; cellulitis/si [Side Effect]; cholangitis/si [Side Effect]; cholecystitis/si [Side Effect]; chronic kidney failure/si [Side Effect]; clinical practice; clinical trial; colon cancer/si [Side Effect]; colon polyp/si [Side Effect]; congestive heart failure/si [Side Effect]; controlled study; decreased appetite/si [Side Effect]; dehydration/si [Side Effect]; diabetic ketoacidosis/si [Side Effect]; diabetic retinopathy/si [Side Effect]; digestive system perforation/si [Side Effect]; disseminated intravascular clotting/si [Side Effect]; dizziness/si [Side Effect]; drug efficacy; drug fatality/si [Side Effect]; drug safety; emphysema/si [Side Effect]; esophagus varices/si [Side Effect]; female; femur neck fracture/si [Side Effect]; follow up; forward heart failure/si [Side Effect]; gastroenteritis/si [Side Effect]; glucagonoma/si [Side Effect]; glucose blood level; glycemic control; heart arrest/si [Side Effect]; heart death/si [Side Effect]; heart failure/si [Side Effect]; heart infarction/si [Side Effect]; heart muscle ischemia/si [Side Effect]; heart ventricle extrasystole/si [Side Effect]; hepatitis B/si [Side Effect]; human; hypoglycemia/si [Side Effect]; hypoglycemic coma/si [Side Effect]; hypophagia/si [Side Effect]; hypotension/si [Side Effect]; ileus/si [Side Effect]; infection/si [Side Effect]; *insulin dependent diabetes mellitus/dt [Drug Therapy]; interstitial lung disease/si [Side Effect]; Japanese; ketoacidosis/si [Side Effect]; kidney carcinoma/si [Side Effect]; kidney failure/si [Side Effect]; liver cancer/si [Side Effect]; liver failure/si [Side Effect]; liver metastasis/si [Side Effect]; major clinical study; male; metastasis/si [Side Effect]; *non insulin dependent diabetes mellitus/dt [Drug Therapy]; observational study; obstructive jaundice/si [Side Effect]; pancreas cancer/si [Side Effect]; patella fracture/si [Side Effect]; peripheral edema/si [Side Effect]; peripheral occlusive artery disease/si [Side Effect]; peritonitis/si [Side Effect]; pneumocephalus/si [Side Effect]; pneumonia/si [Side Effect]; priority journal; prospective study; restenosis/si [Side Effect]; sepsis/si [Side Effect]; sick sinus syndrome/si [Side Effect]; side effect/si [Side Effect]; skin ulcer/si [Side Effect]; spondylitis/si [Side Effect]; spontaneous abortion/si [Side Effect]; stomach cancer/si [Side Effect]; sudden death; thyroid cancer/si [Side Effect]; transitional cell carcinoma/si [Side Effect]; treatment outcome; tuberculous peritonitis/si [Side Effect]; unconsciousness/si [Side Effect]; urethra stricture/si [Side Effect]; urinary tract infection/si [Side Effect]; uterus cancer/si [Side Effect]; vomiting/si [Side Effect]; weight reduction; glucose/ec [Endogenous Compound]; hemoglobin A1c/ec [Endogenous Compound]; *insulin detemir/ae [Adverse Drug Reaction]; *insulin detemir/ct [Clinical Trial]; *insulin detemir/dt [Drug Therapy].

Link to the Ovid Full Text or citation:

Click here for full text options

68.

Belatacept: In adult kidney transplant recipients.

Garnock-Jones K.P.

BioDrugs. 26 (6) (pp 413-424), 2012. Date of Publication: 2012.

AN: 2012635041

Belatacept is a second-generation cytotoxic T-lymphocyte-associated antigen-4-Ig fusion protein, which down-regulates T-cell response, and is used in the prophylaxis of organ rejection in adults receiving a kidney transplant. This article reviews the pharmacologic properties of belatacept and its clinical efficacy and tolerability in kidney transplant recipients.In the well designed, phase III trials BENEFIT and BENEFIT-EXT (in patients receiving kidneys from livingstandard-criteria or extended-criteria donors, respectively), a belatacept-based treatment regimen was noninferior to a cyclosporine (ciclosporin)-based regimen with regard to patient and graft survival and acute graft rejection rate, and was significantly superior to the cyclosporine-based regimen with regard to the rate of renal impairment (in BENEFIT only), at 12 months.Belatacept-based treatment showed long-term efficacy and remained effective after 2, 3, and 4 years with regard to these endpoints.Belatacept was generally well tolerated in patients with kidney transplants from living, standard-criteria, or extended-criteria donors. The most serious adverse events that have been reported with belatacept treatment are post-transplant lymphoproliferative disorder, other malignancies, and serious infections.At month 12, the incidence of new-onset diabetes mellitus after transplant was significantly lower with belatacept than with cyclosporine, and belatacept recipients had significantly lower blood pressure and a significantly smaller increase in certain lipid levels than cyclosporine recipients. Adis © 2012 Springer International Publishing AG. All rights reserved.

Institution

(Garnock-Jones) Adis, 41 Centorian Drive, Private Bag 65901, Mairangi Bay, North Shore 0754, Auckland, New Zealand

Publisher

Adis International Ltd (41 Centorian Drive, Private Bag 65901, Mairangi Bay, Auckland 10 1311, New Zealand)

Emtree Heading

acute graft rejection/co [Complication]; anemia/si [Side Effect]; antihypertensive therapy; area under the curve; article; binding affinity; chemoprophylaxis; cholesterol blood level; chronic allograft nephropathy/co [Complication]; constipation/si [Side Effect]; coughing/si [Side Effect]; creatinine blood level; cryptococcal meningitis/si [Side Effect]; cytomegalovirus infection/si [Side Effect]; diabetes mellitus/si [Side Effect]; diarrhea/si [Side Effect]; drug blood level; drug clearance; drug efficacy; drug fever/si [Side Effect]; drug hypersensitivity/si [Side Effect]; drug induced headache/si [Side Effect]; drug metabolism; drug potency; drug protein binding; drug safety; drug solubility; drug tissue level; drug tolerability; drug withdrawal; dyslipidemia/si [Side Effect]; glomerulus filtration rate; graft recipient; graft survival; graft thrombosis/si [Side Effect]; herpes simplex/si [Side Effect]; human; hyperkalemia/si [Side Effect]; hypertension/dt [Drug Therapy]; hypertension/si [Side Effect]; hypokalemia/si [Side Effect]; immunoregulation; immunosuppressive treatment; induction chemotherapy; infection/si [Side Effect]; kidney disease/si [Side Effect]; kidney graft rejection/dt [Drug Therapy]; kidney graft rejection/pc [Prevention]; *kidney transplantation; leukopenia/si [Side Effect]; lymphocele/si [Side Effect]; maximum plasma concentration; mycosis/si [Side Effect]; nausea/si [Side Effect]; neoplasm/si [Side Effect]; nonhuman; peripheral edema/si [Side Effect]; phase 2 clinical trial (topic); phase 3 clinical trial (topic); Pneumocystis pneumonia/dt [Drug Therapy]; Pneumocystis pneumonia/pc [Prevention]; Polyoma virus nephropathy/si [Side Effect]; posttransplant lymphoproliferative disease/si [Side Effect]; priority journal; progressive multifocal leukoencephalopathy/si [Side Effect]; proteinuria/si [Side Effect]; recommended drug dose; renal graft dysfunction/si [Side Effect]; side effect/si [Side Effect]; single drug dose; skin cancer/si [Side Effect]; skin infection/si [Side Effect]; systematic review; thrombosis/si [Side Effect]; treatment outcome; triacylglycerol blood level; tuberculosis/si [Side Effect]; urinary tract infection/si [Side Effect]; vein thrombosis/si [Side Effect]; virus infection/si [Side Effect]; vomiting/si [Side Effect]; antihypertensive agent/dt [Drug Therapy]; antivirus agent/dt [Drug Therapy]; basiliximab/cb [Drug Combination]; *belatacept/ae [Adverse Drug Reaction]; *belatacept/ct [Clinical Trial]; *belatacept/cb [Drug Combination]; *belatacept/cm [Drug Comparison]; *belatacept/cr [Drug Concentration]; *belatacept/dt [Drug Therapy]; *belatacept/iv [Intravenous Drug Administration]; *belatacept/pk [Pharmacokinetics]; *belatacept/pd [Pharmacology]; calcineurin inhibitor/cm [Drug Comparison]; calcineurin inhibitor/dt [Drug Therapy]; corticosteroid/cb [Drug Combination]; cyclosporin/ae [Adverse Drug Reaction]; cyclosporin/cm [Drug Comparison]; cyclosporin/dt [Drug Therapy]; cyclosporin/po [Oral Drug Administration]; mycophenolic acid 2 morpholinoethyl ester/cb [Drug Combination]; mycophenolic acid 2 morpholinoethyl ester/dt [Drug Therapy]; placebo.

Drug Trade Names and Manufacturers

nulojix

Link to the Ovid Full Text or citation:

Click here for full text options

69.

The role of diabetes co-morbidity for tuberculosis treatment outcomes: a prospective cohort study from Mwanza, Tanzania.

Faurholt-Jepsen D., Range N., Praygod G., Kidola J., Faurholt-Jepsen M., Aabye M.G., Changalucha J., Christensen D.L., Martinussen T., Krarup H., Witte D.R., Andersen T.B., Friis H.

BMC Infectious Diseases. 12 (no pagination), 2012. Article Number: 165. Date of Publication: 27 Jul 2012.

AN: 2012579890

Background: Due to the association between diabetes and pulmonary tuberculosis (TB), diabetes may threaten the control of TB. In a prospective cohort study nested in a nutrition trial, we investigated the role of diabetes on changes in anthropometry, grip strength, and clinical parameters over a five months follow-up period.Methods: Among pulmonary TB patients with known diabetes status, we assessed anthropometry and clinical parameters (e.g. haemoglobin) at baseline and after two and five months of TB treatment. A linear mixed-effects model (repeated measurements) was used to investigate the role of diabetes during recovery.Results: Of 1205 TB patients, the mean (standard deviation) age was 36.6 (13.0) years, 40.9% were females, 48.9% were HIV co-infected, and 16.3% had diabetes. TB patients with diabetes co-morbidity experienced a lower weight gain at two (1.3 kg, CI95% 0.5; 2.0, p = 0.001) and five months (1.0 kg, CI95% 0.3; 1.7, p = 0.007). Similarly, the increase in the level of haemoglobin was lower among TB patients with diabetes co-morbidity after two (DELTA 0.6 g/dL, CI95% 0.3; 0.9 p < 0.001) and five months (DELTA 0.5 g/dL, CI95% 0.2; 0.9 p = 0.004) of TB treatment, respectively.Conclusion: TB patients initiating TB treatment with diabetes co-morbidity experience delayed recovery of body mass and haemoglobin, which are important for the functional recovery from disease. © 2012 Faurholt-Jepsen et al.; licensee BioMed Central Ltd.

Institution

(Faurholt-Jepsen, Faurholt-Jepsen, Friis) Department of Human Nutrition, University of Copenhagen, Frederiksberg, Denmark (Range) Muhimbili Medical Centre, National Institute of Medical Research, Dar Es Salaam, Tanzania

(Praygod, Kidola, Changalucha) Mwanza Medical Centre, National Institute of Medical Research, Mwanza, Tanzania

(Aabye) Clinical Research Centre, University of Copenhagen, Hvidovre Hospital, Hvidovre, Denmark

(Christensen) Department of International Health, University of Copenhagen, Copenhagen, Denmark

(Christensen, Witte) Steno Diabetes Center, Gentofte, Denmark

(Martinussen) Department of Basic Sciences and Environment, University of Copenhagen, Frederiksberg, Denmark

(Krarup) Department of Clinical Biochemistry, Aalborg University Hospital, Aalborg, Denmark

(Andersen) Department of Infectious Diseases, Odense University Hospital, Odense, Denmark

Publisher

BioMed Central Ltd. (Floor 6, 236 Gray's Inn Road, London WC1X 8HB, United Kingdom)

Emtree Heading

adult; arm circumference; article; body mass; CD4 lymphocyte count; cohort analysis; comorbidity; controlled study; *diabetes mellitus; female; follow up; grip strength; human; leukocyte count; *lung tuberculosis/dt [Drug Therapy]; major clinical study; male; mixed infection; prospective study; skinfold thickness; Tanzania; treatment outcome; waist circumference; weight gain; hemoglobin/ec [Endogenous Compound]; tuberculostatic agent/dt [Drug Therapy].

Link to the Ovid Full Text or citation:

Click here for full text options

70.

The pharmacological treatment of bipolar disorder in primary care.

Malhi G.S., Adams D., Berk M.

Medical Journal of Australia. 193 (SUPPL. 4) (pp S24-S30), 2010. Date of Publication: 16 Aug 2010.

AN: 2012123618

Objective: To provide a practical overview of the pharmacological management of adults with bipolar disorder in primary care and the role of general practitioners in the pharmacotherapy of this complex disorder. Data sources: Published guidelines for the treatment of bipolar disorder, plus Cochrane reviews, meta-analyses, review articles and reports from randomised controlled trials that were published up to May 2009. Study selection: Over 500 articles on the treatment of bipolar disorder were reviewed, with an emphasis on meta-analyses and systematic reviews of randomised controlled trials. Where evidence was more limited, open trials and non-controlled data were also reviewed. Data extraction: Key recommendations relevant to GPs were synthesised and rated according to National Health and Medical Research Council levels of evidence. Data synthesis: Lithium, valproate and atypical antipsychotics are first-line treatment options for acute mania, and monotherapy is ideal if it produces an adequate response. For depressive episodes, recommendations are less definitive and the use of antidepressants is controversial. Most patients require maintenance treatment, during which pharmacotherapy should be used to prevent relapse, and psychological and social interventions should be considered. Conclusions: Bipolar disorder is a lifelong episodic illness that affects 1%-2% of the population, many of whom are principally managed by their GPs. Pharmacological treatment with mood-stabilising agents is the primary form of management, although this is ideally provided in conjunction with psychosocial interventions.

Institution

(Malhi, Adams) CADE Clinic, Department of Psychiatry, Royal North Shore Hospital, Sydney, NSW, Australia (Malhi, Adams) Northern Sydney Central Coast Mental Health Drug and Alcohol Services, Northern Sydney Central Coast Area Health Service, Sydney, NSW, Australia

(Malhi) Discipline of Psychiatry, University of Sydney, Sydney, NSW, Australia

(Berk) Department of Clinical and Biomedical Sciences-Barwon Health, University of Melbourne, Melbourne, VIC, Australia

(Berk) The Geelong Clinic, Healthscope, Geelong, VIC, Australia

(Berk) Mental Health Research Institute, Melbourne, VIC, Australia

(Berk) Orygen Youth Health Research Centre, Melbourne, VIC, Australia

Publisher

Australasian Medical Publishing Co. Ltd (Level 2, 26-32 Pyrmont Bridge Road, Pyrmont NSW 2009, Australia)

Emtree Heading

abdominal cramp/si [Side Effect]; abdominal pain/si [Side Effect]; acne/si [Side Effect]; agitation; agranulocytosis/si [Side Effect]; akathisia/si [Side Effect]; aminotransferase blood level; ammonia blood level; anorexia/si [Side Effect]; anxiety; aplastic anemia/si [Side Effect]; appetite; arthralgia/si [Side Effect]; article; asthenia/si [Side Effect]; ataxia/si [Side Effect]; *bipolar depression/dt [Drug Therapy]; *bipolar mania/dt [Drug Therapy]; blood dyscrasia/si [Side Effect]; blurred vision/si [Side Effect]; brain disease/si [Side Effect]; cardiomyopathy/si [Side Effect]; concentration loss/si [Side Effect]; confusion/si [Side Effect]; constipation/si [Side Effect]; diarrhea/si [Side Effect]; diplopia/si [Side Effect]; dizziness/si [Side Effect]; drowsiness/si [Side Effect]; drug dose titration; drug efficacy; drug eruption/si [Side Effect]; drug induced headache/si [Side Effect]; dry skin/si [Side Effect]; dyslipidemia/si [Side Effect]; dystonia/si [Side Effect]; electroconvulsive therapy; epigastric discomfort/si [Side Effect]; epistaxis/si [Side Effect]; extrapyramidal symptom/si [Side Effect]; extrapyramidal syndrome/si [Side Effect]; fatigue/si [Side Effect]; galactorrhea/si [Side Effect]; gastrointestinal hemorrhage/si [Side Effect]; gastrointestinal symptom/si [Side Effect]; general practice; general practitioner; hair loss/si [Side Effect]; heart arrest/si [Side Effect]; heart arrhythmia/si [Side Effect]; heart palpitation/si [Side Effect]; hepatitis/si [Side Effect]; human; hyperammonemia/si [Side Effect]; hypercalcemia/si [Side Effect]; hyperglycemia/si [Side Effect]; hypermagnesemia/si [Side Effect]; hyperparathyroidism/si [Side Effect]; hyperprolactinemia/si [Side Effect]; hyperthyroidism/si [Side Effect]; hyponatremia/si [Side Effect]; hypotension/si [Side Effect]; hypothyroidism/si [Side Effect]; inappropriate vasopressin secretion/si [Side Effect]; indigestion/si [Side Effect]; insomnia/si [Side Effect]; irritability; jaundice/si [Side Effect]; leukocytosis/si [Side Effect]; liver dysfunction/si [Side Effect]; liver failure/si [Side Effect]; liver fibrosis/si [Side Effect]; lupus like syndrome/si [Side Effect]; lupus vulgaris/si [Side Effect]; maculopapular rash/si [Side Effect]; memory disorder/si [Side Effect]; mental health care; mental health service; myalgia/si [Side Effect]; myocarditis/si [Side Effect]; nausea/si [Side Effect]; nephrogenic diabetes insipidus/si [Side Effect]; neuroleptic malignant syndrome/si [Side Effect]; orofacial dyskinesia/si [Side Effect]; orthostatic hypotension/si [Side Effect]; pancreatitis/si [Side Effect]; paresthesia/si [Side Effect]; peripheral edema/si [Side Effect]; *primary medical care; psoriasis/si [Side Effect]; *psychopharmacotherapy; QT interval; rhinitis/si [Side Effect]; rigidity; sedation; seizure/si [Side Effect]; sexual dysfunction/si [Side Effect]; side effect/si [Side Effect]; skin bruising/si [Side Effect]; social psychology; somnolence/si [Side Effect]; Stevens Johnson syndrome/si [Side Effect]; stroke/si [Side Effect]; sweating; systematic review; tachycardia/si [Side Effect]; tardive dyskinesia/si [Side Effect]; taste disorder/si [Side Effect]; thrombocyte aggregation; thrombocytopenia/si [Side Effect]; toxic epidermal necrolysis/si [Side Effect]; transient ischemic attack/si [Side Effect]; treatment outcome; tremor/si [Side Effect]; urine retention/si [Side Effect]; vagina bleeding/si [Side Effect]; vertigo/si [Side Effect]; vomiting/si [Side Effect]; weakness/si [Side Effect]; weight gain; weight reduction; xerostomia/si [Side Effect]; atypical antipsychotic agent/ae [Adverse Drug Reaction]; atypical antipsychotic agent/cb [Drug Combination]; atypical antipsychotic agent/dt [Drug Therapy]; carbamazepine/ae [Adverse Drug Reaction]; carbamazepine/dt [Drug Therapy]; clozapine/ae [Adverse Drug Reaction]; clozapine/cb [Drug Combination]; clozapine/dt [Drug Therapy]; fluoxetine/cb [Drug Combination]; fluoxetine/dt [Drug Therapy]; haloperidol/dt [Drug Therapy]; lamotrigine/ae [Adverse Drug Reaction]; lamotrigine/cb [Drug Combination]; lamotrigine/dt [Drug Therapy]; lithium/ae [Adverse Drug Reaction]; lithium/cb [Drug Combination]; lithium/dt [Drug Therapy]; olanzapine/ae [Adverse Drug Reaction]; olanzapine/cb [Drug Combination]; olanzapine/dt [Drug Therapy]; quetiapine/ae [Adverse Drug Reaction]; quetiapine/cb [Drug Combination]; quetiapine/dt [Drug Therapy]; risperidone/ae [Adverse Drug Reaction]; risperidone/cb [Drug Combination]; risperidone/dt [Drug Therapy]; serotonin uptake inhibitor/ae [Adverse Drug Reaction]; serotonin uptake inhibitor/dt [Drug Therapy]; tranquilizer/dt [Drug Therapy]; valproic acid/ae [Adverse Drug Reaction]; valproic acid/cb [Drug Combination]; valproic acid/dt [Drug Therapy]; ziprasidone/ae [Adverse Drug Reaction]; ziprasidone/cb [Drug Combination]; ziprasidone/dt [Drug Therapy].

Link to the Ovid Full Text or citation:

Click here for full text options

71.

How and when to use biologics in psoriasis.

Kircik L.H., Gonzalez M.E.

Journal of Drugs in Dermatology. 9 (8) (pp s106-s117), 2010. Date of Publication: August 2010.

AN: 2011525801

Institution

(Kircik) Mount Sinai Medical Center, New York, NY, United States (Kircik) Indiana University School of Medicine, Indianapolis, IN, United States

(Gonzalez) Ronald O. Perelman Department of Dermatology, New York University School of Medicine, New York, NY, United States

Publisher

Journal of Drugs in Dermatology (377 Park Avenue South, New York NY 10016, United States)

Emtree Heading

alanine aminotransferase blood level; anaphylaxis/si [Side Effect]; ankylosing spondylitis/dt [Drug Therapy]; antibody blood level; antibody production; antibody response; antibody titer; apoptosis; arthralgia; aspartate aminotransferase blood level; *biological therapy; blood cell count; breast cancer; cardiovascular disease/si [Side Effect]; CD4 lymphocyte count; cell proliferation; chest tightness/si [Side Effect]; childhood disease; clinical assessment; conference paper; congestive heart failure; consultation; Crohn disease/dt [Drug Therapy]; cytokine release; diabetes mellitus; disability; disease course; disease exacerbation/si [Side Effect]; disease severity; drug approval; drug blood level; drug clearance; drug efficacy; drug indication; drug mechanism; drug safety; dyspnea/si [Side Effect]; family history; fatigue/si [Side Effect]; flushing; follow up; headache/si [Side Effect]; hepatitis B; hospitalization; human; Human immunodeficiency virus infection; hypertension/si [Side Effect]; hypotension/si [Side Effect]; infection/si [Side Effect]; infection risk; infusion; injection site pruritus/si [Side Effect]; injection site reaction/si [Side Effect]; joint swelling; liver failure; liver function test; liver toxicity/si [Side Effect]; loading drug dose; long term care; lupus erythematosus/si [Side Effect]; lymphocyte count; lymphoproliferative disease; morning stiffness; mortality; multiple sclerosis/si [Side Effect]; Mycobacterium tuberculosis; nausea/si [Side Effect]; neurologic disease; non melanoma skin cancer; opportunistic infection; outcome assessment; pathophysiology; patient monitoring; phototherapy; plaque type psoriasis/dt [Drug Therapy]; posterior reversible encephalopathy syndrome/si [Side Effect]; postmarketing surveillance; practice guideline; pregnancy; *psoriasis/dt [Drug Therapy]; psoriatic arthritis/dt [Drug Therapy]; PUVA; quality of life; randomized controlled trial (topic); receptor binding; recurrence risk; rheumatoid arthritis/dt [Drug Therapy]; rhinopharyngitis/si [Side Effect]; risk assessment; risk benefit analysis; screening; serodiagnosis; serology; side effect/si [Side Effect]; skin cancer; skin examination; sweating; T lymphocyte activation; Th1 cell; Th17 cell; treatment duration; treatment indication; treatment response; tuberculin test; tuberculosis/si [Side Effect]; ulcerative colitis/dt [Drug Therapy]; unspecified side effect/si [Side Effect]; upper respiratory tract infection/si [Side Effect]; vaccination; virus reactivation; X ray; *adalimumab/ae [Adverse Drug Reaction]; *adalimumab/ct [Clinical Trial]; *adalimumab/do [Drug Dose]; *adalimumab/dt [Drug Therapy]; *adalimumab/pd [Pharmacology]; *adalimumab/sc [Subcutaneous Drug Administration]; alanine aminotransferase/ec [Endogenous Compound]; *alefacept/ae [Adverse Drug Reaction]; *alefacept/cb [Drug Combination]; *alefacept/dt [Drug Therapy]; *alefacept/im [Intramuscular Drug Administration]; *alefacept/pd [Pharmacology]; antihistaminic agent; aspartate aminotransferase/ec [Endogenous Compound]; briakinumab/ae [Adverse Drug Reaction]; briakinumab/ct [Clinical Trial]; briakinumab/dt [Drug Therapy]; CD45RO antigen; drug antibody; *etanercept/ae [Adverse Drug Reaction]; *etanercept/ct [Clinical Trial]; *etanercept/cb [Drug Combination]; *etanercept/cm [Drug Comparison]; *etanercept/dt [Drug Therapy]; *etanercept/pd [Pharmacology]; *etanercept/sc [Subcutaneous Drug Administration]; etretin/cb [Drug Combination]; etretin/dt [Drug Therapy]; hepatitis B core antibody/ec [Endogenous Compound]; immunoglobulin enhancer binding protein; *infliximab/ae [Adverse Drug Reaction]; *infliximab/ct [Clinical Trial]; *infliximab/dt [Drug Therapy]; *infliximab/iv [Intravenous Drug Administration]; *infliximab/pd [Pharmacology]; interleukin 12/ec [Endogenous Compound]; interleukin 17/ec [Endogenous Compound]; interleukin 23/ec [Endogenous Compound]; live vaccine; methotrexate/cb [Drug Combination]; methotrexate/dt [Drug Therapy]; neutralizing antibody; paracetamol; placebo; protein p55/ec [Endogenous Compound]; protein p75/ec [Endogenous Compound]; remicaide; tumor necrosis factor alpha/ec [Endogenous Compound]; tumor necrosis factor alpha receptor/ec [Endogenous Compound]; unclassified drug; *ustekinumab/ae [Adverse Drug Reaction]; *ustekinumab/ct [Clinical Trial]; *ustekinumab/cm [Drug Comparison]; *ustekinumab/dt [Drug Therapy]; *ustekinumab/pk [Pharmacokinetics]; *ustekinumab/pd [Pharmacology].

Drug Trade Names and Manufacturers

Abbott, Amgen [United States], Biogen, Centocor, abt 874, amevive: Biogen, cnto 1275: Centocor, enbrel: Amgen [United States], humira: Abbott, remicaide: Centocor

Link to the Ovid Full Text or citation:

Click here for full text options

72.

Everolimus in patients with autosomal dominant polycystic kidney disease.

Walz G., Budde K., Mannaa M., Nurnberger J., Wanner C., Sommerer C., Kunzendorf U., Banas B., Horl W.H., Obermuller N., Arns W., Pavenstadt H., Gaedeke J., Buchert M., May C., Gschaidmeier H., Kramer S., Eckardt K.-U.

New England Journal of Medicine. 363 (9) (pp 830-840), 2010. Date of Publication: 26 Aug 2010.

AN: 2010466271

BACKGROUND: Autosomal dominant polycystic kidney disease (ADPKD) is a slowly progressive hereditary disorder that usually leads to end-stage renal disease. Although the underlying gene mutations were identified several years ago, efficacious therapy to curtail cyst growth and prevent renal failure is not available. Experimental and observational studies suggest that the mammalian target of rapamycin (mTOR) pathway plays a critical role in cyst growth. METHODS: In this 2-year, double-blind trial, we randomly assigned 433 patients with ADPKD to receive either placebo or the mTOR inhibitor everolimus. The primary outcome was the change in total kidney volume, as measured on magnetic resonance imaging, at 12 and 24 months. RESULTS: Total kidney volume increased between baseline and 1 year by 102 ml in the everolimus group, versus 157 ml in the placebo group (P = 0.02) and between baseline and 2 years by 230 ml and 301 ml, respectively (P = 0.06). Cyst volume increased by 76 ml in the everolimus group and 98 ml in the placebo group after 1 year (P = 0.27) and by 181 ml and 215 ml, respectively, after 2 years (P = 0.28). Parenchymal volume increased by 26 ml in the everolimus group and 62 ml in the placebo group after 1 year (P = 0.003) and by 56 ml and 93 ml, respectively, after 2 years (P = 0.11). The mean decrement in the estimated glomerular filtration rate after 24 months was 8.9 ml per minute per 1.73 m2 of body-surface area in the everolimus group versus 7.7 ml per minute in the placebo group (P = 0.15). Drug-specific adverse events were more common in the everolimus group; the rate of infection was similar in the two groups. CONCLUSIONS: Within the 2-year study period, as compared with placebo, everolimus slowed the increase in total kidney volume of patients with ADPKD but did not slow the progression of renal impairment. (Funded by Novartis; EudraCT number, 2006-001485-16; ClinicalTrials.gov number, NCT00414440.). Copyright © 2010 Massachusetts Medical Society.

Institution

(Walz, Buchert) Renal Division, University Hospital Freiburg, Hugstetter St., 55, 79106 Freiburg, Germany (Budde, Gaedeke) Charite Universitatsmedizin Berlin Campus Mitte, Berlin, Germany

(Mannaa) Campus Virchow, Berlin, Germany

(Nurnberger) University Hospital of Duisburg-Essen, Essen, Germany

(Wanner) University Hospital, Wurzburg, Germany

(Sommerer) University Hospital, Heidelberg, Germany

(Kunzendorf) University Hospital, Kiel, Germany

(Banas) University Hospital, Regensburg, Germany

(Obermuller) University Hospital, Frankfurt, Germany

(Arns) Merheim Medical Center, Cologne, Germany

(Pavenstadt) University Hospital, Munster, Germany

(May, Gschaidmeier, Kramer) Novartis Germany, Nuremberg, Germany

(Eckardt) University of Erlangen, Community Hospital Nurnberg, Erlangen, Germany

(Horl) Medical University of Vienna, Vienna, Austria

Publisher

Massachussetts Medical Society (860 Winter Street, Waltham MA 02451-1413, United States)

Emtree Heading

abdominal pain/si [Side Effect]; acne/si [Side Effect]; adult; anemia/si [Side Effect]; angioneurotic edema/si [Side Effect]; arthralgia/si [Side Effect]; article; autosomal dominant disorder; bacterial gene; benign tumor/si [Side Effect]; body surface; body weight; bronchitis/si [Side Effect]; cholesterol blood level; clinical trial; controlled clinical trial; controlled study; diabetes mellitus/si [Side Effect]; diarrhea/si [Side Effect]; double blind procedure; epistaxis/si [Side Effect]; female; flank pain/si [Side Effect]; folliculitis/si [Side Effect]; gastritis/si [Side Effect]; glomerulus filtration rate; growth; herpes zoster/si [Side Effect]; human; hypercholesterolemia/si [Side Effect]; hyperlipidemia/si [Side Effect]; hypertriglyceridemia/si [Side Effect]; infection rate; kidney parenchyma; *kidney polycystic disease/dt [Drug Therapy]; kidney size; leukopenia/si [Side Effect]; major clinical study; male; malignant neoplastic disease/si [Side Effect]; mammal; mouth ulcer/si [Side Effect]; multicenter study; myalgia/si [Side Effect]; myositis/si [Side Effect]; nausea/si [Side Effect]; nuclear magnetic resonance imaging; observational study; outcome assessment; ovary cyst/si [Side Effect]; peripheral edema/si [Side Effect]; pneumonia/si [Side Effect]; priority journal; randomized controlled trial; rhinopharyngitis/si [Side Effect]; side effect/si [Side Effect]; sinusitis/si [Side Effect]; stomatitis/si [Side Effect]; systolic blood pressure; thrombocytopenia/si [Side Effect]; triacylglycerol blood level; tuberculosis/si [Side Effect]; urinary tract infection/si [Side Effect]; vomiting/si [Side Effect]; *everolimus/ae [Adverse Drug Reaction]; *everolimus/ct [Clinical Trial]; *everolimus/dt [Drug Therapy]; placebo.

Drug Trade Names and Manufacturers

Novartis, certican: Novartis

Link to the Ovid Full Text or citation:

Click here for full text options

73.

Opioid pharmacotherapy for chronic non-cancer pain in the United States: A research guideline for developing an evidence-base.

Chapman C.R., Lipschitz D.L., Angst M.S., Chou R., Denisco R.C., Donaldson G.W., Fine P.G., Foley K.M., Gallagher R.M., Gilson A.M., Haddox J.D., Horn S.D., Inturrisi C.E., Jick S.S., Lipman A.G., Loeser J.D., Noble M., Porter L., Rowbotham M.C., Schoelles K.M., Turk D.C., Volinn E., Von Korff M.R., Webster L.R., Weisner C.M.

Journal of Pain. 11 (9) (pp 807-829), 2010. Date of Publication: September 2010.

AN: 2010471692

This document reports the consensus of an interdisciplinary panel of research and clinical experts charged with reviewing the use of opioids for chronic noncancer pain (CNCP) and formulating guidelines for future research. Prescribing opioids for chronic noncancer pain has recently escalated in the United States. Contrasting with increasing opioid use are: 1) The lack of evidence supporting long-term effectiveness; 2) Escalating misuse of prescription opioids including abuse and diversion; and 3) Uncertainty about the incidence and clinical salience of multiple, poorly characterized adverse drug events (ADEs) including endocrine dysfunction, immunosuppression and infectious disease, opioid-induced hyperalgesia and xerostomia, overdose, falls and fractures, and psychosocial complications. Chief among the limitations of current evidence are: 1) Sparse evidence on long-term opioid effectiveness in chronic pain patients due to the short-term time frame of clinical trials; 2) Insufficiently comprehensive outcome assessment; and 3) Incomplete identification and quantification of ADEs. The panel called for a strategic interdisciplinary approach to the problem domain in which basic scientists and clinicians cooperate to resolve urgent issues and generate a comprehensive evidence base. It offered 4 recommendations in 3 areas: 1) A research strategy for studying the effectiveness of long-term opioid pharmacotherapy; 2) Improvements in evidence-generation methodology; and 3) Potential research topics for generating new evidence. Perspective: Prescribing opioids for CNCP has outpaced the growth of scientific evidence bearing on the benefits and harms of these interventions. The need for a strong evidence base is urgent. This guideline offers a strategic approach to creating a comprehensive evidence base to guide safe and effective management of CNCP. © 2010 by the American Pain Society.

Institution

(Chapman, Lipschitz, Donaldson, Fine, Volinn) Department of Anesthesiology, University of Utah School of Medicine, Salt Lake City, UT, United States (Angst) Department of Anesthesiology, Stanford University, Palo Alto, CA, United States

(Chou) Internal Medicine and Geriatrics, School of Medicine, Oregon Health and Science University, Portland, OR, United States

(Denisco) National Institute on Drug Abuse, Division of Epidemiology, Services and Prevention Research, NIH, Bethesda, MD, United States

(Foley, Inturrisi) Pain and Palliative Care Service, Memorial Sloan Kettering Cancer Center, New York, NY, United States

(Gallagher) Departments of Psychiatry and Anesthesiology and Critical Care, Penn Pain Medicine Center, University of Pennsylvania School of Medicine, Philadelphia, PA, United States

(Gilson) University of Wisconsin School of Medicine and Public Health, Madison, WI, United States

(Inturrisi) Department of Pharmacology, Joan and Sanford I. Weill Medical School, Cornell University, New York, New York, United States

(Haddox) Risk Management and Health Policy, Purdue Pharma, Stamford, CA, United States

(Jick) Department of Epidemiology, Boston University, Boston, MA, United States

(Horn) Department of Medical Informatics, Institute for Clinical Outcomes Research (ICOR), University of Utah School of Medicine, Salt Lake City, UT, United States

(Lipman) Department of Pharmacotherapy, L.S. Skaggs Pharmacy, University of Utah, Salt Lake City, UT, United States

(Loeser) Department of Neurological Surgery, University of Washington, Seattle, WA, United States

(Porter) National Institute of Neurological Disorders, Stroke Systems and Cognitive Neuroscience, National Institutes of Health, Bethesda, MD, United States

(Rowbotham) Pain Clinical Research Center, University of California San Francisco, San Francisco, CA, United States

(Noble, Schoelles) ECRI Institute, Plymouth Meeting, PA, United States

(Turk) Department of Anesthesiology, University of Washington, Seattle, WA, United States

(Von Korff) Center for Health Studies Group Health Cooperative, Seattle, WA, United States

(Webster) Lifetree Pain Clinic, Salt Lake City, UT, United States

(Weisner) Department of Psychiatry, University of California San Francisco, San Francisco, CA, United States

Publisher

Churchill Livingstone Inc. (650 Avenue of the Americas, New York NY 10011, United States)

Emtree Heading

abdominal cramp/si [Side Effect]; bloating/si [Side Effect]; cardiotoxicity/si [Side Effect]; *chronic pain/dt [Drug Therapy]; *chronic pain/th [Therapy]; clinical trial; cognitive defect/si [Side Effect]; comorbidity; constipation/si [Side Effect]; cross tolerance; dental caries/si [Side Effect]; depression/si [Side Effect]; diabetes mellitus/si [Side Effect]; drug abuse; drug efficacy; drug misuse; drug overdose; drug safety; drug tolerability; drug treatment failure; drug withdrawal; endocrine disease/si [Side Effect]; evidence based medicine; falling; fracture/si [Side Effect]; growth hormone deficiency/si [Side Effect]; health care policy; heart arrhythmia/si [Side Effect]; human; hyperalgesia/si [Side Effect]; immune deficiency/si [Side Effect]; incidence; infection/si [Side Effect]; infection sensitivity; intestine function disorder/si [Side Effect]; long term care; low back pain/dt [Drug Therapy]; lung tuberculosis/si [Side Effect]; medical research; nausea/si [Side Effect]; nonhuman; opiate addiction/si [Side Effect]; osteoporosis/si [Side Effect]; patient compliance; periodontal disease/si [Side Effect]; practice guideline; prescription; psychosocial environment; quantitative analysis; review; risk benefit analysis; sedation; side effect/si [Side Effect]; sleep disordered breathing/si [Side Effect]; socioeconomics; sudden death; treatment duration; treatment indication; treatment outcome; United States; vomiting/si [Side Effect]; xerostomia/si [Side Effect]; anticonvulsive agent/dt [Drug Therapy]; codeine/cb [Drug Combination]; codeine/dt [Drug Therapy]; fentanyl/dt [Drug Therapy]; hydrocodone/dt [Drug Therapy]; hydrocodone bitartrate plus paracetamol/dt [Drug Therapy]; hydromorphone/dt [Drug Therapy]; levorphanol/dt [Drug Therapy]; methadone/ae [Adverse Drug Reaction]; methadone/dt [Drug Therapy]; morphine/dt [Drug Therapy]; omega conotoxin MVIIA/dt [Drug Therapy]; omega conotoxin MVIIA/tl [Intrathecal Drug Administration]; *opiate/ae [Adverse Drug Reaction]; *opiate/ct [Clinical Trial]; *opiate/do [Drug Dose]; *opiate/dt [Drug Therapy]; *opiate/tl [Intrathecal Drug Administration]; *opiate/po [Oral Drug Administration]; *opiate/td [Transdermal Drug Administration]; oxycodone/dt [Drug Therapy]; oxymorphone/dt [Drug Therapy]; paracetamol/cb [Drug Combination]; paracetamol/dt [Drug Therapy]; pethidine/dt [Drug Therapy]; psychotropic agent/dt [Drug Therapy]; tricyclic antidepressant agent/dt [Drug Therapy].

Link to the Ovid Full Text or citation:

Click here for full text options

74.

Thai national guidelines for antiretroviral therapy in HIV-1 infected adults and adolescents 2010.

Sungkanuparph S., Techasathit W., Utaipiboon C., Chasombat S., Bhakeecheep S., Leechawengwongs M., Ruxrungtham K., Phanuphak P.

Asian Biomedicine. 4 (4) (pp 515-528), 2010. Date of Publication: August 2010.

AN: 2010572264

In Thailand, more than 150,000 patients are currently treated with antiretroviral drugs under the support of the National AIDS Program (NAP). The appointed Adults and Adolescents Committee consisted of 28 members who are experts in HIV research, patient care or health care policy. Relevant published literature, guidelines, and the most recent relevant clinical trials presented internationally were reviewed. Several peer review and clinical studies conducted in Thailand were included in the review process. Special considerations for patients with co-infection of tuberculosis or hepatitis B were incorporated. Appropriate cut-off of CD4+ T-cell counts when to commence ART among Thai patients have been considered. It is now recommended to start ART at CD4+ T-cell count <350 cells/mm3. For treatment-naive patients, the preferred initial therapy is a nonnucleoside reverse transcriptase inhibitor (NNRTI)-based regimen containing lamivudine plus zidovudine or tenofovir. Stavudine will be phased out in a two-year plan at the national program level. Viral load and CD4+ T-cell counts should be monitored at least once and twice a year. To achieve long-term treatment success, enhancing adherence together with the proper management of antiretroviral-related toxicity is critical. In summary, the major changes from the Thai 2008 guidelines include commencing ART earlier. ART is recommended regardless of CD4+ T cell count if patients have an indication to treat their HBV co-infection. Preferred first regimen uses AZT or TDF, not d4T as the NRTI-backbone. Furthermore, efavirenz is now considered a preferred NNRTI, along with nevirapine.

Institution

(Sungkanuparph) Faculty of Medicine Ramathibodi Hospital, Mahidol University, Bangkok 10400, Thailand (Techasathit) Faculty of Medicine Siriraj Hospital, Mahidol University, Bangkok 10700, Thailand

(Utaipiboon) Global AIDS Program, Thailand MOPH-U.S. CDC Collaboration, Nonthaburi 11000, Thailand

(Chasombat) Bureau of AIDS, TB, and STIs, Department of Disease Control, Ministry of Public Health, Nonthaburi 11000, Thailand

(Bhakeecheep) National Health Security Office, Bangkok 10210, Thailand

(Leechawengwongs) Thai AIDS Society, Bangkok 10310, Thailand

(Ruxrungtham, Phanuphak) Faculty of Medicine, Chulalongkorn University, Bangkok 10330, Thailand

(Ruxrungtham, Phanuphak) HIV-NAT, Thai Red Cross AIDS Research Centre, Bangkok 10330, Thailand

Publisher

IOS Press (Nieuwe Hemweg 6B, Amsterdam 1013 BG, Netherlands)

Emtree Heading

abdominal distension/si [Side Effect]; abdominal pain/si [Side Effect]; abnormal dreaming/si [Side Effect]; aminotransferase blood level; asthenia/si [Side Effect]; atrioventricular block/si [Side Effect]; C banding; CD4 lymphocyte count; clinical trial; confusion/si [Side Effect]; diarrhea/si [Side Effect]; dizziness/si [Side Effect]; drug eruption/si [Side Effect]; drug hypersensitivity/si [Side Effect]; drug induced headache/si [Side Effect]; dyslipidemia/si [Side Effect]; Fanconi renotubular syndrome/si [Side Effect]; health care; hepatitis B; *highly active antiretroviral therapy; human; *Human immunodeficiency virus infection/dt [Drug Therapy]; hyperbilirubinemia/si [Side Effect]; hyperlactatemia/si [Side Effect]; hyperpigmentation/si [Side Effect]; hypertriglyceridemia/si [Side Effect]; insomnia/si [Side Effect]; lactic acidosis/si [Side Effect]; lipoatrophy/si [Side Effect]; lipodystrophy/si [Side Effect]; liver toxicity/si [Side Effect]; lung tuberculosis; macrocytic anemia/si [Side Effect]; mixed infection; nail disease/si [Side Effect]; nausea/si [Side Effect]; nephrogenic diabetes insipidus/si [Side Effect]; nephrolithiasis/si [Side Effect]; nephrotoxicity/si [Side Effect]; neurotoxicity/si [Side Effect]; neutropenia/si [Side Effect]; opportunistic infection; pancreatitis/si [Side Effect]; peripheral neuropathy/si [Side Effect]; PR interval; *practice guideline; pregnant woman; review; side effect/si [Side Effect]; somnolence/si [Side Effect]; Stevens Johnson syndrome/si [Side Effect]; teratogenicity/si [Side Effect]; Thailand; toxic epidermal necrolysis/si [Side Effect]; treatment failure; treatment indication; virus load; vomiting/si [Side Effect]; weakness/si [Side Effect]; abacavir/ae [Adverse Drug Reaction]; abacavir/cb [Drug Combination]; abacavir/dt [Drug Therapy]; atazanavir/ae [Adverse Drug Reaction]; atazanavir/cb [Drug Combination]; atazanavir/dt [Drug Therapy]; darunavir/ae [Adverse Drug Reaction]; didanosine/ae [Adverse Drug Reaction]; didanosine/cb [Drug Combination]; didanosine/dt [Drug Therapy]; efavirenz/ae [Adverse Drug Reaction]; efavirenz/dt [Drug Therapy]; emtricitabine/ae [Adverse Drug Reaction]; etravirine/ae [Adverse Drug Reaction]; indinavir/ae [Adverse Drug Reaction]; indinavir/dt [Drug Therapy]; integrase inhibitor/dt [Drug Therapy]; lamivudine/ae [Adverse Drug Reaction]; lamivudine/cb [Drug Combination]; lamivudine/dt [Drug Therapy]; lopinavir/ae [Adverse Drug Reaction]; lopinavir/cb [Drug Combination]; lopinavir/dt [Drug Therapy]; nevirapine/ae [Adverse Drug Reaction]; nonnucleoside reverse transcriptase inhibitor/dt [Drug Therapy]; proteinase inhibitor/cb [Drug Combination]; proteinase inhibitor/it [Drug Interaction]; proteinase inhibitor/dt [Drug Therapy]; proton pump inhibitor; raltegravir/ae [Adverse Drug Reaction]; raltegravir/dt [Drug Therapy]; rifampicin/it [Drug Interaction]; ritonavir/ae [Adverse Drug Reaction]; ritonavir/cb [Drug Combination]; ritonavir/dt [Drug Therapy]; RNA directed DNA polymerase inhibitor/ct [Clinical Trial]; RNA directed DNA polymerase inhibitor/cb [Drug Combination]; RNA directed DNA polymerase inhibitor/it [Drug Interaction]; RNA directed DNA polymerase inhibitor/dt [Drug Therapy]; saquinavir/ae [Adverse Drug Reaction]; saquinavir/cb [Drug Combination]; saquinavir/dt [Drug Therapy]; stavudine/ae [Adverse Drug Reaction]; stavudine/cb [Drug Combination]; stavudine/dt [Drug Therapy]; tenofovir/ae [Adverse Drug Reaction]; tenofovir/cb [Drug Combination]; tenofovir/dt [Drug Therapy]; zidovudine/ae [Adverse Drug Reaction]; zidovudine/cb [Drug Combination]; zidovudine/dt [Drug Therapy].

Drug Trade Names and Manufacturers

3TC, d4T

Link to the Ovid Full Text or citation:

Click here for full text options

75.

Antiretroviral treatment of adult HIV infection: 2010 recommendations of the International AIDS Society-USA panel.

Thompson M.A., Aberg J.A., Cahn P., Montaner J.S.G., Rizzardini G., Telenti A., Gatell J.M., Gunthard H.F., Hammer S.M., Hirsch M.S., Jacobsen D.M., Reiss P., Richman D.D., Volberding P.A., Yeni P., Schooley R.T.

JAMA - Journal of the American Medical Association. 304 (3) (pp 321-333), 2010. Date of Publication: 21 Jul 2010.

AN: 2010395515

Context: Recent data regarding the consequences of untreated human immunodeficiency virus (HIV) infection and the expansion of treatment choices for antiretroviral-naive and antiretroviral-experienced patients warrant an update of the International AIDS Society-USA guidelines for the use of antiretroviral therapy in adults with HIV infection. Objectives: To provide updated recommendations for management of HIV-infected adults, using antiretroviral drugs and laboratory monitoring tools available in the international, developed-world setting. This report provides guidelines for when to initiate antiretroviral therapy, selection of appropriate initial regimens, patient monitoring, when to change therapy, and what regimens to use when changing. Data Sources and Study Selection: A panel with expertise in HIV research and clinical care reviewed relevant data published or presented at selected scientific conferences since the last panel report through April 2010. Data were identified through a PubMed search, review of scientific conference abstracts, and requests to antiretroviral drug manufacturers for updated clinical trials and adverse event data. Data Extraction and Synthesis: New evidence was reviewed by the panel. Recommendations were drafted by section writing committees and reviewed and edited by the entire panel. The quality and strength of the evidence were rated and recommendations were made by full panel consensus. Conclusions: Patient readiness for treatment should be confirmed before initiation of antiretroviral treatment. Therapy is recommended for asymptomatic patients with a CD4 cell count <500/muL, for all symptomatic patients, and those with specific conditions and comorbidities. Therapy should be considered for asymptomatic patients with CD4 cell count >500/muL. Components of the initial and subsequent regimens must be individualized, particularly in the context of concurrent conditions. Patients receiving antiretroviral treatment should be monitored regularly; treatment failure should be detected and managed early, with the goal of therapy, even in heavily pretreated patients, being HIV-1 RNA suppression below commercially available assay quantification limits. ©2010 American Medical Association. All rights reserved.

Institution

(Thompson) AIDS Research Consortium of Atlanta, Atlanta, GA, United States (Schooley) University of California San Diego, San Diego, CA, United States

(Aberg) New York University School of Medicine, New York, NY, United States

(Cahn) Hospital Juan Fernandez, University of Buenos Aires Medical School, Fundacion Huesped, Argentina

(Gatell) Hospital Clinic-IDIBAPS, University of Barcelona, Barcelona, Spain

(Gunthard) University Hospital Zurich, Division of Infectious Diseases, University of Zurich, Zurich, Switzerland

(Hammer) Columbia University College of Physicians and Surgeons, New York, NY, United States

(Hirsch) Harvard Medical School, Boston, MA, United States

(Jacobsen) International AIDS Society-USA, San Francisco, CA, United States

(Montaner) BC-Centre for Excellence in HIV/AIDS, Providence Health Care, University of British Columbia, Vancouver, BC, Canada

(Reiss) Academic Medical Center, University of Amsterdam, Amsterdam, Netherlands

(Richman) University of California San Diego, Veterans Affairs San Diego Healthcare System, Lausanne, Switzerland

(Rizzardini) Luigi Sacco Hospital, Milan, Italy

(Telenti) University Hospital of Lausanne, Lausanne, Switzerland

(Volberding) University of California San Francisco, San Francisco Veterans Affairs Medical Center, Paris, France

(Yeni) Hopital Bichat-Claude Bernard, Xavier Bichat Medical School, Paris, France

Publisher

American Medical Association (515 North State Street, Chicago IL 60654, United States)

Emtree Heading

*acquired immune deficiency syndrome/dr [Drug Resistance]; *acquired immune deficiency syndrome/dt [Drug Therapy]; *acquired immune deficiency syndrome/ep [Epidemiology]; acute heart infarction/ep [Epidemiology]; adult; African American; aging; aminotransferase blood level; atherosclerosis; bone density; bone marrow toxicity/si [Side Effect]; cardiovascular disease/co [Complication]; cardiovascular disease/ep [Epidemiology]; cardiovascular risk; CD4 lymphocyte count; chronic kidney disease/co [Complication]; chronic kidney disease/ep [Epidemiology]; clinical practice; comorbidity; diabetes mellitus; diarrhea/si [Side Effect]; disease course; dosage schedule comparison; drug efficacy; drug withdrawal; dyslipidemia/si [Side Effect]; gastrointestinal toxicity/si [Side Effect]; glomerulus filtration rate; heart infarction/si [Side Effect]; hepatitis B/dr [Drug Resistance]; hepatitis B/dt [Drug Therapy]; Hepatitis B virus; hepatitis C/dt [Drug Therapy]; Hepatitis C virus; high risk population; *highly active antiretroviral therapy; human; Human immunodeficiency virus 1; *Human immunodeficiency virus 1 infection/dr [Drug Resistance]; *Human immunodeficiency virus 1 infection/dt [Drug Therapy]; *Human immunodeficiency virus 1 infection/ep [Epidemiology]; *Human immunodeficiency virus infection/dr [Drug Resistance]; *Human immunodeficiency virus infection/dt [Drug Therapy]; *Human immunodeficiency virus infection/ep [Epidemiology]; hyperbilirubinemia/si [Side Effect]; hyperlipidemia/co [Complication]; hyperlipidemia/et [Etiology]; hyperlipidemia/si [Side Effect]; hypertension; hypertriglyceridemia/si [Side Effect]; immune reconstitution inflammatory syndrome; insulin resistance/si [Side Effect]; intravenous drug abuse; jaundice/si [Side Effect]; lipodystrophy/co [Complication]; lipodystrophy/et [Etiology]; lipodystrophy/si [Side Effect]; liver disease; liver fibrosis; liver toxicity/co [Complication]; liver toxicity/et [Etiology]; liver toxicity/si [Side Effect]; medical society; mortality; multidrug resistance; nephrolithiasis/co [Complication]; nephrolithiasis/si [Side Effect]; nephrotoxicity/si [Side Effect]; neurotoxicity/si [Side Effect]; nonhuman; opportunistic infection; patient monitoring; practice guideline; pregnant woman; priority journal; QT prolongation/si [Side Effect]; race difference; review; risk reduction; sexual transmission; side effect/si [Side Effect]; treatment failure; treatment indication; tuberculosis; United States; unspecified side effect/si [Side Effect]; vertical transmission; virus hepatitis/dr [Drug Resistance]; virus load; virus reactivation; virus transmission; abacavir/ae [Adverse Drug Reaction]; abacavir/cb [Drug Combination]; abacavir/dt [Drug Therapy]; abacavir plus lamivudine/ae [Adverse Drug Reaction]; abacavir plus lamivudine/cb [Drug Combination]; abacavir plus lamivudine/cm [Drug Comparison]; abacavir plus lamivudine/dt [Drug Therapy]; abacavir plus lamivudine/pr [Pharmaceutics]; *antiretrovirus agent/ae [Adverse Drug Reaction]; *antiretrovirus agent/dt [Drug Therapy]; *antiretrovirus agent/pr [Pharmaceutics]; atazanavir/ae [Adverse Drug Reaction]; atazanavir/cb [Drug Combination]; atazanavir/cm [Drug Comparison]; atazanavir/dt [Drug Therapy]; atazanavir plus ritonavir/ae [Adverse Drug Reaction]; atazanavir plus ritonavir/cm [Drug Comparison]; atazanavir plus ritonavir/do [Drug Dose]; atazanavir plus ritonavir/dt [Drug Therapy]; atazanavir plus ritonavir/pr [Pharmaceutics]; chemokine receptor CCR5; darunavir plus ritonavir/cb [Drug Combination]; darunavir plus ritonavir/cm [Drug Comparison]; darunavir plus ritonavir/dt [Drug Therapy]; darunavir plus ritonavir/pr [Pharmaceutics]; didanosine/ae [Adverse Drug Reaction]; didanosine/dt [Drug Therapy]; efavirenz/ae [Adverse Drug Reaction]; efavirenz/cm [Drug Comparison]; efavirenz/dt [Drug Therapy]; efavirenz/pr [Pharmaceutics]; efavirenz plus emtricitabine plus tenofovir disoproxil/dt [Drug Therapy]; efavirenz plus emtricitabine plus tenofovir disoproxil/pr [Pharmaceutics]; emtricitabine/cb [Drug Combination]; emtricitabine/dt [Drug Therapy]; emtricitabine/pr [Pharmaceutics]; emtricitabine plus tenofovir disoproxil/ae [Adverse Drug Reaction]; emtricitabine plus tenofovir disoproxil/cb [Drug Combination]; emtricitabine plus tenofovir disoproxil/cm [Drug Comparison]; emtricitabine plus tenofovir disoproxil/dt [Drug Therapy]; emtricitabine plus tenofovir disoproxil/pr [Pharmaceutics]; entecavir/dt [Drug Therapy]; fosamprenavir plus ritonavir/ae [Adverse Drug Reaction]; fosamprenavir plus ritonavir/cb [Drug Combination]; fosamprenavir plus ritonavir/cm [Drug Comparison]; fosamprenavir plus ritonavir/do [Drug Dose]; fosamprenavir plus ritonavir/dt [Drug Therapy]; indinavir/ae [Adverse Drug Reaction]; indinavir/dt [Drug Therapy]; indinavir plus ritonavir/ae [Adverse Drug Reaction]; *integrase strand transfer inhibitor/dt [Drug Therapy]; *integrase strand transfer inhibitor/pr [Pharmaceutics]; *integrase strand transfer inhibitor/pd [Pharmacology]; lamivudine/dt [Drug Therapy]; lopinavir plus ritonavir/ae [Adverse Drug Reaction]; lopinavir plus ritonavir/cb [Drug Combination]; lopinavir plus ritonavir/cm [Drug Comparison]; lopinavir plus ritonavir/dt [Drug Therapy]; lopinavir plus ritonavir/pr [Pharmaceutics]; *maraviroc/ae [Adverse Drug Reaction]; *maraviroc/cm [Drug Comparison]; *maraviroc/dt [Drug Therapy]; *maraviroc/pd [Pharmacology]; nevirapine/cb [Drug Combination]; nevirapine/cm [Drug Comparison]; nevirapine/dt [Drug Therapy]; *nonnucleoside reverse transcriptase inhibitor/do [Drug Dose]; *nonnucleoside reverse transcriptase inhibitor/dt [Drug Therapy]; *nonnucleoside reverse transcriptase inhibitor/pr [Pharmaceutics]; *proteinase inhibitor/cb [Drug Combination]; *proteinase inhibitor/do [Drug Dose]; *proteinase inhibitor/it [Drug Interaction]; *proteinase inhibitor/dt [Drug Therapy]; *proteinase inhibitor/pr [Pharmaceutics]; raltegravir/cm [Drug Comparison]; raltegravir/dt [Drug Therapy]; raltegravir/pr [Pharmaceutics]; raltegravir/pd [Pharmacology]; ribavirin/cb [Drug Combination]; ribavirin/dt [Drug Therapy]; ritonavir plus saquinavir/ae [Adverse Drug Reaction]; ritonavir plus saquinavir/cb [Drug Combination]; ritonavir plus saquinavir/cm [Drug Comparison]; ritonavir plus saquinavir/dt [Drug Therapy]; stavudine/ae [Adverse Drug Reaction]; stavudine/dt [Drug Therapy]; tenofovir/ae [Adverse Drug Reaction]; tenofovir/cb [Drug Combination]; tenofovir/cm [Drug Comparison]; tenofovir/cr [Drug Concentration]; tenofovir/do [Drug Dose]; tenofovir/it [Drug Interaction]; tenofovir/dt [Drug Therapy]; tenofovir/pr [Pharmaceutics]; tenofovir/pk [Pharmacokinetics]; unclassified drug; unindexed drug; zidovudine/ae [Adverse Drug Reaction]; zidovudine/cm [Drug Comparison]; zidovudine/dt [Drug Therapy].

Link to the Ovid Full Text or citation:

Click here for full text options

76.

Drug-induced endocrine disorders in the intensive care unit.

Thomas Z., Bandali F., McCowen K., Malhotra A.

Critical Care Medicine. 38 (6 SUPPL.) (pp S219-S230), 2010. Date of Publication: June 2010.

AN: 2010312392

The neuroendocrine response to critical illness is key to the maintenance of homeostasis. Many of the drugs administered routinely in the intensive care unit significantly impact the neuroendocrine system. These agents can disrupt the hypothalamic-pituitary-adrenal axis, cause thyroid abnormalities, and result in dysglycemia. Herein, we review major drug-induced endocrine disorders and highlight some of the controversies that remain in this area. We also discuss some of the more rare drug-induced syndromes that have been described in the intensive care unit. Drugs that may result in an intensive care unit admission secondary to an endocrine-related adverse event are also included. Unfortunately, very few studies have systematically addressed drug-induced endocrine disorders in the critically ill. Timely identification and appropriate management of drug-induced endocrine adverse events may potentially improve outcomes in the critically ill. However, more research is needed to fully understand the impact of medications on endocrine function in the intensive care unit. Copyright © 2010 by the Society of Critical Care Medicine and Lippincott Williams & Wilkins.

Institution

(Thomas, Bandali) Department of Pharmacy Practice and Administration, Ernest Mario School of Pharmacy-Rutgers, State University of New Jersey, New Brunswick, NJ, United States (Thomas) Department of Pharmacy, Hackensack University Medical Center, Hackensack, NJ, United States

(Bandali) Department of Pharmacy, Saint Peter's University Hospital, New Brunswick, NJ, United States

(McCowen, Malhotra) Department of Medicine, Harvard Medical School, Boston, MA, United States

(McCowen) Harvard Vanguard Medical Associates, Boston, MA, United States

(Malhotra) Department of Medicine, Brigham and Women's Hospital, Boston, MA, United States

Publisher

Lippincott Williams and Wilkins (351 West Camden Street, Baltimore MD 21201-2436, United States)

Emtree Heading

abdominal pain/si [Side Effect]; adrenal cortex insufficiency/si [Side Effect]; adrenal disease/si [Side Effect]; adrenal insufficiency/si [Side Effect]; adrenal necrosis/si [Side Effect]; adrenal suppression; bleeding disorder/si [Side Effect]; chronic obstructive lung disease/dt [Drug Therapy]; clinical trial; critically ill patient; diabetes mellitus/si [Side Effect]; drug fatality/si [Side Effect]; drug fever/si [Side Effect]; drug withdrawal; *endocrine disease/si [Side Effect]; endocrine function; enteritis/dt [Drug Therapy]; enzyme activity; enzyme inhibition; epilepsy/dt [Drug Therapy]; euthyroid sick syndrome/si [Side Effect]; gluconeogenesis; goiter/si [Side Effect]; heart arrhythmia/si [Side Effect]; heart atrium fibrillation/dt [Drug Therapy]; heart disease/si [Side Effect]; heart infarction/si [Side Effect]; heart ventricle arrhythmia/si [Side Effect]; heparin induced thrombocytopenia/si [Side Effect]; human; hydrocortisone blood level; hyperglycemia/si [Side Effect]; hyperkalemia/si [Side Effect]; hyperthyroidism/si [Side Effect]; hypoaldosteronism/si [Side Effect]; hypoglycemia; hypotension/dt [Drug Therapy]; hypotension/si [Side Effect]; hypothyroidism/dt [Drug Therapy]; hypothyroidism/si [Side Effect]; insulin release; *intensive care unit; liothyronine blood level; managed care; myxedema/si [Side Effect]; neuroendocrine system; nonhuman; priority journal; review; seizure/dt [Drug Therapy]; septic shock/dt [Drug Therapy]; side effect/si [Side Effect]; stroke/si [Side Effect]; surgical infection/dt [Drug Therapy]; thyroid disease/si [Side Effect]; thyroid hormone metabolism; thyroid hormone release; thyroid hormone synthesis; thyrotoxicosis/dt [Drug Therapy]; thyrotoxicosis/si [Side Effect]; thyrotropin blood level; thyroxine blood level; thyroxine synthesis; treatment outcome; tuberculosis/dt [Drug Therapy]; urinary excretion; vascular disease/dt [Drug Therapy]; amiodarone/ae [Adverse Drug Reaction]; amiodarone/ct [Clinical Trial]; amiodarone/cm [Drug Comparison]; amiodarone/dt [Drug Therapy]; anticoagulant agent/ae [Adverse Drug Reaction]; ciprofloxacin/ae [Adverse Drug Reaction]; ciprofloxacin/cm [Drug Comparison]; cytochrome P450/ec [Endogenous Compound]; dopamine/ae [Adverse Drug Reaction]; dopamine/dt [Drug Therapy]; dronedarone/ae [Adverse Drug Reaction]; dronedarone/ct [Clinical Trial]; dronedarone/cm [Drug Comparison]; dronedarone/dt [Drug Therapy]; etiracetam/cm [Drug Comparison]; etiracetam/dt [Drug Therapy]; etomidate/ae [Adverse Drug Reaction]; etomidate/cm [Drug Comparison]; fluconazole/ae [Adverse Drug Reaction]; fluconazole/ct [Clinical Trial]; fluconazole/dt [Drug Therapy]; glucocorticoid/ae [Adverse Drug Reaction]; glucocorticoid/dt [Drug Therapy]; heparin/ae [Adverse Drug Reaction]; hydrocortisone/dt [Drug Therapy]; hydroxymethylglutaryl coenzyme A reductase inhibitor/ae [Adverse Drug Reaction]; itraconazole/ae [Adverse Drug Reaction]; ketamine/ae [Adverse Drug Reaction]; ketoconazole/ae [Adverse Drug Reaction]; levothyroxine/dt [Drug Therapy]; lithium/ae [Adverse Drug Reaction]; lithium/dt [Drug Therapy]; low molecular weight heparin/ae [Adverse Drug Reaction]; morphine/ae [Adverse Drug Reaction]; morphine/cm [Drug Comparison]; phenobarbital/ae [Adverse Drug Reaction]; phenobarbital/dt [Drug Therapy]; phenytoin/ae [Adverse Drug Reaction]; phenytoin/cm [Drug Comparison]; phenytoin/dt [Drug Therapy]; placebo; prednisone/dt [Drug Therapy]; propylthiouracil/dt [Drug Therapy]; rifampicin/ae [Adverse Drug Reaction]; rifampicin/cm [Drug Comparison]; rifampicin/dt [Drug Therapy]; thiamazole/dt [Drug Therapy]; thionamide derivative/dt [Drug Therapy]; unindexed drug; warfarin/ae [Adverse Drug Reaction].

Link to the Ovid Full Text or citation:

Click here for full text options

77.

Prevention trials: Their place in how we understand the value of prevention strategies.

Colditz G.A., Taylor P.R.

Annual Review of Public Health. 31 (pp 105-120), 2010. Date of Publication: 21 Apr 2010.

AN: 2010294685

Several key factors bear on the interpretation of prevention trials and observational studies that inform prevention strategies. These factors include the underlying disease process and aspects of the intervention: sustainability of behavior change, the time course of the intervention within the disease process, dose and duration of exposure needed to effect risk reduction, durability of the impact of intervention, and methodological problems in implementing and interpreting randomized trials and observational studies to evaluate prevention strategies. The question asked through an intent-to-treat analysis of a randomized controlled trial (RCT) differs from that in the observational setting. Furthermore, the long duration necessary to conduct prevention trials and the resulting lack of adherence to therapy can bias results toward the null. A broader range of approaches to evaluate prevention interventions and programs with improved knowledge synthesis and translation to public health practice will speed our progress toward achieving public health and prevention of chronic diseases. Copyright © 2010 by Annual Reviews. All rights reserved.

Institution

(Colditz) Department of Surgery, Washington University School of Medicine, Saint Louis, MO 63110, United States (Taylor) Genetic Epidemiology Branch, Division of Cancer Epidemiology and Genetics, National Cancer Institute, Bethesda, MD 20892-7236, United States

Publisher

Annual Reviews Inc. (4139 El Camino Way, P.O. Box 10139, Palo Alto CA 94306, United States)

Emtree Heading

behavior change; blood pressure monitoring; breast cancer/dt [Drug Therapy]; breast cancer/pc [Prevention]; calcium intake; cancer prevention; cardiovascular disease/dt [Drug Therapy]; cardiovascular disease/pc [Prevention]; chronic disease; clinical trial; colon cancer/dt [Drug Therapy]; colon cancer/pc [Prevention]; diabetes mellitus/pc [Prevention]; diet; diet supplementation; hepatitis B/dt [Drug Therapy]; hepatitis B/pc [Prevention]; human; hypertension/di [Diagnosis]; information dissemination; lifestyle modification; low drug dose; methodology; nonhuman; observational study; outcome assessment; patient compliance; physical activity; *preventive health service; *preventive medicine; priority journal; public health service; randomized controlled trial; review; risk reduction; screening test; tuberculosis/dt [Drug Therapy]; tuberculosis/pc [Prevention]; uterine cervix cancer/dt [Drug Therapy]; uterine cervix cancer/pc [Prevention]; uterus cancer/si [Side Effect]; vaccination; weight control; acetylsalicylic acid/ct [Clinical Trial]; acetylsalicylic acid/dt [Drug Therapy]; antihypertensive agent/dt [Drug Therapy]; antilipemic agent/dt [Drug Therapy]; BCG vaccine/dt [Drug Therapy]; calcium/ct [Clinical Trial]; calcium/dt [Drug Therapy]; cyclooxygenase 2 inhibitor/dt [Drug Therapy]; eflornithine/ct [Clinical Trial]; eflornithine/cb [Drug Combination]; eflornithine/dv [Drug Development]; eflornithine/dt [Drug Therapy]; eflornithine/to [Drug Toxicity]; hepatitis B vaccine/dt [Drug Therapy]; raloxifene/ct [Clinical Trial]; raloxifene/dt [Drug Therapy]; sulindac/ct [Clinical Trial]; sulindac/cb [Drug Combination]; tamoxifen/ae [Adverse Drug Reaction]; tamoxifen/ct [Clinical Trial]; tamoxifen/dt [Drug Therapy]; Wart virus vaccine/dt [Drug Therapy].

Drug Trade Names and Manufacturers

aspirin

Link to the Ovid Full Text or citation:

Click here for full text options

78.

A guide to treatment of sarcoidosis.

Papanikolaou I.C., Shigemitsu H., Sharma O.P.

Current Drug Therapy. 5 (2) (pp 122-131), 2010. Date of Publication: May 2010.

AN: 2010246183

Not all sarcoidosis patients need treatment. In about 40% of cases, disease will subside spontaneously. For the rest who need to be treated, older drugs like corticosteroids, methotrexate and hydroxychloroquine, as well as the more recent anti-TNF agents, will be effective in suppressing granulomatous inflammation in every organ involved in about 30% of cases. In 20% of the patients, disease will relapse upon discontinuation or tapering of treatment, and in 10% will progress (these two categories constituting chronic disease). In these cases, reinstitution of the same or alternative medication or a combination regimen is warranted. Drugs' side-effects and patients' comorbidities should be taken in consideration during the selection of the appropriate regimen. ©2010 Bentham Science Publishers Ltd.

Institution

(Papanikolaou) 3rd Pulmonary Department, Sismanoglio General Hospital, Athens, Greece (Shigemitsu, Sharma) Pulmonary and Critical Care Department, University of Southern California, Los Angeles, CA, United States

Publisher

Bentham Science Publishers B.V. (P.O. Box 294, Bussum 1400 AG, Netherlands)

Emtree Heading

adult respiratory distress syndrome/si [Side Effect]; alopecia/si [Side Effect]; article; artificial embolism; bacterial infection/si [Side Effect]; bladder cancer/si [Side Effect]; blood toxicity/si [Side Effect]; bone marrow suppression/si [Side Effect]; brain radiation; child care; chronic disease; chronic fatigue syndrome/dt [Drug Therapy]; clinical trial; comorbidity; computer assisted tomography; conjunctival biopsy; diabetes mellitus/si [Side Effect]; disease course; dizziness/si [Side Effect]; drug dose escalation; drug metabolism; drug treatment failure; elderly care; fatigue/dt [Drug Therapy]; fiberoptic bronchoscopy; fungemia/si [Side Effect]; gastrointestinal toxicity/si [Side Effect]; granuloma/si [Side Effect]; granulomatous inflammation; hematologic malignancy/si [Side Effect]; hemorrhagic cystitis/si [Side Effect]; histopathology; human; hypertension/si [Side Effect]; infection/dt [Drug Therapy]; infection prevention; interstitial pneumonia/si [Side Effect]; kidney failure/si [Side Effect]; liver biopsy; liver function test; liver toxicity/si [Side Effect]; low drug dose; lung aspergillosis/dt [Drug Therapy]; lung aspergillosis/su [Surgery]; lung resection; lung toxicity/si [Side Effect]; lung transplantation; lymph node biopsy; lymphoma/dt [Drug Therapy]; mental disease/si [Side Effect]; muscle biopsy; neoplasm/si [Side Effect]; nephrotoxicity/si [Side Effect]; neutropenia/si [Side Effect]; nuclear magnetic resonance imaging; osteoporosis/si [Side Effect]; pancytopenia/si [Side Effect]; peripheral neuropathy/si [Side Effect]; pregnancy; priority journal; pulmonary hypertension/dt [Drug Therapy]; relapse; retina disease/si [Side Effect]; salivary gland biopsy; *sarcoidosis/di [Diagnosis]; *sarcoidosis/dt [Drug Therapy]; *sarcoidosis/su [Surgery]; side effect/si [Side Effect]; skin biopsy; spleen biopsy; teratogenicity/si [Side Effect]; thorax radiography; treatment duration; treatment response; tuberculosis/dt [Drug Therapy]; tuberculosis/pc [Prevention]; unspecified side effect/si [Side Effect]; weight gain; antifungal agent/dt [Drug Therapy]; antifungal agent/po [Oral Drug Administration]; azathioprine/ae [Adverse Drug Reaction]; azathioprine/dt [Drug Therapy]; azathioprine/po [Oral Drug Administration]; azathioprine/pk [Pharmacokinetics]; budesonide/ct [Clinical Trial]; budesonide/dt [Drug Therapy]; budesonide/ih [Inhalational Drug Administration]; calcium ion/ec [Endogenous Compound]; chitotriosidase/ec [Endogenous Compound]; chlorambucil/ae [Adverse Drug Reaction]; chlorambucil/dt [Drug Therapy]; corticosteroid derivative/ct [Clinical Trial]; corticosteroid derivative/dt [Drug Therapy]; corticosteroid derivative/po [Oral Drug Administration]; cyclophosphamide/ae [Adverse Drug Reaction]; cyclophosphamide/dt [Drug Therapy]; cyclophosphamide/po [Oral Drug Administration]; cyclosporin/ae [Adverse Drug Reaction]; cyclosporin/dt [Drug Therapy]; dexmethylphenidate/ct [Clinical Trial]; dexmethylphenidate/dt [Drug Therapy]; dipeptidyl carboxypeptidase/ec [Endogenous Compound]; etanercept/ae [Adverse Drug Reaction]; etanercept/dt [Drug Therapy]; hydroxychloroquine/ae [Adverse Drug Reaction]; hydroxychloroquine/dt [Drug Therapy]; hydroxychloroquine/po [Oral Drug Administration]; ibuprofen/dt [Drug Therapy]; infliximab/ct [Clinical Trial]; infliximab/cb [Drug Combination]; infliximab/dt [Drug Therapy]; infliximab/iv [Intravenous Drug Administration]; interleukin 2 receptor/ec [Endogenous Compound]; isoniazid/dt [Drug Therapy]; ketoconazole/dt [Drug Therapy]; ketoconazole/pd [Pharmacology]; leflunomide/ae [Adverse Drug Reaction]; leflunomide/dt [Drug Therapy]; methotrexate/ae [Adverse Drug Reaction]; methotrexate/dt [Drug Therapy]; methotrexate/po [Oral Drug Administration]; methylprednisolone/dt [Drug Therapy]; mycophenolic acid/ae [Adverse Drug Reaction]; mycophenolic acid/cb [Drug Combination]; mycophenolic acid/dt [Drug Therapy]; mycophenolic acid/po [Oral Drug Administration]; naproxen/dt [Drug Therapy]; pentoxifylline/ae [Adverse Drug Reaction]; pentoxifylline/dt [Drug Therapy]; pentoxifylline/po [Oral Drug Administration]; pentoxifylline/pd [Pharmacology]; prednisolone/ae [Adverse Drug Reaction]; prednisolone/dt [Drug Therapy]; prednisolone/po [Oral Drug Administration]; prednisone/ae [Adverse Drug Reaction]; prednisone/dt [Drug Therapy]; prednisone/po [Oral Drug Administration]; thalidomide/ae [Adverse Drug Reaction]; thalidomide/dt [Drug Therapy]; thalidomide/po [Oral Drug Administration]; tumor necrosis factor alpha antibody/ae [Adverse Drug Reaction]; tumor necrosis factor alpha antibody/dt [Drug Therapy]; vasodilator agent/dt [Drug Therapy].

Link to the Ovid Full Text or citation:

Click here for full text options

79.

Pharmacokinetics of antituberculosis drugs in pulmonary tuberculosis patients with type 2 diabetes.

Ruslami R., Nijland H.M.J., Adhiarta I.G.N., Kariadi S.H.K.S., Alisjahbana B., Aarnoutse R.E., Van Crevel R.

Antimicrobial Agents and Chemotherapy. 54 (3) (pp 1068-1074), 2010. Date of Publication: March 2010.

AN: 2010125679

Altered pharmacokinetics of antituberculosis drugs may contribute to an increased risk of tuberculosis treatment failure for diabetic patients. We previously found that rifampin exposure was 2-fold lower in diabetic than in nondiabetic tuberculosis patients during the continuation phase of treatment. We now examined the influence of diabetes on the pharmacokinetics of antituberculosis drugs in the intensive phase of tuberculosis treatment, and we evaluated the effect of glycemic control. For this purpose, 18 diabetic and 18 gender- and body weight-matched nondiabetic tuberculosis patients were included in an Indonesian setting. Intensive pharmacokinetic sampling was performed for rifampin, pyrazinamide, and ethambutol at steady state. The bioavailability of rifampin was determined by comparing rifampin exposure after oral versus intravenous administration. Pharmacokinetic assessments were repeated for 10 diabetic tuberculosis patients after glycemic control. No differences in the areas under the concentrationtime curves of the drugs in plasma from 0 to 24 h postdose (AUC0-24), the maximum concentrations of the drugs in plasma (Cmax), the times to Cmax (Tmax), and the half-lives of rifampin, pyrazinamide, and ethambutol were found between diabetic and nondiabetic tuberculosis patients in the intensive phase of tuberculosis treatment. For rifampin, oral bioavailability and metabolism were similar in diabetic and nondiabetic patients. The pharmacokinetic parameters of antituberculosis drugs were not correlated with blood glucose levels or glucose control. We conclude that diabetes does not alter the pharmacokinetics of antituberculosis drugs during the intensive phase of tuberculosis treatment. The reduced exposure to rifampin of diabetic patients in the continuation phase may be due to increased body weight and possible differences in hepatic induction. Further research is needed to determine the cause of increased tuberculosis treatment failure among diabetic patients. Copyright © 2010, American Society for Microbiology. All Rights Reserved.

Institution

(Ruslami) Department of Pharmacology, Faculty of Medicine, Padjadjaran University, Bandung, Indonesia (Nijland, Aarnoutse) Department of Clinical Pharmacy, Radboud University Nijmegen Medical Centre, Nijmegen, Netherlands

(Adhiarta, Kariadi) Division of Endocrinology, Faculty of Medicine, Padjadjaran University, Bandung, Indonesia

(Alisjahbana) Division of Tropical Medicine and Infectious Diseases, Faculty of Medicine, Padjadjaran University, Bandung, Indonesia

(Van Crevel) Department of Internal Medicine, Radboud University Nijmegen Medical Centre, Nijmegen, Netherlands

Publisher

American Society for Microbiology (1752 N Street N.W., Washington DC 20036-2904, United States)

Emtree Heading

adult; area under the curve; article; body weight; clinical article; clinical research; clinical trial; comparative study; continuous infusion; controlled study; diabetic patient; drug bioavailability; drug blood level; drug exposure; drug half life; drug metabolism; female; gender; glucose blood level; glycemic control; human; Indonesia; liver; *lung tuberculosis/di [Diagnosis]; *lung tuberculosis/dt [Drug Therapy]; male; maximum plasma concentration; *non insulin dependent diabetes mellitus/di [Diagnosis]; *non insulin dependent diabetes mellitus/dt [Drug Therapy]; population; priority journal; sampling; steady state; treatment failure; antidiabetic agent/dt [Drug Therapy]; desacetylrifampin/cm [Drug Comparison]; desacetylrifampin/pk [Pharmacokinetics]; *ethambutol/ct [Clinical Trial]; *ethambutol/cm [Drug Comparison]; *ethambutol/dt [Drug Therapy]; *ethambutol/po [Oral Drug Administration]; *ethambutol/pk [Pharmacokinetics]; glucose/ec [Endogenous Compound]; human insulin; insulin/dt [Drug Therapy]; insulin/sc [Subcutaneous Drug Administration]; *isoniazid/ct [Clinical Trial]; *isoniazid/dt [Drug Therapy]; *isoniazid/pk [Pharmacokinetics]; *pyrazinamide/ct [Clinical Trial]; *pyrazinamide/cm [Drug Comparison]; *pyrazinamide/dt [Drug Therapy]; *pyrazinamide/po [Oral Drug Administration]; *pyrazinamide/pk [Pharmacokinetics]; *rifampicin/ct [Clinical Trial]; *rifampicin/cm [Drug Comparison]; *rifampicin/dt [Drug Therapy]; *rifampicin/iv [Intravenous Drug Administration]; *rifampicin/po [Oral Drug Administration]; *rifampicin/pk [Pharmacokinetics]; rifampicin derivative/pk [Pharmacokinetics]; unclassified drug.

Drug Trade Names and Manufacturers

Bayer, Novo Nordisk, humulin: Novo Nordisk, rifadin: Bayer

Link to the Ovid Full Text or citation:

Click here for full text options

80.

New insights into T cell biology and T cell-directed therapy for autoimmunity, inflammation, and immunosuppression.

Steward-Tharp S.M., Song Y.-J., Siegel R.M., O'Shea J.J.

The Year in Immunology 2. Annals of the New York Academy of Sciences. 1183 (pp 123-148), 2010. Date of Publication: 10 Jan 2010.

AN: 2010080656

T cell-directed therapies have become mainstays in the management of various autoimmune diseases and organ transplantation. The understanding of T cell biology has expanded greatly since the development of most agents currently in use. Here we discuss important recent discoveries pertaining to T helper cell differentiation, lineage commitment, and function. Within this context, we examine existing T cell-directed therapies, including new agents being evaluated in clinical and preclinical studies. We also use recent findings to speculate on novel targets. © 2009 New York Academy of Sciences.

Institution

(Steward-Tharp, O'Shea) Molecular Immunology and Inflammation Branch, National Institute of Arthritis and Musculoskeletal and Skin Diseases, National Institutes of Health, 10 Center Drive, RM 13C103, Bethesda, MD 20892, United States (Steward-Tharp) Sir William Dunn School of Pathology, University of Oxford, Oxford, United Kingdom

(Song, Siegel) Autoimmunity Branch, National Institute of Arthritis and Musculoskeletal and Skin Diseases, National Institutes of Health, Bethesda, MD, United States

Publisher

Blackwell Publishing Inc. (350 Main Street, Malden MA 02148, United States)

Emtree Heading

acute graft rejection/dt [Drug Therapy]; acute graft rejection/pc [Prevention]; allergic encephalomyelitis/dt [Drug Therapy]; ankylosing spondylitis/dt [Drug Therapy]; antigen presenting cell; asthma/dt [Drug Therapy]; atopic dermatitis/dt [Drug Therapy]; *autoimmune disease/dt [Drug Therapy]; autoimmune thyroiditis/si [Side Effect]; autoimmunity; B cell lymphoma/dt [Drug Therapy]; CD4+ CD25+ T lymphocyte; CD4+ T lymphocyte; cell function; cell homing; cell lineage; chronic lymphatic leukemia/dt [Drug Therapy]; clinical trial; Crohn disease/dt [Drug Therapy]; cytokine production; drug mechanism; drug treatment failure; dyslipidemia/si [Side Effect]; enteritis/dt [Drug Therapy]; flu like syndrome/si [Side Effect]; gastrointestinal symptom/si [Side Effect]; genetic polymorphism; glucose intolerance/si [Side Effect]; graft versus host reaction/dt [Drug Therapy]; helper cell; human; hyper IgE syndrome; hyperkalemia/si [Side Effect]; hypertension/si [Side Effect]; hypomagnesemia/si [Side Effect]; immunological tolerance; *immunosuppressive treatment; immunotherapy; infection/si [Side Effect]; *inflammation; insulin dependent diabetes mellitus/dt [Drug Therapy]; kidney transplantation; lupus erythematosus nephritis/dt [Drug Therapy]; lupus vulgaris/dt [Drug Therapy]; lymphocyte differentiation; lymphocytopenia/si [Side Effect]; major histocompatibility complex; multiple organ failure/si [Side Effect]; multiple sclerosis/dt [Drug Therapy]; myasthenia gravis/dt [Drug Therapy]; neoplasm/si [Side Effect]; nephrotoxicity/si [Side Effect]; neurotoxicity/si [Side Effect]; nonhuman; progressive multifocal leukoencephalopathy/si [Side Effect]; protein function; protein protein interaction; psoriasis/dt [Drug Therapy]; psoriasis vulgaris/dt [Drug Therapy]; psoriatic arthritis/dt [Drug Therapy]; pyoderma gangrenosum/dt [Drug Therapy]; review; rheumatoid arthritis/dt [Drug Therapy]; sepsis/si [Side Effect]; signal transduction; Sjoegren syndrome; skin lupus erythematosus/dt [Drug Therapy]; systemic lupus erythematosus/dt [Drug Therapy]; T cell lymphoma/dt [Drug Therapy]; T lymphocyte activation; Th1 cell; Th17 cell; Th2 cell; ulcerative colitis/dt [Drug Therapy]; vasculitis/dt [Drug Therapy]; abatacept/ct [Clinical Trial]; abatacept/dt [Drug Therapy]; abatacept/pd [Pharmacology]; adalimumab/dt [Drug Therapy]; alefacept/ct [Clinical Trial]; alefacept/dt [Drug Therapy]; alemtuzumab/ae [Adverse Drug Reaction]; alemtuzumab/ct [Clinical Trial]; alemtuzumab/dt [Drug Therapy]; alemtuzumab/pd [Pharmacology]; alicaforsen/ct [Clinical Trial]; alicaforsen/dt [Drug Therapy]; alicaforsen/pd [Pharmacology]; basiliximab/ct [Clinical Trial]; basiliximab/pd [Pharmacology]; belatacept/ct [Clinical Trial]; belatacept/pd [Pharmacology]; bti 322; calcineurin inhibitor/ae [Adverse Drug Reaction]; calcineurin inhibitor/dt [Drug Therapy]; CD3 antibody/dt [Drug Therapy]; CD3 antibody/po [Oral Drug Administration]; CD40 ligand monoclonal antibody/dt [Drug Therapy]; efalizumab/ae [Adverse Drug Reaction]; efalizumab/ct [Clinical Trial]; efalizumab/dt [Drug Therapy]; efalizumab/pd [Pharmacology]; etanercept/dt [Drug Therapy]; fingolimod/ct [Clinical Trial]; fingolimod/dt [Drug Therapy]; fingolimod/pd [Pharmacology]; galiximab/ae [Adverse Drug Reaction]; galiximab/dt [Drug Therapy]; gamma interferon/ec [Endogenous Compound]; infliximab/dt [Drug Therapy]; intercellular adhesion molecule 1 antibody/dt [Drug Therapy]; monclonal antibody jo2/to [Drug Toxicity]; *monoclonal antibody/pd [Pharmacology]; natalizumab/ae [Adverse Drug Reaction]; natalizumab/ct [Clinical Trial]; natalizumab/dt [Drug Therapy]; OKT 3/ae [Adverse Drug Reaction]; OKT 3/ct [Clinical Trial]; OKT 3/dt [Drug Therapy]; OKT 3/pd [Pharmacology]; otelixizumab/ct [Clinical Trial]; otelixizumab/dt [Drug Therapy]; otelixizumab/pd [Pharmacology]; OX40 ligand antibody/dt [Drug Therapy]; siplizumab/ae [Adverse Drug Reaction]; siplizumab/dt [Drug Therapy]; tacrolimus/ct [Clinical Trial]; tacrolimus/dt [Drug Therapy]; tacrolimus/pd [Pharmacology]; teplizumab/ct [Clinical Trial]; teplizumab/pd [Pharmacology]; tgn 1412/ae [Adverse Drug Reaction]; tolerx; unclassified drug; unindexed drug; visilizumab/ct [Clinical Trial]; visilizumab/dt [Drug Therapy]; visilizumab/pd [Pharmacology]; zanolimumab/ct [Clinical Trial]; zanolimumab/dt [Drug Therapy]; zanolimumab/pd [Pharmacology].

Drug Trade Names and Manufacturers

amevive, bms 224818, bti 322, campath, fty 720, isis 2302, lea 29y, nuvion, OKT 3, orencia, prograf, protopic, raptiva, simulect, tgn 1412, tolerx, tysabri

Link to the Ovid Full Text or citation:

Click here for full text options

81.

The safety profile of biologic therapies for juvenile idiopathic arthritis.

Hashkes P.J., Uziel Y., Laxer R.M.

Nature Reviews Rheumatology. 6 (10) (pp 561-571), 2010. Date of Publication: October 2010.

AN: 2010553694

The treatment of juvenile idiopathic arthritis (JIA) has been revolutionized by the use of novel biologic agents that have much improved patients' short-term and, according to early evidence, long-term outcomes. Currently available biologic agents used to treat patients with JIA include tumor necrosis factor (TNF) blockers, various agents that target interleukin (IL)-1 and the IL-6 receptor, T-cell co-stimulation inhibitors and antibodies to B-lymphocyte antigen CD20. These agents are increasingly used early in the course of the disease (often in combination with other immunosuppressive medications) and often for long periods of time, as patients can be difficult to wean from their use. Safety concerns (especially the long-term effects of biologic therapy) are, therefore, being examined more closely. For instance, in 2009, the FDA issued a warning related to the development of malignancies in patients with JIA who had used anti-TNF medications for >2.5 years. In this Review, data related to the safety profile of all currently available biologic agents used to treat JIA are examined, with a particular focus on anti-TNF therapy, the most studied biologic agent for JIA. Safety issues that need further study, including the implementation of registries to monitor long-term drug safety, are also discussed. © 2010 Macmillan Publishers Limited. All rights reserved.

Institution

(Hashkes) Pediatric Rheumatology Unit, Department of Pediatrics, Shaare Zedek Medical Center, PO Box 3235, Jerusalem 91031, Israel (Uziel) Pediatric Rheumatology, Department of Pediatrics, Meir Medical Center, 59 Tchernichovsky Street, Kfar Saba 44281, Israel

(Laxer) Division of Pediatric Rheumatology, Hospital for Sick Children, 555 University Avenue, Toronto, ON M5G 1X8, Canada

Publisher

Nature Publishing Group (Houndmills, Basingstoke, Hampshire RG21 6XS, United Kingdom)

Emtree Heading

acute lymphoblastic leukemia/si [Side Effect]; aggression; alopecia/si [Side Effect]; amnesia/si [Side Effect]; anaphylaxis/si [Side Effect]; anemia/si [Side Effect]; antibody production; anxiety; autoimmune disease/si [Side Effect]; autoimmune hepatitis/si [Side Effect]; bacterial infection/si [Side Effect]; *biological therapy; bronchopneumonia/si [Side Effect]; chill/si [Side Effect]; clinical trial; death; demyelinating disease/si [Side Effect]; depression/si [Side Effect]; dermatomyositis/si [Side Effect]; disease exacerbation/si [Side Effect]; drug eruption/si [Side Effect]; drug hypersensitivity/si [Side Effect]; drug megadose; drug safety; drug targeting; drug withdrawal; dyspnea/si [Side Effect]; enteritis/dt [Drug Therapy]; enteritis/si [Side Effect]; fatigue/si [Side Effect]; fever/si [Side Effect]; food and drug administration; gastroenteritis/si [Side Effect]; gastrointestinal disease/si [Side Effect]; headache/si [Side Effect]; herpes zoster/si [Side Effect]; herpetic stomatitis/si [Side Effect]; Hodgkin disease/si [Side Effect]; human; hypotension/si [Side Effect]; infection/si [Side Effect]; injection site pain/si [Side Effect]; injection site reaction/si [Side Effect]; insomnia/si [Side Effect]; insulin dependent diabetes mellitus/si [Side Effect]; intestine ulcer/si [Side Effect]; *juvenile rheumatoid arthritis/dt [Drug Therapy]; liver dysfunction/si [Side Effect]; loading drug dose; long term care; low drug dose; lung fibrosis/si [Side Effect]; lung tuberculosis/si [Side Effect]; lupus like syndrome/si [Side Effect]; lymphoma/si [Side Effect]; macrophage activation syndrome/si [Side Effect]; malignant neoplastic disease/si [Side Effect]; mental concentration; mental disease/si [Side Effect]; multiple sclerosis/si [Side Effect]; mycobacteriosis/si [Side Effect]; neurologic disease/si [Side Effect]; neurological complication/si [Side Effect]; nonhodgkin lymphoma/si [Side Effect]; opportunistic infection/si [Side Effect]; outcome assessment; pain/si [Side Effect]; pancytopenia/si [Side Effect]; panic/si [Side Effect]; priority journal; psoriasis/si [Side Effect]; psychosis/si [Side Effect]; repeated drug dose; retrobulbar optic neuropathy/si [Side Effect]; review; sarcoidosis/si [Side Effect]; side effect/si [Side Effect]; single drug dose; systemic lupus erythematosus/si [Side Effect]; T cell lymphoma/si [Side Effect]; thorax pain/si [Side Effect]; thyroid cancer/si [Side Effect]; thyroid carcinoma/si [Side Effect]; treatment duration; unspecified side effect/si [Side Effect]; upper respiratory tract infection/si [Side Effect]; urticaria/si [Side Effect]; uveitis/dt [Drug Therapy]; uveitis/si [Side Effect]; vasculitis/dt [Drug Therapy]; vasculitis/si [Side Effect]; vertigo/si [Side Effect]; wheezing/si [Side Effect]; abatacept/ae [Adverse Drug Reaction]; abatacept/ct [Clinical Trial]; abatacept/dt [Drug Therapy]; abatacept/iv [Intravenous Drug Administration]; abatacept/pd [Pharmacology]; adalimumab/ae [Adverse Drug Reaction]; adalimumab/ct [Clinical Trial]; adalimumab/cb [Drug Combination]; adalimumab/cm [Drug Comparison]; adalimumab/dt [Drug Therapy]; adalimumab/pd [Pharmacology]; adalimumab/sc [Subcutaneous Drug Administration]; azathioprine/dt [Drug Therapy]; canakinumab/ae [Adverse Drug Reaction]; canakinumab/ct [Clinical Trial]; canakinumab/dt [Drug Therapy]; canakinumab/pd [Pharmacology]; canakinumab/sc [Subcutaneous Drug Administration]; CD20 antibody/ec [Endogenous Compound]; cyclosporin A/dt [Drug Therapy]; daclizumab/ae [Adverse Drug Reaction]; daclizumab/ct [Clinical Trial]; daclizumab/dt [Drug Therapy]; daclizumab/pd [Pharmacology]; etanercept/ae [Adverse Drug Reaction]; etanercept/ct [Clinical Trial]; etanercept/cb [Drug Combination]; etanercept/cm [Drug Comparison]; etanercept/dt [Drug Therapy]; etanercept/pd [Pharmacology]; etanercept/sc [Subcutaneous Drug Administration]; immunosuppressive agent; infliximab/ae [Adverse Drug Reaction]; infliximab/cb [Drug Combination]; infliximab/cm [Drug Comparison]; infliximab/dt [Drug Therapy]; infliximab/iv [Intravenous Drug Administration]; infliximab/pd [Pharmacology]; interleukin 1/ec [Endogenous Compound]; interleukin 6 receptor/ec [Endogenous Compound]; methotrexate/ae [Adverse Drug Reaction]; methotrexate/ct [Clinical Trial]; methotrexate/cb [Drug Combination]; methotrexate/dt [Drug Therapy]; placebo; recombinant interleukin 1 receptor blocking agent/ae [Adverse Drug Reaction]; recombinant interleukin 1 receptor blocking agent/ct [Clinical Trial]; recombinant interleukin 1 receptor blocking agent/dt [Drug Therapy]; recombinant interleukin 1 receptor blocking agent/pd [Pharmacology]; recombinant interleukin 1 receptor blocking agent/sc [Subcutaneous Drug Administration]; rilonacept/ae [Adverse Drug Reaction]; rilonacept/ct [Clinical Trial]; rilonacept/dt [Drug Therapy]; rilonacept/pd [Pharmacology]; rilonacept/sc [Subcutaneous Drug Administration]; rituximab/dt [Drug Therapy]; rituximab/iv [Intravenous Drug Administration]; rituximab/pd [Pharmacology]; tocilizumab/ae [Adverse Drug Reaction]; tocilizumab/ct [Clinical Trial]; tocilizumab/dt [Drug Therapy]; tocilizumab/iv [Intravenous Drug Administration]; tocilizumab/pd [Pharmacology]; tumor necrosis factor antibody/ae [Adverse Drug Reaction]; tumor necrosis factor antibody/dt [Drug Therapy].

Link to the Ovid Full Text or citation:

Click here for full text options

82.

Pharmacologic treatment of behavioral symptoms associated with autism and other pervasive developmental disorders.

Wink L.K., Erickson C.A., McDougle C.J.

Current Treatment Options in Neurology. 12 (6) (pp 529-538), 2010. Date of Publication: November 2010.

AN: 2010586808

Opinion statement: Pervasive developmental disorders (PDDs), including autistic disorder (autism), Asperger's disorder, and pervasive developmental disorder not otherwise specified (PDD NOS), are neurodevelopmental disorders of childhood onset. These disorders persist throughout the lifespan of affected individuals and are characterized by impaired social behavior and communication, along with repetitive, stereotypic behaviors. Early diagnosis and subsequent behavioral therapy have been shown to improve outcomes for these individuals. Risperidone and aripiprazole have been approved by the United States Food and Drug Administration (FDA) for treatment of irritability associated with autism in children and adolescents. Despite their efficacy, use of these medications is limited by their side effects. In individuals with severe irritability, the first-line treatment is often risperidone. Because of its relatively lower risk of weight gain and metabolic side effects, aripiprazole may be used initially if there is a personal or family history of obesity or diabetes. Monitoring of body mass index and metabolic profiles is indicated with both medications. Stereotypic behaviors associated with autism, though clearly driven by neurobiologic processes, can also be understood as coping mechanisms used to decrease anxiety. From this perspective, therapies targeting reduction of these symptoms may be contraindicated. However, when these symptoms are severe and interfering, pharmacotherapy may be necessary. Serotonin reuptake inhibitors are of limited efficacy in children and adolescents, but risperidone and aripiprazole have been shown to reduce these symptoms. There remains a need for further safety and efficacy research in this area. Hyperactivity and inattention are currently treated with a variety of medications, including guanfacine, which has a relatively benign side effect profile. Stimulant medications are generally avoided as first-line treatment for hyperactivity because of concerns about increased irritability. Currently, social impairment is best addressed through behavioral therapy and social skills training. Novel pharmacotherapies to improve social impairment are in the early stages of research. © 2010 Springer Science+Business Media, LLC.

Institution

(Wink, Erickson, McDougle) Department of Psychiatry, Indiana University School of Medicine, Psychiatry Building-A305, 1111 West 10th Street, Indianapolis, IN 46202-4800, United States

Publisher

Current Science Ltd (34-42 Cleveland Street, London W1P 6LB, United Kingdom)

Emtree Heading

add on therapy; agitation; akathisia/si [Side Effect]; Alzheimer disease/dt [Drug Therapy]; anorexia/si [Side Effect]; antisocial behavior/dt [Drug Therapy]; anxiety disorder/dt [Drug Therapy]; Asperger syndrome/dt [Drug Therapy]; attention deficit disorder/co [Complication]; attention deficit disorder/di [Diagnosis]; attention deficit disorder/dt [Drug Therapy]; attention disturbance/co [Complication]; attention disturbance/dt [Drug Therapy]; *autism/dt [Drug Therapy]; automutilation/dt [Drug Therapy]; *behavior disorder/co [Complication]; *behavior disorder/di [Diagnosis]; *behavior disorder/dt [Drug Therapy]; *behavior disorder/si [Side Effect]; *behavior disorder/th [Therapy]; behavior therapy; blood pressure; body mass; cardiovascular disease/si [Side Effect]; central nervous system disease/si [Side Effect]; child psychiatry; clinical trial; communication disorder/co [Complication]; communication disorder/di [Diagnosis]; communication disorder/dt [Drug Therapy]; communication disorder/th [Therapy]; compulsion/co [Complication]; compulsion/di [Diagnosis]; compulsion/dt [Drug Therapy]; compulsion/th [Therapy]; concentration loss/si [Side Effect]; constipation/si [Side Effect]; coping behavior; diarrhea/si [Side Effect]; disease association; dizziness/si [Side Effect]; drowsiness/si [Side Effect]; drug antagonism; drug approval; drug blood level; drug contraindication; drug efficacy; drug indication; drug mechanism; drug metabolism; drug safety; drug tolerability; drug withdrawal; dry skin/si [Side Effect]; dyslipidemia/si [Side Effect]; early diagnosis; energy metabolism; fatigue/si [Side Effect]; gastrointestinal symptom/si [Side Effect]; headache/si [Side Effect]; heart rate; human; hyperactivity/co [Complication]; hyperactivity/dt [Drug Therapy]; hyperactivity/si [Side Effect]; hyperglycemia/si [Side Effect]; hypersalivation/si [Side Effect]; hypertension/dt [Drug Therapy]; hypotension/si [Side Effect]; impulsiveness; increased appetite/si [Side Effect]; infantile autism/dt [Drug Therapy]; insomnia/si [Side Effect]; intellectual impairment/dt [Drug Therapy]; irritability; mania/si [Side Effect]; metabolic disorder/si [Side Effect]; metabolic parameters; motor dysfunction/si [Side Effect]; nausea/si [Side Effect]; onset age; outcome assessment; personality disorder/dt [Drug Therapy]; pervasive developmental disorder not otherwise specified; *psychopharmacotherapy; QT prolongation/si [Side Effect]; rash/si [Side Effect]; review; risk benefit analysis; sedation; seizure/si [Side Effect]; seizure threshold; sexual dysfunction/si [Side Effect]; side effect/si [Side Effect]; sleep disorder/si [Side Effect]; social adaptation; sociopathy/co [Complication]; sociopathy/di [Diagnosis]; sociopathy/dt [Drug Therapy]; sociopathy/th [Therapy]; somnolence/si [Side Effect]; stereotypy; suicidal ideation/si [Side Effect]; syncope/si [Side Effect]; tardive dyskinesia/si [Side Effect]; tremor/si [Side Effect]; tuberculosis/dt [Drug Therapy]; unspecified side effect/si [Side Effect]; vertigo/si [Side Effect]; vomiting/si [Side Effect]; weight gain; xerostomia/si [Side Effect]; antihypertensive agent/it [Drug Interaction]; aripiprazole/ae [Adverse Drug Reaction]; aripiprazole/ct [Clinical Trial]; aripiprazole/cr [Drug Concentration]; aripiprazole/dt [Drug Therapy]; atomoxetine/ae [Adverse Drug Reaction]; atomoxetine/ct [Clinical Trial]; atomoxetine/cm [Drug Comparison]; atomoxetine/it [Drug Interaction]; atomoxetine/dt [Drug Therapy]; atomoxetine/pd [Pharmacology]; citalopram/ae [Adverse Drug Reaction]; citalopram/ct [Clinical Trial]; citalopram/cm [Drug Comparison]; citalopram/it [Drug Interaction]; citalopram/dt [Drug Therapy]; cycloserine/ae [Adverse Drug Reaction]; cycloserine/ct [Clinical Trial]; cycloserine/cr [Drug Concentration]; cycloserine/it [Drug Interaction]; cycloserine/dt [Drug Therapy]; cycloserine/pd [Pharmacology]; ethionamide/it [Drug Interaction]; fluoxetine/ae [Adverse Drug Reaction]; fluoxetine/ct [Clinical Trial]; fluoxetine/cm [Drug Comparison]; fluoxetine/it [Drug Interaction]; fluoxetine/dt [Drug Therapy]; guanfacine/ae [Adverse Drug Reaction]; guanfacine/ct [Clinical Trial]; guanfacine/dt [Drug Therapy]; isoniazid/it [Drug Interaction]; memantine/ae [Adverse Drug Reaction]; memantine/it [Drug Interaction]; memantine/dt [Drug Therapy]; methylphenidate/ae [Adverse Drug Reaction]; methylphenidate/ct [Clinical Trial]; methylphenidate/cm [Drug Comparison]; methylphenidate/dt [Drug Therapy]; monoamine oxidase inhibitor/it [Drug Interaction]; n methyl dextro aspartic acid receptor blocking agent/it [Drug Interaction]; neuroleptic agent/ae [Adverse Drug Reaction]; oxytocin/ae [Adverse Drug Reaction]; oxytocin/dt [Drug Therapy]; oxytocin/im [Intramuscular Drug Administration]; oxytocin/na [Intranasal Drug Administration]; oxytocin/iv [Intravenous Drug Administration]; oxytocin/pd [Pharmacology]; pimozide/it [Drug Interaction]; placebo; psychostimulant agent/ae [Adverse Drug Reaction]; psychostimulant agent/dt [Drug Therapy]; risperidone/ae [Adverse Drug Reaction]; risperidone/ct [Clinical Trial]; risperidone/dt [Drug Therapy]; risperidone/pk [Pharmacokinetics]; serotonin uptake inhibitor/dt [Drug Therapy]; thioridazine/it [Drug Interaction].

Link to the Ovid Full Text or citation:

Click here for full text options

83.

KDIGO clinical practice guideline for the care of kidney transplant recipients: A summary.

Kasiske B.L., Zeier M.G., Chapman J.R., Craig J.C., Ekberg H., Garvey C.A., Green M.D., Jha V., Josephson M.A., Kiberd B.A., Kreis H.A., McDonald R.A., Newmann J.M., Obrador G.T., Vincenti F.G., Cheung M., Earley A., Raman G., Abariga S., Wagner M., Balk E.M.

Kidney International. 77 (4) (pp 299-311), 2010. Date of Publication: February 2010.

AN: 2010083332

The 2009 Kidney Disease: Improving Global Outcomes (KDIGO) clinical practice guideline on the monitoring, management, and treatment of kidney transplant recipients is intended to assist the practitioner caring for adults and children after kidney transplantation. The guideline development process followed an evidence-based approach, and management recommendations are based on systematic reviews of relevant treatment trials. Critical appraisal of the quality of the evidence and the strength of recommendations followed the Grades of Recommendation Assessment, Development, and Evaluation (GRADE) approach. The guideline makes recommendations for immunosuppression and graft monitoring, as well as prevention and treatment of infection, cardiovascular disease, malignancy, and other complications that are common in kidney transplant recipients, including hematological and bone disorders. Limitations of the evidence, especially the lack of definitive clinical outcome trials, are discussed and suggestions are provided for future research. This summary includes a brief description of methodology and the complete guideline recommendations but does not include the rationale and references for each recommendation, which are published elsewhere. © 2010 International Society of Nephrology.

Institution

(Kasiske) Hennepin County Medical Center, Department of Medicine, Minneapolis, MN, United States (Zeier) University Hospital of Heidelberg, Department of Medicine, Heidelberg, Germany

(Chapman) Westmead Hospital, Department of Renal Medicine, Westmead, Australia

(Craig) Children's Hospital at Westmead, Department of Nephrology, Westmead, Australia

(Ekberg) Lund University, Malmo, Department of Nephrology and Transplantation, Sweden

(Garvey) University of Minnesota, Minneapolis, MN, United States

(Green) Children's Hospital of Pittsburgh, Division of Infectious Diseases, Pittsburgh, PA, United States

(Jha) Postgraduate Medical Institute, Chandigarh, India

(Josephson) University of Chicago, Department of Medicine, Section of Nephrology, Chicago, IL, United States

(Kiberd) Dalhousie University, Department of Medicine, Halifax, Canada

(Kreis) Universite Paris Descartes and Hopital Necker, Service de Transplantation, Paris, France

(McDonald) University of Washington, Seattle Children's Hospital, Division of Nephrology, Seattle, WA, United States

(Newmann) Health Policy Research and Analysis, Reston, VA, United States

(Obrador) Universidad Panamericana School of Medicine, Mexico City, Mexico

(Vincenti) University of California at San Francisco, Department of Medicine, Division of Nephrology, San Francisco, CA, United States

(Cheung) National Kidney Foundation, 30 E. 33rd Street, New York, NY 10016, United States

(Earley, Raman, Abariga, Wagner, Balk) Tufts Center for Kidney Disease Guideline Development and Implementation, Tufts Medical Center, Boston, MA, United States

Publisher

Nature Publishing Group (Houndmills, Basingstoke, Hampshire RG21 6XS, United Kingdom)

Emtree Heading

acute graft rejection/dt [Drug Therapy]; ANCA associated vasculitis/dt [Drug Therapy]; BK virus; bone disease; candidiasis/pc [Prevention]; cardiovascular disease; chickenpox/dt [Drug Therapy]; chronic graft rejection/pc [Prevention]; cigarette smoking; combination chemotherapy; cytomegalovirus infection/dt [Drug Therapy]; cytomegalovirus infection/pc [Prevention]; diabetes mellitus; diet therapy; drug cost; drug megadose; drug monitoring; dyslipidemia; erythrocytosis/dt [Drug Therapy]; female fertility; focal glomerulosclerosis/th [Therapy]; gout; *graft recipient; growth, development and aging; hematologic disease; hemodialysis; hepatitis B/dt [Drug Therapy]; hepatitis C; herpes simplex/dt [Drug Therapy]; herpes simplex/pc [Prevention]; Herpes simplex virus 1; Herpes simplex virus 2; herpes zoster/dt [Drug Therapy]; herpes zoster/pc [Prevention]; human; Human immunodeficiency virus infected patient; hyperoxaluria/dt [Drug Therapy]; hyperoxaluria/th [Therapy]; hypertension; hyperuricemia; immunosuppressive treatment; infection prevention; influenza vaccination; kidney allograft; kidney allograft rejection/pc [Prevention]; kidney biopsy; kidney function; *kidney graft; kidney graft rejection/dt [Drug Therapy]; kidney graft rejection/pc [Prevention]; kidney graft rejection/th [Therapy]; lifestyle; lip cancer; long term care; lymphoproliferative disease; maintenance therapy; male fertility; mental health; obesity; *patient care; patient compliance; patient monitoring; plasmapheresis; Pneumocystis pneumonia/pc [Prevention]; *practice guideline; priority journal; proteinuria/dt [Drug Therapy]; recurrent disease/dt [Drug Therapy]; review; sexual function; skin cancer; treatment outcome; tuberculosis; urinary tract infection/pc [Prevention]; vaccination; virus infection; aciclovir/dt [Drug Therapy]; aciclovir/iv [Intravenous Drug Administration]; aciclovir/po [Oral Drug Administration]; angiotensin receptor antagonist/dt [Drug Therapy]; azathioprine/dt [Drug Therapy]; calcineurin inhibitor/dt [Drug Therapy]; calcium channel blocking agent/dt [Drug Therapy]; CD20 antibody/dt [Drug Therapy]; corticosteroid derivative/dt [Drug Therapy]; cyclophosphamide/dt [Drug Therapy]; cyclosporin A/dt [Drug Therapy]; cytokine receptor antagonist/dt [Drug Therapy]; dipeptidyl carboxypeptidase inhibitor/dt [Drug Therapy]; famciclovir/dt [Drug Therapy]; famciclovir/po [Oral Drug Administration]; ganciclovir/dt [Drug Therapy]; ganciclovir/iv [Intravenous Drug Administration]; ganciclovir/po [Oral Drug Administration]; generic drug/dt [Drug Therapy]; immunoglobulin/dt [Drug Therapy]; immunoglobulin/iv [Intravenous Drug Administration]; interleukin 2 receptor blocking agent/dt [Drug Therapy]; ketoconazole/dt [Drug Therapy]; magnesium oxide/dt [Drug Therapy]; mammalian target of rapamycin inhibitor; mycophenolic acid/dt [Drug Therapy]; mycophenolic acid 2 morpholinoethyl ester; OKT 3/dt [Drug Therapy]; phosphate/dt [Drug Therapy]; prednisone/dt [Drug Therapy]; pyridoxine/dt [Drug Therapy]; tacrolimus/dt [Drug Therapy]; tenofovir/dt [Drug Therapy]; unclassified drug; unindexed drug; valaciclovir/dt [Drug Therapy]; valaciclovir/po [Oral Drug Administration]; valganciclovir/dt [Drug Therapy]; valganciclovir/po [Oral Drug Administration].

Link to the Ovid Full Text or citation:

Click here for full text options

84.

Patient with abdominal pain-concealing gastrointestinal mucormycosis.

Azhar A., Calubiran O.V., Kilaru R.M., Lincoln J.A., Moshenyat I., Basti K., Visconti E.

Gastroenterology and Hepatology. 5 (9) (pp 657-661), 2009. Date of Publication: September 2009.

AN: 2010387024

We presented a case of gastrointestinal mucormycosis with diabetic mellitus and gastric ulcer, complicated by perforation, necrosis, and peritonitis. It is unclear whether the associated pulmonary tuberculosis and alcohol abuse may have also contributed to the patient's debilitating illness. Early diagnosis and aggressive coordinated medical therapy and surgical intervention are key to management and survival. The advent of novel antifungals such as posaconazole may pose a promising alternative to amphotericin B in the treatment of mucormycosis, but it may be limited early on in patients with gastrointestinal mucormycosis, as it is available only in oral formulation. However, a recent study conducted on mice showed that combination therapy of lipid formulations of amphotericin B and posaconazole was not more effective than lipid formulations of amphotericin B alone, and posaconazole monotherapy did not improve survival or reduce fungal burden versus placebo.

Institution

(Azhar) Department of Internal Medicine, Lutheran Medical Center, Brooklyn, NY, United States (Calubiran, Basti, Visconti) Department of Infectious Disease, Lutheran Medical Center, Brooklyn, NY, United States

(Kilaru) Department of Surgery, Lutheran Medical Center, Brooklyn, NY, United States

(Lincoln) Department of Pathology, Lutheran Medical Center, Brooklyn, NY, United States

(Moshenyat) Department of Gastroenterology, Lutheran Medical Center, Brooklyn, NY, United States

Publisher

Gastro-Hep Communications, Inc. (611 Broadway, Suite 310, New York NY 10012, United States)

Emtree Heading

abdominal infection/dt [Drug Therapy]; *abdominal pain/dt [Drug Therapy]; *abdominal pain/su [Surgery]; adult; alcohol abuse; alcoholic pancreatitis; antibiotic sensitivity; article; ascites; biliary tract infection/dt [Drug Therapy]; blood cell count; case report; cholecystectomy; cold clammy skin; computer assisted tomography; cytology; diabetes mellitus; disease course; Enterobacter; Enterobacter cloacae; erythrocyte transfusion; esophagojejunostomy; follow up; fungus hyphae; gallbladder disease/di [Diagnosis]; gallbladder disease/su [Surgery]; gastrectomy; gastrointestinal endoscopy; *gastrointestinal infection/di [Diagnosis]; *gastrointestinal infection/dt [Drug Therapy]; *gastrointestinal mucormycosis/di [Diagnosis]; *gastrointestinal mucormycosis/dt [Drug Therapy]; hemoperitoneum; histopathology; human; human tissue; jejunostomy; laboratory test; laparotomy; lung tuberculosis; male; *mucormycosis/di [Diagnosis]; *mucormycosis/dt [Drug Therapy]; pallor; peritoneal fluid; peritonitis; stomach biopsy; stomach perforation/di [Diagnosis]; stomach perforation/su [Surgery]; stomach ulcer; thorax radiography; tissue culture; total stomach resection; treatment outcome; vomiting; wound infection/dt [Drug Therapy]; amoxicillin plus clavulanic acid/dt [Drug Therapy]; amoxicillin plus clavulanic acid/po [Oral Drug Administration]; amphotericin B/dt [Drug Therapy]; amphotericin B/iv [Intravenous Drug Administration]; aztreonam; cefepime/dt [Drug Therapy]; cefoxitin/iv [Intravenous Drug Administration]; ceftazidime; ceftriaxone; ciprofloxacin; esomeprazole/dt [Drug Therapy]; fentanyl/iv [Intravenous Drug Administration]; fluconazole; fresh frozen plasma; gentamicin/dt [Drug Therapy]; heparin/sc [Subcutaneous Drug Administration]; insulin glargine; meropenem; metoclopramide/dt [Drug Therapy]; metoprolol; metronidazole/dt [Drug Therapy]; metronidazole/po [Oral Drug Administration]; midazolam/iv [Intravenous Drug Administration]; morphine/dt [Drug Therapy]; moxifloxacin; piperacillin; posaconazole/dt [Drug Therapy]; posaconazole/po [Oral Drug Administration]; sulfamethoxazole; sultamicillin; trimethoprim.

Link to the Ovid Full Text or citation:

Click here for full text options

85.

Incidence of fever of unknown origin and subsequent antitubercular medications in hemodialysis patients: A two-year prospective study.

Okada R., Yuzawa Y., Kawamura T., Hamajima N., Watanabe Y., Matsuo S.

Renal Failure. 31 (10) (pp 863-868), 2009. Date of Publication: November 2009.

AN: 2010028104

Background. Hemodialysis (HD) patients are susceptible to atypical tuberculosis (TB), especially among patients presenting with fever of unknown origin (FUO), because of their impaired cellular immunity. Diagnostic trials of anti-TB drugs are therefore recommended in some TB endemic countries, including Japan, though clinical evidence for this therapy is scarce. Methods. We prospectively collected data for incident cases of clinical FUO for two years in 78 of 169 dialysis facilities in Aichi prefecture, located in central Japan. Clinical FUO was defined as sustained fever without any localizing signs and no infiltration on chest x-rays after a one-week antibiotic trial. The baseline characteristics, subsequent body temperatures on the days of HD therapy, and names of antibiotics including anti-TB drugs with the durations of medication were reported until fever alleviation or fever sustainment for over eight weeks. Results. We identified 15 newly developed clinical FUO patients among 8,125 HD patients. The incidence rate was estimated to be 92 (95% CI, 26-158) per 100,000 person-years. This corresponds to 244 cases per year among 264,473 HD patients in Japan. Anti-TB drugs were secondarily prescribed in 8 of 15 clinical FUO patients (53%). No improved fever alleviation was observed when anti-TB drugs were secondarily prescribed compared with cases in which other antibiotics were preferred. Conclusion. We investigated the incidence of FUO in HD patients and found that the rate was not very high, whereas anti-TB drugs were frequently used for FUO cases. The efficacy of this diagnostic therapy should be elucidated in large-scale studies. © 2009 Informa UK, Ltd.

Institution

(Okada, Hamajima) Department of Preventive Medicine/Biostatistics and Medical Decision Making, Nagoya University, Graduate School of Medicine, 65 Tsurumai-cho, Showa-ku, Nagoya 466-8550, Japan (Yuzawa, Matsuo) Department of Nephrology, Nagoya University, Graduate School of Medicine, 65 Tsurumai-cho, Showa-ku, Nagoya, Japan

(Kawamura) Kyoto University, Health Service, Yoshida-Honmachi, Sakyo-ku, Kyoto, Japan

(Watanabe) Aichi Prefectural Association of Dialysis Physicians, 2 Kurobotoke, Momojima-cho, Tsushima, Aichi, Japan

Publisher

Informa Healthcare (69-77 Paul Street, London EC2A 4LQ, United Kingdom)

Emtree Heading

adult; aged; antibiotic therapy; article; atypical mycobacteriosis/di [Diagnosis]; atypical mycobacteriosis/dt [Drug Therapy]; bacterial infection/dt [Drug Therapy]; body temperature; clinical article; comorbidity; diabetic nephropathy/co [Complication]; diabetic nephropathy/th [Therapy]; drug efficacy; female; hemodialysis; *hemodialysis patient; human; incidence; Japan; Kaplan Meier method; male; pleura effusion; priority journal; prospective study; *pyrexia idiopathica/et [Etiology]; survival rate; thorax radiography; treatment outcome; *tuberculosis/di [Diagnosis]; *tuberculosis/dt [Drug Therapy]; antibiotic agent/dt [Drug Therapy]; tuberculostatic agent/dt [Drug Therapy].

Link to the Ovid Full Text or citation:

Click here for full text options

86.

A series of critically challenging case scenarios in moderate to severe psoriasis: A Delphi consensus approach.

Strober B., Berger E., Cather J., Cohen D., Crowley J.J., Gordon K.B., Gottlieb A., Horn E.J., Kavanaugh A.F., Korman N.J., Krueger G.G., Leonardi C.L., Menter A., Schwartzman S., Sobell J.M., Young M.

Journal of the American Academy of Dermatology. 61 (1 SUPPL. 1) (pp S1-S46), 2009. Date of Publication: July 2009.

AN: 2009324737

Clinical trials for systemic psoriasis therapy typically enroll healthy patients and exclude patients with cardiovascular disease, latent tuberculosis, liver disease, histories of malignancies, viral infections, children, and pregnant or breast-feeding women. Physicians often require guidance for optimum management of severe psoriasis in patients that have a combination of underlying disease states. To provide treatment recommendations for complex psoriasis scenarios, a consensus panel comprising 15 experts in psoriatic disease convened to review and discuss available evidence-based data and to arrive at a consensus for treatment options of difficult cases. An application of the Delphi Method was used to select case scenarios, provide medical treatment options, present the case study with existing medical evidence, and anonymously vote on treatment options. The top 10 treatment options were ranked and statistically analyzed to compare the differences between treatments. The final rankings and analysis provide guidance for practical, safe, and efficacious treatment options in a number of complex psoriasis scenarios. © 2009 American Academy of Dermatology, Inc.

Institution

(Strober, Cohen) Department of Dermatology, New York University Medical Center, New York, NY, United States (Berger, Gottlieb, Sobell) Department of Dermatology, Tufts University-New England Medical Center, Boston, MA, United States

(Korman, Young) Department of Dermatology, Case Western Reserve University, Cleveland, OH, United States

(Krueger) Department of Dermatology, University of Utah School of Medicine, Salt Lake City, UT, United States

(Leonardi) Department of Dermatology, Saint Louis University School of Medicine, Saint Louis, MO, United States

(Cather) Modern Dermatology, Dallas, TX, United States

(Crowley) Bakersfield Dermatology and Skin Cancer Medical Group, Bakersfield, CA, United States

(Gordon) ENH Medical Group, Skokie, IL, United States

(Horn) National Psoriasis Foundation, Portland, OR, United States

(Kavanaugh) Center for Innovative Therapy, University of California, San Diego, Medical Center, San Diego, CA, United States

(Menter) Baylor University Medical Center, Dallas, TX, United States

(Schwartzman) Department of Clinical Medicine, Weil Cornell Medical College, New York Presbyterian Hospital, New York, NY, United States

Publisher

Mosby Inc. (11830 Westline Industrial Drive, St. Louis MO 63146, United States)

Emtree Heading

abdominal obesity; absence of side effects/si [Side Effect]; accidental injury/si [Side Effect]; alanine aminotransferase blood level; aminotransferase blood level; amyloidosis/dt [Drug Therapy]; anemia/si [Side Effect]; ankylosing spondylitis/dt [Drug Therapy]; anus atresia/si [Side Effect]; article; aseptic meningitis/si [Side Effect]; aspartate aminotransferase blood level; atopic dermatitis/dt [Drug Therapy]; B cell lymphoma/si [Side Effect]; biological therapy; birth defect/co [Complication]; blastomycosis/dt [Drug Therapy]; body mass; breast feeding education; case study; climatotherapy; clinical trial; coccidioidomycosis/si [Side Effect]; common cold/si [Side Effect]; comorbidity; congestive heart failure/si [Side Effect]; consensus; convulsion/si [Side Effect]; Crohn disease/dt [Drug Therapy]; cutaneous T cell lymphoma/et [Etiology]; Delphi study; demyelinating disease; disease severity; dizziness/si [Side Effect]; drug bioavailability; drug cost; drug dose comparison; drug dose reduction; drug efficacy; drug excretion; drug fatality/si [Side Effect]; drug half life; drug megadose; drug safety; drug tolerability; drug treatment failure; drug withdrawal; enteritis/dt [Drug Therapy]; esophagus atresia/si [Side Effect]; evidence based medicine; evidence based practice; excimer laser; experimental study; headache/si [Side Effect]; health care cost; health insurance; health program; hepatitis B/co [Complication]; hepatitis B/di [Diagnosis]; hepatitis B/et [Etiology]; hepatitis B/pc [Prevention]; Hepatitis B virus; hepatitis C/dt [Drug Therapy]; hepatitis C/si [Side Effect]; high risk patient; histoplasmosis/si [Side Effect]; human; hyperkalemia/si [Side Effect]; hypersensitivity reaction/si [Side Effect]; hypertension/si [Side Effect]; immune response; immunosuppressive treatment; infection/si [Side Effect]; injection site reaction/si [Side Effect]; juvenile rheumatoid arthritis/dt [Drug Therapy]; kidney dysfunction/si [Side Effect]; kidney malformation/si [Side Effect]; listeriosis/si [Side Effect]; liver cirrhosis/si [Side Effect]; liver failure/si [Side Effect]; liver fibrosis/di [Diagnosis]; liver fibrosis/et [Etiology]; liver fibrosis/si [Side Effect]; liver function test; liver injury/si [Side Effect]; liver toxicity/si [Side Effect]; low drug dose; lung cancer/si [Side Effect]; lymphoma/si [Side Effect]; medical personnel; medical research; melanoma/di [Diagnosis]; melanoma/dt [Drug Therapy]; melanoma/et [Etiology]; melanoma/si [Side Effect]; metabolic syndrome X; monotherapy; myalgia/si [Side Effect]; mycosis/si [Side Effect]; non insulin dependent diabetes mellitus; nonhodgkin lymphoma/et [Etiology]; nonhodgkin lymphoma/si [Side Effect]; nonhuman; opportunistic infection/si [Side Effect]; pharyngitis/si [Side Effect]; phototherapy; polyneuropathy/si [Side Effect]; polyradiculoneuropathy/si [Side Effect]; pregnancy; priority journal; progressive multifocal leukoencephalopathy/si [Side Effect]; prophylaxis; *psoriasis/dm [Disease Management]; *psoriasis/dt [Drug Therapy]; *psoriasis/th [Therapy]; psoriasis vulgaris/dt [Drug Therapy]; psoriatic arthritis/dt [Drug Therapy]; PUVA; pyoderma/dt [Drug Therapy]; rash/si [Side Effect]; relaxation training; rheumatoid arthritis/dt [Drug Therapy]; seizure/si [Side Effect]; sensorimotor neuropathy/si [Side Effect]; sepsis/si [Side Effect]; serum sickness/si [Side Effect]; side effect/si [Side Effect]; skin carcinoma/si [Side Effect]; solid tumor/si [Side Effect]; spontaneous abortion/si [Side Effect]; squamous cell carcinoma/si [Side Effect]; teratogenesis; tracheoesophageal fistula/si [Side Effect]; treatment duration; treatment response; tremor/si [Side Effect]; tuberculosis/di [Diagnosis]; tuberculosis/et [Etiology]; tuberculosis/pc [Prevention]; tuberculosis/si [Side Effect]; ultraviolet A radiation; ultraviolet B radiation; uveitis/dt [Drug Therapy]; vaccination; vertebra malformation/si [Side Effect]; virus meningitis/si [Side Effect]; virus reactivation; abatacept/ct [Clinical Trial]; abatacept/dt [Drug Therapy]; abt 874; adalimumab/ae [Adverse Drug Reaction]; adalimumab/ct [Clinical Trial]; adalimumab/cb [Drug Combination]; adalimumab/cm [Drug Comparison]; adalimumab/dt [Drug Therapy]; adalimumab/pe [Pharmacoeconomics]; adalimumab/sc [Subcutaneous Drug Administration]; alefacept/ae [Adverse Drug Reaction]; alefacept/ct [Clinical Trial]; alefacept/dt [Drug Therapy]; alefacept/pe [Pharmacoeconomics]; *antipsoriasis agent; azathioprine/ae [Adverse Drug Reaction]; azathioprine/ct [Clinical Trial]; azathioprine/cb [Drug Combination]; azathioprine/cm [Drug Comparison]; azathioprine/dt [Drug Therapy]; calcipotriol/ae [Adverse Drug Reaction]; calcipotriol/ct [Clinical Trial]; calcipotriol/cb [Drug Combination]; calcipotriol/dt [Drug Therapy]; calcipotriol/to [Drug Toxicity]; calcipotriol/pe [Pharmacoeconomics]; calcipotriol/tp [Topical Drug Administration]; chickenpox vaccine; coal tar/ae [Adverse Drug Reaction]; coal tar/ct [Clinical Trial]; coal tar/dt [Drug Therapy]; corticosteroid/ae [Adverse Drug Reaction]; corticosteroid/ct [Clinical Trial]; corticosteroid/cb [Drug Combination]; corticosteroid/dt [Drug Therapy]; corticosteroid/ar [Intraarticular Drug Administration]; corticosteroid/il [Intralesional Drug Administration]; corticosteroid/pe [Pharmacoeconomics]; corticosteroid/tp [Topical Drug Administration]; cyclosporin/ae [Adverse Drug Reaction]; cyclosporin/ct [Clinical Trial]; cyclosporin/cb [Drug Combination]; cyclosporin/cm [Drug Comparison]; cyclosporin/dt [Drug Therapy]; cyclosporin/pk [Pharmacokinetics]; dithranol/ct [Clinical Trial]; dithranol/dt [Drug Therapy]; dithranol/tp [Topical Drug Administration]; efalizumab/ae [Adverse Drug Reaction]; efalizumab/ct [Clinical Trial]; efalizumab/dt [Drug Therapy]; efalizumab/pe [Pharmacoeconomics]; efalizumab/pk [Pharmacokinetics]; etanercept/ae [Adverse Drug Reaction]; etanercept/ct [Clinical Trial]; etanercept/cb [Drug Combination]; etanercept/cm [Drug Comparison]; etanercept/dt [Drug Therapy]; etanercept/pe [Pharmacoeconomics]; etanercept/sc [Subcutaneous Drug Administration]; etretin/ae [Adverse Drug Reaction]; etretin/ct [Clinical Trial]; etretin/cb [Drug Combination]; etretin/do [Drug Dose]; etretin/dt [Drug Therapy]; etretin/po [Oral Drug Administration]; etretin/pe [Pharmacoeconomics]; hydroxychloroquine/ct [Clinical Trial]; hydroxychloroquine/dt [Drug Therapy]; hydroxyurea/ct [Clinical Trial]; hydroxyurea/cb [Drug Combination]; hydroxyurea/dt [Drug Therapy]; hydroxyurea/pe [Pharmacoeconomics]; infliximab/ae [Adverse Drug Reaction]; infliximab/ct [Clinical Trial]; infliximab/cb [Drug Combination]; infliximab/cm [Drug Comparison]; infliximab/dt [Drug Therapy]; infliximab/pe [Pharmacoeconomics]; leflunomide/ct [Clinical Trial]; leflunomide/dt [Drug Therapy]; mercaptopurine/ct [Clinical Trial]; mercaptopurine/dt [Drug Therapy]; methotrexate/ae [Adverse Drug Reaction]; methotrexate/ct [Clinical Trial]; methotrexate/cb [Drug Combination]; methotrexate/cm [Drug Comparison]; methotrexate/do [Drug Dose]; methotrexate/dt [Drug Therapy]; methotrexate/im [Intramuscular Drug Administration]; methotrexate/po [Oral Drug Administration]; methotrexate/pe [Pharmacoeconomics]; methotrexate/pk [Pharmacokinetics]; methotrexate/sc [Subcutaneous Drug Administration]; mycophenolic acid 2 morpholinoethyl ester/ae [Adverse Drug Reaction]; mycophenolic acid 2 morpholinoethyl ester/ct [Clinical Trial]; mycophenolic acid 2 morpholinoethyl ester/cm [Drug Comparison]; mycophenolic acid 2 morpholinoethyl ester/dt [Drug Therapy]; pimecrolimus/ae [Adverse Drug Reaction]; pimecrolimus/ct [Clinical Trial]; pimecrolimus/dt [Drug Therapy]; pimecrolimus/tp [Topical Drug Administration]; placebo; rituximab/ct [Clinical Trial]; rituximab/dt [Drug Therapy]; salazosulfapyridine/ct [Clinical Trial]; salazosulfapyridine/dt [Drug Therapy]; salazosulfapyridine/pe [Pharmacoeconomics]; tacrolimus/ae [Adverse Drug Reaction]; tacrolimus/ct [Clinical Trial]; tacrolimus/cm [Drug Comparison]; tacrolimus/dt [Drug Therapy]; tacrolimus/po [Oral Drug Administration]; tacrolimus/tp [Topical Drug Administration]; tazarotene/ct [Clinical Trial]; tazarotene/cb [Drug Combination]; tazarotene/dt [Drug Therapy]; tazarotene/tp [Topical Drug Administration]; tioguanine/ct [Clinical Trial]; tioguanine/dt [Drug Therapy]; tumor necrosis factor inhibitor/ae [Adverse Drug Reaction]; tumor necrosis factor inhibitor/ct [Clinical Trial]; tumor necrosis factor inhibitor/cb [Drug Combination]; tumor necrosis factor inhibitor/cm [Drug Comparison]; tumor necrosis factor inhibitor/dt [Drug Therapy]; unindexed drug; ustekinumab/ct [Clinical Trial]; ustekinumab/dt [Drug Therapy]; varicella zoster vaccine.

Drug Trade Names and Manufacturers

Ingram, Merck [United States], abt 874, varivax, zostavax: Merck [United States]

Link to the Ovid Full Text or citation:

Click here for full text options

87.

Treatment of ankylosing spondylitis and other spondyloarthritides.

Braun J., Baraliakos X.

Current Opinion in Rheumatology. 21 (4) (pp 324-334), 2009. Date of Publication: July 2009.

AN: 2009293293

Purpose of review: The management of ankylosing spondylitis and other spondyloarthritides (SpAs) such as psoriatic arthritis (PsA) is becoming more and more complex due to major advances, especially in the pharmacological therapy of these diseases. Biologics such as the anti-tumour necrosis factor agents have the potential to improve almost all outcome parameters usually assessed. The purpose of this review was to collect all available information related to the treatment of ankylosing spondylitis published in the year 2008. This was achieved by a systematic literature search in PubMed. Recent findings: There is increasing knowledge about the short-term and long-term efficacy of anti-tumour necrosis factor agents. As it stands now, radiographic progression of ankylosing spondylitis patients is not decelerated, in contrast to PsA. One major difference between the two SpAs is that ankylosing spondylitis is more an osteoproliferative disease, whereas PsA is more osteodestructive (though it certainly has both elements). Methotrexate offers no additional benefit for the axial symptoms in ankylosing spondylitis. Other biologics such as efalizumab, which is used to treat psoriasis, seems to increase the frequency of arthritis. A whole array of potential side effects has been reported. Cardiovascular comorbidity seems to be rather important for SpA patients. Summary: In this review, we especially cover not only the following themes of new clinical studies, efficacy regarding signs and symptoms, function and quality of life but also structural damage and the link between inflammation and new bone formation. The pharmacokinetics of infliximab including the formation of antibodies and the overall and long-term safety of anti-tumour necrosis factor agents is covered. © 2009 Wolters Kluwer Health Lippincott Williams & Wilkins.

Institution

(Braun, Baraliakos) Rheumazentrum Ruhrgebiet, Herne, Germany (Braun) Rheumazentrum Ruhrgebiet, Landgrafenstr. 15, 44652 Herne, Germany

Publisher

Lippincott Williams and Wilkins (530 Walnut Street,P O Box 327, Philadelphia PA 19106-3621, United States)

Emtree Heading

acute granulocytic leukemia/si [Side Effect]; add on therapy; alopecia areata/si [Side Effect]; amyotrophic lateral sclerosis/si [Side Effect]; *ankylosing spondylitis/dm [Disease Management]; *ankylosing spondylitis/dt [Drug Therapy]; *ankylosing spondylitis/rh [Rehabilitation]; *ankylosing spondylitis/su [Surgery]; *ankylosing spondylitis/th [Therapy]; ankylosis/su [Surgery]; antibody producing cell; antiinflammatory activity; arthritis/si [Side Effect]; aspergillosis/si [Side Effect]; biological therapy; cardiovascular risk; cauda equina syndrome; central nervous system infection/si [Side Effect]; clinical trial; cold injury/si [Side Effect]; comorbidity; coronary artery bypass graft; corticosteroid therapy; cost effectiveness analysis; Crohn disease/si [Side Effect]; dermatitis herpetiformis/si [Side Effect]; diabetes mellitus; diet therapy; disease course; disease exacerbation; drug effect; drug efficacy; drug megadose; drug safety; drug targeting; drug withdrawal; enthesitis/si [Side Effect]; erythema nodosum/dt [Drug Therapy]; functional assessment; group therapy; histopathology; home care; human; hypertension; immunotherapy; iridocyclitis/si [Side Effect]; keratoderma blennorrhagica/si [Side Effect]; kinesiotherapy; lichen planupilaris/si [Side Effect]; lupus vulgaris/si [Side Effect]; Medline; monarthritis/dt [Drug Therapy]; monarthritis/si [Side Effect]; multiple sclerosis/si [Side Effect]; myelitis/dt [Drug Therapy]; myelitis/si [Side Effect]; myeloid leukemia/si [Side Effect]; nevus/si [Side Effect]; nonhuman; nuclear magnetic resonance imaging; ossification; osteotomy; patient education; peripheral neuropathy/si [Side Effect]; physical capacity; physiotherapy; priority journal; prognosis; psoriasis/dt [Drug Therapy]; psoriasis/si [Side Effect]; psoriasis vulgaris/dt [Drug Therapy]; psoriasis vulgaris/si [Side Effect]; psoriatic arthritis/dr [Drug Resistance]; psoriatic arthritis/dt [Drug Therapy]; psoriatic arthritis/si [Side Effect]; pustulosis/si [Side Effect]; pustulosis palmoplantaris/dt [Drug Therapy]; pustulosis palmoplantaris/si [Side Effect]; quality of life; religion; remission; review; rheumatic disease/dt [Drug Therapy]; rheumatic disease/si [Side Effect]; rheumatoid arthritis/dt [Drug Therapy]; sacroiliitis/dt [Drug Therapy]; sarcoidosis/si [Side Effect]; skin toxicity/si [Side Effect]; spine surgery; *spondyloarthropathy/dm [Disease Management]; *spondyloarthropathy/dr [Drug Resistance]; *spondyloarthropathy/dt [Drug Therapy]; *spondyloarthropathy/rh [Rehabilitation]; *spondyloarthropathy/su [Surgery]; *spondyloarthropathy/th [Therapy]; symptomatology; systematic review; Tai Chi; temporomandibular joint disorder/dr [Drug Resistance]; temporomandibular joint disorder/dt [Drug Therapy]; total hip prosthesis; treatment duration; treatment outcome; treatment planning; treatment response; adalimumab/ae [Adverse Drug Reaction]; adalimumab/ct [Clinical Trial]; adalimumab/dt [Drug Therapy]; antinuclear antibody/ec [Endogenous Compound]; bisphosphonic acid derivative/pd [Pharmacology]; C reactive protein/ec [Endogenous Compound]; celecoxib/dt [Drug Therapy]; certolizumab pegol; corticosteroid/dt [Drug Therapy]; double stranded DNA antibody/ec [Endogenous Compound]; efalizumab/ae [Adverse Drug Reaction]; efalizumab/dt [Drug Therapy]; etanercept/ae [Adverse Drug Reaction]; etanercept/dt [Drug Therapy]; etanercept/pd [Pharmacology]; etoricoxib/dt [Drug Therapy]; glucocorticoid/dt [Drug Therapy]; glucocorticoid/ar [Intraarticular Drug Administration]; golimumab/ct [Clinical Trial]; golimumab/dt [Drug Therapy]; hydroxymethylglutaryl coenzyme A reductase inhibitor/pd [Pharmacology]; infliximab/ae [Adverse Drug Reaction]; infliximab/cb [Drug Combination]; infliximab/dt [Drug Therapy]; infliximab/ar [Intraarticular Drug Administration]; low density lipoprotein cholesterol/ec [Endogenous Compound]; methotrexate/cb [Drug Combination]; methotrexate/dt [Drug Therapy]; nonsteroid antiinflammatory agent/dt [Drug Therapy]; placebo; radium 224/ae [Adverse Drug Reaction]; radium 224/dt [Drug Therapy]; rituximab/dt [Drug Therapy]; tumor necrosis factor inhibitor/ae [Adverse Drug Reaction]; tumor necrosis factor inhibitor/ct [Clinical Trial]; tumor necrosis factor inhibitor/cb [Drug Combination]; tumor necrosis factor inhibitor/dt [Drug Therapy].

Link to the Ovid Full Text or citation:

Click here for full text options

88.

Treatment of HIV infection: Swedish recommendations 2009.

Josephson F., Albert J., Flamholc L., Gissln M., Karlstrm O., Moberg L., Navr L., Svedhem V., Svennerholm B., Snnerborg A.

Scandinavian Journal of Infectious Diseases. 41 (11-12) (pp 788-807), 2009. Date of Publication: 2009.

AN: 2009608846

On 4 previous occasions, in 2002, 2003, 2005 and 2007, the Swedish Medical Products Agency (Lkemedelsverket) and the Swedish Reference Group for Antiviral Therapy (RAV) have jointly published recommendations for the treatment of HIV infection. In November 2008, an expert group under the guidance of RAV once more revised the guidelines, of which this is a translation into English. The most important updates in the present guidelines include the following: (a) treatment initiation is now recommended at a CD4 cell count of approximately 350/l; (b) new recommendations for first-line therapy: abacavir/lamivudine or tenofovir/emtricitabine in combination with efavirenz or a boosted protease inhibitor (PI/r); (c) an increased focus on reducing the use of antiretroviral drugs that may cause lipoatrophy; (d) an emphasis on quality assurance of HIV care through the use of InfCare HIV; (e) considerably altered recommendations for the initiation of antiretroviral therapy in children. All infants (<1 y) should start antiretroviral therapy, regardless of immune status. Also, absolute CD4+ cell counts, rather than percentage, may be used to guide treatment initiation in children above the age of 5 y.

Institution

(Josephson) Department of Clinical Pharmacology, Karolinska University Hospital at Huddinge, SE-141 86, Stockholm, Sweden (Albert) Department of Microbiology, Tumour and Cell Biology (MTC), Karolinska Institutet

(Albert) Department of Virology, Immunology and Vaccinology, Swedish Institute for Infectious Disease Control (SMI)

(Flamholc) Department of Infectious Diseases, Malm University Hospital

(Gissln) Department of Infectious Diseases, Sahlgrenska University Hospital, Gteborg

(Karlstrm) Medical Products Agency, Uppsala, Sweden

(Karlstrm, Svedhem, Snnerborg) Department of Infectious Diseases, Karolinska University Hospital

(Moberg) Department of Venereology, South Hospital, Stockholm, Sweden

(Navr) Department of Paediatrics, Karolinska University Hospital

(Svennerholm) Department of Clinical Virology, Sahlgrenska University Hospital, Gteborg

(Snnerborg) Department of Clinical Virology, Karolinska University Hospital, Huddinge, Sweden

Publisher

Informa Healthcare (69-77 Paul Street, London EC2A 4LQ, United Kingdom)

Emtree Heading

absence of side effects/si [Side Effect]; anemia/si [Side Effect]; antiviral therapy; arthralgia/si [Side Effect]; bacterial infection/si [Side Effect]; cardiomyopathy/si [Side Effect]; cardiovascular disease/si [Side Effect]; CD4 lymphocyte count; CD4+ T lymphocyte; central nervous system disease/si [Side Effect]; child care; clinical trial; combination chemotherapy; cost effectiveness analysis; dermatitis/si [Side Effect]; diarrhea/si [Side Effect]; drug contraindication; drug cost; drug dose regimen; drug efficacy; drug monitoring; drug receptor binding; drug safety; drug tolerability; drug treatment failure; drug withdrawal; extrapulmonary tuberculosis/si [Side Effect]; Fanconi renotubular syndrome/si [Side Effect]; fever/si [Side Effect]; follow up; food drug interaction; gene mutation; glomerulus filtration rate; hepatitis/si [Side Effect]; hepatitis A; Hepatitis A virus; hepatitis B; Hepatitis B virus; hepatitis C; hepatomegaly/si [Side Effect]; herpes zoster/si [Side Effect]; *highly active antiretroviral therapy; human; *Human immunodeficiency virus 1; *Human immunodeficiency virus 1 infection/di [Diagnosis]; *Human immunodeficiency virus 1 infection/dm [Disease Management]; *Human immunodeficiency virus 1 infection/dr [Drug Resistance]; *Human immunodeficiency virus 1 infection/dt [Drug Therapy]; *Human immunodeficiency virus 1 infection/ep [Epidemiology]; *Human immunodeficiency virus 1 infection/et [Etiology]; Human immunodeficiency virus infected patient; hyperlipidemia/si [Side Effect]; hypersensitivity reaction/si [Side Effect]; immune status; insulin resistance/si [Side Effect]; interstitial pneumonia/si [Side Effect]; Kaposi sarcoma/si [Side Effect]; kidney disease/si [Side Effect]; kidney failure/si [Side Effect]; laboratory test; lactic acidosis/si [Side Effect]; lipoatrophy/si [Side Effect]; lipohypertrophy/si [Side Effect]; liver injury/si [Side Effect]; liver toxicity/si [Side Effect]; lymphadenopathy/si [Side Effect]; lymphoma/si [Side Effect]; metabolic disorder/si [Side Effect]; mitochondrial toxicity/si [Side Effect]; monotherapy; mother child relation; myalgia/si [Side Effect]; nephrotoxicity/si [Side Effect]; neutropenia/si [Side Effect]; non insulin dependent diabetes mellitus/si [Side Effect]; parotitis/si [Side Effect]; patient compliance; Pneumocystis pneumonia/si [Side Effect]; pneumocystosis/pc [Prevention]; practice guideline; pregnancy; progressive multifocal leukoencephalopathy/si [Side Effect]; prophylaxis; quality control; rash/si [Side Effect]; recommended drug dose; review; RNA virus; Stevens Johnson syndrome/si [Side Effect]; Sweden; teratogenicity/si [Side Effect]; thrombocytopenia/si [Side Effect]; thrush/si [Side Effect]; toxic epidermal necrolysis/si [Side Effect]; toxoplasmosis/si [Side Effect]; treatment contraindication; treatment duration; treatment indication; tubular dysfunction/si [Side Effect]; urinary tract infection/si [Side Effect]; vaccination; virus infection/si [Side Effect]; virus load; virus replication; wasting syndrome/si [Side Effect]; abacavir/ct [Clinical Trial]; abacavir/cm [Drug Comparison]; abacavir/dt [Drug Therapy]; abacavir plus lamivudine/ae [Adverse Drug Reaction]; abacavir plus lamivudine/ct [Clinical Trial]; abacavir plus lamivudine/cm [Drug Comparison]; abacavir plus lamivudine/dt [Drug Therapy]; abacavir plus lamivudine/pe [Pharmacoeconomics]; amprenavir phosphate/ct [Clinical Trial]; amprenavir phosphate/dt [Drug Therapy]; *antiretrovirus agent/ae [Adverse Drug Reaction]; atazanavir/ae [Adverse Drug Reaction]; atazanavir/ct [Clinical Trial]; atazanavir/dt [Drug Therapy]; atazanavir plus ritonavir/ct [Clinical Trial]; atazanavir plus ritonavir/cb [Drug Combination]; atazanavir plus ritonavir/dt [Drug Therapy]; darunavir/ct [Clinical Trial]; darunavir/dt [Drug Therapy]; darunavir plus ritonavir/ct [Clinical Trial]; darunavir plus ritonavir/cm [Drug Comparison]; darunavir plus ritonavir/dt [Drug Therapy]; didanosine/ae [Adverse Drug Reaction]; didanosine/ct [Clinical Trial]; didanosine/cb [Drug Combination]; didanosine/dt [Drug Therapy]; efavirenz/ae [Adverse Drug Reaction]; efavirenz/ct [Clinical Trial]; efavirenz/cb [Drug Combination]; efavirenz/cm [Drug Comparison]; efavirenz/do [Drug Dose]; efavirenz/dt [Drug Therapy]; efavirenz plus emtricitabine plus tenofovir disoproxil/ct [Clinical Trial]; efavirenz plus emtricitabine plus tenofovir disoproxil/dt [Drug Therapy]; emtricitabine/ct [Clinical Trial]; emtricitabine/cb [Drug Combination]; emtricitabine/dt [Drug Therapy]; emtricitabine plus tenofovir disoproxil/ae [Adverse Drug Reaction]; emtricitabine plus tenofovir disoproxil/ct [Clinical Trial]; emtricitabine plus tenofovir disoproxil/cb [Drug Combination]; emtricitabine plus tenofovir disoproxil/cm [Drug Comparison]; emtricitabine plus tenofovir disoproxil/dt [Drug Therapy]; emtricitabine plus tenofovir disoproxil/pe [Pharmacoeconomics]; etravirine/ae [Adverse Drug Reaction]; etravirine/ct [Clinical Trial]; etravirine/dt [Drug Therapy]; indinavir/ct [Clinical Trial]; indinavir/dt [Drug Therapy]; lamivudine/ct [Clinical Trial]; lamivudine/cb [Drug Combination]; lamivudine/dt [Drug Therapy]; lamivudine plus zidovudine; lopinavir/ct [Clinical Trial]; lopinavir/dt [Drug Therapy]; lopinavir plus ritonavir/ae [Adverse Drug Reaction]; lopinavir plus ritonavir/ct [Clinical Trial]; lopinavir plus ritonavir/cb [Drug Combination]; lopinavir plus ritonavir/cm [Drug Comparison]; lopinavir plus ritonavir/dt [Drug Therapy]; maraviroc/ct [Clinical Trial]; maraviroc/cm [Drug Comparison]; maraviroc/dt [Drug Therapy]; maraviroc/pd [Pharmacology]; nelfinavir/ct [Clinical Trial]; nelfinavir/cb [Drug Combination]; nelfinavir/dt [Drug Therapy]; nevirapine/ae [Adverse Drug Reaction]; nevirapine/ct [Clinical Trial]; nevirapine/dt [Drug Therapy]; placebo; raltegravir/ae [Adverse Drug Reaction]; raltegravir/ct [Clinical Trial]; raltegravir/dt [Drug Therapy]; ritonavir/ct [Clinical Trial]; ritonavir/dt [Drug Therapy]; saquinavir/ct [Clinical Trial]; saquinavir/dt [Drug Therapy]; stavudine/ae [Adverse Drug Reaction]; stavudine/ct [Clinical Trial]; stavudine/cb [Drug Combination]; stavudine/dt [Drug Therapy]; tenofovir/ae [Adverse Drug Reaction]; tenofovir/ct [Clinical Trial]; tenofovir/cm [Drug Comparison]; tenofovir/dt [Drug Therapy]; tenofovir disoproxil; tipranavir; unindexed drug; virus RNA/ec [Endogenous Compound]; zidovudine/ae [Adverse Drug Reaction]; zidovudine/ct [Clinical Trial]; zidovudine/cb [Drug Combination]; zidovudine/dt [Drug Therapy].

Drug Trade Names and Manufacturers

aptivus, atripla, celsentri, combivir, intelence, isentress, kivexa, norvir, prezista, retrovir, sustiva, truvada, viread, ziagen

Link to the Ovid Full Text or citation:

Click here for full text options

89.

PENTA 2009 guidelines for the use of antiretroviral therapy in paediatric HIV-1 infection.

Welch S.

HIV Medicine. 10 (10) (pp 591-613), 2009. Date of Publication: November 2009.

AN: 2009590654

PENTA Guidelines aim to provide practical recommendations for treating children with HIV infection in Europe. Changes to guidance since 2004 have been informed by new evidence and by expectations of better outcomes following the ongoing success of antiretroviral therapy (ART). Participation in PENTA trials of simplifying treatment is encouraged. The main changes are in the following sections:'When to start ART': Treatment is recommended for all infants, and at higher CD4 cell counts and percentages in older children, in line with changes to adult guidelines. The number of age bands has been reduced to simplify and harmonize with other paediatric guidelines. Greater emphasis is placed on CD4 cell count in children over 5 years, and guidance is provided where CD4% and CD4 criteria differ.'What to start with': A three-drug regimen of two nucleoside reverse transcriptase inhibitors (NRTIs) with either a nonnucleoside reverse transcriptase inhibitor (NNRTI) or a boosted protease inhibitor (PI) remains the first choice combination. Lamivudine and abacavir are the NRTI backbone of choice for most children, based on long-term follow-up in the PENTA 5 trial. Stavudine is no longer recommended. Whether to start with an NNRTI or PI remains unclear, but PENPACT 1 trial results in 2009 may help to inform this. All PIs should be ritonavir boosted.Recommendations on use of resistance testing, therapeutic drug monitoring and HLA testing draw from data in adults and from European paediatric cohort studies.Recently updated US and WHO paediatric guidelines provide more detailed review of the evidence base. Differences between guidelines are highlighted and explained. © 2009 British HIV Association.

Institution

(Welch) Department of Paediatrics, Heartlands Hospital, Bordesley Green East, Birmingham B9 5SS, United Kingdom

Publisher

Blackwell Publishing Ltd (9600 Garsington Road, Oxford OX4 2XG, United Kingdom)

Emtree Heading

abdominal pain/si [Side Effect]; anemia/si [Side Effect]; antiviral resistance; article; bone demineralization/si [Side Effect]; brain hemorrhage/si [Side Effect]; CD4+ T lymphocyte; child; chronic liver disease/si [Side Effect]; clinical protocol; clinical trial; diabetes mellitus/si [Side Effect]; diagnostic test; diarrhea/si [Side Effect]; drowsiness/si [Side Effect]; drug dose increase; drug efficacy; drug eruption/si [Side Effect]; drug fever/si [Side Effect]; drug formulation; drug induced headache/si [Side Effect]; drug monitoring; drug substitution; drug treatment failure; drug use; drug withdrawal; Europe; fatigue/si [Side Effect]; flatulence/si [Side Effect]; flushing; follow up; gastrointestinal symptom/si [Side Effect]; granulocytopenia/si [Side Effect]; hepatitis/si [Side Effect]; hepatitis B/si [Side Effect]; hepatitis C; human; Human immunodeficiency virus 1; *Human immunodeficiency virus 1 infection/di [Diagnosis]; *Human immunodeficiency virus 1 infection/dt [Drug Therapy]; hypercholesterolemia/si [Side Effect]; hyperlipidemia/si [Side Effect]; jaundice/si [Side Effect]; kidney tubule disorder/si [Side Effect]; lactic acidosis/si [Side Effect]; lipoatrophy/si [Side Effect]; lipodystrophy/si [Side Effect]; liver dysfunction/si [Side Effect]; liver toxicity/si [Side Effect]; lymphocyte count; mood; myopathy/si [Side Effect]; nail pigmentation/si [Side Effect]; nausea/si [Side Effect]; neuropathy/si [Side Effect]; nonhuman; opportunistic infection/dt [Drug Therapy]; opportunistic infection/pc [Prevention]; oral paresthesia/si [Side Effect]; pancreatitis/si [Side Effect]; patient compliance; pediatrics; peripheral neuropathy/si [Side Effect]; Pneumocystis pneumonia/dt [Drug Therapy]; Pneumocystis pneumonia/pc [Prevention]; *practice guideline; priority journal; prophylaxis; pruritus/si [Side Effect]; respiratory tract disease/si [Side Effect]; side effect/si [Side Effect]; skin discoloration/si [Side Effect]; steatosis/si [Side Effect]; Stevens Johnson syndrome/si [Side Effect]; tuberculosis; vivid dream/si [Side Effect]; vomiting/si [Side Effect]; abacavir/ae [Adverse Drug Reaction]; abacavir/ct [Clinical Trial]; abacavir/cb [Drug Combination]; abacavir/do [Drug Dose]; abacavir/dt [Drug Therapy]; abacavir/pr [Pharmaceutics]; abacavir plus lamivudine/ct [Clinical Trial]; abacavir plus lamivudine/do [Drug Dose]; abacavir plus lamivudine/dt [Drug Therapy]; abacavir plus lamivudine plus zidovudine/ct [Clinical Trial]; abacavir plus lamivudine plus zidovudine/do [Drug Dose]; abacavir plus lamivudine plus zidovudine/dt [Drug Therapy]; *antiretrovirus agent/ct [Clinical Trial]; *antiretrovirus agent/dt [Drug Therapy]; atazanavir/ae [Adverse Drug Reaction]; atazanavir/ct [Clinical Trial]; atazanavir/cb [Drug Combination]; atazanavir/do [Drug Dose]; atazanavir/dt [Drug Therapy]; atazanavir/pr [Pharmaceutics]; cotrimoxazole/do [Drug Dose]; cotrimoxazole/dt [Drug Therapy]; darunavir/ae [Adverse Drug Reaction]; darunavir/ct [Clinical Trial]; darunavir/cb [Drug Combination]; darunavir/do [Drug Dose]; darunavir/dt [Drug Therapy]; darunavir/pr [Pharmaceutics]; didanosine/ae [Adverse Drug Reaction]; didanosine/ct [Clinical Trial]; didanosine/cb [Drug Combination]; didanosine/do [Drug Dose]; didanosine/dt [Drug Therapy]; didanosine/pr [Pharmaceutics]; efavirenz/ae [Adverse Drug Reaction]; efavirenz/ct [Clinical Trial]; efavirenz/cb [Drug Combination]; efavirenz/do [Drug Dose]; efavirenz/dt [Drug Therapy]; efavirenz/pr [Pharmaceutics]; efavirenz plus emtricitabine plus tenofovir disoproxil/ct [Clinical Trial]; efavirenz plus emtricitabine plus tenofovir disoproxil/dt [Drug Therapy]; emtricitabine/ae [Adverse Drug Reaction]; emtricitabine/ct [Clinical Trial]; emtricitabine/cb [Drug Combination]; emtricitabine/do [Drug Dose]; emtricitabine/dt [Drug Therapy]; emtricitabine/pr [Pharmaceutics]; emtricitabine plus tenofovir disoproxil/ct [Clinical Trial]; emtricitabine plus tenofovir disoproxil/cb [Drug Combination]; emtricitabine plus tenofovir disoproxil/do [Drug Dose]; emtricitabine plus tenofovir disoproxil/dt [Drug Therapy]; etravirine/ae [Adverse Drug Reaction]; etravirine/ct [Clinical Trial]; etravirine/do [Drug Dose]; etravirine/dt [Drug Therapy]; etravirine/pr [Pharmaceutics]; fosamprenavir/ae [Adverse Drug Reaction]; fosamprenavir/ct [Clinical Trial]; fosamprenavir/cb [Drug Combination]; fosamprenavir/do [Drug Dose]; fosamprenavir/dt [Drug Therapy]; fosamprenavir/pr [Pharmaceutics]; lamivudine/ae [Adverse Drug Reaction]; lamivudine/ct [Clinical Trial]; lamivudine/cb [Drug Combination]; lamivudine/do [Drug Dose]; lamivudine/dt [Drug Therapy]; lamivudine/pr [Pharmaceutics]; lamivudine plus zidovudine/ct [Clinical Trial]; lamivudine plus zidovudine/do [Drug Dose]; lamivudine plus zidovudine/dt [Drug Therapy]; lopinavir plus ritonavir/ae [Adverse Drug Reaction]; lopinavir plus ritonavir/ct [Clinical Trial]; lopinavir plus ritonavir/cb [Drug Combination]; lopinavir plus ritonavir/do [Drug Dose]; lopinavir plus ritonavir/dt [Drug Therapy]; nevirapine/ae [Adverse Drug Reaction]; nevirapine/ct [Clinical Trial]; nevirapine/cb [Drug Combination]; nevirapine/do [Drug Dose]; nevirapine/dt [Drug Therapy]; nevirapine/pr [Pharmaceutics]; nucleoside analog/ct [Clinical Trial]; nucleoside analog/dt [Drug Therapy]; proteinase inhibitor/ae [Adverse Drug Reaction]; proteinase inhibitor/ct [Clinical Trial]; proteinase inhibitor/dt [Drug Therapy]; proteinase inhibitor/pr [Pharmaceutics]; rilpivirine/ct [Clinical Trial]; rilpivirine/do [Drug Dose]; rilpivirine/dt [Drug Therapy]; ritonavir/ae [Adverse Drug Reaction]; ritonavir/ct [Clinical Trial]; ritonavir/cb [Drug Combination]; ritonavir/do [Drug Dose]; ritonavir/dt [Drug Therapy]; ritonavir/pr [Pharmaceutics]; RNA directed DNA polymerase inhibitor/ae [Adverse Drug Reaction]; RNA directed DNA polymerase inhibitor/ct [Clinical Trial]; RNA directed DNA polymerase inhibitor/dt [Drug Therapy]; saquinavir/ae [Adverse Drug Reaction]; saquinavir/ct [Clinical Trial]; saquinavir/cb [Drug Combination]; saquinavir/do [Drug Dose]; saquinavir/dt [Drug Therapy]; saquinavir/pr [Pharmaceutics]; stavudine/ae [Adverse Drug Reaction]; stavudine/ct [Clinical Trial]; stavudine/cb [Drug Combination]; stavudine/do [Drug Dose]; stavudine/dt [Drug Therapy]; stavudine/pr [Pharmaceutics]; tenofovir/ct [Clinical Trial]; tenofovir/cb [Drug Combination]; tenofovir/dt [Drug Therapy]; tenofovir disoproxil/ae [Adverse Drug Reaction]; tenofovir disoproxil/ct [Clinical Trial]; tenofovir disoproxil/do [Drug Dose]; tenofovir disoproxil/dt [Drug Therapy]; tenofovir disoproxil/pr [Pharmaceutics]; tipranavir/ae [Adverse Drug Reaction]; tipranavir/ct [Clinical Trial]; tipranavir/do [Drug Dose]; tipranavir/dt [Drug Therapy]; unindexed drug; zidovudine/ae [Adverse Drug Reaction]; zidovudine/ct [Clinical Trial]; zidovudine/cb [Drug Combination]; zidovudine/do [Drug Dose]; zidovudine/dt [Drug Therapy]; zidovudine/pr [Pharmaceutics].

Drug Trade Names and Manufacturers

Abbott, Bms, Boehringer Ingelheim, Boehringer, Gilead, Glaxo SmithKline, Hoffmann La Roche, Janssen Cilag, Merck Sharp and Dohme, Tibotec, aptivus: Boehringer, atripla: Bms, atripla: Gilead, bactrim: Hoffmann La Roche, combivir: Glaxo SmithKline, emtriva: Gilead, epivir: Glaxo SmithKline, intelence: Janssen Cilag, intelence: Tibotec, invirase: Hoffmann La Roche, kaletra: Abbott, kivexa: Glaxo SmithKline, norvir: Abbott, prezista: Janssen Cilag, prezista: Tibotec, retrovir: Glaxo SmithKline, reyataz: Bms, septrin: Glaxo SmithKline, stocrin: Merck Sharp and Dohme, sustiva: Bms, telzir: Glaxo SmithKline, tmc 125, trizivir: Glaxo SmithKline, truvada: Gilead, videx: Bms, viramune: Boehringer Ingelheim, zeffix: Glaxo SmithKline, zerit: Bms, ziagen: Glaxo SmithKline

Link to the Ovid Full Text or citation:

Click here for full text options

90.

Anti-cytokine antibodies for rheumatic diseases.

Atzeni F., Sarzi-Puttini P.

Current Opinion in Investigational Drugs. 10 (11) (pp 1204-1211), 2009. Date of Publication: November 2009.

AN: 2009585571

Improved knowledge regarding the pathogenetic mechanisms involved in autoimmune diseases has contributed to the development of biological therapies, with advances in cytokine research in particular revolutionizing the treatment of several autoimmune diseases. Anti-TNF agents are the most promising drugs for reducing symptoms, slowing or arresting joint damage, and preventing functional disability in patients with rheumatoid arthritis who fail to respond to standard disease-modifying antirheumatic drugs. Anti-TNF agents may also be useful in the treatment of other rare disorders, such as various forms of vasculitis, polymyositis and dermatomyositis. However, clinical trials of TNF inhibitors and inhibitors of the proinflammatory cytokines IL-1, IL-6, IL-12, IL-15, IL-17 and IL-23 demonstrated that the activity of such compounds is accompanied by a series of adverse events that needs to be addressed. This review describes the efficacy and adverse events associated with anti-cytokine treatments for autoimmune rheumatic diseases.

Institution

(Atzeni, Sarzi-Puttini) L Sacco University Hospital, Rheumatology Unit, via GB Grassi 74, 20127, Milan, Italy

Publisher

Thomson Scientific Ltd. (14 Great Queen Street, London WC2B 5DF, United Kingdom)

Emtree Heading

adverse outcome; allergic asthma/dt [Drug Therapy]; angiofollicular lymph node hyperplasia/dt [Drug Therapy]; ankylosing spondylitis/dt [Drug Therapy]; asthma/dt [Drug Therapy]; autoimmune disease/si [Side Effect]; Behcet disease/dt [Drug Therapy]; cell type; chondritis/dt [Drug Therapy]; chronic obstructive lung disease/dt [Drug Therapy]; Churg Strauss syndrome/dt [Drug Therapy]; clinical feature; clinical trial; colitis/dt [Drug Therapy]; Crohn disease/dt [Drug Therapy]; demyelinating disease/si [Side Effect]; dermatomyositis/dt [Drug Therapy]; drug activity; drug approval; drug binding; drug efficacy; drug half life; drug safety; drug targeting; drug tolerability; drug treatment failure; giant cell arteritis/dt [Drug Therapy]; gout/dt [Drug Therapy]; heart failure/si [Side Effect]; hepatitis/si [Side Effect]; histoplasmosis/si [Side Effect]; human; infection/si [Side Effect]; injection site reaction/si [Side Effect]; juvenile rheumatoid arthritis/dt [Drug Therapy]; listeriosis/si [Side Effect]; liver failure/si [Side Effect]; lung aspergillosis/si [Side Effect]; lupus like syndrome/si [Side Effect]; lymphoma/si [Side Effect]; melanoma/si [Side Effect]; neutropenia/si [Side Effect]; non insulin dependent diabetes mellitus/dt [Drug Therapy]; nonhuman; nose polyp/dt [Drug Therapy]; Pneumocystis pneumonia/si [Side Effect]; polyarteritis nodosa/dt [Drug Therapy]; polymyositis/dt [Drug Therapy]; protein function; psoriasis/dt [Drug Therapy]; psoriasis vulgaris/dt [Drug Therapy]; psoriatic arthritis/dt [Drug Therapy]; retina macula age related degeneration/dt [Drug Therapy]; review; *rheumatoid arthritis/dt [Drug Therapy]; sarcoidosis/dt [Drug Therapy]; side effect/si [Side Effect]; Sjoegren syndrome/dt [Drug Therapy]; skin cancer/si [Side Effect]; skin disease/dt [Drug Therapy]; systematic review; treatment response; tuberculosis/si [Side Effect]; ulcerative colitis/dt [Drug Therapy]; vasculitis/dt [Drug Therapy]; Wegener granulomatosis/dt [Drug Therapy]; adalimumab/ae [Adverse Drug Reaction]; adalimumab/cm [Drug Comparison]; adalimumab/dt [Drug Therapy]; adalimumab/pk [Pharmacokinetics]; adalimumab/pd [Pharmacology]; adalimumab/sc [Subcutaneous Drug Administration]; ain 457/ct [Clinical Trial]; ain 457/dt [Drug Therapy]; amg 714/ct [Clinical Trial]; amg 714/dt [Drug Therapy]; amg 714/pd [Pharmacology]; briakinumab/dt [Drug Therapy]; briakinumab/pd [Pharmacology]; canakinumab/dt [Drug Therapy]; canakinumab/pd [Pharmacology]; certolizumab pegol/ae [Adverse Drug Reaction]; certolizumab pegol/dt [Drug Therapy]; certolizumab pegol/pk [Pharmacokinetics]; certolizumab pegol/pd [Pharmacology]; certolizumab pegol/sc [Subcutaneous Drug Administration]; *cytokine antibody/ct [Clinical Trial]; *cytokine antibody/dt [Drug Therapy]; *cytokine antibody/pd [Pharmacology]; disease modifying antirheumatic drug/dt [Drug Therapy]; etanercept/ae [Adverse Drug Reaction]; etanercept/dt [Drug Therapy]; etanercept/pk [Pharmacokinetics]; etanercept/pd [Pharmacology]; etanercept/sc [Subcutaneous Drug Administration]; golimumab/ae [Adverse Drug Reaction]; golimumab/cb [Drug Combination]; golimumab/dt [Drug Therapy]; golimumab/iv [Intravenous Drug Administration]; golimumab/pk [Pharmacokinetics]; golimumab/pd [Pharmacology]; golimumab/sc [Subcutaneous Drug Administration]; infliximab/ae [Adverse Drug Reaction]; infliximab/cb [Drug Combination]; infliximab/cm [Drug Comparison]; infliximab/dt [Drug Therapy]; infliximab/iv [Intravenous Drug Administration]; infliximab/pk [Pharmacokinetics]; infliximab/pd [Pharmacology]; medi 528/ct [Clinical Trial]; medi 528/dt [Drug Therapy]; mepolizumab/dt [Drug Therapy]; mepolizumab/pd [Pharmacology]; methotrexate/ct [Clinical Trial]; methotrexate/cb [Drug Combination]; methotrexate/dt [Drug Therapy]; placebo; recombinant interleukin 1 receptor blocking agent/ae [Adverse Drug Reaction]; recombinant interleukin 1 receptor blocking agent/ct [Clinical Trial]; recombinant interleukin 1 receptor blocking agent/cb [Drug Combination]; recombinant interleukin 1 receptor blocking agent/dt [Drug Therapy]; recombinant interleukin 11/dt [Drug Therapy]; recombinant interleukin 11/pd [Pharmacology]; rilonacept/ct [Clinical Trial]; rilonacept/dt [Drug Therapy]; steroid/dt [Drug Therapy]; tocilizumab/ae [Adverse Drug Reaction]; tocilizumab/dt [Drug Therapy]; tumor necrosis factor alpha antibody/ae [Adverse Drug Reaction]; tumor necrosis factor alpha antibody/dt [Drug Therapy]; tumor necrosis factor alpha inhibitor/ae [Adverse Drug Reaction]; tumor necrosis factor inhibitor/ae [Adverse Drug Reaction]; tumor necrosis factor inhibitor/ct [Clinical Trial]; tumor necrosis factor inhibitor/cb [Drug Combination]; tumor necrosis factor inhibitor/dt [Drug Therapy]; unclassified drug; ustekinumab/ct [Clinical Trial]; ustekinumab/dt [Drug Therapy]; ustekinumab/pd [Pharmacology].

Drug Trade Names and Manufacturers

Abbott, Glaxo SmithKline, Medimmune, Novartis, ain 457: Novartis, amg 714

Link to the Ovid Full Text or citation:

Click here for full text options

91.

CTLA-4Ig: Uses and future directions.

Ben-Shoshan M.

Recent Patents on Inflammation and Allergy Drug Discovery. 3 (2) (pp 132-142), 2009. Date of Publication: 2009.

AN: 2009579029

Cytotoxic lymphocyte-associated molecule-4 (CTLA-4, CD152) is a member of the CD28 receptor family. Blocking CD28 interaction with its ligands through the use of CTLA-4Ig might contribute to better control of dysregulated immune response processes. The ligands binding to CTLA-4 are the B7 family members, B7-1 (CD80) and B7-2 (CD86). CTLA-4Ig is now a Food and Drug Administration-approved drug for use treating patients with Rheumatoid arthritis (RA) but its use is explored also in other autoimmune diseases, transplantation as well as allergic diseases. Patents related to CTLA-4 function as well as possible clinical applications are discussed in this paper.

Institution

(Ben-Shoshan) Department of Pediatric Allergy and Immunology, McGill University Health Center, Montreal, QC, Canada (Ben-Shoshan) 418 Claremont, Montreal, QC, Canada

Publisher

Bentham Science Publishers B.V. (P.O. Box 294, Bussum 1400 AG, Netherlands)

Emtree Heading

acute graft rejection/co [Complication]; acute graft rejection/dt [Drug Therapy]; acute graft rejection/pc [Prevention]; advanced cancer/dt [Drug Therapy]; allergic asthma/et [Etiology]; allograft; antineoplastic activity; apoptosis; article; autoimmune disease/et [Etiology]; autoimmune disease/pc [Prevention]; autoimmune thyroiditis/et [Etiology]; autoimmunity; binding competition; chronic obstructive lung disease/si [Side Effect]; clinical trial; colon adenoma/si [Side Effect]; comorbidity; coughing/si [Side Effect]; cytokine production; demyelinating disease/si [Side Effect]; diabetes mellitus/dt [Drug Therapy]; diabetes mellitus/et [Etiology]; dizziness/si [Side Effect]; drug dose comparison; drug efficacy; drug fatality/si [Side Effect]; drug hypersensitivity/si [Side Effect]; drug induced headache/si [Side Effect]; drug mechanism; drug potentiation; drug safety; drug structure; drug substitution; drug treatment failure; drug withdrawal; dyspnea/si [Side Effect]; food allergy/et [Etiology]; graft survival; heart arrest/si [Side Effect]; human; immunogenicity; immunomodulation; immunopathogenesis; immunoregulation; immunosuppressive treatment; intestine ischemia/si [Side Effect]; keratoconjunctivitis sicca/si [Side Effect]; lupus erythematosus nephritis/dt [Drug Therapy]; lymphocyte activation; major histocompatibility complex; melanoma/dt [Drug Therapy]; multiple sclerosis/si [Side Effect]; musculoskeletal disease/si [Side Effect]; nonhuman; nose congestion/si [Side Effect]; onset age; opportunistic infection/si [Side Effect]; priority journal; psoriasis/dt [Drug Therapy]; rheumatoid arthritis/dt [Drug Therapy]; risk reduction; skin carcinoma/si [Side Effect]; squamous cell carcinoma/si [Side Effect]; systemic lupus erythematosus/dt [Drug Therapy]; systemic lupus erythematosus/et [Etiology]; tuberculosis/si [Side Effect]; unspecified side effect/si [Side Effect]; upper respiratory tract infection/si [Side Effect]; vasculitis/si [Side Effect]; *abatacept/ae [Adverse Drug Reaction]; *abatacept/ct [Clinical Trial]; *abatacept/cb [Drug Combination]; *abatacept/cm [Drug Comparison]; *abatacept/do [Drug Dose]; *abatacept/it [Drug Interaction]; *abatacept/dt [Drug Therapy]; *abatacept/pd [Pharmacology]; B7 antigen/ec [Endogenous Compound]; belatacept/ct [Clinical Trial]; belatacept/cm [Drug Comparison]; belatacept/dt [Drug Therapy]; calcineurin inhibitor/cm [Drug Comparison]; corticosteroid/ae [Adverse Drug Reaction]; corticosteroid/cb [Drug Combination]; corticosteroid/it [Drug Interaction]; cyclophosphamide/ae [Adverse Drug Reaction]; cyclophosphamide/cb [Drug Combination]; cyclophosphamide/it [Drug Interaction]; cyclophosphamide/dt [Drug Therapy]; cyclosporin/cm [Drug Comparison]; cyclosporin/dt [Drug Therapy]; cytotoxic T lymphocyte antigen 4/ec [Endogenous Compound]; disease modifying antirheumatic drug/dt [Drug Therapy]; hydroxychloroquine/dt [Drug Therapy]; infliximab/cm [Drug Comparison]; leflunomide/dt [Drug Therapy]; methotrexate/cb [Drug Combination]; methotrexate/dt [Drug Therapy]; placebo; tumor necrosis factor alpha inhibitor/dt [Drug Therapy].

Drug Trade Names and Manufacturers

Bristol Myers Squibb

Link to the Ovid Full Text or citation:

Click here for full text options

92.

Long-term safety and effectiveness of etanercept in children with selected categories of juvenile idiopathic arthritis.

Giannini E.H., Ilowite N.T., Lovell D.J., Wallace C.A., Rabinovich C.E., Reiff A., Higgins G., Gottlieb B., Singer N.G., Chon Y., Lin S.-L., Baumgartner S.W.

Arthritis and Rheumatism. 60 (9) (pp 2794-2804), 2009. Date of Publication: September 2009.

AN: 2009461552

Objective. This study was undertaken to evaluate the long-term safety and effectiveness of etanercept alone or in combination with methotrexate (MTX) in children with selected categories of juvenile idiopathic arthritis (JIA). Methods. Patients ages 2-18 years with rheumatoid factor (RF)-positive or RF-negative polyarthritis, systemic JIA, or extended oligoarthritis were eligible for the study. Patients received MTX alone (>10 mg/m2/ week [~0.3 mg/kg/week], maximum dosage 1 mg/kg/week), etanercept alone (0.8 mg/kg/week, maximum dose 50 mg), or etanercept plus MTX for 3 years in an open-label, nonrandomized study. Safety was assessed by measuring rates of adverse events, and effectiveness was assessed using the physician's global assessment of disease activity and the pediatric total joint assessment. Results. A total of 197, 103, and 294 patients were enrolled in the MTX, etanercept, and etanercept plus MTX groups, respectively. Exposure-adjusted rates of adverse events were similar among the 3 treatment groups (18.3, 18.7, and 21.6 per 100 patient-years in the MTX, etanercept, and etanercept plus MTX groups, respectively). Respective rates per 100 patient-years of serious adverse events (4.6, 7.1, and 6.0) and medically important infections (1.3, 1.8, and 2.1) were also similar among the 3 treatment groups. Scores for physician's global assessment and total active joints improved from baseline, and improvement was maintained for the duration of the study. Conclusion. These data confirm the findings of other long-term studies and suggest that etanercept or etanercept plus MTX has an acceptable safety and effectiveness profile in children with selected categories of JIA. Improvement was maintained for 3 years in those continuing to receive medication. © 2009, American College of Rheumatology.

Institution

(Giannini, Lovell) Pediatrics and Rheumatology/Pav 2-129, Children's Hospital Medical Center, 3333 Burnet Avenue, Cincinnati, OH 45229, United States (Ilowite) Albert Einstein College of Medicine, Bronx, NY, United States

(Wallace) Children's Hospital, Regional Medical Center, Seattle, WA, United States

(Rabinovich) Duke University Medical Center, Durham, NC, United States

(Reiff) Children's Hospital Los Angeles, Los Angeles, CA, United States

(Higgins) Ohio State University, Nationwide Children's Hospital, Columbus, OH, United States

(Gottlieb) Schneider Children's Hospital, New Hyde Park, NY, United States

(Singer) University Hospitals Case Medical Center, University Hospitals Rainbow Babies, Children's Hospital, Cleveland, OH, United States

(Chon, Lin, Baumgartner) Amgen Inc., Thousand Oaks, CA, United States

Publisher

John Wiley and Sons Inc. (P.O.Box 18667, Newark NJ 07191-8667, United States)

Emtree Heading

abnormal thinking/si [Side Effect]; abscess/si [Side Effect]; adolescent; adult; agitation; alanine aminotransferase blood level; anemia/si [Side Effect]; anxiety; arthritis/si [Side Effect]; article; aspartate aminotransferase blood level; asthenia/si [Side Effect]; asthma/si [Side Effect]; autoimmune disease/si [Side Effect]; bacterial infection/si [Side Effect]; behavior disorder/si [Side Effect]; blood culture; bronchitis/si [Side Effect]; child; clinical trial; colitis/si [Side Effect]; complex regional pain syndrome type I/si [Side Effect]; congenital malformation/si [Side Effect]; controlled clinical trial; controlled study; depression/si [Side Effect]; dermatomyositis/si [Side Effect]; diabetes mellitus/si [Side Effect]; disease activity; drug efficacy; drug eruption/si [Side Effect]; drug fatality/si [Side Effect]; drug induced cancer/si [Side Effect]; drug safety; drug substitution; drug withdrawal; female; gastroenteritis/si [Side Effect]; gastrointestinal symptom/si [Side Effect]; headache/si [Side Effect]; herpes zoster/si [Side Effect]; hostility; human; hydroureter/si [Side Effect]; idiopathic disease; infection/si [Side Effect]; injection site reaction/si [Side Effect]; *juvenile rheumatoid arthritis/dt [Drug Therapy]; leukopenia/si [Side Effect]; liver function test; lymphoma/si [Side Effect]; major clinical study; male; mental instability/si [Side Effect]; multicenter study; neuritis/si [Side Effect]; neuropathy/si [Side Effect]; open study; optic neuritis/si [Side Effect]; outcome assessment; patient safety; personality disorder/si [Side Effect]; pharyngitis/si [Side Effect]; polyarthritis; preschool child; priority journal; pyelonephritis/si [Side Effect]; randomized controlled trial; rectum cancer/si [Side Effect]; school child; sepsis/si [Side Effect]; side effect/si [Side Effect]; sinusitis/si [Side Effect]; skin infection/si [Side Effect]; thyroiditis/si [Side Effect]; treatment response; tuberculosis/si [Side Effect]; urinary tract infection/si [Side Effect]; uveitis/si [Side Effect]; virus infection/si [Side Effect]; azathioprine/dt [Drug Therapy]; corticosteroid/dt [Drug Therapy]; corticosteroid/ar [Intraarticular Drug Administration]; cyclosporin/dt [Drug Therapy]; disease modifying antirheumatic drug/dt [Drug Therapy]; *etanercept/ae [Adverse Drug Reaction]; *etanercept/ct [Clinical Trial]; *etanercept/cb [Drug Combination]; *etanercept/cm [Drug Comparison]; *etanercept/dt [Drug Therapy]; *etanercept/sc [Subcutaneous Drug Administration]; gold/dt [Drug Therapy]; hydroxychloroquine/dt [Drug Therapy]; leflunomide/dt [Drug Therapy]; *methotrexate/ae [Adverse Drug Reaction]; *methotrexate/ct [Clinical Trial]; *methotrexate/cb [Drug Combination]; *methotrexate/cm [Drug Comparison]; *methotrexate/dt [Drug Therapy]; nonsteroid antiinflammatory agent/dt [Drug Therapy]; prednisone/dt [Drug Therapy]; rheumatoid factor/ec [Endogenous Compound]; salazosulfapyridine/dt [Drug Therapy].

Link to the Ovid Full Text or citation:

Click here for full text options

93.

The common mycobacterial antigens and their importance in the treatment of disease.

Stanford J., Stanford C., Stansby G., Bottasso O., Bahr G., Grange J.

Current Pharmaceutical Design. 15 (11) (pp 1248-1260), 2009. Date of Publication: 2009.

AN: 2009228194

The mycobacteria are one of a number of genera making up the aerobic Actinomycetales. Their antigens demonstrable by immuno-precipitation methods can be divided into four groups. The group i antigens, common to all mycobacterial species, cross-react with their counterparts in animal cells, largely derived from mitochondria. Notable amongst these antigens are the heat-shock, or stress, proteins and possibly bacterial sugars. Tests of cell-mediated immunity show that people can be separated by their responsiveness in skin-test, or lymphocyte proliferation techniques, into four categories of responders. Category 1 individuals respond to all mycobacterial reagents through recognition of the group i antigens. Many chronic diseases are associated with a lack of cell-mediated responsiveness to the group i antigens, and have a raised antibody titre to them. This reflects a predominance of T helper 2 activity and reduced T helper 1 responsiveness as part of the pathogenesis of their diseases, which include chronic bacterial, viral and parasitic infections, allergies, autoimmunities and neoplasms. Packaged together, the group i antigens and the cell-wall adjuvants of selected aerobic Actinomycetales make potent immuno-modulatory reagents. An example is heat-killed Mycobacterium vaccae, useful in both prevention and treatment of disease. Treatment with such reagents results in alleviation of disease, restoration of cellular responsiveness to the common mycobacterial antigens and a decrease in antibody titres to them. This new approach to treatment for such a wide range of diseases has few disadvantageous side effects and can accompany other non-immunosuppressive therapies. © 2009 Bentham Science Publishers Ltd.

Institution

(Stanford, Stanford, Grange) Centre for Infectious Diseases and International Health, Windeyer Institute of Medical Sciences, University College London, 46 Cleveland Street, London W1T 4JF, United Kingdom (Stansby) Northern Vascular Unit, Freeman Hospital, Newcastle-upon-Tyne, United Kingdom

(Bottasso) Instituto de Inmunologia, Facultad de Ciencias Medicas, Universidad Nacional de Rosario, Santa Fe 3100, Rosario 2000, Argentina

(Bahr) Balamand University Medical School, Beirut, Lebanon

Publisher

Bentham Science Publishers B.V. (P.O. Box 294, Bussum 1400 AG, Netherlands)

Emtree Heading

adjuvant therapy; antibody titer; antigen antibody reaction; asthma/dt [Drug Therapy]; atherosclerosis/dt [Drug Therapy]; atopic dermatitis/dt [Drug Therapy]; autoimmunity; bacterial strain; Buruli ulcer; cellular immunity; clinical trial; coronary artery disease/su [Surgery]; cross reaction; disease course; drug effect; drug efficacy; drug response; drug safety; drug synthesis; eukaryote; highly active antiretroviral therapy; human; Human immunodeficiency virus infection/dt [Drug Therapy]; immunomodulation; immunopathology; immunoreactivity; immunoregulation; immunotherapy; insulin dependent diabetes mellitus/dt [Drug Therapy]; leishmaniasis/dt [Drug Therapy]; lepromatous leprosy/dt [Drug Therapy]; lupus vulgaris/dt [Drug Therapy]; lymphocyte proliferation; malaria/dt [Drug Therapy]; malignant neoplastic disease/dt [Drug Therapy]; malignant transformation; mycobacteriosis; Mycobacterium vaccae; nonhuman; priority journal; protein function; protein localization; psoriasis/dt [Drug Therapy]; regulatory T lymphocyte; review; rheumatoid arthritis/dt [Drug Therapy]; skin test; systemic disease/dt [Drug Therapy]; Th1 cell; Th2 cell; transluminal coronary angioplasty; treatment outcome; trypanosomiasis/dt [Drug Therapy]; tuberculosis/dt [Drug Therapy]; tuberculosis/pc [Prevention]; antiretrovirus agent/dt [Drug Therapy]; bacterium antibody; BCG vaccine/dt [Drug Therapy]; heat shock protein; *Mycobacterium antigen/dv [Drug Development]; *Mycobacterium antigen/dt [Drug Therapy]; Mycobacterium vaccine/ct [Clinical Trial]; Mycobacterium vaccine/dt [Drug Therapy]; placebo.

Link to the Ovid Full Text or citation:

Click here for full text options

94.

Drug therapy of pemphigus vulgaris.

Ahmed A.R.

Giornale Italiano di Dermatologia e Venereologia. 142 (4) (pp 391-408), 2007. Date of Publication: August 2007.

AN: 2007548048

Pemphigus vulgaris (PV) was and continues to remain a potentially fatal, autoimmune, mucocutaneous blistering disease. It most frequently affects the oral cavity and skin or both. Other mucous membranes of eye, nose, pharynx, larynx, esophagus, trachea, vagina, penis, anal canal, and the nails can be affected. It is characterized by an intraepidermal suprabasilar cleft, with in vivo deposition of IgG on perilesional tissue and a circulating autoantibody to keratinocyte cell surface molecules. Since their initial use, systemic corticosteroids have remained the mainstay of treatment. New agents have been used as they became available. Amongst the immunosuppressive agents available, the most commonly used are azathioprine, mycophenolate mofetil in combination with prednisone. Similar results with cyclophosphamide when used in a dexamethasone - cyclophosphamide pulse therapy protocol are observed. Immunoablative therapy without stem cell rescue has been abandoned. Plasmapheresis has limited value, as does extracorporeal photophoresis. Immunoadsorption shows promise but requires additional research to determine the optimal condition for the variables associated with the procedure. On the horizon the biologic agents show the most promise. Intravenous immunoglobulin (IVIg) is safe, produces long term remissions, has few side effects, but requires the presence of an infrastructure for delivery. It is slow to act. Not all patients have a complete response. Studies to attempt to convert poor responders to high responders are underway but not completed. A monoclonal antibody (rituximab) to CD20 present on pathogenic autoantibody producing cells has the potential to produce long term remissions. Used according to a lymphoma protocol, it can result in severe infection and death, besides a reasonable rate of recurrence. Its use with IVIg on a unique protocol may produce better results and less fatal infections. There is a renewed interest in the treatment of PV. Many investigators believe that it is a good model for autoimmunity. Lessons learned from treating pemphigus may be applied to other autoimmune diseases.

Institution

(Ahmed) Center for Blistering Diseases, New England Baptist Hospital, Boston, MA, United States (Ahmed) Center for Blistering Diseases, New England Baptist Hospital, 70 Parker Hill Avenue, Boston, MA 02120, United States

Publisher

Edizioni Minerva Medica

Emtree Heading

adjuvant therapy; alopecia/si [Side Effect]; amenorrhea/si [Side Effect]; B lymphocyte; bladder cancer/si [Side Effect]; blister; bronchospasm/si [Side Effect]; cell surface; chill/si [Side Effect]; clinical feature; clinical trial; combination chemotherapy; diabetes mellitus/si [Side Effect]; disease severity; drug blood level; drug dose titration; drug efficacy; drug fatality/si [Side Effect]; drug megadose; drug safety; drug withdrawal; dyspnea/si [Side Effect]; fever/si [Side Effect]; follow up; Gram negative sepsis/si [Side Effect]; heart disease/si [Side Effect]; hematologic disease/si [Side Effect]; hemorrhagic cystitis/si [Side Effect]; herpes zoster/si [Side Effect]; human; immunoadsorption; immunosuppressive treatment; immunotherapy; infection/si [Side Effect]; infertility/si [Side Effect]; keratinocyte; leukopenia/si [Side Effect]; lung abscess/si [Side Effect]; lung tuberculosis/si [Side Effect]; monotherapy; mouth ulcer/si [Side Effect]; multiple cycle treatment; mycosis/si [Side Effect]; nausea/si [Side Effect]; obesity/si [Side Effect]; opportunistic infection/si [Side Effect]; outcome assessment; pathogenesis; *pemphigus vulgaris/di [Diagnosis]; *pemphigus vulgaris/dt [Drug Therapy]; *pemphigus vulgaris/et [Etiology]; *pemphigus vulgaris/th [Therapy]; photochemotherapy; phototherapy; plasmapheresis; pneumonia/si [Side Effect]; pruritus/si [Side Effect]; quality of life; recurrent disease; remission; review; septicemia/si [Side Effect]; skin manifestation/si [Side Effect]; stem cell; symptom; T lymphocyte; thrombocytopenia/si [Side Effect]; treatment outcome; treatment response; tuberculosis/si [Side Effect]; unspecified side effect/si [Side Effect]; virus infection/si [Side Effect]; autoantibody; *azathioprine/cb [Drug Combination]; *azathioprine/dt [Drug Therapy]; CD20 antibody; chlorambucil/ae [Adverse Drug Reaction]; chlorambucil/dt [Drug Therapy]; *corticosteroid/ae [Adverse Drug Reaction]; *corticosteroid/cb [Drug Combination]; *corticosteroid/do [Drug Dose]; *corticosteroid/dt [Drug Therapy]; *cyclophosphamide/ae [Adverse Drug Reaction]; *cyclophosphamide/ct [Clinical Trial]; *cyclophosphamide/cb [Drug Combination]; *cyclophosphamide/dt [Drug Therapy]; *cyclophosphamide/iv [Intravenous Drug Administration]; cyclosporin/dt [Drug Therapy]; dapsone/ae [Adverse Drug Reaction]; dapsone/cb [Drug Combination]; dapsone/dt [Drug Therapy]; *dexamethasone/ae [Adverse Drug Reaction]; *dexamethasone/ct [Clinical Trial]; *dexamethasone/cb [Drug Combination]; *dexamethasone/dt [Drug Therapy]; *dexamethasone/iv [Intravenous Drug Administration]; etanercept/dt [Drug Therapy]; etanercept/sc [Subcutaneous Drug Administration]; folic acid/cb [Drug Combination]; gold/ae [Adverse Drug Reaction]; gold/dt [Drug Therapy]; gold/im [Intramuscular Drug Administration]; immunoglobulin/ae [Adverse Drug Reaction]; immunoglobulin/cb [Drug Combination]; immunoglobulin/dt [Drug Therapy]; immunoglobulin/iv [Intravenous Drug Administration]; immunoglobulin/pk [Pharmacokinetics]; immunoglobulin/pd [Pharmacology]; immunoglobulin G/ec [Endogenous Compound]; immunosuppressive agent/cb [Drug Combination]; immunosuppressive agent/dt [Drug Therapy]; infliximab/ae [Adverse Drug Reaction]; infliximab/dt [Drug Therapy]; methotrexate/ae [Adverse Drug Reaction]; methotrexate/cb [Drug Combination]; methotrexate/do [Drug Dose]; methotrexate/dt [Drug Therapy]; methotrexate/im [Intramuscular Drug Administration]; methotrexate/pk [Pharmacokinetics]; methotrexate/pd [Pharmacology]; methylprednisolone/dt [Drug Therapy]; methylprednisolone/iv [Intravenous Drug Administration]; *mycophenolic acid 2 morpholinoethyl ester/ae [Adverse Drug Reaction]; *mycophenolic acid 2 morpholinoethyl ester/cb [Drug Combination]; *mycophenolic acid 2 morpholinoethyl ester/dt [Drug Therapy]; *mycophenolic acid 2 morpholinoethyl ester/pd [Pharmacology]; pemphigus antibody; *prednisone/ae [Adverse Drug Reaction]; *prednisone/cb [Drug Combination]; *prednisone/do [Drug Dose]; *prednisone/dt [Drug Therapy]; *prednisone/po [Oral Drug Administration]; rituximab/ae [Adverse Drug Reaction]; rituximab/do [Drug Dose]; rituximab/dt [Drug Therapy]; steroid/cb [Drug Combination]; steroid/dt [Drug Therapy]; tumor necrosis factor alpha inhibitor/dt [Drug Therapy]; vincristine/ae [Adverse Drug Reaction]; vincristine/cb [Drug Combination]; vincristine/dt [Drug Therapy].

Link to the Ovid Full Text or citation:

Click here for full text options

95.

Toxicity of HIV protease inhibitors: Clinical considerations.

Boesecke C., Cooper D.A.

Current Opinion in HIV and AIDS. 3 (6) (pp 653-659), 2008. Date of Publication: November 2008.

AN: 2009322399

PURPOSE OF REVIEW: To review recent studies reporting toxicity, adverse events, side effects, and drug-drug interactions related to the use of HIV protease inhibitors, with particular focus on possible clinical implications. RECENT FINDINGS: Toxicity-associated adverse events still remain a major concern when prescribing HIV protease inhibitors. Among those, diarrhea, lipid, and liver enzyme elevations are predominant. Also, with protease inhibitors being metabolized by the hepatic cytochrome P450, a significant number of drug-drug interactions has to be taken into account in patients who often require coadministration of drugs to treat supplementary diseases, for example, tuberculosis. Hence, scientific interest in this area continues to be high, especially in characterizing individual toxicities of particular protease inhibitors as well as in comparing them among each other. SUMMARY: Protease inhibitors are still a cornerstone of combination antiretroviral therapy. A profound knowledge of the unwanted, but manageable, effects of protease inhibitor therapy is required to maintain an otherwise efficient and well tolerated antiretroviral therapy. Further research will elucidate the potential role of protease inhibitors both in double-boosted salvage therapy and in resource-limited settings. © 2008 Lippincott Williams & Wilkins, Inc.

Institution

(Boesecke, Cooper) National Centre in HIV Epidemiology and Clinical Research, University of New South Wales, 376 Victoria Street, Sydney, NSW 2010, Australia (Cooper) Centre for Immunology, St. Vincent's Hospital, University of New South Wales, Sydney, NSW, Australia

Publisher

Lippincott Williams and Wilkins (250 Waterloo Road, London SE1 8RD, United Kingdom)

Emtree Heading

abnormal laboratory result/si [Side Effect]; acquired immune deficiency syndrome/dt [Drug Therapy]; alanine aminotransferase blood level; alopecia/si [Side Effect]; antiviral resistance; *antiviral therapy; aspartate aminotransferase blood level; brain hemorrhage/si [Side Effect]; cholesterol blood level; clinical trial; combination chemotherapy; diabetes mellitus/si [Side Effect]; diarrhea/si [Side Effect]; drug blood level; drug contraindication; drug dosage form comparison; drug dose comparison; drug dose reduction; drug efficacy; drug eruption/si [Side Effect]; drug induced headache/si [Side Effect]; drug inhibition; drug megadose; drug metabolism; drug monitoring; drug safety; drug substitution; drug tolerability; *drug toxicity; drug treatment failure; dry skin/si [Side Effect]; fatigue/si [Side Effect]; gastrointestinal symptom/si [Side Effect]; glucose tolerance; hepatitis/si [Side Effect]; hepatitis B/dt [Drug Therapy]; hepatitis C/dt [Drug Therapy]; human; Human immunodeficiency virus infected patient; Human immunodeficiency virus infection/dr [Drug Resistance]; Human immunodeficiency virus infection/dt [Drug Therapy]; hyperbilirubinemia/si [Side Effect]; hyperlipidemia/si [Side Effect]; ingrown nail/si [Side Effect]; lipoatrophy/si [Side Effect]; lipodystrophy/si [Side Effect]; lipoprotein blood level; liver toxicity/si [Side Effect]; low drug dose; maintenance therapy; metabolic disorder/si [Side Effect]; metabolic syndrome X/si [Side Effect]; nausea/si [Side Effect]; nephrolithiasis/si [Side Effect]; oral paresthesia/si [Side Effect]; paresthesia/si [Side Effect]; priority journal; review; sex difference; side effect/si [Side Effect]; thrombocyte aggregation; treatment outcome; treatment response; triacylglycerol blood level; tuberculosis/dt [Drug Therapy]; vomiting/si [Side Effect]; weight gain; abacavir plus lamivudine/ae [Adverse Drug Reaction]; abacavir plus lamivudine/cb [Drug Combination]; abacavir plus lamivudine/cm [Drug Comparison]; abacavir plus lamivudine/dt [Drug Therapy]; abacavir plus lamivudine/to [Drug Toxicity]; amprenavir phosphate/ae [Adverse Drug Reaction]; amprenavir phosphate/cb [Drug Combination]; amprenavir phosphate/cm [Drug Comparison]; amprenavir phosphate/it [Drug Interaction]; amprenavir phosphate/dt [Drug Therapy]; amprenavir phosphate/to [Drug Toxicity]; atazanavir/ae [Adverse Drug Reaction]; atazanavir/cb [Drug Combination]; atazanavir/cr [Drug Concentration]; atazanavir/it [Drug Interaction]; atazanavir/dt [Drug Therapy]; atazanavir/to [Drug Toxicity]; atazanavir plus ritonavir/ae [Adverse Drug Reaction]; atazanavir plus ritonavir/cm [Drug Comparison]; atazanavir plus ritonavir/dt [Drug Therapy]; atazanavir plus ritonavir/to [Drug Toxicity]; cytochrome P450/ec [Endogenous Compound]; darunavir/ae [Adverse Drug Reaction]; darunavir/cr [Drug Concentration]; darunavir/dt [Drug Therapy]; darunavir/to [Drug Toxicity]; darunavir plus ritonavir/ae [Adverse Drug Reaction]; darunavir plus ritonavir/cr [Drug Concentration]; darunavir plus ritonavir/dt [Drug Therapy]; darunavir plus ritonavir/to [Drug Toxicity]; efavirenz/ae [Adverse Drug Reaction]; efavirenz/ct [Clinical Trial]; efavirenz/cb [Drug Combination]; efavirenz/cm [Drug Comparison]; efavirenz/dt [Drug Therapy]; emtricitabine/cb [Drug Combination]; emtricitabine/cm [Drug Comparison]; emtricitabine/dt [Drug Therapy]; indinavir/ae [Adverse Drug Reaction]; indinavir/ct [Clinical Trial]; indinavir/dt [Drug Therapy]; indinavir/to [Drug Toxicity]; indinavir plus ritonavir/dt [Drug Therapy]; indinavir plus ritonavir/to [Drug Toxicity]; ketoconazole/dt [Drug Therapy]; lopinavir/ae [Adverse Drug Reaction]; lopinavir/cb [Drug Combination]; lopinavir/cr [Drug Concentration]; lopinavir/dt [Drug Therapy]; lopinavir/to [Drug Toxicity]; lopinavir plus ritonavir/ae [Adverse Drug Reaction]; lopinavir plus ritonavir/ct [Clinical Trial]; lopinavir plus ritonavir/cb [Drug Combination]; lopinavir plus ritonavir/cm [Drug Comparison]; lopinavir plus ritonavir/dt [Drug Therapy]; lopinavir plus ritonavir/to [Drug Toxicity]; nelfinavir/ae [Adverse Drug Reaction]; nelfinavir/cb [Drug Combination]; nelfinavir/it [Drug Interaction]; nelfinavir/dt [Drug Therapy]; nelfinavir/to [Drug Toxicity]; nevirapine/cb [Drug Combination]; nevirapine/dt [Drug Therapy]; nevirapine/to [Drug Toxicity]; omeprazole/cb [Drug Combination]; omeprazole/it [Drug Interaction]; paroxetine/cb [Drug Combination]; paroxetine/cr [Drug Concentration]; paroxetine/it [Drug Interaction]; phenytoin/cb [Drug Combination]; phenytoin/cr [Drug Concentration]; phenytoin/it [Drug Interaction]; *proteinase inhibitor/dt [Drug Therapy]; *proteinase inhibitor/to [Drug Toxicity]; rifabutin/cb [Drug Combination]; rifabutin/dt [Drug Therapy]; rifampicin/ae [Adverse Drug Reaction]; rifampicin/cb [Drug Combination]; rifampicin/it [Drug Interaction]; ritonavir/ae [Adverse Drug Reaction]; ritonavir/cb [Drug Combination]; ritonavir/cm [Drug Comparison]; ritonavir/it [Drug Interaction]; ritonavir/dt [Drug Therapy]; ritonavir/to [Drug Toxicity]; ritonavir plus saquinavir/ae [Adverse Drug Reaction]; ritonavir plus saquinavir/ct [Clinical Trial]; ritonavir plus saquinavir/cb [Drug Combination]; ritonavir plus saquinavir/cm [Drug Comparison]; ritonavir plus saquinavir/cr [Drug Concentration]; ritonavir plus saquinavir/it [Drug Interaction]; ritonavir plus saquinavir/dt [Drug Therapy]; ritonavir plus saquinavir/to [Drug Toxicity]; ritonavir plus tipranavir/ae [Adverse Drug Reaction]; ritonavir plus tipranavir/ct [Clinical Trial]; ritonavir plus tipranavir/do [Drug Dose]; ritonavir plus tipranavir/dt [Drug Therapy]; ritonavir plus tipranavir/to [Drug Toxicity]; saquinavir/ae [Adverse Drug Reaction]; saquinavir/cb [Drug Combination]; saquinavir/cr [Drug Concentration]; saquinavir/it [Drug Interaction]; saquinavir/dt [Drug Therapy]; saquinavir/to [Drug Toxicity]; tenofovir/cb [Drug Combination]; tenofovir/cm [Drug Comparison]; tenofovir/dt [Drug Therapy]; tenofovir/to [Drug Toxicity]; tipranavir/ae [Adverse Drug Reaction]; tipranavir/dt [Drug Therapy]; tipranavir/to [Drug Toxicity]; unindexed drug; voriconazole/cb [Drug Combination]; voriconazole/it [Drug Interaction].

Drug Trade Names and Manufacturers

viracept

Link to the Ovid Full Text or citation:

Click here for full text options

96.

Inflammatory bowel disease (IBD): What is the role of conventional treatments?. <Maladies Inflammatoires Chroniques Intestinales (MICI): Quelle place pour les traitements conventionnels?.>

Beaugerie L.

Acta Endoscopica. 38 (4) (pp 359-374), 2008. Date of Publication: 2008.

AN: 2008500752

Conventional treatments for IBD include 5-amino-salicylate derivatives, corticosteroids and 'conventional' immunosuppressors (as opposed to biotechnological treatments), called 'immunomodulators' which essentially include thiopurines, methotrexate, cyclosporin and cyclosporin analogs. Here, we focus on the residual indications of corticosteroids and recent developments regarding the indications and good use of thiopurines and methotrexate, notably drawing from the recent European and American recommendations. Conventional treatments play a role in therapeutic strategies for IBD, and tend to be globally more ambitious and 'aggressive,' due to the diversification of the therapeutic arsenal and the observation of the relative failure of previously adopted strategies. The current objective is to obtain stable, prolonged clinical remission in the majority of patients, with the ideal being that one day the expression of IBD will be limited to the first attack. Since current treatments are only suspensive, this evolution makes sense only if the major risks related to the use of immunosuppressive treatments (severe infections and cancer) are known and mastered. © Springer 2008.

Institution

(Beaugerie) Service de Gastroenterologie et Nutrition, Hopital Saint-Antoine et Universite Pierre et Marie Curie, 184 rue du Faubourg Saint-Antoine, F-75571 Paris Cedex 12, France

Publisher

Springer Paris

Emtree Heading

antiinflammatory activity; arthralgia/si [Side Effect]; article; aspergillosis/si [Side Effect]; blood toxicity/si [Side Effect]; cancer risk; candidiasis/si [Side Effect]; cholestasis/si [Side Effect]; clinical trial; coughing/si [Side Effect]; Crohn disease/dt [Drug Therapy]; cytolysis; diabetes mellitus/si [Side Effect]; diarrhea/si [Side Effect]; drug choice; drug contraindication; drug dose reduction; drug efficacy; drug eruption/si [Side Effect]; drug fatality/si [Side Effect]; drug fever/si [Side Effect]; drug hypersensitivity/si [Side Effect]; drug indication; drug induced headache/si [Side Effect]; drug monitoring; drug substitution; drug treatment failure; drug withdrawal; drunkenness/si [Side Effect]; endocrine disease/si [Side Effect]; *enteritis/dr [Drug Resistance]; *enteritis/dt [Drug Therapy]; erythroblastopenia/si [Side Effect]; evening dosage; fatigue/si [Side Effect]; fibrosis/si [Side Effect]; flu like syndrome/si [Side Effect]; herpes/si [Side Effect]; human; infection/si [Side Effect]; interstitial immunoallergic pneumonia/si [Side Effect]; jaundice/si [Side Effect]; liver cirrhosis/si [Side Effect]; liver toxicity/si [Side Effect]; long term care; low drug dose; lymphocytopenia/si [Side Effect]; metabolic disorder/si [Side Effect]; nausea/si [Side Effect]; nausea and vomiting/si [Side Effect]; nephrotoxicity/si [Side Effect]; opportunistic infection/si [Side Effect]; pancreatitis/si [Side Effect]; patient monitoring; pneumonia/si [Side Effect]; relapse; remission; side effect/si [Side Effect]; triacylglycerol lipase blood level; tuberculosis/si [Side Effect]; ulcerative colitis/dt [Drug Therapy]; unspecified side effect/si [Side Effect]; verruca vulgaris/si [Side Effect]; virus infection/si [Side Effect]; acetylsalicylic acid/it [Drug Interaction]; allopurinol/it [Drug Interaction]; allopurinol/pd [Pharmacology]; azathioprine/do [Drug Dose]; azathioprine/dt [Drug Therapy]; budesonide/ae [Adverse Drug Reaction]; budesonide/dt [Drug Therapy]; calcineurin inhibitor/dt [Drug Therapy]; corticosteroid/ae [Adverse Drug Reaction]; corticosteroid/cm [Drug Comparison]; corticosteroid/do [Drug Dose]; corticosteroid/dt [Drug Therapy]; corticosteroid/iv [Intravenous Drug Administration]; corticosteroid/po [Oral Drug Administration]; cyclosporin/dt [Drug Therapy]; cyclosporin/iv [Intravenous Drug Administration]; cyclosporin derivative/dt [Drug Therapy]; hydrocortisone; immunosuppressive agent/ae [Adverse Drug Reaction]; immunosuppressive agent/cm [Drug Comparison]; infliximab/dt [Drug Therapy]; mercaptopurine/ae [Adverse Drug Reaction]; mercaptopurine/cb [Drug Combination]; mercaptopurine/cm [Drug Comparison]; mercaptopurine/do [Drug Dose]; mercaptopurine/it [Drug Interaction]; mercaptopurine/dt [Drug Therapy]; mesalazine/cb [Drug Combination]; mesalazine/it [Drug Interaction]; mesalazine/dt [Drug Therapy]; mesalazine/pd [Pharmacology]; methotrexate/ae [Adverse Drug Reaction]; methotrexate/ct [Clinical Trial]; methotrexate/ad [Drug Administration]; methotrexate/cm [Drug Comparison]; methotrexate/it [Drug Interaction]; methotrexate/dt [Drug Therapy]; methotrexate/im [Intramuscular Drug Administration]; methotrexate/po [Oral Drug Administration]; methotrexate/sc [Subcutaneous Drug Administration]; methylprednisolone/dt [Drug Therapy]; methylprednisolone/po [Oral Drug Administration]; methylprednisolone/pa [Parenteral Drug Administration]; phenylbutazone/it [Drug Interaction]; placebo; prednisolone/dt [Drug Therapy]; tetracosactide; trimethoprim/it [Drug Interaction]; tumor necrosis factor antibody/ae [Adverse Drug Reaction]; tumor necrosis factor antibody/cm [Drug Comparison]; tumor necrosis factor antibody/dt [Drug Therapy].

Drug Trade Names and Manufacturers

synacthene

Link to the Ovid Full Text or citation:

Click here for full text options

97.

Reducing bias from test misclassification in burden of disease studies: Use of test to actual positive ratio - New test parameter.

Campbell H., Biloglav Z., Rudan I.

Croatian Medical Journal. 49 (3) (pp 402-414), 2008. Date of Publication: June 2008.

AN: 2008327823

Aim: To address the problem of estimating disease frequency identified by a diagnostic test, which may not represent the actual number of persons with disease in a community, but rather the number of persons who tested positive. Those two values may be very different, their relationship depending on the properties of the diagnostic test applied and true prevalence of the disease in a population. Methods: We defined a new test parameter, the ratio of Test to Actual Positives (TAP), which summarizes the properties of the diagnostic test applied and true prevalence of the disease in a population, and propose that is the most useful summary measure of the potential for bias in disease frequency estimates. Results: A consideration of the relationship between the sensitivity (Se) and specificity (Sp) of the diagnostic test and the true prevalence of disease in a population can inform study design by highlighting the potential for disease misclassification bias. The effects of a decrease in Sp on the TAP ratio at very low disease prevalence are dramatic, as at 80% Sp (and any Se value including 100%), the measured disease frequency will represent a 25-fold overestimate. At a disease prevalence of 0.10, the Sp needs to be 90% or greater to achieve a TAP ratio of 1.0. However, unlike at lower levels of disease prevalence, the test Se is also an important determinant of the TAP ratio. A TAP ratio of 1.0 can be achieved by a Sp of 95% and intermediate Se (40%-60%); or a Sp of 99% and very high Se (over 90%). This illustrates how a test with poor performance characteristics in a clinical setting can perform well in a disease burden study in a population. In circumstances in which the TAP ratio suggests a potential for a large bias, we suggest correction procedures that limit disease misclassification bias and which are often counter-intuitive. We also illustrate how these methods can improve the power of intervention studies, which define outcomes by use of a diagnostic test. Conclusions: Optimal screening test characteristics for use in a population-based survey are likely to be different to those when the test is used in a clinical setting. Calibrating the test a priori to bring the TAP ratio closer to unity deals with the possible large bias in disease burden estimates based on application of diagnostic (screening) test.

Institution

(Campbell, Rudan) Department of Public Health Sciences, University of Edinburgh Medical School, Edinburgh, United Kingdom (Biloglav) Andrija Stampar School of Public Health, University of Zagreb Medical School, Zagreb, Croatia

(Rudan) Croatian Center for Global Health, University of Split Medical School, Split, Croatia

(Rudan) Croatian Centre for Global Health, University of Split Medical School, Soltanska 2, 21000 Split, Croatia

Publisher

Pabst Science Publishers

Emtree Heading

article; child; clinical trial; controlled clinical trial; controlled study; *diabetes mellitus/ep [Epidemiology]; diagnostic test; disease classification; *disease surveillance; human; *Human immunodeficiency virus infection/ep [Epidemiology]; iodine deficiency/ep [Epidemiology]; major clinical study; morbidity; observer bias; outcome assessment; Papanicolaou test; *pneumonia/ep [Epidemiology]; polymerase chain reaction; prevalence; randomized controlled trial; screening test; sensitivity and specificity; *trichomoniasis/ep [Epidemiology]; tuberculin test; *tuberculosis/ep [Epidemiology]; world health organization.

Link to the Ovid Full Text or citation:

Click here for full text options

98.

Chronic heart failure in Japan: Implications of the CHART studies.

Shiba N., Shimokawa H.

Vascular Health and Risk Management. 4 (1) (pp 103-113), 2008. Date of Publication: 2008.

AN: 2008158492

The prognosis of patients with chronic heart failure (CHF) still remains poor, despite the recent advances in medical and surgical treatment. Furthermore, CHF is a major public health problem in most industrialized countries where the elderly population is rapidly increasing. Although the prevalence and mortality of CHF used to be relatively low in Japan, the disorder has been markedly increasing due to the rapid aging of the society and the Westernization of lifestyle that facilitates the development of coronary artery disease. The Chronic Heart Failure Analysis and Registry in the Tohoku District (CHART)-1 study was one of the largest cohorts in Japan. The study has clarified the characteristics and prognosis of Japanese patients with CHF, demonstrating that their prognosis was similarly poor compared with those in Western countries. However, we still need evidence for the prevention and treatment of CHF based on the large cohort studies or randomized treatment trials in the Japanese population. Since the strategy for CHF management is now changing from treatment to prevention, a larger-size prospective cohort, called the CHART-2 study, has been initiated to evaluate the risk factors of CHF in Japan. This review summarizes the current status of CHF studies in Japan and discusses their future perspectives. © 2008 Shiba and Shimokawa, publisher and licensee Dove Medical Press Ltd.

Institution

(Shiba, Shimokawa) Department of Cardiovascular Medicine, Department of Evidence-Based Cardiovascular Medicine, Tohoku University Graduate School of Medicine, 1-1 Seiryo-machi, Aobaku, Sendai City 980-8574, Japan

Publisher

DOVE Medical Press Ltd.

Emtree Heading

aging; cardiovascular disease; cause of death; clinical trial; cost effectiveness analysis; diabetes mellitus; drug dose comparison; drug efficacy; echocardiography; gastroenteritis; health insurance; *heart failure/di [Diagnosis]; *heart failure/dm [Disease Management]; *heart failure/dt [Drug Therapy]; *heart failure/et [Etiology]; *heart failure/pc [Prevention]; *heart failure/su [Surgery]; heart left ventricle ejection fraction; heart left ventricle enddiastolic volume; human; hypercholesterolemia/dt [Drug Therapy]; hypertension/dt [Drug Therapy]; Japan; medical care; optimal drug dose; pneumonia; prognosis; proportional hazards model; quality of life; race difference; review; social status; treatment outcome; tuberculosis; angiotensin 2 receptor antagonist/dt [Drug Therapy]; azosemide/ct [Clinical Trial]; azosemide/cm [Drug Comparison]; azosemide/dt [Drug Therapy]; beta adrenergic receptor blocking agent/dt [Drug Therapy]; candesartan/ct [Clinical Trial]; candesartan/dt [Drug Therapy]; carvedilol/ct [Clinical Trial]; carvedilol/do [Drug Dose]; carvedilol/dt [Drug Therapy]; dipeptidyl carboxypeptidase inhibitor/dt [Drug Therapy]; furosemide/ct [Clinical Trial]; furosemide/cm [Drug Comparison]; furosemide/dt [Drug Therapy]; olmesartan/ct [Clinical Trial]; olmesartan/dt [Drug Therapy]; pitavastatin/ct [Clinical Trial]; pitavastatin/dt [Drug Therapy]; placebo; valsartan/ct [Clinical Trial]; valsartan/dt [Drug Therapy].

Link to the Ovid Full Text or citation:

Click here for full text options

99.

Review: Fixed-dose drug combinations improve medication compliance compared with free-drug regimens: Commentary.

Messerli F.H., Bergmann J.-F.

Evidence-Based Medicine. 13 (1) (pp 18), 2008. Date of Publication: February 2008.

AN: 2008080691

Institution

(Messerli) Columbia University College of Physicians and Surgeons, New York, NY, United States (Bergmann) Hopital Lariboisiere, University Paris 7, Paris, France

Publisher

BMJ Publishing Group

Emtree Heading

*chronic disease/dt [Drug Therapy]; clinical trial; combination chemotherapy; controlled clinical trial; diabetes mellitus/dt [Drug Therapy]; human; Human immunodeficiency virus infection/dt [Drug Therapy]; hypertension/dt [Drug Therapy]; outcome assessment; patient attitude; patient compliance; risk reduction; short survey; treatment duration; tuberculosis/dt [Drug Therapy]; *drug/ct [Clinical Trial]; *drug/do [Drug Dose]; *drug/dt [Drug Therapy].

Link to the Ovid Full Text or citation:

Click here for full text options

100.

European evidence-based Consensus on the management of ulcerative colitis: Current management.

Travis S.P.L., Stange E.F., Lemann M., Oresland T., Bemelman W.A., Chowers Y., Colombel J.F., D'Haens G., Ghosh S., Marteau P., Kruis W., Mortensen N.J.McC., Penninckx F., Gassull M.

Journal of Crohn's and Colitis. 2 (1) (pp 24-62), 2008. Date of Publication: March 2008.

AN: 2008056224

Publisher

Elsevier

Emtree Heading

appendectomy; basal cell carcinoma/si [Side Effect]; bone marrow suppression/si [Side Effect]; clinical feature; clinical trial; colon resection; colonoscopy; combination chemotherapy; controlled clinical trial; diabetes mellitus/si [Side Effect]; diarrhea/si [Side Effect]; disease activity; disease course; drug dosage form comparison; drug dose escalation; drug dose increase; drug efficacy; drug fatality/si [Side Effect]; drug mechanism; drug megadose; drug response; drug safety; follow up; gastrointestinal biopsy; gastrointestinal symptom/si [Side Effect]; headache/si [Side Effect]; histoplasmosis/si [Side Effect]; human; hypertension/si [Side Effect]; hypomagnesemia/si [Side Effect]; ileostomy; interstitial nephritis/si [Side Effect]; intestine function; kidney failure/si [Side Effect]; laparoscopy; monotherapy; nausea/si [Side Effect]; nephrotic syndrome/si [Side Effect]; neuropathy/si [Side Effect]; pancreatitis/si [Side Effect]; paresthesia/si [Side Effect]; perioperative period; pneumonia/si [Side Effect]; postoperative complication; postoperative period; priority journal; proctocolectomy; randomized controlled trial; rash/si [Side Effect]; relapse; review; risk factor; single drug dose; steroid therapy; thrombocytopenia/si [Side Effect]; toxic megacolon/co [Complication]; treatment outcome; tremor/si [Side Effect]; tuberculosis/si [Side Effect]; *ulcerative colitis/di [Diagnosis]; *ulcerative colitis/dt [Drug Therapy]; *ulcerative colitis/su [Surgery]; 12 sulfodehydroabietic acid/dt [Drug Therapy]; 12 sulfodehydroabietic acid/pd [Pharmacology]; acetarsol/ct [Clinical Trial]; acetarsol/cm [Drug Comparison]; acetarsol/dt [Drug Therapy]; adalimumab/cm [Drug Comparison]; adalimumab/dt [Drug Therapy]; adalimumab/sc [Subcutaneous Drug Administration]; aminosalicylic acid/cb [Drug Combination]; aminosalicylic acid/dt [Drug Therapy]; aminosalicylic acid/po [Oral Drug Administration]; amoxicillin/cb [Drug Combination]; amoxicillin/dt [Drug Therapy]; azathioprine/ae [Adverse Drug Reaction]; azathioprine/ct [Clinical Trial]; azathioprine/dt [Drug Therapy]; azathioprine/po [Oral Drug Administration]; beclometasone dipropionate/dt [Drug Therapy]; betamethasone/dt [Drug Therapy]; betamethasone/iv [Intravenous Drug Administration]; bismuth carbomer/ct [Clinical Trial]; bismuth carbomer/dt [Drug Therapy]; bismuth carbomer/pd [Pharmacology]; bismuth derivative/ct [Clinical Trial]; bismuth derivative/dt [Drug Therapy]; bismuth derivative/pd [Pharmacology]; budesonide/dt [Drug Therapy]; corticosteroid/dt [Drug Therapy]; corticosteroid/iv [Intravenous Drug Administration]; corticosteroid/po [Oral Drug Administration]; corticosteroid/rc [Rectal Drug Administration]; cyclosporin A/ae [Adverse Drug Reaction]; cyclosporin A/ct [Clinical Trial]; cyclosporin A/cm [Drug Comparison]; cyclosporin A/dt [Drug Therapy]; cyclosporin A/iv [Intravenous Drug Administration]; cyclosporin A/po [Oral Drug Administration]; cyclosporin A/pd [Pharmacology]; hydrocortisone/dt [Drug Therapy]; hydrocortisone/iv [Intravenous Drug Administration]; infliximab/ae [Adverse Drug Reaction]; infliximab/ct [Clinical Trial]; infliximab/cm [Drug Comparison]; infliximab/do [Drug Dose]; infliximab/dt [Drug Therapy]; lidocaine/dt [Drug Therapy]; lidocaine/pd [Pharmacology]; mesalazine/ae [Adverse Drug Reaction]; mesalazine/ct [Clinical Trial]; mesalazine/cb [Drug Combination]; mesalazine/cm [Drug Comparison]; mesalazine/do [Drug Dose]; mesalazine/dt [Drug Therapy]; mesalazine/po [Oral Drug Administration]; mesalazine/pd [Pharmacology]; mesalazine/rc [Rectal Drug Administration]; mesalazine/tp [Topical Drug Administration]; mesren; methotrexate/ct [Clinical Trial]; methotrexate/cm [Drug Comparison]; methotrexate/dt [Drug Therapy]; methotrexate/po [Oral Drug Administration]; methylprednisolone/do [Drug Dose]; methylprednisolone/dt [Drug Therapy]; methylprednisolone/iv [Intravenous Drug Administration]; metronidazole/cb [Drug Combination]; nicotine/ct [Clinical Trial]; nicotine/cb [Drug Combination]; nicotine/cm [Drug Comparison]; nicotine/dt [Drug Therapy]; nicotine/pd [Pharmacology]; nicotine/td [Transdermal Drug Administration]; nicotine tartrate/dt [Drug Therapy]; nicotine tartrate/pd [Pharmacology]; placebo; prednisolone/ct [Clinical Trial]; prednisolone/cm [Drug Comparison]; prednisolone/dt [Drug Therapy]; prednisolone/po [Oral Drug Administration]; rebamipide/dt [Drug Therapy]; rebamipide/pd [Pharmacology]; ropivacaine/ct [Clinical Trial]; ropivacaine/do [Drug Dose]; ropivacaine/dt [Drug Therapy]; ropivacaine/pd [Pharmacology]; salazosulfapyridine/cm [Drug Comparison]; salazosulfapyridine/dt [Drug Therapy]; tacrolimus/ae [Adverse Drug Reaction]; tacrolimus/ct [Clinical Trial]; tacrolimus/cm [Drug Comparison]; tacrolimus/dt [Drug Therapy]; tacrolimus/po [Oral Drug Administration]; tetracycline/cb [Drug Combination]; unclassified drug; unindexed drug.

Drug Trade Names and Manufacturers

asacol, claversal, lialda, mesasal, mesren, pentasa, rowasa, salofalk

Link to the Ovid Full Text or citation:

Click here for full text options

101.

SCORTEN: Does it need modification?.

Vaishampayan S., Das A., Verma R.

Indian Journal of Dermatology, Venereology and Leprology. 74 (1) (pp 35-37), 2008. Date of Publication: 01 Jan 2008.

AN: 2008035832

Background: Toxic epidermal necrolysis (TEN) is a drug induced acute life threatening condition with mortality ranging from about 15 to 60%. A 'severity of illness' score termed as SCORTEN has been developed to predict mortality in TEN cases at the time of admission. It is calculated by giving one point for each of predetermined seven variables, evaluated during first 24 hours of admission. Total score ranging from 1-7 predicts a probability of mortality from 0.03 to 0.90. Aim: A prospective study was conducted to analyze efficacy of 'SCORTEN' in TEN cases to predict mortality during their management. Methods: All cases of TEN reporting for management to the hospital were assessed using 'SCORTEN' on day one and day five to predict probable mortality, this data was then compared with ultimate outcome. Results: During the study period, we treated 10 cases of TEN, all induced by drugs, patient's age ranging from 03 to 70 years and body surface area (BSA) involvement from 10 to 95%. Three cases succumbed to death. These cases were analyzed with SCORTEN to predict probability of mortality at the time of admission and day five. We encountered some variations from the original study. It was observed that if patients are analyzed with SCORTEN on a daily/alternate day basis, it will serve as a better predictor of mortality. Conclusion: Body surface area (BSA) involvement and age probably need more weightage in calculations. Besides malignancy, tuberculosis and pre-existing diabetes also need to be included while predicting mortality.

Institution

(Vaishampayan, Das, Verma) Department of Dermatology, Armed Forces Medical College and Command Hospital (SC), Pune, Maharashtra, India (Vaishampayan) 155 Base Hospital c/o 99 APO

Publisher

Medknow Publications

Emtree Heading

adolescent; adult; aged; article; body surface; child; clinical article; clinical assessment; clinical effectiveness; clinical trial; controlled study; female; hospital admission; hospital care; human; male; mortality; outcome assessment; prediction; probability; *scoring system; *scorten; *toxic epidermal necrolysis.

Link to the Ovid Full Text or citation:

Click here for full text options

102.

Evaluation of an intermittent six-month regimen in new pulmonary tuberculosis patients with diabetes mellitus.

Balasubramanian R., Ramanathan U., Thyagarajan K., Ramachandran R., Rajaram K., Bhaskar D., Shantharam, Hariharan R.S., Narayanan P.R.

The Indian journal of tuberculosis. 54 (4) (pp 168-176), 2007. Date of Publication: Oct 2007.

AN: 18072529

BACKGROUND: The treatment of tuberculosis (TB) with category I regimen of the Revised National Tuberculosis Control Programme (RNTCP) for patients with diabetes mellitus (DM) needs evaluation. OBJECTIVE: To assess the cure and relapse rates in 3 years, among the new smear-positive TB patients with Type-2 DM (DMTB) treated with CAT-I regimen (2E3H3R3Z3/4R3H3) of RNTCP. METHODOLOGY: TB suspects attending the diabetology units and the TB research centre (TRC) Chennai, were investigated. Eligible DMTB cases were enrolled. Baseline estimation of cardiac, renal, liver function tests and glycosylated-HBA1c were undertaken. All patients received 2E3H3R3Z3/4R3H3 under supervision at TRC. Clinical and sputum (smear and culture) examinations and monitoring of diabetic status were undertaken every month up to 24 months, then once in 3 months up to 36 months. RESULTS: Of 100 patients admitted, 7 were excluded for various reasons from analysis. Of 93 patients, 87 (94%) had a favourable response at the end of treatment. Pre and post treatment mean glycosylated-HBA1c were 9.7% and 8.4% (>7% poor control). During follow-up period, 6 died and one lost to follow-up. Of the remaining, four relapsed. CONCLUSION: Category-I regimen, recommended for all the new smear-positive patients in the Indian TB programme, is effective in the treatment of DMTB patients, despite poor control of diabetes.

Institution

(Balasubramanian, Ramanathan, Thyagarajan, Ramachandran, Rajaram, Bhaskar, Shantharam, Hariharan, Narayanan) Tuberculosis Research Centre (ICMR), Chennai.

Emtree Heading

adult; aged; article; clinical trial; *diabetes mellitus/dt [Drug Therapy]; female; follow up; human; *lung tuberculosis/co [Complication]; *lung tuberculosis/dt [Drug Therapy]; male; microbiology; middle aged; radiography; recurrent disease; sputum; time; treatment outcome; tuberculostatic agent/ae [Adverse Drug Reaction]; tuberculostatic agent/dt [Drug Therapy].

Link to the Ovid Full Text or citation:

Click here for full text options

103.

Abatacept: A novel treatment for moderate-to-severe rheumatoid arthritis.

Reynolds J., Shojania K., Marra C.A.

Pharmacotherapy. 27 (12 I) (pp 1693-1701), 2007. Date of Publication: December 2007.

AN: 2007599773

Rheumatoid arthritis is a chronic autoimmune disease that often leads to functional disability and reduced quality of life. The pathogenesis of synovial inflammation that is associated with this disease is thought to result from T-cell activation. To become fully activated, T cells require an antigen-specific signal through the T-cell receptor and a second signal through a costimulatory receptor. Abatacept is the first drug in a new class of disease-modifying antirheumatic drugs (DMARDs) known as selective costimulation modulators. Costimulation modulators block the second signal and decrease T-cell activation. Abatacept has been approved by the United States Food and Drug Administration for reducing signs and symptoms, inducing major clinical response, slowing the progression of structural damage, and improving physical function in adults with moderate-to-severe active rheumatoid arthritis who have had an inadequate response to at least one other DMARD, such as methotrexate or tumor necrosis factor (TNF)-alpha inhibitors. Randomized controlled trials have shown that abatacept improves both clinical outcomes and health-related quality of life in patients who have had an inadequate response to other DMARDs. Abatacept has been shown to be well tolerated. In clinical trials, however, abatacept treatment was associated with a higher rate of infections compared with placebo. This finding was compounded when abatacept was used with TNF-alpha inhibitors; thus, this combination should be avoided. Abatacept appears to be a useful treatment option for patients with rheumatoid arthritis who have previously failed other DMARDs. However, additional clinical trials evaluating its long-term effect on patient safety and disease outcomes are needed.

Institution

(Reynolds, Shojania) Division of Rheumatology, Faculty of Medicine, University of British Columbia, Vancouver, BC, Canada (Marra) Faculty of Pharmaceutical Sciences, University of British Columbia, Vancouver, BC, Canada

(Marra) Arthritis Research Centre of Canada, Vancouver, BC, Canada

(Marra) Centre for Health Evaluation and Outcome Sciences, St. Paul's Hospital, Vancouver, BC, Canada

(Marra) Centre for Health Evaluation and Outcome Sciences, St. Paul's Hospital, 620B, 1081 Burrard Street, Vancouver, BC V6Z 1Y6, Canada

Publisher

Pharmacotherapy Publications Inc.

Emtree Heading

add on therapy; asthma; basal cell carcinoma/si [Side Effect]; chronic obstructive lung disease/si [Side Effect]; clinical trial; combination chemotherapy; comorbidity; controlled clinical trial; coughing/si [Side Effect]; diabetes mellitus; disease course; disease severity; dizziness/si [Side Effect]; drug blood level; drug clearance; drug effect; drug efficacy; drug fatality/si [Side Effect]; drug potentiation; drug safety; drug tolerability; drug withdrawal; dyspnea/si [Side Effect]; headache/si [Side Effect]; heart failure; human; hypertension/si [Side Effect]; immunogenicity; infection/si [Side Effect]; injection site reaction/si [Side Effect]; monotherapy; nausea/si [Side Effect]; neoplasm/si [Side Effect]; outcome assessment; pathogenesis; quality of life; randomized controlled trial; review; *rheumatoid arthritis/dt [Drug Therapy]; *rheumatoid arthritis/et [Etiology]; rhinopharyngitis/si [Side Effect]; squamous cell carcinoma/si [Side Effect]; synovitis/et [Etiology]; T lymphocyte activation; treatment duration; treatment indication; treatment response; tuberculosis/si [Side Effect]; unspecified side effect/si [Side Effect]; upper respiratory tract infection/si [Side Effect]; *abatacept/ae [Adverse Drug Reaction]; *abatacept/ct [Clinical Trial]; *abatacept/cb [Drug Combination]; *abatacept/it [Drug Interaction]; *abatacept/dt [Drug Therapy]; *abatacept/iv [Intravenous Drug Administration]; *abatacept/pk [Pharmacokinetics]; *abatacept/pd [Pharmacology]; disease modifying antirheumatic drug/ct [Clinical Trial]; disease modifying antirheumatic drug/cb [Drug Combination]; disease modifying antirheumatic drug/dt [Drug Therapy]; disease modifying antirheumatic drug/po [Oral Drug Administration]; etanercept/ae [Adverse Drug Reaction]; etanercept/ct [Clinical Trial]; etanercept/cb [Drug Combination]; etanercept/it [Drug Interaction]; etanercept/dt [Drug Therapy]; infliximab/ct [Clinical Trial]; infliximab/cb [Drug Combination]; infliximab/dt [Drug Therapy]; methotrexate/ct [Clinical Trial]; methotrexate/cb [Drug Combination]; methotrexate/dt [Drug Therapy]; placebo; recombinant interleukin 1 receptor blocking agent/ct [Clinical Trial]; recombinant interleukin 1 receptor blocking agent/cb [Drug Combination]; recombinant interleukin 1 receptor blocking agent/dt [Drug Therapy]; tumor necrosis factor alpha inhibitor/ae [Adverse Drug Reaction]; tumor necrosis factor alpha inhibitor/ct [Clinical Trial]; tumor necrosis factor alpha inhibitor/cb [Drug Combination]; tumor necrosis factor alpha inhibitor/it [Drug Interaction]; tumor necrosis factor alpha inhibitor/dt [Drug Therapy].

Link to the Ovid Full Text or citation:

Click here for full text options

104.

Fluoroquinolones for the treatment of pulmonary tuberculosis.

Moadebi S., Harder C.K., Fitzgerald M.J., Elwood K.R., Marra F.

Drugs. 67 (14) (pp 2077-2099), 2007. Date of Publication: 2007.

AN: 2007487057

Tuberculosis (TB) continues to be a significant problem globally. Treatment includes a multiple drug regimen with isoniazid, rifampicin (rifampin), pyrazinamide and ethambutol. Often, one of these medications needs to be replaced as a result of adverse events or because Mycobacterium tuberculosis develops resistance against one these first-line agents. Fluoroquinolones, particularly the newer ones, possess good in vitro (levofloxacin, gatifloxacin, moxifloxacin) and in vivo (gatifloxacin and moxifloxacin) bactericidal activity against M. tuberculosis, making them attractive agents for the treatment of pulmonary TB.All relevant clinical trials, cohort studies and case reports investigating the clinical efficacy and tolerability of fluoroquinolones when used for the treatment of pulmonary TB were evaluated for this review. Specifically, efficacy and safety in the following indications were investigated: (i) first-line treatment of drug-sensitive pulmonary TB; (ii) first-line treatment for multi-drug resistant (MDR) TB; and (iii) treatment of patients with drug intolerance. Twenty-seven articles met our inclusion criteria; nine articles presented data from randomised, controlled or cohort studies. Seven studies used fluoroquinolones as first-line agents in drug-sensitive TB (1469 patients), 15 studies used fluoroquinolones to treat MDR-TB (1025 patients) and six studies (951 patients) investigated the use of fluoroquinolones in patients intolerant to other TB medications.In patients with susceptible M. tuberculosis strains, substitution with a fluoroquinolone did not have an effect on cure or radiological improvement at 8 weeks or failure at 12 months. Substitution of older fluoroquinolones into a regimen, especially ciprofloxacin, resulted in a higher rate of relapse and a longer time to sputum-culture conversions. The use of fluoroquinolones in patients with MDR-TB is supported by some trials where others show a lack of improvement in efficacy of a regimen.Our review of the literature does not support the use of older fluoroquinolones, especially ciprofloxacin, as substitute agents for drug-sensitive or drug-resistant TB. However, newer fluoroquinolones, such as moxifloxacin, may be a reasonable alternative based on results from one large clinical trial. Fluoroquinolones have an important role as substitute agents for those who are intolerant of first-line TB agents. © 2007 Adis Data Information BV. All rights reserved.

Institution

(Moadebi, Fitzgerald, Elwood, Marra) BC Centre for Disease Control, Vancouver, BC, Canada (Harder, Elwood, Marra) University of British Columbia, Vancouver, BC, Canada

(Fitzgerald) Centre for Clinical Epidemiology and Evaluation, Vancouver, BC, Canada

(Marra) BC Centre for Disease Control, Vaccine and Pharmacy Services, 655 West 12th Avenue, Vancouver, BC V5Z 1L8, Canada

Publisher

Adis International Ltd

Emtree Heading

absence of side effects/si [Side Effect]; acute kidney failure/si [Side Effect]; add on therapy; ageusia/si [Side Effect]; case study; central nervous system disease/si [Side Effect]; clinical study; clinical trial; cohort analysis; disease severity; drug dose comparison; drug effect; drug efficacy; drug hypersensitivity; drug induced headache/si [Side Effect]; drug megadose; drug safety; drug substitution; drug tolerability; drug treatment failure; drug withdrawal; gastrointestinal disease/si [Side Effect]; gastrointestinal symptom/si [Side Effect]; heart arrhythmia/si [Side Effect]; human; insulin dependent diabetes mellitus/si [Side Effect]; low drug dose; *lung tuberculosis/dt [Drug Therapy]; Medline; monotherapy; myalgia/si [Side Effect]; nausea/si [Side Effect]; outcome assessment; photosensitivity/si [Side Effect]; rash/si [Side Effect]; review; single drug dose; sputum culture; sputum smear; statistical analysis; treatment duration; treatment planning; treatment response; unspecified side effect/si [Side Effect]; vertigo/si [Side Effect]; vomiting/si [Side Effect]; aminosalicylic acid/ae [Adverse Drug Reaction]; aminosalicylic acid/ct [Clinical Trial]; aminosalicylic acid/dt [Drug Therapy]; capreomycin/ae [Adverse Drug Reaction]; capreomycin/ct [Clinical Trial]; capreomycin/dt [Drug Therapy]; capreomycin/im [Intramuscular Drug Administration]; *ciprofloxacin/ae [Adverse Drug Reaction]; *ciprofloxacin/ct [Clinical Trial]; *ciprofloxacin/cm [Drug Comparison]; *ciprofloxacin/do [Drug Dose]; *ciprofloxacin/dt [Drug Therapy]; clofazimine/ae [Adverse Drug Reaction]; clofazimine/ct [Clinical Trial]; clofazimine/dt [Drug Therapy]; clofazimine/im [Intramuscular Drug Administration]; cycloserine/ae [Adverse Drug Reaction]; cycloserine/ct [Clinical Trial]; cycloserine/dt [Drug Therapy]; ethambutol/ae [Adverse Drug Reaction]; ethambutol/ct [Clinical Trial]; ethambutol/cm [Drug Comparison]; ethambutol/dt [Drug Therapy]; ethionamide/ae [Adverse Drug Reaction]; ethionamide/ct [Clinical Trial]; ethionamide/cb [Drug Combination]; ethionamide/dt [Drug Therapy]; isoniazid/ae [Adverse Drug Reaction]; isoniazid/ct [Clinical Trial]; isoniazid/cb [Drug Combination]; isoniazid/cm [Drug Comparison]; isoniazid/dt [Drug Therapy]; kanamycin/ae [Adverse Drug Reaction]; kanamycin/ct [Clinical Trial]; kanamycin/cb [Drug Combination]; kanamycin/dt [Drug Therapy]; kanamycin/im [Intramuscular Drug Administration]; levofloxacin/ae [Adverse Drug Reaction]; levofloxacin/ct [Clinical Trial]; levofloxacin/cm [Drug Comparison]; levofloxacin/dt [Drug Therapy]; *moxifloxacin/ae [Adverse Drug Reaction]; *moxifloxacin/ct [Clinical Trial]; *moxifloxacin/cb [Drug Combination]; *moxifloxacin/dt [Drug Therapy]; *ofloxacin/ae [Adverse Drug Reaction]; *ofloxacin/ct [Clinical Trial]; *ofloxacin/cm [Drug Comparison]; *ofloxacin/do [Drug Dose]; *ofloxacin/dt [Drug Therapy]; pefloxacin/ae [Adverse Drug Reaction]; pefloxacin/ct [Clinical Trial]; pefloxacin/cb [Drug Combination]; pefloxacin/dt [Drug Therapy]; protionamide/ae [Adverse Drug Reaction]; protionamide/ct [Clinical Trial]; protionamide/dt [Drug Therapy]; pyrazinamide/ae [Adverse Drug Reaction]; pyrazinamide/ct [Clinical Trial]; pyrazinamide/cb [Drug Combination]; pyrazinamide/cm [Drug Comparison]; pyrazinamide/dt [Drug Therapy]; quinoline derived antiinfective agent/ae [Adverse Drug Reaction]; quinoline derived antiinfective agent/ct [Clinical Trial]; quinoline derived antiinfective agent/dt [Drug Therapy]; rifabutin/ae [Adverse Drug Reaction]; rifabutin/ct [Clinical Trial]; rifabutin/dt [Drug Therapy]; rifampicin/ae [Adverse Drug Reaction]; rifampicin/ct [Clinical Trial]; rifampicin/cb [Drug Combination]; rifampicin/cm [Drug Comparison]; rifampicin/dt [Drug Therapy]; sparfloxacin/ae [Adverse Drug Reaction]; sparfloxacin/ct [Clinical Trial]; sparfloxacin/cb [Drug Combination]; sparfloxacin/dt [Drug Therapy]; streptomycin/ae [Adverse Drug Reaction]; streptomycin/ct [Clinical Trial]; streptomycin/dt [Drug Therapy]; streptomycin/im [Intramuscular Drug Administration]; thioacetazone/ae [Adverse Drug Reaction]; thioacetazone/ct [Clinical Trial]; thioacetazone/cb [Drug Combination]; thioacetazone/dt [Drug Therapy].

Link to the Ovid Full Text or citation:

Click here for full text options

105.

Immunological properties of engineered nanomaterials.

Dobrovolskaia M.A., McNeil S.E.

Nature Nanotechnology. 2 (8) (pp 469-478), 2007. Date of Publication: August 2007.

AN: 2007387404

Most research on the toxicology of nanomaterials has focused on the effects of nanoparticles that enter the body accidentally. There has been much less research on the toxicology of nanoparticles that are used for biomedical applications, such as drug delivery or imaging, in which the nanoparticles are deliberately placed in the body. Moreover, there are no harmonized standards for assessing the toxicity of nanoparticles to the immune system (immunotoxicity). Here we review recent research on immunotoxicity, along with data on a range of nanotechnology-based drugs that are at different stages in the approval process. Research shows that nanoparticles can stimulate and/or suppress the immune responses, and that their compatibility with the immune system is largely determined by their surface chemistry. Modifying these factors can significantly reduce the immunotoxicity of nanoparticles and make them useful platforms for drug delivery. © 2007 Nature Publishing Group.

Institution

(Dobrovolskaia, McNeil) Nanotechnology Characterization Laboratory, Advanced Technology Program, NCI-Frederick, 1050 Boyles St., Frederick, MD 21702, United States

Publisher

Nature Publishing Group

Emtree Heading

antigenicity; autoimmune disease/si [Side Effect]; blood clotting time; breast cancer/dt [Drug Therapy]; cancer chemotherapy; clinical trial; complement activation; cytotoxicity; drug administration route; drug delivery system; drug hypersensitivity/si [Side Effect]; endocytosis; erythrocyte aplasia/si [Side Effect]; head and neck cancer/dt [Drug Therapy]; hemolysis; hepatitis C/dt [Drug Therapy]; human; *immune response; *immune system; immunogenicity; immunotoxicity; infection/si [Side Effect]; inflammation; insulin dependent diabetes mellitus/dt [Drug Therapy]; Kaposi sarcoma/dt [Drug Therapy]; lung cancer/dt [Drug Therapy]; lung non small cell cancer/dt [Drug Therapy]; melanoma/dt [Drug Therapy]; mycosis/si [Side Effect]; nonhuman; ovary cancer/dt [Drug Therapy]; particle size; patent ductus arteriosus/dt [Drug Therapy]; pinocytosis; priority journal; review; skin allergy/si [Side Effect]; T cell lymphoma/si [Side Effect]; thrombocyte aggregation; treatment outcome; tuberculosis/si [Side Effect]; biovant; calcium phosphate/ct [Clinical Trial]; calcium phosphate/dt [Drug Therapy]; calcium phosphate/im [Intramuscular Drug Administration]; calcium phosphate/pr [Pharmaceutics]; cyclodextrin/ct [Clinical Trial]; cyclodextrin/dt [Drug Therapy]; cyclodextrin/iv [Intravenous Drug Administration]; cyclodextrin/pr [Pharmaceutics]; dendrimer/pr [Pharmaceutics]; doxorubicin/dt [Drug Therapy]; doxorubicin/iv [Intravenous Drug Administration]; doxorubicin/pr [Pharmaceutics]; fullerene derivative/pr [Pharmaceutics]; gold/pr [Pharmaceutics]; ibuprofen lysine/dt [Drug Therapy]; ibuprofen lysine/iv [Intravenous Drug Administration]; ibuprofen lysine/pr [Pharmaceutics]; infliximab/ae [Adverse Drug Reaction]; infliximab/dt [Drug Therapy]; infliximab/iv [Intravenous Drug Administration]; infliximab/pr [Pharmaceutics]; liposome/pr [Pharmaceutics]; *nanomaterial/pr [Pharmaceutics]; *nanoparticle/pr [Pharmaceutics]; nanoshell/pr [Pharmaceutics]; neoprofen; paclitaxel/ae [Adverse Drug Reaction]; paclitaxel/dt [Drug Therapy]; paclitaxel/iv [Intravenous Drug Administration]; paclitaxel/pr [Pharmaceutics]; polyamidoamine/pr [Pharmaceutics]; quantum dot/pr [Pharmaceutics]; recombinant alpha2a interferon/ae [Adverse Drug Reaction]; recombinant alpha2a interferon/dt [Drug Therapy]; recombinant alpha2a interferon/im [Intramuscular Drug Administration]; recombinant alpha2a interferon/sc [Subcutaneous Drug Administration]; recombinant erythropoietin/ae [Adverse Drug Reaction]; recombinant erythropoietin/dt [Drug Therapy]; recombinant erythropoietin/iv [Intravenous Drug Administration]; recombinant erythropoietin/pr [Pharmaceutics]; recombinant erythropoietin/sc [Subcutaneous Drug Administration]; recombinant human insulin/ct [Clinical Trial]; recombinant human insulin/dt [Drug Therapy]; recombinant human insulin/pr [Pharmaceutics]; recombinant human insulin/sc [Subcutaneous Drug Administration]; ultrasmall superparamagnetic iron oxide/ae [Adverse Drug Reaction]; ultrasmall superparamagnetic iron oxide/iv [Intravenous Drug Administration]; ultrasmall superparamagnetic iron oxide/pr [Pharmaceutics]; unclassified drug; vivagel/pr [Pharmaceutics].

Drug Trade Names and Manufacturers

Abraxis, Advanced Magnetics, Amgen, Bedford, BioSante, Bms, centrocor, cytimmune, Flamel, Hoffmann La Roche, Imarx, insert, Introgen, nanospectra, Ortho, Ovation, Pharmacia Upjohn, Supergen, Triton, abraxane: Abraxis, basulin: Flamel, biovant: BioSante, doxil: Ortho, epogen: Amgen, neoprofen: Ovation, roferon a: Hoffmann La Roche

Link to the Ovid Full Text or citation:

Click here for full text options

106.

Antiretroviral treatment of HIV infection: Swedish recommendations 2007.

Josephson F., Albert J., Flamholc L., Gisslen M., Karlstrom O., Lindgren S.-R., Naver L., Sandstrom E., Svedhem-Johansson V., Svennerholm B., Sonnerborg A.

Scandinavian Journal of Infectious Diseases. 39 (6-7) (pp 486-507), 2007. Date of Publication: 2007.

AN: 2007321043

On 3 previous occasions, in 2002, 2003 and 2005, the Swedish Medical Products Agency (Lakemedelsverket) and the Swedish Reference Group for Antiviral Therapy (RAV) have jointly published recommendations for the treatment of HIV infection. An expert group, under the guidance of RAV, has now revised the text again. Since the publication of the previous treatment recommendations, 1 new drug for the treatment of HIV has been approved - the protease inhibitor (PI) darunavir (Prezista). Furthermore, 3 new drugs have become available: the integrase inhibitor raltegravir (MK-0518), the CCR5-inhibitor maraviroc (Celsentri), both of which have novel mechanisms of action, and the non-nucleoside reverse transcriptase inhibitor (NNRTI) etravirine (TMC-125). The new guidelines differ from the previous ones in several respects. The most important of these are that abacavir is now preferred to tenofovir and zidovudine, as a first line drug in treatment-naive patients, and that initiation of antiretroviral treatment is now recommended before the CD4 cell count falls below 250/mul, rather than 200/mul. Furthermore, recommendations on the treatment of HIV infection in children have been added to the document. As in the case of the previous publication, recommendations are evidence-graded in accordance with the Oxford Centre for Evidence Based Medicine, 2001 (see [http://www.cebm.net/levels_of_evidence.asp#levels](http://www.cebm.net/levels_of_evidence.asp#levels)).

Institution

(Josephson) Department of Clinical Pharmacology, Karolinska University Hospital, Uppsala, Sweden (Albert) Department of Microbiology, Tumour and Cell Biology (MTC), Karolinska Institute, Swedish Institute for Infectious Disease Control (SMI), Uppsala, Sweden

(Flamholc) Department of Infectious Diseases, Malmo University Hospital, Uppsala, Sweden

(Gisslen) Department of Infectious Diseases, Uppsala, Sweden

(Karlstrom) Medical Products Agency, Uppsala, Sweden

(Karlstrom, Svedhem-Johansson, Sonnerborg) Department of Infectious Diseases, South Hospital, Stockholm, Sweden

(Lindgren) Department of Obstetrics and Gynaecology, South Hospital, Stockholm, Sweden

(Naver) Department of Paediatrics, South Hospital, Stockholm, Sweden

(Sandstrom) Department of Venerology, South Hospital, Stockholm, Sweden

(Svennerholm) Clinical Virology, Sahlgrenska University Hospital, Goteborg, Sweden

(Sonnerborg) Department of Clinical Pharmacology, Karolinska University Hospital, Uppsala, Sweden

Publisher

Informa Healthcare

Emtree Heading

CD4 lymphocyte count; CD4+ T lymphocyte; central nervous system disease/si [Side Effect]; clinical trial; consensus; diabetes mellitus/si [Side Effect]; drug dose regimen; drug eruption/si [Side Effect]; drug exposure; drug hypersensitivity/si [Side Effect]; drug potentiation; drug substitution; drug treatment failure; drug withdrawal; dyslipidemia/si [Side Effect]; edema/si [Side Effect]; food; food drug interaction; gastrointestinal symptom/si [Side Effect]; heart infarction/si [Side Effect]; hepatitis A/dt [Drug Therapy]; hepatitis A/pc [Prevention]; hepatitis B/dt [Drug Therapy]; hepatitis B/pc [Prevention]; hepatitis C/dt [Drug Therapy]; human; *Human immunodeficiency virus infection/dt [Drug Therapy]; *Human immunodeficiency virus infection/pc [Prevention]; hyperbilirubinemia/si [Side Effect]; hyperlactatemia/si [Side Effect]; hyperlipidemia/si [Side Effect]; hypotension/si [Side Effect]; insulin resistance/si [Side Effect]; lactic acidosis/si [Side Effect]; lipoatrophy/si [Side Effect]; liver dysfunction/si [Side Effect]; liver injury/si [Side Effect]; lymphadenopathy/si [Side Effect]; metabolic disorder/si [Side Effect]; mitochondrial toxicity/si [Side Effect]; monotherapy; mother child relation; mucosa inflammation/si [Side Effect]; mycobacteriosis/dt [Drug Therapy]; nephrotoxicity/si [Side Effect]; non insulin dependent diabetes mellitus/si [Side Effect]; patient compliance; publication; review; side effect/si [Side Effect]; skin manifestation/si [Side Effect]; Stevens Johnson syndrome/si [Side Effect]; subcutaneous fat; superinfection/dt [Drug Therapy]; toxic epidermal necrolysis/si [Side Effect]; tuberculosis/dt [Drug Therapy]; viremia/dt [Drug Therapy]; virus transmission; abacavir/ae [Adverse Drug Reaction]; abacavir/ct [Clinical Trial]; abacavir/cb [Drug Combination]; abacavir/dt [Drug Therapy]; amprenavir phosphate/cb [Drug Combination]; amprenavir phosphate/dt [Drug Therapy]; *antiretrovirus agent/ae [Adverse Drug Reaction]; *antiretrovirus agent/it [Drug Interaction]; *antiretrovirus agent/dt [Drug Therapy]; atazanavir/ae [Adverse Drug Reaction]; atazanavir/ct [Clinical Trial]; atazanavir/cb [Drug Combination]; atazanavir/dt [Drug Therapy]; celsentri; chemokine receptor CCR5 antagonist/ct [Clinical Trial]; chemokine receptor CCR5 antagonist/dt [Drug Therapy]; darunavir/cb [Drug Combination]; darunavir/it [Drug Interaction]; darunavir/dt [Drug Therapy]; didanosine/ae [Adverse Drug Reaction]; didanosine/it [Drug Interaction]; didanosine/dt [Drug Therapy]; efavirenz/ae [Adverse Drug Reaction]; efavirenz/ct [Clinical Trial]; efavirenz/cb [Drug Combination]; efavirenz/it [Drug Interaction]; efavirenz/dt [Drug Therapy]; efavirenz/to [Drug Toxicity]; emtricitabine/cm [Drug Comparison]; emtricitabine/dt [Drug Therapy]; enfuvirtide/dt [Drug Therapy]; etravirine/dt [Drug Therapy]; indinavir/cb [Drug Combination]; indinavir/dt [Drug Therapy]; integrase inhibitor/ct [Clinical Trial]; integrase inhibitor/dt [Drug Therapy]; lamivudine/ae [Adverse Drug Reaction]; lamivudine/cm [Drug Comparison]; lamivudine/dt [Drug Therapy]; lamivudine plus zidovudine/dt [Drug Therapy]; lopinavir/dt [Drug Therapy]; lopinavir plus ritonavir/cb [Drug Combination]; lopinavir plus ritonavir/do [Drug Dose]; lopinavir plus ritonavir/it [Drug Interaction]; lopinavir plus ritonavir/dt [Drug Therapy]; maraviroc/dt [Drug Therapy]; mk 0518; nelfinavir/dt [Drug Therapy]; nevirapine/ae [Adverse Drug Reaction]; nevirapine/cb [Drug Combination]; nevirapine/dt [Drug Therapy]; prezista; proteinase inhibitor/ae [Adverse Drug Reaction]; proteinase inhibitor/cb [Drug Combination]; proteinase inhibitor/dt [Drug Therapy]; raltegravir/dt [Drug Therapy]; ritonavir/ae [Adverse Drug Reaction]; ritonavir/ct [Clinical Trial]; ritonavir/cb [Drug Combination]; ritonavir/it [Drug Interaction]; ritonavir/dt [Drug Therapy]; RNA directed DNA polymerase inhibitor/ae [Adverse Drug Reaction]; RNA directed DNA polymerase inhibitor/cm [Drug Comparison]; RNA directed DNA polymerase inhibitor/dt [Drug Therapy]; saquinavir/cb [Drug Combination]; saquinavir/dt [Drug Therapy]; stavudine/ae [Adverse Drug Reaction]; stavudine/ct [Clinical Trial]; stavudine/dt [Drug Therapy]; tenofovir/ae [Adverse Drug Reaction]; tenofovir/ct [Clinical Trial]; tenofovir/cb [Drug Combination]; tenofovir/cm [Drug Comparison]; tenofovir/dt [Drug Therapy]; tenofovir disoproxil; tipranavir/ae [Adverse Drug Reaction]; tipranavir/cb [Drug Combination]; tipranavir/dt [Drug Therapy]; unclassified drug; unindexed drug; zidovudine/ae [Adverse Drug Reaction]; zidovudine/dt [Drug Therapy].

Drug Trade Names and Manufacturers

aptivus, celsentri, combivir, crixivan, emtriva, epivir, fuzeon, invirase, kaletra, mk 0518, norvir, prezista, retrovir, reyataz, stocrin, t 20, telzir, tmc 114, tmc 125, videx, viracept, viramune, viread, zerit, ziagen

Link to the Ovid Full Text or citation:

Click here for full text options

107.

Tumor necrosis factor antagonists: Preliminary evidence for an emerging approach in the treatment of ocular inflammation.

Theodossiadis P.G., Markomichelakis N.N., Sfikakis P.P.

Retina. 27 (4) (pp 399-413), 2007. Date of Publication: April/May 2007.

AN: 2007194777

The anti-tumor necrosis factor (TNF) monoclonal antibody infliximab and the soluble TNF receptor etanercept inhibit the pleiotropic actions of TNF and are widely used for the treatment of rheumatoid arthritis (RA), juvenile idiopathic arthritis (JIA), spondyloarthropathies (SpA), Crohn's disease, and psoriasis with an acceptable safety profile. A pathogenetic role of TNF in ocular inflammatory conditions has recently emerged from small trials reporting preliminary results on the efficacy of these agents in patients with noninfectious uveitis, regardless of the origin of the disease. The authors review the published experience, derived mostly from investigator-sponsored trials and uncontrolled case series, on the use of TNF antagonists in approximately 280 patients with various ocular conditions who were inadequately controlled on currently available therapy. These reports suggest that TNF antagonists, mainly infliximab, which may have better efficacy than etanercept, are useful in the treatment of ocular inflammation associated with Adamantiades-Behcet's disease, RA, JIA, SpA, Crohn's, sarcoidosis, and Graves' disease ophthalmopathy. Infliximab was also beneficial in small numbers of patients with idiopathic uveitis or scleritis, birdshot retinochoroiditis, uveitic and diabetic cystoid macular edema, and age-related macular degeneration. The currently available data are nonrandomized and thus preliminary, providing the foundation and justification for randomized trials to assess efficacy and safety. Until such results are available, knowledge regarding the use of anti-TNF regimens in ophthalmology is incomplete. However, the preliminary evidence points to a growing optimism for targeting TNF in patients with ocular inflammation. © The Ophthalmic Communications Society, Inc.

Institution

(Theodossiadis) Second Department of Ophthalmology, Laikon Hospital, Athens University Medical School (Sfikakis) First Department of Propedeutic and Internal Medicine, Laikon Hospital, Athens University Medical School

(Markomichelakis) Ocular Inflammation and Immunology Service, General Hospital, Greece

(Theodossiadis) 13 Lykiou Street, Kolonaki, Athens, Greece

Publisher

Lippincott Williams and Wilkins

Emtree Heading

ankylosing spondylitis/dt [Drug Therapy]; aspergillosis/si [Side Effect]; autoimmune disease/si [Side Effect]; cancer recurrence/si [Side Effect]; cataract/si [Side Effect]; chorioretinitis/dt [Drug Therapy]; clinical trial; congestive heart failure/si [Side Effect]; continuous infusion; Crohn disease/dt [Drug Therapy]; demyelinating disease/si [Side Effect]; diabetic macular edema/dt [Drug Therapy]; disease association; drug efficacy; drug hypersensitivity/si [Side Effect]; drug mechanism; drug safety; endocrine ophthalmopathy; endometrium cancer/si [Side Effect]; *eye inflammation/co [Complication]; *eye inflammation/dt [Drug Therapy]; *eye inflammation/et [Etiology]; glaucoma/si [Side Effect]; Graves disease; heart failure/si [Side Effect]; histoplasmosis/si [Side Effect]; human; immunosuppressive treatment; infection/si [Side Effect]; intraocular hypertension/si [Side Effect]; juvenile rheumatoid arthritis/dt [Drug Therapy]; listeriosis/si [Side Effect]; lung infection/si [Side Effect]; lupus like syndrome/si [Side Effect]; lymphoma/si [Side Effect]; malignant neoplastic disease/si [Side Effect]; multiple sclerosis/si [Side Effect]; myositis/si [Side Effect]; neurologic disease/si [Side Effect]; nonhuman; optic neuritis/si [Side Effect]; Pneumocystis pneumonia/si [Side Effect]; psoriasis/dt [Drug Therapy]; psoriasis/si [Side Effect]; psoriatic arthritis/dt [Drug Therapy]; retina macula age related degeneration/dt [Drug Therapy]; review; rheumatoid arthritis/dt [Drug Therapy]; sarcoidosis/dt [Drug Therapy]; scleritis; solid tumor/si [Side Effect]; spondyloarthropathy/dt [Drug Therapy]; systemic disease; thrombosis/si [Side Effect]; treatment failure; tuberculosis/si [Side Effect]; uveitis/co [Complication]; uveitis/dt [Drug Therapy]; uveitis/et [Etiology]; vitreous hemorrhage/si [Side Effect]; adalimumab/cb [Drug Combination]; adalimumab/dt [Drug Therapy]; antirheumatic agent/cb [Drug Combination]; antirheumatic agent/dt [Drug Therapy]; azathioprine/cb [Drug Combination]; azathioprine/dt [Drug Therapy]; corticosteroid/cb [Drug Combination]; corticosteroid/dt [Drug Therapy]; cyclosporin/cb [Drug Combination]; cyclosporin/dt [Drug Therapy]; etanercept/ae [Adverse Drug Reaction]; etanercept/ct [Clinical Trial]; etanercept/cb [Drug Combination]; etanercept/cm [Drug Comparison]; etanercept/dt [Drug Therapy]; etanercept/pd [Pharmacology]; infliximab/ae [Adverse Drug Reaction]; infliximab/ct [Clinical Trial]; infliximab/cb [Drug Combination]; infliximab/cm [Drug Comparison]; infliximab/cr [Drug Concentration]; infliximab/dt [Drug Therapy]; infliximab/iv [Intravenous Drug Administration]; infliximab/pd [Pharmacology]; methotrexate/ct [Clinical Trial]; methotrexate/cb [Drug Combination]; methotrexate/dt [Drug Therapy]; mycophenolic acid 2 morpholinoethyl ester/dt [Drug Therapy]; placebo; salazosulfapyridine/cb [Drug Combination]; salazosulfapyridine/dt [Drug Therapy]; steroid/dt [Drug Therapy]; steroid/tp [Topical Drug Administration]; tacrolimus/dt [Drug Therapy]; *tumor necrosis factor antibody/ae [Adverse Drug Reaction]; *tumor necrosis factor antibody/ct [Clinical Trial]; *tumor necrosis factor antibody/cb [Drug Combination]; *tumor necrosis factor antibody/dt [Drug Therapy].

Link to the Ovid Full Text or citation:

Click here for full text options

108.

Treatment outcome of pulmonary tuberculosis in eastern Taiwan - Experience at a Medical Center.

Lee J.-J., Wu R.-L., Lee Y.-S., Wu Y.-C., Chiang C.-Y.

Journal of the Formosan Medical Association. 106 (1) (pp 25-30), 2007. Date of Publication: January 2007.

AN: 2007077270

Background/Purpose: The World Health Organization (WHO) has proposed that treatment outcome is an important indicator of tuberculosis control. This study investigated the outcome of tuberculosis treatment at a medical center in eastern Taiwan. Methods: A total of 166 pulmonary tuberculosis patients notified by Tzu Chi Hospital in 2002 were included in this study. Treatment outcome data were collected at the local level and categorized according to WHO recommendations as cured, treatment completed, failed, died, defaulted, or transferred. Outcomes of the 166 patients, as reported by the National Tuberculosis Program were obtained from the Taiwan Center for Disease Control (Taiwan CDC) for comparison. Results: Of the 166 patients, outcome was classified as cured in 46 (27.7%), treatment completed in 73 (44.0%), died in 27 (16.3%), treatment failed in five (3.0%), and defaulted in 15 (9.0%). Males were more likely to die or to default than females, and the elderly were more likely to die than younger patients. Patients with comorbidities were significantly more likely to die than patients without (p=0.025). Patients with a history of tuberculosis were more likely to default (p = 0.050). Smear-positive patients were more likely to fail, and patients without cavitation on chest radiograph were more likely to have successful treatment. Outcomes of 26 (15.7%) cases in this cohort were unavailable (18 cases) or inaccurate (8 cases) on the Taiwan CDC website. Conclusion: The unsatisfactory outcome of pulmonary tuberculosis treatment in this medical center in eastern Taiwan highlights the importance of implementing directly observed treatment short course strategy. Improvement in the quality of data reported by the National Tuberculosis Program is urgently needed. © 2007 Elsevier & Formosan Medical Association.

Institution

(Lee) Department of Internal Medicine, Buddhist Tzu Chi General Hospital, Tzu Chi University, Hualien, Taiwan (Republic of China) (Wu) Department of Public Health, Tzu Chi University, Hualien, Taiwan (Republic of China)

(Lee, Wu) Center for Disease Control, Taipei, Taiwan (Republic of China)

(Chiang) International Union Against Tuberculosis and Lung Diseases, Paris, France

(Chiang) Kuang-Fu South Road, Taipei 105, Taiwan (Republic of China)

Publisher

Scientific Communications International Ltd

Emtree Heading

adolescent; adult; aged; article; cancer; child; clinical trial; comorbidity; diabetes mellitus; drug fever/si [Side Effect]; drug substitution; drug tolerability; drug withdrawal; female; human; liver cirrhosis; lung cavitation/di [Diagnosis]; lung cavitation/dt [Drug Therapy]; *lung tuberculosis/di [Diagnosis]; *lung tuberculosis/dr [Drug Resistance]; *lung tuberculosis/dt [Drug Therapy]; major clinical study; male; patient attitude; pneumoconiosis; sputum examination; survival rate; Taiwan; thorax radiography; toxic hepatitis/si [Side Effect]; treatment outcome; uremia; *ethambutol/ae [Adverse Drug Reaction]; *ethambutol/cb [Drug Combination]; *ethambutol/dt [Drug Therapy]; *isoniazid/ae [Adverse Drug Reaction]; *isoniazid/cb [Drug Combination]; *isoniazid/dt [Drug Therapy]; *pyrazinamide/ae [Adverse Drug Reaction]; *pyrazinamide/cb [Drug Combination]; *pyrazinamide/dt [Drug Therapy]; *rifampicin/ae [Adverse Drug Reaction]; *rifampicin/cb [Drug Combination]; *rifampicin/dt [Drug Therapy].

Link to the Ovid Full Text or citation:

Click here for full text options

109.

HCV-related advanced fibrosis/cirrhosis: Randomized controlled trial of pegylated interferon alpha-2a and ribavirin.

Helbling B., Jochum W., Stamenic I., Knopfli M., Cerny A., Borovicka J., Gonvers J.-J., Wilhelmi M., Dinges S., Mullhaupt B., Esteban A., Meyer-Wyss B., Renner E.L.

Journal of Viral Hepatitis. 13 (11) (pp 762-769), 2006. Date of Publication: November 2006.

AN: 2006511425

In patients with hepatitis C virus (HCV)-related advanced fibrosis/cirrhosis, 30% of sustained HCV clearance has been reported with pegylated interferon alpha-2a (PEG-IFN) alone, but the efficacy and tolerability of the PEG-IFN/ribavirin (RBV) combination remain poorly defined. A total of 124 treatment-naive patients with biopsy proved HCV-related advanced fibrosis/cirrhosis (Ishak score F4-F6, Child-Pugh score <7) were randomized to 48 weeks of PEG-IFN (180 mug sc weekly) and standard dose of RBV (1000/1200 mg po daily, STD) or PEG-IFN (180 mug sc weekly) and low-dose of RBV (600/800 mg po daily, LOW). Sustained virologic response (SVR) rates with PEG-IFN/STD RBV (52%) were higher - albeit not significantly - than that with PEG-IFN/LOW RBV (38%, P = 0.153). In multivariate analysis, genotype 2/3 and a baseline platelet count >150 x 109/L were independently associated with SVR. The likelihood of SVR was <7% if viraemia had not declined by >2 log or to undetectable levels after 12 weeks. Nine adverse events in the STD RBV and 15 in the LOW RBV group were classified as severe (including two deaths); dose reductions for intolerance were required in 78% and 57% (P = 0.013), and treatment was terminated early in 23% and 27% of patients (P = n.s.). The benefit/risk ratio of treating compensated HCV-cirrhotics with STD PEG-IFN/RBV is favourable. © 2006 The Authors.

Institution

(Helbling, Stamenic, Wilhelmi, Dinges, Mullhaupt, Renner) Division of Gastroenterology and Hepatology, University Hospital, Zurich, Switzerland (Helbling, Borovicka) Division of Gastroenterology/Hepatology, Cantonal Hospital, St Gallen, Switzerland

(Jochum) Department of Pathology, University Hospital, Zurich, Switzerland

(Knopfli, Cerny) Department of Internal Medicine, Cantonal Hospital, Lugano, Switzerland

(Gonvers) Division of Gastroenterology, University Hospital, Lausanne, Switzerland

(Esteban) Roche Pharma (Schweiz) AG, Reinach, Switzerland

(Meyer-Wyss) Division of Gastroenterology, St. Claraspital, Basel, Switzerland

(Renner) Department of Internal Medicine, Health Sciences Center, University of Manitoba, Winnipeg, Man., Canada

(Renner) Department of Internal Medicine, University of Manitoba, John Buhler Research Centre, McDermot Avenue, Winnipeg, Man. R3A 1R9, Canada

Publisher

Blackwell Publishing Ltd (9600 Garsington Road, Oxford OX4 2XG, United Kingdom)

Emtree Heading

abdominal bleeding/si [Side Effect]; acute coronary syndrome/si [Side Effect]; adult; aged; article; cancer recurrence/si [Side Effect]; cardiovascular disease/si [Side Effect]; clinical trial; controlled clinical trial; controlled study; depression/si [Side Effect]; diabetes mellitus/si [Side Effect]; dose response; drug dose reduction; drug dose regimen; drug effect; drug efficacy; drug fatality/si [Side Effect]; eczema/si [Side Effect]; endocrine disease/si [Side Effect]; eye disease/si [Side Effect]; female; gastroenteritis/si [Side Effect]; gastrointestinal disease/si [Side Effect]; genotype; hemiparesis/si [Side Effect]; *hepatitis C/dt [Drug Therapy]; *hepatitis C/et [Etiology]; Hepatitis C virus; human; infection/si [Side Effect]; insomnia/si [Side Effect]; leukopenia/si [Side Effect]; liver biopsy; liver cell carcinoma/si [Side Effect]; *liver cirrhosis/co [Complication]; *liver cirrhosis/di [Diagnosis]; *liver fibrosis/co [Complication]; *liver fibrosis/di [Diagnosis]; lumbar disk hernia/si [Side Effect]; lung tuberculosis/si [Side Effect]; major clinical study; male; mental disease/si [Side Effect]; metabolic disorder/si [Side Effect]; multicenter study; multivariate analysis; neoplasm/si [Side Effect]; nervousness; neurologic disease/si [Side Effect]; nonhodgkin lymphoma/si [Side Effect]; open study; outcome assessment; peroneus nerve paralysis/si [Side Effect]; pleura empyema/si [Side Effect]; pneumonia/si [Side Effect]; polyneuropathy/si [Side Effect]; priority journal; psychosis/si [Side Effect]; randomized controlled trial; risk benefit analysis; septic shock/si [Side Effect]; skin disease/si [Side Effect]; suicide attempt/si [Side Effect]; thrombocyte count; thrombocytopenia/si [Side Effect]; tongue carcinoma/si [Side Effect]; viremia; visual impairment/si [Side Effect]; *peginterferon alpha2a/ae [Adverse Drug Reaction]; *peginterferon alpha2a/ct [Clinical Trial]; *peginterferon alpha2a/cb [Drug Combination]; *peginterferon alpha2a/do [Drug Dose]; *peginterferon alpha2a/dt [Drug Therapy]; *peginterferon alpha2a/sc [Subcutaneous Drug Administration]; *ribavirin/ae [Adverse Drug Reaction]; *ribavirin/ct [Clinical Trial]; *ribavirin/cb [Drug Combination]; *ribavirin/do [Drug Dose]; *ribavirin/dt [Drug Therapy]; *ribavirin/po [Oral Drug Administration].

Drug Trade Names and Manufacturers

Roche Pharma [Switzerland]

Link to the Ovid Full Text or citation:

Click here for full text options

110.

Peptide Immunotherapy.

Larche M.

Immunology and Allergy Clinics of North America. 26 (2) (pp 321-332), 2006. Date of Publication: May 2006.

AN: 2006401402

Synthetic peptides representing T-cell epitopes of allergens and autoantigens have been employed to induce antigen-specific tolerance in vivo in experimental models and the clinical setting. Delivery of peptides orally or by injection leads to reduced reactivity to antigen accompanied by the induction of T cells with a regulatory phenotype. Peptide therapy may provide a safe, effective, and economically viable approach for disease-modifying therapy in autoimmune and allergic diseases. © 2006 Elsevier Inc. All rights reserved.

Institution

(Larche) Department of Allergy and Clinical Immunology, Faculty of Medicine, Imperial College, Sir Alexander Fleming Bldg, Rm 360, London, SW7 2AZ, United Kingdom

Publisher

W.B. Saunders

Emtree Heading

allergic encephalomyelitis/dt [Drug Therapy]; allergic rhinitis/dt [Drug Therapy]; *allergy/dm [Disease Management]; *allergy/dt [Drug Therapy]; *allergy/pc [Prevention]; anaphylaxis/dt [Drug Therapy]; anaphylaxis/pc [Prevention]; antigen specificity; arthritis/dt [Drug Therapy]; arthritis/et [Etiology]; arthritis/pc [Prevention]; asthma/dt [Drug Therapy]; *autoimmune disease/dt [Drug Therapy]; autoimmunity; cat; CD4+ T lymphocyte; cell function; cell interaction; clinical assessment; clinical trial; concentration response; cytokine production; cytokine release; dander; diabetes mellitus/dt [Drug Therapy]; diabetes mellitus/pc [Prevention]; drug binding; drug efficacy; drug hypersensitivity/dt [Drug Therapy]; drug hypersensitivity/si [Side Effect]; drug mechanism; drug reexposure; drug safety; drug tolerability; drug tolerance; erythema/si [Side Effect]; experimental model; human; immunological tolerance; immunoreactivity; immunostimulation; *immunotherapy; in vitro study; in vivo study; injection; major histocompatibility complex; multiple sclerosis/dt [Drug Therapy]; Mycobacterium tuberculosis; nebulization; nonhuman; peripheral blood mononuclear cell; phenotype; priority journal; prophylaxis; provocation test; pruritus/si [Side Effect]; quality of life; regulatory T lymphocyte; review; rhinitis; side effect/si [Side Effect]; T lymphocyte; T lymphocyte activation; Th1 cell; Th2 cell; treatment duration; treatment outcome; treatment response; unspecified side effect/si [Side Effect]; adrenalin/dt [Drug Therapy]; allergen/ct [Clinical Trial]; allergen/cb [Drug Combination]; allergen/dt [Drug Therapy]; allergen/dl [Intradermal Drug Administration]; allergen/na [Intranasal Drug Administration]; allergen/pd [Pharmacology]; aluminum potassium sulfate/cb [Drug Combination]; aluminum potassium sulfate/pd [Pharmacology]; autoantigen; bee venom; collagen type 2/dt [Drug Therapy]; collagen type 2/na [Intranasal Drug Administration]; collagen type 2/pd [Pharmacology]; cytokine/ec [Endogenous Compound]; epitope; glutamate decarboxylase 65/dt [Drug Therapy]; glutamate decarboxylase 65/na [Intranasal Drug Administration]; glutamate decarboxylase 65/pd [Pharmacology]; heat shock protein/do [Drug Dose]; heat shock protein/dt [Drug Therapy]; heat shock protein/po [Oral Drug Administration]; heat shock protein 60/dt [Drug Therapy]; heat shock protein 60/na [Intranasal Drug Administration]; heat shock protein 60/pd [Pharmacology]; heat shock protein 60/sc [Subcutaneous Drug Administration]; insect venom; insulin derivative/dt [Drug Therapy]; insulin derivative/na [Intranasal Drug Administration]; insulin derivative/sc [Subcutaneous Drug Administration]; interleukin 10/ec [Endogenous Compound]; major histocompatibility antigen class 2/ec [Endogenous Compound]; myelin basic protein/ae [Adverse Drug Reaction]; myelin basic protein/ad [Drug Administration]; myelin basic protein/dt [Drug Therapy]; myelin basic protein/na [Intranasal Drug Administration]; myelin basic protein/po [Oral Drug Administration]; myelin basic protein/pd [Pharmacology]; myelin basic protein/sc [Subcutaneous Drug Administration]; phospholipase A2/ct [Clinical Trial]; phospholipase A2/cb [Drug Combination]; phospholipase A2/dt [Drug Therapy]; phospholipase A2/pd [Pharmacology]; sodium chloride; *synthetic peptide/ae [Adverse Drug Reaction]; *synthetic peptide/ct [Clinical Trial]; *synthetic peptide/ad [Drug Administration]; *synthetic peptide/cb [Drug Combination]; *synthetic peptide/do [Drug Dose]; *synthetic peptide/dt [Drug Therapy]; *synthetic peptide/ih [Inhalational Drug Administration]; *synthetic peptide/dl [Intradermal Drug Administration]; *synthetic peptide/na [Intranasal Drug Administration]; *synthetic peptide/ip [Intraperitoneal Drug Administration]; *synthetic peptide/iv [Intravenous Drug Administration]; *synthetic peptide/po [Oral Drug Administration]; *synthetic peptide/pd [Pharmacology]; *synthetic peptide/sc [Subcutaneous Drug Administration]; transforming growth factor beta/ec [Endogenous Compound].

Link to the Ovid Full Text or citation:

Click here for full text options

111.

Evaluation of subcutaneous proleukin (interleukin-2) in a randomized international trial (ESPRIT): Geographical and gender differences in the baseline characteristics of participants.

Pett S.L., Wand H., Law M.G., Arduino R., Lopez J.C., Knysz B., Pereira L.C., Pollack S., Reiss P., Tambussi G., Abrams D.I., Cooper D.A., Darbyshire J.H., Duncan W.R., Emery S., Clifford Lane H., Lehrman S., Lundgren J.D., Neaton J.D., Aguilar L., Angel E.B., Aquilia S., Belloso W., Benetucci J., Bittar V., Cahn P., Casiro A., Contarelli J., Corral J., Daciuk L., David D., Ines F., Fridman D., Galache V., Guaragna G., Ivalo S., Laplume H., Lasala M.B., Lattes R., Lasovsky J., Lopardo G., Losso M., Lourtau L., Lupo S., Maranzana A., Marson C., Massera L., del Lujan Sanchez M., Somenzini C., Tocci M., Algar S., Anderson J., Austin D., Baker D., Blavius K., Bloch M., Boyle M., Bradford D., Carrall L., Carr A., Chuah J., Curry M., D'Arcy-Evans C., Dobson P., Doong N., Ferguson W., Finlayson R., French M., Gold J., Habel P., Hoy J., Hudson J., James R., Jeganathan S., Leung J., Lowe K., MacRae K., Mallal S., McMurchie M., Medland N., Mijch A., Miller S., Murray J., Orth D., Patching J., Primrose R., Quan D., Rawlinson M., Ree H., Richardson R., Rogers G., Roney J., Roth N., Shaw D., Soo T.M., Sowden D., Street A., Vale R.

HIV Clinical Trials. 7 (2) (pp 70-85), 2006. Date of Publication: March/April 2006.

AN: 2006345637

Background: ESPRIT, is a phase III, open-label, randomized, international clinical trial evaluating the effects of subcutaneous recombinant interleukin-2 (rIL-2) plus antiretroviral therapy (ART) versus ART alone on HIV-disease progression and death in HIV-1-infected individuals with CD4+ T-cells >300 cells/muL. Objectives: To describe the baseline characteristics of participants randomized to ESPRIT overall and by geographic location. Method: Baseli ne characteristics of randomized participants were summarized by region. Results: 4,150 patients were enrolled in ESPRIT from 254 sites in 25 countries. 41%, 27%, 16%, 11%, and 5% were enrolled in Europe, North America, South America, Asia, and Australia, respectively. The median age was 40 years, 81% were men, and 76%, 11%, and 9% were Caucasian, Asian, and African American or African, respectively. 44% of women enrolled (n = 769) were enrolled in Thailand and Argentina. Overall, 55% and 38% of the cohort acquired HIV through male homosexual and heterosexual contact, respectively. 25% had a prior history of AIDS-defining illness; Pneumocystis jirovecii pneumonia, M. tuberculosis, and esophageal candida were most commonly reported. Median nadir and baseline CD4+ T-cell counts were 199 and 458 cells/muL, respectively. 6% and 13% were hepatitis B or C virus coinfected, respectively. Median duration of antiretroviral therapy (ART) was 4.2 years; the longest median duration was in Australia (5.2 years) and the shortest was in Asia (2.3 years). 17%, 13%, and 69% of participants began ART before 1995, between 1996 and 1997, and from 1998 onward, respectively. 86% used ART from two or more ART classes, with 49% using a protease inhibitor-based regimen and 46% using a nonnucleoside reverse transcriptase inhibitor-based regimen. 78% had plasma HIV RNA below detection (<500 cp/mL). Conclusion: ESPRIT has enrolled a diverse population of HIV-infected individuals including large populations of women and patients of African-American/African and Asian ethnicity often underrepresented in HIV research. As a consequence, the results of the study may have wide global applicability. © 2006 Thomas Land Publishers, Inc.

Institution

(Pett, Wand, Law) National Centre in HIV Epidemiology and Clinical Research, University of New South Wales, 376 Victoria St., Sydney, NSW 2010, Australia (Arduino) University of Texas Medical School of Houston, Houston, TX, United States

(Lopez) Hospital Universitario Gergorio Maranon, Servicio de Micobiologica y Enf. Infecciosas, Madrid, Spain

(Knysz) Medical University of Wroclaw, Department of Infectiou Diseases, Wroclaw University School of Medicine, Wroclaw, Poland

(Pereira) Instituto de Infectologia Emillio Ribas, Sao Paulo, Brazil

(Pollack) Rambam Medical Center, Immunology Allergy and AIDS Institute, B. Rappaport Faculty of Medicine, Technion, Haifa, Israel

(Reiss) Academish Medisch Centrum - Amsterdam, Amsterdam, Netherlands

(Tambussi) Ospedale San Raffaele, Divisione Malattie Infettive - Centro San Luigi, Milano, Italy

(Aguilar, Angel, Aquilia, Belloso, Benetucci, Bittar, Cahn, Casiro, Contarelli, Corral, Daciuk, David, Ines, Fridman, Galache, Guaragna, Ivalo, Laplume, Lasala, Lattes, Lasovsky, Lopardo, Losso, Lourtau, Lupo, Maranzana, Marson, Massera, del Lujan Sanchez, Somenzini, Tocci) Argentina National Coordinating Center, Argentina

(Algar, Anderson, Austin, Baker, Blavius, Bloch, Boyle, Bradford, Carrall, Carr, Chuah, Curry, D'Arcy-Evans, Dobson, Doong, Ferguson, Finlayson, French, Gold, Habel, Hoy, Hudson, James, Jeganathan, Leung, Lowe, MacRae, Mallal, McMurchie, Medland, Mijch, Miller, Murray, Orth, Patching, Primrose, Quan, Rawlinson, Ree, Richardson, Rogers, Roney, Roth, Shaw, Soo, Sowden, Street, Street, Vale) Australia National Trial Coordinating Center, Australia

Publisher

Thomas Land Publishers Inc.

Emtree Heading

adult; African American; AIDS related complex; anamnesis; Argentina; article; Asia; Asian; Australia; bacterial pneumonia; candidiasis; CD4 lymphocyte count; clinical trial; cohort analysis; controlled clinical trial; controlled study; death; diabetes mellitus/dt [Drug Therapy]; diabetes mellitus/si [Side Effect]; disease course; disease duration; disease transmission; drug efficacy; drug safety; dyslipidemia/dt [Drug Therapy]; dyslipidemia/si [Side Effect]; ethnology; Europe; female; geographic distribution; heart infarction/si [Side Effect]; hepatitis B/dt [Drug Therapy]; hepatitis C/dt [Drug Therapy]; herpes simplex/dt [Drug Therapy]; herpes simplex/pc [Prevention]; herpes simplex/si [Side Effect]; heterosexuality; HIV associated lipodystrophy/si [Side Effect]; human; Human immunodeficiency virus; *Human immunodeficiency virus infection/dt [Drug Therapy]; hypertension/dt [Drug Therapy]; hypertension/si [Side Effect]; intravenous drug abuse; lipid storage; major clinical study; male; male homosexual; methodology; Negro; nonhuman; North America; open study; phase 3 clinical trial; priority journal; randomized controlled trial; sex difference; side effect/si [Side Effect]; South America; stroke/si [Side Effect]; superinfection; Thailand; time series analysis; treatment duration; treatment outcome; tuberculosis; virus load; antihypertensive agent/dt [Drug Therapy]; antilipemic agent/dt [Drug Therapy]; *antiretrovirus agent/ae [Adverse Drug Reaction]; *antiretrovirus agent/ct [Clinical Trial]; *antiretrovirus agent/cb [Drug Combination]; *antiretrovirus agent/cm [Drug Comparison]; *antiretrovirus agent/dt [Drug Therapy]; hepatitis B surface antigen/ec [Endogenous Compound]; hepatitis B vaccine/dt [Drug Therapy]; herpes vaccine/dt [Drug Therapy]; insulin/dt [Drug Therapy]; oral antidiabetic agent/dt [Drug Therapy]; oral antidiabetic agent/po [Oral Drug Administration]; proteinase inhibitor/ae [Adverse Drug Reaction]; proteinase inhibitor/ct [Clinical Trial]; proteinase inhibitor/cb [Drug Combination]; proteinase inhibitor/cm [Drug Comparison]; proteinase inhibitor/dt [Drug Therapy]; *recombinant interleukin 2/ad [Drug Administration]; *recombinant interleukin 2/cb [Drug Combination]; *recombinant interleukin 2/cm [Drug Comparison]; *recombinant interleukin 2/dt [Drug Therapy]; *recombinant interleukin 2/sc [Subcutaneous Drug Administration]; ritonavir/ae [Adverse Drug Reaction]; ritonavir/ct [Clinical Trial]; ritonavir/cb [Drug Combination]; ritonavir/cm [Drug Comparison]; ritonavir/dt [Drug Therapy]; RNA directed DNA polymerase inhibitor/ae [Adverse Drug Reaction]; RNA directed DNA polymerase inhibitor/ct [Clinical Trial]; RNA directed DNA polymerase inhibitor/cb [Drug Combination]; RNA directed DNA polymerase inhibitor/cm [Drug Comparison]; RNA directed DNA polymerase inhibitor/dt [Drug Therapy]; virus RNA/ec [Endogenous Compound].

Drug Trade Names and Manufacturers

proleukin

Link to the Ovid Full Text or citation:

Click here for full text options

112.

Current HIV treatment guidelines - An overview.

Rathbun R.C., Lockhart S.M., Stephens R.J.

Current Pharmaceutical Design. 12 (9) (pp 1045-1063), 2006. Date of Publication: March 2006.

AN: 2006186388

Use of highly active antiretroviral therapy has resulted in significant reductions in HIV-related morbidity and mortality. Current therapeutic approaches target cellular entry, viral transcription, and maturation of newly formed virus. Combination therapy is necessary to provide durable suppression of viral replication and immune reconstitution. A variety of consensus treatment guidelines addressing prophylaxis and treatment of HIV infection and opportunistic infections have been developed to serve as resources for clinicians. A summary of U.S. Department of Health and Human Services Guidelines for Use of Antiretroviral Agents in HIV-1-Infected Adults and Adolescents and International AIDS Society-USA Panel recommendations for Treatment of Adult HIV infection are presented. Considerations for selection of antiretroviral therapy in special populations (e.g., pregnancy, coinfection with tuberculosis, hepatitis B and C virus) are highlighted. U.S. Public Health Service guidelines for management of occupational exposure to HIV and initiation of postexposure prophylaxis are discussed as well as World Health Organization recommendations for use of antiretroviral therapy in resource-limited settings. The pathophysiology of HIV infection, viral load testing methods, viral dynamics, and classification of antiretrovirals are also briefly reviewed. © 2006 Bentham Science Publishers Ltd.

Institution

(Rathbun, Lockhart) College of Pharmacy, University of Oklahoma Health Sciences Center, Oklahoma City, OK, United States (Stephens) College of Pharmacy, University of Oklahoma Schusterman Center, Tulsa, OK, United States

Publisher

Bentham Science Publishers B.V.

Emtree Heading

abdominal pain/si [Side Effect]; bloating/si [Side Effect]; central nervous system disease/si [Side Effect]; clinical trial; diabetes insipidus/si [Side Effect]; diarrhea/si [Side Effect]; disease association; disorders of mitochondrial functions/si [Side Effect]; drug classification; drug dose regimen; drug efficacy; drug eruption/si [Side Effect]; drug half life; drug hypersensitivity/si [Side Effect]; drug mechanism; dyspnea/si [Side Effect]; Fanconi renotubular syndrome/si [Side Effect]; fatigue/si [Side Effect]; fatty liver/si [Side Effect]; fever/si [Side Effect]; gastrointestinal symptom/si [Side Effect]; Gilbert disease/si [Side Effect]; hepatitis B; hepatitis C; highly active antiretroviral therapy; human; *Human immunodeficiency virus infection/dt [Drug Therapy]; *Human immunodeficiency virus infection/et [Etiology]; hyperlipidemia/dt [Drug Therapy]; hyperlipidemia/si [Side Effect]; hypotension/si [Side Effect]; kidney failure/si [Side Effect]; kidney tubule necrosis/si [Side Effect]; lactic acidosis/si [Side Effect]; liver toxicity/si [Side Effect]; malaise/si [Side Effect]; metabolic disorder/dt [Drug Therapy]; metabolic disorder/si [Side Effect]; myopathy/si [Side Effect]; nausea/si [Side Effect]; nephrolithiasis/si [Side Effect]; nephrotoxicity/si [Side Effect]; occupational exposure; pancreatitis/si [Side Effect]; pathophysiology; patient selection; polyneuropathy/si [Side Effect]; practice guideline; priority journal; reverse transcription polymerase chain reaction; review; Stevens Johnson syndrome/si [Side Effect]; treatment failure; tuberculosis; virus load; virus replication; visual hallucination/si [Side Effect]; vivid dream/si [Side Effect]; vomiting/si [Side Effect]; abacavir/ae [Adverse Drug Reaction]; abacavir/ct [Clinical Trial]; abacavir/cb [Drug Combination]; abacavir/cm [Drug Comparison]; abacavir/dt [Drug Therapy]; abacavir/pk [Pharmacokinetics]; adefovir/ae [Adverse Drug Reaction]; adefovir/dt [Drug Therapy]; amprenavir phosphate/ct [Clinical Trial]; amprenavir phosphate/cb [Drug Combination]; amprenavir phosphate/cm [Drug Comparison]; amprenavir phosphate/dt [Drug Therapy]; *antiretrovirus agent/ae [Adverse Drug Reaction]; *antiretrovirus agent/ct [Clinical Trial]; *antiretrovirus agent/cb [Drug Combination]; *antiretrovirus agent/cm [Drug Comparison]; *antiretrovirus agent/do [Drug Dose]; *antiretrovirus agent/dt [Drug Therapy]; *antiretrovirus agent/pk [Pharmacokinetics]; *antiretrovirus agent/pd [Pharmacology]; atazanavir/ae [Adverse Drug Reaction]; atazanavir/ct [Clinical Trial]; atazanavir/cb [Drug Combination]; atazanavir/dt [Drug Therapy]; atazanavir/pk [Pharmacokinetics]; CD4 antigen/ec [Endogenous Compound]; cidofovir/ae [Adverse Drug Reaction]; cidofovir/dt [Drug Therapy]; delavirdine/dt [Drug Therapy]; didanosine/ct [Clinical Trial]; didanosine/cb [Drug Combination]; didanosine/cm [Drug Comparison]; didanosine/dt [Drug Therapy]; didanosine/pk [Pharmacokinetics]; efavirenz/ae [Adverse Drug Reaction]; efavirenz/ct [Clinical Trial]; efavirenz/cb [Drug Combination]; efavirenz/cm [Drug Comparison]; efavirenz/do [Drug Dose]; efavirenz/dt [Drug Therapy]; efavirenz/pk [Pharmacokinetics]; emtricitabine/ct [Clinical Trial]; emtricitabine/cb [Drug Combination]; emtricitabine/cm [Drug Comparison]; emtricitabine/dt [Drug Therapy]; enfuvirtide/dt [Drug Therapy]; enfuvirtide/pd [Pharmacology]; hydroxymethylglutaryl coenzyme A reductase inhibitor/dt [Drug Therapy]; indinavir/ae [Adverse Drug Reaction]; indinavir/dt [Drug Therapy]; indinavir/pk [Pharmacokinetics]; lamivudine/ct [Clinical Trial]; lamivudine/cb [Drug Combination]; lamivudine/cm [Drug Comparison]; lamivudine/dt [Drug Therapy]; lamivudine/pk [Pharmacokinetics]; lopinavir plus ritonavir/ct [Clinical Trial]; lopinavir plus ritonavir/cb [Drug Combination]; lopinavir plus ritonavir/dt [Drug Therapy]; nelfinavir/ct [Clinical Trial]; nelfinavir/cb [Drug Combination]; nelfinavir/dt [Drug Therapy]; nevirapine/ae [Adverse Drug Reaction]; nevirapine/do [Drug Dose]; nevirapine/dt [Drug Therapy]; nevirapine/pk [Pharmacokinetics]; proteinase inhibitor/ae [Adverse Drug Reaction]; proteinase inhibitor/ct [Clinical Trial]; proteinase inhibitor/cb [Drug Combination]; proteinase inhibitor/cm [Drug Comparison]; proteinase inhibitor/dt [Drug Therapy]; proteinase inhibitor/pk [Pharmacokinetics]; proteinase inhibitor/pd [Pharmacology]; ritonavir/ct [Clinical Trial]; ritonavir/cb [Drug Combination]; ritonavir/cm [Drug Comparison]; ritonavir/dt [Drug Therapy]; ritonavir/pk [Pharmacokinetics]; RNA directed DNA polymerase inhibitor/ae [Adverse Drug Reaction]; RNA directed DNA polymerase inhibitor/ct [Clinical Trial]; RNA directed DNA polymerase inhibitor/cb [Drug Combination]; RNA directed DNA polymerase inhibitor/cm [Drug Comparison]; RNA directed DNA polymerase inhibitor/do [Drug Dose]; RNA directed DNA polymerase inhibitor/dt [Drug Therapy]; RNA directed DNA polymerase inhibitor/pk [Pharmacokinetics]; RNA directed DNA polymerase inhibitor/pd [Pharmacology]; saquinavir/ct [Clinical Trial]; saquinavir/cb [Drug Combination]; saquinavir/cm [Drug Comparison]; saquinavir/dt [Drug Therapy]; saquinavir/pk [Pharmacokinetics]; stavudine/ct [Clinical Trial]; stavudine/cb [Drug Combination]; stavudine/dt [Drug Therapy]; tenofovir/ae [Adverse Drug Reaction]; tenofovir/ct [Clinical Trial]; tenofovir/cb [Drug Combination]; tenofovir/cm [Drug Comparison]; tenofovir/dt [Drug Therapy]; tenofovir disoproxil/dt [Drug Therapy]; tipranavir/dt [Drug Therapy]; unindexed drug; virus protein/ec [Endogenous Compound]; zalcitabine/dt [Drug Therapy]; zidovudine/ct [Clinical Trial]; zidovudine/cb [Drug Combination]; zidovudine/cm [Drug Comparison]; zidovudine/dt [Drug Therapy].

Drug Trade Names and Manufacturers

t 20

Link to the Ovid Full Text or citation:

Click here for full text options

113.

Clinical islet transplantation in type 1 diabetes mellitus: Results of Australia's first trial.

O'Connell P.J., Hawthorne W.J., Holmes-Walker D.J., Nankivell B.J., Gunton J.E., Patel A.T., Walters S.N., Pleass H.C.C., Allen R.D.M., Chapman J.R.

Medical Journal of Australia. 184 (5) (pp 221-225), 2006. Date of Publication: 06 Mar 2006.

AN: 2006134855

Objective: To determine whether pancreatic islet transplantation can control diabetes and prevent severe life-threatening hypoglycaemia. Design, setting and participants: A single-arm observation study of six patients undergoing islet transplantation. All patients had had type 1 diabetes mellitus for over 5 years and documented episodes of repeated severe hypoglycaemia. Islets were isolated from donor pancreases digested by Liberase. Separated islets were infused into the recipient's liver via the portal vein. Patients were immunosuppressed with daclizumab, sirolimus and tacrolimus. The transplants were performed at Westmead Hospital, NSW, between October 2002 and February 2005. Main outcome measures: Normal blood glucose control without administration of exogenous insulin; demonstration of islet function and abolition of hypoglycaemia. Results: Five of the patients received two islet infusions, and the sixth was withdrawn after one infusion following a portal vein thrombosis. Three patients became insulin-independent, with excellent glycaemic control. Two had islet function with circulating C-peptide, improved glycaemic control, reduced insulin requirement and abolition of severe hypoglycaemia. However, over a 2-year period, graft function deteriorated. Recipients who were initially insulin free remained C-peptide positive but required supplemental insulin. Complications included one postoperative bleed, two portal vein thromboses (which resolved completely), presumed recurrence of tuberculosis in one patient, and deterioration in renal function in one patient. Conclusions: Islet transplantation is effective at improving glycaemic control and hypoglycaemia unawareness in the short to medium term. However, problems with long-term safety of immunosuppression, islet-induced thrombosis and early detection of loss of islet function remain to be addressed.

Institution

(O'Connell, Hawthorne, Nankivell, Patel, Walters, Pleass, Allen, Chapman) National Pancreas Transplant Unit, University of Sydney, Westmead Hospital, Darcy Road, Westmead, NSW 2145, Australia (Holmes-Walker, Gunton) Department of Endocrinology, Westmead Hospital, Darcy Road, Westmead, NSW 2145, Australia

Publisher

Australasian Medical Publishing Co. Ltd

Emtree Heading

adult; ankle edema/si [Side Effect]; article; Australia; blood transfusion; cell isolation; *cell transplantation; clinical article; cytomegalovirus infection/co [Complication]; cytomegalovirus infection/dt [Drug Therapy]; cytomegalovirus infection/pc [Prevention]; diabetes control; disease duration; disease severity; drug safety; early diagnosis; follow up; glucose blood level; glycemic control; graft failure/co [Complication]; graft rejection/co [Complication]; graft rejection/dt [Drug Therapy]; graft rejection/pc [Prevention]; graft survival; herpes virus infection/co [Complication]; herpes virus infection/dt [Drug Therapy]; herpes virus infection/pc [Prevention]; human; hypercholesterolemia/si [Side Effect]; hypoglycemia/co [Complication]; hypoglycemia/pc [Prevention]; immunosuppressive treatment; *insulin dependent diabetes mellitus/dr [Drug Resistance]; *insulin dependent diabetes mellitus/dt [Drug Therapy]; *insulin dependent diabetes mellitus/su [Surgery]; kidney dysfunction/si [Side Effect]; liver toxicity/di [Diagnosis]; liver toxicity/si [Side Effect]; long term care; mouth ulcer/si [Side Effect]; observational study; *pancreas islet; pancreas islet cell function; pneumocystosis/co [Complication]; pneumocystosis/dt [Drug Therapy]; pneumocystosis/pc [Prevention]; portal vein; portal vein thrombosis/co [Complication]; portal vein thrombosis/dt [Drug Therapy]; portal vein thrombosis/pc [Prevention]; postoperative hemorrhage/si [Side Effect]; postoperative hemorrhage/su [Surgery]; postoperative hemorrhage/th [Therapy]; protein blood level; recurrent disease/dt [Drug Therapy]; recurrent disease/si [Side Effect]; treatment outcome; tuberculosis/dt [Drug Therapy]; tuberculosis/si [Side Effect]; C peptide/ec [Endogenous Compound]; cefazolin/dt [Drug Therapy]; cotrimoxazole/dt [Drug Therapy]; cotrimoxazole/po [Oral Drug Administration]; daclizumab/ae [Adverse Drug Reaction]; daclizumab/dt [Drug Therapy]; glucose/ec [Endogenous Compound]; heparin/ae [Adverse Drug Reaction]; heparin/do [Drug Dose]; heparin/dt [Drug Therapy]; heparin/iv [Intravenous Drug Administration]; heparin/sc [Subcutaneous Drug Administration]; imipenem/dt [Drug Therapy]; insulin/dt [Drug Therapy]; mycophenolic acid 2 morpholinoethyl ester/dt [Drug Therapy]; rapamycin/ae [Adverse Drug Reaction]; rapamycin/dt [Drug Therapy]; tsukubaenolide/ae [Adverse Drug Reaction]; tsukubaenolide/dt [Drug Therapy]; tuberculostatic agent/dt [Drug Therapy]; valaciclovir/dt [Drug Therapy]; valaciclovir/po [Oral Drug Administration].

Drug Trade Names and Manufacturers

Astellas, Hoffmann La Roche, Wyeth

Link to the Ovid Full Text or citation:

Click here for full text options

114.

Twenty-four-week follow-up examination of a leukocytapheresis therapy in rheumatoid arthritis.

Izumi Y., Tominaga M., Iwanaga N., Huang M., Tanaka F., Aratake K., Arima K., Tamai M., Kamachi M., Nakamura H., Ida H., Origuchi T., Kawakami A., Eguchi K.

Modern Rheumatology. 16 (1) (pp 20-23), 2006. Date of Publication: February 2006.

AN: 2006098910

Several clinical trials have demonstrated that leukocytapheresis (LCAP) is a safe and effective therapy for patients with refractory rheumatoid arthritis (RA). However, most of those reports were limited to short-term clinical observation. We have treated 11 RA patients with LCAP and observed them for 24 weeks after the final administration. The 11 cases included 3 diabetes patients, 2 patients with interstitial pneumonia, 1 patient with diffuse panbronchiolitis, and 1 patient with old pulmonary tuberculosis. Alternative therapies for all of these patients were considered difficult. Once-a-week LCAP administration was added for 5 weeks to the previous therapeutic regime in all patients, and the treatment efficacy was prospectively qualified. At 4 weeks after the final LCAP therapy, 8 of the 11 patients (73%) had achieved an American College of Rheumatology (ACR) 20% response, and 3 of the 11 (27%) had achieved both ACR 50% and ACR 70% responses. Although the efficacy decreased after the observation periods, an ACR 20% response was maintained in 5 patients (45%) at 24 weeks. Although only a limited number of patients were examined in this study, the results suggested that LCAP therapy will be beneficial to RA patients, including patients who cannot be treated with tumor necrosis factor inhibitors or conventional disease-modifying antirheumatic drugs. © Japan College of Rheumatology 2006.

Institution

(Izumi, Iwanaga, Huang, Tanaka, Aratake, Arima, Tamai, Kamachi, Nakamura, Ida, Kawakami, Eguchi) First Department of Internal Medicine, Graduate School of Biochemical Sciences, Nagasaki University, 1-7-1 Sakamoto, Nagasaki 852-8501, Japan (Tominaga) Department of Nephrology, Nijigaoka Hospital, Nagasaki, Japan

(Origuchi) Nagasaki University, School of Health Sciences, 1-7-1 Sakamoto, Nagasaki 852-8501, Japan

Publisher

Springer-Verlag Tokyo

Emtree Heading

adult; aged; article; bronchiolitis/co [Complication]; bronchiolitis/dt [Drug Therapy]; clinical article; controlled study; *cytapheresis; diabetes mellitus/co [Complication]; disease activity; disease association; disease classification; disease severity; drug dose regimen; drug hypersensitivity/si [Side Effect]; female; follow up; human; interstitial pneumonia/co [Complication]; Japan; *leukapheresis; lung tuberculosis/co [Complication]; male; outcomes research; priority journal; Pseudomonas aeruginosa; *rheumatoid arthritis/di [Diagnosis]; *rheumatoid arthritis/dr [Drug Resistance]; *rheumatoid arthritis/dt [Drug Therapy]; *rheumatoid arthritis/th [Therapy]; scoring system; statistical analysis; statistical significance; antibiotic agent/dt [Drug Therapy]; bucillamine/dt [Drug Therapy]; C reactive protein/ec [Endogenous Compound]; cyclosporin/dt [Drug Therapy]; disease modifying antirheumatic drug/ae [Adverse Drug Reaction]; disease modifying antirheumatic drug/dt [Drug Therapy]; methotrexate/do [Drug Dose]; methotrexate/dt [Drug Therapy]; mizoribine/dt [Drug Therapy]; nonsteroid antiinflammatory agent/dt [Drug Therapy]; penicillamine/dt [Drug Therapy]; prednisolone/do [Drug Dose]; prednisolone/dt [Drug Therapy]; salazosulfapyridine/dt [Drug Therapy].

Link to the Ovid Full Text or citation:

Click here for full text options

115.

Sinogenic intracranial empyema in children.

Adame N., Hedlund G., Byington C.L.

Pediatrics. 116 (3) (pp e461-e467), 2005. Date of Publication: September 2005.

AN: 2006431198

Background. Sinogenic intracranial empyema (SIE) is an uncommon complication of sinusitis that can lead to devastating neurologic sequelae. Early recognition of the clinical findings is critical so that proper management can be instituted. Objective. To describe the symptoms, signs, and laboratory and imaging findings from one of the largest pediatric SIE case series reported. Methods. Descriptive study of a retrospective cohort of all children admitted to Primary Children's Medical Center with SIE between June 2000 and February 2004. Children were identified by a computerized search of Primary Children's Medical Center medical records using the terms "sinusitis" and "brain/subdural/epidural empyema." Erythrocyte sedimentation rate (ESR) and C-reactive protein (CRP) values of children with SIE were compared with a group of children with uncomplicated sinusitis cared for in the same health care system as outpatients. The medical records of the uncomplicated sinusitis group were not reviewed for any clinical or radiographic data other than CRP and ESR values. Results. Twelve children with SIE were identified. The median age of children with SIE was 11.5 years. Symptoms were usually present 10 days (median) before diagnosis and included headache (10), fever (11), nausea/vomiting (7), mental-status changes (5), and seizures (3). Physical findings included abnormal neurologic examination (9), Pott's puffy tumor (4), and orbital cellulitis (3). Using the Intermountain Health Care system's computerized database, 142 children with uncomplicated sinusitis treated as outpatients were identified. Children with SIE had markedly higher CRP levels (median: 10.05 mg/dL) and ESRs (median: 87 mm/hour) than those with uncomplicated sinusitis (median CRP: 0.7 mg/dL; median ESR: 6 mm/hour). Four children had hyperglycemia. Four children had a lumbar puncture at presentation, and the findings were normal for all of them. Craniofacial imaging included computed tomography (CT) and magnetic resonance imaging (MRI). SIE was not detected in 4 patients who had nonenhanced CT. Axial imaging alone was unable to demonstrate SIE in 1 child with sphenoid and ethmoid sinusitis, and coronal images were needed to demonstrate its presence and extent. The initial facial/orbital imaging studies in 2 patients with physical signs of complicated sinusitis (orbital cellulitis and Pott's puffy tumor) were not adequate to detect SIE. Using contrast-enhanced head CT or MRI, SIE was diagnosed in all 12 children. Conclusions. Children with sinusitis and any neurologic finding, signs of complicated sinusitis such as Pott's puffy tumor or orbital cellulitis, or persistent headache, fever, or nausea and vomiting after antibiotic therapy should have additional evaluation for SIE. Children with hyperglycemia or diabetes may be at increased risk for SIE. The ESR and CRP levels are markedly elevated in children with SIE and may be useful screening tools. MRI with gadolinium is the preferred method to diagnose SIE. If MRI is unavailable, a contrast-enhanced head CT with axial and coronal planes should be obtained. Nonenhanced CT alone lacks sensitivity, and a normal study may be falsely reassuring. Copyright © 2005 by the American Academy of Pediatrics.

Institution

(Adame, Byington) Department of Pediatrics, Primary Children's Medical Center, University of Utah, Salt Lake City, UT, United States (Hedlund) Department of Medical Imaging, Primary Children's Medical Center, Salt Lake City, UT, United States

(Adame) Department of Pediatrics, Division of Critical Care, 7703 Floyd Curl Dr, San Antonio, TX 78229-3900, United States

Publisher

American Academy of Pediatrics

Emtree Heading

adult; article; clinical feature; clinical trial; controlled clinical trial; controlled study; diagnostic imaging; *empyema/co [Complication]; *empyema/di [Diagnosis]; erythrocyte sedimentation rate; female; glucose blood level; health care system; human; hyperglycemia; male; medical record; neurologic examination; orbit cellulitis/co [Complication]; preschool child; priority journal; school child; screening; sinusitis/dm [Disease Management]; symptomatology; temperature measurement; treatment outcome; tuberculous spondylitis/co [Complication]; C reactive protein/ec [Endogenous Compound]; glucose/ec [Endogenous Compound]; glycosylated hemoglobin/ec [Endogenous Compound].

Link to the Ovid Full Text or citation:

Click here for full text options

116.

Comparison of sirolimus with low-dose tacrolimus versus sirolimus-based calcineurin inhibitor-free regimen in live donor renal transplantation.

Hamdy A.F., El-Agroudy A.E., Bakr M.A., Mostafa A., El-Baz M., El-Shahawy E.-M., Ghoneim M.A.

American Journal of Transplantation. 5 (10) (pp 2531-2538), 2005. Date of Publication: October 2005.

AN: 2006295498

Between May 2001 and January 2003, 132 live donor renal allotransplant recipients were included in a prospective, randomized controlled trial where they were divided into two groups. All patients received steroids and basiliximab induction therapy. For maintenance immunosuppression, tacrolimus and sirolimus were used in group A. In group B, mycophenolate mofetil (MMF) and sirolimus were utilized. Patients were followed up for a minimum of 24 months. One-year patient and graft survival rates were not significantly different between group A (96.9%, 92.3%) and group B (100%, 98.4%), respectively. However, the incidence of biopsy-proven acute rejection was less in group B but the difference was not statistically significant (13.5% vs. 18.5% in group A). Statistically significant better renal function was encountered among group B patients at two years post-transplantation as measured by serum creatinine (1.25 vs. 1.43 mg/dl; P = 0.017) and calculated glomerular filtration rate (GFR) (94.9 vs. 79.6 ml/min; P = 0.005). One year protocol biopsies showed insignificant differences relative to chronic allograft damage index (CADI) between either group (Group A: 2.41 vs. Group B: 2.69; P = 0.436). Conclusion: Similar outcome was noted among patients in whom calcineurin inhibitors were not included in their immunosuppressive regimen. The long term impact of this observation on graft survival and function needs longer follow up. Copyright © Blackwell Munksgaard 2005.

Institution

(Hamdy, El-Agroudy, Bakr, Mostafa, El-Baz, El-Shahawy, Ghoneim) Urology and Nephrology Center, Mansoura University, Mansoura, Egypt

Publisher

Blackwell Publishing Ltd

Emtree Heading

abdominal disease/si [Side Effect]; acute graft rejection/co [Complication]; acute graft rejection/dt [Drug Therapy]; acute graft rejection/pc [Prevention]; adult; article; avascular necrosis/si [Side Effect]; chronic graft rejection/co [Complication]; chronic graft rejection/dt [Drug Therapy]; chronic graft rejection/pc [Prevention]; creatinine blood level; diabetes mellitus/si [Side Effect]; diarrhea/si [Side Effect]; female; follow up; gastrointestinal hemorrhage/si [Side Effect]; glomerulus filtration rate; graft survival; herpes zoster/si [Side Effect]; human; human tissue; hyperlipidemia/si [Side Effect]; immunosuppressive treatment; incidence; kidney biopsy; kidney function; *kidney transplantation; leukopenia/si [Side Effect]; living donor; lung tuberculosis/si [Side Effect]; maintenance therapy; major clinical study; male; mycosis/si [Side Effect]; nephrotoxicity/si [Side Effect]; priority journal; prospective study; proteinuria/si [Side Effect]; recipient; side effect/si [Side Effect]; survival rate; thrombocytopenia/si [Side Effect]; treatment outcome; urinary tract infection/si [Side Effect]; basiliximab/cb [Drug Combination]; basiliximab/dt [Drug Therapy]; basiliximab/iv [Intravenous Drug Administration]; *calcineurin inhibitor; creatinine/ec [Endogenous Compound]; liver enzyme/ec [Endogenous Compound]; methylprednisolone/dt [Drug Therapy]; methylprednisolone/iv [Intravenous Drug Administration]; mycophenolic acid 2 morpholinoethyl ester/cb [Drug Combination]; mycophenolic acid 2 morpholinoethyl ester/dt [Drug Therapy]; mycophenolic acid 2 morpholinoethyl ester/po [Oral Drug Administration]; prednisolone/dt [Drug Therapy]; prednisolone/po [Oral Drug Administration]; *rapamycin/ae [Adverse Drug Reaction]; *rapamycin/cb [Drug Combination]; *rapamycin/cm [Drug Comparison]; *rapamycin/dt [Drug Therapy]; *rapamycin/po [Oral Drug Administration]; steroid/cb [Drug Combination]; steroid/dt [Drug Therapy]; *tacrolimus/ae [Adverse Drug Reaction]; *tacrolimus/cm [Drug Comparison]; *tacrolimus/do [Drug Dose]; *tacrolimus/dt [Drug Therapy].

Drug Trade Names and Manufacturers

Fujisawa [Jordan], Hoffmann La Roche [Italy], Novartis, Wyeth Ayerst [United States], cellcept: Hoffmann La Roche [Italy], fk 506, prograf: Fujisawa [Jordan], rapamune: Wyeth Ayerst [United States], simulect: Novartis

Link to the Ovid Full Text or citation:

Click here for full text options

117.

ANCA-associated renal vasculitis at the end of the twentieth century - A disease of older patients.

Harper L., Savage C.O.

Rheumatology. 44 (4) (pp 495-501), 2005. Date of Publication: April 2005.

AN: 2005480793

Objective. Antineutrophil cytoplasmic antibody (ANCA)-associated vasculitides are increasingly recognized in older patients. However, it is unknown whether disease presentation and response to treatment differs from younger patients. We aimed to examine the presentation, response to treatment and outcome of patients over 65 yr of age compared with a younger cohort. Methods. This retrospective, single centre, sequential cohort study reports presenting features and outcome of 233 consecutive new patients with ANCA-associated vasculitis between 1990 and 2000. Results. The median age of all patients was 65 yr (range 16-90 yr). Older patients (>65 yr) presented with more severe renal involvement at presentation (P<0.001). Older patients were as likely to respond to treatment or undergo relapse as the younger patients. Older patients receiving immunosuppression had an increased risk of infection (P = 0.0027). Survival was worse in the older group (P = 0.016) and death occurred early. Mortality was associated with poor renal function (creatinine >400 mumol/l), infection and low serum albumin. Leucopenia was associated with severe renal impairment (P = 0.0048) and increased risk of infection (P = 0.0006). Multivariate analysis determined that serum creatinine >400 mumol/l and age were independent risk factors for poor prognosis. Conclusion. ANCA-associated vasculitis occurs frequently in older patients and physicians should maintain a high index of suspicion. Older patients have a poorer prognosis due to more severe renal involvement and increased sensitivity to adverse effects of treatment. This study highlights the importance of careful dosing of cyclophosphamide: In those aged over 65 yr a 25% dose reduction is safe and reduces the risk of leucopenia. This study further highlights the importance of renal function on prognosis and the need for less toxic treatment regimens. © British Society for Rheumatology 2005; all rights reserved.

Institution

(Harper, Savage) Division of Immunology and Infection, The Medical School, University of Birmingham, Edgbaston, Birmingham B15 2TT, United Kingdom

Publisher

Oxford University Press

Emtree Heading

adolescent; adult; age distribution; aged; *ANCA associated vasculitis/di [Diagnosis]; *ANCA associated vasculitis/dt [Drug Therapy]; article; bladder cancer/si [Side Effect]; cerebrovascular disease/si [Side Effect]; clinical feature; clinical trial; cohort analysis; controlled clinical trial; controlled study; creatinine blood level; death; deep vein thrombosis/si [Side Effect]; diabetes mellitus/si [Side Effect]; disease association; disease severity; drug dose reduction; drug dose regimen; drug fatality/si [Side Effect]; female; fracture/si [Side Effect]; health care facility; heart infarction/si [Side Effect]; human; immunosuppressive treatment; infection/dt [Drug Therapy]; infection/pc [Prevention]; infection/si [Side Effect]; *kidney disease/di [Diagnosis]; *kidney disease/dt [Drug Therapy]; kidney dysfunction/si [Side Effect]; leukopenia/si [Side Effect]; lower respiratory tract infection/si [Side Effect]; lung embolism/si [Side Effect]; major clinical study; male; malignant neoplastic disease/si [Side Effect]; mortality; multivariate analysis; neutropenia/si [Side Effect]; physician; pneumonia/si [Side Effect]; priority journal; prognosis; relapse; retrospective study; risk assessment; sensitivity analysis; sepsis/si [Side Effect]; skin cancer/si [Side Effect]; survival rate; treatment outcome; tuberculosis/si [Side Effect]; azathioprine/dt [Drug Therapy]; corticosteroid/ae [Adverse Drug Reaction]; corticosteroid/dt [Drug Therapy]; cotrimoxazole/dt [Drug Therapy]; creatinine/ec [Endogenous Compound]; cyclophosphamide/ae [Adverse Drug Reaction]; cyclophosphamide/ct [Clinical Trial]; cyclophosphamide/do [Drug Dose]; cyclophosphamide/dt [Drug Therapy]; immunosuppressive agent/ae [Adverse Drug Reaction]; immunosuppressive agent/ct [Clinical Trial]; immunosuppressive agent/do [Drug Dose]; immunosuppressive agent/dt [Drug Therapy]; methylprednisolone/dt [Drug Therapy]; prednisolone/dt [Drug Therapy]; serum albumin/ec [Endogenous Compound].

Drug Trade Names and Manufacturers

septrin

Link to the Ovid Full Text or citation:

Click here for full text options

118.

Treating the patient in pain.

Katz W.A., Rothenberg R.

Journal of Clinical Rheumatology. 11 (2 SUPPL.) (pp S16-S28), 2005. Date of Publication: April 2005.

AN: 2005199081

Physicians may choose from a variety of pharmacologic and nonpharmacologic options to treat patients with painful rheumatic diseases. Osteoarthritis (OA) is the most common type of arthritis requiring pain management. New disease-modifying antirheumatic drugs and biologic response modifiers can improve disease states in patients with rheumatoid arthritis (RA). After the inflammatory component of RA is minimized with such agents, treatment goals shift to those similar to secondary OA and other degenerative joint diseases. Relief of pain and improvement in functional status are essential components of effective therapy. A pure analgesic such as acetaminophen and nonsteroidal antiinflammatory drugs, including the cyclooxygenase-2-selective inhibitors for those at risk for gastrointestinal side effects, may be used at the lowest effective doses. Combination therapy for acetaminophen and an opioid may maximize pain relief and provide greater speed and duration of action than the separate components. Use of the atypical opioid tramadol with acetaminophen often results in an improved side-effect profile compared with stronger opioids, with similar levels of pain relief. Adjunctive therapy with agents such as topical analgesics, intraarticular hyaluron, tricyclic antidepressants, anticonvulsants, muscle relaxants, and anxiolytics may also be helpful. Nonpharmacologic therapies such as exercise, physical therapy, and psychologic counseling may also diminish pain and improve outcome in patients with rheumatic diseases. One may also consider yoga, acupuncture, biofeedback, massage, relaxation techniques, and other alternative therapies. Copyright © 2005 by Lippincott Williams & Wilkins.

Institution

(Katz) Department of Medicine, Univ. of PA School of Medicine, Philadelphia, PA, United States (Rothenberg) Department of Medicine, George Washington Univ. Med. Center, Washington, DC, United States

Publisher

Lippincott Williams and Wilkins

Emtree Heading

acupuncture; adjuvant therapy; alopecia/si [Side Effect]; alternative medicine; analgesia; *arthralgia/co [Complication]; *arthralgia/dm [Disease Management]; *arthralgia/dt [Drug Therapy]; *arthralgia/th [Therapy]; article; bleeding/si [Side Effect]; bone marrow suppression/si [Side Effect]; cardiovascular disease/si [Side Effect]; clinical practice; clinical trial; comorbidity; comparative study; constipation/si [Side Effect]; corticosteroid therapy; cryotherapy; Cushingoid syndrome/si [Side Effect]; diabetes mellitus/si [Side Effect]; diagnostic test; diet; digestive system ulcer/si [Side Effect]; disease association; disease exacerbation/si [Side Effect]; disease severity; dizziness/si [Side Effect]; doctor patient relation; drowsiness/si [Side Effect]; drug contraindication; drug cross reactivity; drug dosage form; drug dose regimen; drug effect; drug efficacy; drug hypersensitivity/si [Side Effect]; drug indication; drug potency; drug potentiation; edema/si [Side Effect]; exercise; feedback system; fluid retention; fracture healing/si [Side Effect]; functional assessment; gastrointestinal hemorrhage/si [Side Effect]; gastrointestinal symptom/si [Side Effect]; habituation; human; hypertension/si [Side Effect]; hyperthermic therapy; infection risk; kidney disease/si [Side Effect]; liver dysfunction/si [Side Effect]; liver level; liver toxicity/si [Side Effect]; low drug dose; massage; nausea/si [Side Effect]; occupational therapy; osteoarthritis/dt [Drug Therapy]; osteoporosis/si [Side Effect]; *pain/co [Complication]; *pain/dm [Disease Management]; *pain/dt [Drug Therapy]; *pain/th [Therapy]; pain assessment; pathophysiology; patient care; patient compliance; patient counseling; patient education; physician attitude; physiotherapy; polypharmacy; postoperative pain/co [Complication]; postoperative pain/dt [Drug Therapy]; priority journal; psychotherapy; relaxation training; respiration depression/si [Side Effect]; *rheumatic disease/dt [Drug Therapy]; rheumatoid arthritis/dt [Drug Therapy]; risk assessment; sedation; seizure; sepsis/si [Side Effect]; side effect/si [Side Effect]; social support; somnolence/si [Side Effect]; tablet formulation; tachyphylaxis; treatment outcome; treatment planning; tuberculosis/si [Side Effect]; vomiting/si [Side Effect]; weight gain; xerostomia/si [Side Effect]; yoga; *analgesic agent/ae [Adverse Drug Reaction]; *analgesic agent/ct [Clinical Trial]; *analgesic agent/ad [Drug Administration]; *analgesic agent/cb [Drug Combination]; *analgesic agent/cm [Drug Comparison]; *analgesic agent/do [Drug Dose]; *analgesic agent/it [Drug Interaction]; *analgesic agent/dt [Drug Therapy]; *analgesic agent/po [Oral Drug Administration]; *analgesic agent/pa [Parenteral Drug Administration]; *analgesic agent/pr [Pharmaceutics]; *analgesic agent/pk [Pharmacokinetics]; *analgesic agent/pd [Pharmacology]; *analgesic agent/tp [Topical Drug Administration]; anticonvulsive agent/dt [Drug Therapy]; antiinflammatory agent/ae [Adverse Drug Reaction]; antiinflammatory agent/dt [Drug Therapy]; antiinflammatory agent/pd [Pharmacology]; antipyretic agent; antipyretic analgesic agent/ae [Adverse Drug Reaction]; antipyretic analgesic agent/dt [Drug Therapy]; antipyretic analgesic agent/pd [Pharmacology]; anxiolytic agent/dt [Drug Therapy]; celecoxib/ae [Adverse Drug Reaction]; celecoxib/dt [Drug Therapy]; celecoxib/pd [Pharmacology]; corticosteroid/cb [Drug Combination]; corticosteroid/dt [Drug Therapy]; corticosteroid/pd [Pharmacology]; cyclooxygenase 2 inhibitor/ae [Adverse Drug Reaction]; cyclooxygenase 2 inhibitor/cb [Drug Combination]; cyclooxygenase 2 inhibitor/cm [Drug Comparison]; cyclooxygenase 2 inhibitor/do [Drug Dose]; cyclooxygenase 2 inhibitor/dt [Drug Therapy]; cyclooxygenase 2 inhibitor/pk [Pharmacokinetics]; cyclooxygenase 2 inhibitor/pd [Pharmacology]; diclofenac/ae [Adverse Drug Reaction]; diclofenac/dt [Drug Therapy]; diclofenac/pd [Pharmacology]; disease modifying antirheumatic drug/ae [Adverse Drug Reaction]; disease modifying antirheumatic drug/cb [Drug Combination]; disease modifying antirheumatic drug/cm [Drug Comparison]; disease modifying antirheumatic drug/do [Drug Dose]; disease modifying antirheumatic drug/dt [Drug Therapy]; disease modifying antirheumatic drug/po [Oral Drug Administration]; disease modifying antirheumatic drug/pd [Pharmacology]; hyaluronic acid/ad [Drug Administration]; hyaluronic acid/cb [Drug Combination]; hyaluronic acid/cm [Drug Comparison]; hyaluronic acid/dt [Drug Therapy]; hyaluronic acid/pd [Pharmacology]; hydroxychloroquine/ae [Adverse Drug Reaction]; hydroxychloroquine/cb [Drug Combination]; hydroxychloroquine/dt [Drug Therapy]; hydroxychloroquine/pd [Pharmacology]; hypnotic agent/dt [Drug Therapy]; ibuprofen/ae [Adverse Drug Reaction]; ibuprofen/dt [Drug Therapy]; ibuprofen/pd [Pharmacology]; leflunomide/ae [Adverse Drug Reaction]; leflunomide/cb [Drug Combination]; leflunomide/dt [Drug Therapy]; leflunomide/pd [Pharmacology]; long acting drug/dt [Drug Therapy]; methotrexate/ae [Adverse Drug Reaction]; methotrexate/cb [Drug Combination]; methotrexate/dt [Drug Therapy]; methotrexate/pd [Pharmacology]; muscle relaxant agent/dt [Drug Therapy]; naproxen/ae [Adverse Drug Reaction]; naproxen/dt [Drug Therapy]; naproxen/pd [Pharmacology]; nonsteroid antiinflammatory agent/ae [Adverse Drug Reaction]; nonsteroid antiinflammatory agent/cb [Drug Combination]; nonsteroid antiinflammatory agent/cm [Drug Comparison]; nonsteroid antiinflammatory agent/do [Drug Dose]; nonsteroid antiinflammatory agent/dt [Drug Therapy]; nonsteroid antiinflammatory agent/po [Oral Drug Administration]; nonsteroid antiinflammatory agent/pk [Pharmacokinetics]; nonsteroid antiinflammatory agent/pd [Pharmacology]; opiate/ae [Adverse Drug Reaction]; opiate/cb [Drug Combination]; opiate/cm [Drug Comparison]; opiate/do [Drug Dose]; opiate/dt [Drug Therapy]; opiate/pd [Pharmacology]; opiate/tp [Topical Drug Administration]; paracetamol/ae [Adverse Drug Reaction]; paracetamol/ct [Clinical Trial]; paracetamol/cb [Drug Combination]; paracetamol/cm [Drug Comparison]; paracetamol/do [Drug Dose]; paracetamol/dt [Drug Therapy]; paracetamol/pr [Pharmaceutics]; paracetamol/pk [Pharmacokinetics]; paracetamol/pd [Pharmacology]; recombinant interleukin 1 receptor blocking agent/ae [Adverse Drug Reaction]; recombinant interleukin 1 receptor blocking agent/cb [Drug Combination]; recombinant interleukin 1 receptor blocking agent/dt [Drug Therapy]; recombinant interleukin 1 receptor blocking agent/pd [Pharmacology]; salazosulfapyridine/ae [Adverse Drug Reaction]; salazosulfapyridine/cb [Drug Combination]; salazosulfapyridine/dt [Drug Therapy]; salazosulfapyridine/pd [Pharmacology]; short acting drug/dt [Drug Therapy]; tramadol/ae [Adverse Drug Reaction]; tramadol/ct [Clinical Trial]; tramadol/cb [Drug Combination]; tramadol/cm [Drug Comparison]; tramadol/do [Drug Dose]; tramadol/dt [Drug Therapy]; tramadol/pr [Pharmaceutics]; tramadol/pd [Pharmacology]; tramadol/tp [Topical Drug Administration]; tricyclic antidepressant agent/dt [Drug Therapy]; tumor necrosis factor antibody/ae [Adverse Drug Reaction]; tumor necrosis factor antibody/cb [Drug Combination]; tumor necrosis factor antibody/dt [Drug Therapy]; tumor necrosis factor antibody/pd [Pharmacology]; unindexed drug.

Link to the Ovid Full Text or citation:

Click here for full text options

119.

Benefit/risk of therapies for rheumatoid arthritis: Underestimation of the "side effects" or risks of RA leads to underestimation of the benefit/risk of therapies.

Pincus T., Kavanaugh A., Sokka T.

Clinical and Experimental Rheumatology. 22 (5 SUPPL. 35) (pp S2-S11), 2004. Date of Publication: 2004.

AN: 2004428782

Most physicians are familiar with the side effects or risks of drugs used to treat rheumatoid arthritis (RA), but relatively less familiar with the "side effects" or risks associated with RA itself. RA is not thought to have the same potential severity as a cardiovascular or neoplastic disease by most physicians, the public, or even some rheumatologists, although relative rates of predicted mortality in some patients with RA are in the range of some people with coronary artery disease or Hodgkin's disease. Many reasons may be identified to explain why the risks of RA have been underestimated: RA does not lead to acute life-threatening situations; population-based data have suggested that most people who meet criteria for RA have a mild or self-limited process; acute attributed causes of death in people with RA are superficially similar to those in the general population; clinical trials have suggested many therapies that are efficacious over a period of 3-12 months; few long-term longitudinal studies were performed prior to the 1980s; medical recommendations made during the 1950s-1980s suggested that simple therapies were adequate for most patients; and quantitative information concerning patient status is generally not included in standard rheumatology care. As more information has emerged concerning severe long-term outcomes in the "natural history" of RA (as treated prior to the 1990s, new strategies of aggressive intervention have been developed. Furthermore, basic research has led to new therapies. It appears that the benefit/risk ratio of therapies for RA has increased substantially over the last two decades, and the outlook for patients with RA is much better at this time than in previous years. © Copyright Clinical and Experimental Rheumatology 2004.

Institution

(Pincus, Sokka) Division of Rheumatology/Immunology, Department of Medicine, Vanderbilt University Medical Center, 203 Oxford House, Nashville, TN 37232-4500, United States (Kavanaugh) The Center for Innovative Therapy, Div. Rheumatol. Allergy/Immunology, Univ. California San Diego Sch. Med., La Jolla, CA, United States

(Sokka) Jyvaskyla Central Hospital, Jyvaskyla, Finland

Publisher

Clinical and Experimental Rheumatology S.A.S.

Emtree Heading

bladder disease/si [Side Effect]; cardiovascular disease; cause of death; central nervous system disease/si [Side Effect]; clinical trial; diabetes mellitus/si [Side Effect]; disease course; disease severity; drug efficacy; drug research; drug use; gastrointestinal disease/si [Side Effect]; hematologic disease/si [Side Effect]; Hodgkin disease; human; hypertension/si [Side Effect]; ischemic heart disease; kidney disease/si [Side Effect]; liver disease/si [Side Effect]; longitudinal study; lymphoma/si [Side Effect]; mortality; neoplasm; osteopenia/si [Side Effect]; population research; prediction; priority journal; quantitative analysis; retinopathy/si [Side Effect]; review; *rheumatoid arthritis/dt [Drug Therapy]; risk assessment; *risk benefit analysis; teratogenicity; treatment outcome; tuberculosis/si [Side Effect]; adalimumab/ae [Adverse Drug Reaction]; adalimumab/cm [Drug Comparison]; adalimumab/dt [Drug Therapy]; *antirheumatic agent/ae [Adverse Drug Reaction]; *antirheumatic agent/ct [Clinical Trial]; *antirheumatic agent/cb [Drug Combination]; *antirheumatic agent/cm [Drug Comparison]; *antirheumatic agent/dt [Drug Therapy]; *antirheumatic agent/to [Drug Toxicity]; azathioprine/ae [Adverse Drug Reaction]; azathioprine/dt [Drug Therapy]; corticosteroid/ae [Adverse Drug Reaction]; corticosteroid/ct [Clinical Trial]; corticosteroid/cb [Drug Combination]; corticosteroid/cm [Drug Comparison]; corticosteroid/dt [Drug Therapy]; cyclophosphamide/ae [Adverse Drug Reaction]; cyclophosphamide/dt [Drug Therapy]; cyclosporin A/ae [Adverse Drug Reaction]; cyclosporin A/dt [Drug Therapy]; etanercept/ae [Adverse Drug Reaction]; etanercept/cm [Drug Comparison]; etanercept/dt [Drug Therapy]; gold salt/ae [Adverse Drug Reaction]; gold salt/cm [Drug Comparison]; gold salt/dt [Drug Therapy]; hydroxychloroquine/ae [Adverse Drug Reaction]; hydroxychloroquine/ct [Clinical Trial]; hydroxychloroquine/cb [Drug Combination]; hydroxychloroquine/cm [Drug Comparison]; hydroxychloroquine/dt [Drug Therapy]; infliximab/ae [Adverse Drug Reaction]; infliximab/cm [Drug Comparison]; infliximab/dt [Drug Therapy]; leflunomide/ae [Adverse Drug Reaction]; leflunomide/cm [Drug Comparison]; leflunomide/dt [Drug Therapy]; leflunomide/to [Drug Toxicity]; methotrexate/ae [Adverse Drug Reaction]; methotrexate/ct [Clinical Trial]; methotrexate/cb [Drug Combination]; methotrexate/cm [Drug Comparison]; methotrexate/dt [Drug Therapy]; nonsteroid antiinflammatory agent/ae [Adverse Drug Reaction]; nonsteroid antiinflammatory agent/dt [Drug Therapy]; penicillamine/ae [Adverse Drug Reaction]; penicillamine/cm [Drug Comparison]; penicillamine/dt [Drug Therapy]; prednisolone/ct [Clinical Trial]; prednisolone/cb [Drug Combination]; prednisolone/cm [Drug Comparison]; prednisolone/dt [Drug Therapy]; prednisone/ct [Clinical Trial]; prednisone/cb [Drug Combination]; prednisone/cm [Drug Comparison]; prednisone/dt [Drug Therapy]; recombinant interleukin 1 receptor blocking agent/ae [Adverse Drug Reaction]; recombinant interleukin 1 receptor blocking agent/cm [Drug Comparison]; recombinant interleukin 1 receptor blocking agent/dt [Drug Therapy]; salazosulfapyridine/ct [Clinical Trial]; salazosulfapyridine/cb [Drug Combination]; salazosulfapyridine/cm [Drug Comparison]; salazosulfapyridine/dt [Drug Therapy].

Link to the Ovid Full Text or citation:

Click here for full text options

120.

Randomised trial of simulect versus placebo for control of acute rejection in renal allograft recipients.

Bingyi S., Yeyong Q., Ming C., Chunbai M., Wenqiang Z.

Transplantation Proceedings. 35 (1) (pp 192-194), 2003. Date of Publication: February 2003.

AN: 2003074700

Institution

(Bingyi, Yeyong, Ming, Chunbai, Wenqiang) Department of Urology, Hospital of PLA, Beijing, China (Bingyi) Department of Urology, 309 Hospital of PLA, Beijing 100091, China

Publisher

Elsevier Inc.

Emtree Heading

adult; cadaver donor; chill/si [Side Effect]; clinical article; clinical trial; conference paper; controlled clinical trial; controlled study; coughing/si [Side Effect]; cytokine release; diabetes mellitus/si [Side Effect]; drug effect; drug tolerability; female; fever/si [Side Effect]; *graft rejection/dt [Drug Therapy]; *graft rejection/pc [Prevention]; headache/si [Side Effect]; human; human tissue; hypertension/si [Side Effect]; infection/si [Side Effect]; kidney; *kidney allograft; kidney function; liver function; lung edema/si [Side Effect]; lymphoproliferative disease/si [Side Effect]; male; priority journal; randomized controlled trial; side effect/si [Side Effect]; treatment outcome; tuberculosis/dt [Drug Therapy]; azathioprine/ae [Adverse Drug Reaction]; azathioprine/cb [Drug Combination]; azathioprine/do [Drug Dose]; azathioprine/dt [Drug Therapy]; azathioprine/iv [Intravenous Drug Administration]; azathioprine/pd [Pharmacology]; *basiliximab/ae [Adverse Drug Reaction]; *basiliximab/ct [Clinical Trial]; *basiliximab/dt [Drug Therapy]; *basiliximab/iv [Intravenous Drug Administration]; *basiliximab/pd [Pharmacology]; cyclosporin A/ae [Adverse Drug Reaction]; cyclosporin A/cb [Drug Combination]; cyclosporin A/do [Drug Dose]; cyclosporin A/dt [Drug Therapy]; cyclosporin A/iv [Intravenous Drug Administration]; cyclosporin A/pd [Pharmacology]; hydrocortisone/ae [Adverse Drug Reaction]; hydrocortisone/cb [Drug Combination]; hydrocortisone/do [Drug Dose]; hydrocortisone/dt [Drug Therapy]; hydrocortisone/iv [Intravenous Drug Administration]; hydrocortisone/pd [Pharmacology]; interleukin 2 receptor/ec [Endogenous Compound]; monoclonal antibody/ae [Adverse Drug Reaction]; monoclonal antibody/ct [Clinical Trial]; monoclonal antibody/dt [Drug Therapy]; monoclonal antibody/iv [Intravenous Drug Administration]; monoclonal antibody/pd [Pharmacology]; placebo; prednisone/ae [Adverse Drug Reaction]; prednisone/cb [Drug Combination]; prednisone/do [Drug Dose]; prednisone/dt [Drug Therapy]; prednisone/iv [Intravenous Drug Administration]; prednisone/pd [Pharmacology]; steroid/ae [Adverse Drug Reaction]; steroid/cb [Drug Combination]; steroid/dt [Drug Therapy]; steroid/iv [Intravenous Drug Administration]; steroid/pd [Pharmacology]; tuberculostatic agent/dt [Drug Therapy]; tuberculostatic agent/pd [Pharmacology].

Drug Trade Names and Manufacturers

neoral, simulect

Link to the Ovid Full Text or citation:

Click here for full text options

121.

CMT-3 CollaGenex.

Fingleton B.

Current Opinion in Investigational Drugs. 4 (12) (pp 1460-1467), 2003. Date of Publication: December 2003.

AN: 2004038585

CMT-3 is an orally active matrix metalloprotease inhibitor, and one of a series of inhibitors of multiple proteases and cytokines, under development by CollaGenex. The compound is currently undergoing phase II trials for the potential treatment of cancer, in particular metastatic cancer and HIV-related Kaposi's sarcoma. © Current Drugs.

Institution

(Fingleton) Vanderbilt University, Medical Center, Department of Cancer Biology, 23rd and Pierce Avenues, Nashville, TN 37232-6840, United States

Publisher

Current Drugs Ltd

Emtree Heading

anemia/si [Side Effect]; anorexia/si [Side Effect]; antineoplastic activity; aplastic anemia/si [Side Effect]; arthralgia/si [Side Effect]; blister/si [Side Effect]; *cancer/dr [Drug Resistance]; *cancer/dt [Drug Therapy]; cancer chemotherapy; clinical trial; constipation/si [Side Effect]; diabetic nephropathy/co [Complication]; diabetic nephropathy/dt [Drug Therapy]; dizziness/si [Side Effect]; dose response; drug absorption; drug bioavailability; drug blood level; drug contraindication; drug efficacy; drug fever/si [Side Effect]; drug metabolism; drug potency; drug synthesis; drug tolerability; drug withdrawal; erythema/si [Side Effect]; fatigue/si [Side Effect]; headache/si [Side Effect]; hemolytic anemia/si [Side Effect]; highly active antiretroviral therapy; human; Human immunodeficiency virus infection/dt [Drug Therapy]; Kaposi sarcoma/co [Complication]; Kaposi sarcoma/dt [Drug Therapy]; liver disease/si [Side Effect]; lung infiltrate/si [Side Effect]; lupus vulgaris/si [Side Effect]; megaloblastic anemia/si [Side Effect]; metastasis/co [Complication]; metastasis/dr [Drug Resistance]; metastasis/dt [Drug Therapy]; musculoskeletal disease/si [Side Effect]; nausea/si [Side Effect]; neurotoxicity/si [Side Effect]; nonhuman; osteoarthritis/dt [Drug Therapy]; osteoporosis/dt [Drug Therapy]; pain/si [Side Effect]; photosensitivity/dt [Drug Therapy]; photosensitivity/pc [Prevention]; photosensitivity/si [Side Effect]; phototoxicity/dt [Drug Therapy]; phototoxicity/pc [Prevention]; phototoxicity/si [Side Effect]; prophylaxis; review; rheumatoid arthritis/dt [Drug Therapy]; sideroblastic anemia/si [Side Effect]; structure activity relation; sunburn/dt [Drug Therapy]; sunburn/pc [Prevention]; sunburn/si [Side Effect]; systemic disease/si [Side Effect]; treatment failure; vomiting/si [Side Effect]; *4 dedimethylaminosancycline/ae [Adverse Drug Reaction]; *4 dedimethylaminosancycline/ct [Clinical Trial]; *4 dedimethylaminosancycline/an [Drug Analysis]; *4 dedimethylaminosancycline/cb [Drug Combination]; *4 dedimethylaminosancycline/cm [Drug Comparison]; *4 dedimethylaminosancycline/cr [Drug Concentration]; *4 dedimethylaminosancycline/dv [Drug Development]; *4 dedimethylaminosancycline/do [Drug Dose]; *4 dedimethylaminosancycline/dt [Drug Therapy]; *4 dedimethylaminosancycline/po [Oral Drug Administration]; *4 dedimethylaminosancycline/pk [Pharmacokinetics]; *4 dedimethylaminosancycline/pd [Pharmacology]; anticonvulsive agent/ct [Clinical Trial]; anticonvulsive agent/cb [Drug Combination]; anticonvulsive agent/dt [Drug Therapy]; anticonvulsive agent/pd [Pharmacology]; antineoplastic agent/ae [Adverse Drug Reaction]; antineoplastic agent/ct [Clinical Trial]; antineoplastic agent/an [Drug Analysis]; antineoplastic agent/cb [Drug Combination]; antineoplastic agent/cm [Drug Comparison]; antineoplastic agent/cr [Drug Concentration]; antineoplastic agent/dv [Drug Development]; antineoplastic agent/do [Drug Dose]; antineoplastic agent/dt [Drug Therapy]; antineoplastic agent/po [Oral Drug Administration]; antineoplastic agent/pk [Pharmacokinetics]; antineoplastic agent/pd [Pharmacology]; antiretrovirus agent/cb [Drug Combination]; antiretrovirus agent/dt [Drug Therapy]; arthrostat/dt [Drug Therapy]; arthrostat/pd [Pharmacology]; cmt 1; cmt 4; cmt 5; cmt 6; cmt 7; cmt 8; cytokine inhibitor/ae [Adverse Drug Reaction]; cytokine inhibitor/ct [Clinical Trial]; cytokine inhibitor/an [Drug Analysis]; cytokine inhibitor/cb [Drug Combination]; cytokine inhibitor/cm [Drug Comparison]; cytokine inhibitor/cr [Drug Concentration]; cytokine inhibitor/dv [Drug Development]; cytokine inhibitor/do [Drug Dose]; cytokine inhibitor/dt [Drug Therapy]; cytokine inhibitor/po [Oral Drug Administration]; cytokine inhibitor/pk [Pharmacokinetics]; cytokine inhibitor/pd [Pharmacology]; dermastat/dt [Drug Therapy]; dermastat/pd [Pharmacology]; doxycycline/ae [Adverse Drug Reaction]; doxycycline/ct [Clinical Trial]; doxycycline/cm [Drug Comparison]; doxycycline/dt [Drug Therapy]; doxycycline/po [Oral Drug Administration]; doxycycline/pd [Pharmacology]; matrix metalloproteinase inhibitor/ae [Adverse Drug Reaction]; matrix metalloproteinase inhibitor/ct [Clinical Trial]; matrix metalloproteinase inhibitor/an [Drug Analysis]; matrix metalloproteinase inhibitor/cb [Drug Combination]; matrix metalloproteinase inhibitor/cm [Drug Comparison]; matrix metalloproteinase inhibitor/cr [Drug Concentration]; matrix metalloproteinase inhibitor/dv [Drug Development]; matrix metalloproteinase inhibitor/do [Drug Dose]; matrix metalloproteinase inhibitor/dt [Drug Therapy]; matrix metalloproteinase inhibitor/po [Oral Drug Administration]; matrix metalloproteinase inhibitor/pk [Pharmacokinetics]; matrix metalloproteinase inhibitor/pd [Pharmacology]; nephrostat/dt [Drug Therapy]; nephrostat/pd [Pharmacology]; nsc 683551; osteostat/dt [Drug Therapy]; osteostat/pd [Pharmacology]; placebo; proteinase inhibitor/ae [Adverse Drug Reaction]; proteinase inhibitor/ct [Clinical Trial]; proteinase inhibitor/an [Drug Analysis]; proteinase inhibitor/cb [Drug Combination]; proteinase inhibitor/cm [Drug Comparison]; proteinase inhibitor/cr [Drug Concentration]; proteinase inhibitor/dv [Drug Development]; proteinase inhibitor/do [Drug Dose]; proteinase inhibitor/dt [Drug Therapy]; proteinase inhibitor/po [Oral Drug Administration]; proteinase inhibitor/pk [Pharmacokinetics]; proteinase inhibitor/pd [Pharmacology]; rheumastat/dt [Drug Therapy]; rheumastat/pd [Pharmacology]; sunscreen/dt [Drug Therapy]; tetracycline derivative/ae [Adverse Drug Reaction]; tetracycline derivative/ct [Clinical Trial]; tetracycline derivative/an [Drug Analysis]; tetracycline derivative/cb [Drug Combination]; tetracycline derivative/cm [Drug Comparison]; tetracycline derivative/cr [Drug Concentration]; tetracycline derivative/dv [Drug Development]; tetracycline derivative/do [Drug Dose]; tetracycline derivative/dt [Drug Therapy]; tetracycline derivative/po [Oral Drug Administration]; tetracycline derivative/pk [Pharmacokinetics]; tetracycline derivative/pd [Pharmacology]; unclassified drug.

Drug Trade Names and Manufacturers

Collagenex, arthrostat, cmt 1, cmt 3: Collagenex, cmt 4, cmt 5, cmt 6, cmt 7, cmt 8, col 3, dermastat, metastat, nephrostat, nsc 683551, osteostat, rheumastat

Link to the Ovid Full Text or citation:

Click here for full text options

122.

Adverse events of standardized regimens of corticosteroids for prophylaxis and treatment of nerve function impairment in leprosy: Results from the 'TRIPOD' trials.

Richardus J.H., Withington S.G., Anderson A.M., Croft R.P., Nicholls P.G., Van Brakel W.H., Smith W.C.S.

Leprosy Review. 74 (4) (pp 319-327), 2003. Date of Publication: December 2003.

AN: 2004014112

Reactions in leprosy causing nerve function impairment (NFI) are increasingly treated with standardized regimens of corticosteroids, often under field conditions. Safety concerns led to an assessment of adverse events of corticosteroids, based on data of three trials studying prevention of NFI (the TRIPOD study). A multicentre, randomized, double-blind placebo-controlled trial was conducted in leprosy control programmes in Nepal and Bangladesh. Treatment was with prednisolone according to fixed schedules for 16 weeks, starting in one trial with 20 mg/day (prophylactic regimen: total dosage 1.96 g) and in the other two trials with 40 mg/day (therapeutic regimen: total dosage 2.52 g). Minor adverse events were defined as moon face, fungal infections, acne, and gastric pain requiring antacid. Major adverse events were defined as psychosis, peptic ulcer, glaucoma, cataract, diabetes and hypertension. Also, the occurrence of infected plantar, palmar, and corneal ulceration was monitored, together with occurrence of TB. Considering all three trials together, minor adverse events were observed in 130/815 patients (16%). Of these, 51/414 (12%) were in the placebo group and 79/401 (20%) in the prednisolone group. The relative risk for minor adverse events in the prednisolone group was 1.6 (P= 0.004). Adverse events with a significantly increased risk were acne, fungal infections and gastric pain. Major adverse events were observed in 15/815 patients (2%); 7/414 (2%) in the placebo group and 8/401 (2%) in the prednisolone group. No major adverse events had a significantly increased risk in the prednisolone arm of the trials. No cases of TB were observed in 300 patients who could be followed-up for 24 months. Standardized regimens of corticosteroids for both prophylaxis and treatment of reactions and NFI in leprosy under field conditions in developing countries are safe when a standard pre-treatment examination is performed, treatment for minor conditions can be carried out by field staff, referral for specialized medical care is possible, and sufficient follow-up is done during and after treatment.

Institution

(Richardus) Department of Public Health, Erasmus MC, University Medical Center Rotterdam, P.O. Box 1738, 3000 DR Rotterdam, Netherlands (Withington) Leprosy Mission Bangladesh, House 17A, Banani (Old) DOHS, Road 3, Dhaka 1206, Bangladesh

(Anderson) Intl. Nepal Fellowship-RELEASE, P.O. BOX 28, Pokhara, Nepal

(Croft) 56a St Peter's Road, Early, Reading RG6 1PH, United Kingdom

(Nicholls, Smith) Department of Public Health, University of Aberdeen, Polwarth Building, Foresterhill, Aberdeen, AB25 2ZD, United Kingdom

(Van Brakel) KIT Leprosy Unit, Wibautstraat 137 J, 1097 DN Amsterdam, Netherlands

Publisher

British Leprosy Relief Association

Emtree Heading

acne/si [Side Effect]; adolescent; adult; article; Bangladesh; cataract/si [Side Effect]; clinical trial; controlled clinical trial; controlled study; cornea ulcer/si [Side Effect]; corticosteroid therapy; diabetes mellitus/si [Side Effect]; disability; double blind procedure; glaucoma/si [Side Effect]; human; hypertension/si [Side Effect]; infection/co [Complication]; *leprosy/di [Diagnosis]; major clinical study; moon face/si [Side Effect]; multicenter study; mycosis/co [Complication]; Nepal; *nerve function impairment/co [Complication]; *nerve function impairment/dt [Drug Therapy]; *nerve function impairment/pc [Prevention]; *neuropathy/co [Complication]; *neuropathy/dt [Drug Therapy]; *neuropathy/pc [Prevention]; peptic ulcer/si [Side Effect]; psychosis/si [Side Effect]; randomized controlled trial; risk assessment; side effect/si [Side Effect]; stomach pain/si [Side Effect]; treatment outcome; tuberculosis/co [Complication]; *corticosteroid/ae [Adverse Drug Reaction]; *corticosteroid/ct [Clinical Trial]; *corticosteroid/do [Drug Dose]; *corticosteroid/dt [Drug Therapy]; placebo; *prednisolone/ae [Adverse Drug Reaction]; *prednisolone/ct [Clinical Trial]; *prednisolone/do [Drug Dose]; *prednisolone/dt [Drug Therapy].

Link to the Ovid Full Text or citation:

Click here for full text options

123.

T-cell-activation inhibitors in rheumatoid arthritis.

Lorenz H.-M.

BioDrugs. 17 (4) (pp 263-270), 2003. Date of Publication: 2003.

AN: 2003336612

As rheumatoid arthritis (RA) is a chronic inflammatory disabling disease and a cure is not available, optimisation of therapeutic strategies is mandatory. Within recent years many new details of the inflammatory cascade(s) have been elaborated, leading to new therapeutic options such as neutralisation of tumour necrosis factor-alpha (TNFalpha). T-cell inhibition is another new approach to the treatment of RA. However, it is important to note two points: first, the role of T lymphocytes in the initiation and/or perpetuation of RA is still debated controversially, Second, there are few truly T-cell-specific agents that have proven to be effective and are established in the treatment of inflammatory disorders. Leflunomide may be considered one such agent; another in development is the fusion protein CTLA4-Ig. From a clinical perspective, studies demonstrating efficacy of these agents might represent the strongest support for a role of T cells in RA. In addition to leflunomide and CTLA4-Ig, therapeutic agents with activity against T cells, including anti-CD4 antibodies, cyclosporin, tacrolimus and T-cell receptor (TCR)-Vbeta-chain vaccination strategies, have been studied in patients with RA. Combination therapies including any of these T-cell-activation inhibitors with non-T-cell-specific agents such as methotrexate, antimalarials or anti-TNFalpha biologicals may prove the most effective strategies in controlling this complex disease.

Institution

(Lorenz) Department of Medicine III, Rheumatology Inst. Clin. Immunol., University of Erlangen-Nuremberg, Erlangen, Germany (Lorenz) Department of Medicine III, Clinical Immunology, University of Erlangen-Nuremberg, Krankenhausstr. 12, Erlangen, 91054, Germany

Publisher

Adis International Ltd

Emtree Heading

alopecia/si [Side Effect]; bone destruction/co [Complication]; cartilage degeneration/co [Complication]; cell specificity; chronic inflammation/dt [Drug Therapy]; chronic inflammation/et [Etiology]; clinical feature; clinical protocol; clinical trial; diarrhea/si [Side Effect]; disease course; drug dose regimen; drug efficacy; drug formulation; drug hypersensitivity/si [Side Effect]; drug infusion; drug mechanism; drug safety; drug specificity; drug surveillance program; drug tolerability; dyspepsia/si [Side Effect]; ecchymosis/si [Side Effect]; enzyme inhibition; gastroenteritis/si [Side Effect]; headache/si [Side Effect]; human; hypercholesterolemia/si [Side Effect]; hyperglycemia/si [Side Effect]; hypertension/si [Side Effect]; leukocytoclastic vasculitis/si [Side Effect]; liver necrosis/si [Side Effect]; liver toxicity/si [Side Effect]; low drug dose; lung tuberculosis/si [Side Effect]; lymphocyte function; lymphocytopenia/si [Side Effect]; muscle cramp/si [Side Effect]; nausea/si [Side Effect]; nephrotoxicity/si [Side Effect]; non insulin dependent diabetes mellitus/si [Side Effect]; nonhuman; outcomes research; pancreatitis/si [Side Effect]; pathophysiology; pneumonia/si [Side Effect]; priority journal; pruritus/si [Side Effect]; rash/si [Side Effect]; review; *rheumatoid arthritis/dt [Drug Therapy]; *rheumatoid arthritis/et [Etiology]; Stevens Johnson syndrome/si [Side Effect]; *T lymphocyte activation; treatment planning; tremor/si [Side Effect]; vaccination; vasculitis/si [Side Effect]; vasodilatation; antimalarial agent/cb [Drug Combination]; antimalarial agent/dt [Drug Therapy]; antirheumatic agent/dt [Drug Therapy]; belatacept/ae [Adverse Drug Reaction]; belatacept/ct [Clinical Trial]; belatacept/cb [Drug Combination]; belatacept/dv [Drug Development]; belatacept/do [Drug Dose]; belatacept/dt [Drug Therapy]; belatacept/iv [Intravenous Drug Administration]; belatacept/pd [Pharmacology]; CD4 antigen/ec [Endogenous Compound]; chloroquine/ct [Clinical Trial]; chloroquine/cm [Drug Comparison]; chloroquine/dt [Drug Therapy]; cyclosporin A/ae [Adverse Drug Reaction]; cyclosporin A/ct [Clinical Trial]; cyclosporin A/cb [Drug Combination]; cyclosporin A/cm [Drug Comparison]; cyclosporin A/do [Drug Dose]; cyclosporin A/dt [Drug Therapy]; cyclosporin A/pr [Pharmaceutics]; cyclosporin A/pk [Pharmacokinetics]; cyclosporin A/pd [Pharmacology]; cytotoxic T lymphocyte antigen 4/ae [Adverse Drug Reaction]; cytotoxic T lymphocyte antigen 4/ct [Clinical Trial]; cytotoxic T lymphocyte antigen 4/cb [Drug Combination]; cytotoxic T lymphocyte antigen 4/do [Drug Dose]; cytotoxic T lymphocyte antigen 4/dt [Drug Therapy]; cytotoxic T lymphocyte antigen 4/iv [Intravenous Drug Administration]; cytotoxic T lymphocyte antigen 4/pd [Pharmacology]; dihydroorotate dehydrogenase/ec [Endogenous Compound]; etanercept/ct [Clinical Trial]; etanercept/cb [Drug Combination]; etanercept/dt [Drug Therapy]; HLA DR antigen/ec [Endogenous Compound]; HLA DR4 antigen/ec [Endogenous Compound]; hybrid protein/dt [Drug Therapy]; hybrid protein/pd [Pharmacology]; *immunomodulating agent/cb [Drug Combination]; *immunomodulating agent/dt [Drug Therapy]; *immunomodulating agent/pd [Pharmacology]; infliximab/ae [Adverse Drug Reaction]; infliximab/cb [Drug Combination]; infliximab/dt [Drug Therapy]; interleukin 1 receptor blocking agent/dt [Drug Therapy]; interleukin 1 receptor blocking agent/pd [Pharmacology]; interleukin 6 receptor/ec [Endogenous Compound]; ir 501/ct [Clinical Trial]; ir 501/dt [Drug Therapy]; ir 501/im [Intramuscular Drug Administration]; keliximab/ae [Adverse Drug Reaction]; keliximab/ct [Clinical Trial]; keliximab/do [Drug Dose]; keliximab/dt [Drug Therapy]; leflunomide/ae [Adverse Drug Reaction]; leflunomide/ct [Clinical Trial]; leflunomide/cb [Drug Combination]; leflunomide/cm [Drug Comparison]; leflunomide/do [Drug Dose]; leflunomide/dt [Drug Therapy]; leflunomide/pd [Pharmacology]; methotrexate/ae [Adverse Drug Reaction]; methotrexate/ct [Clinical Trial]; methotrexate/cb [Drug Combination]; methotrexate/cm [Drug Comparison]; methotrexate/do [Drug Dose]; methotrexate/dt [Drug Therapy]; monoclonal antibody 4162w94/ae [Adverse Drug Reaction]; monoclonal antibody 4162w94/ct [Clinical Trial]; monoclonal antibody 4162w94/dt [Drug Therapy]; OKT 4/ae [Adverse Drug Reaction]; OKT 4/ct [Clinical Trial]; OKT 4/dt [Drug Therapy]; placebo; salazosulfapyridine/ct [Clinical Trial]; salazosulfapyridine/cm [Drug Comparison]; salazosulfapyridine/dt [Drug Therapy]; T lymphocyte activation inhibitor/cb [Drug Combination]; T lymphocyte activation inhibitor/dt [Drug Therapy]; T lymphocyte activation inhibitor/pd [Pharmacology]; T lymphocyte receptor beta chain/ct [Clinical Trial]; T lymphocyte receptor beta chain/dt [Drug Therapy]; T lymphocyte receptor beta chain/im [Intramuscular Drug Administration]; T lymphocyte receptor beta chain/pd [Pharmacology]; tacrolimus/ae [Adverse Drug Reaction]; tacrolimus/ct [Clinical Trial]; tacrolimus/do [Drug Dose]; tacrolimus/dt [Drug Therapy]; tacrolimus/pd [Pharmacology]; tumor necrosis factor alpha/ec [Endogenous Compound]; tumor necrosis factor alpha antibody/ct [Clinical Trial]; tumor necrosis factor alpha antibody/cb [Drug Combination]; tumor necrosis factor alpha antibody/dt [Drug Therapy]; unclassified drug; unindexed drug.

Drug Trade Names and Manufacturers

ir 501, lea 29y

Link to the Ovid Full Text or citation:

Click here for full text options

124.

Patient acceptance of educational voice messages: A review of controlled clinical studies.

Krishna S., Balas E.A., Boren S.A., Maglaveras N.

Methods of Information in Medicine. 41 (5) (pp 360-369), 2002. Date of Publication: 2002.

AN: 2002452778

Objectives: The objective of this study was to evaluate controlled evidence on the impact of automated computer-based telephone messaging technology upon health outcomes, cost savings ond acceptance by patients, caregivers and core providers. Methods: Systematic searches of electronik databases were conducted to find controlled clinical studies of automoted phone messaging used in patient care. Studies were selected based on the three criteria: 1) rondomized controlled trials or controlled trials; 2) patients receiving health care related education, information, advice or reminder for a specific action to be token in their home setting; and 3) use of automated computer-based phone technology to deliver the messages. Informotion abstracted from studies included informotion about the institution, persans targeted, intervention and its effect on heolth outcomes, costs and acceptance by patients ond caregivers. Results. A total of nineteen studies were identified for review. Sixteen studies were randomized controlled trials and three were controlled studies with no randomizotion. Studies were ploced in twa categories, preventive care education and chronic care studies. Preventive core education studies covered childhood immunizations, medication compliance, influenza vaccinations, tuberculosis and health prevention activities and chronic care studies were related to cholesterol, diabetes, hypertension and congestive heart failure. More than 80% of studies showed significant impact upon measurable health outcomes. Conclusions: Controlled evidence substantiates the efficacy of automated telephone communication in improving the quality of care. Educational voice messages are acceptable to patients and represent an important opportunity to enhance telemedicine and telehealth applications.

Institution

(Krishna, Boren) Center for Health Care Quality, University of Missouri, Columbia, MO, United States (Balas) School of Public Health, Saint Louis University, 3545 Lafayette Avenue, St. Louis, MO 63104, United States

(Maglaveras) Lab. of Medical Informatics, Aristotle University, Thessaloniki, Greece

Publisher

Schattauer GmbH

Emtree Heading

article; *automation; caregiver; chronic disease; clinical trial; congestive heart failure; data base; diabetes mellitus; health care cost; health care quality; human; hypertension; immunization; influenza vaccination; patient compliance; *patient education; preventive medicine; priority journal; resource management; *telecommunication; *telephone; treatment outcome; tuberculosis; cholesterol.

Link to the Ovid Full Text or citation:

Click here for full text options

125.

Why evidence-based medicine?.

Jamrozik K.

Bailliere's Best Practice and Research in Clinical Anaesthesiology. 15 (4) (pp 505-518), 2001. Date of Publication: 2001.

AN: 2002066451

This chapter considers briefly what counts as 'evidence' and describes three historical strands, in medical science generally, in epidemiology and in clinical trials, that have converged to give rise to the concept of evidence-based medicine. It then returns to the randomized controlled trial as the fundamental building block of evidence-based practice. Finally, through considering whether all trials are equally useful, it demonstrates that discerning what is the best way to proceed in the clinical management of individual patients and problems is not necessarily a simple matter.

Institution

(Jamrozik) Department of Primary Health Care and General Practice, Imperial College of Science, Technology and Medicine, Reynolds Building, St Dunstan's Road, London, W6 8RP, United Kingdom

Publisher

Bailliere Tindall Ltd

Emtree Heading

ascites/dt [Drug Therapy]; cardiovascular disease/dt [Drug Therapy]; clinical trial; controlled clinical trial; controlled study; deep vein thrombosis/dt [Drug Therapy]; deep vein thrombosis/pc [Prevention]; drug efficacy; drug indication; epidemic; epidemiological data; *evidence based medicine; fever/dt [Drug Therapy]; health care quality; health promotion; history of medicine; human; insulin dependent diabetes mellitus/dt [Drug Therapy]; lung embolism/dt [Drug Therapy]; lung embolism/pc [Prevention]; lung tuberculosis/di [Diagnosis]; lung tuberculosis/dt [Drug Therapy]; medical decision making; medical research; medicine; meta analysis; outcomes research; patient care; pharyngitis/dt [Drug Therapy]; priority journal; public health; publication; randomized controlled trial; review; risk factor; screening test; scurvy/dt [Drug Therapy]; scurvy/pc [Prevention]; statistical analysis; stroke; syphilis/dt [Drug Therapy]; theory; thorax radiography; validation process; acetylsalicylic acid/ct [Clinical Trial]; acetylsalicylic acid/dt [Drug Therapy]; acetylsalicylic acid/pd [Pharmacology]; arsphenamine/dt [Drug Therapy]; citrus fruit extract/dt [Drug Therapy]; digitalis/dt [Drug Therapy]; hydroxymethylglutaryl coenzyme A reductase inhibitor/ct [Clinical Trial]; hydroxymethylglutaryl coenzyme A reductase inhibitor/dt [Drug Therapy]; hydroxymethylglutaryl coenzyme A reductase inhibitor/pd [Pharmacology]; insulin/ct [Clinical Trial]; insulin/dt [Drug Therapy]; insulin/pd [Pharmacology]; penicillin G/ct [Clinical Trial]; penicillin G/dt [Drug Therapy]; penicillin G/pd [Pharmacology]; streptomycin/ct [Clinical Trial]; streptomycin/dt [Drug Therapy]; streptomycin/pd [Pharmacology].

Link to the Ovid Full Text or citation:

Click here for full text options

126.

Effectiveness and problems of PZA-containing 6-month regimen for the treatment of new pulmonary tuberculosis patients.

Wada M.

Kekkaku. 76 (1) (pp 33-43), 2000. Date of Publication: 2000.

AN: 2001058097

One third of the world population has been infected with Mycobacterium tuberculosis, and the number of tuberculosis will increase worldwide without more effective programs of tuberculosis control. Despite of the presence of very potent anti-tuberculosis drugs the global tuberculosis situation is still very serious, and such gloomy feature are caused, at least partly, by the failures in the treatment of tuberculosis. The most important factor for the failure in chemotherapy is incompliance of the patients to the regimens. History of the chemotherapy of tuberculosis can be said as the history of the efforts to reduce such defaulters. Modern chemotherapy of tuberculosis has started from the discovery of streptomycin. Streptomycin monotherapy could improve temporally symptoms and bacteriological status, but could not cure the patients with moderately advanced pulmonary tuberculosis because of the emerge of drug-resistant tuberculosis. This problem was overcome by combining use of para-aminosalicylate and/or isoniazid developed later on. About 97% of patients with pulmonary tuberculosis became bacteriologically quiescent by the 12 months of streptomycin, para-aminosalicylate and isoniazid. Since 1950s through 1970s three drug combination of streptomycin, para-aminosalicylate and isoniazid had been the standard regimen for the treatment of tuberculosis. By the introduction of rifampicin, the duration of chemotherapy could be shortened to 9 months. Subsequent to the successful animal experiments carried out by Grosset which demonstrated that the addition of pyrazinamide for initial 2 months to the standard two-drug combination (isoniazid and rifampicin) could remarkably shorten the duration of chemotherapy, many clinical trials have been done all over the world to compare the efficacy and safety of pyrazinamide-containing intensified short-course regimen with those of standard regimen without pyrazinamide. Sputum negative conversion rates after 2 months of treatment with PZA-regimen was 70 ~ 95%, and the relapse rates after the completion of the treatment course were less than 4%. The incidence of adverse events was less than 4%. The pyrazinamide-containing 6 months short-course regimens has been established as a new standard regimen for the initial treatment of pulmonary tuberculosis worldwide. But, in Japan, this regimen had not been adapted as the standard until April 1996 because of undue fear for high incidence of liver toxicity induced by pyrazinamide. However, in many clinical trials carried out in various parts of the world did not show any causative relationship between the higher incidence of liver toxicity and pyrazinamide. According to our own experience in Fukujuji Hospital, Japan Anti-tuberculosis Association, the frequency of drug induced hepatitis among 632 patients with normal liver function at the onset of chemotherapy was 7.9 percent (50/632) when treated with pyrazinamide-containing regimens, and was similar to that among 412 patients treated with other regimens without pyrazinamide (7.3 percent 30/412). These figures were higher than those reported in the literatures. The risk factors of drug-induced hepatitis so far reported included elderly, positive hepatitis C virus antibody, low serum albumin and so on. Such known risk factors could not wholly explain the higher rate of liver dysfunction observed among our Japanese patients. We have examined additional factors affecting the frequency of drug-induced hepatitis in our hospital, and noticed that the past history of gastrectomy and over-dosing of isoniazid (> 7.5 mg/kg) and/or pyrazinamide (> 30 mg) were relating to the higher incidence of drug-induced hepatitis. Another important finding is that the relapse rate among patients complicated with diabetes mellitus is significantly higher than that of the patients without diabetes millitus (6.31/100 person-years vs 0.90/100 person-years, P < 0.001). Further research will need whether the patients complicated with diabetes mellitus have any immunological deficient to kill Mycobacterium tuberculosis. WHO, CDC and ATS recommended that 4-drug regimen including pyrazinamide for the initial treatment of all cases of tuberculosis. Considering that the incidence of initial resistance to isoniazid is 4.4% in Japan, we should start to treat all cases of newly diagnosed tuberculosis with pyrazinamide - containing regimen (isoniazid, rifampicin, pyrazinamide, plus streptomycin or ethambutol). To do this, further studies on the risk factors of drug-induced hepatitis are urgently needed.

Institution

(Wada) 3-1-24 Matsuyama, Kiyose-shi, Tokyo 204-8533, Japan

Publisher

Japanese Society for Tuberculosis

Emtree Heading

clinical trial; conference paper; diabetes insipidus; drug efficacy; gastrectomy; hepatitis/si [Side Effect]; human; Japan; liver toxicity/si [Side Effect]; *lung tuberculosis/dr [Drug Resistance]; *lung tuberculosis/dt [Drug Therapy]; *lung tuberculosis/ep [Epidemiology]; Mycobacterium tuberculosis; patient compliance; treatment failure; tuberculosis control; albumin/ec [Endogenous Compound]; aminosalicylic acid/cb [Drug Combination]; aminosalicylic acid/dt [Drug Therapy]; ethambutol/cb [Drug Combination]; ethambutol/dt [Drug Therapy]; hepatitis C antibody/ec [Endogenous Compound]; isoniazid/cb [Drug Combination]; isoniazid/do [Drug Dose]; isoniazid/dt [Drug Therapy]; *pyrazinamide/ae [Adverse Drug Reaction]; *pyrazinamide/ct [Clinical Trial]; *pyrazinamide/cb [Drug Combination]; *pyrazinamide/do [Drug Dose]; *pyrazinamide/dt [Drug Therapy]; rifampicin/cb [Drug Combination]; rifampicin/dt [Drug Therapy]; streptomycin/cb [Drug Combination]; streptomycin/dt [Drug Therapy].

Link to the Ovid Full Text or citation:

Click here for full text options

127.

Long-term peritoneal dialysis outcome in a single center.

Cancarini G.C., Sandrini M., Vizzardi V., Scaini P., Mombelloni S., Pola A., Maiorca R.

Peritoneal Dialysis International. 20 (SUPPL. 2) (pp S121-S126), 2000. Date of Publication: 2000.

AN: 2000221310

Institution

(Cancarini, Sandrini, Vizzardi, Scaini, Mombelloni, Pola, Maiorca) Department of Nephrology, University of Brescia, Division of Nephrology, Brescia, Italy (Cancarini) Divisione di Nefrologia, Azienda Ospedaliera, Spedali Civili di Brescia, Piazza Ospedale 1, Brescia 25123, Italy

Publisher

Multimed Inc.

Emtree Heading

adult; age; aged; article; biocompatibility; cerebrovascular disease; clinical trial; cohort analysis; comorbidity; continuous ambulatory peritoneal dialysis; female; hemodialysis; host resistance; human; hypertension; insulin dependent diabetes mellitus; ischemic heart disease; kidney failure/th [Therapy]; kidney transplantation; major clinical study; male; non insulin dependent diabetes mellitus; peripheral vascular disease; *peritoneal dialysis; priority journal; respiratory tract infection; risk factor; survival rate; treatment indication; treatment outcome; tuberculosis; peritoneal dialysis fluid.

Link to the Ovid Full Text or citation:

Click here for full text options

128.

Assessment of a combined preparation of isoniazid, rifampicin and pyrazinamide (Rifater) in the initial phase of chemotherapy in three 6- month regimens for smear-positive pulmonary tuberculosis: A five-year follow- up report.

Teo S.K.

International Journal of Tuberculosis and Lung Disease. 3 (2) (pp 126-132), 1999. Date of Publication: February 1999.

AN: 1999116554

SETTING: Singapore Tuberculosis Service. OBJECTIVE: To assess the acceptability, efficacy and relapse rate of a combined formulation of three drugs-isoniazid, rifampicin and pyrazinamide (Rifater)-given in the initial phase of chemotherapy in three 6-month regimens (2SHRZ/4H3R3, 1SHRZ/5H3R3 and 2HRZ/4H3R3) under direct observation for all patients. DESIGN: A randomised, controlled, unblinded study comparing a group of patients treated with Rifater and another given the three component drugs as separate formulations. RESULTS: The 310 patients admitted to the study were divided into two groups of 155 patients. The frequency of side effects was similar in both groups. Of 271 patients with drug-sensitive strains who had completed treatment without interruption, sputum cultures converted in all patients. At the end of 5 years, there were 15 relapses: three (2.2%) in the separate drugs group and 12 (9.3%) in the Rifater group. Exclusion of two cases in the Rifater group, one with silicotuberculosis and another with no bacteriological confirmation of diagnosis, gave a relapse rate of 7.9% (P = 0.03 for the comparison of relapse rates in the two groups). CONCLUSION: A combined formulation of three drugs given daily in the initial phase of 6- month short-course therapy, followed by intermittent treatment with isoniazid and rifampicin given three times a week under direct observation for all patients, appears to be less effective than treatment with the component drugs given as separate formulations.

Institution

(Teo) Department of Medicine, Alexandra Hospital, Singapore, Singapore (Teo) 378 Alexandra Road, Singapore 159964, Singapore

Publisher

International Union against Tubercul. and Lung Dis.

Emtree Heading

article; clinical trial; combination chemotherapy; controlled clinical trial; controlled study; diabetes mellitus; drug efficacy; follow up; human; Human immunodeficiency virus infection; *lung tuberculosis/dr [Drug Resistance]; *lung tuberculosis/dt [Drug Therapy]; *lung tuberculosis/ep [Epidemiology]; major clinical study; Mycobacterium tuberculosis; priority journal; randomized controlled trial; recurrence risk; Singapore; sputum culture; treatment outcome; *isoniazid/ct [Clinical Trial]; *isoniazid/cb [Drug Combination]; *isoniazid/cm [Drug Comparison]; *isoniazid/do [Drug Dose]; *isoniazid/dt [Drug Therapy]; *pyrazinamide/ct [Clinical Trial]; *pyrazinamide/cb [Drug Combination]; *pyrazinamide/cm [Drug Comparison]; *pyrazinamide/do [Drug Dose]; *pyrazinamide/dt [Drug Therapy]; *rifampicin/ct [Clinical Trial]; *rifampicin/cb [Drug Combination]; *rifampicin/cm [Drug Comparison]; *rifampicin/do [Drug Dose]; *rifampicin/dt [Drug Therapy]; *rifater/ct [Clinical Trial]; *rifater/cb [Drug Combination]; *rifater/cm [Drug Comparison]; *rifater/do [Drug Dose]; *rifater/dt [Drug Therapy]; streptomycin/cb [Drug Combination]; streptomycin/dt [Drug Therapy].

Drug Trade Names and Manufacturers

Bracco [Italy], Lepetit [Italy], rifater: Lepetit [Italy]

Link to the Ovid Full Text or citation:

Click here for full text options
