# Supplementary material for: Effect of glycemic control and type of diabetes treatment on TB treatment outcomes among people with TB-diabetes: A systematic review (updated August 2024)
Source: PLoS One. 2025 Jul 18;20(7):e0328619. doi: 10.1371/journal.pone.0328619 (PMC12273911; doi:10.1371/journal.pone.0328619)
Supplement: S1 Appendix — (ZIP) [file pone.0328619.s004.zip › S1 appendix_old/EMBASE/298-1_with RISK search reuslts no 15.doc]

Database: Embase <1996 to 2016 September 16>

Search Strategy:

--------------------------------------------------------------------------------

1	tuberculosis.mp. or exp tuberculosis/ (151164)

2	exp treatment outcome/ or treatment outcome*.mp. (1189741)

3	diabetes mellitus.mp. or exp diabetes mellitus/ (627157)

4	diabetes mellitus/ (362441)

5	DM.mp. (48079)

6	diabetes.mp. (663193)

7	mellitus.mp. (588341)

8	3 or 4 or 5 or 6 or 7 (715025)

9	1 and 2 and 8 (724)

10	cohort studies.mp. or cohort analysis/ (263408)

11	exp risk factor/ or cohort.mp. or exp risk/ (2148599)

12	10 or 11 (2148599)

13	9 and 10 (81)

14	9 and 12 (304)

15	limit 14 to human (298)

16	limit 13 to human (81)

17	trial*.mp. or exp controlled clinical trial/ or exp "clinical trial (topic)"/ or exp "randomized controlled trial (topic)"/ or exp "controlled clinical trial (topic)"/ (1555047)

18	random.ab. (191735)

19	randomized controlled trial.pt. (0)

20	controlled clinical trial.pt. (0)

21	randomized.ab. (454621)

22	placebo.ab. (188623)

23	clinical trials as topic.sh. (15)

24	trial.ti. (176210)

25	17 or 18 or 19 or 20 or 21 or 22 or 23 or 24 (1831290)

26	9 and 25 (128)

27	limit 26 to human (128)

***************************

1.

Long-term study of infliximab for psoriasis in daily practice: drug survival depends on combined treatment, obesity and infusion reactions.

Spertino J., Lopez-Ferrer A., Vilarrasa E., Puig L.

Journal of the European Academy of Dermatology and Venereology. 28 (11) (pp 1514-1521), 2014. Date of Publication: 01 Nov 2014.

AN: 2014775069

Background: Infiximab has been shown to be highly effective in phase III clinical trials, but limited information is available regarding drug survival and maintenance of efficacy beyond 1 year in real-life clinical setting. Objectives: To analyse the efficacy and safety of infliximab in a large number of patients with a long follow-up and to identify clinical factors associated with long-term drug survival. Methods: A retrospective review of patients with moderate-to-severe psoriasis treated with infliximab from March 2004 to August 2012 at a tertiary dermatology centre was carried out. Results: In total, 63 treatment courses with infliximab were administered to 56 patients. The mean duration of treatment was 31.6 months. The only significant positive predictor of drug survival was combination treatment [hazard ratio (HR) vs. monotherapy 2.90, 95% confidence interval (CI) 1.42-5.92]. Significant negative predictors of drug survival were obesity (HR 0.40, 95% CI 0.19-0.87) and infusion reactions (HR 0.40, 95% CI 0.19-0.87). Infusion reactions occurred in 13 (23%) of our patients and were a reason for discontinuation of treatment in 5. Conclusions: This retrospective review of a cohort of patients with moderate-to-severe psoriasis treated with infliximab in daily practice shows that the PASI75 response rates at 24 and 52 weeks of treatment are similar to those of the pivotal studies, but 37 courses of treatment (59%) had to be discontinued after a median of 12 months. The major cause for discontinuation was loss of response, in 18 cases. Combination treatment, obesity and infusion reactions were found to be predictors of drug survival.

Institution

(Spertino, Lopez-Ferrer, Vilarrasa, Puig) Department of Dermatology, Hospital de la Santa Creu i Sant Pau, Universitat Autonoma de Barcelona, Barcelona, Catalonia, Spain

Publisher

Blackwell Publishing Ltd

Emtree Heading

adult; aged; aminotransferase blood level; antibody titer; arthralgia/si [Side Effect]; arthralgia/dt [Drug Therapy]; backache/si [Side Effect]; bacterial skin disease/si [Side Effect]; basal cell carcinoma/si [Side Effect]; biological therapy; body mass; body temperature; chemoprophylaxis; chest tightness/si [Side Effect]; cholesteatoma/si [Side Effect]; clinical evaluation; *combination drug therapy; controlled study; diabetes mellitus/si [Side Effect]; dizziness/si [Side Effect]; *drug efficacy; *drug safety; drug treatment failure; drug withdrawal; dyspnea/si [Side Effect]; face erythema/si [Side Effect]; female; fever/si [Side Effect]; fever/dt [Drug Therapy]; fibromyalgia/si [Side Effect]; flushing; follow up; gastroenteritis/si [Side Effect]; headache/si [Side Effect]; headache/si [Side Effect]; heart palpitation/si [Side Effect]; human; hypertension/si [Side Effect]; hypertransaminasemia/si [Side Effect]; hypotension/si [Side Effect]; *infusion related reaction/si [Side Effect]; knee prosthesis; large cell lung carcinoma/si [Side Effect]; latent tuberculosis/di [Diagnosis]; latent tuberculosis/pc [Prevention]; latent tuberculosis/si [Side Effect]; latent tuberculosis/dt [Drug Therapy]; leukoplakia/si [Side Effect]; *long term survival; lung tuberculosis/di [Diagnosis]; lung tuberculosis/pc [Prevention]; lung tuberculosis/dt [Drug Therapy]; lupus like syndrome/si [Side Effect]; major clinical study; male; medical record review; monotherapy; myalgia/si [Side Effect]; myalgia/dt [Drug Therapy]; Mycobacterium tuberculosis test kit; nausea/si [Side Effect]; *obesity; otitis/si [Side Effect]; paraproteinemia/si [Side Effect]; phototherapy; priority journal; prosthesis infection/co [Complication]; pruritus/si [Side Effect]; *psoriasis; *psoriasis vulgaris/th [Therapy]; *psoriasis vulgaris/dt [Drug Therapy]; retrospective study; review; rigor/si [Side Effect]; side effect/si [Side Effect]; squamous cell carcinoma/si [Side Effect]; stridor/si [Side Effect]; survival rate; survival time; sweating; systemic therapy; systolic blood pressure; thorax pain/si [Side Effect]; treatment duration; tuberculin test; ultraviolet A radiation; upper respiratory tract infection/si [Side Effect]; urticaria/si [Side Effect]; vagina candidiasis/si [Side Effect]; viral skin disease/si [Side Effect]; wheezing/si [Side Effect]; adalimumab/dt [Drug Therapy]; aminotransferase/ec [Endogenous Compound]; antinuclear antibody/ec [Endogenous Compound]; cyclosporin A/ae [Adverse Drug Reaction]; cyclosporin A/cb [Drug Combination]; cyclosporin A/cm [Drug Comparison]; cyclosporin A/dt [Drug Therapy]; efalizumab/dt [Drug Therapy]; etanercept/dt [Drug Therapy]; etretin/ae [Adverse Drug Reaction]; etretin/cb [Drug Combination]; etretin/cm [Drug Comparison]; etretin/dt [Drug Therapy]; *infliximab/ae [Adverse Drug Reaction]; *infliximab/cb [Drug Combination]; *infliximab/cm [Drug Comparison]; *infliximab/dt [Drug Therapy]; isoniazid/dt [Drug Therapy]; methotrexate/ae [Adverse Drug Reaction]; methotrexate/cb [Drug Combination]; methotrexate/cm [Drug Comparison]; methotrexate/dt [Drug Therapy]; nonsteroid antiinflammatory agent/dt [Drug Therapy]; psoralen; ustekinumab/dt [Drug Therapy].

Link to the Ovid Full Text or citation:

Click here for full text options

2.

Prolonged hospitalisation for immigrants and high risk patients with positive smear pulmonary tuberculosis.

Luzzati R., Confalonieri M., Cazzadori A., Della Loggia P., Cifaldi R., Fabris C., Biolo M., Borelli M., Longo C., Concia E.

Monaldi Archives for Chest Disease - Cardiac Series. 75 (2) (pp 141-145), 2011. Date of Publication: 2011.

AN: 20160591601

Background and objective. Tuberculosis (TB) occurring in immigrants and resistance to drugs are major problems for TB control in Western countries. Directly observed therapy (DOT) reduces disease transmission, but this approach may have poor results among illegal immigrants. Our aim was to evaluate a prolonged hospitalisation programme to improve early outcome of TB treatment in high risk patients. Methods. All the consecutive adult patients with sputum smear-positive pulmonary TB admitted to 2 Italian referral TB Centres were evaluated. Hospital-based DOT was provided to high risk patients up-to smear conversion. Demographic, microbiological and clinical conditions, as potential factors associated with confirmed smear conversion at 60 and 90 days of anti-tuberculous therapy were evaluated. Results. 122 patients were studied, 45.9% of them were immigrants (20% illegal) from high-prevalence TB countries. HIV testing was negative in all cases. Twelve patients had M. tuberculosis resistant to > 1 first-line anti-tuberculous agents. The rate of defaulting from TB treatment was 7.3%. Sputum smear became negative in 84.4% cases after 60 days and 93.3% cases after 90 days. At such time, smear conversion rates were similar among different high risk subgroups such as illegal immigrants (95.9%), legal foreign-born (92.5%) and Italian persons (94.8%). Persistent sputum smear positivity was independently correlated with the extent of pulmonary lesions at 60 (p<0.0001) and 90 days (p=0.038) of hospital-based DOT. Conclusions. These findings suggest that prolonged hospitalisation for illegal immigrants and high risk TB patients, may positively influence the early outcome of TB treatment despite of drug resistance and legal status.

Institution

(Luzzati) Infectious Diseases Unit, University Hospital, Trieste, Italy (Confalonieri, Cifaldi, Biolo, Longo) Department of Pulmonology, University Hospital, Strada di Fiume 447, Trieste 34149, Italy

(Cazzadori, Della Loggia, Concia) Infectious Diseases Unit, University Hospital, Verona, Italy

(Fabris) Department of Microbiology, University Hospital, Trieste, Italy

(Borelli) Department of Mathematics and Computer Science, University of Trieste, Italy

Publisher

Fondazione Salvatore Maugeri (Via Ferrata 8, Pavia 27100, Italy)

Emtree Heading

adult; alcohol abuse; antibiotic resistance; article; bacterium isolation; chronic obstructive lung disease; clinical effectiveness; controlled study; demography; diabetes mellitus; disease association; female; follow up; health care cost; *high risk patient; HIV test; hospital admission; hospital discharge; *hospitalization; human; illegitimacy; immigrant; Italian (citizen); liver disease; *lung tuberculosis/di [Diagnosis]; *lung tuberculosis/dt [Drug Therapy]; major clinical study; male; malignant neoplastic disease; medical history; microbiological examination; Mycobacterium tuberculosis; neurologic disease; patient referral; peripheral lung lesion/di [Diagnosis]; prevalence; *prolonged hospitalization; smear; treatment duration; *treatment outcome; tuberculosis/dt [Drug Therapy]; isoniazid/dt [Drug Therapy]; rifampicin/dt [Drug Therapy]; tuberculostatic agent/dt [Drug Therapy].

Link to the Ovid Full Text or citation:

Click here for full text options

3.

Diabetic tuberculosis.

Kalra S., Atreja A.

Journal of the Pakistan Medical Association. 66 (9) (pp 1194-1195), 2016. Date of Publication: September 2016.

AN: 20160595722

Diabetes and tuberculosis coexist together, and influence each other's natural history and treatment outcomes significantly. This assumes clinical as well as public health significance. This article describes these associations, and discusses action that can be taken at the primary care level to tackle this challenge.

Institution

(Kalra) Department of Endocrinology, Bharti Hospital, Karnal, India (Atreja) Department of Pulmonary Medicine, MMIMSR, Mullana, India

Publisher

Pakistan Medical Association

Emtree Heading

article; clinical feature; *diabetes mellitus/et [Etiology]; *diabetic tuberculosis/et [Etiology]; disease association; human; infection risk; mortality; Mycobacterium tuberculosis; pathogenesis; recurrent disease; risk assessment; screening; sputum analysis; systematic review (topic); treatment failure; *tuberculosis/et [Etiology]; antidiabetic agent; glucose/ec [Endogenous Compound]; interleukin 10/ec [Endogenous Compound]; tuberculostatic agent.

Link to the Ovid Full Text or citation:

Click here for full text options

4.

Screening for diabetes mellitus in tuberculosis patients in a referral center in Iran.

Baghaei P., Tabarsi P., Marjani M., Moniri A., Masjedi M.R.

Infectious Diseases. 47 (7) (pp 472-476), 2015. Date of Publication: 2015.

AN: 20160253952

Background: A significant link between diabetes mellitus (DM) and tuberculosis (TB) has been widely demonstrated. DM increases the risk of TB in all aspects. The aims of this study were to assess the prevalence of DM among newly diagnosed TB patients, to screen these patients for DM, and to determine the number needed to screen (NNS) to diagnose new cases of DM. Methods: A prospective cohort descriptive study was carried out in Iranian adults admitted to the National Research Institute of Tuberculosis and Lung Disease from 2012 to 2013 with a new diagnosis of TB. Glycated hemoglobin (HbA1c) and fasting blood glucose (FBG) were measured for all patients. Results: Of the 293 patients included, 101 (34.5%) had DM. DM was newly diagnosed in 45 (19%) patients. The number needed to screen was 5 to identify one new DM case. Age > 40 years was associated with DM in this population (odds ratio (OR)=3.91, 95% confidence interval (CI)=1.47 - 10.38). Conclusion: Screening for DM should be performed routinely in patients with TB and may improve treatment outcomes.

Institution

(Baghaei, Tabarsi, Marjani, Moniri, Masjedi) Clinical Tuberculosis and Epidemiology Research Center, National Research Institute of Tuberculosis and Lung Diseases (NRITLD), Shahid Beheshti University of Medical Sciences, Tehran, Iran, Islamic Republic of

Publisher

Taylor and Francis Ltd.

Emtree Heading

adult; age; article; cohort analysis; descriptive research; *diabetes mellitus/di [Diagnosis]; diabetic patient; fasting blood glucose; female; gender; glucose blood level; human; impaired fasting glucose; impaired glucose tolerance; Iran; *lung tuberculosis/di [Diagnosis]; major clinical study; male; number needed to screen; prevalence; prospective study; screening test; sensitivity and specificity; glucose/ec [Endogenous Compound]; hemoglobin A1c/ec [Endogenous Compound].

Link to the Ovid Full Text or citation:

Click here for full text options

5.

Prevalence, risk factors, management, and treatment outcomes of first-line antituberculous drug-induced liver injury: a prospective cohort study.

Sun Q., Zhang Q., Gu J., Sun W.-W., Wang P., Bai C., Xiao H.-P., Sha W.

Pharmacoepidemiology and Drug Safety. 25 (8) (pp 908-917), 2016. Date of Publication: 01 Aug 2016.

AN: 20160572220

Purpose: Antituberculosis drug-induced liver injury (ATDILI) is one of the most deleterious side effects associated with chemotherapy against tuberculosis (TB). In this study, our objective was to determine the incidence, risk factors, and management of ATDILI and analyze its impact on the treatment outcome in patients receiving standard anti-TB chemotherapy. Methods: A prospective cohort study of ATDILI prevalence was conducted in 938 enrolled patients of the 1426 TB cases in Shanghai from March 2011 to September 2012. Patients were followed up until February 2014. Univariate and multivariate logistic regression analyses were used to determine the risk factors of ATDILI. Successful therapeutic outcome, rates of drug resistance conversion, sputum smear/culture conversion, and lung cavity closure were analyzed. Results: Hepatitis B surface antigen/hepatitis B e antigen-positive hepatitis B carriers, complicated with systemic lupus erythematosus, albumin < 25 g/L, and chronic alcoholism were independent risk factors for ATDILI. Of the 121 cases with ATDILI (incidence rate of 12.9%), 84 (69.4%) used modified anti-TB therapy after recovery of liver function. Compared with the non-ATDILI group, patients with ATDILI exhibited remarkably decreased lung cavity closure rate (84.6% vs. 93.0%, P < 0.001) along with significantly reduced sputum smear/culture conversion rate (85.4% vs. 94.0%, P < 0.001). Conclusions: Our findings indicated that 12.9% patients developed ATDILI during standard anti-TB therapy, resulting in poor therapeutic outcome. Hepatitis B carriers with systemic lupus erythematosus, albumin < 25 g/L, and chronic alcoholism manifested increased risks for ATDILI. Copyright © 2016 John Wiley & Sons, Ltd.

Institution

(Sun, Zhang, Gu, Sun, Wang, Xiao, Sha) Clinic and Research Center of Tuberculosis, Shanghai Key Laboratory of Tuberculosis, Shanghai Pulmonary Hospital, Tongji University School of Medicine, Shanghai, China (Sun, Bai) Department of Respiratory and Critical Care Medicine, Changhai Hospital, the Second Military Medical University, Shanghai, China

Publisher

John Wiley and Sons Ltd (Southern Gate, Chichester, West Sussex PO19 8SQ, United Kingdom)

Emtree Heading

adult; age; albumin blood level; alcoholism; article; body mass; cohort analysis; convalescence; diabetes mellitus; extrapulmonary tuberculosis; female; follow up; gender; hemoglobin blood level; hepatitis B; human; hypertension; incidence; liver function; lung cavity; major clinical study; male; prevalence; priority journal; prognosis; prospective study; risk factor; smoking; sputum culture; systemic lupus erythematosus; *toxic hepatitis/ep [Epidemiology]; *toxic hepatitis/et [Etiology]; *toxic hepatitis/si [Side Effect]; treatment outcome; albumin/ec [Endogenous Compound]; hemoglobin/ec [Endogenous Compound]; hepatitis B surface antigen; hepatitis B(e) antigen; *tuberculostatic agent/ae [Adverse Drug Reaction].

Link to the Ovid Full Text or citation:

Click here for full text options

6.

Factors affecting patient outcome in primary cutaneous aspergillosis.

Tatara A.M., Mikos A.G., Kontoyiannis D.P.

Medicine (United States). 95 (26) (no pagination), 2016. Article Number: e3747. Date of Publication: 01 Jun 2016.

AN: 20160538979

Primary cutaneous aspergillosis (PCA) is an uncommon infection of the skin. There is a paucity of organized literature regarding this entity in regard to patient characteristics, associated Aspergillus species, and treatment modalities on outcome (disease recurrence, disease dissemination, and mortality). We reviewed all published reports of PCA from 1967 to 2015. Cases were deemed eligible if they included the following: patient baseline characteristics (age, sex, underlying condition), evidence of proven or probable PCA, primary treatment strategy, and outcome. We identified 130 eligible cases reported from 1967 to 2015. The patients were predominantly male (63.8%) with a mean age of 30.4 +/- 22.1 years. Rates of PCA recurrence, dissemination, and mortality were 10.8%, 18.5%, and 31.5%, respectively. In half of the cases, there was an association with a foreign body. Seven different Aspergillus species were reported to cause PCA. Systemic antifungal therapy without surgery was the most common form of therapy (60% of cases). Disease dissemination was more common in patients with underlying systemic conditions and occurred on average 41.4 days after PCA diagnosis (range of 3-120 days). In a multivariate linear regression model of mortality including only patients with immunosuppressive conditions, dissemination and human immunodeficiency virus/acquired immune deficiency syndrome were statistically significantly associated with increased mortality. Nearly one-third of patients with PCA die with the disease. Dissemination and host status are critical in patient outcome.

Institution

(Tatara, Mikos) Department of Bioengineering, Rice University, United States (Tatara, Kontoyiannis) Department of Infectious Diseases Infection Control and Employee Health, Unit 1416, University of Texas MD Anderson Cancer Center, 1515 Holcombe Boulevard, Houston, TX 77030, United States

Publisher

Lippincott Williams and Wilkins

Emtree Heading

acquired immune deficiency syndrome; adult; aged; agricultural worker; article; *aspergillosis/di [Diagnosis]; *aspergillosis/ep [Epidemiology]; *aspergillosis/pc [Prevention]; Aspergillus flavus; Aspergillus fumigatus; Aspergillus terreus; burn; computer assisted tomography; concurrent infection; cytomegalovirus infection; diabetes mellitus; disease association; early diagnosis; Escherichia coli infection; female; foreign body; human; Human immunodeficiency virus infection; immunocompromised patient; immunosuppressive treatment; major clinical study; male; mixed infection; morbidity; mortality rate; Mycobacterium tuberculosis; organ transplantation; outcome assessment; pharmaceutical care; Pneumocystis jiroveci; pneumocystosis; *primary cutaneous aspergillosis/di [Diagnosis]; *primary cutaneous aspergillosis/ep [Epidemiology]; *primary cutaneous aspergillosis/pc [Prevention]; priority journal; recurrent disease; risk factor; sputum culture; Staphylococcus capitis; Staphylococcus infection; systematic review; systemic therapy; thorax radiography; tuberculosis; antifungal agent; corticosteroid; galactomannan/ec [Endogenous Compound].

Link to the Ovid Full Text or citation:

Click here for full text options

7.

Clinical spectrum and outcome of pulmonary nocardiosis: 5-year experience.

Singh A., Chhina D., Soni R.K., Kakkar C., Sidhu U.S.

Lung India. 33 (4) (pp 398-403), 2016. Date of Publication: July-August 2016.

AN: 20160517919

Background: Pulmonary nocardiosis is a rare but a life-threatening infection caused by Nocardia spp. The diagnosis is often missed and delayed resulting in delay in appropriate treatment and thus higher mortality. Aim: In this study, we aim to evaluate the clinical spectrum and outcome of patients with pulmonary nocardiosis. Methods: A retrospective, 5-year (2009-2014) review of demographic profile, risk factors, clinical manifestations, imaging findings, treatment, and outcome of patients with pulmonary nocardiosis admitted to a tertiary care hospital. Results: The median age of the study subjects was 54 years (range, 16-76) and majority of them (75%) were males. The risk factors for pulmonary nocardiosis identified in our study were long-term steroid use (55.6%), chronic lung disease (52.8%), diabetes (27.8%), and solid-organ transplantation (22.2%). All the patients were symptomatic, and the most common symptoms were cough (91.7%), fever (78%), and expectoration (72%). Almost two-third of the patients were initially misdiagnosed and the alternative diagnosis included pulmonary tuberculosis (n = 7), community-acquired pneumonia (n = 5), lung abscess (n = 4), invasive fungal infection (n = 3), lung cancer (n = 2), and Wegener's granulomatosis (n = 2). The most common radiographic features were consolidation (77.8%) and nodules (56%). The mortality rate for indoor patients was 33% despite treatment. Higher mortality rate was observed among those who had brain abscess (100.0%), HIV positivity (100%), need for mechanical ventilation (87.5%), solid-organ transplantation (50%), and elderly (age > 60 years) patients (43%). Conclusion: The diagnosis of pulmonary nocardiosis is often missed and delayed resulting in delay in appropriate treatment and thus high mortality. A lower threshold for diagnosing pulmonary nocardiosis needs to be exercised, in chest symptomatic patients with underlying chronic lung diseases or systemic immunosuppression, for the early diagnosis, and treatment of this uncommon but potentially lethal disease. Despite treatment mortality remains high, especially in those with brain abscess, HIV positivity, need for mechanical ventilation, solid-organ transplantation, and elderly.

Institution

(Singh, Sidhu) Department of Pulmonary Medicine, Dayanand Medical College and Hospital, Ludhiana, Punjab, India (Chhina) Department of Microbiology, Dayanand Medical College and Hospital, Ludhiana, Punjab, India

(Soni) Department of Social and Preventive Medicine, Dayanand Medical College and Hospital, Ludhiana, Punjab, India

(Kakkar) Department of Radiology, Dayanand Medical College and Hospital, Ludhiana, Punjab, India

Publisher

Medknow Publications (B9, Kanara Business Centre, off Link Road, Ghatkopar (E), Mumbai 400 075, India)

Emtree Heading

abdominal abscess/su [Surgery]; abdominal pain; abnormal laboratory result; Acinetobacter infection/di [Diagnosis]; adolescent; adult; aged; antibiotic sensitivity; antibiotic therapy; article; artificial ventilation; brain abscess; chronic drug administration; chronic lung disease; clinical feature; community acquired pneumonia/di [Diagnosis]; corticosteroid therapy; coughing; crackle; diabetes mellitus; diagnostic error; diagnostic value; disease duration; dyspnea; empyema/su [Surgery]; female; fever; graft recipient; headache; hemoptysis; Hospital Mortality; human; Human immunodeficiency virus infected patient; Human immunodeficiency virus infection; human tissue; immunosuppressive treatment; Klebsiella pneumoniae infection/di [Diagnosis]; leukocytosis; lung abscess/di [Diagnosis]; lung cancer/di [Diagnosis]; *lung infection/di [Diagnosis]; *lung infection/et [Etiology]; *lung infection/th [Therapy]; *lung infection/dt [Drug Therapy]; lung lavage; lung tuberculosis/di [Diagnosis]; major clinical study; malaise; male; medical record review; mixed infection/di [Diagnosis]; mortality rate; neutrophilia; night sweat; *nocardiosis/di [Diagnosis]; *nocardiosis/et [Etiology]; *nocardiosis/th [Therapy]; *nocardiosis/dt [Drug Therapy]; organ transplantation; outcome assessment; pancytopenia/si [Side Effect]; Pseudomonas infection/di [Diagnosis]; *pulmonary nocardiosis/di [Diagnosis]; *pulmonary nocardiosis/et [Etiology]; *pulmonary nocardiosis/th [Therapy]; *pulmonary nocardiosis/dt [Drug Therapy]; rash/si [Side Effect]; retrospective study; risk factor; seizure; sensory dysfunction; skin abscess; skin ulcer; sputum analysis; symptom; systemic mycosis/di [Diagnosis]; tertiary care center; thorax pain; thorax radiography; thrombocytopenia/si [Side Effect]; thrombocytosis; tracheal aspiration procedure; transthoracic aspiration; treatment response; tuberculosis/di [Diagnosis]; uremia/si [Side Effect]; ventilated patient; Wegener granulomatosis/di [Diagnosis]; weight reduction; wheezing; amikacin/cb [Drug Combination]; amikacin/dt [Drug Therapy]; antibiotic agent/ae [Adverse Drug Reaction]; antibiotic agent/dt [Drug Therapy]; cefotaxime/cb [Drug Combination]; cefotaxime/dt [Drug Therapy]; corticosteroid/to [Drug Toxicity]; cotrimoxazole/cb [Drug Combination]; cotrimoxazole/dt [Drug Therapy]; imipenem/cb [Drug Combination]; imipenem/dt [Drug Therapy]; immunosuppressive agent/to [Drug Toxicity]; linezolid/cb [Drug Combination]; linezolid/dt [Drug Therapy]; minocycline/cb [Drug Combination]; minocycline/dt [Drug Therapy].

Link to the Ovid Full Text or citation:

Click here for full text options

8.

Health and ethical consequences of outsourcing pivotal clinical trials to Latin America: A cross-sectional, descriptive study.

Homedes N., Ugalde A.

PLoS ONE. 11 (6) (no pagination), 2016. Article Number: e0157756. Date of Publication: June 2016.

AN: 20160489947

Introduction: The implications of conducting clinical trials in low and middle income countries on the financial accessibility and safety of the pharmaceutical products available in those markets have not been studied. Regulatory practices and ethical declarations lead to the commercialization of the new products, referred to as New Molecular Entities (NMEs), in the countries where tested as soon as they are approved in high surveillance countries. Patients and patients' associations use the Latin American courts to access new and expensive treatments, regardless of their safety profile and therapeutic value. Design and Objectives: Cross-sectional, descriptive study. To determine the therapeutic value and safety profile of the NMEs approved by the Food and Drug Administration (FDA) in 2011 and 2012 that had been tested in Latin America, and the implications of their market approval for the pharmaceutical budgets in the countries where tested. Setting: Latin America. Measures: To assess the therapeutic value and safety of the NMEs commercialized in the different countries we used f independent drug bulletins. The prices of the NMEs for the consumers were obtained from the pharmaceutical price observatories of the countries were the medicines had been tested. If the price was not available in the observatories, it was obtained from pharmaceutical distributors. We used the countries' minimum wage and per capita income to calculate the financial accessibility of a course of treatment with the NMEs. Results: We found that 33 NMEs approved by the FDA in 2011 and 2012 have been tested in Latin America. Of these, 26 had been evaluated by independent drug bulletins and only five were found to add some value to a subset of patients and had significant side-effects. The pharmaceutical prices were very high, varied widely across countries and were unrelated to the countries' income per capita or minimum wage. Conclusion: The implementation of clinical trials in Latin America results in the commercialization of medicines with questionable safety profiles and limited therapeutic value, putting patients at risk and causing budgetary strains in pharmaceutical budgets.

Institution

(Homedes) Management Policy and Community Health, School of Public Health, University of Texas, El Paso, TX, United States (Homedes) Department of International Health, Georgetown University, Washington, DC, United States

(Ugalde) Department of Sociology, University of Texas, Austin, TX, United States

Publisher

Public Library of Science

Emtree Heading

acute coronary syndrome/dt [Drug Therapy]; age related macular degeneration/dt [Drug Therapy]; Argentina; article; atrial fibrillation/dt [Drug Therapy]; breast cancer/dt [Drug Therapy]; castration resistant prostate cancer/dt [Drug Therapy]; Chile; chronic hepatitis C/dt [Drug Therapy]; chronic myeloid leukemia/dt [Drug Therapy]; chronic obstructive lung disease/dt [Drug Therapy]; Colombia; controlled clinical trial; controlled study; cross sectional study; Cushing syndrome/dt [Drug Therapy]; deep vein thrombosis/dt [Drug Therapy]; drug approval; drug cost; drug efficacy; drug safety; *financial management; government regulation; human; Human immunodeficiency virus infection/dt [Drug Therapy]; kidney graft rejection/dt [Drug Therapy]; lung embolism/dt [Drug Therapy]; *medical ethics; melanoma/dt [Drug Therapy]; metastatic colorectal cancer/dt [Drug Therapy]; multidrug resistant tuberculosis/dt [Drug Therapy]; non insulin dependent diabetes mellitus/dt [Drug Therapy]; non small cell lung cancer/dt [Drug Therapy]; outcome assessment; overall survival; Philadelphia 1 chromosome/dt [Drug Therapy]; progression free survival; risk assessment; risk benefit analysis; seizure/dt [Drug Therapy]; *South and Central America; systemic lupus erythematosus/dt [Drug Therapy]; thyroid medullary carcinoma/dt [Drug Therapy]; aclidinium bromide/ct [Clinical Trial]; aclidinium bromide/pe [Pharmacoeconomics]; aclidinium bromide/dt [Drug Therapy]; aflibercept/ct [Clinical Trial]; aflibercept/pe [Pharmacoeconomics]; aflibercept/dt [Drug Therapy]; apixaban/ct [Clinical Trial]; apixaban/pe [Pharmacoeconomics]; apixaban/dt [Drug Therapy]; bedaquiline/ct [Clinical Trial]; bedaquiline/pe [Pharmacoeconomics]; bedaquiline/dt [Drug Therapy]; belatacept/ct [Clinical Trial]; belatacept/pe [Pharmacoeconomics]; belatacept/dt [Drug Therapy]; belimumab/ct [Clinical Trial]; belimumab/pe [Pharmacoeconomics]; belimumab/dt [Drug Therapy]; bosutinib/ct [Clinical Trial]; bosutinib/pe [Pharmacoeconomics]; bosutinib/dt [Drug Therapy]; cabozantinib/ct [Clinical Trial]; cabozantinib/pe [Pharmacoeconomics]; cabozantinib/dt [Drug Therapy]; cobicistat/ct [Clinical Trial]; cobicistat/pe [Pharmacoeconomics]; cobicistat/dt [Drug Therapy]; crizotinib/ct [Clinical Trial]; crizotinib/pe [Pharmacoeconomics]; crizotinib/dt [Drug Therapy]; elvitegravir/ct [Clinical Trial]; elvitegravir/pe [Pharmacoeconomics]; elvitegravir/dt [Drug Therapy]; emtricitabine/ct [Clinical Trial]; emtricitabine/pe [Pharmacoeconomics]; emtricitabine/dt [Drug Therapy]; enzalutamide/ct [Clinical Trial]; enzalutamide/pe [Pharmacoeconomics]; enzalutamide/dt [Drug Therapy]; indacaterol/ct [Clinical Trial]; indacaterol/pe [Pharmacoeconomics]; indacaterol/dt [Drug Therapy]; ipilimumab/ct [Clinical Trial]; ipilimumab/pe [Pharmacoeconomics]; ipilimumab/dt [Drug Therapy]; linagliptin/ct [Clinical Trial]; linagliptin/pe [Pharmacoeconomics]; linagliptin/dt [Drug Therapy]; pasireotide/ct [Clinical Trial]; pasireotide/pe [Pharmacoeconomics]; pasireotide/dt [Drug Therapy]; perampanel/ct [Clinical Trial]; perampanel/pe [Pharmacoeconomics]; perampanel/dt [Drug Therapy]; pertuzumab/ct [Clinical Trial]; pertuzumab/pe [Pharmacoeconomics]; pertuzumab/dt [Drug Therapy]; regorafenib/ct [Clinical Trial]; regorafenib/pe [Pharmacoeconomics]; regorafenib/dt [Drug Therapy]; rilpivirine/ct [Clinical Trial]; rilpivirine/pe [Pharmacoeconomics]; rilpivirine/dt [Drug Therapy]; rivaroxaban/ct [Clinical Trial]; rivaroxaban/pe [Pharmacoeconomics]; rivaroxaban/dt [Drug Therapy]; roflumilast/ct [Clinical Trial]; roflumilast/pe [Pharmacoeconomics]; roflumilast/dt [Drug Therapy]; telaprevir/ct [Clinical Trial]; telaprevir/pe [Pharmacoeconomics]; telaprevir/dt [Drug Therapy]; tenofovir/ct [Clinical Trial]; tenofovir/pe [Pharmacoeconomics]; tenofovir/dt [Drug Therapy]; ticagrelor/ct [Clinical Trial]; ticagrelor/pe [Pharmacoeconomics]; ticagrelor/dt [Drug Therapy]; vandetanib/ct [Clinical Trial]; vandetanib/pe [Pharmacoeconomics]; vandetanib/dt [Drug Therapy].

Drug Trade Names and Manufacturers

Almirall, Astellas, Astra Zeneca, Bayer, Bms, Boehringer, Discovery, Eisai, Exelixis, Forest, Genentech, Genzyme, Gilead, Glaxo SmithKline, Hoffmann La Roche, Janssen, Labaz, Novartis, Pfizer, Rafo, Sobi, Takeda, Teva, Vertex, arcapta neohaler: Novartis, aubagio: Genzyme, benlysta: Glaxo SmithKline, bosulif: Pfizer, brilinta: Astra Zeneca, caprelsa: Astra Zeneca, cometriq: Exelixis, cometriq: Sobi, daliresp: Forest, daxas: Takeda, edarbi: Takeda, edurant: Janssen, eklaire genuari: Almirall, elelyso: Pfizer, elicuis: Bms, eliquis: Bms, eylea: Bayer, eylia: Bayer, fycompa: Eisai, granix: Teva, incivek: Janssen, incivek: Vertex, inlyta: Pfizer, neutroval: Teva, nulojix: Bms, onbrize: Novartis, perjeta: Genentech, perjeta: Hoffmann La Roche, potiga: Glaxo SmithKline, signifor: Novartis, sirturo: Janssen, stivarga: Bayer, stribild: Gilead, surfaxin: Discovery, tradjenta: Boehringer, tudorza pressair: Forest, uplyso: Pfizer, xalkori: Pfizer, xarelto: Bayer, xarelto: Janssen, xeljan: Pfizer, xtandi: Rafo, xtandi: Astellas, yerboy: Bms, yervoy: Bms, zaltrap: Labaz

Link to the Ovid Full Text or citation:

Click here for full text options

9.

Alcohol: Taking a population perspective.

Gilmore W., Chikritzhs T., Stockwell T., Jernigan D., Naimi T., Gilmore I.

Nature Reviews Gastroenterology and Hepatology. 13 (7) (pp 426-434), 2016. Date of Publication: 01 Jul 2016.

AN: 20160382888

Alcohol consumption is a global phenomenon, as is the resultant health, social and economic harm. The nature of these harms varies with different drinking patterns and with the societal and political responses to the burden of harm; nevertheless, alcohol-related chronic diseases have a major effect on health. Strong evidence exists for the effectiveness of different strategies to minimize this damage and those policies that target price, availability and marketing of alcohol come out best, whereas those using education and information are much less effective. However, these policies can be portrayed as anti-libertarian and so viewing them in the context of alcohol-related harm to those other than the drinker, such as the most vulnerable in society, is important. When this strategy is successful, as in Scotland, it has been possible to pass strong and effective legislation, such as for a minimum unit price for alcohol.

Institution

(Gilmore, Chikritzhs) National Drug Research Institute, Faculty of Health Sciences, Curtin University, GPO Box U1987, Perth 6845, Australia (Stockwell) Centre for Addictions Research of British Columbia, Department of Psychology, University of Victoria, PO Box 1700 STN CSC, Victoria, BC V8W 2Y2, Canada

(Jernigan) Johns Hopkins Bloomberg School of Public Health, Johns Hopkins University, 615 North Wolfe Street, Baltimore, MD 21205, United States

(Naimi) Section of General Internal Medicine, Boston University Medical Center, Boston University, 801 Massachusetts Avenue, Boston, MA 02118, United States

(Gilmore) University of Liverpool, Liverpool Science Park, 131 Mount Pleasant, Liverpool L3 5TF, United Kingdom

Publisher

Nature Publishing Group (Houndmills, Basingstoke, Hampshire RG21 6XS, United Kingdom)

Emtree Heading

advertising; *alcohol consumption; alcohol intoxication; alcoholism; automutilation; cardiovascular disease; cost of living; depression; diabetes mellitus; education; epilepsy; fetal alcohol syndrome; gastrointestinal disease; government; health care policy; health hazard; human; Human immunodeficiency virus infection; information; liver disease; marketing; neoplasm; outcome assessment; priority journal; public health; review; risk factor; Scotland; socioeconomics; tuberculosis; *alcohol/to [Drug Toxicity].

Link to the Ovid Full Text or citation:

Click here for full text options

10.

The influence of smoking on pulmonary tuberculosis in diabetic and non-diabetic patients.

Bai K.-J., Lee J.-J., Chien S.-T., Suk C.-W., Chiang C.-Y.

PLoS ONE. 11 (6) (no pagination), 2016. Article Number: e0156677. Date of Publication: 01 Jun 2016.

AN: 20160457069

Background: Both smoking and diabetes can increase the risk and influence the manifestations and outcomes of tuberculosis (TB). It is not clear whether the influence of smoking on pulmonary TB differs between non-diabetic and diabetic patients. Herein, we assessed the manifestations and outcomes of TB in relation to smoking in both diabetic and non-diabetic TB patients. Methodology/Principal Findings: All diabetic culture-positive pulmonary TB patients notified from 2005-2010 at three teaching hospitals in Taiwan were enrolled. A culture-positive pulmonary TB patient without DM who was notified to the health authority immediately prior to each diabetic TB patient was selected for comparison. The 972 patients in this study cohort included 365 (37.6%) nondiabetic non-smokers, 149 (15.3%) non-diabetic smokers, 284 (29.2%) diabetic non-smokers, and 174 (17.9%) diabetic smokers. The adjusted relative risk of a pretreatment positive smear for a smoker compared with a non-smoker was 2.19 (95% CI 1.38-3.47) in non-diabetic patients and 2.23 (95% CI 1.29-3.87) in diabetic culture-positive pulmonary TB patients. The adjusted relative risk for a positive smear among diabetic smokers was 5.61 (95% CI 3.35-9.41) compared with non-diabetic non-smokers. Smoking was significantly associated with an increased frequency of bilateral lung parenchyma involvement (AdjOR 1.84, 95% CI 1.16-2.93), far-advanced pulmonary TB (AdjOR 1.91, 95% CI 1.04-3.50), cavitary lesions (AdjOR 2.03, 95% CI 1.29-3.20), and unfavorable outcomes of TB (AdjOR 2.35, 95% CI 1.02-5.41) in non-diabetic patients. However, smoking was not associated with cavitary lung parenchyma lesions regarding the location, number or size of the cavity in diabetic TB patients. Conclusions/Significance: Smoking and diabetes have joint effects on a pretreatment positive smear. Diabetic smokers had more than a 5-fold increased risk of a pretreatment positive smear than did non-diabetic non-smokers, indicating remarkable joint effects of diabetes and smoking on the risk of TB transmission.

Institution

(Bai, Suk, Chiang) Division of Pulmonary Medicine, Department of Internal Medicine, Wan Fang Hospital, Taipei Medical University, Taipei, Taiwan (Republic of China) (Bai) School of Respiratory Therapy, College of Medicine, Taipei Medical University, Taipei, Taiwan (Republic of China)

(Lee) Department of Internal Medicine, Buddhist Tzu Chi General Hospital, Tzu Chi University, Hualien, Taiwan (Republic of China)

(Chien) Chest Hospital, Department of Health and Welfare, Tainan, Taiwan (Republic of China)

(Chiang) Department of Internal Medicine, School of Medicine, College of Medicine, Taipei Medical University, Taipei, Taiwan (Republic of China)

(Chiang) International Union Against Tuberculosis and Lung Disease, Paris, France

Publisher

Public Library of Science

Emtree Heading

adult; aged; article; cohort analysis; controlled study; *diabetic patient; disease association; disease course; disease severity; female; human; *lung tuberculosis/di [Diagnosis]; major clinical study; male; outcome assessment; *smoking; sputum culture; sputum smear; Taiwan.

Link to the Ovid Full Text or citation:

Click here for full text options

11.

Diabetes mellitus in patients with pulmonary tuberculosis in an aging population in Shanghai, China: Prevalence, clinical characteristics and outcomes.

Wu Z., Guo J., Huang Y., Cai E., Zhang X., Pan Q., Yuan Z., Shen X.

Journal of Diabetes and its Complications. 30 (2) (pp 237-241), 2016. Date of Publication: 01 Mar 2016.

AN: 20160220922

Aims To determine the prevalence of diabetes mellitus among pulmonary tuberculosis patients and the difference of clinical characteristics and outcomes between pulmonary tuberculosis patients with and without diabetes mellitus in an aging population in Shanghai, China. Methods This is a retrospective population-based study. 201 newly diagnosed pulmonary tuberculosis patients in Changning District, Shanghai during 2007-2008 were included. Clinical characteristics and outcomes were collected. Determination of diabetes mellitus was based on the medical records before pulmonary tuberculosis was diagnosed. Results The prevalence of diabetes mellitus among pulmonary tuberculosis patients was 19.9% (40/201). Pulmonary tuberculosis patients with diabetes mellitus were more likely to be old (> 50, OR = 5.23, 95% CI = 2.07-13.25), to have pulmonary cavities (OR = 3.02, 95% CI = 1.31-6.98), to be sputum smear positive (OR = 2.90, 95% CI = 1.12-7.51), and to have extension of anti-tuberculosis treatment duration (OR = 2.68, 95% CI 1.17-6.14). Besides, they had a higher 2nd month sputum smear positive proportion (OR = 2.97, 95% CI 1.22-7.22) and a higher 5-year recurrence rate (OR = 5.87, 95% CI 1.26-27.40). Conclusions High prevalence, severe clinical characteristics and poor outcomes of pulmonary tuberculosis patients with diabetes mellitus highlight the necessity of early bi-directional screening and co-management of these two diseases in Shanghai, China.

Institution

(Wu, Guo, Pan, Yuan, Shen) Department of Tuberculosis Control, Shanghai Municipal Center for Disease Control and Prevention, Shanghai, China (Huang, Cai, Zhang) Shanghai Changning Center for Disease Control and Prevention, Shanghai, China

Publisher

Elsevier Inc.

Emtree Heading

adolescent; adult; age; aged; antibiotic therapy; article; China; controlled study; *diabetes mellitus/ep [Epidemiology]; female; human; lung cavity; *lung tuberculosis/dt [Drug Therapy]; major clinical study; male; outcome assessment; population research; prevalence; priority journal; recurrence risk; recurrent infection; retrospective study; sputum smear; treatment duration; ethambutol/cb [Drug Combination]; ethambutol/dt [Drug Therapy]; isoniazid/cb [Drug Combination]; isoniazid/dt [Drug Therapy]; pyrazinamide/cb [Drug Combination]; pyrazinamide/dt [Drug Therapy]; rifampicin/cb [Drug Combination]; rifampicin/dt [Drug Therapy]; streptomycin/cb [Drug Combination]; streptomycin/dt [Drug Therapy].

Link to the Ovid Full Text or citation:

Click here for full text options

12.

Treatment effect analysis of the standard regimen and the optimized regimen for retreatment pulmonary tuberculosis complicated with diabetes.

Du J., Gao W., Ma Y., Zhong Q., Liang X., Fu Y., Ji B., Xie L., Huang X., Ge Q., Han X., Tian X., Shu W., Li L.

Zhonghua jie he he hu xi za zhi = Zhonghua jiehe he huxi zazhi = Chinese journal of tuberculosis and respiratory diseases. 38 (12) (pp 886-891), 2015. Date of Publication: 01 Dec 2015.

AN: 26850888

OBJECTIVE: To analyze the therapeutic effects of the standard regimen and the optimized regimen in retreatment pulmonary tuberculosis complicated with diabetes mellitus (DM). METHODS: In a multi-center cohort study, patients with smear positive retreatment pulmonary tuberculosis (TB) with DM and those without DM [excluding multi-drug resistance (MDR), extensively drug-resistant (XDR) and non-tuberculosis Mycobacterium pulmonary disease(NTM)] were enrolled. There were a total of 178 cases, including 60 smear positive retreatment TB patients with DM and 118 without DM, who were randomly divided into 4 groups: Optimized group 1 [individualized treatment in 30 DM cases, 29 males, age (48 +/- 11)], retreatment group 1 [standard retreatment regimen in 30 DM cases, 28 males, age(48 +/- 10)], Optimized group 2[individual regimen in 57 non-DM cases, 37 males, age (41 +/- 14)], and retreatment group 2 [standard retreatment regimen in 61 non-DM cases, 49 males, age (43 +/- 13)]. Patients in the optimized group were treated with optimized individualized regimen based on DST result, with 3-4 sensitive drugs in the regimen, while those in the retreatment group were treated with national standard retreatment regimen. The therapeutic effect of different groups were recorded and the related factors of treatment outcome were analyzed with Chi-square test and multi-factor analysis.

RESULTS: The treatment success rates of the optimized group 1 and the retreatment group 1 were 83.3%(25/30) and 60.0%(18/30), respectively, and the difference was statistically significant (chi(2)=4.02, P=0.045<0.05). The treatment failure rate of the optimized group 1 (6.7%, 2/30) and the retreatment group 1(30.0%, 9/30) was statistically different (chi(2)=5.46, P=0.02<0.05). The outcome difference between the optimized group 2 and the retreatment group 2 showed no statistical significance. Multi-factor analysis showed that treatment regimen, DM, gender and drug resistance were the significant factors related with treatment outcome. The probability of treatment success using the individualized treatment regimen was 2.7 times higher than that using the standard regimen (P=0.025). The risk of treatment failure of the drug resistance cases was 2.8 times higher than that of the drug sensitive cases (P=0.038). The probability of treatment success in DM cases was 0.4 times that in non-DM cases (P<0.05).

CONCLUSION: The outcome of the optimized regimen group was better than that of the standard regimen group, and retreatment TB patients complicated with DM faced a higher risk of treatment failure, which should receive more attention.

Institution

(Du, Gao, Ma, Zhong, Liang, Fu, Ji, Xie, Huang, Ge, Han, Tian, Shu) Beijing Chest Hospital, Capital Medical University, Beijing Tuberculosis and Thoracic Tumor Research Institute, Beijing 101149, China	(Li) Email:

Emtree Heading

adult; cohort analysis; controlled study; diabetes mellitus; female; human; *lung tuberculosis; male; middle aged; randomized controlled trial; retreatment; treatment outcome.

Link to the Ovid Full Text or citation:

Click here for full text options

13.

Differential proteome analysis of peripheral blood mononuclear cells from patients of concurrent active tuberculosis and type 2 diabetes mellitus.

Jyoti K., Sanjay B.K., Indu V., Sadhna S.

Indian Journal of Clinical Biochemistry. Conference: 42nd National Conference of Association of Clinical Biochemists of India, ACBICON 2015 Chandigarh India. Conference Start: 20151125 Conference End: 20151128. Conference Publication: (var.pagings). 30 (pp S122), 2015. Date of Publication: December 2015.

AN: 72285348

Diabetes almost triples the risk of developing active tuberculosis and is also a risk factor for its adverse treatment outcomes. Eight of the ten countries with the highest incidence of diabetes mellitus worldwide are also classified as high-burden countries for tuberculosis by the World Health Organization. Though decreased immunity in diabetic patients is thought to be the cause for this cooccurrence, pathophysiology of association of tuberculosis and diabetes mellitus is poorly understood. Proteomics is an important tool which can be applied to understand the pathophysiology of concurrent cases of tuberculosis and diabetes. The objective of the study was to analyze the differential proteome of PBMCs in concurrent active tuberculosis and type 2 diabetes patients. Forty one candidates were enrolled in the study and were categorized in four different groups: i) patients with pulmonary naive tuberculosis, ii) associated pulmonary naive tuberculosis and type 2 diabetes, iii) only type 2 diabetes and iv) healthy controls. PBMC were isolated and subjected to proteome analysis 2DE-MALDI-MS/MS. Proteins were identified by MASCOT and specific functions for the proteins were correlated with SwissProt database. In the study a total of 272 +/- 29 spots were obtained on the 2DE gel. Spot analysis was done by Image Master Pentium 6 software. Two or more fold change in spot area was considered significant for alteration in protein expression. A total of 24 proteins were found to have altered expression among the groups. Out of which Vimentin is highly expressed on M. tuberculosis infected human monocytes and is involved in binding to NKp46 receptors of NK cells. Another protein Maff-interacting protein that acts as transcription factor is involved in upregulated production of IL10 Hyaluronan synthase 1 in MDMs infected with M. tuberculosis which favours bacterial growth. The study concludes that involvement of these differentially expressed molecules in various cellular networks can be predicted which might be linked to the co pathogenesis of tuberculosis and diabetes.

Institution

(Jyoti, Indu, Sadhna) Department of Biochemistry, Chandigarh, India (Sanjay) Department of Endocrinology, Institute of Medical Education and Research, Chandigarh, India

Publisher

Springer India

Emtree Heading

*peripheral blood mononuclear cell; *patient; *human; *tuberculosis; *non insulin dependent diabetes mellitus; *India; diabetes mellitus; pathophysiology; diabetic patient; protein expression; computer program; data base; natural killer cell; SWISS-PROT; risk factor; proteomics; monocyte; immunity; world health organization; bacterial growth; treatment outcome; pathogenesis; risk; *proteome; protein; transcription factor MafF; transcription factor; natural cytotoxicity triggering receptor 1; synthetase; hyaluronic acid; vimentin.

Link to the Ovid Full Text or citation:

Click here for full text options

14.

Glycated hemoglobin screening identifies patients admitted for retreatment of tuberculosis at risk for diabetes in Tanzania.

Sariko M.L., Mpagama S.G., Gratz J., Kisonga R., Saidi Q., Kibiki G.S., Heysell S.K.

Journal of Infection in Developing Countries. 10 (4) (pp 423-426), 2016. Date of Publication: April 2016.

AN: 20160342821

Introduction: World Health Organization recommendations of bidirectional screening for tuberculosis (TB) and diabetes have been met with varying levels of uptake by national TB programs in resource-limited settings. Methodology: Kibong'oto Infectious Diseases Hospital (KIDH) is a referral hospital for TB from northern Tanzania, and the national referral hospital for multidrug-resistant (MDR)-TB. Glycated hemoglobin (HgbA1c) testing was done on patients admitted to KIDH for newly diagnosed TB, retreatment TB, and MDR-TB, to determine the point prevalence of diabetes (HgbA1c > 6.5%) and prediabetes (HgbA1c 5.7%-6.4%). Results: Of 148 patients hospitalized at KIDH over a single week, 59 (38%) had no prior TB treatment, 22 (15%) were retreatment cases, and 69 (47%) had MDR-TB. Only 3 (2%) had a known history of diabetes. A total of 144 (97%) had successful screening, of which 110 (77%) had an HgbA1c < 5.6%, 28 (19%) had > 5.7 < 6.5, and 6 (4%) had > 6.5. Comparing subjects with prediabetes or diabetes to those with normal A1c levels, retreatment patients were significantly more likely to have a A1c > 5.7% (odds ratio: 3.2, 95% CI: 1.2-9.0; p = 0.02) compared to those without prior TB treatment. No retreatment case was a known diabetic, thus the number needed to screen to diagnose one new case of diabetes among retreatment cases was 11. Conclusions: Diabetes prevalence by HgbA1c was less common than expected, but higher HgA1c values were significantly more frequent among retreatment cases, allowing for a rational, resource-conscious screening approach.

Institution

(Sariko, Saidi, Kibiki) Kilimanjaro Clinical Research Institute, Moshi, Tanzania (Mpagama, Kisonga) Kibong'oto Infectious Diseases Hospital, Kilimanjaro, Sanya Juu, Tanzania

(Gratz, Heysell) Division of Infectious Diseases and International Health, University of Virginia, Charlottesville, VA, United States

Publisher

Journal of Infection in Developing Countries

Emtree Heading

adult; article; blood analysis; body mass; controlled study; cross sectional study; *diabetes mellitus/di [Diagnosis]; drug treatment failure; female; high risk population; human; impaired glucose tolerance; major clinical study; male; *multidrug resistant tuberculosis/di [Diagnosis]; *multidrug resistant tuberculosis/dr [Drug Resistance]; point of care testing; prevalence; retreatment; Tanzania; *glycosylated hemoglobin/ec [Endogenous Compound]; hemoglobin A1c/ec [Endogenous Compound].

Link to the Ovid Full Text or citation:

Click here for full text options

15.

Global urine metabolomics in patients treated with first-line tuberculosis drugs and identification of a novel metabolite of ethambutol.

Das M.K., Arya R., Debnath S., Debnath R., Lodh A., Bishwal S.C., Das A., Nanda R.K.

Antimicrobial Agents and Chemotherapy. 60 (4) (pp 2257-2264), 2016. Date of Publication: April 2016.

AN: 20160308876

Population level variation of drug metabolism phenotype (DMP) has great implications in treatment outcome, drug-related side effects, and resistance development. In this study, we used a gas chromatography-time of flight-mass spectrometry (GC-TOF-MS)-based untargeted urine metabolomics approach to understand the DMP of a tuberculosis (TB) patient cohort (n = 20) from Tripura, a state in the northeastern part of India. Urine samples collected at different postdose time points (2 h, 6 h, 12 h, 24 h, 36 h, and 48 h) from these newly diagnosed TB patients receiving first-line anti-TB drugs were analyzed, and we have successfully detected three of the four first-line drugs, viz., isoniazid (INH), ethambutol (ETB), andpyrazinamide (PZA). The majority of their known metabolites, acetyl-isoniazid (AcINH), isonicotinic acid (INA), isonicotinuric acid (INTA), 2,2'-(ethylenediimino)-dibutyric acid (EDBA), 5-hydroxypyrazinamide (5OH-PZA), pyrazinoic acid (POA), and 5-hydroxypyrazinoic acid (5OH-POA), were also detected. Analyzing the variation in abundances of drugs and their known metabolites and calculating the metabolic ratios in these samples, we offer comprehensive DMP information on this small patient cohort that represents Tripura, India. The majority (75%) of these patients are found to be slow acetylators of INH. The average metabolic ratios of POA/PZA and 5OH-POA/POA are 3.16 +/- 3.03 and 6.09 +/- 6.15, respectively. Employing correlation analysis of the metabolomics metadata and a manual prediction of drug catabolism, we have proposed 2-aminobutyric acid (AABA) as a novel metabolite of ETB. These observations indicate the usefulness of GC-MS-based metabolomics to characterize the DMP at a population level and also to identify novel drug metabolites.

Institution

(Das, Arya, Bishwal, Nanda) Immunology Group, International Centre for Genetic Engineering and Biotechnology (ICGEB), New Delhi, India (Debnath, Debnath, Lodh, Das) Department of Tuberculosis and Respiratory Disease, Agartala Government Medical College, Agartala, Tripura, India

(Bishwal) School of Life Sciences, Sambalpur University, Sambalpur, Odisha, India

Publisher

American Society for Microbiology

Emtree Heading

adult; aged; article; aspartate aminotransferase blood level; body mass; chemical structure; chronic kidney disease; clinical article; cohort analysis; computer assisted tomography; controlled study; diabetes mellitus; drug megadose; drug metabolism; female; gas chromatography; human; *lung tuberculosis/dt [Drug Therapy]; male; metabolic ratio; *metabolomics; obesity; priority journal; sputum cytodiagnosis; time of flight mass spectrometry; underweight; *urinalysis; very elderly; 2 aminobutyric acid; 2,2' (ethylenediimino)dibutyric acid; 5 hydroxypyrazinamide; 5 hydroxypyrazinoic acid; acetylisoniazid; alanine aminotransferase/ec [Endogenous Compound]; alkaline phosphatase; *ethambutol/dt [Drug Therapy]; *isoniazid/dt [Drug Therapy]; isonicotinic acid; isonicotinuric acid; *pyrazinamide/dt [Drug Therapy]; pyrazinoic acid; unclassified drug.

Link to the Ovid Full Text or citation:

Click here for full text options

16.

Diabetes mellitus is associated with increased mortality during tuberculosis treatment: A prospective cohort study among tuberculosis patients in South-Eastern Amahra Region, Ethiopia.

Workneh M.H., Bjune G.A., Yimer S.A.

Infectious Diseases of Poverty. 5 (1) (no pagination), 2016. Article Number: 22. Date of Publication: March 21, 2016.

AN: 20160294293

Background: There is growing evidence suggesting that diabetes mellitus (DM) affects disease presentation and treatment outcome in tuberculosis (TB) patients. This study aimed at investigating the role of DM on clinical presentations and treatment outcomes among newly diagnosed TB patients. Methods: A prospective cohort study was conducted in South-Eastern Amhara Region, Ethiopia from September 2013 till March 2015. Study subjects were consecutively recruited from 44 randomly selected health facilities in the study area. Participants were categorized into two patient groups, namely, patients with TB and DM (TBDM) and TB patients without DM (TBNDM). Findings on clinical presentations and treatment outcomes were compared between the two patient groups. Cox proportional hazard regression analysis was applied to identify factors associated with death. Results: Out of 1314 TB patients enrolled in the study, 109 (8.3 %) had coexisting DM. TBDM comorbidity [adjusted hazard ratio (AHR) 3.96; 95 % confidence interval (C.I.) (1.76-8.89)], and TB coinfection with human immunodeficiency virus (HIV) [AHR 2.59; 95 % C.I. (1.21-5.59)] were associated with increased death. TBDM and TBNDM patients did not show significant difference in clinical symptoms at baseline and during anti-TB treatment period. However, at the 2nd month of treatment, TBDM patients were more symptomatic compared to patients in the TBNDM group. Conclusions: The study showed that DM is associated with increased death during TB treatment. DM has no association with clinical presentation of TB except at the end of the intensive phase treatment. Routine screening of TB patients for DM is recommended for early diagnosis and treatment of patients with TBDM comorbidity.

Institution

(Workneh, Bjune, Yimer) University of Oslo, Institute of Health and Society, Faculty of Medicine, Oslo, Norway (Workneh, Yimer) Amhara Regional State Health Bureau, Bahir-Dar, Ethiopia

(Yimer) Oslo University Hospital, Department of Microbiology, Oslo, Norway

(Yimer) Norwegian Institute of Public Health, Department of Bacteriology and Immunology, Oslo, Norway

Publisher

BioMed Central Ltd.

Emtree Heading

adolescent; adult; aged; article; comorbidity; *diabetes mellitus; disease association; Ethiopia; female; human; major clinical study; male; mortality; prevalence; priority journal; prospective study; treatment outcome; *tuberculosis.

Link to the Ovid Full Text or citation:

Click here for full text options

17.

Changing Trends in Complications and Mortality Rates among US Youth and Young Adults with HIV Infection in the Era of Combination Antiretroviral Therapy.

Mirani G., Williams P.L., Chernoff M., Abzug M.J., Levin M.J., Seage G.R., Oleske J.M., Purswani M.U., Hazra R., Traite S., Zimmer B., Van Dyke R.B.

Clinical Infectious Diseases. 61 (12) (pp 1850-1861), 2015. Date of Publication: 15 Dec 2015.

AN: 20160250451

Background. Combination antiretroviral therapy (cART) has resulted in a dramatic decrease in human immunodeficiency virus (HIV)-related opportunistic infections and deaths in US youth, but both continue to occur. Methods. We estimated the incidence of complications and deaths in IMPAACT P1074, a long-term US-based prospective multicenter cohort study conducted from April 2008 to June 2014. Incidence rates of selected diagnoses and trends over time were compared with those from a previous observational cohort study, P219C (2004-2007). Causes of death and relevant demographic and clinical features were reviewed. Results. Among 1201 HIV-infected youth in P1074 (87% perinatally infected; mean [standard deviation] age at last chart review, 20.9 [5.4] years), psychiatric and neurodevelopmental disorders, asthma, pneumonia, and genital tract infections were among the most common comorbid conditions. Compared with findings in P219C, conditions with significantly increased incidence included substance or alcohol abuse, latent tuberculosis, diabetes mellitus, atypical mycobacterial infections, vitamin D deficiency or metabolic bone disorders, anxiety disorders, and fractures; the incidence of pneumonia decreased significantly. Twenty-eight deaths occurred, yielding a standardized mortality rate 31.5 times that of the US population. Those who died were older, less likely to be receiving cART, and had lower CD4 cell counts and higher viral loads. Most deaths (86%) were due to HIV-related medical conditions. Conclusions. Opportunistic infections and deaths are less common among HIV-infected youth in the US in the cART era, but the mortality rate remains elevated. Deaths were associated with poor HIV control and older age. Emerging complications, such as psychiatric, inflammatory, metabolic, and genital tract diseases, need to be addressed.

Institution

(Mirani, Van Dyke) Tulane University School of Medicine, New Orleans, LA, United States (Williams, Chernoff, Traite) Center for Biostatistics in AIDS Research, United States

(Williams) Department of Biostatistics, Harvard T. H. Chan School of Public Health, 655 Huntington Ave, Boston, MA 02115, United States

(Williams, Seage) Department of Epidemiology, Harvard T. H. Chan School of Public Health, Boston, MA, United States

(Abzug, Levin) University of Colorado, School of Medicine and Children's Hospital Colorado, Aurora, United States

(Oleske) Rutgers New Jersey Medical School, Newark, United States

(Purswani) Albert Einstein College of Medicine, Bronx-Lebanon Hospital Center, Bronx, United States

(Zimmer) Frontier Science Technology and Research Foundation, Amherst, NY, United States

(Hazra) Eunice Kennedy Shriver National Institute of Child Health and Human Development, Bethesda, MD, United States

Publisher

Oxford University Press

Emtree Heading

adolescent; adult; age; alcohol abuse; anxiety disorder; appendicitis; article; asthma; atypical mycobacteriosis; Black person; body mass; cause of death; CD4 lymphocyte count; cellulitis; Chlamydia; chlamydiasis; clinical feature; cohort analysis; *combination chemotherapy; communication disorder; controlled study; diabetes mellitus; disease severity; dyslipidemia; eczema; esophagus candidiasis; ethnicity; failure to thrive; female; fetus death; follow up; fracture; gastroesophageal reflux; gender; genital tract infection; gonorrhea; hepatitis; herpes simplex; herpes zoster; *highly active antiretroviral therapy; Hispanic; human; Human immunodeficiency virus; *Human immunodeficiency virus infection/ep [Epidemiology]; *Human immunodeficiency virus infection/dt [Drug Therapy]; hypertension; incidence; induced abortion; intrauterine growth retardation; iron deficiency anemia; kidney disease; latent tuberculosis; learning disorder; lipodystrophy; major clinical study; male; medical record review; mental disease; metabolic bone disease; mood disorder; *mortality; mortality rate; neutropenia; observational study; opportunistic infection; outcome assessment; papillomavirus infection; perinatal infection; pneumonia; posttraumatic stress disorder; preeclampsia; premature labor; priority journal; prospective study; pulmonary candidiasis; spontaneous abortion; substance abuse; syphilis; trend study; trichomoniasis; virus load; vitamin deficiency; wasting syndrome; young adult; *antiretrovirus agent/dt [Drug Therapy]; virus RNA; vitamin D.

Link to the Ovid Full Text or citation:

Click here for full text options

18.

Screening for diabetes mellitus in tuberculosis patients in a referral center in Iran.

Baghaei P., Tabarsi P., Marjani M., Moniri A., Masjedi M.R.

Infectious Diseases. 47 (7) (pp 472-476), 2015. Date of Publication: 2015.

AN: 20160253952

Background: A significant link between diabetes mellitus (DM) and tuberculosis (TB) has been widely demonstrated. DM increases the risk of TB in all aspects. The aims of this study were to assess the prevalence of DM among newly diagnosed TB patients, to screen these patients for DM, and to determine the number needed to screen (NNS) to diagnose new cases of DM. Methods: A prospective cohort descriptive study was carried out in Iranian adults admitted to the National Research Institute of Tuberculosis and Lung Disease from 2012 to 2013 with a new diagnosis of TB. Glycated hemoglobin (HbA1c) and fasting blood glucose (FBG) were measured for all patients. Results: Of the 293 patients included, 101 (34.5%) had DM. DM was newly diagnosed in 45 (19%) patients. The number needed to screen was 5 to identify one new DM case. Age > 40 years was associated with DM in this population (odds ratio (OR)=3.91, 95% confidence interval (CI)=1.47 - 10.38). Conclusion: Screening for DM should be performed routinely in patients with TB and may improve treatment outcomes.

Institution

(Baghaei, Tabarsi, Marjani, Moniri, Masjedi) Clinical Tuberculosis and Epidemiology Research Center, National Research Institute of Tuberculosis and Lung Diseases (NRITLD), Shahid Beheshti University of Medical Sciences, Tehran, Iran, Islamic Republic of

Publisher

Taylor and Francis Ltd.

Emtree Heading

adult; age; article; cohort analysis; descriptive research; *diabetes mellitus/di [Diagnosis]; diabetic patient; fasting blood glucose; female; gender; glucose blood level; human; impaired fasting glucose; impaired glucose tolerance; Iran; *lung tuberculosis/di [Diagnosis]; major clinical study; male; number needed to screen; prevalence; prospective study; screening test; sensitivity and specificity; glucose/ec [Endogenous Compound]; hemoglobin A1c/ec [Endogenous Compound].

Link to the Ovid Full Text or citation:

Click here for full text options

19.

Social, economic, and immunological impacts of TB treatment in Eastern rural China.

Xiao X., Yuan L., Qiu L., Lu W., Jiang W., Zhao Q.

International Journal of Infectious Diseases. Conference: 17th International Congress on Infectious Diseases Hyderabad India. Conference Start: 20160302 Conference End: 20160305. Conference Publication: (var.pagings). 45 (pp 250), 2016. Date of Publication: April 2016.

AN: 72245512

Background: Drug resistant TB in China has been rapidly growing and becoming a cause for critical public health concern. It was reported that the acquired drug-resistant TB mainly results from incompliance to treatment, uncompleted treatment, irregular treatment and unqualified health services. Methods & Materials: Case-cohort was established in four counties in eastern rural China. All registered active TB patients from April 2013 to March 2014 were investigated using structure questionnaire to collect demographic, geographic, socioeconomic information and disease profile. Enzyme-linked immunosorbent assay (ELISA) was used to assess the level of serum cytokines (IFN-gamma, IL - 4) before anti-TB treatment. All the participants were followed up 6 or 8 months until treatment was completed. Results: In total, 1404 patients were recruited, in which 1270 (94.5%) were newly diagnosed TB patients, 447 (33.8%) were smearpositive (SS+). The average age was 49.5+/-18.8, and 76.2% was male. The serum IFN-gamma and IL-4 before anti-TB treatment was 7.936 and 12.292 pg/ml respectively. 80.9% of newly diagnosed TB patients received standard anti-TB treatment, while 87.7% retreatment TB patients received standard anti-TB treatment. The successfully treated rate was 92.5% and 78.6% of new TB patients and retreatment TB patients respectively. The sputum conversion rate of SS+ TB patients after 2 month anti-TB treatment was 96.5%, while the cure rate and successful rate was 80.5% and 90.0% respectively. 92.6% smear-negative patients completed anti-TB treatment. The successful rate in Jiangsu (97.3%), the more developed area, was higher than in Jiangxi (87.6%) (OR = 6.276, 95% CI: 2.528 15.579). Patients with BMI<18.5 may be more risk to fail the treatment compared with the health BMI (18.5-23.9) patients (OR = 3.196, 95% CI: 1.592 6.418). There was no relationship between serum cytokines level and treatment outcomes. The cure rate of TB comorbidity with diabetes was same as the one without diabetes. Conclusion: The sputum conversion rate, cure rate and successful rate of TB patients were high in eastern rural China. Improving patients' nutrition, keeping a healthy BMI could be the key point of increasing successfully treated rate of tuberculosis. More research should be done in future to evaluate the impact of the serum cytokines during anti-TB treatment.

Institution

(Xiao, Yuan, Jiang, Zhao) School of Public Health, Fudan University, Shanghai, China (Qiu) Jiangxi Provincial Center For Disease Control And Prevention, Jiangxi, China

(Lu) Jiangsu Provincial Center For Disease Control And Prevention, Jiangsu, China

Publisher

Elsevier

Emtree Heading

*infection; *China; human; patient; serum; sputum; retreatment; diabetes mellitus; drug resistant tuberculosis; comorbidity; treatment outcome; male; health; risk; smear; enzyme linked immunosorbent assay; health service; tuberculosis; nutrition; questionnaire; public health; cytokine; interleukin 4; interferon.

Link to the Ovid Full Text or citation:

Click here for full text options

20.

Tuberculosis.

Dheda K., Barry C.E., Maartens G.

The Lancet. 387 (10024) (pp 1211-1226), 2016. Date of Publication: 19 Mar 2016.

AN: 2015381148

Summary Although the worldwide incidence of tuberculosis has been slowly decreasing, the global disease burden remains substantial (~9 million cases and ~1.5 million deaths in 2013), and tuberculosis incidence and drug resistance are rising in some parts of the world such as Africa. The modest gains achieved thus far are threatened by high prevalence of HIV, persisting global poverty, and emergence of highly drug-resistant forms of tuberculosis. Tuberculosis is also a major problem in health-care workers in both low-burden and high-burden settings. Although the ideal preventive agent, an effective vaccine, is still some time away, several new diagnostic technologies have emerged, and two new tuberculosis drugs have been licensed after almost 50 years of no tuberculosis drugs being registered. Efforts towards an effective vaccine have been thwarted by poor understanding of what constitutes protective immunity. Although new interventions and investment in control programmes will enable control, eradication will only be possible through substantial reductions in poverty and overcrowding, political will and stability, and containing co-drivers of tuberculosis, such as HIV, smoking, and diabetes.

Institution

(Dheda) Lung Infection and Immunity Unit, Division of Pulmonology and UCT Lung Institute, University of Cape Town, Cape Town, South Africa (Maartens) Division of Clinical Pharmacology, University of Cape Town, Cape Town, South Africa

(Barry) Department of Medicine, Institute of Infectious Disease and Molecular Medicine, Faculty of Health Sciences, University of Cape Town, Cape Town, South Africa

(Dheda, Barry, Maartens) Tuberculosis Research Section, Laboratory of Clinical Infectious Diseases, National Institute of Allergy and Infectious Diseases (NIAID), National Institutes of Health (NIH), Bethesda, MD, United States

Publisher

Lancet Publishing Group

Emtree Heading

antibiotic sensitivity; article; contact examination; diagnostic procedure; diagnostic test; disease transmission; drug resistant tuberculosis; human; Human immunodeficiency virus infection; immunopathogenesis; incidence; infection control; infection risk; latent tuberculosis; mixed infection; multidrug resistant tuberculosis; outcome assessment; pathogenesis; prevalence; priority journal; prognosis; prophylaxis; short course therapy; *tuberculosis/ep [Epidemiology]; *tuberculosis/et [Etiology]; BCG vaccine; corticosteroid; tuberculostatic agent.

Link to the Ovid Full Text or citation:

Click here for full text options

21.

Diabetes and Other Risk Factors for Multi-drug Resistant Tuberculosis in a Mexican Population with Pulmonary Tuberculosis: Case Control Study.

Gomez-Gomez A., Magana-Aquino M., Lopez-Meza S., Aranda-Alvarez M., Diaz-Ornelas D.E., Hernandez-Segura M.G., Salazar-Lezama M.A, Castellanos-Joya M., Noyola D.E.

Archives of medical research. 46 (2) (pp 142-148), 2015. Date of Publication: 01 Feb 2015.

AN: 25704633

RESULTS: Age, gender, malnutrition, being a health-care worker, HIV/AIDS status, and drug abuse were not significantly different between MDR-TB and non-MDR-TB patients. Significant differences between MDR-TB and non-MDR-TB patients were DM (47.2 vs. 28.1%; p = 0.028); previous anti-TB treatments (3 vs. 0, respectively; p <0.001), and duration of first anti-TB treatment (8 vs. 6 months, respectively; p <0.001). CONCLUSIONS: MDR-TB and DM are associated in 47.2% of MDR TB cases (17/36) in this study. Other recognized factors were not found to be significantly different in MDR-TB compared to non-MDR-TB in this study. Cost-feasible strategies must be implemented in the treatment of DM-TB in order to prevent the selection of MDR-TB.

BACKGROUND AND AIMS: Multidrug resistant tuberculosis (MDR-TB) poses problems in treatment, costs and treatment outcomes. It is not known if classically described risk factors for MDR-TB in other countries are the same in Mexico and the frequency of the association between diabetes mellitus (DM) and MDR-TB in our country is not clear. We undertook this study to analyze risk factors associated with the development of MDR-TB, with emphasis on DM.

METHODS: A case-control study in the state of San Luis Potosi (SLP), Mexico was carried out. All pulmonary MDR-TB patients diagnosed in the state of SLP between 1998 and 2013 (36 cases) evaluated at a state pharmacoresistant tuberculosis (TB) clinic and committee; 139 controls were randomly selected from all pulmonary non-multidrug-resistant tuberculosis (non-MDR-TB) cases identified between 2003 and 2008. Cases and controls were diagnosed and treated under programmatic conditions.

Institution

(Gomez-Gomez) Division de Medicina Interna, Hospital Central "Dr. Ignacio Morones Prieto", San Luis Potosi, Mexico; Departamento de Medicina Interna, Facultad de Medicina, Universidad Autonoma de San Luis Potosi, San Luis Potosi, Mexico; Comite Estatal de Tuberculosis Farmacorresistente, San Luis Potosi, Mexico. Electronic address: agomez.cer@prodigy.net.mx (Magana-Aquino) Division de Medicina Interna, Hospital Central "Dr. Ignacio Morones Prieto", San Luis Potosi, Mexico; Departamento de Medicina Interna, Facultad de Medicina, Universidad Autonoma de San Luis Potosi, San Luis Potosi, Mexico; Comite Estatal de Tuberculosis Farmacorresistente, San Luis Potosi, Mexico

(Lopez-Meza) Programa de Atencion al Adulto Mayor, San Luis Potosi, Mexico

(Aranda-Alvarez) Direccion de Politicas y Calidad en Salud, San Luis Potosi, Mexico

(Diaz-Ornelas) Programa de Micobacteriosis Servicios de Salud del Estado de San Luis Potosi, San Luis Potosi, Mexico

(Hernandez-Segura) Servicio de Medicina Preventiva, Hospital Central "Dr. Ignacio Morones Prieto", San Luis Potosi, Mexico

(Salazar-Lezama) Asesor Nacional en Tuberculosis Farmacorresistente, Instituto Nacional de Enfermedades Respiratorias, Mexico, D.F., Mexico

(Castellanos-Joya) Direccion Programa Nacional de Tuberculosis, Secretaria de Salud, Mexico, D.F., Mexico

(Noyola) Departmento de Microbiologia, Facultad de Medicina, Universidad Autonoma de San Luis Potosi, San Luis Potosi, Mexico

Emtree Heading

adult; bacterium identification; case control study; diabetes mellitus; drug effects; female; *genetics; health care cost; health care personnel; human; male; Mexico; microbial sensitivity test; *microbiology; middle aged; mortality; multidrug resistance; Mycobacterium tuberculosis; outpatient department; *pathology; risk factor; Tuberculosis, Multidrug-Resistant/dt [Drug Therapy]; Tuberculosis, Pulmonary/dt [Drug Therapy]; isoniazid/dt [Drug Therapy]; rifampicin/dt [Drug Therapy]; tuberculostatic agent/dt [Drug Therapy].

Link to the Ovid Full Text or citation:

Click here for full text options

22.

Transient hyperglycemia in patients with tuberculosis in Tanzania: Implications for diabetes screening algorithms.

Boillat-Blanco N., Ramaiya K.L., Mganga M., Minja L.T., Bovet P., Schindler C., Von Eckardstein A., Gagneux S., Daubenberger C., Reither K., Probst-Hensch N.

Journal of Infectious Diseases. 213 (7) (pp 1163-1172), 2016. Date of Publication: 2016.

AN: 20160240084

Background. Diabetes mellitus (DM) increases tuberculosis risk while tuberculosis, as an infectious disease, leads to hyperglycemia. We compared hyperglycemia screening strategies in controls and patients with tuberculosis in Dar es Salaam, Tanzania. Methods. Consecutive adults with tuberculosis and sex- and age-matched volunteers were included in a case-control study between July 2012 and June 2014. All underwent DM screening tests (fasting capillary glucose [FCG] level, 2-hour CG [2-hCG] level, and glycated hemoglobin A1c [HbA1c] level) at enrollment, and cases were tested again after receipt of tuberculosis treatment. Association of tuberculosis and its outcome with hyperglycemia was assessed using logistic regression analysis adjusted for sex, age, body mass index, human immunodeficiency virus infection status, and socioeconomic status. Patients with tuberculosis and newly diagnosed DM were not treated for hyperglycemia. Results. At enrollment, DM prevalence was significantly higher among patients with tuberculosis (n = 539; FCG level > 7 mmol/ L, 4.5% of patients, 2-hCG level > 11 mmol/L, 6.8%; and HbA1c level > 6.5%, 9.3%), compared with controls (n = 496; 1.2%, 3.1%, and 2.2%, respectively). The association between hyperglycemia and tuberculosis disappeared after tuberculosis treatment (adjusted odds ratio [aOR] for the FCG level: 9.6 [95% confidence interval {CI}, 3.7-24.7] at enrollment vs 2.4 [95% CI, .7-8.7] at follow-up; aOR for the 2-hCG level: 6.6 [95% CI, 4.0-11.1] vs 1.6 [95% CI, .8-2.9]; and aOR for the HbA1c level, 4.2 [95% CI, 2.9-6.0] vs 1.4 [95% CI, .9-2.0]). Hyperglycemia, based on the FCG level, at enrollment was associated with tuberculosis treatment failure or death (aOR, 3.3; 95% CI, 1.2-9.3). Conclusions. Transient hyperglycemia is frequent during tuberculosis, and DM needs confirmation after tuberculosis treatment. Performance of DM screening at tuberculosis diagnosis gives the opportunity to detect patients at risk of adverse outcome.

Institution

(Boillat-Blanco, Minja, Reither) Ifakara Health Institute, Tanzania (Ramaiya) Shree Hindu Mandal Hospital, Tanzania

(Ramaiya) Muhimbili University of Health Sciences, Tanzania

(Mganga) Kinondoni Municipal Council, National Tuberculosis Program, Dar es Salaam, Tanzania

(Boillat-Blanco, Schindler, Gagneux, Daubenberger, Reither, Probst-Hensch) Swiss Tropical and Public Health Institute, Tanzania

(Boillat-Blanco, Schindler, Gagneux, Daubenberger, Reither, Probst-Hensch) Department of Sciences, University of Basel, Switzerland

(Boillat-Blanco) Infectious Diseases Service, Lausanne University Hospital, Rue du Bugnon 46, Lausanne 1011, Switzerland

(Bovet) Institute of Social and Preventive Medicine, Lausanne University Hospital, Switzerland

(Von Eckardstein) Institute of Clinical Chemistry, University of Zurich, University Hospital of Zurich, Switzerland

Publisher

Oxford University Press

Emtree Heading

adult; age; article; body mass; capillary blood; case control study; controlled study; *diabetes mellitus/di [Diagnosis]; diet restriction; disease association; drug treatment failure; female; follow up; glucose blood level; human; Human immunodeficiency virus infection; *hyperglycemia; logistic regression analysis; major clinical study; male; mortality; prevalence; priority journal; prognosis; risk assessment; screening test; sex difference; social status; Tanzania; *tuberculosis/dt [Drug Therapy]; hemoglobin A1c/ec [Endogenous Compound]; tuberculostatic agent/dt [Drug Therapy].

Link to the Ovid Full Text or citation:

Click here for full text options

23.

Can china achieve the who global targets for tb control by 2035?.

Huynh G.H.

Transactions of the Royal Society of Tropical Medicine and Hygiene. 110 (3) (pp 161-162), 2015. Article Number: trv105. Date of Publication: 07 Oct 2015.

AN: 20160200407

To reach the ambitious WHO TB global targets by 2035, it is likely that China will need a comprehensive strategy that builds on its existing high-quality directly observed treatment, short-course program. This will require optimizing the use of existing tools within a changing health system landscape. In addition, new tools are needed to identify and treat TB in high-risk groups and in older people, who are a growing driver of disease incidence. Lastly, strategies are needed to address the proximate risk factors and social determinants that underlie trends in TB burden.

Institution

(Huynh) Massachusetts Institute of Technology, Cambridge, MA 02139, United States

Publisher

Oxford University Press

Emtree Heading

age; *China; control strategy; decentralization; diabetes mellitus; disease course; disease transmission; drug monitoring; drug resistant tuberculosis; drug sensitivity; health care access; health care management; health care planning; health care quality; health care system; health service; high risk population; hospital planning; human; incidence; indoor air pollution; *lung tuberculosis/pc [Prevention]; malnutrition; mathematical model; medication compliance; morbidity; mortality rate; note; prevalence; retreatment; risk factor; risk reduction; short course therapy; smoking; social determinants of health; treatment outcome; *tuberculosis control; *world health organization.

Link to the Ovid Full Text or citation:

Click here for full text options

24.

Does enhanced diabetes management reduce the risk and improve the outcome of tuberculosis?.

Lo H.-Y., Yang S.-L., Lin H.-H., Bai K.-J., Lee J.-J., Lee T.-I., Chiang C.-Y.

International Journal of Tuberculosis and Lung Disease. 20 (3) (pp 376-382), 2016. Date of Publication: 01 Mar 2016.

AN: 20160148052

SETTING: The Bureau of National Health Insurance (NHI) has implemented a pay-for-performance (p4p) programme for diabetes mellitus (DM) in Taiwan. OBJECTIVE : To investigate whether patients with DM enrolled in the p4p programme (DM-p4p) are less likely to develop tuberculosis (TB) and whether they have a better outcome than patients with DMnot enrolled in the p4p programme (DM-non-p4p) if they do develop TB. DESIGN: A random sample of 79 471 DM-p4p, 100 000 DM-non-p4p and 100 000 non-diabetic patients (non-DM) was obtained from the 2008-2009 NHI database, and the patients were matched with the National TB Registry to determine whether they had developed TB by the end of 2010. RESULTS : The average annual incidence of TB was respectively 259.9 (95%CI 230.2-293.4), 137.5 (95%CI 116.4-162.5) and 74.1 (95%CI 59.0-93.0) per 100 000 population among DM-non-p4p, DM-p4p and non-DM patients. The relative risk of death over treatment success was 1.79 (95%CI 1.05-3.04) among DM-non-p4p and 1.69 (95%CI 0.84-3.40) among non-DM patients, relative to DM-p4p patients. CONCLUSIONS : Enhanced case management of DM reduced risk and improved outcomes of TB among patients with DM.

Institution

(Lo, Yang) Taiwan Centers for Disease Control, Ministry of Health and Welfare, Taipei, Taiwan (Republic of China) (Lin) Institute of Epidemiology and Preventive Medicine, National Taiwan University, Taipei, Taiwan (Republic of China)

(Bai) School of Respiratory Therapy, College of Medicine, Taipei Medical University, Taipei, Taiwan (Republic of China)

(Bai, Chiang) Division of Pulmonary Medicine, Department of Internal Medicine, Wan Fang Hospital, Taipei Medical University, 111 Hsin-Long Road, Taipei, Taiwan (Republic of China)

(Lee) Department of Internal Medicine, Tzu Chi General Hospital and Tzu Chi University, Hualien, Taiwan (Republic of China)

(Lee) Division of Endocrinology and Metabolism, Department of Internal Medicine, Wan Fang Hospital, Taipei Medical University, Taipei, Taiwan (Republic of China)

(Lee) Department of General Medicine, School of Medicine, College of Medicine, Taipei Medical University, Taipei, Taiwan (Republic of China)

(Chiang) Department of Internal Medicine, School of Medicine, College of Medicine, Taipei Medical University, Taipei, Taiwan (Republic of China)

(Chiang) International Union Against Tuberculosis and Lung Disease, Paris, France

Publisher

International Union against Tubercul. and Lung Dis.

Emtree Heading

adult; aged; article; case management; cohort analysis; controlled study; *diabetes mellitus; female; *health insurance; human; incidence; major clinical study; male; mortality; *pay-for-performance; priority journal; *risk reduction; treatment outcome; *tuberculosis; very elderly.

Link to the Ovid Full Text or citation:

Click here for full text options

25.

Tuberculosis prevention and control: We need to pay heed to diabetes care.

Kapur A.

International Journal of Tuberculosis and Lung Disease. 20 (3) (pp 287), 2016. Date of Publication: 01 Mar 2016.

AN: 20160148036

Institution

(Kapur) World Diabetes Foundation, Gentofte, Denmark

Publisher

International Union against Tubercul. and Lung Dis.

Emtree Heading

adaptive immunity; CD4 lymphocyte count; chronic inflammation; clinical feature; *diabetes control; editorial; human; Human immunodeficiency virus infection; hyperglycemia; infection risk; innate immunity; insulin resistance; outcome assessment; *patient care; primary medical care; priority journal; total quality management; *tuberculosis control; world health organization.

Link to the Ovid Full Text or citation:

Click here for full text options

26.

Tuberculosis in older versus younger adult patients: A retrospective comparison of patient characteristics and treatment outcomes at a major UK referral centre.

Abbara A., Buell K.G., Sullivan J.A.L., Collin S.M., Kon O.M., Hansel T., John L., Davidson R.N.

Thorax. Conference: British Thoracic Society Winter Meeting 2015 London United Kingdom. Conference Start: 20151202 Conference End: 20151204. Conference Publication: (var.pagings). 70 (pp A203), 2015. Date of Publication: December 2015.

AN: 72199832

Introduction and objectives Tuberculosis (TB) in older persons presents challenges related to diagnosis, management, comorbidities and polypharmacy potentially contributing to increased morbidity and mortality. This retrospective cohort review compares the baseline characteristics, diagnosis, management and outcome between older patients (OPs) (over 65 years) and younger patients (YPs) (25-35 years.) Method All patients >65 years treated at Northwick Park Hospital during 2002-2014 were identified from London TB register; a comparison group of patients aged 25-35 years were randomly selected. Clinical, microbiological, radiological and biochemical parameters together with management and outcomes were obtained from electronic records. Characteristics of patients were compared between the two groups using Chi-squared and Kruskal-Wallis tests; analyses were performed using Stata (Stata Corp. 2013). Results The comparison groups comprised 313 patients aged >65 years and 339 patients aged 25-35. Demographics, site of disease, TB culture, treatment regimens and outcomes are recorded in Table 1. 35.6% of OPs and 29.6% of YPs were symptomatic for >2 months at review in secondary care. Median duration to starting treatment from review was 17 days (IQR: 4-57) in OPs compared to 2 (IQR: 1-19) in YPs (p = 0.001). 44.8% of OPs experienced drug toxicity compared to 27.3% of YPs (p = <0.001.) Gastrointestinal symptoms affected 24.8% and 9.6% of OPs and YPs respectively (p < 0.001). There was no difference in prevalence of rash (4.8% in OPs,) arthralgia (2.4% of OPs,) or drug induced liver injury (6.4% of OPs, p = 0.32.) Comorbidities were higher in OPs, with diabetes present in 34.5%, hypertension in 52.6% and renal failure in 17.9% compared to 1.5%, 1.2% and 1.8% in YPs (all p = 0.001). 58.8% of >65 and 37.9% of YPs had inpatient admissions, with 30% of OPs admitted for >10 days (p = <0.001). Completion was 78.7% and mortality 16.0% amongst OPs, versus 91.2% with no deaths amongst YPs (both p = <0.001). Conclusion These data characterise the delays in presentation and treatment initiation in older patients who also experience a more complicated treatment course with an increased side effect profile, more variation from standard quadruple therapy, lower completion rates and poorer outcomes. This, together with longer inpatient stays impacts patients, but also has financial implications for services.

Institution

(Abbara, John, Davidson) London North West NHS Healthcare Trust, London, United Kingdom (Buell, Sullivan) Imperial College, London, United Kingdom

(Collin) University of Bristol, Bristol, United Kingdom

(Kon, Hansel) Imperial NHS Healthcare Trust, London, United Kingdom

Publisher

BMJ Publishing Group

Emtree Heading

*patient; *human; *treatment outcome; *winter; *society; *United Kingdom; *adult; *tuberculosis; mortality; hospital patient; diagnosis; prevalence; drug toxicity; hospital; morbidity; secondary health care; Kruskal Wallis test; diabetes mellitus; kidney failure; therapy; gastrointestinal symptom; rash; arthralgia; toxic hepatitis; hypertension; parameters; register; death; side effect; polypharmacy.

Link to the Ovid Full Text or citation:

Click here for full text options

27.

Incidence of tuberculosis is high in chronic kidney disease patients in South East England and drug resistance common.

Ostermann M., Palchaudhuri P., Riding A., Begum P., Milburn H.J.

Renal Failure. 38 (2) (pp 256-261), 2016. Date of Publication: 07 Feb 2016.

AN: 20160022470

The risk of tuberculosis (TB) is significantly increased in chronic kidney disease (CKD). Data on TB in CKD in the UK are sparse; most information stems from countries with high background prevalence. The aim of this study was to estimate the incidence of TB in CKD patients in South East London and to describe the epidemiology, treatment, and outcome. CKD patients with TB between 1994 and 2010 were identified retrospectively. Data were collected on type of renal replacement therapy, the method of TB diagnosis, disease site, treatment regimens, and risk factors. Forty patients were identified of whom 67.5% had CKD stages IV-V. Sixty-five percent were from non-UK born ethnic minorities. Median time from diagnosis of CKD to TB development was 12 months (range 0-192 months). Cumulative incidence of TB was 1267/100,000 [95% confidence interval (CI): 630-1904; 85 x background UK rate] in hemodialysis patients; 398/100,000 (95% CI: 80-1160; 26 x background UK rate) in peritoneal dialysis; and 522/100,000 (CI: 137-909; 35 x background UK rate) in transplant recipients. Sixty-three percent of patients had pulmonary TB and 25% of patients with culture-positive TB had resistant isolates. Fifty percent of patients were immunosuppressed due to drugs, diabetes, and/or retroviral disease. Treatment regimens were according to recent national guidance in 73% of cases. Seventy-six percent of patients experienced side effects. Greater awareness of risk factors, drug resistance, treatment regimens, and potential side effects is needed.

Institution

(Ostermann) Department of Renal Medicine, Guys and St Thomas NHS Foundation Trust, London, United Kingdom (Ostermann, Milburn) Guys and St Thomas NHS Foundation Trust, Kings College London, Great Maze Pond, London SE1 9RT, United Kingdom

(Palchaudhuri, Riding, Begum, Milburn) Department of Respiratory Medicine, Guys and St Thomas NHS Foundation Trust, London, United Kingdom

Publisher

Taylor and Francis Ltd

Emtree Heading

adult; anorexia; article; atypical mycobacteriosis; *chronic kidney disease; clinical article; cohort analysis; female; fever; graft recipient; hemodialysis patient; hemoptysis; human; hyperparathyroidism/dt [Drug Therapy]; hyperphosphatasemia/dt [Drug Therapy]; immunocompromized patient; incidence; interferon gamma release assay; kidney transplantation; male; peritoneal dialysis; priority journal; renal replacement therapy; retrospective study; thorax radiography; treatment duration; treatment outcome; *tuberculosis/di [Diagnosis]; *tuberculosis/dr [Drug Resistance]; *tuberculosis/ep [Epidemiology]; United Kingdom; unspecified side effect/si [Side Effect]; vitamin blood level; vitamin supplementation; weight reduction; cycloserine; ethambutol; immunosuppressive agent; isoniazid; phosphate binding agent/dt [Drug Therapy]; pyrazinamide; streptomycin; *tuberculostatic agent/ae [Adverse Drug Reaction]; vitamin D.

Link to the Ovid Full Text or citation:

Click here for full text options

28.

Efficacy and effect of free treatment on multidrug-resistant tuberculosis.

Zhang Q., Wu Z., Zhang Z., Sha W., Shen X., Xiao H.

Experimental and Therapeutic Medicine. 11 (3) (pp 777-782), 2016. Date of Publication: March 2016.

AN: 20160084966

Multidrug-resistant tuberculosis (MDR-TB) is a serious public health and social issue. It pertains to the type of tuberculosis that is resistant simultaneously to isoniazid and rifampicin. MDR-TB has a high mortality and is expensive to treat. The aim of the present study was to examine the therapeutic effects of individualized free treatment and the relevant influencing factors on the treatment outcome for MDR-TB. A prospective study module was used to analyze the therapeutic outcome of MDR-TB with individualized free treatment for 160 patients between 2011 and 2014. Statistical analysis was performed using the Chi-square or Fisher's exact test, and the odds ratio was calculated using a logistic regression analysis model. In total, 160 patients were enrolled in the study for treatment of MDR-TB. From these, 88 cases completed the course of treatment, and 70 cases were successfully treated. Of the remaining 72 cases, 37 cases exhibited treatment failure, 18 cases were suspended during treatment and 17 patients succumbed to the disease. The results showed that the confounding factors were: i) retreatment (p<0.05); ii) occurrence of diabetes (p<0.001); iii) lesion without improvement in radiography during treatment (p=0.001); iv) positive sputum culture for Mycobacterium tuberculosis after 3-month treatment (p<0.05); and v) termination of treatment due to adverse reaction (p<0.05). These factors were associated with poor treatment outcomes by logistic regression analysis. Adverse drug reaction was observed in 33 cases and treatment was terminated or changed permanently in 29 of these cases. The most common adverse reaction was liver function damage caused by pyrazinamide and leucopenia caused by rifabutin. One patient suffered from serious liver failure. In conclusion, the success rate of long treatment course for MDR-TB is not high due to many adverse reactions. Occurrence of diabetes is the main factor that caused poor efficacy.

Institution

(Zhang, Sha, Xiao) Clinic and Research Center of Tuberculosis, Shanghai Key Laboratory of Tuberculosis, Shanghai Pulmonary Hospital, Tongji University School of Medicine, Shanghai 200433, China (Wu, Zhang, Shen) Shanghai Center for Disease Control and Prevention, Shanghai 200065, China

Publisher

Spandidos Publications (10 Vriaxidos Street, Athens 116 10, Greece)

Emtree Heading

adult; article; bacteriology; drug efficacy; female; human; leukopenia/si [Side Effect]; liver injury/si [Side Effect]; logistic regression analysis; major clinical study; male; medical record review; middle aged; mortality; *multidrug resistant tuberculosis/dt [Drug Therapy]; risk factor; treatment outcome; treatment planning; amikacin/dt [Drug Therapy]; aminosalicylic acid/ae [Adverse Drug Reaction]; aminosalicylic acid/dt [Drug Therapy]; isoniazid/ae [Adverse Drug Reaction]; isoniazid/dt [Drug Therapy]; protionamide/ae [Adverse Drug Reaction]; protionamide/dt [Drug Therapy]; pyrazinamide/ae [Adverse Drug Reaction]; pyrazinamide/dt [Drug Therapy]; rifabutin/ae [Adverse Drug Reaction]; rifabutin/dt [Drug Therapy].

Link to the Ovid Full Text or citation:

Click here for full text options

29.

Diabetes and tuberculosis co-epidemic: The Bali Declaration.

Kapur A., Harries A.D., Lonnroth K., Wilson P., Sulistyowati L.S.

The Lancet Diabetes and Endocrinology. 4 (1) (pp 8-10), 2016. Date of Publication: 01 Jan 2016.

AN: 20151026283

Institution

(Kapur) World Diabetes Foundation, Gentofte, Denmark (Harries) International Union Against Tuberculosis and Lung Disease, Paris 75006, France

(Harries) London School of Hygiene and Tropical Medicine, Keppel Street, London, United Kingdom

(Lonnroth) World Health Organization, Geneva, Switzerland

(Lonnroth) Department of Public Health Sciences, Karolinska Institutet, Stockholm, Sweden

(Wilson) International Diabetes Federation, Brussels, Belgium

(Sulistyowati) Directorate of Non-Communicable Diseases Control, Directorate of Diseases Control and Environmental Health, Ministry of Health, Jakarta, Indonesia

Publisher

Lancet Publishing Group

Emtree Heading

clinical feature; communicable disease; *diabetes mellitus/ep [Epidemiology]; *diabetes mellitus/dt [Drug Therapy]; *epidemic; glucose tolerance; human; Indonesia; infection risk; medical documentation; morbidity; mortality; Mycobacterium tuberculosis; non communicable disease; note; poverty; practice guideline; prevalence; priority journal; recurrent infection; sustainable development; treatment failure; *tuberculosis/ep [Epidemiology]; *tuberculosis/dt [Drug Therapy]; urban population; rifampicin/to [Drug Toxicity]; rifampicin/dt [Drug Therapy]; sulfonylurea derivative/po [Oral Drug Administration]; sulfonylurea derivative/dt [Drug Therapy].

Link to the Ovid Full Text or citation:

Click here for full text options

30.

Diabetes is associated with worse clinical presentation in tuberculosis patients from Brazil: A retrospective cohort study.

Gil-Santana L., Almeida J.L., Oliveira C.A.M., Hickson L.S., Daltro C., Castro S., Kornfeld H., Netto E.M., Andrade B.B.

PLoS ONE. 11 (1) (no pagination), 2016. Article Number: e0146876. Date of Publication: 11 Jan 2016.

AN: 20160054999

Background. The rising prevalence of diabetes mellitus (DM) worldwide, especially in developing countries, and the persistence of tuberculosis (TB) as a major public health issue in these same regions, emphasize the importance of investigating this association. Here, we compared the clinical profile and disease outcomes of TB patients with or without coincident DM in a TB reference center in Brazil. Methods. We performed a retrospective analysis of a TB patient cohort (treatment naive) of 408 individuals recruited at a TB primary care center in Brazil between 2004 and 2010. Data on diagnosis of TB and DM were used to define the groups. The study groups were compared with regard to TB disease presentation at diagnosis as well as to clinical outcomes such as cure and mortality rates upon anti-tuberculosis therapy (ATT) initiation. A composite score utilizing clinical, radiological and microbiological parameters was used to compare TB severity between the groups. Results. DM patients were older than non-diabetic TB patients. In addition, diabetic individuals more frequently presented with cough, night sweats, hemoptysis and malaise than those without DM. The overall pattern of lung lesions assessed by chest radiographic examination was similar between the groups. Compared to non-diabetic patients, those with TB-diabetes exhibited positive acid-fast bacilli in sputum samples more frequently at diagnosis and at 30 days after ATT initiation. Notably, higher values of the TB severity score were significantly associated with TB-diabetes comorbidity after adjustment for confounding factors. Moreover, during ATT, diabetic patients required more frequent transfers to TB reference hospitals for complex clinical management. Nevertheless, overall mortality and cure rates were indistinguishable between the study groups. Conclusions. These findings reinforce the idea that diabetes negatively impacts pulmonary TB severity. Our study argues for the systematic screening for DM in TB reference centers in endemic areas.

Institution

(Gil-Santana, Almeida, Andrade) Unidade de Medicina Investigativa, Laboratorio Integrado de Microbiologia e Imunorregulacao, Centro de Pesquisas Goncalo Moniz, Fundacao Oswaldo Cruz, Salvador, Bahia, Brazil (Gil-Santana, Almeida, Andrade) School of Medicine, Faculdades de Tecnologia e Ciencias, Salvador, Bahia, Brazil

(Gil-Santana, Almeida, Andrade) Multinational Organization Network Sponsoring Translational and Epidemiological Research (MONSTER), Fundacao Jose Silveira, Salvador, Brazil

(Oliveira, Hickson, Castro, Netto, Andrade) Research Center, Instituto Brasileiro para Investigacao da Tuberculose (IBIT), Fundacao Jose Silveira, Salvador, Bahia, Brazil

(Daltro, Netto) Hospital Universitario Pro. Edgard Santos, Universidade Federal da Bahia, Salvador, Bahia, Brazil

(Kornfeld) Department of Medicine, University of Massachusetts Medical School, Worcester, MA, United States

Publisher

Public Library of Science

Emtree Heading

acid fast bacterium; adult; alcoholism; article; body mass; Brazil; clinical feature; cohort analysis; comorbidity; controlled study; coughing; *diabetes mellitus; disease association; disease severity; extrapulmonary tuberculosis; female; hemoptysis; human; Human immunodeficiency virus infection; major clinical study; malaise; male; mortality; Mycobacterium tuberculosis; neoplasm; night sweat; outcome assessment; prevalence; primary medical care; retrospective study; scoring system; sex ratio; smoking; thorax radiography; tuberculin test; *tuberculosis; illicit drug.

Link to the Ovid Full Text or citation:

Click here for full text options

31.

Battling tuberculosis in an island context with a high burden of communicable and non-communicable diseases: epidemiology, progress, and lessons learned in Kiribati, 2000 to 2012.

Hoy D., Kienene T., Reiher B., Roth A., Tira T., McKenzie J., Merilles O.E., Viney K.

International journal of infectious diseases : IJID : official publication of the International Society for Infectious Diseases. 30 (pp 135-141), 2015. Date of Publication: 01 Jan 2015.

AN: 25455798

OBJECTIVES: To examine the epidemiology of tuberculosis (TB) in Kiribati from 2000 to 2012, document lessons learned, and recommend ways to mitigate the burden of TB in Kiribati. METHODS: A descriptive study was performed using data on TB case notifications, prevalence, incidence, mortality, and treatment outcomes from global reports and data files. Progress towards meeting the Millennium Development Goal TB target (to reduce TB incidence by 2015) and the Regional Strategy to Stop Tuberculosis in the Western Pacific 2011-2015 targets (to reduce TB prevalence and mortality by half by 2015 relative to the level in 2000) was examined.

RESULTS: TB case notifications and the estimated incidence and prevalence have increased in Kiribati since 2000. From 2000 to 2012, Kiribati reported a total of 3863 TB notifications; in 2012, the case notification rate was 343/100 000 population. The majority (89%) of TB patients complete treatment and/or are cured, and the estimated TB mortality rate has remained relatively stable at around 16/100 000 population. HIV testing of TB patients has increased over recent years from 8% of notifications tested in 2003 to 43% tested in 2012. Of all 818 tests, only four (0.5%) patients were confirmed HIV-positive. Drug-resistant TB has been detected in a small number of cases.

CONCLUSIONS: TB rates continue to increase in Kiribati and the 2015 goals for TB control are unlikely to be met. This is probably due to the complex mix of risk factors present in Kiribati, including smoking, diabetes, alcohol use, crowded living, and poverty. A comprehensive approach to address these risk factors is needed to mitigate the burden of TB in Kiribati.

Institution

(Hoy) Secretariat of the Pacific Community, BP D5, 98848 Noumea, New Caledonia. Electronic address: damianh@spc.int (Kienene) Kiribati Ministry of Health and Medical Services, Kiribati

(Reiher) Kiribati Ministry of Health and Medical Services, Kiribati

(Roth) Secretariat of the Pacific Community, BP D5, 98848 Noumea, New Caledonia

(Tira) Kiribati Ministry of Health and Medical Services, Kiribati

(McKenzie) Secretariat of the Pacific Community, BP D5, 98848 Noumea, New Caledonia

(Merilles) Secretariat of the Pacific Community, BP D5, 98848 Noumea, New Caledonia

(Viney) National Centre of Epidemiology and Population Health, Australian National University, Canberra, Australia

Emtree Heading

adolescent; adult; aged; child; Communicable Diseases/ep [Epidemiology]; Federated States of Micronesia; female; human; incidence; infant; male; middle aged; mortality; newborn; poverty; preschool child; prevalence; risk factor; smoking; treatment outcome; tuberculosis/ep [Epidemiology]; Tuberculosis, Multidrug-Resistant/ep [Epidemiology]; young adult.

Link to the Ovid Full Text or citation:

Click here for full text options

32.

Results of the implementation of a pilot model for the bidirectional screening and joint management of patients with pulmonary tuberculosis and diabetes mellitus in Mexico.

Castellanos-Joya M., Delgado-Sanchez G., Ferreyra-Reyes L., Cruz-Hervert P., Ferreira-Guerrero E., Ortiz-Solis G., Jimenez M.I., Salazar L.L., Montero-Campos R., Mongua-Rodriguez N., Baez-Saldana R., Bobadilla-del-Valle M., Gonzalez-Roldan J.F., Ponce-de-Leon A., Sifuentes-Osornio J., Garcia-Garcia L.

PloS one. 9 (9) (pp e106961), 2014. Date of Publication: 2014.

AN: 25229236

BACKGROUND: Recently, the World Health Organisation and the International Union Against Tuberculosis and Lung Disease published a Collaborative Framework for the Care and Control of Tuberculosis (TB) and Diabetes (DM) (CFTB/DM) proposing bidirectional screening and joint management. OBJECTIVE: To evaluate the feasibility and effectiveness of the CFTB/DM in Mexico.

DESIGN: Prospective observational cohort.

SETTING: 15 primary care units in 5 states in Mexico.

PARTICIPANTS: Patients aged >20 years diagnosed with DM or pulmonary TB who sought care at participating clinics.

INTERVENTION: The WHO/Union CFTB/DM was adapted and implemented according to official Mexican guidelines. We recruited participants from July 2012 to April 2013 and followed up until March 2014. Bidirectional screening was performed. Patients diagnosed with TB and DM were invited to receive TB treatment under joint management.

MAIN OUTCOME MEASURES: Diagnoses of TB among DM, of DM among TB, and treatment outcomes among patients with DM and TB.

RESULTS: Of 783 DM patients, 11 (1.4%) were unaware of their TB. Of 361 TB patients, 16 (4.4%) were unaware of their DM. 95 TB/DM patients accepted to be treated under joint management, of whom 85 (89.5%) successfully completed treatment. Multiple linear regression analysis with change in HbA1c and random capillary glucose as dependent variables revealed significant decrease with time (regression coefficients (beta) = -0.660, (95% confidence interval (CI), -0.96 to -0.35); and beta = -1.889 (95% CI, -2.77 to -1.01, respectively)) adjusting by sex, age and having been treated for a previous TB episode. Patients treated under joint management were more likely to experience treatment success than patients treated under routine DM and TB programs as compared to historical (adjusted OR (aOR), 2.8, 95%CI 1.28-6.13) and same period (aOR 2.37, 95% CI 1.13-4.96) comparison groups.

CONCLUSIONS: Joint management of TB and DM is feasible and appears to improve clinical outcomes.

Institution

(Castellanos-Joya) Direccion del Programa de Micobacteriosis, Centro Nacional de Programas Preventivos y Control de Enfermedades, Mexico, Distrito Federal, Mexico (Delgado-Sanchez) Centro de Investigacion sobre Enfermedades Infecciosas, Instituto Nacional de Salud Publica, Cuernavaca, Morelos, Mexico

(Ferreyra-Reyes) Centro de Investigacion sobre Enfermedades Infecciosas, Instituto Nacional de Salud Publica, Cuernavaca, Morelos, Mexico

(Cruz-Hervert) Centro de Investigacion sobre Enfermedades Infecciosas, Instituto Nacional de Salud Publica, Cuernavaca, Morelos, Mexico

(Ferreira-Guerrero) Centro de Investigacion sobre Enfermedades Infecciosas, Instituto Nacional de Salud Publica, Cuernavaca, Morelos, Mexico

(Ortiz-Solis) Direccion del Programa de Salud en el Adulto y en el Anciano, Centro Nacional de Programas Preventivos y Control de Enfermedades, Mexico, Distrito Federal, Mexico

(Jimenez) Direccion del Programa de Micobacteriosis, Centro Nacional de Programas Preventivos y Control de Enfermedades, Mexico, Distrito Federal, Mexico

(Salazar) Direccion del Programa de Micobacteriosis, Centro Nacional de Programas Preventivos y Control de Enfermedades, Mexico, Distrito Federal, Mexico

(Montero-Campos) Centro de Investigacion sobre Enfermedades Infecciosas, Instituto Nacional de Salud Publica, Cuernavaca, Morelos, Mexico

(Mongua-Rodriguez) Centro de Investigacion sobre Enfermedades Infecciosas, Instituto Nacional de Salud Publica, Cuernavaca, Morelos, Mexico

(Baez-Saldana) Centro de Investigacion sobre Enfermedades Infecciosas, Instituto Nacional de Salud Publica, Cuernavaca, Morelos, Mexico

(Bobadilla-del-Valle) Laboratorio de Microbiologia, Instituto Nacional de Ciencias Medicas y de Nutricion "Salvador Zubiran", Mexico, Distrito Federal, Mexico

(Gonzalez-Roldan) Direccion General, Centro Nacional de Programas Preventivos y Control de Enfermedades, Mexico, Distrito Federal, Mexico

(Ponce-de-Leon) Laboratorio de Microbiologia, Instituto Nacional de Ciencias Medicas y de Nutricion "Salvador Zubiran", Mexico, Distrito Federal, Mexico

(Sifuentes-Osornio) Direccion Medica, Instituto Nacional de Ciencias Medicas y de Nutricion "Salvador Zubiran", Mexico, Distrito Federal, Mexico

(Garcia-Garcia) Centro de Investigacion sobre Enfermedades Infecciosas, Instituto Nacional de Salud Publica, Cuernavaca, Morelos, Mexico

Emtree Heading

aged; diabetes mellitus/di [Diagnosis]; disease management; human; Mexico; middle aged; prospective study; Tuberculosis, Pulmonary/di [Diagnosis].

Link to the Ovid Full Text or citation:

Click here for full text options

33.

Survival benefit associated with early cyclosporine treatment for dermatomyositis-associated interstitial lung disease.

Go D.J., Park J.K., Kang E.H., Kwon H.M., Lee Y.J., Song Y.W., Lee E.B.

Rheumatology International. 36 (1) (pp 125-131), 2016. Date of Publication: 01 Jan 2016.

AN: 2015246394

Interstitial lung disease (ILD) is the most common cause of death in dermatomyositis (DM). Cyclosporine A (CsA) has shown to be effective in DM-associated ILD (DM-ILD). This study aimed to define the optimal time of CsA administration. A total of 47 patients with DM-ILD, who were treated with CsA at Seoul National University Hospital between January 1998 and June 2013, were enrolled. ILD was diagnosed based on typical chest high-resolution computed tomography (HRCT) findings. Patients with early and delayed CsA treatment were compared in regard to the mortality and ILD progression on HRCT. The early (n = 16) and the delayed treatment group (n = 31) did not differ in regard to baseline clinical characteristics including HRCT scores and pulmonary function. Patients with clinically amyopathic DM (CADM) were more common in the early treatment group. The mortality rate was significantly lower in the early treatment group than in the delayed treatment group (p = 0.009). The survival benefit of early CsA treatment remained significant even after adjusting for age, degree of dyspnea, CADM status, and the year of CsA treatment (hazard ratio 0.057, 95 % confidence interval 0.007-0.472). CsA stabilized disease progression on HRCT in the early treatment group (p = 0.738). Delay in CsA treatment is associated with a worse survival in patients with DM-ILD. Early CsA treatment should be considered at DM-ILD diagnosis especially in patients at a higher risk of developing a rapidly progressive ILD.

Institution

(Go, Park, Kwon, Song, Lee) Division of Rheumatology, Department of Internal Medicine, Seoul National University College of Medicine, Seoul, South Korea (Go, Park, Song) Department of Molecular Medicine and Biopharmaceutical Sciences, Graduate School of Convergence Science and Technology, Medical Research Institute, Seoul National University, Seoul, South Korea

(Kang, Lee) Division of Rheumatology, Department of Internal Medicine, Seoul National University Bundang Hospital, Seongnam, South Korea

Publisher

Springer Verlag

Emtree Heading

adult; article; clinical article; cohort analysis; *dermatomyositis; disease association; disease course; drug withdrawal; dyspnea; female; hand tremor/si [Side Effect]; high resolution computer tomography; human; infection/si [Side Effect]; *interstitial lung disease/co [Complication]; *interstitial lung disease/dt [Drug Therapy]; leukopenia/si [Side Effect]; lung function; male; middle aged; mortality; nephrotoxicity/si [Side Effect]; priority journal; rash/si [Side Effect]; retrospective study; *survival; therapy delay; thorax pain/si [Side Effect]; *time to treatment; treatment outcome; tuberculosis/si [Side Effect]; upper abdominal pain/si [Side Effect]; azathioprine/cm [Drug Comparison]; azathioprine/dt [Drug Therapy]; corticosteroid/cm [Drug Comparison]; corticosteroid/dt [Drug Therapy]; cyclophosphamide/iv [Intravenous Drug Administration]; cyclophosphamide/cm [Drug Comparison]; cyclophosphamide/dt [Drug Therapy]; *cyclosporin A/ae [Adverse Drug Reaction]; *cyclosporin A/dt [Drug Therapy]; *cyclosporin A/iv [Intravenous Drug Administration]; *cyclosporin A/po [Oral Drug Administration]; methotrexate/cm [Drug Comparison]; methotrexate/dt [Drug Therapy].

Link to the Ovid Full Text or citation:

Click here for full text options

34.

A few cases of increased FDG uptake on 18F-FDG PET/CT in residual lesions of tuberculosis at the time of treatment completion.

Lee M., Jung I., Kim Y., Lee J.

Chest. Conference: CHEST 2015 Montreal, QC Canada. Conference Start: 20151024 Conference End: 20151028. Conference Publication: (var.pagings). 148 (4 MEETING ABSTRACT) (no pagination), 2015. Date of Publication: October 2015.

AN: 72120174

Introduction: The presence of radiographic lesions such as cavity is one of the risk factors for treatment failure or relapse of pulmonary tuberculosis (TB). A functional imaging technique that monitors glucose metabolism in tissues, 18F-fluorodeoxyglucose positron emission/computed tomography (18F-FDG PET/CT) has been reported recently to detect metabolic activity of TB lesions in the lung which could reveal therapeutic response in early time point. Measuring of FDG uptake at the time of treatment completion could give us information about the association of increased FDG uptake with risk of relapse afterwards. CASE PRESENTATION: Five cases of pulmonary TB had been treated with first-line anti-TB drugs (isoniazid, rifampicin, ethambutol, and pyrazinamide). 4 males and a female, age ranged from 31 to 64 years old are all new TB patients with no history of previous treatment. At the time of diagnosis sputum smears for acid-fast bacilli(AFB) were positive as were cultures for Mycobacterium Tuberculosis. All of them have no co-morbidity of diabetes or cancer. Their chest x-ray showed cavitary lesions in upper lung field in two patients. Their compliance of treatment was good with no interruption of medication by any reasons. Sputum microbiologic tests converted to negative and treament completed after 6 months or 9 months in total. They checked 18FFDG PET/CT at the time of treatment completion. 4 out of 5 showed increased FDG uptake of residual lesions especially in cavity wall or residual parenchymal lesions. DISCUSSION: Tuberculosis (TB) needs very long duration of combination antibiotic therapy required to achieve relapse-free cure. The World Health Organization recommends the standard treatment length as 6 months for drug susceptible TB and 20 months for multidrug-resistant and extensively drug-resistant TB (M/XDR-TB). The optimal duration for treatment of TB likely differs between individuals and depends on a variety of variables, such as the extent of the disease. Personalization of the duration of treatment for TB, especially for patients with M/XDR-TB, would be desired but there has been little information of biomarkers to decide for relapse-free cure. In recent literatures, 18F-FDG PET/CT correlates with treatment outcome in MDR-TB and could be useful as an early therapeutic marker. Given that the increased FDG uptake shows active granulomatous inflammation of TB lesions, 18F-FDG PET/CT at the time of scheduled completion could be a marker to predict the risk of relapse. Conclusions: More study is needed to evaluate the association of increased FDG uptake of residual lesions in the lung at treatment completion with the risk of relapse since 18F-FDG PET/CT at the time of scheduled completion could be used as a decision maker of personalized treatment duration to minimize the risk of relapse in the future, especially for the treatment of drug resistant TB.

Institution

(Lee, Jung, Kim, Lee) INTERNATIONAL TB RESEARCH CENTER, Seoul, South Korea

Publisher

American College of Chest Physicians

Emtree Heading

*tuberculosis; relapse; risk; lung; human; patient; treatment duration; imaging; risk factor; lung tuberculosis; treatment failure; world health organization; sputum; treatment response; treatment outcome; male; female; diabetes mellitus; tomography; positron; morbidity; tissues; Mycobacterium tuberculosis; glucose metabolism; neoplasm; acid fast bacterium; drug therapy; sputum smear; antibiotic therapy; extensively drug resistant tuberculosis; diagnosis; monitor; granulomatous inflammation; drug resistant tuberculosis; thorax radiography; *fluorodeoxyglucose f 18; marker; isoniazid plus rifampicin; pyrazinamide; biological marker; ethambutol.

Link to the Ovid Full Text or citation:

Click here for full text options

35.

The analysis of the reasons of failures in treatment at MDR TB pationts.

Golubchikov P., Mishustin S., Zemlyanaya N.

European Respiratory Journal. Conference: European Respiratory Society Annual Congress 2011 Amsterdam Netherlands. Conference Start: 20110924 Conference End: 20110928. Conference Publication: (var.pagings). 38 (no pagination), 2011. Date of Publication: 01 Sep 2011.

AN: 72120943

In the Tomsk region from year 2004 to year 2008 there have been registered 88 cases of treatment failure of MDR TB within the program of Global Fund "Comprehensive strategy for reduction of burden of MDR tuberculosis". That has made 13,4% of total number of the patients registered for treatment. The given cohort had heavy social composition: 48 patients were unemployed, 5 patients were homeless, 52 persons suffered alcoholism, 7 drug addicts. In 10 patients on an initiation of treatment XDR was found. From 88 patients new cases - 14 persons, repeated - 74 persons. The number of reasons had their input into the lack of treatment success: * low adherence to controllable treatment 48,9% (the number of the missed doses in the intensive phase fluctuated from 19 to 38%), rendering patients some social support didn't influence their compliance: * serious lung damage - 36,3% * serious sideeffects - 12,5% (owing to accompanying diseases hepatitis, stomach ulcer, diabetes) * refusal to undergo surgical treatment - 2,3%. With the purpose to decrease treatment failures in patients with MDR TB since 2010 additional measures to the existing program have been developed: Expansion of the program "Companion" in rural areas on managing difficult patients. Strengthening of actions for the decrease in alcoholic dependence in patients undergoing chemotherapy. Uninterrupted provision of medicines for side-effect treatment in places of treatment. Wider use of surgical treatment. Optimization of early TB case finding of among the population.

Publisher

European Respiratory Society

Emtree Heading

*society; *European; human; patient; treatment failure; alcoholism; surgery; lung injury; stomach ulcer; social support; rural area; diseases; unemployment; hepatitis; drug dependence; population; diabetes mellitus; case finding; problem patient; chemotherapy; side effect; tuberculosis.

Link to the Ovid Full Text or citation:

Click here for full text options

36.

Dose response protective effect of inhaled corticosteroid on osteoporosis in patients with chronic obstructive pulmonary disease.

Kuo H.-C.

Respirology. Conference: 20th Congress of the Asian Pacific Society of Respirology Kuala Lumpur Malaysia. Conference Start: 20151203 Conference End: 20151206. Conference Publication: (var.pagings). 20 (pp 32), 2015. Date of Publication: December 2015.

AN: 72115886

Importance: Whether the use of inhaled corticosteroid (ICS) in chronic obstructive pulmonary disease (COPD) patients can protect the incidence of osteoporosis remains undetermined. Objective: to assess the incidence of osteoporosis in COPD patients with ICS use and without. Design: Retrospective cohort and population-based study Participants: From January 1996 through December 2011, from 23 million Taiwan Health Insurance database, we got newly diagnosed female patients with COPD (n = 33125, ICD-9-CM 491, 492, 496). We analysed the one million beneficiaries randomly selected from all insurant from 1997 to 2009, which has been demonstrated to be representative of the entire population. Patients were excluded if they were younger than 40 years of age, osteoporosis diagnosed prior to the diagnosis of COPD and cases of Asthma (ICD-9 CM code 493.X) before the index date. In total, 10723 patients with COPD were included in the cohort study group. Interventions: Users (n =812) and nonusers (n =9911) of ICS. Main outcome measurement: We used Cox Proportional hazards regression models to estimate hazard ratios (HRS) for osteoporosis incidence for ICS users vs non-users. Results: From 1996 to 2011 trace, the incidence rate of osteoporosis per100,000 person years is 4,395 in non-users and 2,709 in ICS users (HR: 0.73, 95% CI:0.63-084) adjusted for age, income, and medical diseases (pulmonary tuberculosis, bacterial pneumonia, bronchiectasis, pulmonary fibrosis, hypertension, and diabetes mellitus). The ICS accumulative dose is associated with lower risk for osteoporosis (0 mg ~ < 20 mg, HR: 0.80, 95% CI 0.65~0.97; >20 mg ~ < 60 mg, HR: 0.70, 95% CI 0.52~0.92; >60 mg, HR: 0.66, 95% CI 0.50~0.86; p < 0.0001). Conclusion and relevance: COPD patients with inhaled corticosteroid treatment exhibit associated with dose response protective effect for osteoporosis.

Institution

(Kuo) Kaohsiung Chang Gung Memorial Hospital, South Korea

Publisher

Blackwell Publishing

Emtree Heading

*osteoporosis; *patient; *human; *chronic obstructive lung disease; *Asian; *society; *dose response; population; ICD-9-CM; cohort analysis; income; asthma; Taiwan; diseases; lung tuberculosis; bacterial pneumonia; hazard ratio; proportional hazards model; diagnosis; data base; incidence; health insurance; risk; outcome assessment; diabetes mellitus; hypertension; bronchiectasis; lung fibrosis; female; corticosteroid therapy; ICD-9; *corticosteroid.

Link to the Ovid Full Text or citation:

Click here for full text options

37.

Do kinetics of interferon-gamma responses predict TB relapse?.

Chee C., Mar K.-W.K., Gan S.-H., Barkham T., Wang Y.-T.

European Respiratory Journal. Conference: European Respiratory Society Annual Congress 2011 Amsterdam Netherlands. Conference Start: 20110924 Conference End: 20110928. Conference Publication: (var.pagings). 38 (no pagination), 2011. Date of Publication: 01 Sep 2011.

AN: 72117861

Evidence that quantitative T-cell interferon-gamma responses to M. tuberculosis-specific antigens may reflect mycobacterial burden has raised interest in these responses as biomarkers for treatment outcomes. A cohort of 263 HIV-negative, culture-positive pulmonary TB patients enrolled between August 2005 and May 2007 who completed treatment and who were tested with QuantiFERON-Gold In-tube (QFT-IT) and T-SPOT.TB assays pre- and post-treatment were matched with the national TB registry for any renotification as at December 31 2010. Four patients relapsed with culture-positive, pansensitive pulmonary TB (relapse rate 1.5%). The time to relapse was 11, 17, 35 and 44 months. All were male. At the time of the first TB episode, their mean age was 45.4 years old, two were diabetic, two were cigarette smokers; all were baseline sputum AFB smearpositive; two had cavitary disease. All received directly-observed, 6-month chemotherapy with four first-line drugs in the intensive phase, followed by thrice weekly Rifampicin and Isoniazid in the continuation phase. All had one negative sputum culture (done once only) at two months of treatment. All four patients were IGRA positive at baseline. The QFT-IT was negative at end of treatment and 6 months post treatment-completion in three and four patients respectively. All remained T-SPOT positive at treatment completion, while two reverted their T-SPOT results 6 months thereafter. Compared to the whole treatment cohort, a lower proportion of relapsed patients tested IGRA positive at 6 months post-treatment completion (0% vs 46.3% for QFT-IT; 50% vs 79.2% for T-SPOT). Our observations suggest that the kinetics of interferon-gamma responses are not useful for predicting disease relapse.

Publisher

European Respiratory Society

Emtree Heading

*relapse; *European; *society; *kinetics; patient; human; enzyme linked immunospot assay; antigen specificity; recurrence risk; tuberculosis; register; T lymphocyte; tube; assay; interferon gamma release assay; sputum culture; diabetes mellitus; male; chemotherapy; treatment outcome; sputum; smoking; Human immunodeficiency virus; *gamma interferon; gold; isoniazid; rifampicin; biological marker.

Link to the Ovid Full Text or citation:

Click here for full text options

38.

Diabetes mellitus and pulmonary tuberculosis-Portuguese retrospective cohort.

Da Costa J.C., Gomes M., Duarte R.

European Respiratory Journal. Conference: European Respiratory Society Annual Congress 2015 Amsterdam Netherlands. Conference Start: 20150926 Conference End: 20150930. Conference Publication: ( var.pagings). 46 (no pagination), 2015. Date of Publication: 01 Sep 2015.

AN: 72108571

I: Diabetes mellitus (DM) is recognized as a risk factor to development and poor outcome on Tuberculosis (TB) patients. The goal of this study was to evaluate the association of DM and pulmonary TB in Portugal. M: Retrospective cohort study. National database was revised from 2008 to 2013. All patients with pulmonary TB reported were included. A global evaluation of the prevalence of DM on TB patients, presentation features and main TB treatment outcomes was performed. The trend of DM prevalence from 2008-13 was analysed. Statistical analysis was performed using IBM SPSS version 21; a significance level was defined to p<0.05. T student and Chi-square tests were used. R: Sample size: 11771 TB cases (10615 naive TB cases and 1156 retreatment cases). The prevalence of DM was 6.0%. There was a male predominance on DM group (DMg) and non-DM group (nDM) (71.0 vs 70.4%, p=0.725). The mean age was 60.5 (+/-15.2) on DMg and 46.1 (+/-17.3) years on nDM (p<0.001). There was no difference on the proportion of patients with retreatments on both groups (10.8% DMg vs 9.8% nDM, p=0.370), neither in the drug resistance profiles. There was a trend to severe treatment toxicity on DMg (4.6% vs 3.1%, p=0.06). Diabetic patients had more unsuccessful outcomes (treatment failure and death), p<0.001. From 2008 to 2013, DM prevalence on TB patients varied from 5.4% to 6.9%. D: DM patients had worse TB outcomes. This is of particular relevance since population is getting older and DM is increasing its prevalence.

Publisher

European Respiratory Society

Emtree Heading

*Portuguese (citizen); *human; *lung tuberculosis; *diabetes mellitus; *European; *society; patient; prevalence; treatment outcome; Portugal; statistical analysis; population; male; data base; risk factor; treatment failure; tuberculosis; retreatment; cohort analysis; chi square test; sample size; drug resistance; toxicity; diabetic patient; student; death; data analysis software.

Link to the Ovid Full Text or citation:

Click here for full text options

39.

Risk factors for multidrug-resistant tuberculosis among patients with pulmonary tuberculosis at the central chest institute of Thailand.

Chuchottaworn C., Thanachartwet V., Sangsayunh P., Than T.Z.M., Sahassananda D., Surabotsophon M., Desakorn V.

PLoS ONE. 10 (10) (no pagination), 2015. Article Number: e0139986. Date of Publication: 07 Oct 2015.

AN: 2015550285

There are limited data available on the risk factors for multidrug-resistant tuberculosis (MDR-TB). Therefore, we here conducted a retrospective matched case-control study among adults with pulmonary TB who received treatment at the Central Chest Institute of Thailand (CCIT) between January 2007 and December 2013, in order to determine the risk factors associated with MDR-TB among patients with pulmonary TB. We identified 145 patients with pulmonary MDR-TB (cases) and 145 patients with drug-sensitive pulmonary TB (controls). Multivariate analysis identified the independent risk factors for MDR-TB as follows: (1) > 2 episodes of prior pulmonary TB (odds ratio [OR] 39.72, 95% confidence interval (95% CI) 7.86-200.66), (2) duration of illness > 60 days (OR 3.08, 95% CI 1.52 -6.22), (3) sputum acid fast bacilli smear 3+ (OR 13.09, 95% CI 4.64-36.91), (4) presence of lung cavities (OR 3.82, 95% CI 1.89-7.73), and (5) presence of pleural effusion (OR 2.75, 95% CI 1.06-7.16). Prior pulmonary TB management with a non-category I regimen (P = 0.012) and having treatment failure or default as treatment outcomes (P = 0.036) were observed in a higher proportion among patients with MDR-TB. Particular characteristics of lung cavities, including the maximum diameter > 30 mm (P < 0.001), the number of cavities > 3 (P = 0.001), bilateral involvement (P < 0.001), and > 2 lung zones involved (P = 0.001) were more commonly observed in patients with MDR-TB. In conclusion, these clinical factors and chest radiographic findings associated with MDR-TB among patients with pulmonary TB may help physicians to provide proper management of cases for prevention of the development and spread of MDR-TB in future.

Institution

(Chuchottaworn, Sangsayunh) Division of Respiratory Medicine, Central Chest Institute of Thailand (CCIT), Nonthaburi, Thailand (Thanachartwet, Than, Desakorn) Department of Clinical Tropical Medicine, Faculty of Tropical Medicine, Mahidol University, Bangkok, Thailand

(Sahassananda) Information Technology Unit, Faculty of Tropical Medicine, Mahidol University, Bangkok, Thailand

(Surabotsophon) Division of Pulmonary and Critical Care Medicine, Ramkhamhaeng Hospital, Bangkok, Thailand

Publisher

Public Library of Science

Emtree Heading

acid fast bacterium; adult; article; atelectasis/di [Diagnosis]; cardiovascular disease; cohort analysis; controlled study; coughing; diabetes mellitus; disease duration; female; hemoptysis; human; Human immunodeficiency virus infection; lung disease; lung nodule; *lung tuberculosis/dr [Drug Resistance]; *lung tuberculosis/dt [Drug Therapy]; major clinical study; male; microbiological examination; mortality; *multidrug resistant tuberculosis/dr [Drug Resistance]; *multidrug resistant tuberculosis/dt [Drug Therapy]; Mycobacterium tuberculosis; pleura effusion; prevalence; retrospective study; risk factor; short course therapy; sputum smear; Thailand; thorax radiography; treatment failure; treatment outcome; ethambutol/dt [Drug Therapy]; isoniazid/dt [Drug Therapy]; pyrazinamide/dt [Drug Therapy]; rifampicin/dt [Drug Therapy].

Link to the Ovid Full Text or citation:

Click here for full text options

40.

Progressive Disseminated Histoplasmosis and HIV/AIDS: a Dermatological Perspective.

Chang P., Meaux T.

Current Fungal Infection Reports. 9 (4) (pp 213-219), 2015. Date of Publication: 01 Dec 2015.

AN: 2015463852

Progressive disseminated histoplasmosis (PDH) occurs as a result of internal spread of Histoplasma capsulatum from the lungs to organs rich in monocytes. This typically occurs in immunosuppressed patients, such as those with HIV/AIDS. Skin involvement is found in 10 % of all patients with PDH in the USA and in up to 25 % of those with AIDS. Crusted papular or plaque-like lesions are most common, with others being nodular, pustular, ulcerated, vegetative, acneiform, or wart-like. Lesions may be caused by the fungus itself or an immune response to the infection, such as erythema nodosum or erythema multiform. Histopathology demonstrates necrosis of the superficial dermal vessels. The differential diagnosis is broad and varies depending on the location and morphology of the lesion(s). Culture is the gold standard for diagnosis, and first-line treatment is amphotericin B for 1-2 weeks at a dose of 3 mg/kg/day.

Institution

(Chang, Meaux) Dermatology Service Social Security General Hospital-IGSS, 9. Street 7-55 Zone 9, Guatemala City 01009, Guatemala (Meaux) Dermatology Elective, Hospital General de Enfermedades IGSS, 9. Street 7-55 Zone 9, Guatemala City 01009, Guatemala

Publisher

Current Medicine Group LLC 1

Emtree Heading

*acquired immune deficiency syndrome/pc [Prevention]; *acquired immune deficiency syndrome/dt [Drug Therapy]; Africa; age; Ajellomyces capsulatus; alcoholism; Argentina; blastomycosis; Brazil; cancer radiotherapy; candidiasis/di [Diagnosis]; cellulitis/di [Diagnosis]; coccidioidomycosis; Colombia; complement fixation test; coughing; Crohn disease/di [Diagnosis]; cryptococcosis/di [Diagnosis]; cytosol; diabetes mellitus; disease association; drug tolerability; erythema multiforme; erythema nodosum/di [Diagnosis]; erythroderma/di [Diagnosis]; fever; fine needle aspiration biopsy; fungus culture; Germany; giant cell granuloma; gold standard; highly active antiretroviral therapy; histiocyte; histopathology; *histoplasmosis/co [Complication]; *histoplasmosis/di [Diagnosis]; *histoplasmosis/ep [Epidemiology]; *histoplasmosis/pc [Prevention]; *histoplasmosis/dt [Drug Therapy]; human; Human immunodeficiency virus infected patient; immunocompromised patient; immunodiffusion; immunosuppressive treatment; incidence; Italy; kidney transplantation; leishmaniasis/di [Diagnosis]; lung lavage; lymphocyte; lymphocytopenia; lymphoma/di [Diagnosis]; microscopy; molluscum contagiosum/di [Diagnosis]; multiplex polymerase chain reaction; Mycobacterium haemophilum; neutrophil; nonhuman; penicilliosis/di [Diagnosis]; physical examination; prevalence; primary cutaneous histoplasmosis; *progressive disseminated histoplasmosis/co [Complication]; *progressive disseminated histoplasmosis/di [Diagnosis]; *progressive disseminated histoplasmosis/ep [Epidemiology]; *progressive disseminated histoplasmosis/pc [Prevention]; *progressive disseminated histoplasmosis/dt [Drug Therapy]; pyoderma gangrenosum/di [Diagnosis]; randomized controlled trial(topic); relapse; review; risk factor; rosacea/di [Diagnosis]; sensitivity and specificity; Sjoegren syndrome; smear; squamous cell carcinoma/di [Diagnosis]; syphilis/di [Diagnosis]; systemic mycosis/di [Diagnosis]; treatment outcome; Trichosporon; Trichosporon inkin; tuberculosis/di [Diagnosis]; United States; weight reduction; amphotericin B/ct [Clinical Trial]; amphotericin B/dt [Drug Therapy]; amphotericin B deoxycholate/ct [Clinical Trial]; amphotericin B deoxycholate/dt [Drug Therapy]; C reactive protein/ec [Endogenous Compound]; fluconazole/ct [Clinical Trial]; fluconazole/dt [Drug Therapy]; integrase inhibitor/dt [Drug Therapy]; itraconazole/ct [Clinical Trial]; itraconazole/dt [Drug Therapy]; methenamine; nonnucleoside reverse transcriptase inhibitor/dt [Drug Therapy]; posaconazole/ct [Clinical Trial]; posaconazole/dt [Drug Therapy]; proteinase inhibitor/dt [Drug Therapy]; RNA directed DNA polymerase inhibitor/dt [Drug Therapy]; voriconazole/ct [Clinical Trial]; voriconazole/dt [Drug Therapy].

Link to the Ovid Full Text or citation:

Click here for full text options

41.

Trends in the incidence and causes of severe visual impairment and blindness in children from Israel.

Mezer E., Chetrit A., Kalter-Leibovici O., Kinori M., Ben-Zion I., Wygnanski-Jaffe T.

Journal of AAPOS. 19 (3) (pp 260-265e1), 2015. Date of Publication: 01 Jun 2015.

AN: 2015504062

Purpose To describe trends in the incidence and causes of legal childhood blindness in Israel, one of the few countries worldwide that maintain a national registry of the blind. Methods We performed a historical cohort study of annual reports of the National Registry of the Blind (NRB) between 1999 and 2013. All data regarding demographic information, year of registration and cause of blindness of children 0-18 years of age registered for blind certification were obtained from the annual reports of the NRB. Causes of legal blindness analyzed were optic atrophy, retinitis pigmentosa, retinopathy of prematurity (ROP), albinism, other retinal disorders, cataract, and glaucoma. The main outcome measure was the incidence of new cases of certified legal blindness. Results The incidence of newly registered legally blind children in Israel almost halved from 7.7 per 100,000 in 1999 to 3.1 per 100,000 in 2013. The decline was mainly attributable to a decreased incidence of blindness resulting from retinitis pigmentosa and ROP. The incidence of registered cases due to cerebral visual impairment increased. Conclusions During the past decade the incidence of severe childhood visual impairment and blindness declined in Israel. A continuous decline in consanguineous marriages among the Jewish and Arab populations in Israel may have contributed to the decrease in the rate of vision loss due to retinitis pigmentosa in children.

Author NameID

Mezer, Eedy; ORCID: <http://orcid.org/0000-0002-2818-7538>

Institution

(Mezer) Department of Ophthalmology, Rambam Health Care Campus, Haifa, Israel (Mezer) Ruth and Bruce Rappaport Faculty of Medicine, Technion-Israel Institute of Technology, Haifa, Israel

(Chetrit, Kalter-Leibovici) Unit of Cardiovascular Epidemiology, Gertner Institute for Epidemiology and Health Policy Research, Tel Hashomer, Israel

(Kalter-Leibovici, Kinori, Ben-Zion, Wygnanski-Jaffe) Sackler Faculty of Medicine, Tel Aviv University, Tel Aviv, Israel

(Kinori, Ben-Zion, Wygnanski-Jaffe) Goldschleger Eye Institute, Sheba Medical Center, Tel Hashomer, Israel

Publisher

Mosby Inc.

Emtree Heading

adolescent; adult; age related macular degeneration; albinism; amblyopia; anophthalmia; article; *blindness/co [Complication]; *blindness/ep [Epidemiology]; *blindness/et [Etiology]; cataract; child; *childhood disease/co [Complication]; *childhood disease/ep [Epidemiology]; *childhood disease/et [Etiology]; cohort analysis; congenital cataract/cn [Congenital Disorder]; congenital glaucoma/cn [Congenital Disorder]; cornea opacity; demography; diabetic retinopathy; disease severity; female; glaucoma; human; incidence; infant; Israel; keratoconus; lung tuberculosis; major clinical study; male; microphthalmia; myopic maculopathy; newborn; nystagmus; optic nerve atrophy; outcome assessment; primary glaucoma; priority journal; retina detachment; retina disease; retinitis pigmentosa; retrolental fibroplasia; secondary glaucoma; senile cataract; uveitis; *visual impairment/co [Complication]; *visual impairment/ep [Epidemiology]; *visual impairment/et [Etiology]; vitreous hemorrhage.

Link to the Ovid Full Text or citation:

Click here for full text options

42.

Years lived with disability (YLDs) for 1160 sequelae of 289 diseases and injuries 1990-2010: A systematic analysis for the Global Burden of Disease Study 2010.

Vos T., Flaxman A.D., Naghavi M., Lozano R., Michaud C., Ezzati M., Shibuya K., Salomon J.A., Abdalla S., Aboyans V., Abraham J., Ackerman I., Aggarwal R., Ahn S.Y., Ali M.K., Alvarado M., Anderson H.R., Anderson L.M., Andrews K.G., Atkinson C., Baddour L.M., Bahalim A.N., Barker-Collo S., Barrero L.H., Bartels D.H., Basanez M.-G., Baxter A., Bell M.L., Benjamin E.J., Bennett D., Bernabe E., Bhalla K., Bhandari B., Bikbov B., Abdulhak A.B., Birbeck G., Black J.A., Blencowe H., Blore J.D., Blyth F., Bolliger I., Bonaventure A., Boufous S., Bourne R., Boussinesq M., Braithwaite T., Brayne C., Bridgett L., Brooker S., Brooks P., Brugha T.S., Bryan-Hancock C., Bucello C., Buchbinder R., Buckle G., Budke C.M., Burch M., Burney P., Burstein R., Calabria B., Campbell B., Canter C.E., Carabin H., Carapetis J., Carmona L., Cella C., Charlson F., Chen H., Cheng A.T.-A., Chou D., Chugh S.S., Coffeng L.E., Colan S.D., Colquhoun S., Colson K.E., Condon J., Connor M.D., Cooper L.T., Corriere M., Cortinovis M., De Vaccaro K.C., Couser W., Cowie B.C., Criqui M.H., Cross M., Dabhadkar K.C., Dahiya M., Dahodwala N., Damsere-Derry J., Danaei G., Davis A., De Leo D., Degenhardt L., Dellavalle R., Delossantos A., Denenberg J., Derrett S., Des Jarlais D.C., Dharmaratne S.D., Dherani M., Diaz-Torne C., Dolk H., Dorsey E.R., Driscoll T., Duber H., Ebel B., Edmond K., Elbaz A., Ali S.E., Erskine H., Erwin P.J., Espindola P., Ewoigbokhan S.E., Farzadfar F., Feigin V., Felson D.T., Ferrari A., Ferri C.P., Fevre E.M., Finucane M.M., Flaxman S., Flood L., Foreman K., Forouzanfar M.H., Fowkes F.G.R., Franklin R., Fransen M., Freeman M.K., Gabbe B.J., Gabriel S.E., Gakidou E., Ganatra H.A., Garcia B., Gaspari F., Gillum R.F., Gmel G., Gonzalez-Medina D., Gosselin R., Grainger R., Grant B., Groeger J., Guillemin F., Gunnell D., Gupta R., Haagsma J., Hagan H., Halasa Y.A., Hall W., Haring D., Haro J.M., Harrison J.E., Havmoeller R., Hay R.J., Higashi H., Hill C., Hoen B., Hoffman H., Hotez P.J., Hoy D., Huang J.J., Ibeanusi S.E., Jacobsen K.H., James S.L., Jarvis D., Jasrasaria R., Jayaraman S., Johns N., Jonas J.B., Karthikeyan G., Kassebaum N., Kawakami N., Keren A., Khoo J.-P., King C.H., Knowlton L.M., Kobusingye O., Koranteng A., Krishnamurthi R., Laden F., Lalloo R., Laslett L.L., Lathlean T., Leasher J.L., Lee Y.Y., Leigh J., Levinson D., Lim S.S., Limb E., Lin J.K., Lipnick M., Lipshultz S.E., Liu W., Loane M., Ohno S.L., Lyons R., Ma J., Mabweijano J., MacIntyre M.F., Malekzadeh R., Mallinger L., Manivannan S., Marcenes W., March L., Margolis D.J., Marks G.B., Marks R., Matsumori A., Matzopoulos R., Mayosi B.M., McAnulty J.H., McDermott M.M., McGill N., McGrath J., Medina-Mora M.E., Meltzer M., Mensah G.A., Merriman T.R., Meyer A.-C., Miglioli V., Miller M., Miller T.R., Mitchell P.B., Mock C., Mocumbi A.O., Moffitt T.E., Mokdad A.A., Monasta L., Montico M., Moran A., Morawska L., Mori R., Murdoch M.E., Mwaniki M.K., Naidoo K., Nair M.N., Naldi L., Narayan K.M.V., Nelson P.K., Nelson R.G., Nevitt M.C., Newton C.R., Nolte S., Norman P., Norman R., O'Donnell M., O'Hanlon S., Olives C., Omer S.B., Ortblad K., Osborne R., Ozgediz D., Page A., Pahari B., Pandian J.D., Rivero A.P., Patten S.B., Pearce N., Padilla R.P., Perez-Ruiz F., Perico N., Pesudovs K., Phillips D., Phillips M.R., Pierce K., Pion S., Polanczyk G.V., Polinder S., Pope III C.A., Popova S., Porrini E., Pourmalek F., Prince M., Pullan R.L., Ramaiah K.D., Ranganathan D., Razavi H., Regan M., Rehm J.T., Rein D.B., Remuzzi G., Richardson K., Rivara F.P., Roberts T., Robinson C., De Leon F.R., Ronfani L., Room R., Rosenfeld L.C., Rushton L., Sacco R.L., Saha S., Sampson U., Sanchez-Riera L., Sanman E., Schwebel D.C., Scott J.G., Segui-Gomez M., Shahraz S., Shepard D.S., Shin H., Shivakoti R., Singh D., Singh G.M., Singh J.A., Singleton J., Sleet D.A., Sliwa K., Smith E., Smith J.L., Stapelberg N.J.C., Steer A., Steiner T., Stolk W.A., Stovner L.J., Sudfeld C., Syed S., Tamburlini G., Tavakkoli M., Taylor H.R., Taylor J.A., Taylor W.J., Thomas B., Thomson W.M., Thurston G.D., Tleyjeh I.M., Tonelli M., Towbin J.A., Truelsen T., Tsilimbaris M.K., Ubeda C., Undurraga E.A., Van Der Werf M.J., Van Os J., Vavilala M.S., Venketasubramanian N., Wang M., Wang W., Watt K., Weatherall D.J., Weinstock M.A., Weintraub R., Weisskopf M.G., Weissman M.M., White R.A., Whiteford H., Wiebe N., Wiersma S.T., Wilkinson J.D., Williams H.C., Williams S.R.M., Witt E., Wolfe F., Woolf A.D., Wulf S., Yeh P.-H., Zaidi A.K.M., Zheng Z.-J., Zonies D., Lopez A.D., Murray C.J.L., Moradi-Lakeh M.

The Lancet. 380 (9859) (pp 2163-2196), 2012. Date of Publication: 2012.

AN: 2012732549

Background Non-fatal health outcomes from diseases and injuries are a crucial consideration in the promotion and monitoring of individual and population health. The Global Burden of Disease (GBD) studies done in 1990 and 2000 have been the only studies to quantify non-fatal health outcomes across an exhaustive set of disorders at the global and regional level. Neither effort quantified uncertainty in prevalence or years lived with disability (YLDs). Methods Of the 291 diseases and injuries in the GBD cause list, 289 cause disability. For 1160 sequelae of the 289 diseases and injuries, we undertook a systematic analysis of prevalence, incidence, remission, duration, and excess mortality. Sources included published studies, case notification, population-based cancer registries, other disease registries, antenatal clinic serosurveillance, hospital discharge data, ambulatory care data, household surveys, other surveys, and cohort studies. For most sequelae, we used a Bayesian meta-regression method, DisMod-MR, designed to address key limitations in descriptive epidemiological data, including missing data, inconsistency, and large methodological variation between data sources. For some disorders, we used natural history models, geospatial models, back-calculation models (models calculating incidence from population mortality rates and case fatality); or registration completeness models; (models adjusting for incomplete registration with health-system access and other covariates). Disability weights for 220 unique health states were used to capture the severity of health loss. YLDs by cause at age, sex, country, and year levels were adjusted for comorbidity with simulation methods. We included uncertainty estimates at all stages of the analysis. Findings Global prevalence for all ages combined in 2010 across the 1160 sequelae ranged from fewer than one case per 1 million people to 350 000 cases per 1 million people. Prevalence and severity of health loss were weakly correlated (correlation coefficient -0 37). In 2010, there were 777 million YLDs from all causes, up from 583 million in 1990. The main contributors to global YLDs were mental and behavioural disorders, musculoskeletal disorders, and diabetes or endocrine diseases. The leading specific causes of YLDs were much the same in 2010 as they were in 1990: low back pain, major depressive disorder, iron-deficiency anaemia, neck pain, chronic obstructive pulmonary disease, anxiety disorders, migraine, diabetes, and falls. Age-specific prevalence of YLDs increased with age in all regions and has decreased slightly from 1990 to 2010. Regional patterns of the leading causes of YLDs were more similar compared with years of life lost due to premature mortality. Neglected tropical diseases, HIV/AIDS, tuberculosis, malaria, and anaemia were important causes of YLDs in sub-Saharan Africa. Interpretation Rates of YLDs per 100 000 people have remained largely constant over time but rise steadily with age. Population growth and ageing have increased YLD numbers and crude rates over the past two decades. Prevalences of the most common causes of YLDs, such as mental and behavioural disorders and musculoskeletal disorders, have not decreased. Health systems will need to address the needs of the rising numbers of individuals with a range of disorders that largely cause disability but not mortality. Quantification of the burden of non-fatal health outcomes will be crucial to understand how well health systems are responding to these challenges. Effective and affordable strategies to deal with this rising burden are an urgent priority for health systems in most parts of the world.

Institution

(Baxter, Erskine, Ferrari, Keren, Saha, Whiteford) Queensland Centre for Mental Health Research, Australia (Vos, Blore, Charlson, Hay, Leasher, Norman, Page, Lopez) School Population of Healtho, Australia

(Ganatra, Halasa, Patten) Centre for Clinical Research, Australia

(McGill) Queensland Brain Institute, University of Queensland, Brisbane, QLD, Australia

(Flaxman, Naghavi, Lozano, Ahn, Alvarado, Andrews, Atkinson, Bolliger, Burstein, Campbell, Colson, Delossantos, Dharmaratne, Forouzanfar, Fransen, Gabriel, Gmel, Hall, Haro, Jarvis, Jayaraman, Levinson, Loane, Mabweijano, Malekzadeh, Moffitt, Nair, Ortblad, Phillips, Pierce, Ranganathan, Roberts, Rosenfeld, Sanman, Wang, Wulf, Murray) Institute for Health Metrics and Evaluation, University of Washington, 2301 Fifth Avenue, Seattle, WA 98121, United States

(Anderson) Department of Epidemiology, School of Public Health, United States

(Karthikeyan) Department of Anesthesiology and Pain Medicine, United States

(Couser, Duber, Ebel, Mitchell, Olives, Rivara, Vavilala) University of Washington, Seattle, WA, United States

(Michaud) China Medical Board, Boston, MA, United States

(Ezzati) MRCHPA Centre for Environment and Health, Department of Epidemiology and Biostatistics, School of Public Health, United Kingdom

(Basanez, Burney, Foreman, James, O'Hanlon, Rushton) Imperial College London, London, United Kingdom

(Shibuya) Department of Global Health Policy, Japan

(Kassebaum) University of Tokyo, Tokyo, Japan

(Finucane) Department of Biostatistics, United States

(Salomon) School of Public Health, United States

(Danaei, Krishnamurthi, Limb, Miglioli, Singh, Sudfeld, Tavakkoli, Weisskopf, White) 1Department of Epidemiology, United States

(Lipshultz) Harvard Humanitarian Initiative, United States

(King) Boston Childrenaes Hospital, United States

(Colan) Brigham and Womenaes Hospital, United States

(Jasrasaria) Harvard Medical School, Harvard University, Boston, MA, United States

(Bartels) Sudanese Public Health Consultancy Group, Sudan

(Abdalla) Department of Cardiology, Dupuytren University Hospital, Limoges, France

(Aboyans) University of Texas, San Antonio, TX, United States

(Abraham) Centre for International Child Health, Australia

(Steer) Department of Pediatrics, Australia

(Weintraub) Centre for Health Policy, Programs and Economics, Australia

(Degenhardt) School of Population Health, Australia

(Room) University of Melbourne, Melbourne, VIC, Australia

(Ackerman, Brooks, Marks, Taylor) Sanjay Gandhi Postgraduate Institute of Medical Sciences, Lucknow, India

(Aggarwal) Schools of Public Health and Medicine, United States

(Omer) Emory University, Atlanta, GA, United States

(Ali) St Georgeaes University of London, London, United Kingdom

(Dabhadkar, Gakidou, Narayan) Mayo Clinic, Rochester, MN, United States

(Anderson, Lim) Independent Consultant, Geneva, Switzerland

(Gunnell) University of Auckland, Auckland, New Zealand

(Baddour, Erwin, Gabbe) Department of Industrial Engineering, School of Engineering, Pontificia Universidad Javeriana, Bogota, Colombia

(Bahalim) Global Partners in Anesthesia and Surgery, United States

(Barker-Collo) Yale University, New Haven, CT, United States

(Barrero) School of Medicine, United States

(Ozgediz) Boston University, Boston, MA, United States

(Bell, Hoy) Clinical Trial Service Unit and Epidemiological Studies Unit, United Kingdom

(Felson) University of Oxford, Oxford, United Kingdom

(Benjamin) Dental Institute, United Kingdom

(Bennett) Hospital NHS Trust, United Kingdom

(Bernabe) Institute of Psychiatry, Kingaes College London, London, United Kingdom

(Havmoeller) Institute of Dentistry, United Kingdom

(Prince) Queen Mary University of London, London, United Kingdom

(Dahiya) Moscow State University of Medicine and Dentistry, Research Institute of Transplantology and Artificial Organs, Moscow, Russian Federation

(Bhandari) King Fahad Medical City, Riyadh, Saudi Arabia

(Manivannan) Michigan State University, East Lansing, MI, United States

(Bikbov) MRC Epidemiology Unit, Cambridge, United Kingdom

(Abdulhak) London School of Hygiene and Tropical Medicine, London, United Kingdom

(Tleyjeh) Department of Rheumatololgy, Northern Clinical School, Australia

(Birbeck) Faculty of Health Sciences, Australia

(Black) Institute of Bone and Joint Research, Australia

(Blencowe, Brooker, Edmond, Pearce, Pullan, Smith) Sydney School of Public Health, Australia

(Smith) University of Sydney, Sydney, NSW, Australia

(Marcenes, Sanchez-Riera) Inserm, Paris, France

(Driscoll, Lee) Transport and Road Safety Research, Australia

(Blyth, Bridgett, Cross, Margolis, McDermott) National Drug and Alcohol Research Centre, Australia

(Bonaventure, Elbaz) University of New South Wales, Sydney, NSW, Australia

(Boufous) Vision and Eye Research Unit, Anglia Ruskin University, Cambridge, United Kingdom

(Calabria, Degenhardt, Nelson, Singleton) Institut de Recherche pour le Developpement, Martinique

(Bucello, Miller) Moorfields Eye Hospital, London, United Kingdom

(Bourne) University of Cambridge, Cambridge, United Kingdom

(Boussinesq) University of Leicester, Leicester, United Kingdom

(Pion) Flinders University, Adelaide, SA, Australia

(Braithwaite) Cabrini Institute, Malvern, VIC, Australia

(Brayne, Richardson) Department of Epidemiology and Preventive Medicine, Australia

(Brugha) Monash University, Melbourne, VIC, Australia

(Bryan-Hancock) Bloomberg School of Public Health, United States

(Flood, Haro, Laslett, Pesudovs) Johns Hopkins University, Baltimore, MD, United States

(Buchbinder) Texas AandM University, College Station, TX, United States

(Freeman) Great Ormond Street Hospital, London, United Kingdom

(Buchbinder, Hotez) Washington University, St Louis, MO, United States

(Buckle, Mallinger) University of Oklahoma, Health Sciences Center, Oklahoma City, OK, United States

(Dorsey, Shivakoti) Telethon Institute for Child Health Research, Centre for Child Health Research, Australia

(Budke) University of Western Australia, Perth, WA, Australia

(Burch) Universidad Camilo Jose Cela, Villanueva de la Canada, Spain

(Canter) Mario Negri Institute for Pharmacological Research, Bergamo, Italy

(Carabin) National Institute of Environmental Health Sciences, Research Triangle Park, NC, United States

(Carapetis) Institute of Biomedical Sciences, Academia Sinica, Taipei, Taiwan (Republic of China)

(Norman) Heart Institute, United States

(Carmona) Cedars-Sinai Medical Center, Los Angeles, CA, United States

(Cella, Cortinovis, Garcia, Meyer, Perico, Remuzzi) Department of Public Health, United States

(Chen) Erasmus MC, University Medical Center Rotterdam, Rotterdam, Netherlands

(Cheng) Menzies School of Health Research, Darwin, NT, Australia

(Harrison) National Health Services, Fife, Edinburgh, United Kingdom

(Chugh) University of Edinburgh, Edinburgh, United Kingdom

(Polinder) University of the Witwatersrand, Johannesburg, South Africa

(Coffeng, Gupta, Stolk) Loyola University Medical School, Chicago, IL, United States

(Colquhoun, Condon) School of Public Health Sciences, Wake Forest University, Winston-Salem, NC, United States

(Connor, Fowkes) Hospital Dr Gustavo N Collado, Puerto Chitre, Panama

(Connor, Fevre) Victorian Infectious Diseases Reference Laboratory, Melbourne, VIC, Australia

(Cooper) University of California, San Diego, San Diego, CA, United States

(Corriere) University of Pennsylvania, Philadelphia, PA, United States

(De Vaccaro) Building and Road Research Institute, Kumasi, Ghana

(Cowie) MRC Hearing and Communication Group, Manchester, United Kingdom

(Criqui, Denenberg) School of Dentistry and Oral Health, Australia

(Dahodwala, March) Population and Social Health Research Program, Australia

(Damsere-Derry) Griffith University, Brisbane, QLD, Australia

(Davis, Laden) Denver VA Medical Center, Denver, CO, United States

(Laden) University of Otago, Dunedin, New Zealand

(De Leo, Stapelberg) Beth Israel Medical Center, New York City, NY, United States

(Dellavalle) University of Peradeniya, Peradeniya, Sri Lanka

(Derrett, Gosselin, Mensah, Taylor, Thomson) University of Liverpool, Liverpool, United Kingdom

(Des Jarlais) Hospital de la Santa Creu i Sant Pau, Barcelona, Spain

(Dharmaratne) University of Ulster, Ulster, United Kingdom

(Dherani) Federal Ministry of Health, Khartoum, Sudan

(Diaz-Torne) Hospital Maciel, Montevideo, Uruguay

(Dolk, Liu) Emerald Public Health Consulting Services Ltd, Abuja, Nigeria

(Moffitt) Digestive Disease Research Center, Iran, Islamic Republic of

(Espindola, Farzadfar, Moradi-Lakeh) Tehran University of Medical Sciences, Tehran, Iran, Islamic Republic of

(Ewoigbokhan) National Institute for Stroke and Applied Neurosciences, New Zealand

(MacIntyre) Auckland Technical University, Auckland, New Zealand

(Montico) Medical School, Brazil

(Feigin) Federal University of Sao Paulo, Sao Paulo, Brazil

(Koranteng, Witt) Carnegie Mellon University, Pittsburgh, PA, United States

(Polanczyk) James Cook University, Townsville, QLD, Australia

(Ferri) Howard University, College of Medicine, Washington, DC, United States

(Watt) Addiction Info Switzerland, Lausanne, Switzerland

(Gaspari) Department of Epidemiology and Biostatistics, United States

(Gillum) University of California, San Francisco, San Francisco, CA, United States

(Nevitt) College of Medicine, SUNY Downstate Medical Center, Brooklyn, NY, United States

(Gonzalez-Medina, Lin, Merriman, Robinson) National Center for Injury Prevention and Control, United States

(Grant) Centers for Disease Control and Prevention, Atlanta, GA, United States

(Sleet) Universite de Lorraine, Nancy, France

(Wiersma) University of Bristol, Bristol, United Kingdom

(Groeger) New York University, New York City, NY, United States

(Guillemin) Brandeis University, Waltham, MA, United States

(Haagsma) Parc Sanitari Sant Joan de Deu, CIBERSAM, Universitat de Barcelona, Sant Boi de Llobregat, Spain

(Thurston) Karolinska University Hospital, Stockholm, Sweden

(Hagan) Queen Elizabeth Hospital, Adelaide, SA, Australia

(Shahraz) Universite de Franche-Comte, Besancon, France

(Shepard) Centre Hospitalier Regional, Universitaire de Besancon, Besancon, France

(Undurraga) National Institute on Deafness and Other Communication Disorders, United States

(Haring) National Institute of Diabetes and Digestive and Kidney Diseases, National Institutes of Health, Bethesda, MD, United States

(Harrison) National School of Tropical Medicine, Baylor College of Medicine, Waco, TX, United States

(Higashi) University of Port Harcourt, Port Harcourt, Nigeria

(Hill) George Mason University, Fairfax, VA, United States

(Hill) Department of Ophthalmology, Medical Faculty Mannheim, Ruprecht Karls University, Heidelberg, Germany

(Hoen) All India Institute of Medical Sciences, New Delhi, India

(Nelson) Department of Cardiology, Hebrew University Hadassah Medical School, Jerusalem, Israel

(Hoffman) Case Western Reserve University, Cleveland, OH, United States

(Huang) School of Public Health, Makerere University, Kampala, Uganda

(Ibeanusi, Johns) University of South Africa, Johannesburg, South Africa

(Jonas) Kwame Nkrumah University of Science and Technology, Kumasi, Ghana

(Kawakami) University of Tasmania, TAS, Australia

(Khoo) Nova Southeastern University, Fort Lauderdale, FL, United States

(Knowlton) Ministry of Health, Jerusalem, Israel

(Knowlton) Miller School of Medicine, University of Miami, Miami, FL, United States

(Kobusingye) Swansea University, Swansea, United Kingdom

(Lalloo) Mulago Hospital, Kampala, Uganda

(Lathlean) Asian Pacific Society of Cardiology, Kyoto, Japan

(Leigh, Lipnick, Sacco, Wilkinson) Medical Research Council, Tygerberg, South Africa

(Ohno) Hatter Institute, South Africa

(Lyons) Department of Medicine, South Africa

(Marks) University of Cape Town, Cape Town, South Africa

(Matsumori) Legacy Health System, Portland, OR, United States

(Sliwa) Northwestern University Feinberg, School of Medicine, Evanston, IL, United States

(Meltzer) National Institute on Psychiatry Ramon de la Fuente, Mexico City, Mexico

(Matsumori, Matzopoulos) Thomas Jefferson University, Philadelphia, PA, United States

(Mayosi) Pacific Institute for Research and Evaluation, Calverton, MD, United States

(McAnulty) National Institute of Health, Maputo, Mozambique

(McGrath) University Eduardo Mondlane, Maputo, Mozambique

(Medina-Mora) Duke University, Durham, NC, United States

(Miller) Institute for Maternal and Child Health, IRCCS Burlo Garofolo, Trieste, Italy

(Mock) Columbia University, New York City, NY, United States

(Mock) Queensland University of Technology, Brisbane, QLD, Australia

(Mocumbi, Mokdad, Monasta, Ronfani, Tamburlini) National Center for Child Health and Development, Tokyo, Japan

(Moran, Weissman) Watford General Hospital, Watford, United Kingdom

(Morawska) Kemri-Wellcome Trust, Kilifi, Kenya

(Mori) AVRI, University of KwaZulu-Natal, Durban, South Africa

(Murdoch) Centro Studi GISED, Bergamo, Italy

(Mwaniki) Charite- Universitatsmedizin Berlin, Berlin, Germany

(Naidoo) HRB-Clinical Research Facility, National University of Ireland Galway, Galway, Ireland

(Naldi) Deakin University, Melbourne, VIC, Australia

(Newton) B P Koirala Institute of Health Sciences, Dharan, Nepal

(O'Donnell) Betty Cowan Research and Innovation Center, Ludhiana, India

(Osborne) Hospital Juan XXIII, La Paz, Bolivia

(Pahari) University of Calgary, Calgary, AB, Canada

(Pandian) Instituto Nacional de Enfermedades Respiratorias, Mexico City, Mexico

(Rivero) Hospital Universitario Cruces, Barakaldo, Spain

(Patten) Shanghai Mental Health Center, School of Medicine, China

(Padilla) School of Public Health, Shanghai Jiao Tong University, Shanghai, China

(Perez-Ruiz) Brigham Young University, Provo, UT, United States

(Phillips) Centre for Addiction and Mental Health, Toronto, ON, Canada

(Zheng) Hospital Universitario de Canarias, Tenerif, Spain

(Pope III) Faculty of Medicine, School of Population and Public Health, University of British Columbia, Vancouver, BC, Canada

(Popova) Vector Control Research Centre, Pondicherry, India

(Rehm) Center for Disease Analysis, Louisville, CO, United States

(Porrini) University of California, Berkeley, Berkeley, CA, United States

(Pourmalek) NORC, University of Chicago, Chicago, IL, United States

(Ramaiah) Complejo Hospitalario Caja De Seguro Social, Panama City, Panama

(Razavi) Centre for Alcohol Policy Research, Turning Point Alcohol and Drug Centre, Fitzroy, SA, Australia

(Regan, Sampson) Vanderbilt University, Nashville, TN, United States

(Rein, Schwebel) University of Alabama at Birmingham, Birmingham, AL, United States

(De Leon) Ministry of Interior, Madrid, Spain

(Room) Health Canada, Ottawa, ON, Canada

(Stovner) Queens Medical Center, Honolulu, HI, United States

(Singh) Department of Neuroscience, Norway

(Segui-Gomez, Taylor) Norwegian University of Science and Technology, Trondheim, Norway

(Shin) Aga Khan University, Karachi, Pakistan

(Tonelli) Drexel University, School of Public Health, Philadelphia, PA, United States

(Steiner, Tsilimbaris, Wiebe) Alberta Kidney Disease Network, Canada

(Towbin, Zaidi) University of Alberta, Edmonton, AB, Canada

(Truelsen) Cincinnati Childrenaes Hospital, Cincinnati, OH, United States

(Syed) Department of Neurology, Copenhagen University Hospital, Herlev, Denmark

(Ubeda) University of Crete Medical School, Crete, Greece

(Van Der Werf) Instituto Nacional de Epidemiologia, ANLIS, Malbran, Argentina

(Van Os) KNCV Tuberculosis Foundation, The Hague, Netherlands

(Thomas) Maastricht University Medical Centre, Maastricht, Netherlands

(Wang) National University of Singapore, Singapore

(Weinstock) Beijing Neurosurgical Institute, Capital Medical University, Beijing, China

(Grainger) Brown University, Providence, RI, United States

(Venketasubramanian, Williams) Royal Childrenaes Hospital, Critical Care and Neurosciences Theme, Murdoch Childrenaes Research Institute, Melbourne, VIC, Australia

(Williams, Woolf) University of Nottingham, Nottingham, United Kingdom

(Williams) Hollywood Orthopaedic Group, Perth, WA, Australia

(Wolfe) Arthritis Research, Wichita, KS, United States

(Weintraub) Royal Cornwall Hospital, Truro, United Kingdom

(Weintraub, Yeh) London School of Economics, London, United Kingdom

(Zonies) Landstuhl Regional Medical Center, Landstuhl, Germany

(Ma) National Center for Non-Communicable Disease Control and Prevention, China Centers for Disease Control, Beijing, China

Publisher

Lancet Publishing Group

Emtree Heading

acquired immune deficiency syndrome/co [Complication]; acquired immune deficiency syndrome/dt [Drug Therapy]; acquired immune deficiency syndrome/ep [Epidemiology]; age distribution; aging; anemia/co [Complication]; anemia/ep [Epidemiology]; antiviral therapy; anxiety disorder/co [Complication]; anxiety disorder/ep [Epidemiology]; article; behavior disorder/co [Complication]; behavior disorder/ep [Epidemiology]; chronic obstructive lung disease/co [Complication]; chronic obstructive lung disease/ep [Epidemiology]; comorbidity; diabetes mellitus/co [Complication]; diabetes mellitus/ep [Epidemiology]; *disability; disability severity; disease duration; endocrine disease/co [Complication]; endocrine disease/ep [Epidemiology]; falling; health care system; human; Human immunodeficiency virus infection/co [Complication]; Human immunodeficiency virus infection/ep [Epidemiology]; *iatrogenic disease/co [Complication]; *iatrogenic disease/ep [Epidemiology]; incidence; iron deficiency anemia/co [Complication]; iron deficiency anemia/ep [Epidemiology]; low back pain/co [Complication]; low back pain/ep [Epidemiology]; major depression/co [Complication]; major depression/ep [Epidemiology]; malaria/co [Complication]; malaria/ep [Epidemiology]; mental disease/co [Complication]; mental disease/ep [Epidemiology]; migraine/co [Complication]; migraine/ep [Epidemiology]; mortality; musculoskeletal disease/co [Complication]; musculoskeletal disease/ep [Epidemiology]; neck pain/co [Complication]; neck pain/ep [Epidemiology]; outcome assessment; population growth; prevalence; priority journal; remission; systematic review; tropical disease/co [Complication]; tropical disease/ep [Epidemiology]; tuberculosis/co [Complication]; tuberculosis/ep [Epidemiology]; *years lived with disability; anti human immunodeficiency virus agent/dt [Drug Therapy].

Link to the Ovid Full Text or citation:

Click here for full text options

43.

Outcomes of open repair of mycotic descending thoracic and thoracoabdominal aortic aneurysms.

Lau C., Gaudino M., De Biasi A.R., Munjal M., Girardi L.N.

Annals of Thoracic Surgery. 100 (5) (pp 1712-1717), 2015. Date of Publication: November 2015.

AN: 2015323381

Background The purpose of this study was to evaluate the short- and intermediate-term outcomes of open repair of mycotic thoracic and thoracoabdominal aneurysms. Contemporary surgical and perioperative techniques were utilized. Methods From November 1997 to May 2014, 14 consecutive patients underwent open repair of descending thoracic (n = 9, 64.3%) and thoracoabdominal (n = 5, 35.7%) mycotic aortic aneurysms. All procedures were performed through the left side of the chest. Infected tissue was completely debrided and excised. Aortic continuity was restored in situ with a Dacron prosthesis (Macquet Corp, Oakland, NJ). Soft tissue coverage of the prosthesis was performed when anatomy and patient condition permitted. Perioperative outcomes, intermediate-term survival, and reinfection rates were examined. Results All patients presented with either aneurysm-related symptoms or a clinical picture of sepsis. Diagnosis was confirmed utilizing computed tomography imaging. Mean age was 66 +/- 13 years, 8 patients (57.1%) were male, and mean aneurysm size was 5.9 +/- 1.3 cm. All patients were hypertensive, 3 (21.4%) had prior coronary revascularization, 7 (50%) had chronic pulmonary disease, 5 (35.7%) had diabetes mellitus, and 2 (14.3%) had end-stage renal disease requiring dialysis. Twelve patients (85.7%) had aneurysm-related pain, and 9 (64.3%) of them had contained rupture. Mean time from onset of illness to surgery was 36 days (range, 0 to 153). On preoperative blood cultures, 4 (28.6%) grew Staphylococcus aureus, 4 (28.6%) grew gram negative organisms, 2 (14.3%) grew mycobacterium, and 4 cultures (28.6%) had negative results. Empiric broad-spectrum antibiotics were initiated on all patients and adjusted based on final cultures. A majority of patients underwent repair utilizing a clamp-and-sew technique (n = 10, 71.4%); the remainder (n = 4, 28.6%) required repair under profound hypothermic circulatory arrest. After radical debridement of the infected tissue, grafts were placed in the normal anatomic position; 6 (42.9%) patients had additional soft tissue coverage, 5 (35.7%) utilizing an omental flap and 1 (7.1%), a serratus muscle flap. There was 1 in-hospital death (7.1%) secondary to ischemic bowel. Four patients (28.6%) required tracheostomy, and 1 (7.1%) had recurrent nerve injury. None of the patients incurred spinal cord injury, stroke, or new onset renal failure requiring dialysis. After surgery, all patients were given 6 weeks of intravenous antibiotics. Lifelong suppression therapy was maintained with oral antibiotics. There were no episodes of prosthetic graft infection on follow-up. Univariate analysis revealed that New York Heart Association functional class, diabetes, and preoperative renal dysfunction were preoperative risk factors for major adverse events. Mean follow-up time was 26.5 months (median 8.2; range, 1 to 142). Actuarial 5-year survival was 71%. Conclusions Open repair of mycotic descending thoracic and thoracoabdominal aortic aneurysms remains the gold standard of therapy. Aggressive intraoperative debridement with in situ prosthetic reconstruction permits a high rate of success in this very high risk cohort of patients. Lifelong antibiotic suppression therapy may prevent late prosthetic graft infection.

Institution

(Lau, Gaudino, De Biasi, Munjal, Girardi) Department of Cardiothoracic Surgery, Weill Cornell Medical College, New York, NY, United States

Publisher

Elsevier USA

Emtree Heading

adult; aged; article; blood culture; cardiac patient; cardiovascular risk; cerebrovascular accident; chronic lung disease; clinical article; cohort analysis; computer assisted tomography; deep hypothermic circulatory arrest; diabetes mellitus; end stage renal disease/th [Therapy]; female; follow up; gold standard; heart muscle revascularization; human; hypertension; male; mean arterial pressure; *mycotic aneurysm/su [Surgery]; *mycotic descending thoracic aortic aneurysm/su [Surgery]; New York Heart Association class; outcome assessment; peritoneal dialysis; positron emission tomography; priority journal; prospective study; salmonellosis; spinal cord injury; Staphylococcus aureus; sternotomy; surgical patient; *thoracoabdominal aorta aneurysm/su [Surgery]; tracheostomy; treatment duration; tuberculosis/dt [Drug Therapy]; nafcillin; oxacillin; piperacillin plus tazobactam; sultamicillin; vancomycin/dt [Drug Therapy].

Link to the Ovid Full Text or citation:

Click here for full text options

44.

A Cytomegalovirus Retinitis Screening Program: Evaluation of Enrollment Criteria for HIV Patients in Singapore.

Shah J.M., Leo S.W., Lee T.L., Tan X.L., Pan J.C., Wong E.P., Yong V.K., Lim T.H., Teoh S.C.

Ocular Immunology and Inflammation. 23 (5) (pp 362-370), 2015. Date of Publication: 03 Sep 2015.

AN: 2015319915

Purpose: Identification of optimal enrollment criteria for a CMVR screening program suitable for a resource-limited environment.Methods: A prospective audit was performed on newly diagnosed HIV patients referred for CMVR screening with any of the following four criteria: (1) visual symptoms, (2) low CD4+ counts (<50 cells/muL), (3) AIDS-defining illnesses (ADI), and/or (4) opportunistic infections (OI). Odds ratios for each of the demographic factors and enrollment criteria were calculated. Sensitivities, specificities, and workload reduction for the various combinations were determined.Results: A total of 348 screening visits for 176 HIV patients were performed. While individually only ADI was statistically significant for increased CMVR risk, the combination of CD4+ counts <50 cells/L with either ADI or visual symptoms or all 3 criteria were also statistically significant. Two enrollment criteria, ADI and ADI with CD4+ <50 cells/L, demonstrated good sensitivities, specificities, and workload reduction.Conclusion: We propose ADI and possibly CD4+ counts <50 cells/L as enrollment criteria for CMVR screening.

Institution

(Shah) Yong Loo Lin School of Medicine, National University of Singapore, Singapore, Singapore (Leo) Dr Leo Adult and Paediatric Eye Specialist, Singapore, Singapore

(Leo, Lee, Tan, Pan, Wong, Yong, Lim, Teoh) National Healthcare Group Eye Institute, Department of Ophthalmology, Tan Tock Seng Hospital, 11 Jalan Tan Tock Seng, Singapore 308433, Singapore

(Pan) Nobel Eye and Vision Centre, Singapore, Singapore

Publisher

Taylor and Francis Ltd

Emtree Heading

adult; article; blurred vision; cataract/di [Diagnosis]; CD4+ T lymphocyte; *Cytomegalovirus retinitis/di [Diagnosis]; diabetic retinopathy/di [Diagnosis]; diagnostic test accuracy study; eye discomfort; eye examination; female; human; *Human immunodeficiency virus infected patient; Human immunodeficiency virus infection; infection risk; major clinical study; male; medical audit; ocular tuberculosis/di [Diagnosis]; opportunistic infection; outcome assessment; patient coding; patient referral; *program evaluation; prospective study; *screening; sensitivity and specificity; Singapore; vitreous hemorrhage/di [Diagnosis]; workload.

Link to the Ovid Full Text or citation:

Click here for full text options

45.

New strain of tuberculosis G24767: Old disease a continuous threat.

Maldonado Quintana H.J., Fernandez R., Colon M., Adorno J., Betancourt M.C., Malpica Santiago J.C., Torellas Ruiz P., Corcino V.

American Journal of Respiratory and Critical Care Medicine. Conference: American Thoracic Society International Conference, ATS 2015 Denver, CO United States. Conference Start: 20150515 Conference End: 20150520. Conference Publication: (var.pagings). 191 (no pagination), 2015. Date of Publication: 2015.

AN: 72051103

Multi-drug resistant tuberculosis (MDR-TB) is defined as a Mycobacterium tuberculosis Strain resistant to two or more first-line antituberculous drugs. Multi-drug resistance can be primary or acquired. Primary when there is no history or evidence of previous antituberculous drug treatment and acquired in previously treated subjects for at least one month and those with treatment failures or relapses. We present a 59 year old male patient who develop shortness of breath, dry cough and associated weight loss of 55kg in the last 7 months. Medical history pertinent for diabetes mellitus. He used to work at a healthcare facility, lived in a concrete house with no pets and frequently travel to Ecuador. PPD skin test was performed on a yearly basis with negative results. CXR reveal a right upper lobe opacity for which flexible bronchoscopy was done yielding aerobic non-motile bacillus. Sputum AFB were positive and patient was treated for 36 days until negative sputum samples was reported. Patient was discharge home, follow by the health department in four drug anti tuberculosis therapy. Follow up AFB sputum samples were positive again for Mycobacterium Tuberculosis after 2 months. Sputum samples were send to the Centers of Disease Control and found a MDR tuberculosis resistant to isoniazid, rifampin, ethambutol and streptomycin. Genotyping was done for further classification and a newly discover genomic G24767 strain, was reported. Considering this complicated case of Tuberculosis, treatment with Amikacin, Linezolid, and Levofloxacin, secondary lines drugs, were given with improvement of patient's symptoms. Patient will continue treatment for one year. Tuberculosis is a global threat to society despite improvement in therapy as it continues to be an economic burden especially in underdevelop countries. The downfall of global economics and growing travel destinations in developing countries has escalade the exposure of organism not previously encountered in industrialized nations. Most cases of MDR-TB are reported on immunosuppressed patients with risk factors and from endemic areas. Nevertheless new strains with higher transmission degree are emerging as a threat in patients who have low risk factors for the development of MDR-TB. MDR-TB patients have three times higher transmission rate than non MDR- TB patients. The strain G24767 is the first genomic sequence not classified in the known TB cluster of the United States and Puerto Rico population. Its genomic pattern of resistance for first line drugs and borderline resistance to secondary drug is an alarming feature for the development of XDR-TB on US territory.

Institution

(Maldonado Quintana, Fernandez, Colon, Adorno, Betancourt, Malpica Santiago, Torellas Ruiz, Corcino) San Juan City Hospital, San Juan, Puerto Rico

Publisher

American Thoracic Society

Emtree Heading

*American; *society; *tuberculosis; human; patient; sputum; therapy; Mycobacterium tuberculosis; risk factor; bacillus (rod shaped bacterium); travel; male; relapse; drug therapy; diabetes mellitus; drug resistance; medical history; weight reduction; treatment failure; health care facility; pet animal; Ecuador; skin test; follow up; public health service; coughing; dyspnea; disease control; multidrug resistant tuberculosis; genotype; classification; Bacillus; United States; economics; organisms; exposure; developing country; bronchoscopy; Puerto Rico; population; tuberculostatic agent; ethambutol; levofloxacin; isoniazid plus rifampicin; streptomycin; amikacin; linezolid; tuberculin.

Link to the Ovid Full Text or citation:

Click here for full text options

46.

Duration of anti-tuberculous treatment within nine months is inversely associated with two-year relapse rate in diabetic patients.

Wang J.-Y., Lee C.-H.

American Journal of Respiratory and Critical Care Medicine. Conference: American Thoracic Society International Conference, ATS 2014 San Diego, CA United States. Conference Start: 20140516 Conference End: 20140521. Conference Publication: (var.pagings). 189 (no pagination), 2014. Date of Publication: 2014.

AN: 72042088

Background: Diabetes mellitus (DM) is the most common co-morbidity of tuberculosis (TB) and it increases the risk of unfavourable treatment outcome. This study investigated whether extending anti-TB treatment from 6 to 9 months reduces the risk of relapse. Methods: A nationwide cohort of DM patients with pulmonary TB receiving regular anti-TB treatment for less than 9.5 months and without positive culture after 2 months of treatment were identified from the National Health Insurance Research Database (NHIRD) of Taiwan. Factors predicting TB relapse within 2 years after complete treatment, including duration of anti-TB treatment, were explored using Cox regression analysis. Findings: The two-year relapse rate in all TB patients declined from 4.76% in 1997 to 0.81% in 2008. Among the 5713 DM patients with pulmonary TB, per-year increment in age (HR 0.96 [0.95-0.98]), number of years at TB diagnosis after 2002 (HR 0.83 [0.75-0.91]), chronic obstructive pulmonary disease (HR 1.80 [1.09-2.96]), and per-month increment in anti-TB treatment duration (HR 0.83 [0.70-0.99]) were independent predictors of TB relapse. Interpretation: There is a consistent decline in the 2-year relapse rate in overall TB population in Taiwan from 1997 to 2008. Cases of TB relapse in DM patients may be prevented by extending anti-TB treatment by 3 months. Follow-up care with a high suspicion of relapse may be necessary for young DM patients. (Figure Presented).

Institution

(Wang) National Taiwan University Hospital, Taipei, Taiwan (Republic of China) (Lee) Taipei Tzuchi Hospital, Taipei, Taiwan (Republic of China)

Publisher

American Thoracic Society

Emtree Heading

*human; *recurrence risk; *diabetic patient; *American; *society; relapse; patient; Taiwan; risk; treatment outcome; proportional hazards model; data base; tuberculosis; national health insurance; morbidity; follow up; chronic obstructive lung disease; regression analysis; diagnosis; treatment duration; population; diabetes mellitus.

Link to the Ovid Full Text or citation:

Click here for full text options

47.

Spinal tuberculosis: A retrospective chart review.

Sezgi C., Taylan M., Kaya H., Sen H.S., Abakay O., Bulut M., Abakay A.

Acta Medica Mediterranea. 30 (3) (pp 725-730), 2014. Date of Publication: 2014.

AN: 2014424239

Aims: Background and objectives: This study aimed to investigate the clinical characteristics, diagnostic methods, and therapeutic outcomes in patients with spinal tuberculosis (TB) in Southeastern Turkey Methods: Patients diagnosed with spinal TB at Dicle University Hospital, Diyarbakir, Turkey, between October 2005 to December 2010 were enrolled in the study retrospectively. Patients were evaluated for the following: Clinical presentation, underlying diseases, laboratory results, imaging findings, medical therapies, and treatment outcomes Results: A total of 23 patients including 14 men and 9 women with spinal TB, also known as Pott's disease, were enrolled. The study subjects' ages ranged from 17 to 69 years with a mean age of 38.4 years. Patients most often presented with back pain (69.5%, n=16) and fever (56.5%, n=13), and the most frequent physical finding was spinal tenderness (91.3%). Only 17.4% of the patients demonstrated neurological deficits and no paraplegia was observed. The time interval between onset of symptoms and treatment initiation was 3.2 months. Of all the patients, 47.8% exhibited Pott's disease in the thoracolumbar region while 34.8% only had thoracic involvement. On average, 2.5 vertebrae were diseased in this cohort. Furthermore, 82.6% of the patients had paraspinal and psoas abscesses. All patients were treated with anti-TB therapy. However, 26.1% required additional surgery, and one patient with miliary TB died. The mean treatment course lasted for 12.3 months. Conclusions: It may be possible to refine methods of spinal TB detection and diagnosis by studying thoracolumbar pathology in young adults with Pott's disease in endemic countries such as Turkey. By studying the clinical progression of this disease, the delay between symptom onset and diagnosis may be decreased so that complications such as paraplegia and surgical interventions are avoided.

Institution

(Sezgi, Taylan, Kaya, Sen, Abakay, Abakay) Dicle University School of Medicine, Department of Pulmonary Diseases, Diyarbakir, Turkey (Bulut) Dicle University School of Medicine, Department of Orthopedy, Diyarbakir, Turkey

Publisher

Acta Medica Mediterranea

Emtree Heading

adult; article; backache; cause of death; chronic kidney failure; clinical article; clinical feature; computer assisted tomography; erythrocyte sedimentation rate; female; fever; hepatitis B; human; leukocyte count; limb weakness; male; medical record review; non insulin dependent diabetes mellitus; nuclear magnetic resonance imaging; outcome assessment; paraplegia; paraspinal abscess; paresthesia; psoas abscess; retrospective study; spinal cord compression/su [Surgery]; spinal tenderness; spine disease; spine instability/su [Surgery]; spine malformation/su [Surgery]; spine surgery; thoracic spine; thoracolumbar spine; time; treatment duration; *tuberculous spondylitis/di [Diagnosis]; *tuberculous spondylitis/dt [Drug Therapy]; *tuberculous spondylitis/su [Surgery]; Turkey (republic); unspecified side effect/si [Side Effect]; X ray; C reactive protein/ec [Endogenous Compound]; ethambutol/ae [Adverse Drug Reaction]; ethambutol/dt [Drug Therapy]; isoniazid/ae [Adverse Drug Reaction]; isoniazid/dt [Drug Therapy]; pyrazinamide/ae [Adverse Drug Reaction]; pyrazinamide/dt [Drug Therapy]; rifampicin/ae [Adverse Drug Reaction]; rifampicin/dt [Drug Therapy]; streptomycin/ae [Adverse Drug Reaction]; streptomycin/dt [Drug Therapy].

Link to the Ovid Full Text or citation:

Click here for full text options

48.

Latent tuberculosis infection in patients with chronic plaque psoriasis: Evidence from the Italian Psocare Registry.

Cazzaniga S., Maccarone M., Picardo M., Naldi L., Griseta V., Miracapillo A., Azzini M., Mocci L., Michelini M., Offidani A., Bernardini L., Campanati A., Ricotti G., Giacchetti A., Norat M., Gualco F., Castelli A., Cuccia A., Diana A., Roncarolo G., Belli M.A., Baldassarre M.A., Santoro G., Vena G.A., Lo Console F., Filotico R., Mastrandrea V., Brunetti B., Musumeci F., Carrabba E., Dal Mas P., Annicchiarico F., Benvegnu B., Spaziani G., Cusano F., Saletta Iannazzone S., Galluccio A., Pezza M., Marchesi L., Imberti G., Reseghetti A., Barbera C., Reggiani M., Lanzoni A., Patrizi A., Bardazzi F., Antonucci A., De Tommaso S., Balestri R., Wallnofer W., Ingannamorte F., Calzavara-Pinton P., Iannazzi S., Zane C., Capezzera R., Bassisi S., Rossi M.T., Altamura V., Vigl W., Nobile C., Aste N., Murgia S., Mugheddu C., Scuderi G., Baglieri F., Di Dio C., Cilioni Grilli E., Mastronardi C., Agnusdei C.P., Antrilli A., Aulisa L., Raimondo U., Scotto Di Luzio G., Battarra V.C., Farro P., Plaitano R., Micali G., Musumeci M.L., Massimino D., Li Calzi M., La Greca S., Pettinato M., Sapienza G., Valenti G., De Giacomo P.F., D'Amico D., Arcangeli F., Brunelli D., Ghetti E., Tulli A., Assi G., Laria G., Prestinari F., Spadafora S., Coppola M., Caresana G., Pezzarossa E., Domaneschi E., Felisi C., Donato L., Bertero M., Musso L., Pa Lazzini S., Bruscino P., Agozzino U.C., Ottaviani M., Simoncini C., Virgili A., Osti F., Fabbri P., Volpi W., Caproni M., Lotti T., Prignano F., Buggiani G., Troiano M., Fenizi G., Altobella A., Amoruso A., Condello M., Goffredo A., Righini M.G., Alessandrini F., Satolli F., Zampetti M., Bertani E., Fossati S., Parodi A., Burlando M., Fiorucci C., Nigro A., Ghigliotti G., Massone L., Moise G.M., Serrai M., Cannata G., Campagnoli A.M., Daly M., Leporati C., Peila R., Filosa G., Bugatti L., Nicolini M., Nazzari G., Cestari R., Anastasio F., Larussa F.M., Pollice N., De Francesco F., Mazzocchetti G., Peris K., Fargnoli M.C., Di Cesare A., De Angelis L., Flati G., Biamonte A.S., Quarta G., Congedo M., Carcaterra A., Strippoli D., Fideli D., Marsili F., Celli M., Ceccarini M., Bachini L., D'Oria M., Schirripa V., De Filippi C., Martini P., Lapucci E., Mazzatenta C., Ghilardi A., Simonacci M., Bettacchi A., Gasco R., Zanca A., Battistini S., Dattola S., Vernaci R., Postorino F., Zampieri P.F., Padovan C., Gonzalez Intchaurraga M.A., Ladurner J., Guarneri B., Cannavo S., Manfre C., Borgia F., Puglisi Guerra A., Cattaneo A., Carrera C., Fracchiolla C., Mozzanica N., Prezzemolo L., Menni S., Lodi A., Martino P., Monti M., Mancini L., Sacrini F., Altomare G.F., Taglioni M., Lovati C., Mercuri S.R., Schiesari G., Giannetti A., Conti A., Lasagni C., Greco M., Ronsini G., Schianchi S., Fiorentini C., Niglietta S., Maglietta R., Padalino C., Crippa D., Pini M., Rossi E., Tosi D., Armas M., Ruocco V., Ayala F., Balato N., Gaudiello F., Cimmino G.F., Monfrecola G., Gallo L., Argenziano G., Fulgione E., Berruti G., Mozzillo R., Ceparano S., De Michele I., Giorgiano D., Leigheb G., Deledda S., Peserico A., Alaibac M., Piaserico S., Schiesari L., Dan G., Mattei I., Oro E., Arico M., Bongiorno M.R., Angileri R., Amato S., Todaro F., Milioto M., Bellastro R., Di Nuzzo S., De Panfilis G., Zanni M., Borroni G., Cananzi R., Brazzelli V., Lisi P., Stingeni L., Hansel K., Pierfelice V., Donelli S., Rastelli D., Gasperini M., Barachini P., Cecchi R., Bartoli L., Pavesi M., De Paola S., Corradin M.T., Ricciuti F., Piccirillo A., Viola L., Tataranni M., Mautone M.G., Lo Scocco G., Niccoli M.C., Brunasso Vernetti A.M.G., Gaddoni G., Resta F., Casadio M.C., Arcidiaco M.C., Luvara M.C., Albertini G., Di Lernia V., Guareschi E., Catrani S., Morri M., Amerio P., De Simone C., D'Agostino M., Agostino I., Calvieri S., Cantoresi F., Richetta A., Sorgi P., Carnevale C., Nicolucci F., Berardesca E., Ardigo M., De Felice C., Gubinelli E., Chimenti S., Talamonti M., Camplone G., Cruciani G., Riccardi F., Barbati R., Pagani W., Malagoli P.G., Pellicano R., Donadio D., Di Vito C., Cottoni F., Montesu M.A., Pirodda C., Addis G., Marongiu P., Farris A., Cacciapuoti M., Verrini A., Desirello G., Gnone M., Fimiani M., Pellegrino M., Castelli G., Zappala L., Sesana G., Ingordo V., Vozza E., Di Giuseppe D., Fasciocco D., Nespoli P., Papini M., Cicoletti M., Bernengo M.G., Ortoncelli M., Bonvicino A., Capella G., Doveil G.C., Forte M., Peroni A., Salomone B., Savoia P., Pippione M., Zichichi L., Frazzitta M., De Luca G., Zumiani G., Tasin L., Simonetto D., Ros S., Trevisan G., Patamia M., Miertusova S., Patrone P., Frattasio A., Piccirillo F., La Spina S., Di Gaetano L., Marzocchi V., Motolese A., Venturi C., Sedona P., Gai F., Pasquinucci S., Bellazzi R.M., Silvestri T., Girolomoni G., Gisondi P., Veller Fornasa C., Trevisan G.P.

British Journal of Dermatology. 172 (6) (pp 1613-1620), 2015. Date of Publication: 01 Jun 2015.

AN: 2015090164

Background The nationwide prevalence of latent tuberculosis infection (LTBI) in Italian patients with psoriasis has never been investigated. Objectives To estimate the nationwide prevalence of LTBI in Italian patients with psoriasis who are candidates for systemic treatment. Methods Data were obtained from the Psocare Registry on those patients (n = 4946) with age > 18 years, systemic treatment at entry specified and tuberculin skin test (TST) performed according to the Mantoux method. LTBI diagnosis was based on a positive TST result in the absence of any clinical, radiological or microbiological evidence of active tuberculosis. Results Latent tuberculosis infection was diagnosed in 8.3% of patients with psoriasis (409 of 4946). The prevalence of LTBI was lower in patients on biologics than in those on conventional systemic treatments, ranging from 4.3% (19 of 444) of patients on adalimumab to 31% (eight of 26) of those on psoralen-ultraviolet A (P < 0.05). Independent factors associated with LTBI were male sex [odds ratio (OR) 1.30, 95% confidence interval (CI) 1.04-1.62; P = 0.02], age over 55 years (OR 2.93, 95% CI 2.18-3.93; P < 0.001) and being entered into a conventional treatment (OR 3.83, 95% CI 3.10-4.74; P < 0.001). Positive history of tuberculosis was seen in 1% of patients (n = 49). Conclusions The nationwide prevalence of LTBI in Italian patients with psoriasis candidate to systemic treatment is high, and screening is recommended prior to biological treatment.

Institution

(Gisondi, Girolomoni) Department of Medicine, Section of Dermatology and Venereology, University of Verona, Piazzale A. Stefani 1, Verona I-37126, Italy (Cazzaniga, Naldi) GISED Study Centre, Ospedali Riuniti, Bergamo, Italy

(Chimenti) Department of Dermatology, University of Rome 'Tor Vergata', Rome, Italy

(Maccarone) Italian Psoriatic Patient Association (ADIPSO), Rome, Italy

(Picardo) Laboratory of Cutaneous Physiopathology, San Gallicano Dermatological Institute, Rome, Italy

(Griseta, Miracapillo) U.O.C. Dermatologia e Venereologia Ospedale Generale Regionale F. Miulli, Acquaviva delle Fonti, Italy

(Azzini, Mocci, Michelini) S.O.C. Dermatologia SS. Antonio e Biagio e C. Arrigo, Alessandria, Italy

(Offidani, Bernardini, Campanati) U.O. Clinica Dermatologica, Ancona, Italy

(Ricotti, Giacchetti) U.O. Dermatologia INRCA/IRCCS, Ancona, Italy

(Norat, Gualco) U.O. Dermatologia Ospedale Beauregard, Aosta, Italy

(Castelli, Cuccia, Diana) U.O. Dermatologia Ospedale S. Donato, Arezzo, Italy

(Roncarolo) S.O.C. Dermatologia Ospedale di Asti, Italy

(Belli, Baldassarre) U.O. Dermatologia Ospedale S.G. Moscati, Avellino, Italy

(Santoro) U.O.C. Dermatologia P.O. Cutroni Zodda, Barcellona (Me.), Italy

(Vena, Lo Console, Filotico, Mastrandrea) U.O. Dermatologia II Azienda Ospedaliera Policlinico Consorziale, Bari, Italy

(Brunetti, Musumeci) U.O. Dermatologica Ospedale di Battipaglia, Italy

(Carrabba, Dal Mas, Annicchiarico, Benvegnu, Spaziani) U.O. di Dermosifilopatia Ospedale S. Martino, Belluno, Italy

(Cusano, Saletta Iannazzone) U.O. Dermatologia Azienda Ospedaliera Rummo, Benevento, Italy

(Galluccio, Pezza) U.O. Dermatologia Ospedale S. Cuore di Gesu Fatebenefratelli, Benevento, Italy

(Marchesi, Imberti, Reseghetti) USC Dermatologia A.O. Ospedali Riuniti di Bergamo, Italy

(Barbera) U.O. Dermatologia Ospedale degli Infermi, Biella, Italy

(Reggiani, Lanzoni) U.O. di Dermatologia Presidio Ospedaliero Bellaria Maggiore, Bologna, Italy

(Patrizi, Bardazzi, Antonucci, De Tommaso, Balestri) U.O. Dermatologia Policlinico S. Orsola Malpighi, Bologna, Italy

(Wallnofer, Ingannamorte) Divisione Dermatologica Bolzano, Bolzano, Italy

(Calzavara-Pinton, Iannazzi, Zane, Capezzera, Bassisi, Rossi) Divisione Dermatologica, Azienda Spedali Civili di Brescia, Italy

(Altamura) U.O. Complessa di Dermatologia, P.O. Perrino, Brindisi, Italy

(Vigl, Nobile) U.O. Dermatologia Ospedale di Brunico, Italy

(Aste, Murgia, Mugheddu) Clinica Dermatologica Universita di Cagliari, Italy

(Scuderi, Baglieri, Di Dio) U.O. Dermatologia A.O. Ospedale S. Elia, Caltanissetta, Italy

(Cilioni Grilli) U.O. Dermatologia Ospedale B. Eustachio, Camerino, Italy

(Mastronardi, Agnusdei, Antrilli) U.O. Dermatologia P.O. Cardarelli, Campobasso, Italy

(Aulisa) S.O.C. Dermatologia Ospedale Casale Monferrato, Italy

(Raimondo, Scotto Di Luzio, Battarra, Farro, Plaitano) U.O. Dermatologia A.O. San Sebastiano, Caserta, Italy

(Micali, Musumeci, Massimino, Li Calzi) Clinica Dermatologica, Universita di Catania A.O.V. Emanuele, Catania, Italy

(La Greca) U.O. Dermatologia A.O. Garibaldi S. L. Curro A. Tomaselli, Catania, Italy

(Pettinato, Sapienza) U.O.C. di Dermatologica A.O. Universitaria V. Emanuele, Catania, Italy

(Valenti, De Giacomo, D'Amico) U.O. Dermatologia A.O. Pugliese Ciaccio, Catanzaro, Italy

(Arcangeli, Brunelli, Ghetti) U.O. Dermatologia Ospedale di Cesena, Italy

(Tulli, Assi, Amerio) Clinica Dermatologica, Universita di Chieti, Italy

(Laria, Prestinari) U.S. Complessa di Dermosifilopatia Ospedale S. Anna, Como, Italy

(Spadafora, Coppola) U.O. Dermatologia P.O. Mariano Santo, Cosenza, Italy

(Caresana, Pezzarossa, Domaneschi, Felisi) Istituti Ospitalieri di Cremona Servizio Ospedaliero di Dermatologia, Cremona, Italy

(Donato) U.O. Dermatologia P.O. Crotone, Italy

(Bertero, Musso, Pa Lazzini) S.O.C. Dermatologia Ospedale Santa Croce e Carle, Cuneo, Italy

(Bruscino) U.O. Dermatologia Ospedale S. Verdiana, Empoli, Italy

(Agozzino) U.O. di Dermatologia e M.S.T. A.O. U I, Enna, Italy

(Ottaviani, Simoncini) U.O. Dermatologia Ospedale Civile di Fabriano, Italy

(Virgili, Osti) Sezione di Dermatologia Azienda Ospedaliera Universitaria Ferrara, Italy

(Fabbri, Volpi, Caproni) Dip. Scienze Dermatologiche, Universita di Firenze, Italy

(Lotti, Prignano, Buggiani, Troiano) U.O. Complessa Dermatologica di Fisioterapia Dermatologica, Firenze, Italy

(Fenizi, Altobella, Amoruso, Condello, Goffredo) U.O. Dermatologia Azienda Ospedaliero-Universitaria, Foggia, Italy

(Righini, Alessandrini, Satolli) U.O. Dermatologia Ospedale G.B. Morgagni-L. Pieranto, Forli, Italy

(Zampetti) Azienda USL Roma H U.O.C. Dermatologia Aziendale Ospedale di Frascati, Italy

(Bertani, Fossati) U.O. Dermatologia A.O. S.A. Abate, Gallarate, Italy

(Parodi, Burlando, Fiorucci) Di.S.E.M. Sezione di Dermatologia, Genova, Italy

(Nigro, Ghigliotti) U.O. Dermatologia Ospedale S. Martino, Genova, Italy

(Massone) U.O. Complessa di Dermatologia dell'Ente Ospedaliero Galliera, Genova, Italy

(Moise) SOC Dermatologia Azienda per i Servizi Sanitari n. 2 Isontina, Gorizia, Italy

(Serrai) U.O. Dermatologia Presidio Ospedaliero Misericordia, Grosseto, Italy

(Cannata, Campagnoli) U.O. Complessa di Dermatologia Ospedale Civile, Imperia, Italy

(Daly, Leporati, Peila) U.O. Dermatologia Ospedali Riuniti di Ivrea, Italy

(Filosa, Bugatti, Nicolini) U.O. Dermatologia Ospedale A. Murri, Jesi, Italy

(Nazzari, Cestari) U.O. di Dermatologia Ospe dale Civile Sant'Anna, La Spezia, Italy

(Anastasio, Larussa) U.O. Dermatologia Ospedale Civile, Lamezia Terme, Italy

(Pollice, De Francesco, Mazzocchetti) Reparto di Dermatologia Ospedale di Lanciano, Lanciano, Italy

(Peris, Fargnoli, Di Cesare, De Angelis) Dermatologia Oncologica e Molecolare, L'Aquila, Italy

(Flati, Biamonte) U.O.C. Dermatologia Ospedale Regionale S. Salvatore, L'Aquila, Italy

(Quarta, Congedo) U.O. Dermatologia Ospedale V. Fazzi Lecce, Italy

(Carcaterra, Strippoli, Fideli) Dermatologia Presidio A. Manzoni, Lecco, Italy

(Marsili, Celli) U.O.C. Dermatologia Ospedale Versilia, Lido di Camaiore, Italy

(Ceccarini, Bachini, D'Oria) U.O. Dermatologia Ospedali Riuniti di Livorno, Italy

(Schirripa) P.O. Siderno ASL9 Locri, Italy

(De Filippi) U.O. Dermatologia A.O. della Provincia di Lodi, Italy

(Martini, Lapucci, Mazzatenta, Ghilardi) U.O. Dermatologia Ospedale Campo di Marte, Lucca, Italy

(Simonacci, Bettacchi, Gasco) U.O. di Dermatologia Ospedale di Macerata, Macerata, Italy

(Zanca) U.O. Dermatologia Ospedale S. Carlo Poma, Mantova, Italy

(Battistini) U.O. Dermatologia P.O. Massa, Italy

(Dattola, Vernaci, Postorino) Servizio Dermatologia, P.O. Melito Porto Salvo, Melito, Italy

(Zampieri, Padovan, Gonzalez Intchaurraga, Ladurner) Divisione Dermatologia e Venerologia Ospedale Franz Tappeiner, Merano, Italy

(Guarneri, Cannavo, Manfre, Borgia) U.O.C. di Dermatologia A.O.U.G. Martino, Messina, Italy

(Puglisi Guerra) U.O. Dermatologia A.O. Papardo, Messina, Italy

(Sedona) U.O. Dermatologia Dermatologia Ospedale Umberto I, Mestre, Italy

(Cattaneo, Carrera, Fracchiolla, Mozzanica, Prezzemolo) Centro per lo Studio e la Cura della Psoriasi, IRCCS Ospedale Maggiore di Milano, U.O. di Dermatologia, Milano, Italy

(Menni, Lodi, Martino) Clinica Dermatologica Universitaria AO San Paolo, Milano, Italy

(Monti, Mancini, Sacrini) U.O. Dermatologia Istituto Clinico Humanitas, Milano, Italy

(Altomare, Taglioni, Lovati) Servizio di Dermatologia Istituto Ortopedico Galeazzi, Milano, Italy

(Mercuri) Dermatologia IRCCS Fondazione Centro San Raffaele del Monte Tabor, Milano, Italy

(Schiesari) U.O. di Dermatologia Ospedale di Mirano, Mirano, Italy

(Giannetti, Conti, Lasagni, Greco, Ronsini, Schianchi, Fiorentini, Niglietta, Maglietta, Padalino) Clinica Dermatologica di Modena, Modena, Italy

(Crippa, Pini, Rossi, Tosi, Armas) U.S.C. di Dermosifilopatia A.O. San Gerardo, Monza, Italy

(Ruocco) U.O. di Clinica Dermatologica, Napoli, Italy

(Ayala, Balato, Gaudiello, Cimmino, Monfrecola, Gallo) Sezione di Dermatologia Dipartimento di Patologia Sistematica, Napoli, Italy

(Argenziano, Fulgione) D.A.S. Dermatologia e Venereologia, U.O. Malattie Veneree e Dermatologia Parassitaria, Seconda Universita di Napoli, Italy

(Berruti, Mozzillo) U.O.C. di Dermatologia P.O. San Gennaro, Napoli, Italy

(Ceparano, De Michele) DH Dermatologico, P.O. Ascalesi, Napoli, Italy

(Giorgiano) U.O. Dermatologia, P.O. Tortora Pagani, Nocera Pagani, Italy

(Leigheb) Clinica Dermatologica Universita del Piemonte Orientale, C/O Ospedale Maggiore della Carita, Novara, Italy

(Deledda) U.O. Dermatologia Ospedale S.F. Nuoro, Nuoro, Italy

(Peserico, Alaibac, Piaserico, Schiesari, Dan, Mattei, Oro) Clinica Dermatologica Universita di Padova, Padova, Italy

(Arico, Bongiorno, Angileri) Cattedra di Dermatologia, UOC di Dermatologia e Malattie Sessualmente Trasmesse Policlinico P. Giaccone Palermo, Italy

(Amato, Todaro, Milioto, Bellastro) U.O. di Dermatologia ARNAS Civico-Di Gristina-M. Ascoli, Palermo, Italy

(Di Nuzzo, De Panfilis, Zanni) Centro di Fotodermatologia, Parma, Italy

(Borroni, Cananzi, Brazzelli) Clinica Dermatologica Universita di Pavia IRCCS Policlinico S. Matteo, Pavia, Italy

(Lisi, Stingeni, Hansel) Sezione di Dermatologia Clinica, Allergologica e Venereologica, Dipartimento di Specialita Medico-Chirurgiche e Sanita Pubblica, Universita di Perugina, Italy

(Pierfelice) U.O. Dermatologia Ospedale Civile Pescara, Italy

(Donelli, Rastelli, Gasperini) U.O. Semplice Dipartimentale di Dermatologia Ospedale Piacenza, Italy

(Barachini) U.O. Dermatologia Azienda Ospedaliera Pisana, Pisa, Italy

(Cecchi, Bartoli, Pavesi) U.O. Dermatologia, Ospedale di Pistoia, Pistoia, Italy

(De Paola) U.O. Semplice Dermatologia Ospedale di Polla e S. Arsenio, Polla, Italy

(Corradin) U.O. Dermatologia Azienda Ospedaliera Santa Maria degli Angeli, Pordenone, Italy

(Ricciuti, Piccirillo, Viola, Tataranni, Mautone) U.O. Dermatologia Centro MTS, Potenza, Italy

(Lo Scocco, Niccoli, Brunasso Vernetti) U.O. Dermatologia Ospedale Misericordia e Dolce, Prato, Italy

(Gaddoni, Resta, Casadio) U.O. Aziendale di Dermatologia di Ravenna, Ravenna, Italy

(Arcidiaco, Luvara) U.O. Dermatologia Azienda Ospedaliera Bianchi-Melacrino-Morelli, Reggio Calabria, Italy

(Albertini, Di Lernia, Guareschi) Struttura Complessa di Dermatologia Azienda Ospedaliera Arcispedale, Reggio Emilia, Italy

(Catrani, Morri) U.O. Dermatologia Ospedale Infermi, Rimini, Italy

(Amerio, De Simone, D'Agostino, Agostino) Clinica Dermatologica Policlinico Gemelli, Roma, Italy

(Calvieri, Cantoresi, Richetta, Sorgi, Carnevale, Nicolucci) Dip. Malattiecutanee-Veneree, Chiurgia Plastica-Ricostruttiva Universit a studi di Roma, Roma, Italy

(Berardesca, Ardigo, De Felice) Istituto Dermatologico S. Gallicano, Roma, Italy

(Gubinelli) IDI IRCCS, Roma, Italy

(Chimenti, Talamonti) Clinica Dermatologica, Universita Studi di Roma, Roma, Italy

(Camplone) U.O. Dermatologia Azienda Ospedaliera Sant'Andrea, Roma, Italy

(Cruciani, Riccardi) U.O. Dermatologia Azienda Ospedaliera San Camillo Forlanini, Roma, Italy

(Barbati) U.O. Dermatologia Ospedale S. Eugenio, Roma, Italy

(Zumiani) U.O. Dermatologia Ospedale S. Maria del Carmine, Rovereto, Italy

(Pagani) S.O.C. Dermatologia Ospedale Civico, Rovigo, Italy

(Malagoli) Ambulatorio di Dermatologia Policlinico San Donato, S. Donato Milanese, Italy

(Pellicano) U.O. Dermatologia IRCCS Casa Sollievo della Sofferenza, S. Giovanni Rotondo, Italy

(Donadio, Di Vito) U.O.C. Dermatologia Ospedale S. Giovanni di Dio, Salerno, Italy

(Cottoni, Montesu, Pirodda, Addis, Marongiu) Clinica Dermatologica Universita di Sassari, Sassari, Italy

(Farris, Cacciapuoti, Verrini) U.O. di Dermatologia Ospedale San Paolo, Savona, Italy

(Desirello, Gnone) U.O. di Dermatologia Ospedale Civile di Sestri Levante, Sestri Levante, Italy

(Fimiani, Pellegrino) Azienda Ospedaliera Senese D.A.I. Med. Clin. e Sci. Immunologiche Applicate S.C. di Dermatologia, Siena, Italy

(Castelli, Zappala) U.O.C. di Dermatologia A.O.U.I., Siracusa, Italy

(Sesana) Dermatologia A.O. della Valtellina e della Valchiavenna, Sondrio, Italy

(Ingordo) Ospedale Marina Militare Taranto Rep. Dermatologia, Taranto, Italy

(Vozza, Di Giuseppe) U.O. Complessa di Dermatologia e Chirurgia Dermatologica, Taranto, Italy

(Fasciocco, Nespoli) U.O. Dermatologia P.O. G. Mazzini, Teramo, Italy

(Papini, Cicoletti) Clinica Dermatologica di Terni, Terni, Italy

(Bernengo, Ortoncelli, Bonvicino, Capella, Doveil, Forte, Peroni, Salomone, Savoia) SCDU Dermosifilopatia 2 Ospedale S. Lazzaro, Torino, Italy

(Pippione) SCDU Dermosifilopatia 3 Ospedale S. Lazzaro, Torino, Italy

(Zichichi, Frazzitta, De Luca) U.O. Dermatologia Azienda Ospedaliera S.A. Abate, Trapani, Italy

(Zumiani, Tasin) U.O. Dermatologia Ospedale S.C. di Trento, Trento, Italy

(Simonetto, Ros) U.O. di Dermatologia Ospedale Ca Foncello, Treviso, Italy

(Trevisan, Patamia, Miertusova) Istituto di Clinica Dermatologica, Trieste, Italy

(Patrone, Frattasio, Piccirillo, La Spina, Di Gaetano) Clinica Dermatologica, Udine, Italy

(Marzocchi) S.O.C. Dermatologia Azienda Ospedaliera Santa Maria della Misericordia di Udine, Udine, Italy

(Motolese, Venturi) U.O. Dermatologia Ospedale II Circolo, Varese, Italy

(Sedona, Gai, Pasquinucci) U.O. Dermatologia Venezia Mestre Ospedale SS. Giovanni e Paolo, Venezia, Italy

(Bellazzi, Silvestri) S.O.C. Dermatologia Ospedale di Vercelli, Vercelli, Italy

(Girolomoni, Gisondi) Clinica Dermatologica Ospedale Civile Maggiore, Verona, Italy

(Veller Fornasa, Trevisan) U.O. Dermatologia Vicenza, Italy

Publisher

Blackwell Publishing Ltd

Emtree Heading

adult; age; article; controlled study; diabetes mellitus/dt [Drug Therapy]; diagnostic test accuracy study; disease registry; female; human; *latent tuberculosis/di [Diagnosis]; *latent tuberculosis/ep [Epidemiology]; major clinical study; male; medical history; microbiological examination; middle aged; multicenter study; predictive value; prevalence; priority journal; *psoriasis vulgaris/di [Diagnosis]; *psoriasis vulgaris/th [Therapy]; *psoriasis vulgaris/dt [Drug Therapy]; PUVA; risk factor; sex difference; systemic therapy; thorax radiography; treatment outcome; tuberculin test; adalimumab/dt [Drug Therapy]; antidiabetic agent/dt [Drug Therapy]; cyclosporin A/dt [Drug Therapy]; etanercept/dt [Drug Therapy]; etretin/dt [Drug Therapy]; infliximab/dt [Drug Therapy]; methotrexate/dt [Drug Therapy]; psoralen/dt [Drug Therapy]; psoralen/po [Oral Drug Administration].

Link to the Ovid Full Text or citation:

Click here for full text options

49.

Characteristics and treatment outcomes of tuberculosis cases by risk groups, Japan, 2007-2010.

Uchimura K., Ngamvithayapong-Yanai J., Kawatsu L., Ohkado A., Yoshiyama T., Shimouchi A., Ito K., Ishikawa N.

Western Pacific surveillance and response journal : WPSAR. 4 (1) (pp 11-18), 2013. Date of Publication: 01 Jan 2013.

AN: 23908950

INTRODUCTION: We studied the characteristics and treatment outcomes of the following risk groups for tuberculosis (TB): those with HIV and diabetes mellitus (DM), contact cases, the homeless, foreigners, health care workers (HCW) and the elderly. METHOD: A descriptive cross-sectional study was conducted by analysing the Japanese tuberculosis surveillance data of all cases registered between 2007 and 2010 (n = 96 689).

RESULTS: The annual proportion of TB cases by risk group was stable over the study period, although there was a slight but significant increase observed for foreigners and elderly cases. Homeless and elderly TB cases had the highest DM co-morbidity (16.6% and 15.3%). HIV co-infection was low in all TB cases (0.2%) yet highest in foreigners (1.3%). HIV status of 45% of TB cases was unknown. The proportion of multi drug resistant (MDR) TB was similar among all risk groups (0.0-0.9%) except foreigners, at 3.4%. Males in most risk groups had higher mortality than females; the mortality of all TB cases in all age groups for both males and females were 3.6-24 times higher than the general population.

DISCUSSION: Reasons for the high proportion of "HIV status unknown" should be investigated and improved. Contact tracing among foreign cases with MDR-TB should be a priority. Homeless persons should be screened for DM together with TB. Programs to enhance health and nutrition status may benefit tuberculosis prevention among the elderly. Tuberculosis screening and TB education are important for HCW.

Institution

(Uchimura, Ngamvithayapong-Yanai, Kawatsu, Ohkado, Yoshiyama, Shimouchi, Ito, Ishikawa) The Research Institute of Tuberculosis, Japan Anti-tuberculosis Association, Tokyo, Japan

Emtree Heading

adolescent; adult; age; aged; child; Coinfection/ep [Epidemiology]; complication; cross- sectional study; Diabetes Complications/ep [Epidemiology]; female; *health care personnel; *homelessness; human; Human immunodeficiency virus; Human immunodeficiency virus infection; infant; Japan; male; middle aged; *migrant; newborn; preschool child; public health; risk; sex difference; treatment outcome; tuberculosis/ep [Epidemiology]; tuberculosis/th [Therapy]; Tuberculosis, Multidrug-Resistant/ep [Epidemiology]; very elderly; young adult.

Link to the Ovid Full Text or citation:

Click here for full text options

50.

The double burden of infectious diseases and diabetes-A bidirectional relationship.

Sobngwi E., Mbanya J.C.

European Endocrinology. 11 (2) (pp 112-113), 2015. Date of Publication: 2015.

AN: 2015370960

There is growing evidence for an etiological interaction between infectious diseases and diabetes, as well as for bidirectional influence of clinical presentation, spread, and outcomes. Some HIV treatments increase diabetes risk, and some infectious diseases may determine unique phenotypes of diabetes. Individuals who have type 2 diabetes have increased risk for tuberculosis and viral hepatitis and have poorer treatment outcomes. Joint noncommunicable diseases (NCDs) and infectious diseases clinics are the ideal method of tackling the double burden of these diseases in developing countries.

Institution

(Sobngwi) National Obesity Centre, Yaounde Central Hospital, Yaounde, Cameroon (Mbanya) Department of Internal Medicine and Specialties, Faculty of Medicine and Biomedical Sciences, University of Yaounde 1, Yaounde, Cameroon

(Mbanya) Laboratory of Molecular Medicine and Metabolism, The Biotechnology Centre, University of Yaounde 1, Yaounde, Cameroon

Publisher

Touch Briefings

Emtree Heading

Africa south of the Sahara; article; developing country; *diabetes mellitus/ep [Epidemiology]; *diabetes mellitus/pc [Prevention]; *diabetes mellitus/th [Therapy]; *diabetes mellitus/dt [Drug Therapy]; disease association; disease predisposition; exercise; health care access; heredity; high risk population; human; Human immunodeficiency virus infection/dt [Drug Therapy]; *infection/ep [Epidemiology]; insulin resistance; life expectancy; lifestyle modification; metabolic syndrome X; mixed infection; morbidity; mortality; non communicable disease; non insulin dependent diabetes mellitus; phenotype; prevalence; risk; treatment outcome; tuberculosis; virus hepatitis; antiretrovirus agent/dt [Drug Therapy]; metformin/dt [Drug Therapy].

Link to the Ovid Full Text or citation:

Click here for full text options

51.

Treatment outcomes among drug-susceptible tuberculosis patients in Latvia, 2006-2010.

Lucenko I., Riekstina V., Perevoscikovs J., Mozgis D., Khogali M., Gadoev J., Decolombani P., Kumar A.M.V.

Public Health Action. 4 (pp S54-S58), 2014. Date of Publication: 21 Oct 2014.

AN: 2015334779

Setting: Latvia, an Eastern European country with a high burden of tuberculosis (TB). Objective: To describe treatment outcomes among new drug-susceptible TB patients and assess the association of treatment outcomes with selected social determinants and risk factors. Design: A retrospective cohort study of patients aged >15 years registered during 2006-2010, with a review of records in the National Tuberculosis Registry. Results: Of 2476 patients, 1704 (69%) were male; the median age was 42 years. About two thirds of patients were unemployed or retired, 7% were human immunodeficiency virus (HIV) positive and 35% had a history of alcohol use. Treatment success was achieved in 2167 (88%) patients. Older age, unemployment, HIV infection and alcohol use were found to be independently associated with unsuccessful treatment (death, loss to follow- up, failure, transfer out and other). For many variables, including HIV infection, diabetes mellitus and tobacco use, it was not possible to distinguish between 'not recorded' and 'not present' in the registry. Conclusion: The treatment success rate among new drug-susceptible TB patients exceeded the 85% global target for TB control. Additional attention and support is required for most vulnerable patients, such as those who are unemployed or retired, HIV infected and alcohol users. The National TB Registry should be revised to improve definitions and staff should be trained for proper data collection and recording.

Institution

(Lucenko, Perevoscikovs, Mozgis) Centre for Disease, Prevention and Control of Latvia, Riga, Latvia (Riekstina) Riga East Clinical, University Hospital, Centre of Tuberculosis and Lung Diseases, Riga, Latvia

(Mozgis) Riga Stradins University, Riga, Latvia

(Khogali) Medical Department, Operational Research Unit, Medecins Sans Frontieres (MSF), Operational Centre, Brussels, Luxembourg

(Gadoev) World Health Organization (WHO) Country Office, Tashkent, Uzbekistan

(Decolombani) WHO Regional Office for Europe, Copenhagen, Denmark

(Kumar) International Union, Against Tuberculosis and Lung Disease, South-East, Asia Regional Office, New Delhi, India

Publisher

International Union Against Tuberculosis and Lung Disease (The Union) (68 boulevard Saint Michel, Paris 75006, France)

Emtree Heading

adolescent; adult; aged; alcohol consumption; article; disease control; *drug sensitivity; female; follow up; human; Human immunodeficiency virus infection; Latvia; major clinical study; male; retrospective study; risk factor; short course therapy; treatment failure; *treatment outcome; *tuberculosis/dt [Drug Therapy]; ethambutol/dt [Drug Therapy]; isoniazid/dt [Drug Therapy]; pyrazinamide/dt [Drug Therapy]; rifampicin/dt [Drug Therapy].

Link to the Ovid Full Text or citation:

Click here for full text options

52.

Long-term Outcomes of ABO-Incompatible Living Donor Kidney Transplantation: A Comparative Analysis.

Shin E., Kwon S.W., Yang W.S., Baeck C., Yu H., Cho H., Park S.-K.

Transplantation Proceedings. 47 (6) (pp 1720-1726), 2015. Date of Publication: 01 Jul 2015.

AN: 2015305941

Background Although ABO-incompatible kidney transplantation has become more common due to organ shortage, few studies on long-term outcomes have been performed in the Korean population. Methods A retrospective review of medical records was conducted for individuals who underwent living donor kidney transplantation at Asan Medical Center from February 2009 to January 2012. Results A total of 469 patients were included; the mean age was 42.8 +/- 11.8 years, and the median follow-up period was 45 (range, 1-65) months. ABO-incompatible recipients (73) were compared with ABO-compatible patients (396). Patient survival was similar between the ABO-incompatible group (97.3% and 95.9% at 1 and 3 years) and the ABO-compatible group (99.0% and 98.5% at 1 and 3 years; P =.136). Death-censored graft survival was also comparable between groups (98.6% vs 99.7% at 1 year; 98.6% vs 98.7% at 3 years; P =.386). Graft function, acute rejection, and postoperative complications were not significantly different between groups. Additionally, high body mass index and multiple human leukocyte antigen mismatches were significant risk factors for acute rejection (OR: 1.08, 95% CI: 1.01-1.16, P =.033; and OR: 1.20, 95% CI: 1.02-1.40, P =.025, respectively). Conclusion ABO-incompatible kidney transplantation could be a safe option when ABO-compatible donors are not available.

Institution

(Shin, Yang, Baeck, Yu, Cho, Park) Division of Nephrology, Department of Internal Medicine, Asan Medical Center, 388-1 Pungnap-2 dong, Songpa-gu, Seoul 138-736, South Korea (Kwon) Department of Laboratory Medicine, University of Ulsan College of Medicine, Asan Medical Center, Seoul, South Korea

Publisher

Elsevier USA

Emtree Heading

acute graft rejection/di [Diagnosis]; acute graft rejection/dt [Drug Therapy]; adolescent; adult; aged; article; avascular necrosis/co [Complication]; bk virus infection/co [Complication]; bleeding/co [Complication]; *blood group ABO incompatibility; body mass; catheter thrombosis/co [Complication]; child; comparative study; controlled study; creatinine blood level; cytomegalovirus infection/co [Complication]; diabetes mellitus/co [Complication]; drug dose reduction; female; follow up; glomerulus filtration rate; graft recipient; graft survival; hematoma/co [Complication]; herpes simplex/co [Complication]; herpes virus infection/co [Complication]; HLA matching; human; human tissue; immunosuppressive treatment; infectious complication/co [Complication]; intestine injury/co [Complication]; intravenous catheter; kidney allograft; kidney graft rejection/di [Diagnosis]; kidney graft rejection/dt [Drug Therapy]; *kidney transplantation; *living donor; long term care; lymphocele/co [Complication]; major clinical study; male; medical record; meningitis/co [Complication]; mortality; *outcome assessment; parvovirus infection/co [Complication]; Pneumocystis pneumonia/co [Complication]; pneumonia/co [Complication]; postoperative complication/co [Complication]; priority journal; retrospective study; risk factor; septic shock/co [Complication]; single drug dose; survival rate; tuberculosis/co [Complication]; urinary tract infection/co [Complication]; creatinine/ec [Endogenous Compound]; methylprednisolone/dt [Drug Therapy]; mycophenolate mofetil/dt [Drug Therapy]; rituximab; tacrolimus/dt [Drug Therapy].

Link to the Ovid Full Text or citation:

Click here for full text options

53.

The influence of mining and human immunodeficiency virus infection among patients admitted for retreatment of tuberculosis in Northern Tanzania.

Mpagama S.G., Lekule I.A., Mbuya A.W., Kisonga R.M., Heysell S.K.

American Journal of Tropical Medicine and Hygiene. 93 (2) (pp 212-215), 2015. Date of Publication: 01 Aug 2015.

AN: 2015302749

In tuberculosis (TB)-prevalent settings, patients admitted for retreatment of TB may account for a high burden of poor treatment outcome. We performed a retrospective cohort study to characterize retreatment patients and outcomes at a TB referral hospital in northern Tanzania. From 2009 to 2013, 185 patients began a retreatment regimen, the majority for relapse after prior treatment completion. Men accounted for an unexpected majority (88%), 36 (20%) were human immunodeficiency virus (HIV) infected and for 45 (24%) mining was their primary occupation. A poor outcome (death, default, or persistent smear positivity after 7 months of treatment) was found in 37 (23%). HIV infection was the only significant predictor of poor outcome (adjusted odds ratio [aOR] = 2.50, 95% confidence interval [CI] = 1.07-5.83, P = 0.034). Interventions to minimize need for retreatment or improve retreatment success may be regionally specific. In our setting, community-based diagnosis and management among at-risk subpopulations such as miners and those HIV infected appear of highest yield.

Institution

(Heysell) Division of Infectious Diseases and International Health, University of Virginia, P.O. Box 81340, Charlottesville, VA 22908, United States (Mpagama, Lekule, Mbuya, Kisonga) Kibong'oto Infectious Disease Hospital, Kilimanjaro, Tanzania

Publisher

American Society of Tropical Medicine and Hygiene (111 Deer Lake Road, Suite 100,Deerfield, Illinois 60015, United States)

Emtree Heading

adult; aged; alcohol consumption; article; CD4 lymphocyte count; diabetes mellitus; female; hospital admission; human; Human immunodeficiency virus infected patient; *Human immunodeficiency virus infection; major clinical study; male; multidrug resistant tuberculosis; patient referral; pneumoconiosis; relapse; *retreatment; smoking; sputum culture; sputum smear; Tanzania; *tuberculosis/dt [Drug Therapy]; ethambutol/dt [Drug Therapy]; isoniazid/dt [Drug Therapy]; pyrazinamide/dt [Drug Therapy]; rifampicin/dt [Drug Therapy]; streptomycin/dt [Drug Therapy].

Link to the Ovid Full Text or citation:

Click here for full text options

54.

Tuberculosis is frequently diagnosed within 12 months of diabetes mellitus.

Heo E.Y., Choi N.-K., Yang B.R., Koo B.K., Hwang S.-S., Lee C.-H., Kang Y.A.

International Journal of Tuberculosis and Lung Disease. 19 (9) (pp 1098-1101), 2015. Date of Publication: 01 Sep 2015.

AN: 2015299235

BACKGROUND: Evidence regarding the effects of tuberculosis (TB) screening among patients with diabetes mellitus (DM) in intermediate TB burden countries is insufficient, and the most appropriate time point for TB screening is unclear. OBJ ECT IVE : To investigate trends in TB incidence among newly diagnosed DM patients. DESIGN: A retrospective cohort study of the claims database of the Health Insurance Review and Assessment Service in Korea was performed. Participants were newly diagnosed with type 2 DM in 2009. The study outcome was TB incidence between 2009 and 2011 among participants according to duration of type 2 DM. RESULT S : A cohort of 331 601 patients with newly diagnosed type 2 DMin 2009 was identified. During the 3-year follow-up period, 1533 patients were diagnosed with TB. The estimated incidence of TB among newly diagnosed type 2 DM patients was 18/10 000 patientyears (py) (95%CI 17.5-19.4). TB incidence was 33/ 10 000 py (95%CI 30.0-35.6) in the first 6 months, and 19/10 000 py (95%CI 16.5-20.6) in the following 6-month period. CONCLUS IONS : The risk of developing TB was increased among DM patients, particularly during the first 12 months after DM diagnosis.

Institution

(Heo, Yang, Koo) Department of Internal Medicine, Boramae Medical Center, Seoul National University, Seoul Metropolitan Government, Seoul, South Korea (Choi) Medical Research Collaborating Center, College of Medicine, Seoul National University, Seoul National University Hospital, Seoul, South Korea

(Choi) Seoul National University Medical Research Center, Institute of Environmental Medicine, Seoul, South Korea

(Yang) Department of Preventive Medicine, Seoul National University College of Medicine, Seoul, South Korea

(Hwang) Department of Social and Preventive Medicine, Inha University School of Medicine, Incheon, South Korea

(Lee) Department of Internal Medicine, College of Medicine, Seoul National University, Seoul National University Hospital, Seoul, South Korea

(Kang) Department of Internal Medicine, College of Medicine, Yonsei University, Severance Hospital, 50-1 Yonsei-ro, Seodaemun-gu, Seoul 120-752, South Korea

Publisher

International Union against Tubercul. and Lung Dis.

Emtree Heading

adult; aged; article; cohort analysis; controlled study; data base; *diabetes mellitus; diabetic patient; disease duration; female; follow up; human; incidence; infection risk; major clinical study; male; non insulin dependent diabetes mellitus/dt [Drug Therapy]; outcome assessment; priority journal; retrospective study; screening; *tuberculosis/di [Diagnosis]; *tuberculosis/dt [Drug Therapy]; 2,4 thiazolidinedione derivative/dt [Drug Therapy]; alpha glucosidase inhibitor/dt [Drug Therapy]; aminosalicylic acid/dt [Drug Therapy]; cycloserine/dt [Drug Therapy]; ethambutol/dt [Drug Therapy]; insulin/dt [Drug Therapy]; isoniazid plus rifampicin/dt [Drug Therapy]; meglitinide/dt [Drug Therapy]; metformin/dt [Drug Therapy]; protionamide/dt [Drug Therapy]; pyrazinamide/dt [Drug Therapy]; sulfonylurea/dt [Drug Therapy].

Link to the Ovid Full Text or citation:

Click here for full text options

55.

Epidemiology and in-hospital outcome of stroke in South Ethiopia.

Deresse B., Shaweno D.

Journal of the Neurological Sciences. 355 (1-2) (pp 138-142), 2015. Date of Publication: 15 Aug 2015.

AN: 2015099941

Introduction Although the burden of stroke in Sub-Saharan Africa, including Ethiopia, is increasing, there are few available data on stroke in Ethiopia. Objective To describe the magnitude of risk factors, sub-types and in-hospital outcome of stroke at Hawassa University Referral Hospital, Ethiopia. Methods A prospective hospital-based study was conducted with all adult patients admitted to Hawassa University Referral Hospital with stroke diagnosis between May 2013 and April 2014. Computerized tomography scan was performed in all patients to confirm the type of stroke. Stroke severity at admission was assessed by the National Institute of Health Stroke Scale. Stroke outcome at discharge was measured using the modified Rankin stroke scale. Results A total of 163 stroke patients were recruited during the study period, of which 82 (50.3%) patients had ischemic stroke while 81 (49.7%) had hemorrhagic stroke. Stroke risk factors included hypertension (50.9%), cardiac diseases (16.6%), diabetes mellitus (7.4%), alcohol (10.4%), cigarette smoking (4.9%) and tuberculous meningitis (3.1%). In-hospital stroke mortality was 14.7%. The main predictors of in-hospital stroke mortality were stroke severity at admission, hemorrhagic stroke, decreased level of consciousness and seizure. Conclusion The proportion of hemorrhagic stroke is higher than in Western countries. Hypertension is the most common risk factor for stroke. More than half of the patients were discharged with severe disability. We recommend establishing stroke units in resource limited countries like Ethiopia in order to reduce stroke mortality and post stroke disability.

Author NameID

Deresse, Birrie; ORCID: <http://orcid.org/0000-0003-4371-6087>

Institution

(Deresse) Neurology Unit, Department of Internal Medicine, School of Medicine, College of Medicine and Health Sciences, Hawassa University, Hawassa, Ethiopia (Shaweno) School of Public and Environmental Health, College of Medicine and Health Sciences, Hawassa University, Hawassa, Ethiopia

Publisher

Elsevier

Emtree Heading

adolescent; adult; aged; alcohol consumption; article; aspiration pneumonia; atherosclerosis; *brain hemorrhage/ep [Epidemiology]; *brain hemorrhage/et [Etiology]; brain infarction; *brain ischemia/ep [Epidemiology]; *brain ischemia/et [Etiology]; brain stem infarction; cardioembolic stroke; carotid atherosclerosis; cerebellum infarction; computer assisted tomography; consciousness disorder; controlled study; diabetes mellitus; disease severity; dyslipidemia; echocardiography; electrocardiography; Ethiopia; family history; female; headache; heart disease; hospital acquired pneumonia; human; Human immunodeficiency virus infection; hypertension; intracranial hypertension; lumbar puncture; major clinical study; male; middle aged; obesity; outcome assessment; priority journal; prospective study; Rankin scale; risk assessment; risk factor; seizure; smoking; subarachnoid hemorrhage/ep [Epidemiology]; transient ischemic attack; tuberculous meningitis.

Link to the Ovid Full Text or citation:

Click here for full text options

56.

Causes of prematurity in the Bloemfontein Academic Complex.

Brits H., Adriaanse M., Rall D.-M., van Biljon M., van der Walt A., Wasserman E., Joubert G.

South African Family Practice. 57 (3) (no pagination), 2015. Date of Publication: 21 Jul 2015.

AN: 2015211314

Background: Prematurity is globally the leading cause of neonatal mortality, morbidity and long-term disability. The Millennium Development Goals (MDG) of the United Nations, and specifically MDG 4, address child mortality. Neonatal mortality rates contribute to 37% of all under-five mortality, with the largest proportion (30%) due to prematurity. The overall purpose of this study was to determine factors associated with prematurity and to identify treatable and preventable causes at the Bloemfontein Academic Complex in order to decrease the morbidity and mortality associated with prematurity in line with MDG. Methods: A case control study design was used including all premature babies referred to the Kangaroo Care Unit of the National District Hospital between December 2010 and March 2011. Each baby was included with his/her mother as a pair. For each premature baby and mother pair a term baby and mother pair was included as a control. Data were collected on a data sheet from the mothers' antenatal records, and from maternal notes as well as the neonatal follow-up and discharge notes. Data gathered included baseline characteristics, habits, diseases and medication of the mother during the antenatal period and delivery as well as baseline demographics of the babies. Results: A total of 194 mothers and 198 babies were included in the study with 109 case mothers and 85 control mothers. Pre-existing medical conditions were more prevalent in the premature group (69%) compared with the control group (27%) with p < 0.0001. These conditions included hypertension, HIV disease and syphilis. Possible causes for prematurity identified in this study included teenage mothers and premature rupture of membranes. Conclusion: Risk factors identified for prematurity were: teenage mothers, pre-existing medical conditions in the mother, and preterm rupture of membranes. Smoking and alcohol consumption during pregnancy could not be identified as risk factors for prematurity. It is recommended that patients with any of the above-mentioned identified risk factors be classified as high risk for the development of prematurity and shorter follow-up intervals and more aggressive management of pre-existing medical conditions should be practised.

Institution

(Brits) Department of Family Medicine, University of the Free State, Bloemfontein, South Africa (Adriaanse, Rall, van Biljon, van der Walt, Wasserman) School of Medicine, University of the Free State, Bloemfontein, South Africa

(Joubert) Department of Biostatistics, University of the Free State, Bloemfontein, South Africa

Publisher

Medpharm Publications (PO Box 14804, Lyttelton, Gauteng 0157, South Africa)

Emtree Heading

adolescent mother; adult; alcohol consumption; article; case control study; controlled study; diabetes mellitus; disease association; female; fetus lung maturity; follow up; hospital discharge; human; Human immunodeficiency virus infection; hypertension; infant; infection; major clinical study; multiple pregnancy; outcome assessment; premature fetus membrane rupture; *prematurity; prenatal diagnosis; risk assessment; smoking; syphilis; tuberculosis; folic acid; iron; magnesium sulfate.

Link to the Ovid Full Text or citation:

Click here for full text options

57.

Serum drug concentrations of INH and RMP predict 2-month sputum culture results in tuberculosis patients.

Mah A., Kharrat H., Ahmed R., Gao Z., Der E., Hansen E., Long R., Kunimoto D., Cooper R.

International Journal of Tuberculosis and Lung Disease. 19 (2) (pp 210-215), 2015. Date of Publication: 01 Feb 2015.

AN: 2015200884

BACKGROUND: In Alberta provincial tuberculosis (TB) clinics, serum drug concentrations (SDCs) are measured in patients with human immunodeficiency virus, diabetes mellitus or at extremes of weight, or showing slow clinical response to treatment, to guide treatment. DESIGN: A retrospective review was performed of TB cases in Northern Alberta with SDCs measured from 1998 to 2013. Adequacy of SDC was based on the maximum concentration (Cmax) achieved in serum, with rifampicin (RMP) values <8 mug/ml and isoniazid (INH) values <3 mug/ml for daily dosing and <9 mug/ml for intermittent dosing considered inadequate. Clinical variables and microbiological outcomes were then compared between the adequate and inadequate groups. RESULTS: Of 134 pulmonary TB cases with SDCs for INH and/or RMP, we found a significant increase in 2-month sputum culture positivity in the cohort with inadequate concentrations of INH compared to those with adequate INH concentrations (42.5% vs. 18.3%, P = 0.0084). A similar trend was seen in the cohort with inadequate concentrations of RMP (39% vs. 21%, P = 0.0725). CONCLUS IONS: Among our study population, low SDCs of INH and, to a lesser extent, RMP, appear to be associated with reduced sputum culture conversion after 2 months of treatment.

Institution

(Mah, Ahmed, Long, Kunimoto, Cooper) Department of Medicine, University of Alberta, Edmonton, AB, Canada (Kharrat) Faculty of Medicine and Dentistry, University of Alberta, Edmonton, AB, Canada

(Gao, Hansen) Alberta Health Services TB Program, Alberta Health Services, Edmonton, AB, Canada

(Der) Central TB Services, Alberta Health Services, Calgary, AB, Canada

Publisher

International Union against Tubercul. and Lung Dis.

Emtree Heading

adult; antibiotic sensitivity; Canada; cohort analysis; comorbidity; *drug blood level; drug intermittent therapy; drug treatment failure; female; human; Human immunodeficiency virus infection; insulin dependent diabetes mellitus; *lung tuberculosis/dt [Drug Therapy]; major clinical study; male; maximum plasma concentration; medical record review; non insulin dependent diabetes mellitus; patient compliance; prediction; priority journal; relapse; retrospective study; review; sputum culture; treatment duration; treatment outcome; ethambutol/dt [Drug Therapy]; *isoniazid/cr [Drug Concentration]; *isoniazid/dt [Drug Therapy]; pyrazinamide/dt [Drug Therapy]; *rifampicin/cr [Drug Concentration]; *rifampicin/dt [Drug Therapy].

Link to the Ovid Full Text or citation:

Click here for full text options

58.

Diabetes mellitus and tuberculosis.

Kansal H.M., Srivastava S., Bhargava S.K.

Journal International Medical Sciences Academy. 28 (1) (pp 58-60), 2015. Date of Publication: 01 Jan 2015.

AN: 2015136959

Diabetes and tuberculosis are the twin epidemics which has a major impact on morbidity and mortality of either disorder. Improved understanding of the bidirectional relationship is necessary to reduce the dual burden of the disease. The present article focuses on the association of the two disorders and salient features of the two disorders when they occur together.

Institution

(Kansal) Department of Pulmonary Medicine, School of Medical Sciences and Research, Sharda University, Greater Noida, Uttar Pradesh, India (Srivastava) Department of Medicine, School of Medical Sciences and Research, Sharda University, Greater Noida, Uttar Pradesh, India

(Bhargava) Dept of Radiology and Imaging, School of Medical Sciences and Research, Sharda University, Greater Noida, Uttar Pradesh, India

Publisher

International Medical Sciences Academy

Emtree Heading

article; clinical feature; *diabetes mellitus/ep [Epidemiology]; drug interaction; epidemic; human; morbidity; mortality; outcome assessment; risk factor; screening; sputum; *tuberculosis/dr [Drug Resistance]; *tuberculosis/ep [Epidemiology]; tuberculostatic agent.

Link to the Ovid Full Text or citation:

Click here for full text options

59.

Duration of treatment in pulmonary tuberculosis: Are international guidelines on the management of tuberculosis missing something?.

Atif M., Sulaiman S.A.S., Shafie A.A., Babar Z.U.

Public Health. 129 (6) (pp 777-782), 2015. Date of Publication: 01 Jun 2015.

AN: 2015054629

Background: Despite evidence of an association between tuberculosis (TB) treatment outcomes and the performance of national tuberculosis programmes (NTP), no study to date has rigorously documented the duration of treatment among TB patients. As such, this study was conducted to report the durations of the intensive and continuation phases of TB treatment and their predictors among new smear-positive pulmonary tuberculosis (PTB) patients in Malaysia. Study design: Descriptive, non-experimental, follow-up cohort study. Methods: This study was conducted at the Chest Clinic of Penang General Hospital between March 2010 and February 2011. The medical records and TB notification forms of all new smear-positive PTB patients, diagnosed during the study period, were reviewed to obtain sociodemographic and clinical data. Based on standard guidelines, the normal benchmarks for the durations of the intensive and continuation phases of PTB treatment were taken as two and four months, respectively. A patient in whom the clinicians decided to extend the intensive phase of treatment by >2 weeks was categorized as a case with a prolonged intensive phase. The same criterion applied for the continuation phase. Multiple logistic regression analysis was performed to find independent factors associated with the duration of TB treatment. Data were analyzed using Predictive Analysis Software Version 19.0. Results: Of the 336 patients included in this study, 261 completed the intensive phase of treatment, and 226 completed the continuation phase of treatment. The mean duration of TB treatment (n = 226) was 8.19 (standard deviation 1.65) months. Half (49.4%, 129/261) of the patients completed the intensive phase of treatment in two months, whereas only 37.6% (85/226) of the patients completed the continuation phase of treatment in four months. On multiple logistic regression analysis, being a smoker, being underweight and having a history of cough for >4 weeks at TB diagnosis were found to be predictive of a prolonged intensive phase of treatment. Diabetes mellitus and the presence of lung cavities at the start of treatment were the only predictors found for a prolonged continuation phase of treatment. Conclusions: The average durations of the intensive and continuation phases of treatment among PTB patients were longer than the targets recommended by the World Health Organization. As there are no internationally agreed criteria, it was not possible to judge how well the Malaysian NTP performed in terms of managing treatment duration among PTB patients.

Institution

(Atif) Department of Pharmacy, Islamia University of Bahawalpur, Punjab, Pakistan (Sulaiman) Discipline of Clinical Pharmacy, School of Pharmaceutical Sciences, Universiti Sains Malaysia, Penang, Malaysia

(Shafie) Discipline of Social and Administrative Pharmacy, School of Pharmaceutical Sciences, Universiti Sains Malaysia, Penang, Malaysia

(Babar) Division of Pharmacy Practice, School of Pharmacy, Faculty of Medical and Health Sciences, University of Auckland, Auckland, New Zealand

Publisher

Elsevier

Emtree Heading

adult; article; Chinese; cohort analysis; comorbidity; continuation phase; coughing; diabetes mellitus; female; human; hypertension; intensive phase; lung cavity; *lung tuberculosis/dt [Drug Therapy]; major clinical study; Malay (people); Malaysia; male; medical history; middle aged; *practice guideline; risk factor; smoking; Tamil (people); *treatment duration; treatment outcome; treatment planning; underweight; ethambutol/cb [Drug Combination]; ethambutol/dt [Drug Therapy]; isoniazid/cb [Drug Combination]; isoniazid/dt [Drug Therapy]; pyrazinamide/cb [Drug Combination]; pyrazinamide/dt [Drug Therapy]; rifampicin/cb [Drug Combination]; rifampicin/dt [Drug Therapy].

Link to the Ovid Full Text or citation:

Click here for full text options

60.

Smoking on treatment outcomes among tuberculosis patients.

Khan A.H., Israr M., Khan A., Aftab R.A., Khan T.M.

American Journal of the Medical Sciences. 349 (6) (pp 505-509), 2015. Date of Publication: 06 Jun 2015.

AN: 2015105159

Background: Smoking is a risk factor not only for the development of cancer and coronary heart disease but also for tuberculosis (TB). The aim of this study was to determine the prevalence of smoking in patients with TB, identify demographic and clinical characteristics associated with smoking and to evaluate TB treatment outcomes in the smokers. Method: A retrospective cohort study of patients with TB was conducted at Khyber Teaching Hospital, Peshawar, Pakistan. All patients with pulmonary and extrapulmonary TB and those coinfected with HIV, hepatitis or diabetes mellitus were included in the study. The patients were categorized into smokers and nonsmokers. Treatment outcomes were evaluated by smear testing at the end of the treatment. Results: Of 472 enrolled subjects, 68 (14.4%) were smokers. The prevalence of smoking among male and female patients with TB was 11.8% and 2.5%, respectively. Univariate analysis indicated that the gender, age group and marital status of patients with TB were associated with smoking. The results indicated that patient gender (P 0.05), age: 15 to 24 years (P 0.05) and age >55 years (P 0.004) were risk factors associated with smoking among TB patients. Of the 68 smokers with TB, the treatment outcomes among 54 patients (79.4%) were unsuccessful. The treatment outcomes was statistically significantly associated with smoking (odds ratio: 2.58, P 0.004). Conclusions: Findings from the current study proved smoking to be one of the main factors associated with the occurrence of TB and significantly reducing the outcomes of TB therapy.

Institution

(Khan, Aftab) Department of Clinical Pharmacy, School of Pharmaceutical Sciences, Universiti Sains Malaysia, Penang 11800, Malaysia (Israr) Department of Respiratory Diseases, District TB Office Bannu, Bannu, Pakistan

(Khan) Medical B Unit, Khyber Teaching Hospital, Peshawar, Pakistan

(Khan) School of Pharmacy, Monash University, Selangor Darul Ehsan, Malaysia

Publisher

Lippincott Williams and Wilkins

Emtree Heading

adolescent; adult; article; clinical feature; controlled clinical trial; controlled study; demography; diabetes mellitus; *extrapulmonary tuberculosis/dt [Drug Therapy]; female; groups by age; hepatitis; human; Human immunodeficiency virus infection; *lung tuberculosis/dt [Drug Therapy]; major clinical study; male; marriage; mixed infection; prevalence; retrospective study; risk factor; *smoking; sputum smear; *treatment outcome; *tuberculostatic agent/dt [Drug Therapy].

Link to the Ovid Full Text or citation:

Click here for full text options

61.

Diabetes mellitus is associated with cavities, smear grade, and multidrug-resistant tuberculosis in Georgia.

Magee M.J., Kempker R.R., Kipiani M., Gandhi N.R., Darchia L., Tukvadze N., Howards P.P., Narayan K.M.V., Blumberg H.M.

International Journal of Tuberculosis and Lung Disease. 19 (6) (pp 685-692), 2015. Date of Publication: 01 Jun 2015.

AN: 2015052742

SETTING: National tuberculosis (TB) treatment facility in the country of Georgia. OBJECTIVE: To determine the prevalence of diabetes mellitus (DM) and pre-DM among patients with TB using glycosylated-hemoglobin (HbA1c), and to estimate the association between DM and clinical characteristics and response to anti-tuberculosis treatment. DESIGN: A cohort study was conducted from 2011 to 2014 at the National Centre for TB and Lung Disease in Tbilisi. Patients aged >35 years with pulmonary TB were included. HbA1c was used to defineDM(>6.5%), pre-DM (>5.7-6.4%), and no DM (<5.7%). Interviews and medical chart abstraction were performed. Regression analyses estimated associations between DM and 1) baseline TB characteristics and 2) anti-tuberculosis treatment outcomes. RESULTS : A total of 318 newly diagnosed patients with TB were enrolled. The prevalence of DM and pre-DM was 11.6% and 16.4%, respectively. In multivariable analyses, patients with TB-DM had more cavitation (adjusted OR [aOR] 2.26), higher smear grade (aOR 2.37), and more multidrug-resistant TB (MDR-TB) (aOR 2.27) than patients without DM. The risk of poor anti-tuberculosis treatment outcomes was similar among patients with and those without DM(28.1%vs. 23.6%). CONCLUSION: DM and pre-DM were common among adults with newly diagnosed pulmonary TB in Tbilisi, Georgia, and DM was associated with more clinical symptoms, and MDR-TB, at presentation.

Institution

(Magee) Division of Epidemiology and Biostatistics, School of Public Health, Georgia State University, One Park Place NE, Atlanta, GA 30303, United States (Magee, Gandhi, Howards, Narayan, Blumberg) Department of Epidemiology and Global Health, Rollins School of Public Health, Emory University, Atlanta, GA, United States

(Kempker, Gandhi, Blumberg) Division of Infectious Diseases, Department of Medicine, Emory University School of Medicine, Atlanta, GA, United States

(Kipiani, Darchia, Tukvadze) National Center for Tuberculosis and Lung Diseases, Tbilisi, Georgia

Publisher

International Union against Tubercul. and Lung Dis.

Emtree Heading

acid fast bacterium; adult; aged; alcohol consumption; article; clinical feature; cohort analysis; controlled study; *diabetes mellitus/di [Diagnosis]; disease association; female; follow up; Georgia (republic); hemoptysis; human; impaired glucose tolerance/di [Diagnosis]; *lung cavitation; major clinical study; male; middle aged; *multidrug resistant tuberculosis/dt [Drug Therapy]; Mycobacterium tuberculosis; prevalence; priority journal; risk assessment; risk factor; smoking; sputum culture; *sputum smear; treatment outcome; hemoglobin A1c/ec [Endogenous Compound]; tuberculostatic agent/dt [Drug Therapy].

Link to the Ovid Full Text or citation:

Click here for full text options

62.

Nonopioid Medications for Pain.

Tauben D.

Physical Medicine and Rehabilitation Clinics of North America. 26 (2) (pp 219-248), 2015. Date of Publication: 01 May 2015.

AN: 2015049328

Evidence of nonopioid analgesic effectiveness exceeds that for long-term opioids in chronic noncancer pain (CNCP), most with lower risk. Non-drug therapies such as cognitive behavioral therapy and physical activation are safer and also effective. Nonsteroidal antiinflammatory drugs are useful for inflammatory and nociceptive pain, share renal and variable gastrointestinal, bleeding and cardiovascular side effects. Antidepressants with noradrenergic activity (such as tricyclics and seroton-norepinephrine reuptake inhibitors) and neuromodulating anticonvulsant drugs (gabapentinoids and sodium-channel blockers) are proven to be effective for neuropathic and centralized pain. Ketamine and cannabinoids are other studied analgesics but have a less well-proven role in CNCP.

Institution

(Tauben) Division of Pain Medicine, Department of Anesthesiology and Pain Medicine, UW Center for Pain Relief, University of Washington, 1959 Northeast Pacific Street, Box 356540, Seattle, WA 98195, United States (Tauben) Division of General Internal Medicine, Department of Medicine, UW Center for Pain Relief, University of Washington, 1959 Northeast Pacific Street, Box 356540, Seattle, WA 98195, United States

Publisher

W.B. Saunders

Emtree Heading

acupuncture; alcoholic beverage; *analgesia; analgesic activity; analgesic nephropathy/si [Side Effect]; anticholinergic syndrome/si [Side Effect]; application site pain/si [Side Effect]; application site reaction/si [Side Effect]; arthritis/dt [Drug Therapy]; asthma/si [Side Effect]; balance disorder/si [Side Effect]; cannabis addiction/si [Side Effect]; cardiovascular disease; cardiovascular risk; chronic bronchitis/si [Side Effect]; *chronic pain/dm [Disease Management]; *chronic pain/et [Etiology]; *chronic pain/th [Therapy]; *chronic pain/dt [Drug Therapy]; clinical trial(topic); cognitive defect/si [Side Effect]; cognitive therapy; comparative effectiveness; constipation/si [Side Effect]; cost control; diabetic nephropathy/dt [Drug Therapy]; diabetic neuropathy/dt [Drug Therapy]; dose kidney function relation; drug absorption; drug alcohol interaction; drug bioavailability; drug choice; drug classification; drug contraindication; drug dose reduction; drug efficacy; drug hypersensitivity/si [Side Effect]; drug mechanism; drug megadose; drug misuse; drug monitoring; drug potency; drug potentiation; drug safety; drug selectivity; drug tolerability; drug withdrawal; edema/si [Side Effect]; endometriosis/dt [Drug Therapy]; enthesopathy/dt [Drug Therapy]; evidence based medicine; face pain/dt [Drug Therapy]; fever/dt [Drug Therapy]; fibromyalgia/dt [Drug Therapy]; fracture nonunion/si [Side Effect]; glucose intolerance/si [Side Effect]; hallucination/si [Side Effect]; headache/dt [Drug Therapy]; health care cost; human; hyperpyrexia/si [Side Effect]; hypnosis; inflammatory pain/dt [Drug Therapy]; insomnia/th [Therapy]; insomnia/dt [Drug Therapy]; interstitial nephritis/si [Side Effect]; intraocular hypertension/si [Side Effect]; kidney papilla necrosis/si [Side Effect]; kinesiotherapy; knee osteoarthritis/dt [Drug Therapy]; liver failure/si [Side Effect]; low drug dose; lupus vulgaris/dt [Drug Therapy]; maximum plasma concentration; medical decision making; memory disorder/si [Side Effect]; migraine/dt [Drug Therapy]; multiple sclerosis/dt [Drug Therapy]; muscle spasm/dt [Drug Therapy]; myositis/dt [Drug Therapy]; nausea/si [Side Effect]; nephrotoxicity/si [Side Effect]; nephrotoxicity/si [Side Effect]; neuritis/dt [Drug Therapy]; neurolysis/si [Side Effect]; neuromodulation; neuropathic pain/dt [Drug Therapy]; nociceptive pain/dt [Drug Therapy]; nonhuman; orthostatic hypotension/si [Side Effect]; osteoarthritis/dt [Drug Therapy]; outcome assessment; pain assessment; pathophysiology; patient satisfaction; pelvis pain syndrome/dt [Drug Therapy]; peripheral neuropathy/dt [Drug Therapy]; pharmacodynamics; physical activity; polypharmacy; postherpetic neuralgia/dt [Drug Therapy]; postoperative nausea and vomiting/dt [Drug Therapy]; prescription; priority journal; prostate hypertrophy/si [Side Effect]; psychosocial care; QT prolongation/si [Side Effect]; quality of life; radiculopathy/dt [Drug Therapy]; randomized controlled trial(topic); review; Reye syndrome/si [Side Effect]; rhabdomyolysis/si [Side Effect]; rheumatic disease/dt [Drug Therapy]; rheumatic polymyalgia/dt [Drug Therapy]; rheumatoid arthritis/dt [Drug Therapy]; risk benefit analysis; sedation; sensitization; sexual dysfunction/si [Side Effect]; side effect/si [Side Effect]; sleep quality; sodium retention; spondyloarthropathy/dt [Drug Therapy]; stiff man syndrome/dt [Drug Therapy]; tendinitis/dt [Drug Therapy]; tension headache/dt [Drug Therapy]; trigeminus neuralgia/dt [Drug Therapy]; urine retention/si [Side Effect]; xerostomia/si [Side Effect]; yoga; acetylsalicylic acid/do [Drug Dose]; acetylsalicylic acid/ae [Adverse Drug Reaction]; acetylsalicylic acid/cb [Drug Combination]; anticonvulsive agent/ae [Adverse Drug Reaction]; anticonvulsive agent/dt [Drug Therapy]; antidepressant agent/pd [Pharmacology]; antidepressant agent/it [Drug Interaction]; antidepressant agent/dt [Drug Therapy]; baclofen/po [Oral Drug Administration]; baclofen/ae [Adverse Drug Reaction]; baclofen/dt [Drug Therapy]; benzodiazepine derivative/dt [Drug Therapy]; calcium channel blocking agent/dt [Drug Therapy]; cannabis/ct [Clinical Trial]; cannabis/ae [Adverse Drug Reaction]; cannabis/dt [Drug Therapy]; capsaicin/td [Transdermal Drug Administration]; capsaicin/pd [Pharmacology]; capsaicin/do [Drug Dose]; capsaicin/ae [Adverse Drug Reaction]; capsaicin/dt [Drug Therapy]; carbamazepine/pd [Pharmacology]; carbamazepine/dt [Drug Therapy]; corticosteroid/ae [Adverse Drug Reaction]; corticosteroid/ei [Epidural Drug Administration]; corticosteroid/dt [Drug Therapy]; diclofenac/td [Transdermal Drug Administration]; diclofenac/pk [Pharmacokinetics]; diclofenac/ae [Adverse Drug Reaction]; diclofenac/dt [Drug Therapy]; diphenhydramine/dt [Drug Therapy]; ketamine/po [Oral Drug Administration]; ketamine/td [Transdermal Drug Administration]; ketamine/pd [Pharmacology]; ketamine/ae [Adverse Drug Reaction]; ketamine/bd [Buccal Drug Administration]; ketamine/dt [Drug Therapy]; lidocaine/td [Transdermal Drug Administration]; lidocaine/dt [Drug Therapy]; methylprednisolone/po [Oral Drug Administration]; methylprednisolone/do [Drug Dose]; methylprednisolone/dt [Drug Therapy]; muscle relaxant agent/dt [Drug Therapy]; nonsteroid antiinflammatory agent/pd [Pharmacology]; nonsteroid antiinflammatory agent/ae [Adverse Drug Reaction]; nonsteroid antiinflammatory agent/cb [Drug Combination]; nonsteroid antiinflammatory agent/it [Drug Interaction]; nonsteroid antiinflammatory agent/iv [Intravenous Drug Administration]; nonsteroid antiinflammatory agent/po [Oral Drug Administration]; nonsteroid antiinflammatory agent/td [Transdermal Drug Administration]; nonsteroid antiinflammatory agent/dt [Drug Therapy]; paracetamol/pd [Pharmacology]; paracetamol/ae [Adverse Drug Reaction]; paracetamol/dt [Drug Therapy]; phenacetin/ae [Adverse Drug Reaction]; phenacetin/cb [Drug Combination]; phenacetin/dt [Drug Therapy]; phenytoin/it [Drug Interaction]; prednisone/po [Oral Drug Administration]; prednisone/do [Drug Dose]; prednisone/dt [Drug Therapy]; serotonin noradrenalin reuptake inhibitor/ct [Clinical Trial]; serotonin noradrenalin reuptake inhibitor/pd [Pharmacology]; serotonin noradrenalin reuptake inhibitor/ae [Adverse Drug Reaction]; serotonin noradrenalin reuptake inhibitor/dt [Drug Therapy]; serotonin uptake inhibitor/ct [Clinical Trial]; serotonin uptake inhibitor/pd [Pharmacology]; serotonin uptake inhibitor/dt [Drug Therapy]; sodium channel blocking agent/dt [Drug Therapy]; spasmolytic agent/dt [Drug Therapy]; tizanidine/pd [Pharmacology]; tizanidine/ae [Adverse Drug Reaction]; tizanidine/dt [Drug Therapy]; tramadol/pd [Pharmacology]; tramadol/dt [Drug Therapy]; tricyclic antidepressant agent/ct [Clinical Trial]; tricyclic antidepressant agent/pd [Pharmacology]; tricyclic antidepressant agent/ae [Adverse Drug Reaction]; tricyclic antidepressant agent/dt [Drug Therapy]; unindexed drug; warfarin/it [Drug Interaction].

Drug Trade Names and Manufacturers

pennsaid

Link to the Ovid Full Text or citation:

Click here for full text options

63.

Diabetes and other risk factors for multi-drug resistant tuberculosis in a mexican population with pulmonary tuberculosis: Case control study.

Gomez-Gomez A., Magana-Aquino M., Lopez-Meza S., Aranda-Alvarez M., Diaz-Ornelas D.E., Hernandez-Segura M.G., Salazar-Lezama M.T., Castellanos-Joya M., Noyola D.E.

Archives of Medical Research. 46 (2) (pp 142-148), 2015. Date of Publication: 01 Feb 2015.

AN: 2015002238

Background and Aims: Multidrug resistant tuberculosis (MDR-TB) poses problems in treatment, costs and treatment outcomes. It is not known if classically described risk factors for MDR-TB in other countries are the same in Mexico and the frequency of the association between diabetes mellitus (DM) and MDR-TB in our country is not clear. We undertook this study to analyze risk factors associated with the development of MDR-TB, with emphasis on DM. Methods: A case-control study in the state of San Luis Potosi (SLP), Mexico was carried out. All pulmonary MDR-TB patients diagnosed in the state of SLP between 1998 and 2013 (36 cases) evaluated at a state pharmacoresistant tuberculosis (TB) clinic and committee; 139 controls were randomly selected from all pulmonary non-multidrug-resistant tuberculosis (non-MDR-TB) cases identified between 2003 and 2008. Cases and controls were diagnosed and treated under programmatic conditions. Results: Age, gender, malnutrition, being a health-care worker, HIV/AIDS status, and drug abuse were not significantly different between MDR-TB and non-MDR-TB patients. Significant differences between MDR-TB and non-MDR-TB patients were DM (47.2 vs. 28.1%; p=0.028); previous anti-TB treatments (3 vs. 0, respectively; p<0.001), and duration of first anti-TB treatment (8 vs. 6months, respectively; p<0.001). Conclusions: MDR-TB and DM are associated in 47.2% of MDR TB cases (17/36) in this study. Other recognized factors were not found to be significantly different in MDR-TB compared to non-MDR-TB in this study. Cost-feasible strategies must be implemented in the treatment of DM-TB in order to prevent the selection of MDR-TB.

Institution

(Gomez-Gomez, Magana-Aquino) Division de Medicina Interna, Hospital Central Dr. Ignacio Morones Prieto, San Luis Potosi, Mexico (Gomez-Gomez, Magana-Aquino) Departamento de Medicina Interna, Facultad de Medicina, Universidad Autonoma de San Luis Potosi, San Luis Potosi, Mexico

(Gomez-Gomez, Magana-Aquino) Comite Estatal de Tuberculosis Farmacorresistente, San Luis Potosi, Mexico

(Lopez-Meza) Programa de Atencion al Adulto Mayor, San Luis Potosi, Mexico

(Aranda-Alvarez) Direccion de Politicas y Calidad en Salud, San Luis Potosi, Mexico

(Diaz-Ornelas) Programa de Micobacteriosis Servicios de Salud del Estado de San Luis Potosi, San Luis Potosi, Mexico

(Hernandez-Segura) Servicio de Medicina Preventiva, Hospital Central Dr. Ignacio Morones Prieto, San Luis Potosi, Mexico

(Salazar-Lezama) Asesor Nacional en Tuberculosis Farmacorresistente, Instituto Nacional de Enfermedades Respiratorias, Mexico, D.F., Mexico

(Castellanos-Joya) Direccion Programa Nacional de Tuberculosis, Secretaria de Salud, Mexico, D.F., Mexico

(Noyola) Departmento de Microbiologia, Facultad de Medicina, Universidad Autonoma de San Luis Potosi, San Luis Potosi, Mexico

Publisher

Elsevier Inc.

Emtree Heading

acquired immune deficiency syndrome; adult; age; antibiotic sensitivity; article; controlled study; *diabetes mellitus; drug abuse; female; health care personnel; human; Human immunodeficiency virus infected patient; Human immunodeficiency virus infection; *infection risk; *lung tuberculosis; major clinical study; male; malnutrition; Mexican; *multidrug resistant tuberculosis/dr [Drug Resistance]; polymerase chain reaction; population based case control study; sex difference; smoking; sputum culture; treatment duration; tuberculostatic agent.

Link to the Ovid Full Text or citation:

Click here for full text options

64.

Diabetes and the risk of infections with immunomodulator therapy in inflammatory bowel diseases.

Ananthakrishnan A.N., Cagan A., Cai T., Gainer V.S., Shaw S.Y., Churchill S., Karlson E.W., Murphy S.N., Kohane I., Liao K.P.

Alimentary Pharmacology and Therapeutics. 41 (11) (pp 1141-1148), 2015. Date of Publication: 01 Jun 2015.

AN: 2015929997

Background Infections are an important concern in patients using immunosuppressive therapy for their inflammatory bowel disease (IBD). Diabetes affects nearly 10% of Americans. Whether it confers an additional risk with immunosuppression in IBD has not been examined previously. Aim To examine the association between diabetes and infections with immunomodulator use in IBD Methods Using a validated, multi-institutional IBD cohort, we identified all patients who received at least one prescription for immunomodulators (thiopurines, methotrexate). Our primary outcome was infection within 1 year of the prescription of the immunomodulator. Multivariable logistic regression adjusting for relevant confounders was used to estimate the independent association with diabetes. Results Our study included 2766 patients receiving at least one prescription for immunomodulators among whom 210 (8%) developed an infection within 1 year. Patients who developed an infection were likely to be older, have more comorbidities, more likely to have received a prescription for steroids but similar in initiation of anti-TNF therapy within that year. Only 8% of those without an infection had diabetes compared to 19% of those who developed an infection within 1 year [odds ratio (OR) 2.74, 95% confidence interval (CI) 1.88-3.98, P < 0.001]. On multivariate analysis, diabetes was independently associated with a nearly two-fold increase in risk of infections (OR: 1.80, 95% CI: 1.20-2.68). There was no increase in risk of infections with addition of anti-TNF therapy (OR: 1.14, 95% CI: 0.80-1.63). Conclusion Diabetes is an independent risk factor for infection in IBD patients using immunomodulator therapy.

Institution

(Ananthakrishnan) Division of Gastroenterology, Massachusetts General Hospital, Boston, MA, United States (Ananthakrishnan, Shaw, Karlson, Murphy, Kohane, Liao) Harvard Medical School, Boston, MA, United States

(Ananthakrishnan, Shaw, Murphy) Department of Medicine, Massachusetts General Hospital, Boston, MA, United States

(Cagan, Gainer) Research is and Computing, Partners HealthCare, Charlestown, MA, United States

(Cai) Department of Biostatistics, Harvard School of Public Health, Boston, MA, United States

(Shaw) Center for Systems Biology, Massachusetts General Hospital, Boston, MA, United States

(Churchill, Murphy, Kohane) I2b2 National Center for Biomedical Computing, Brigham and Women's Hospital, Boston, MA, United States

(Karlson, Liao) Division of Rheumatology, Allergy and Immunology, Brigham and Women's Hospital, Boston, MA, United States

(Murphy) Department of Neurology, Massachusetts General Hospital, Boston, MA, United States

(Kohane) Children's Hospital Boston, Boston, MA, United States

Publisher

Blackwell Publishing Ltd

Emtree Heading

adult; article; aspergillosis; bacteremia; cellulitis; cohort analysis; comorbidity; controlled study; Crohn disease; cytomegalovirus infection; *diabetes mellitus; disease association; Epstein Barr virus infection; female; follow up; herpes simplex; herpes zoster; human; *immunosuppressive treatment; infection/si [Side Effect]; *infection risk; *inflammatory bowel disease/dt [Drug Therapy]; influenza; lung abscess; major clinical study; male; meningitis; outcome assessment; pneumonia; population research; prescription; priority journal; sepsis; Streptococcus infection; tuberculosis; ulcerative colitis; urinary tract infection; azathioprine/ae [Adverse Drug Reaction]; azathioprine/dt [Drug Therapy]; *immunomodulating agent/ae [Adverse Drug Reaction]; *immunomodulating agent/dt [Drug Therapy]; mercaptopurine/ae [Adverse Drug Reaction]; mercaptopurine/dt [Drug Therapy]; methotrexate/ae [Adverse Drug Reaction]; methotrexate/dt [Drug Therapy].

Link to the Ovid Full Text or citation:

Click here for full text options

65.

Effect of diabetes on tuberculosis control in 13 countries with high tuberculosis: A modelling study.

Pan S.-C., Ku C.-C., Kao D., Ezzati M., Fang C.-T., Lin H.-H.

The Lancet Diabetes and Endocrinology. 3 (5) (pp 323-330), 2015. Date of Publication: 01 May 2015.

AN: 2015851385

Background: Diabetes increases the risk of tuberculosis incidence and the risk of adverse treatment outcomes in patients with tuberculosis. Because prevalence of diabetes is increasing in low-income and middle-income countries where the burden of tuberculosis is high, prevention of diabetes carries the potential to improve tuberculosis control worldwide. Methods: We used dynamic tuberculosis transmission models to analyse the potential effect of diabetes on tuberculosis epidemiology in 13 countries with high tuberculosis burden. We used data for previous diabetes prevalence in each country and constructed scenarios to represent the potential ranges of future diabetes prevalence. The country-specific model was calibrated to the estimated trend of tuberculosis incidence. We estimated the tuberculosis burden that can be reduced by alternative scenarios of diabetes prevention. Findings: If the prevalence of diabetes continues to rise as it has been in the past decade in the 13 countries (base case scenario), by 2035, the cumulative reduction in tuberculosis incidence would be 8.8% (95% credible interval [CrI] 4.0-15.8) and mortality would be 34.0% (30.3-39.6). Lowering the prevalence of diabetes by an absolute level of 6.6-13.8% could accelerate the decline of tuberculosis incidence by an absolute level of 11.5-25.2% and tuberculosis mortality by 8.7-19.4%. Compared with the base case scenario, stopping the rise of diabetes would avoid 6.0 million (95% CrI 5.1-6.9) incident cases and 1.1 million (1.0-1.3) tuberculosis deaths in 13 countries during 20 years. If interventions reduce diabetes incidence by 35% by 2025, 7.8 million (6.7-9.0) tuberculosis cases and 1.5 million (1.3-1.7) tuberculosis deaths could be averted by 2035. Interpretation: The diabetes epidemic could substantially affect tuberculosis epidemiology in high burden countries. The communicable disease and non-communicable disease sectors need to move beyond conventional boundaries and link with each other to form a joint response to diabetes and tuberculosis. Funding: Taiwan National Science Council.

Institution

(Pan, Fang) Department of Internal Medicine, National Taiwan University Hospital, Taipei, Taiwan (Republic of China) (Pan, Ku, Fang, Lin) Institute of Epidemiology and Preventive Medicine, College of Public Health, National Taiwan University, Taipei, Taiwan (Republic of China)

(Kao) Department of Epidemiology and Biostatistics, University of British Columbia, Vancouver, Canada

(Ezzati) MRC-PHE Centre for Environment and Health, Department of Epidemiology and Biostatistics, School of Public Health, Imperial College, London, United Kingdom

Publisher

Lancet Publishing Group

Emtree Heading

Afghanistan; article; bacterial transmission; Bangladesh; Brazil; Cambodia; China; compartment model; death; *diabetes mellitus/ep [Epidemiology]; epidemic; human; incidence; India; Indonesia; mortality; Myanmar; Pakistan; Philippines; prevalence; priority journal; recurrence risk; risk factor; Russian Federation; Thailand; *tuberculosis/ep [Epidemiology]; *tuberculosis/pc [Prevention]; *tuberculosis control; Viet Nam.

Link to the Ovid Full Text or citation:

Click here for full text options

66.

Optimal duration of anti-TB treatment in patients with diabetes: Nine or six months?.

Wang J.-Y., Lee M.-C., Shu C.-C., Lee C.-H., Lee L.-N., Chao K.-M., Chang F.-Y.

Chest. 147 (2) (pp 520-528), 2015. Date of Publication: 01 Feb 2015.

AN: 2015970600

BACKGROUND: Diabetes mellitus (DM) increases the risk of TB recurrence. This study investigated whether 9-month anti-TB treatment is associated with a lower risk of TB recurrence within 2 years after complete treatment than 6-month treatment in patients with DM with an emphasis on the impact of directly observed therapy, short course (DOTs). METHODS: Patients with pulmonary but not extrapulmonary TB receiving treatment of 173 to 277 days between 2002 and 2010 were identified from the National Health Insurance Research Database of Taiwan. Patients with DM were then selected and classified into two groups based on anti-TB treatment duration (9 months vs 6 months). Factors predicting 2-year TB recurrence were explored using Cox regression analysis. RESULTS: Among 12,688 patients with DM and 43,195 patients without DM, the 2-year TB recurrence rate was 2.20% and 1.38%, respectively (P < .001). Of the patients with DM, recurrence rate decreased from 3.54% to 1.19% after implementation of DOTs (P < .001). A total of 4,506 (35.5%) were classified into 9-month anti-TB treatment group. Although a 9-month anti-TB treatment was associated with a lower recurrence rate (hazard ratio, 0.76 [95% CI, 0.59-0.97]), the benefit disappeared (hazard ratio, 0.69 [95% CI, 0.43-1.11]) under DOTs. Other predictors of recurrence included older age, male sex, malignancy, earlier TB diagnosis year, culture positivity after 2 months of anti-TB treatment, and anti-TB treatment being < 80% consistent with standard regimen. CONCLUSIONS: The 2-year TB recurrence rate is higher in a diabetic population in Taiwan and can be reduced by treatment supervision. Extending the anti-TB treatment by 3 months may also decrease the recurrence rate when treatment is not supervised.

Institution

(Wang) Department of Internal Medicine, National Taiwan University Hospital, Taipei, Taiwan (Republic of China) (Shu) Department of Traumatology, National Taiwan University Hospital, Taipei, Taiwan (Republic of China)

(Lee) Department of Laboratory Medicine, National Taiwan University Hospital, Taipei, Taiwan (Republic of China)

(Lee) Department of Pharmacy, Taipei Tzuchi Hospital, Buddhist Tzuchi Medical Foundation, New Taipei, Taiwan (Republic of China)

(Lee) Department of Pulmonary Medicine, Wanfang Hospital, Taipei Medical University, Hsing-Long Rd, Taipei 116, Taiwan (Republic of China)

(Chao) Graduate Institute of Biomedical Electronics and Bioinformatics, National Taiwan University, Taipei, Taiwan (Republic of China)

(Chang) Centers for Disease Control, Taipei, Taiwan (Republic of China)

Publisher

American College of Chest Physicians

Emtree Heading

adult; age; aged; article; bacterium culture; clinical supervision; controlled study; *diabetes mellitus/dt [Drug Therapy]; disease association; disease course; early diagnosis; female; human; *lung tuberculosis/dt [Drug Therapy]; major clinical study; male; patient compliance; prediction; priority journal; recurrence risk; recurrent infection; risk assessment; risk factor; sex difference; short course therapy; Taiwan; *treatment duration; treatment outcome; antidiabetic agent/dt [Drug Therapy]; ethambutol/dt [Drug Therapy]; isoniazid/dt [Drug Therapy]; pyrazinamide/dt [Drug Therapy]; rifampicin/dt [Drug Therapy]; *tuberculostatic agent/dt [Drug Therapy].

Link to the Ovid Full Text or citation:

Click here for full text options

67.

Long-term outcome in patients with obscure gastrointestinal bleeding after capsule endoscopy.

Tan W., Ge Z.Z., Gao Y.J., Li X.B., Dai J., Fu S.W., Zhang Y., Xue H.B., Zhao Y.J.

Journal of Digestive Diseases. 16 (3) (pp 125-134), 2015. Date of Publication: 01 Mar 2015.

AN: 2015862539

Objective: This study aimed to identify the risk factors associated with rebleeding and long-term outcomes after capsule endoscopy (CE) for obscure gastrointestinal bleeding (OGIB) in a follow-up study. Methods: Data of consecutive patients who underwent CE due to OGIB from June 2002 to January 2012 were retrospectively reviewed. The Cox proportional hazard model was used to evaluate the risk factors associated with rebleeding, while Kaplan-Meier survival curves and the log-rank test were used to analyze cumulative rebleeding rates. Results: The overall rebleeding rate after CE in patients with OGIB was 28.6% (97/339) during a median follow-up of 48 months (range 12-112 months). Multivariate analysis showed that age >60 years (hazard ratio [HR] 2.473, 95% confidence interval [CI] 1.576-3.881, P=0.000), positive CE findings (HR 3.393, 95% CI 1.931-5.963, P=0.000), hemoglobin <70g/L before CE (HR 2.010, 95% CI 1.261-3.206, P=0.003), nonspecific treatments (HR 2.500, 95% CI 1.625-3.848, P=0.000) and the use of anticoagulants, antiplatelet or non-steroidal anti-inflammatory drugs after CE (HR 2.851, 95% CI 1.433-5.674, P=0.003) were independent risk factors associated with rebleeding. Univariate analysis showed that chronic hepatitis was independently associated with rebleeding in CE-negative patients (P=0.021). Conclusions: CE has a significant impact on the long-term outcome of patients with OGIB. Further investigation and close follow-up in patients with OGIB and those with negative CE findings are necessary.

Institution

(Tan, Ge, Gao, Li, Dai, Fu, Zhang, Xue, Zhao) Key Laboratory of Gastroenterology and Hepatology, Ministry of Health, Division of Gastroenterology and Hepatology, Ren Ji Hospital, Sch. of Medicine, Shanghai Jiao Tong Univ. Shanghai Insti. of Digestive Disease, Shanghai, China (Tan) Department of Gastroenterology, Changzheng Hospital, Second Military Medical University, 415 Fengyang Road, Shanghai 200003, China

Publisher

Blackwell Publishing

Emtree Heading

adult; angiodysplasia/di [Diagnosis]; angiodysplasia/dt [Drug Therapy]; article; autoimmune disease; blood transfusion; *capsule endoscopy; Chinese medicine; chronic hepatitis; chronic kidney failure; colonoscopy; Crohn disease/dt [Drug Therapy]; diabetes mellitus; digital subtraction angiography; diverticulosis/di [Diagnosis]; drug use; female; follow up; *gastrointestinal hemorrhage; heart disease; hormonal therapy; human; hypertension; liver cirrhosis; lung tuberculosis; lymphangiectasis/di [Diagnosis]; major clinical study; medical record review; occult blood; outcome assessment; parasitosis/di [Diagnosis]; priority journal; recurrent disease; retrospective study; risk factor; small intestine tumor/di [Diagnosis]; aminosalicylic acid/dt [Drug Therapy]; anticoagulant agent; antithrombocytic agent; hemoglobin/ec [Endogenous Compound]; nonsteroid antiinflammatory agent; proton pump inhibitor; thalidomide/dt [Drug Therapy].

Link to the Ovid Full Text or citation:

Click here for full text options

68.

Mortality among MDR-TB cases: Comparison with drug-susceptible tuberculosis and associated factors.

Chung-Delgado K., Guillen-Bravo S., Revilla-Montag A., Bernabe-Ortiz A.

PLoS ONE. 10 (3) (no pagination), 2015. Article Number: e0119332. Date of Publication: 19 Mar 2015.

AN: 2015861675

Background: An increase in multidrug-resistant tuberculosis (MDR-TB) cases is evident worldwide. Its management implies a complex treatment, high costs, more toxic anti-tuberculosis drug use, longer treatment time and increased treatment failure and mortality. The aims of this study were to compare mortality between MDR and drug-susceptible cases of tuberculosis, and to determine risk factors associated with mortality among MDR-TB cases. Methods and Results: A retrospective cohort study was performed using data from clinical records of the National Strategy for Prevention and Control of Tuberculosis in Lima, Peru. In the first objective, MDR-TB, compared to drug-susceptible cases, was themain exposure variable and time to death, censored at 180 days, the outcome of interest. For the second objective, different variables obtained from clinical records were assessed as potential risk factors for death among MDR-TB cases. Cox regression analysis was used to determine hazard ratios (HR) and 95%confidence intervals (95%CI). A total of 1,232 patients were analyzed: mean age 30.9 +/-14.0 years, 60.0%were males. 61 patients (5.0%) died during treatment, whereas the MDR-TB prevalence was 19.2%. MDR-TB increased the risk of death during treatment (HR = 7.5; IC95%: 4.1-13.4) when compared to presumed drug-susceptible cases after controlling for potential confounders. Education level (p = 0.01), previous TB episodes (p<0.001), diabetes history (p<0.001) and HIV infection (p = 0.04) were factors associated with mortality among MDR-TB cases. Conclusions: MDR-TB is associated with an increased risk of death during treatment. Lower education, greater number of previous TB episodes, diabetes history, and HIV infection were independently associated with mortality among MDR-TB cases. New strategies for appropriate MDR-TB detection and management should be implemented, including drug sensitivity tests, diabetes and HIV screening, as well as guarantee for a complete adherence to therapy.

Institution

(Chung-Delgado, Guillen-Bravo, Revilla-Montag, Bernabe-Ortiz) Escuela de Medicina, Universidad Peruana de Ciencias Aplicadas, Lima, Peru

Publisher

Public Library of Science

Emtree Heading

adult; article; cohort analysis; controlled study; diabetes mellitus; *drug susceptible tuberculosis; drug susceptible tuberculosis/dt [Drug Therapy]; educational status; female; high risk patient; human; Human immunodeficiency virus infection; major clinical study; male; medical history; mixed infection; *mortality; *multidrug resistant tuberculosis/dt [Drug Therapy]; outcome assessment; Peru; prevalence; retrospective study; risk assessment; *risk factor; *tuberculosis/dt [Drug Therapy]; tuberculostatic agent/dt [Drug Therapy].

Link to the Ovid Full Text or citation:

Click here for full text options

69.

Safety of inhaled corticosteroids for treating chronic obstructive pulmonary disease.

Matera M.G., Cardaci V., Cazzola M., Rogliani P.

Expert Opinion on Drug Safety. 14 (4) (pp 533-541), 2015. Date of Publication: 01 Apr 2015.

AN: 2015836674

Introduction: The frequent use of inhaled corticosteroids (ICSs), especially at higher doses, has been accompanied by concern about both systemic and local side effects. Patients suffering from chronic obstructive pulmonary disease (COPD) are more at risk from side effects, likely because of the use of higher doses of ICS in COPD to overcome corticosteroid unresponsiveness.Areas covered: There is considerable concern about increased incidence of pneumonia, osteoporosis and hyperglycemia in diabetic patients and cataracts. The local side effects of ICSs, such as hoarseness and pharyngeal discomfort, oral and oropharyngeal candidiasis, cough during inhalation, and a sensation of thirst, are not usually serious but are of clinical importance because they may lead to patients discontinuing therapy.Expert opinion: The possibility that ICSs induce adverse side effects should not lead us to avoid their use in patients in whom clinical evidence suggests that they may be helpful. However, clinicians should balance the potential benefits of ICSs in COPD against their potential side effects and always consider using the lowest possible dose to achieve the best possible management.

Institution

(Matera) Second University of Naples, Department of Experimental Medicine, Via del Parco Margherita 24, Naples 80121, Italy (Cardaci) San Raffaele Pisana Hospital, Department of Pulmonary Rehabilitation, IRCCS, Rome, Italy

(Cazzola, Rogliani) University of Rome Tor Vergata, Department of Systems Medicine, Via Montpellier 1, Rome 00133, Italy

Publisher

Informa Healthcare

Emtree Heading

cataract/si [Side Effect]; *chronic lung disease; chronic obstructive lung disease/dt [Drug Therapy]; *corticosteroid therapy; coughing/si [Side Effect]; diabetes mellitus/si [Side Effect]; drug safety; drug withdrawal; dysphonia/si [Side Effect]; fracture/si [Side Effect]; glaucoma/si [Side Effect]; hoarseness/si [Side Effect]; human; hyperglycemia/si [Side Effect]; larynx disorder/si [Side Effect]; lung tuberculosis/si [Side Effect]; oropharynx candidiasis/si [Side Effect]; osteoporosis/si [Side Effect]; perioral dermatitis/si [Side Effect]; pharynx disease/si [Side Effect]; pneumonia/si [Side Effect]; review; risk assessment; skin manifestation/si [Side Effect]; thrush/si [Side Effect]; treatment outcome; *corticosteroid/ae [Adverse Drug Reaction]; *corticosteroid/dt [Drug Therapy]; *corticosteroid/ih [Inhalational Drug Administration].

Link to the Ovid Full Text or citation:

Click here for full text options

70.

Pulmonary tuberculosis in outpatients in Sabah, Malaysia: Advanced disease but low incidence of HIV co-infection.

William T., Parameswaran U., Lee W.K., Yeo T.W., Anstey N.M., Ralph A.P.

BMC Infectious Diseases. 15 (1) (no pagination), 2015. Article Number: 758. Date of Publication: January 31, 2015.

AN: 2015799705

Background: Tuberculosis (TB) is generally well controlled in Malaysia, but remains an important problem in the nation's eastern states. In order to better understand factors contributing to high TB rates in the eastern state of Sabah, our aims were to describe characteristics of patients with TB at a large outpatient clinic, and determine the prevalence of HIV co-infection. Additionally, we sought to test sensitivity and specificity of the locally-available point-of-care HIV test kits. Methods: We enrolled consenting adults with smear-positive pulmonary TB for a 2-year period at Luyang Clinic, Kota Kinabalu, Malaysia. Participants were questioned about ethnicity, smoking, prior TB, disease duration, symptoms and comorbidities. Chest radiographs were scored using a previously devised tool. HIV was tested after counselling using 2 point-of-care tests for each patient: the test routinely in use at the TB clinic (either Advanced QualityTM Rapid Anti-HIV 1&2, FACTS anti-HIV 1/2 RAPID or HIV (1 + 2) Antibody Colloidal Gold), and a comparator test (Abbott DetermineTM HIV-1/2, Inverness Medical). Positive tests were confirmed by enzyme immunoassay (EIA), particle agglutination and line immunoassay. Results: 176 participants were enrolled; 59 (33.5%) were non-Malaysians and 104 (59.1%) were male. Smoking rates were high (81/104 males, 77.9%), most had cavitary disease (51/145, 64.8%), and 81/176 (46.0%) had haemoptysis. The median period of symptoms prior to treatment onset was 8 weeks. Diabetes was present in 12. People with diabetes or other comorbidities had less severe TB, suggesting different healthcare seeking behaviours in this group. All participants consented to HIV testing: three (1.7%) were positive according to DetermineTM and EIA, but one of these tested negative on the point-of-care test available at the clinic (Advanced QualityTM Rapid Anti-HIV 1&2). The low number of positive tests and changes in locally-available test type meant that accurate estimates of sensitivity and specificity were not possible. Conclusion: Patients had advanced disease at diagnosis, long diagnostic delays, low HIV co-infection rates, high smoking rates among males, and migrants may be over-represented. These findings provide important insights to guide local TB control efforts. Caution is required in using some point-of-care HIV tests, and ongoing quality control measures are of major importance.

Institution

(William, Parameswaran) Infectious Diseases Unit, Queen Elizabeth Hospital, Kota Kinabalu, Sabah, Malaysia (William, Parameswaran, Yeo, Anstey, Ralph) Infectious Diseases Society Sabah-Menzies School of Health, Research Clinical Research Unit, Kota Kinabalu, Sabah, Malaysia

(William) Sabah Department of Health, Kota Kinabalu, Sabah, Malaysia

(Lee) Luyang Outpatient Clinic, Kota Kinabalu, Sabah, Malaysia

(Yeo, Anstey, Ralph) Global and Tropical Health Division, Menzies School of Health Research, Darwin, NT, Australia

(Anstey, Ralph) Department of Medicine, Royal Darwin Hospital, Darwin, NT, Australia

Publisher

BioMed Central Ltd.

Emtree Heading

adolescent; aged; agglutination test; article; attitude to illness; clinical feature; comorbidity; controlled study; diabetes mellitus; diagnostic accuracy; diagnostic test accuracy study; disease association; disease duration; disease severity; enzyme immunoassay; female; help seeking behavior; hemoptysis; HIV rapid test; human; Human immunodeficiency virus; *Human immunodeficiency virus infection/di [Diagnosis]; incidence; *lung tuberculosis; major clinical study; Malaysia; male; *mixed infection/di [Diagnosis]; outcome assessment; outpatient; patient counseling; point of care testing; risk factor; scoring system; sensitivity and specificity; smoking; sputum smear; thorax radiography.

Link to the Ovid Full Text or citation:

Click here for full text options

71.

Acquired immunodeficiencies and tuberculosis: Focus on HIV/AIDS and diabetes mellitus.

Ronacher K., Joosten S.A., van Crevel R., Dockrell H.M., Walzl G., Ottenhoff T.H.M.

Immunological Reviews. 264 (1) (pp 121-137), 2015. Date of Publication: 01 Mar 2015.

AN: 2015767401

The spread of human immunodeficiency virus (HIV) infection within Africa led to marked increases in numbers of cases of tuberculosis (TB), and although the epidemic peaked in 2006, there were still 1.8 million new cases in 2013, with 29.2 million prevalent cases. Half of all TB cases in Africa are in those with HIV co-infection. A brief review of the well-documented main immunological mechanisms of HIV-associated increased susceptibility to TB is presented. However, a new threat is facing TB control, which presents itself in the form of a rapid increase in the number of people living with type II diabetes mellitus (T2DM), particularly in areas that are already hardest hit by the TB epidemic. T2DM increases susceptibility to TB threefold, and the TB burden attributable to T2DM is 15%. This review addresses the much smaller body of research information available on T2DM-TB, compared to HIV-TB comorbidity. We discuss the altered clinical presentation of TB in the context of T2DM comorbidity, changes in innate and adaptive immune responses, including lymphocyte subsets and T-cell phenotypes, the effect of treatment of the different comorbidities, changes in biomarker expression and genetic predisposition to the respective morbidities, and other factors affecting the comorbidity. Although significant gains have been made in improving our understanding of the underlying mechanisms of T2DM-associated increased susceptibility, knowledge gaps still exist that require urgent attention.

Institution

(Ronacher, Walzl) DST/NRF Centre of Excellence for Biomedical Tuberculosis Research, MRC Centre for Tuberculosis Research, Division of Molecular Biology and Human Genetics, Faculty of Medicine and Health Sciences, Stellenbosch University, Cape Town, South Africa (Joosten, Ottenhoff) Department of Infectious Diseases, Leiden University Medical Centre, Leiden, Netherlands

(van Crevel) Department of Medicine, Radboud University Medical Center, Nijmegen, Netherlands

(Dockrell) Immunology and Infection Department, Faculty of Infectious and Tropical Diseases, London School of Hygiene and Tropical Medicine, London, United Kingdom

Publisher

Blackwell Publishing Ltd

Emtree Heading

*acquired immune deficiency syndrome; adaptive immunity; article; B lymphocyte; bone marrow cell; CD4+ T lymphocyte; comorbidity; cytokine production; diabetic patient; genetic risk; human; *Human immunodeficiency virus infection/dt [Drug Therapy]; immune reconstitution inflammatory syndrome/si [Side Effect]; innate immunity; lymphocyte subpopulation; mixed infection; natural killer cell; *non insulin dependent diabetes mellitus/dt [Drug Therapy]; nonhuman; priority journal; protein expression; T lymphocyte; Th17 cell; Th22 cell; treatment outcome; *tuberculosis/dt [Drug Therapy]; tuberculosis control; advanced glycation end product; anti human immunodeficiency virus agent/dt [Drug Therapy]; antiretrovirus agent/ae [Adverse Drug Reaction]; biological marker; complement receptor; gamma interferon; glibenclamide/dt [Drug Therapy]; glutathione; hemoglobin A1c; interleukin 17; interleukin 18; metformin/dt [Drug Therapy]; nonnucleoside reverse transcriptase inhibitor/dt [Drug Therapy]; proteinase inhibitor/dt [Drug Therapy]; tuberculostatic agent/dt [Drug Therapy]; tumor necrosis factor alpha; vitamin D.

Link to the Ovid Full Text or citation:

Click here for full text options

72.

Battling tuberculosis in an island context with a high burden of communicable and non-communicable diseases: Epidemiology, progress, and lessons learned in Kiribati, 2000 to 2012.

Hoy D., Kienene T., Reiher B., Roth A., Tira T., McKenzie J., Merilles O.E.A., Viney K.

International Journal of Infectious Diseases. 30 (pp e135-e141), 2015. Date of Publication: January 01, 2015.

AN: 2014630166

Objectives: To examine the epidemiology of tuberculosis (TB) in Kiribati from 2000 to 2012, document lessons learned, and recommend ways to mitigate the burden of TB in Kiribati. Methods: A descriptive study was performed using data on TB case notifications, prevalence, incidence, mortality, and treatment outcomes from global reports and data files. Progress towards meeting the Millennium Development Goal TB target (to reduce TB incidence by 2015) and the Regional Strategy to Stop Tuberculosis in the Western Pacific 2011-2015 targets (to reduce TB prevalence and mortality by half by 2015 relative to the level in 2000) was examined. Results: TB case notifications and the estimated incidence and prevalence have increased in Kiribati since 2000. From 2000 to 2012, Kiribati reported a total of 3863 TB notifications; in 2012, the case notification rate was 343/100000 population. The majority (89%) of TB patients complete treatment and/or are cured, and the estimated TB mortality rate has remained relatively stable at around 16/100000 population. HIV testing of TB patients has increased over recent years from 8% of notifications tested in 2003 to 43% tested in 2012. Of all 818 tests, only four (0.5%) patients were confirmed HIV-positive. Drug-resistant TB has been detected in a small number of cases. Conclusions: TB rates continue to increase in Kiribati and the 2015 goals for TB control are unlikely to be met. This is probably due to the complex mix of risk factors present in Kiribati, including smoking, diabetes, alcohol use, crowded living, and poverty. A comprehensive approach to address these risk factors is needed to mitigate the burden of TB in Kiribati.

Institution

(Hoy, Roth, McKenzie, Merilles) Secretariat of the Pacific Community, BP D5, Noumea 98848, New Caledonia (Kienene, Reiher, Tira) Kiribati Ministry of Health and Medical Services, Kiribati

(Viney) National Centre of Epidemiology and Population Health, Australian National University, Canberra, Australia

Publisher

Elsevier

Emtree Heading

article; female; HIV test; human; incidence; infection rate; Kiribati; *lung tuberculosis/dr [Drug Resistance]; *lung tuberculosis/ep [Epidemiology]; major clinical study; male; mortality; prevalence; risk factor; treatment outcome.

Link to the Ovid Full Text or citation:

Click here for full text options

73.

The incidence and risk factor of acute serum creatinine elevation more than 1.5 mg/dL among the patients treated with tenofovir/emtricitabine-containing HAART regimens.

Chang H-H., Jin S., Kim S.-W.

International Journal of Antimicrobial Agents. Conference: 10th International Symposium on Antimicrobial Agents and Resistance Incheon South Korea. Conference Start: 20150514 Conference End: 20150515. Conference Publication: (var.pagings). 45 (pp S121-S122), 2015. Date of Publication: May 2015.

AN: 71915997

Background: The combination of tenofovir disproxil fumarate and emtricitabine (TDF/FTC) has been the 1st choice NRTI according to many reliable antiretroviral treatment guidelines because of its high efficacy. However, TDF-related renal toxicity is a challenging issue regarding its use in the clinical practice by many overseas reports. We conducted this study to evaluate the incidence and risk factor of acute serum creatinine elevation more than 1.5 mg/ dL among the TDF/FTC-based HAART treated patients. Methods: We undertook the retrospective medical records evaluation of 205 HIV-infected patients treated with TDF/FTCcontaining regimens in Department of Allergy and Infectious Disease of Kyungpook National University Hospital between 1 February 2010 and 30 April 2014. Among 205 patients, 72 patients were excluded because of too short treatment duration (<6 days), lack of blood chemistry or other blood laboratory data, and impossibility of review of patient's medical records. A total 136 patient's data [91 cases in TDF/FTC+protease inhibitors(PI) treated group (group 1), 20 cases in TDF/FTC+non-nucleoside reverse transcriptase inhibitors(NNRTI) treated group (group 2) and 25 cases in TDF/FTC+integrase inhibitors(INSTI) treated group (group 3)] was included in statistical analysis. Results: The patients' age, gender distribution, comorbidity such as syphilis, hepatitis C, hepatitis B, CMV infection, tuberculosis, and underlying diseases like hypertension, diabetes mellitus, chronic renal diseases, and thyroid diseases were not different between three groups. The incidences of co-administration of analgesics or anti-psychotic medication were not different either. The initial CD4 count at the initiation of treatment in each group was not different statistically (279+/-202/mm3 in group 1 vs. 347+/-300/mm3 in group 2 vs. 345+/-203/mm3 in group 3, p=0.270), but initial HIV RNA level (log10 copies/mL) was significantly higher in group 1 and 2 (3.398+/- 2.109 in group 1 vs. 3.160+/- 2.432 in group 2 vs. 1.919+/- 2.213 in group 3, p=0.022). The only 4 cases (4.9% in PI-treated patients group) showed the acute serum creatinine elevation more than 1.5 mg/dL, and all the case belonged to group 1. One case was the patient treated with LPV/r+ TDF/FTC, and the others with ATV/r+ TDF/ETC. In DRV/r+ TDF/ETC treated patients group. There was no case of acute serum Cr elevation (4.3% in LPV/r group vs. 16.7% in ATV/r group vs. 0% in DRV/r group, p=0.013). In group 2 and 3, there was no case showing serum Cr elevation (4.9% in group 1 vs. 0% in group 2 and 3, p=0.403). Medication change rate and overall treatment failure rate were not different between groups. Increment of CD4 count after HAART and the increment of serum creatinine level were not different between groups either. Conclusion: This study has limitations such as small number of patients included and only single center experience, but TDF/ FTC+ATV/r regimen showed relatively higher rate of serum Cr elevation. This finding reinforces the importance of close followup of HIV-infected patients who have treated with TDF/FTC+ ATV/r regimen.

Institution

(Chang, Jin, Kim) Division of Allergy and Infectious Diseases, Department of Internal Medicine, Kyungpook National University Hospital, Daegu, South Korea

Publisher

Elsevier

Emtree Heading

*nephrotoxicity; *risk factor; *creatinine blood level; *patient; *human; *highly active antiretroviral therapy; *Human immunodeficiency virus; serum; medical record; Human immunodeficiency virus infected patient; drug therapy; university hospital; hepatitis C; allergy; infection; hepatitis B; treatment duration; syphilis; comorbidity; follow up; statistical analysis; clinical practice; treatment failure; laboratory; hypertension; blood; diseases; tuberculosis; cytomegalovirus infection; diabetes mellitus; chronic kidney disease; thyroid disease; psychosis; blood chemistry; gender; patient coding; *anti human immunodeficiency virus agent; *tenofovir; *antiinfective agent; CD4 antigen; fumaric acid; emtricitabine; integrase inhibitor; nonnucleoside reverse transcriptase inhibitor; proteinase inhibitor; analgesic agent; RNA; RNA directed DNA polymerase inhibitor.

Link to the Ovid Full Text or citation:

Click here for full text options

74.

Tuberculosis screening for diabetic patients in community clinics in Kunming, China.

Stone A., Ling L., Innes A., Lin X.

Journal of General Internal Medicine. Conference: 38th Annual Meeting of the Society of General Internal Medicine Toronto, ON Canada. Conference Start: 20150422 Conference End: 20150425. Conference Publication: (var.pagings). 30 (pp S289), 2015. Date of Publication: April 2015.

AN: 71877979

BACKGROUND: China has a rising epidemic of diabetes mellitus (DM) with a prevalence of 11.6 %, representing up to 113.9 million people. China also has an estimated 12 million cases of tuberculosis (TB). Diabetic patients have up to a 3-fold increased risk of developing active TB in addition to higher risk of TB treatment failure and even death. The present intervention was developed through the USAID Control and Prevention of TB (CAP-TB) project from FHI 360 in conjunction with partners the International Union Against Lung Disease and Tuberculosis and the Yunnan Anti-TB Association as a method to increase detection of TB in high-risk groups. This study aims to determine whether a simple screening tool for TB can be implemented by primary care providers in community clinics in Kunming, China to increase TB case detection in diabetic patients. Primary Objective: To build health workforce capacity of primary care providers in the region to improve diagnosis and treatment of TB among high-risk groups. Secondary Objective: To determine whether TB screening in diabetic patients by primary care providers should be recommended in China. METHODS: Primary care physicians and nurses at 5 designated district health centers and 5 sub-centers in the Xishan District of Kunming, Yunnan Province, China were trained to screen diabetic patients for TB. A symptom questionnaire was used at each clinic visit for each diabetic patient: cough for >2 weeks, night sweats for >4 weeks, fever for >4 weeks, weight loss over the previous 4 weeks, and symptoms of extra-pulmonary TB. A "yes" answer to any of these questions generated a referral for evaluation in the district TB clinic. Data were collected monthly on number of diabetic patients screened, number of positive screens, patients referred to and seen at the district TB clinic, and the number of patients diagnosed with TB. From this data, the case notification rate was determined. In addition to quantitative data collection, quarterly site visits were conducted to elicit initial success with enacting the screening protocol as well as to discuss barriers to screening with the trained providers. RESULTS: From June 2013 to April 2014, 2942 diabetic patients were screened for TB in the designated clinics. Of these, 278 patients had TB symptoms and 209 patients arrived at TB clinic for diagnostic testing resulting in 3 confirmed cases of TB. Six patients had positive symptom screens but had chest x-rays that were read as inactive or old TB. The case notification rate was 102 per 100,000 screened as compared to a baseline notification rate in the general population of Yunnan province of 55 per 100,000. CONCLUSIONS: Providers at health centers in Kunming, China are able to implement a simple protocol integrating TB screening into diabetes care. However, while many diabetic patients were screened, few screened positive resulting in a relatively low case notification rate. Success of implementation (measured by number of patients screened) also varied between sites. Poor provider understanding of reasons for screening, including risk factors for and symptoms of TB, was a barrier to successful detection. Other barriers included poor integration of screening into provider workflow and patient resistance to referral for diagnosis. Ongoing provider and patient education is needed on early case detection of TB and the importance of active screening for timely diagnosis and treatment of TB among diabetic patients. Screening for TB in diabetic patients in areas of high prevalence of both diseases is likely important to decrease the incidence of TB. However, even among those referred for TB diagnosis, relatively few had active TB. More information is needed to determine the most cost-effective and efficient means to screen these high-risk patients for TB. In the future, a new intervention aimed at focusing on patients at the hospital level in diabetic outpatient clinics (as opposed to community primary care settings) in Kunming will be piloted. Other interventions may also prove to be more effective, such as targeting a certain hemoglobin A1C to stratify for those with poorly controlled diabetes. Data on screening thus far has not been sufficient to recommend wider implementation, but it is necessary to continue to pursue new strategies as diabetes becomes a greater issue in areas of high TB prevalence.

Institution

(Ling, Innes) CAP-TB, USAID Regional Development Mission Asia, Bangkok, Thailand (Lin) Yunnan Center for Disease Control and Prevention, Kunming, China

(Innes) Family Health International, Bangkok, Thailand

(Ling) Family Health International, Kunming, China

(Stone) University of California, San Francisco, CA, United States

Publisher

Springer New York LLC

Emtree Heading

*screening; *diabetic patient; *human; *community; *hospital; *China; *society; *internal medicine; *tuberculosis; patient; diagnosis; diabetes mellitus; primary medical care; prevalence; high risk population; risk; health center; general practitioner; outpatient department; high risk patient; health; diseases; patient education; risk factor; population; prevention; information processing; treatment failure; weight reduction; death; positive syndrome; lung disease; epidemic; fever; workflow; night sweat; coughing; questionnaire; nurse; thorax radiography; hemoglobin.

Link to the Ovid Full Text or citation:

Click here for full text options

75.

Temporal trends in critical events complicating HIV infection: 1999-2010 multicentre cohort study in France.

Barbier F., Roux A., Canet E., Martel-Samb P., Aegerter P., Wolff M., Guidet B., Azoulay E.

Intensive Care Medicine. 40 (12) (pp 1906-1915), 2014. Date of Publication: 21 Nov 2014.

AN: 2014877889

Purpose: Multicentre data are limited to appraise the management and prognosis of critically ill human immunodeficiency virus (HIV)-infected patients. We sought to describe temporal trends in demographic and clinical characteristics, indications for intensive care and outcome in this patient population. Methods: We conducted a cohort study of unselected HIV-infected patients admitted between 1999 and 2010 to 34 French ICUs contributing to the CUB-Rea prospective database.

Results: We included 6,373 consecutive patients. Over the 12-year period, increases occurred in median age (39 years in 1999-2001; 47 years in 2008-2010, p < 0.0001) and prevalence of comorbidities (notably malignancies, from 6.7 to 16.4 %, p < 0.0001). Admissions for respiratory failure (39.8 % overall), shock (8.1 %) and coma (22.7 %) decreased (p < 0.0001), while those for sepsis (19.3 %) remained stable. The main final diagnoses were bacterial sepsis (24.6 %) and non-bacterial acquired immune deficiency syndrome (AIDS)-defining diseases (steady decline from 26.0 to 17.5 %, p < 0.0001). Patients increasingly received mechanical ventilation (from 42.9 to 54.0 %) and renal replacement therapy (from 9.6 to 16.8 %) (p < 0.0001), whereas vasopressor use remained stable (27.4 %). ICU readmissions increased after 2004 (p < 0.0001). ICU and hospital mortality (17.6 and 26.9 %, respectively) dropped markedly in the most severely ill patients requiring multiple life-sustaining therapies. Malignancies and chronic liver disease were heavily associated with hospital mortality by multivariate analysis, while the most common AIDS-defining complications (Pneumocystis jirovecii pneumonia, cerebral toxoplasmosis and tuberculosis) had no independent impact.

Conclusions: Progressive ageing, increasing prevalence of comorbidities (mainly malignancies), a steady decline in AIDS-related illnesses and improved benefits from life-sustaining therapies were the main temporal trends in HIV-infected patients requiring ICU admission.

Institution

(Barbier) Medical Intensive Care Unit (ICU), La Source Hospital, Orleans, France (Roux) Respiratory ICU, Georges Pompidou European Hospital, Paris, France

(Canet, Azoulay) Medical ICU, Saint-Louis Hospital, Paris, France

(Martel-Samb, Aegerter) Department of Public Health and Clinical Research, Ambroise Pare Hospital, Boulogne-Billancourt, France

(Wolff) Medical and Infectious Diseases ICU, Bichat-Claude Bernard Hospital, Paris, France

(Guidet) Medical ICU, Saint-Antoine Hospital, Paris, France

Publisher

Springer Verlag

Emtree Heading

*acquired immune deficiency syndrome/ep [Epidemiology]; acute respiratory failure/ep [Epidemiology]; adult; AIDS related complex/co [Complication]; AIDS related complex/ep [Epidemiology]; article; cardiovascular disease/ep [Epidemiology]; chronic kidney disease/ep [Epidemiology]; cohort analysis; colorectal cancer/ep [Epidemiology]; combination antiretroviral therapy; *comorbidity; *critically ill patient; diabetes mellitus/ep [Epidemiology]; female; France; hepatitis B/ep [Epidemiology]; hepatitis C/ep [Epidemiology]; highly active antiretroviral therapy; Hodgkin disease/ep [Epidemiology]; hospital admission; human; Human immunodeficiency virus infected patient; *Human immunodeficiency virus infection/ep [Epidemiology]; *Human immunodeficiency virus infection/dt [Drug Therapy]; intensive care unit; Kaposi sarcoma/ep [Epidemiology]; liver cancer/ep [Epidemiology]; liver cirrhosis/ep [Epidemiology]; lung cancer/ep [Epidemiology]; major clinical study; male; mixed infection/ep [Epidemiology]; mortality; multicenter study; nonhodgkin lymphoma/ep [Epidemiology]; outcome assessment; Pneumocystis pneumonia/co [Complication]; Pneumocystis pneumonia/ep [Epidemiology]; prevalence; respiratory tract disease/ep [Epidemiology]; toxoplasmosis/co [Complication]; toxoplasmosis/ep [Epidemiology]; tuberculosis/co [Complication]; tuberculosis/ep [Epidemiology]; antiretrovirus agent/dt [Drug Therapy].

Link to the Ovid Full Text or citation:

Click here for full text options

76.

Medical Devices and Diagnostics for Cardiovascular Diseases in Low-Resource Settings.

McGuire H., Weigl B.H.

Journal of Cardiovascular Translational Research. 7 (8) (pp 737-748), 2014. Date of Publication: 15 Nov 2014.

AN: 2014842968

Noncommunicable diseases (NCDs), including cardiovascular diseases and diabetes, have emerged as an underappreciated health threat with enormous economic and public health implications for populations in low-resource settings. In order to address these diseases, devices that are to be used in low-resource settings have to conform to requirements that are generally more challenging than those developed for traditional markets. Characteristics and issues that must be considered when working in low- and middle-income countries (LMICs) include challenging environmental conditions, a complex supply chain, sometimes inadequate operator training, and cost. Somewhat counterintuitively, devices for low-resource setting (LRS) markets need to be of at least as high quality and reliability as those for developed countries to be setting-appropriate and achieve impact. Finally, the devices need to be designed and tested for the populations in which they are to be used in order to achieve the performance that is needed. In this review, we focus on technologies for primary and secondary health-care settings and group them according to the continuum of care from prevention to treatment.

Institution

(McGuire) PATH, Washington, DC, United States (Weigl) PATH, Seattle, WA, United States

Publisher

Springer New York LLC

Emtree Heading

analytical equipment; article; behavior change; blood glucose meter; blood pressure monitor; blood pressure monitoring; calibration; *cardiovascular disease/di [Diagnosis]; *cardiovascular disease/pc [Prevention]; chronic respiratory tract disease; computer program; diabetes mellitus/di [Diagnosis]; diabetes mellitus/pc [Prevention]; *diagnostic test; dyslipidemia/di [Diagnosis]; environmental factor; follow up; health care access; health care cost; health care distribution; health care financing; health care quality; highly active antiretroviral therapy; human; Human immunodeficiency virus infection/dt [Drug Therapy]; hypertension/di [Diagnosis]; hypertension/dt [Drug Therapy]; low and middle income country; *low resource setting; malignant neoplastic disease; *medical device; medical education; medical information system; medical technology; non communicable disease/di [Diagnosis]; non communicable disease/dm [Disease Management]; non communicable disease/pc [Prevention]; outcome assessment; patient care; patient monitoring; point of care testing; preventive medicine; primary health care; risk assessment; risk factor; screening test; secondary health care; *socioeconomics; tuberculosis; antiretrovirus agent/dt [Drug Therapy]; beta adrenergic receptor blocking agent/dt [Drug Therapy]; calcium channel blocking agent/dt [Drug Therapy]; dipeptidyl carboxypeptidase inhibitor/dt [Drug Therapy]; diuretic agent/dt [Drug Therapy]; vasodilator agent/dt [Drug Therapy].

Link to the Ovid Full Text or citation:

Click here for full text options

77.

The growing challenge of nontuberculous mycobacteria.

Schluger N.W.

Chest. 146 (3) (pp 529-530), 2014. Date of Publication: 01 Sep 2014.

AN: 2014805480

Institution

(Schluger) Department of Medicine, Columbia University, Mailman School of Public Health, 622 W 168th St, New York, NY 10032, United States

Publisher

American College of Chest Physicians

Emtree Heading

asthma/dt [Drug Therapy]; *atypical mycobacteriosis/ep [Epidemiology]; *atypical mycobacteriosis/dt [Drug Therapy]; atypical Mycobacterium; bronchiectasis; chronic kidney disease; chronic obstructive lung disease/dt [Drug Therapy]; coughing; diabetes mellitus; disease association; editorial; end stage renal disease; human; Human immunodeficiency virus infection; immunosuppressive treatment; incidence; infection risk; infection sensitivity; mortality; Mycobacterium avium; nonhuman; rheumatoid arthritis; risk assessment; treatment outcome; tuberculosis; ethambutol/dt [Drug Therapy]; macrolide/dt [Drug Therapy]; rifabutin/dt [Drug Therapy]; rifampicin/dt [Drug Therapy]; rifamycin/dt [Drug Therapy]; steroid/dt [Drug Therapy].

Link to the Ovid Full Text or citation:

Click here for full text options

78.

NCDs and an outcome-based approach to global health.

Bollyky T.J., Emanuel E.J., Goosby E.P., Satcher D., Shalala D.E., Thompson T.G.

The Lancet. 384 (9959) (pp 2003-2004), 2014. Date of Publication: 06 Dec 2014.

AN: 2014621616

Institution

(Bollyky) Council on Foreign Relations, Washington, DC 20006, United States (Emanuel) Department of Medical Ethics and Health Policy, Perelman School of Medicine, University of Pennsylvania, Philadelphia, PA, United States

(Goosby) Institute for Global Health Delivery and Diplomacy, School of Medicine, University of California, San Francisco, San Francisco, CA, United States

(Satcher) Satcher Health Leadership Institute, Center of Excellence on Health Disparities, Morehouse School of Medicine, Atlanta, GA, United States

(Shalala) Office of President, University of Miami, Coral Gables, FL, United States

(Thompson) Thompson Family Holdings, Washington, DC, United States

Publisher

Lancet Publishing Group

Emtree Heading

acquired immune deficiency syndrome; budget; cardiovascular disease; cause of death; child health; communicable disease; developing country; diabetes mellitus; economic aspect; epidemic; family planning; government regulation; health care cost; *health status; household; human; Human immunodeficiency virus; income; lowest income group; malaria; maternal welfare; medical care; mental health; neoplasm; *non communicable disease; note; outcome assessment; physical disability; physical inactivity; population risk; prematurity; primary medical care; public health; quality adjusted life year; risk assessment; risk factor; tobacco; tuberculosis; world health organization; antihypertensive agent.

Link to the Ovid Full Text or citation:

Click here for full text options

79.

A nationwide population-based study of the risk of tuberculosis in different solid organ transplantations in Taiwan.

Chen C.-H., Shu K.-H., Ho H.-C., Cheng S.-B., Lin C.-C., Wei H.-J., Lin C.-H., Chang S.-N., Wu M.-J.

Transplantation Proceedings. 46 (4) (pp 1032-1035), 2014. Date of Publication: May 2014.

AN: 2014325628

Advances in immunosuppressants for solid organ transplantation (SOT) have improved prevention and treatment of acute rejection as well as reduced the risk of chronic graft damage. However, SOT recipients are prone to developing opportunistic infections because of their long-term immunosuppressed status. Tuberculosis (TB) is a serious opportunistic infection that is associated with increased morbidity and mortality in SOT recipients. However, nationwide population-based research specifically focused on the associations between kidney transplantation (KTx), liver transplantation (LTx), and heart transplantation (HTx), and subsequent TB infection is lacking. This study was conducted using Taiwan's National Health Insurance Research Database, which provided claims data for SOT recipients from 2000 to 2009. Clinical features, treatment, and outcomes were analyzed to determine the risk for TB after SOT. In total, 153 (3.2%) RTx, 19 (1.1%) LTx, and 26 (2.8%) HTx recipients became infected with TB. Compared with non-TB patients, HTx recipients with TB had significantly higher prevalence of older age (P =.037), hypertension (P <.001), and coronary artery disease (CAD) (P =.002). There were also greater percentages of male sex (P =.018), diabetes (P =.029), hyperlipidemia (P =.016), CAD (P <.001), and chronic obstructive pulmonary disease (COPD) (P <.001) in RTx recipients with TB than in those without. In conclusion, posttransplantation TB is a serious problem worldwide, and a high index of suspicion is warranted to ensure early diagnosis and prompt initiation of treatment for TB among SOT patients. In this preliminary study, KTx recipients had a higher risk of TB infection than LTx and HTx recipients, and the high-risk factors were male sex, diabetes, hyperlipidemia, CAD, and COPD. The use of optimal immunosuppressive agents to minimize acute rejection, monitoring of high-risk recipients, prompt diagnosis, and appropriate treatment are required for the management of TB infection in endemic areas such as Taiwan. © 2014 by Elsevier Inc. All rights reserved.

Institution

(Chen, Shu, Wu) Division of Nephrology, Department of Medicine, Taichung Veterans General Hospital, 1650 Taiwan Boulevard, Taichung 40705, Taiwan (Republic of China) (Ho) Division of Urology, Taichung Veterans General Hospital, Taichung, Taiwan (Republic of China)

(Cheng, Wei) Liver Transplantation Center, Department of Surgery, Taichung Veterans General Hospital, Taichung, Taiwan (Republic of China)

(Lin) Department of Pediatrics, Taichung Veterans General Hospital, Taichung, Taiwan (Republic of China)

(Lin, Chang) Department of Medical Research, Taichung Veterans General Hospital, Taichung, Taiwan (Republic of China)

(Wu) School of Medicine, Chung Shan Medical University, Taichung, Taiwan (Republic of China)

(Chen, Shu, Wu) School of Medicine, China Medical University, Taichung, Taiwan (Republic of China)

(Wu) Institute of Biomedical Science, National Chung Hsing University, Taichung, Taiwan (Republic of China)

(Chen) Department of Life Science, Tunghai University, Taichung, Taiwan (Republic of China)

Publisher

Elsevier USA

Emtree Heading

acute graft rejection; adult; chronic obstructive lung disease; clinical feature; conference paper; coronary artery disease; diabetes mellitus; early diagnosis; female; graft recipient; high risk patient; human; hyperlipidemia; hypertension; major clinical study; male; *organ transplantation; outcome assessment; population research; priority journal; Taiwan; *tuberculosis; azathioprine; cyclosporin; prednisolone; rifampicin; tacrolimus.

Link to the Ovid Full Text or citation:

Click here for full text options

80.

Diabetes mellitus, smoking status, and rate of sputum culture conversion in patients with multidrug-resistant tuberculosis: a cohort study from the country of Georgia.

Magee M.J., Kempker R.R., Kipiani M., Tukvadze N., Howards P.P., Narayan K.M., Blumberg H.M.

PloS one. 9 (4) (pp e94890), 2014. Date of Publication: 2014.

AN: 24736471

INTRODUCTION: Diabetes mellitus (DM) is a risk factor for active tuberculosis (TB) but little is known about the effect of DM on culture conversion among patients with multidrug-resistant (MDR)-TB. The primary aim was to estimate the association between DM and rate of TB sputum culture conversion. A secondary objective was to estimate the association between DM and the risk of poor treatment outcomes among patients with MDR-TB. MATERIALS AND METHODS: A cohort of all adult patients starting MDR-TB treatment in the country of Georgia between 2009-2011 was followed during second-line TB therapy. Cox proportional models were used to estimate the adjusted hazard rate of sputum culture conversion. Log-binomial regression models were used to estimate the cumulative risk of poor TB treatment outcome.

RESULTS: Among 1,366 patients with sputum culture conversion information, 966 (70.7%) had culture conversion and the median time to conversion was 68 days (interquartile range 50-120). The rate of conversion was similar among patients with MDR-TB and DM (adjusted hazard ratio [aHR] 0.95, 95%CI 0.71-1.28) compared to patients with MDR-TB only. The rate of culture conversion was significantly less in patients that currently smoked (aHR 0.82, 95%CI 0.71-0.95), had low body mass index (aHR 0.71, 95%CI 0.59-0.84), second-line resistance (aHR 0.56, 95%CI 0.43-0.73), lung cavities (aHR 0.70, 95%CI 0.59-0.83) and with disseminated TB (aHR 0.75, 95%CI 0.62-0.90). The cumulative risk of poor treatment outcome was also similar among TB patients with and without DM (adjusted risk ratio [aRR] 1.03, 95%CI 0.93-1.14).

CONCLUSIONS: In adjusted analyses, DM did not impact culture conversion rates in a clinically meaningful way but smoking did.

Institution

(Magee) Department of Epidemiology, Rollins School of Public Health, Emory University, Atlanta, Georgia, United States of America; Hubert Department of Global Health, Rollins School of Public Health, Emory University, Atlanta, Georgia, United States of America (Kempker) Division of Infectious Diseases, Department of Medicine, Emory University School of Medicine, Atlanta, Georgia, United States of America

(Kipiani) National Center for Tuberculosis and Lung Diseases, Tbilisi, Georgia

(Tukvadze) National Center for Tuberculosis and Lung Diseases, Tbilisi, Georgia

(Howards) Department of Epidemiology, Rollins School of Public Health, Emory University, Atlanta, Georgia, United States of America

(Narayan) Department of Epidemiology, Rollins School of Public Health, Emory University, Atlanta, Georgia, United States of America; Hubert Department of Global Health, Rollins School of Public Health, Emory University, Atlanta, Georgia, United States of America

(Blumberg) Department of Epidemiology, Rollins School of Public Health, Emory University, Atlanta, Georgia, United States of America; Hubert Department of Global Health, Rollins School of Public Health, Emory University, Atlanta, Georgia, United States of America; Division of Infectious Diseases, Department of Medicine, Emory University School of Medicine, Atlanta, Georgia, United States of America

Emtree Heading

adolescent; adult; aged; cohort analysis; *diabetes mellitus; epidemiology; female; Georgia; human; male; *microbiology; middle aged; *smoking; sputum; treatment outcome; Tuberculosis, Multidrug-Resistant/dt [Drug Therapy]; Tuberculosis, Multidrug-Resistant/ep [Epidemiology]; Tuberculosis, Pulmonary/dt [Drug Therapy]; Tuberculosis, Pulmonary/ep [Epidemiology]; young adult; tuberculostatic agent/dt [Drug Therapy].

Link to the Ovid Full Text or citation:

Click here for full text options

81.

[Evaluation of risk factors for failed/defaulted on treatment outcomes of pulmonary tuberculosis in Osaka City].

Matsumoto K., Komukai J., Kasai S., Hirota S., Koda S., Terakawa K., Shimouchi A.

Kekkaku : [Tuberculosis]. 89 (6) (pp 593-599), 2014. Date of Publication: Jun 2014.

AN: 25095644

In this study, we analyzed the relationship between the risk of discontinuing medication and patient outcomes. Newly registered patients with pulmonary tuberculosis from Osaka City who required outpatient treatment in 2011 were included in the study. We assessed the number of patient cures and the number of patients who completed medication as outcomes for successful treatment and the number of failed treatments and the number of treatments that were discontinued by patients as outcomes for failed and discontinued treatments. As related factors, we examined the risk of discontinuing medication, implementation of directly observed treatments, short course (DOTS), and planned duration of treatment. To assess the risk of discontinuing medication, we examined the following medical risk factors: (1) drug resistance to isoniazid or rifampicin, (2) diabetes, (3) use of immunosuppressive/anticancer drugs, (4) use of adrenal corticosteroid, (5) artificial dialysis, (6) human immunodeficiency virus infection/acquired immunodeficiency syndrome, (7) liver damage, and (8) side effects. The social risk factors were (1) being without a fixed address at the time of registration, (2) a history of discontinuing treatment, (3) lack of assistance with medication, (4) being elderly and requiring nursing care, (5) alcohol/drug dependence, (6) serious mental disease, (7) financial problems, (8) lack of the awareness of being ill, (9) keeping irregular hours, and (10) others. We identified 568 cases of successful treatment and 41 cases of failed and discontinued treatment. Multiple logistic regression analysis was performed, with successful treatment considered as the dependent variable 0 and failed and discontinued treatment considered as the dependent variable 1. The medical/social risk factors, positive/negative sputum smear test results, the planned duration of treatment (6 months/9 months or more), and the implementation of B type or higher DOTS were included as independent variables. The significant medical risk factors were drug resistance to isoniazid or rifampicin, the use of immunosuppressive/anticancer drugs, and side effects, with odds ratios of 4.55, 4.68, and 2.68, respectively. Further, a planned duration of treatment of 9 months or more and the implementation of B type or higher DOTS were associated with odd ratios of 4.51 and 0.35, respectively. These results highlight the need to assess risk factors for discontinuing treatment and to adopt measures to overcome these factors, such as the type of DOTS being implemented, in each case.

Emtree Heading

article; directly observed therapy; female; human; Japan; *lung tuberculosis/dt [Drug Therapy]; male; middle aged; risk factor; treatment failure; treatment outcome.

Link to the Ovid Full Text or citation:

Click here for full text options

82.

[Treatment of pulmonary tuberculosis in a low-prevalence urban area. Compliance and sputum conversion]. <Tratamiento de la tuberculosis pulmonar en un area urbana de baja prevalencia. Cumplimiento y negativizacion bacteriologica.>

Stoffel C., Lorenz R., Arce M., Rico M., Fernandez L., Imaz M.S.

Medicina. 74 (1) (pp 9-18), 2014. Date of Publication: 2014.

AN: 24561834

Several factors could be responsible for a delay in bacteriological conversion of sputum in patients with pulmonary tuberculosis (pTB) even under proper treatment. We aimed to determine those factors associated with follow-up test compliance and bacteriologic conversion at month two of treatment in patients with pTB who were receiving directly controlled treatment (DOT) in an urban area with low TB burden (notification rate: 14.9 cases/100 000, 2010-2011). We retrospectively analyzed the clinical, demographic, radiological, microbiological and therapeutic characteristics of 196 new smear-positive pTB cases. The proportion of patients who underwent bacteriological examination was 79.1% (155/196) and 74.7% (121/162) at the second and last month of treatment. Smear examinations were significantly more frequent in patients with co-morbidities or receiving DOT in health facilities with laboratory access. Diabetes (OR 17.4, 95% CI 2.0 - 27.0) and high smear grade (OR 13.8, 95% CI 1.8 - 108.0) were highly associated with persistent positive smears. Cavitation (OR 4.6, 95%, CI 1.1 - 20.5) and diabetes (OR 3.3, 95% CI 1.1 - 10.5) were associated with persistent positive cultures. Smear examination results at two months of treatment indicated a sensitivity of 63.9% (23/36) and a specificity of 82.4% (98/119) in relation to culture. The limited predictive value of smear examination relative to culture results indicated the need to find other markers for sputum conversion. Identification of risk factors associated with persistent sputum positivity could contribute to patient care and resource allocation. Prolonged infectiousness in patients with diabetes could indicate the need to review TB control strategies.

Institution

(Stoffel) Programa de Enfermedades Respiratorias y Tuberculosis de Santa Fe, Ministerio de Salud de la Provincia de Santa Fe, Argentina.

Emtree Heading

Argentina/ep [Epidemiology]; article; bacteriological conversion; directly observed therapy; female; health; human; *lung tuberculosis/dt [Drug Therapy]; *lung tuberculosis/ep [Epidemiology]; male; microbiology; middle aged; *patient compliance; prevalence; retrospective study; risk factor; *sputum; statistics; therapy; treatment outcome; tuberculostatic agent/dt [Drug Therapy].

Link to the Ovid Full Text or citation:

Click here for full text options

83.

Treatment guidelines for latent tuberculosis infection.

Prevention Committee of the Japanese Society for Tuberculosis, Treatment Committee of the Japanese Society for Tuberculosis

Kekkaku : [Tuberculosis]. 89 (1) (pp 21-37), 2014. Date of Publication: Jan 2014.

AN: 24654427

The treatment of latent tuberculosis infection (LTBI) has been established as valid for patients at high risk for developing active tuberculosis. Treatment of LTBI is also considered an important strategy for eliminating tuberculosis (TB) in Japan. In recent years, interferon-gamma release assays have come into widespread use; isoniazid (INH) preventive therapy for HIV patients has come to be recommended worldwide; and there have been increases in both types of biologics used in the treatment of immune diseases as well as the diseases susceptible to treatment. In light of the above facts, the Prevention Committee and the Treatment Committee of the Japanese Society for Tuberculosis have jointly drafted these guidelines. In determining subjects for LTBI treatment, the following must be considered: 1) risk of TB infection/ development; 2) infection diagnosis; 3) chest image diagnosis; 4) the impact of TB development; 5) the possible manifestation of side effects; and 6) the prospects of treatment completion. LTBI treatment is actively considered when relative risk is deemed 4 or higher, including risk factors such as the following: HIV/AIDS, organ transplants (immunosuppressant use), silicosis, dialysis due to chronic renal failure, recent TB infection (within 2 years), fibronodular shadows in chest radiographs (untreated old TB), the use of biologics, and large doses of corticosteroids. Although the risk is lower, the following risk factors require consideration of LTBI treatment when 2 or more of them are present: use of oral or inhaled corticosteroids, use of other immunosuppressants, diabetes, being underweight, smoking, gastrectomy, and so on. In principle, INH is administered for a period of 6 or 9 months. When INH cannot be used, rifampicin is administered for a period of 4 or 6 months. It is believed that there are no reasons to support long-term LTBI treatment for immunosuppressed patients in Japan, where the risk of infection is not considered markedly high. For pregnant women, HIV-positive individuals, heavy drinkers, and individuals with a history of liver injury, regular liver function tests are necessary when treatment is initiated and when symptoms are present. There have been reports of TB developing during LTBI treatment; therefore, attention should be paid to TB development symptoms. When administering LTBI treatment, patients must be educated about side effects, the risk of developing TB onset, and the risks associated with discontinuing medication. Treatment outcomes and support for continuation of treatment are evaluated in cooperation with health centers. As stipulated by the Infectious Diseases Control Law, doctors are required to notify a health center when an individual develops TB. Based on this notification, the health center registers the patient, sends a public health nurse to visit the patient and give instructions, and provides medication adherence support. The patient applies at a health center for public expenses for medical care at a designated TB care facility. Pending approval in a review by an infectious disease examination council, the patient's copayment is reduced.

Emtree Heading

AIDS related complex/co [Complication]; health service; human; Human immunodeficiency virus infection/co [Complication]; Japan; *latent tuberculosis/co [Complication]; *latent tuberculosis/dt [Drug Therapy]; *latent tuberculosis/pc [Prevention]; organization and management; patient education; *practice guideline; review; risk factor; immunosuppressive agent/ae [Adverse Drug Reaction]; isoniazid/ad [Drug Administration]; rifampicin/ad [Drug Administration]; tuberculostatic agent/ad [Drug Administration].

Link to the Ovid Full Text or citation:

Click here for full text options

84.

Predictors of pulmonary tuberculosis treatment outcomes in South Korea: a prospective cohort study, 2005-2012.

Choi H., Lee M., Chen R.Y., Kim Y., Yoon S., Joh J.S., Park S.K., Dodd L.E., Lee J., Song T., Cai Y., Goldfeder L.C., Via L.E., Carroll M.W., Barry C.E., Cho S.-N.

BMC infectious diseases. 14 (pp 360), 2014. Date of Publication: 2014.

AN: 24990578

BACKGROUND: Tuberculosis remains an important health concern in many countries. The aim of this study was to identify predictors of unfavorable outcomes at the end of treatment (EOT) and at the end of study (EOS; 40 months after EOT) in South Korea. METHODS: New or previously treated tuberculosis patients were recruited into a prospective observational cohort study at two hospitals in South Korea. To identify predictors of unfavorable outcomes at EOT and EOS, logistic regression analysis was performed.

RESULTS: The proportion of multidrug-resistant tuberculosis (MDR-TB) was 8.2% in new cases and 57.9% in previously treated cases. Of new cases, 68.6% were cured, as were 40.7% of previously treated cases. At EOT, diabetes, >3 previous TB episodes, >1 significant regimen change, and MDR-TB were significantly associated with treatment failure or death. At EOS, age >35, body-mass index (BMI) <18.5, diabetes, and MDR-TB were significantly associated with treatment failure, death, or relapse. Among cases that were cured at EOT, age >50 and a BMI <18.5 were associated with subsequent death or relapse during follow-up to EOS. Treatment interruption was associated with service sector employees or laborers, bilateral lesions on chest X-ray, and previous treatment failure or treatment interruption history.

CONCLUSIONS: Risk factors for poor treatment outcomes at EOT and EOS include both patient factors (diabetes status, age, BMI) and disease factors (history of multiple previous treatment episodes, MDR-TB). In this longitudinal, observational cohort study, diabetes mellitus and MDR-TB were risk factors for poor treatment outcomes and relapse. Measures to help ensure that the first tuberculosis treatment episode is also the last one may improve treatment outcomes.

TRIAL REGISTRATION: ClinicalTrials.gov ID: NCT00341601.

Institution

(Cho) International Tuberculosis Research Center, Changwon, Republic of Korea. raycho@yonsei.kr

Emtree Heading

adult; cohort analysis; female; human; male; middle aged; prospective study; risk factor; South Korea; treatment failure; treatment outcome; Tuberculosis, Multidrug-Resistant/dt [Drug Therapy]; Tuberculosis, Multidrug-Resistant/ep [Epidemiology]; Tuberculosis, Pulmonary/dt [Drug Therapy]; Tuberculosis, Pulmonary/ep [Epidemiology]; tuberculostatic agent/dt [Drug Therapy].

Link to the Ovid Full Text or citation:

Click here for full text options

85.

Does Alcohol consumption during multidrug-resistant tuberculosis treatment affect outcome?. A population-based study in Kerala, India.

Duraisamy K., Mrithyunjayan S., Ghosh S., Nair S.A., Balakrishnan S., Subramoniapillai J., Oeltmann J.E., Moonan P.K., Kumar A.M.

Annals of the American Thoracic Society. 11 (5) (pp 712-718), 2014. Date of Publication: 01 Jun 2014.

AN: 24735096

RATIONALE: India reports the largest number of multidrug-resistant tuberculosis cases in the world; yet, no longitudinal study has assessed factors related to treatment outcomes under programmatic conditions in the public sector. OBJECTIVES: To describe demographic, clinical, and risk characteristics associated with treatment outcomes for all patients with multidrug-resistant tuberculosis registered in the Revised National Tuberculosis Control Programme, Kerala State, India from January 1, 2009 to June 30, 2010.

METHODS: Cox regression methods were used to calculate adjusted hazard ratios with 95% confidence intervals (CIs) to assess factors associated with an unsuccessful treatment outcome.

MEASUREMENTS AND MAIN RESULTS: Of 179 patients with multidrug-resistant tuberculosis registered, 112 (63%) had successful treatment outcomes (77 bacteriologically cured, 35 treatment completed) and 67 (37%) had unsuccessful treatment outcomes (30 died, 26 defaulted, 9 failed treatment, 1 stopped treatment because of drug-related adverse events, and 1 developed extensively drug-resistant tuberculosis). The hazard for unsuccessful outcome was significantly higher among patients who consumed alcohol during treatment (adjusted hazard ratio, 4.3; 95% CI, 1.1-17.6) than those who did not. Persons who consumed alcohol during treatment, on average, missed 18 more intensive-phase doses (95% CI, 13-22) than those who did not. Although many patients had diabetes (33%), were ever smokers (39%), or had low body mass index (47%), these factors were not associated with outcome.

CONCLUSION: Overall treatment success was greater than global and national averages; however, outcomes among patients consuming alcohol remained poor. Integration of care for multidrug-resistant tuberculosis and alcoholism should be considered to improve treatment adherence and outcomes.

Institution

(Duraisamy, Mrithyunjayan, Ghosh, Nair, Balakrishnan, Subramoniapillai, Oeltmann, Moonan, Kumar) 1 Department of Tuberculosis, WHO Country Office for India, New Delhi, India

Emtree Heading

adult; clinical trial; drinking behavior; female; follow up; *health survey; human; incidence; India; isolation and purification; male; microbiology; middle aged; multicenter study; Mycobacterium tuberculosis; retrospective study; *risk assessment; risk factor; sputum; survival rate; treatment outcome; trends; Tuberculosis, Multidrug-Resistant/dt [Drug Therapy]; Tuberculosis, Multidrug-Resistant/ep [Epidemiology]; tuberculostatic agent/dt [Drug Therapy].

Link to the Ovid Full Text or citation:

Click here for full text options

86.

Microbiology and epidemiology of infectious spinal disease.

Jeong S.-J., Choi S.-W., Youm J.-Y., Kim H.-W., Ha H.-G., Yi J.-S.

Journal of Korean Neurosurgical Society. 56 (1) (pp 21-27), 2014. Date of Publication: 01 Jul 2014.

AN: 2015074358

Objective: Infectious spinal disease is regarded as an infection by a specific organism that affects the vertebral body, intervertebral disc and adjacent perivertebral soft tissue. Its incidence seems to be increasing as a result of larger proportion of the older patients with chronic debilitating disease, the rise of intravenous drug abuser, and the increase in spinal procedure and surgery. In Korea, studies assessing infectious spinal disease are rare and have not been addressed in recent times. The objectives of this study are to describe the epidemiology of all kind of spinal infectious disease and their clinical and microbiological characteristics as well as to assess the diagnostic methodology and the parameters related to the outcomes. Methods: A retrospective study was performed in all infectious spinal disease cases presenting from January 2005 to April 2010 to three tertiary teaching hospitals within a city of 1.5 million in Korea. Patient demographics, risk factors, clinical features, and outcomes were assessed. Risk factors entailed the presence of diabetes, chronic renal failure, liver cirrhosis, immunosuppressants, remote infection, underlying malignancy and previous spinal surgery or procedure. We comparatively analyzed the results between the groups of pyogenic and tuberculous spinal infection. SPSS version 14 statistical software was used to perform the analyses of the data. The threshold for statistical significance was established at p<0.05. Results: Ninety-two cases fulfilled the inclusion criteria and were reviewed. Overall, patients of tuberculous spinal infection (TSI) and pyogenic spinal infection (PSI) entailed 20 (21.7%) and 72 (78.3%) cases, respectively. A previous spinal surgery or procedure was the most commonly noted risk factor (39.1%), followed by diabetes (15.2%). The occurrence of both pyogenic and tuberculous spondylitis was predominant in the lumbar spine. Discs are more easily invaded in PSI. At initial presentation, white cell blood count and C-reactive protein levels were higher in PSI compared to TSI (p<0.05). Etiological agents were identified in 53.3%, and the most effective method for identification of etiological agents was tissue culture (50.0%). Staphyococcus aureus was the most commonly isolated infective agent associated with pyogenic spondylitis, followed by E. coli. Surgical treatment was performed in 31.5% of pyogenic spondylitis and in 35.0% of tuberculous spondylitis cases. Conclusion: Many previous studies in Korea usually reported that tuberculous spondylitis is the predominant infection. However, in our study, the number of pyogenic infection was 3 times greater than that of tuberculous spinal disease. Etiological agents were identified in a half of all infectious spinal disease. For better outcomes, we should try to identify the causative microorganism before antibiotic therapy and make every effort to improve the result of culture and biopsy.

Institution

(Jeong, Choi, Youm) Department of Neurosurgery, School of Medicine, Chungnam National University, Daejeon, South Korea (Kim, Ha) Department of Neurosurgery, Konyang University Hospital, Daejeon, South Korea

(Yi) Department of Neurosurgery, The Catholic University of Korea College of Medicine, Daejeon St. Mary's Hospital, Daejeon, South Korea

Publisher

Korean Neurosurgical Society

Emtree Heading

adult; article; clinical feature; comorbidity; erythrocyte sedimentation rate; Escherichia coli; female; human; leukocyte count; major clinical study; male; *microbiology; Mycobacterium; outcome assessment; Pseudomonas; retrospective study; risk factor; *spinal cord infection/ep [Epidemiology]; spondylitis; Staphylococcus aureus; Streptococcus; C reactive protein/ec [Endogenous Compound].

Link to the Ovid Full Text or citation:

Click here for full text options

87.

Screening for diabetes mellitus in patients diagnosed with pulmonary tuberculosis.

Pablo-Villamor M.P., Benedicto J.P., Benedicto M.T.J.U., Perez V.M.

Phillippine Journal of Internal Medicine. 52 (4) (pp 1-9), 2014. Date of Publication: 01 Oct 2014.

AN: 2015714619

Background: The epidemic of diabetes mellitus (DM) poses a threat for global tuberculosis (TB) control. Objective: This study attempts to assess the value of screening for diabetes in patients with pulmonary tuberculosis and reviews the disease burden, clinical and radiographic manifestations, rates of sputum smear positivity and time to conversion, treatment outcomes and fatality rates, in the local setting.

Methods: This is a prospective observational cohort study involving adults diagnosed with pulmonary tuberculosis at the PTSI TB DOTS out-patient clinic, regardless of sputum-smear status, from July 2011-November 2012. A diabetes screening tool was used and patients were screened for presence of DM. Treatment outcomes were also determined.

Results: Of the 38 patients enrolled, seven (18.4%, 95% confidence interval 7.7-34.3) were diagnosed with DM. This is higher than the estimated 12.9% in 2010 and 14.4% projected estimate in 2030 in our country by a report of WHO as well as in reported prevalence of DM among patients with PTB in large studies done in China (12.4%) and India (13%). There was no significant difference noted in the basic profile, clinical and radiographic presentation, sputum conversion and treatment outcomes among patients with DM and without DM who were being treated for PTB.

Conclusion: This study demonstrates the value and feasibility of screening for DM among patients with PTB. Although the findings of this study are consistent with most of previous similar studies, the estimate on the true prevalence of DM may not be very accurate because of the small sample size. Hence, a multi-center study with a larger sample size must be conducted to more accurately measure the true prevalence of DM among patients with TB and to determine associations of various clinical and radiographic presentations and clinical outcomes.

Institution

(Pablo-Villamor) Medical Specialist II, Vicente Sotto Memorial Medical Center, Philippines (Benedicto) Philippine Tuberculosis Society Inc, Philippines

(Benedicto) Diagnostic Section Department of Radiology, PGH, Philippines

(Perez) Quezon Institute and Central Chest Clinic and Dispensary Philippine Tuberculosis Society Inc, Philippines

Publisher

Philippine College of Physicians

Emtree Heading

adult; article; China; clinical article; clinical feature; cohort analysis; controlled study; *diabetes mellitus/di [Diagnosis]; diagnostic value; disease association; fatality; feasibility study; female; human; India; *lung tuberculosis; male; observational study; outpatient care; prevalence; prospective study; radiography; risk factor; sample size; *screening test; short course therapy; sputum smear; systematic review; treatment outcome.

Link to the Ovid Full Text or citation:

Click here for full text options

88.

Association between diabetes mellitus and tuberculosis in United States-Born and foreign-born populations in San Francisco.

Suwanpimolkul G., Grinsdale J.A., Jarlsberg L.G., Higashi J., Osmond D.H., Hopewell P.C., Kato-Maeda M.

PLoS ONE. 9 (12) (no pagination), 2014. Article Number: e114442. Date of Publication: 05 Dec 2014.

AN: 2014959756

Setting: The impact of diabetes on tuberculosis in United States and foreign-born populations in San Francisco has not been studied. Objective: To determine the characteristics, prevalence and temporal trends of diabetes in US and foreign-born persons attending the San Francisco Tuberculosis Clinic. Design: We analyzed data from individuals seeking medical attention at the San Francisco Tuberculosis Clinic. We included patients with diagnosis of tuberculosis, latent infection, or not infected with Mycobacterium tuberculosis. We assessed the temporal trend and the characteristics of individuals with and without diabetes. Result: Between 2005 and 2012, there were 4371 (19.0%) individuals without evidence of tuberculosis infection, 17,856 (77.6%) with latent tuberculosis, and 791 (3.4%) with tuberculosis. 66% were born in the United States, China, Mexico, and the Philippines. The prevalence of diabetes was the highest among individuals with tuberculosis and increased during the study period. Patients with tuberculosis and diabetes were more likely to be male, older than 45 years and born in the Philippines. There was a disproportionate association of TB and DM relative to LTBI and DM among Filipinos in individuals older than 45 years old. Conclusions: Our data suggest that Filipinos older than 45 years old are more likely to have tuberculosis probably due to a higher prevalence of diabetes. In San Francisco, tuberculosis-screening programs in individuals with diabetes and latent tuberculosis may be beneficial in patients older than 45 years old especially from the Philippines.

Institution

(Suwanpimolkul, Jarlsberg, Hopewell, Kato-Maeda) Curry International Tuberculosis Center, University of California San Francisco, San Francisco, CA, United States (Suwanpimolkul) Faculty of Medicine, Chulalongkorn University, King Chulalongkorn Memorial Hospital, Bangkok, Thailand

(Grinsdale, Higashi) Tuberculosis Control, San Francisco Department of Public Health, San Francisco, CA, United States

(Osmond) Department of Epidemiology and Biostatistics, University of California San Francisco, San Francisco, CA, United States

(Grinsdale) San Francisco Department of Public Health, Office of Equity and Quality Improvement, Population Health Division, San Francisco, CA, United States

Publisher

Public Library of Science

Emtree Heading

African American; American; American Indian; article; birthplace; China; controlled study; death; *diabetes mellitus/di [Diagnosis]; *disease association; disease predisposition; Ethiopia; female; Filipino (people); Hispanic; human; interferon gamma release assay; latent tuberculosis/dr [Drug Resistance]; major clinical study; male; mass screening; Mexico; multidrug resistance; Mycobacterium tuberculosis; observational study; Pacific Islander; Philippines; prevalence; retrospective study; risk factor; sputum smear; treatment failure; treatment outcome; trend study; tuberculin test; *tuberculosis/dr [Drug Resistance]; tuberculosis control; United States; West African.

Link to the Ovid Full Text or citation:

Click here for full text options

89.

Survival analysis of adult tuberculosis disease.

Ajagbe O.B., Kabair Z., O'Connor T.

PLoS ONE. 9 (11) (no pagination), 2014. Article Number: 112838. Date of Publication: 19 Nov 2014.

AN: 2014954075

Background: We conducted a survival analysis of all the confirmed cases of Adult Tuberculosis (TB) patients treated in Cork-City, Ireland. The aim of this study was to estimate Survival time (ST), including median time of survival and to assess the association and impact of covariates (TB risk factors) to event status and ST. The outcome of the survival analysis is reported in this paper. Methods: We used a retrospective cohort study research design to review data of 647 bacteriologically confirmed TB patients from the medical record of two teaching hospitals. Mean age 49 years (Range 18-112). We collected information on potential risk factors of all confirmed cases of TB treated between 2008-2012. For the survival analysis, the outcome of interest was 'treatment failure' or 'death' (whichever came first). A univariate descriptive statistics analysis was conducted using a non-parametric procedure, Kaplan-Meier (KM) method to estimate overall survival (OS), while the Cox proportional hazard model was used for the multivariate analysis to determine possible association of predictor variables and to obtain adjusted hazard ratio. P value was set at <0.05, log likelihood ratio test at >0.10. Data were analysed using SPSS version 15.0.

Results: There was no significant difference in the survival curves of male and female patients. (Log rank statistic = 0.194, df = 1, p = 0.66) and among different age group (Log rank statistic = 1.337, df = 3, p = 0.72). The mean overall survival (OS) was 209 days (95%CI: 92-346) while the median was 51 days (95% CI: 35.7-66). The mean ST for women was 385 days (95%CI: 76.6-694) and for men was 69 days (95%CI: 48.8-88.5). Multivariate Cox regression showed that patient who had history of drug misuse had 2.2 times hazard than those who do not have drug misuse. Smokers and alcohol drinkers had hazard of 1.8 while patients born in country of high endemicity (BICHE) had hazard of 6.3 and HIV co-infection hazard was 1.2.

Conclusion: There was no significant difference in survival curves of male and female and among age group. Women had a higher ST compared to men. But men had a higher hazard rate compared to women. Anti-TNF, immunosuppressive medication and diabetes were found to be associated with longer ST, while alcohol, smoking, RICHE, BICHE was associated with shorter ST.

Institution

(Ajagbe, Kabair) Department of Public Health and Epidemiology, University College Cork, Cork, Ireland (O'Connor) Mercy University Hospital, Cork, Ireland

Publisher

Public Library of Science

Emtree Heading

adult; age; aged; alcohol consumption; article; cohort analysis; diabetes mellitus; disease association; drug misuse; endemic disease; ethnicity; female; hazard ratio; human; Human immunodeficiency virus infection; Ireland; Kaplan Meier method; major clinical study; male; medical history; medical record review; middle aged; mixed infection; mortality; multivariate analysis; overall survival; prediction; proportional hazards model; retrospective study; risk factor; sex difference; smoking; *survival rate; survival time; teaching hospital; treatment failure; *tuberculosis/pc [Prevention]; *tuberculosis/dt [Drug Therapy]; very elderly; BCG vaccine/dt [Drug Therapy]; immunosuppressive agent; tuberculostatic agent/dt [Drug Therapy]; tumor necrosis factor inhibitor.

Link to the Ovid Full Text or citation:

Click here for full text options

90.

Ulcerative colitis: Epidemiology, diagnosis, and management.

Feuerstein J.D., Cheifetz A.S.

Mayo Clinic Proceedings. 89 (11) (pp 1553-1563), 2014. Date of Publication: 01 Nov 2014.

AN: 2014868903

Ulcerative colitis is a chronic idiopathic inflammatory bowel disease characterized by continuous mucosal inflammation that starts in the rectum and extends proximally. Typical presenting symptoms include bloody diarrhea, abdominal pain, urgency, and tenesmus. In some cases, extraintestinal manifestations may be present as well. In the right clinical setting, the diagnosis of ulcerative colitis is based primarily on endoscopy, which typically reveals evidence of continuous colonic inflammation, with confirmatory biopsy specimens having signs of chronic colitis. The goals of therapy are to induce and maintain remission, decrease the risk of complications, and improve quality of life. Treatment is determined on the basis of the severity of symptoms and is classically a step-up approach. 5-Aminosalycilates are the mainstay of treatment for mild to moderate disease. Patients with failed 5-aminosalycilate therapy or who present with more moderate to severe disease are typically treated with corticosteroids followed by transition to a steroid-sparing agent with a thiopurine, anti-tumor necrosis factor agent, or adhesion molecule inhibitor. Despite medical therapies, approximately 15% of patients still require proctocolectomy. In addition, given the potential risks of complications from the disease itself and the medications used to treat the disease, primary care physicians play a key role in optimizing the preventive care to reduce the risk of complications.

Institution

(Feuerstein, Cheifetz) Department of Medicine, Beth Israel Deaconess Medical Center, Harvard Medical School, 330 Brookline Ave E/DANA 501, Boston, MA 02215, United States

Publisher

Elsevier Ltd

Emtree Heading

adrenal insufficiency/si [Side Effect]; antibiotic therapy; avascular necrosis/si [Side Effect]; bile duct carcinoma; bone marrow suppression/si [Side Effect]; cataract/si [Side Effect]; clinical feature; colon resection; colorectal cancer/co [Complication]; colorectal cancer/di [Diagnosis]; congestive heart failure/si [Side Effect]; cost effectiveness analysis; delirium/si [Side Effect]; demyelinating disease/si [Side Effect]; diabetes mellitus/si [Side Effect]; diarrhea/si [Side Effect]; disease severity; general practitioner; genetic risk; genetic variability; glaucoma/si [Side Effect]; headache/si [Side Effect]; hemolytic anemia/si [Side Effect]; hepatitis/si [Side Effect]; hepatitis B/si [Side Effect]; histopathology; human; hyperkalemia/si [Side Effect]; hypertension/si [Side Effect]; hypertrichosis/si [Side Effect]; ileostomy; infection/si [Side Effect]; infusion related reaction/si [Side Effect]; injection site reaction/si [Side Effect]; insomnia/si [Side Effect]; interstitial nephritis/si [Side Effect]; intestine biopsy; laboratory test; leukopenia/si [Side Effect]; lupus erythematosus/si [Side Effect]; lymphoma/si [Side Effect]; melanoma/si [Side Effect]; mood disorder/si [Side Effect]; nausea/si [Side Effect]; nephrotoxicity/si [Side Effect]; non melanoma skin cancer/si [Side Effect]; nonhodgkin lymphoma/si [Side Effect]; osteopenia/si [Side Effect]; osteoporosis/si [Side Effect]; pancreatitis/si [Side Effect]; paradoxical drug reaction/si [Side Effect]; pathogenesis; patient compliance; pharynx disease/si [Side Effect]; physician attitude; primary sclerosing cholangitis; proctocolectomy; progressive multifocal leukoencephalopathy/si [Side Effect]; psoriasis/si [Side Effect]; review; risk assessment; risk factor; seizure/si [Side Effect]; skin cancer/si [Side Effect]; smoking habit; treatment outcome; tuberculosis/si [Side Effect]; *ulcerative colitis/di [Diagnosis]; *ulcerative colitis/ep [Epidemiology]; *ulcerative colitis/et [Etiology]; *ulcerative colitis/su [Surgery]; *ulcerative colitis/dt [Drug Therapy]; vomiting/si [Side Effect]; wound healing impairment/si [Side Effect]; 6 mercaptopurine derivative/ae [Adverse Drug Reaction]; 6 mercaptopurine derivative/cb [Drug Combination]; 6 mercaptopurine derivative/dt [Drug Therapy]; adalimumab/po [Oral Drug Administration]; adalimumab/sc [Subcutaneous Drug Administration]; adalimumab/pd [Pharmacology]; adalimumab/dt [Drug Therapy]; aminosalicylic acid/ae [Adverse Drug Reaction]; aminosalicylic acid/po [Oral Drug Administration]; aminosalicylic acid/rc [Rectal Drug Administration]; aminosalicylic acid/dt [Drug Therapy]; azathioprine/po [Oral Drug Administration]; azathioprine/dt [Drug Therapy]; balsalazide/po [Oral Drug Administration]; balsalazide/rc [Rectal Drug Administration]; balsalazide/dt [Drug Therapy]; budesonide/po [Oral Drug Administration]; budesonide/rc [Rectal Drug Administration]; budesonide/iv [Intravenous Drug Administration]; budesonide/dt [Drug Therapy]; calcineurin inhibitor/po [Oral Drug Administration]; calcineurin inhibitor/iv [Intravenous Drug Administration]; calcineurin inhibitor/ae [Adverse Drug Reaction]; calcineurin inhibitor/dt [Drug Therapy]; corticosteroid/po [Oral Drug Administration]; corticosteroid/rc [Rectal Drug Administration]; corticosteroid/iv [Intravenous Drug Administration]; corticosteroid/ae [Adverse Drug Reaction]; corticosteroid/dt [Drug Therapy]; cyclosporin/po [Oral Drug Administration]; cyclosporin/iv [Intravenous Drug Administration]; cyclosporin/dt [Drug Therapy]; golimumab/po [Oral Drug Administration]; golimumab/sc [Subcutaneous Drug Administration]; golimumab/pd [Pharmacology]; golimumab/dt [Drug Therapy]; infliximab/po [Oral Drug Administration]; infliximab/iv [Intravenous Drug Administration]; infliximab/sc [Subcutaneous Drug Administration]; infliximab/cb [Drug Combination]; infliximab/dt [Drug Therapy]; mercaptopurine/cb [Drug Combination]; mercaptopurine/dt [Drug Therapy]; mesalazine/po [Oral Drug Administration]; mesalazine/rc [Rectal Drug Administration]; mesalazine/dt [Drug Therapy]; natalizumab/pd [Pharmacology]; natalizumab/ae [Adverse Drug Reaction]; natalizumab/dt [Drug Therapy]; nonsteroid antiinflammatory agent/dt [Drug Therapy]; prednisone/po [Oral Drug Administration]; prednisone/rc [Rectal Drug Administration]; prednisone/iv [Intravenous Drug Administration]; prednisone/dt [Drug Therapy]; probiotic agent/dt [Drug Therapy]; steroid/iv [Intravenous Drug Administration]; steroid/dt [Drug Therapy]; tacrolimus/po [Oral Drug Administration]; tacrolimus/iv [Intravenous Drug Administration]; tacrolimus/dt [Drug Therapy]; tumor necrosis factor inhibitor/ae [Adverse Drug Reaction]; tumor necrosis factor inhibitor/cb [Drug Combination]; tumor necrosis factor inhibitor/iv [Intravenous Drug Administration]; tumor necrosis factor inhibitor/sc [Subcutaneous Drug Administration]; tumor necrosis factor inhibitor/dt [Drug Therapy]; vedolizumab/iv [Intravenous Drug Administration]; vedolizumab/pd [Pharmacology]; vedolizumab/ae [Adverse Drug Reaction]; vedolizumab/dt [Drug Therapy].

Link to the Ovid Full Text or citation:

Click here for full text options

91.

Pulmonary tuberculosis increases the risk of pulmonary thromboembolism: A nationwide population-based cohort study.

Chung W.-S., Lin C.-L., Chen Y.-F., Hsu W.-H., Kao C.-H.

Thrombosis and Haemostasis. 112 (6) (pp 1325-1327), 2014. Date of Publication: 2014.

AN: 2014948743

Institution

(Chung) Department of Internal Medicine, Taichung Hospital, Ministry of Health and Welfare, Executive Yuan, Taichung, Taiwan (Republic of China) (Chung, Hsu, Kao) Graduate Institute of Clinical Medical Science and School of Medicine, College of Medicine, China Medical University, Taichung, Taiwan (Republic of China)

(Chung, Chen) Department of Healthcare Administration, Central Taiwan University of Science and Technology, Taichung, Taiwan (Republic of China)

(Lin) Management Office for Health Data, China Medical University Hospital, Taichung, Taiwan (Republic of China)

(Kao) Department of Nuclear Medicine and PET Center, China Medical University Hospital, Taichung, Taiwan (Republic of China)

Publisher

Schattauer GmbH

Emtree Heading

adult; aged; cerebrovascular accident; clinical article; cohort analysis; comorbidity; controlled study; diabetes mellitus; female; hazard ratio; heart atrium fibrillation; heart failure; hormone substitution; human; hyperlipidemia; hypertension; incidence; leg fracture; letter; *lung embolism; *lung tuberculosis; male; Mycobacterium tuberculosis; national health insurance; neoplasm; outcome assessment; risk factor; venous thromboembolism; anticoagulant agent; antithrombocytic agent; contraceptive agent.

Link to the Ovid Full Text or citation:

Click here for full text options

92.

Extra-pulmonary tuberculosis: the comorbidityof the near future?.

Sotgiu G., Gegia M., Migliori G.B.

International Journal of Tuberculosis and Lung Disease. 18 (12) (pp 1389), 2014. Date of Publication: 01 Dec 2014.

AN: 2014916764

Institution

(Sotgiu) Clinical Epidemiology and Medical Statistics Unit, Department of Biomedical Sciences, University of Sassari, Sassari, Italy (Gegia) USAID Georgia TB Prevention Project, University Research Co., LLC Branch in Georgia, Tbilisi, Georgia

(Migliori) World Health Organization Collaborating Centre for TB and Lung Diseases, Fondazione S. Maugeri, Care and Research Institute, Tradate, Italy

Publisher

International Union against Tubercul. and Lung Dis.

Emtree Heading

acquired immune deficiency syndrome; bacterial strain; bacterium identification; *comorbidity; diabetes mellitus; disease association; disease severity; editorial; *extrapulmonary tuberculosis; health hazard; human; Human immunodeficiency virus; Human immunodeficiency virus infection; infection risk; medical research; Mycobacterium tuberculosis; outcome assessment; risk assessment; risk factor.

Link to the Ovid Full Text or citation:

Click here for full text options

93.

Clinical management of concurrent diabetes and tuberculosis and the implications for patient services.

Riza A.L., Pearson F., Ugarte-Gil C., Alisjahbana B., Van de Vijver S., Panduru N.M., Hill P.C., Ruslami R., Moore D., Aarnoutse R., Critchley J.A., van Crevel R.

The Lancet Diabetes and Endocrinology. 2 (9) (pp 740-753), 2014. Date of Publication: 01 Sep 2014.

AN: 2014859615

Diabetes triples the risk for active tuberculosis, thus the increasing burden of type 2 diabetes will help to sustain the present tuberculosis epidemic. Recommendations have been made for bidirectional screening, but evidence is scarce about the performance of specific tuberculosis tests in individuals with diabetes, specific diabetes tests in patients with tuberculosis, and screening and preventive therapy for latent tuberculosis infections in individuals with diabetes. Clinical management of patients with both diseases can be difficult. Tuberculosis patients with diabetes have a lower concentration of tuberculosis drugs and a higher risk of drug toxicity than tuberculosis patients without diabetes. Good glycaemic control, which reduces long-term diabetes complications and could also improve tuberculosis treatment outcomes, is hampered by chronic inflammation, drug-drug interactions, suboptimum adherence to drug treatments, and other factors. Besides drug treatments for tuberculosis and diabetes, other interventions, such as education, intensive monitoring, and lifestyle interventions, might be needed, especially for patients with newly diagnosed diabetes or those who need insulin. From a health systems point of view, delivery of optimum care and integration of services for tuberculosis and diabetes is a huge challenge in many countries. Experience from the combined tuberculosis and HIV/AIDS epidemic could serve as an example, but more studies are needed that include economic assessments of recommended screening and systems to manage concurrent tuberculosis and diabetes.

Institution

(Riza, van Crevel) Department of Internal Medicine, Radboud University Medical Center, Nijmegen, Netherlands (Aarnoutse) Department of Pharmacy, Radboud University Medical Center, Nijmegen, Netherlands

(Riza, Panduru) Human Genomics Laboratory, University of Medicine and Pharmacy of Craiova, Craiova, Romania

(Pearson, Critchley) Population Health Research Institute, St Georges University, London, United Kingdom

(Ugarte-Gil) Universidad Peruana Cayetano Heredia, Lima, Peru

(Ugarte-Gil) Department of International Health, Johns Hopkins Bloomberg School of Public Health, Baltimore, MD, United States

(Alisjahbana) Center for TB-HIV research, Medical Faculty, Padjadjaran University, Hasan Sadikin Hosptial, Bandung, Indonesia

(Van de Vijver) African Population and Health Research Center, Nairobi, Kenya

(Van de Vijver) Department of Global Health, Academic Medical Center, University of Amsterdam, Amsterdam Institute for Global Health and Development, Amsterdam, Netherlands

(Panduru) 2nd Clinical Department, Carol Davila University of Medicine and Pharmacy, Bucharest, Romania

(Hill) Centre for International Health, Faculty of Medicine, Otago University, Dunedin, New Zealand

(Ruslami) Department of Pharmacology and Therapy, Faculty of Medicine, Padjadjaran University, Bandung, Indonesia

(Moore) London School of Hygiene and Tropical Medicine, London, United Kingdom

Publisher

Lancet Publishing Group

Emtree Heading

blood glucose monitoring; clinical feature; *diabetes mellitus/di [Diagnosis]; *diabetes mellitus/ep [Epidemiology]; diagnostic test; disease association; drug choice; drug cost; drug safety; glycemic control; health care availability; health care delivery; health economics; *health service; human; latent tuberculosis/di [Diagnosis]; lifestyle modification; *managed care; needs assessment; outcome assessment; patient education; patient monitoring; pharmaceutical care; public health service; review; risk assessment; screening test; *tuberculosis/di [Diagnosis]; *tuberculosis/ep [Epidemiology]; *tuberculosis/dt [Drug Therapy]; ethambutol/dt [Drug Therapy]; pyrazinamide/dt [Drug Therapy]; rifampicin/dt [Drug Therapy].

Link to the Ovid Full Text or citation:

Click here for full text options

94.

Improving tuberculosis prevention and care through addressing the global diabetes epidemic: From evidence to policy and practice.

Lonnroth K., Roglic G., Harries A.D.

The Lancet Diabetes and Endocrinology. 2 (9) (pp 730-739), 2014. Date of Publication: 01 Sep 2014.

AN: 2014859614

Diabetes triples the risk of tuberculosis and is also a risk factor for adverse tuberculosis treatment outcomes, including death. Prevalence of diabetes is increasing globally, but most rapidly in low-income and middle-income countries where tuberculosis is a grave public health problem. Growth in this double disease burden creates additional obstacles for tuberculosis care and prevention. We review how the evolution of evidence on the link between tuberculosis and diabetes has informed global policy on collaborative activities, and how practice is starting to change as a consequence. We conclude that coordinated planning and service delivery across communicable and non-communicable disease programmes is necessary, feasible, and creates synergies that will help to reduce the burden of both tuberculosis and diabetes.

Institution

(Lonnroth) Departments of Global TB Programme, World Health Organization, Geneva, Switzerland (Roglic) Management of Non-communicable Diseases, World Health Organization, Geneva, Switzerland

(Harries) The International Union Against TB and Lung Diseases, Paris, France

Publisher

Lancet Publishing Group

Emtree Heading

chemoprophylaxis; *clinical practice; *diabetes mellitus; disease association; epidemic; evidence based medicine; health care access; *health care policy; health service; human; infection prevention; infection risk; medication compliance; *patient care; review; risk assessment; screening test; *total quality management; treatment response; tuberculosis/dt [Drug Therapy]; *tuberculosis control; isoniazid/dt [Drug Therapy]; rifapentine/dt [Drug Therapy].

Link to the Ovid Full Text or citation:

Click here for full text options

95.

Diabetes and tuberculosis-a wake-up call.

Anonymous

The Lancet Diabetes and Endocrinology. 2 (9) (pp 677), 2014. Date of Publication: 01 Sep 2014.

AN: 2014859612

Publisher

Lancet Publishing Group

Emtree Heading

communicable disease; *diabetes mellitus; disease association; disease severity; editorial; health care access; health program; human; Human immunodeficiency virus infection; infection prevention; infection risk; non communicable disease; non insulin dependent diabetes mellitus; obesity; patient care; treatment outcome; *tuberculosis; tuberculosis control.

Link to the Ovid Full Text or citation:

Click here for full text options

96.

Treatment outcomes for patients with multidrug-resistant tuberculosis in post-earthquake Port-au-Prince, Haiti.

Charles M., Vilbrun S.C., Koenig S.P., Hashiguchi L.M., Mabou M.M., Ocheretina O., Pape J.W.

American Journal of Tropical Medicine and Hygiene. 91 (4) (pp 715-721), 2014. Date of Publication: 01 Oct 2014.

AN: 2014825083

We report outcomes and 12-month survival for the first cohort of patients to undergo multidrug-resistant tuberculosis (MDR-TB) treatment after the earthquake in Haiti. From March 3, 2010 to March 28, 2013, 110 patients initiated treatment of laboratory-confirmed MDR-TB at the Groupe Haitien d'Etude du Sarcome de Kaposi et des Infections Opportunistes (GHESKIO) Center in Port-au-Prince, Haiti. Twenty-seven patients (25%) were human immunodeficiency virus (HIV)-positive. As of October 31, 2013, 95 (86%) patients were either cured or alive on treatment, 4 (4%) patients defaulted, and 11 (10%) patients died. Culture conversion occurred by 30 days in 14 (13%) patients, 60 days in 49 (45%) patients, and 90 days in 81 (74%) patients. The probabilities of survival to 12 months were 96% (95% confidence interval [95% CI] = 89-99) and 85% (95% CI = 64-94) for HIV-negative and -positive patients, respectively. Despite adverse conditions, outcomes for patients with MDR-TB are highly encouraging. Major efforts are underway to scale up community directly observed therapy and expand care to other regions of Haiti.

Institution

(Charles) Division of Global Health Protection, Center for Global Health, Centers for Disease Control and Prevention, Atlanta, GA 30333, United States (Vilbrun, Hashiguchi, Mabou, Pape) Les Centres Groupe Haitien d'Etude du Sarcome de Kaposi et des Infections Opportunistes (GHESKIO), Port-au-Prince, Haiti

(Koenig) Brigham and Women's Hospital, Harvard Medical School, Boston, MA, United States

(Ocheretina) Center for Global Health, Division of Infectious Diseases, Weill Cornell Medical College, New York, NY, United States

Publisher

American Society of Tropical Medicine and Hygiene (111 Deer Lake Road, Suite 100,Deerfield, Illinois 60015, United States)

Emtree Heading

acquired immune deficiency syndrome/dt [Drug Therapy]; adult; ambulatory care; antibiotic resistance; *antibiotic therapy; antiviral therapy; anxiety disorder/si [Side Effect]; arthralgia/si [Side Effect]; article; cause of death; depression; diabetes mellitus/dt [Drug Therapy]; directly observed therapy; drug hypersensitivity/si [Side Effect]; drug megadose; drug withdrawal; earthquake; female; follow up; gastrointestinal symptom/si [Side Effect]; gynecomastia/si [Side Effect]; Haiti; hearing impairment/si [Side Effect]; hemoglobin blood level; home care; human; Human immunodeficiency virus infection/dt [Drug Therapy]; hypothyroidism/si [Side Effect]; major clinical study; male; medication compliance; mortality; *multidrug resistant tuberculosis/dt [Drug Therapy]; Mycobacterium tuberculosis; nephrotoxicity/si [Side Effect]; ototoxicity/si [Side Effect]; peripheral neuropathy/si [Side Effect]; pneumothorax/co [Complication]; psychosis/si [Side Effect]; psychosocial care; respiratory failure/co [Complication]; seizure/si [Side Effect]; skin disease/si [Side Effect]; sputum culture; survival rate; survival time; therapy delay; treatment duration; treatment outcome; treatment response; amikacin/ae [Adverse Drug Reaction]; amikacin/dt [Drug Therapy]; aminosalicylic acid/ae [Adverse Drug Reaction]; aminosalicylic acid/dt [Drug Therapy]; amoxicillin plus clavulanic acid/ae [Adverse Drug Reaction]; amoxicillin plus clavulanic acid/dt [Drug Therapy]; capreomycin/ae [Adverse Drug Reaction]; capreomycin/dt [Drug Therapy]; cycloserine/ae [Adverse Drug Reaction]; cycloserine/dt [Drug Therapy]; ethambutol/ae [Adverse Drug Reaction]; ethambutol/dt [Drug Therapy]; ethionamide/ae [Adverse Drug Reaction]; ethionamide/dt [Drug Therapy]; hemoglobin/ec [Endogenous Compound]; isoniazid/ae [Adverse Drug Reaction]; isoniazid/dt [Drug Therapy]; isoniazid/do [Drug Dose]; kanamycin/ae [Adverse Drug Reaction]; kanamycin/dt [Drug Therapy]; levofloxacin/ae [Adverse Drug Reaction]; levofloxacin/dt [Drug Therapy]; lopinavir/cb [Drug Combination]; lopinavir/dt [Drug Therapy]; moxifloxacin/ae [Adverse Drug Reaction]; moxifloxacin/dt [Drug Therapy]; ofloxacin/ae [Adverse Drug Reaction]; ofloxacin/dt [Drug Therapy]; pyrazinamide/ae [Adverse Drug Reaction]; pyrazinamide/dt [Drug Therapy]; rifampicin/ae [Adverse Drug Reaction]; rifampicin/dt [Drug Therapy]; ritonavir/cb [Drug Combination]; ritonavir/dt [Drug Therapy]; RNA directed DNA polymerase inhibitor/cb [Drug Combination]; RNA directed DNA polymerase inhibitor/dt [Drug Therapy]; streptomycin/ae [Adverse Drug Reaction]; streptomycin/dt [Drug Therapy]; *tuberculostatic agent/ae [Adverse Drug Reaction]; *tuberculostatic agent/dt [Drug Therapy].

Link to the Ovid Full Text or citation:

Click here for full text options

97.

Results of the implementation of a pilot model for the bidirectional screening and joint management of patients with pulmonary Tuberculosis and Diabetes mellitus in Mexico.

Castellanos-Joya M., Delgado-Sanchez G., Ferreyra-Reyes L., Cruz-Hervert P., Ferreira-Guerrero E., Ortiz-Solis G., Jimenez M.I., Salazar L.L., Montero-Campos R., Mongua-Rodriguez N., Baez-Saldana R., Bobadilla-Del-Valle M., Gonza Lez-Roldan J.F., Ponce-De-Leon A., Sifuentes-Osornio J., Garcia-Garcia L.

PLoS ONE. 9 (9) (no pagination), 2014. Article Number: e106961. Date of Publication: 17 Sep 2014.

AN: 2014804910

Objective: To evaluate the feasibility and effectiveness of the CFTB/DM in Mexico. Design. Prospective observational cohort. Setting. 15 primary care units in 5 states in Mexico. Participants: Patients aged >20 years diagnosed with DM or pulmonary TB who sought care at participating clinics. Intervention: The WHO/Union CFTB/DM was adapted and implemented according to official Mexican guidelines. We recruited participants from July 2012 to April 2013 and followed up until March 2014. Bidirectional screening was performed. Patients diagnosed with TB and DM were invited to receive TB treatment under joint management. Main outcome measures. Diagnoses of TB among DM, of DM among TB, and treatment outcomes among patients with DM and TB. Background: Recently, the World Health Organisation and the International Union Against Tuberculosis and Lung Disease published a Collaborative Framework for the Care and Control of Tuberculosis (TB) and Diabetes (DM) (CFTB/DM) proposing bidirectional screening and joint management.

Results: Of 783 DM patients, 11 (1.4%) were unaware of their TB. Of 361 TB patients, 16 (4.4%) were unaware of their DM. 95 TB/DM patients accepted to be treated under joint management, of whom 85 (89.5%) successfully completed treatment. Multiple linear regression analysis with change in HbA1c and random capillary glucose as dependent variables revealed significant decrease with time (regression coefficients (beta) =20.660, (95% confidence interval (CI), 20.96 to 20.35); and beta =21.889 (95% CI, 22.77 to 21.01, respectively)) adjusting by sex, age and having been treated for a previous TB episode. Patients treated under joint management were more likely to experience treatment success than patients treated under routine DM and TB programs as compared to historical (adjusted OR (aOR), 2.8, 95%CI 1.28-6.13) and same period (aOR 2.37, 95% CI 1.13-4.96) comparison groups.

Conclusions: Joint management of TB and DM is feasible and appears to improve clinical outcomes.

Institution

(Castellanos-Joya, Jimenez, Salazar) Direccion Del Programa de Micobacteriosis, Centro Nacional de Programas Preventivos y Control de Enfermedades, Mexico, Distrito Federal, Mexico (Delgado-Sanchez, Ferreyra-Reyes, Cruz-Hervert, Ferreira-Guerrero, Montero-Campos, Mongua-Rodriguez, Baez-Saldana, Garcia-Garcia) Centro de Investigacion Sobre Enfermedades Infecciosas, Instituto Nacional de Salud Publica, Cuernavaca, Morelos, Mexico

(Ortiz-Solis) Direccion Del Programa de Salud en El Adulto y en El Anciano, Centro Nacional de Programas Preventivos y Control de Enfermedades, Mexico, Distrito Federal, Mexico

(Bobadilla-Del-Valle, Ponce-De-Leon) Laboratorio de Microbiologia, Instituto Nacional de Ciencias Medicas y de Nutricion Salvador Zubira N, Mexico, Distrito Federal, Mexico

(Gonza Lez-Roldan) Direccion General, Centro Nacional de Programas Preventivos y Control de Enfermedades, Mexico, Distrito Federal, Mexico

(Sifuentes-Osornio) Direccion Medica, Instituto Nacional de Ciencias Medicas y de Nutricion Salvador Zubiran, Mexico, Distrito Federal, Mexico

Publisher

Public Library of Science

Emtree Heading

adult; age; aged; article; *bidirectional screening; clinical effectiveness; cohort analysis; Collaborative Framework for the Care and Control of Tuberculosis and Diabetes; control strategy; controlled study; *diabetes mellitus/di [Diagnosis]; *diabetes mellitus/ep [Epidemiology]; *diabetes mellitus/pc [Prevention]; *diabetes mellitus/dt [Drug Therapy]; disease control; feasibility study; female; follow up; glucose blood level; *health care planning; hemoglobin blood level; human; human cell; *lung tuberculosis/di [Diagnosis]; *lung tuberculosis/ep [Epidemiology]; *lung tuberculosis/pc [Prevention]; *lung tuberculosis/dt [Drug Therapy]; major clinical study; male; Mexico; multiple linear regression analysis; observational study; *patient care; physical activity; pilot study; prospective study; screening; sex difference; glucose/ec [Endogenous Compound]; hemoglobin A1c/ec [Endogenous Compound]; insulin/dt [Drug Therapy]; isoniazid/dt [Drug Therapy]; oral antidiabetic agent/po [Oral Drug Administration]; oral antidiabetic agent/dt [Drug Therapy]; rifampicin/dt [Drug Therapy].

Link to the Ovid Full Text or citation:

Click here for full text options

98.

Update on Janus Kinase Antagonists in Inflammatory Bowel Disease.

Boland B.S., Sandborn W.J., Chang J.T.

Gastroenterology Clinics of North America. 43 (3) (pp 603-617), 2014. Date of Publication: September 2014.

AN: 2014534256

Janus kinase (JAK) inhibitors have emerged as a novel orally administered small-molecule therapy for the treatment of ulcerative colitis and possibly Crohn disease. These molecules are designed to selectively target the activity of specific JAKs and to offer a targeted mechanism of action without risk of immunogenicity. Based on data from clinical trials in rheumatoid arthritis and phase 2 studies in inflammatory bowel disease, tofacitinib and other JAK inhibitors are likely to become a new form of medical therapy for the treatment of inflammatory bowel disease. © 2014 Elsevier Inc.

Institution

(Boland, Sandborn, Chang) Division of Gastroenterology, Department of Medicine, Inflammatory Bowel Disease Center, University of California San Diego, 9500 Gilman Drive, La Jolla, CA 92093, United States (Boland, Sandborn, Chang) Digestive Diseases Research Development Center, University of California San Diego, 9500 Gilman Drive, La Jolla, CA 92093, United States

Publisher

W.B. Saunders

Emtree Heading

abdominal pain/si [Side Effect]; autoimmune disease/dt [Drug Therapy]; bone marrow suppression; bronchiectasis/si [Side Effect]; bronchitis/si [Side Effect]; cancer risk; cardiovascular disease/si [Side Effect]; cellulitis/si [Side Effect]; cholesterol blood level; colitis/dt [Drug Therapy]; congestive heart failure/si [Side Effect]; Crohn disease/dt [Drug Therapy]; diabetic foot/si [Side Effect]; disease exacerbation/si [Side Effect]; drug dose comparison; drug efficacy; drug fatality/si [Side Effect]; drug mechanism; drug safety; drug tolerability; drug withdrawal; dyslipidemia/si [Side Effect]; enzyme analysis; enzyme deficiency; enzyme inhibition; genetic association; graft rejection/dt [Drug Therapy]; herpes zoster/si [Side Effect]; human; infection/si [Side Effect]; infection risk; *inflammatory bowel disease/dt [Drug Therapy]; influenza/si [Side Effect]; lipoprotein blood level; liver abscess/si [Side Effect]; liver toxicity/si [Side Effect]; lung tuberculosis/si [Side Effect]; lymphoma/si [Side Effect]; malignant neoplastic disease/si [Side Effect]; multicenter study (topic); myelofibrosis/dt [Drug Therapy]; nausea/si [Side Effect]; nephrotoxicity/si [Side Effect]; neutropenia/si [Side Effect]; neutrophil count; nonhuman; opportunistic infection/si [Side Effect]; outcome assessment; perianal abscess/si [Side Effect]; phase 1 clinical trial (topic); phase 2 clinical trial(topic); phase 3 clinical trial (topic); pneumonia/si [Side Effect]; protein expression; protein family; psoriasis/dt [Drug Therapy]; pulmonary cryptococcosis/si [Side Effect]; randomized controlled trial (topic); review; rheumatoid arthritis/dt [Drug Therapy]; rhinopharyngitis/si [Side Effect]; sepsis/si [Side Effect]; side effect/si [Side Effect]; signal transduction; single nucleotide polymorphism; solid tumor/si [Side Effect]; treatment duration; tuberculous pleurisy/si [Side Effect]; ulcerative colitis/dt [Drug Therapy]; vomiting/si [Side Effect]; vulva abscess/si [Side Effect]; vulva disease/si [Side Effect]; adalimumab/cb [Drug Combination]; adalimumab/cm [Drug Comparison]; adalimumab/dt [Drug Therapy]; baricitinib/dt [Drug Therapy]; baricitinib/pd [Pharmacology]; certolizumab pegol/ct [Clinical Trial]; certolizumab pegol/dt [Drug Therapy]; cytokine receptor/ec [Endogenous Compound]; cytokine receptor 1/ec [Endogenous Compound]; cytokine receptor 2/ec [Endogenous Compound]; decernotinib/ct [Clinical Trial]; decernotinib/dt [Drug Therapy]; decernotinib/pd [Pharmacology]; disease modifying antirheumatic drug/cb [Drug Combination]; disease modifying antirheumatic drug/dt [Drug Therapy]; glpg 0634/ct [Clinical Trial]; glpg 0634/dt [Drug Therapy]; glpg 0634/pd [Pharmacology]; glpg 0974/ct [Clinical Trial]; glpg 0974/dt [Drug Therapy]; glpg 0974/po [Oral Drug Administration]; glpg 0974/pd [Pharmacology]; high density lipoprotein/ec [Endogenous Compound]; Janus kinase 1/ec [Endogenous Compound]; Janus kinase 2/ec [Endogenous Compound]; Janus kinase 3/ec [Endogenous Compound]; *Janus kinase inhibitor/dt [Drug Therapy]; jnj 54781532/ct [Clinical Trial]; jnj 54781532/dt [Drug Therapy]; jnj 54781532/pd [Pharmacology]; low density lipoprotein cholesterol/ec [Endogenous Compound]; methotrexate/cb [Drug Combination]; methotrexate/dt [Drug Therapy]; peficitinib/pd [Pharmacology]; placebo; ruxolitinib/ct [Clinical Trial]; ruxolitinib/cm [Drug Comparison]; ruxolitinib/dt [Drug Therapy]; ruxolitinib/pd [Pharmacology]; STAT protein/ec [Endogenous Compound]; tofacitinib/ae [Adverse Drug Reaction]; tofacitinib/ct [Clinical Trial]; tofacitinib/cb [Drug Combination]; tofacitinib/cm [Drug Comparison]; tofacitinib/do [Drug Dose]; tofacitinib/dt [Drug Therapy]; tofacitinib/pd [Pharmacology]; unclassified drug.

Drug Trade Names and Manufacturers

AbbVie, Astellas, Galapagos, Incyte, Janssen, Lilly, Vertex, asp 015k: Astellas, cp 690550, glpg 0634: AbbVie, glpg 0634: Galapagos, glpg 0974: Galapagos, incb 028050, jnj 54781532: Janssen, vx 509: Vertex

Link to the Ovid Full Text or citation:

Click here for full text options

99.

Hepatocellular carcinoma: Clinicopathological profile and challenges of management in a resource-limited setting.

Jaka H., Mshana S.E., Rambau P.F., Masalu N., Chalya P.L., Kalluvya S.E.

World Journal of Surgical Oncology. 12 (1) (no pagination), 2014. Article Number: 246. Date of Publication: 02 Aug 2014.

AN: 2014521484

Background: Hepatocellular carcinoma is one of the most common cancers worldwide and its incidence is reported to be increasing in resource-limited countries. There is a paucity of published data regarding hepatocellular carcinoma in Tanzania, and the study area in particular. This study describes the clinicopathological profile of hepatocellular carcinoma in our local setting and highlights the challenging problems in the management of this disease.Methods: This was a retrospective study of histopathologically confirmed cases of hepatocellular carcinoma seen at Bugando Medical Center between March 2009 and February 2013.Results: A total of 142 patients (M: F = 2.2: 1) were studied representing 4.6% of all malignancies. The median age of patients was 45 years. Hepatitis B virus infection (66.2%) and heavy alcohol consumption (60.6%) were the most frequently identified risk factors for hepatocellular carcinoma. The majority of patients (88.0%) presented late with advanced stages. HBsAg was positive in 66.2% of the patients and Hepatitis C Virus antibody in 16.9%. Thirteen (9.2%) patients tested positive for HIV infection. Most patients (52.8%) had both right and left lobe involvement. The trabecular pattern (47.9%) was the most frequent histopathological type. None of patients had curative therapy because of the advanced nature of the disease. Coagulopathy (45.7%) was the most common complications. The overall mortality rate was 46.5% and it was significantly associated with comorbidity, HIV positivity, CD4+ count <200 cells/mul, high histological grade, advanced stage of the tumor, presence of distant metastases at the time of diagnosis, and associated complications (P < 0.001). The overall median duration of hospital stay was 14 days. The majority of patients (71.1%) were lost to follow-up at the end of the follow-up period.Conclusions: Hepatocellular carcinoma patients in this region are relatively young at diagnosis and the majority of them present late with an advanced stage and high rate of distant metastasis. Lack of awareness of the disease, poor accessibility to healthcare facilities, and lack of screening programs in this region may contribute to advanced disease at the time of diagnosis. There is a need for early detection, adequate treatment, and proper follow-up to improve treatment outcome. © 2014 Jaka et al.

Institution

(Jaka, Kalluvya) Department of Internal Medicine, Catholic University of Health and Allied Sciences- Bugando, P.O. Box 1464, Mwanza, Tanzania (Mshana) Department of Microbiology and Immunology, Catholic University of Health and Allied Sciences- Bugando, P.O. Box 1464, Mwanza, Tanzania

(Rambau) Department of Pathology, Catholic University of Health and Allied Sciences- Bugando, Bugando, P.O. Box 1464, Mwanza, Tanzania

(Masalu) Department of Oncology, Catholic University of Health and Allied Sciences- Bugando, Bugando, P.O. Box 1464, Mwanza, Tanzania

(Chalya) Department of Surgery, Catholic University of Health and Allied Sciences- Bugando, Bugando, P.O. Box 1464, Mwanza, Tanzania

Emtree Heading

adult; alcohol consumption; article; bacterial peritonitis/co [Complication]; blood clotting disorder; blood transfusion; cancer mortality; cause of death; CD4 lymphocyte count; chronic kidney failure; comorbidity; diabetes mellitus; distant metastasis; female; follow up; health care facility; hemoperitoneum; hemorrhagic shock; hepatic encephalopathy/co [Complication]; Hepatitis B virus; hepatorenal syndrome/co [Complication]; histopathology; human; Human immunodeficiency virus infection; hypertension; length of stay; *liver cell carcinoma/dm [Disease Management]; liver failure; lung metastasis; major clinical study; male; mixed infection; retrospective study; risk factor; treatment outcome; tuberculosis; tumor bleeding; upper gastrointestinal bleeding/co [Complication]; aflatoxin B1; hepatitis B surface antigen/ec [Endogenous Compound]; hepatitis C antibody/ec [Endogenous Compound].

Link to the Ovid Full Text or citation:

Click here for full text options

100.

Factors associated with tuberculosis by HIV status in the Brazilian national surveillance system: A cross sectional study.

do Prado T.N., Miranda A.E., de Souza F.M., dos Santos Dias E., Sousa L.K.F., Arakaki-Sanchez D., Sanchez M.N., Golub J.E., Maciel E.L.

BMC Infectious Diseases. 14 (1) (no pagination), 2014. Article Number: 415. Date of Publication: 28 Jul 2014.

AN: 2014521163

Background: Over the last decade tuberculosis (TB) incidence and mortality in Brazil have been steadily declining. However, this downward trend has not been observed among HIV-infected patients. We describe the epidemiological and clinical profile of TB patients by HIV status using the Brazilian National Surveillance System.Methods: All TB diagnoses with HIV status information between January 1, 2007 and December 31, 2011 were categorized as either HIV or non-HIV at time of TB diagnosis. Co-infected patients (TB-HIV) were compared to TB patients with no HIV-infection using a hierarchical logistic regression model using Stata 13.0.Results: The prevalence of TB-HIV co-infection was 19% among adults > 15 years of age. We analyzed data from 243,676 individuals, of whom 46,466 were TB-HIV and 197,210 were only TB cases. The following factors increased risk of co-infection: male sex (OR: 1.06, 95% CI 1.03-1.10), 20 to 39 years of age (OR = 4.82, 95% CI 4.34-5.36), black (OR = 1.08, 95% CI 1.04-1.13), 4-7 years of education (OR = 1.13, 95% CI 1.19-1.28), diagnosed following default (OR = 2.65, 95% CI 1.13-6.25), presenting with pulmonary and extra-pulmonary forms of TB simultaneously (OR = 2.80, 95% CI 1.56-5.02), presenting with histopathologic examination suggestive of TB (OR = 2.15, 95% CI 1.13-4.07). Co-infected patients were less likely to live in rural areas (OR = 0.45, 95% CI 0.42-0.48), have diabetes (OR = 0.45, 95% CI 0.40-0.50) and be smear positive (OR = 0.55, 95% CI 0.32-0.95), and co-infected patients had higher risk of default (OR = 2.96, 95% CI 2.36-3.71) and death from TB (OR = 5.16, 95% CI 43.04-5.77).Conclusions: The prevalence of co-infection with HIV among TB patients is 19% in Brazil. By identifying predictors of co-infection targeted interventions can be developed to prevent both TB and HIV, and to diagnose each disease earlier and ultimately decrease poor treatment outcomes and death. © 2014 do Prado et al.; licensee BioMed Central Ltd.

Institution

(do Prado, de Souza, dos Santos Dias, Sousa, Maciel) Lab-Epi UFES - Laboratorio de Epidemiologia, Universidade Federal do Espirito Santo, Av. Marechal Campos, 1468- Maruipe, Vitoria, ES, Brazil (do Prado, dos Santos Dias, Sousa, Maciel) School of Nursing, Federal University of Espirito Santo, Espirito Santo, Brazil

(do Prado, Miranda, Maciel) Post-Graduate Program in Infectious Diseases, Federal University of Espirito Santo, Espirito Santo, Brazil

(Miranda, Maciel) Post-Graduate Program in Public Health, Federal University of Espirito Santo, Espirito Santo, Brazil

(Arakaki-Sanchez) TB National Consultant, Pan American Health Organization, Sao Paulo, Brazil

(Sanchez) Department of Public Health, University of Brasilia, Federal District, Brazil

(Golub) Center for Tuberculosis Research, Johns Hopkins University School of Medicine, Baltimore, MD, United States

Emtree Heading

adolescent; adult; article; Black person; Brazilian; controlled study; cross-sectional study; diabetes mellitus; disease association; educational status; ethnic group; extrapulmonary tuberculosis/di [Diagnosis]; female; *health status; health survey; high risk patient; histopathology; human; *Human immunodeficiency virus infection; human tissue; logistic regression analysis; lung tuberculosis/di [Diagnosis]; major clinical study; male; mixed infection; mortality; outcome assessment; prevalence; rural area; smear; symptomatology; *tuberculosis/di [Diagnosis]; *tuberculosis/ep [Epidemiology].

Link to the Ovid Full Text or citation:

Click here for full text options
