# Supplementary material for: Effect of glycemic control and type of diabetes treatment on TB treatment outcomes among people with TB-diabetes: A systematic review (updated August 2024)
Source: PLoS One. 2025 Jul 18;20(7):e0328619. doi: 10.1371/journal.pone.0328619 (PMC12273911; doi:10.1371/journal.pone.0328619)
Supplement: S1 Appendix — (ZIP) [file pone.0328619.s004.zip › S1 appendix_old/EMBASE/298-2_with RISK search reuslts no 15.doc]

Database: Embase <1996 to 2016 September 16>

Search Strategy:

--------------------------------------------------------------------------------

1	tuberculosis.mp. or exp tuberculosis/ (151164)

2	exp treatment outcome/ or treatment outcome*.mp. (1189741)

3	diabetes mellitus.mp. or exp diabetes mellitus/ (627157)

4	diabetes mellitus/ (362441)

5	DM.mp. (48079)

6	diabetes.mp. (663193)

7	mellitus.mp. (588341)

8	3 or 4 or 5 or 6 or 7 (715025)

9	1 and 2 and 8 (724)

10	cohort studies.mp. or cohort analysis/ (263408)

11	exp risk factor/ or cohort.mp. or exp risk/ (2148599)

12	10 or 11 (2148599)

13	9 and 10 (81)

14	9 and 12 (304)

15	limit 14 to human (298)

16	limit 13 to human (81)

17	trial*.mp. or exp controlled clinical trial/ or exp "clinical trial (topic)"/ or exp "randomized controlled trial (topic)"/ or exp "controlled clinical trial (topic)"/ (1555047)

18	random.ab. (191735)

19	randomized controlled trial.pt. (0)

20	controlled clinical trial.pt. (0)

21	randomized.ab. (454621)

22	placebo.ab. (188623)

23	clinical trials as topic.sh. (15)

24	trial.ti. (176210)

25	17 or 18 or 19 or 20 or 21 or 22 or 23 or 24 (1831290)

26	9 and 25 (128)

27	limit 26 to human (128)

***************************

101.

Treatment outcome of new smear positive pulmonary tuberculosis patients in Penang, Malaysia.

Atif M., Sulaiman S.A.S., Shafie A.A., Ali I., Asif M., Babar Z.-U.-D.

BMC Infectious Diseases. 14 (1) (no pagination), 2014. Article Number: 399. Date of Publication: 19 Mar 2014.

AN: 2014498044

Background: According to the World Health Organization's recent report, in Malaysia, tuberculosis (TB) treatment success rate for new smear positive pulmonary tuberculosis (PTB) patients is still below the global success target of 85%. In this study, we evaluated TB treatment outcome among new smear positive PTB patients, and identified the predictors of unsuccessful treatment outcome and longer duration of treatment (i.e., > 6 months).Methods: The population in this study consisted of all new smear positive PTB patients who were diagnosed at the chest clinic of Penang General Hospital between March 2010 and February 2011. During the study period, a standardized data collection form was used to obtain socio-demographic, clinical and treatment related data of the patients from their medical charts and TB notification forms (Tuberculosis Information System; TBIS). These data sources were reviewed at the time of the diagnosis of the patients and then at the subsequent follow-up visits until their final treatment outcomes were available. The treatment outcomes of the patients were reported in line with six outcome categories recommended by World Health Organization. Multiple logistic regression analysis was used to find the independent risk factors for unsuccessful treatment outcome and longer treatment duration. Data were analyzed using the PASW (Predictive Analysis SoftWare, version 19.0. Armonk, NY: IBM Corp).Results: Among the 336 PTB patients (236 male and 100 female) notified during the study period, the treatment success rate was 67.26% (n = 226). Out of 110 patients in unsuccessful outcome category, 30 defaulted from the treatment, 59 died and 21 were transferred to other health care facilities. The mean duration of TB treatment was 8.19 (SD 1.65) months. In multiple logistic regression analysis, risk factors for unsuccessful treatment outcome were foreign nationality, male gender and being illiterate. Similarly, risk factors for mortality due to TB included high-grade sputum and presence of lung cavities at the start of treatment, being alcoholic and elderly. Likewise, concurrent diabetes, presence of lung cavities at the start of the treatment and being a smoker were the significant predictors of longer treatment duration.Conclusion: Our findings indicated that the treatment success rate among the new smear positive PTB patients was less than the success target set by World Health Organization. The proportion of patients in the successful outcome category may be increased by closely monitoring the treatment progress of the patients with aforementioned high risk characteristics. Similarly, more aggressive follow-up of the treatment defaulters and transferred out patients could also improve the TB treatment success rate. © 2014 Atif et al.; licensee BioMed Central Ltd.

Institution

(Atif, Sulaiman) Discipline of Clinical Pharmacy, School of Pharmaceutical Sciences, Universiti Sains Malaysia, Penang, Malaysia (Atif) Department of Pharmacy, The Islamia University of Bahawalpur, Punjab, Pakistan

(Shafie) Discipline of Social and Administrative Pharmacy, School of Pharmaceutical Sciences, Universiti Sains Malaysia, Penang, Malaysia

(Ali) Respiratory Department, Penang General Hospital, Penang, Malaysia

(Asif) Department of Pharmacology, School of Pharmaceutical Sciences, Universiti Sains Malaysia, Penang, Malaysia

(Babar) Division of Pharmacy Practice, School of Pharmacy, University of Auckland, Auckland, New Zealand

Publisher

BioMed Central Ltd.

Emtree Heading

adult; aged; article; cause of death; controlled study; disease association; female; human; lung cavity; *lung tuberculosis; major clinical study; Malaysia; male; middle aged; mortality; outcome assessment; patient assessment; prediction; risk assessment; risk factor; sputum smear; treatment duration; treatment outcome; young adult.

Link to the Ovid Full Text or citation:

Click here for full text options

102.

Infection risk in patients with rheumatoid arthritis treated with etanercept or adalimumab.

Chiang Y.-C., Kuo L.-N., Yen Y.-H., Tang C.-H., Chen H.-Y.

Computer Methods and Programs in Biomedicine. 116 (3) (pp 319-327), 2014. Date of Publication: October 2014.

AN: 2014482167

Objectives: To compare the risk of infection for rheumatoid arthritis (RA) patients who took etanercept or adalimumab medication in a nationwide population. Methods: RA patients who took etanercept or adalimumab were identified in the Taiwan's National Health Insurance Research Database. The composite outcome of serious infections, including hospitalization for infection, reception of an antimicrobial injection, and tuberculosis were followed for 365 days. A Kaplan-Meier survival curve with a log-rank test and Cox proportional hazards regression were used to compare risks of infection between the two cohorts of tumor necrosis factor (TNF)-alpha antagonists users. Hazard ratios (HRs) were obtained and adjusted with propensity scores and clinical factors. Sensitivity analyses and subgroup analyses were also performed. Results: In total, 1660 incident etanercept users and 484 incident adalimumab users were eligible for the analysis. The unadjusted HR for infection of the etanercept users was significantly higher than that of the adalimumab users (HR: 1.93; 95% confidence interval (CI): 1.09-3.42; p= 0.024). The HRs were 2.04 (95% CI: 1.14-3.65; p= 0.016) and 2.02 (95% CI: 1.13-3.61; p= 0.018) after adjusting for propensity scores and for propensity scores in addition to clinical factors, respectively. The subgroup analyses revealed that HRs for composite infection was significantly higher in patient subgroups of older age, female, as well as patients who did not have DM, COPD, and hospitalization history at the baseline. Conclusion: In this head-to-head cohort study involving a nationwide population of patients with RA, etanercept users demonstrated a higher risk of infection than adalimumab users. Results of this study suggest the possible existence of an intra-class difference in infection risk among TNF-alpha antagonists. © 2014 Elsevier Ireland Ltd.

Institution

(Chiang, Kuo, Yen, Chen) Department of Pharmacy, Wan Fang Hospital, Taipei Medical University, Taipei, Taiwan (Republic of China) (Chiang, Kuo, Yen, Chen) School of Pharmacy, Taipei Medical University, Taipei, Taiwan (Republic of China)

(Tang) School of Health Care Administration, Taipei Medical University, Taipei, Taiwan (Republic of China)

Publisher

Elsevier Ireland Ltd

Emtree Heading

adult; article; female; high risk patient; hospitalization; human; *infection/et [Etiology]; *infection/si [Side Effect]; infection risk; Kaplan Meier method; major clinical study; male; outcome assessment; propensity score; *rheumatoid arthritis/dt [Drug Therapy]; Taiwan; *adalimumab/ae [Adverse Drug Reaction]; *adalimumab/dt [Drug Therapy]; *etanercept/ae [Adverse Drug Reaction]; *etanercept/dt [Drug Therapy]; tumor necrosis factor inhibitor/ae [Adverse Drug Reaction]; tumor necrosis factor inhibitor/dt [Drug Therapy].

Link to the Ovid Full Text or citation:

Click here for full text options

103.

Predictors of pulmonary tuberculosis treatment outcomes in South Korea: A prospective cohort study, 2005-2012.

Choi H., Lee M., Chen R.Y., Kim Y., Yoon S., Joh J.S., Park S.K., Dodd L.E., Lee J., Song T., Cai Y., Goldfeder L.C., Via L.E., Carroll M.W., Barry III C.E., Cho S.-N.

BMC Infectious Diseases. 14 (1) (no pagination), 2014. Article Number: 360. Date of Publication: 02 Jul 2014.

AN: 2014465794

Background: Tuberculosis remains an important health concern in many countries. The aim of this study was to identify predictors of unfavorable outcomes at the end of treatment (EOT) and at the end of study (EOS; 40 months after EOT) in South Korea.Methods: New or previously treated tuberculosis patients were recruited into a prospective observational cohort study at two hospitals in South Korea. To identify predictors of unfavorable outcomes at EOT and EOS, logistic regression analysis was performed.Results: The proportion of multidrug-resistant tuberculosis (MDR-TB) was 8.2% in new cases and 57.9% in previously treated cases. Of new cases, 68.6% were cured, as were 40.7% of previously treated cases. At EOT, diabetes, >3 previous TB episodes, >1 significant regimen change, and MDR-TB were significantly associated with treatment failure or death. At EOS, age >35, body-mass index (BMI) <18.5, diabetes, and MDR-TB were significantly associated with treatment failure, death, or relapse. Among cases that were cured at EOT, age >50 and a BMI <18.5 were associated with subsequent death or relapse during follow-up to EOS. Treatment interruption was associated with service sector employees or laborers, bilateral lesions on chest X-ray, and previous treatment failure or treatment interruption history.Conclusions: Risk factors for poor treatment outcomes at EOT and EOS include both patient factors (diabetes status, age, BMI) and disease factors (history of multiple previous treatment episodes, MDR-TB). In this longitudinal, observational cohort study, diabetes mellitus and MDR-TB were risk factors for poor treatment outcomes and relapse. Measures to help ensure that the first tuberculosis treatment episode is also the last one may improve treatment outcomes.Trial registration: ClinicalTrials.gov ID: NCT00341601. © 2014 Choi et al.; licensee BioMed Central Ltd.

Institution

(Choi, Lee, Kim, Yoon, Lee, Song, Cho) International Tuberculosis Research Center, Changwon, South Korea (Choi) BK21PLUS Program in Public Health Sciences, Department of Health Sciences, Graduate School, Korea University, Seoul, South Korea

(Chen, Cai, Goldfeder, Via, Carroll, Barry III) Tuberculosis Research Section, Laboratory of Clinical Infectious Disease, National Institute of Allergy and Infectious Diseases (NIAID), National Institutes of Health (NIH), Bethesda, Maryland, United States

(Joh) Department of Internal Medicine, National Medical Center, Seoul, South Korea

(Park) National Masan Tuberculosis Hospital, Changwon, South Korea

(Dodd) Biostatistics Research Branch, NIAID, NIH, Bethesda, Maryland, United States

(Cho) Department of Microbiology and Institute for Immunology, Immunological Diseases, Yonsei University College of Medicine, Brain Korea 21 Project for Medical Science, Seoul, South Korea

Publisher

BioMed Central Ltd.

Emtree Heading

adult; article; body mass; cohort analysis; controlled study; death; diabetes mellitus; female; follow up; human; *lung tuberculosis; major clinical study; male; multidrug resistant tuberculosis; observational study; prediction; prognosis; prospective study; relapse; South Korea; thorax radiography; treatment failure; *treatment outcome; treatment withdrawal.

Link to the Ovid Full Text or citation:

Click here for full text options

104.

Is there an effect of glucose lowering treatment on incidence and prognosis of tuberculosis? A systematic review.

Jorgensen M.E., Faurholt-Jepsen D.

Current Diabetes Reports. 14 (7) (no pagination), 2014. Article Number: 505. Date of Publication: July 2014.

AN: 2014443596

The association between diabetes and incidence of tuberculosis is well established, and observational studies have shown poor treatment outcome in tuberculosis related to hyperglycemia. The WHO recommends screening for diabetes among all patients with tuberculosis and optimized glycemic control aiming at improving tuberculosis outcome. However, no intervention studies support this notion. Patients with tuberculosis are often vulnerable with high degree of comorbidity, and, therefore, at high risk of adverse effects of intensive glucose control. Controlled intervention studies of the effect of glucose lowering treatment on tuberculosis outcomes are clearly warranted to justify screening for- and tight control of diabetes. © 2014 Springer Science+Business Media New York.

Institution

(Jorgensen) Steno Diabetes Center, Niels Steensensvej 2, DK-2820 Gentofte, Denmark (Faurholt-Jepsen) Department of Nutrition, Exercise, and Sports, University of Copenhagen, Frederiksberg, Denmark

(Faurholt-Jepsen) Department of Infectious Diseases, Rigshospitalet, Copenhagen, Denmark

Publisher

Current Medicine Group LLC 1

Emtree Heading

area under the curve; article; clinical trial (topic); comorbidity; diabetes control; *diabetes mellitus/dt [Drug Therapy]; diabetic patient; disease association; drug bioavailability; drug efficacy; drug potentiation; drug safety; drug treatment failure; Embase; glucose blood level; glycemic control; high risk patient; human; *hyperglycemia; incidence; insulin resistance; Medical Subject Headings; Medline; multidrug resistant tuberculosis; observational study; prevalence; prognosis; screening test; sputum culture; systematic review; thorax radiography; treatment outcome; *tuberculosis; antidiabetic agent/dt [Drug Therapy]; ethambutol; *glucose/ec [Endogenous Compound]; insulin; isoniazid; placebo; pyrazinamide; rifampicin; saxagliptin.

Link to the Ovid Full Text or citation:

Click here for full text options

105.

Does alcohol consumption during multidrug-resistant tuberculosis treatment affect outcome? A population-based study in Kerala, India.

Duraisamy K., Mrithyunjayan S., Ghosh S., Nair S.A., Balakrishnan S., Subramoniapillai J., Oeltmann J.E., Moonan P.K., Kumar A.M.V.

Annals of the American Thoracic Society. 11 (5) (pp 712-715), 2014. Date of Publication: June 2014.

AN: 2014444288

Rationale: India reports the largest number of multidrug-resistant tuberculosis cases in the world; yet, no longitudinal study has assessed factors related to treatment outcomes under programmatic conditions in the public sector. Objectives: To describe demographic, clinical, and risk characteristics associated with treatment outcomes for all patients with multidrug-resistant tuberculosis registered in the Revised National Tuberculosis Control Programme, Kerala State, India from January 1, 2009 to June 30, 2010. Methods: Cox regression methods were used to calculate adjusted hazard ratios with 95% confidence intervals (CIs) to assess factors associated with an unsuccessful treatment outcome. Measurements and Main Results: Of 179 patients with multidrug-resistant tuberculosis registered, 112 (63%) had successful treatment outcomes (77 bacteriologically cured, 35 treatment completed) and 67 (37%) had unsuccessful treatment outcomes (30 died, 26 defaulted, 9 failed treatment, 1 stopped treatment because of drug-related adverse events, and 1 developed extensively drug-resistant tuberculosis). The hazard for unsuccessful outcome was significantly higher among patients who consumed alcohol during treatment (adjusted hazard ratio, 4.3; 95% CI, 1.1-17.6) than those who did not. Persons who consumed alcohol during treatment, on average, missed 18 more intensive-phase doses (95% CI, 13-22) than those who did not. Although many patients had diabetes (33%), were ever smokers (39%), or had low body mass index (47%), these factors were not associated with outcome. Conclusion: Overall treatment success was greater than global and national averages; however, outcomes among patients consuming alcohol remained poor. Integration of care for multidrug-resistant tuberculosis and alcoholism should be considered to improve treatment adherence and outcomes. Copyright © 2014 by the American Thoracic Society.

Institution

(Duraisamy, Nair, Balakrishnan) Department of Tuberculosis, WHO Country Office for India, New Delhi 110029, India (Mrithyunjayan, Subramoniapillai) State TB Cell, Direct. of Health Services, Thiruvananthapuram, Kerala, India

(Ghosh, Oeltmann, Moonan) U.S. Centers for Disease Control and Prevention, Division of TB Elimination, Atlanta, GA, United States

(Kumar) International Union Against Tuberculosis and Lung Disease (The Union), South-East Asia Regional Office, New Delhi, India

Publisher

American Thoracic Society

Emtree Heading

adolescent; adult; *alcohol consumption; antibiotic sensitivity; arthralgia/si [Side Effect]; article; body mass; cohort analysis; controlled study; demography; diabetes mellitus; drinking behavior; drug treatment failure; extensively drug resistant tuberculosis/di [Diagnosis]; extensively drug resistant tuberculosis/dt [Drug Therapy]; female; follow up; gastrointestinal symptom/si [Side Effect]; health program; human; India; jaundice/si [Side Effect]; major clinical study; male; *multidrug resistant tuberculosis/di [Diagnosis]; *multidrug resistant tuberculosis/dt [Drug Therapy]; *multidrug resistant tuberculosis/ep [Epidemiology]; Mycobacterium tuberculosis; nephrotoxicity/si [Side Effect]; neuropathy/si [Side Effect]; obesity; ototoxicity/si [Side Effect]; outcome assessment; population research; retrospective study; smoking; suicidal ideation/si [Side Effect]; tuberculosis control; *alcohol; cycloserine/ae [Adverse Drug Reaction]; cycloserine/dt [Drug Therapy]; ethambutol/ae [Adverse Drug Reaction]; ethambutol/dt [Drug Therapy]; ethionamide/ae [Adverse Drug Reaction]; ethionamide/dt [Drug Therapy]; isoniazid/ae [Adverse Drug Reaction]; isoniazid/dt [Drug Therapy]; kanamycin/ae [Adverse Drug Reaction]; kanamycin/dt [Drug Therapy]; levofloxacin/ae [Adverse Drug Reaction]; levofloxacin/dt [Drug Therapy]; ofloxacin/ae [Adverse Drug Reaction]; ofloxacin/dt [Drug Therapy]; pyrazinamide/ae [Adverse Drug Reaction]; pyrazinamide/dt [Drug Therapy]; rifampicin/ae [Adverse Drug Reaction]; rifampicin/dt [Drug Therapy]; streptomycin/ae [Adverse Drug Reaction]; streptomycin/dt [Drug Therapy].

Link to the Ovid Full Text or citation:

Click here for full text options

106.

Spinal tuberculosis: A retrospective chart review.

Sezgi C., Taylan M., Kaya H., Sen H.S., Abakay O., Bulut M., Abakay A.

Acta Medica Mediterranea. 30 (3) (pp 725-730), 2014. Date of Publication: 2014.

AN: 2014424239

Aims: Background and objectives: This study aimed to investigate the clinical characteristics, diagnostic methods, and therapeutic outcomes in patients with spinal tuberculosis (TB) in Southeastern Turkey Methods: Patients diagnosed with spinal TB at Dicle University Hospital, Diyarbakir, Turkey, between October 2005 to December 2010 were enrolled in the study retrospectively. Patients were evaluated for the following: Clinical presentation, underlying diseases, laboratory results, imaging findings, medical therapies, and treatment outcomes Results: A total of 23 patients including 14 men and 9 women with spinal TB, also known as Pott's disease, were enrolled. The study subjects' ages ranged from 17 to 69 years with a mean age of 38.4 years. Patients most often presented with back pain (69.5%, n=16) and fever (56.5%, n=13), and the most frequent physical finding was spinal tenderness (91.3%). Only 17.4% of the patients demonstrated neurological deficits and no paraplegia was observed. The time interval between onset of symptoms and treatment initiation was 3.2 months. Of all the patients, 47.8% exhibited Pott's disease in the thoracolumbar region while 34.8% only had thoracic involvement. On average, 2.5 vertebrae were diseased in this cohort. Furthermore, 82.6% of the patients had paraspinal and psoas abscesses. All patients were treated with anti-TB therapy. However, 26.1% required additional surgery, and one patient with miliary TB died. The mean treatment course lasted for 12.3 months. Conclusions: It may be possible to refine methods of spinal TB detection and diagnosis by studying thoracolumbar pathology in young adults with Pott's disease in endemic countries such as Turkey. By studying the clinical progression of this disease, the delay between symptom onset and diagnosis may be decreased so that complications such as paraplegia and surgical interventions are avoided.

Institution

(Sezgi, Taylan, Kaya, Sen, Abakay, Abakay) Dicle University School of Medicine, Department of Pulmonary Diseases, Diyarbakir, Turkey (Bulut) Dicle University School of Medicine, Department of Orthopedy, Diyarbakir, Turkey

Publisher

A. CARBONE Editore

Emtree Heading

adult; article; backache; cause of death; chronic kidney failure; clinical article; clinical feature; computer assisted tomography; erythrocyte sedimentation rate; female; fever; hepatitis B; human; leukocyte count; limb weakness; male; medical record review; non insulin dependent diabetes mellitus; nuclear magnetic resonance imaging; outcome assessment; paraplegia; paraspinal abscess; paresthesia; psoas abscess; retrospective study; spinal cord compression/su [Surgery]; spinal tenderness; spine disease; spine instability/su [Surgery]; spine malformation/su [Surgery]; spine surgery; thoracic spine; thoracolumbar spine; time; treatment duration; *tuberculous spondylitis/di [Diagnosis]; *tuberculous spondylitis/dt [Drug Therapy]; *tuberculous spondylitis/su [Surgery]; Turkey (republic); unspecified side effect/si [Side Effect]; X ray; C reactive protein/ec [Endogenous Compound]; ethambutol/ae [Adverse Drug Reaction]; ethambutol/dt [Drug Therapy]; isoniazid/ae [Adverse Drug Reaction]; isoniazid/dt [Drug Therapy]; pyrazinamide/ae [Adverse Drug Reaction]; pyrazinamide/dt [Drug Therapy]; rifampicin/ae [Adverse Drug Reaction]; rifampicin/dt [Drug Therapy]; streptomycin/ae [Adverse Drug Reaction]; streptomycin/dt [Drug Therapy].

Link to the Ovid Full Text or citation:

Click here for full text options

107.

Cohort analysis of antenatal care and delivery outcomes in pregnancy: A basis for improving maternal health.

Harries A.D., Jahn A., Ben-Smith A., Gadabu O.J., Douglas G.P., Seita A., Khader A., Zachariah R.

Public Health Action. 4 (2) (pp 75-78), 2014. Date of Publication: 01 Jun 2014.

AN: 2014421981

Cohort analysis has been the cornerstone of tuberculosis (TB) monitoring and evaluation for nearly two decades; these principles have been adapted for patients with the human immunodeficiency virus/acquired immune-deficiency syndrome on antiretroviral treatment and patients with diabetes mellitus and hypertension. We now make the case for using cohort analyses for monitoring pregnant women during antenatal care, up to and including childbirth. We believe that this approach would strengthen the current monitoring and evaluation systems used in antenatal care by providing more precise information at regular time intervals. Accurate real-time data on how many pregnant women are enrolled in antenatal care, their characteristics, the interventions they are receiving and the outcomes for mother and child should provide a solid basis for action to reduce maternal mortality. © 2014 The Union.

Institution

(Harries) International Union Against Tuberculosis and Lung Disease, Paris, France (Harries) London School of Hygiene and Tropical Medicine, London, United Kingdom

(Jahn) Department for HIV and AIDS, Ministry of Health, Lilongwe, Malawi

(Jahn) International Training and Education Centre for Health, Malawi

(Jahn) University of Washington, Seattle, WA, United States

(Ben-Smith) Department of Biomedical Informatics, University of Pittsburgh, Pittsburgh, PA, United States

(Gadabu) Baobab Health Trust, Lilongwe, Malawi

(Douglas) Center for Health Informatics for the Underserved, University of Pittsburgh, Pittsburgh, PA, United States

(Seita, Khader) United Nations Relief and Works Agency for Palestine Refugees in the Near East, UNRWA Headquarters, Amman, Jordan

(Zachariah) Medecins Sans Frontieres, MSF, Medical Department, Brussels Operational Centre, Luxembourg, Luxembourg

Publisher

International Union Against Tuberculosis and Lung Disease (The Union) (68 boulevard Saint Michel, Paris 75006, France)

Emtree Heading

article; clinical evaluation; cohort analysis; diabetes mellitus; female; follow up; highly active antiretroviral therapy; human; Human immunodeficiency virus infection; hypertension; maternal mortality; *maternal welfare; patient care; *pregnancy outcome; *prenatal care; public health; risk factor; treatment outcome; tuberculosis.

Link to the Ovid Full Text or citation:

Click here for full text options

108.

Predictors of non-traumatic coma in a pediatric cohort from a South Indian tertiary care center: Results of a multivariate analysis.

Gowda V.K.N., Bannigidad N.B., Kumar P., Srikanteswara P.-K., Shivananda, Govindraj, Vykuntaraju S.H., Ramaswamy P.

Journal of Pediatric Neurology. 12 (1) (pp 35-43), 2014. Date of Publication: 2014.

AN: 2014381538

Objective: To study the etiology and clinical profile of non-traumatic coma in children at tertiary care center and to determine the predictors of outcome. Methods: One hundred and four consecutive children between 2 mo-12 yr were studied. The clinical signs at admission; vital signs, Glasgow coma scale, respiratory pattern, papillary reflex, extra-ocular movements, fundus picture and motor deficits were recorded. Etiology of coma was determined by clinical history, examination and relevant investigations. Their progress was monitored clinically, biochemically and with multi-system monitors. Outcome was recorded as survived or died. Results: Etiology of coma in 65% cases was intracranial infections; other causes were metabolic (20%). Sixty-seven percent recovered completely, 16% had residual neurodeficits, 16% died. Survival was better in children with intracranial infections (13%) as compared to metabolic coma (33%). On multivariate logistic regression, bradycardia, hypotension, abnormal respiratory pattern (especially, ataxic type), duration of coma more than 48 h, Glasgow coma scale < 7 at admission, unequal and non-reactive pupils, papilledema, abnormal extra-ocular movements, motor deficits, signs of meningitis correlated with mortality. Requirement of ventilatory support and abnormal computerized tomography findings correlated with mortality. Conclusions: Intracranial infections were the most common cause of non-traumatic coma in children; the most common cause of death being metabolic coma. Simple clinical signs and relevant investigations served as prognostic indicators of outcome. © 2014-IOS Press.

Institution

(Gowda) Department of Pediatric Neurology, Indira Gandhi Institute of Child Health, Bangalore, India (Bannigidad, Kumar, Shivananda, Govindraj, Ramaswamy) Department of Pediatrics, Indira Gandhi Institute of Child Health, Bangalore, India

(Srikanteswara) Department of Neurology, Bangalore Medical College and Research Institute, Bangalore, India

(Vykuntaraju) Department of Anatomy, Kempegowda Institute of Medical Sciences, Bangalore, India

(Gowda) Bangalore Child Neurology and Rehabilitation Center, HANS Complex, 8/A 1st Main 1st Cross, Vijayanagar, Bangalore 560040, India

Publisher

IOS Press (Nieuwe Hemweg 6B, Amsterdam 1013 BG, Netherlands)

Emtree Heading

abnormal respiratory sound; anterior fontanel; article; assisted ventilation; bacterial meningitis; bradycardia; cause of death; child; *childhood disease/dt [Drug Therapy]; *childhood disease/et [Etiology]; *childhood disease/th [Therapy]; childhood mortality; clinical examination; cohort analysis; *coma/et [Etiology]; *coma/th [Therapy]; computer assisted tomography; controlled study; convulsion; dehydration; diabetic ketoacidosis; epileptic state; extraocular muscle; eye movement; female; fever; Glasgow coma scale; glucose blood level; headache; hemiparesis; hemiplegia; hepatic encephalopathy; hospital admission; human; hyperventilation; hypotension; hypoxia; Indian; intensive care unit; jaundice; loose feces; major clinical study; male; malnutrition; meningitis; organophosphate poisoning/dt [Drug Therapy]; outcome assessment; pallor; papilledema; prognosis; quadriplegia; rash; retrospective study; Reye syndrome; snakebite/dt [Drug Therapy]; sodium blood level; teaching hospital; tendon reflex; tertiary care center; tuberculous meningitis; vital sign; vomiting; glucose/ec [Endogenous Compound]; organophosphorus compound/to [Drug Toxicity]; snake venom antiserum/dt [Drug Therapy]; sodium/ec [Endogenous Compound].

Link to the Ovid Full Text or citation:

Click here for full text options

109.

Tuberculosis, diabetes mellitus, and Malaysia.

Khan A.H.

Archives of Pharmacy Practice. 5 (2) (pp 45-46), 2014. Date of Publication: 2014.

AN: 2014372724

Institution

(Khan) Department of Clinical Pharmacy, School of Pharmaceutical Sciences, Universiti Sains Malaysia, Penang 11800, Malaysia

Publisher

Archives of Global Professionals

Emtree Heading

acquired immune deficiency syndrome; *diabetes mellitus/dm [Disease Management]; diabetic patient; disease predisposition; early diagnosis; editorial; extrapulmonary tuberculosis/co [Complication]; government; health care cost; health care policy; health literacy; human; immigrant; immunity; incidence; infection risk; Malaysia; mortality; poverty; prevalence; risk factor; short course therapy; silicosis; smoking; treatment outcome; *tuberculosis/di [Diagnosis]; *tuberculosis/dm [Disease Management]; *tuberculosis/dt [Drug Therapy]; *tuberculosis/ep [Epidemiology]; tuberculosis control; tuberculostatic agent/dt [Drug Therapy].

Link to the Ovid Full Text or citation:

Click here for full text options

110.

Tobacco smoking, alcohol drinking, diabetes, low body mass index and the risk of self-reported symptoms of active tuberculosis: Individual Participant Data (IPD) meta-analyses of 72,684 individuals in 14 high tuberculosis burden countries.

Patra J., Jha P., Rehm J., Suraweera W.

PLoS ONE. 9 (5) (no pagination), 2014. Article Number: e96433. Date of Publication: 02 May 2014.

AN: 2014330403

Background: The effects of multiple exposures on active tuberculosis (TB) are largely undetermined. We sought to establish a dose-response relationship for smoking, drinking, and body mass index (BMI) and to investigate the independent and joint effects of these and diabetes on the risk of self-reported symptoms of active TB disease. Methods and Findings: We analyzed 14 national studies in 14 high TB-burden countries using self-reports of blood in cough/phlegm and cough lasting > = 3 weeks in the last year as the measures of symptoms of active TB. The random effect estimates of the relative risks (RR) between active TB and smoking, drinking, diabetes, and BMI<18.5 kg/m 2 were reported for each gender. Floating absolute risks were used to examine dyads of exposure. Adjusted for age and education, the risks of active TB were significantly associated with diabetes and BMI<18.5 kg/m2 in both sexes, with ever drinking in men and with ever smoking in women. Stronger dose-response relationships were seen in women than in men for smoking amount, smoking duration and drinking amount but BMI<18.5 kg/m2 showed a stronger dose-response relationship in men. In men, the risks from joint exposures were statistically significant for diabetics with BMI<18.5 kg/m2 (RR = 6.4), diabetics who smoked (RR = 3.8), and diabetics who drank alcohol (RR = 3.2). The risks from joint risk factors were generally larger in women than in men, with statistically significant risks for diabetics with BMI<18.5 kg/m2 (RR = 10.0), diabetics who smoked (RR = 5.4) and women with BMI<18.5 kg/m2 who smoked (RR = 5.0). These risk factors account for 61% of male and 34% of female estimated TB incidents in these 14 countries. Conclusions: Tobacco, alcohol, diabetes, and low BMI are significant individual risk factors but in combination are associated with triple or quadruple the risk of development of recent active TB. These risk factors might help to explain the wide variation in TB across countries. © 2014 Patra et al.

Institution

(Patra, Jha, Suraweera) Centre for Global Health Research, St. Michael's Hospital, Toronto, ON, Canada (Patra, Jha, Rehm) Dalla Lana School of Public Health, University of Toronto, Toronto, ON, Canada

(Rehm) Centre for Addiction and Mental Health, Toronto, ON, Canada

Publisher

Public Library of Science

Emtree Heading

adult; article; *body mass; controlled study; coughing; *diabetes mellitus; disease association; disease duration; dose response; *drinking behavior; female; geographic distribution; human; major clinical study; male; middle aged; outcomes research; pathogenesis; risk factor; self report; sex difference; *smoking; smoking habit; *tuberculosis/et [Etiology]; young adult.

Link to the Ovid Full Text or citation:

Click here for full text options

111.

Impact of underlying diabetes and presence of lung cavities on treatment outcomes in patients with pulmonary tuberculosis.

Nakamura A., Hagiwara E., Hamai J., Taguri M., Terauchi Y.

Diabetic Medicine. 31 (6) (pp 707-713), 2014. Date of Publication: June 2014.

AN: 2014339266

Aims: We investigated the effects of diabetes and the presence of lung cavities on treatment outcomes in patients with pulmonary tuberculosis. Methods: We conducted a retrospective review of the clinical records of all consecutive patients admitted to the Kanagawa Cardiovascular and Respiratory Centre with the diagnosis of pulmonary tuberculosis. The study outcomes examined were time to sputum culture conversion and percentage of patients with sputum culture conversion by the time 2 months of treatment, and these outcomes were compared between patients with and without diabetes. Results: Of the 260 patients enrolled in the study, 69 were diagnosed as having diabetes mellitus, while the remaining 191 did not have diabetes. The percentage of patients with cavities was higher in the patients with diabetes (71.0%) than in those without (45.5%; P = 0.0003). The time to sputum culture conversion was significantly longer in the patients with diabetes than in those without (P = 0.0005), and the percentage of patients with a positive sputum culture at 2 months was higher in the patients with diabetes (43.5%) than in those without (18.8%; P = 0.0001). Multivariate analyses revealed that the presence/absence of lung cavities was a more important determinant of treatment outcomes than the presence/absence of diabetes. Conclusions: The presence of lung cavities was found to be a more important determinant of the treatment outcomes than that of diabetes per se in patients with pulmonary tuberculosis. © 2014 The Authors.

Institution

(Nakamura, Hamai, Terauchi) Department of Endocrinology and Metabolism, Graduate School of Medicine, Yokohama City University, Yokohama, Japan (Hagiwara) Department of Respiratory Medicine, Kanagawa Cardiovascular and Respiratory Centre, Yokohama, Japan

(Taguri) Department of Biostatistics and Epidemiology, Yokohama City University, Yokohama, Japan

Publisher

Blackwell Publishing Ltd

Emtree Heading

adult; age; aged; antimicrobial therapy; article; *diabetes mellitus/dt [Drug Therapy]; directly observed therapy; female; glucose blood level; hemoglobin blood level; human; *lung cavity; *lung tuberculosis/dt [Drug Therapy]; major clinical study; male; medical record review; risk factor; sex difference; sputum culture; thorax radiography; *treatment outcome; very elderly; ethambutol/cb [Drug Combination]; ethambutol/dt [Drug Therapy]; glucose/ec [Endogenous Compound]; hemoglobin A1c/ec [Endogenous Compound]; insulin/dt [Drug Therapy]; isoniazid/cb [Drug Combination]; isoniazid/dt [Drug Therapy]; oral antidiabetic agent/dt [Drug Therapy]; pyrazinamide/cb [Drug Combination]; pyrazinamide/dt [Drug Therapy]; rifampicin/cb [Drug Combination]; rifampicin/dt [Drug Therapy].

Link to the Ovid Full Text or citation:

Click here for full text options

112.

Determinants of default from pulmonary tuberculosis treatment in Kuwait.

Zhang Q., Gaafer M., El Bayoumy I.

The Scientific World Journal. 2014 (no pagination), 2014. Article Number: 672825. Date of Publication: 2014.

AN: 2014322837

Objectives. To determine the prevalence and risk factors of default from pulmonary tuberculosis treatment in Kuwait. Design. Retrospective study. Patients and methods. We studied all patients who were registered for pulmonary tuberculosis treatment between January 1, 2010, and December 31, 2012, and admitted into TB wards in El Rashid Center or treated in the outpatient clinic in TB Control Unit. Results. There were 110 (11.5%) patients who defaulted from treatment. Fifty-six percent of those who defaulted did so in the first 2 months of treatment and 86.4% of them were still bacteriologically positive at the time of default. Key risk factors associated with noncompliance were male sex, low educational level, non-Kuwaiti nations, history of default, and history of concomitant diabetes mellitus, liver disease, or lung cancer. Multiple drug resistance was also associated with default from treatment. Conclusion. Default from treatment may be partially responsible for the persistent relatively high rates of tuberculosis in Kuwait. Health professionals and policy makers should ensure that all barriers to treatment are removed and that incentives are used to encourage treatment compliance. © 2014 Qing Zhang et al.

Institution

(Zhang) Tuberculosis Diagnosis and Treatment Center, Shanghai, Pulmonary Hospital, Tongji University, 507 Zheng Min Road, Shanghai 200433, China (Gaafer) Community, Environmental and Occupational Medicine Department, Minufiya University, Egypt

(El Bayoumy) Public Health Department, Tanta Faculty of Medicine, Egypt

(El Bayoumy) Ports and Borders Health Division, P.O. Box 35180, 36052 Shaab, Kuwait

Publisher

ScientificWorld Ltd. (Venture West, New Greenham Park, Newbury, Berkshire RG19 6HX, United Kingdom)

Emtree Heading

adolescent; adult; article; child; educational status; female; groups by age; health care policy; hospital admission; human; infant; infection control; Kuwait; *lung tuberculosis; major clinical study; male; *medical care; newborn; outpatient department; *patient compliance; prevalence; retrospective study; risk factor; sputum smear; treatment outcome; treatment refusal.

Link to the Ovid Full Text or citation:

Click here for full text options

113.

Diabetes mellitus, smoking status, and rate of sputum culture conversion in patients with multidrug-resistant tuberculosis: A cohort study from the country of Georgia.

Magee M.J., Kempker R.R., Kipiani M., Tukvadze N., Howards P.P., Venkat Narayan K.M., Blumberg H.M.

PLoS ONE. 9 (4) (no pagination), 2014. Article Number: e94890. Date of Publication: 15 Apr 2014.

AN: 2014302165

Introduction: Diabetes mellitus (DM) is a risk factor for active tuberculosis (TB) but little is known about the effect of DM on culture conversion among patients with multidrug-resistant (MDR)-TB. The primary aim was to estimate the association between DM and rate of TB sputum culture conversion. A secondary objective was to estimate the association between DM and the risk of poor treatment outcomes among patients with MDR-TB. Materials and Methods: A cohort of all adult patients starting MDR-TB treatment in the country of Georgia between 2009-2011 was followed during second-line TB therapy. Cox proportional models were used to estimate the adjusted hazard rate of sputum culture conversion. Log-binomial regression models were used to estimate the cumulative risk of poor TB treatment outcome. Results: Among 1,366 patients with sputum culture conversion information, 966 (70.7%) had culture conversion and the median time to conversion was 68 days (interquartile range 50-120). The rate of conversion was similar among patients with MDR-TB and DM (adjusted hazard ratio [aHR] 0.95, 95%CI 0.71-1.28) compared to patients with MDR-TB only. The rate of culture conversion was significantly less in patients that currently smoked (aHR 0.82, 95%CI 0.71-0.95), had low body mass index (aHR 0.71, 95%CI 0.59-0.84), second-line resistance (aHR 0.56, 95%CI 0.43-0.73), lung cavities (aHR 0.70, 95%CI 0.59-0.83) and with disseminated TB (aHR 0.75, 95%CI 0.62-0.90). The cumulative risk of poor treatment outcome was also similar among TB patients with and without DM (adjusted risk ratio [aRR] 1.03, 95%CI 0.93-1.14). Conclusions: In adjusted analyses, DM did not impact culture conversion rates in a clinically meaningful way but smoking did. © 2014 Magee et al.

Institution

(Magee, Howards, Venkat Narayan, Blumberg) Department of Epidemiology, Rollins School of Public Health, Emory University, Atlanta, GA, United States (Magee, Venkat Narayan, Blumberg) Hubert Department of Global Health, Rollins School of Public Health, Emory University, Atlanta, GA, United States

(Kempker, Blumberg) Division of Infectious Diseases, Department of Medicine, Emory University, Atlanta, GA, United States

(Kipiani, Tukvadze) National Center for Tuberculosis and Lung Diseases, Tbilisi, GA, Georgia

Publisher

Public Library of Science

Emtree Heading

adult; article; bacterium isolate; *biotransformation; body mass; cohort analysis; *diabetes mellitus; disease association; female; human; lung cavity; major clinical study; male; miliary tuberculosis; *multidrug resistant tuberculosis/dr [Drug Resistance]; *multidrug resistant tuberculosis/dt [Drug Therapy]; Mycobacterium tuberculosis; nonhuman; risk assessment; *smoking; *sputum culture; treatment duration; treatment outcome; United States; capreomycin/dt [Drug Therapy]; cycloserine/dt [Drug Therapy]; isoniazid/dt [Drug Therapy]; kanamycin/dt [Drug Therapy]; levofloxacin/dt [Drug Therapy]; protionamide/dt [Drug Therapy]; pyrazinamide/dt [Drug Therapy]; rifampicin/dt [Drug Therapy].

Link to the Ovid Full Text or citation:

Click here for full text options

114.

Effect of diabetes on treatment outcome of smear-positive pulmonary tuberculosis - A report from South India.

Viswanathan V., Vigneswari A., Selvan K., Satyavani K., Rajeswari R., Kapur A.

Journal of Diabetes and its Complications. 28 (2) (pp 162-165), 2014. Date of Publication: March-April 2014.

AN: 2014142715

Aim To assess the effect of diabetes on tuberculosis (TB) treatment outcome and sputum conversion among new smear-positive (NSP) cases registered under Directly Observed Treatment Short Course in South India. Methods Details on sputum conversion and TB treatment outcome were collected from case records of NSP cases (N = 332; M/F 247:85) registered at TB units of three districts in Tamil Nadu. Subjects were screened for diabetes and categorized as diabetic (TBDM) (n = 96; 73:23) and non-diabetic (TBnonDM) (n = 149; 109:40). Results Among 245 selected TB patients, 93.5% were cured, 1.6% completed TB treatment, 2% had TB treatment failure (TF), and 0.4% had treatment default (TD), 0.4% with MDR-TB and 2% death rate. At the end of intensive phase of TB treatment, 14.7% remained sputum positive in the TBDM group, whereas it was 3.5% in the TBnonDM group. Mean duration (days) for sputum conversion was higher in the TBDM group (64.2 +/- 10.5) compared to the TBnonDM group (61.5 +/- 7.5) (p < 0.001). TF rate was higher (4.2% vs 0.7%) and MDR-TB (1%) was also seen in the TBDM group. No death and TD were seen among DM whereas it was 3.4 and 0.7% in TBnonDM. Conclusions Delayed sputum conversion and high TB treatment failure rates were common in NSP cases with diabetes. © 2014 Elsevier Inc. All rights reserved.

Institution

(Viswanathan, Vigneswari, Selvan, Satyavani, Rajeswari, Kapur) M.V. Hospital for Diabetes, Prof. M. Viswanathan Diabetes Research Centre, WHO Collaborating Centre for Education, Research and Training in Diabetes, No. 4, West Madha Church Road, Royapuram, Chennai 600 013, Tamil Nadu, India

Publisher

Elsevier Inc. (360 Park Avenue South, New York NY 10010, United States)

Emtree Heading

adult; aged; article; controlled study; *diabetes mellitus/dt [Drug Therapy]; drug treatment failure; female; glycemic control; high risk population; human; India; *laboratory diagnosis; *lung tuberculosis/dt [Drug Therapy]; major clinical study; male; mortality; multidrug resistant tuberculosis; *new smear positive; priority journal; screening; short course therapy; *sputum smear; *treatment outcome; ethambutol/cb [Drug Combination]; ethambutol/dt [Drug Therapy]; insulin/cb [Drug Combination]; insulin/dt [Drug Therapy]; isoniazid/cb [Drug Combination]; isoniazid/dt [Drug Therapy]; oral antidiabetic agent/cb [Drug Combination]; oral antidiabetic agent/dt [Drug Therapy]; pyrazinamide/cb [Drug Combination]; pyrazinamide/dt [Drug Therapy]; rifampicin/cb [Drug Combination]; rifampicin/dt [Drug Therapy]; streptomycin/cb [Drug Combination]; streptomycin/dt [Drug Therapy].

Link to the Ovid Full Text or citation:

Click here for full text options

115.

Infection-related mortality is higher for kidney allograft recipients with pretransplant diabetes mellitus.

Hayer M.K., Farrugia D., Begaj I., Ray D., Sharif A.

Diabetologia. 57 (3) (pp 554-561), 2014. Date of Publication: March 2014.

AN: 2014143513

Aims/hypothesis: The risk of infection-related mortality in kidney allograft recipients with pre-existing diabetes mellitus is unknown. We determined the risk of infection-related mortality after kidney transplantation in a population-based cohort stratified by diagnosis of pre-existing diabetes mellitus. Methods: We linked data between two national registries (Hospital Episode Statistics and the Office for National Statistics) to select all mortality events after kidney transplantation in England between April 2001 and March 2012. The primary outcome measure was infection-related mortality after transplantation comparing diabetic with non-diabetic recipients. Results: A total of 19,103 kidney allograft recipients were analysed; 2,968 (15.5%) were known to have diabetes before kidney transplantation. After transplantation, 2,085 deaths (10.9%) occurred (median follow-up 4.4 years [interquartile range 2.2-7.3]), with 434 classified as secondary to infection (20.8% of all deaths). Risk of overall (16.0% vs 10.0%, p < 0.001) and infection-related (3.3% vs 2.1%, p < 0.001) mortality after kidney transplantation was higher for diabetic than non-diabetic recipients, respectively. No cytomegalovirus-related deaths occurred in diabetic recipients compared with 5.7% in non-diabetic recipients (p < 0.007), with a trend towards more unspecified sepsis in diabetic recipients (30.6% vs 22.6%, respectively, p = 0.070). Diabetes at the time of transplantation was an independent risk factor predicting infection-related mortality in kidney allograft recipients after transplantation (HR 1.71 [95% CI 1.36, 2.15], p < 0.001). Conclusions/interpretation: Infection-related mortality is more common in kidney allograft recipients with pre-existing diabetes mellitus. Further work is required to determine whether attenuated immunosuppression is beneficial for diabetic kidney allograft recipients. © 2013 Springer-Verlag Berlin Heidelberg.

Institution

(Hayer, Farrugia, Sharif) Department of Nephrology and Transplantation, Renal Institute of Birmingham, Queen Elizabeth Hospital, Edgbaston, Birmingham B15 2WB, United Kingdom (Begaj, Ray) Department of Medical Informatics, Queen Elizabeth Hospital, Birmingham, United Kingdom

Publisher

Springer Verlag (Tiergartenstrasse 17, Heidelberg D-69121, Germany)

Emtree Heading

adult; aged; article; brain infection/co [Complication]; brain infection/ep [Epidemiology]; bronchopneumonia/co [Complication]; bronchopneumonia/ep [Epidemiology]; cellulitis/co [Complication]; cellulitis/ep [Epidemiology]; cholecystitis/co [Complication]; cholecystitis/ep [Epidemiology]; cohort analysis; colitis/co [Complication]; colitis/ep [Epidemiology]; controlled study; Cytomegalovirus; *cytomegalovirus infection/co [Complication]; *cytomegalovirus infection/ep [Epidemiology]; *diabetes mellitus; diabetic patient; disease registry; *end stage renal disease/su [Surgery]; endocarditis/co [Complication]; endocarditis/ep [Epidemiology]; female; *graft infection/co [Complication]; *graft infection/ep [Epidemiology]; graft recipient; hazard ratio; hepatitis/co [Complication]; hepatitis/ep [Epidemiology]; human; incidence; kidney allograft; *kidney transplantation; major clinical study; male; middle aged; multicenter study; mycosis/co [Complication]; mycosis/ep [Epidemiology]; osteomyelitis/co [Complication]; osteomyelitis/ep [Epidemiology]; outcome assessment; peritonitis/co [Complication]; peritonitis/ep [Epidemiology]; priority journal; risk factor; sepsis/co [Complication]; skin ulcer/co [Complication]; skin ulcer/ep [Epidemiology]; surgical mortality; surgical patient; surgical risk; trend study; tuberculosis/co [Complication]; tuberculosis/ep [Epidemiology]; United Kingdom; urinary tract infection/co [Complication]; urinary tract infection/ep [Epidemiology].

Link to the Ovid Full Text or citation:

Click here for full text options

116.

Impact of diabetes on treatment outcomes and long-term survival in multidrug-resistant tuberculosis.

Kang Y.A., Kim S.Y., Jo K.-W., Kim H.J., Park S.-K., Kim T.-H., Kim E.K., Lee K.M., Lee S.S., Park J.S., Koh W.-J., Kim D.Y., Shim T.S.

Respiration. 86 (6) (pp 472-478), 2014. Date of Publication: January 2014.

AN: 2014071108

Background: Few studies have investigated the impact of diabetes mellitus (DM), a globally increasing metabolic disease, on treatment outcomes and long-term survival in patients with multidrug-resistant forms of tuberculosis (MDR-TB). Objectives: We analyzed outcomes in a large cohort to assess the impact of DM on treatment outcomes of patients with MDR-TB. Methods: MDR-TB patients newly diagnosed or retreated between 2000 and 2002 and followed for 8-11 years were retrospectively analyzed with respect to the effect of DM as a comorbidity on their treatment outcome and long-term survival. Results: Of 1,407 patients with MDR-TB, 239 (17.0%) had coexisting DM. The mean age and body mass index were higher in MDR-TB patients with DM [MDR-TBDM(+)] than in those without DM [MDR-TBDM(-)]. Patients with MDR-TB and a comorbidity of DM had a significantly lower treatment success rate than those without a history of DM (36.0 vs. 47.2%, p = 0.002). In addition, DM was the negative predictor for MDR-TB treatment success in multivariate analyses [odds ratio 0.51, 95% confidence interval (CI) 0.26-0.99]. Mean survival times were also lower in MDR-TBDM(+) than in MDR-TBDM(-) patients (102 vs. 114 months, p = 0.001), with DM as a significant predictor of poor long-term survival in multivariate analyses (hazard ratio 1.59, 95% CI 1.01-2.50). Conclusions: Among MDR-TB patients, DM was a relatively common comorbidity. In patients undergoing treatment for MDR-TB and followed for 8-11 years, it was found to be independently associated with an increased risk of both treatment failure and death. Copyright © 2013 S. Karger AG, Basel.

Institution

(Kang, Kim) Department of Internal Medicine, Yonsei University College of Medicine, Seoul, South Korea (Jo, Shim) Division of Pulmonary and Critical Care Medicine, University of Ulsan College of Medicine, Asan Medical Center, 388-1 Pungnap-dong, Songpa-gu, Seoul, South Korea

(Kim) Department of Epidemiology, Korean Institute of Tuberculosis, Seoul, South Korea

(Kim) Department of Internal Medicine, Hanyang University College of Medicine, Seoul, South Korea

(Koh) Division of Pulmonary and Critical Care Medicine, Department of Medicine, Sungkyunkwan University School of Medicine, Seoul, South Korea

(Park) Clinical Research Center, National Masan Hospital, Masan, South Korea

(Kim) Division of Pulmonary and Critical Care Medicine, Department of Internal Medicine, Pochon CHA University, Seongnam, South Korea

(Lee) Department of Internal Medicine, Chungbuk National University College of Medicine, Chungbuk National University Hospital, Cheongju, South Korea

(Lee) Department of Internal Medicine, Inje University School of Medicine, Ilsan Paik Hospital, Goyang, South Korea

(Park) Department of Internal Medicine, Dankook University College of Medicine, Dankook University Hospital, Cheonan, South Korea

(Kim) Department of Thoracic Surgery, National Mokpo Hospital, Mokpo, South Korea

Publisher

S. Karger AG (Allschwilerstrasse 10, P.O. Box, Basel CH-4009, Switzerland)

Emtree Heading

adolescent; adult; aged; antibiotic resistance; article; body mass; child; cohort analysis; comorbidity; *diabetes mellitus; female; human; *long term survival; major clinical study; male; middle aged; *multidrug resistant tuberculosis/dt [Drug Therapy]; outcome assessment; predictor variable; priority journal; retrospective study; school child; short course therapy; survival time; treatment duration; treatment outcome; very elderly; young adult; capreomycin/dt [Drug Therapy]; enviomycin/dt [Drug Therapy]; kanamycin/dt [Drug Therapy].

Link to the Ovid Full Text or citation:

Click here for full text options

117.

Frequency of tuberculosis among diabetic patients in the People's Republic of China.

Wang H.-T., Zhang J., Ji L.-C., You S.-H., Bai Y., Dai W., Wang Z.-Y.

Therapeutics and Clinical Risk Management. 10 (1) (pp 45-49), 2014. Date of Publication: 2014.

AN: 2014060297

The People's Republic of China has nearly the highest incidence of both diabetes mellitus (DM) and tuberculosis (TB) worldwide. DM increases the risk of TB by two to three times and adversely affects TB treatment outcomes. The increasing epidemic of DM in the People's Republic of China is due to decreased physical activity, unhealthy diet, and obesity. Over the last 20 years, the excellent free China National Tuberculosis Program has been set up, and the "DOTS" (directly observed treatment + short-course chemotherapy) model for TB control has successfully reduced the burden of TB, but the disease is still a considerable problem. Given the high burden of TB and DM in the People's Republic of China and the relationship between the two diseases, it is sensible to screen DM patients for TB. A bidirectional screening of the two diseases was conducted in the People's Republic of China from 2011 to 2012, which identified a TB incidence in patients with DM of about 958 per 100,000. Here, we report the findings of our recent study on the incidence of TB among diabetic patients in the People's Republic of China. The data agree with those of previous reports. © 2014 Wang et al. This work is published by Dove Medical Press Limited.

Institution

(Wang, Zhang, Ji, You, Bai) Department of Otorhinolaryngology, Head, and Neck Surgery, Chinese PLA General Hospital, China (Dai) Department of Medical Record Administration, Chinese PLA General Hospital, China

(Wang) The 3rd TB Department, Chinese PLA 309 Hospital, Beijing, China

Publisher

Dove Medical Press Ltd. (PO Box 300-008, Albany, Auckland, New Zealand)

Emtree Heading

article; bacterial transmission; CD28 gene; China; controlled study; *diabetes mellitus; disease association; disease marker; gene; gene frequency; Hk2 gene; human; incidence; infection rate; infection risk; lymphocyte activation; molecular dynamics; nonhuman; outcome assessment; patient assessment; risk assessment; risk factor; risk reduction; screening test; *tuberculosis/ep [Epidemiology]; biological marker/ec [Endogenous Compound]; CD28 antigen/ec [Endogenous Compound].

Link to the Ovid Full Text or citation:

Click here for full text options

118.

Impact of diabetes mellitus on clinical parameters and treatment outcomes of diagnosed pulmonary tuberculosis patients.

Amar J.B., Safta B.B., Dhahri B., Azzabi S., Baccar M.A., Aouina H., Bouacha H.

European Respiratory Journal. Conference: European Respiratory Society Annual Congress 2014 Munich Germany. Conference Start: 20140906 Conference End: 20140906. Conference Publication: (var.pagings). 44 (no pagination), 2014. Date of Publication: 01 Sep 2014.

AN: 71851866

Tuberculosis (TB) is an infectious disease which is still a major cause of morbidity and mortality throughout the world. People with diabetes have a three times higher risk of developing active TB than people without diabetes. Aim: The aim of this study is to determine the impact of diabetes on clinical and radiological manifestations and treatment outcome of pulmonary TB (PTB) patients. Methods: We have retrospectively analyzed clinical features in 30 diabetic patients treated for TB; this group was compared to a control group of 65 non-diabetic tuberculosis patients. Results: In the diabetic group, the mean age is 52 years (22-74); 52% of patients had type I diabetes. The duration of diabetes course at the time of PTB diagnosis varied from 1 month to 16 years. The most frequent symptom was weight loss found in 85% of cases. Haemoptysis was noted in 50% and fever in 61% of patients. Radiographic abnormalities were bilateral in 74 % of patients; lower lung lesion was observed in 52% of patients. The percentage of patients with cavities was higher in the patients with diabetes (74%) than in those without (38%; P< 0.05). The diagnosis of PTB was made by microscopic sputum examination and sputum culture in all patients. Five patients had extrapulmonary TB: liver (2 cases), blood (2 cases) and bone in one case. Therapy length is similar between two groups. No drug resistance was noted. Insulin was required in all patients with type II diabetes. Diabetes is associated with a higher frequency of lung after-effects compared with non-diabetic (52% vs 20%; p<0.05). Conclusion: PT seems to aggravate diabetes course and diabetes seems to increase radiological after-effects.

Institution

(Amar, Safta, Dhahri, Azzabi, Baccar, Aouina, Bouacha) Pulmonary Department, Charles Nicolle Hospital, Tunis, Tunisia

Publisher

European Respiratory Society

Emtree Heading

*diabetes mellitus; *parameters; *treatment outcome; *lung tuberculosis; *patient; *human; *European; *society; tuberculosis; diagnosis; weight reduction; infection; morbidity; insulin dependent diabetes mellitus; control group; drug resistance; bone; diabetic patient; non insulin dependent diabetes mellitus; hemoptysis; fever; clinical feature; risk; liver; mortality; sputum culture; blood; therapy; sputum examination; lung lesion; lung; insulin.

Link to the Ovid Full Text or citation:

Click here for full text options

119.

Comorbidities and MDR-TB treatment outcomes in Georgia-2009-11 cohort.

Kikvidze M., Ikiashvili L.

European Respiratory Journal. Conference: European Respiratory Society Annual Congress 2014 Munich Germany. Conference Start: 20140906 Conference End: 20140906. Conference Publication: (var.pagings). 44 (no pagination), 2014. Date of Publication: 01 Sep 2014.

AN: 71851788

Background. Treatment of MDR-TB is complex and chemotherapy course's outcome depends on many factors. Comorbidities may complicate TB disease and effect treatment response. Aims and objectives. The aim of the study was comparison of chemotherapy response among patients with and those without comorbidities in a setting of individualized MDR-TB care and adequate management of all diseases. Methods. We conducted a retrospective cohort study of 1970 drug-resistant tuberculosis patients integrated in DOTS+ programme in 2009-2011 years in Georgia. Patients were routinely investigated for HIV, diabetes mellitus (DM) and viral hepatitis. Documentary approved diagnoses of other diseases were taken in account. Dispersive analysis using Mann-Whitney/Wilcoxon Two-Sample Test was provided to compare the treatment outcomes of patients with and those without comorbidity. Results. Of 1970 patients with drug-resistant tuberculosis 486(24,7%) had one or more comorbidity. 223 patient(11,3%) had liver diseases, DM-104(5,2%), HIV-72(3,7%), Psychiatric diseases - 53(2,7%), other-55(2,9%). Definition "success" received 966(49%) patient:44,0% with comorbidities vs 50,7%-without(p=0,01),"Default"-30,5% vs 28,4%, "Failure"- 4,1% vs 4,0(p>0,05, CI-95%),"Death"- 10,7% vs 5,7%(p<0,05, CI-95%). Mortality rate was highest during HIV-co-infection(RR-3, 73, 95% CI 2,38-5,86, p<0, 05). Definition "success" was lowest during HIV-co-infection(RR-0,67, 95%CI48-0,93, p<0,05) and chronic viral hepatitis "C"(RR-0,79, 95%CI 0,66-0,95, p<0,05). Conclusions. Our cohort study revealed that comorbidities had serious impact on treatment outcomes andwere associated with significant lower successful treatment outcomes and about twice higher death rate.

Institution

(Kikvidze, Ikiashvili) Department of MDR-TB, National Center for Tuberculosis and Lung Diseases (NCTBLD), Tbilisi, Georgia

Publisher

European Respiratory Society

Emtree Heading

*treatment outcome; *European; *society; patient; human; drug resistant tuberculosis; cohort analysis; mixed infection; virus hepatitis; chemotherapy; mortality; diseases; Human immunodeficiency virus; comorbidity; death; mental disease; liver disease; diagnosis; diabetes mellitus; treatment response; short course therapy.

Link to the Ovid Full Text or citation:

Click here for full text options

120.

Gender differences and TB treatment outcome among anti tuberculosis drug induced hepatitis patients.

Nadarajah K., Khan A.H., Hadi M.A., Aftab R.A., Ahmad N.

Respirology. Conference: 19th Congress of the Asian Pacific Society of Respirology Bali Indonesia. Conference Start: 20141113 Conference End: 20141116. Conference Publication: (var.pagings). 19 (pp 246), 2014. Date of Publication: November 2014.

AN: 71678367

Background: Anti-tubercle drug-induced hepatotoxicity (ATDH) causes substantial morbidity and mortality and diminishes treatment effectiveness. Aims And Objective: The aim of current study was to note the association between gender differences among anti-tuberculosis drug induced hepatotoxity patients and to evaluate TB treatment outcomes. Method: A retrospective cohort study was conducted at Hospital Pulau Pinang Malaysia. All TB patients presented between January 2007 and September 2013 were included for the current study. Patients with inadequate medical records and patients receiving concurrent potential hepatotoxic medications along with anti-TB treatment were excluded from current study. A valid data collection form was used for collecting patient demographic and clinical data. Data was analyzed by using SPSS version 20. Results: A total of 3093 TB patients were included for the final analysis. One hundred and ninety patients suffered from drug induced hepatitis, of which 146 (76.8%) were male and 44 (23.3%) were female patients. The mean age of male patients suffering from drug induced hepatitits was 47.49 (SD +/- 14.28) years whereas the mean age for female patients was 45.0 (SD +/- 14.96). Area of residence (p 0.001), smoking habit (p < 0.001), alcohol consumption (p < 0.001) and intravenous drug users (p < 0.001) had statistical significant association with drug induced hepatitis patient gender. Anti-tuberculosis induced drug hepatitis patients with diabetes mellitus (p 0.046) and HIV/AIDS 107 (p 0.014) also had statistical significant association with gender. A total of 82 (72.6%) male and 31(27.4%) female patients treatment outcome were successful, 21(87.5%) male and 3 (12.5%) female patients were defaulter, 12 (85.7%) male and 2 (14.3%) female patients were transferred out, 22 (81.5) male and 5 (18.5) female patients expired whereas 9 (75%) male and 3 (25%) female patients treatment was continued. Conclusion: Multiple factors are associated with patient gender in the development of anti- tubercle drug induced hepatitis. Therefore careful monitoring and regular patient assessment can optimize TB treatment outcomes in such patients.

Institution

(Nadarajah, Khan, Aftab, Ahmad) Department of Clinical Pharmacy, School of Pharmaceutical Sciences Universiti Sains Malaysia, Penang 11800, Malaysia (Hadi) Monash University Malaysia, Bandar Sunway 47500, Malaysia

Publisher

Blackwell Publishing

Emtree Heading

*treatment outcome; *tuberculosis; *toxic hepatitis; *patient; *human; *Asian; *society; *sex difference; female; male; implantable cardioverter defibrillator; gender; liver toxicity; hepatitis; intravenous drug abuse; alcohol consumption; smoking habit; clinical study; therapy; mortality; information processing; drug therapy; medical record; Malaysia; betel nut; cohort analysis; morbidity; patient assessment; diabetes mellitus; monitoring; hospital; data analysis software.

Link to the Ovid Full Text or citation:

Click here for full text options

121.

Characteristics and treatment outcomes of MDR tuberculosis in Taiwan.

Yu M.-C., Chen H.-Y., Chien S.-H., Jou R.

International Journal of Infectious Diseases. Conference: 16th International Congress on Infectious Diseases, ICID 2014 Cape Town South Africa. Conference Start: 20140402 Conference End: 20140405. Conference Publication: (var.pagings). 21 (pp 57), 2014. Date of Publication: April 2014.

AN: 71633880

Background: In Taiwan, the MDR-TB among new TB cases was 1%, and 6% in retreated TB cases. Facing the challenge of MDR-TB, a designated government-organized and hospital-initiated with an enhanced direct observed treatment program was implemented in May2007 to provide patient-centered care and better management for MDR-TB cases. In this study, we investigated characteristics of MDR-TB cases notified in 2007-2009 in northern Taiwan and identified possible risk factors associated with treatment outcomes. Methods & Materials: In 2007-2009, 151 MDR-TB cases were confirmed. Demographic, clinical characteristics of patients and bacteriological and genetic data of Mycobacterium tuberculosis strains were analyzed. The treatment outcome at 30 months was evaluated. Comparisons were performed using Mantel-Haenszel chi-squared method or Fisher's exact. Statistical significance was defined as a p value < 0.05. Results: Of the 151 MDR-TB cases, 106 (70.2%) were males and 45 (29.8%) females with a mean age of 52.0 and 45.4 years old, respectively. Of the 151 MDR-TB cases, 54.3% were new cases and 23.2% cases were either pre-XDR or XDR cases. Among 39.7% of cases with comobility, 25.8% had diabetes mellitus. Furthermore, 49.5% of patients were infected with Beijing genotype strains and 23.8% were resulted from recent transmission. Outcome was determined for 126 (83.4%) cases enrolled in a government-organized and hospital-initiated MDR-TB treatment consortium. Excluding two cases transferred out, 85.5% had favorable outcome and 18 had unfavorable outcomes including 16 death and 2 failure cases with severe complications due to anti-TB medication.Wefound that age group elder than 65 years old (p < 0.01), BMI < 18.5 (p = 0.01), M. tuberculosis strains resistant to streptomycin (p = 0.02) were factors for unfavorable treatment outcome. Conclusion: High proportion ofnew MDR-TB cases suggesting the need for stringent control measures including early diagnosis, and strengthened contact tracing strategies. Nevertheless, a designated patient-center management program adopting individualized regimens has resulted in good favorable outcome in northern Taiwan.

Institution

(Yu, Chien) Wan Fang Hospital, Taipei Medical University, Taipei, Taiwan (Republic of China) (Chen, Jou) Centers for Disease Control, Taipei, Taiwan (Republic of China)

Publisher

Elsevier

Emtree Heading

*treatment outcome; *tuberculosis; *Taiwan; *infection; human; patient; hospital; statistical significance; government; Mycobacterium tuberculosis; contact examination; male; female; death; risk factor; genotype; diabetes mellitus; groups by age; early diagnosis; patient care; streptomycin.

Link to the Ovid Full Text or citation:

Click here for full text options

122.

Should HIV and tuberculosis services be integrated in settings with high TB/HIV co-infection?.

Harley B.

International Journal of Infectious Diseases. Conference: 16th International Congress on Infectious Diseases, ICID 2014 Cape Town South Africa. Conference Start: 20140402 Conference End: 20140405. Conference Publication: (var.pagings). 21 (pp 44), 2014. Date of Publication: April 2014.

AN: 71633851

Integration of TB and HIV services in settings with high coinfection, has received a lot of attention yet remains a challenge. Direction fromWHOcame in 2001 following the first meeting of the global working group on TB/HIV; the terminology at the time was "programme interaction" and "collaboration", which has shifted to "integration", but what remains is lack of clarity as to exactly what integration means, how to achieve it and the benefits and challenges associated with it. Data from many settings shows very high co-infection rates: this overlapping epidemiology and the goal of providing client-centred care are at the core of the push to integrate services. It has been easier to achieve integration of TB and HIV services at single, well defined points within the traditional, vertical, TB and HIV services and less so for services which are provided over a length of time. There is scanty and conflicting evidence that integration per se leads to better treatment outcomes or is more cost effective. Other challenges in making integration a reality include infection control and buy-in and integration at all levels of funding, service delivery and programme support, as well as the lag in development and solidly embedding changes in the system such as in policy, training and monitoring and evaluation (including stationery, manual and electronic registers and audit tools). Implementing integrated TB and HIV services has also often been tackled in conjunction with other changes in the system, such as decentralisation or the addition of new services (HIV related) with limited or no extra resources being provided, including for the change management process. There are also broader integration issues, such as with sexual and reproductive health and substance abuse, and with the growing cohort of ageing ART clients at increased risk of cardiovascular disease even with viral suppression, overlapping epidemiology of hypertension and diabetes with HIV is set to increase. In conclusion, there is a push to integrate TB and HIV services, but much remains for this integration to be optimally implemented in diverse settings and to find its place in the landscape of broader health system changes.

Institution

(Harley) Cape Town Municipality, Cape Town, South Africa

Publisher

Elsevier

Emtree Heading

*tuberculosis; *infection; *mixed infection; *Human immunodeficiency virus; human; epidemiology; funding; infection rate; treatment outcome; infection control; substance abuse; health care delivery; monitoring; landscape; book; register; medical audit; change management; reproductive health; diabetes mellitus; aging; risk; cardiovascular disease; hypertension; policy; embedding; health care.

Link to the Ovid Full Text or citation:

Click here for full text options

123.

Risk factors for poor treatment outcomes in patients with MDR-TB and XDR-TB in China: Retrospective multicenter investigation.

Tang S., Tan S., Yao L., Li F., Li L., Guo Z., Liu Y., Hao X., Li Y., Ding X., Zhang Z., Tong L., Huang J.

Chest. Conference: CHEST World Congress 2014 Annual Meeting Madrid Spain. Conference Start: 20140321 Conference End: 20140324. Conference Publication: (var.pagings). 145 (3 MEETING ABSTRACT) (no pagination), 2014. Date of Publication: March 2014.

AN: 71428761

PURPOSE: Multidrug resistant tuberculosis (MDR-TB) and extensively drug-resistant (XDR-TB) is a major public health threat in China. To investigate risk factors for poor treatment outcomes in patients with MDR-TB and XDR-TB in China. METHODS: We retrospectively analyzed TB Patients with culture-proven MDR-TB and HIV-negative from July 2006 to June 2010 in five large-scale Tuberculosis Specialized Hospitals in China. RESULTS: A total of 586 HIV-negative patients with MDR-TB were included in the final analysis. Among them, 169 cases (28.8%) had XDR-TB. The patients were divided into XDR-TB group (n=169) and other MDR-TB group (MDR-TB excluding XDR-TB) (n=417). In total, 240 patients (40.95%) had treatment Success, and 346 (59.05%) had Poor treatment outcomes. The treatment success rate in other MDR-TB group was 52.2%, significantly higher than that in XDR-TB group (13%, P<0.001), whereas poor treatment outcomes were more common in patients with XDR-TB than in patients with other MDRTB (87 vs.47.8%; P<0.001). In multivariate logistic regression analysis, Poor Treatment Outcomes were associated with duration of previous anti-TB treatment of more than one year, a BMI less than 18.5 kg/m2, XDR, retreatment, diabetes, tumor, decreased albumin, cavitation and 5~6 lung fields with disease. In multivariate logistic regression analysis, having a tumor was the only independent risk factor associated with death. CONCLUSIONS: The prevalence of MDR- and XDR-TB is high in some areas in China with poor treatment outcome. The presence of XDR, low BMI, hypoalbuminemia, comorbidity and previous anti-TB treatment are independent poor prognostic factors in patients with MDR-TB. Effective TB control strategies should be implemented to prevent the development and spread of MDR- and XDR-TB in China.

Institution

(Tang, Tan, Yao, Li, Li, Guo, Liu, Hao, Li, Ding, Zhang, Tong, Huang) Shanghai Pulmonary Hospital, Shanghai, China

Publisher

American College of Chest Physicians

Emtree Heading

*risk factor; *treatment outcome; *human; *China; *patient; multivariate logistic regression analysis; neoplasm; multidrug resistant tuberculosis; prevalence; death; diabetes mellitus; lung; retreatment; hospital; Human immunodeficiency virus; control strategy; comorbidity; hypoalbuminemia; tuberculosis; public health; albumin.

Link to the Ovid Full Text or citation:

Click here for full text options

124.

The double burden.

Faurholt-Jepsen D.

Danish medical journal. 60 (7) (pp B4673), 2013. Date of Publication: 01 Jul 2013.

AN: 23809978

One third of the world's population is latently infected with Mycobacterium tuberculosis, and with the lifestyle changes succeeding the on-going urbanization, populations already burdened by tuberculosis are experiencing a dramatic increase in chronic diseases, with diabetes being a serious challenge. Tuberculosis and diabetes are not only becoming co-existing diseases. In fact, the diseases interact, and there is evidence to suggest that especially diabetes disease increases the susceptibility for developing active tuberculosis disease. Furthermore, it is plausible that tuberculosis leads to, either transient or permanent, impairment of the glucose metabolism, which ultimately will turn into diabetes. A number of studies from the Americas, Europe, Asia, and, most lately, from sub-Saharan Africa have reported strong association between tuberculosis and diabetes; on average, the estimated risk of active tuberculosis is thrice as high among people with diabetes. The study from sub-Saharan Africa was conducted in Tanzania and is the basis of this thesis. Based on available evidence on the association between tuberculosis and diabetes, the primary aim of the study was to assess the role of diabetes for tuberculosis risk, manifestations, treatment outcomes and survival in a Tanzanian population of tuberculosis patients and non-tuberculosis neighbourhood controls. The study was conducted in Mwanza City in northern Tanzania, with a population exceeding half a million inhabitants, with tuberculosis and HIV being common infections in the region, but with little knowledge about the prevalence of diabetes. We recruited newly diagnosed pulmonary tuberculosis patients from spring 2006 and continuously till the fall 2009, with all participating in a nutritional intervention running in parallel with the medical tuberculosis treatment. All participants underwent diabetes and HIV testing as well as a series of measurements such as anthropometric, clinical and paraclinical parameters. The population was followed up during treatment (2 and 5 months) to assess treatment outcome as well as after one year to assess their survival status. Based on data from 1,250 tuberculosis patients and 350 neighbourhood controls, we found that 38 and 21%, respectively, had impaired glycaemia, and that the prevalence of diabetes was 17 and 9% among tuberculosis patients and controls, respectively. This difference in prevalence between patients and controls was equivalent to an adjusted odds ratio of more than four, indicating a strong association between tuberculosis and diabetes. Furthermore, we found that diabetes was associated with tuberculosis among both participants with or without HIV co-infection. Despite the strong association, diabetes had only moderate clinical implications when the tuberculosis patients initiated the tuberculosis treatment; the patients with diabetes co-morbidity had a minor elevation in the immune response and more frequently reported to have fever. Furthermore, diabetes did not seem to delay time to sputum conversion during treatment. Nevertheless, diabetes co-morbidity led to impaired treatment outcome with slower recovery of weight and haemoglobin and a more than four times higher mortality rate within the initial phase of tuberculosis treatment. In conclusion, in the African region, the double burden of tuberculosis and diabetes is becoming a major health problem. Although the tuberculosis incidence has stabilized during the last decade, the increasing incidence of diabetes will possibly interfere with tuberculosis control and may, consequently, make the tuberculosis incidence increase again. Future research strategies should focus on enhanced diagnostic tools to identify tuberculosis patients with diabetes co-morbidity, and on the role of disease-disease, drug-disease and drug-drug interactions between tuberculosis and diabetes diseases and treatments.

Institution

(Faurholt-Jepsen) Department of Human Nutrition, University of Copenhagen, Rolighedsvej 30, 1958 Frederiksberg C, Denmark

Emtree Heading

adolescent; adult; aged; case control study; clinical trial; complication; cross-sectional study; female; follow up; human; Human immunodeficiency virus infection; male; middle aged; mortality; non insulin dependent diabetes mellitus; prospective study; risk factor; statistical model; survival; Tanzania; treatment outcome; Tuberculosis, Pulmonary/di [Diagnosis]; Tuberculosis, Pulmonary/dt [Drug Therapy]; very elderly; young adult; tuberculostatic agent/dt [Drug Therapy].

Link to the Ovid Full Text or citation:

Click here for full text options

125.

Impact of diabetes on treatment outcomes and long-term survival in multidrug-resistant tuberculosis.

Kang Y.A., Kim S.Y., Jo K.-W., Kim H.J., Park S.-K., Kim T.-H., Kim E.K., Lee K.M., Lee S.S., Park J.S., Koh W.-J., Kim D.Y., Shim T.S.

Respiration. 86 (6) (pp 472-478), 2013. Date of Publication: 2013.

AN: 2015973079

Background: Few studies have investigated the impact of diabetes mellitus (DM), a globally increasing metabolic disease, on treatment outcomes and long-term survival in patients with multidrug-resistant forms of tuberculosis (MDR-TB). Objectives: We analyzed outcomes in a large cohort to assess the impact of DM on treatment outcomes of patients with MDR-TB. Methods: MDR-TB patients newly diagnosed or retreated between 2000 and 2002 and followed for 8-11 years were retrospectively analyzed with respect to the effect of DM as a comorbidity on their treatment outcome and long-term survival. Results: Of 1,407 patients with MDR-TB, 239 (17.0%) had coexisting DM. The mean age and body mass index were higher in MDR-TB patients with DM [MDR-TBDM(+)] than in those without DM [MDR-TBDM(-)]. Patients with MDR-TB and a comorbidity of DM had a significantly lower treatment success rate than those without a history of DM (36.0 vs. 47.2%, p = 0.002). In addition, DM was the negative predictor for MDR-TB treatment success in multivariate analyses [odds ratio 0.51, 95% confidence interval (CI) 0.26-0.99]. Mean survival times were also lower in MDR-TBDM(+) than in MDR-TBDM(-) patients (102 vs. 114 months, p = 0.001), with DM as a significant predictor of poor long-term survival in multivariate analyses (hazard ratio 1.59, 95% CI 1.01-2.50). Conclusions: Among MDR-TB patients, DM was a relatively common comorbidity. In patients undergoing treatment for MDR-TB and followed for 8-11 years, it was found to be independently associated with an increased risk of both treatment failure and death.

Institution

(Kang, Kim) Department of Internal Medicine, Yonsei University College of Medicine, South Korea (Jo, Shim) Division of Pulmonary and Critical Care Medicine, University of Ulsan College of Medicine, Asan Medical Center, 388-1 Pungnap-dong, Songpa-gu, Seoul, South Korea

(Kim) Department of Epidemiology, Korean Institute of Tuberculosis, South Korea

(Kim) Department of Internal Medicine, Hanyang University College of Medicine, South Korea

(Koh) Department of Medicine, Samsung Medical Center, Sungkyunkwan University School of Medicine, Seoul, South Korea

(Park) Clinical Research Center, National Masan Hospital, Masan, South Korea

(Kim) Department of Internal Medicine, College of Medicine, Pochon CHA University, Seongnam, South Korea

(Lee) Department of Internal Medicine, Chungbuk National University College of Medicine, Chungbuk National University Hospital, Cheongju, South Korea

(Lee) Department of Internal Medicine, Inje University School of Medicine, Ilsan Paik Hospital, Goyang, South Korea

(Park) Department of Internal Medicine, Dankook University College of Medicine, Dankook University Hospital, Cheonan, South Korea

(Kim) Department of Thoracic Surgery, National Mokpo Hospital, Mokpo, South Korea

Publisher

S. Karger AG

Emtree Heading

adolescent; adult; aged; article; comorbidity; controlled study; *diabetes mellitus; female; follow up; human; *long term survival; major clinical study; male; *multidrug resistant tuberculosis; outcome assessment; priority journal; *prognosis; retrospective study; survival time; treatment outcome.

Link to the Ovid Full Text or citation:

Click here for full text options

126.

Impact of diabetes on treatment outcomes and long-term survival in multidrug-resistant tuberculosis.

Kang Y.A., Kim S.Y., Jo K.-W., Kim H.J., Park S.-K., Kim T.-H., Kim E.K., Lee K.M., Lee S.S., Park J.S., Koh W.-J., Kim D.Y., Shim T.S.

Respiration. 86 (6) (pp 472-478), 2013. Date of Publication: 2013.

AN: 2015947462

Background: Few studies have investigated the impact of diabetes mellitus (DM), a globally increasing metabolic disease, on treatment outcomes and long-term survival in patients with multidrug-resistant forms of tuberculosis (MDR-TB). Objectives: We analyzed outcomes in a large cohort to assess the impact of DM on treatment outcomes of patients with MDR-TB. Methods: MDR-TB patients newly diagnosed or retreated between 2000 and 2002 and followed for 8-11 years were retrospectively analyzed with respect to the effect of DM as a comorbidity on their treatment outcome and long-term survival. Results: Of 1,407 patients with MDR-TB, 239 (17.0%) had coexisting DM. The mean age and body mass index were higher in MDR-TB patients with DM [MDR-TBDM(+)] than in those without DM [MDR-TBDM(-)]. Patients with MDR-TB and a comorbidity of DM had a significantly lower treatment success rate than those without a history of DM (36.0 vs. 47.2%, p = 0.002). In addition, DM was the negative predictor for MDR-TB treatment success in multivariate analyses [odds ratio 0.51, 95% confidence interval (CI) 0.26-0.99]. Mean survival times were also lower in MDR-TBDM(+) than in MDR-TBDM(-) patients (102 vs. 114 months, p = 0.001), with DM as a significant predictor of poor long-term survival in multivariate analyses (hazard ratio 1.59, 95% CI 1.01-2.50). Conclusions: Among MDR-TB patients, DM was a relatively common comorbidity. In patients undergoing treatment for MDR-TB and followed for 8-11 years, it was found to be independently associated with an increased risk of both treatment failure and death.

Institution

(Kang, Kim) Department of Internal Medicine, Yonsei University College of Medicine, Asan Medical Center, Seoul, South Korea (Jo, Shim) Division of Pulmonary and Critical Care Medicine, University of Ulsan College of Medicine, Asan Medical Center, 388-1 Pungnap-dong, Songpa-gu, Seoul, South Korea

(Kim) Department of Epidemiology, Korean Institute of Tuberculosis, Sungkyunkwan University School of Medicine, Seoul, South Korea

(Kim) Department of Internal Medicine, Hanyang University College of Medicine, Sungkyunkwan University School of Medicine, Seoul, South Korea

(Koh) Department of Medicine, Samsung Medical Center, Sungkyunkwan University School of Medicine, Seoul, South Korea

(Park) Clinical Research Center, National Masan Hospital, Masan, South Korea

(Kim) Department of Internal Medicine, College of Medicine, Pochon CHA University, Seongnam, South Korea

(Lee) Department of Internal Medicine, Chungbuk National University College of Medicine, Chungbuk National University Hospital, Cheongju, South Korea

(Lee) Department of Internal Medicine, Inje University School of Medicine, Ilsan Paik Hospital, Goyang, South Korea

(Park) Department of Internal Medicine, Dankook University College of Medicine, Dankook University Hospital, Cheonan, South Korea

(Kim) Department of Thoracic Surgery, National Mokpo Hospital, Mokpo, South Korea

Publisher

S. Karger AG

Emtree Heading

acid fast bacterium; adult; aged; article; bacterium culture; body mass; chronic kidney disease; chronic lung disease; cohort analysis; comorbidity; connective tissue disease; controlled study; *diabetes mellitus; diabetic patient; drug sensitivity; extensively drug resistant tuberculosis; female; human; hyperthyroidism; hypothyroidism; Korean (people); long term survival; major clinical study; male; malnutrition; mental disease; *multidrug resistant tuberculosis; outcome assessment; postgastrectomy syndrome; priority journal; randomized controlled trial; retrospective study; short course therapy; survival time; thorax radiography; treatment duration.

Link to the Ovid Full Text or citation:

Click here for full text options

127.

Diabetes mellitus and tuberculosis facts and controversies.

Baghaei P., Marjani M., Javanmard P., Tabarsi P., Masjedi M.R.

Journal of Diabetes and Metabolic Disorders. 12 (1) (no pagination), 2013. Article Number: 58. Date of Publication: December 20, 2013.

AN: 2014846891

Tuberculosis (TB) and diabetes mellitus (DM) are both important health issues. A bidirectional association between them has been demonstrated by many researchers. The link of DM and TB is more prominent in developing countries where TB is endemic and the burden of diabetes mellitus is increasing. The association between diabetes and tuberculosis may be the next challenge for global tuberculosis control worldwide. Proper planning and collaboration are necessary to reduce the dual burden of diabetes and TB. One model similar to the TB-HIV program for prevention, screening and treatment of both diseases can be the best approach. In this paper, we review existing data and discuss the matters of controversy that would be helpful for determining research priorities in different countries.

Institution

(Baghaei, Marjani, Tabarsi) Clinical Tuberculosis and Epidemiology Research Center, National Research Institute of Tuberculosis and Lung Diseases (NRITLD), Shahid Beheshti University of Medical Sciences, Tehran, Iran, Islamic Republic of (Javanmard) Department of Internal Medicine, SUNY Stony Brook University, Stony Brook, NY, United States

(Masjedi) Chronic Respiratory Diseases Research Center, National Research Institute of Tuberculosis and Lung Diseases (NRITLD), Shahid Beheshti University of Medical Sciences, Tehran, Iran, Islamic Republic of

Publisher

BioMed Central Ltd.

Emtree Heading

adverse drug reaction; clinical feature; *diabetes mellitus/di [Diagnosis]; *diabetes mellitus/ep [Epidemiology]; disease activity; disease association; drug blood level; human; hyperglycemia; incidence; latent tuberculosis; liver toxicity/si [Side Effect]; multidrug resistant tuberculosis/dr [Drug Resistance]; prevalence; radiography; review; risk assessment; risk factor; screening; sputum culture; treatment outcome; treatment response; *tuberculosis/ep [Epidemiology]; *tuberculosis/pc [Prevention]; *tuberculosis/dt [Drug Therapy]; ethambutol/dt [Drug Therapy]; isoniazid/dt [Drug Therapy]; pyrazinamide/dt [Drug Therapy]; rifampicin/cr [Drug Concentration]; rifampicin/dt [Drug Therapy]; sulfonylurea/cr [Drug Concentration]; tuberculostatic agent/cr [Drug Concentration]; tuberculostatic agent/ae [Adverse Drug Reaction]; tuberculostatic agent/dt [Drug Therapy].

Link to the Ovid Full Text or citation:

Click here for full text options

128.

Tuberculosis and diabetes mellitus: Time for an integrated approach.

Babu S., Salve H., Krishnan A.

National Medical Journal of India. 26 (6) (pp 342-343), 2013. Date of Publication: November/December 2013.

AN: 2014479332

Institution

(Babu, Salve, Krishnan) Centre for Community Medicine, All India Institute of Medical Sciences, New Delhi, India

Publisher

All India Institute of Medical Sciences

Emtree Heading

Australia; cohort analysis; comorbidity; *diabetes mellitus; disease association; disease predisposition; disease registry; disease transmission; dose response; health center; human; impaired glucose tolerance; *integrated health care system; mass screening; note; opportunistic infection; prevalence; relapse; sputum culture; treatment outcome; *tuberculosis; hemoglobin A1c/ec [Endogenous Compound].

Link to the Ovid Full Text or citation:

Click here for full text options

129.

Cohort monitoring - As a tool to improve diabetes care services.

Kapur A., Harries A.D.

Diabetes Research and Clinical Practice. 102 (3) (pp 260-264), 2013. Date of Publication: December 2013.

AN: 2013773681

The need to stem the rising tide of non-communicable diseases (NCDs) including diabetes has been recognised at the highest levels through the UN political declaration. Diabetes care services are largely unavailable in the primary care setting in most developing countries and where available the services are unstructured, with poor record keeping, stock outs and frequent disruption in supplies. With no systematic monitoring of care and programme implementation, treatment outcomes are poor and are consequently associated with a high economic burden. Systematic evaluation of programmes through cohort monitoring has been shown to be effective in large-scale interventions for two chronic infectious diseases-tuberculosis and HIV/AIDS. Can the same simple tool of cohort monitoring be applied to improve diabetes care delivery in the developing world? Pilot projects show it is possible, but scale up and expansion would require investment in information technology. In a scenario where systems for NCD are just beginning to be set up, it makes sense to learn from and build further on the initial pilot programmes. © 2013 Elsevier Ireland Ltd.

Institution

(Kapur) World Diabetes Foundation, Gentofte, Denmark (Harries) International Union Against Tuberculosis and Lung Diseases, Paris, France

(Harries) London School of Hygiene and Tropical Medicine, London, United Kingdom

Publisher

Elsevier Ireland Ltd (P.O. Box 85, Limerick, Ireland)

Emtree Heading

cause of death; chronic disease; *cohort monitoring; *diabetes mellitus; disease registry; electronic medical record; health program; *health service; human; intervention study; non communicable disease; note; *patient monitoring; risk factor; treatment outcome; vital sign.

Link to the Ovid Full Text or citation:

Click here for full text options

130.

Prevalence of type 2 diabetes among newly detected pulmonary tuberculosis patients in china: A community based cohort study.

Wang Q., Ma A., Han X., Zhao S., Cai J., Ma Y., Zhao J., Wang Y., Dong H., Zhao Z., Wei L., Yu T., Chen P., Schouten E.G., Kok F.J., Kapur A.

PLoS ONE. 8 (12) (no pagination), 2013. Article Number: e82660. Date of Publication: 18 Dec 2013.

AN: 2014080630

Background: Patients with type 2 diabetes (DM) have a higher risk of developing pulmonary tuberculosis (PTB); moreover, DM co-morbidity in PTB is associated with poor PTB treatment outcomes. Community based prevalence data on DM and prediabetes (pre-DM) among TB patients is lacking, particularly from the developing world. Therefore we conducted a prospective study to investigate the prevalence of DM and pre-DM and evaluated the risk factors for the presence of DM among newly detected PTB patients in rural areas of China. Methods and Findings: In a prospective community based study carried out from 2010 to 2012, a representative sample of 6382 newly detected PTB patients from 7 TB clinics in Linyi were tested for DM. A population of 6674 non-TB controls from the same community was similarly tested as well. The prevalence of DM in TB patients (6.3%) was higher than that in non-TB controls (4.7%, p<0.05). PTB patients had a higher odds of DM than non-TB controls (adjusted OR 3.17, 95% CI 1.14-8.84). The prevalence of DM increased with age and was significantly higher in TB patients in the age categories above 30 years (p<0.05). Among TB patients, those with normal weight (BMI 18.5-23.9) had the lowest prevalence of DM (5.8%). Increasing age, family history of DM, positive sputum smear, cavity on chest X-ray and higher yearly income (>10000 RMB yuan) were positively associated and frequent outdoor activity was negatively associated with DM in PTB patients. Conclusions: The prevalence of DM in PTB patients was higher than in non-TB controls with a 3 fold higher adjusted odds ratio of having DM. Given the increasing DM prevalence and still high burden of TB in China, this association may represent a new public health challenge concerning the prevention and treatment of both diseases. © 2013 Wang et al.

Institution

(Wang, Ma, Han, Cai) Nutrition Institute, Qingdao University, Qingdao, China (Zhao, Ma) Linyi Chest Hospital, Linyi, China

(Zhao) Yishui Tuberculosis Clinic, Linyi, China

(Wang) Tancheng Tuberculosis Clinic, Linyi, China

(Dong) Yinan Tuberculosis Clinic, Linyi, China

(Zhao) Lanshan Tuberculosis Clinic, Linyi, China

(Wei) Feixian Tuberculosis Clinic, Linyi, China

(Yu) Pingyi Tuberculosis Clinic, Linyi, China

(Chen) Cangshan Tuberculosis Clinic, Linyi, China

(Schouten, Kok) Division of Human Nutrition, Wageningen University, Wageningen, Netherlands

(Kapur) World Diabetes Foundation, Gentofte, Denmark

Publisher

Public Library of Science (185 Berry Street, Suite 1300, San Francisco CA 94107, United States)

Emtree Heading

adult; age distribution; article; body mass; China; cholesterol blood level; cohort analysis; controlled study; family history; female; glucose blood level; human; income; lung cavity; *lung tuberculosis; major clinical study; male; *non insulin dependent diabetes mellitus/ep [Epidemiology]; obesity; prevalence; prospective study; risk assessment; risk factor; rural area; sputum smear; triacylglycerol blood level; underweight; cholesterol/ec [Endogenous Compound]; glucose/ec [Endogenous Compound]; triacylglycerol/ec [Endogenous Compound].

Link to the Ovid Full Text or citation:

Click here for full text options

131.

Risk factors for poor treatment outcomes in patients with MDR-TB and XDR-TB in China: Retrospective multi-center investigation.

Tang S., Tan S., Yao L., Li F., Li L., Guo X., Liu Y., Hao X., Li Y., Ding X., Zhang Z., Tong L., Huang J.

PLoS ONE. 8 (12) (no pagination), 2013. Article Number: 0082943. Date of Publication: 05 Dec 2013.

AN: 2014028091

Background: The treatment of patients with MDR- and XDR-TB is usually more complex, toxic and costly and less effective than treatment of other forms of TB. However, there is little information available on risk factors for poor outcomes in patients with MDR- and XDR-TB in China. Methodology/Principal Findings: We retrospectively analyzed the clinical records of HIV-negative TB Patients with culture-proven MDR- or XDR-TB who were registered from July 2006 to June 2011 at five large-scale Tuberculosis Specialized Hospitals in China. Among 1662 HIV-seronegative TB cases which were culture-positive for M. tuberculosis complex and had positive sputum-smear microscopy results, 965 cases (58.1%) were DR-TB, and 586 cases (35.3%) were classified as having MDR-TB, accounting for 60.7% of DR-TB. 169 cases (10.2%) were XDRTB, accounting for 17.5% of DR-TB, 28.8% of MDR-TB. The MDR-TB patients were divided into XDR-TB group (n=169) and other MDR-TB group (non-XDR MDR-TB) (n=417). In total, 240 patients (40.95%) had treatment success, and 346 (59.05%) had poor treatment outcomes. The treatment success rate in other MDR-TB group was 52.2%, significantly higher than that in the XDR-TB group (13%, P<0.001). In multivariate logistic regression analysis, poor outcomes were associated with duration of previous anti-TB treatment of more than one year (OR, 0.077; 95% CI, 0.011-0.499, P<0.001), a BMI less than 18.5 kg/m2 (OR, 2.185; 95% CI, 1.372-3.478, P<0.001), XDR (OR, 13.368; 95% CI, 6.745-26.497, P<0.001), retreatment (OR, 0.171; 95% CI, 0.093-0.314, P<0.001), diabetes (OR, 0.305; 95% CI, 0.140-0.663, P=0.003), tumor (OR, 0.095; 95% CI, 0.011-0.795, P=0.03), decreased albumin (OR, 0.181; 95% CI, 0.118-0.295, P<0.001), cavitation (OR, 0.175; 95% CI, 0.108-0.286, P<0.001). Conclusions/Significance: The patients with MDR-TB and XDR-TB have poor treatment outcomes in China.The presence of extensive drug resistance, low BMI, hypoalbuminemia, comorbidity, cavitary disease and previous anti- TB treatment are independent prognostic factors for poor outcome in patients with MDR-TB. © 2013 Tang et al.

Institution

(Tang, Huang) Department of Respiratory Medicine, First Affiliated Hospital, Soochow University, Suzhou, China (Tang, Yao, Liu, Hao, Zhang) Tuberculosis Center for Diagnosis and Treatment, Shanghai Pulmonary Hospital, Tongji University School of Medicine, Shanghai, China

(Tan, Li, Ding, Tong) Department of Tuberculosis, Guangzhou Chest Hospital, State Key Laboratory of Respiratory Disease, Guangzhou, China

(Li) Hangzhou Red Cross Hospital, Hangzhou, China

(Li) Tianjin Haihe Hospital, Tianjin, China

(Guo) Henan Infectious Hospital, Zhengzhou, China

Publisher

Public Library of Science (185 Berry Street, Suite 1300, San Francisco CA 94107, United States)

Emtree Heading

adult; adverse outcome; albumin blood level; article; bacterium culture; bacterium identification; body mass; China; comorbidity; diabetes mellitus; disease association; disease classification; drug resistant tuberculosis/co [Complication]; drug resistant tuberculosis/di [Diagnosis]; drug resistant tuberculosis/dr [Drug Resistance]; drug resistant tuberculosis/dt [Drug Therapy]; drug resistant tuberculosis/ep [Epidemiology]; drug resistant tuberculosis/et [Etiology]; *extensively drug resistant tuberculosis/co [Complication]; *extensively drug resistant tuberculosis/di [Diagnosis]; *extensively drug resistant tuberculosis/dr [Drug Resistance]; *extensively drug resistant tuberculosis/dt [Drug Therapy]; *extensively drug resistant tuberculosis/ep [Epidemiology]; *extensively drug resistant tuberculosis/et [Etiology]; female; health survey; human; lung cavitation; major clinical study; male; microscopy; middle aged; minimum inhibitory concentration; morbidity; multicenter study; *multidrug resistant tuberculosis/co [Complication]; *multidrug resistant tuberculosis/di [Diagnosis]; *multidrug resistant tuberculosis/dr [Drug Resistance]; *multidrug resistant tuberculosis/dt [Drug Therapy]; *multidrug resistant tuberculosis/ep [Epidemiology]; *multidrug resistant tuberculosis/et [Etiology]; Mycobacterium tuberculosis; nonhuman; outcome assessment; *prognosis; retrospective study; risk factor; sputum smear; treatment failure; amikacin/an [Drug Analysis]; capreomycin/an [Drug Analysis]; ethambutol/an [Drug Analysis]; isoniazid/an [Drug Analysis]; ofloxacin/an [Drug Analysis]; rifampicin/an [Drug Analysis]; streptomycin/an [Drug Analysis]; tuberculostatic agent/dt [Drug Therapy].

Link to the Ovid Full Text or citation:

Click here for full text options

132.

Clinical challenges in the co-management of diabetes mellitus and tuberculosis in southern Africa.

Reid M.J.A., McFadden N., Tsima B.M.

Journal of Endocrinology, Metabolism and Diabetes of South Africa. 18 (3) (pp 135-140), 2013. Date of Publication: 2013.

AN: 2014035641

Over the past 20 years, tuberculosis incidence in southern Africa has increased at an alarming rate, fuelled primarily by the human immunodeficiency virus epidemic. The emerging prevalence of diabetes mellitus in the region represents a new threat to tuberculosis control. The intersecting double burden is a cause for concern since diabetes mellitus increases the risk of tuberculosis and results in poor treatment outcomes. This review article discusses the evidence of a causal association between these two conditions, and examines the numerous clinical challenges that relate to tuberculosis and diabetes mellitus co-management. Diabetes is associated with a more advanced age and body weight in patients with tuberculosis, although not with a specific clinical presentation of tuberculosis. Rifampicin adversely alters glycaemic control by lowering the concentrations of most oral antidiabetic drugs. Poor glycaemic control, possibly exacerbated by tuberculosis and anti-tuberculous therapy, is an important contributing factor to tuberculosis case fatality and relapse. Clinicians need to be aware of these clinical and pharmacological challenges when co-managing these complex diseases. © SEMDSA.

Institution

(Reid, McFadden) Perleman School of Medicine, University of Pennsylvania, United States (Reid, Tsima) School of Medicine, University of Botswana, Botswana

Publisher

South African Medical Association (Private Bag X1, Pinelands 7430, South Africa)

Emtree Heading

*Africa; body weight; clinical feature; *diabetes mellitus/dt [Drug Therapy]; *diabetes mellitus/ep [Epidemiology]; disease association; disease exacerbation; epidemic; fatality; glycemic control; human; Human immunodeficiency virus; incidence; infection risk; public health; recurrence risk; review; treatment outcome; *tuberculosis/dt [Drug Therapy]; *tuberculosis/ep [Epidemiology]; tuberculosis control; oral antidiabetic agent/cr [Drug Concentration]; oral antidiabetic agent/dt [Drug Therapy]; oral antidiabetic agent/iv [Intravenous Drug Administration]; rifampicin/dt [Drug Therapy].

Link to the Ovid Full Text or citation:

Click here for full text options

133.

Poor performance status is a strong predictor for death in patients with smear-positive pulmonary TB admitted to two Japanese hospitals.

Horita N., Miyazawa N., Yoshiyama T., Kojima R., Omori N., Kaneko T., Ishigatsubo Y.

Transactions of the Royal Society of Tropical Medicine and Hygiene. 107 (7) (pp 451-456), 2013. Article Number: trt037. Date of Publication: July 2013.

AN: 2013767794

Background: Estimation of performance status (PS) has been assessed as a tool to determine which patients with newly diagnosed pulmonary TB (PTB) are most at risk of dying. This simple prediction rule has not been validated in patients with PTB with different background characteristics and from different geographic areas. Methods: A retrospective cohort study was conducted in two Japanese hospitals in different regions and included 432 inpatients with newly diagnosed smear-positive non-multidrug-resistant lung TB without HIV infection. The patients had a mean +/- SD age of 64.9 +/- 19.7 years and 135 were female (31.3%). Detailed nursing charts were reviewed to estimate PS, which was graded 0 (best condition), 1, 2, 3 or 4 (worst condition), for each patient. Results: Single variable and multivariable Cox regression analyses models revealed that a one-point increase in PS was associated with a 2.8-fold (95% CI 2.2-3.6) and 2.3-fold (95% CI 1.8-3.0), adjusted for age, gender, comorbidities and treatment regimen, increase in the HR for death (p < 0.001 for both models). Kaplan-Meier curves also showed a significant difference in mortality among different PS groups (p < 0.001). Conclusion: PS was strongly associated with mortality from PTB in the study cohort. Estimation of PS at the start of treatment for newly diagnosed PTB patients could be a useful tool in case management in resource-limited countries. © Royal Society of Tropical Medicine and Hygiene 2013. All rights reserved.

Institution

(Horita, Ishigatsubo) Department of Internal Medicine and Clinical Immunology, Yokohama City University Graduate School of Medicine, Yokohama, Japan (Horita, Miyazawa, Kojima, Omori) Department of Respiratory Medicine, Saiseikai Yokohamashi Nanbu Hospital, Yokohama, Japan

(Yoshiyama) Division of Respiratory Medicine, Japan Antituberculosis Association Fukujuji Hospital, Tokyo, Japan

(Kaneko) Respiratory Disease Center, Yokohama City University Medical Center, Yokohama, Japan

Publisher

Oxford University Press (Great Clarendon Street, Oxford OX2 6DP, United Kingdom)

Emtree Heading

adult; aged; article; cause of death; chronic kidney disease; chronic liver disease; chronic respiratory tract disease; clinical observation; cohort analysis; comorbidity; daily life activity; diabetes mellitus; dying; female; follow up; *health status; heart disease; human; Japanese; *lung tuberculosis; major clinical study; male; *mortality; *performance status; prognosis; retrospective study; treatment outcome; X ray; aspartate aminotransferase/ec [Endogenous Compound]; creatinine/ec [Endogenous Compound]; hemoglobin/ec [Endogenous Compound].

Link to the Ovid Full Text or citation:

Click here for full text options

134.

Profile of HIV-infected patients receiving second-line antiretroviral therapy in a resource-limited setting in Nigeria.

Onyedum C.C., Iroezindu M.O., Chukwuka C.J., Anyaene C.A., Obi F.I., Young E.E.

Transactions of the Royal Society of Tropical Medicine and Hygiene. 107 (10) (pp 608-614), 2013. Article Number: trt071. Date of Publication: October 2013.

AN: 2013767756

Background: Second-line antiretroviral therapy (ART) accounts for lessthan5%of totalARTin resource-limited settings. We described the baseline characteristics, reasons for switch and treatment outcomes of Nigerian patients receiving second-line ART. Methods: In this retrospective cohort study we recorded the baseline characteristics of HIV-infected adults whose treatment regimen was switched from a non-nucleoside reverse transcriptase inhibitor, a first-line agent, to a protease inhibitor-based second-line regimen. The duration of follow-up was 12 months. Results: Of 4229 patients who started first-line therapy, 186 (4.4%) were switched to second-line therapy after a mean duration of 16.6+/-7.6 months. Their mean age was 41.8+/-9.6 years and 59.1% were women. The median (range) viral load andCD4 cell counts at switch were 4.7 (4.1-6.3) log10 copies/ml and 71 (6-610) cells/mul, respectively. The predominantreason for switchwas virological failure (79.0%). Only55.4%and36.6%of patientshadCD4 cell count and viral load at 12 months. About 82%, 79% and 82% of patients with available data achieved virological suppression at 3 months, 6 months and 12 months respectively (p 1/4 0.81). The proportion of patients who achieved >50%rise in CD4 cell count increased from55.8% at 3 months to 78.6% at 12 months (p 1/4 0.0002). Conclusion: The rate of switch to second-line therapy was low but there were good treatment outcomes among patients with available data. Attrition rate was high. Regular viral load monitoring, improved availability/affordability of second-line regimens and retention in care should become priorities in resource-limited settings. © The Author 2013. Published by Oxford University Press on behalf of Royal Society of Tropical Medicine and Hygiene. All rights reserved.

Institution

(Onyedum, Iroezindu, Chukwuka, Young) Department of Medicine, College of Medicine, University of Nigeria Teaching Hospital (UNTH), Enugu, Nigeria (Anyaene) APIN/CDC Centre, UNTH, Nigeria

(Obi) Department of Community Medicine, UNTH, Nigeria

Publisher

Oxford University Press (Great Clarendon Street, Oxford OX2 6DP, United Kingdom)

Emtree Heading

adolescent; adult; alcohol consumption; antibiotic prophylaxis; antiviral resistance; *antiviral therapy; article; blood transfusion; CD4 lymphocyte count; cohort analysis; comorbidity; diabetes mellitus; drug substitution; drug treatment failure; educational status; female; follow up; heterosexuality; *highly active antiretroviral therapy; human; Human immunodeficiency virus 1; *Human immunodeficiency virus 1 infection/dt [Drug Therapy]; Human immunodeficiency virus 2; *Human immunodeficiency virus 2 infection/dt [Drug Therapy]; Human immunodeficiency virus infected patient; hypertension; immune response; limit of detection; major clinical study; male; marriage; medication compliance; Nigeria; nonhuman; patient compliance; pneumonia; retrospective study; *second line antiretroviral therapy; treatment duration; treatment outcome; tuberculosis; virus load; virus transmission; world health organization; cotrimoxazole; efavirenz plus emtricitabine plus tenofovir disoproxil/dt [Drug Therapy]; emtricitabine plus tenofovir disoproxil/cb [Drug Combination]; emtricitabine plus tenofovir disoproxil/dt [Drug Therapy]; *Human immunodeficiency virus proteinase inhibitor/dt [Drug Therapy]; lamivudine plus nevirapine plus stavudine/dt [Drug Therapy]; lamivudine plus nevirapine plus zidovudine/dt [Drug Therapy]; lopinavir plus ritonavir/cb [Drug Combination]; lopinavir plus ritonavir/dt [Drug Therapy]; *nonnucleoside reverse transcriptase inhibitor/dt [Drug Therapy]; tenofovir plus emtricitabine plus nevirapine/dt [Drug Therapy]; unclassified drug; virus RNA/ec [Endogenous Compound]; zidovudine/cb [Drug Combination]; zidovudine/dt [Drug Therapy].

Link to the Ovid Full Text or citation:

Click here for full text options

135.

Clinical and laboratory characteristics of penicilliosis marneffei among patients with and without HIV infection in Northern Thailand: A retrospective study.

Kawila R., Chaiwarith R., Supparatpinyo K.

BMC Infectious Diseases. 13 (1) (no pagination), 2013. Article Number: 464. Date of Publication: 05 Oct 2013.

AN: 2013718369

Background: Penicilliosis marneffei is increasingly observed in individuals without HIV infection. This study aimed to compare the clinical and laboratory features among HIV infected and uninfected individuals with penicilliosis marneffei.Methods: A retrospective cohort study was conducted between January 1, 2007 and December 31, 2011 at Chiang Mai University Hospital. We included individuals who were >15 years of age and presented with culture-proven P. marneffei infection.Results: 116 HIV-infected and 34 HIV-uninfected patients were enrolled. Comparing to HIV-infected patients, HIV-uninfected patients were older; less likely to have fever, splenomegaly, and umbilicated skin lesions; more likely to have Sweet's syndrome and bone and joint infections; had higher white blood cell count, platelet count, and CD4 cell count; had lower alanine transaminase (ALT); and less likely to have positive fungal blood cultures. The mortality rates were 20.7% and 29.4% among HIV infected and uninfected patients, respectively.Conclusions: Clinical manifestations of penicilliosis marneffei are different between patients with and without HIV infection. Physician's awareness of this disease in HIV-uninfected patients may prompt the diagnosis and timely treatment, and can lead to a better outcome. © 2013 Kawila et al.; licensee BioMed Central Ltd.

Institution

(Kawila, Chaiwarith, Supparatpinyo) Department of Medicine, Faculty of Medicine, Chiang Mai University, Chiang Mai 50200, Thailand (Supparatpinyo) Research Institute for Health Sciences, Faculty of Medicine, Chiang Mai University, Chiang Mai, Thailand

Publisher

BioMed Central Ltd. (Floor 6, 236 Gray's Inn Road, London WC1X 8HB, United Kingdom)

Emtree Heading

acute febrile neutrophilic dermatosis; adolescent; adult; age; article; bone infection; candidiasis/co [Complication]; CD4 lymphocyte count; clinical feature; cohort analysis; colon cancer; comorbidity; convalescence; cryptococcosis/co [Complication]; cytomegalovirus infection/co [Complication]; diabetes mellitus; female; fever; fungus culture; herpes zoster/co [Complication]; histoplasmosis/co [Complication]; human; *Human immunodeficiency virus infection; infectious arthritis; isosporiasis/co [Complication]; leukocytosis; lymphoma; major clinical study; male; mixed connective tissue disease/dt [Drug Therapy]; mortality; myasthenia gravis; nocardiosis/co [Complication]; opportunistic infection/co [Complication]; *penicilliosis/di [Diagnosis]; *penicilliosis/dt [Drug Therapy]; *penicilliosis/et [Etiology]; Penicillium marneffei; retrospective study; salmonellosis/co [Complication]; skin defect; splenomegaly; systemic lupus erythematosus/dt [Drug Therapy]; Thailand; thrombocytosis; treatment duration; treatment outcome; tuberculosis/co [Complication]; university hospital; alanine aminotransferase/ec [Endogenous Compound]; amphotericin B deoxycholate/dt [Drug Therapy]; amphotericin B deoxycholate/iv [Intravenous Drug Administration]; azathioprine/dt [Drug Therapy]; CD4 antigen/ec [Endogenous Compound]; itraconazole/dt [Drug Therapy]; itraconazole/po [Oral Drug Administration]; prednisolone/dt [Drug Therapy].

Link to the Ovid Full Text or citation:

Click here for full text options

136.

Diabetes mellitus and smoking among tuberculosis patients in a tertiary care centre in Karnataka, India.

Jali M.V., Mahishale V.K., Hiremath M.B., Satyanarayana S., Kumar A.M.V., Nagaraja S.B., Isaakidis P.

Public Health Action. 3 (SUPPL.1) (pp S51-S53), 2013. Date of Publication: November 2013.

AN: 2013709957

Diabetes mellitus (DM) and smoking are risk factors for adverse outcomes in the treatment of tuberculosis (TB). In a tertiary care hospital at Belgaum in the South Indian State of Karnataka, all TB patients aged >18 years consecutively diagnosed from February to September 2012 were evaluated for DM and smoking. Of 307 TB patients, 35.5% were found to have DM, 9.8% were current smokers, and 3.6% had DM and were also smokers. Measures to assess and address both these factors need to be taken into account during TB treatment. © 2013 The Union.

Institution

(Jali, Hiremath) KLES Diabetes Centre, KLES Dr Prabhakar Kore Hospital and Medical Research Centre, Belgaum, Karnataka, India (Mahishale) Department of Pulmonary Medicine, KLES Dr Prabha-kar Kore Hospital and Medical Research Centre, Belgaum, Karnataka, India

(Satyanarayana, Kumar) International Union Against Tuberculosis and Lung Disease, South-East Asia Office, New Delhi, India

(Nagaraja) Employees State Insurance Corporation (ESIC), Post Graduate Institute of Medical Science and Research and Model Hospital, Rajaji Nagar, Bangalore, India

(Isaakidis) Medecins Sans Frontieres, Mumbai, India

Publisher

International Union Against Tuberculosis and Lung Disease (68 boulevard Saint Michel, Paris 75006, France)

Emtree Heading

adult; aged; article; comorbidity; cross-sectional study; *diabetes mellitus/ep [Epidemiology]; extrapulmonary tuberculosis; female; glucose blood level; human; Human immunodeficiency virus infection; impaired fasting glucose; India; major clinical study; male; multidrug resistant tuberculosis; prevalence; random blood glucose level; retreatment; risk factor; *smoking; smoking cessation; sputum smear; treatment outcome; *tuberculosis.

Link to the Ovid Full Text or citation:

Click here for full text options

137.

New screening technologies for type 2 diabetes mellitus appropriate for use in tuberculosis patients.

Adepoyibi T., Weigl B., Greb H., Neogi T., McGuire H.

Public Health Action. 3 (SUPPL.1) (pp S10-S17), 2013. Date of Publication: November 2013.

AN: 2013709949

Type 2 diabetes mellitus (DM), which is epidemic in lowand middle-income countries (LMICs), may threaten gains made in tuberculosis (TB) control, as DM is both a major risk factor for developing active TB and it can lead to adverse TB treatment outcomes. Despite World Health Organization guidance that all TB patients should be screened for DM, most facilities in LMICs that manage TB patients do not currently perform screening for DM, due in part to the cost and complexity involved. DM screening is further complicated by the presentation of transient hyperglycemia in many TB patients, as well as differences in diabetes risk factors (e.g., body mass index) between TB patients and the general public. In this article, we review existing and new technologies for DM screening that may be more suitable for TB patients in LMICs. Such methods should be rapid, they should not require fasting, and they should allow the provider to differentiate between transient and longer-term hyperglycemia, using inexpensive tools that require little training and no specialized infrastructure. Several methods that are currently under development, such as point-of-care glycated hemoglobin and glycated albumin assays, non-invasive advanced glycation end-product readers, and sudomotor function-based screening devices, offer interesting performance characteristics and warrant evaluation in populations with TB. © 2013 The Union.

Institution

(Adepoyibi, Weigl, Greb, Neogi, McGuire) PATH, Washington, DC, United States (Neogi) University of Washington School of Medicine, Department of Family Medicine, Seattle, WA, United States

Publisher

International Union Against Tuberculosis and Lung Disease (68 boulevard Saint Michel, Paris 75006, France)

Emtree Heading

chemical analysis; disposable equipment; electrochemical detection; epidemic; glucose blood level; human; neuropathy; *non insulin dependent diabetes mellitus/di [Diagnosis]; non invasive measurement; oral glucose tolerance test; *point of care testing; questionnaire; review; risk assessment; screening; sensitivity and specificity; sweating; systematic review (topic); *tuberculosis; world health organization; advanced glycation end product/ec [Endogenous Compound]; biological marker; glycosylated albumin/ec [Endogenous Compound]; hemoglobin A1c/ec [Endogenous Compound].

Link to the Ovid Full Text or citation:

Click here for full text options

138.

Nutritional Status of Adult Patients with Pulmonary Tuberculosis in Rural Central India and Its Association with Mortality.

Bhargava A., Chatterjee M., Jain Y., Chatterjee B., Kataria A., Bhargava M., Kataria R., D'Souza R., Jain R., Benedetti A., Pai M., Menzies D.

PLoS ONE. 8 (10) (no pagination), 2013. Article Number: e77979. Date of Publication: 24 Oct 2013.

AN: 2013674017

Under-nutrition is a known risk factor for TB and can adversely affect treatment outcomes. However, data from India are sparse, despite the high burden of TB as well as malnutrition in India. We assessed the nutritional status at the time of diagnosis and completion of therapy, and its association with deaths during TB treatment, in a consecutive cohort of 1695 adult patients with pulmonary tuberculosis in rural India during 2004 - 2009.Multivariable logistic regression was used to obtain adjusted estimates of the association of nutritional status with deaths during treatment. At the time of diagnosis, median BMI and body weights were 16.0 kg/m2and 42.1 kg in men, and 15.0 kg/m2and 34.1 kg in women, indicating that 80% of women and 67% of men had moderate to severe under-nutrition (BMI<17.0 kg/m2). Fifty two percent of the patients (57% of men and 48% of women) had stunting indicating chronic under-nutrition. Half of women and one third of men remained moderately to severely underweight at the end of treatment. 60 deaths occurred in 1179 patients (5%) in whom treatment was initiated. Severe under-nutrition at diagnosis was associated with a 2 fold higher risk of death. Overall, a majority of patients had evidence of chronic severe under-nutrition at diagnosis, which persisted even after successful treatment in a significant proportion of them. These findings suggest the need for nutritional support during treatment of pulmonary TB in this rural population. © 2013 Bhargava et al.

Institution

(Bhargava) Department of Internal Medicine, Himalayan Institute of Medical Sciences, Jolly Grant, Uttarakhand, India (Chatterjee) Krishak Maitri Hospital, Chengail, West Bengal, India

(Jain, Kataria, Kataria, Jain) Jan Swasthya Sahyog, Village and P.O.Ganiyari, Chhattisgarh, India

(Chatterjee) Department of Microbiology, Himalayan Institute of Medical Sciences, Jolly Grant, Uttarakhand, India

(Bhargava) Department of Community Medicine, Himalayan Institute of Medical Sciences, Jolly Grant, Uttarakhand, India

(D'Souza) Centre for Public Health and Equity, Bhopal, Madhya Pradesh, India

(Benedetti, Menzies) Respiratory Epidemiology and Clinical Research Unit, Montreal Chest Institute, McGill University Health Centre, Montreal, QC, Canada

(Pai) Department of Epidemiology, Biostatistics and Occupational Health, McGill University, Montreal, QC, Canada

Publisher

Public Library of Science (185 Berry Street, Suite 1300, San Francisco CA 94107, United States)

Emtree Heading

adult; age distribution; anemia; article; body height; body mass; body weight; cohort analysis; comorbidity; controlled study; diabetes mellitus; disease association; female; human; Human immunodeficiency virus infection; India; *lung tuberculosis/ep [Epidemiology]; major clinical study; male; malnutrition; *mortality; *nutritional status; nutritional support; prevalence; retrospective study; rural area; sex difference; treatment outcome; underweight.

Link to the Ovid Full Text or citation:

Click here for full text options

139.

Tuberculosis and diabetes mellitus interaction: A current clinical perspective.

Kibirige D., Ssekitoleko R., Worodria W.

Infectious Diseases in Clinical Practice. 21 (6) (pp 355-358), 2013. Date of Publication: November 2013.

AN: 2013703849

ABSTRACT: The disease burden from tuberculosis (TB) and diabetes mellitus (DM) is increasing globally. Current evidence suggests that DM increases the odds of developing TB. This risk is highest in the low- and middle-income countries, where the burden of TB is high. Immune dysfunction due to DM increases the propensity to develop TB. Both DM and TB complicate each other and present enormous clinical challenges. This review article discusses the close interaction between the 2 comorbidities and also advocate for consideration of integration of dual-screening strategies for DM and TB in clinical care especially in areas with high prevalence of both diseases. © 2013 by Lippincott Williams & Wilkins.

Institution

(Kibirige) Department of Medicine, St Raphael of St Francis Hospital Nsambya, PO Box 7146, Kampala, Uganda (Ssekitoleko) Infectious Diseases Unit, Mulago National Referral and Teaching Hospital, Kampala, Uganda

(Ssekitoleko, Worodria) Department of Medicine, Makerere University College of Health Sciences, Kampala, Uganda

(Worodria) Pulmonology Unit, Mulago National Referral and Teaching Hospital, Kampala, Uganda

Publisher

Lippincott Williams and Wilkins (530 Walnut Street,P O Box 327, Philadelphia PA 19106-3621, United States)

Emtree Heading

comorbidity; *diabetes mellitus/di [Diagnosis]; *diabetes mellitus/dt [Drug Therapy]; *diabetes mellitus/et [Etiology]; diabetic patient; disease activity; disease association; glucose absorption; glycemic control; human; hyperglycemia; intestine absorption; meta analysis (topic); mixed infection; multidrug resistant tuberculosis; oral glucose tolerance test; prevalence; review; risk assessment; risk factor; screening; systematic review (topic); treatment outcome; *tuberculosis/di [Diagnosis]; *tuberculosis/dt [Drug Therapy]; *tuberculosis/et [Etiology]; vitamin D deficiency; glibenclamide/dt [Drug Therapy]; glibenclamide/po [Oral Drug Administration]; gliclazide/dt [Drug Therapy]; gliclazide/po [Oral Drug Administration]; glimepiride/dt [Drug Therapy]; glimepiride/po [Oral Drug Administration]; insulin/ec [Endogenous Compound]; pyrazinamide/dt [Drug Therapy]; rifampicin/dt [Drug Therapy]; vitamin D/ec [Endogenous Compound].

Link to the Ovid Full Text or citation:

Click here for full text options

140.

Adalimumab for the treatment of psoriasis in real life: A retrospective cohort of 119 patients at a single Spanish centre.

Lopez-Ferrer A., Vilarrasa E., Gich I.J., Puig L.

British Journal of Dermatology. 169 (5) (pp 1141-1147), 2013. Date of Publication: November 2013.

AN: 2013696878

Background Patients with moderate-to-severe psoriasis treated with adalimumab in daily clinical practice are different from those in clinical trials, and outcomes may differ in different geographical settings. Objectives To analyse the efficacy, retention of treatment and adverse events in a cohort of such patients at a referral centre in Barcelona, Spain. Methods Data from a cohort of 119 consecutive patients treated between January 2008 and March 2013 were retrospectively collected. Drug survival was analysed by the Kaplan-Meier method with log-rank test and Cox regression. Results The mean duration of treatment was 25 months (median 22, range 2-60). The 75% improvement in Psoriasis Area and Severity Index (PASI 75) response rates at 16 weeks, 6 months and 1 year of treatment were 64%, 58% and 53%, respectively (intention-to-treat analysis). The corresponding PASI 90 values were 49%, 52% and 50%. Biologic-naive patients (41%) had significantly higher PASI 75 and PASI 90 response rates at 6 months and 1 year. On multivariate analysis, only PASI 90 response at 6 months was significantly associated with treatment retention (P = 0.0009), with a hazard ratio of 7.3 (95% confidence interval 2.3-23.6). Forty-eight adverse events (AEs) occurred in 29 patients, and were serious in eight (0.032 events per patient-year). Paradoxical flares of psoriasis or arthritis were seen in five patients. Infections accounted for seven serious AEs, and were the reason for discontinuation in two patients. Conclusions PASI 90 response at 6 months was the only independent variable predicting drug survival on multivariate analysis. Infections, including de novo infection by Mycobacterium tuberculosis, accounted for seven serious AEs. What's already known about this topic? There are few reports on the use of adalimumab for the treatment of moderate-to-severe psoriasis in clinical practice according to the European Medicines Agency. Psoriasis Area and Severity Index (PASI) 75% response rates at 16 weeks and 6 months were approximately 60% in a previously published U.K. series. Male sex and the presence of arthritis have been associated with decreased drug survival in one study. What does this study add? Biologic-naive status and efficacy parameters denoting a good or excellent response appear to be associated with a higher probability of drug survival. Combination treatment increased PASI response rates at 6 months and might provide an explanation for the relatively high rate of PASI 90 responders in our cohort. Infections, including de novo infection by Mycobacterium tuberculosis, accounted for most serious adverse events, and paradoxical flares of psoriasis and psoriatic arthritis were relatively frequent. © 2013 British Association of Dermatologists.

Institution

(Lopez-Ferrer, Vilarrasa, Puig) Department of Dermatology, Hospital de la Santa Creu i Sant Pau, Universitat Autonoma de Barcelona, Sant Antoni Maria Claret 167, 08025 Barcelona, Catalonia, Spain (Gich) Department of Clinical Epidemiology and Public Health, Hospital de la Santa Creu i Sant Pau, Universitat Autonoma de Barcelona, Sant Antoni Maria Claret 167, 08025 Barcelona, Catalonia, Spain

Publisher

Blackwell Publishing Ltd (9600 Garsington Road, Oxford OX4 2XG, United Kingdom)

Emtree Heading

abscess/si [Side Effect]; adult; aged; alcoholism; article; bronchitis/si [Side Effect]; cohort analysis; comparative study; controlled study; diabetes mellitus; disease course; drug efficacy; drug safety; drug withdrawal; dyslipidemia; female; furunculosis/si [Side Effect]; gastroenteritis/si [Side Effect]; heart infarction/si [Side Effect]; herpes simplex/si [Side Effect]; human; hyperglycemia/si [Side Effect]; hypertension/si [Side Effect]; hypertriglyceridemia/si [Side Effect]; injection site reaction/si [Side Effect]; intention to treat analysis; latent tuberculosis; leukopenia/si [Side Effect]; major clinical study; male; medical record review; miliary tuberculosis/si [Side Effect]; muscle cramp/si [Side Effect]; nonalcoholic fatty liver; obesity; open study; paronychia/si [Side Effect]; patient monitoring; patient referral; pneumonia/si [Side Effect]; priority journal; prostatitis/si [Side Effect]; pruritus/si [Side Effect]; *psoriasis/dt [Drug Therapy]; Psoriasis Area and Severity Index; psoriasis vulgaris; psoriatic arthritis/di [Diagnosis]; psoriatic arthritis/dt [Drug Therapy]; retrospective study; scabies/si [Side Effect]; Spain; survival; thrombocytopenia/si [Side Effect]; thrush/si [Side Effect]; treatment duration; treatment outcome; treatment response; tuberculosis/dt [Drug Therapy]; urinary tract infection/si [Side Effect]; vasculitis/si [Side Effect]; *adalimumab/ae [Adverse Drug Reaction]; *adalimumab/ct [Clinical Trial]; *adalimumab/cb [Drug Combination]; *adalimumab/dt [Drug Therapy]; *adalimumab/pd [Pharmacology]; cyclosporin A/cb [Drug Combination]; cyclosporin A/dt [Drug Therapy]; etretin/cb [Drug Combination]; etretin/dt [Drug Therapy]; infliximab/dt [Drug Therapy]; leflunomide/dt [Drug Therapy]; methotrexate/cb [Drug Combination]; methotrexate/dt [Drug Therapy]; tuberculostatic agent/dt [Drug Therapy].

Link to the Ovid Full Text or citation:

Click here for full text options

141.

Mycobacterium tuberculosis infection following kidney transplantation.

Boubaker K., Gargah T., Abderrahim E., Ben Abdallah T., Kheder A.

BioMed Research International. 2013 (no pagination), 2013. Article Number: 347103. Date of Publication: 2013.

AN: 2013680823

Introduction and Aims. Post-transplant tuberculosis (TB) is a problem in successful long-term outcome of renal transplantation recipients. Our objective was to describe the pattern and risk factors of TB infection and the prognosis in our transplant recipients. Patients and Methods. This study was a retrospective review of the records of 491 renal transplant recipients in our hospital during the period from January 1986 to December 2009. The demographic data, transplant characteristics, clinical manifestations, diagnostic criteria, treatment protocol, and long-term outcome of this cohort of patients were analyzed. Results. 16 patients (3,2%) developed post-transplant TB with a mean age of 32,5 +/- 12,7 (range: 13-60) years and a mean post-transplant period of 36,6months (range: 12,3 months-15,9 years). The forms of the diseases were pulmonary in 10/16 (62,6%), disseminated in 3/16 (18,7%), and extrapulmonary in 3/16 (18,7%). Graft dysfunction was observed in 7 cases (43,7%) with tissue-proof acute rejection in 3 cases and loss of the graft in 4 cases. Hepatotoxicity developed in 3 patients (18,7%) during treatment. Recurrences were observed in 4 cases after early stop of treatment. Two patients (12.5%) died. Conclusion. Extra pulmonary and disseminated tuberculosis were observed in third of our patients. More than 9months of treatment may be necessary to prevent recurrence. © 2013 Karima Boubaker et al.

Institution

(Boubaker, Abderrahim, Kheder) Internal Medecine Department, Charles Nicole Hospital, Boulevard 9 Avril, Bab Souika, 1006 Tunis, Tunisia (Gargah) Pediatric Department, Charles Nicole Hospital, Tunisia

(Ben Abdallah) Research Laboratory of Immunology (LR03SP01), Tunisia

Publisher

Hindawi Publishing Corporation (410 Park Avenue, 15th Floor, 287 pmb, New York NY 10022, United States)

Emtree Heading

adolescent; adult; article; Aspergillus; Candida albicans; Cytomegalovirus; diabetic nephropathy; female; glomerulonephritis; human; hypertension; hyperuricemia; interstitial nephritis; kidney failure; *kidney transplantation; liver toxicity; lupus erythematosus nephritis; major clinical study; male; outcome assessment; pathogenesis; prognosis; recurrent disease; renal graft dysfunction; retrospective study; risk assessment; risk factor; treatment duration; *tuberculosis; azathioprine; cyclosporin; ethambutol; isoniazid plus rifampicin; mycophenolic acid 2 morpholinoethyl ester; pyrazinamide; tacrolimus.

Link to the Ovid Full Text or citation:

Click here for full text options

142.

Outcome of Tuberculosis Treatment in Patients with Diabetes Mellitus Treated in the Revised National Tuberculosis Control Programme in Malappuram District, Kerala, India.

KV N., Duraisamy K., Balakrishnan S., M S., S J.S., Sagili K.D., Satyanarayana S., MV A.K., Enarson D.A.

PLoS ONE. 8 (10) (no pagination), 2013. Article Number: e76275. Date of Publication: 14 Oct 2013.

AN: 2013645467

Settings:Kerala State, India has reported the greatest dual burden of Tuberculosis (TB) and Diabetes Mellitus (DM). Malappuram district in Kerala has monitored and recorded DM status and its control from 2010 under Revised National Tuberculosis Control Program (RNTCP).Objectives:To assess, under programme conditions, comprehensiveness of recording DM status among TB cases and the TB treatment outcomes among DM patients (disaggregated by glycemic control) and compare with-non DM patients.Design:This retrospective record review included 3,116TB patients from April 2010 to September 2011.DM was defined as per international guidelines and TB treatment outcomes were categorized as favourable(cured and treatment completed) and unfavourable(death, default, failure and transfer out). Relative Risk (RR) and 95% confidence intervals(CI) were calculated to assess the risk of unfavourable outcomes.Results:DM status was recorded in 90% of TB cases and 667 (24%) had DM. 17% of DM patients and 23% of patients with unknown DM status had unfavourable outcomes but this difference was not statistically significant. Unadjusted RR for poor glycemic control or unknown control status for unfavourable outcome were (2.00; 95% CI 0.97-4.13) and (2.14; 95% CI 1.11-4.13).Conclusion:This study could not confirm an adverse association between DM or its control during treatment and the course of response to TB treatment.DM screening in TB cases and recording of DM care needs to be improved to enable more conclusive evidence. © 2013 KV et al.

Institution

(KV) District Tuberculosis Centre, Manjeri, Malappuram District, Kerala, India (Duraisamy, Balakrishnan) Department of Tuberculosis, WHO Country Office for India, New Delhi, India

(M) State TB Training and Demonstration Centre, Trivandrum, Kerala, India

(S) State TB Cell, Trivandrum, Kerala, India

(Sagili, Satyanarayana, MV) International Union Against Tuberculosis and Lung Disease, South East Asia Regional Office, New Delhi, India

(Enarson) International Union Against Tuberculosis and Lung Disease, Montreal, QC, Canada

Publisher

Public Library of Science (185 Berry Street, Suite 1300, San Francisco CA 94107, United States)

Emtree Heading

adolescent; adult; article; controlled study; death; diabetes control; *diabetes mellitus/di [Diagnosis]; diabetic patient; documentation; female; glycemic control; human; India; major clinical study; male; medical record review; patient care; patient transport; practice guideline; risk assessment; screening; treatment failure; *treatment outcome; *tuberculosis; tuberculosis control.

Link to the Ovid Full Text or citation:

Click here for full text options

143.

Outcome of bilateral adrenalectomy in Cushing's syndrome: A systematic review.

Ritzel K., Beuschlein F., Mickisch A., Osswald A., Schneider H.J., Schopohl J., Reincke M.

Journal of Clinical Endocrinology and Metabolism. 98 (10) (pp 3939-3948), 2013. Date of Publication: October 2013.

AN: 2013638749

Background: The outcome of bilateral adrenalectomy (BADx) in patients with Cushing's syndrome (CS) is not well characterized. Methods: A literature search was performed with the search terms "bilateral adrenalectomy" or "total adrenalectomy" and "Cushing's" or "Cushing." Immediate and long-term outcomes after BADx in CS were analyzed using descriptive statistics (median [range]). Results: From 549 screened publications, 37 studies met inclusion criteria (1320 patients, 82% having Cushing's disease [CD], 13% having ectopic CS, and 5% having primary adrenal hyperplasia). Surgical morbidity and mortality of BADx (23 studies, 739 patients) were 18% (6-31) and 3% (0-15), respectively. In patients with CD, surgical mortality was below 1%. Although residual cortisol secretion due to accessory adrenal tissue or adrenal remnants was found in 3-34% (5 studies, 236 patients), less than 2% had a relapse of CS. Symptoms of hypercortisolism (eg, hypertension, obesity, or depression) improved in the majority of the patients after BADx (7 studies, 195 patients). The number of adrenal crises per 100 patient-years was 9.3 (6 studies, 203 patients). Nelson's syndrome occurred in 21% (0-47) of the patients (24 studies, 768 patients). Mortality (23 studies, 690 patients) was 17% (0-88) ata follow-up of 41 months (14-294). Remarkably, 46% of thepatientsdied in the first year after surgery. The median ages at death were 62 years (CD) and 53 years (ectopic CS). Conclusion: BADx is relatively safe and provides adequate success. Excess mortality within the first year after surgery suggests that intensive clinical care for patients after BADx is warranted. Copyright © 2013 by The Endocrine Society.

Institution

(Ritzel, Beuschlein, Mickisch, Osswald, Schneider, Schopohl, Reincke) Medizinische Klinik und Poliklinik IV, Klinikum der Universitat Munchen, Ziemssenstrasse 1, D-80336 Munchen, Germany

Publisher

Endocrine Society (8401 Connecticut Ave. Suite 900, Chevy Chase MD 20815, United States)

Emtree Heading

adrenal cortex insufficiency/co [Complication]; adrenal hyperplasia; *adrenalectomy; bleeding/co [Complication]; body mass; bronchitis/co [Complication]; cardiovascular disease/co [Complication]; cause of death; cerebrovascular accident/co [Complication]; clinical assessment; comorbidity; corticotropin blood level; *Cushing syndrome/dt [Drug Therapy]; *Cushing syndrome/su [Surgery]; depression; diabetes mellitus; diverticulitis/co [Complication]; emotional disorder; fatigue; follow up; gastrointestinal hemorrhage/co [Complication]; general condition improvement; heart infarction/co [Complication]; high risk patient; human; hydrocortisone release; hydrocortisone urine level; hypercortisolism; hyperpigmentation; hypertension; hypophysis tumor/co [Complication]; incidence; intestine obstruction/co [Complication]; laparoscopic adrenalectomy; laparoscopic surgery; length of stay; lung embolism/co [Complication]; malignant neoplastic disease/co [Complication]; menstrual cycle; morbidity; multiple organ failure/co [Complication]; muscle weakness; myeloid leukemia/co [Complication]; Nelson syndrome/co [Complication]; obesity; phenotype; pneumonia/co [Complication]; postoperative complication; postoperative period; preoperative evaluation; preoperative treatment; priority journal; psychosis/co [Complication]; quality of life; relapse; remission; respiratory failure/co [Complication]; review; septicemia/co [Complication]; suicide/co [Complication]; surgical infection/co [Complication]; surgical infection/dt [Drug Therapy]; surgical infection/pc [Prevention]; surgical injury/co [Complication]; surgical mortality; symptom; systematic review; thromboembolism/co [Complication]; treatment outcome; treatment response; tuberculosis/co [Complication]; urinary tract infection/co [Complication]; adrenergic receptor blocking agent/dt [Drug Therapy]; antibiotic agent/dt [Drug Therapy]; corticotropin/ec [Endogenous Compound]; hydrocortisone/ec [Endogenous Compound]; ketoconazole/dt [Drug Therapy]; metyrapone/dt [Drug Therapy].

Link to the Ovid Full Text or citation:

Click here for full text options

144.

Diabetes mellitus and tuberculosis: Pattern of tuberculosis, two-month smear conversion and treatment outcomes in Guangzhou, China.

Mi F., Tan S., Liang L., Harries A.D., Hinderaker S.G., Lin Y., Yue W., Chen X., Liang B., Gong F., Du J.

Tropical Medicine and International Health. 18 (11) (pp 1379-1385), 2013. Date of Publication: November 2013.

AN: 2013674768

Objective: There is a high burden of both diabetes (DM) and tuberculosis (TB) in China. We evaluated the association between DM and the pattern of disease, 2-month sputum smear conversion and treatment outcomes of patients with TB in Guangzhou, China. Method: All patients registered with TB from September 2011 to June 2012 were screened for DM and assessed for treatment outcomes in relation to presence or absence of DM and quality of DM control using patient registers, treatment cards and electronic record systems. Results: There were 1589 patients with TB of whom 189 (12%) had DM. Among those with DM, there was a significantly higher proportion of men, persons aged 35 years and older and persons with smear-positive pulmonary tuberculosis (PTB) (P < 0.01). In patients with DM and new smear-positive PTB, there was a higher proportion who had positive sputum smears at 2 months (21.7% vs. 5.6%, RR 3.85, 95%CI 2.24-6.63), who were lost-to-follow-up (5.2% vs. 1.7%, RR 3.23, 95%CI 1.08-9.63) and who failed treatment (10.3% vs. 2.3%, RR 4.46, 95%CI 1.96-10.18) compared with patients who had no DM. There was no significant association between these adverse outcomes and DM control as measured by 2 and 6-month fasting blood glucose. Conclusion: Diabetes mellitus in new smear-positive patients with PTB was associated with failure to sputum smear convert at 2 months and adverse treatment outcomes of loss-to-follow-up and failure. Further research is needed to understand the reasons for these findings and to determine whether the current length of treatment of 6 months is adequate. © 2013 John Wiley & Sons Ltd.

Institution

(Mi, Liang, Yue, Chen, Du) Beijing Chest Hospital, Capital Medical University, Beijing, China (Mi, Liang, Yue, Chen, Du) Beijing Tuberculosis and Thoracic Tumor Research Institute, Beijing, China

(Tan, Liang, Gong) Guangzhou Chest Hospital, Guangzhou, China

(Tan, Liang, Gong) State Key Laboratory of Respiratory Diseases, Guangzhou, China

(Harries) International Union Against Tuberculosis and Lung Disease, Paris, France

(Harries) London School of Hygiene and Tropical Medicine, London, United Kingdom

(Hinderaker) University of Bergen, Bergen, Norway

(Lin) The China Union Office, Beijing, China

Publisher

Blackwell Publishing Ltd (9600 Garsington Road, Oxford OX4 2XG, United Kingdom)

Emtree Heading

adult; antibiotic therapy; article; China; clinical feature; cohort analysis; cross-sectional study; demography; diabetes control; *diabetes mellitus/ep [Epidemiology]; disease association; female; follow up; glucose blood level; human; *lung tuberculosis/dt [Drug Therapy]; *lung tuberculosis/ep [Epidemiology]; major clinical study; male; prevalence; retrospective study; sputum smear; treatment outcome; ethambutol/cb [Drug Combination]; ethambutol/dt [Drug Therapy]; glucose/ec [Endogenous Compound]; isoniazid/cb [Drug Combination]; isoniazid/dt [Drug Therapy]; pyrazinamide/cb [Drug Combination]; pyrazinamide/dt [Drug Therapy]; rifampicin/cb [Drug Combination]; rifampicin/dt [Drug Therapy].

Link to the Ovid Full Text or citation:

Click here for full text options

145.

Treatment optimization: An outline for future success.

Flexner C., Plumley B., Brown Ripin D.H.

Current Opinion in HIV and AIDS. 8 (6) (pp 523-527), 2013. Date of Publication: November 2013.

AN: 2013669462

PURPOSE OF REVIEW: In this issue of Current Opinion, the Guest Editors and their colleagues provide a comprehensive overview of current activities aimed at optimizing global HIV treatment. In this introduction, we outline current goals and approaches that will be described in more detail elsewhere in this issue. RECENT FINDINGS: Two recent conferences, the first and second Conference on Antiretroviral Drug Optimization (CADO), brought together experts from academia, governments, foundations, the pharmaceutical industry, and community activists to develop a global HIV-treatment research agenda for the coming decade focused on better therapies and how to make them accessible to a broader population of people living with HIV. Important recommendations included a focus on more efficient process chemistry for antiretroviral drugs, investigation of antiretroviral dose reduction as a possible optimization strategy, recognition of the increasing importance of concurrent infections and comorbidities especially tuberculosis and aging-related diseases, and identifying a highly effective and affordable nontoxic, once-daily fixed-dose combination regimen for first-line treatment. SUMMARY: HIV treatment optimization is a process intended to enhance the long-term efficacy, adherence, tolerability, safety, convenience, and affordability of combination ART. The ultimate goal of this process is to expand access to well tolerated and effective lifetime treatment to all those in need. © 2013 Wolters Kluwer Health.

Institution

(Flexner) Johns Hopkins University School of Medicine and Bloomberg School of Public Health, Osler 527, 600 N, Wolfe Street, Baltimore, MD 21287-5554, United States (Plumley) Pangaea Global AIDS Foundation, Oakland, CA, United States

(Brown Ripin) Clinton Health Access Initiative, Boston, MA, United States

Publisher

Lippincott Williams and Wilkins (250 Waterloo Road, London SE1 8RD, United Kingdom)

Emtree Heading

absence of side effects/si [Side Effect]; *antiviral therapy; cardiovascular disease; comorbidity; concurrent infection; diabetes mellitus; drug bioavailability; drug cost; drug efficacy; drug safety; drug tolerability; drug treatment failure; drug withdrawal; hepatitis B/si [Side Effect]; human; Human immunodeficiency virus; *Human immunodeficiency virus infection/dt [Drug Therapy]; *Human immunodeficiency virus infection/pc [Prevention]; hypertension; low drug dose; medication compliance; mixed infection; patient compliance; phase 3 clinical trial (topic); priority journal; recurrent infection/si [Side Effect]; review; risk reduction; tuberculosis/dt [Drug Therapy]; unspecified side effect/si [Side Effect]; *anti human immunodeficiency virus agent/dt [Drug Therapy]; *anti human immunodeficiency virus agent/pe [Pharmacoeconomics]; cobicistat/cb [Drug Combination]; cobicistat/dt [Drug Therapy]; darunavir/cb [Drug Combination]; darunavir/cm [Drug Comparison]; darunavir/dt [Drug Therapy]; dolutegravir/ct [Clinical Trial]; dolutegravir/cb [Drug Combination]; dolutegravir/dt [Drug Therapy]; efavirenz/cb [Drug Combination]; efavirenz/dt [Drug Therapy]; emtricitabine/ae [Adverse Drug Reaction]; emtricitabine/cb [Drug Combination]; emtricitabine/dt [Drug Therapy]; Human immunodeficiency virus proteinase inhibitor/cm [Drug Comparison]; Human immunodeficiency virus proteinase inhibitor/dt [Drug Therapy]; lamivudine/ae [Adverse Drug Reaction]; lamivudine/cb [Drug Combination]; lamivudine/dt [Drug Therapy]; raltegravir/ae [Adverse Drug Reaction]; raltegravir/dt [Drug Therapy]; rifampicin/dt [Drug Therapy]; ritonavir/cb [Drug Combination]; ritonavir/dt [Drug Therapy]; tenofovir/cb [Drug Combination]; tenofovir/dt [Drug Therapy]; tenofovir/po [Oral Drug Administration]; tenofovir alafenamide/ct [Clinical Trial]; tenofovir alafenamide/cb [Drug Combination]; tenofovir alafenamide/dt [Drug Therapy]; tenofovir disoproxil/dt [Drug Therapy].

Link to the Ovid Full Text or citation:

Click here for full text options

146.

Diabetes mellitus and tuberculosis.

Koo B.K.

Diabetes and Metabolism Journal. 37 (4) (pp 249-251), 2013. Date of Publication: 2013.

AN: 2013588454

Institution

(Koo) Department of Internal Medicine, Boramae Medical Center, Seoul National University College of Medicine, 20 Boramae-ro 5-gil, Dongjak-gu, Seoul 156-707, South Korea

Publisher

Korean Diabetes Association (Room 1010, Renalssance Tower Bldg 456, Gongdeok-dong, Mapo-gu, Seoul 121-706, South Korea)

Emtree Heading

article; bacterial load; *diabetes mellitus/dt [Drug Therapy]; *diabetes mellitus/ep [Epidemiology]; disease association; disease severity; drug treatment failure; geographic distribution; hemoglobin blood level; human; hyperglycemia; incidence; India; infection risk; Korea; nonhuman; prevalence; prognosis; screening test; thorax radiography; *tuberculosis/di [Diagnosis]; *tuberculosis/ep [Epidemiology]; antidiabetic agent/dt [Drug Therapy]; hemoglobin A1c/ec [Endogenous Compound].

Link to the Ovid Full Text or citation:

Click here for full text options

147.

Incidence and risk factors for treatment failure with infliximab in psoriasis.

Escande H., Livideanu C.B., Steiner A., Lahfa M., Marguery M.C., Mazereeuw J.H., Meyer N., Labadie F.G., Aquilina C., Viraben R., Gourraud P.A., Paul C.

Journal of the European Academy of Dermatology and Venereology. 27 (10) (pp 1323-1324), 2013. Date of Publication: October 2013.

AN: 2013616264

Institution

(Escande, Livideanu, Steiner, Lahfa, Marguery, Mazereeuw, Meyer, Labadie, Aquilina, Viraben, Paul) Department of Dermatology, Paul Sabatier University, Toulouse University Hospital, Toulouse, France (Gourraud) Methodomics, Mortagne-sur-Sevre, Vendee, France

Publisher

Blackwell Publishing Ltd (9600 Garsington Road, Oxford OX4 2XG, United Kingdom)

Emtree Heading

alcoholism; anaphylaxis/si [Side Effect]; asthenia/si [Side Effect]; autoimmune hepatitis/si [Side Effect]; cytolysis; depression inventory; diabetes mellitus; drug efficacy; *drug treatment failure; drug withdrawal; dyslipidemia; erythrodermic psoriasis; guttate psoriasis; human; hypertension; letter; lung aspergillosis/si [Side Effect]; major clinical study; miliary tuberculosis/si [Side Effect]; obesity; priority journal; *psoriasis/dt [Drug Therapy]; psoriasis vulgaris; psoriatic arthritis; pustular psoriasis; *risk factor; sex difference; side effect/si [Side Effect]; smoking; treatment outcome; tuberculous lymphadenitis/si [Side Effect]; *infliximab/ae [Adverse Drug Reaction]; *infliximab/dt [Drug Therapy].

Drug Trade Names and Manufacturers

remicade

Link to the Ovid Full Text or citation:

Click here for full text options

148.

Cutaneous tuberculosis in Tunisia.

Abdelmalek R., Mebazaa A., Berriche A., Kilani B., Ben Osman A., Mokni M., Tiouiri Benaissa H.

Medecine et Maladies Infectieuses. 43 (9) (pp 374-378), 2013. Date of Publication: 2013.

AN: 2013592488

Introduction: Tuberculosis is endemic in Tunisia. Pulmonary tuberculosis is the most common presentation in our country. Cutaneous presentations are rare (1-2% of cases). The diagnosis of cutaneous tuberculosis (CT) is difficult. Histological and clinical presentations are polymorphous, many differential diagnoses are available, and it is difficult to isolate Mycobacterium. Objective: We had for aim to study the epidemiological and clinical features of CT in Tunisia, and to compare presentations before and after 1990. Patients and methods: We conducted a retrospective study between January1991 and December2011, in which we included all cases of CT observed at the Infectious Diseases and Dermatology Units of the Tunis la Rabta Hospital. Results: Hundred and thirty-seven patients were included, with a mean age of 43.8. years; 72.3% were female patients. Hundred and fifty locations were observed, most of which on the head and neck. Scrofuloderma was the most frequent presentation, observed in 65% of cases. The diagnosis was confirmed by histology and/or microbiology in 75.8% of cases. The treatment was prescribed for a mean 11.3. months, leading to full recovery in most cases. Conclusion: CT is still reported in Tunisia. The diagnosis relies mainly on histology. Controlling this mutilating tuberculosis requires a global control of this disease, and especially lymph node location, given the high rate of scrofuloderma. © 2013 Elsevier Masson SAS.

Institution

(Abdelmalek, Berriche, Kilani, Tiouiri Benaissa) Service des maladies infectieuses, Faculte de medecine de Tunis, Universite Tunis EL Manar, hOpital la Rabta, Tunis, Tunisia (Mebazaa, Ben Osman, Mokni) Service de dermatologie, Faculte de medecine de Tunis, Universite Tunis EL Manar, hOpital la Rabta, Tunis, Tunisia

Publisher

Elsevier Masson SAS (62 rue Camille Desmoulins, Issy les Moulineaux Cedex 92442, France)

Emtree Heading

adolescent; adult; aged; antibiotic resistance; article; bacterial skin disease; bacterium identification; child; clinical feature; comparative study; controlled study; delayed diagnosis; diabetes mellitus; endemic disease/di [Diagnosis]; endemic disease/dr [Drug Resistance]; endemic disease/ep [Epidemiology]; female; histopathology; human; Human immunodeficiency virus infection; lupus vulgaris; major clinical study; male; Mycobacterium tuberculosis; nonhuman; outcome assessment; preschool child; retrospective study; risk factor; scrofuloderma; skin biopsy; skin toxicity/si [Side Effect]; *skin tuberculosis/di [Diagnosis]; *skin tuberculosis/dr [Drug Resistance]; *skin tuberculosis/dt [Drug Therapy]; *skin tuberculosis/ep [Epidemiology]; toxicoderma/si [Side Effect]; treatment duration; tuberculin test; tuberculous gumma; Tunisia; ethambutol/ae [Adverse Drug Reaction]; ethambutol/cb [Drug Combination]; ethambutol/dt [Drug Therapy]; isoniazid/ae [Adverse Drug Reaction]; isoniazid/cb [Drug Combination]; isoniazid/dt [Drug Therapy]; pyrazinamide/ae [Adverse Drug Reaction]; pyrazinamide/cb [Drug Combination]; pyrazinamide/dt [Drug Therapy]; quinoline derived antiinfective agent/dt [Drug Therapy]; rifampicin/ae [Adverse Drug Reaction]; rifampicin/cb [Drug Combination]; rifampicin/dt [Drug Therapy]; streptomycin/ae [Adverse Drug Reaction]; streptomycin/cb [Drug Combination]; streptomycin/dt [Drug Therapy].

Link to the Ovid Full Text or citation:

Click here for full text options

149.

Filamentous basidiomycetes in the clinical laboratory.

Brandt M.E.

Current Fungal Infection Reports. 7 (3) (pp 219-223), 2013. Date of Publication: September 2013.

AN: 2013550598

Filamentous basidiomycetes are difficult to identify in the clinical laboratory, mostly due to lack of sporulation, and their role as agents of fungal infection can be difficult to assess. More cases of infection with these agents are being reported as more laboratories gain proficiency with the recognition of their subtle morphologic features and the use of DNA-based methods for identification. Most infections occur in the respiratory tract and sinuses, although brain infection has been reported. Susceptibility testing suggests that these agents will respond well to azole drugs other than fluconazole. © 2013 Springer Science+Business Media New York (outside the USA).

Institution

(Brandt) Mycotic Diseases Branch, Centers for Disease Control and Prevention, Mailstop G-11, 1600 Clifton Road, Atlanta GA 30333, United States

Publisher

Current Medicine Group LLC (400 Market St, Ste 700 Philadelphia PA 19106, United States)

Emtree Heading

antifungal susceptibility; article; asthma; autopsy; bacterial pneumonia; *Basidiomycetes; basidiospore; blood transfusion; bone marrow biopsy; bone marrow transplantation; brain abscess; brain biopsy; brain damage; brain infection; brain tissue; bronchopneumonia; chronic cough; chronic granulomatous disease; chronic obstructive lung disease/et [Etiology]; *clinical laboratory; Coccidioides; diabetes mellitus; diabetic patient; DNA sequence; DRESS syndrome; dyspnea; endoscopic surgery; follow up; frontal cortex; fungal sinusitis; fungus ball; headache; high risk patient; hospital discharge; human; immunocompromised patient; immunosuppressive treatment; leukemia/th [Therapy]; life cycle; lung infection; lung infiltrate; lung injury; lung mycosis; maxilla sinusitis; meningitis; minimum inhibitory concentration; mycosis; nail infection; nonhuman; nose obstruction; nuclear magnetic resonance imaging; onychomycosis; orbit tumor; respiratory failure; Schizophyllum commune; septic shock; species identification; Sporothrix; steroid therapy; stomach carcinoma; treatment duration; treatment failure; tuberculosis/et [Etiology]; ulcer; umbilical cord blood; university hospital; Volvariella; amphotericin; amphotericin B; amphotericin B lipid complex; caspofungin; cycloheximide; echinocandin; fluconazole/iv [Intravenous Drug Administration]; itraconazole; posaconazole/po [Oral Drug Administration]; pyrrole; voriconazole.

Link to the Ovid Full Text or citation:

Click here for full text options

150.

Management of human immunodeficiency virus in primary care.

Sell J.K.

Primary Care - Clinics in Office Practice. 40 (3) (pp 589-617), 2013. Date of Publication: September 2013.

AN: 2013520318

This article presents an overview of current human immunodeficiency (HIV) management for primary care practitioners. Discussion is focused on appropriate screening, antiretroviral treatment, opportunistic infection prophylaxis, laboratory testing and prevention. Improved screening can identify the 20-25% of persons living with HIV in the United States who remain undiagnosed. Expansion of treatment recommendations to include all HIV-infected persons and expanded opportunities for prophylaxis will likely significantly increase the number of persons who receive antiretroviral treatment. Understanding of opportunistic infection prophylaxis, proper vaccination, and comorbid risk factor modification can improve life expectancy for many patients living with chronic stable HIV infection. © 2013 Elsevier Inc.

Institution

(Sell) Penn State Hershey Department of Family and Community Medicine, Penn State Milton S. Hershey Medical Center, 500 University Drive, Hershey, PA 17033, United States

Publisher

W.B. Saunders (Independence Square West, Philadelphia PA 19106-3399, United States)

Emtree Heading

atypical mycobacteriosis/dt [Drug Therapy]; atypical mycobacteriosis/pc [Prevention]; CD4 lymphocyte count; chickenpox/dt [Drug Therapy]; chickenpox/pc [Prevention]; comorbidity; coronary artery disease/si [Side Effect]; cryptococcal meningitis; diabetes mellitus/si [Side Effect]; diagnostic procedure; drug eruption/si [Side Effect]; drug treatment failure; dyslipidemia/si [Side Effect]; fatty liver/si [Side Effect]; gastrointestinal intolerance/si [Side Effect]; gastrointestinal symptom/si [Side Effect]; glucose intolerance/si [Side Effect]; health care cost; hepatitis A/dt [Drug Therapy]; hepatitis A/pc [Prevention]; hepatitis B/dt [Drug Therapy]; hepatitis B/pc [Prevention]; human; *Human immunodeficiency virus infection/di [Diagnosis]; *Human immunodeficiency virus infection/dm [Disease Management]; *Human immunodeficiency virus infection/dr [Drug Resistance]; *Human immunodeficiency virus infection/dt [Drug Therapy]; *Human immunodeficiency virus infection/pc [Prevention]; hyperlipidemia/si [Side Effect]; immunization; infection prevention; influenza/dt [Drug Therapy]; influenza/pc [Prevention]; laboratory test; lactic acidosis/si [Side Effect]; life expectancy; lipodystrophy/si [Side Effect]; liver toxicity/si [Side Effect]; occupational exposure; opportunistic infection/pc [Prevention]; peripheral neuropathy/si [Side Effect]; pneumococcal infection/dt [Drug Therapy]; pneumococcal infection/pc [Prevention]; Pneumocystis pneumonia/dt [Drug Therapy]; Pneumocystis pneumonia/pc [Prevention]; pregnancy; *primary medical care; priority journal; review; risk factor; screening; Stevens Johnson syndrome/si [Side Effect]; toxoplasmosis/dt [Drug Therapy]; toxoplasmosis/pc [Prevention]; tuberculosis/dt [Drug Therapy]; tuberculosis/pc [Prevention]; United States; abacavir plus lamivudine/dt [Drug Therapy]; abacavir plus lamivudine plus zidovudine/dt [Drug Therapy]; antiretrovirus agent/dt [Drug Therapy]; atovaquone/cb [Drug Combination]; atovaquone/dt [Drug Therapy]; azithromycin/dt [Drug Therapy]; chickenpox vaccine/dt [Drug Therapy]; clarithromycin/dt [Drug Therapy]; cotrimoxazole/dt [Drug Therapy]; dapsone/cb [Drug Combination]; dapsone/dt [Drug Therapy]; didanosine/ae [Adverse Drug Reaction]; didanosine/dt [Drug Therapy]; efavirenz plus emtricitabine plus tenofovir disoproxil/dt [Drug Therapy]; emtricitabine plus tenofovir disoproxil/dt [Drug Therapy]; folinic acid/cb [Drug Combination]; folinic acid/dt [Drug Therapy]; hepatitis A vaccine/dt [Drug Therapy]; hepatitis B vaccine/dt [Drug Therapy]; Human immunodeficiency virus vaccine/dt [Drug Therapy]; influenza vaccine/dt [Drug Therapy]; isoniazid/dt [Drug Therapy]; lamivudine plus zidovudine/dt [Drug Therapy]; nonnucleoside reverse transcriptase inhibitor/ae [Adverse Drug Reaction]; nonnucleoside reverse transcriptase inhibitor/dt [Drug Therapy]; pentamidine/dt [Drug Therapy]; Pneumococcus vaccine/dt [Drug Therapy]; proteinase inhibitor/ae [Adverse Drug Reaction]; proteinase inhibitor/dt [Drug Therapy]; pyridoxine/dt [Drug Therapy]; pyrimethamine/cb [Drug Combination]; pyrimethamine/dt [Drug Therapy]; rifabutin/dt [Drug Therapy]; rifampicin/dt [Drug Therapy]; RNA directed DNA polymerase inhibitor/ae [Adverse Drug Reaction]; RNA directed DNA polymerase inhibitor/dt [Drug Therapy]; stavudine/ae [Adverse Drug Reaction]; stavudine/dt [Drug Therapy]; unindexed drug.

Drug Trade Names and Manufacturers

Abbott [United States], Bristol Myers Squibb [United States], Gilead [United States], Glaxo SmithKline [United Kingdom], Pfizer [United States], atripla: Bristol Myers Squibb [United States], atripla: Gilead [United States], combivir: Glaxo SmithKline [United Kingdom], epzicom: Glaxo SmithKline [United Kingdom], trizivir: Glaxo SmithKline [United Kingdom], truvada: Gilead [United States]

Link to the Ovid Full Text or citation:

Click here for full text options

151.

The impact of diabetes mellitus and its control on the development of tuberculosis: A nationwide longitudinal study in Taiwan.

Lee M.-C., Lee C.-H., Shu C.-C., Pong W.-B., Lan C.-C., Wang J.-Y., Lee L.-N., Chao K.-M.

Pharmacoepidemiology and Drug Safety. 22 (9) (pp 995-1003), 2013. Date of Publication: September 2013.

AN: 2013527414

Purpose: Diabetic mellitus (DM) is a well-known risk factor of tuberculosis (TB). However, there is paucity of reports on the impact of diabetic control and adherence to anti-diabetic treatment on the risk of TB. This nationwide cohort study aimed to address these issues. Methods: In the Longitudinal Health Insurance Database 2005 of Taiwan, DM cases and matched control subjects were selected. Potential risk factors for developing TB were investigated using the Cox proportional hazards regression analysis. Time-dependent variables were used to measure drug prescription, adherence, and number of DM-associated admissions from 360 to 90days prior to each outcome event. Results: The 49903 DM patients identified (52.5% males) had a mean age of 61.9+/-14.2years. Among DM patients and control subjects, the independent risk factors of TB were age, male sex, chronic obstructive pulmonary disease (COPD), autoimmune disease, and DM (hazard ratio [HR] 1.293 [1.154-1.449]). Among DM patients, time-dependent Cox analysis revealed that age, male sex, COPD, end-stage renal disease, maximum average daily dose of oral hypoglycemic agent (HR 1.13 [1.071-1.193]), insulin use during admission (HR 1.462 [1.087-1.966]), adherence to anti-diabetic medication (0.577 [0.429-0.776]), and number of DM-associated admissions (1.789 [1.231-2.601]) were independent factors associated with the development of TB. Conclusions: The risk of TB parallels DM severity. Some cases of TB can be prevented by fostering adherence to anti-diabetic medication. © 2013 John Wiley & Sons, Ltd.

Institution

(Lee) Department of Pharmacy, Buddhist Tzu Chi General Hospital, Taipei Branch, Taiwan (Republic of China) (Lee, Chao) Graduate Institute of Biomedical Electronics and Bioinformatics, National Taiwan University, Taipei, Taiwan (Republic of China)

(Lee, Pong, Lan) Department of Internal Medicine, Buddhist Tzu Chi General Hospital, Taipei Branch, Taiwan (Republic of China)

(Lee, Lan) School of Medicine, Tzu Chi University, Hualien, Taiwan (Republic of China)

(Shu) Department of Traumatology, National Taiwan University Hospital, Taipei, Taiwan (Republic of China)

(Wang) Department of Internal Medicine, National Taiwan University Hospital, Taipei, Taiwan (Republic of China)

(Lee) Department of Laboratory Medicine, National Taiwan University Hospital, Taipei, Taiwan (Republic of China)

Publisher

John Wiley and Sons Ltd (Southern Gate, Chichester, West Sussex PO19 8SQ, United Kingdom)

Emtree Heading

adult; age; aged; article; autoimmune disease; chronic obstructive lung disease; cohort analysis; comorbidity; controlled study; diabetes control; *diabetes mellitus/dt [Drug Therapy]; diabetic patient; drug megadose; female; follow up; gender; hospital admission; human; insulin dependent diabetes mellitus; kidney failure; liver cirrhosis; longitudinal study; lowest income group; major clinical study; male; medication compliance; organ transplantation; outcome assessment; prescription; priority journal; risk factor; Taiwan; *tuberculosis/dt [Drug Therapy]; *tuberculosis control; corticosteroid; insulin/dt [Drug Therapy]; oral antidiabetic agent/dt [Drug Therapy]; prescription drug; tuberculostatic agent/dt [Drug Therapy].

Link to the Ovid Full Text or citation:

Click here for full text options

152.

Incidence and outcome of newly-diagnosed tuberculosis in schizophrenics: A 12-year, nationwide, retrospective longitudinal study.

Kuo S.-C., Chen Y.-T., Li S.-Y., Lee Y.-T., Yang A.C., Chen T.-L., Liu C.-J., Chen T.-J., Su I.-J., Fung C.-P.

BMC Infectious Diseases. 13 (1) (no pagination), 2013. Article Number: 351. Date of Publication: 29 Jul 2013.

AN: 2013486755

Background: To control tuberculosis (TB), it is critical to identify at risk populations. Schizophrenia is recognized as an important risk factor for TB. However, previous studies have been confounded by comorbidities, and reports of TB infection outcomes are rare. Therefore, the current nation-wide study aimed to compare the adjusted incidence and outcome of TB diseases in schizophrenics and the general population.Method: Using the National Health Insurance Research Database from 1998 to 2009, this retrospective longitudinal study included 60,409 schizophrenics and general population matched for age, Charlson's score, and comorbidities. Diagnosis of TB was based on the international classification of disease, ninth revision and use of anti-TB drugs. Unfavorable outcome for TB was defined as death, loss to follow-up, or use of anti-TB treatment for more than 9 months.Results: The adjusted incidence of TB in schizophrenics was significantly higher than in the general population [hazard ratio, 1.52; 95% confidence interval (CI), 1.29-1.79; p < 0.001; Kaplan-Meier log-rank test, p < 0.001]. Cox regression revealed age and male gender as risk factors for newly-diagnosed TB. The outcome of TB was comparable in schizophrenics and the general population [odds ratio (OR), 0.78; 95% CI, 0.55-1.09; p =0.144]. Logistic regression revealed a statistical trend for diabetes mellitus to predict poor outcome in schizophrenics with TB (OR, 2.30; 95% CI, 0.96-5.74; p = 0.062).Conclusions: Schizophrenics are at increased risk for TB, and screening may be warranted for those living in areas with high prevalence of TB. © 2013 Kuo et al.; licensee BioMed Central Ltd.

Institution

(Kuo, Li, Lee, Chen, Fung) Institute of Clinical Medicine, National Yang-Ming University, School of Medicine, Taipei, Taiwan (Republic of China) (Kuo, Lee, Chen, Fung) Division of Infectious Diseases, Taipei Veterans General Hospital, No. 201, Sec. 2, Shih-Pai Road, Taipei 112, Taiwan (Republic of China)

(Kuo, Su) National Institute of Infectious Diseases and Vaccinology, National Health Research Institutes, Miaoli County, Taiwan (Republic of China)

(Chen, Li) Division of Nephrology, Taipei Veterans General Hospital, Taipei, Taiwan (Republic of China)

(Chen) Department of Medicine, Taipei City Hospital Heping Fuyou Branch, Taipei, Taiwan (Republic of China)

(Lee) Department of Medicine, Chutung Veterans Hospital, Chutung, Taiwan (Republic of China)

(Yang) Department of Psychiatry, Taipei Veterans General Hospital, Taipei, Taiwan (Republic of China)

(Yang) Division of Psychiatry, School of Medicine, National Yang-Ming University, Taipei, Taiwan (Republic of China)

(Liu) Institute of Public Health, School of Medicine, National Yang-Ming University, Taipei, Taiwan (Republic of China)

(Liu) Division of Hematology and Oncology, Department of Medicine, Taipei Veterans General Hospital, Taipei, Taiwan (Republic of China)

(Liu) Department of Internal Medicine, National Yang-Ming University Hospital, Yilan, Taiwan (Republic of China)

(Chen) Department of Family Medicine, Taipei Veterans General Hospital, Taipei, Taiwan (Republic of China)

Publisher

BioMed Central Ltd. (Floor 6, 236 Gray's Inn Road, London WC1X 8HB, United Kingdom)

Emtree Heading

adult; age; article; Charlson Comorbidity Index; controlled study; drug use; female; human; ICD-9; incidence; infection risk; longitudinal study; major clinical study; male; mortality; outcome assessment; retrospective study; risk assessment; *schizophrenia; sex difference; treatment duration; *tuberculosis/dt [Drug Therapy]; *tuberculosis/ep [Epidemiology]; tuberculostatic agent/dt [Drug Therapy].

Link to the Ovid Full Text or citation:

Click here for full text options

153.

Selective uptake of influenza vaccine and pregnancy outcomes.

Cantu J., Biggio J., Jauk V., Wetta L., Andrews W., Tita A.

Journal of Maternal-Fetal and Neonatal Medicine. 26 (12) (pp 1207-1211), 2013. Date of Publication: August 2013.

AN: 2013447781

Objective: To describe the characteristics of pregnant women who accept the influenza vaccine and evaluate the relationship between vaccination and adverse pregnancy outcomes. Methods: Retrospective cohort study of women receiving prenatal care during the 2009-2011 influenza seasons. Vaccination status was ascertained through our perinatal record system and clinic vaccination logs. Pregnancy outcomes included a primary composite of miscarriage, fetal demise, preterm birth (PTB) <37 weeks and neonatal demise. Stratification and logistic regression were used to adjust for potential confounders. Results: Of 3104 eligible pregnant women, 1094 (35%) received the influenza vaccine. Women vaccinated were more likely to be older, obese, primiparae, and have medical complications or a prior PTB. In univariable analyses, flu vaccination was associated with increased adverse composite outcome and PTB. After multivariable adjustments, vaccination was no longer associated with adverse outcomes in women with medical complications but remained associated with adverse outcomes among those without known co-morbidity. Conclusions: Vaccination was associated with an increased adverse composite outcome in pregnant women without identified co-morbidity but not those with co-morbidities. This association is likely due to selection bias, which should be considered in planning of observational studies of the impact of vaccination on pregnancy outcomes. © 2013 Informa UK Ltd. All rights reserved: reproduction in whole or part not permitted.

Institution

(Cantu, Biggio, Jauk, Wetta, Andrews, Tita) Center for Women's Reproductive Health, University of Alabama at Birmingham, Birmingham, AL, United States

Publisher

Informa Healthcare (69-77 Paul Street, London EC2A 4LQ, United Kingdom)

Emtree Heading

adult; age distribution; antiviral therapy; article; clinical feature; cohort analysis; *drug uptake; female; fetus death; gestational age; hepatitis C/co [Complication]; human; Human immunodeficiency virus infection; influenza/dt [Drug Therapy]; influenza/pc [Prevention]; influenza vaccination; low birth weight; lung tuberculosis; lupus vulgaris/co [Complication]; maternal hypertension/co [Complication]; medical record; newborn death; obesity; outcome assessment; preeclampsia; pregnancy complication; pregnancy diabetes mellitus/co [Complication]; *pregnancy outcome; pregnant woman; premature labor; prenatal care; primipara; priority journal; retrospective study; sickle cell anemia/co [Complication]; small for date infant; spontaneous abortion; stillbirth; *influenza vaccine/dt [Drug Therapy]; oseltamivir/dt [Drug Therapy].

Link to the Ovid Full Text or citation:

Click here for full text options

154.

Antibiotic resistance rates in causative agents of infections in diabetic patients: Rising concerns.

Boyanova L., Mitov I.

Expert Review of Anti-Infective Therapy. 11 (4) (pp 411-420), 2013. Date of Publication: April 2013.

AN: 2013428188

The vicious cycle is that hyperglycemia (>11.1 mmol/l) or other diabetes-associated factors facilitate or worsen the development of infections and vice versa, the infections deteriorate the glycemic control of the patients. Diabetic patients are prone to some infections, infection recurrences and poor outcomes. Immunocompromised state and frequent antibiotic use are associated with antibiotic resistance of the bacterial pathogens, such as Mycobacterium tuberculosis (in some studies), methicillin-resistant Staphylococcus aureus, Streptococcus pneumoniae, Gram-negative bacteria such as Pseudomonas aeruginosa and Acinetobacter baumannii, bacteria in diabetic foot infections and different opportunistic and rare pathogens or multidrug-resistant strains. Prompt diagnostics and susceptibility testing, early and aggressive surgical and/or antibiotic therapy, and, importantly, good glycemic control are of utmost importance for treatment of antibiotic-resistant infections in diabetic patients. © 2013 2013 Expert Reviews Ltd.

Institution

(Boyanova, Mitov) Department of Medical Microbiology, Medical University of Sofia, Zdrave Street 2, 1431 Sofia, Bulgaria

Publisher

Expert Reviews Ltd. (2 Albert Place, London N3 1QB, United Kingdom)

Emtree Heading

Acinetobacter baumannii; *antibiotic resistance; antibiotic sensitivity; bacterial strain; blood glucose monitoring; cellulitis; *diabetes mellitus/dt [Drug Therapy]; diabetic foot/dt [Drug Therapy]; drug penetration; drug use; early diagnosis; emphysematous cholecystitis; emphysematous pyelonephritis; external otitis; glycemic control; Gram negative bacterium; Gram positive bacterium; Helicobacter pylori; human; hyperglycemia; immunocompromised patient; *infection; infection risk; lower respiratory tract infection/dt [Drug Therapy]; methicillin resistant Staphylococcus aureus; mixed infection; Mycobacterium tuberculosis; nonhuman; osteomyelitis; outcome assessment; peritonitis; Pseudomonas aeruginosa; recurrent infection; review; risk reduction; sepsis; skin infection; Streptococcus pneumoniae; tuberculosis/dt [Drug Therapy]; unspecified side effect/si [Side Effect]; urinary tract infection/pc [Prevention]; amoxicillin; *antibiotic agent/dt [Drug Therapy]; *antibiotic agent/po [Oral Drug Administration]; *antibiotic agent/pa [Parenteral Drug Administration]; *antibiotic agent/pk [Pharmacokinetics]; aztreonam; beta lactamase; carbapenem; cefixime; cefotaxime; ceftazidime; ceftriaxone; cefuroxime; cephalosporin derivative; ciprofloxacin; clindamycin/dt [Drug Therapy]; clindamycin/po [Oral Drug Administration]; gatifloxacin; gentamicin; glitazone derivative/dt [Drug Therapy]; glucose/ec [Endogenous Compound]; imipenem; insulin/dt [Drug Therapy]; lincosamide; macrolide; metallo beta lactamase/ec [Endogenous Compound]; metformin/ae [Adverse Drug Reaction]; metformin/dt [Drug Therapy]; nitrofurantoin; quinoline derived antiinfective agent/dt [Drug Therapy]; quinoline derived antiinfective agent/po [Oral Drug Administration]; rifampicin/dt [Drug Therapy]; sparfloxacin; streptogramin derivative; sulfonylurea/dt [Drug Therapy]; unindexed drug.

Link to the Ovid Full Text or citation:

Click here for full text options

155.

Beyond pregnancy - The neglected burden of mortality in young women of reproductive age in Bangladesh: A prospective cohort study.

Labrique A.B., Sikder S.S., Wu L., Rashid M., Ali H., Ullah B., Shamim A.A., Mehra S., Klemm R., Banu H., West Jr. K.P., Christian P.

BJOG: An International Journal of Obstetrics and Gynaecology. 120 (9) (pp 1085-1089), 2013. Date of Publication: August 2013.

AN: 2013441665

Objective To describe proportionate mortality and causes of death unrelated to pregnancy. Design Prospective cohort study. Setting Rural northwest Bangladesh. Population A cohort of 133 617 married women of reproductive age. Methods Verbal autopsies were conducted for women who died whilst under surveillance in the cohort trial. Physician-assigned causes of death based on verbal autopsies were used to categorise deaths. Main outcome measures The proportion of deaths due to non-communicable diseases, infectious diseases, injury or pregnancy. Results Of the 1107 deaths occurring among women between 2001 and 2007, 48% were attributed to non-communicable diseases, 22% to pregnancy, 17% to infections, 9% to injury and 4% to other causes. Conclusions Although focus on pregnancy-related mortality remains important, more attention is warranted on non-communicable diseases among women of reproductive age. © 2013 The Authors BJOG An International Journal of Obstetrics and Gynaecology © 2013 RCOG.

Institution

(Labrique, Sikder, Wu, Mehra, Klemm, West Jr., Christian) Department of International Health, Johns Hopkins Bloomberg School of Public Health, 615 N. Wolfe St., Baltimore, MD 21205, United States (Rashid, Ali, Shamim) JiVitA Maternal and Child Health Research Project Gaibandha, Poschim Para, Bangladesh

(Ullah) Maternal and Neonatal Health Initiative, United Nations Population Fund Narail, Aladatpur, Bangladesh

(Banu) Department of Gynecology, Institute of Child and Mother Health Matuail, Dhaka, Bangladesh

Publisher

Blackwell Publishing Ltd (9600 Garsington Road, Oxford OX4 2XG, United Kingdom)

Emtree Heading

adult; anaphylactic shock; anemia; anorexia nervosa; asthma; autopsy; Bangladesh; bleeding; cause of death; cerebrovascular accident; cohort analysis; dehydration; diabetes mellitus; diarrhea; diphtheria; epilepsy; female; gastrointestinal disease; heart failure; heart infarction; homicide; human; infection; injury; ischemic heart disease; kidney disease; lung embolism; major clinical study; malaria; malnutrition; married woman; meningitis; *mortality; neoplasm; non communicable disease; outcome assessment; pericardial effusion; *pregnancy; priority journal; prospective study; rabies; respiratory tract infection; review; rheumatic disease; sepsis; suicide; tetanus; tonsillitis; tuberculosis; typhoid fever.

Link to the Ovid Full Text or citation:

Click here for full text options

156.

The double burden of diabetes and tuberculosis - Public health implications.

Kapur A., Harries A.D.

Diabetes Research and Clinical Practice. 101 (1) (pp 10-19), 2013. Date of Publication: July 2013.

AN: 2013412760

Diabetes mellitus (DM) and tuberculosis (TB) have existed for thousands of years and even now the global disease burden from DM and TB is huge. The incidence of TB is declining slowly but it still remains a big problem in many populous large low and middle income countries. On the other, hand the burden of diabetes is increasing very rapidly, particularly in the very same countries where TB is endemic. The intersecting double burden is therefore ominous particularly as several studies and systematic reviews have indicated that DM increases the risk of TB disease and results in poor treatment outcomes. To address the double burden, WHO and the International Union Against Tuberculosis and Lung Disease (The Union) in 2011 launched a collaborative framework for the care and control of diabetes and tuberculosis, to encourage collaborative research and implement bidirectional screening of the two diseases in routine settings. This review article (i) explores some of the new evidence for the association between TB and DM, (ii) discusses issues with regard to clinical presentation and outcomes, (iii) presents the evidence, challenges and strategies for bidirectional screening based on field studies to implement the framework and (iv) finally presents suggestions on how diabetes care delivery may benefit from the lessons of the TB DOTS approach and public health principles for structured care delivery. © 2012 Elsevier Ireland Ltd.

Institution

(Kapur) World Diabetes Foundation, Gentofte, Denmark (Harries) International Union Against Tuberculosis and Lung Diseases, Paris, France

(Harries) London School of Hygiene and Tropical Medicine, London, United Kingdom

Publisher

Elsevier Ireland Ltd (P.O. Box 85, Limerick, Ireland)

Emtree Heading

body mass; clinical feature; comorbidity; diabetes control; *diabetes mellitus/di [Diagnosis]; *diabetes mellitus/dt [Drug Therapy]; *diabetes mellitus/ep [Epidemiology]; *diabetes mellitus/et [Etiology]; disease association; disease predisposition; drug treatment failure; financial management; glycemic control; health care cost; health care delivery; health care planning; health program; hemoglobin blood level; high risk patient; human; impaired glucose tolerance; incidence; infection risk; insulin resistance; insurance; nonhuman; outcome assessment; pathogenesis; prevalence; *public health problem; recurrent disease; review; screening test; sputum smear; *tuberculosis/di [Diagnosis]; *tuberculosis/dt [Drug Therapy]; *tuberculosis/ep [Epidemiology]; *tuberculosis/et [Etiology]; tuberculosis control; glibenclamide/dt [Drug Therapy]; glibenclamide/po [Oral Drug Administration]; gliclazide/dt [Drug Therapy]; gliclazide/po [Oral Drug Administration]; glimepiride/dt [Drug Therapy]; glimepiride/po [Oral Drug Administration]; glipizide/dt [Drug Therapy]; glipizide/po [Oral Drug Administration]; glycosylated hemoglobin/ec [Endogenous Compound]; isoniazid/dt [Drug Therapy]; rifampicin/dt [Drug Therapy]; tuberculostatic agent/dt [Drug Therapy].

Link to the Ovid Full Text or citation:

Click here for full text options

157.

The double burden: The role of diabetes for tuberculosis risk, manifestations, treatment outcomes and survival.

Faurholt-Jepsen D.

Danish Medical Journal. 60 (7) (pp 18), 2013. Date of Publication: July 2013.

AN: 2013412092

One third of the world's population is latently infected with Mycobacterium tuberculosis, and with the lifestyle changes succeeding the on-going urbanization, populations already burdened by tuberculosis are experiencing a dramatic increase in chronic diseases, with diabetes being a serious challenge. Tuberculosis and diabetes are not only becoming co-existing diseases. In fact, the diseases interact, and there is evidence to suggest that especially diabetes disease increases the susceptibility for developing active tuberculosis disease. Furthermore, it is plausible that tuberculosis leads to, either transient or permanent, impairment of the glucose metabolism, which ultimately will turn into diabetes. A number of studies from the Americas, Europe, Asia, and, most lately, from sub-Saharan Africa have reported strong association between tuberculosis and diabetes; on average, the estimated risk of active tuberculosis is thrice as high among people with diabetes. The study from sub-Saharan Africa was conducted in Tanzania and is the basis of this thesis. Based on available evidence on the association between tuberculosis and diabetes, the primary aim of the study was to assess the role of diabetes for tuberculosis risk, manifestations, treatment outcomes and survival in a Tanzanian population of tuberculosis patients and non-tuberculosis neighbourhood controls. The study was conducted in Mwanza City in northern Tanzania, with a population exceeding half a million inhabitants, with tuberculosis and HIV being common infections in the region, but with little knowledge about the prevalence of diabetes. We recruited newly diagnosed pulmonary tuberculosis patients from spring 2006 and continuously till the fall 2009, with all participating in a nutritional intervention running in parallel with the medical tuberculosis treatment. All participants underwent diabetes and HIV testing as well as a series of measurements such as anthropometric, clinical and paraclinical parameters. The population was followed up during treatment (2 and 5 months) to assess treatment outcome as well as after one year to assess their survival status. Based on data from 1250 tuberculosis patients and 350 neighbourhood controls, we found that 38 and 21%, respectively, had impaired glycaemia, and that the prevalence of diabetes was 17 and 9% among tuberculosis patients and controls, respectively. This difference in prevalence between patients and controls was equivalent to an adjusted odds ratio of more than four, indicating a strong association between tuberculosis and diabetes. Furthermore, we found that diabetes was associated with tuberculosis among both participants with or without HIV co-infection. Despite the strong association, diabetes had only moderate clinical implications when the tuberculosis patients initiated the tuberculosis treatment; the patients with diabetes co-morbidity had a minor elevation in the immune response and more frequently reported to have fever. Furthermore, diabetes did not seem to delay time to sputum conversion during treatment. Nevertheless, diabetes co-morbidity led to impaired treatment outcome with slower recovery of weight and haemoglobin and a more than four times higher mortality rate within the initial phase of tuberculosis treatment. In conclusion, in the African region, the double burden of tuberculosis and diabetes is becoming a major health problem. Although the tuberculosis incidence has stabilized during the last decade, the increasing incidence of diabetes will possibly interfere with tuberculosis control and may, consequently, make the tuberculosis incidence increase again. Future research strategies should focus on enhanced diagnostic tools to identify tuberculosis patients with diabetes co-morbidity, and on the role of disease-disease, drug-disease and drug-drug interactions between tuberculosis and diabetes diseases and treatments.

Institution

(Faurholt-Jepsen) Department of Human Nutrition, University of Copenhagen, Rolighedsvej 30, 1958 Frederiksberg C, Denmark

Publisher

Danish Medical Association (Kristianiagade 14, Copenhagen DK-1263, Denmark)

Emtree Heading

anthropometry; article; death; *diabetes mellitus/di [Diagnosis]; disease association; disease control; follow up; human; Human immunodeficiency virus infection/di [Diagnosis]; medical record review; mortality; neutrophil count; prevalence; questionnaire; risk factor; sputum culture; staff training; survival; *treatment outcome; *tuberculosis/di [Diagnosis]; *tuberculosis/ep [Epidemiology]; weight gain; acute phase protein/ec [Endogenous Compound]; C reactive protein/ec [Endogenous Compound]; CD4 antigen/ec [Endogenous Compound]; ethambutol plus isoniazid; ethambutol plus isoniazid plus pyrazinamide plus rifampicin; glucose/ec [Endogenous Compound]; hemoglobin/ec [Endogenous Compound]; isoniazid plus rifampicin; orosomucoid/ec [Endogenous Compound].

Link to the Ovid Full Text or citation:

Click here for full text options

158.

Diabetes is a strong predictor of mortality during tuberculosis treatment: A prospective cohort study among tuberculosis patients from Mwanza, Tanzania.

Faurholt-Jepsen D., Range N., Praygod G., Jeremiah K., Faurholt-Jepsen M., Aabye M.G., Changalucha J., Christensen D.L., Grewal H.M.S., Martinussen T., Krarup H., Witte D.R., Andersen A.B., Friis H.

Tropical Medicine and International Health. 18 (7) (pp 822-829), 2013. Date of Publication: July 2013.

AN: 2013391100

Objective: Strong evidence suggests diabetes may be associated with tuberculosis (TB) and could influence TB treatment outcomes. We assessed the role of diabetes on sputum culture conversion and mortality among patients undergoing TB treatment. Methods: A total of 1250 Tanzanian TB patients were followed prospectively during TB treatment with sputum culture after 2 and 5 months. Survival status was assessed at least 1 year after initiation of treatment. At baseline, all participants underwent testing for diabetes and HIV, and the serum concentration of the acute phase reactant alpha-1 glycoprotein (AGP) was determined. Results: There were no differences between participants with and without diabetes regarding the proportion of positive cultures at 2 (3.8% vs. 5.8%) and 5 (1.3% vs. 0.9%) months (P > 0.46). However, among patients with a positive TB culture, relatively more patients with diabetes died before the 5-month follow-up. Within the initial 100 days of TB treatment, diabetes was associated with a fivefold increased risk of mortality (RR 5.09, 95% CI 2.36; 11.02, P < 0.001) among HIV uninfected, and a twofold increase among HIV co-infected patient (RR 2.33 95% CI 1.20; 4.53, P = 0.012), while diabetes was not associated with long-term mortality. Further adjustment with AGP did not change the estimates. Conclusion: Diabetes considerably increases risk of early mortality during TB treatment. The effect may not be explained by increased severity of TB, but could be due to impaired TB treatment response. Research is needed to clarify the mechanism and to assess whether glycaemic control improves survival. © 2013 Blackwell Publishing Ltd.

Institution

(Faurholt-Jepsen, Faurholt-Jepsen, Friis) Department of Nutrition, Exercise and Sports, University of Copenhagen, Frederiksberg, Denmark (Range) Muhimbili Research Centre, National Institute for Medical Research, Dar Es Salaam, Tanzania

(Praygod, Jeremiah, Changalucha) Mwanza Research Centre, National Institute for Medical Research, Mwanza, Tanzania

(Aabye) Clinical Research Centre, University of Copenhagen, Hvidovre Hospital, Hvidovre, Denmark

(Christensen) Department of International Health, University of Copenhagen, Copenhagen, Denmark

(Grewal) The Gade Institute, University of Bergen, Bergen, Norway

(Martinussen) Biostatistics and Mathematics, University of Copenhagen, Copenhagen, Denmark

(Krarup) Department of Clinical Biochemistry, Aalborg University Hospital, Aalborg, Denmark

(Witte) Steno Diabetes Center, Gentofte, Denmark

(Andersen) Department of Infectious Diseases, Odense University Hospital, Odense, Denmark

Publisher

Blackwell Publishing Ltd (9600 Garsington Road, Oxford OX4 2XG, United Kingdom)

Emtree Heading

adult; article; cohort analysis; comorbidity; *diabetes mellitus; female; human; Human immunodeficiency virus infection; lung tuberculosis/dt [Drug Therapy]; major clinical study; male; mixed infection; mortality; risk factor; sex difference; sputum culture; survival rate; Tanzania; *tuberculosis/dt [Drug Therapy]; alpha1 glycoprotein/ec [Endogenous Compound]; tuberculostatic agent/dt [Drug Therapy].

Link to the Ovid Full Text or citation:

Click here for full text options

159.

The biological and clinical activity of anti-malarial drugs in autoimmune disorders.

Taherian E., Rao A., Malemud C.J., Askari A.D.

Current Rheumatology Reviews. 9 (1) (pp 45-62), 2013. Date of Publication: 2013.

AN: 2013358663

Chloroquine and hydroxychloroquine are 4-aminoquinoline compounds commonly employed as anti-malarial drugs. Chloroquine and its synthetic analogue, hydroxychloroquine also belong to the disease-modifying anti-rheumatic drug class because these drugs are immunosuppressive. The immunosuppressive activity of chloroquine and hydroxychloroquine is likely to account for their capacity to reduce T-cell and B-cell hyperactivity as well as pro-inflammatory cytokine gene expression. This review evaluated experimental and clinical trials results as well as clinical response data relative to the use of chloroquine and/or hydroxychloroquine as first-line medical therapies in systemic lupus erythematosus, rheumatoid arthritis, primary Sjogren's syndrome, the anti-phospholipid syndrome and in the treatment of sarcoidosis. A primary outcomes measure in these clinical trials was the extent to which chloroquine and/or hydroxychloroquine reduced disease progression or exacerbations and/or the use and dosage of corticosteroids. The relative efficacy of chloroquine and hydroxychloroquine in modifying the clinical course of these autoimmune disorders is balanced against evidence that these drugs induce adverse effects which may reduce their use and effectiveness in the therapy of autoimmune disorders. © 2013 Bentham Science Publishers.

Institution

(Taherian, Rao, Malemud, Askari) Department of Medicine, Division of Rheumatic Diseases, Case Western Reserve University School of Medicine and University Hospitals Case Medical Center, Cleveland, OH 44106, United States (Malemud) Department of Anatomy, Case Western Reserve University School of Medicine, University Hospitals Case Medical Center, Cleveland, OH 44106, United States

Publisher

Bentham Science Publishers B.V. (P.O. Box 294, Bussum 1400 AG, Netherlands)

Emtree Heading

anticoagulation; antigen antibody complex; *antimalarial activity; *antiphospholipid syndrome; Apgar score; arterial stiffness; artery thrombosis/co [Complication]; artery thrombosis/pc [Prevention]; arthralgia; atherosclerosis; atomic force microscopy; autologous mixed lymphocyte reaction; birth weight; body mass; bone density; bone erosion; burning sensation; cardiomyopathy; cardiovascular risk; cell surface; cholesterol blood level; clinical practice; cohort analysis; coronary artery disease; cross-sectional study; deep vein thrombosis/pc [Prevention]; diabetes mellitus; disease activity; disease course; disease exacerbation; drug dose titration; drug efficacy; dyslipidemia; echography; ex vivo study; fat intake; follow up; glucose tolerance; heart left ventricle filling pressure; heart muscle biopsy; human; hypercalcemia; hypertension; in vitro study; in vivo study; insulin release; insulin sensitivity; joint function; kidney failure; kidney injury; lean body weight; lung embolism/pc [Prevention]; lung function; lung infiltrate; lung lesion; lupus erythematosus nephritis/dt [Drug Therapy]; lupus erythematosus nephritis/pc [Prevention]; lymphadenopathy; morning stiffness; mouth mucosa; myalgia; Mycobacterium tuberculosis; myopathy; North America; open study; outcome assessment; overall survival; phospholipid bilayer; pigment epithelium; pneumonia; postmenopause; postoperative period; pregnancy; pregnancy outcome; priority journal; propensity score; prospective study; pulse wave; randomized controlled trial (topic); remission; retinopathy/dt [Drug Therapy]; retrospective study; review; *rheumatoid arthritis/dt [Drug Therapy]; rheumatology; risk factor; sarcoidosis/dt [Drug Therapy]; sarcoidosis/et [Etiology]; *Sjoegren syndrome; skin defect; skin pigmentation; SLEDAI; *systemic lupus erythematosus/di [Diagnosis]; *systemic lupus erythematosus/dt [Drug Therapy]; *systemic lupus erythematosus/et [Etiology]; thorax radiography; thrombocyte aggregation; tissue injury; transthoracic echocardiography; world health organization; xerostomia; azathioprine; CD8 antigen/ec [Endogenous Compound]; chlorambucil; *chloroquine/dt [Drug Therapy]; cholesterol/ec [Endogenous Compound]; colchicine; cyclosporin; *hydroxychloroquine/cb [Drug Combination]; *hydroxychloroquine/dt [Drug Therapy]; *hydroxychloroquine/po [Oral Drug Administration]; interleukin 1/ec [Endogenous Compound]; interleukin 15/ec [Endogenous Compound]; interleukin 18/ec [Endogenous Compound]; interleukin 2/ec [Endogenous Compound]; interleukin 6/ec [Endogenous Compound]; interleukin 8/ec [Endogenous Compound]; leflunomide; lipocortin 5; low density lipoprotein/ec [Endogenous Compound]; methotrexate/cb [Drug Combination]; mycophenolic acid 2 morpholinoethyl ester/dt [Drug Therapy]; placebo; prednisone; quinoprox; salazosulfapyridine/cb [Drug Combination]; triacylglycerol/ec [Endogenous Compound]; tumor necrosis factor alpha/ec [Endogenous Compound]; unclassified drug.

Drug Trade Names and Manufacturers

plaquenil, quinoprox

Link to the Ovid Full Text or citation:

Click here for full text options

160.

Risk factors and characteristics of post-transplant tuberculosis in an endemic area.

Joo D.J., Seok Kim B., Jin Kim S., Ha Huh K., Soo Kim M., Ju M.K., Kim S.I.I., Seun Kim Y.

Annals of Transplantation. 18 (1) (pp 163-173), 2013. Date of Publication: 2013.

AN: 2013282096

Tuberculosis (TB) is a major post-transplant concern in endemic areas. This report summarizes the clinical characteristics, risk factors, and effects of post-transplant TB on graft and patient survival at a single center in Korea. We retrospectively analyzed data from 2799 kidney recipients at Yonsei University Health System between April 1979 and August 2008 to determine the incidence, outcome, and risk factors affecting the development of TB infections and the effect of TB on graft and patient survival rates. TB developed in 144 (109 males and 35 females; mean age, 37+/-12 years) out of 2799 (5.1%) recipients. Newly developed TB occurred in 116 recipients (81%) and recurrent TB occurred in 28 (19%) recipients with a pre-transplant history of a mycobacterial infection. The mean interval to TB diagnosis was 55.6+/-47.9 months after transplantation without a peak interval incidence for 8 years after transplantation. Based on Cox regression analysis, a history of TB was the strongest risk factor (hazard ratio [HR] PERSOONLY =11.618) affecting the development of TB. TB negatively affected graft and patient survival after kidney transplantation. Non-pleurisy extrapulmonary and miliary TB resulted in inferior treatment results and a poor prognosis in the early treatment period. A history of TB is the strongest predictor of post-transplant TB. Therefore, patients with a pre-transplant history of TB should be carefully monitored. © Ann Transplant.

Institution

(Joo, Ha Huh, Soo Kim, Ju, Kim, Seun Kim) Departments of Surgery Severance Hospital, Yonsei University Health System, Seoul, South Korea (Joo, Seok Kim, Ha Huh, Soo Kim, Ju, Kim, Seun Kim) Research Institute for Transplantation, Yonsei University College of Medicine, Seoul, South Korea

(Seok Kim) Internal Medicine, Severance Hospital, Yonsei University Health System, Seoul, South Korea

(Jin Kim) Department of Surgery, CHA Bundang Medical Center, CHA University, Seongnam, South Korea

Publisher

Medical Science International (ul. Ustrzycka 11, Warsaw 02-141, Poland)

Emtree Heading

acute graft rejection/dt [Drug Therapy]; adult; article; clinical feature; diabetes mellitus; extrapulmonary tuberculosis; female; follow up; gender; graft survival; Hepatitis B virus; human; major clinical study; male; medical history; miliary tuberculosis; morbidity; outcome assessment; *post transplant tuberculosis/di [Diagnosis]; *post transplant tuberculosis/dt [Drug Therapy]; prognosis; retrospective study; risk factor; survival; survival rate; *tuberculosis/di [Diagnosis]; *tuberculosis/dt [Drug Therapy]; tuberculous pleurisy; adenosine deaminase/ec [Endogenous Compound]; azathioprine/cb [Drug Combination]; azathioprine/dt [Drug Therapy]; basiliximab/dt [Drug Therapy]; calcineurin inhibitor/cb [Drug Combination]; calcineurin inhibitor/dt [Drug Therapy]; cyclosporin A/cb [Drug Combination]; cyclosporin A/dt [Drug Therapy]; ethambutol/cb [Drug Combination]; ethambutol/dt [Drug Therapy]; isoniazid/cb [Drug Combination]; isoniazid/dt [Drug Therapy]; methylprednisolone/dt [Drug Therapy]; mycophenolic acid 2 morpholinoethyl ester/cb [Drug Combination]; mycophenolic acid 2 morpholinoethyl ester/dt [Drug Therapy]; OKT 3; prednisolone/cb [Drug Combination]; prednisolone/dt [Drug Therapy]; pyrazinamide/cb [Drug Combination]; pyrazinamide/dt [Drug Therapy]; rifampicin/cb [Drug Combination]; rifampicin/dt [Drug Therapy]; steroid/cb [Drug Combination]; steroid/dt [Drug Therapy]; streptomycin/cb [Drug Combination]; streptomycin/dt [Drug Therapy]; tacrolimus/dt [Drug Therapy]; thymocyte antibody.

Link to the Ovid Full Text or citation:

Click here for full text options

161.

Clinical characteristics, drug resistance, and treatment outcomes among tuberculosis patients with diabetes in Peru.

Magee M.J., Bloss E., Shin S.S., Contreras C., Huaman H.A., Ticona J.C., Bayona J., Bonilla C., Yagui M., Jave O., Cegielski J.P.

International Journal of Infectious Diseases. 17 (6) (pp e404-e412), 2013. Date of Publication: June 2013.

AN: 2013289598

Objectives: Diabetes is a risk factor for active tuberculosis (TB). Data are limited regarding the association between diabetes and TB drug resistance and treatment outcomes. We examined characteristics of TB patients with and without diabetes in a Peruvian cohort at high risk for drug-resistant TB. Among TB patients with diabetes (TB-DM), we studied the association between diabetes clinical/management characteristics and TB drug resistance and treatment outcomes. Methods: During 2005-2008, adults with suspected TB with respiratory symptoms in Lima, Peru, who received rapid drug susceptibility testing (DST), were prospectively enrolled and followed during treatment. Bivariate and Kaplan-Meier analyses were used to examine the relationships of diabetes characteristics with drug-resistant TB and TB outcomes. Results: Of 1671 adult TB patients enrolled, 186 (11.1%) had diabetes. TB-DM patients were significantly more likely than TB patients without diabetes to be older, have had no previous TB treatment, and to have a body mass index (BMI) >18.5kg/m2 (p<0.05). In patients without and with previous TB treatment, the prevalence of multidrug-resistant TB was 23% and 26%, respectively, among patients without diabetes, and 12% and 28%, respectively, among TB-DM patients. Among 149 TB-DM patients with DST results, 104 (69.8%) had drug-susceptible TB and 45 (30.2%) had drug-resistant TB, of whom 29 had multidrug-resistant TB. There was no association between diabetes characteristics and drug-resistant TB. Of 136 TB-DM patients with outcome information, 107 (78.7%) had a favorable TB outcome; active diabetes management was associated with a favorable outcome. Conclusions: Diabetes was common in a cohort of TB patients at high risk for drug-resistant TB. Despite prevalent multidrug-resistant TB among TB-DM patients, the majority had a favorable TB treatment outcome. © 2013 International Society for Infectious Diseases.

Institution

(Magee) Rollins School of Public Health, Emory University, Atlanta, United States (Bloss, Cegielski) US Centers for Disease Control and Prevention, 1600 Clifton Rd, MS E-10, Atlanta, GA 30333, United States

(Shin, Bayona) Partners in Health, Boston, United States

(Shin, Contreras, Bayona) Socios En Salud, Lima, Peru

(Huaman, Ticona, Bonilla, Jave) Ministerio de Salud del Peru, Lima, Peru

(Yagui) Instituto Nacional de Salud, Lima, Peru

(Yagui) Universidad Nacional Mayor de San Marcos, Lima, Peru

Publisher

Elsevier (P.O. Box 211, Amsterdam 1000 AE, Netherlands)

Emtree Heading

adolescent; adult; article; body mass; cohort analysis; diabetes control; *diabetes mellitus/dt [Drug Therapy]; diabetic patient; disease association; *drug resistant tuberculosis; drug sensitivity; female; high risk patient; human; insulin treatment; laboratory; major clinical study; male; *multidrug resistant tuberculosis; outcome assessment; Peru; prospective study; sputum culture; insulin/dt [Drug Therapy]; oral antidiabetic agent/dt [Drug Therapy].

Link to the Ovid Full Text or citation:

Click here for full text options

162.

Risk factors influencing rebleeding after bronchial artery embolization on the management of hemoptysis associated with pulmonary tuberculosis.

Hwang H.-G., Lee H.-S., Choi J.-S., Seo K.-H., Kim Y.-H., Na J.-O.

Tuberculosis and Respiratory Diseases. 74 (3) (pp 111-119), 2013. Date of Publication: March 2013.

AN: 2013246252

Background: Hemoptysis due to pulmonary tuberculosis (TB) frequently develops in Korea where the prevalence of TB is intermediate. The effect of bronchial artery embolization (BAE) on the control of massive hemoptysis has been well known. This study is designed to identify the risk factors contributing to rebleeding after BAE in patients with TB. Methods: We retrospectively evaluated risk factors and the time for rebleeding after BAE in 72 patients presenting with hemoptysis. Results: The overall immediate success rate of BAE was 93.1% (67 of 72 patients). Of the 29 patients (40.3%) who showed rebleeding after BAE, 13 patients experienced rebleeding within 1 month, and 14 patients between 1 month to 1 year. The existence of a shunt in angiographic finding, aspergilloma, and diabetes mellitus were risk factors of rebleeding after BAE in multivariate analysis. Conclusion: BAE was very effective for obtaining immediate bleeding control in hemoptysis associated with active TB or post-TB sequelae. It is important to observe whether or not rebleeding occurs up to 1 year of BAE especially in TB patients with aspergilloma, DM, or a shunt. Even rebleeding can be managed well by second BAE. Copyright©2013. The Korean Academy of Tuberculosis and Respiratory Diseases. All rights reserved.

Institution

(Hwang) Department of Internal Medicine, Soonchunhyang University Gumi Hospital, Soonchunhyang University College of Medicine, Gumi, South Korea (Lee, Choi, Seo, Kim, Na) Department of Internal Medicine, Soonchunhyang University Cheonan Hospital, Soonchunhyang University College of Medicine, 31 Suncheonhyang 6-gil, Dongnam-gu, Cheonan 330-930, South Korea

Publisher

Korean National Tuberculosis Association (121-150 Dangsandong-6-ga Yeoungdeungpogu, Seoul 150-046, South Korea)

Emtree Heading

adult; aged; angiography; article; *artificial embolism; aspergilloma; *bronchial artery embolization; clinical evaluation; diabetes mellitus; female; *hemoptysis/th [Therapy]; human; *lung tuberculosis/dt [Drug Therapy]; major clinical study; male; outcome assessment; recurrent disease; retrospective study; risk factor; ethambutol/dt [Drug Therapy]; isoniazid/dt [Drug Therapy]; pyrazinamide/dt [Drug Therapy]; rifampicin/dt [Drug Therapy].

Link to the Ovid Full Text or citation:

Click here for full text options

163.

Impact of diabetes mellitus on treatment outcomes of tuberculosis patients in tertiary care setup.

Sulaiman S.A.S., Khan A.H., Muttalif A.R., Hassali M.A., Ahmad N., Iqubal M.S.

American Journal of the Medical Sciences. 345 (4) (pp 321-325), 2013. Date of Publication: April 2013.

AN: 2013237429

Background: Concurrent diabetes mellitus (DM) with tuberculosis (TB) has an increased risk of treatment failure. This study was aimed to evaluate treatment outcomes in patients with TB with and without DM. Methods: A retrospective cohort study was conducted at respiratory clinic of Hospital Pulau Pinang, Malaysia. All TB-registered patients from January 2006 to December 2007 were included in the study. A validated data collection form was used for collecting data. World Health Organization criterion was used for categorizing treatment outcomes. Data were analyzed by using SPSS 16. Results: Of 1267 patients, 338 patients (26.7%) had concurrent TB-DM. In multivariate analysis, TB-DM was more likely to be present in Chinese (odds ratio [OR] = 1.401, P = 0.011), patients having age of 46 to 60 years (OR = 3.168, P < 0.001) and >60 years (OR = 2.524, P < 0.001) and patients with pulmonary TB (OR = 2.079, P < 0.001). Nine hundred and eighty-five (78.8%) patients were successfully treated. No statistically significant difference was observed between 2 groups: patients with TB-DM and patients with only TB. Successful treatment outcomes were observed in patients having age of 46 to 60 (OR = 1.567, P = 0.001), whereas male gender (OR = 0.721, P = 0.049) and patients with relapse TB (OR = 0.494, P = 0.002) were less likely to have successful treatment outcome. Conclusions: High prevalence of TB-DM in the study signifies the fact that patients with DM are at high risk of developing TB. Treatment outcomes in both groups were comparable. The gender-based and age-based disparity in TB treatment outcomes in this study indicates the importance of gender-specific and age-specific strategies of TB management. Copyright © 2013 Lippincott Williams and Wilkins.

Institution

(Sulaiman, Khan, Ahmad, Iqubal) Department of Clinical Pharmacy, School of Pharmaceutical Sciences, Universiti Sains Malaysia, 11800 Penang, Malaysia (Muttalif) Department of Respiratory Medicine, Penang General Hospital, Penang, Malaysia

(Hassali) Discipline of Social and Administrative Pharmacy, School of Pharmaceutical Sciences, Universiti Sains Malaysia, 11800 Penang, Malaysia

Publisher

Lippincott Williams and Wilkins (530 Walnut Street,P O Box 327, Philadelphia PA 19106-3621, United States)

Emtree Heading

adult; article; clinical feature; *diabetes mellitus; diabetic patient; female; human; *lung tuberculosis; major clinical study; male; prevalence; relapse; retrospective study; tertiary health care; treatment outcome; world health organization.

Link to the Ovid Full Text or citation:

Click here for full text options

164.

Approval of a tuberculosis drug based on a paradoxical surrogate measure.

Avorn J.

JAMA - Journal of the American Medical Association. 309 (13) (pp 1349-1350), 2013. Date of Publication: 03 Apr 2013.

AN: 2013208618

Institution

(Avorn) Division of Pharmacoepidemiology and Pharmacoeconomics, Brigham and Women's Hospital, Harvard Medical School, Boston, MA 02120, United States

Publisher

American Medical Association (515 North State Street, Chicago IL 60654, United States)

Emtree Heading

cardiovascular risk; cholesterol blood level; diabetes mellitus/dt [Drug Therapy]; *drug approval; drug efficacy; drug mechanism; drug treatment failure; enzyme inhibition; heart infarction; heart ventricle fibrillation/si [Side Effect]; hemoglobin blood level; high risk patient; human; hypercholesterolemia/dt [Drug Therapy]; liver toxicity/si [Side Effect]; *multidrug resistant tuberculosis/dr [Drug Resistance]; *multidrug resistant tuberculosis/dt [Drug Therapy]; outcome assessment; priority journal; QT prolongation/si [Side Effect]; risk assessment; risk benefit analysis; short survey; sputum culture; bedaquiline/ae [Adverse Drug Reaction]; bedaquiline/dt [Drug Therapy]; bedaquiline/pd [Pharmacology]; ezetimibe/dt [Drug Therapy]; ezetimibe plus simvastatin; hemoglobin A1c/ec [Endogenous Compound]; low density lipoprotein cholesterol/ec [Endogenous Compound]; placebo; proton transporting adenosine triphosphatase/ec [Endogenous Compound]; rosiglitazone/dt [Drug Therapy]; sirturo; unclassified drug.

Drug Trade Names and Manufacturers

avandia, sirturo, vytorin, zetia

Link to the Ovid Full Text or citation:

Click here for full text options

165.

Commentary: Tuberculosis, diabetes and smoking: A burden greater than the sum of its parts.

Lyons J.G., Stewart S.

International Journal of Epidemiology. 42 (1) (pp 230-232), 2013. Article Number: dys217. Date of Publication: February 2013.

AN: 2013207969

Institution

(Lyons, Stewart) Preventative Health, Baker IDI Heart and Diabetes Institute, Melbourne, VIC, Australia

Publisher

Oxford University Press (Great Clarendon Street, Oxford OX2 6DP, United Kingdom)

Emtree Heading

acquired immune deficiency syndrome; cardiovascular disease; comorbidity; disease transmission; follow up; health care policy; human; Human immunodeficiency virus infection; lifestyle; lowest income group; morbidity; mortality; *non insulin dependent diabetes mellitus; note; obesity; outcome assessment; priority journal; risk factor; *smoking; smoking cessation; socioeconomics; *tuberculosis; world health organization.

Link to the Ovid Full Text or citation:

Click here for full text options

166.

Impact of Diabetes and Smoking on Mortality in Tuberculosis.

Reed G.W., Choi H., Lee S.Y., Lee M., Kim Y., Park H., Lee J., Zhan X., Kang H., Hwang S.H., Carroll M., Cai Y., Cho S.-N., Barry III C.E., Via L.E., Kornfeld H.

PLoS ONE. 8 (2) (no pagination), 2013. Article Number: e58044. Date of Publication: 28 Feb 2013.

AN: 2013139362

Background: Diabetes mellitus is a risk factor for tuberculosis (TB) disease. There is evidence that diabetes also influences TB severity and treatment outcomes but information is incomplete and some published results have been inconsistent. Methods: A longitudinal cohort study was conducted at the National Masan Tuberculosis Hospital in the Republic of Korea. Subjects presenting with a first episode of TB or for retreatment of TB were followed from enrollment through completion of treatment. Demographic, clinical, and microbiological variables were recorded, along with assessment of outcomes. Results were compared in TB patients with and without diabetes or smoking history. Data were adjusted for gender, age, cohort, educational level and alcohol consumption. Results: The combined cohorts comprised 657 subjects. Diabetes was present in 25% and was associated with greater radiographic severity and with recurrent or relapsed TB. Diabetes and cigarette smoking independently increased the risk of death in the first 12 months after enrollment. Estimating the combined impact of diabetes and smoking yielded a hazard ratio of 5.78. Only 20% of diabetic subjects were non-smokers; 54% smoked >1 pack daily. In this cohort, the impact of diabetes on mortality was greater in patients younger than 50 years, compared to older patients. Conclusions: In this cohort of Korean patients, diabetes exacerbated the severity of TB disease. Diabetic subjects who smoked >1 pack of cigarettes daily were at particularly high risk of death from TB. Strategies to improve TB outcomes could productively focus resources for patient education and TB prevention on the vulnerable population of younger diabetics, particularly those who also smoke.

Institution

(Reed) Department of Preventive and Behavioral Medicine, University of Massachusetts Medical School, Worcester, MA, United States (Choi, Lee, Lee, Kim, Park, Lee, Cho) International Tuberculosis Research Center, Changwon, South Korea

(Cho) Department of Microbiology, Yonsei University College of Medicine, Seoul, South Korea

(Zhan) Information Services Department, University of Massachusetts Medical School, Worcester, MA, United States

(Kang, Hwang) National Masan Tuberculosis Hospital, Changwon, South Korea

(Carroll, Cai, Barry III, Via) Tuberculosis Research Section, Laboratory of Clinical Infectious Disease, National Institute of Allergy and Infectious Diseases, Bethesda, MD, United States

(Kornfeld) Department of Medicine, University of Massachusetts Medical School, Worcester, MA, United States

Publisher

Public Library of Science (185 Berry Street, Suite 1300, San Francisco CA 94107, United States)

Emtree Heading

adult; adverse outcome; article; cohort analysis; comorbidity; controlled study; *diabetes mellitus; disease exacerbation; disease severity; female; hazard ratio; human; longitudinal study; *lung tuberculosis/dt [Drug Therapy]; major clinical study; male; mortality; multidrug resistant tuberculosis; recurrent infection; relapse; retreatment; *smoking; South Korea; survival rate; thorax radiography; ethambutol/cb [Drug Combination]; ethambutol/dt [Drug Therapy]; isoniazid/cb [Drug Combination]; isoniazid/dt [Drug Therapy]; pyrazinamide/cb [Drug Combination]; pyrazinamide/dt [Drug Therapy]; rifampicin/cb [Drug Combination]; rifampicin/dt [Drug Therapy].

Link to the Ovid Full Text or citation:

Click here for full text options

167.

The pieces of the puzzle - Disseminated bcg infection with mycotic abdominal aneurysm identified on a blood culture.

Denkinger C.M., Aroesty J.M., Weinberg A.N., Karchmer A.W.

Infectious Diseases in Clinical Practice. 21 (1) (pp 17-20), 2013. Date of Publication: January 2013.

AN: 2013023183

Intravesicular bacillus Calmette-Guerin (BCG) instillation is a widely used immunotherapy for superficial bladder cancer. Although this treatment is generally well tolerated with primarily local adverse effects, systemic adverse effects, including fever, pneumonitis, and hepatitis, have been reported. A disseminated infection with the development of mycotic aneurysms is very rare and results in significant morbidity and mortality.We report an immunocompetent patient with disseminated BCG infection and mycotic aneurysms diagnosed based on imaging and a positive mycolytic blood culture and subsequently confirmed by histopathology. We review the literature of other cases of disseminated BCG infection with mycotic aneurysms and summarize the classic presentation, the treatment, and outcomes. Copyright © 2013 by Lippincott Williams & Wilkins.

Institution

(Denkinger, Aroesty, Karchmer) Department of Medicine, Division of Infectious Diseases, Beth Israel Deaconess Medical Center, 110 Francis St, Boston, MA 02215, United States (Weinberg) Department of Medicine, Massachusetts General Hospital, Boston, MA, United States

Publisher

Lippincott Williams and Wilkins (530 Walnut Street,P O Box 327, Philadelphia PA 19106-3621, United States)

Emtree Heading

*abdominal aorta aneurysm/su [Surgery]; abdominal radiography; aged; anemia; anorexia; antibiotic therapy; artery anastomosis; bacterium identification; bladder carcinoma/dt [Drug Therapy]; bladder carcinoma/th [Therapy]; blood culture; bone marrow biopsy; *bovine tuberculosis/dt [Drug Therapy]; *bovine tuberculosis/si [Side Effect]; cachexia; cancer surgery; case report; chronic obstructive lung disease; computer assisted tomography; congestive cardiomyopathy; coughing; diabetes mellitus; disease duration; disease severity; *disseminated bacillus calmette guerin infection/dt [Drug Therapy]; *disseminated bacillus calmette guerin infection/si [Side Effect]; drug instillation; drug substitution; drug withdrawal; duodenum; dyspnea; enteritis; fatigue; fever; gastrointestinal endoscopy; heart atrium fibrillation; high risk patient; histopathology; human; hypertension; ileum; iliac artery; iliac artery aneurysm/su [Surgery]; immunocompetence; incubation time; kidney perfusion; leukocyte; low level laser therapy; male; medical history; muscle atrophy; Mycobacterium bovis; *mycotic aneurysm/dt [Drug Therapy]; *mycotic aneurysm/si [Side Effect]; *mycotic aneurysm/su [Surgery]; outcome assessment; pelvis radiography; physical examination; prostate cancer/su [Surgery]; prostatectomy; renal artery; review; saccular aneurysm/su [Surgery]; sedimentation rate; spleen artery; surgical risk; thorax radiography; toxic hepatitis/si [Side Effect]; transitional cell carcinoma/dt [Drug Therapy]; transitional cell carcinoma/th [Therapy]; treatment duration; upper gastrointestinal tract; urine culture; weakness; weight reduction; wheezing; *BCG vaccine/ae [Adverse Drug Reaction]; *BCG vaccine/dt [Drug Therapy]; ethambutol/dt [Drug Therapy]; indium 111; isoniazid/ae [Adverse Drug Reaction]; isoniazid/dt [Drug Therapy]; moxifloxacin/dt [Drug Therapy]; rifampicin/dt [Drug Therapy].

Link to the Ovid Full Text or citation:

Click here for full text options

168.

Association of diabetes Mellitus and tuberculosis in San Francisco.

Suwanpimolkul G., Grinsdale J., Jarlsberg L.G., Higashi J., Osmond D., Hopewell P.C., Kato-Maeda M.

American Journal of Respiratory and Critical Care Medicine. Conference: American Thoracic Society International Conference, ATS 2013 Philadelphia, PA United States. Conference Start: 20130517 Conference End: 20130522. Conference Publication: (var.pagings). 187 (no pagination), 2013. Date of Publication: 2013.

AN: 71981016

Rationale: Diabetes Mellitus (DM) is a risk factor for tuberculosis (TB). We explored the epidemiological, clinical and bacteriological characteristics of the association between Mycobacterium tuberculosis (MTB) infection and disease and DM among US-born individuals and foreign born persons from Latin America, China and the Philippines. Method: We performed a retrospective analysis of the clinical and epidemiological characteristics of individuals receiving services or managed by the San Francisco Department of Public Health TB clinic, as well as the analysis of the bacteriological data from patients with TB. The diagnosis of DM and epidemiological data was obtained from the TB patient management database. The classification of persons exposed to and or infected with MTB was based on the American Thoracic Society classification. Results: Between January 2005 and March 2012, 20,539 individuals were evaluated/managed at the TB clinic, 35% were born in the US, 28% were from countries in Latin America, 22% from China and 15% from the Philippines. Seventy-two percent (14,899) had latent TB infection (TB category 2), 17% (3,429) were not infected with MTB (TB categories 0 and 1) and 3% (627) had TB (TB category 3). The frequency of DM among TB category 0/1 for US, Latin America, China and the Philippines was: 4.4%, 4.3%, 3.9% and 6.8%, respectively. The frequency of DM among TB category 2 was similar in all groups: 6.4%, 7.2%, 6.3% and 7.8%, respectively, and the frequency of DM in category 3 was 9.2%, 12%, 17% and 32%, respectively. Compared with US, Individuals from China and the Philippines had a significantly higher proportion of DM and TB (US vs China, z-score 2.14 and p=0.032; US vs. the Philippines, z-score 2.71, p=0.006). The proportion of TB with DM in all population increased significantly in the last seven years (form 15.1% in 2005 to 26.5% in 2012; p-value for trend=0.01). Poor treatment outcome (death, adverse event, default, treatment failure) was not significantly different among TB versus TB with DM patients. Discussion /Conclusions. The proportion of TB with DM has increased significantly in the past 7 years in San Francisco. Data suggest that DM is more frequent in individuals from the Philippines, and TB and DM is more frequent in foreign-born persons from China and the Philippines. Prospective studies are needed to determine the incidence of TB among patients with DM in the different populations and to determine the need for targeted screening and prevention activities in these groups.

Institution

(Suwanpimolkul, Jarlsberg, Osmond, Hopewell, Kato-Maeda) Curry International Tuberculosis Center, Division of Pulmonary and Critical Care Medicine, University of California San Francisco, San Francisco, CA, United States (Grinsdale, Higashi) San Francisco Tuberculosis Control Section, San Francisco Department of Public Health, San Francisco, CA, United States

Publisher

American Thoracic Society

Emtree Heading

*diabetes mellitus; *tuberculosis; *United States; *American; *society; human; Philippines; China; South and Central America; patient; hospital; classification; population; infection; death; treatment outcome; diagnosis; statistical significance; public health; data base; patient care; screening; treatment failure; prospective study; epidemiological data; risk factor; prevention.

Link to the Ovid Full Text or citation:

Click here for full text options

169.

Impact of diabetes mellitus on treatment outcomes of patients with active tuberculosis in an endemic area.

Socaci A., Nini G., Marica C.

European Respiratory Journal. Conference: European Respiratory Society Annual Congress 2013 Barcelona Spain. Conference Start: 20130907 Conference End: 20130911. Conference Publication: (var.pagings). 42 (no pagination), 2013. Date of Publication: 01 Sep 2013.

AN: 71843977

Introduction. Diabetes mellitus (DM) is an emerging chronic health condition of developed and developing countries. Due to the association between diabetes and pulmonary tuberculosis (TB), diabetes may threaten the control of TB. Aims. We conducted a study to determine the prevalence of DM among TB patients, and to determine and compare treatment outcomes among TB patients with versus without DM. Methods. A retrospective cohort study included all patients with culture-confirmed TB diagnosed in 2010 and 2011, in west of the country. Results. Of 228 TB patients, 42 (18.4%) had DM. Patients with diabetes had 2.0 times higher odds of death than patients without diabetes (p = 0.18). Of the diabetic TB patients, 9.5% died within one month after starting TB treatment, compared with 3.5% of patients without diabetes (p = 0.09) Time to sputum culture conversion was longer in patients with diabetes than patients without diabetes (median 49 versus 39 days, p = 0.09). Two-month culture conversion proportions were similar (69% and 70%). There was no difference in the proportion of patients with baseline drug resistance, comparing patients with diabetes to patients without diabetes (14.3% versus 15.3%, p = 0.87). Treatment failure occurred in 4.1% of patients without diabetes and 6.7% of patients with diabetes (p = 0.51). Conclusions. In our TB patient population, DM was a risk factor for death, and it had a negative impact on treatment outcome. Enhanced medical vigilance, especially during the early part of TB treatment, is warranted.

Publisher

European Respiratory Society

Emtree Heading

*tuberculosis; *chronic disease; *treatment outcome; *diabetes mellitus; *patient; *human; *European; *society; death; lung tuberculosis; developing country; cohort analysis; sputum culture; health; prevalence; risk factor; population; drug resistance; treatment failure; alertness.

Link to the Ovid Full Text or citation:

Click here for full text options

170.

Impact of diabetes mellitus on drug-resistant tuberculosis treatment outcomes in Georgia-cohort study.

Kikvidze M., Mikiashvili L.

European Respiratory Journal. Conference: European Respiratory Society Annual Congress 2013 Barcelona Spain. Conference Start: 20130907 Conference End: 20130911. Conference Publication: (var.pagings). 42 (no pagination), 2013. Date of Publication: 01 Sep 2013.

AN: 71843894

Background. Diabetes mellitus (DM) is associated with an increased risk of tuberculosis (TB). There is growing evidence that this comorbidity might affect TB-disease presentation and treatment response. Aims and objectives. We investigated the level of impact of diabetes mellitus on drug-resistant tuberculosis (DR-TB) treatment outcomes in condition of adequate management of both diseases. Methods. Cohort study was provided among 1154 drug-resistant tuberculosis patients integrated in DOTS+ programme in 2009-2010 years in Georgia. Every patient was routinely investigated for DM. To compare the treatment outcomes of patients with and those without diabetes comorbidity dispersive analysis using Kruskal-Wallis - H criteria was provided. Results. Among 1154 DR-TB patients only 69 (5, 98%) had diabetes mellitus. All of them developed pulmonary form of TB and were HIV-negative. 61 (88, 4%) patient was male, mean age was 46, 8 (Standard deviation-12,67), mean BMI-23,44 (St.dev.- 3,78). In 3 (4,3%) patient was revealed extensively-DR-TB, in 58 (84,1%) - multi-DR-TB, and in 7 (10, 1%) - poly-DR-TB. 91,3% patients had II type of DM and 86,9% received insulin therapy. Definition: "successful treatment" received 55% diabetic patient vs 56 % non-diabetic, "Default"-36,2% vs 27,6%; "Failure" - 5,8 % vs 4,7 %; "Death" - 4,3% vs 9,6 % (p>0,05). Conclusions. We found no significant difference in proportion of successful treatment outcomes, though default rate was rather higher in patients with diabetes than non-diabetes in the setting of adequate treatment options and management in Georgia.

Institution

(Kikvidze, Mikiashvili) MDR-TB Department, National Center for Tuberculosis and Lung Diseases (NCTBLD), Tbilisi 0101, Georgia

Publisher

European Respiratory Society

Emtree Heading

*diabetes mellitus; *drug resistant tuberculosis; *treatment outcome; *cohort analysis; *European; *society; *tuberculosis; human; patient; comorbidity; treatment response; risk; death; diabetic patient; insulin treatment; spinal spacer; male; diseases; Human immunodeficiency virus; short course therapy.

Link to the Ovid Full Text or citation:

Click here for full text options

171.

Clinical profile of MDR-TB patients and their early response to DOTS PLUS: An Indian perspective.

Javia A., Mehta D., Ganchi F., Rathod B., Chopada K., Bhanderi M.

European Respiratory Journal. Conference: European Respiratory Society Annual Congress 2013 Barcelona Spain. Conference Start: 20130907 Conference End: 20130911. Conference Publication: (var.pagings). 42 (no pagination), 2013. Date of Publication: 01 Sep 2013.

AN: 71843868

Background:The emergence MDR-TB is an obstacle to effective TB control in India Aim:to analyze the MDR TB patients(Pt) & to evaluate their response to DOTS PLUS Methods: A prospective cohort study of 58 MDR-TB Pts. Pt's detailed demographic data recorded & put on DOTS PLUS.Clinical evaluation, sputum smear, culture & DST at the end of 3,4,5,6 and 9 month were done to monitor progress during treatment. Treatment outcomes were noted at the end of initiation phase (IP) Results:MDR-TB were common in males (M:F=3:2),in age group 18-35 yrs(64%),in lower socioeconomic class(88%),in Pts with BMI <18.5(89% M,96% F),in rural areas(48%),with bilateral(85%) & cavitory(67%) lesion on CXR.Commonest symptoms were cough(100%),fever(95%) & weight loss(60%). Associated with DM(8.6%) & HIV(5.2%).High grade sputum & culture positivity (+2 & +3) were more prevalent(60% & 69% respectively). 85% had resistance to both H & R. End IP smear & culture conversion rate was 72.4% and 56.9% respectively while 15.5% showed smear conversion but culture non-conversion. Mean smear & culture conversion time was 3.4 & 4.12 months respectively. Low smear & culture conversion rate was seen in Pts with H& R resistance,bilateral & cavitory lesions on CXR and initial high grade smear & culture positivity. 6 Pts(10.3%) died and 4 Pts(6.9%) defaulted during IP. 33% Pts had ADR, the commonest being GI disturbance(42%),hearing loss(32%) & psychiatric disturbance(16%) Conclusion:Early diagnosis & treatment of MDR-TB is essential. Resistance to both H & R,bilateral & cavitory lesion in CXR,high grade initial smear & culture positivity,low BMI and DM is associated with poor outcome. We will continue our study till completion of treatment.

Institution

(Javia, Mehta, Ganchi, Rathod, Chopada, Bhanderi) Pulmonary Medicine, M P Shah Medical College, Jamnagar, Gujarat 361008, India

Publisher

European Respiratory Society

Emtree Heading

*tuberculosis; *human; *diagnosis; *patient; *Indian; *European; *society; *short course therapy; smear; sputum smear; cohort analysis; weight reduction; gastrointestinal symptom; male; social class; groups by age; rural area; coughing; fever; treatment outcome; sputum; India; hearing impairment; early diagnosis; Human immunodeficiency virus.

Link to the Ovid Full Text or citation:

Click here for full text options

172.

Effect of diabetes on treatment outcome of smear positive pulmonary tuberculosis: A report from south india.

Viswanathan V., Aravindalochanan V., Kuppusamy S., Kumpatla S., Rajan R., Kapur A.

Diabetes. Conference: 73rd Scientific Sessions of the American Diabetes Association Chicago, IL United States. Conference Start: 20130621 Conference End: 20130625. Conference Publication: (var.pagings). 62 (pp A45-A46), 2013. Date of Publication: July 2013.

AN: 71286620

Aim was to assess the effect of diabetes on tuberculosis (TB) treatment outcome and sputum conversion among new smear positive (NSP) cases registered under Directly Observed Treatment Short Course in India. A cohort of NSP cases (N=332; M:F 247:85) registered at TB units of 3 districts in Tamil Nadu, viz, Thiruvallur, Kanchipuram and Chennai, from Jan-Mar 2011 were selected from a diabetes screening study. They were followed up for 8 months during TB treatment and details on sputum conversion and TB treatment outcome as per RNTCP were collected. Study subjects were categorized as diabetic (DM) (n=96; 73:23), pre-diabetic (PDM) (n=87; 65:22) and non-diabetic (NonDM) (n=149; 109:40) based on their OGTT values at the time of registration in DOTS. HbA1c% was estimated. Appropriate statistical analyses were done to compare outcome indicators between groups using SPSS version 16.0. Among the 332 TB patients, 305 (91.9%) were cured of TB, 6 (1.8%) completed treatment, 10 (3%) died, 5 (1.5%) had treatment default (TD), 5 (1.5%) had treatment failure (TF) and 1 (0.3%) developed MDR-TB. Mean age of DM group was significantly higher than PDM and NonDM groups (p<0.001). At the end of intensive phase of CAT-1 TB treatment, 14.7% remained sputum positive in DM group, which was higher than that of PDM (8.3%) and NonDM (3.5%). Mean duration (days) taken for sputum conversion was higher in DM (64.2+/-10.5) than PDM (61.8+/-7.2) and NonDM (61.5+/-7.5) [p=0.041]. TF rate was higher in DM group (4.2%) than NonDM (0.7%), and MDR-TB (1%) was seen in DM group. No Death and TD were seen among DM whereas it was 5.7 and 4.6% in PDM and 3.4 and 0.7% in NonDM, respectively. A positive weak correlation between baseline HbA1c and days needed for sputum conversion was observed only in DM group [r=0.2; p=0.08]. Delayed sputum conversion and high failure rates in TB treatment outcome were common in NSP pulmonary TB patients with diabetes. There were no reported deaths and treatment defaulters in TB patients with diabetes.

Institution

(Viswanathan, Aravindalochanan, Kuppusamy, Kumpatla, Rajan, Kapur) Chennai, India, Gentofte, Denmark

Publisher

American Diabetes Association Inc.

Emtree Heading

*diabetes mellitus; *lung tuberculosis; *smear; *treatment outcome; *India; human; sputum; patient; death; short course therapy; treatment failure; statistical analysis; astronomy; tuberculosis; registration; screening; hemoglobin A1c.

Link to the Ovid Full Text or citation:

Click here for full text options

173.

Impact of poor glycemic control on severity of pulmonary tuberculosis in new smear positive patients with type 2 diabetes mellitus.

Sindhuri A., Mahishale V., Patil B.B.

Lung India. Conference: National Conference on Pulmonary Diseases, NAPCON 2013 Chennai India. Conference Start: 20131128 Conference End: 20131130. Conference Publication: (var.pagings). 30 (pp S7), 2013. Date of Publication: October-December 2013.

AN: 71281996

Introduction: Diabetes mellitus (DM) is a known risk factor for tuberculosis (TB). Little is known about the effect of poor glycemic control on severity and outcome of TB. Aim: To study the effect of poor glycemic control on severity of pulmonary TB and outcome of TB treatment. Materials and Methods: In a tertiary care hospital, 70 patients, newly diagnosed with smear positive pulmonary TB and with coexistent type-II DM were enrolled. All patients underwent clinical, bacteriological, biochemical and radiological examinations at the initial enrolment and were followed-up for a total duration of 6 months. Clinical characteristics and outcome were compared between patients with poor glycemic control and optimal glycemic control. Results: At initial presentation, patients with poor glycemic control (hemoglobin A1C >8%) had more severe disease as compared to patients with optimal glycemic control, with greater sputum mycobacterial burden (sputum grade 3 50% vs. 4%, grade 2 30% vs. 14%; P < 0.001) and far advanced lesions on chest X-ray (40% vs. 2%; P < 0.001). After 2 months, sputum microscopy showed more positive results in patients with poor glycemic control (35% vs. 5%; P < 0.004). At the end of treatment these patients had greater failure rates with sputum smear positivity (23% vs. 4.3%; P < 0.04). Conclusions: Poorly controlled diabetes in pulmonary TB leads to severe disease with greater mycobacterial burden and far advanced lesions radiologically It was also observed that poor diabetic control had delayed sputum conversion and increased treatment failure rates among TB patients.

Institution

(Sindhuri, Mahishale, Patil) Department of Pulmonary Medicine, KLE University, Jawaharlal Nehru Medical College, Belgaum, Karnataka, India

Publisher

Medknow Publications and Media Pvt. Ltd

Emtree Heading

*glycemic control; *lung tuberculosis; *smear; *patient; *human; *non insulin dependent diabetes mellitus; *lung disease; sputum; diabetes mellitus; sputum cytodiagnosis; sputum smear; thorax radiography; radiodiagnosis; tertiary care center; treatment failure; diabetes control; tuberculosis; risk factor; hemoglobin.

Link to the Ovid Full Text or citation:

Click here for full text options

174.

The co-management of tuberculosis and diabetes: Challenges and opportunities in the developing world.

Sullivan T., Amor Y.B.

PLoS Medicine. 9 (7) (no pagination), 2012. Article Number: e1001269. Date of Publication: July 2012.

AN: 2012453742

Institution

(Sullivan) Department of Medicine, Mount Sinai Hospital, New York, NY, United States (Amor) The Earth Institute, Columbia University, New York, NY, United States

Publisher

Public Library of Science (185 Berry Street, Suite 1300, San Francisco CA 94107, United States)

Emtree Heading

article; cost effectiveness analysis; cost of illness; death; *diabetes mellitus; disease association; disease control; health care system; human; Human immunodeficiency virus infection; incidence; obesity; prevalence; risk factor; treatment failure; *tuberculosis.

Link to the Ovid Full Text or citation:

Click here for full text options

175.

[Tuberculosis of finger bones: three cases]. <Tuberculose digitale : a propos de trois cas.>

Loussaief C., Ammari F., Toumi A., Ben Brahim H., Ben Rhomdane F., Chakroun M.

La Revue de medecine interne / fondee ... par la Societe nationale francaise de medecine interne. 33 (4) (pp e22-24), 2012. Date of Publication: Apr 2012.

AN: 21549458

Tuberculosis dactylis is exceptional. We report tuberculous dactylitis in three women who were 51, 44, and 62-year-old, respectively. The diagnosis was suspected on chronic and insidious clinical presentation, and confirmed by histology. Disease course was favourable with antibuberculosis regimen but two patients had permanent hand disability. Clinical and therapeutic issues are discussed in the context of an endemic country. Copyright © 2011 Societe nationale Francaise de medecine interne (SNFMI). Published by Elsevier SAS. All rights reserved.

Institution

(Loussaief) Service des maladies infectieuses, EPS Fattouma Bourguiba, 5016 Monastir, Tunisie.

Emtree Heading

adult; article; biopsy; case report; female; *finger phalanx; human; *immunocompromised patient; microbiology; middle aged; *non insulin dependent diabetes mellitus/co [Complication]; pathology; radiography; risk factor; time; treatment outcome; tuberculin test; *tuberculous arthritis/co [Complication]; *tuberculous arthritis/di [Diagnosis]; *tuberculous arthritis/dt [Drug Therapy]; *tuberculostatic agent/dt [Drug Therapy].

Link to the Ovid Full Text or citation:

Click here for full text options

176.

Treatment outcomes of new smear positive pulmonary tuberculosis patients in North East Libya.

Solliman M.A., Hassali M.A., Al-Haddad M.S., Sulaiman S.A.S., Shafie A.A., Atif M., Saleem F.

Latin American Journal of Pharmacy. 31 (4) (pp 567-573), 2012. Date of Publication: -.

AN: 2012414592

A retrospective study conducted in North East of Libya to evaluate treatment outcome of smear positive pulmonary tuberculosis (PTB) patients and factors associated with unsuccessful outcome and treatment default has been developed. Three hundred and twenty seven patients were notified in Kuwaifia chest hospital, Benghazi and Shahat chest hospital, Shahat, during 2007-2008. Using the World Health Organization criteria, treatment completion and cure rate was 1.2% and 57.5%, respectively. Treatment failure occurred in 7(2.1%) cases. Ninety (27.5%) patients defaulted treatment, 11 (3.4%) died and 26 (8%) transferred out. Over all, successful and unsuccessful treatment outcome was 58.7% and 41.3%, respectively. Libyan nationality was the only predictor of successful treatment outcome while Libyan nationality and diabetes mellitus had negative association with treatment default. Improving clinical and laboratory infrastructure in peripheral areas, educating defaulters about benefits of completing therapy and stratifying foreigners as high risk groups could improve success rate. Measures should be taken to improve professional commitment and expertise of health care professionals.

Institution

(Solliman, Hassali, Shafie, Saleem) Discipline of Social and Administrative Pharmacy, School of Pharmaceutical Sciences, Universiti Sains Malaysia, Malaysia (Al-Haddad) College of Pharmacy, Taif University, Saudi Arabia

(Sulaiman, Atif) Discipline of Clinical Pharmacy, School of Pharmaceutical Sciences, Universiti Sains Malaysia, Malaysia

Publisher

Colegio de Farmaceuticos de la Provincia de Buenos Aires (Calle 5 No. 966, La Plata 1900, Argentina)

Emtree Heading

adult; article; clinical evaluation; diabetes mellitus; disease association; drug treatment failure; female; high risk population; human; Libyan Arab Jamahiriya; *lung tuberculosis/dt [Drug Therapy]; major clinical study; male; *outcome assessment; retrospective study; sputum smear; treatment outcome; unspecified side effect/si [Side Effect]; ethambutol/dt [Drug Therapy]; isoniazid/dt [Drug Therapy]; pyrazinamide/dt [Drug Therapy]; rifampicin/dt [Drug Therapy]; *tuberculostatic agent/ae [Adverse Drug Reaction]; *tuberculostatic agent/dt [Drug Therapy].

Link to the Ovid Full Text or citation:

Click here for full text options

177.

Emerging clinico-epidemiological trends in melioidosis: Analysis of 95 cases from western coastal India.

Vidyalakshmi K., Lipika S., Vishal S., Damodar S., Chakrapani M.

International Journal of Infectious Diseases. 16 (7) (pp e491-e497), 2012. Date of Publication: July 2012.

AN: 2012352623

Objectives: To study the clinico-epidemiological trends in melioidosis, an emerging disease in the western coastal region of India. Methods: Data of 95 patients with melioidosis in the western coastal region of India were retrospectively analyzed with respect to monthly rainfall, risk factors, clinical presentations, and outcome. Results: A strong linear correlation was seen between average monthly rainfall and the occurrence of cases (p= 0.002). Mortality was seen only in patients with bacteremia (p< 0.001). Nine (40.9%) patients with septic shock died (p< 0.001). Age >40 years and diabetes mellitus were seen in 75.8% of cases, each. Pneumonia was the most common clinical presentation (32.6%), followed by musculoskeletal disease (20%), melioidotic lymphadenopathy (7.4%), and dental abscess (6.3%). Only 36.8% of patients had exposure to wet soil/surface water. Conclusions: Melioidosis is quite prevalent in the western coastal region of India, and is strongly associated with rainfall, age, and diabetes mellitus. Higher proportions of musculoskeletal, dental, and lymph node melioidosis were seen in this region as compared to endemic areas. Bacteremic melioidosis has a poorer prognosis than non-bacteremic melioidosis. The presence of septic shock is a strong predictor of mortality. Percutaneous inoculation may not be the main portal of entry for Burkholderia pseudomallei in this region. © 2012 International Society for Infectious Diseases.

Institution

(Vidyalakshmi) Department of Microbiology, Kasturba Medical College, Manipal University, Light House Hill Road, Mangalore, Karnataka, 575001, India (Lipika) Department of Medical Microbiology, Post Graduate Institute of Medical Education and Research, Chandigarh, India

(Vishal) Department of Medicine, Kasturba Medical College, Manipal University, Manipal, Karnataka, India

(Damodar, Chakrapani) Department of Medicine, Kasturba Medical College, Manipal University, Mangalore, Karnataka, India

Publisher

Elsevier (P.O. Box 211, Amsterdam 1000 AE, Netherlands)

Emtree Heading

adult; age distribution; aged; article; aspiration biopsy; bacteremia; bacterial arthritis; blood culture; Burkholderia pseudomallei; child; clinical feature; comorbidity; diabetes mellitus; erythrocyte sedimentation rate; female; human; India; leukocyte count; lymphadenopathy; major clinical study; male; *melioidosis/dt [Drug Therapy]; *melioidosis/ep [Epidemiology]; mortality; musculoskeletal disease; Mycobacterium tuberculosis; outcome assessment; pleura effusion; pneumonia; preschool child; psoas abscess; retrospective study; risk factor; septic shock; septicemia; soil; spondylitis; tooth abscess; tuberculosis/dt [Drug Therapy]; ceftazidime/dt [Drug Therapy]; ceftazidime/iv [Intravenous Drug Administration]; chloramphenicol/dt [Drug Therapy]; chloramphenicol/iv [Intravenous Drug Administration]; cotrimoxazole/dt [Drug Therapy]; cotrimoxazole/iv [Intravenous Drug Administration]; doxycycline/dt [Drug Therapy]; doxycycline/iv [Intravenous Drug Administration]; rain; surface water; tuberculostatic agent/dt [Drug Therapy].

Link to the Ovid Full Text or citation:

Click here for full text options

178.

All you need to read in the other general journals.

Anonymous

BMJ (Online). 344 (7861) (no pagination), 2012. Article Number: e4055. Date of Publication: 16 Jun 2012.

AN: 2012345872

Publisher

BMJ Publishing Group (Tavistock Square, London WC1H 9JR, United Kingdom)

Emtree Heading

add on therapy; antibiotic resistance; article; bleeding/si [Side Effect]; *brain cancer; brain hemorrhage/si [Side Effect]; cancer incidence; cancer risk; cardiovascular disease/dt [Drug Therapy]; cardiovascular disease/pc [Prevention]; cardiovascular surgery; *cognitive therapy; *computer assisted tomography; depression/th [Therapy]; drug tolerability; drug withdrawal; gastrointestinal hemorrhage/si [Side Effect]; glucose blood level; glycemic control; health care quality; human; hypoglycemia/si [Side Effect]; intermethod comparison; *leukemia; lifestyle modification; low drug dose; medical decision making; multicenter study (topic); *multidrug resistant tuberculosis/dr [Drug Resistance]; nausea/si [Side Effect]; *non insulin dependent diabetes mellitus/dt [Drug Therapy]; *non insulin dependent diabetes mellitus/pc [Prevention]; *non insulin dependent diabetes mellitus/th [Therapy]; open study; pancreatitis/si [Side Effect]; patient compliance; prediction; priority journal; psychotherapy; radiation dose; randomized controlled trial (topic); remission; risk assessment; risk benefit analysis; side effect/si [Side Effect]; surgical mortality; survival rate; *telemedicine; treatment outcome; unnecessary procedure; weight gain; weight reduction; *acetylsalicylic acid/ae [Adverse Drug Reaction]; *acetylsalicylic acid/do [Drug Dose]; *acetylsalicylic acid/dt [Drug Therapy]; bedaquiline; delamanid; ethambutol; *exendin 4/ae [Adverse Drug Reaction]; *exendin 4/ct [Clinical Trial]; *exendin 4/cm [Drug Comparison]; *exendin 4/dt [Drug Therapy]; *glimepiride/ae [Adverse Drug Reaction]; *glimepiride/ct [Clinical Trial]; *glimepiride/cm [Drug Comparison]; *glimepiride/dt [Drug Therapy]; glucose/ec [Endogenous Compound]; hemoglobin A1c/ec [Endogenous Compound]; isoniazid; kanamycin; *metformin/dt [Drug Therapy]; ofloxacin; rifampicin; streptomycin; *troponin T/ec [Endogenous Compound].

Link to the Ovid Full Text or citation:

Click here for full text options

179.

Disease severity score could not predict the outcomes in peritoneal dialysis-associated peritonitis.

Dong J., Li Z., Xu R., Chen Y., Luo S., Li Y.

Nephrology Dialysis Transplantation. 27 (6) (pp 2496-2501), 2012. Date of Publication: June 2012.

AN: 2012322939

Background. We aim to explore if disease severity score (DSS) at onset is associated with dialysate white cell counts, the severity of causative organisms and the risk for treatment failure of peritoneal dialysis (PD)-associated peritonitis in an adult PD cohort. Methods .Our prospective cohort study recorded all peritonitis episodes between 2008 and 2010. The DSS, demographic data and clinical characteristics were recorded at the onset of peritonitis. The dialysate cells were counted at regular intervals and organism culture were examined too. Treatment failure of peritonitis was defined as peritonitis-associated death and transfer to haemodialysis. Results. A total of 219 episodes of peritonitis in 146 PD patients were recorded, 21.9% of which resulted in treatment failure. There were no significant differences in dialysate white cell counts on the fifth and seventh day and the distribution of causative organism between groups with varied DSS level. DSS could not predict treatment failure including peritonitis-related death and transfer to haemodialysis after adjusting for age, gender, diabetes, dialysis duration, dialysate white cell count on the third day, the presence of Staphylococcus aureus, gram-negative organisms and polymicrobial organisms. Conclusion. Our study demonstrated that DSS at onset was not associated with prolonged elevation of dialysate white cell counts, severity of causative organisms and outcome of peritonitis episodes in adult PD patients. © 2012 The Author.

Institution

(Dong, Li, Xu, Chen, Luo, Li) Department of Medicine, Peking University, First Medical Hospital, Peking, China (Li) Department of Nephrology, West China Hospital of Sichuan University, Sichuan, China

Publisher

Oxford University Press (Great Clarendon Street, Oxford OX2 6DP, United Kingdom)

Emtree Heading

abdominal pain; Acinetobacter; adult; age; aged; article; cohort analysis; controlled study; Corynebacterium; diabetes mellitus; dialysate; dialysate white cell count; *disease severity; *disease severity score; Enterococcus; Escherichia coli; female; fever; fungus; gender; Gram negative bacterium; Gram positive bacterium; hemodialysis; human; Klebsiella; leukocyte count; major clinical study; male; mortality; *peritoneal dialysis; *peritonitis/co [Complication]; priority journal; prospective study; Pseudomonas; *scoring system; Staphylococcus; Staphylococcus aureus; Streptococcus; treatment duration; treatment failure; treatment outcome; tuberculosis.

Link to the Ovid Full Text or citation:

Click here for full text options

180.

Pulmonary Tuberculosis and delay in anti-tuberculous treatment are important risk factors for chronic obstructive pulmonary disease.

Lee C.-H., Lee M.-C., Lin H.-H., Shu C.-C., Wang J.-Y., Lee L.-N., Chao K.-M.

PLoS ONE. 7 (5) (no pagination), 2012. Article Number: e37978. Date of Publication: 25 May 2012.

AN: 2012302785

Objective: Tuberculosis (TB) remains the leading cause of death among infectious diseases worldwide. It has been suggested as an important risk factor of chronic obstructive pulmonary disease (COPD), which is also a major cause of morbidity and mortality. This study investigated the impact of pulmonary TB and anti-TB treatment on the risk of developing COPD. Design, Setting, and Participants: This cohort study used the National Health Insurance Database of Taiwan, particularly the Longitudinal Health Insurance Database 2005 to obtain 3,176 pulmonary TB cases and 15,880 control subjects matched in age, sex, and timing of entering the database. Main Outcome Measures: Hazard ratios of potential risk factors of COPD, especially pulmonary TB and anti-TB treatment. Results: The mean age of pulmonary TB cases was 51.9+/-19.2. The interval between the initial study date and commencement of anti-TB treatment (delay in anti-TB treatment) was 75.8+/-65.4 days. Independent risk factors for developing COPD were age, male, low income, and history of pulmonary TB (hazard ratio 2.054 [1.768-2.387]), while diabetes mellitus was protective. The impact of TB persisted for six years after TB diagnosis and was significant in women and subjects aged >70 years. Among TB patients, delay in anti-TB treatment had a dose-response relationship with the risk of developing COPD. Conclusions: Some cases of COPD may be preventable by controlling the TB epidemic, early TB diagnosis, and prompt initiation of appropriate anti-TB treatment. Follow-up care and early intervention for COPD may be necessary for treated TB patients. © 2012 Lee et al.

Institution

(Lee, Chao) Graduate Institute of Biomedical Electronics and Bioinformatics, National Taiwan University, Taipei, Taiwan (Republic of China) (Lee) Department of Internal Medicine, Buddhist Tzu Chi General Hospital, Taipei Branch, New Taipei, Taiwan (Republic of China)

(Lee) School of Medicine, Tzu Chi University, Hualien, Taiwan (Republic of China)

(Lee) Department of Pharmacy, Buddhist Tzu Chi General Hospital, Taipei Branch, New Taipei, Taiwan (Republic of China)

(Lin) Graduate Institute of Epidemiology and Preventive Medicine, National Taiwan University, Taipei, Taiwan (Republic of China)

(Shu) Department of Traumatology, National Taiwan University Hospital, Taipei, Taiwan (Republic of China)

(Shu, Wang) Department of Internal Medicine, National Taiwan University Hospital, Taipei, Taiwan (Republic of China)

(Lee) Department of Laboratory Medicine, National Taiwan University Hospital, Taipei, Taiwan (Republic of China)

Publisher

Public Library of Science (185 Berry Street, Suite 1300, San Francisco CA 94107, United States)

Emtree Heading

adult; age; aged; article; *chronic obstructive lung disease/di [Diagnosis]; cohort analysis; controlled study; data base; diabetes mellitus; disease association; female; gender; human; lowest income group; *lung tuberculosis/di [Diagnosis]; major clinical study; male; medical history; national health insurance; outcome assessment; patient attitude; patient compliance; patient participation; *risk assessment; risk factor; Taiwan; *therapy delay; tuberculosis/dt [Drug Therapy]; *tuberculostatic agent/dt [Drug Therapy].

Link to the Ovid Full Text or citation:

Click here for full text options

181.

High Mortality Rate of Infectious Diseases in Dialysis Patients: A Comparison With the General Population in Japan.

Wakasugi M., Kawamura K., Yamamoto S., Kazama J.J., Narita I.

Therapeutic Apheresis and Dialysis. 16 (3) (pp 226-231), 2012. Date of Publication: June 2012.

AN: 2012296508

Infectious disease is the second leading cause of death among dialysis patients, and it is generally assumed that the mortality rate of infectious disease is considerably higher in dialysis patients than in the general population. There are no comprehensive studies on this issue and on the contribution of each category of infectious disease to excess mortality in dialysis patients in Japan. We used mortality data reported to the Japanese Society for Dialysis Therapy and national Vital Statistics data for 2008 and 2009. We calculated standardized mortality ratios and compared the mortality rates for each category of infectious disease. During the 2-year study period, 274683 and 10435 deaths from infectious diseases were recorded in 126 million people and 273237 dialysis patients, respectively. The standardized mortality ratio for all infectious diseases was 7.5 (95% confidence interval, 7.3-7.6) in dialysis patients with respect to the general population in Japan. The categories of infectious disease with a significantly higher standardized mortality ratio among the dialysis patients were sepsis, peritonitis, influenza, tuberculosis, and pneumonia and in that order. In particular, the mortality rate of sepsis contributed to 69.5% of the difference in infectious disease mortality between dialysis patients and the general population. This study underlines markedly increased mortality from infectious diseases, particularly from sepsis, in dialysis patients compared with the general population. © 2012 The Authors. Therapeutic Apheresis and Dialysis © 2012 International Society for Apheresis.

Institution

(Wakasugi) Center for Inter-organ Communication Research, Niigata University Graduate School of Medical and Dental Science, Niigata, Japan (Kawamura, Yamamoto, Narita) Division of Clinical Nephrology and Rheumatology, Niigata University Graduate School of Medical and Dental Science, Niigata, Japan

(Kazama) Division of Blood Purification Therapy, Niigata University Medical and Dental Hospital, Niigata, Japan

Publisher

Blackwell Publishing Inc. (350 Main Street, Malden MA 02148, United States)

Emtree Heading

adolescent; adult; aged; article; child; controlled study; *diabetic nephropathy/th [Therapy]; epidemiological data; female; *hemodialysis; *hemodialysis patient; human; infant; *infection/ep [Epidemiology]; *influenza/ep [Epidemiology]; Japan; major clinical study; male; medical record; medical society; mortality; *peritoneal dialysis; *peritonitis/ep [Epidemiology]; *pneumonia/ep [Epidemiology]; population; preschool child; priority journal; risk factor; school child; *sepsis/ep [Epidemiology]; standardized mortality ratio; treatment outcome; *tuberculosis/ep [Epidemiology]; vital statistics.

Link to the Ovid Full Text or citation:

Click here for full text options

182.

Role of diabetes in the prognosis and therapeutic outcome of tuberculosis.

Syed Suleiman S.A., Ishaq Aweis D.M., Mohamed A.J., Razakmuttalif A., Moussa M.A.A.

International Journal of Endocrinology. 2012 (no pagination), 2012. Article Number: 645362. Date of Publication: 2012.

AN: 2012278888

Background. Increased susceptibility of diabetic mellitus (DM) patients to infection, including tuberculosis (TB), is well documented. The prevalence of DM in Malaysia is reaching epidemic proportions. In this study, we sought to assess risk factors for TB and the impact of DM on the outcome of TB treatment. Methods. TB patients, diabetic patients, and diabetic patients with TB were divided into three groups of 200 subjects each. Data were obtained from patients' medical files at the beginning and end of the study period. Prevalence rates of DM and HIV among TB patients were assessed. Prognosis, TB-related complications, anatomical site of infection, and duration of infection and diabetes were also examined. Results. The prevalence rates of HIV and DM amongst TB patients were 7.7 and 30%, respectively. The diabetic TB patient group contained more males (72%) and smokers (45.5%) compared to the nondiabetic group (58.3% and 33.5%, resp.). Approximately 74% of diabetic patients were Mycobacterium sputum positive compared to only 51% of nondiabetic patients. Diabetic patients were also more likely to develop pulmonary TB (87%) compared to nondiabetic TB patients (59%). Diabetic TB patients had a higher mortality rate (7.5%) compared to the TB only and DM only groups (1 and 2%, resp.). The duration of TB symptoms was longer in nondiabetic TB patients compared to diabetic TB patients (4.5 versus 2.6 months, resp.). Diabetes antedated TB by a mean time of 4 years. Conclusions. We found a higher number of sputum-smear-positive cases and pulmonary TB cases as well as a greater number of males and higher mortality rate in diabetic patients compared to nondiabetic patients. Copyright © 2012 Syed Azhar Syed Suleiman et al.

Institution

(Syed Suleiman) School of Pharmaceutical Sciences, USM, 11800 Penang, Malaysia (Ishaq Aweis) Department of Clinical Pharmacy, School of Pharmaceutical Sciences, USM, 11800 Penang, Malaysia

(Ishaq Aweis) Ahmadi Hospital, Kuwait Oil Company, Kuwait

(Mohamed) Department of Pharmacology, School of Pharmaceutical Sciences, USM, 11800 Penang, Malaysia

(Mohamed) Faculty of Medicine, Benadir University, Mogadishu, Somalia

(Razakmuttalif) Respiratory Clinic, Hospital Pulau Pinang (HPP), Malaysia

(Moussa) Department of Community Medicine and Behavioural Sciences, Faculty of Medicine, Kuwait University, Kuwait

Publisher

Hindawi Publishing Corporation (410 Park Avenue, 15th Floor, 287 pmb, New York NY 10022, United States)

Emtree Heading

adult; article; cohort analysis; controlled study; *diabetes mellitus/et [Etiology]; disease duration; female; high risk population; human; Human immunodeficiency virus infection/ep [Epidemiology]; lung tuberculosis; major clinical study; male; mortality; Mycobacterium; prevalence; priority journal; *prognosis; risk factor; sex difference; smoking; *treatment outcome; *tuberculosis.

Link to the Ovid Full Text or citation:

Click here for full text options

183.

Predictors of mortality in staphylococcus aureus bacteremia.

van Hal S.J., Jensen S.O., Vaska V.L., Espedido B.A., Paterson D.L., Gosbell I.B.

Clinical Microbiology Reviews. 25 (2) (pp 362-386), 2012. Date of Publication: April 2012.

AN: 2012205805

Staphylococcus aureus bacteremia (SAB) is an important infection with an incidence rate ranging from 20 to 50 cases/100,000 population per year. Between 10% and 30% of these patients will die from SAB. Comparatively, this accounts for a greater number of deaths than for AIDS, tuberculosis, and viral hepatitis combined. Multiple factors influence outcomes for SAB patients. The most consistent predictor of mortality is age, with older patients being twice as likely to die. Except for the presence of comorbidities, the impacts of other host factors, including gender, ethnicity, socioeconomic status, and immune status, are unclear. Pathogen-host interactions, especially the presence of shock and the source of SAB, are strong predictors of outcomes. Although antibiotic resistance may be associated with increased mortality, questions remain as to whether this reflects pathogen-specific factors or poorer responses to antibiotic therapy, namely, vancomycin. Optimal management relies on starting appropriate antibiotics in a timely fashion, resulting in improved outcomes for certain patient subgroups. The roles of surgery and infectious disease consultations require further study. Although the rate of mortality from SAB is declining, it remains high. Future international collaborative studies are required to tease out the relative contributions of various factors to mortality, which would enable the optimization of SAB management and patient outcomes. © 2012, American Society for Microbiology. All Rights Reserved.

Institution

(van Hal, Gosbell) Department of Microbiology and Infectious Diseases, Sydney South West Pathology Service-Liverpool, South Western Sydney Local Health Network, Sydney, NSW, Australia (van Hal, Jensen, Espedido, Gosbell) Antibiotic Resistance and Mobile Elements Group, Microbiology and Infectious Diseases Unit, School of Medicine, University of Western Sydney, Sydney, NSW, Australia

(Jensen, Espedido, Gosbell) Ingham Institute of Applied Medical Research, Sydney, NSW, Australia

(Vaska, Paterson) The University of Queensland, UQ Centre for Clinical Research (UQCCR), Herston, QLD, Australia

Publisher

American Society for Microbiology (1752 N Street N.W., Washington DC 20036-2904, United States)

Emtree Heading

acquired immune deficiency syndrome; alcoholism; bacterial endocarditis; bactericidal activity; bone and joint infections/ep [Epidemiology]; Caucasian; cellular immunity; central nervous system infection/ep [Epidemiology]; chronic kidney failure; cohort analysis; confidence interval; congestive heart failure; crowding; device infection/co [Complication]; diabetes mellitus; drug fatality/si [Side Effect]; ethnicity; gender; hematologic malignancy; host pathogen interaction; human; humoral immunity; immune deficiency/cn [Congenital Disorder]; immune status; in vitro study; innate immunity; intensive care unit; liver cirrhosis; meta analysis (topic); methicillin resistant Staphylococcus aureus; methicillin resistant Staphylococcus aureus infection/dt [Drug Therapy]; mortality; multidrug resistance; multivariate analysis; neutropenia; newborn intensive care; penicillin resistance; phagocyte; postoperative complication/co [Complication]; prospective study; prostate cancer; prosthetic valve endocarditis/co [Complication]; prosthetic valve endocarditis/et [Etiology]; randomized controlled trial (topic); retrospective study; review; risk; sepsis; shock; social status; socioeconomics; soft tissue infection/co [Complication]; *staphylococcal bacteremia/dr [Drug Resistance]; *staphylococcal bacteremia/ep [Epidemiology]; *staphylococcal bacteremia/et [Etiology]; Staphylococcus infection/dt [Drug Therapy]; surgical infection/co [Complication]; survival prediction; systematic review (topic); treatment failure; tuberculosis; United States; unspecified side effect/si [Side Effect]; virus hepatitis; cefazolin/dt [Drug Therapy]; cloxacillin/dt [Drug Therapy]; cotrimoxazole/cm [Drug Comparison]; cotrimoxazole/dt [Drug Therapy]; exotoxin; flucloxacillin; gentamicin/ae [Adverse Drug Reaction]; levofloxacin; linezolid/ae [Adverse Drug Reaction]; linezolid/cm [Drug Comparison]; meticillin; rifampicin/pk [Pharmacokinetics]; vancomycin/cm [Drug Comparison]; vancomycin/dt [Drug Therapy].

Link to the Ovid Full Text or citation:

Click here for full text options

184.

Risk of tuberculosis among people with diabetes mellitus: An Australian nationwide cohort study.

Dobler C.C., Flack J.R., Marks G.B.

BMJ Open. 2 (1) (no pagination), 2012. Article Number: 000666. Date of Publication: 2012.

AN: 2012134172

Objective: Previous studies that have found an increased risk for tuberculosis (TB) in people with diabetes mellitus (DM) have been conducted in segments of the population and have not adjusted for important potential confounders. We sought to determine the RR for TB in the presence of DM in a national population with data on confounding factors in order to inform the decision-making process about latent tuberculosis infection (LTBI) screening in people with diabetes. Design: Whole population historical cohort study. Setting: All Australian States and Territories with a mean TB incidence of 5.8/100 000. Participants: Cases of TB in people with DM were identified by record linkage using the National Diabetes Services Scheme Database and TB notification databases for the years 2001-2006. Primary and secondary outcome measures: Primary outcome was notified cases of TB. Secondary outcome was notified cases of culture-confirmed TB. RR of TB was estimated with adjustment for age, sex, TB incidence in country of birth and indigenous status. Results: There were 6276 cases of active TB among 19 855 283 people living in Australia between 2001 and 2006. There were 271 (188 culture positive) cases of TB among 802 087 members of the DM cohort and 130 cases of TB among 273 023 people using insulin. The crude RR of TB was 1.78 (95% CI 1.17 to 2.73) in all people with DM and 2.16 (95% CI 1.19 to 3.93) in people with DM using insulin. The adjusted RRs were 1.48 (95% CI 1.04 to 2.10) and 2.27 (95% CI 1.41 to 3.66), respectively. Conclusions: The presence of DM alone does not justify screening for LTBI. However, when combined with other risk factors for TB, the presence of DM may be sufficient to justify screening and treatment for LTBI.

Institution

(Dobler, Marks) Department of Respiratory and Environmental Epidemiology, Woolcock Institute of Medical Research, University of Sydney, Sydney, Australia (Dobler, Marks) Department of Respiratory Medicine, Liverpool Hospital, Sydney, Australia

(Flack) Diabetes Centre, Bankstown-Lidcombe Hospital, Sydney, Australia

Publisher

BMJ Publishing Group (Tavistock Square, London WC1H 9JR, United Kingdom)

Emtree Heading

article; Australia; diabetes mellitus/dt [Drug Therapy]; *diabetic patient; disease surveillance; female; high risk population; human; *latent tuberculosis; major clinical study; male; medical decision making; outcome assessment; prevalence; risk factor; screening test; insulin/dt [Drug Therapy].

Link to the Ovid Full Text or citation:

Click here for full text options

185.

Unresolved antiretroviral treatment management issues in HIV-infected children.

Heidari S., Mofenson L.M., Hobbs C.V., Cotton M.F., Marlink R., Katabira E.

Journal of Acquired Immune Deficiency Syndromes. 59 (2) (pp 161-169), 2012. Date of Publication: 01 Feb 2012.

AN: 2012086339

Antiretroviral therapy in children has expanded dramatically in low-income and middle-income countries. The World Health Organization revised its pediatric HIV guidelines to recommend initiation of antiretroviral therapy in all HIV-infected children younger than 2 years, regardless of CD4 count or clinical stage. The number of children starting life-long antiretroviral therapy should therefore expand dramatically over time. The early initiation of antiretroviral therapy has indisputable benefits for children, but there is a paucity of definitive information on the potential adverse effects. In this review, a comprehensive literature search was conducted to provide an overview of our knowledge about the complications of treating pediatric HIV. Antiretroviral therapy in children, as in adults, is associated with enhanced survival, reduction in opportunistic infections, improved growth and neurocognitive function, and better quality of life. Despite antiretroviral therapy, HIV-infected children may continue to lag behind their uninfected peers in growth and development. In addition, epidemic concurrent conditions, such as tuberculosis, malaria, and malnutrition, can combine with HIV to yield more rapid disease progression and poor treatment outcomes. Additional studies are required to evaluate the long-term effects of antiretroviral therapy in HIV-infected infants, children, and adolescents, particularly in resource-limited countries where concomitant infections and conditions may enhance the risk of adverse effects. There is an urgent need to evaluate drug-drug interactions in children to determine optimal treatment regimens for both HIV and coinfections. Copyright © 2012 Lippincott Williams & Wilkins.

Institution

(Heidari) Department of Research Promotion, International AIDS Society, Geneva, Switzerland (Mofenson) Pediatric, Adolescent and Maternal AIDS Branch, National Institutes of Health, Bethesda, United States

(Hobbs) Laboratory of Malaria Immunology and Vaccinology, National Institute of Allergy and Infectious Disease, National Institutes of Health, Rockville, United States

(Cotton) Department of Pediatrics and Child Health, Stellenbosch University and Tygerberg Children's Hospital, South Africa

(Marlink) Harvard School of Public Health, Boston, United States

(Marlink) Elizabeth Glaser Pediatric AIDS Foundation, Los Angeles, CA, United States

(Katabira) Makerere University College of Health Sciences, Kampala, Uganda

Publisher

Lippincott Williams and Wilkins (530 Walnut Street,P O Box 327, Philadelphia PA 19106-3621, United States)

Emtree Heading

anemia/si [Side Effect]; body weight; bone development; bone mineral; cardiovascular disease/ep [Epidemiology]; CD4 lymphocyte count; child; child development; child growth; child nutrition; childhood disease; clinical practice; cognitive defect; comorbidity; diabetes mellitus/ep [Epidemiology]; drug safety; drug substitution; drug withdrawal; dyslipidemia/co [Complication]; dyslipidemia/et [Etiology]; gastrointestinal toxicity/si [Side Effect]; *highly active antiretroviral therapy; HIV associated dementia/co [Complication]; human; Human immunodeficiency virus infected patient; *Human immunodeficiency virus infection/dt [Drug Therapy]; hypercholesterolemia/co [Complication]; hypercholesterolemia/et [Etiology]; hypercholesterolemia/si [Side Effect]; hyperlipidemia/dt [Drug Therapy]; hyperlipidemia/si [Side Effect]; immune reconstitution inflammatory syndrome/si [Side Effect]; insulin resistance/co [Complication]; insulin resistance/et [Etiology]; lipodystrophy/co [Complication]; lipodystrophy/et [Etiology]; lipodystrophy/si [Side Effect]; liver toxicity/si [Side Effect]; *malaria/dt [Drug Therapy]; *malaria/ep [Epidemiology]; *malaria/pc [Prevention]; *malnutrition/co [Complication]; *malnutrition/ep [Epidemiology]; *malnutrition/pc [Prevention]; *malnutrition/th [Therapy]; metabolic disorder/co [Complication]; metabolic disorder/et [Etiology]; neurotoxicity/si [Side Effect]; neutropenia/si [Side Effect]; peripheral neuropathy/si [Side Effect]; practice guideline; preschool child; priority journal; QT prolongation/si [Side Effect]; rash/si [Side Effect]; review; risk factor; side effect/si [Side Effect]; survival rate; treatment outcome; *tuberculosis/dt [Drug Therapy]; *tuberculosis/ep [Epidemiology]; *tuberculosis/pc [Prevention]; *tuberculosis/si [Side Effect]; virus load; vitamin D deficiency/ep [Epidemiology]; world health organization; abacavir/ae [Adverse Drug Reaction]; abacavir/dt [Drug Therapy]; amodiaquine/ae [Adverse Drug Reaction]; amodiaquine/it [Drug Interaction]; amodiaquine/dt [Drug Therapy]; *antimalarial agent/it [Drug Interaction]; *antimalarial agent/dt [Drug Therapy]; *antimalarial agent/pk [Pharmacokinetics]; *antiretrovirus agent/ae [Adverse Drug Reaction]; *antiretrovirus agent/cr [Drug Concentration]; *antiretrovirus agent/it [Drug Interaction]; *antiretrovirus agent/dt [Drug Therapy]; *antiretrovirus agent/pk [Pharmacokinetics]; artemether/it [Drug Interaction]; artemether/dt [Drug Therapy]; artemether/pk [Pharmacokinetics]; BCG vaccine/ae [Adverse Drug Reaction]; BCG vaccine/dt [Drug Therapy]; benflumetol/ae [Adverse Drug Reaction]; benflumetol/it [Drug Interaction]; benflumetol/dt [Drug Therapy]; benflumetol/pk [Pharmacokinetics]; cotrimoxazole/ae [Adverse Drug Reaction]; cotrimoxazole/dt [Drug Therapy]; cycloserine/ae [Adverse Drug Reaction]; cycloserine/dt [Drug Therapy]; didanosine/ae [Adverse Drug Reaction]; didanosine/dt [Drug Therapy]; efavirenz/ae [Adverse Drug Reaction]; efavirenz/cr [Drug Concentration]; efavirenz/it [Drug Interaction]; efavirenz/dt [Drug Therapy]; efavirenz/pk [Pharmacokinetics]; hydroxymethylglutaryl coenzyme A reductase inhibitor/dt [Drug Therapy]; isoniazid/ae [Adverse Drug Reaction]; isoniazid/dt [Drug Therapy]; lopinavir/ae [Adverse Drug Reaction]; lopinavir/it [Drug Interaction]; lopinavir/dt [Drug Therapy]; nelfinavir/ae [Adverse Drug Reaction]; nelfinavir/dt [Drug Therapy]; nevirapine/ae [Adverse Drug Reaction]; nevirapine/dt [Drug Therapy]; *nonnucleoside reverse transcriptase inhibitor/ae [Adverse Drug Reaction]; *nonnucleoside reverse transcriptase inhibitor/it [Drug Interaction]; *nonnucleoside reverse transcriptase inhibitor/dt [Drug Therapy]; *proteinase inhibitor/ae [Adverse Drug Reaction]; *proteinase inhibitor/dt [Drug Therapy]; rifampicin/ae [Adverse Drug Reaction]; rifampicin/it [Drug Interaction]; rifampicin/dt [Drug Therapy]; ritonavir/ae [Adverse Drug Reaction]; ritonavir/it [Drug Interaction]; ritonavir/dt [Drug Therapy]; stavudine/ae [Adverse Drug Reaction]; stavudine/dt [Drug Therapy]; tenofovir/ae [Adverse Drug Reaction]; tenofovir/dt [Drug Therapy]; *tuberculostatic agent/ae [Adverse Drug Reaction]; *tuberculostatic agent/it [Drug Interaction]; *tuberculostatic agent/dt [Drug Therapy]; virus RNA; zidovudine/ae [Adverse Drug Reaction]; zidovudine/dt [Drug Therapy].

Link to the Ovid Full Text or citation:

Click here for full text options

186.

Predictive factors for anti-tuberculosis treatment failure.

Lesnic E., Vasile Z.

European Respiratory Journal. Conference: European Respiratory Society Annual Congress 2012 Vienna Austria. Conference Start: 20120901 Conference End: 20120905. Conference Publication: (var.pagings). 40 (no pagination), 2012. Date of Publication: 01 Sep 2012.

AN: 71926136

The effectiveness of DOTS achieved 59% in Moldova in 2011. The result of an inadequate treatment is the treatment failure. Its increasing rate (7.5% in 2011) leads to accumulation of MDR sources in the population and puts more problems to the National TB Control Program. Treatment outcome depends by the social categories of patients, TB extension and co-morbidities. Aim of study is evaluation of predictive factors for anti-TB treatment failure (TF). Was compared risk factors for TF in a study group (SG) of 110 patients with pulmonary TB failed after 5 months of DOTS and a control group (CG) of 100 patients cured with DOTS. Results: males 60% SG vs 50% CG, average age 35,6 SG vs 42,5 CG, unemployed 76% SG vs 48% CG, with bad living conditions 63% SG vs 48% CG (p<0.05). Were detected 80% of patients from both groups by passive way with known TB contact 26% SG vs 20% CG. All patients (100%) had destructive forms of TB with positive sputum, but bilateral extension had 84% of SG vs 24% in CG(p<0.05). Susceptibility test detected primary resistance in 55% SG vs 12% CG. Co-morbidities had 56% of SG vs 24% in CG, most frequent: alcoholism, hepatitis, diabetes, HIV infection. Noncompliance with treatment had 20% from SG vs 100% compliance in CG. Treatment outcome in SG: cured 42%, 30% dead,10% defaulted,18% continue another treatment regimen vs 100% cured in CG. Conclusions predictive factors for TF: are treatment not adapted to susceptibility testing, noncompliance with treatment, low social status, extensive TB with comorbidities.

Publisher

European Respiratory Society

Emtree Heading

*treatment failure; *European; *society; *tuberculosis; human; patient; treatment outcome; morbidity; short course therapy; hepatitis; diabetes mellitus; male; alcoholism; Moldova; sputum; population; control group; social status; Human immunodeficiency virus infection; risk factor; unemployment; Human immunodeficiency virus.

Link to the Ovid Full Text or citation:

Click here for full text options

187.

Treatment outcome of multi-drug-resistant tuberculosis patients at DOTS Plus site, Trivandrum.

Nair D.S., Anitha Kumari K., Nair S.

Lung India. Conference: National Conference on Pulmonary Diseases, NAPCON 2011 New Delhi India. Conference Start: 20111127 Conference End: 20111130. Conference Publication: (var.pagings). 29 (pp S21), 2012. Date of Publication: March 2012.

AN: 71210730

Background: The DOTS Plus site at Trivandrum started providing treatment to multi-drug-resistant tuberculosis (MDR TB) patients in December 2008. It provides DOTS Plus treatment to seven districts in South Kerala. This study looks into the treatment outcome of MDR-TB patients started on Cat IV RNTCP treatment. Objectives: 1. To study the treatment outcome of MDR TB patients on Cat IV RNTCP treatment. 2. To study the risk factors associated with adverse outcomes. Study Design: Cross-sectional study. Study Setting: DOTS-Plus site, Trivandrum, located at the Department of Pulmonary Medicine, Medical College, Trivandrum. Study Period: July 2010 to July 2011. Data Collection and Analysis: Patient details collected using a structured questionnaire after getting informed consent and analyzed by Epi-info-3.5.1. Results: Of the 70 MDR TB patients started on Cat IV ATT, 55 were males and 15 were females, the mean age being 42.2 years. 15.7% of the patients were declared cured, 21% have completed treatment, 31.5% have defaulted and 31.5% patients died. The risk factors associated with death during treatment in this study are low body mass index (BMI), higher age and male sex. Diabetes mellitus had no significant association with death or default during treatment for this cohort. Conclusion: Main factors associated with adverse outcomes were low BMI, higher age and male sex. Strategies should be designed to address these so as to improve the treatment outcomes for MDR patients.

Institution

(Nair, Anitha Kumari, Nair) Department of Pulmonary Medicine, Medical College, Trivandrum, Kerala, India

Publisher

Medknow Publications and Media Pvt. Ltd

Emtree Heading

*treatment outcome; *multidrug resistant tuberculosis; *human; *lung disease; *patient; *short course therapy; cat; male; adverse outcome; risk factor; death; body mass; cross-sectional study; informed consent; study design; structured questionnaire; information processing; medical school; diabetes mellitus; pulmonology; female.

Link to the Ovid Full Text or citation:

Click here for full text options

188.

Tuberculosis treatment outcomes in patients with and without diabetes mellitus.

Khan A.H., Sulaiman S.A.S., Abdul Muttalif A.R., Hassali M.A., Ahmad N.

Value in Health. Conference: ISPOR 15th Annual European Congress Berlin Germany. Conference Start: 20121103 Conference End: 20121107. Conference Publication: (var.pagings). 15 (7) (pp A387), 2012. Date of Publication: November 2012.

AN: 70916571

OBJECTIVES: To evaluate treatment outcomes in tuberculosis patients with and without diabetes mellitus. METHODS: This was a retrospective cohort study conducted at respiratory clinic of Hospital Pulau Pinang (HPP), Malaysia. All Tuberculosis (TB) patients who were presented to the clinic from January 2006 to December 2007 were included in the study. A purpose developed valid data collection form was used for collecting demographic and clinical data. Treatment outcomes initially recorded as cured, treatment completed, defaulted, transferred out, expired and treatment continued were then classified into two categories; successful and unsuccessful treatment. Cured and treatment completed patients were placed in treatment successful category while the rest were placed in the category of unsuccessful treatment. Data was analyzed by using SPSS 16. RESULTS: Final analysis included 1266 patients.Three hundred and thirty eight patients (26.7%) had DM along with TB. In multivariate analysis TB-DM was more likely to be present in Chinese (OR=1.470, p-value=0.003), married patients (OR=1.408, p-value=0.011) and patients having age 46-60 years (OR=2.002, p-value<0.001), and>60 years (OR = 1.594, p-value = 0.010). Nine hundred and eighty five (78.8%) patients were successfully treated. Successful Treatment was observed in patients having age of 46-60 years (OR=1.567, p-value=0.001), whereas male gender (OR=0.721, p-value = 0.049) and patients with relapse TB (OR = 0.494, p-value = 0.002) were less likely to have successful treatment. CONCLUSIONS: In the present study Chinese ethnicity, age (> 46 years) and being married were the predictors of prevalence of TB-DM. Majority patients were successfully treated. Male gender and patients with relapse TB were the predictors of treatment failure. No statistically significant difference in treatment outcomes was observed between TB patients with and without diabetes mellitus.

Institution

(Khan, Sulaiman) Universiti Sains Malaysia, Penang, Pulau Pinang, Malaysia (Abdul Muttalif) Respiratory Institute of Malaysia, Kula Lumpur, Malaysia

(Hassali) Universiti Sains Malaysia, Minden, Penang, Malaysia

(Ahmad) Universiti Sains Malaysia, Pinang, Penang, Malaysia

Publisher

Elsevier Ltd

Emtree Heading

*treatment outcome; *patient; *human; *diabetes mellitus; *tuberculosis; statistical significance; hospital; gender; male; relapse; betel nut; multivariate analysis; cohort analysis; clinical study; information processing; prevalence; Malaysia; ethnicity; treatment failure.

Link to the Ovid Full Text or citation:

Click here for full text options

189.

Risk factors for failure in treatment of Pulmonary Tuberculosis (PTB): Study from a rural medical college hospital in south India.

Venkatarathnamma P.N., Bhatta S.A.

International Journal of Infectious Diseases. Conference: 15th International Congress on Infectious Diseases, ICID 2012 Bangkok Thailand. Conference Start: 20120613 Conference End: 20120616. Conference Publication: (var.pagings). 16 (pp e278), 2012. Date of Publication: June 2012.

AN: 70869129

Background: Tuberculosis (TB) is amajor global health problem, with India accounting for one-fifth of the global TB cases. Each year nearly 2 million people in India develop TB, of which around 0.87 million are infectious cases and annually around 330,000 die due to TB. Modern Anti-tubercular treatment (ATT) can cure virtually all patients provided correct combination treatment is taken. Some patients with tuberculosis fail, relapse or default from initial treatment. There is increased risk of morbidity and transmission, among those on Category II ATT regimen. Methods: The objective was to evaluate the factors responsible for initial treatment failure and default, subsequent retreatment and outcome. Methods: All cases of pulmonary tuberculosis started on Category I RNTCP (Revised National Tuberculosis Control Programme called DOTS elsewhere) from January 2011 to December 2011 were analysed for treatment given, risk factors of treatment failure and outcome. Results: A total of 245 cases were diagnosed as PTB(Pulmonary Tubeculosis) and started on Category I ATT. 18 were retreatment cases who had relapse, or defaulted, with male: female ratio of 17:1. Of these 18, whowere started on category II regimen,risk factors for initial treatment failure were undernourishment (100%), illiteracy (44%), rural patients (72%), smoking (55%), deliberate neglect (16%), alcohol abuse (27%), Diabetes mellitus (16%), vomiting (16%) and ex-miners. Retreatment was successful in 9 of these patients, while 3 patients further defaulted. 3 patients on retreatment died, 1 due to massive hemoptysis and 2 due to respiratory failure. 3 were lost for follow up. Conclusion: Retreatment of TB patients is a challenge to the TB control services, as they require longer treatment duration with at least 5 drugs (including an injectable). They are also more likely to harbour and transmit drug-resistant tuberculosis with poor treatment outcomes. Default from treatment was high in rural areas. Preventing the need for retreatment in the first place is the best strategy. Identifying patients that confer higher risk of relapse, failure, or default from primary TB treatment, and targeted interventions such as health education, substance abuse counselling, enhanced tracking, or reinforcement of DOTS supervision may help to reduce the need for retreatment, resulting in cost savings and diminished morbidity and transmission.

Institution

(Venkatarathnamma, Bhatta) Sri Devaraj Urs Medical College, Kolar, Karnataka, India

Publisher

Elsevier

Emtree Heading

*lung tuberculosis; *medical school; *hospital; *India; *infection; *risk factor; human; patient; retreatment; relapse; treatment failure; morbidity; tuberculosis; risk; smoking; malnutrition; female; counseling; tuberculosis control; health education; male; cost control; rural area; alcohol abuse; treatment outcome; health; drug resistant tuberculosis; treatment duration; follow up; respiratory failure; hemoptysis; miner; vomiting; diabetes mellitus; substance abuse; reinforcement; short course therapy.

Link to the Ovid Full Text or citation:

Click here for full text options

190.

Pancreatic tuberculosis mimicking carcinoma: A case series and review of the imaging and literature.

Fletcher T., Din I., Aston S., Miller A., Beeching N., Joekes E.

Clinical Microbiology and Infection. Conference: 22nd European Congress of Clinical Microbiology and Infectious Diseases London United Kingdom. Conference Start: 20120331 Conference End: 20120403. Conference Publication: (var.pagings). 18 (pp 556-557), 2012. Date of Publication: April 2012.

AN: 70823671

Introduction: The incidence of tuberculosis (TB) in developed countries is increasing and pulmonary disease remains the most common presentation. Abdominal tuberculosis is a frequent site of extra-pulmonary infection and has a wide spectrum of clinical presentation. Isolated pancreatic TB is rare and is normally reported in association with immunosuppression. The presentation and imaging often mimic pancreatic carcinoma, with tuberculosis only diagnosed after laparotomy and histological examination. Methods: We present a rare case series of five patients with confirmed pancreatic tuberculosis that have been managed in our regional infectious disease unit in the previous 10 years. Two cases were diagnosed post-operatively following Whipple's procedure for suspected carcinoma. We also discuss the imaging characteristics of pancreatic TB, and the diagnostic role of endoscopic ultrasound-guided fine needle aspiration (EUS-FNA) in pancreatic lesions. Discussion: This case series highlights that the incidence of pancreatic tuberculosis is low and the diagnosis and appropriate treatment opportunity are often missed. It should always be considered in patients with suspected pancreatic malignancy. We present five cases which also demonstrate that pancreatic TB is not always associated with immunosuppression. Patients often have recognised risk factors, particularly their country of origin and it has a wide spectrum of clinical and radiological presentation. 556-557 P1948 Comparative evaluation of severe and mild forms of extrapulmonary tuberculosis: 202 cases E. Kursun*, T. Turunc, Y.Z. Demiroglu, H. Arslan (Ankara, TR) Objectives: Tuberculosis can involve any organ system in the body. In this study, we aimed to comparative evaluation of severe and mild forms of the extrapulmonary tuberculosis (EPT) in our clinic. Methods: In this prospective study, 202 HIV seronegative patients of EPT who admitted to our department between January 2001 and October 2011 were included. The patients were evaluated with respect to epidemiological and clinical features, laboratory results and treatment outcome in two groups such as severe and mild forms of EPT. Results: The age range of the patients were 16-84 years old (mean age: 46.2 +/- 16.6) and 109 of them (54%) patients were female. The most common sites of involvement were lymph nodes (37.1%). There was an underlying disease in 56 (27.2%) of the patients (diabetes mellitus in 12.9%; chronic renal dysfunction in 10.4%, malignancy in 4%), history of passed tuberculosis infection in 21 (10.4%) and history of contact with tuberculosis patient in 41 (%20.3). According to criteria of Word Health Organization (WHO), tuberculosis which is miliary with meningeal, pericardial, pleural effusion that is either bilateral or diffuse, which has spinal, intestinal, or more than one bone and joint involvement or genitourinary involvement is classified as severe clinical form; where as tuberculosis that goes with lymphadenitis, unilateral pleural effusion, involvement of peripheral joints and skin, excluding spine is classified as mild clinical form. Severe and mild forms of EPT were present in 97 (48%) and 105 (52%) of the patients, respectively. In comparison of the patients with severe and mild forms of EPT, the severe form were detected more frequently in males (p = 0.01), the positivity rates of culture and acid-fast staining were higher in patients with severe form (p < 0.001 and p = 0.02, respectively). The mortality rate was also found higher in patients with severe form (p = 0.01). Histopathological evaluation was more useful in the mild group. Conclusion: Investigation of epidemiological and clinical characteristics of extrapulmonary tuberculosis on the large series of cases may be essential in early diagnosis and treatment and also prevention of disease especially in endemic countries.

Institution

(Fletcher, Din, Aston, Miller, Beeching, Joekes) LiverpoolUnited Kingdom

Publisher

Blackwell Publishing Ltd

Emtree Heading

*carcinoma; *case study; *imaging; *microbiology; *infection; *tuberculosis; human; patient; extrapulmonary tuberculosis; pleura effusion; immunosuppressive treatment; diagnosis; abdominal tuberculosis; pancreas carcinoma; lung disease; skin; bone; lung infection; developed country; female; examination; lymphadenitis; spine; procedures; mortality; endoscopic echography; laparotomy; treatment outcome; laboratory; clinical feature; prospective study; lymph node; diabetes mellitus; health care organization; hospital; organ systems; risk factor; male; staining; early diagnosis; prevention; aspiration biopsy; Human immunodeficiency virus; acid.

Link to the Ovid Full Text or citation:

Click here for full text options

191.

Chronic disease in the Caribbean: Strategies to respond to the public health challenge in the region.

Ferguson T.S., Tulloch-Reid M.K., Cunningham-Myrie C.A., Davidson-Sadler T., Copeland S., Lewis-Fuller E., Wilks R.J.

West Indian Medical Journal. 60 (4) (pp 397-411), 2011. Date of Publication: July 2011.

AN: 2012529275

With the advent of the epidemiological transition, chronic non-communicable diseases (CNCDs) have emerged as the leading cause of death globally. In this paper, we present an overview of the burden of CNCDs in the Caribbean region and use Jamaica as a case-study to review the impact of policy initiatives and interventions implemented in response to the CNCD epidemic. The findings show that while Jamaica has implemented several policy initiatives aimed at stemming the tide of the CNCD epidemic, a comparison of data from two national health and lifestyle surveys conducted in Jamaica in 2000/01 and 2007/08 revealed that there was an increase in the prevalence of intermediate CNCD risk factors such as hypertension and obesity. We therefore present recommended strategies which we believe will enhance the current CNCD response and thus reduce, or at least stem, the current epidemic of CNCDs.

Institution

(Ferguson, Tulloch-Reid, Cunningham-Myrie, Wilks) Tropical Medicine Research Institute (Epidemiology Research Unit), The University of the West Indies, Kingston, Jamaica (Davidson-Sadler, Copeland, Lewis-Fuller) Ministry of Health, Jamaica

Publisher

University of the West Indies (Mona, Kingston 7, Jamaica)

Emtree Heading

article; cancer control; cancer epidemiology; cancer mortality; cardiovascular disease; Central America; *chronic disease/ep [Epidemiology]; *chronic disease/pc [Prevention]; chronic kidney disease; congenital malformation/ep [Epidemiology]; congenital malformation/pc [Prevention]; decision making; diabetes mellitus/ep [Epidemiology]; diabetes mellitus/pc [Prevention]; dietary intake; disease control; disease surveillance; documentation; endocrine disease/ep [Epidemiology]; endocrine disease/pc [Prevention]; epidemic; evidence based medicine; gastrointestinal disease/ep [Epidemiology]; gastrointestinal disease/pc [Prevention]; government; health behavior; health care organization; health care policy; health care quality; health center; health service; health survey; heart infarction; help seeking behavior; human; Human immunodeficiency virus; hypothesis; immobilization; information dissemination; Jamaica; law; lifestyle; malignant neoplastic disease/ep [Epidemiology]; malignant neoplastic disease/pc [Prevention]; morbidity; mortality; nephritis/ep [Epidemiology]; nephritis/pc [Prevention]; neuropsychiatry; organization and management; outcome assessment; pneumonia/ep [Epidemiology]; pneumonia/pc [Prevention]; practice guideline; premature mortality; respiratory tract disease/ep [Epidemiology]; respiratory tract disease/pc [Prevention]; risk factor; smoking cessation; social marketing; stroke; syphilis/ep [Epidemiology]; syphilis/pc [Prevention]; tuberculosis; urogenital tract disease/ep [Epidemiology]; urogenital tract disease/pc [Prevention]; wellbeing.

Link to the Ovid Full Text or citation:

Click here for full text options

192.

TB and diabetes - the dual epidemic: Is it a matter of concern?.

Behera D.

Indian Journal of Tuberculosis. 58 (4) (pp 143-147), 2011. Date of Publication: 2011.

AN: 2012421276

Institution

(Behera) LRS Institute of TB and Respiratory Diseases, New Delhi, India

Publisher

Tuberculosis Association of India (3 Red Cross Road, New Delhi 110001, India)

Emtree Heading

abdominal obesity; adverse outcome; blood sampling; cause of death; developing country; diabetes control; *diabetes mellitus/dt [Drug Therapy]; *diabetes mellitus/ep [Epidemiology]; diabetic nephropathy/co [Complication]; disease association; disease surveillance; editorial; *epidemic; geographic distribution; glycemic control; human; Human immunodeficiency virus infection; hyperglycemia/si [Side Effect]; hypertension; impaired glucose tolerance; liver toxicity/si [Side Effect]; lowest income group; lung tuberculosis; outcome assessment; prevalence; risk assessment; risk factor; rural area; short course therapy; *tuberculosis/dt [Drug Therapy]; *tuberculosis/ep [Epidemiology]; tuberculosis control; urban area; urban population; oral antidiabetic agent/ae [Adverse Drug Reaction]; oral antidiabetic agent/it [Drug Interaction]; oral antidiabetic agent/dt [Drug Therapy]; oral antidiabetic agent/po [Oral Drug Administration]; rifampicin/ae [Adverse Drug Reaction]; rifampicin/it [Drug Interaction]; rifampicin/dt [Drug Therapy]; tuberculostatic agent/ae [Adverse Drug Reaction]; tuberculostatic agent/dt [Drug Therapy].

Link to the Ovid Full Text or citation:

Click here for full text options

193.

The timing of death in patients with tuberculosis who die during anti-tuberculosis treatment in Andhra Pradesh, South India.

Jonnalagada S., Harries A.D., Zachariah R., Satyanarayana S., Tetali S., Keshav Chander G., Rao S., Rao R., Peri S., Anchala R., Kannuri N.K.

BMC public health. 11 (pp 921), 2011. Date of Publication: 2011.

AN: 22166132

India has 2.0 million estimated tuberculosis (TB) cases per annum with an estimated 280,000 TB-related deaths per year. Understanding when in the course of TB treatment patients die is important for determining the type of intervention to be offered and crucially when this intervention should be given. The objectives of the current study were to determine in a large cohort of TB patients in India:- i) treatment outcomes including the number who died while on treatment, ii) the month of death and iii) characteristics associated with "early" death, occurring in the initial 8 weeks of treatment. This was a retrospective study in 16 selected Designated Microscopy Centres (DMCs) in Hyderabad, Krishna and Adilabad districts of Andhra Pradesh, South India. A review was performed of treatment cards and medical records of all TB patients (adults and children) registered and placed on standardized anti-tuberculosis treatment from January 2005 to September 2009. There were 8,240 TB patients (5183 males) of whom 492 (6%) were known to have died during treatment. Case-fatality was higher in those previously treated (12%) and lower in those with extra-pulmonary TB (2%). There was an even distribution of deaths during anti-tuberculosis treatment, with 28% of all patients dying in the first 8 weeks of treatment. Increasing age and new as compared to recurrent TB disease were significantly associated with "early death". In this large cohort of TB patients, deaths occurred with an even frequency throughout anti-TB treatment. Reasons may relate to i) the treatment of the disease itself, raising concerns about drug adherence, quality of anti-tuberculosis drugs or the presence of undetected drug resistance and ii) co-morbidities, such as HIV/AIDS and diabetes mellitus, which are known to influence mortality. More research in this area from prospective and retrospective studies is needed.

Institution

(Jonnalagada) LEPRA India - Blue Peter Public Health & Research Centre, Near TEC Building, Cherlapally, Hyderabad 501301, Andhra Pradesh, India.

Emtree Heading

adult; article; female; human; India/ep [Epidemiology]; *lung tuberculosis/dt [Drug Therapy]; male; medical audit; middle aged; mortality; retrospective study; time; treatment outcome; *tuberculostatic agent/ad [Drug Administration]; *tuberculostatic agent/dt [Drug Therapy].

Link to the Ovid Full Text or citation:

Click here for full text options

194.

Is there delay in diagnosis of pulmonary tuberculosis in an intermediate to low TB incidence setting.

Malbasa M., Pesut D.

Pneumologia. 60 (3) (pp 138-142), 2011. Date of Publication: 2011.

AN: 22097435

A cross-sectional study on pulmonary TB diagnosis delay in an intermediate TB incidence setting showed average patient's delay of 44+/-61.65days and total delay of 103+/-148days. Alcoholism, lack of TB cases in family, diabetes mellitus, relapse, cough or tachycardia (p<0.01), absence of hemoptysis, dyspnea and anemia (p<0.01), age >40 (p<0.05), negative auscultation and positive sputum smear findings (p<0.05) were significantly associated with patient's delay >30 days. Age <40 years, negative auscultation and sputum smear findings (p<0.01), female sex, city as residence (p<0.05), absence of cough, sputum, weight loss, fever, excavation (p<0.01), and night sweats (p<0.05) were significantly associated with total delay > 103 days. Further population education and continual medical education are waranted.

Institution

(Malbasa) School of Medicine, University of Belgrade, Medicine Research Centre, Serbia (Pesut) School of Medicine, University of Belgrade, Internal Medicine Department, Serbia

(Pesut) Teaching Hospital of Lung Diseases, Clinical Centre of Serbia, Visegradska 26/20, 11000 Belgrade, Serbia

Publisher

Romanian Society of Pneumology (Sos. Viilor 90, sector 5, Bucharest RO-050159, Romania)

Emtree Heading

adult; aged; anemia; article; coughing; cross-sectional study; *delayed diagnosis; dyspnea; female; hemoptysis; human; incidence; isolation and purification; *lung tuberculosis/co [Complication]; *lung tuberculosis/di [Diagnosis]; *lung tuberculosis/dt [Drug Therapy]; *lung tuberculosis/ep [Epidemiology]; *lung tuberculosis/pc [Prevention]; male; microbiology; middle aged; *Mycobacterium tuberculosis; *patient education; prognosis; questionnaire; risk assessment; risk factor; sputum; time; treatment outcome; Yugoslavia/ep [Epidemiology]; tuberculostatic agent/dt [Drug Therapy].

Link to the Ovid Full Text or citation:

Click here for full text options

195.

Pitavastatin: Novel effects on lipid parameters.

Chapman M.J.

Atherosclerosis Supplements. 12 (3) (pp 277-284), 2011. Date of Publication: November 2011.

AN: 2011675734

Atherogenic dyslipidemia is characterised by high levels of triglycerides, low levels of high-density lipoprotein-cholesterol (HDL-C), and moderate to marked elevations in low-density lipoprotein-cholesterol (LDL-C) concentrations; such dyslipidemia is further characterised by high apolipoprotein B (apoB): apolipoprotein A1 (apoA1) ratios. Numerous clinical trials have demonstrated that statins are effective in lowering LDL-C and reducing cardiovascular (CV) risk in people with dyslipidemia. However, the most effective treatments should target all of the key atherogenic features, rather than LDL-C alone. Pitavastatin is a new member of the statin class whose distinct pharmacological features translate into a broad spectrum of action on both apoB-containing and apoA1-containing lipoprotein components of the atherogenic lipid profile. The efficacy and safety of this statin has been demonstrated by a large clinical development programme conducted both in Japanese and Caucasian populations. Phase III and IV studies in a wide range of patients with primary hypercholesterolemia or combined dyslipidemia showed that 12 weeks' treatment with pitavastatin l-4 mg was well tolerated, significantly improved lipid profiles (including LDL-C, TG, and HDL-C levels) and increased the EAS-/NCEP ATP Ill-recommended LDL-C target attainment rate to a similar or greater degree as comparable doses of atorvastatin, simvastatin, or pravastatin. Results were similar across all patient groups and were generally sustained after 52 weeks of treatment. However, whereas the effects of atorvastatin and simvastatin on HDL-C levels remained constant over the long term, pitavastatin-treated patients experienced progressive and maintained elevations in HDL-C, ultimately increasing by up to 14.3% vs. initial baseline. In this context, it is significant that the in vitro studies of Yamashita et al. [J Atheroscler Thromb 2010;17:436-51] have shown pitavastatin to be distinguished by its potent stimulation of apoA1 production in hepatocyte-like cells. These findings suggest that pitavastatin may be highly efficacious in raising levels of lipid-poor apoA1 particles, which are known to be highly active in ABCA1-mediated cellular cholesterol efflux, an observation which is pertinent to the excessive accumulation of cholesterol in macrophage foam cells of the atherosclerotic plaque. Indeed, the intravascular remodelling and maturation of lipid-poor apoA1 particles is known to drive flux of apoA1, cholesterol and phospholipid through the HDL pathway. It is equally relevant that pitavastatin therapy has been shown to be efficacious in markedly reducing coronary atheroma volume in acute coronary syndrome patients in the JAPAN-ACS trial, a therapeutic effect which may be linked to its impact on apoA1/HDL metabolism and function. Overall, Phase III and IV studies demonstrate that pitavastatin 1-4 mg is well tolerated, attenuates the atherogenic lipid profile and increases LDL-C target attainment rates with a similar or greater efficacy to comparable doses of atorvastatin, simvastatin and pravastatin. Furthermore, pitavastatin may be particularly beneficial in high-risk patients with elevated concentrations of TG-rich lipoproteins and low levels of HDL-C, and in whom the atheroprotective function of HDL particles is typically defective; significantly, such patients typically exhibit persistent, residual cardiometabolic risk even when LDL-C is at goal. In this context, it is relevant that such patient groups cover a wide spectrum of metabolic diseases, including metabolic syndrome, type 2 diabetes, coronary disease, familial and non-familial forms of hypercholesterolemia, auto-immune diseases such as rheumatoid arthritis and lupus, renal disease and some forms of hepatic insufficiency. © 2011 Elsevier Ireland Ltd.

Institution

(Chapman) Institut National de la Sante et de la Recherche Medicale (INSERM) UMR-S939, Pavillon Benjamin Delessert, Hopital de la Pitie, 83 Blud l'Hopital, 75651 Paris Cedex 13, France

Publisher

Elsevier Ireland Ltd (P.O. Box 85, Limerick, Ireland)

Emtree Heading

acute coronary syndrome; article; atheroma; atherosclerosis; atherosclerotic plaque; autoimmune disease; cardiovascular risk; Caucasian; coronary artery disease; drug dose titration; drug efficacy; drug mechanism; drug tolerability; *dyslipidemia/dt [Drug Therapy]; high risk population; human; *hypercholesterolemia/dt [Drug Therapy]; in vitro study; Japanese; kidney disease; liver failure; lupus vulgaris; macrophage; metabolic syndrome X; myopathy/si [Side Effect]; myositis/si [Side Effect]; non insulin dependent diabetes mellitus; phase 3 clinical trial (topic); phase 4 clinical trial (topic); priority journal; rhabdomyolysis/si [Side Effect]; rheumatoid arthritis; treatment outcome; apolipoprotein A1/ec [Endogenous Compound]; apolipoprotein B/ec [Endogenous Compound]; atorvastatin/cm [Drug Comparison]; atorvastatin/dt [Drug Therapy]; cholesterol/ec [Endogenous Compound]; high density lipoprotein cholesterol/ec [Endogenous Compound]; hydroxymethylglutaryl coenzyme A reductase inhibitor/pd [Pharmacology]; *lipid/ec [Endogenous Compound]; low density lipoprotein cholesterol/ec [Endogenous Compound]; phospholipid/ec [Endogenous Compound]; *pitavastatin/ae [Adverse Drug Reaction]; *pitavastatin/ct [Clinical Trial]; *pitavastatin/cm [Drug Comparison]; *pitavastatin/dt [Drug Therapy]; *pitavastatin/pd [Pharmacology]; pravastatin/ae [Adverse Drug Reaction]; pravastatin/cm [Drug Comparison]; pravastatin/dt [Drug Therapy]; simvastatin/cm [Drug Comparison]; simvastatin/dt [Drug Therapy]; triacylglycerol/ec [Endogenous Compound].

Link to the Ovid Full Text or citation:

Click here for full text options

196.

Predictors of treatment failure among pulmonary tuberculosis patients in mulago hospital, Uganda.

Namukwaya E., Nakwagala F.N., Mulekya F., Mayanja-Kizza H., Mugerwa R.

African Health Sciences. 11 (SPEC. ISSUE) (pp S105-S111), 2011. Date of Publication: 2011.

AN: 2011559530

Introduction: Early identification of Tuberculosis (TB) treatment failure using cost effective means is urgently needed in developing nations. The study set out to describe affordable predictors of TB treatment failure in an African setting. Objective: To determine the predictors of treatment failure among patients with sputum smear positive pulmonary TB at Mulago hospital. The study was carried out in the TB clinic of Mulago hospital Kampala, Uganda. This was an unmatched case control study where fifty patients with a diagnosis of TB treatment failure (cases) and 100 patients declared cured after completing anti TB treatment (controls) were recruited into the study. Cases were compared with controls to determine predictors of treatment failure. Results: Significant predictors of treatment failure in this study included a positive sputum smear at 2 months of TB treatment (OR 20.63, 95%CI 5.42-78.41) and poor adherence to anti TB treatment (OR 14.59, 95%CI 3.04-70.15). Conclusion: This study identified a treatment related and a simple laboratory predictor of TB treatment failure in Mulago hospital which may be used in resource limited settings for early recognition of those at risk and early intervention.

Institution

(Namukwaya, Nakwagala, Mayanja-Kizza, Mugerwa) Department of Medicine, College of Health Sciences, Makerere University Kampala, Uganda (Mulekya) School of Public Health, Makerere University Kampala, Uganda

Publisher

Makerere University, Medical School (P.O. Box 7072, Kampala, Uganda)

Emtree Heading

adult; article; bacterial load; case control study; clinical article; controlled study; diabetes mellitus; disease severity; *drug treatment failure; early diagnosis; early intervention; female; fever; glucose blood level; health care access; human; Human immunodeficiency virus infected patient; laboratory test; lung cavitation; *lung tuberculosis/di [Diagnosis]; *lung tuberculosis/dt [Drug Therapy]; male; patient compliance; prediction; risk factor; sputum smear; treatment refusal; Uganda; ethambutol/cb [Drug Combination]; ethambutol/dt [Drug Therapy]; isoniazid/cb [Drug Combination]; isoniazid/dt [Drug Therapy]; pyrazinamide/cb [Drug Combination]; pyrazinamide/dt [Drug Therapy]; rifampicin/cb [Drug Combination]; rifampicin/dt [Drug Therapy].

Link to the Ovid Full Text or citation:

Click here for full text options

197.

Drug repositioning: Re-investigating existing drugs for new therapeutic indications.

Padhy B.M., Gupta Y.K.

Journal of Postgraduate Medicine. 57 (2) (pp 153-160), 2011. Date of Publication: April-June 2011.

AN: 2011479478

Drug discovery and development is an expensive, time-consuming, and risky enterprise. In order to accelerate the drug development process with reduced risk of failure and relatively lower costs, pharmaceutical companies have adopted drug repositioning as an alternative. This strategy involves exploration of drugs that have already been approved for treatment of other diseases and/or whose targets have already been discovered. Various techniques including data mining, bioinformatics, and usage of novel screening platforms have been used for identification and screening of potential repositioning candidates. However, challenges in clinical trials and intellectual property issues may be encountered during the repositioning process. Nevertheless, such initiatives not only add value to the portfolio of pharmaceutical companies but also provide an opportunity for academia and government laboratories to develop new and innovative uses of existing drugs for infectious and neglected diseases, especially in emerging countries like India.

Institution

(Padhy) Department of Pharmacology, Hamdard Institute of Medical Sciences and Research, New Delhi, India (Gupta) Department of Pharmacology, All India Institute of Medical Sciences, New Delhi, India

Publisher

Medknow Publications and Media Pvt. Ltd (B9, Kanara Business Centre, off Link Road, Ghatkopar (E), Mumbai 400 075, India)

Emtree Heading

acne/dt [Drug Therapy]; acquired immune deficiency syndrome/dt [Drug Therapy]; acute heart infarction; African trypanosomiasis/dt [Drug Therapy]; alcoholism/dt [Drug Therapy]; amyotrophic lateral sclerosis/dt [Drug Therapy]; angina pectoris/dt [Drug Therapy]; angiogenesis; anthelmintic activity; attention deficit disorder/dt [Drug Therapy]; autoimmune disease; bacterial infection/dt [Drug Therapy]; bioinformatics; breast cancer/dt [Drug Therapy]; cachexia; Caenorhabditis elegans; cell assay; clinical trial (topic); computer program; data base; data mining; depression/dt [Drug Therapy]; developed country; developing country; diabetes mellitus/dt [Drug Therapy]; Drosophila melanogaster; drug control; drug cost; drug efficacy; drug identification; drug manufacture; drug marketing; drug mechanism; drug quality; *drug repositioning; drug safety; drug screening; drug treatment failure; epilepsy/dt [Drug Therapy]; erectile dysfunction/dt [Drug Therapy]; erythema nodosum leprosum/dt [Drug Therapy]; Europe; ex vivo study; eye disease; eyelash; fibromyalgia/dt [Drug Therapy]; food and drug administration; genomics; glaucoma/dt [Drug Therapy]; gout/dt [Drug Therapy]; government; hematopoietic stem cell; high throughput screening; hirsutism/dt [Drug Therapy]; human; Human immunodeficiency virus; Human immunodeficiency virus infection/dt [Drug Therapy]; hypertension/dt [Drug Therapy]; in vivo study; India; infection; inflammation/dt [Drug Therapy]; influenza/dt [Drug Therapy]; leishmaniasis/dt [Drug Therapy]; leprosy/dt [Drug Therapy]; library; lifestyle; lymphatic filariasis; malaria/dt [Drug Therapy]; male type alopecia/dt [Drug Therapy]; melanoma/dt [Drug Therapy]; migraine/dt [Drug Therapy]; migraine/pc [Prevention]; morbidity; morning sickness/dt [Drug Therapy]; mortality; multiple myeloma/dt [Drug Therapy]; mycosis/dt [Drug Therapy]; neoplasm/dt [Drug Therapy]; neurologic disease; neuropathic pain/dt [Drug Therapy]; nonhuman; obesity; osteoarthritis; osteoporosis/dt [Drug Therapy]; pain/dt [Drug Therapy]; Parkinson disease/dt [Drug Therapy]; patent; pediatrics; pericarditis/dt [Drug Therapy]; pharmacogenetics; phase 1 clinical trial (topic); phenotype; Plasmodium falciparum; Plasmodium vivax; pregnant woman; promyelocytic leukemia/dt [Drug Therapy]; proteomics; psoriasis/dt [Drug Therapy]; recurrent disease/dt [Drug Therapy]; restless legs syndrome/dt [Drug Therapy]; review; rheumatoid arthritis/dt [Drug Therapy]; risk; smoking cessation; stress incontinence/dt [Drug Therapy]; *treatment indication; tropical disease; tuberculosis/dt [Drug Therapy]; United States; visceral leishmaniasis/dt [Drug Therapy]; yaws; acetylsalicylic acid/dt [Drug Therapy]; amantadine/dt [Drug Therapy]; amfebutamone/dt [Drug Therapy]; amphotericin/dt [Drug Therapy]; atomoxetine/dt [Drug Therapy]; auranofin/dt [Drug Therapy]; bimatoprost/dt [Drug Therapy]; bromocriptine/dt [Drug Therapy]; ceftriaxone/dt [Drug Therapy]; clofazimine/dt [Drug Therapy]; colchicine/dt [Drug Therapy]; dapsone/dt [Drug Therapy]; disulfiram/dt [Drug Therapy]; duloxetine/dt [Drug Therapy]; eflornithine/dt [Drug Therapy]; gabapentin/dt [Drug Therapy]; methotrexate/dt [Drug Therapy]; milnacipran/dt [Drug Therapy]; miltefosine/dt [Drug Therapy]; minocycline/dt [Drug Therapy]; minoxidil/dt [Drug Therapy]; plerixafor/dt [Drug Therapy]; propranolol/dt [Drug Therapy]; raloxifene/ct [Clinical Trial]; raloxifene/dt [Drug Therapy]; retinoic acid/dt [Drug Therapy]; ropinirole/dt [Drug Therapy]; sildenafil/ct [Clinical Trial]; sildenafil/dt [Drug Therapy]; thalidomide/dt [Drug Therapy]; unindexed drug; zidovudine/dt [Drug Therapy].

Drug Trade Names and Manufacturers

aspirin

Link to the Ovid Full Text or citation:

Click here for full text options

198.

Series: "Update on tuberculosis" - Tuberculosis and noncommunicable diseases: Neglected links and missed opportunities.

Creswell J., Raviglione M., Ottmani S., Migliori G.B., Uplekar M., Blanc L., Sotgiu G., Lonnroth K.

European Respiratory Journal. 37 (5) (pp 1269-1282), 2011. Date of Publication: 01 May 2011.

AN: 2011248701

Globally, the incidence of tuberculosis (TB) is declining very slowly, and the noncommunicable disease (NCD) burden for many countries is steadily increasing. Several NCDs, such as diabetes mellitus, alcohol use disorders and smoking-related conditions, are responsible for a significant proportion of TB cases globally, and in the European region, represent a larger attributable fraction for TB disease than HIV. Concrete steps are needed to address NCDs and their risk factors. We reviewed published studies involving TB and NCDs, and present a review and discussion of how they are linked, the implications for case detection and management, and how prevention efforts may be strengthened by integration of services. These NCDs put patients at increased risk for developing TB and at risk for poor treatment outcomes. However, they also present an opportunity to provide better care through increased casedetection activities, improved clinical management and better access to care for both TB and NCDs. Hastening the global decline in TB incidence may be assisted by strengthening these types of activities.

Institution

(Creswell, Raviglione, Ottmani, Uplekar, Blanc, Lonnroth) Stop TB Dept., World Health Organization, 20 Avenue Appia, Geneva 1211, Switzerland (Migliori) WHO Collaborating Centre for TB and Lung Disease, Fondazione S. Maugeri, Care and Research Institute, Tradate, Italy

(Sotgiu) Hygiene and Preventive Medicine Institute, University of Sassari, Sassari, Italy

Publisher

European Respiratory Society (4 Ave Sainte-Luce, Lausanne CH-1003, Switzerland)

Emtree Heading

alcohol consumption; *alcoholism; awareness; cellular immunity; chronic obstructive lung disease; cost effectiveness analysis; *diabetes mellitus/dt [Drug Therapy]; disease association; disease transmission; health care cost; health program; host resistance; human; incidence; liver toxicity/si [Side Effect]; *lung tuberculosis/dm [Disease Management]; *lung tuberculosis/dt [Drug Therapy]; malnutrition; mental disease; nonhuman; peripheral neuropathy/si [Side Effect]; priority journal; review; risk assessment; risk factor; silicosis; *smoking; social status; substance abuse; systematic review; tuberculosis control; glipizide/it [Drug Interaction]; glipizide/dt [Drug Therapy]; isoniazid/ae [Adverse Drug Reaction]; isoniazid/dt [Drug Therapy]; rifampicin/ae [Adverse Drug Reaction]; rifampicin/it [Drug Interaction]; rifampicin/dt [Drug Therapy].

Link to the Ovid Full Text or citation:

Click here for full text options

199.

The impact of diabetes on tuberculosis treatment outcomes: A systematic review.

Baker M.A., Harries A.D., Jeon C.Y., Hart J.E., Kapur A., Lonnroth K., Ottmani S.-E., Goonesekera S.D., Murray M.B.

BMC Medicine. 9 (no pagination), 2011. Article Number: 81. Date of Publication: 01 Jul 2011.

AN: 2011445871

Background: Multiple studies of tuberculosis treatment have indicated that patients with diabetes mellitus may experience poor outcomes.We performed a systematic review and meta-analysis to quantitatively summarize evidence for the impact of diabetes on tuberculosis outcomes.Methods: We searched PubMed, EMBASE and the World Health Organization Regional Indexes from 1 January 1980 to 31 December 2010 and references of relevant articles for reports of observational studies that included people with diabetes treated for tuberculosis. We reviewed the full text of 742 papers and included 33 studies of which 9 reported culture conversion at two to three months, 12 reported the combined outcome of failure and death, 23 reported death, 4 reported death adjusted for age and other potential confounding factors, 5 reported relapse, and 4 reported drug resistant recurrent tuberculosis.Results: Diabetes is associated with an increased risk of failure and death during tuberculosis treatment. Patients with diabetes have a risk ratio (RR) for the combined outcome of failure and death of 1.69 (95% CI, 1.36 to 2.12). The RR of death during tuberculosis treatment among the 23 unadjusted studies is 1.89 (95% CI, 1.52 to 2.36), and this increased to an effect estimate of 4.95 (95% CI, 2.69 to 9.10) among the 4 studies that adjusted for age and other potential confounding factors. Diabetes is also associated with an increased risk of relapse (RR, 3.89; 95% CI, 2.43 to 6.23). We did not find evidence for an increased risk of tuberculosis recurrence with drug resistant strains among people with diabetes. The studies assessing sputum culture conversion after two to three months of tuberculosis therapy were heterogeneous with relative risks that ranged from 0.79 to 3.25.Conclusions: Diabetes increases the risk of failure and death combined, death, and relapse among patients with tuberculosis. This study highlights a need for increased attention to treatment of tuberculosis in people with diabetes, which may include testing for suspected diabetes, improved glucose control, and increased clinical and therapeutic monitoring. © 2011 Baker et al; licensee BioMed Central Ltd.

Institution

(Baker) Division of Infectious Diseases, Massachusetts General Hospital, Boston, MA, United States (Baker, Goonesekera, Murray) Department of Epidemiology, Harvard School of Public Health, Boston, MA, United States

(Harries) International Union Against Tuberculosis and Lung Disease, Paris, France

(Harries) London School of Hygiene and Tropical Medicine, Keppel Street, London, United Kingdom

(Jeon) Center for Infectious Disease Epidemiologic Research, Columbia University, New York, NY, United States

(Hart) The Warren Alpert Medical School of Brown University, Providence, RI, United States

(Kapur) World Diabetes Foundation, Lyngby, Denmark

(Lonnroth, Ottmani) Stop-TB Department, World Health Organization, Geneva, Switzerland

(Murray) Division of Global Health Equity, Brigham and Women's Hospital, Boston, MA, United States

(Jeon) Department of Epidemiology, Harvard School of Public Health, Boston, MA, United States

Publisher

BioMed Central Ltd. (Floor 6, 236 Gray's Inn Road, London WC1X 8HB, United Kingdom)

Emtree Heading

article; death; *diabetes mellitus; drug resistant tuberculosis; evidence based medicine; genetic heterogeneity; human; meta analysis; recurrence risk; relapse; risk factor; sputum culture; systematic review; treatment failure; treatment outcome; *tuberculosis/dr [Drug Resistance]; *tuberculosis/dt [Drug Therapy]; ethambutol/dt [Drug Therapy]; pyrazinamide/dt [Drug Therapy]; rifampicin/dt [Drug Therapy].

Link to the Ovid Full Text or citation:

Click here for full text options

200.

Autoimmunity and inflammation: Murine models and translational studies.

Hall S.W., Cooke A.

Mammalian Genome. 22 (7-8) (pp 377-389), 2011. Date of Publication: August 2011.

AN: 2011439859

Autoimmune and inflammatory diseases, including type 1 diabetes, multiple sclerosis, inflammatory bowel disease, and rheumatoid arthritis, constitute an important and growing public health burden. However, in many cases our understanding of disease biology is limited and available therapies vary greatly in their efficacy and safety. Animal models of autoimmune and inflammatory diseases have provided valuable tools to researchers investigating their aetiology, pathology, and novel therapeutic strategies. Although such models vary in the degree to which they reflect human autoimmune and inflammatory diseases and caution is required in the extrapolation of animal data to the clinical setting, therapeutic approaches first evaluated in established animal models, including collagen-induced arthritis, experimental autoimmune encephalomyelitis, and the nonobese diabetic mouse, have successfully progressed to clinical investigation and practice. Similarly, these models have proven useful in providing support for basic hypotheses regarding the underlying causes and pathology of autoimmune and inflammatory diseases. Here we review selected murine models of autoimmunity and inflammation and efforts to translate findings from these models into both basic insights into disease biology and novel therapeutic strategies. © 2011 Springer Science+Business Media, LLC.

Institution

(Hall, Cooke) Department of Pathology, University of Cambridge, Tennis Court Road, Cambridge CB2 1QP, United Kingdom

Publisher

Springer New York (233 Springer Street, New York NY 10013-1578, United States)

Emtree Heading

animal model; arthritis/dt [Drug Therapy]; arthritis/th [Therapy]; arthropathy/dt [Drug Therapy]; *autoimmune disease/et [Etiology]; BioBreeding rat; cell migration; chronic arthritis/th [Therapy]; colitis/dt [Drug Therapy]; collagen disease/dt [Drug Therapy]; collagen disease/th [Therapy]; Crohn disease/et [Etiology]; diabetes mellitus/dt [Drug Therapy]; diabetes mellitus/et [Etiology]; diabetes mellitus/pc [Prevention]; differential diagnosis; disease association; disease course; disease severity; dose response; drug protein binding; drug safety; drug targeting; encephalomyelitis/di [Diagnosis]; encephalomyelitis/dt [Drug Therapy]; encephalomyelitis/et [Etiology]; encephalomyelitis/pc [Prevention]; encephalomyelitis/th [Therapy]; enteritis/et [Etiology]; *experimental model; experimental mouse; gene dosage; gene mutation; gold standard; Hashimoto disease/et [Etiology]; heredity; human; immunomodulation; immunosuppressive treatment; in vitro study; in vivo study; infection risk; *inflammatory disease/dt [Drug Therapy]; *inflammatory disease/et [Etiology]; innate immunity; insulin dependent diabetes mellitus/dt [Drug Therapy]; insulin dependent diabetes mellitus/et [Etiology]; insulin dependent diabetes mellitus/th [Therapy]; insulitis/dt [Drug Therapy]; low drug dose; monotherapy; multiple sclerosis/di [Diagnosis]; multiple sclerosis/dt [Drug Therapy]; multiple sclerosis/et [Etiology]; nonhuman; pathology; postprimary tuberculosis/si [Side Effect]; progressive multifocal leukoencephalopathy/di [Diagnosis]; progressive multifocal leukoencephalopathy/et [Etiology]; progressive multifocal leukoencephalopathy/si [Side Effect]; protein function; protein processing; protein targeting; psoriasis/dt [Drug Therapy]; relapse/dt [Drug Therapy]; review; rheumatoid arthritis/dt [Drug Therapy]; rheumatoid arthritis/et [Etiology]; rheumatoid arthritis/th [Therapy]; risk reduction; sex ratio; side effect/si [Side Effect]; symbiont; *translational research; treatment duration; treatment outcome; ulcerative colitis/et [Etiology]; vaccination; virus reactivation; bacterial protein/pd [Pharmacology]; beta1a interferon/cb [Drug Combination]; beta1a interferon/dt [Drug Therapy]; CD3 antibody/dt [Drug Therapy]; CD3 antibody/pd [Pharmacology]; collagen type 2/dt [Drug Therapy]; collagen type 2/dl [Intradermal Drug Administration]; collagen type 2/ip [Intraperitoneal Drug Administration]; collagen type 2/pr [Pharmaceutics]; collagen type 2/pd [Pharmacology]; etanercept/ae [Adverse Drug Reaction]; etanercept/dt [Drug Therapy]; excretory secretoty glycoprotein/dt [Drug Therapy]; excretory secretoty glycoprotein/pd [Pharmacology]; excretory secretoty glycoprotein/sc [Subcutaneous Drug Administration]; Freund adjuvant; glycan lacto n fucopentaose III/dt [Drug Therapy]; glycan lacto n fucopentaose III/pd [Pharmacology]; glycan lacto n fucopentaose III/sc [Subcutaneous Drug Administration]; infliximab/ct [Clinical Trial]; infliximab/cb [Drug Combination]; infliximab/cm [Drug Comparison]; infliximab/pd [Pharmacology]; insulin/dt [Drug Therapy]; methotrexate/ct [Clinical Trial]; methotrexate/cb [Drug Combination]; methotrexate/dt [Drug Therapy]; methotrexate/pd [Pharmacology]; myelin basic protein; myelin oligodendrocyte glycoprotein; n (3 oxododecanoyl)homoserine lactone/dt [Drug Therapy]; n (3 oxododecanoyl)homoserine lactone/ip [Intraperitoneal Drug Administration]; n (3 oxododecanoyl)homoserine lactone/pd [Pharmacology]; natalizumab/ae [Adverse Drug Reaction]; natalizumab/ct [Clinical Trial]; natalizumab/cb [Drug Combination]; natalizumab/dt [Drug Therapy]; natalizumab/pd [Pharmacology]; otelixizumab/ct [Clinical Trial]; otelixizumab/pd [Pharmacology]; placebo; polysaccharide/pd [Pharmacology]; polysaccharide a/dt [Drug Therapy]; polysaccharide a/pd [Pharmacology]; protein antibody/pd [Pharmacology]; Schistosoma vaccine/dt [Drug Therapy]; Schistosoma vaccine/ip [Intraperitoneal Drug Administration]; teplizumab/ct [Clinical Trial]; teplizumab/pd [Pharmacology]; tumor necrosis factor alpha/ec [Endogenous Compound]; tumor necrosis factor alpha antibody/dt [Drug Therapy]; tumor necrosis factor alpha antibody/pd [Pharmacology]; tumor necrosis factor alpha inhibitor/ae [Adverse Drug Reaction]; tumor necrosis factor alpha inhibitor/cm [Drug Comparison]; tumor necrosis factor alpha inhibitor/dt [Drug Therapy]; unclassified drug; very late activation antigen 4/ec [Endogenous Compound]; very late activation antigen 4 antibody/dt [Drug Therapy]; very late activation antigen 4 antibody/pd [Pharmacology].

Link to the Ovid Full Text or citation:

Click here for full text options
