# Supplementary material for: Effect of glycemic control and type of diabetes treatment on TB treatment outcomes among people with TB-diabetes: A systematic review (updated August 2024)
Source: PLoS One. 2025 Jul 18;20(7):e0328619. doi: 10.1371/journal.pone.0328619 (PMC12273911; doi:10.1371/journal.pone.0328619)
Supplement: S1 Appendix — (ZIP) [file pone.0328619.s004.zip › S1 appendix_old/EMBASE/298-3_with RISK search reuslts no 15.doc]

Database: Embase <1996 to 2016 September 16>

Search Strategy:

--------------------------------------------------------------------------------

1	tuberculosis.mp. or exp tuberculosis/ (151164)

2	exp treatment outcome/ or treatment outcome*.mp. (1189741)

3	diabetes mellitus.mp. or exp diabetes mellitus/ (627157)

4	diabetes mellitus/ (362441)

5	DM.mp. (48079)

6	diabetes.mp. (663193)

7	mellitus.mp. (588341)

8	3 or 4 or 5 or 6 or 7 (715025)

9	1 and 2 and 8 (724)

10	cohort studies.mp. or cohort analysis/ (263408)

11	exp risk factor/ or cohort.mp. or exp risk/ (2148599)

12	10 or 11 (2148599)

13	9 and 10 (81)

14	9 and 12 (304)

15	limit 14 to human (298)

16	limit 13 to human (81)

17	trial*.mp. or exp controlled clinical trial/ or exp "clinical trial (topic)"/ or exp "randomized controlled trial (topic)"/ or exp "controlled clinical trial (topic)"/ (1555047)

18	random.ab. (191735)

19	randomized controlled trial.pt. (0)

20	controlled clinical trial.pt. (0)

21	randomized.ab. (454621)

22	placebo.ab. (188623)

23	clinical trials as topic.sh. (15)

24	trial.ti. (176210)

25	17 or 18 or 19 or 20 or 21 or 22 or 23 or 24 (1831290)

26	9 and 25 (128)

27	limit 26 to human (128)

***************************

201.

Peritoneal dialysis as the first-line renal replacement therapy in patients with autosomal dominant polycystic kidney disease.

Li L., Szeto C.-C., Kwan B.C.-H., Chow K.-M., Leung C.-B., Kam-Tao Li P.

American Journal of Kidney Diseases. 57 (6) (pp 903-907), 2011. Date of Publication: June 2011.

AN: 2011285196

Background: Autosomal dominant polycystic kidney disease (ADPKD) is the most common hereditary cause of kidney failure. Peritoneal dialysis (PD) often is avoided because of concerns about hernias and peritonitis. Study Design: Retrospective matched-cohort study. Setting & Participants: 42 consecutive patients with ADPKD and 84 nondiabetic patients matched by time of PD therapy initiation. Predictors: ADPKD and comorbid conditions. Outcomes: Patient survival, technique survival, peritonitis-free survival; peritonitis rate; abdominal hernias. Measurements: Dialysis adequacy and nutritional indexes; rate of decrease in residual renal function; bacteriologic cause of peritonitis. Results: 5-year actuarial survival of the ADPKD and control groups was 71.0% and 69.7% (P = 0.4), whereas technique survival was 51.6% and 37.3%, respectively (P = 0.2). There was no difference in overall rates of peritonitis between the ADPKD and control groups (0.51 vs 0.53 episodes/patient-year; P = 0.3), and the incidence of Gram-negative peritonitis also was similar (0.16 vs 0.14 episodes/patient year; P = 0.5). Abdominal wall hernia was significantly more common in the ADPKD than control group (14 vs 6 cases; P < 0.001), but all patients were able to resume PD therapy after surgical repair. Limitations: Retrospective study with limited sample size. Conclusion: PD is a feasible treatment option for most patients with ADPKD with end-stage renal disease. Although patients with ADPKD have a higher risk of abdominal wall hernia, their overall survival rate and risk of peritonitis are similar to those of other nondiabetic PD patients. © 2011 National Kidney Foundation, Inc.

Institution

(Li) Department of Nephrology, Shanghai Changzheng Hospital, Second Military Medical University, Shanghai, China (Szeto, Kwan, Chow, Leung, Kam-Tao Li) Department of Medicine and Therapeutics, Prince of Wales Hospital, Chinese University of Hong Kong, Shatin, Hong Kong

Publisher

W.B. Saunders (Independence Square West, Philadelphia PA 19106-3399, United States)

Emtree Heading

abdominal wall hernia; Acinetobacter; adult; aged; article; *autosomal dominant polycystic kidney disease/cn [Congenital Disorder]; *autosomal dominant polycystic kidney disease/th [Therapy]; blood stain; cerebrovascular disease; clinical article; cohort analysis; comorbidity; controlled study; diabetes mellitus; disease free survival; Enterococcus; Escherichia coli; female; follow up; human; hypertension; incidence; ischemic heart disease; *kidney polycystic disease/cn [Congenital Disorder]; *kidney polycystic disease/th [Therapy]; Klebsiella; male; Mycobacterium tuberculosis; nonhuman; nutritional status; overall survival; *peritoneal dialysis; peritonitis; Pseudomonas; *renal replacement therapy; retrospective study; sample size; Staphylococcus aureus; Streptococcus; therapy effect; treatment outcome.

Link to the Ovid Full Text or citation:

Click here for full text options

202.

Invasive aspergillosis in developing countries.

Chakrabarti A., Chatterjee S.S., Das A., Shivaprakash M.R.

Medical Mycology. 49 (SUPPL. 1) (pp S35-S47), 2011. Date of Publication: April 2011.

AN: 2011149743

To review invasive aspergillosis (IA) in developing countries, we included those countries, which are mentioned in the document of the International Monetary Fund (IMF), called the Emerging and Developing Economies List, 2009. A PubMed/Medline literature search was performed for studies concerning IA reported during 1970 through March 2010 from these countries. IA is an important cause of morbidity and mortality of hospitalized patients of developing countries, though the exact frequency of the disease is not known due to inadequate reporting and facilities to diagnose. Only a handful of centers from India, China, Thailand, Pakistan, Bangladesh, Sri Lanka, Malaysia, Iran, Iraq, Saudi Arabia, Egypt, Sudan, South Africa, Turkey, Hungary, Brazil, Chile, Colombia, and Argentina had reported case series of IA. As sub-optimum hospital care practice, hospital renovation work in the vicinity of immunocompromised patients, overuse or misuse of steroids and broad-spectrum antibiotics, use of contaminated infusion sets/fluid, and increase in intravenous drug abusers have been reported from those countries, it is expected to find a high rate of IA among patients with high risk, though hard data is missing in most situations. Besides classical risk factors for IA, liver failure, chronic obstructive pulmonary disease, diabetes, and tuberculosis are the newly recognized underlying diseases associated with IA. In Asia, Africa and Middle East sino-orbital or cerebral aspergillosis, and Aspergillus endophthalmitis are emerging diseases and Aspergillus flavus is the predominant species isolated from these infections. The high frequency of A. flavus isolation from these patients may be due to higher prevalence of the fungus in the environment. Cerebral aspergillosis cases are largely due to an extension of the lesion from invasive Aspergillus sinusitis. The majority of the centers rely on conventional techniques including direct microscopy, histopathology, and culture to diagnose IA. Galactomannan, beta-D glucan test, and DNA detection in IA are available only in a few centers. Mortality of the patients with IA is very high due to delays in diagnosis and therapy. Antifungal use is largely restricted to amphotericin B deoxycholate and itraconazole, though other anti-Aspergillus antifungal agents are available in those countries. Clinicians are aware of good outcome after use of voriconazole/liposomal amphotericin B/caspofungin, but they are forced to use amphotericin B deoxycholate or itraconazole in public-sector hospitals due to economic reasons. © 2011 ISHAM.

Institution

(Chakrabarti, Chatterjee, Shivaprakash) Department of Medical Microbiology, PGIMER, Chandigarh 160012, India (Das) Department of Histopathology, Postgraduate Institute of Medical Education and Research, Chandigarh, India

Publisher

Informa Healthcare (69-77 Paul Street, London EC2A 4LQ, United Kingdom)

Emtree Heading

Africa; Argentina; Asia; aspergillosis; aspergillosis endocarditis; Aspergillus endophthalmitis; Aspergillus flavus; Bangladesh; bone marrow transplantation; Brazil; central nervous system aspergillosis/su [Surgery]; Chile; China; chronic obstructive lung disease; Colombia; conference paper; delayed diagnosis; dermatomycosis; developing country; diabetes mellitus; disease association; drug abuse; Egypt; fungal endocarditis; fungal endophthalmitis; hospital care; human; Hungary; immunocompromised patient; India; infection; *invasive aspergillosis/dt [Drug Therapy]; Iran; Iraq; leukemia; liver failure; lung aspergillosis; Malaysia; medical practice; Medline; Middle East; morbidity; mortality; nonhuman; Pakistan; risk assessment; Saudi Arabia; South Africa; Sri Lanka; Sudan; systematic review; Thailand; transplantation; treatment outcome; tuberculosis; Turkey (republic); amphotericin B/cm [Drug Comparison]; amphotericin B/dt [Drug Therapy]; amphotericin B deoxycholate/dt [Drug Therapy]; amphotericin B lipid complex/dt [Drug Therapy]; antibiotic agent; caspofungin/dt [Drug Therapy]; fungal DNA/ec [Endogenous Compound]; itraconazole/dt [Drug Therapy]; steroid; voriconazole/cm [Drug Comparison]; voriconazole/dt [Drug Therapy].

Link to the Ovid Full Text or citation:

Click here for full text options

203.

Adverse events associated with treatment of latent tuberculosis in the general population.

Smith B.M., Schwartzman K., Bartlett G., Menzies D.

CMAJ. 183 (3) (pp E173-E179), 2011. Date of Publication: 22 Feb 2011.

AN: 2011112612

Background: Guidelines recommend treatment of latent tuberculosis in patients at increased risk for active tuberculosis. Studies investigating the association of therapy with serious adverse events have not included the entire treated population nor accounted for comorbidities or occurrence of similar events in the untreated general population. Our objective was to estimate the risk of adverse events requiring hospital admission that were associated with therapy for latent tuberculosis infection in the general population. Methods: Using administrative health data from the province of Quebec, we created a historical cohort of all residents dispensed therapy for latent tuberculosis between 1998 and 2003. Each patient was matched on age, sex and postal region with two untreated residents. The observation period was 18 months (from 6 months before to 12 months after initiation of therapy). The primary outcome was hospital admission for therapy-associated adverse events. Results: During the period of observation, therapy for latent tuberculosis was dispensed to 9145 residents, of whom 95% started isoniazid and 5% started rifampin. Pretreatment comorbid illness was significantly more common among patients receiving such therapy compared with the matched untreated cohort. Of all patients dispensed therapy, 45 (0.5%) were admitted to hospital for a hepatic event compared with 15 (0.1%) of the untreated patients. For people over age 65 years, the odds of hospital admission for a hepatic event among patients treated for latent tuberculosis infection was significantly greater than among matched untreated people after adjustment for comorbidities (odds ratio [OR] 6.4, 95% CI 2.2-18.3). Excluding patients with comorbid illness, there were two excess admissions to hospital for hepatic events per 100 patients initiating therapy compared with the rate among untreated people over 65 years (95% CI 0.1-3.87). Interpretation: The risk of adverse events requiring hospital admission increased significantly among patients over 65 years receiving treatment for latent tuberculosis infection. The decision to treat latent tuberculosis infection in elderly patients should be made after careful consideration of risks and benefits. © 2011 Canadian Medical Association or its licensors.

Institution

(Smith, Schwartzman, Menzies) Respiratory Division, Montreal Chest Institute, McGill University, Montreal, QC, Canada (Schwartzman, Menzies) Respiratory Epidemiology and Clinical Research Unit, Montreal Chest Institute, McGill University, Montreal, QC, Canada

(Bartlett) Department of Family Medicine, McGill University Health Centre, McGill University, Montreal, QC, Canada

Publisher

Canadian Medical Association (1867 Alta Vista Drive, Ottawa KIG5W8, Canada)

Emtree Heading

adult; aged; article; chronic lung disease/si [Side Effect]; comorbidity; controlled study; dermatitis/si [Side Effect]; diabetes mellitus/si [Side Effect]; drug intoxication/si [Side Effect]; dyspepsia/si [Side Effect]; female; gastrointestinal disease/si [Side Effect]; hospital admission; human; kidney disease/si [Side Effect]; *latent tuberculosis; liver disease/si [Side Effect]; major clinical study; male; peptic ulcer/si [Side Effect]; thrombocytopenia/si [Side Effect]; toxic hepatitis/si [Side Effect]; treatment outcome; vomiting/si [Side Effect]; *isoniazid/ae [Adverse Drug Reaction]; *rifampicin/ae [Adverse Drug Reaction].

Link to the Ovid Full Text or citation:

Click here for full text options

204.

Outcomes of overseas kidney transplantation in chronic haemodialysis patients in Taiwan.

Hsu C.-C., Lee C.-H., Hwang S.-J., Huang S.-W., Yang W.-C., Chang Y.-K., Tsai D.F.-C., Kuo K.N.

Nephrology. 16 (3) (pp 341-348), 2011. Date of Publication: March 2011.

AN: 2011112328

Aim: Overseas kidney transplantation has often been reported to have unsatisfactory outcomes. This study aims to compare post-transplantation outcomes between overseas and domestic kidney transplant (KT) recipients in Taiwan. Methods: The Taiwanese National Health Insurance Research Database was used to identify 310 domestic and 643 overseas KT recipients, who survived for longer than 1 month after the transplantation, in a cohort of 45 453 chronic haemodialysis patients in 1997-2002. Cox proportional hazards models were used to assess risks of mortality and graft failure. Results: The 1, 3 and 5 year survival rates for domestic KT recipients were 96.5%, 93.3% and 91.6%, respectively, while those for overseas KT recipients were 94.9%, 87.9% and 77.1%, respectively (P = 0.015). For the overseas group, those who received a KT before 2001 had significantly higher hazard ratios of mortality and graft failure (2.85 and 1.71, respectively). However, for those receiving a KT in 2001-2002, no significant outcome difference could be found between overseas and domestic recipients. Conclusion: The risk disparity between overseas and domestic KT recipients is mainly attributable to when the transplantation was performed. In attempting to dissuade potential recipients from organ trafficking, merely emphasizing the previously acknowledged poor outcomes no longer suffices as a valid reason. © 2011 The Authors. Nephrology © 2011 Asian Pacific Society of Nephrology.

Institution

(Hsu, Chang, Kuo) Institute of Population Health Sciences, National Health Research Institutes, Zhunan, Taiwan (Republic of China) (Hsu) Department of Health Services Administration, China Medical University and Hospital, Taichung, Taiwan (Republic of China)

(Lee) Bureau of National Health Insurance, Taipei, Taiwan (Republic of China)

(Hwang) Department of Medicine, Kaohsiung Medical University Hospital, College of Medicine, Kaohsiung, Taiwan (Republic of China)

(Huang) Division of Urology, Department of Surgery, Saint Martin de Porres Hospital, Chiayi, Taiwan (Republic of China)

(Yang) Department of Medicine, Taipei Veterans General Hospital, National Yang-Ming University, Taipei, Taiwan (Republic of China)

(Tsai) Department of Social Medicine, National Taiwan University, National Taiwan University Hospital, Taipei, Taiwan (Republic of China)

Publisher

Blackwell Publishing (550 Swanston Street, Carlton South VIC 3053, Australia)

Emtree Heading

adult; article; brain abscess/co [Complication]; cerebrovascular disease/co [Complication]; comorbidity; controlled study; cytomegalovirus infection/co [Complication]; data base; diabetes mellitus/co [Complication]; endocarditis/co [Complication]; female; graft failure/co [Complication]; graft recipient; graft rejection/co [Complication]; graft rejection/dt [Drug Therapy]; graft rejection/pc [Prevention]; graft survival; hematoma/co [Complication]; *hemodialysis patient; hepatitis B/co [Complication]; hepatitis C/co [Complication]; herpes simplex/co [Complication]; herpes zoster/co [Complication]; human; hydronephrosis/co [Complication]; hypertension/co [Complication]; incidence; ischemic heart disease/co [Complication]; kidney cancer/co [Complication]; kidney infection/co [Complication]; *kidney transplantation; kidney vein thrombosis/co [Complication]; liver abscess/co [Complication]; lymphocele/co [Complication]; major clinical study; male; medical tourism; meningitis/co [Complication]; mortality; outcome assessment; *overseas kidney transplantation; postoperative complication/co [Complication]; postoperative infection/co [Complication]; priority journal; risk assessment; septicemia/co [Complication]; survival rate; survivor; Taiwan; tuberculosis/co [Complication]; urinary tract obstruction/co [Complication]; urinoma/co [Complication]; lymphocyte antibody/dt [Drug Therapy]; methylprednisolone/dt [Drug Therapy]; thymocyte antibody/dt [Drug Therapy].

Link to the Ovid Full Text or citation:

Click here for full text options

205.

Tuberculous lymphadenitis at Penang General Hospital, Malaysia.

Khan A.H., Sulaiman S.A.S., Muttalif A.R., Hassali M.A., Khan T.M.

Medical Principles and Practice. 20 (1) (pp 80-84), 2011. Date of Publication: December 2010.

AN: 2010701334

Objective: To evaluate the incidence, treatment and clinical outcomes of tuberculous (TB) lymphadenitis at Penang General Hospital, Malaysia. Materials and Methods: Penang General Hospital is the referral center for all tuberculosis patients in the state of Penang. Patient records were reviewed to identify patients with confirmed diagnosis of TB lymphadenitis between January 2006 and December 2008. Data were analyzed using SPSS version 15. Results: Of 1,548 tuberculosis cases, 109 (7.0%) patients had TB lymphadenitis. The mean age was 36.4 +/- 12.87 years and of the 109 patients with TB lymphadenitis, 35 (33.0%), 37 (34.0%) and 36 (33.0%) were observed for 2006, 2007 and 2008, respectively. Ethnically, 45 (41.3%) were Malay followed by 37 Chinese (33.9%). Among risk factors for TB lymphadenitis, HIV and diabetes mellitus were seen in 17 (15.6%) and 11 (10.0%) patients, respectively. Cough and fever were the most frequently reported symptoms. In a majority of cases (n = 90, 82.5%) positive results were obtained for fine-needle aspiration (FNA). Directly observed therapy was given to all patients. Sixty-two (56.9%) patients were successfully treated, and 5 (4.6%) patients died during the treatment. Conclusion: There was no increase in the incidence of TB lymphadenitis over the 3-year study period. The incidence was slightly higher in male than female gender and in Malay (ethnic group). Diabetes mellitus and HIV were the most commonly reported risk factors. FNA is the most reliable diagnostic test. Copyright © 2010 S. Karger AG, Basel.

Institution

(Muttalif) Department of Respiratory Medicine, Penang General Hospital, Malaysia (Hassali) Discipline of Social and Administrative Pharmacy, School of Pharmaceutical Sciences, Universiti Sains Malaysia, Malaysia

(Khan, Sulaiman, Khan) School of Pharmaceutical Sciences, Universiti Sains Malaysia, Penang, Malaysia

(Khan) College of Clinical Pharmacy, King Faisal University, Al-Ahsa, Saudi Arabia

Publisher

S. Karger AG (Allschwilerstrasse 10, P.O. Box, Basel CH-4009, Switzerland)

Emtree Heading

abdominal swelling; adolescent; adult; aged; article; aspiration biopsy; backache; child; chill; coughing; diabetes mellitus; drug treatment failure; dyspnea; ethnicity; fatigue; female; fever; general hospital; hemoptysis; human; Human immunodeficiency virus infection; lethargy; lymphadenopathy; major clinical study; Malaysia; male; medical record review; night sweat; retrospective study; risk factor; school child; symptom; thorax pain; thorax radiography; treatment duration; *tuberculous lymphadenitis/di [Diagnosis]; *tuberculous lymphadenitis/dt [Drug Therapy]; weight reduction; ethambutol/cb [Drug Combination]; ethambutol/dt [Drug Therapy]; isoniazid/cb [Drug Combination]; isoniazid/dt [Drug Therapy]; pyrazinamide/cb [Drug Combination]; pyrazinamide/dt [Drug Therapy]; rifampicin/cb [Drug Combination]; rifampicin/dt [Drug Therapy]; streptomycin/cb [Drug Combination]; streptomycin/dt [Drug Therapy].

Link to the Ovid Full Text or citation:

Click here for full text options

206.

Risk factors and outcome of treatment in patients with psoas abscesses.

Kara A., Kizilarslanoglu M.C., Alp S., Akova M.

Clinical Microbiology and Infection. Conference: 21st ECCMID/27th ICC Milan Italy. Conference Start: 20110507 Conference End: 20110510. Conference Publication: (var.pagings). 17 (pp S284), 2011. Date of Publication: May 2011.

AN: 70599756

Objectives: Although psoas abscesses (PAs) have not been reported frequently in the literature, they remain a significant therapeutic challenge. We retrospectively evaluated demographic characteristics, symptomatology, predisposing factors, microbiology and treatment outcomes of patients with PAs in a large tertiary care university hospital. Methods:We have electronically searched Hacettepe University Hospital database. There were 83 patients who were diagnosed clinically and radiologically with PA between 2003 and 2009. Individual patient files were searched for detailed patient characteristics, possible risk factors and underlying diseases, infecting microorganisms and treatment outcomes. Results: 37 (44.6%) of 83 patients were female. Median age was 51 years (range: 18-83). Mean abscess size was 6.4 centimeters (range: 1-25). Fever and pain were the most common symptoms. In 37 patients, abscesses were localized at right psoas, in 40 patients at left and 6 patients had bilaterally. Multiple abscess formation were determined in 10 patients. The most common underlying diseases were malignancies (16.9%) (4 with bladder carcinoma, 3 with colon cancer, 2 with multiple myeloma and one of each with lung, breast, endometrium, ovarian and prostate cancer), diabetes mellitus (13.2%), Crohn's disease (4.8%), and rheumatological diseases (4.8%). 13 patients (15.7%) had no underlying disease and 46 (55.4%) of patients had a history of an abdominal operation. 19 (22.9%) patients had previously and incompletely been treated for PA. The most common isolated microorganisms were Staphylococcus aureus (18 MSSA, 5 MRSA), Escherichia coli (n = 13; 3 of which with ESBL production) and Mycobacterium tuberculosis (n = 11). 68 patients (81.9%) were treated successfully with percutaneous drainage and with appropriate antimicrobial therapy. Four patients (4.8%) died due to complications related to PA. Conclusion: We report a high-rate of previous surgical intervention as a predisposing factor for PAs. M. tuberculosis was also a frequent cause of PAs which might be a consequence of intermediate-high prevalence of tuberculosis in Turkey. High rates of treatment success were achieved by a combination of drainage and antimicrobial treatment.

Institution

(Kara, Kizilarslanoglu, Alp, Akova) AnkaraTurkey

Publisher

Blackwell Publishing Ltd

Emtree Heading

*patient; *human; *psoas abscess; *risk factor; abscess; treatment outcome; university hospital; disease predisposition; antimicrobial therapy; microorganism; tuberculosis; Mycobacterium tuberculosis; percutaneous drainage; prevalence; female; demography; symptomatology; microbiology; tertiary health care; data base; fever; pain; surgery; Crohn disease; bladder carcinoma; colon cancer; multiple myeloma; lung; breast; endometrium; prostate cancer; diabetes mellitus; abdominal surgery; Staphylococcus aureus; Escherichia coli; methicillin resistant Staphylococcus aureus; nitrogen 13; extended spectrum beta lactamase.

Link to the Ovid Full Text or citation:

Click here for full text options

207.

Clinical characteristics and treatment outcomes of tuberculosis in hospitals of two large cities in Thailand.

Manosuthi W., Kawkitinarong K., Suwanpimolkul G., Chokbumrungsuk C., Jirawattanapisal T., Ruxrungtham K., Akksilp S.

Clinical Microbiology and Infection. Conference: 21st ECCMID/27th ICC Milan Italy. Conference Start: 20110507 Conference End: 20110510. Conference Publication: (var.pagings). 17 (pp S50), 2011. Date of Publication: May 2011.

AN: 70599050

Background: Tuberculosis (TB) is a major public health threat of large cities in many countries. The current clinical situation of TB in large cities of Thailand has not been well established. Methods: A retrospective cohort study was conducted among patients diagnosed active TB in 6 hospitals in Bangkok and Nonthaburi between January 2008 and December 2008. Patients were classified into HIV sero-positive, sero-negative, and undetermined sero-status; and were followed until December 2009. Results: A total of 813 patients were identified. Mean+/-SD age was 41+/-14 years, 62% were male, and mean+/-SD body weight was 53+/-11 kilograms. The first three co-morbidities included 38% HIV infection, 6% diabetes, and 2% chronic liver disease. Of all, 66% were diagnosed isolated pulmonary TB. Median (IQR) duration from first presentation to TB diagnosis was 3 (0-10) days. Of all, 43% of patients performed TB culture and 16% did anti-TB drug susceptibility test. Isoniazid, rifampicin, and multi-drug resistance were found in 11%, 6% and 5%, respectively. For 1-year TB treatment outcomes, 52.1%, 19.4%, 11.9%, 8.7%, 6.8%, 1.0% were cured/completed treated, transferred out, defaulted, on-going treated, dead and failed, respectively. Survival rates at 2, 6, 12 and 24 months were 93%, 85%, 81%, and 81% in HIV sero-positve group; 96%, 94%, 92%, and 92% in HIV sero-negative group; 98%, 97%, 97%, and 97% in undetermined sero-status (log rank test, P<0.001). Cox's proportional hazard model showed that death was associated with TB/HIV co-infection (P<0.001, HR= 2.801), low body weight (P = 0.005, HR= 1.637), old age (P = 0.005, HR= 1.439), and extrapulmonary/disseminated TB (P = 0.021, HR= 2.184). Conclusions: HIV infection and diabetes are the common co-morbidities in TB patients. Unfavorable outcome of TB treatment is relatively high, particularly in patients with HIV.

Institution

(Manosuthi, Kawkitinarong, Suwanpimolkul, Chokbumrungsuk, Jirawattanapisal, Ruxrungtham, Akksilp) Nonthaburi, BangkokThailand

Publisher

Blackwell Publishing Ltd

Emtree Heading

*Thailand; *city; *treatment outcome; *hospital; *tuberculosis; human; Human immunodeficiency virus; patient; Human immunodeficiency virus infection; morbidity; body weight; diabetes mellitus; survival rate; log rank test; proportional hazards model; death; mixed infection; senescence; public health; cohort analysis; male; chronic liver disease; diagnosis; drug sensitivity; drug resistance; isoniazid; rifampicin.

Link to the Ovid Full Text or citation:

Click here for full text options

208.

The effect of smoking on the treatment outcome in patients with pulmonary tuberculosis: A prospective cohort study.

Castillo M.

Respirology. Conference: 16th Congress of the Asian Pacific Society of Respirology Shanghai China. Conference Start: 20111103 Conference End: 20111106. Conference Publication: (var.pagings). 16 (pp 73), 2011. Date of Publication: November 2011.

AN: 70575950

Smoking and tuberculosis have great impact on health. Smoking is prevalent worldwide occurring in 20% of adults while Tuberculosis is a universal problem. Studies have shown that although smoking has no etiological role in TB, higher prevalence of TB has been observed in smokers. However, only few studies have evaluated the impact of smoking on TB treatment. General Objective To determine the effect of smoking on the treatment outcome among patients with pulmonary tuberculosis. Specific Objectives To determine the prevalence of smoking among patients with PTB, incidence of treatment success and failure among patients treated for PTB and to determine the association between smoking and treatment outcomes. Methodology A prospective cohort study done in Dr. Jose Rodriguez Memorial Hospital, DOTS Centre included all patients diagnosed with PTB, ages 18 years and above. Demographics and smoking history were obtained by standardized interview. CXR and sputum AFB were done. Sputum examination was repeated on 2nd, 4th month and on completion of treatment. Result A total of 197 pulmonary tuberculosis patients were enrolled in the DOTs Center during the study period. Of the 111 patients available for interview, only 105 patients were included in the assessment. There were 38 non smoker (36.2%) and 65 with smoking history (39 current smoker and 28 previous smoker). Patient's mean age was 42.6 +/- 15.4 (mean +/-SD). Female patients predominate among non smokers (73.7%) while male gender among current and previous smokers (84.6% and 82.1% respectively). Co-existing illnesses are hypertension, diabetes, asthma and COPD. Current smokers have more progressive CXR findings as manifested by either far advanced/ cavitary lesion (33.3%). Only 67 out of 197 patients had 2 or more sputum positive smear on baseline examination. The rest had either doubtful or negative smear but was assessed by the TB Diagnostic Committee. Among the 67 smear positive patients, 40 patients are +3 (38.1%), and 27 patient are +2 (25.7%). The remaining 38 subjects are either sputum +1 (14.3%) or sputum smear negative (21.9%). Of the 105 patients, 5 patients were classified treatment failure, 1 non-smoker, 2 previous smokers and 2 current smokers. Conclusion: Patients under DOTS program have high success rate. Although the study did not reach statistical significance, there was a strong trend on the association of smoking with cavitary or far advanced PTB and treatment failure.

Institution

(Castillo) Division of Pulmonary, Critical Care Medicine, Philippine Heart Center, Quezon, Philippines

Publisher

Blackwell Publishing

Emtree Heading

*smoking; *lung tuberculosis; *human; *patient; *society; *treatment outcome; *Asian; *cohort analysis; sputum; smear; interview; prevalence; tuberculosis; treatment failure; short course therapy; health; adult; methodology; hospital; sputum examination; male; gender; general aspects of disease; hypertension; diabetes mellitus; asthma; examination; diagnosis; sputum smear; statistical significance; female.

Link to the Ovid Full Text or citation:

Click here for full text options

209.

A comparative study of dot and sat in rural tertiary care hospital: A two years retrospective study.

Jagadish Babu D., Jyothi K.

International Journal of Pharmaceutical Sciences and Research. 3 (7) (pp 2283-2286), 2012. Date of Publication: July 2012.

AN: 2013131174

Directly observed therapy (DOT) of tuberculosis patients talking their drugs is supposed to improve treatment completion and outcome .we compared DOT and Self Administered therapy (SAT), in which patients on the same drug regimen are not, observed taking their pills, to assess the effect of each on the success of tuberculosis treatment. This was the two year (2010 and 2011) retrospective study of tuberculosis patients admitted to the AH and RC.A total of 308 patients cases were analyzed. The majority of the cases were within 21-40 years TB patients were 38.96% of the total. Among the all TB patients with chronic illness others were 18(58.44%) more. Among 308 patients TB patients 131(42.53%) TB patients undergone DOT and 177 (57.46%) TB patient's undergone SAT therapy. The comparative outcome between DOT and SAT were 110(86.61%) of cure and in SAT were 90 (57.32%). Only 5(03.93%) treatment failure, acquired drug resistance 01(00.78%), follow up lost were 11(08.66%) in DOT as compared to SAT 23(14.64%), 06(03.82%), 23(14.64%) respectively. There is no relapse and death in DOT therapy but in SAT therapy relapse were 10(06.36%) and deaths were 05(03.18%). There were large and significant difference in the cure, treatment failure, relapse and death rates between TB patient who receive DOT and SAT therapy respectively.

Institution

(Jagadish Babu, Jyothi) Sri Adichunchanagari College of Pharmacy, Department of Clinical Pharmacy, B.G.nagara-571 448, Mandaya District, Karnataka, India

Publisher

Society of Pharmaceutical Sciences and Research (1117, Sector 12 A, Panchakula, Haryana, India)

Emtree Heading

adult; age distribution; aged; antibiotic resistance; article; bacterial transmission; clinical assessment; comparative effectiveness; comparative study; controlled study; diabetes mellitus; *directly observed therapy; *drug self administration; drug treatment failure; follow up; human; hypertension; infant; infection risk; intermethod comparison; ischemic heart disease; lung tuberculosis/dt [Drug Therapy]; major clinical study; mortality; occupational exposure; outcome assessment; relapse; retrospective study; risk reduction; social stigma; tertiary health care; tuberculostatic agent/dt [Drug Therapy].

Link to the Ovid Full Text or citation:

Click here for full text options

210.

Years lived with disability (YLDs) for 1160 sequelae of 289 diseases and injuries 1990-2010: A systematic analysis for the Global Burden of Disease Study 2010.

Vos T., Flaxman A.D., Naghavi M., Lozano R., Michaud C., Ezzati M., Shibuya K., Salomon J.A., Abdalla S., Aboyans V., Abraham J., Ackerman I., Aggarwal R., Ahn S.Y., Ali M.K., Alvarado M., Anderson H.R., Anderson L.M., Andrews K.G., Atkinson C., Baddour L.M., Bahalim A.N., Barker-Collo S., Barrero L.H., Bartels D.H., Basanez M.-G., Baxter A., Bell M.L., Benjamin E.J., Bennett D., Bernabe E., Bhalla K., Bhandari B., Bikbov B., Abdulhak A.B., Birbeck G., Black J.A., Blencowe H., Blore J.D., Blyth F., Bolliger I., Bonaventure A., Boufous S., Bourne R., Boussinesq M., Braithwaite T., Brayne C., Bridgett L., Brooker S., Brooks P., Brugha T.S., Bryan-Hancock C., Bucello C., Buchbinder R., Buckle G., Budke C.M., Burch M., Burney P., Burstein R., Calabria B., Campbell B., Canter C.E., Carabin H., Carapetis J., Carmona L., Cella C., Charlson F., Chen H., Cheng A.T.-A., Chou D., Chugh S.S., Coffeng L.E., Colan S.D., Colquhoun S., Colson K.E., Condon J., Connor M.D., Cooper L.T., Corriere M., Cortinovis M., De Vaccaro K.C., Couser W., Cowie B.C., Criqui M.H., Cross M., Dabhadkar K.C., Dahiya M., Dahodwala N., Damsere-Derry J., Danaei G., Davis A., De Leo D., Degenhardt L., Dellavalle R., Delossantos A., Denenberg J., Derrett S., Des Jarlais D.C., Dharmaratne S.D., Dherani M., Diaz-Torne C., Dolk H., Dorsey E.R., Driscoll T., Duber H., Ebel B., Edmond K., Elbaz A., Ali S.E., Erskine H., Erwin P.J., Espindola P., Ewoigbokhan S.E., Farzadfar F., Feigin V., Felson D.T., Ferrari A., Ferri C.P., Fevre E.M., Finucane M.M., Flaxman S., Flood L., Foreman K., Forouzanfar M.H., Fowkes F.G.R., Franklin R., Fransen M., Freeman M.K., Gabbe B.J., Gabriel S.E., Gakidou E., Ganatra H.A., Garcia B., Gaspari F., Gillum R.F., Gmel G., Gonzalez-Medina D., Gosselin R., Grainger R., Grant B., Groeger J., Guillemin F., Gunnell D., Gupta R., Haagsma J., Hagan H., Halasa Y.A., Hall W., Haring D., Haro J.M., Harrison J.E., Havmoeller R., Hay R.J., Higashi H., Hill C., Hoen B., Hoffman H., Hotez P.J., Hoy D., Huang J.J., Ibeanusi S.E., Jacobsen K.H., James S.L., Jarvis D., Jasrasaria R., Jayaraman S., Johns N., Jonas J.B., Karthikeyan G., Kassebaum N., Kawakami N., Keren A., Khoo J.-P., King C.H., Knowlton L.M., Kobusingye O., Koranteng A., Krishnamurthi R., Laden F., Lalloo R., Laslett L.L., Lathlean T., Leasher J.L., Lee Y.Y., Leigh J., Levinson D., Lim S.S., Limb E., Lin J.K., Lipnick M., Lipshultz S.E., Liu W., Loane M., Ohno S.L., Lyons R., Ma J., Mabweijano J., MacIntyre M.F., Malekzadeh R., Mallinger L., Manivannan S., Marcenes W., March L., Margolis D.J., Marks G.B., Marks R., Matsumori A., Matzopoulos R., Mayosi B.M., McAnulty J.H., McDermott M.M., McGill N., McGrath J., Medina-Mora M.E., Meltzer M., Mensah G.A., Merriman T.R., Meyer A.-C., Miglioli V., Miller M., Miller T.R., Mitchell P.B., Mock C., Mocumbi A.O., Moffitt T.E., Mokdad A.A., Monasta L., Montico M., Moran A., Morawska L., Mori R., Murdoch M.E., Mwaniki M.K., Naidoo K., Nair M.N., Naldi L., Narayan K.M.V., Nelson P.K., Nelson R.G., Nevitt M.C., Newton C.R., Nolte S., Norman P., Norman R., O'Donnell M., O'Hanlon S., Olives C., Omer S.B., Ortblad K., Osborne R., Ozgediz D., Page A., Pahari B., Pandian J.D., Rivero A.P., Patten S.B., Pearce N., Padilla R.P., Perez-Ruiz F., Perico N., Pesudovs K., Phillips D., Phillips M.R., Pierce K., Pion S., Polanczyk G.V., Polinder S., Pope III C.A., Popova S., Porrini E., Pourmalek F., Prince M., Pullan R.L., Ramaiah K.D., Ranganathan D., Razavi H., Regan M., Rehm J.T., Rein D.B., Remuzzi G., Richardson K., Rivara F.P., Roberts T., Robinson C., De Leon F.R., Ronfani L., Room R., Rosenfeld L.C., Rushton L., Sacco R.L., Saha S., Sampson U., Sanchez-Riera L., Sanman E., Schwebel D.C., Scott J.G., Segui-Gomez M., Shahraz S., Shepard D.S., Shin H., Shivakoti R., Singh D., Singh G.M., Singh J.A., Singleton J., Sleet D.A., Sliwa K., Smith E., Smith J.L., Stapelberg N.J.C., Steer A., Steiner T., Stolk W.A., Stovner L.J., Sudfeld C., Syed S., Tamburlini G., Tavakkoli M., Taylor H.R., Taylor J.A., Taylor W.J., Thomas B., Thomson W.M., Thurston G.D., Tleyjeh I.M., Tonelli M., Towbin J.A., Truelsen T., Tsilimbaris M.K., Ubeda C., Undurraga E.A., Van Der Werf M.J., Van Os J., Vavilala M.S., Venketasubramanian N., Wang M., Wang W., Watt K., Weatherall D.J., Weinstock M.A., Weintraub R., Weisskopf M.G., Weissman M.M., White R.A., Whiteford H., Wiebe N., Wiersma S.T., Wilkinson J.D., Williams H.C., Williams S.R.M., Witt E., Wolfe F., Woolf A.D., Wulf S., Yeh P.-H., Zaidi A.K.M., Zheng Z.-J., Zonies D., Lopez A.D., Murray C.J.L., Moradi-Lakeh M.

The Lancet. 380 (9859) (pp 2163-2196), 2012. Date of Publication: December 2012.

AN: 2012732549

Background Non-fatal health outcomes from diseases and injuries are a crucial consideration in the promotion and monitoring of individual and population health. The Global Burden of Disease (GBD) studies done in 1990 and 2000 have been the only studies to quantify non-fatal health outcomes across an exhaustive set of disorders at the global and regional level. Neither effort quantified uncertainty in prevalence or years lived with disability (YLDs). Methods Of the 291 diseases and injuries in the GBD cause list, 289 cause disability. For 1160 sequelae of the 289 diseases and injuries, we undertook a systematic analysis of prevalence, incidence, remission, duration, and excess mortality. Sources included published studies, case notification, population-based cancer registries, other disease registries, antenatal clinic serosurveillance, hospital discharge data, ambulatory care data, household surveys, other surveys, and cohort studies. For most sequelae, we used a Bayesian meta-regression method, DisMod-MR, designed to address key limitations in descriptive epidemiological data, including missing data, inconsistency, and large methodological variation between data sources. For some disorders, we used natural history models, geospatial models, back-calculation models (models calculating incidence from population mortality rates and case fatality); or registration completeness models; (models adjusting for incomplete registration with health-system access and other covariates). Disability weights for 220 unique health states were used to capture the severity of health loss. YLDs by cause at age, sex, country, and year levels were adjusted for comorbidity with simulation methods. We included uncertainty estimates at all stages of the analysis. Findings Global prevalence for all ages combined in 2010 across the 1160 sequelae ranged from fewer than one case per 1 million people to 350 000 cases per 1 million people. Prevalence and severity of health loss were weakly correlated (correlation coefficient -0 37). In 2010, there were 777 million YLDs from all causes, up from 583 million in 1990. The main contributors to global YLDs were mental and behavioural disorders, musculoskeletal disorders, and diabetes or endocrine diseases. The leading specific causes of YLDs were much the same in 2010 as they were in 1990: low back pain, major depressive disorder, iron-deficiency anaemia, neck pain, chronic obstructive pulmonary disease, anxiety disorders, migraine, diabetes, and falls. Age-specific prevalence of YLDs increased with age in all regions and has decreased slightly from 1990 to 2010. Regional patterns of the leading causes of YLDs were more similar compared with years of life lost due to premature mortality. Neglected tropical diseases, HIV/AIDS, tuberculosis, malaria, and anaemia were important causes of YLDs in sub-Saharan Africa. Interpretation Rates of YLDs per 100 000 people have remained largely constant over time but rise steadily with age. Population growth and ageing have increased YLD numbers and crude rates over the past two decades. Prevalences of the most common causes of YLDs, such as mental and behavioural disorders and musculoskeletal disorders, have not decreased. Health systems will need to address the needs of the rising numbers of individuals with a range of disorders that largely cause disability but not mortality. Quantification of the burden of non-fatal health outcomes will be crucial to understand how well health systems are responding to these challenges. Effective and affordable strategies to deal with this rising burden are an urgent priority for health systems in most parts of the world.

Institution

(Baxter, Erskine, Ferrari, Keren, Saha, Whiteford) Queensland Centre for Mental Health Research, Australia (Vos, Blore, Charlson, Hay, Leasher, Norman, Page, Lopez) School Population of Healtho, Australia

(Ganatra, Halasa, Patten) Centre for Clinical Research, Australia

(McGill) Queensland Brain Institute, University of Queensland, Brisbane, QLD, Australia

(Flaxman, Naghavi, Lozano, Ahn, Alvarado, Andrews, Atkinson, Bolliger, Burstein, Campbell, Colson, Delossantos, Dharmaratne, Forouzanfar, Fransen, Gabriel, Gmel, Hall, Haro, Jarvis, Jayaraman, Levinson, Loane, Mabweijano, Malekzadeh, Moffitt, Nair, Ortblad, Phillips, Pierce, Ranganathan, Roberts, Rosenfeld, Sanman, Wang, Wulf, Murray) Institute for Health Metrics and Evaluation, University of Washington, 2301 Fifth Avenue, Seattle, WA 98121, United States

(Anderson) Department of Epidemiology, School of Public Health, United States

(Karthikeyan) Department of Anesthesiology and Pain Medicine, United States

(Couser, Duber, Ebel, Mitchell, Olives, Rivara, Vavilala) University of Washington, Seattle, WA, United States

(Michaud) China Medical Board, Boston, MA, United States

(Ezzati) MRCHPA Centre for Environment and Health, Department of Epidemiology and Biostatistics, School of Public Health, United Kingdom

(Basanez, Burney, Foreman, James, O'Hanlon, Rushton) Imperial College London, London, United Kingdom

(Shibuya) Department of Global Health Policy, Japan

(Kassebaum) University of Tokyo, Tokyo, Japan

(Finucane) Department of Biostatistics, United States

(Salomon) School of Public Health, United States

(Danaei, Krishnamurthi, Limb, Miglioli, Singh, Sudfeld, Tavakkoli, Weisskopf, White) 1Department of Epidemiology, United States

(Lipshultz) Harvard Humanitarian Initiative, United States

(King) Boston Childrenaes Hospital, United States

(Colan) Brigham and Womenaes Hospital, United States

(Jasrasaria) Harvard Medical School, Harvard University, Boston, MA, United States

(Bartels) Sudanese Public Health Consultancy Group, Sudan

(Abdalla) Department of Cardiology, Dupuytren University Hospital, Limoges, France

(Aboyans) University of Texas, San Antonio, TX, United States

(Abraham) Centre for International Child Health, Australia

(Steer) Department of Pediatrics, Australia

(Weintraub) Centre for Health Policy, Programs and Economics, Australia

(Degenhardt) School of Population Health, Australia

(Room) University of Melbourne, Melbourne, VIC, Australia

(Ackerman, Brooks, Marks, Taylor) Sanjay Gandhi Postgraduate Institute of Medical Sciences, Lucknow, India

(Aggarwal) Schools of Public Health and Medicine, United States

(Omer) Emory University, Atlanta, GA, United States

(Ali) St Georgeaes University of London, London, United Kingdom

(Dabhadkar, Gakidou, Narayan) Mayo Clinic, Rochester, MN, United States

(Anderson, Lim) Independent Consultant, Geneva, Switzerland

(Gunnell) University of Auckland, Auckland, New Zealand

(Baddour, Erwin, Gabbe) Department of Industrial Engineering, School of Engineering, Pontificia Universidad Javeriana, Bogota, Colombia

(Bahalim) Global Partners in Anesthesia and Surgery, United States

(Barker-Collo) Yale University, New Haven, CT, United States

(Barrero) School of Medicine, United States

(Ozgediz) Boston University, Boston, MA, United States

(Bell, Hoy) Clinical Trial Service Unit and Epidemiological Studies Unit, United Kingdom

(Felson) University of Oxford, Oxford, United Kingdom

(Benjamin) Dental Institute, United Kingdom

(Bennett) Hospital NHS Trust, United Kingdom

(Bernabe) Institute of Psychiatry, Kingaes College London, London, United Kingdom

(Havmoeller) Institute of Dentistry, United Kingdom

(Prince) Queen Mary University of London, London, United Kingdom

(Dahiya) Moscow State University of Medicine and Dentistry, Research Institute of Transplantology and Artificial Organs, Moscow, Russian Federation

(Bhandari) King Fahad Medical City, Riyadh, Saudi Arabia

(Manivannan) Michigan State University, East Lansing, MI, United States

(Bikbov) MRC Epidemiology Unit, Cambridge, United Kingdom

(Abdulhak) London School of Hygiene and Tropical Medicine, London, United Kingdom

(Tleyjeh) Department of Rheumatololgy, Northern Clinical School, Australia

(Birbeck) Faculty of Health Sciences, Australia

(Black) Institute of Bone and Joint Research, Australia

(Blencowe, Brooker, Edmond, Pearce, Pullan, Smith) Sydney School of Public Health, Australia

(Smith) University of Sydney, Sydney, NSW, Australia

(Marcenes, Sanchez-Riera) Inserm, Paris, France

(Driscoll, Lee) Transport and Road Safety Research, Australia

(Blyth, Bridgett, Cross, Margolis, McDermott) National Drug and Alcohol Research Centre, Australia

(Bonaventure, Elbaz) University of New South Wales, Sydney, NSW, Australia

(Boufous) Vision and Eye Research Unit, Anglia Ruskin University, Cambridge, United Kingdom

(Calabria, Degenhardt, Nelson, Singleton) Institut de Recherche pour le Developpement, Martinique

(Bucello, Miller) Moorfields Eye Hospital, London, United Kingdom

(Bourne) University of Cambridge, Cambridge, United Kingdom

(Boussinesq) University of Leicester, Leicester, United Kingdom

(Pion) Flinders University, Adelaide, SA, Australia

(Braithwaite) Cabrini Institute, Malvern, VIC, Australia

(Brayne, Richardson) Department of Epidemiology and Preventive Medicine, Australia

(Brugha) Monash University, Melbourne, VIC, Australia

(Bryan-Hancock) Bloomberg School of Public Health, United States

(Flood, Haro, Laslett, Pesudovs) Johns Hopkins University, Baltimore, MD, United States

(Buchbinder) Texas AandM University, College Station, TX, United States

(Freeman) Great Ormond Street Hospital, London, United Kingdom

(Buchbinder, Hotez) Washington University, St Louis, MO, United States

(Buckle, Mallinger) University of Oklahoma, Health Sciences Center, Oklahoma City, OK, United States

(Dorsey, Shivakoti) Telethon Institute for Child Health Research, Centre for Child Health Research, Australia

(Budke) University of Western Australia, Perth, WA, Australia

(Burch) Universidad Camilo Jose Cela, Villanueva de la Canada, Spain

(Canter) Mario Negri Institute for Pharmacological Research, Bergamo, Italy

(Carabin) National Institute of Environmental Health Sciences, Research Triangle Park, NC, United States

(Carapetis) Institute of Biomedical Sciences, Academia Sinica, Taipei, Taiwan (Republic of China)

(Norman) Heart Institute, United States

(Carmona) Cedars-Sinai Medical Center, Los Angeles, CA, United States

(Cella, Cortinovis, Garcia, Meyer, Perico, Remuzzi) Department of Public Health, United States

(Chen) Erasmus MC, University Medical Center Rotterdam, Rotterdam, Netherlands

(Cheng) Menzies School of Health Research, Darwin, NT, Australia

(Harrison) National Health Services, Fife, Edinburgh, United Kingdom

(Chugh) University of Edinburgh, Edinburgh, United Kingdom

(Polinder) University of the Witwatersrand, Johannesburg, South Africa

(Coffeng, Gupta, Stolk) Loyola University Medical School, Chicago, IL, United States

(Colquhoun, Condon) School of Public Health Sciences, Wake Forest University, Winston-Salem, NC, United States

(Connor, Fowkes) Hospital Dr Gustavo N Collado, Puerto Chitre, Panama

(Connor, Fevre) Victorian Infectious Diseases Reference Laboratory, Melbourne, VIC, Australia

(Cooper) University of California, San Diego, San Diego, CA, United States

(Corriere) University of Pennsylvania, Philadelphia, PA, United States

(De Vaccaro) Building and Road Research Institute, Kumasi, Ghana

(Cowie) MRC Hearing and Communication Group, Manchester, United Kingdom

(Criqui, Denenberg) School of Dentistry and Oral Health, Australia

(Dahodwala, March) Population and Social Health Research Program, Australia

(Damsere-Derry) Griffith University, Brisbane, QLD, Australia

(Davis, Laden) Denver VA Medical Center, Denver, CO, United States

(Laden) University of Otago, Dunedin, New Zealand

(De Leo, Stapelberg) Beth Israel Medical Center, New York City, NY, United States

(Dellavalle) University of Peradeniya, Peradeniya, Sri Lanka

(Derrett, Gosselin, Mensah, Taylor, Thomson) University of Liverpool, Liverpool, United Kingdom

(Des Jarlais) Hospital de la Santa Creu i Sant Pau, Barcelona, Spain

(Dharmaratne) University of Ulster, Ulster, United Kingdom

(Dherani) Federal Ministry of Health, Khartoum, Sudan

(Diaz-Torne) Hospital Maciel, Montevideo, Uruguay

(Dolk, Liu) Emerald Public Health Consulting Services Ltd, Abuja, Nigeria

(Moffitt) Digestive Disease Research Center, Iran, Islamic Republic of

(Espindola, Moradi-Lakeh) Tehran University of Medical Sciences, Tehran, Iran, Islamic Republic of

(Ewoigbokhan) National Institute for Stroke and Applied Neurosciences, New Zealand

(MacIntyre) Auckland Technical University, Auckland, New Zealand

(Farzadfar, Montico) Medical School, Brazil

(Feigin) Federal University of Sao Paulo, Sao Paulo, Brazil

(Koranteng, Witt) Carnegie Mellon University, Pittsburgh, PA, United States

(Polanczyk) James Cook University, Townsville, QLD, Australia

(Ferri) Howard University, College of Medicine, Washington, DC, United States

(Watt) Addiction Info Switzerland, Lausanne, Switzerland

(Gaspari) Department of Epidemiology and Biostatistics, United States

(Gillum) University of California, San Francisco, San Francisco, CA, United States

(Nevitt) College of Medicine, SUNY Downstate Medical Center, Brooklyn, NY, United States

(Gonzalez-Medina, Lin, Merriman, Robinson) National Center for Injury Prevention and Control, United States

(Grant) Centers for Disease Control and Prevention, Atlanta, GA, United States

(Sleet) Universite de Lorraine, Nancy, France

(Wiersma) University of Bristol, Bristol, United Kingdom

(Groeger) New York University, New York City, NY, United States

(Guillemin) Brandeis University, Waltham, MA, United States

(Haagsma) Parc Sanitari Sant Joan de Deu, CIBERSAM, Universitat de Barcelona, Sant Boi de Llobregat, Spain

(Thurston) Karolinska University Hospital, Stockholm, Sweden

(Hagan) Queen Elizabeth Hospital, Adelaide, SA, Australia

(Shahraz) Universite de Franche-Comte, Besancon, France

(Shepard) Centre Hospitalier Regional, Universitaire de Besancon, Besancon, France

(Undurraga) National Institute on Deafness and Other Communication Disorders, United States

(Haring) National Institute of Diabetes and Digestive and Kidney Diseases, National Institutes of Health, Bethesda, MD, United States

(Harrison) National School of Tropical Medicine, Baylor College of Medicine, Waco, TX, United States

(Higashi) University of Port Harcourt, Port Harcourt, Nigeria

(Hill) George Mason University, Fairfax, VA, United States

(Hill) Department of Ophthalmology, Medical Faculty Mannheim, Ruprecht Karls University, Heidelberg, Germany

(Hoen) All India Institute of Medical Sciences, New Delhi, India

(Nelson) Department of Cardiology, Hebrew University Hadassah Medical School, Jerusalem, Israel

(Hoffman) Case Western Reserve University, Cleveland, OH, United States

(Huang) School of Public Health, Makerere University, Kampala, Uganda

(Ibeanusi, Johns) University of South Africa, Johannesburg, South Africa

(Jonas) Kwame Nkrumah University of Science and Technology, Kumasi, Ghana

(Kawakami) University of Tasmania, TAS, Australia

(Khoo) Nova Southeastern University, Fort Lauderdale, FL, United States

(Knowlton) Ministry of Health, Jerusalem, Israel

(Knowlton) Miller School of Medicine, University of Miami, Miami, FL, United States

(Kobusingye) Swansea University, Swansea, United Kingdom

(Lalloo) Mulago Hospital, Kampala, Uganda

(Lathlean) Asian Pacific Society of Cardiology, Kyoto, Japan

(Leigh, Lipnick, Sacco, Wilkinson) Medical Research Council, Tygerberg, South Africa

(Ohno) Hatter Institute, South Africa

(Lyons) Department of Medicine, South Africa

(Marks) University of Cape Town, Cape Town, South Africa

(Matsumori) Legacy Health System, Portland, OR, United States

(Sliwa) Northwestern University Feinberg, School of Medicine, Evanston, IL, United States

(Meltzer) National Institute on Psychiatry Ramon de la Fuente, Mexico City, Mexico

(Matsumori, Matzopoulos) Thomas Jefferson University, Philadelphia, PA, United States

(Mayosi) Pacific Institute for Research and Evaluation, Calverton, MD, United States

(McAnulty) National Institute of Health, Maputo, Mozambique

(McGrath) University Eduardo Mondlane, Maputo, Mozambique

(Medina-Mora) Duke University, Durham, NC, United States

(Miller) Institute for Maternal and Child Health, IRCCS Burlo Garofolo, Trieste, Italy

(Mock) Columbia University, New York City, NY, United States

(Mock) Queensland University of Technology, Brisbane, QLD, Australia

(Mocumbi, Mokdad, Monasta, Ronfani, Tamburlini) National Center for Child Health and Development, Tokyo, Japan

(Moran, Weissman) Watford General Hospital, Watford, United Kingdom

(Morawska) Kemri-Wellcome Trust, Kilifi, Kenya

(Mori) AVRI, University of KwaZulu-Natal, Durban, South Africa

(Murdoch) Centro Studi GISED, Bergamo, Italy

(Mwaniki) Charite- Universitatsmedizin Berlin, Berlin, Germany

(Naidoo) HRB-Clinical Research Facility, National University of Ireland Galway, Galway, Ireland

(Naldi) Deakin University, Melbourne, VIC, Australia

(Newton) B P Koirala Institute of Health Sciences, Dharan, Nepal

(O'Donnell) Betty Cowan Research and Innovation Center, Ludhiana, India

(Osborne) Hospital Juan XXIII, La Paz, Bolivia

(Pahari) University of Calgary, Calgary, AB, Canada

(Pandian) Instituto Nacional de Enfermedades Respiratorias, Mexico City, Mexico

(Rivero) Hospital Universitario Cruces, Barakaldo, Spain

(Patten) Shanghai Mental Health Center, School of Medicine, China

(Padilla) School of Public Health, Shanghai Jiao Tong University, Shanghai, China

(Perez-Ruiz) Brigham Young University, Provo, UT, United States

(Phillips) Centre for Addiction and Mental Health, Toronto, ON, Canada

(Zheng) Hospital Universitario de Canarias, Tenerif, Spain

(Pope III) Faculty of Medicine, School of Population and Public Health, University of British Columbia, Vancouver, BC, Canada

(Popova) Vector Control Research Centre, Pondicherry, India

(Rehm) Center for Disease Analysis, Louisville, CO, United States

(Porrini) University of California, Berkeley, Berkeley, CA, United States

(Pourmalek) NORC, University of Chicago, Chicago, IL, United States

(Ramaiah) Complejo Hospitalario Caja De Seguro Social, Panama City, Panama

(Razavi) Centre for Alcohol Policy Research, Turning Point Alcohol and Drug Centre, Fitzroy, SA, Australia

(Regan, Sampson) Vanderbilt University, Nashville, TN, United States

(Rein, Schwebel) University of Alabama at Birmingham, Birmingham, AL, United States

(De Leon) Ministry of Interior, Madrid, Spain

(Room) Health Canada, Ottawa, ON, Canada

(Stovner) Queens Medical Center, Honolulu, HI, United States

(Singh) Department of Neuroscience, Norway

(Segui-Gomez, Taylor) Norwegian University of Science and Technology, Trondheim, Norway

(Shin) Aga Khan University, Karachi, Pakistan

(Tonelli) Drexel University, School of Public Health, Philadelphia, PA, United States

(Steiner, Tsilimbaris, Wiebe) Alberta Kidney Disease Network, Canada

(Towbin, Zaidi) University of Alberta, Edmonton, AB, Canada

(Truelsen) Cincinnati Childrenaes Hospital, Cincinnati, OH, United States

(Syed) Department of Neurology, Copenhagen University Hospital, Herlev, Denmark

(Ubeda) University of Crete Medical School, Crete, Greece

(Van Der Werf) Instituto Nacional de Epidemiologia, ANLIS, Malbran, Argentina

(Van Os) KNCV Tuberculosis Foundation, The Hague, Netherlands

(Thomas) Maastricht University Medical Centre, Maastricht, Netherlands

(Wang) National University of Singapore, Singapore

(Weinstock) Beijing Neurosurgical Institute, Capital Medical University, Beijing, China

(Grainger) Brown University, Providence, RI, United States

(Venketasubramanian, Williams) Royal Childrenaes Hospital, Critical Care and Neurosciences Theme, Murdoch Childrenaes Research Institute, Melbourne, VIC, Australia

(Williams, Woolf) University of Nottingham, Nottingham, United Kingdom

(Williams) Hollywood Orthopaedic Group, Perth, WA, Australia

(Wolfe) Arthritis Research, Wichita, KS, United States

(Weintraub) Royal Cornwall Hospital, Truro, United Kingdom

(Weintraub, Yeh) London School of Economics, London, United Kingdom

(Zonies) Landstuhl Regional Medical Center, Landstuhl, Germany

(Ma) National Center for Non-Communicable Disease Control and Prevention, China Centers for Disease Control, Beijing, China

Publisher

Lancet Publishing Group (Langford Lane, Kidlington, Oxford OX5 1GB, United Kingdom)

Emtree Heading

acquired immune deficiency syndrome/co [Complication]; acquired immune deficiency syndrome/dt [Drug Therapy]; acquired immune deficiency syndrome/ep [Epidemiology]; age distribution; aging; anemia/co [Complication]; anemia/ep [Epidemiology]; antiviral therapy; anxiety disorder/co [Complication]; anxiety disorder/ep [Epidemiology]; article; behavior disorder/co [Complication]; behavior disorder/ep [Epidemiology]; chronic obstructive lung disease/co [Complication]; chronic obstructive lung disease/ep [Epidemiology]; comorbidity; diabetes mellitus/co [Complication]; diabetes mellitus/ep [Epidemiology]; *disability; disability severity; disease duration; endocrine disease/co [Complication]; endocrine disease/ep [Epidemiology]; falling; health care system; human; Human immunodeficiency virus infection/co [Complication]; Human immunodeficiency virus infection/ep [Epidemiology]; *iatrogenic disease/co [Complication]; *iatrogenic disease/ep [Epidemiology]; incidence; iron deficiency anemia/co [Complication]; iron deficiency anemia/ep [Epidemiology]; low back pain/co [Complication]; low back pain/ep [Epidemiology]; major depression/co [Complication]; major depression/ep [Epidemiology]; malaria/co [Complication]; malaria/ep [Epidemiology]; mental disease/co [Complication]; mental disease/ep [Epidemiology]; migraine/co [Complication]; migraine/ep [Epidemiology]; mortality; musculoskeletal disease/co [Complication]; musculoskeletal disease/ep [Epidemiology]; neck pain/co [Complication]; neck pain/ep [Epidemiology]; outcome assessment; population growth; prevalence; priority journal; remission; systematic review; tropical disease/co [Complication]; tropical disease/ep [Epidemiology]; tuberculosis/co [Complication]; tuberculosis/ep [Epidemiology]; *years lived with disability; anti human immunodeficiency virus agent/dt [Drug Therapy].

Link to the Ovid Full Text or citation:

Click here for full text options

211.

Inflammatory bowel disease: Perioperative pharmacological considerations.

Kumar A., Auron M., Aneja A., Mohr F., Jain A., Shen B.

Mayo Clinic Proceedings. 86 (8) (pp 748-757), 2011. Date of Publication: August 2011.

AN: 2011419773

The perioperative management of patients with inflammatory bowel disease is challenging given the altered immune system that results from a variety of biologic and immunomodulator therapies. Clinicians are often faced with challenges and complicated equations when deciding on the type and dose of medication. To understand the effect of these medications and review the evidence regarding the management of these medications in the perioperative setting, a PubMed-based literature search (January 1, 1960, through April 1, 2011) was conducted using the following search terms: perioperative management, risk, outcome, inflammatory bowel disease, ulcerative colitis, Crohn's disease, aminosalicylates, glucocorticoids, purine analogues, cyclosporine, methotrexate, biologic therapy, infliximab, and thromboembolism. The 414 articles identified were manually sorted to exclude those that did not address perioperative risk, outcomes, and medications in the abstracts, yielding 84 articles for review. Additional references were obtained from the citations within the retrieved articles. This review surveys the findings of the selected articles and presents guidelines and resources for perioperative medication management for patients with inflammatory bowel disease undergoing surgery. © 2011 Mayo Foundation for Medical Education and Research.

Institution

(Kumar, Auron) Department of Hospital Medicine, Children's Hospital, Cleveland Clinic, Cleveland, OH, United States (Mohr) Department of Pediatric Gastroenterology, Children's Hospital, Cleveland Clinic, Cleveland, OH, United States

(Shen) Digestive Disease Institute, Cleveland Clinic, Cleveland, OH, United States

(Aneja) Department of Medicine, MetroHealth Medical Center, Cleveland, OH, United States

(Jain) Department of Gastroenterology, MetroHealth Medical Center, Cleveland, OH, United States

Publisher

Elsevier Ltd

Emtree Heading

adrenal insufficiency/si [Side Effect]; anastomosis; antibacterial activity; antiinflammatory activity; artery thrombosis; biological therapy; bleeding/pc [Prevention]; bleeding/th [Therapy]; blood glucose monitoring; bone marrow suppression/si [Side Effect]; bone marrow toxicity/si [Side Effect]; colorectal cancer; colorectal surgery; combination chemotherapy; compression garment; Crohn disease/dt [Drug Therapy]; Crohn disease/su [Surgery]; deep vein thrombosis/co [Complication]; diabetes mellitus/si [Side Effect]; disease association; drug absorption; drug activity; drug dose escalation; drug elimination; drug excretion; drug half life; drug hypersensitivity/si [Side Effect]; drug megadose; drug potentiation; drug safety; drug withdrawal; elective surgery; emergency surgery; *enteritis/dt [Drug Therapy]; *enteritis/pc [Prevention]; *enteritis/su [Surgery]; *enteritis/th [Therapy]; evidence based practice; gastrointestinal hemorrhage/si [Side Effect]; glaucoma/si [Side Effect]; glucose intolerance/si [Side Effect]; hemolytic anemia/si [Side Effect]; homozygosity; hospital discharge; hospitalization; human; hyperglycemia/si [Side Effect]; hyperhomocysteinemia; ileal pouch anal anastomosis; immobilization; immunosuppressive treatment; infection/si [Side Effect]; infection risk; intraocular pressure; liver toxicity/si [Side Effect]; low drug dose; lung embolism; maintenance therapy; monotherapy; mortality; mucosa inflammation/si [Side Effect]; mutational analysis; nephrotoxicity/si [Side Effect]; nerve ending; nonhuman; operation duration; opportunistic infection/si [Side Effect]; osteopenia/si [Side Effect]; osteoporosis/si [Side Effect]; outcome assessment; pancreatitis/si [Side Effect]; patient monitoring; perioperative period; Pneumocystis pneumonia/dt [Drug Therapy]; Pneumocystis pneumonia/pc [Prevention]; pneumonia/si [Side Effect]; postoperative infection/co [Complication]; postoperative period; practice guideline; progressive multifocal leukoencephalopathy/si [Side Effect]; recommended drug dose; relapse; remission; review; risk assessment; risk factor; seizure/si [Side Effect]; sepsis/co [Complication]; sepsis/si [Side Effect]; side effect/si [Side Effect]; surgical stress; surgical technique; systematic review; thrombocytopenia/si [Side Effect]; thromboembolism; treatment duration; treatment indication; tuberculosis/si [Side Effect]; ulcerative colitis/dt [Drug Therapy]; ulcerative colitis/su [Surgery]; vein thrombosis; venous thromboembolism/dt [Drug Therapy]; venous thromboembolism/pc [Prevention]; venous thromboembolism/th [Therapy]; wound healing; wound healing impairment/si [Side Effect]; 5 acetamidosalicylic acid/dt [Drug Therapy]; 5 acetamidosalicylic acid/pk [Pharmacokinetics]; azathioprine/cb [Drug Combination]; azathioprine/dt [Drug Therapy]; azathioprine/pd [Pharmacology]; blood clotting factor 10a inhibitor/dt [Drug Therapy]; blood clotting factor 5 Leiden/ec [Endogenous Compound]; corticotropin/ec [Endogenous Compound]; cotrimoxazole/dt [Drug Therapy]; cyclosporin/ae [Adverse Drug Reaction]; cyclosporin/dt [Drug Therapy]; cyclosporin/pk [Pharmacokinetics]; enoxaparin; fondaparinux/dt [Drug Therapy]; fondaparinux/sc [Subcutaneous Drug Administration]; glucocorticoid/ae [Adverse Drug Reaction]; glucocorticoid/cb [Drug Combination]; glucocorticoid/dt [Drug Therapy]; glucocorticoid/ih [Inhalational Drug Administration]; glucocorticoid/tp [Topical Drug Administration]; heparin/dt [Drug Therapy]; heparin/sc [Subcutaneous Drug Administration]; hydrocortisone/dt [Drug Therapy]; hydrocortisone/iv [Intravenous Drug Administration]; infliximab/ae [Adverse Drug Reaction]; infliximab/dt [Drug Therapy]; infliximab/pk [Pharmacokinetics]; infliximab/pd [Pharmacology]; low molecular weight heparin/dt [Drug Therapy]; mercaptopurine/ae [Adverse Drug Reaction]; mercaptopurine/cb [Drug Combination]; mercaptopurine/dt [Drug Therapy]; mercaptopurine/pk [Pharmacokinetics]; mesalazine/ae [Adverse Drug Reaction]; mesalazine/dt [Drug Therapy]; mesalazine/pk [Pharmacokinetics]; mesalazine/pd [Pharmacology]; methotrexate/ae [Adverse Drug Reaction]; methotrexate/ct [Clinical Trial]; methotrexate/it [Drug Interaction]; methotrexate/dt [Drug Therapy]; methotrexate/pd [Pharmacology]; natalizumab/ae [Adverse Drug Reaction]; natalizumab/dt [Drug Therapy]; natalizumab/pd [Pharmacology]; nitrous oxide/ae [Adverse Drug Reaction]; nitrous oxide/it [Drug Interaction]; prednisone/dt [Drug Therapy]; purine derivative/cb [Drug Combination]; purine derivative/dt [Drug Therapy]; salazosulfapyridine/ae [Adverse Drug Reaction]; salazosulfapyridine/dt [Drug Therapy]; salazosulfapyridine/pk [Pharmacokinetics]; salazosulfapyridine/pd [Pharmacology]; sulfapyridine/dt [Drug Therapy]; sulfapyridine/pd [Pharmacology].

Link to the Ovid Full Text or citation:

Click here for full text options

212.

Association of diabetes and tuberculosis - A major public health challenge.

Aravindalochanan V., Viswanathan V., Kumpatla S.

Journal of the Indian Medical Association. 110 (8) (pp 646-648), 2012. Date of Publication: August 2012.

AN: 2012747667

The burden of tuberculosis and diabetes is remarkably high in India. There is an increased risk for diabetic subjects to have active form of tuberculosis compared to their normal counterparts. The chance of reactivation of past tuberculosis infection is also common among the people with diabetes. In addition to that, few studies had reported that proportion of poor tuberculosis treatment outcomes such as treatment failure and death was higher in people with both tuberculosis and diabetes. A recent report from Tamil Nadu showed a very high prevalence of diabetes and prediabetes among tuberculosis patients. These findings strongly suggest the need of universal screening for diabetes among tuberculosis patients. There are several questions to be answered to assist in the formulation of strategies to address this dual burden in developing countries like India. The main purpose of the article is to summarise and discuss the available evidences and to define the area of future research to identify a cost-effective screening method for diabetes among tuberculosis patients and the best mode of reporting and managing the two diseases for the better control of tuberculosis and diabetes.

Institution

(Aravindalochanan, Viswanathan, Kumpatla) MV Hospital for Diabetes, Prof M Viswanathan Diabetes Research Centre, WHO Collaborating Centre for Research, Education and Training in Diabetes, Chennai 600013, India

Publisher

Indian Medical Association (53 Creek Row, Calcutta 700014, India)

Emtree Heading

article; death; *diabetes mellitus/dm [Disease Management]; *diabetes mellitus/dt [Drug Therapy]; disease association; human; liver toxicity; patient care; postprimary tuberculosis; public health; risk factor; screening; treatment failure; *tuberculosis/dt [Drug Therapy]; oral antidiabetic agent/it [Drug Interaction]; oral antidiabetic agent/dt [Drug Therapy]; rifampicin/it [Drug Interaction]; rifampicin/dt [Drug Therapy].

Link to the Ovid Full Text or citation:

Click here for full text options

213.

Tuberculosis in an Urban Area in China: Differences between Urban Migrants and Local Residents.

Shen X., Xia Z., Li X., Wu J., Wang L., Li J., Jiang Y., Guo J., Chen J., Hong J., Yuan Z., Pan Q., DeRiemer K., Sun G., Gao Q., Mei J.

PLoS ONE. 7 (11) (no pagination), 2012. Article Number: e51133. Date of Publication: 30 Nov 2012.

AN: 2012718962

Background: The increase in urban migrants is one of major challenges for tuberculosis control in China. The different characteristics of tuberculosis cases between urban migrants and local residents in China have not been investigated before. Methodology/Principal Findings: We performed a retrospective study of all pulmonary tuberculosis patients reported in Songjiang district, Shanghai, to determine the demographic, clinical and microbiological characteristics of tuberculosis cases between urban migrants and local residents. We calculated the odds ratios (OR) and performed multivariate logistic regression to identify the characteristics that were independently associated with tuberculosis among urban migrants. A total of 1,348 pulmonary tuberculosis cases were reported during 2006-2008, among whom 440 (32.6%) were local residents and 908 (67.4%) were urban migrants. Urban migrant (38.9/100,000 population) had higher tuberculosis rates than local residents (27.8/100,000 population), and the rates among persons younger than age 35 years were 3 times higher among urban migrants than among local residents. Younger age (adjusted OR per additional year at risk = 0.92, 95% CI: 0.91-0.94, p<0.001), poor treatment outcome (adjusted OR = 4.12, 95% CI: 2.65-5.72, p<0.001), and lower frequency of any comorbidity at diagnosis (adjusted OR = 0.20, 95% CI: 0.13-0.26, p = 0.013) were significantly associated with tuberculosis patients among urban migrants. There were poor treatment outcomes among urban migrants, mainly from transfers to another jurisdiction (19.3% of all tuberculosis patients among urban migrants). Conclusions/Significance: A considerable proportion of tuberculosis cases in Songjiang district, China, during 2006-2008 occurred among urban migrants. Our findings highlight the need to develop and implement specific tuberculosis control strategies for urban migrants, such as more exhaustive case finding, improved case management and follow-up, and use of directly observed therapy (DOT). © 2012 Shen et al.

Institution

(Shen, Sun, Gao) Key Laboratory of Medical Molecular Virology, Shanghai Medical College, Fudan University, Shanghai, China (Shen, Xia, Li, Wu, Wang, Li, Jiang, Guo, Chen, Yuan, Pan, Mei) Department of Tuberculosis Control, Shanghai Municipal Center for Disease Control and Prevention, Shanghai, China

(Hong) Department of Tuberculosis Control, Songjiang District Center for Disease Control and Prevention, Shanghai, China

(DeRiemer) University of California, Davis, School of Medicine, Davis, CA, United States

Publisher

Public Library of Science (185 Berry Street, Suite 1300, San Francisco CA 94107, United States)

Emtree Heading

adult; age distribution; antibiotic resistance; article; cardiovascular disease; China; chronic obstructive lung disease; comorbidity; diabetes mellitus; disease association; female; hepatitis B; high risk population; human; infection rate; infection risk; lung cavitation; *lung tuberculosis; major clinical study; male; pneumonia; retrospective study; sex difference; sputum smear; thorax radiography; treatment outcome; *urban population; ethambutol; isoniazid; rifampicin; streptomycin.

Link to the Ovid Full Text or citation:

Click here for full text options

214.

The PREDICTIVETM Study: A multinational, prospective observational study to evaluate the safety and efficacy of insulin detemir treatment in patients with type 1 and 2 diabetes-data from the Japan cohort.

Namba M., Kaku K., Yoshioka N., Yamada Y., Watada H., Ueki K., Terauchi Y., Tobe K., Araki E., Shuto Y., Kadowaki T.

Diabetology International. 3 (1) (pp 11-20), 2012. Date of Publication: March 2012.

AN: 2012706617

The PREDICTIVETM Study of Japan was an open-label, non-randomized 52-week observational study to evaluate the safety and efficacy of insulin detemir when used in routine clinical practice. Patients with either type 1 or 2 diabetes in whom insulin detemir was to be prescribed but without a previous history of using insulin detemir were eligible to participate. The primary endpoint was an incidence of serious adverse drug reaction (SADR) for the entire observational period. Data were collected at baseline, 12, 26, and 52 weeks. A total of 3,519 patients were enrolled in 540 institutions between 2008 and 2009. Thirty patients (0. 9%) reported 32 SADRs. The rate of total hypoglycemic episodes was 1. 51 episodes/patient-year in patients with type 1 diabetes and 0. 23 episodes/patient-year in patients with type 2 diabetes. The rate of total hypoglycemic episodes did not increase during the observational period. Significant reductions were observed at 52 weeks in HbA1c levels (0. 51% in type 1 diabetes, P < 0. 001; 0. 80% in type 2 diabetes, P < 0. 001), fasting glucose level (reduction of 22. 1 mg/dl in type 1 diabetes, P < 0. 001; 25. 6 mg/dl in type 2 diabetes, P < 0. 001), and intrapatient fasting glucose variability (coefficient of variance; reduction of 11. 4% in type 1 diabetes, P < 0. 001; 4. 1% for type 2 diabetes, P < 0. 001). Our results from the 52-week follow-up data of 3,345 Japanese patients with diabetes demonstrated that insulin detemir contributed to a lower incidence of SADRs (<1%) and suggested that treatment with detemir could provide better glycemic control without increasing hypoglycemic episodes. © 2011 The Japan Diabetes Society.

Institution

(Namba) Division of Diabetes and Metabolism, Department of Internal Medicine, Hyogo College of Medicine, Mukogawa-cho 1-1, Nishinomiya, Hyogo, 663-8501, Japan (Kaku) Division of Diabetes, Endocrinology and Metabolism, Department of Internal Medicine, Kawasaki Medical School, Kurashiki, Okayama, Japan

(Yoshioka) Department of Division of Diabetes and Metabolism, Sapporo Medical Center NTT East Corporation, Sapporo, Hokkaido, Japan

(Yamada) Department of Endocrinology, Diabetes and Geriatric Medicine, Akita University Graduate School of Medicine, Akita, Japan

(Watada) Division of Metabolism and Endocrinology, Department of Internal Medicine, Juntendo University, Tokyo, Japan

(Ueki, Kadowaki) Department of Metabolic Diseases, Graduate School of Medicine, University of Tokyo, Tokyo, Japan

(Terauchi) Division of Endocrinology and Metabolism, Yokohama City University Graduate School of Medicine, Yokohama, Kanagawa, Japan

(Tobe) Department of Internal Medicine, Faculty of Medicine, University of Toyama, Toyama, Japan

(Araki) Department of Metabolic Medicine, Faculty of Life Sciences, Kumamoto University, Kumamoto, Japan

(Shuto) Safety Management Department, Novo Nordisk Pharma Ltd., Tokyo, Japan

Publisher

Springer Japan (1-11-11 Kudan-kita, Chiyoda-ku, No. 2 Funato Bldg., Tokyo 102-0073, Japan)

Emtree Heading

abdominal pain/si [Side Effect]; acute heart failure/si [Side Effect]; acute heart infarction/si [Side Effect]; acute pancreatitis/si [Side Effect]; acute pyelonephritis/si [Side Effect]; adult; angina pectoris/si [Side Effect]; article; aspiration pneumonia/si [Side Effect]; bile duct carcinoma/si [Side Effect]; bile duct stone/si [Side Effect]; bloating/si [Side Effect]; brain hemorrhage/si [Side Effect]; brain infarction/si [Side Effect]; cardiopulmonary arrest/si [Side Effect]; carotid artery obstruction/si [Side Effect]; carpal tunnel syndrome/si [Side Effect]; cataract/si [Side Effect]; cellulitis/si [Side Effect]; cholangitis/si [Side Effect]; cholecystitis/si [Side Effect]; chronic kidney failure/si [Side Effect]; clinical practice; clinical trial; colon cancer/si [Side Effect]; colon polyp/si [Side Effect]; congestive heart failure/si [Side Effect]; controlled study; decreased appetite/si [Side Effect]; dehydration/si [Side Effect]; diabetic ketoacidosis/si [Side Effect]; diabetic retinopathy/si [Side Effect]; digestive system perforation/si [Side Effect]; disseminated intravascular clotting/si [Side Effect]; dizziness/si [Side Effect]; drug efficacy; drug fatality/si [Side Effect]; drug safety; emphysema/si [Side Effect]; esophagus varices/si [Side Effect]; female; femur neck fracture/si [Side Effect]; follow up; forward heart failure/si [Side Effect]; gastroenteritis/si [Side Effect]; glucagonoma/si [Side Effect]; glucose blood level; glycemic control; heart arrest/si [Side Effect]; heart death/si [Side Effect]; heart failure/si [Side Effect]; heart infarction/si [Side Effect]; heart muscle ischemia/si [Side Effect]; heart ventricle extrasystole/si [Side Effect]; hepatitis B/si [Side Effect]; human; hypoglycemia/si [Side Effect]; hypoglycemic coma/si [Side Effect]; hypophagia/si [Side Effect]; hypotension/si [Side Effect]; ileus/si [Side Effect]; infection/si [Side Effect]; *insulin dependent diabetes mellitus/dt [Drug Therapy]; interstitial lung disease/si [Side Effect]; Japanese; ketoacidosis/si [Side Effect]; kidney carcinoma/si [Side Effect]; kidney failure/si [Side Effect]; liver cancer/si [Side Effect]; liver failure/si [Side Effect]; liver metastasis/si [Side Effect]; major clinical study; male; metastasis/si [Side Effect]; *non insulin dependent diabetes mellitus/dt [Drug Therapy]; observational study; obstructive jaundice/si [Side Effect]; pancreas cancer/si [Side Effect]; patella fracture/si [Side Effect]; peripheral edema/si [Side Effect]; peripheral occlusive artery disease/si [Side Effect]; peritonitis/si [Side Effect]; pneumocephalus/si [Side Effect]; pneumonia/si [Side Effect]; priority journal; prospective study; restenosis/si [Side Effect]; sepsis/si [Side Effect]; sick sinus syndrome/si [Side Effect]; side effect/si [Side Effect]; skin ulcer/si [Side Effect]; spondylitis/si [Side Effect]; spontaneous abortion/si [Side Effect]; stomach cancer/si [Side Effect]; sudden death; thyroid cancer/si [Side Effect]; transitional cell carcinoma/si [Side Effect]; treatment outcome; tuberculous peritonitis/si [Side Effect]; unconsciousness/si [Side Effect]; urethra stricture/si [Side Effect]; urinary tract infection/si [Side Effect]; uterus cancer/si [Side Effect]; vomiting/si [Side Effect]; weight reduction; glucose/ec [Endogenous Compound]; hemoglobin A1c/ec [Endogenous Compound]; *insulin detemir/ae [Adverse Drug Reaction]; *insulin detemir/ct [Clinical Trial]; *insulin detemir/dt [Drug Therapy].

Link to the Ovid Full Text or citation:

Click here for full text options

215.

Morbidity and mortality at five years after initiating category I treatment among patients with new sputum smear positive pulmonary tuberculosis.

Lisha P.V., James P.T., Ravindran C.

Indian Journal of Tuberculosis. 59 (2) (pp 83-91), 2012. Date of Publication: April 2012.

AN: 2012589607

Background: Evaluation of disease outcome is central to the assessment of tuberculosis control programmes. Most of the follow up studies in RNTCP are short term. Five year follow up studies have not been done previously in this region. The objective of the present study is to evaluate the outcome of Category I treatment in smear positive tuberculosis, five years after treatment in terms of relapse, sequelae and death and to know the associated factors. Material and Methods: Patients who had registered for Category 1 treatment during the period 2002 - 2004 were followed up at five years with clinical evaluation, Chest X-ray, ESR and sputum AFB smear. Results: Of the 224 patients who were studied, 81% patients were males. Addictions, including smoking and alcoholism, were prevalent in 136 patients (61%). Treatment success rate at six months was 94.2%. At the end of five years, 124 patients (57.9%) were symptomatic, 59% patients had radiological sequelae, relapse in 10 patients (4.5%), and mortality in 12 (5.4%) patients. Diabetes mellitus was the most common comorbid illness. Smoking and age > 45 years were associated with radiological sequelae. Smoking was significantly associated with mortality. Smokers had worse outcomes in all parameters. Conclusions: Relapse rate was 4.5% and overall mortality was 5.4% at the end of five years. Significant proportion of patients has radiological sequelae. Smoking was the preventable risk factor associated with sequelae, relapse and mortality.

Institution

(Lisha, James, Ravindran) Institute of Chest Diseases, Government Medical College, Kozhikode (Kerala), India

Publisher

Tuberculosis Association of India (3 Red Cross Road, New Delhi 110001, India)

Emtree Heading

acid fast bacterium; adolescent; adult; age distribution; aged; alcoholism; article; cause of death; clinical evaluation; comorbidity; controlled study; diabetes mellitus; disease association; erythrocyte sedimentation rate; female; follow up; human; *lung tuberculosis; major clinical study; male; outcome assessment; relapse; risk assessment; risk factor; sex difference; smoking habit; sputum smear; symptomatology; thorax radiography.

Link to the Ovid Full Text or citation:

Click here for full text options

216.

Emerging Adverse Cutaneous Drug Reactions.

Hagen J.W., Magro C.M., Crowson A.N.

Dermatologic Clinics. 30 (4) (pp 695-730), 2012. Date of Publication: October 2012.

AN: 2012573810

The past several decades have seen the advent and rapidly expanding use of biological agents in the treatment of chronic disease states. As increasingly large pools of patients have been enrolled in treatment protocols using these agents, physicians have become acquainted with both desired and adverse events associated with their use. Dermatologists frequently encounter patients affected by cutaneous drug reactions associated with the use of biological agents, thereby becoming familiar with the full range of side effects reported in the literature. This review discusses these adverse cutaneous effects, their underlying mechanisms, and efforts to predict and minimize their occurrence. © 2012 Elsevier Inc.

Institution

(Hagen) Tri-Institutional MD-PhD Program, Weill Medical College of Cornell University, New York, NY 10065, United States (Magro) Department of Pathology and Laboratory Medicine, Weill Medical College of Cornell University, New York, NY 10065, United States

(Crowson) Department of Dermatology, University of Oklahoma, Oklahoma, OK, United States

(Crowson) Department of Pathology, University of Oklahoma, Tulsa, OK, United States

(Crowson) Department of Surgery, University of Oklahoma, Tulsa, OK, United States

(Crowson) St John Medical Center, Regional Medical Laboratory, Tulsa, OK, United States

Publisher

W.B. Saunders (Independence Square West, Philadelphia PA 19106-3399, United States)

Emtree Heading

abscess/si [Side Effect]; acne/si [Side Effect]; acute disease/si [Side Effect]; acute generalized exanthematous pustulosis/si [Side Effect]; advanced cancer/dt [Drug Therapy]; alopecia areata/si [Side Effect]; anhidrosis/si [Side Effect]; B cell lymphoma/dt [Drug Therapy]; basal cell carcinoma/si [Side Effect]; breast cancer/dt [Drug Therapy]; bullous dermatitis/si [Side Effect]; capillary leak syndrome/si [Side Effect]; cellulitis/si [Side Effect]; chickenpox/si [Side Effect]; chronic disease/dt [Drug Therapy]; chronic drug administration; cold injury/si [Side Effect]; colorectal cancer/dt [Drug Therapy]; contact dermatitis/si [Side Effect]; Crohn disease/dt [Drug Therapy]; cutaneous T cell lymphoma/si [Side Effect]; dermatitis/si [Side Effect]; dermatitis herpetiformis/si [Side Effect]; dermatofibroma/si [Side Effect]; dermatomyositis/si [Side Effect]; diabetic neuropathy/dt [Drug Therapy]; disease association; DRESS syndrome/si [Side Effect]; drug approval; drug dose escalation; drug efficacy; drug eruption/si [Side Effect]; drug response; drug safety; drug tolerability; drug withdrawal; ectropion/si [Side Effect]; eczema/si [Side Effect]; erythema multiforme/dt [Drug Therapy]; erythema multiforme/si [Side Effect]; erythroderma/si [Side Effect]; eye disease/si [Side Effect]; eyelash trichomegaly/si [Side Effect]; face edema/si [Side Effect]; face erythema/si [Side Effect]; follicular lymphoma/si [Side Effect]; folliculitis/si [Side Effect]; genital herpes/si [Side Effect]; graft necrosis/si [Side Effect]; granuloma annulare/si [Side Effect]; guttate psoriasis/si [Side Effect]; head and neck carcinoma/dt [Drug Therapy]; herpes simplex/si [Side Effect]; herpes zoster/si [Side Effect]; human; impetigo/si [Side Effect]; infection/si [Side Effect]; infectious complication/si [Side Effect]; injection site edema/si [Side Effect]; injection site erythema/si [Side Effect]; injection site pain/si [Side Effect]; injection site pruritus/si [Side Effect]; injection site reaction/dt [Drug Therapy]; injection site reaction/si [Side Effect]; interstitial lung disease/si [Side Effect]; keratoacanthoma/si [Side Effect]; large cell lymphoma/dt [Drug Therapy]; large cell lymphoma/si [Side Effect]; leukocytoclastic vasculitis/si [Side Effect]; lichenoid/si [Side Effect]; lichenoid eruption/si [Side Effect]; lung tuberculosis/si [Side Effect]; lupus like syndrome/si [Side Effect]; lymphoproliferative disease/dt [Drug Therapy]; lymphoproliferative disease/si [Side Effect]; melanoma/si [Side Effect]; Merkel cell tumor/si [Side Effect]; metastasis/dt [Drug Therapy]; metastasis/si [Side Effect]; molluscum contagiosum/si [Side Effect]; mucinosis/si [Side Effect]; mucosa inflammation/si [Side Effect]; multiple sclerosis/dt [Drug Therapy]; mycosis fungoides/si [Side Effect]; neoplasm/dt [Drug Therapy]; nephrogenic systemic fibrosis/si [Side Effect]; neurodermatitis/si [Side Effect]; nevus/si [Side Effect]; nonhuman; nonmelanoma skin cancer/si [Side Effect]; nummular dermatitis/si [Side Effect]; onychomycosis/si [Side Effect]; outcome assessment; papular rash/si [Side Effect]; paronychia/si [Side Effect]; photosensitivity/si [Side Effect]; pityriasis rosea/si [Side Effect]; pityriasis rubra pilaris/si [Side Effect]; pneumonia/si [Side Effect]; pompholyx/si [Side Effect]; prediction; priority journal; pseudodermatomyositis; psoriasis/dt [Drug Therapy]; psoriasis/si [Side Effect]; psoriasis vulgaris/dt [Drug Therapy]; purpura; pustulosis palmoplantaris/si [Side Effect]; radiation dermatitis/si [Side Effect]; recurrent cancer/dt [Drug Therapy]; retiform purpura; retina macula degeneration/dt [Drug Therapy]; review; rheumatoid arthritis/dt [Drug Therapy]; risk assessment; risk factor; rosacea/si [Side Effect]; sarcoidosis/si [Side Effect]; seborrheic keratosis/si [Side Effect]; Sezary syndrome/si [Side Effect]; skin allergy/si [Side Effect]; skin cancer/si [Side Effect]; skin candidiasis/si [Side Effect]; *skin disease/si [Side Effect]; skin infection/si [Side Effect]; skin lupus erythematosus/si [Side Effect]; skin lymphoma/si [Side Effect]; skin manifestation/si [Side Effect]; skin tumor/si [Side Effect]; squamous cell carcinoma/dt [Drug Therapy]; squamous cell carcinoma/si [Side Effect]; Stevens Johnson syndrome/si [Side Effect]; systemic disease/si [Side Effect]; systemic lupus erythematosus/si [Side Effect]; T cell lymphoma/dt [Drug Therapy]; T cell lymphoma/si [Side Effect]; tinea corporis/si [Side Effect]; tinea versicolor/si [Side Effect]; toxic epidermal necrolysis/si [Side Effect]; ulcer; upper respiratory tract infection/si [Side Effect]; urticaria/si [Side Effect]; verruca vulgaris/si [Side Effect]; vesicular rash/si [Side Effect]; virus infection/si [Side Effect]; Wells syndrome/si [Side Effect]; wound healing impairment/si [Side Effect]; xeroderma pigmentosum/si [Side Effect]; adalimumab/ae [Adverse Drug Reaction]; adalimumab/dt [Drug Therapy]; alefacept/ae [Adverse Drug Reaction]; alefacept/cm [Drug Comparison]; alefacept/dt [Drug Therapy]; alemtuzumab/ae [Adverse Drug Reaction]; alemtuzumab/dt [Drug Therapy]; basiliximab/ae [Adverse Drug Reaction]; bevacizumab/ae [Adverse Drug Reaction]; bevacizumab/cm [Drug Comparison]; bevacizumab/dt [Drug Therapy]; brentuximab vedotin/ae [Adverse Drug Reaction]; brentuximab vedotin/dt [Drug Therapy]; cetuximab/ae [Adverse Drug Reaction]; cetuximab/dt [Drug Therapy]; corticosteroid/dt [Drug Therapy]; cyclosporin/ae [Adverse Drug Reaction]; daclizumab; denileukin diftitox/ae [Adverse Drug Reaction]; denileukin diftitox/dt [Drug Therapy]; disease modifying antirheumatic drug/ae [Adverse Drug Reaction]; disease modifying antirheumatic drug/cm [Drug Comparison]; efalizumab/ae [Adverse Drug Reaction]; efalizumab/ct [Clinical Trial]; efalizumab/dt [Drug Therapy]; etanercept/ae [Adverse Drug Reaction]; etanercept/cm [Drug Comparison]; etanercept/dt [Drug Therapy]; ibritumomab tiuxetan/ae [Adverse Drug Reaction]; ibritumomab tiuxetan/cb [Drug Combination]; infliximab/ae [Adverse Drug Reaction]; infliximab/dt [Drug Therapy]; methotrexate/ae [Adverse Drug Reaction]; methotrexate/cb [Drug Combination]; methotrexate/dt [Drug Therapy]; monoclonal antibody/ae [Adverse Drug Reaction]; natalizumab/ae [Adverse Drug Reaction]; natalizumab/dt [Drug Therapy]; paclitaxel/ae [Adverse Drug Reaction]; paclitaxel/dt [Drug Therapy]; panitumumab/ae [Adverse Drug Reaction]; panitumumab/dt [Drug Therapy]; placebo; prednisone/ae [Adverse Drug Reaction]; prednisone/dt [Drug Therapy]; ranibizumab/ae [Adverse Drug Reaction]; ranibizumab/cm [Drug Comparison]; ranibizumab/dt [Drug Therapy]; ranibizumab/vi [Intravitreal Drug Administration]; rituximab/ae [Adverse Drug Reaction]; rituximab/cb [Drug Combination]; rituximab/dt [Drug Therapy]; steroid/dt [Drug Therapy]; steroid/po [Oral Drug Administration]; steroid/tp [Topical Drug Administration]; taxane derivative/ae [Adverse Drug Reaction]; taxane derivative/cb [Drug Combination]; trastuzumab/ae [Adverse Drug Reaction]; trastuzumab/cb [Drug Combination]; trastuzumab/dt [Drug Therapy]; tumor necrosis factor alpha antibody/ae [Adverse Drug Reaction]; tumor necrosis factor alpha antibody/cm [Drug Comparison]; tumor necrosis factor alpha inhibitor/ae [Adverse Drug Reaction]; tumor necrosis factor alpha inhibitor/cb [Drug Combination]; tumor necrosis factor alpha inhibitor/cm [Drug Comparison]; tumor necrosis factor alpha inhibitor/dt [Drug Therapy]; unindexed drug.

Drug Trade Names and Manufacturers

adcetris, amevive, avastin, erbitux, herceptin, lucentis, ontak, raptiva, rituxan, simulect, tysabri, vectibix, zenapax

Link to the Ovid Full Text or citation:

Click here for full text options

217.

The role of diabetes co-morbidity for tuberculosis treatment outcomes: a prospective cohort study from Mwanza, Tanzania.

Faurholt-Jepsen D., Range N., Praygod G., Kidola J., Faurholt-Jepsen M., Aabye M.G., Changalucha J., Christensen D.L., Martinussen T., Krarup H., Witte D.R., Andersen T.B., Friis H.

BMC Infectious Diseases. 12 (no pagination), 2012. Article Number: 165. Date of Publication: 27 Jul 2012.

AN: 2012579890

Background: Due to the association between diabetes and pulmonary tuberculosis (TB), diabetes may threaten the control of TB. In a prospective cohort study nested in a nutrition trial, we investigated the role of diabetes on changes in anthropometry, grip strength, and clinical parameters over a five months follow-up period.Methods: Among pulmonary TB patients with known diabetes status, we assessed anthropometry and clinical parameters (e.g. haemoglobin) at baseline and after two and five months of TB treatment. A linear mixed-effects model (repeated measurements) was used to investigate the role of diabetes during recovery.Results: Of 1205 TB patients, the mean (standard deviation) age was 36.6 (13.0) years, 40.9% were females, 48.9% were HIV co-infected, and 16.3% had diabetes. TB patients with diabetes co-morbidity experienced a lower weight gain at two (1.3 kg, CI95% 0.5; 2.0, p = 0.001) and five months (1.0 kg, CI95% 0.3; 1.7, p = 0.007). Similarly, the increase in the level of haemoglobin was lower among TB patients with diabetes co-morbidity after two (DELTA 0.6 g/dL, CI95% 0.3; 0.9 p < 0.001) and five months (DELTA 0.5 g/dL, CI95% 0.2; 0.9 p = 0.004) of TB treatment, respectively.Conclusion: TB patients initiating TB treatment with diabetes co-morbidity experience delayed recovery of body mass and haemoglobin, which are important for the functional recovery from disease. © 2012 Faurholt-Jepsen et al.; licensee BioMed Central Ltd.

Institution

(Faurholt-Jepsen, Faurholt-Jepsen, Friis) Department of Human Nutrition, University of Copenhagen, Frederiksberg, Denmark (Range) Muhimbili Medical Centre, National Institute of Medical Research, Dar Es Salaam, Tanzania

(Praygod, Kidola, Changalucha) Mwanza Medical Centre, National Institute of Medical Research, Mwanza, Tanzania

(Aabye) Clinical Research Centre, University of Copenhagen, Hvidovre Hospital, Hvidovre, Denmark

(Christensen) Department of International Health, University of Copenhagen, Copenhagen, Denmark

(Christensen, Witte) Steno Diabetes Center, Gentofte, Denmark

(Martinussen) Department of Basic Sciences and Environment, University of Copenhagen, Frederiksberg, Denmark

(Krarup) Department of Clinical Biochemistry, Aalborg University Hospital, Aalborg, Denmark

(Andersen) Department of Infectious Diseases, Odense University Hospital, Odense, Denmark

Publisher

BioMed Central Ltd. (Floor 6, 236 Gray's Inn Road, London WC1X 8HB, United Kingdom)

Emtree Heading

adult; arm circumference; article; body mass; CD4 lymphocyte count; cohort analysis; comorbidity; controlled study; *diabetes mellitus; female; follow up; grip strength; human; leukocyte count; *lung tuberculosis/dt [Drug Therapy]; major clinical study; male; mixed infection; prospective study; skinfold thickness; Tanzania; treatment outcome; waist circumference; weight gain; hemoglobin/ec [Endogenous Compound]; tuberculostatic agent/dt [Drug Therapy].

Link to the Ovid Full Text or citation:

Click here for full text options

218.

Clinical and temporal patterns of severe pneumonia causing critical illness during Hajj.

Mandourah Y., Al-Radi A., Ocheltree A.H., Ocheltree S.R., Fowler R.A.

BMC Infectious Diseases. 12 (no pagination), 2012. Article Number: 117. Date of Publication: 16 May 2012.

AN: 2012573403

Background: Pneumonia is a leading cause of hospitalization during Hajj and susceptibility and transmission may be exacerbated by extreme spatial and temporal crowding. We describe the number and temporal onset, co-morbidities, and outcomes of severe pneumonia causing critical illness among pilgrims.Method: A cohort study of all critically ill Hajj patients, of over 40 nationalities, admitted to 15 hospitals in 2 cities in 2009 and 2010. Demographic, clinical, and laboratory data, and variables necessary for calculation of the Acute Physiology and Chronic Health Evaluation IV scores were collected.Results: There were 452 patients (64.6% male) who developed critical illness. Pneumonia was the primary cause of critical illness in 123 (27.2%) of all intensive care unit (ICU) admissions during Hajj. Pneumonia was community (Hajj)-acquired in 66.7%, aspiration-related in 25.2%, nosocomial in 3.3%, and tuberculous in 4.9%. Pneumonia occurred most commonly in the second week of Hajj, 95 (77.2%) occurred between days 5-15 of Hajj, corresponding to the period of most extreme pilgrim density. Mechanical ventilation was performed in 69.1%. Median duration of ICU stay was 4 (interquartile range [IQR] 1-8) days and duration of ventilation 4 (IQR 3-6) days. Commonest preexisting co-morbidities included smoking (22.8%), diabetes (32.5%), and COPD (17.1%). Short-term mortality (during the 3-week period of Hajj) was 19.5%.Conclusion: Pneumonia is a major cause of critical illness during Hajj and occurs amidst substantial crowding and pilgrim density. Increased efforts at prevention for at risk pilgrim prior to Hajj and further attention to spatial and physical crowding during Hajj may attenuate this risk. © 2012 Mandourah et al.; licensee BioMed Central Ltd.

Institution

(Mandourah) Department of Intensive Care, Riyadh Military Hospital, P.O. Box 7897, 11159, Riyadh, Saudi Arabia (Al-Radi) Department of Oncology, Oncology Center at King Abdul Aziz Hospital and Oncology Center, Jeddah, Saudi Arabia

(Ocheltree) Department of Internal Medicine, North West Armed Forces Hospital, Tabuk, Saudi Arabia

(Ocheltree) Department of Intensive Care, King Abdul-Aziz Hospital and Oncology Center, Jeddah, Saudi Arabia

(Fowler) Department of Critical Care Medicine, Department of Medicine Sunnybrook Hospital, University of Toronto, Toronto, Canada

Publisher

BioMed Central Ltd. (Floor 6, 236 Gray's Inn Road, London WC1X 8HB, United Kingdom)

Emtree Heading

adult; aged; article; artificial ventilation; aspiration pneumonia; community acquired pneumonia; comorbidity; controlled study; *critical illness/co [Complication]; diabetes mellitus; disease association; disease severity; female; hospital admission; hospital infection; human; intensive care unit; length of stay; major clinical study; male; mortality; outcome assessment; *pneumonia/th [Therapy]; *religion; smoking; treatment duration; tuberculosis.

Link to the Ovid Full Text or citation:

Click here for full text options

219.

When TB associates with diabetes.

Perez-Guzman C., Serna-Vela F.J., Vargas M.H.

Expert Review of Anti-Infective Therapy. 10 (7) (pp 727-728), 2012. Date of Publication: July 2012.

AN: 2012520518

Institution

(Perez-Guzman, Serna-Vela) Research Unit, State Health Ministry of Aguascalientes, Aguascalientes, Mexico (Vargas) Research Unit, National Institute of Respiratory Diseases, Mexico

(Vargas) Medical Research Unit in Respiratory Diseases, Mexican Institute of Social Security, Mexico City, Mexico

Publisher

Expert Reviews Ltd. (2 Albert Place, London N3 1QB, United Kingdom)

Emtree Heading

Arabian; *diabetes mellitus/pc [Prevention]; diabetic patient; disease association; drug absorption; drug bioavailability; drug elimination; drug metabolism; editorial; ethnic group; glucose blood level; human; hyperglycemia; *lung tuberculosis/co [Complication]; *lung tuberculosis/dt [Drug Therapy]; metabolic regulation; risk factor; treatment failure; treatment outcome; urinary tract infection; ethambutol/dt [Drug Therapy]; ethambutol/pk [Pharmacokinetics]; glucose/ec [Endogenous Compound]; pyrazinamide/dt [Drug Therapy]; pyrazinamide/pk [Pharmacokinetics]; rifampicin/dt [Drug Therapy]; rifampicin/pk [Pharmacokinetics]; tuberculostatic agent/dt [Drug Therapy]; tuberculostatic agent/pk [Pharmacokinetics].

Link to the Ovid Full Text or citation:

Click here for full text options

220.

Predictors of tuberculosis treatment outcomes. <Preditores dos desfechos do tratamento da tuberculose.>

Orofino R.L., do Brasil P.E.A., Trajman A., Schmaltz C.A.S., Dalcolmo M., Rolla V.C.

Jornal Brasileiro de Pneumologia. 38 (1) (pp 88-97), 2012. Date of Publication: January/February 2012.

AN: 22407045

Objective: To analyze tuberculosis treatment outcomes and their predictors. Methods: This was a retrospective longitudinal cohort study involving tuberculosis patients treated between 2004 and 2006 at the Instituto de Pesquisa Evandro Chagas, in the city of Rio de Janeiro. We estimated adjusted risk ratios (ARRs) for the predictors of treatment outcomes. Results: Among 311 patients evaluated, the rates of cure, treatment abandonment, treatment failure, and mortality were 72%, 19%, 2%, and 6%, respectively. Changes in the treatment regimen due to adverse events occurred in 8%. The factors found to reduce the probability of cure were alcoholism (ARR, 0.30), use of the streptomycin+ethambutol+ofloxacin (SEO) regimen (ARR, 0.32), HIV infection without the use of antiretroviral therapy (ART; ARR, 0.36), and use of the rifampin+isoniazid+pyrazinamide+ethambutol regimen (ARR, 0.58). Being younger and being alcoholic both increased the probability of abandonment (ARR, 3.84 and 1.76, respectively). It was impossible to determine the ARR for the remaining outcomes due to their low prevalence. However, using the relative risk (RR), we identified the following potential predictors of mortality: use of the SEO regimen (RR, 11.43); HIV infection without ART (RR, 9.64); disseminated tuberculosis (RR, 9.09); lack of bacteriological confirmation (RR, 4.00); diabetes mellitus (RR, 3.94); and homosexual/bisexual behavior (RR, 2.97). Low income was a potential predictor of treatment failure (RR, 11.70), whereas disseminated tuberculosis and HIV infection with ART were potential predictors of changes in the regimen due to adverse events (RR, 3.57 and 2.46, respectively). Conclusions: The SEO regimen should not be used for extended periods. The data confirm the importance of ART and suggest the need to use it early.

Institution

(Orofino, Schmaltz, Rolla) Laboratory for Clinical Research on Mycobacterial Disease, Evandro Chagas Clinical Research Institute, Oswaldo Cruz Foundation, Rio de Janeiro, Brazil (do Brasil) Evandro Chagas Clinical Research Institute, Oswaldo Cruz Foundation, Rio de Janeiro, Brazil

(Trajman) Gama Filho University, Rio de Janeiro, Brazil

(Trajman) McGill University, Montreal, Canada

(Dalcolmo) Outpatient Clinic, Helio Fraga Referral Center for Tuberculosis, Oswaldo Cruz Foundation, Rio de Janeiro, Brazil

Publisher

Sociedade Brasileira de Pneumologia e Tisiologia (SEPS 714/914, Bloco E, Sala 116, Brasilia 70390-145, Brazil)

Emtree Heading

adult; article; classification; drug combination/ae [Adverse Drug Reaction]; epidemiology; female; highly active antiretroviral therapy/ae [Adverse Drug Reaction]; human; Human immunodeficiency virus infection/co [Complication]; Human immunodeficiency virus infection/dt [Drug Therapy]; *lung tuberculosis/dt [Drug Therapy]; male; mortality; *patient; patient compliance; socioeconomics; statistics; treatment failure; treatment outcome; *ethambutol/ae [Adverse Drug Reaction]; *ofloxacin/ae [Adverse Drug Reaction]; *streptomycin/ae [Adverse Drug Reaction]; *tuberculostatic agent/ae [Adverse Drug Reaction].

Link to the Ovid Full Text or citation:

Click here for full text options

221.

Cohort monitoring of persons with hypertension: An illustrated example from a primary healthcare clinic for Palestine refugees in Jordan.

Khader A., Farajallah L., Shahin Y., Hababeh M., Abu-Zayed I., Kochi A., Harries A.D., Zachariah R., Kapur A., Venter W., Seita A.

Tropical Medicine and International Health. 17 (9) (pp 1163-1170), 2012. Date of Publication: September 2012.

AN: 2012510847

Objective Recording and reporting systems borrowed from the DOTS framework for tuberculosis control can be used to record, monitor and report on chronic disease. In a primary healthcare clinic run by UNRWA in Amman, Jordan, serving Palestine refugees with hypertension, we set out to illustrate the method of cohort reporting for persons with hypertension by presenting on quarterly and cumulative case finding, cumulative and 12-month analysis of cohort outcomes and to assess how these data may inform and improve the quality of hypertension care services. Method This was a descriptive study using routine programme data collected through E-Health. Results There were 97 newly registered patients with hypertension in quarter 1, 2012, and a total of 4130 patients with hypertension ever registered since E-Health started in October 2009. By 31 March 2012, 3119 (76%) of 4130 patients were retained in care, 878 (21%) had failed to present to a healthcare worker in the last 3months and the remainder had died, transferred out or were lost to follow-up. Cumulative and 12-month cohort outcome analysis indicated deficiencies in several components of clinical performance related to blood pressure measurements and fasting blood glucose tests to screen simultaneously for diabetes. Between 8% and 15% of patients with HT had serious complications such as cardiovascular disease and stroke. Conclusion Cohort analysis is a valuable tool for the monitoring and management of non-communicable chronic diseases such as HT. © 2012 Blackwell Publishing Ltd.

Institution

(Khader, Farajallah, Shahin, Hababeh, Venter, Seita) United Nations Relief and Works Agency for Palestine Refugees in the Near East, UNRWA HQ (A), Amman, Jordan (Abu-Zayed) Field Health Programme, UNRWA, Amman, Jordan

(Kochi) Tokyo University Medical School, Tokyo, Japan

(Harries) International Union Against Tuberculosis and Lung Disease, Paris, France

(Harries) London School of Hygiene and Tropical Medicine, London, United Kingdom

(Zachariah) Medecins sans Frontieres, Medical Department, Operational Research Unit, Brussels Operational Center, Luxembourg, Luxembourg

(Kapur) World Diabetes Foundation, Gentofte, Denmark

Publisher

Blackwell Publishing Ltd (9600 Garsington Road, Oxford OX4 2XG, United Kingdom)

Emtree Heading

adult; article; blood pressure measurement; cardiovascular disease/co [Complication]; cohort analysis; descriptive research; diabetes mellitus; female; follow up; glucose blood level; health care quality; human; *hypertension/dt [Drug Therapy]; Jordan; major clinical study; male; mortality; outcomes research; Palestine; patient monitoring; primary health care; refugee; retrospective study; stroke/co [Complication]; amiloride/dt [Drug Therapy]; atenolol/dt [Drug Therapy]; diltiazem/dt [Drug Therapy]; enalapril/dt [Drug Therapy]; furosemide/dt [Drug Therapy]; glucose/ec [Endogenous Compound]; hydrochlorothiazide/dt [Drug Therapy]; methyldopa/dt [Drug Therapy]; nifedipine/dt [Drug Therapy]; propranolol/dt [Drug Therapy].

Link to the Ovid Full Text or citation:

Click here for full text options

222.

Non-healing arm wound with a discharging sinus in an elderly patient with diabetes.

Bansal S., Jindal S., Biswas R.

BMJ case reports. 2010 (no pagination), 2010. Date of Publication: 2010.

AN: 22767481

A 48-year-old Filipino female presented with unilateral acute onset painful red eye, blurred vision and yellow discharge. On examination she had a corrected visual acuity of 6/5 in the right eye and 6/18 in the left eye. There was a left-sided periorbital swelling, with chemosis involving the bulbar conjunctiva on the temporal aspect. Ocular motility showed limitation of left-sided abduction with mild limitation of laevoelevation and laevodepression. She was afebrile and systemic examination was unremarkable. Medical history included diagnosis of Crohn's disease since the age of 20. She was on oral mesalamine 1 g for mildly active colitis. Full blood count was normal but erythrocyte sedimentation rate and C reactive protein were raised. Blood culture and conjunctival swab were negative. Contrast-enhanced CT scan demonstrated enlargement of the lacrimal gland. She was managed conservatively with acetaminophen and codeine for pain and swelling. She recovered completely in 2 weeks with no sequelae.

Institution

(Bansal) Department of Medicine, People's College of Medical Sciences and Research Centre, Bhopal, India.

Emtree Heading

aged; arm; article; case report; *Crohn disease/co [Complication]; *Crohn disease/di [Diagnosis]; *Crohn disease/dt [Drug Therapy]; *diabetes mellitus/di [Diagnosis]; *diabetes mellitus/dt [Drug Therapy]; follow up; human; lacrimal apparatus; *lacrimal gland disease/di [Diagnosis]; *lacrimal gland disease/th [Therapy]; male; methodology; pathophysiology; radiography; risk assessment; shoulder; *skin ulcer/di [Diagnosis]; *skin ulcer/th [Therapy]; treatment outcome; *tuberculous arthritis/di [Diagnosis]; *tuberculous arthritis/dt [Drug Therapy]; wound drainage; wound healing; antiinfective agent/dt [Drug Therapy]; tuberculostatic agent/dt [Drug Therapy].

Link to the Ovid Full Text or citation:

Click here for full text options

223.

Gender differences in the prevalence of tuberculous lymphadenitis at the state of penang, Malaysia: Findings from a cross-sectional study.

Khan A.H., Sulaiman S.A.S., Muttalif A.R., Hassali M.A., Abdullah R.

Archives of Pharmacy Practice. 1 (1) (pp 7-11), 2010. Date of Publication: 2010.

AN: 2012184531

Objective: To evaluate the prevalence of tuberculous lymphadenitis on the basis of gender in the northern state of Malaysia. Materials and Methods: Current study was divided into two phases (Retrospective and Prospective), which was conducted at the Department of Chest in General Hospital Penang. Patient records were reviewed retrospectively, and prospectively to identify patients with confirmed diagnosis of tuberculous lymphadenitis under the clinical signs and symptoms, and laboratory tests from January 2006 to December 2008. Data were analyzed using SPSS version 16. Results: One hundred and nine patients (7.2%) with confirmed tuberculosis lymphadenitis cases were found, among 1516 of total TB cases. The mean age of the patients was 36.4+/-12.87. The male to female ratio was 1.4:1. Ethnically, 45 (41.3%) were Malay followed by Chinese 37 (33.9%). Among the risk factors were, HIV and diabetes mellitus, 17 (15.6%) and 11(10.0%) respectively. Cough and fever were the most common symptoms in both gender groups. Night sweat, cough, shortness of breathing were observed more in male than female, while swelling of lymph nodes, loss of appetite, loss of weight, haemoptysis, and chest pain were higher in female. In the majority, 90 (82.5%) positive results were obtained by fine needle aspiration (FNA) cytology. Overall sixty two (56.9%) patients were successfully treated, 5 (4.6%) patients died during the treatment, while male were higher in successfully treated (59.7%). Conclusion: Incidence of TB Lymphadenitis was found higher among Malay men. Diabetes mellitus and HIV are the most commonly reported risk factors and co-morbidities playing an important role in treatment success while, 12 months treatment were observed among 10 (9.2%) of cases. Treatment successful rate were higher in male as compare to female.

Institution

(Khan, Sulaiman) Department of Clinical Pharmacy, School of Pharmaceutical Sciences, Universiti Sains Malaysia, 11800 Penang, Malaysia (Muttalif) Department of Respiratory Medicine, Penang General Hospital, Penang, Malaysia

(Hassali) Discipline of Social and Administrative Pharmacy, School of Pharmaceutical Sciences, University Sains Malaysia, 11800 Penang, Malaysia

(Abdullah) Hospital Pharmacists, Umum Hospital Sarawak, Malaysia

Publisher

Archives of Global Professionals (Regent Court, Sheffield S1 4DA, United Kingdom)

Emtree Heading

acid fast bacterium; adult; alcohol consumption; anorexia; article; aspiration biopsy; comorbidity; controlled study; coughing; cross-sectional study; diabetes mellitus; dyspnea; ethnicity; female; fever; hemoptysis; human; Human immunodeficiency virus infection; laboratory test; lymphadenopathy; major clinical study; Malaysia; male; medical record review; night sweat; outcome assessment; physical examination; prevalence; risk factor; *sex difference; smoking; sputum culture; thorax pain; tuberculin test; *tuberculous lymphadenitis/di [Diagnosis]; *tuberculous lymphadenitis/ep [Epidemiology]; weight reduction.

Link to the Ovid Full Text or citation:

Click here for full text options

224.

How and when to use biologics in psoriasis.

Kircik L.H., Gonzalez M.E.

Journal of Drugs in Dermatology. 9 (8) (pp s106-s117), 2010. Date of Publication: August 2010.

AN: 2011525801

Institution

(Kircik) Mount Sinai Medical Center, New York, NY, United States (Kircik) Indiana University School of Medicine, Indianapolis, IN, United States

(Gonzalez) Ronald O. Perelman Department of Dermatology, New York University School of Medicine, New York, NY, United States

Publisher

Journal of Drugs in Dermatology (377 Park Avenue South, New York NY 10016, United States)

Emtree Heading

alanine aminotransferase blood level; anaphylaxis/si [Side Effect]; ankylosing spondylitis/dt [Drug Therapy]; antibody blood level; antibody production; antibody response; antibody titer; apoptosis; arthralgia; aspartate aminotransferase blood level; *biological therapy; blood cell count; breast cancer; cardiovascular disease/si [Side Effect]; CD4 lymphocyte count; cell proliferation; chest tightness/si [Side Effect]; childhood disease; clinical assessment; conference paper; congestive heart failure; consultation; Crohn disease/dt [Drug Therapy]; cytokine release; diabetes mellitus; disability; disease course; disease exacerbation/si [Side Effect]; disease severity; drug approval; drug blood level; drug clearance; drug efficacy; drug indication; drug mechanism; drug safety; dyspnea/si [Side Effect]; family history; fatigue/si [Side Effect]; flushing; follow up; headache/si [Side Effect]; hepatitis B; hospitalization; human; Human immunodeficiency virus infection; hypertension/si [Side Effect]; hypotension/si [Side Effect]; infection/si [Side Effect]; infection risk; infusion; injection site pruritus/si [Side Effect]; injection site reaction/si [Side Effect]; joint swelling; liver failure; liver function test; liver toxicity/si [Side Effect]; loading drug dose; long term care; lupus erythematosus/si [Side Effect]; lymphocyte count; lymphoproliferative disease; morning stiffness; mortality; multiple sclerosis/si [Side Effect]; Mycobacterium tuberculosis; nausea/si [Side Effect]; neurologic disease; non melanoma skin cancer; opportunistic infection; outcome assessment; pathophysiology; patient monitoring; phototherapy; plaque type psoriasis/dt [Drug Therapy]; posterior reversible encephalopathy syndrome/si [Side Effect]; postmarketing surveillance; practice guideline; pregnancy; *psoriasis/dt [Drug Therapy]; psoriatic arthritis/dt [Drug Therapy]; PUVA; quality of life; randomized controlled trial (topic); receptor binding; recurrence risk; rheumatoid arthritis/dt [Drug Therapy]; rhinopharyngitis/si [Side Effect]; risk assessment; risk benefit analysis; screening; serodiagnosis; serology; side effect/si [Side Effect]; skin cancer; skin examination; sweating; T lymphocyte activation; Th1 cell; Th17 cell; treatment duration; treatment indication; treatment response; tuberculin test; tuberculosis/si [Side Effect]; ulcerative colitis/dt [Drug Therapy]; unspecified side effect/si [Side Effect]; upper respiratory tract infection/si [Side Effect]; vaccination; virus reactivation; X ray; *adalimumab/ae [Adverse Drug Reaction]; *adalimumab/ct [Clinical Trial]; *adalimumab/do [Drug Dose]; *adalimumab/dt [Drug Therapy]; *adalimumab/pd [Pharmacology]; *adalimumab/sc [Subcutaneous Drug Administration]; alanine aminotransferase/ec [Endogenous Compound]; *alefacept/ae [Adverse Drug Reaction]; *alefacept/cb [Drug Combination]; *alefacept/dt [Drug Therapy]; *alefacept/im [Intramuscular Drug Administration]; *alefacept/pd [Pharmacology]; antihistaminic agent; aspartate aminotransferase/ec [Endogenous Compound]; briakinumab/ae [Adverse Drug Reaction]; briakinumab/ct [Clinical Trial]; briakinumab/dt [Drug Therapy]; CD45RO antigen; drug antibody; *etanercept/ae [Adverse Drug Reaction]; *etanercept/ct [Clinical Trial]; *etanercept/cb [Drug Combination]; *etanercept/cm [Drug Comparison]; *etanercept/dt [Drug Therapy]; *etanercept/pd [Pharmacology]; *etanercept/sc [Subcutaneous Drug Administration]; etretin/cb [Drug Combination]; etretin/dt [Drug Therapy]; hepatitis B core antibody/ec [Endogenous Compound]; immunoglobulin enhancer binding protein; *infliximab/ae [Adverse Drug Reaction]; *infliximab/ct [Clinical Trial]; *infliximab/dt [Drug Therapy]; *infliximab/iv [Intravenous Drug Administration]; *infliximab/pd [Pharmacology]; interleukin 12/ec [Endogenous Compound]; interleukin 17/ec [Endogenous Compound]; interleukin 23/ec [Endogenous Compound]; live vaccine; methotrexate/cb [Drug Combination]; methotrexate/dt [Drug Therapy]; neutralizing antibody; paracetamol; placebo; protein p55/ec [Endogenous Compound]; protein p75/ec [Endogenous Compound]; remicaide; tumor necrosis factor alpha/ec [Endogenous Compound]; tumor necrosis factor alpha receptor/ec [Endogenous Compound]; unclassified drug; *ustekinumab/ae [Adverse Drug Reaction]; *ustekinumab/ct [Clinical Trial]; *ustekinumab/cm [Drug Comparison]; *ustekinumab/dt [Drug Therapy]; *ustekinumab/pk [Pharmacokinetics]; *ustekinumab/pd [Pharmacology].

Drug Trade Names and Manufacturers

Abbott, Amgen [United States], Biogen, Centocor, abt 874, amevive: Biogen, cnto 1275: Centocor, enbrel: Amgen [United States], humira: Abbott, remicaide: Centocor

Link to the Ovid Full Text or citation:

Click here for full text options

225.

Implications of the global increase of diabetes for tuberculosis control and patient care.

Ruslami R., Aarnoutse R.E., Alisjahbana B., Van Der Ven A.J.A.M., Van Crevel R.

Tropical Medicine and International Health. 15 (11) (pp 1289-1299), 2010. Date of Publication: November 2010.

AN: 2011057422

Objectives To review the current knowledge about tuberculosis (TB) and diabetes, assessing the implication of the global increase of diabetes for TB control and patient care. Methods Systematic literature review. Results Using public databases, it can be estimated that 12.6% (95% CI 9.2-17.3%) of new TB cases in the 10 countries with the highest TB burden will be attributable to TB in 2030, a relative increase of 25.5% compared to 2010. Diabetes is associated with a higher age and body weight among patients with TB, but probably not with a specific clinical presentation of TB. Rifampicin hampers glycemic control by increasing the metabolism of most oral antidiabetic drugs, while diabetes patients may have lower concentrations of anti-TB drugs. This might be one factor contributing to higher TB treatment failure rates. Conclusions The global epidemic of diabetes has implications for control and treatment of TB. Prospective studies are needed to improve prevention, early detection and treatment of concomitant diabetes and TB, especially in developing countries. © 2010 Blackwell Publishing Ltd.

Institution

(Ruslami) Department of Pharmacology, Faculty of Medicine, Universitas Padjadjaran, Hasan Sadikin Hospital, Bandung, Indonesia (Ruslami, Alisjahbana) Health Research Unit, Faculty of Medicine - Universitas Padjadjaran, Hasan Sadikin Hospital, Bandung, Indonesia

(Aarnoutse) Department of Clinical Pharmacy, Radboud University Nijmegen Medical Centre, Nijmegen, Netherlands

(Alisjahbana) Department of Internal Medicine, Faculty of Medicine, Univerisitas Padjadjaran /Hasan Sadikin Hospital, Bandung, Indonesia

(Van Der Ven, Van Crevel) Department of Internal Medicine, Radboud University Nijmegen Medical Centre, Nijmegen, Netherlands

Publisher

Blackwell Publishing Ltd (9600 Garsington Road, Oxford OX4 2XG, United Kingdom)

Emtree Heading

age; article; Bangladesh; body weight; China; Congo; data base; *diabetes mellitus/dt [Drug Therapy]; disease association; drug metabolism; Ethiopia; glycemic control; human; India; Indonesia; knowledge; multicenter study; Mycobacterium tuberculosis; Nigeria; non insulin dependent diabetes mellitus/dt [Drug Therapy]; Pakistan; *patient care; patient compliance; Philippines; risk factor; South Africa; sputum culture; systematic review; treatment failure; *tuberculosis/dt [Drug Therapy]; *tuberculosis control; antidiabetic agent/dt [Drug Therapy]; metformin/dt [Drug Therapy]; nateglinide/dt [Drug Therapy]; pioglitazone/dt [Drug Therapy]; repaglinide/dt [Drug Therapy]; rifampicin/dt [Drug Therapy]; rifampicin/pk [Pharmacokinetics]; rosiglitazone/dt [Drug Therapy]; sulfonylurea derivative/dt [Drug Therapy].

Link to the Ovid Full Text or citation:

Click here for full text options

226.

Opioid pharmacotherapy for chronic non-cancer pain in the United States: A research guideline for developing an evidence-base.

Chapman C.R., Lipschitz D.L., Angst M.S., Chou R., Denisco R.C., Donaldson G.W., Fine P.G., Foley K.M., Gallagher R.M., Gilson A.M., Haddox J.D., Horn S.D., Inturrisi C.E., Jick S.S., Lipman A.G., Loeser J.D., Noble M., Porter L., Rowbotham M.C., Schoelles K.M., Turk D.C., Volinn E., Von Korff M.R., Webster L.R., Weisner C.M.

Journal of Pain. 11 (9) (pp 807-829), 2010. Date of Publication: September 2010.

AN: 2010471692

This document reports the consensus of an interdisciplinary panel of research and clinical experts charged with reviewing the use of opioids for chronic noncancer pain (CNCP) and formulating guidelines for future research. Prescribing opioids for chronic noncancer pain has recently escalated in the United States. Contrasting with increasing opioid use are: 1) The lack of evidence supporting long-term effectiveness; 2) Escalating misuse of prescription opioids including abuse and diversion; and 3) Uncertainty about the incidence and clinical salience of multiple, poorly characterized adverse drug events (ADEs) including endocrine dysfunction, immunosuppression and infectious disease, opioid-induced hyperalgesia and xerostomia, overdose, falls and fractures, and psychosocial complications. Chief among the limitations of current evidence are: 1) Sparse evidence on long-term opioid effectiveness in chronic pain patients due to the short-term time frame of clinical trials; 2) Insufficiently comprehensive outcome assessment; and 3) Incomplete identification and quantification of ADEs. The panel called for a strategic interdisciplinary approach to the problem domain in which basic scientists and clinicians cooperate to resolve urgent issues and generate a comprehensive evidence base. It offered 4 recommendations in 3 areas: 1) A research strategy for studying the effectiveness of long-term opioid pharmacotherapy; 2) Improvements in evidence-generation methodology; and 3) Potential research topics for generating new evidence. Perspective: Prescribing opioids for CNCP has outpaced the growth of scientific evidence bearing on the benefits and harms of these interventions. The need for a strong evidence base is urgent. This guideline offers a strategic approach to creating a comprehensive evidence base to guide safe and effective management of CNCP. © 2010 by the American Pain Society.

Institution

(Chapman, Lipschitz, Donaldson, Fine, Volinn) Department of Anesthesiology, University of Utah School of Medicine, Salt Lake City, UT, United States (Angst) Department of Anesthesiology, Stanford University, Palo Alto, CA, United States

(Chou) Internal Medicine and Geriatrics, School of Medicine, Oregon Health and Science University, Portland, OR, United States

(Denisco) National Institute on Drug Abuse, Division of Epidemiology, Services and Prevention Research, NIH, Bethesda, MD, United States

(Foley, Inturrisi) Pain and Palliative Care Service, Memorial Sloan Kettering Cancer Center, New York, NY, United States

(Gallagher) Departments of Psychiatry and Anesthesiology and Critical Care, Penn Pain Medicine Center, University of Pennsylvania School of Medicine, Philadelphia, PA, United States

(Gilson) University of Wisconsin School of Medicine and Public Health, Madison, WI, United States

(Inturrisi) Department of Pharmacology, Joan and Sanford I. Weill Medical School, Cornell University, New York, New York, United States

(Haddox) Risk Management and Health Policy, Purdue Pharma, Stamford, CA, United States

(Jick) Department of Epidemiology, Boston University, Boston, MA, United States

(Horn) Department of Medical Informatics, Institute for Clinical Outcomes Research (ICOR), University of Utah School of Medicine, Salt Lake City, UT, United States

(Lipman) Department of Pharmacotherapy, L.S. Skaggs Pharmacy, University of Utah, Salt Lake City, UT, United States

(Loeser) Department of Neurological Surgery, University of Washington, Seattle, WA, United States

(Porter) National Institute of Neurological Disorders, Stroke Systems and Cognitive Neuroscience, National Institutes of Health, Bethesda, MD, United States

(Rowbotham) Pain Clinical Research Center, University of California San Francisco, San Francisco, CA, United States

(Noble, Schoelles) ECRI Institute, Plymouth Meeting, PA, United States

(Turk) Department of Anesthesiology, University of Washington, Seattle, WA, United States

(Von Korff) Center for Health Studies Group Health Cooperative, Seattle, WA, United States

(Webster) Lifetree Pain Clinic, Salt Lake City, UT, United States

(Weisner) Department of Psychiatry, University of California San Francisco, San Francisco, CA, United States

Publisher

Churchill Livingstone Inc. (650 Avenue of the Americas, New York NY 10011, United States)

Emtree Heading

abdominal cramp/si [Side Effect]; bloating/si [Side Effect]; cardiotoxicity/si [Side Effect]; *chronic pain/dt [Drug Therapy]; *chronic pain/th [Therapy]; clinical trial; cognitive defect/si [Side Effect]; comorbidity; constipation/si [Side Effect]; cross tolerance; dental caries/si [Side Effect]; depression/si [Side Effect]; diabetes mellitus/si [Side Effect]; drug abuse; drug efficacy; drug misuse; drug overdose; drug safety; drug tolerability; drug treatment failure; drug withdrawal; endocrine disease/si [Side Effect]; evidence based medicine; falling; fracture/si [Side Effect]; growth hormone deficiency/si [Side Effect]; health care policy; heart arrhythmia/si [Side Effect]; human; hyperalgesia/si [Side Effect]; immune deficiency/si [Side Effect]; incidence; infection/si [Side Effect]; infection sensitivity; intestine function disorder/si [Side Effect]; long term care; low back pain/dt [Drug Therapy]; lung tuberculosis/si [Side Effect]; medical research; nausea/si [Side Effect]; nonhuman; opiate addiction/si [Side Effect]; osteoporosis/si [Side Effect]; patient compliance; periodontal disease/si [Side Effect]; practice guideline; prescription; psychosocial environment; quantitative analysis; review; risk benefit analysis; sedation; side effect/si [Side Effect]; sleep disordered breathing/si [Side Effect]; socioeconomics; sudden death; treatment duration; treatment indication; treatment outcome; United States; vomiting/si [Side Effect]; xerostomia/si [Side Effect]; anticonvulsive agent/dt [Drug Therapy]; codeine/cb [Drug Combination]; codeine/dt [Drug Therapy]; fentanyl/dt [Drug Therapy]; hydrocodone/dt [Drug Therapy]; hydrocodone bitartrate plus paracetamol/dt [Drug Therapy]; hydromorphone/dt [Drug Therapy]; levorphanol/dt [Drug Therapy]; methadone/ae [Adverse Drug Reaction]; methadone/dt [Drug Therapy]; morphine/dt [Drug Therapy]; omega conotoxin MVIIA/dt [Drug Therapy]; omega conotoxin MVIIA/tl [Intrathecal Drug Administration]; *opiate/ae [Adverse Drug Reaction]; *opiate/ct [Clinical Trial]; *opiate/do [Drug Dose]; *opiate/dt [Drug Therapy]; *opiate/tl [Intrathecal Drug Administration]; *opiate/po [Oral Drug Administration]; *opiate/td [Transdermal Drug Administration]; oxycodone/dt [Drug Therapy]; oxymorphone/dt [Drug Therapy]; paracetamol/cb [Drug Combination]; paracetamol/dt [Drug Therapy]; pethidine/dt [Drug Therapy]; psychotropic agent/dt [Drug Therapy]; tricyclic antidepressant agent/dt [Drug Therapy].

Link to the Ovid Full Text or citation:

Click here for full text options

227.

Candidaemia in disseminated TB.

Sheety A.J., Adhikari P., Rao S., Ramapuraruj J., Kotians S., Kamath A.

Journal of Clinical and Diagnostic Research. 4 (6) (pp 3465-3467), 2010. Date of Publication: 2010.

AN: 2010708937

Context: Disseminated TB patients are invariably immunocompromised and are susceptible to opportunistic infections. Candidaemia is an under-recognised infection in India. Therefore, we planned to evaluate the prevalence, survival and risk factors for the development of this infection in disseminated TB patients in a tertiary care hospital in southern India. Aims: To assess the prevalence of invasive candidiasis/candidaemia in disseminated TB patients in India and to determine the survival of these patients and risk factors for the development of candidaemia in disseminated TB patients Settings and Design: A prospective study conducted after obtaining the institutional ethical committee's approval. Methods and Material: Disseminated TB patients who came between February 2005 and February 2006 were included in the study after obtaining informed consent from the patients with assured confidentiality. The sources of the fungal culture were blood and ascitic fluid. Statistical analysis: Fisher's test, the level of significance was set at < 0.05 Results: 103 disseminated TB patients were enrolled into this study, but the prevalence of candidaemia was just 5%. Intravenous broad spectrum antibiotics (P<0.001), oral broad spectrum antibiotics (P<0.01) and indwelling catheters (P<0.01) were the three important risk factors. Non candida albicans species were predominantly present. Conclusions: Candidaemia has a low prevalence in Indian climatic conditions. If the risk factors associated with this infection are avoided, survival can be improved.

Institution

(Sheety) Department Pharmacology (Adhikari, Rao, Ramapuraruj) Department Medicine

(Kotians) Department Community medicine, Kasturba Medical College, Mangalore campus, Manipal University, Manipal, India

(Kamath) Department Forensic Medicine

Publisher

Journal of Clinical and Diagnostic Research (71 Veer Nagar,G.T. Road, Delhi 110007, India)

Emtree Heading

antibiotic resistance; article; *candidemia/di [Diagnosis]; *candidemia/dt [Drug Therapy]; catheter infection; comorbidity; controlled study; early diagnosis; Fisher exact test; human; Human immunodeficiency virus infected patient; Human immunodeficiency virus infection/dt [Drug Therapy]; human tissue; India; infection risk; insulin dependent diabetes mellitus/dt [Drug Therapy]; major clinical study; *miliary tuberculosis/di [Diagnosis]; *miliary tuberculosis/dr [Drug Resistance]; *miliary tuberculosis/dt [Drug Therapy]; nonhodgkin lymphoma/dt [Drug Therapy]; outcome assessment; overall survival; prevalence; prognosis; prospective study; risk factor; sensitivity and specificity; survival rate; tertiary health care; amphotericin B/dt [Drug Therapy]; cyclophosphamide/dt [Drug Therapy]; doxorubicin/dt [Drug Therapy]; ethambutol/dt [Drug Therapy]; fluconazole/dt [Drug Therapy]; fluconazole/po [Oral Drug Administration]; insulin/dt [Drug Therapy]; insulin/sc [Subcutaneous Drug Administration]; isoniazid/dt [Drug Therapy]; lamivudine/dt [Drug Therapy]; nevirapine/dt [Drug Therapy]; pyrazinamide/dt [Drug Therapy]; rifampicin/dt [Drug Therapy]; vincristine/dt [Drug Therapy]; zidovudine/dt [Drug Therapy].

Link to the Ovid Full Text or citation:

Click here for full text options

228.

Here is diabetes in the Lancet's tuberculosis Series!.

Lonnroth K., Raviglione M.C.

The Lancet. 376 (9757) (pp 1987-1988), 2010. Date of Publication: December 11-17, 2010.

AN: 2010684645

Institution

(Lonnroth, Raviglione) Stop TB Department, WHO, 1211 Geneva, Switzerland

Publisher

Elsevier Limited (32 Jamestown Road, London NW1 7BY, United Kingdom)

Emtree Heading

adverse outcome; alcoholism; *diabetes mellitus; human; letter; priority journal; risk factor; smoking; treatment outcome; *tuberculosis.

Link to the Ovid Full Text or citation:

Click here for full text options

229.

Successful medical management of renal zygomycosis: A summary of two cases and a review of the Indian literature.

Marak R.S.K., Misra R., Ansari M.S., Dixit A., Poornima, Prasad K.N., Dhole T.N.

Medical Mycology. 48 (8) (pp 1088-1095), 2010. Date of Publication: December 2010.

AN: 2010631009

We present two cases of renal zygomycosis caused by Apophysomyces elegans and Mycocladus corymbifer in previously healthy immunocompetent males and an overview of the disease in India. In both cases a percutaneous nephrostomy (PCN) was performed and the etiologic agents were identified by direct microscopy and culture. Amphotericin B was administered and both patients recovered completely. A review of the literature revealed 42 cases of renal zygomycosis in India. The majority of them were from the Postgraduate Institute of Medical Education and Research, Chandigarh, in North India. In contrast to cases from the developed world where transplant recipients and patients with hematological malignancies seem to be most vulnerable to zygomycosis, the most common risk factor in India is uncontrolled diabetes mellitus. However, renal zygomycosis is an exception and the patients in both of our cases had no identifiable underlying disorder and recovered successfully without nephrectomy. It is important to emphasize that treatment of A. elegans must be aggressive and lipid formulations of antifungals are typically favored due to their limited side effects profile and ability of the clinician to use higher doses. A high index of clinical suspicion and knowledge of the varied manifestations in diagnosing this condition cannot be overemphasized. © 2010 ISHAM.

Institution

(Marak, Misra, Dixit, Poornima, Prasad, Dhole) Department of Microbiology, Sanjay Gandhi Postgraduate Institute of Medical Sciences, Lucknow, India (Ansari) Departments of Urology, Sanjay Gandhi Postgraduate Institute of Medical Sciences, Lucknow, India

Publisher

Informa Healthcare (69-77 Paul Street, London EC2A 4LQ, United Kingdom)

Emtree Heading

adolescent; adult; antibiotic therapy; article; case report; chill; computer assisted tomography; debridement; diabetes mellitus; differential diagnosis; drug formulation; dysuria; fever; flank pain; fungus hyphae; human; hydronephrosis/di [Diagnosis]; immunocompetence; India; intravenous pyelography; kidney tuberculosis/di [Diagnosis]; male; percutaneous nephrostomy; pyuria; rigor; risk factor; treatment outcome; ultrasound; urinalysis; vomiting; Zygomycetes; *zygomycosis/dm [Disease Management]; *zygomycosis/dt [Drug Therapy]; *zygomycosis/et [Etiology]; *zygomycosis/su [Surgery]; amphotericin B/dt [Drug Therapy]; amphotericin B/iv [Intravenous Drug Administration]; antibiotic agent.

Link to the Ovid Full Text or citation:

Click here for full text options

230.

Therapeutic drug monitoring for slow response to tuberculosis treatment in a state control program, Virginia, USA.

Heysell S.K., Moore J.L., Keller S.J., Houpt E.R.

Emerging Infectious Diseases. 16 (10) (pp 1546-1553), 2010. Date of Publication: October 2010.

AN: 2010592688

Therapeutic drug monitoring may be useful in tuberculosis management, but programmatic implementation is understudied. We performed a retrospective cohort study to determine prevalence of lower than expected levels of isoniazid, rifampin, ethambutol, and pyrazinamide measured at time of estimated peak serum concentration. Patients were tested for serum concentration at 2 hours after medication administration. When patients were tested, 22 had concen- trations lower than expected range for rifampin, 23 of 39 patients had low levels of isoniazid, and 8 of 26 patients had low levels of ethambutol; all 20 patients tested for pyrazinamide were within expected range. Over 26 months, 42 patients met criteria for slow response. Diabetes was associated with slow response (p<0.001), and persons with diabetes were more likely than persons without diabetes to have low rifampin levels (p = 0.03). Dosage adjustment of rifampin was more likely to elevate serum concentration to the target range than adjustment of isoniazid given in daily doses (p = 0.01).

Institution

(Heysell, Houpt) University of Virginia, Charlottesville, VA, United States (Moore, Keller) Virginia Department of Health, Richmond, VA, United States

Publisher

Centers for Disease Control and Prevention (CDC) (1600 Clifton Road, Mailstop C-12, Atlanta GA 30333, United States)

Emtree Heading

adult; aged; article; cohort analysis; controlled study; diabetes mellitus; drug blood level; drug dose increase; *drug monitoring; drug program; drug sensitivity; female; human; *lung tuberculosis/dt [Drug Therapy]; major clinical study; male; maximum plasma concentration; outcome assessment; retrospective study; *treatment response; United States; *ethambutol/an [Drug Analysis]; *ethambutol/cb [Drug Combination]; *ethambutol/cr [Drug Concentration]; *ethambutol/dt [Drug Therapy]; *isoniazid/an [Drug Analysis]; *isoniazid/cb [Drug Combination]; *isoniazid/cr [Drug Concentration]; *isoniazid/do [Drug Dose]; *isoniazid/dt [Drug Therapy]; *pyrazinamide/an [Drug Analysis]; *pyrazinamide/cb [Drug Combination]; *pyrazinamide/cr [Drug Concentration]; *pyrazinamide/dt [Drug Therapy]; *rifampicin/an [Drug Analysis]; *rifampicin/cb [Drug Combination]; *rifampicin/cr [Drug Concentration]; *rifampicin/dt [Drug Therapy].

Link to the Ovid Full Text or citation:

Click here for full text options

231.

Factors associated with multidrug-resistant tuberculosis: Comparison of patients born inside and outside of the Czech Republic.

Bartu V., Kopecka E., Havelkova M.

Journal of International Medical Research. 38 (3) (pp 1156-1163), 2010. Date of Publication: 2010.

AN: 2010398935

Multidrug-resistant tuberculosis (MDR-TB) is defined as resistance of Mycobacterium tuberculosis complex (MTB) to at least isoniazid and rifampicin. The aim of this study was to evaluate and compare a cohort of 50 patients with MDR-TB according to birthplace, resistance type, clinical outcome, length of bacteriological positivity of sputum and length of hospitalization. Thirty-three of the patients were born in the Czech Republic (group A) and 17 were immigrants to the Czech Republic (group B). Patients in group B were significantly younger (mean [range] age 33 [19-56] years) than those in group A (mean [range] age 48 [33-80] years). Primary resistance was present in 16 (48%) cases in group A and in 13 (76%) cases in group B. There were 36 (72%) cured patients, five (10%) remained on treatment and nine (18%) died; no patients failed or transferred out of the study. The mean length of bacteriological positivity of sputum samples was 5.9 months and the mean length of hospitalization was 8.2 months. Resistance to capreomycin was an important predictor of poor outcome. © 2010 Field House Publishing LLP.

Institution

(Bartu) Department of Respiratory Diseases, Medicon, Prague, Czech Republic (Kopecka) Central Unit for MDR-TB, Department of Respiratory Diseases 1st Medical School, Faculty Thomayer Hospital, Prague, Czech Republic

(Havelkova) Mycobacteriology Unit and National Reference Laboratory for Mycobacteria NIPH, WHO/IUATLD Supranational Reference Laboratory for Mycobacteria, Prague, Czech Republic

Publisher

Field House Publishing (6 Sompting Ave, Worthing BN14 8HN, United Kingdom)

Emtree Heading

adult; aged; article; birthplace; cardiovascular disease; chronic obstructive lung disease; clinical article; comorbidity; controlled study; Czech Republic; diabetes mellitus; drug treatment failure; female; human; immigrant; length of stay; liver disease; male; *multidrug resistant tuberculosis/dr [Drug Resistance]; *multidrug resistant tuberculosis/dt [Drug Therapy]; Mycobacterium tuberculosis; nonhuman; smoking habit; sputum culture; treatment duration; amikacin/cb [Drug Combination]; amikacin/dt [Drug Therapy]; aminosalicylic acid/cb [Drug Combination]; aminosalicylic acid/dt [Drug Therapy]; capreomycin/dt [Drug Therapy]; ciprofloxacin/cb [Drug Combination]; ciprofloxacin/dt [Drug Therapy]; clofazimine/cb [Drug Combination]; clofazimine/dt [Drug Therapy]; cycloserine/cb [Drug Combination]; cycloserine/dt [Drug Therapy]; ethambutol/dt [Drug Therapy]; isoniazid/dt [Drug Therapy]; pyrazinamide/dt [Drug Therapy]; quinoline derived antiinfective agent/dt [Drug Therapy]; rifampicin/dt [Drug Therapy]; streptomycin/dt [Drug Therapy].

Link to the Ovid Full Text or citation:

Click here for full text options

232.

Antiretroviral treatment of adult HIV infection: 2010 recommendations of the International AIDS Society-USA panel.

Thompson M.A., Aberg J.A., Cahn P., Montaner J.S.G., Rizzardini G., Telenti A., Gatell J.M., Gunthard H.F., Hammer S.M., Hirsch M.S., Jacobsen D.M., Reiss P., Richman D.D., Volberding P.A., Yeni P., Schooley R.T.

JAMA - Journal of the American Medical Association. 304 (3) (pp 321-333), 2010. Date of Publication: 21 Jul 2010.

AN: 2010395515

Context: Recent data regarding the consequences of untreated human immunodeficiency virus (HIV) infection and the expansion of treatment choices for antiretroviral-naive and antiretroviral-experienced patients warrant an update of the International AIDS Society-USA guidelines for the use of antiretroviral therapy in adults with HIV infection. Objectives: To provide updated recommendations for management of HIV-infected adults, using antiretroviral drugs and laboratory monitoring tools available in the international, developed-world setting. This report provides guidelines for when to initiate antiretroviral therapy, selection of appropriate initial regimens, patient monitoring, when to change therapy, and what regimens to use when changing. Data Sources and Study Selection: A panel with expertise in HIV research and clinical care reviewed relevant data published or presented at selected scientific conferences since the last panel report through April 2010. Data were identified through a PubMed search, review of scientific conference abstracts, and requests to antiretroviral drug manufacturers for updated clinical trials and adverse event data. Data Extraction and Synthesis: New evidence was reviewed by the panel. Recommendations were drafted by section writing committees and reviewed and edited by the entire panel. The quality and strength of the evidence were rated and recommendations were made by full panel consensus. Conclusions: Patient readiness for treatment should be confirmed before initiation of antiretroviral treatment. Therapy is recommended for asymptomatic patients with a CD4 cell count <500/muL, for all symptomatic patients, and those with specific conditions and comorbidities. Therapy should be considered for asymptomatic patients with CD4 cell count >500/muL. Components of the initial and subsequent regimens must be individualized, particularly in the context of concurrent conditions. Patients receiving antiretroviral treatment should be monitored regularly; treatment failure should be detected and managed early, with the goal of therapy, even in heavily pretreated patients, being HIV-1 RNA suppression below commercially available assay quantification limits. ©2010 American Medical Association. All rights reserved.

Institution

(Thompson) AIDS Research Consortium of Atlanta, Atlanta, GA, United States (Schooley) University of California San Diego, San Diego, CA, United States

(Aberg) New York University School of Medicine, New York, NY, United States

(Cahn) Hospital Juan Fernandez, University of Buenos Aires Medical School, Fundacion Huesped, Argentina

(Gatell) Hospital Clinic-IDIBAPS, University of Barcelona, Barcelona, Spain

(Gunthard) University Hospital Zurich, Division of Infectious Diseases, University of Zurich, Zurich, Switzerland

(Hammer) Columbia University College of Physicians and Surgeons, New York, NY, United States

(Hirsch) Harvard Medical School, Boston, MA, United States

(Jacobsen) International AIDS Society-USA, San Francisco, CA, United States

(Montaner) BC-Centre for Excellence in HIV/AIDS, Providence Health Care, University of British Columbia, Vancouver, BC, Canada

(Reiss) Academic Medical Center, University of Amsterdam, Amsterdam, Netherlands

(Richman) University of California San Diego, Veterans Affairs San Diego Healthcare System, Lausanne, Switzerland

(Rizzardini) Luigi Sacco Hospital, Milan, Italy

(Telenti) University Hospital of Lausanne, Lausanne, Switzerland

(Volberding) University of California San Francisco, San Francisco Veterans Affairs Medical Center, Paris, France

(Yeni) Hopital Bichat-Claude Bernard, Xavier Bichat Medical School, Paris, France

Publisher

American Medical Association (515 North State Street, Chicago IL 60654, United States)

Emtree Heading

*acquired immune deficiency syndrome/dr [Drug Resistance]; *acquired immune deficiency syndrome/dt [Drug Therapy]; *acquired immune deficiency syndrome/ep [Epidemiology]; acute heart infarction/ep [Epidemiology]; adult; African American; aging; aminotransferase blood level; atherosclerosis; bone density; bone marrow toxicity/si [Side Effect]; cardiovascular disease/co [Complication]; cardiovascular disease/ep [Epidemiology]; cardiovascular risk; CD4 lymphocyte count; chronic kidney disease/co [Complication]; chronic kidney disease/ep [Epidemiology]; clinical practice; comorbidity; diabetes mellitus; diarrhea/si [Side Effect]; disease course; dosage schedule comparison; drug efficacy; drug withdrawal; dyslipidemia/si [Side Effect]; gastrointestinal toxicity/si [Side Effect]; glomerulus filtration rate; heart infarction/si [Side Effect]; hepatitis B/dr [Drug Resistance]; hepatitis B/dt [Drug Therapy]; Hepatitis B virus; hepatitis C/dt [Drug Therapy]; Hepatitis C virus; high risk population; *highly active antiretroviral therapy; human; Human immunodeficiency virus 1; *Human immunodeficiency virus 1 infection/dr [Drug Resistance]; *Human immunodeficiency virus 1 infection/dt [Drug Therapy]; *Human immunodeficiency virus 1 infection/ep [Epidemiology]; *Human immunodeficiency virus infection/dr [Drug Resistance]; *Human immunodeficiency virus infection/dt [Drug Therapy]; *Human immunodeficiency virus infection/ep [Epidemiology]; hyperbilirubinemia/si [Side Effect]; hyperlipidemia/co [Complication]; hyperlipidemia/et [Etiology]; hyperlipidemia/si [Side Effect]; hypertension; hypertriglyceridemia/si [Side Effect]; immune reconstitution inflammatory syndrome; insulin resistance/si [Side Effect]; intravenous drug abuse; jaundice/si [Side Effect]; lipodystrophy/co [Complication]; lipodystrophy/et [Etiology]; lipodystrophy/si [Side Effect]; liver disease; liver fibrosis; liver toxicity/co [Complication]; liver toxicity/et [Etiology]; liver toxicity/si [Side Effect]; medical society; mortality; multidrug resistance; nephrolithiasis/co [Complication]; nephrolithiasis/si [Side Effect]; nephrotoxicity/si [Side Effect]; neurotoxicity/si [Side Effect]; nonhuman; opportunistic infection; patient monitoring; practice guideline; pregnant woman; priority journal; QT prolongation/si [Side Effect]; race difference; review; risk reduction; sexual transmission; side effect/si [Side Effect]; treatment failure; treatment indication; tuberculosis; United States; unspecified side effect/si [Side Effect]; vertical transmission; virus hepatitis/dr [Drug Resistance]; virus load; virus reactivation; virus transmission; abacavir/ae [Adverse Drug Reaction]; abacavir/cb [Drug Combination]; abacavir/dt [Drug Therapy]; abacavir plus lamivudine/ae [Adverse Drug Reaction]; abacavir plus lamivudine/cb [Drug Combination]; abacavir plus lamivudine/cm [Drug Comparison]; abacavir plus lamivudine/dt [Drug Therapy]; abacavir plus lamivudine/pr [Pharmaceutics]; *antiretrovirus agent/ae [Adverse Drug Reaction]; *antiretrovirus agent/dt [Drug Therapy]; *antiretrovirus agent/pr [Pharmaceutics]; atazanavir/ae [Adverse Drug Reaction]; atazanavir/cb [Drug Combination]; atazanavir/cm [Drug Comparison]; atazanavir/dt [Drug Therapy]; atazanavir plus ritonavir/ae [Adverse Drug Reaction]; atazanavir plus ritonavir/cm [Drug Comparison]; atazanavir plus ritonavir/do [Drug Dose]; atazanavir plus ritonavir/dt [Drug Therapy]; atazanavir plus ritonavir/pr [Pharmaceutics]; chemokine receptor CCR5; darunavir plus ritonavir/cb [Drug Combination]; darunavir plus ritonavir/cm [Drug Comparison]; darunavir plus ritonavir/dt [Drug Therapy]; darunavir plus ritonavir/pr [Pharmaceutics]; didanosine/ae [Adverse Drug Reaction]; didanosine/dt [Drug Therapy]; efavirenz/ae [Adverse Drug Reaction]; efavirenz/cm [Drug Comparison]; efavirenz/dt [Drug Therapy]; efavirenz/pr [Pharmaceutics]; efavirenz plus emtricitabine plus tenofovir disoproxil/dt [Drug Therapy]; efavirenz plus emtricitabine plus tenofovir disoproxil/pr [Pharmaceutics]; emtricitabine/cb [Drug Combination]; emtricitabine/dt [Drug Therapy]; emtricitabine/pr [Pharmaceutics]; emtricitabine plus tenofovir disoproxil/ae [Adverse Drug Reaction]; emtricitabine plus tenofovir disoproxil/cb [Drug Combination]; emtricitabine plus tenofovir disoproxil/cm [Drug Comparison]; emtricitabine plus tenofovir disoproxil/dt [Drug Therapy]; emtricitabine plus tenofovir disoproxil/pr [Pharmaceutics]; entecavir/dt [Drug Therapy]; fosamprenavir plus ritonavir/ae [Adverse Drug Reaction]; fosamprenavir plus ritonavir/cb [Drug Combination]; fosamprenavir plus ritonavir/cm [Drug Comparison]; fosamprenavir plus ritonavir/do [Drug Dose]; fosamprenavir plus ritonavir/dt [Drug Therapy]; indinavir/ae [Adverse Drug Reaction]; indinavir/dt [Drug Therapy]; indinavir plus ritonavir/ae [Adverse Drug Reaction]; *integrase strand transfer inhibitor/dt [Drug Therapy]; *integrase strand transfer inhibitor/pr [Pharmaceutics]; *integrase strand transfer inhibitor/pd [Pharmacology]; lamivudine/dt [Drug Therapy]; lopinavir plus ritonavir/ae [Adverse Drug Reaction]; lopinavir plus ritonavir/cb [Drug Combination]; lopinavir plus ritonavir/cm [Drug Comparison]; lopinavir plus ritonavir/dt [Drug Therapy]; lopinavir plus ritonavir/pr [Pharmaceutics]; *maraviroc/ae [Adverse Drug Reaction]; *maraviroc/cm [Drug Comparison]; *maraviroc/dt [Drug Therapy]; *maraviroc/pd [Pharmacology]; nevirapine/cb [Drug Combination]; nevirapine/cm [Drug Comparison]; nevirapine/dt [Drug Therapy]; *nonnucleoside reverse transcriptase inhibitor/do [Drug Dose]; *nonnucleoside reverse transcriptase inhibitor/dt [Drug Therapy]; *nonnucleoside reverse transcriptase inhibitor/pr [Pharmaceutics]; *proteinase inhibitor/cb [Drug Combination]; *proteinase inhibitor/do [Drug Dose]; *proteinase inhibitor/it [Drug Interaction]; *proteinase inhibitor/dt [Drug Therapy]; *proteinase inhibitor/pr [Pharmaceutics]; raltegravir/cm [Drug Comparison]; raltegravir/dt [Drug Therapy]; raltegravir/pr [Pharmaceutics]; raltegravir/pd [Pharmacology]; ribavirin/cb [Drug Combination]; ribavirin/dt [Drug Therapy]; ritonavir plus saquinavir/ae [Adverse Drug Reaction]; ritonavir plus saquinavir/cb [Drug Combination]; ritonavir plus saquinavir/cm [Drug Comparison]; ritonavir plus saquinavir/dt [Drug Therapy]; stavudine/ae [Adverse Drug Reaction]; stavudine/dt [Drug Therapy]; tenofovir/ae [Adverse Drug Reaction]; tenofovir/cb [Drug Combination]; tenofovir/cm [Drug Comparison]; tenofovir/cr [Drug Concentration]; tenofovir/do [Drug Dose]; tenofovir/it [Drug Interaction]; tenofovir/dt [Drug Therapy]; tenofovir/pr [Pharmaceutics]; tenofovir/pk [Pharmacokinetics]; unclassified drug; unindexed drug; zidovudine/ae [Adverse Drug Reaction]; zidovudine/cm [Drug Comparison]; zidovudine/dt [Drug Therapy].

Link to the Ovid Full Text or citation:

Click here for full text options

233.

Tuberculosis at extremes of age.

Schaaf H.S., Collins A., Bekker A., Davies P.D.O.

Respirology. 15 (5) (pp 747-763), 2010. Date of Publication: July 2010.

AN: 2010351445

Although tuberculosis (TB) has its highest burden among young adults, especially since the advent of HIV infection, two other groups with low immunity, the very young (<1 year) with immature immunity and the elderly (>65 years) with waning immunity, are vulnerable groups not to be forgotten. This review describes the epidemiology, clinical aspects, public health aspects and outcome of TB in patients at the extremes of age. The epidemiology differs therein that TB in infants occurs in developing countries with high incidences of TB and HIV, while TB in the elderly occurs in developed countries with ageing populations. The clinical presentation may be non-specific, history of contact with TB is often not known and TB is often not considered at these age extremes, and when the diagnosis is considered, disease progression may already be advanced. Anti-TB treatment regimens are the same as in other age groups, but drug dosages may need adjustment according to weight, renal function, liver function and other potentially complicating factors. Adverse events are more difficult to observe and both the young and the elderly are reliant on others for adherence to treatment. Mortality at both age extremes is higher than in the general TB population. For all the above reasons, public health measures to: prevent transmission of infection; identify those infected and providing preventive therapy; high index of suspicion in order to make an early diagnosis; and timely initiation of treatment are important in both the very young and the elderly. © 2010 The Authors.

Institution

(Schaaf, Bekker) Department of Paediatrics and Child Health, Stellenbosch University, Tygerberg Children's Hospital, Tygerberg, South Africa (Collins, Davies) Tuberculosis Research and Resources Unit, Liverpool Heart and Chest Hospital, Liverpool L14 3PE, United Kingdom

Publisher

Blackwell Publishing (550 Swanston Street, Carlton South VIC 3053, Australia)

Emtree Heading

abdominal distension; acid fast bacterium; aged; alcohol consumption; anorexia; apnea; ascites; BCG vaccination; blood flow; Brazil; chronic kidney disease; clinical feature; congenital tuberculosis; coughing; cyanosis; developed country; developing country; diabetes mellitus; directly observed therapy; disease course; disease predisposition; disease severity; disease transmission; DNA fingerprinting; dyspnea; early diagnosis; facial nerve paralysis; failure to thrive; fever; follow up; groups by age; health survey; hearing loss; hemoptysis; hepatomegaly; human; Human immunodeficiency virus; Human immunodeficiency virus prevalence; incidence; infant; infection control; infection prevention; infection risk; injection site abscess; irritability; jaundice; kidney function; liver dysfunction; liver function; liver toxicity; low birth weight; lung infiltrate; lung resection; lymphadenopathy; major clinical study; malnutrition; mastoiditis; Medline; mental disease; mixed infection; monitoring; morbidity; mortality; Mycobacterium bovis; Mycobacterium tuberculosis; nonhuman; otorrhea; outcome assessment; papular rash; patient compliance; Pneumocystis jiroveci; Pneumocystis pneumonia; prematurity; priority journal; prophylaxis; public health; pustule; respiratory distress; review; screening; senescence; sepsis; shock; short course therapy; splenomegaly; stridor; tachypnea; thorax pain; thorax radiography; tuberculin test; *tuberculosis/di [Diagnosis]; *tuberculosis/ep [Epidemiology]; *tuberculosis/su [Surgery]; tuberculous meningitis; tuberculous osteomyelitis; urogenital tuberculosis; visual disorder; weakness; weight; weight reduction; wheezing; world health organization; acetohydrazide; antiretrovirus agent; cotrimoxazole; digoxin; ethambutol; ethionamide; gamma interferon; hydrazine; immunosuppressive agent; isoniazid; phenytoin; pyrazinamide; pyridoxine; rifampicin; streptomycin.

Link to the Ovid Full Text or citation:

Click here for full text options

234.

Prevention trials: Their place in how we understand the value of prevention strategies.

Colditz G.A., Taylor P.R.

Annual Review of Public Health. 31 (pp 105-120), 2010. Date of Publication: 21 Apr 2010.

AN: 2010294685

Several key factors bear on the interpretation of prevention trials and observational studies that inform prevention strategies. These factors include the underlying disease process and aspects of the intervention: sustainability of behavior change, the time course of the intervention within the disease process, dose and duration of exposure needed to effect risk reduction, durability of the impact of intervention, and methodological problems in implementing and interpreting randomized trials and observational studies to evaluate prevention strategies. The question asked through an intent-to-treat analysis of a randomized controlled trial (RCT) differs from that in the observational setting. Furthermore, the long duration necessary to conduct prevention trials and the resulting lack of adherence to therapy can bias results toward the null. A broader range of approaches to evaluate prevention interventions and programs with improved knowledge synthesis and translation to public health practice will speed our progress toward achieving public health and prevention of chronic diseases. Copyright © 2010 by Annual Reviews. All rights reserved.

Institution

(Colditz) Department of Surgery, Washington University School of Medicine, Saint Louis, MO 63110, United States (Taylor) Genetic Epidemiology Branch, Division of Cancer Epidemiology and Genetics, National Cancer Institute, Bethesda, MD 20892-7236, United States

Publisher

Annual Reviews Inc. (4139 El Camino Way, P.O. Box 10139, Palo Alto CA 94306, United States)

Emtree Heading

behavior change; blood pressure monitoring; breast cancer/dt [Drug Therapy]; breast cancer/pc [Prevention]; calcium intake; cancer prevention; cardiovascular disease/dt [Drug Therapy]; cardiovascular disease/pc [Prevention]; chronic disease; clinical trial; colon cancer/dt [Drug Therapy]; colon cancer/pc [Prevention]; diabetes mellitus/pc [Prevention]; diet; diet supplementation; hepatitis B/dt [Drug Therapy]; hepatitis B/pc [Prevention]; human; hypertension/di [Diagnosis]; information dissemination; lifestyle modification; low drug dose; methodology; nonhuman; observational study; outcome assessment; patient compliance; physical activity; *preventive health service; *preventive medicine; priority journal; public health service; randomized controlled trial; review; risk reduction; screening test; tuberculosis/dt [Drug Therapy]; tuberculosis/pc [Prevention]; uterine cervix cancer/dt [Drug Therapy]; uterine cervix cancer/pc [Prevention]; uterus cancer/si [Side Effect]; vaccination; weight control; acetylsalicylic acid/ct [Clinical Trial]; acetylsalicylic acid/dt [Drug Therapy]; antihypertensive agent/dt [Drug Therapy]; antilipemic agent/dt [Drug Therapy]; BCG vaccine/dt [Drug Therapy]; calcium/ct [Clinical Trial]; calcium/dt [Drug Therapy]; cyclooxygenase 2 inhibitor/dt [Drug Therapy]; eflornithine/ct [Clinical Trial]; eflornithine/cb [Drug Combination]; eflornithine/dv [Drug Development]; eflornithine/dt [Drug Therapy]; eflornithine/to [Drug Toxicity]; hepatitis B vaccine/dt [Drug Therapy]; raloxifene/ct [Clinical Trial]; raloxifene/dt [Drug Therapy]; sulindac/ct [Clinical Trial]; sulindac/cb [Drug Combination]; tamoxifen/ae [Adverse Drug Reaction]; tamoxifen/ct [Clinical Trial]; tamoxifen/dt [Drug Therapy]; Wart virus vaccine/dt [Drug Therapy].

Drug Trade Names and Manufacturers

aspirin

Link to the Ovid Full Text or citation:

Click here for full text options

235.

Significance of spirometry in diabetic patients.

Meo S.A.

International Journal of Diabetes Mellitus. 2 (1) (pp 47-50), 2010. Date of Publication: April 2010.

AN: 2010157620

Spirometry is a widely used pulmonary function test (PFT), ideally suited to describing the effects of obstruction or restriction on lung function. It is a powerful diagnostic tool that plays a significant role in the early diagnosis of lung damage and its associated structures. It is also used to monitor the therapeutic efficacy of various treatment regimes and the course of the disease. The spirometric parameters have gained more popularity when it has been reported that impaired Forced Vital Capacity (FVC) and Forced Expiratory Volume in 1 s (FEV1) are emerging novel risk factors for type 2 diabetes mellitus. These spirometric parameter derangements have been evident on spirometry long before the clinical diagnosis of diabetes mellitus or insulin resistance. In spite of this, spirometry is not used routinely as part of a management system in diabetic patients. Its role is neither fully explored, nor fully utilized to achieve quality of life when managing diabetes mellitus. The aim of the present review is to highlight the evidence based significance of spirometry in the light of peer reviewed published literature. It may serve as a brief reference for diabetes management teams to enable spirometry to be included in the algorithm of the routine assessment of diabetic patients. © 2009 International Journal of Diabetes Mellitus.

Institution

(Meo) Department of Physiology, College of Medicine, King Khalid University Hospital, Saudi Arabia

Publisher

Elsevier Ltd (Langford Lane, Kidlington, Oxford OX5 1GB, United Kingdom)

Emtree Heading

breathing muscle; clinical feature; *diabetes mellitus/di [Diagnosis]; diabetic patient; disease severity; forced expiratory volume; forced vital capacity; human; lung disease/di [Diagnosis]; lung function; lung function test; lung gas exchange; lung infection/co [Complication]; lung tuberculosis/co [Complication]; muscle stretching; non insulin dependent diabetes mellitus/di [Diagnosis]; nonhuman; outcome assessment; peak expiratory flow; priority journal; review; risk factor; *spirometry.

Link to the Ovid Full Text or citation:

Click here for full text options

236.

Acute Tuberculosis.

Schlossberg D.

Infectious Disease Clinics of North America. 24 (1) (pp 139-146), 2010. Date of Publication: March 2010.

AN: 2010103997

Both primary and reactivation tuberculosis may present as an acute process and mimic community-acquired pneumonia. Tuberculosis should always be included in the initial differential diagnosis, and suspicion should be heightened by a variety of clinical and epidemiologic clues, as well as by multiple underlying conditions. This article reviews the pathophysiology, risk factors, and clinical manifestations of acute presentations of tuberculosis. © 2010 Elsevier Inc. All rights reserved.

Institution

(Schlossberg) Temple University School of Medicine, Philadelphia, PA, United States (Schlossberg) University of Pennsylvania School of Medicine, Philadelphia, PA, United States

(Schlossberg) Tuberculosis Control Program, Philadelphia Department of Public Health, 500 South Broad Street, Philadelphia, PA 19146, United States

Publisher

W.B. Saunders (Independence Square West, Philadelphia PA 19106-3399, United States)

Emtree Heading

acute disease; atelectasis/co [Complication]; bronchoscopy; community acquired pneumonia; coughing; diabetes mellitus; disease transmission; drug treatment failure; dyspnea; emphysema/co [Complication]; erythema nodosum/co [Complication]; exposure; fever; gastrectomy; head and neck carcinoma; hemodialysis patient; human; immune deficiency; jejunoileal bypass; kidney failure; lung calcification; lung cavitation; lung infiltrate; lung parenchyma; lymphadenopathy; malaise; Mycobacterium tuberculosis; night sweat; pathogenesis; pleura effusion; pneumonia; pneumothorax; postprimary tuberculosis; primary tuberculosis; relapse; review; risk factor; silicosis; sore throat; thorax pain; thorax radiography; *tuberculosis/di [Diagnosis]; *tuberculosis/dr [Drug Resistance]; *tuberculosis/dt [Drug Therapy]; *tuberculosis/et [Etiology]; weight reduction; corticosteroid/dt [Drug Therapy]; quinoline derived antiinfective agent/dt [Drug Therapy].

Link to the Ovid Full Text or citation:

Click here for full text options

237.

Reduced health provider delay and tuberculosis mortality due to an improved hospital programme.

Liu Y.-C., Lin H.-H., Chen Y.-S., Su I.-J., Huang T.-S., Tsai H.-C., Wann S.-R., Lee S.S.-J.

International Journal of Tuberculosis and Lung Disease. 14 (1) (pp 72-78), 2010. Date of Publication: January 2010.

AN: 2010093408

SETTING: A referral hospital in Kaohsiung,Taiwan. OBJECTIVE: To evaluate the impact of an in-hospital tuberculosis (TB) quality care programme initiated in May 2005 on health provider delay and outcome of newly diagnosed TB cases. DESIGN: Retrospective chart review of newly diagnosed TB cases presenting in 2002 and 2006. Health provider delay, clinical manifestations, management and outcome were recorded. RESULTS: Overall, 327 patients before (2002) and 262 patients after (2006) the programme began were enrolled. Patients were older men (mean age 65.9 years) and 23.4% (138/589) had diabetes; 84.4% had received anti-tuberculosis treatment. The programme shortened the time for doctors to order a chest X-ray (P < 0.01), and the reporting time for smear (P < 0.0001) and culture (P < 0.0001). On multivariable analysis, risk factors for attributable mortality included age >65 years (OR 4.4, 95%CI 1.8-10.9, P = 0.001) and liver cirrhosis (OR 4.3, 95%CI 1.1-16.6, P = 0.04). Treatment reduced mortality by 81% (OR 0.2, 95%CI 0.1-0.4, P < 0.001) and the programme halved overall mortality (OR 0.5, 95%CI 0.3-0.8, P = 0.01), and reduced attributable mortality by 62% (OR 0.4, 95%CI 0.2-0.8, P < 0.01). CONCLUSION: Intervention at the hospital level for quality control of TB care was instrumental in reducing health provider delay and led to a significant reduction in mortality. ©2010 The Union.

Institution

(Liu) Section of Infectious Diseases, Department of Internal Medicine, Shuang-Ho Hospital, Taipei, Taiwan (Republic of China) (Liu) Taipei Medical University, Taipei, Taiwan (Republic of China)

(Lin) Section of Infectious Diseases, E-Da Hospital, I-Shou University, Kaohsiung, Taiwan (Republic of China)

(Lin, Chen, Huang, Tsai, Wann, Lee) National Yang-Ming University, Taipei, Taiwan (Republic of China)

(Chen, Tsai, Wann, Lee) Section of Infectious Diseases, Department of Internal Medicine, Kaohsiung Veterans General Hospital, Kaohsiung, Taiwan (Republic of China)

(Su) Division of Clinical Research, National Health Research Institutes, Zhunan, Miaoli

(Huang) Section of Microbiology and Infectious Diseases, Kaohsiung Veterans General Hospital, Kaohsiung, Taiwan (Republic of China)

(Huang) Department of Medical Technology, Foo-Yin Institute of Technology, Kaohsiung, Taiwan (Republic of China)

Publisher

International Union against Tubercul. and Lung Dis. (68 boulevard Saint-Michel, Paris 75006, France)

Emtree Heading

adult; aged; article; bacterium culture; clinical feature; female; *health care personnel; health care quality; *health program; human; liver cirrhosis; major clinical study; male; medical record review; *mortality; multivariate analysis; outcome assessment; priority journal; quality control; retrospective study; risk factor; smear; *therapy delay; thorax radiography; *tuberculosis/di [Diagnosis]; *tuberculosis/ep [Epidemiology].

Link to the Ovid Full Text or citation:

Click here for full text options

238.

Pharmacologic treatment of behavioral symptoms associated with autism and other pervasive developmental disorders.

Wink L.K., Erickson C.A., McDougle C.J.

Current Treatment Options in Neurology. 12 (6) (pp 529-538), 2010. Date of Publication: November 2010.

AN: 2010586808

Opinion statement: Pervasive developmental disorders (PDDs), including autistic disorder (autism), Asperger's disorder, and pervasive developmental disorder not otherwise specified (PDD NOS), are neurodevelopmental disorders of childhood onset. These disorders persist throughout the lifespan of affected individuals and are characterized by impaired social behavior and communication, along with repetitive, stereotypic behaviors. Early diagnosis and subsequent behavioral therapy have been shown to improve outcomes for these individuals. Risperidone and aripiprazole have been approved by the United States Food and Drug Administration (FDA) for treatment of irritability associated with autism in children and adolescents. Despite their efficacy, use of these medications is limited by their side effects. In individuals with severe irritability, the first-line treatment is often risperidone. Because of its relatively lower risk of weight gain and metabolic side effects, aripiprazole may be used initially if there is a personal or family history of obesity or diabetes. Monitoring of body mass index and metabolic profiles is indicated with both medications. Stereotypic behaviors associated with autism, though clearly driven by neurobiologic processes, can also be understood as coping mechanisms used to decrease anxiety. From this perspective, therapies targeting reduction of these symptoms may be contraindicated. However, when these symptoms are severe and interfering, pharmacotherapy may be necessary. Serotonin reuptake inhibitors are of limited efficacy in children and adolescents, but risperidone and aripiprazole have been shown to reduce these symptoms. There remains a need for further safety and efficacy research in this area. Hyperactivity and inattention are currently treated with a variety of medications, including guanfacine, which has a relatively benign side effect profile. Stimulant medications are generally avoided as first-line treatment for hyperactivity because of concerns about increased irritability. Currently, social impairment is best addressed through behavioral therapy and social skills training. Novel pharmacotherapies to improve social impairment are in the early stages of research. © 2010 Springer Science+Business Media, LLC.

Institution

(Wink, Erickson, McDougle) Department of Psychiatry, Indiana University School of Medicine, Psychiatry Building-A305, 1111 West 10th Street, Indianapolis, IN 46202-4800, United States

Publisher

Current Science Ltd (34-42 Cleveland Street, London W1P 6LB, United Kingdom)

Emtree Heading

add on therapy; agitation; akathisia/si [Side Effect]; Alzheimer disease/dt [Drug Therapy]; anorexia/si [Side Effect]; antisocial behavior/dt [Drug Therapy]; anxiety disorder/dt [Drug Therapy]; Asperger syndrome/dt [Drug Therapy]; attention deficit disorder/co [Complication]; attention deficit disorder/di [Diagnosis]; attention deficit disorder/dt [Drug Therapy]; attention disturbance/co [Complication]; attention disturbance/dt [Drug Therapy]; *autism/dt [Drug Therapy]; automutilation/dt [Drug Therapy]; *behavior disorder/co [Complication]; *behavior disorder/di [Diagnosis]; *behavior disorder/dt [Drug Therapy]; *behavior disorder/si [Side Effect]; *behavior disorder/th [Therapy]; behavior therapy; blood pressure; body mass; cardiovascular disease/si [Side Effect]; central nervous system disease/si [Side Effect]; child psychiatry; clinical trial; communication disorder/co [Complication]; communication disorder/di [Diagnosis]; communication disorder/dt [Drug Therapy]; communication disorder/th [Therapy]; compulsion/co [Complication]; compulsion/di [Diagnosis]; compulsion/dt [Drug Therapy]; compulsion/th [Therapy]; concentration loss/si [Side Effect]; constipation/si [Side Effect]; coping behavior; diarrhea/si [Side Effect]; disease association; dizziness/si [Side Effect]; drowsiness/si [Side Effect]; drug antagonism; drug approval; drug blood level; drug contraindication; drug efficacy; drug indication; drug mechanism; drug metabolism; drug safety; drug tolerability; drug withdrawal; dry skin/si [Side Effect]; dyslipidemia/si [Side Effect]; early diagnosis; energy metabolism; fatigue/si [Side Effect]; gastrointestinal symptom/si [Side Effect]; headache/si [Side Effect]; heart rate; human; hyperactivity/co [Complication]; hyperactivity/dt [Drug Therapy]; hyperactivity/si [Side Effect]; hyperglycemia/si [Side Effect]; hypersalivation/si [Side Effect]; hypertension/dt [Drug Therapy]; hypotension/si [Side Effect]; impulsiveness; increased appetite/si [Side Effect]; infantile autism/dt [Drug Therapy]; insomnia/si [Side Effect]; intellectual impairment/dt [Drug Therapy]; irritability; mania/si [Side Effect]; metabolic disorder/si [Side Effect]; metabolic parameters; motor dysfunction/si [Side Effect]; nausea/si [Side Effect]; onset age; outcome assessment; personality disorder/dt [Drug Therapy]; pervasive developmental disorder not otherwise specified; *psychopharmacotherapy; QT prolongation/si [Side Effect]; rash/si [Side Effect]; review; risk benefit analysis; sedation; seizure/si [Side Effect]; seizure threshold; sexual dysfunction/si [Side Effect]; side effect/si [Side Effect]; sleep disorder/si [Side Effect]; social adaptation; sociopathy/co [Complication]; sociopathy/di [Diagnosis]; sociopathy/dt [Drug Therapy]; sociopathy/th [Therapy]; somnolence/si [Side Effect]; stereotypy; suicidal ideation/si [Side Effect]; syncope/si [Side Effect]; tardive dyskinesia/si [Side Effect]; tremor/si [Side Effect]; tuberculosis/dt [Drug Therapy]; unspecified side effect/si [Side Effect]; vertigo/si [Side Effect]; vomiting/si [Side Effect]; weight gain; xerostomia/si [Side Effect]; antihypertensive agent/it [Drug Interaction]; aripiprazole/ae [Adverse Drug Reaction]; aripiprazole/ct [Clinical Trial]; aripiprazole/cr [Drug Concentration]; aripiprazole/dt [Drug Therapy]; atomoxetine/ae [Adverse Drug Reaction]; atomoxetine/ct [Clinical Trial]; atomoxetine/cm [Drug Comparison]; atomoxetine/it [Drug Interaction]; atomoxetine/dt [Drug Therapy]; atomoxetine/pd [Pharmacology]; citalopram/ae [Adverse Drug Reaction]; citalopram/ct [Clinical Trial]; citalopram/cm [Drug Comparison]; citalopram/it [Drug Interaction]; citalopram/dt [Drug Therapy]; cycloserine/ae [Adverse Drug Reaction]; cycloserine/ct [Clinical Trial]; cycloserine/cr [Drug Concentration]; cycloserine/it [Drug Interaction]; cycloserine/dt [Drug Therapy]; cycloserine/pd [Pharmacology]; ethionamide/it [Drug Interaction]; fluoxetine/ae [Adverse Drug Reaction]; fluoxetine/ct [Clinical Trial]; fluoxetine/cm [Drug Comparison]; fluoxetine/it [Drug Interaction]; fluoxetine/dt [Drug Therapy]; guanfacine/ae [Adverse Drug Reaction]; guanfacine/ct [Clinical Trial]; guanfacine/dt [Drug Therapy]; isoniazid/it [Drug Interaction]; memantine/ae [Adverse Drug Reaction]; memantine/it [Drug Interaction]; memantine/dt [Drug Therapy]; methylphenidate/ae [Adverse Drug Reaction]; methylphenidate/ct [Clinical Trial]; methylphenidate/cm [Drug Comparison]; methylphenidate/dt [Drug Therapy]; monoamine oxidase inhibitor/it [Drug Interaction]; n methyl dextro aspartic acid receptor blocking agent/it [Drug Interaction]; neuroleptic agent/ae [Adverse Drug Reaction]; oxytocin/ae [Adverse Drug Reaction]; oxytocin/dt [Drug Therapy]; oxytocin/im [Intramuscular Drug Administration]; oxytocin/na [Intranasal Drug Administration]; oxytocin/iv [Intravenous Drug Administration]; oxytocin/pd [Pharmacology]; pimozide/it [Drug Interaction]; placebo; psychostimulant agent/ae [Adverse Drug Reaction]; psychostimulant agent/dt [Drug Therapy]; risperidone/ae [Adverse Drug Reaction]; risperidone/ct [Clinical Trial]; risperidone/dt [Drug Therapy]; risperidone/pk [Pharmacokinetics]; serotonin uptake inhibitor/dt [Drug Therapy]; thioridazine/it [Drug Interaction].

Link to the Ovid Full Text or citation:

Click here for full text options

239.

Tuberculosis treatment outcomes: A retrospective cohort analysis of smoking versus non-smoking patients in the state of Penang, Malaysia.

Dujaili J.A., Sulaiman S.A.S., Awaisu A., Muttalif A.R., Jaafar A.Q.

Pharmacotherapy. Conference: 2010 Annual Meeting of the American College of Clinical Pharmacy, ACCP 2010 Austin, TX United States. Conference Start: 20101017 Conference End: 20101020. Conference Publication: (var.pagings). 30 (10) (pp 402e-403e), 2010. Date of Publication: October 2010.

AN: 71764599

Purpose: The association between tobacco smoking and tuberculosis (TB) is increasingly coming to light and the literature is laden with the evidence of this association. However, only a few observational studies specifically investigated the association between smoking and TB treatment outcomes. The present study aims to compare the treatment outcomes between smoking and non-smoking TB patients. Methods: A retrospective cohort study comparing smoking and nonsmoking TB patients with regards to treatment outcomes was conducted. The data were extracted from medical records of newly diagnosed TB patients of both sexes, above 15 years of age whose smoking status was documented and who registered at a chest clinic of a tertiary care hospital in Penang, Malaysia from January 1 2006 to June 30 2008. Category II (TB relapse, treatment failure and treatment default), Category III (chronic cases) and HIV co-infected TB patients were excluded from the study. Furthermore, other Category I cases comprising of pregnant women, patients with diabetes and those with incomplete data were also excluded. Results: Smoking was significantly associated with male gender, alcohol use, and intravenous drug use. Ever smokers had increased likelihood of treatment failure (OR=7.48; CI 0.93, 60.30), default (OR=7.17; CI 2.76, 18.62) and were less likely to be cured (OR=0.34; CI 0.21, 0.49). After controlling for the effects of confounders using multivariate logistic regression, ever smokers were still less likely to be cured (aOR=0.31; CI 0.17, 0.57) and more likely to default treatment (aOR=3.24; CI 1.01, 10.45). Dying from TB did not reach statistical significance. Conclusion: This study further reaffirms that smoking is an independent predictor of poor TB treatment outcomes and prognosis. Therefore, smoking cessation interventions should be offered to all TB patients who are smokers when they are undergoing TB treatment.

Institution

(Dujaili, Sulaiman, Awaisu, Jaafar) School of Pharmaceutical Science, Universiti Sains Malaysia, Minden, Malaysia (Muttalif) Department of Respiratory Medicine, Penang Hospital, Jalan Residensi, Malaysia

Publisher

Pharmacotherapy Publications Inc.

Emtree Heading

*smoking; *treatment outcome; *cohort analysis; *patient; *human; *Malaysia; *American; *college; *clinical pharmacy; *tuberculosis; treatment failure; pregnant woman; tertiary care center; relapse; male; female; smoking cessation; prognosis; hospital; logistic regression analysis; thorax; medical record; drug use; alcohol consumption; gender; statistical significance; diabetes mellitus; observational study; Human immunodeficiency virus.

Link to the Ovid Full Text or citation:

Click here for full text options

240.

Tuberculosis and diabetes mellitus: merging epidemics.

Sen T., Joshi S.R., Udwadia Z.F.

The Journal of the Association of Physicians of India. 57 (pp 399-404), 2009. Date of Publication: 01 May 2009.

AN: 19634288

The link between tuberculosis (TB) and diabetes mellitus (DM) has occupied the center stage of discussion. Experts have raised concern about the merging epidemics of tuberculosis and diabetes particularly in the low to medium income countries like India and China that have the highest burden of TB in the world, and are experiencing the fastest increase in the prevalence of DM. There is good evidence that DM makes a substantial contribution to TB incidence. The huge prevalence of DM in India, may be contributing to the increasing prevalence of TB. This review looks at the link between these two merging epidemics. We discuss the epidemiology, clinical features, microbiology and radiology, and management and treatment outcomes of patients with tuberculosis and diabetes mellitus.

Institution

(Sen, Joshi, Udwadia) Hinduja Hospital and National Research Centre, Mahim, Mumbai, India

Emtree Heading

diabetes mellitus/ep [Epidemiology]; epidemic; health; human; incidence; India; prevalence; risk factor; *statistics and numerical data; Tuberculosis, Pulmonary/di [Diagnosis]; Tuberculosis, Pulmonary/dt [Drug Therapy]; Tuberculosis, Pulmonary/ep [Epidemiology]; tuberculostatic agent/dt [Drug Therapy].

Link to the Ovid Full Text or citation:

Click here for full text options

241.

Clinical manifestations and outcome of tuberculosis in diabetic patients admitted to King Abdulaziz University Hospital in Jeddah, Saudi Arabia.

Siddiqui A.M.

Journal of Taibah University Medical Sciences. 4 (2) (pp 148-155), 2009. Date of Publication: 2009.

AN: 2011086886

Objectives: Compare epidemiology, clinical manifestations and outcome of tuberculosis between diabetic and non-diabetic patients admitted to King Abdulaziz University Hospital in Jeddah, Saudi Arabia. Methods: Review the medical data of adult inpatients with tuberculosis from January 2002 to December 2007. Demographic data, location of tuberculosis, sputum positivity for acid fast bacilli, duration of hospitalization and outcome (relapses, drug resistance and inhospital mortality) were compared between the two groups. Results: From total of 216 tuberculosis cases, 35 (16%) had diabetes. These were older in age. 68% diabetics were over 50 years compared to 16% non-diabetics. (p=0.0001). Ratio of Saudis to non-Saudis with tuberculosis was greater in diabetics than in non-diabetics (p=0.003). Pulmonary tuberculosis was the most common site affected and was associated with poor glycemic control (p = 0.013). Smear positivity for acid fast bacilli was not different in diabetics and non-diabetics (p = 0.66). Outcome was favorable and not significantly different in diabetic patients (p = 0.096) Conclusion: Saudi diabetic patients have increased risk of tuberculosis. Pulmonary tuberculosis is most common in diabetics and is associated with poor glucose control. Diabetes does not alter the outcome of tuberculosis significantly.

Institution

(Siddiqui) Department of Internal Medicine, King Abdulaziz University Hospital, Jeddah 21589, Saudi Arabia

Publisher

Darussalam Publisher (P.o Box 22743, Riyadh 11416, Saudi Arabia)

Emtree Heading

adult; age distribution; aged; antibiotic therapy; article; blood glucose monitoring; controlled study; demography; diabetes mellitus; *diabetic patient; disease association; hospital admission; hospitalization; human; length of stay; *lung tuberculosis/dt [Drug Therapy]; major clinical study; medical record review; mortality; relapse; retrospective study; risk assessment; Saudi Arabia; sputum analysis; treatment outcome; university hospital; tuberculostatic agent/dt [Drug Therapy].

Link to the Ovid Full Text or citation:

Click here for full text options

242.

A series of critically challenging case scenarios in moderate to severe psoriasis: A Delphi consensus approach.

Strober B., Berger E., Cather J., Cohen D., Crowley J.J., Gordon K.B., Gottlieb A., Horn E.J., Kavanaugh A.F., Korman N.J., Krueger G.G., Leonardi C.L., Menter A., Schwartzman S., Sobell J.M., Young M.

Journal of the American Academy of Dermatology. 61 (1 SUPPL. 1) (pp S1-S46), 2009. Date of Publication: July 2009.

AN: 2009324737

Clinical trials for systemic psoriasis therapy typically enroll healthy patients and exclude patients with cardiovascular disease, latent tuberculosis, liver disease, histories of malignancies, viral infections, children, and pregnant or breast-feeding women. Physicians often require guidance for optimum management of severe psoriasis in patients that have a combination of underlying disease states. To provide treatment recommendations for complex psoriasis scenarios, a consensus panel comprising 15 experts in psoriatic disease convened to review and discuss available evidence-based data and to arrive at a consensus for treatment options of difficult cases. An application of the Delphi Method was used to select case scenarios, provide medical treatment options, present the case study with existing medical evidence, and anonymously vote on treatment options. The top 10 treatment options were ranked and statistically analyzed to compare the differences between treatments. The final rankings and analysis provide guidance for practical, safe, and efficacious treatment options in a number of complex psoriasis scenarios. © 2009 American Academy of Dermatology, Inc.

Institution

(Strober, Cohen) Department of Dermatology, New York University Medical Center, New York, NY, United States (Berger, Gottlieb, Sobell) Department of Dermatology, Tufts University-New England Medical Center, Boston, MA, United States

(Korman, Young) Department of Dermatology, Case Western Reserve University, Cleveland, OH, United States

(Krueger) Department of Dermatology, University of Utah School of Medicine, Salt Lake City, UT, United States

(Leonardi) Department of Dermatology, Saint Louis University School of Medicine, Saint Louis, MO, United States

(Cather) Modern Dermatology, Dallas, TX, United States

(Crowley) Bakersfield Dermatology and Skin Cancer Medical Group, Bakersfield, CA, United States

(Gordon) ENH Medical Group, Skokie, IL, United States

(Horn) National Psoriasis Foundation, Portland, OR, United States

(Kavanaugh) Center for Innovative Therapy, University of California, San Diego, Medical Center, San Diego, CA, United States

(Menter) Baylor University Medical Center, Dallas, TX, United States

(Schwartzman) Department of Clinical Medicine, Weil Cornell Medical College, New York Presbyterian Hospital, New York, NY, United States

Publisher

Mosby Inc. (11830 Westline Industrial Drive, St. Louis MO 63146, United States)

Emtree Heading

abdominal obesity; absence of side effects/si [Side Effect]; accidental injury/si [Side Effect]; alanine aminotransferase blood level; aminotransferase blood level; amyloidosis/dt [Drug Therapy]; anemia/si [Side Effect]; ankylosing spondylitis/dt [Drug Therapy]; anus atresia/si [Side Effect]; article; aseptic meningitis/si [Side Effect]; aspartate aminotransferase blood level; atopic dermatitis/dt [Drug Therapy]; B cell lymphoma/si [Side Effect]; biological therapy; birth defect/co [Complication]; blastomycosis/dt [Drug Therapy]; body mass; breast feeding education; case study; climatotherapy; clinical trial; coccidioidomycosis/si [Side Effect]; common cold/si [Side Effect]; comorbidity; congestive heart failure/si [Side Effect]; consensus; convulsion/si [Side Effect]; Crohn disease/dt [Drug Therapy]; cutaneous T cell lymphoma/et [Etiology]; Delphi study; demyelinating disease; disease severity; dizziness/si [Side Effect]; drug bioavailability; drug cost; drug dose comparison; drug dose reduction; drug efficacy; drug excretion; drug fatality/si [Side Effect]; drug half life; drug megadose; drug safety; drug tolerability; drug treatment failure; drug withdrawal; enteritis/dt [Drug Therapy]; esophagus atresia/si [Side Effect]; evidence based medicine; evidence based practice; excimer laser; experimental study; headache/si [Side Effect]; health care cost; health insurance; health program; hepatitis B/co [Complication]; hepatitis B/di [Diagnosis]; hepatitis B/et [Etiology]; hepatitis B/pc [Prevention]; Hepatitis B virus; hepatitis C/dt [Drug Therapy]; hepatitis C/si [Side Effect]; high risk patient; histoplasmosis/si [Side Effect]; human; hyperkalemia/si [Side Effect]; hypersensitivity reaction/si [Side Effect]; hypertension/si [Side Effect]; immune response; immunosuppressive treatment; infection/si [Side Effect]; injection site reaction/si [Side Effect]; juvenile rheumatoid arthritis/dt [Drug Therapy]; kidney dysfunction/si [Side Effect]; kidney malformation/si [Side Effect]; listeriosis/si [Side Effect]; liver cirrhosis/si [Side Effect]; liver failure/si [Side Effect]; liver fibrosis/di [Diagnosis]; liver fibrosis/et [Etiology]; liver fibrosis/si [Side Effect]; liver function test; liver injury/si [Side Effect]; liver toxicity/si [Side Effect]; low drug dose; lung cancer/si [Side Effect]; lymphoma/si [Side Effect]; medical personnel; medical research; melanoma/di [Diagnosis]; melanoma/dt [Drug Therapy]; melanoma/et [Etiology]; melanoma/si [Side Effect]; metabolic syndrome X; monotherapy; myalgia/si [Side Effect]; mycosis/si [Side Effect]; non insulin dependent diabetes mellitus; nonhodgkin lymphoma/et [Etiology]; nonhodgkin lymphoma/si [Side Effect]; nonhuman; opportunistic infection/si [Side Effect]; pharyngitis/si [Side Effect]; phototherapy; polyneuropathy/si [Side Effect]; polyradiculoneuropathy/si [Side Effect]; pregnancy; priority journal; progressive multifocal leukoencephalopathy/si [Side Effect]; prophylaxis; *psoriasis/dm [Disease Management]; *psoriasis/dt [Drug Therapy]; *psoriasis/th [Therapy]; psoriasis vulgaris/dt [Drug Therapy]; psoriatic arthritis/dt [Drug Therapy]; PUVA; pyoderma/dt [Drug Therapy]; rash/si [Side Effect]; relaxation training; rheumatoid arthritis/dt [Drug Therapy]; seizure/si [Side Effect]; sensorimotor neuropathy/si [Side Effect]; sepsis/si [Side Effect]; serum sickness/si [Side Effect]; side effect/si [Side Effect]; skin carcinoma/si [Side Effect]; solid tumor/si [Side Effect]; spontaneous abortion/si [Side Effect]; squamous cell carcinoma/si [Side Effect]; teratogenesis; tracheoesophageal fistula/si [Side Effect]; treatment duration; treatment response; tremor/si [Side Effect]; tuberculosis/di [Diagnosis]; tuberculosis/et [Etiology]; tuberculosis/pc [Prevention]; tuberculosis/si [Side Effect]; ultraviolet A radiation; ultraviolet B radiation; uveitis/dt [Drug Therapy]; vaccination; vertebra malformation/si [Side Effect]; virus meningitis/si [Side Effect]; virus reactivation; abatacept/ct [Clinical Trial]; abatacept/dt [Drug Therapy]; abt 874; adalimumab/ae [Adverse Drug Reaction]; adalimumab/ct [Clinical Trial]; adalimumab/cb [Drug Combination]; adalimumab/cm [Drug Comparison]; adalimumab/dt [Drug Therapy]; adalimumab/pe [Pharmacoeconomics]; adalimumab/sc [Subcutaneous Drug Administration]; alefacept/ae [Adverse Drug Reaction]; alefacept/ct [Clinical Trial]; alefacept/dt [Drug Therapy]; alefacept/pe [Pharmacoeconomics]; *antipsoriasis agent; azathioprine/ae [Adverse Drug Reaction]; azathioprine/ct [Clinical Trial]; azathioprine/cb [Drug Combination]; azathioprine/cm [Drug Comparison]; azathioprine/dt [Drug Therapy]; calcipotriol/ae [Adverse Drug Reaction]; calcipotriol/ct [Clinical Trial]; calcipotriol/cb [Drug Combination]; calcipotriol/dt [Drug Therapy]; calcipotriol/to [Drug Toxicity]; calcipotriol/pe [Pharmacoeconomics]; calcipotriol/tp [Topical Drug Administration]; chickenpox vaccine; coal tar/ae [Adverse Drug Reaction]; coal tar/ct [Clinical Trial]; coal tar/dt [Drug Therapy]; corticosteroid/ae [Adverse Drug Reaction]; corticosteroid/ct [Clinical Trial]; corticosteroid/cb [Drug Combination]; corticosteroid/dt [Drug Therapy]; corticosteroid/ar [Intraarticular Drug Administration]; corticosteroid/il [Intralesional Drug Administration]; corticosteroid/pe [Pharmacoeconomics]; corticosteroid/tp [Topical Drug Administration]; cyclosporin/ae [Adverse Drug Reaction]; cyclosporin/ct [Clinical Trial]; cyclosporin/cb [Drug Combination]; cyclosporin/cm [Drug Comparison]; cyclosporin/dt [Drug Therapy]; cyclosporin/pk [Pharmacokinetics]; dithranol/ct [Clinical Trial]; dithranol/dt [Drug Therapy]; dithranol/tp [Topical Drug Administration]; efalizumab/ae [Adverse Drug Reaction]; efalizumab/ct [Clinical Trial]; efalizumab/dt [Drug Therapy]; efalizumab/pe [Pharmacoeconomics]; efalizumab/pk [Pharmacokinetics]; etanercept/ae [Adverse Drug Reaction]; etanercept/ct [Clinical Trial]; etanercept/cb [Drug Combination]; etanercept/cm [Drug Comparison]; etanercept/dt [Drug Therapy]; etanercept/pe [Pharmacoeconomics]; etanercept/sc [Subcutaneous Drug Administration]; etretin/ae [Adverse Drug Reaction]; etretin/ct [Clinical Trial]; etretin/cb [Drug Combination]; etretin/do [Drug Dose]; etretin/dt [Drug Therapy]; etretin/po [Oral Drug Administration]; etretin/pe [Pharmacoeconomics]; hydroxychloroquine/ct [Clinical Trial]; hydroxychloroquine/dt [Drug Therapy]; hydroxyurea/ct [Clinical Trial]; hydroxyurea/cb [Drug Combination]; hydroxyurea/dt [Drug Therapy]; hydroxyurea/pe [Pharmacoeconomics]; infliximab/ae [Adverse Drug Reaction]; infliximab/ct [Clinical Trial]; infliximab/cb [Drug Combination]; infliximab/cm [Drug Comparison]; infliximab/dt [Drug Therapy]; infliximab/pe [Pharmacoeconomics]; leflunomide/ct [Clinical Trial]; leflunomide/dt [Drug Therapy]; mercaptopurine/ct [Clinical Trial]; mercaptopurine/dt [Drug Therapy]; methotrexate/ae [Adverse Drug Reaction]; methotrexate/ct [Clinical Trial]; methotrexate/cb [Drug Combination]; methotrexate/cm [Drug Comparison]; methotrexate/do [Drug Dose]; methotrexate/dt [Drug Therapy]; methotrexate/im [Intramuscular Drug Administration]; methotrexate/po [Oral Drug Administration]; methotrexate/pe [Pharmacoeconomics]; methotrexate/pk [Pharmacokinetics]; methotrexate/sc [Subcutaneous Drug Administration]; mycophenolic acid 2 morpholinoethyl ester/ae [Adverse Drug Reaction]; mycophenolic acid 2 morpholinoethyl ester/ct [Clinical Trial]; mycophenolic acid 2 morpholinoethyl ester/cm [Drug Comparison]; mycophenolic acid 2 morpholinoethyl ester/dt [Drug Therapy]; pimecrolimus/ae [Adverse Drug Reaction]; pimecrolimus/ct [Clinical Trial]; pimecrolimus/dt [Drug Therapy]; pimecrolimus/tp [Topical Drug Administration]; placebo; rituximab/ct [Clinical Trial]; rituximab/dt [Drug Therapy]; salazosulfapyridine/ct [Clinical Trial]; salazosulfapyridine/dt [Drug Therapy]; salazosulfapyridine/pe [Pharmacoeconomics]; tacrolimus/ae [Adverse Drug Reaction]; tacrolimus/ct [Clinical Trial]; tacrolimus/cm [Drug Comparison]; tacrolimus/dt [Drug Therapy]; tacrolimus/po [Oral Drug Administration]; tacrolimus/tp [Topical Drug Administration]; tazarotene/ct [Clinical Trial]; tazarotene/cb [Drug Combination]; tazarotene/dt [Drug Therapy]; tazarotene/tp [Topical Drug Administration]; tioguanine/ct [Clinical Trial]; tioguanine/dt [Drug Therapy]; tumor necrosis factor inhibitor/ae [Adverse Drug Reaction]; tumor necrosis factor inhibitor/ct [Clinical Trial]; tumor necrosis factor inhibitor/cb [Drug Combination]; tumor necrosis factor inhibitor/cm [Drug Comparison]; tumor necrosis factor inhibitor/dt [Drug Therapy]; unindexed drug; ustekinumab/ct [Clinical Trial]; ustekinumab/dt [Drug Therapy]; varicella zoster vaccine.

Drug Trade Names and Manufacturers

Ingram, Merck [United States], abt 874, varivax, zostavax: Merck [United States]

Link to the Ovid Full Text or citation:

Click here for full text options

243.

Epidemiologic characteristics of the tuberculosis cases followed up at Sanliurfa Central Tuberculosi Control Dispensary between 2001 and 2006 years. <2001-2006 Yillari arasinda sanliurfa merkez verem savas dispanserinde takip edilen tuberkuloz olgularinin epidemiyolojik ozellikleri.>

Kocakoglu S., Simsek Z., Ceylan E.

Turk Toraks Dergisi. 10 (1) (pp 9-14), 2009. Date of Publication: 2009.

AN: 2009305363

Introduction: In this study, it was aimed to evaluate the descriptive characteristics, treatment failure and related factors of tuberculosis cases followed up at Sanliurfa Central Tuberculosis Control Dispensary between 2001 and 2006. Material and Method: One thousand, one hundred and three case records could be reached of 1287 patients under treatment during these years (85.7%). Of these, 59.7% was male and the mean age was 27.9 years. Results: The rate of extrapulmonary tuberculosis (EP.TB) was 28.8%. The percentage of EP TB was significantly higher in females than in males (p<0.05). While the percentage of diagnosis by the positive sputum analysis was 48. 1 %, the diagnostic tuberculosis culture was 0.8%. Of the patients, 49.80/6 EP TB cases had organ biopsy. The percentage of the cure status of the cases with positive sputum analysis was 21.8%, and 59.5% completed the treatment successfully. Although they had positive sputum analysis, 2.6% of the cases had stopped the therapy. Other comorbid diseases were present in 8.3% of the cases. Of these, DM was the most common disease - with the rate of 41.5%. Treatment failure rate was between 17.3%6-3.3% and showed an annual decrease in rate. The treatment failure status was seen to be significantly higher in the elderly, uneducated, unemployed cases, seasonal workers, and those with side effects (p<0.05). No significant relation between gender, comorbidity, affected organ and treatment failure was found (p>0.05). Conclusion: Findings of this study showed, the necessity for improvement of the services according of the characteristics of the province and infrastructure and development of the health education program regarding health professionals and the community in order to achieve effective ana sustainable control, diagnosis and treatment of TB in Sanliurfa.

Institution

(Kocakoglu) Ispir Devlet Hastanesi, Erzurum, Turkey (Simsek) Harran Universitesi, Tip Fakultesi, Halk Sagligi Anabilim Dali, Sanliurfa, Turkey

(Ceylan) Harran Universitesi, Tip Fakultesi, Gogus Hastaliklari Anabilim Dali, Sanliurfa, Turkey

Publisher

AVES Publishing Co. (Kizilelma CAD. 5/3, Findikzade- Istanbul 34096, Turkey)

Emtree Heading

adult; article; comorbidity; culture medium; diabetes mellitus; educational status; extrapulmonary tuberculosis/di [Diagnosis]; female; geriatrics; human; *infection control; lung biopsy; major clinical study; male; risk factor; sex difference; sputum analysis; treatment failure; *tuberculosis/di [Diagnosis]; *tuberculosis/ep [Epidemiology]; unemployment; worker.

Link to the Ovid Full Text or citation:

Click here for full text options

244.

Treatment of ankylosing spondylitis and other spondyloarthritides.

Braun J., Baraliakos X.

Current Opinion in Rheumatology. 21 (4) (pp 324-334), 2009. Date of Publication: July 2009.

AN: 2009293293

Purpose of review: The management of ankylosing spondylitis and other spondyloarthritides (SpAs) such as psoriatic arthritis (PsA) is becoming more and more complex due to major advances, especially in the pharmacological therapy of these diseases. Biologics such as the anti-tumour necrosis factor agents have the potential to improve almost all outcome parameters usually assessed. The purpose of this review was to collect all available information related to the treatment of ankylosing spondylitis published in the year 2008. This was achieved by a systematic literature search in PubMed. Recent findings: There is increasing knowledge about the short-term and long-term efficacy of anti-tumour necrosis factor agents. As it stands now, radiographic progression of ankylosing spondylitis patients is not decelerated, in contrast to PsA. One major difference between the two SpAs is that ankylosing spondylitis is more an osteoproliferative disease, whereas PsA is more osteodestructive (though it certainly has both elements). Methotrexate offers no additional benefit for the axial symptoms in ankylosing spondylitis. Other biologics such as efalizumab, which is used to treat psoriasis, seems to increase the frequency of arthritis. A whole array of potential side effects has been reported. Cardiovascular comorbidity seems to be rather important for SpA patients. Summary: In this review, we especially cover not only the following themes of new clinical studies, efficacy regarding signs and symptoms, function and quality of life but also structural damage and the link between inflammation and new bone formation. The pharmacokinetics of infliximab including the formation of antibodies and the overall and long-term safety of anti-tumour necrosis factor agents is covered. © 2009 Wolters Kluwer Health Lippincott Williams & Wilkins.

Institution

(Braun, Baraliakos) Rheumazentrum Ruhrgebiet, Herne, Germany (Braun) Rheumazentrum Ruhrgebiet, Landgrafenstr. 15, 44652 Herne, Germany

Publisher

Lippincott Williams and Wilkins (530 Walnut Street,P O Box 327, Philadelphia PA 19106-3621, United States)

Emtree Heading

acute granulocytic leukemia/si [Side Effect]; add on therapy; alopecia areata/si [Side Effect]; amyotrophic lateral sclerosis/si [Side Effect]; *ankylosing spondylitis/dm [Disease Management]; *ankylosing spondylitis/dt [Drug Therapy]; *ankylosing spondylitis/rh [Rehabilitation]; *ankylosing spondylitis/su [Surgery]; *ankylosing spondylitis/th [Therapy]; ankylosis/su [Surgery]; antibody producing cell; antiinflammatory activity; arthritis/si [Side Effect]; aspergillosis/si [Side Effect]; biological therapy; cardiovascular risk; cauda equina syndrome; central nervous system infection/si [Side Effect]; clinical trial; cold injury/si [Side Effect]; comorbidity; coronary artery bypass graft; corticosteroid therapy; cost effectiveness analysis; Crohn disease/si [Side Effect]; dermatitis herpetiformis/si [Side Effect]; diabetes mellitus; diet therapy; disease course; disease exacerbation; drug effect; drug efficacy; drug megadose; drug safety; drug targeting; drug withdrawal; enthesitis/si [Side Effect]; erythema nodosum/dt [Drug Therapy]; functional assessment; group therapy; histopathology; home care; human; hypertension; immunotherapy; iridocyclitis/si [Side Effect]; keratoderma blennorrhagica/si [Side Effect]; kinesiotherapy; lichen planupilaris/si [Side Effect]; lupus vulgaris/si [Side Effect]; Medline; monarthritis/dt [Drug Therapy]; monarthritis/si [Side Effect]; multiple sclerosis/si [Side Effect]; myelitis/dt [Drug Therapy]; myelitis/si [Side Effect]; myeloid leukemia/si [Side Effect]; nevus/si [Side Effect]; nonhuman; nuclear magnetic resonance imaging; ossification; osteotomy; patient education; peripheral neuropathy/si [Side Effect]; physical capacity; physiotherapy; priority journal; prognosis; psoriasis/dt [Drug Therapy]; psoriasis/si [Side Effect]; psoriasis vulgaris/dt [Drug Therapy]; psoriasis vulgaris/si [Side Effect]; psoriatic arthritis/dr [Drug Resistance]; psoriatic arthritis/dt [Drug Therapy]; psoriatic arthritis/si [Side Effect]; pustulosis/si [Side Effect]; pustulosis palmoplantaris/dt [Drug Therapy]; pustulosis palmoplantaris/si [Side Effect]; quality of life; religion; remission; review; rheumatic disease/dt [Drug Therapy]; rheumatic disease/si [Side Effect]; rheumatoid arthritis/dt [Drug Therapy]; sacroiliitis/dt [Drug Therapy]; sarcoidosis/si [Side Effect]; skin toxicity/si [Side Effect]; spine surgery; *spondyloarthropathy/dm [Disease Management]; *spondyloarthropathy/dr [Drug Resistance]; *spondyloarthropathy/dt [Drug Therapy]; *spondyloarthropathy/rh [Rehabilitation]; *spondyloarthropathy/su [Surgery]; *spondyloarthropathy/th [Therapy]; symptomatology; systematic review; Tai Chi; temporomandibular joint disorder/dr [Drug Resistance]; temporomandibular joint disorder/dt [Drug Therapy]; total hip prosthesis; treatment duration; treatment outcome; treatment planning; treatment response; adalimumab/ae [Adverse Drug Reaction]; adalimumab/ct [Clinical Trial]; adalimumab/dt [Drug Therapy]; antinuclear antibody/ec [Endogenous Compound]; bisphosphonic acid derivative/pd [Pharmacology]; C reactive protein/ec [Endogenous Compound]; celecoxib/dt [Drug Therapy]; certolizumab pegol; corticosteroid/dt [Drug Therapy]; double stranded DNA antibody/ec [Endogenous Compound]; efalizumab/ae [Adverse Drug Reaction]; efalizumab/dt [Drug Therapy]; etanercept/ae [Adverse Drug Reaction]; etanercept/dt [Drug Therapy]; etanercept/pd [Pharmacology]; etoricoxib/dt [Drug Therapy]; glucocorticoid/dt [Drug Therapy]; glucocorticoid/ar [Intraarticular Drug Administration]; golimumab/ct [Clinical Trial]; golimumab/dt [Drug Therapy]; hydroxymethylglutaryl coenzyme A reductase inhibitor/pd [Pharmacology]; infliximab/ae [Adverse Drug Reaction]; infliximab/cb [Drug Combination]; infliximab/dt [Drug Therapy]; infliximab/ar [Intraarticular Drug Administration]; low density lipoprotein cholesterol/ec [Endogenous Compound]; methotrexate/cb [Drug Combination]; methotrexate/dt [Drug Therapy]; nonsteroid antiinflammatory agent/dt [Drug Therapy]; placebo; radium 224/ae [Adverse Drug Reaction]; radium 224/dt [Drug Therapy]; rituximab/dt [Drug Therapy]; tumor necrosis factor inhibitor/ae [Adverse Drug Reaction]; tumor necrosis factor inhibitor/ct [Clinical Trial]; tumor necrosis factor inhibitor/cb [Drug Combination]; tumor necrosis factor inhibitor/dt [Drug Therapy].

Link to the Ovid Full Text or citation:

Click here for full text options

245.

Impact of diabetes mellitus on treatment outcomes of patients with active tuberculosis.

Dooley K.E., Tang T., Golub J.E., Dorman S.E., Cronin W.

American Journal of Tropical Medicine and Hygiene. 80 (4) (pp 634-639), 2009. Date of Publication: April 2009.

AN: 2009288272

Diabetes mellitus (DM) is an emerging chronic health condition of developed and developing countries. We conducted a retrospective cohort study of patients with active, culture-confirmed tuberculosis (TB) in Maryland to determine the impact of DM on TB treatment outcomes. Of 297 TB patients, 42 (14%) had DM. Patients with diabetes had 2.0 times higher odds of death than patients without diabetes (95% confidence interval [CI] 0.74-5.2, P - 0.18). Adjusting for human immunodeficiency virus (HIV), age, weight, and foreign birth, the odds of death were 6.5 times higher in patients with diabetes than patients without diabetes (95% CI 1.1-38.0, P = 0.039). In pulmonary TB patients, time to sputum culture conversion was longer in patients with diabetes than patients without diabetes (median 49 versus 39 days, P = 0.09). Two-month culture conversion proportions were similar (70% and 69%). Treatment failure occurred in 4.1% of patients without diabetes and 6.7% of patients with diabetes (P = 0.51). In conclusion, DM was a risk factor for death in Maryland TB patients. There was a trend toward increased time to culture conversion; two-month culture conversion proportions, however, were similar. Copyright © 2009 by The American Society of Tropical Medicine and Hygiene.

Institution

(Dooley) Division of TB Control, Refugee and Migrant Health, Maryland Department of Health and Mental Hygiene, 201 W. Preston Street, Baltimore, MD 21201 (Tang) Division of Clinical Pharmacology and Infectious Diseases, Johns Hopkins University, School of Medicine, 600 N. Wolfe Street Osier 527, Baltimore, MD 21287

(Golub) Johns Hopkins University Bloomberg, School of Medicine, 615 N.Wolfe Street, Baltimore, MD 21205

(Dorman) Center for Tuberculosis Research, Johns Hopkins University, School of Medicine, 1550 Orleans Street, Baltimore, MD 21231

(Cronin) Center for Tuberculosis Research, Johns Hopkins University, School of Medicine

Publisher

American Society of Tropical Medicine and Hygiene (111 Deer Lake Road, Suite 100,Deerfield, Illinois 60015, United States)

Emtree Heading

adult; article; bacterium culture; cohort analysis; confidence interval; controlled study; *diabetes mellitus; disease association; drug sensitivity; ethnicity; female; human; Human immunodeficiency virus; lung tuberculosis/dt [Drug Therapy]; major clinical study; male; miliary tuberculosis; mortality; multidrug resistance; Mycobacterium tuberculosis; retrospective study; risk assessment; risk factor; sputum analysis; treatment failure; treatment outcome; treatment response; *tuberculosis/dt [Drug Therapy]; United States; tuberculostatic agent/dt [Drug Therapy].

Link to the Ovid Full Text or citation:

Click here for full text options

246.

Risk Factors for Early Mortality and Morbidity After Pneumonectomy: A Reappraisal.

Mansour Z., Kochetkova E.A., Santelmo N., Meyer P., Wihlm J.-M., Quoix E., Massard G.

Annals of Thoracic Surgery. 88 (6) (pp 1737-1743), 2009. Date of Publication: December 2009.

AN: 2009631340

Background: Pneumonectomy remains a high-risk procedure. Comprehensive patient selection should be based on analysis of proven risk factors. Methods: The records of 323 pneumonectomy patients were retrospectively reviewed. Multiple demographic data were collected. End points were operative mortality at 30 and at 90 days, major procedurally related complications, and cardiovascular events. Univariate and multivariate statistical analyses were performed. Results: Smoking habits, chronic obstructive pulmonary disease (COPD) status, induction chemotherapy status, diabetes, and obesity had no statistical influence on short-term outcomes. After right pneumonectomy, 30-day mortality (p = 0.045) and the incidence of bronchopleural fistulas (p = 0.009) were increased. Multivariate analysis for postoperative bronchopleural fistulas discovered that right pneumonectomies are the sole risk factor (p = 0.015). Univariate analysis for postoperative atrial fibrillation showed that male gender, age 70 and older, hypertension, and dyslipidemia are risk factors. Multivariate analysis found no definite risk factor. Patients with coronary artery disease had more postoperative cardiovascular events (p = 0.003). Among patients free of coronary artery disease, COPD led to an increased 90-day mortality rate (p = 0.028). Conclusions: Patients with right pneumonectomies are at increased risk. Postoperative cardiovascular events are more frequent in coronary artery disease patients. COPD is a risk factor in patients free of coronary disease. © 2009 The Society of Thoracic Surgeons.

Institution

(Mansour, Kochetkova, Santelmo, Wihlm, Massard) Service de Chirurgie Thoracique, Hopitaux Universitaires de Strasbourg, Strasbourg, France (Meyer) Departement de Biostatistiques, Hopitaux Universitaires de Strasbourg, Strasbourg, France

(Quoix) Departement de Pneumologie, Hopitaux Universitaires de Strasbourg, Strasbourg, France

Publisher

Elsevier USA (6277 Sea Harbor Drive, Orlando FL 32862 8239, United States)

Emtree Heading

adult; adult respiratory distress syndrome/co [Complication]; age distribution; aged; artery disease; article; bronchiectasis/su [Surgery]; bronchopleural fistula/co [Complication]; carcinoid/su [Surgery]; cardiovascular disease/co [Complication]; chemotherapy; chronic obstructive lung disease; coronary artery disease; diabetes mellitus; dyslipidemia; empyema/co [Complication]; female; heart atrium fibrillation/co [Complication]; human; hypertension; lung cyst/su [Surgery]; lung metastasis/su [Surgery]; *lung resection; lung tuberculosis/su [Surgery]; lymphoma/su [Surgery]; major clinical study; male; medical record review; mesothelioma/su [Surgery]; *morbidity; obesity; postoperative period; priority journal; retrospective study; *risk factor; sex difference; smoking habit; *surgical mortality; surgical patient; surgical risk; treatment outcome.

Link to the Ovid Full Text or citation:

Click here for full text options

247.

Tuberculosis and diabetes mellitus: convergence of two epidemics.

Dooley K.E., Chaisson R.E.

The Lancet Infectious Diseases. 9 (12) (pp 737-746), 2009. Date of Publication: December 2009.

AN: 2009629628

The link between diabetes mellitus and tuberculosis has been recognised for centuries. In recent decades, tuberculosis incidence has declined in high-income countries, but incidence remains high in countries that have high rates of infection with HIV, high prevalence of malnutrition and crowded living conditions, or poor tuberculosis control infrastructure. At the same time, diabetes mellitus prevalence is soaring globally, fuelled by obesity. There is growing evidence that diabetes mellitus is an important risk factor for tuberculosis and might affect disease presentation and treatment response. Furthermore, tuberculosis might induce glucose intolerance and worsen glycaemic control in people with diabetes. We review the epidemiology of the tuberculosis and diabetes epidemics, and provide a synopsis of the evidence for the role of diabetes mellitus in susceptibility to, clinical presentation of, and response to treatment for tuberculosis. In addition, we review potential mechanisms by which diabetes mellitus can cause tuberculosis, the effects of tuberculosis on diabetic control, and pharmacokinetic issues related to the co-management of diabetes and tuberculosis. © 2009 Elsevier Ltd. All rights reserved.

Institution

(Dooley, Chaisson) Division of Infectious Diseases, Center for Tuberculosis Research, Johns Hopkins University School of Medicine, Baltimore, MD, United States (Dooley) Division of Clinical Pharmacology, Johns Hopkins University School of Medicine, Baltimore, MD, United States

Publisher

Lancet Publishing Group (84 Theobald's Road, London WC1X 8RR, United Kingdom)

Emtree Heading

area under the curve; clinical feature; cultural anthropology; developed country; developing country; diabetes control; *diabetes mellitus/co [Complication]; *diabetes mellitus/dm [Disease Management]; *diabetes mellitus/dt [Drug Therapy]; *diabetes mellitus/ep [Epidemiology]; *diabetes mellitus/et [Etiology]; disease association; disease severity; drug metabolism; epidemic; evidence based medicine; glucose intolerance; glucose tolerance; glycemic control; health care cost; human; hyperglycemia/si [Side Effect]; hyperinsulinemia/si [Side Effect]; incidence; insulin dependent diabetes mellitus/dt [Drug Therapy]; Mycobacterium tuberculosis; non insulin dependent diabetes mellitus/dt [Drug Therapy]; nonhuman; outcome assessment; priority journal; radiodiagnosis; review; risk factor; thorax radiography; treatment failure; treatment response; *tuberculosis/co [Complication]; *tuberculosis/di [Diagnosis]; *tuberculosis/dm [Disease Management]; *tuberculosis/dt [Drug Therapy]; *tuberculosis/ep [Epidemiology]; *tuberculosis/et [Etiology]; 2,4 thiazolidinedione derivative/dt [Drug Therapy]; glibenclamide/it [Drug Interaction]; glibenclamide/dt [Drug Therapy]; glibenclamide/po [Oral Drug Administration]; glibenclamide/pk [Pharmacokinetics]; glipizide/it [Drug Interaction]; glipizide/dt [Drug Therapy]; glipizide/po [Oral Drug Administration]; glipizide/pk [Pharmacokinetics]; insulin/it [Drug Interaction]; insulin/dt [Drug Therapy]; isoniazid/dt [Drug Therapy]; nateglinide/it [Drug Interaction]; nateglinide/dt [Drug Therapy]; pioglitazone/it [Drug Interaction]; pioglitazone/dt [Drug Therapy]; pyridoxine/dt [Drug Therapy]; repaglinide/it [Drug Interaction]; repaglinide/dt [Drug Therapy]; repaglinide/pk [Pharmacokinetics]; rifampicin/ae [Adverse Drug Reaction]; rifampicin/cr [Drug Concentration]; rifampicin/it [Drug Interaction]; rifampicin/dt [Drug Therapy]; rifampicin/pk [Pharmacokinetics]; rifampicin/pd [Pharmacology]; rosiglitazone/it [Drug Interaction]; rosiglitazone/dt [Drug Therapy]; rosiglitazone/pk [Pharmacokinetics]; sulfonylurea/it [Drug Interaction]; sulfonylurea/dt [Drug Therapy].

Link to the Ovid Full Text or citation:

Click here for full text options

248.

Association of tuberculosis and diabetes mellitus: Immunological mechanisms involving susceptibility. <Asociacion de tuberculosis y diabetes mellitus: Mecanismos inmunologicos involucrados en la susceptibilidad.>

Gonzalez-hernandez Y., Sada Diaz E., Escobar-Gutierrez A., Munos Torrico M., Torres Rojas M.

Revista del Instituto Nacional de Enfermedades Respiratorias. 22 (1) (pp 48-55), 2009. Date of Publication: 2009.

AN: 2009619034

Tuberculosis (TB) is one of the most important infectious diseases as a cause of death worldwide. Diabetes mellitus (DM) constitutes a major risk factor for TB, and in Mexican population its role as risk factor seems to be even more relevant than HIV infection. Studies in patients with type 2 DM (DM2) have shown that the risk of developing TB is 1.8 to 8 times higher than in non-diabetic population. It has also been shown that the presence of DM2 increases the failure rate to anti-tuberculous drugs and the susceptibility to infection with drug-resistant strains of M. tuberculosis. On the other hand, immunological studies in patients with DM have revealed defects in polymorphonuclear leukocytes functions (phagocytosis, opsonic activity by complement and intracellular bactericidal activity), as well as the release of IL-1 and production of IL-6, TNF-alpha and IFN-gamma by mononuclear cells. In experimental animal models, diminished production of cytokines such as IL-12, IL-6 and IL-10 is associated with high concentrations of blood glucose, suggesting a possible role of hyperglycemia in the immune response abnormality causative of increased susceptibility to infections. Alveolar macrophages from DM2 patients who develop active TB display less activation and decreased in IFN-gamma production. These data suggest that alterations in the innate and adaptive immune responses in patients with DM participate in the increased susceptibility to infection and in the development of active TB.

Institution

(Gonzalez-hernandez, Sada Diaz, Munos Torrico, Torres Rojas) Departamento de Microbiologia, Instituto Nacional de Enfermedades Respiratorias Ismael Cosio Villegas, Calzada de Tlalpan 4502, colonia Seccion XVI, Mexico, D.F., 4080, Mexico (Escobar-Gutierrez) Departamento de Investigaciones Inmunologicas, Instituto de Diagnostico Y Referencia Epidemiologicos

Publisher

Instituto Nacional de Enfermedades Respiratorias (Calzada de Tlalpan 4502, Mexico D.F. CP 14080, Mexico)

Emtree Heading

adaptive immunity; bactericidal activity; cause of death; cytokine production; *diabetes mellitus; disease association; *disease predisposition; drug treatment failure; glucose blood level; human; Human immunodeficiency virus infection; hyperglycemia; immune response; innate immunity; leukocyte function; lung alveolus macrophage; mononuclear cell; Mycobacterium tuberculosis; neutrophil; non insulin dependent diabetes mellitus; nonhuman; phagocytosis; review; risk factor; *tuberculosis/dr [Drug Resistance]; *tuberculosis/dt [Drug Therapy]; gamma interferon/ec [Endogenous Compound]; glucose/ec [Endogenous Compound]; interleukin 10/ec [Endogenous Compound]; interleukin 12/ec [Endogenous Compound]; interleukin 6/ec [Endogenous Compound]; tuberculostatic agent/dt [Drug Therapy]; tumor necrosis factor alpha/ec [Endogenous Compound].

Link to the Ovid Full Text or citation:

Click here for full text options

249.

PENTA 2009 guidelines for the use of antiretroviral therapy in paediatric HIV-1 infection.

Welch S.

HIV Medicine. 10 (10) (pp 591-613), 2009. Date of Publication: November 2009.

AN: 2009590654

PENTA Guidelines aim to provide practical recommendations for treating children with HIV infection in Europe. Changes to guidance since 2004 have been informed by new evidence and by expectations of better outcomes following the ongoing success of antiretroviral therapy (ART). Participation in PENTA trials of simplifying treatment is encouraged. The main changes are in the following sections:'When to start ART': Treatment is recommended for all infants, and at higher CD4 cell counts and percentages in older children, in line with changes to adult guidelines. The number of age bands has been reduced to simplify and harmonize with other paediatric guidelines. Greater emphasis is placed on CD4 cell count in children over 5 years, and guidance is provided where CD4% and CD4 criteria differ.'What to start with': A three-drug regimen of two nucleoside reverse transcriptase inhibitors (NRTIs) with either a nonnucleoside reverse transcriptase inhibitor (NNRTI) or a boosted protease inhibitor (PI) remains the first choice combination. Lamivudine and abacavir are the NRTI backbone of choice for most children, based on long-term follow-up in the PENTA 5 trial. Stavudine is no longer recommended. Whether to start with an NNRTI or PI remains unclear, but PENPACT 1 trial results in 2009 may help to inform this. All PIs should be ritonavir boosted.Recommendations on use of resistance testing, therapeutic drug monitoring and HLA testing draw from data in adults and from European paediatric cohort studies.Recently updated US and WHO paediatric guidelines provide more detailed review of the evidence base. Differences between guidelines are highlighted and explained. © 2009 British HIV Association.

Institution

(Welch) Department of Paediatrics, Heartlands Hospital, Bordesley Green East, Birmingham B9 5SS, United Kingdom

Publisher

Blackwell Publishing Ltd (9600 Garsington Road, Oxford OX4 2XG, United Kingdom)

Emtree Heading

abdominal pain/si [Side Effect]; anemia/si [Side Effect]; antiviral resistance; article; bone demineralization/si [Side Effect]; brain hemorrhage/si [Side Effect]; CD4+ T lymphocyte; child; chronic liver disease/si [Side Effect]; clinical protocol; clinical trial; diabetes mellitus/si [Side Effect]; diagnostic test; diarrhea/si [Side Effect]; drowsiness/si [Side Effect]; drug dose increase; drug efficacy; drug eruption/si [Side Effect]; drug fever/si [Side Effect]; drug formulation; drug induced headache/si [Side Effect]; drug monitoring; drug substitution; drug treatment failure; drug use; drug withdrawal; Europe; fatigue/si [Side Effect]; flatulence/si [Side Effect]; flushing; follow up; gastrointestinal symptom/si [Side Effect]; granulocytopenia/si [Side Effect]; hepatitis/si [Side Effect]; hepatitis B/si [Side Effect]; hepatitis C; human; Human immunodeficiency virus 1; *Human immunodeficiency virus 1 infection/di [Diagnosis]; *Human immunodeficiency virus 1 infection/dt [Drug Therapy]; hypercholesterolemia/si [Side Effect]; hyperlipidemia/si [Side Effect]; jaundice/si [Side Effect]; kidney tubule disorder/si [Side Effect]; lactic acidosis/si [Side Effect]; lipoatrophy/si [Side Effect]; lipodystrophy/si [Side Effect]; liver dysfunction/si [Side Effect]; liver toxicity/si [Side Effect]; lymphocyte count; mood; myopathy/si [Side Effect]; nail pigmentation/si [Side Effect]; nausea/si [Side Effect]; neuropathy/si [Side Effect]; nonhuman; opportunistic infection/dt [Drug Therapy]; opportunistic infection/pc [Prevention]; oral paresthesia/si [Side Effect]; pancreatitis/si [Side Effect]; patient compliance; pediatrics; peripheral neuropathy/si [Side Effect]; Pneumocystis pneumonia/dt [Drug Therapy]; Pneumocystis pneumonia/pc [Prevention]; *practice guideline; priority journal; prophylaxis; pruritus/si [Side Effect]; respiratory tract disease/si [Side Effect]; side effect/si [Side Effect]; skin discoloration/si [Side Effect]; steatosis/si [Side Effect]; Stevens Johnson syndrome/si [Side Effect]; tuberculosis; vivid dream/si [Side Effect]; vomiting/si [Side Effect]; abacavir/ae [Adverse Drug Reaction]; abacavir/ct [Clinical Trial]; abacavir/cb [Drug Combination]; abacavir/do [Drug Dose]; abacavir/dt [Drug Therapy]; abacavir/pr [Pharmaceutics]; abacavir plus lamivudine/ct [Clinical Trial]; abacavir plus lamivudine/do [Drug Dose]; abacavir plus lamivudine/dt [Drug Therapy]; abacavir plus lamivudine plus zidovudine/ct [Clinical Trial]; abacavir plus lamivudine plus zidovudine/do [Drug Dose]; abacavir plus lamivudine plus zidovudine/dt [Drug Therapy]; *antiretrovirus agent/ct [Clinical Trial]; *antiretrovirus agent/dt [Drug Therapy]; atazanavir/ae [Adverse Drug Reaction]; atazanavir/ct [Clinical Trial]; atazanavir/cb [Drug Combination]; atazanavir/do [Drug Dose]; atazanavir/dt [Drug Therapy]; atazanavir/pr [Pharmaceutics]; cotrimoxazole/do [Drug Dose]; cotrimoxazole/dt [Drug Therapy]; darunavir/ae [Adverse Drug Reaction]; darunavir/ct [Clinical Trial]; darunavir/cb [Drug Combination]; darunavir/do [Drug Dose]; darunavir/dt [Drug Therapy]; darunavir/pr [Pharmaceutics]; didanosine/ae [Adverse Drug Reaction]; didanosine/ct [Clinical Trial]; didanosine/cb [Drug Combination]; didanosine/do [Drug Dose]; didanosine/dt [Drug Therapy]; didanosine/pr [Pharmaceutics]; efavirenz/ae [Adverse Drug Reaction]; efavirenz/ct [Clinical Trial]; efavirenz/cb [Drug Combination]; efavirenz/do [Drug Dose]; efavirenz/dt [Drug Therapy]; efavirenz/pr [Pharmaceutics]; efavirenz plus emtricitabine plus tenofovir disoproxil/ct [Clinical Trial]; efavirenz plus emtricitabine plus tenofovir disoproxil/dt [Drug Therapy]; emtricitabine/ae [Adverse Drug Reaction]; emtricitabine/ct [Clinical Trial]; emtricitabine/cb [Drug Combination]; emtricitabine/do [Drug Dose]; emtricitabine/dt [Drug Therapy]; emtricitabine/pr [Pharmaceutics]; emtricitabine plus tenofovir disoproxil/ct [Clinical Trial]; emtricitabine plus tenofovir disoproxil/cb [Drug Combination]; emtricitabine plus tenofovir disoproxil/do [Drug Dose]; emtricitabine plus tenofovir disoproxil/dt [Drug Therapy]; etravirine/ae [Adverse Drug Reaction]; etravirine/ct [Clinical Trial]; etravirine/do [Drug Dose]; etravirine/dt [Drug Therapy]; etravirine/pr [Pharmaceutics]; fosamprenavir/ae [Adverse Drug Reaction]; fosamprenavir/ct [Clinical Trial]; fosamprenavir/cb [Drug Combination]; fosamprenavir/do [Drug Dose]; fosamprenavir/dt [Drug Therapy]; fosamprenavir/pr [Pharmaceutics]; lamivudine/ae [Adverse Drug Reaction]; lamivudine/ct [Clinical Trial]; lamivudine/cb [Drug Combination]; lamivudine/do [Drug Dose]; lamivudine/dt [Drug Therapy]; lamivudine/pr [Pharmaceutics]; lamivudine plus zidovudine/ct [Clinical Trial]; lamivudine plus zidovudine/do [Drug Dose]; lamivudine plus zidovudine/dt [Drug Therapy]; lopinavir plus ritonavir/ae [Adverse Drug Reaction]; lopinavir plus ritonavir/ct [Clinical Trial]; lopinavir plus ritonavir/cb [Drug Combination]; lopinavir plus ritonavir/do [Drug Dose]; lopinavir plus ritonavir/dt [Drug Therapy]; nevirapine/ae [Adverse Drug Reaction]; nevirapine/ct [Clinical Trial]; nevirapine/cb [Drug Combination]; nevirapine/do [Drug Dose]; nevirapine/dt [Drug Therapy]; nevirapine/pr [Pharmaceutics]; nucleoside analog/ct [Clinical Trial]; nucleoside analog/dt [Drug Therapy]; proteinase inhibitor/ae [Adverse Drug Reaction]; proteinase inhibitor/ct [Clinical Trial]; proteinase inhibitor/dt [Drug Therapy]; proteinase inhibitor/pr [Pharmaceutics]; rilpivirine/ct [Clinical Trial]; rilpivirine/do [Drug Dose]; rilpivirine/dt [Drug Therapy]; ritonavir/ae [Adverse Drug Reaction]; ritonavir/ct [Clinical Trial]; ritonavir/cb [Drug Combination]; ritonavir/do [Drug Dose]; ritonavir/dt [Drug Therapy]; ritonavir/pr [Pharmaceutics]; RNA directed DNA polymerase inhibitor/ae [Adverse Drug Reaction]; RNA directed DNA polymerase inhibitor/ct [Clinical Trial]; RNA directed DNA polymerase inhibitor/dt [Drug Therapy]; saquinavir/ae [Adverse Drug Reaction]; saquinavir/ct [Clinical Trial]; saquinavir/cb [Drug Combination]; saquinavir/do [Drug Dose]; saquinavir/dt [Drug Therapy]; saquinavir/pr [Pharmaceutics]; stavudine/ae [Adverse Drug Reaction]; stavudine/ct [Clinical Trial]; stavudine/cb [Drug Combination]; stavudine/do [Drug Dose]; stavudine/dt [Drug Therapy]; stavudine/pr [Pharmaceutics]; tenofovir/ct [Clinical Trial]; tenofovir/cb [Drug Combination]; tenofovir/dt [Drug Therapy]; tenofovir disoproxil/ae [Adverse Drug Reaction]; tenofovir disoproxil/ct [Clinical Trial]; tenofovir disoproxil/do [Drug Dose]; tenofovir disoproxil/dt [Drug Therapy]; tenofovir disoproxil/pr [Pharmaceutics]; tipranavir/ae [Adverse Drug Reaction]; tipranavir/ct [Clinical Trial]; tipranavir/do [Drug Dose]; tipranavir/dt [Drug Therapy]; unindexed drug; zidovudine/ae [Adverse Drug Reaction]; zidovudine/ct [Clinical Trial]; zidovudine/cb [Drug Combination]; zidovudine/do [Drug Dose]; zidovudine/dt [Drug Therapy]; zidovudine/pr [Pharmaceutics].

Drug Trade Names and Manufacturers

Abbott, Bms, Boehringer Ingelheim, Boehringer, Gilead, Glaxo SmithKline, Hoffmann La Roche, Janssen Cilag, Merck Sharp and Dohme, Tibotec, aptivus: Boehringer, atripla: Bms, atripla: Gilead, bactrim: Hoffmann La Roche, combivir: Glaxo SmithKline, emtriva: Gilead, epivir: Glaxo SmithKline, intelence: Janssen Cilag, intelence: Tibotec, invirase: Hoffmann La Roche, kaletra: Abbott, kivexa: Glaxo SmithKline, norvir: Abbott, prezista: Janssen Cilag, prezista: Tibotec, retrovir: Glaxo SmithKline, reyataz: Bms, septrin: Glaxo SmithKline, stocrin: Merck Sharp and Dohme, sustiva: Bms, telzir: Glaxo SmithKline, tmc 125, trizivir: Glaxo SmithKline, truvada: Gilead, videx: Bms, viramune: Boehringer Ingelheim, zeffix: Glaxo SmithKline, zerit: Bms, ziagen: Glaxo SmithKline

Link to the Ovid Full Text or citation:

Click here for full text options

250.

CTLA-4Ig: Uses and future directions.

Ben-Shoshan M.

Recent Patents on Inflammation and Allergy Drug Discovery. 3 (2) (pp 132-142), 2009. Date of Publication: 2009.

AN: 2009579029

Cytotoxic lymphocyte-associated molecule-4 (CTLA-4, CD152) is a member of the CD28 receptor family. Blocking CD28 interaction with its ligands through the use of CTLA-4Ig might contribute to better control of dysregulated immune response processes. The ligands binding to CTLA-4 are the B7 family members, B7-1 (CD80) and B7-2 (CD86). CTLA-4Ig is now a Food and Drug Administration-approved drug for use treating patients with Rheumatoid arthritis (RA) but its use is explored also in other autoimmune diseases, transplantation as well as allergic diseases. Patents related to CTLA-4 function as well as possible clinical applications are discussed in this paper.

Institution

(Ben-Shoshan) Department of Pediatric Allergy and Immunology, McGill University Health Center, Montreal, QC, Canada (Ben-Shoshan) 418 Claremont, Montreal, QC, Canada

Publisher

Bentham Science Publishers B.V. (P.O. Box 294, Bussum 1400 AG, Netherlands)

Emtree Heading

acute graft rejection/co [Complication]; acute graft rejection/dt [Drug Therapy]; acute graft rejection/pc [Prevention]; advanced cancer/dt [Drug Therapy]; allergic asthma/et [Etiology]; allograft; antineoplastic activity; apoptosis; article; autoimmune disease/et [Etiology]; autoimmune disease/pc [Prevention]; autoimmune thyroiditis/et [Etiology]; autoimmunity; binding competition; chronic obstructive lung disease/si [Side Effect]; clinical trial; colon adenoma/si [Side Effect]; comorbidity; coughing/si [Side Effect]; cytokine production; demyelinating disease/si [Side Effect]; diabetes mellitus/dt [Drug Therapy]; diabetes mellitus/et [Etiology]; dizziness/si [Side Effect]; drug dose comparison; drug efficacy; drug fatality/si [Side Effect]; drug hypersensitivity/si [Side Effect]; drug induced headache/si [Side Effect]; drug mechanism; drug potentiation; drug safety; drug structure; drug substitution; drug treatment failure; drug withdrawal; dyspnea/si [Side Effect]; food allergy/et [Etiology]; graft survival; heart arrest/si [Side Effect]; human; immunogenicity; immunomodulation; immunopathogenesis; immunoregulation; immunosuppressive treatment; intestine ischemia/si [Side Effect]; keratoconjunctivitis sicca/si [Side Effect]; lupus erythematosus nephritis/dt [Drug Therapy]; lymphocyte activation; major histocompatibility complex; melanoma/dt [Drug Therapy]; multiple sclerosis/si [Side Effect]; musculoskeletal disease/si [Side Effect]; nonhuman; nose congestion/si [Side Effect]; onset age; opportunistic infection/si [Side Effect]; priority journal; psoriasis/dt [Drug Therapy]; rheumatoid arthritis/dt [Drug Therapy]; risk reduction; skin carcinoma/si [Side Effect]; squamous cell carcinoma/si [Side Effect]; systemic lupus erythematosus/dt [Drug Therapy]; systemic lupus erythematosus/et [Etiology]; tuberculosis/si [Side Effect]; unspecified side effect/si [Side Effect]; upper respiratory tract infection/si [Side Effect]; vasculitis/si [Side Effect]; *abatacept/ae [Adverse Drug Reaction]; *abatacept/ct [Clinical Trial]; *abatacept/cb [Drug Combination]; *abatacept/cm [Drug Comparison]; *abatacept/do [Drug Dose]; *abatacept/it [Drug Interaction]; *abatacept/dt [Drug Therapy]; *abatacept/pd [Pharmacology]; B7 antigen/ec [Endogenous Compound]; belatacept/ct [Clinical Trial]; belatacept/cm [Drug Comparison]; belatacept/dt [Drug Therapy]; calcineurin inhibitor/cm [Drug Comparison]; corticosteroid/ae [Adverse Drug Reaction]; corticosteroid/cb [Drug Combination]; corticosteroid/it [Drug Interaction]; cyclophosphamide/ae [Adverse Drug Reaction]; cyclophosphamide/cb [Drug Combination]; cyclophosphamide/it [Drug Interaction]; cyclophosphamide/dt [Drug Therapy]; cyclosporin/cm [Drug Comparison]; cyclosporin/dt [Drug Therapy]; cytotoxic T lymphocyte antigen 4/ec [Endogenous Compound]; disease modifying antirheumatic drug/dt [Drug Therapy]; hydroxychloroquine/dt [Drug Therapy]; infliximab/cm [Drug Comparison]; leflunomide/dt [Drug Therapy]; methotrexate/cb [Drug Combination]; methotrexate/dt [Drug Therapy]; placebo; tumor necrosis factor alpha inhibitor/dt [Drug Therapy].

Drug Trade Names and Manufacturers

Bristol Myers Squibb

Link to the Ovid Full Text or citation:

Click here for full text options

251.

Vitrectomy for endogenous fungal endophthalmitis.

Shen X., Xu G.

Ocular Immunology and Inflammation. 17 (3) (pp 148-152), 2009. Date of Publication: June 2009.

AN: 2009432889

Purpose: The purpose of this study was to evaluate the predisposing risk factors and treatment outcomes of endogenous fungal endophthalmitis (EFE). Methods: Records of 25 patients (29 eyes), diagnosed with EFE and treated at Shanghai Eye, Ear, Nose Throat Hospital from January 2002 to December 2006, were retrospectively reviewed. Four patients had bilateral disease. Final visual acuity and recurrence of EFE were evaluated. Results: Of these 25 patients diagnosed with EFE, 20 patients (22 eyes) were treated by pars plana vitrectomy. Of the remaining 5 patients (7 eyes), 3 cases (4 eyes) were cured by antifungal drugs through systemic treatment and intravitreal injection, but another 2 cases (3 eyes) were only given intravitreal injection because of severe general conditions. Of the 22 eyes that underwent pars plana vitrectomy, 16 (73%) eyes gained visual acuity of counting fingers or better. Recurrent EFE was happened in 2 eyes. Vitrectomy was repeated in 1 eye, another was subsequently enucleated due to phthisis. Vitreous culture-proven or smear-proven EFE occurred in 27 or 2 eyes, respectively. Candida albicans occurred in 17 of 29 eyes (59%), and other causative organisms were yeast fungus (3 eyes), Aspergillus niger (3 eyes), Actinomyces (2 eyes),Aspergillus flavus (1 eye), and Fusarium (1 eye). Conclusion: Candida albicans were the most common causative organisms in EFE. The most common predisposing risk factors include recent major operation and intravenous administration in rural settings. Most patients with EFE will gain useful vision (counting fingers) after pars plana vitrectomy.

Institution

(Shen) Department of Ophthalmology, Ruijin Hospital, Jiaotong University, Ruijin 2nd Road 197, Shanghai, 200025, China (Xu) Department of Ophthalmology, Eye, Ear, Nose, Throat (EENT) Hospital, Fudan University, Shanghai, China

Publisher

Informa Healthcare (69-77 Paul Street, London EC2A 4LQ, United Kingdom)

Emtree Heading

abdomen liomyosarcoma; abortion; abscess; Actinomyces; adult; article; Aspergillus flavus; Aspergillus niger; blood smear; breast cancer; buttock abscess; Candida albicans; ciliary disk; clinical article; diabetes mellitus; disease predisposition; disseminated intravascular clotting; *endogenous fungal endophthalmitis/dt [Drug Therapy]; *endogenous fungal endophthalmitis/su [Surgery]; *eye inflammation/dt [Drug Therapy]; *eye inflammation/su [Surgery]; female; Fusarium; hemodialysis; human; intravenous drug abuse; kidney graft; lensectomy; liver abscess; liver cancer; lung tuberculosis; lymphoma; male; *mycosis/dt [Drug Therapy]; *mycosis/su [Surgery]; myosarcoma; nonhuman; observational study; pancreas cancer; pancreatitis; postoperative period; puerperium; recurrent disease/co [Complication]; recurrent disease/su [Surgery]; reoperation; retinitis; retrospective study; risk factor; stomach cancer; systemic therapy; tooth extraction; treatment outcome; ulcerative colitis; visual acuity; *vitrectomy; yeast; amphotericin B/cb [Drug Combination]; amphotericin B/dt [Drug Therapy]; amphotericin B/vi [Intravitreal Drug Administration]; fluconazole/ad [Drug Administration]; fluconazole/cb [Drug Combination]; fluconazole/dt [Drug Therapy]; silicone oil.

Link to the Ovid Full Text or citation:

Click here for full text options

252.

Diabetes and the risk of tuberculosis: A neglected threat to public health?.

Stevenson C.R., Critchley J.A., Forouhi N.G., Roglic G., Williams B.G., Dye C., Unwin N.C.

Chronic Illness. 3 (3) (pp 228-245), 2007. Date of Publication: September 2007.

AN: 2008318588

Objectives: Tuberculosis (TB) remains a major global public health problem. In the past, a relationship between TB and diabetes mellitus (DM) was recognized, and its importance was acknowledged through joint treatment clinics. However, this is rarely highlighted in current research or control priorities. This paper aims to evaluate the evidence for an association between these two diseases. Methods: A Medline literature search was undertaken, supplemented by checking references and contacting experts. We critically appraised studies that quantified the association between TB and DM, and were published after 1995. We assessed study quality according to criteria such as sample size, method of selection of cases and controls, losses to follow-up, quality and method of control of confounding, and summarized the results narratively and in tabular form. Results: All studies identified statistically significant and clinically important associations, with the increase in risk or odds of TB varying between 1.5- and 7.8-fold for those with DM. Risk was highest at younger ages. Most studies had not measured and controlled adequately for potential major confounders. Discussion: There is strong evidence for an association between TB and DM, which has potential public health implications. Further well-designed studies are needed to assess the magnitude precisely. © SAGE Publications 2007.

Institution

(Stevenson, Forouhi) Medical Research Council Epidemiology Unit, Elsie Widdowson Laboratory, Fulbourn Road, Cambridge CB1 9NL, United Kingdom (Critchley, Unwin) Institute of Health and Society, Newcastle University, William Leech Building, Newcastle upon Tyne NE2 4HH, United Kingdom

(Roglic) Department of Chronic Diseases and Health Promotion, World Health Organization, Avenue Appia 20, 1211 Geneva 27, Switzerland

(Williams, Dye) Tuberculosis Monitoring and Evaluation, World Health Organization, Avenue Appia 20, 1211 Geneva 27, Switzerland

Publisher

SAGE Publications Ltd (55 City Road, London EC1Y 1SP, United Kingdom)

Emtree Heading

age distribution; correlation analysis; data extraction; *diabetes mellitus; disease association; disease severity; evaluation; gender; glycemic control; human; immune system; iron overload; kidney failure; Medline; microangiopathy; mortality; *public health; public health problem; review; risk factor; sample size; social status; statistical significance; treatment failure; *tuberculosis; vitamin deficiency; trace element.

Link to the Ovid Full Text or citation:

Click here for full text options

253.

Global epidemiology of tuberculosis: Prospects for control.

Lonnroth K., Raviglione M.

Seminars in Respiratory and Critical Care Medicine. 29 (5) (pp 481-491), 2008. Date of Publication: October 2008.

AN: 2008474656

The burden of tuberculosis (TB) is now very slowly decreasing globally. However, the rate of decline is too slow to reach all the epidemiological impact targets set for 2015. The prospects for reaching the TB elimination target set for 2050 are even bleaker. Implementation of the World Health Organization's Stop TB Strategy is currently lagging behind the envisioned scale-up pace, particularly with regard to TB/HIV collaborative activities and management of drug resistant TB. To ensure long-term TB control there is, first, a need to ensure that all components of the Stop TB Strategy are scaled up according to plans, with special attention to improved access for the poor. However, this may not be enough. Recent analyses suggest that the impact of current efforts to reduce TB incidence is less than expected and that improved diagnostic and curative efforts need to be combined with additional preventive efforts. New and more effective vaccines and drugs for preventive treatment would revolutionize TB control. A stronger focus on prevention would also entail more concerted actions to limit the impact of TB risk factors, including HIV/AIDS, smoking, malnutrition, alcoholism, diabetes, crowded living conditions, and indoor air pollution, which may all contribute a considerable proportion of the global TB burden. Copyright © 2008 by Thieme Medical Publishers, Inc.

Institution

(Lonnroth, Raviglione) Stop TB Department, World Health Organization, Geneva, Switzerland (Lonnroth) Stop TB Department, World Health Organization, 20 Avenue Appia, CH-1211 Geneva, Switzerland

Publisher

Thieme Medical Publishers, Inc.

Emtree Heading

acquired immune deficiency syndrome; alcoholism; antibiotic resistance; Chile; China; cohort analysis; Cuba; diabetes mellitus; drug sensitivity; health program; health service; highly active antiretroviral therapy; human; Human immunodeficiency virus; Human immunodeficiency virus infection/dt [Drug Therapy]; incidence; India; indoor air pollution; infection risk; lifestyle; malnutrition; mixed infection; mortality; multidrug resistance; Peru; prevalence; prevention and control; priority journal; public health; review; risk factor; risk reduction; screening test; smoking; socioeconomics; treatment outcome; *tuberculosis/di [Diagnosis]; *tuberculosis/dt [Drug Therapy]; *tuberculosis/ep [Epidemiology]; *tuberculosis/pc [Prevention]; *tuberculosis control; Uruguay; world health organization; antiretrovirus agent/dt [Drug Therapy]; BCG vaccine/dt [Drug Therapy]; cotrimoxazole/dt [Drug Therapy]; isoniazid/dt [Drug Therapy]; tuberculostatic agent/dt [Drug Therapy].

Link to the Ovid Full Text or citation:

Click here for full text options

254.

Drug-related hospitalizations at a tertiary level hospital in Bangalore: A prospective study.

Koneri R., Prakasam K., Mishra V., Rajan H.

Journal of Clinical and Diagnostic Research. 2 (2) (pp 736-740), 2008. Date of Publication: 2008.

AN: 2008202628

The Objective of the present study was to determine the causality, severity, preventability, classification of adverse drug events, and-drug therapeutic failures resulting in hospitalization, at a tertiary level hospital in Bangalore. Prospective data was collected from a total of 155 consecutive adult patients hospitalized during a period of six months due to drug related events at the Kempegowda Institute of Medical Sciences, Bangalore. The prevalence of Drug-related hospitalizations was (6.4%) [95% Cl 5.6%-7.7%] in the study. Multiple drug therapy in patients was associated with drug related hospitalizations. 50 % of the admissions were due to Adverse Drug Reactions (ADR), 38% due to Dose Related Therapeutic Failure (DTF), and only 12% were due to Self or Intentional Poisoning (SIP.). On sub-group analysis, 64% of ADR were noted to be due to normal side effects; and 68% of DTF were due to non-compliance. Using Naranjo's probability scale for causality assessment; 58% of Drug Related Hospital Admission (DRHA) was classified as definite; whereas 36% was probable. Out of these, 84% of DRHA were predictable, whereas 16% were non-predictable. 72% of DRHA was managed by altering the dose. The prevalence of Drug-related hospitalizations is high in this hospital, which merits further research and intervention.

Institution

(Koneri) Department of Pharmacology, KIMS Pharmacy College, Bangalore 70, India

Publisher

Journal of Clinical and Diagnostic Research

Emtree Heading

adult; aged; article; asthma/dt [Drug Therapy]; cardiovascular disease/dt [Drug Therapy]; confidence interval; diabetes mellitus/dt [Drug Therapy]; disease classification; drug dose titration; *drug induced disease/ep [Epidemiology]; *drug induced disease/et [Etiology]; *drug induced disease/si [Side Effect]; drug overdose; drug treatment failure; epilepsy/dt [Drug Therapy]; female; *hospitalization; human; hypertension/dt [Drug Therapy]; India; infection/dt [Drug Therapy]; inflammation/dt [Drug Therapy]; length of stay; major clinical study; male; patient compliance; prevalence; probability; prospective study; respiratory tract disease/dt [Drug Therapy]; risk assessment; risk factor; self poisoning; tertiary health care; tuberculosis/dt [Drug Therapy]; antidiabetic agent/ae [Adverse Drug Reaction]; antidiabetic agent/do [Drug Dose]; antidiabetic agent/dt [Drug Therapy]; antidiabetic agent/to [Drug Toxicity]; antiretrovirus agent/ae [Adverse Drug Reaction]; antiretrovirus agent/do [Drug Dose]; antiretrovirus agent/dt [Drug Therapy]; antiretrovirus agent/to [Drug Toxicity]; cardiovascular agent/ae [Adverse Drug Reaction]; cardiovascular agent/do [Drug Dose]; cardiovascular agent/dt [Drug Therapy]; cardiovascular agent/to [Drug Toxicity]; *drug/ae [Adverse Drug Reaction]; *drug/do [Drug Dose]; *drug/to [Drug Toxicity]; histamine H1 receptor antagonist/ae [Adverse Drug Reaction]; histamine H1 receptor antagonist/do [Drug Dose]; histamine H1 receptor antagonist/dt [Drug Therapy]; histamine H1 receptor antagonist/to [Drug Toxicity]; nonsteroid antiinflammatory agent/ae [Adverse Drug Reaction]; nonsteroid antiinflammatory agent/do [Drug Dose]; nonsteroid antiinflammatory agent/dt [Drug Therapy]; nonsteroid antiinflammatory agent/to [Drug Toxicity]; respiratory tract agent/ae [Adverse Drug Reaction]; respiratory tract agent/do [Drug Dose]; respiratory tract agent/dt [Drug Therapy]; respiratory tract agent/to [Drug Toxicity]; steroid/ae [Adverse Drug Reaction]; steroid/do [Drug Dose]; steroid/dt [Drug Therapy]; steroid/to [Drug Toxicity]; tuberculostatic agent/ae [Adverse Drug Reaction]; tuberculostatic agent/do [Drug Dose]; tuberculostatic agent/dt [Drug Therapy]; tuberculostatic agent/to [Drug Toxicity].

Link to the Ovid Full Text or citation:

Click here for full text options

255.

Chronic heart failure in Japan: Implications of the CHART studies.

Shiba N., Shimokawa H.

Vascular Health and Risk Management. 4 (1) (pp 103-113), 2008. Date of Publication: 2008.

AN: 2008158492

The prognosis of patients with chronic heart failure (CHF) still remains poor, despite the recent advances in medical and surgical treatment. Furthermore, CHF is a major public health problem in most industrialized countries where the elderly population is rapidly increasing. Although the prevalence and mortality of CHF used to be relatively low in Japan, the disorder has been markedly increasing due to the rapid aging of the society and the Westernization of lifestyle that facilitates the development of coronary artery disease. The Chronic Heart Failure Analysis and Registry in the Tohoku District (CHART)-1 study was one of the largest cohorts in Japan. The study has clarified the characteristics and prognosis of Japanese patients with CHF, demonstrating that their prognosis was similarly poor compared with those in Western countries. However, we still need evidence for the prevention and treatment of CHF based on the large cohort studies or randomized treatment trials in the Japanese population. Since the strategy for CHF management is now changing from treatment to prevention, a larger-size prospective cohort, called the CHART-2 study, has been initiated to evaluate the risk factors of CHF in Japan. This review summarizes the current status of CHF studies in Japan and discusses their future perspectives. © 2008 Shiba and Shimokawa, publisher and licensee Dove Medical Press Ltd.

Institution

(Shiba, Shimokawa) Department of Cardiovascular Medicine, Department of Evidence-Based Cardiovascular Medicine, Tohoku University Graduate School of Medicine, 1-1 Seiryo-machi, Aobaku, Sendai City 980-8574, Japan

Publisher

DOVE Medical Press Ltd.

Emtree Heading

aging; cardiovascular disease; cause of death; clinical trial; cost effectiveness analysis; diabetes mellitus; drug dose comparison; drug efficacy; echocardiography; gastroenteritis; health insurance; *heart failure/di [Diagnosis]; *heart failure/dm [Disease Management]; *heart failure/dt [Drug Therapy]; *heart failure/et [Etiology]; *heart failure/pc [Prevention]; *heart failure/su [Surgery]; heart left ventricle ejection fraction; heart left ventricle enddiastolic volume; human; hypercholesterolemia/dt [Drug Therapy]; hypertension/dt [Drug Therapy]; Japan; medical care; optimal drug dose; pneumonia; prognosis; proportional hazards model; quality of life; race difference; review; social status; treatment outcome; tuberculosis; angiotensin 2 receptor antagonist/dt [Drug Therapy]; azosemide/ct [Clinical Trial]; azosemide/cm [Drug Comparison]; azosemide/dt [Drug Therapy]; beta adrenergic receptor blocking agent/dt [Drug Therapy]; candesartan/ct [Clinical Trial]; candesartan/dt [Drug Therapy]; carvedilol/ct [Clinical Trial]; carvedilol/do [Drug Dose]; carvedilol/dt [Drug Therapy]; dipeptidyl carboxypeptidase inhibitor/dt [Drug Therapy]; furosemide/ct [Clinical Trial]; furosemide/cm [Drug Comparison]; furosemide/dt [Drug Therapy]; olmesartan/ct [Clinical Trial]; olmesartan/dt [Drug Therapy]; pitavastatin/ct [Clinical Trial]; pitavastatin/dt [Drug Therapy]; placebo; valsartan/ct [Clinical Trial]; valsartan/dt [Drug Therapy].

Link to the Ovid Full Text or citation:

Click here for full text options

256.

Clinical characteristics of pulmonary tuberculosis patients from a southern Taiwan hospital-based survey.

Wang C.-S., Chen H.-C., Yang C.-J., Tsai J.-R., Chong I.-W., Hwang J.-J., Huang M.-S.

Kaohsiung Journal of Medical Sciences. 24 (1) (pp 17-24), 2008. Date of Publication: January 2008.

AN: 2008095219

This study aimed to identify the clinical characteristics of culture-positive pulmonary tuberculosis (TB) patients from a southern Taiwan hospital-based survey between August 1, 2003 and July 31, 2006. Demographics, symptoms, susceptibility patterns, sputum acid-fast bacilli (AFB) stain status and treatment outcomes were recorded. The medical records of 154 patients who presented to the Kaohsiung Municipal Hsiao-Kang Hospital were analyzed retrospectively. The mean age of patients was 59.5 years; 115 patients were male and 39 were female. Diabetes mellitus (48/154; 31.2%) was the most frequent risk factor for pulmonary TB infection. Nearly all patients (139/154; 90.3%) had a cough. Fever was only seen in 27.9% and hemoptysis in 14.9% of patients. The combined resistance rates of Mycobacterium tuberculosis to the tested first-line agents were as follows: isoniazid, 3.2%; rifampin, 7.8%; ethambutol, 5.8%; and streptomycin, 2.6%. The combined resistance rate to any one of four first-line drugs was 12.3%. The combined resistance rate to ofloxacin was 3.9%. The combined resistance rate of multidrug resistant-TB was 1.9%. Sputum AFB stains were positive in 68.2% of cases. Analysis of treatment outcomes showed overall treatment success at 76.6%. The proportions of patients who died, defaulted treatment or in whom treatment failed were 16.2%, 3.9% and 0.0%, respectively. In conclusion, our study showed: (1) a higher frequency of pulmonary TB in male subjects than in other areas of Taiwan; (2) a higher frequency of cough and lower frequency of fever and hemoptysis than previous studies; (3) that the combined resistance rates to isoniazid and streptomycin were lower than both average levels in Taiwan and the global combined drug resistance rate; and (4) a higher proportion of patients responding to treatment and lower proportions of patients suffering mortality, defaulting treatment or not responding to treatment compared with other areas of Taiwan. With regard to resistance rates, the combined resistance rate to ethambutol was similar to the average level in Taiwan and higher than the global combined drug resistance rate. However, the combined resistance rate to rifampin was higher than both the average level in Taiwan and the global combined drug resistance rate. The combined resistance rates to at least any one of four first-line drugs and multidrug resistant-TB were lower than the average levels in Taiwan and higher than the global combined drug resistance rate. Our results may help to identify local variations in the disease and improve the effectiveness of TB infection control programs. © 2008 Elsevier. All rights reserved.

Institution

(Wang, Chen) Department of Internal Medicine, Kaohsiung Municipal Hsiao-Kang Hospital, Kaohsiung, Taiwan (Republic of China) (Yang, Tsai, Chong, Hwang, Huang) Division of Pulmonary and Critical Care Medicine, Department of Internal Medicine, Kaohsiung Medical University Hospital, Kaohsiung, Taiwan (Republic of China)

(Chong, Hwang, Huang) Department of Internal Medicine, College of Medicine, Kaohsiung Medical University, Kaohsiung, Taiwan (Republic of China)

(Huang) Division of Pulmonary and Critical Care Medicine, Department of Internal Medicine, Kaohsiung Medical University Hospital, 100 Shih-Chuan 1st Road, Kaohsiung 807, Taiwan (Republic of China)

Publisher

Elsevier (Singapore) Pte Ltd

Emtree Heading

acid fast bacterium; adult; aged; antibiotic resistance; article; clinical feature; combination chemotherapy; controlled study; coughing; diabetes mellitus; female; fever; health survey; hemoptysis; hospital based case control study; human; infection risk; infection sensitivity; *lung tuberculosis/dt [Drug Therapy]; *lung tuberculosis/ep [Epidemiology]; major clinical study; male; medical record; morbidity; mortality; multidrug resistance; Mycobacterium tuberculosis; sex difference; sputum culture; Taiwan; treatment failure; treatment outcome; ethambutol/cb [Drug Combination]; ethambutol/dt [Drug Therapy]; isoniazid/cb [Drug Combination]; isoniazid/dt [Drug Therapy]; ofloxacin/dt [Drug Therapy]; rifampicin/cb [Drug Combination]; rifampicin/dt [Drug Therapy]; streptomycin/cb [Drug Combination]; streptomycin/dt [Drug Therapy].

Link to the Ovid Full Text or citation:

Click here for full text options

257.

Review: Fixed-dose drug combinations improve medication compliance compared with free-drug regimens: Commentary.

Messerli F.H., Bergmann J.-F.

Evidence-Based Medicine. 13 (1) (pp 18), 2008. Date of Publication: February 2008.

AN: 2008080691

Institution

(Messerli) Columbia University College of Physicians and Surgeons, New York, NY, United States (Bergmann) Hopital Lariboisiere, University Paris 7, Paris, France

Publisher

BMJ Publishing Group

Emtree Heading

*chronic disease/dt [Drug Therapy]; clinical trial; combination chemotherapy; controlled clinical trial; diabetes mellitus/dt [Drug Therapy]; human; Human immunodeficiency virus infection/dt [Drug Therapy]; hypertension/dt [Drug Therapy]; outcome assessment; patient attitude; patient compliance; risk reduction; short survey; treatment duration; tuberculosis/dt [Drug Therapy]; *drug/ct [Clinical Trial]; *drug/do [Drug Dose]; *drug/dt [Drug Therapy].

Link to the Ovid Full Text or citation:

Click here for full text options

258.

European evidence-based Consensus on the management of ulcerative colitis: Current management.

Travis S.P.L., Stange E.F., Lemann M., Oresland T., Bemelman W.A., Chowers Y., Colombel J.F., D'Haens G., Ghosh S., Marteau P., Kruis W., Mortensen N.J.McC., Penninckx F., Gassull M.

Journal of Crohn's and Colitis. 2 (1) (pp 24-62), 2008. Date of Publication: March 2008.

AN: 2008056224

Publisher

Elsevier

Emtree Heading

appendectomy; basal cell carcinoma/si [Side Effect]; bone marrow suppression/si [Side Effect]; clinical feature; clinical trial; colon resection; colonoscopy; combination chemotherapy; controlled clinical trial; diabetes mellitus/si [Side Effect]; diarrhea/si [Side Effect]; disease activity; disease course; drug dosage form comparison; drug dose escalation; drug dose increase; drug efficacy; drug fatality/si [Side Effect]; drug mechanism; drug megadose; drug response; drug safety; follow up; gastrointestinal biopsy; gastrointestinal symptom/si [Side Effect]; headache/si [Side Effect]; histoplasmosis/si [Side Effect]; human; hypertension/si [Side Effect]; hypomagnesemia/si [Side Effect]; ileostomy; interstitial nephritis/si [Side Effect]; intestine function; kidney failure/si [Side Effect]; laparoscopy; monotherapy; nausea/si [Side Effect]; nephrotic syndrome/si [Side Effect]; neuropathy/si [Side Effect]; pancreatitis/si [Side Effect]; paresthesia/si [Side Effect]; perioperative period; pneumonia/si [Side Effect]; postoperative complication; postoperative period; priority journal; proctocolectomy; randomized controlled trial; rash/si [Side Effect]; relapse; review; risk factor; single drug dose; steroid therapy; thrombocytopenia/si [Side Effect]; toxic megacolon/co [Complication]; treatment outcome; tremor/si [Side Effect]; tuberculosis/si [Side Effect]; *ulcerative colitis/di [Diagnosis]; *ulcerative colitis/dt [Drug Therapy]; *ulcerative colitis/su [Surgery]; 12 sulfodehydroabietic acid/dt [Drug Therapy]; 12 sulfodehydroabietic acid/pd [Pharmacology]; acetarsol/ct [Clinical Trial]; acetarsol/cm [Drug Comparison]; acetarsol/dt [Drug Therapy]; adalimumab/cm [Drug Comparison]; adalimumab/dt [Drug Therapy]; adalimumab/sc [Subcutaneous Drug Administration]; aminosalicylic acid/cb [Drug Combination]; aminosalicylic acid/dt [Drug Therapy]; aminosalicylic acid/po [Oral Drug Administration]; amoxicillin/cb [Drug Combination]; amoxicillin/dt [Drug Therapy]; azathioprine/ae [Adverse Drug Reaction]; azathioprine/ct [Clinical Trial]; azathioprine/dt [Drug Therapy]; azathioprine/po [Oral Drug Administration]; beclometasone dipropionate/dt [Drug Therapy]; betamethasone/dt [Drug Therapy]; betamethasone/iv [Intravenous Drug Administration]; bismuth carbomer/ct [Clinical Trial]; bismuth carbomer/dt [Drug Therapy]; bismuth carbomer/pd [Pharmacology]; bismuth derivative/ct [Clinical Trial]; bismuth derivative/dt [Drug Therapy]; bismuth derivative/pd [Pharmacology]; budesonide/dt [Drug Therapy]; corticosteroid/dt [Drug Therapy]; corticosteroid/iv [Intravenous Drug Administration]; corticosteroid/po [Oral Drug Administration]; corticosteroid/rc [Rectal Drug Administration]; cyclosporin A/ae [Adverse Drug Reaction]; cyclosporin A/ct [Clinical Trial]; cyclosporin A/cm [Drug Comparison]; cyclosporin A/dt [Drug Therapy]; cyclosporin A/iv [Intravenous Drug Administration]; cyclosporin A/po [Oral Drug Administration]; cyclosporin A/pd [Pharmacology]; hydrocortisone/dt [Drug Therapy]; hydrocortisone/iv [Intravenous Drug Administration]; infliximab/ae [Adverse Drug Reaction]; infliximab/ct [Clinical Trial]; infliximab/cm [Drug Comparison]; infliximab/do [Drug Dose]; infliximab/dt [Drug Therapy]; lidocaine/dt [Drug Therapy]; lidocaine/pd [Pharmacology]; mesalazine/ae [Adverse Drug Reaction]; mesalazine/ct [Clinical Trial]; mesalazine/cb [Drug Combination]; mesalazine/cm [Drug Comparison]; mesalazine/do [Drug Dose]; mesalazine/dt [Drug Therapy]; mesalazine/po [Oral Drug Administration]; mesalazine/pd [Pharmacology]; mesalazine/rc [Rectal Drug Administration]; mesalazine/tp [Topical Drug Administration]; mesren; methotrexate/ct [Clinical Trial]; methotrexate/cm [Drug Comparison]; methotrexate/dt [Drug Therapy]; methotrexate/po [Oral Drug Administration]; methylprednisolone/do [Drug Dose]; methylprednisolone/dt [Drug Therapy]; methylprednisolone/iv [Intravenous Drug Administration]; metronidazole/cb [Drug Combination]; nicotine/ct [Clinical Trial]; nicotine/cb [Drug Combination]; nicotine/cm [Drug Comparison]; nicotine/dt [Drug Therapy]; nicotine/pd [Pharmacology]; nicotine/td [Transdermal Drug Administration]; nicotine tartrate/dt [Drug Therapy]; nicotine tartrate/pd [Pharmacology]; placebo; prednisolone/ct [Clinical Trial]; prednisolone/cm [Drug Comparison]; prednisolone/dt [Drug Therapy]; prednisolone/po [Oral Drug Administration]; rebamipide/dt [Drug Therapy]; rebamipide/pd [Pharmacology]; ropivacaine/ct [Clinical Trial]; ropivacaine/do [Drug Dose]; ropivacaine/dt [Drug Therapy]; ropivacaine/pd [Pharmacology]; salazosulfapyridine/cm [Drug Comparison]; salazosulfapyridine/dt [Drug Therapy]; tacrolimus/ae [Adverse Drug Reaction]; tacrolimus/ct [Clinical Trial]; tacrolimus/cm [Drug Comparison]; tacrolimus/dt [Drug Therapy]; tacrolimus/po [Oral Drug Administration]; tetracycline/cb [Drug Combination]; unclassified drug; unindexed drug.

Drug Trade Names and Manufacturers

asacol, claversal, lialda, mesasal, mesren, pentasa, rowasa, salofalk

Link to the Ovid Full Text or citation:

Click here for full text options

259.

Tuberculosis among the health-care workers of the University of Ilorin Teaching Hospital.

Salami A.K., Katibi I.A.

Tropical Doctor. 37 (4) (pp 251), 2007. Date of Publication: October 2007.

AN: 2009291434

Institution

(Salami, Katibi) Department Ofmedicine, University of Ilorin, PMB 1515, Ilorin, Nigeria

Publisher

Royal Society of Medicine Press Ltd (P.O. Box 9002, London W1A 0ZA, United Kingdom)

Emtree Heading

adult; article; chronic obstructive lung disease/ep [Epidemiology]; clinical article; diabetes mellitus/ep [Epidemiology]; female; health care personnel; human; Human immunodeficiency virus infection/ep [Epidemiology]; liver cirrhosis/ep [Epidemiology]; *lung tuberculosis/di [Diagnosis]; *lung tuberculosis/ep [Epidemiology]; male; Mycobacterium tuberculosis; nursing; risk; teaching hospital; treatment outcome; tuberculosis/di [Diagnosis]; tuberculosis/ep [Epidemiology]; tuberculous spondylitis.

Link to the Ovid Full Text or citation:

Click here for full text options

260.

Fluoroquinolones for the treatment of pulmonary tuberculosis.

Moadebi S., Harder C.K., Fitzgerald M.J., Elwood K.R., Marra F.

Drugs. 67 (14) (pp 2077-2099), 2007. Date of Publication: 2007.

AN: 2007487057

Tuberculosis (TB) continues to be a significant problem globally. Treatment includes a multiple drug regimen with isoniazid, rifampicin (rifampin), pyrazinamide and ethambutol. Often, one of these medications needs to be replaced as a result of adverse events or because Mycobacterium tuberculosis develops resistance against one these first-line agents. Fluoroquinolones, particularly the newer ones, possess good in vitro (levofloxacin, gatifloxacin, moxifloxacin) and in vivo (gatifloxacin and moxifloxacin) bactericidal activity against M. tuberculosis, making them attractive agents for the treatment of pulmonary TB.All relevant clinical trials, cohort studies and case reports investigating the clinical efficacy and tolerability of fluoroquinolones when used for the treatment of pulmonary TB were evaluated for this review. Specifically, efficacy and safety in the following indications were investigated: (i) first-line treatment of drug-sensitive pulmonary TB; (ii) first-line treatment for multi-drug resistant (MDR) TB; and (iii) treatment of patients with drug intolerance. Twenty-seven articles met our inclusion criteria; nine articles presented data from randomised, controlled or cohort studies. Seven studies used fluoroquinolones as first-line agents in drug-sensitive TB (1469 patients), 15 studies used fluoroquinolones to treat MDR-TB (1025 patients) and six studies (951 patients) investigated the use of fluoroquinolones in patients intolerant to other TB medications.In patients with susceptible M. tuberculosis strains, substitution with a fluoroquinolone did not have an effect on cure or radiological improvement at 8 weeks or failure at 12 months. Substitution of older fluoroquinolones into a regimen, especially ciprofloxacin, resulted in a higher rate of relapse and a longer time to sputum-culture conversions. The use of fluoroquinolones in patients with MDR-TB is supported by some trials where others show a lack of improvement in efficacy of a regimen.Our review of the literature does not support the use of older fluoroquinolones, especially ciprofloxacin, as substitute agents for drug-sensitive or drug-resistant TB. However, newer fluoroquinolones, such as moxifloxacin, may be a reasonable alternative based on results from one large clinical trial. Fluoroquinolones have an important role as substitute agents for those who are intolerant of first-line TB agents. © 2007 Adis Data Information BV. All rights reserved.

Institution

(Moadebi, Fitzgerald, Elwood, Marra) BC Centre for Disease Control, Vancouver, BC, Canada (Harder, Elwood, Marra) University of British Columbia, Vancouver, BC, Canada

(Fitzgerald) Centre for Clinical Epidemiology and Evaluation, Vancouver, BC, Canada

(Marra) BC Centre for Disease Control, Vaccine and Pharmacy Services, 655 West 12th Avenue, Vancouver, BC V5Z 1L8, Canada

Publisher

Adis International Ltd

Emtree Heading

absence of side effects/si [Side Effect]; acute kidney failure/si [Side Effect]; add on therapy; ageusia/si [Side Effect]; case study; central nervous system disease/si [Side Effect]; clinical study; clinical trial; cohort analysis; disease severity; drug dose comparison; drug effect; drug efficacy; drug hypersensitivity; drug induced headache/si [Side Effect]; drug megadose; drug safety; drug substitution; drug tolerability; drug treatment failure; drug withdrawal; gastrointestinal disease/si [Side Effect]; gastrointestinal symptom/si [Side Effect]; heart arrhythmia/si [Side Effect]; human; insulin dependent diabetes mellitus/si [Side Effect]; low drug dose; *lung tuberculosis/dt [Drug Therapy]; Medline; monotherapy; myalgia/si [Side Effect]; nausea/si [Side Effect]; outcome assessment; photosensitivity/si [Side Effect]; rash/si [Side Effect]; review; single drug dose; sputum culture; sputum smear; statistical analysis; treatment duration; treatment planning; treatment response; unspecified side effect/si [Side Effect]; vertigo/si [Side Effect]; vomiting/si [Side Effect]; aminosalicylic acid/ae [Adverse Drug Reaction]; aminosalicylic acid/ct [Clinical Trial]; aminosalicylic acid/dt [Drug Therapy]; capreomycin/ae [Adverse Drug Reaction]; capreomycin/ct [Clinical Trial]; capreomycin/dt [Drug Therapy]; capreomycin/im [Intramuscular Drug Administration]; *ciprofloxacin/ae [Adverse Drug Reaction]; *ciprofloxacin/ct [Clinical Trial]; *ciprofloxacin/cm [Drug Comparison]; *ciprofloxacin/do [Drug Dose]; *ciprofloxacin/dt [Drug Therapy]; clofazimine/ae [Adverse Drug Reaction]; clofazimine/ct [Clinical Trial]; clofazimine/dt [Drug Therapy]; clofazimine/im [Intramuscular Drug Administration]; cycloserine/ae [Adverse Drug Reaction]; cycloserine/ct [Clinical Trial]; cycloserine/dt [Drug Therapy]; ethambutol/ae [Adverse Drug Reaction]; ethambutol/ct [Clinical Trial]; ethambutol/cm [Drug Comparison]; ethambutol/dt [Drug Therapy]; ethionamide/ae [Adverse Drug Reaction]; ethionamide/ct [Clinical Trial]; ethionamide/cb [Drug Combination]; ethionamide/dt [Drug Therapy]; isoniazid/ae [Adverse Drug Reaction]; isoniazid/ct [Clinical Trial]; isoniazid/cb [Drug Combination]; isoniazid/cm [Drug Comparison]; isoniazid/dt [Drug Therapy]; kanamycin/ae [Adverse Drug Reaction]; kanamycin/ct [Clinical Trial]; kanamycin/cb [Drug Combination]; kanamycin/dt [Drug Therapy]; kanamycin/im [Intramuscular Drug Administration]; levofloxacin/ae [Adverse Drug Reaction]; levofloxacin/ct [Clinical Trial]; levofloxacin/cm [Drug Comparison]; levofloxacin/dt [Drug Therapy]; *moxifloxacin/ae [Adverse Drug Reaction]; *moxifloxacin/ct [Clinical Trial]; *moxifloxacin/cb [Drug Combination]; *moxifloxacin/dt [Drug Therapy]; *ofloxacin/ae [Adverse Drug Reaction]; *ofloxacin/ct [Clinical Trial]; *ofloxacin/cm [Drug Comparison]; *ofloxacin/do [Drug Dose]; *ofloxacin/dt [Drug Therapy]; pefloxacin/ae [Adverse Drug Reaction]; pefloxacin/ct [Clinical Trial]; pefloxacin/cb [Drug Combination]; pefloxacin/dt [Drug Therapy]; protionamide/ae [Adverse Drug Reaction]; protionamide/ct [Clinical Trial]; protionamide/dt [Drug Therapy]; pyrazinamide/ae [Adverse Drug Reaction]; pyrazinamide/ct [Clinical Trial]; pyrazinamide/cb [Drug Combination]; pyrazinamide/cm [Drug Comparison]; pyrazinamide/dt [Drug Therapy]; quinoline derived antiinfective agent/ae [Adverse Drug Reaction]; quinoline derived antiinfective agent/ct [Clinical Trial]; quinoline derived antiinfective agent/dt [Drug Therapy]; rifabutin/ae [Adverse Drug Reaction]; rifabutin/ct [Clinical Trial]; rifabutin/dt [Drug Therapy]; rifampicin/ae [Adverse Drug Reaction]; rifampicin/ct [Clinical Trial]; rifampicin/cb [Drug Combination]; rifampicin/cm [Drug Comparison]; rifampicin/dt [Drug Therapy]; sparfloxacin/ae [Adverse Drug Reaction]; sparfloxacin/ct [Clinical Trial]; sparfloxacin/cb [Drug Combination]; sparfloxacin/dt [Drug Therapy]; streptomycin/ae [Adverse Drug Reaction]; streptomycin/ct [Clinical Trial]; streptomycin/dt [Drug Therapy]; streptomycin/im [Intramuscular Drug Administration]; thioacetazone/ae [Adverse Drug Reaction]; thioacetazone/ct [Clinical Trial]; thioacetazone/cb [Drug Combination]; thioacetazone/dt [Drug Therapy].

Link to the Ovid Full Text or citation:

Click here for full text options

261.

The effect of type 2 diabetes mellitus on the presentation and treatment response of pulmonary tuberculosis.

Alisjahbana B., Sahiratmadja E., Nelwan E.J., Purwa A.M., Ahmad Y., Ottenhoff T.H.M., Nelwan R.H.H., Parwati I., Van Der Meer J.W.M., Van Crevel R.

Clinical Infectious Diseases. 45 (4) (pp 428-435), 2007. Date of Publication: 15 Aug 2007.

AN: 2007381906

Background. Diabetes mellitus (DM) is a known risk factor for tuberculosis (TB), and with the increasing prevalence of type 2 DM in less developed regions, many patients with TB will have concomitant DM. Presently, little is known about the effect of DM on the clinical presentation and treatment outcome of TB. Methods. In an urban setting in Indonesia, 737 patients with pulmonary TB were screened for DM and were followed up prospectively during TB treatment. Clinical characteristics and outcome were compared between patients with TB who had DM and patients with TB who did not have DM. Results. DM was diagnosed in 14.8% of patients with TB and was associated with older age and a greater body weight. On presentation, diabetic patients with TB had more symptoms but had no evidence of more-severe TB. After 2 months, results of sputum microscopic examination was more often positive in diabetic patients (18.1% vs. 10.0%). After 6 months, 22.2% of cultured sputum specimens from diabetic patients were positive for Mycobacterium tuberculosis (adjusted odds ratio, 7.65; P = .004). Conclusion. DM seems to have a negative effect on the outcome of TB treatment. The underlying mechanisms for the different response to treatment in diabetic patients with TB must be explored. Screening for DM and subsequent glycemic control may improve the outcome of TB treatment. © 2007 by the Infectious Diseases Society of America. All rights reserved.

Institution

(Alisjahbana, Purwa, Ahmad) Internal Medicine, Medical Faculty, Padjadjaran University, Bandung, Indonesia (Parwati) Clinical Pathology Department, Medical Faculty, Padjadjaran University, Bandung, Indonesia

(Sahiratmadja) Eijkman Institute of Molecular Biology, Medical Faculty, University of Indonesia, Jakarta, Indonesia

(Nelwan, Nelwan) Infectious Disease Working Group, Medical Faculty, University of Indonesia, Jakarta, Indonesia

(Ottenhoff) Department of Immunohematology and Blood Transfusion, Leiden University Medical Center, Leiden, Netherlands

(Van Der Meer, Van Crevel) Department of Internal Medicine, Radboud University, Nijmegen Medical Center, Netherlands

(Van Crevel) Dept. of Internal Medicine, Radboud University, Medical Center Nijmegen, PO Box 9101, 6500 HB, Nijmegen, Netherlands

Publisher

University of Chicago Press

Emtree Heading

adult; article; body weight; clinical feature; comorbidity; female; glycemic control; human; Indonesia; infection risk; *lung tuberculosis/dt [Drug Therapy]; major clinical study; male; Mycobacterium tuberculosis; *non insulin dependent diabetes mellitus; priority journal; prospective study; screening; sputum analysis; treatment response; urban area; antidiabetic agent/dt [Drug Therapy]; antidiabetic agent/po [Oral Drug Administration]; ethambutol/cb [Drug Combination]; ethambutol/dt [Drug Therapy]; isoniazid/cb [Drug Combination]; isoniazid/dt [Drug Therapy]; pyrazinamide/cb [Drug Combination]; pyrazinamide/dt [Drug Therapy]; rifampicin/cb [Drug Combination]; rifampicin/dt [Drug Therapy].

Link to the Ovid Full Text or citation:

Click here for full text options

262.

Study of association between use of complementary and alternative medicine and non-compliance with modern medicine in patients presenting to the emergency department.

Jose V., Bhalla A., Sharma N., Hota D., Sivaprasad S., Pandhi P.

Journal of Postgraduate Medicine. 53 (2) (pp 96-101), 2007. Date of Publication: 01 Apr 2007.

AN: 2007257222

Context: Complementary and alternative medicines (CAMs) are extensively used by the public. Noncompliance is an important cause of therapy failure. Aim: This study was done to determine prevalence of emergency admission due to noncompliance with modern medicine following switching over to CAM and to identify any significant association for CAM use among noncompliers. Setting and Design: This cross-sectional study was conducted in the emergency unit of a tertiary healthcare institute. Materials and Methods: Demographic factors and system affected were compared between compliers and noncompliers. Prevalence, reasons and nature of noncompliance were determined. Age, gender, outcome, relation strength and potential preventability of noncompliance, precipitating and previous disease and noncompliant drugs were compared for significant association between CAM using and other noncompliers. Statistical Analysis: Student's 't' test, Chi square test and odds ratio were used. Results: Of the 506 patients interviewed 168 (33%) were noncompliant. In 160 (95%) patients noncompliance was due to under-dosing. Lack of knowledge and CAM use constituted 144 (86%) noncompliance-related admissions. Thirty-three (7%) admissions were strongly related to noncompliance and CAM use. Age, gender, outcome, drug use and diseases except chronic obstructive pulmonary disease (COPD) and asthma showed no association while relation strength and potential preventability of emergency admission was less with CAM-using noncompliers. Noncompliance was observed for hypertension, diabetes, COPD and asthma, seizure disorder, tuberculosis and hemophilia besides hepatic and renal failure. The CAM noncompliers used CAM more for modern medicine incurable or unaffordable than curable diseases. Conclusion: Advice for regular treatment and frequent monitoring can decrease CAM use-related noncompliance admissions.

Institution

(Jose, Hota, Pandhi) Department of Pharmacology, Postgraduate Institute of Medical Education and Research, Chandigarh -160 012, India (Bhalla, Sharma, Sivaprasad) Department of Internal Medicine, Postgraduate Institute of Medical Education and Research, Chandigarh -160 012, India

Publisher

Medknow Publications

Emtree Heading

adult; age distribution; *alternative medicine; article; asthma; chi square test; chronic obstructive lung disease; controlled study; cross-sectional study; demography; diabetes mellitus; *emergency care; emergency ward; female; hemophilia; hospital admission; human; human experiment; hypertension; kidney failure; liver failure; male; outcome assessment; patient compliance; prevalence; risk assessment; seizure; sex ratio; statistical analysis; Student t test; tertiary health care; tuberculosis.

Link to the Ovid Full Text or citation:

Click here for full text options

263.

Treatment outcome of pulmonary tuberculosis in eastern Taiwan - Experience at a Medical Center.

Lee J.-J., Wu R.-L., Lee Y.-S., Wu Y.-C., Chiang C.-Y.

Journal of the Formosan Medical Association. 106 (1) (pp 25-30), 2007. Date of Publication: January 2007.

AN: 2007077270

Background/Purpose: The World Health Organization (WHO) has proposed that treatment outcome is an important indicator of tuberculosis control. This study investigated the outcome of tuberculosis treatment at a medical center in eastern Taiwan. Methods: A total of 166 pulmonary tuberculosis patients notified by Tzu Chi Hospital in 2002 were included in this study. Treatment outcome data were collected at the local level and categorized according to WHO recommendations as cured, treatment completed, failed, died, defaulted, or transferred. Outcomes of the 166 patients, as reported by the National Tuberculosis Program were obtained from the Taiwan Center for Disease Control (Taiwan CDC) for comparison. Results: Of the 166 patients, outcome was classified as cured in 46 (27.7%), treatment completed in 73 (44.0%), died in 27 (16.3%), treatment failed in five (3.0%), and defaulted in 15 (9.0%). Males were more likely to die or to default than females, and the elderly were more likely to die than younger patients. Patients with comorbidities were significantly more likely to die than patients without (p=0.025). Patients with a history of tuberculosis were more likely to default (p = 0.050). Smear-positive patients were more likely to fail, and patients without cavitation on chest radiograph were more likely to have successful treatment. Outcomes of 26 (15.7%) cases in this cohort were unavailable (18 cases) or inaccurate (8 cases) on the Taiwan CDC website. Conclusion: The unsatisfactory outcome of pulmonary tuberculosis treatment in this medical center in eastern Taiwan highlights the importance of implementing directly observed treatment short course strategy. Improvement in the quality of data reported by the National Tuberculosis Program is urgently needed. © 2007 Elsevier & Formosan Medical Association.

Institution

(Lee) Department of Internal Medicine, Buddhist Tzu Chi General Hospital, Tzu Chi University, Hualien, Taiwan (Republic of China) (Wu) Department of Public Health, Tzu Chi University, Hualien, Taiwan (Republic of China)

(Lee, Wu) Center for Disease Control, Taipei, Taiwan (Republic of China)

(Chiang) International Union Against Tuberculosis and Lung Diseases, Paris, France

(Chiang) Kuang-Fu South Road, Taipei 105, Taiwan (Republic of China)

Publisher

Scientific Communications International Ltd

Emtree Heading

adolescent; adult; aged; article; cancer; child; clinical trial; comorbidity; diabetes mellitus; drug fever/si [Side Effect]; drug substitution; drug tolerability; drug withdrawal; female; human; liver cirrhosis; lung cavitation/di [Diagnosis]; lung cavitation/dt [Drug Therapy]; *lung tuberculosis/di [Diagnosis]; *lung tuberculosis/dr [Drug Resistance]; *lung tuberculosis/dt [Drug Therapy]; major clinical study; male; patient attitude; pneumoconiosis; sputum examination; survival rate; Taiwan; thorax radiography; toxic hepatitis/si [Side Effect]; treatment outcome; uremia; *ethambutol/ae [Adverse Drug Reaction]; *ethambutol/cb [Drug Combination]; *ethambutol/dt [Drug Therapy]; *isoniazid/ae [Adverse Drug Reaction]; *isoniazid/cb [Drug Combination]; *isoniazid/dt [Drug Therapy]; *pyrazinamide/ae [Adverse Drug Reaction]; *pyrazinamide/cb [Drug Combination]; *pyrazinamide/dt [Drug Therapy]; *rifampicin/ae [Adverse Drug Reaction]; *rifampicin/cb [Drug Combination]; *rifampicin/dt [Drug Therapy].

Link to the Ovid Full Text or citation:

Click here for full text options

264.

HCV-related advanced fibrosis/cirrhosis: Randomized controlled trial of pegylated interferon alpha-2a and ribavirin.

Helbling B., Jochum W., Stamenic I., Knopfli M., Cerny A., Borovicka J., Gonvers J.-J., Wilhelmi M., Dinges S., Mullhaupt B., Esteban A., Meyer-Wyss B., Renner E.L.

Journal of Viral Hepatitis. 13 (11) (pp 762-769), 2006. Date of Publication: November 2006.

AN: 2006511425

In patients with hepatitis C virus (HCV)-related advanced fibrosis/cirrhosis, 30% of sustained HCV clearance has been reported with pegylated interferon alpha-2a (PEG-IFN) alone, but the efficacy and tolerability of the PEG-IFN/ribavirin (RBV) combination remain poorly defined. A total of 124 treatment-naive patients with biopsy proved HCV-related advanced fibrosis/cirrhosis (Ishak score F4-F6, Child-Pugh score <7) were randomized to 48 weeks of PEG-IFN (180 mug sc weekly) and standard dose of RBV (1000/1200 mg po daily, STD) or PEG-IFN (180 mug sc weekly) and low-dose of RBV (600/800 mg po daily, LOW). Sustained virologic response (SVR) rates with PEG-IFN/STD RBV (52%) were higher - albeit not significantly - than that with PEG-IFN/LOW RBV (38%, P = 0.153). In multivariate analysis, genotype 2/3 and a baseline platelet count >150 x 109/L were independently associated with SVR. The likelihood of SVR was <7% if viraemia had not declined by >2 log or to undetectable levels after 12 weeks. Nine adverse events in the STD RBV and 15 in the LOW RBV group were classified as severe (including two deaths); dose reductions for intolerance were required in 78% and 57% (P = 0.013), and treatment was terminated early in 23% and 27% of patients (P = n.s.). The benefit/risk ratio of treating compensated HCV-cirrhotics with STD PEG-IFN/RBV is favourable. © 2006 The Authors.

Institution

(Helbling, Stamenic, Wilhelmi, Dinges, Mullhaupt, Renner) Division of Gastroenterology and Hepatology, University Hospital, Zurich, Switzerland (Helbling, Borovicka) Division of Gastroenterology/Hepatology, Cantonal Hospital, St Gallen, Switzerland

(Jochum) Department of Pathology, University Hospital, Zurich, Switzerland

(Knopfli, Cerny) Department of Internal Medicine, Cantonal Hospital, Lugano, Switzerland

(Gonvers) Division of Gastroenterology, University Hospital, Lausanne, Switzerland

(Esteban) Roche Pharma (Schweiz) AG, Reinach, Switzerland

(Meyer-Wyss) Division of Gastroenterology, St. Claraspital, Basel, Switzerland

(Renner) Department of Internal Medicine, Health Sciences Center, University of Manitoba, Winnipeg, Man., Canada

(Renner) Department of Internal Medicine, University of Manitoba, John Buhler Research Centre, McDermot Avenue, Winnipeg, Man. R3A 1R9, Canada

Publisher

Blackwell Publishing Ltd (9600 Garsington Road, Oxford OX4 2XG, United Kingdom)

Emtree Heading

abdominal bleeding/si [Side Effect]; acute coronary syndrome/si [Side Effect]; adult; aged; article; cancer recurrence/si [Side Effect]; cardiovascular disease/si [Side Effect]; clinical trial; controlled clinical trial; controlled study; depression/si [Side Effect]; diabetes mellitus/si [Side Effect]; dose response; drug dose reduction; drug dose regimen; drug effect; drug efficacy; drug fatality/si [Side Effect]; eczema/si [Side Effect]; endocrine disease/si [Side Effect]; eye disease/si [Side Effect]; female; gastroenteritis/si [Side Effect]; gastrointestinal disease/si [Side Effect]; genotype; hemiparesis/si [Side Effect]; *hepatitis C/dt [Drug Therapy]; *hepatitis C/et [Etiology]; Hepatitis C virus; human; infection/si [Side Effect]; insomnia/si [Side Effect]; leukopenia/si [Side Effect]; liver biopsy; liver cell carcinoma/si [Side Effect]; *liver cirrhosis/co [Complication]; *liver cirrhosis/di [Diagnosis]; *liver fibrosis/co [Complication]; *liver fibrosis/di [Diagnosis]; lumbar disk hernia/si [Side Effect]; lung tuberculosis/si [Side Effect]; major clinical study; male; mental disease/si [Side Effect]; metabolic disorder/si [Side Effect]; multicenter study; multivariate analysis; neoplasm/si [Side Effect]; nervousness; neurologic disease/si [Side Effect]; nonhodgkin lymphoma/si [Side Effect]; open study; outcome assessment; peroneus nerve paralysis/si [Side Effect]; pleura empyema/si [Side Effect]; pneumonia/si [Side Effect]; polyneuropathy/si [Side Effect]; priority journal; psychosis/si [Side Effect]; randomized controlled trial; risk benefit analysis; septic shock/si [Side Effect]; skin disease/si [Side Effect]; suicide attempt/si [Side Effect]; thrombocyte count; thrombocytopenia/si [Side Effect]; tongue carcinoma/si [Side Effect]; viremia; visual impairment/si [Side Effect]; *peginterferon alpha2a/ae [Adverse Drug Reaction]; *peginterferon alpha2a/ct [Clinical Trial]; *peginterferon alpha2a/cb [Drug Combination]; *peginterferon alpha2a/do [Drug Dose]; *peginterferon alpha2a/dt [Drug Therapy]; *peginterferon alpha2a/sc [Subcutaneous Drug Administration]; *ribavirin/ae [Adverse Drug Reaction]; *ribavirin/ct [Clinical Trial]; *ribavirin/cb [Drug Combination]; *ribavirin/do [Drug Dose]; *ribavirin/dt [Drug Therapy]; *ribavirin/po [Oral Drug Administration].

Drug Trade Names and Manufacturers

Roche Pharma [Switzerland]

Link to the Ovid Full Text or citation:

Click here for full text options

265.

Stroke: An Indian perspective.

Desai S.D., Behari M.

Journal International Medical Sciences Academy. 19 (2) (pp 136-138), 2006. Date of Publication: April/June 2006.

AN: 2007217053

Stroke is a very common disorder affecting older people. Community surveys of different parts of the country indicate a crude prevalence of stroke of 203/1, 00,000 populations above 20 years which is much lower than that reported from the west. The causes of low prevalence of stroke in India are predominance of young population in India in which stroke is less prevalent and high early mortality leaving behind fewer survivors. The mortality rate due to stroke in India is 7.3/ 1, 00,000. Thus, deaths due to stroke are 22 times higher than malaria, 1.4 times that due to tuberculosis and 4 times that due to rheumatic heart disease. About 10-15 % of stroke occurs in population < 40 year old. Certain risk factors evaluated in Indian centers are hyperhomocystienemia, elevated anticardiolipin antibody, and elevated lipoprotein, in addition to hypertension, smoking and diabetes mellitus. Most centers show the ratio of ischemic to hemorrhagic stroke as 10-15% to 90- 85 %. As regards the vascular involvement both intracranial and extra-cranial involvement occurs in Indian patients. On the other hand in west <5% have intracranial disease and in the Far East <5% have extra-cranial disease.

Institution

(Desai, Behari) Department of Neurology, CNC, AIIMS, Ansari Nager New Delhi, India

Publisher

International Medical Sciences Academy

Emtree Heading

acute disease/dt [Drug Therapy]; atherosclerosis; *cerebrovascular accident/dt [Drug Therapy]; conference paper; human; India; mortality; prevalence; rheumatic heart disease; risk factor; treatment outcome; fibrinolytic agent/dt [Drug Therapy].

Link to the Ovid Full Text or citation:

Click here for full text options

266.

Heartfile's contribution to heath systems strengthening in Pakistan.

Nishtar S.

Eastern Mediterranean Health Journal. 12 (SUPPL. 2) (pp S38-S53), 2006. Date of Publication: 2006.

AN: 2007012979

Key health indicators in Pakistan remain relatively intransigent. While there has been some improvement at intermediate outcome and process levels, several challenges remain, including an inattention to health systems strengthening. Within this context the NGO Heartfile has worked to bring about changes at a health policy and systems level through creation of a policy-level institutional mechanism for systems strengthening and a national health reform agenda based on systems strengthening and an intersectoral approach to health. By generating evidence, Heartfile has also assisted in strengthening the evidence-policy linkage, and in developing contemporary concepts for health policy and planning and service delivery.

Institution

(Nishtar) Heartfile and Pakistan's Health Policy Think Tank, Chak Shahzad, Islamabad, Pakistan

Publisher

World Health Organization

Emtree Heading

acute diarrhea/ep [Epidemiology]; acute diarrhea/et [Etiology]; article; blindness/et [Etiology]; blindness/pc [Prevention]; breast cancer/ep [Epidemiology]; breast cancer/et [Etiology]; child health; childhood mortality; decision making; diabetes mellitus/ep [Epidemiology]; diabetes mellitus/et [Etiology]; disease surveillance; funding; goal attainment; *health care; health care planning; *health care policy; health program; health promotion; health service; health status; hepatitis B/ep [Epidemiology]; hepatitis B/et [Etiology]; hepatitis B/pc [Prevention]; hepatitis C/ep [Epidemiology]; hepatitis C/et [Etiology]; hepatitis C/pc [Prevention]; human; Human immunodeficiency virus infection/ep [Epidemiology]; Human immunodeficiency virus infection/et [Etiology]; immunization; intravenous drug abuse; leprosy; liver cirrhosis/co [Complication]; malaria/ep [Epidemiology]; malaria/et [Etiology]; malaria/pc [Prevention]; maternal welfare; medical research; mental health; Pakistan; public health; respiratory tract infection/ep [Epidemiology]; respiratory tract infection/et [Etiology]; risk factor; seroprevalence; skin leishmaniasis/ep [Epidemiology]; skin leishmaniasis/et [Etiology]; smoking; traffic accident; treatment outcome; tuberculosis/ep [Epidemiology]; tuberculosis/et [Etiology]; tuberculosis/pc [Prevention]; virus hemorrhagic fever; world health organization.

Link to the Ovid Full Text or citation:

Click here for full text options

267.

Tuberculosis of the bone marrow: Clinico-pathotogical study of 22 cases from Saudi Arabia.

Hakawi A.M., Alrajhi A.A.

International Journal of Tuberculosis and Lung Disease. 10 (9) (pp 1041-1044), 2006. Date of Publication: September 2006.

AN: 2006447198

OBJECTIVE: To study the clinical and pathological patterns of tuberculosis (TB) involving the bone marrow in a tertiary care setting. METHODS: A retrospective study of cases of bone marrow TB diagnosed and treated at King Faisal Specialist Hospital and Research Center from 1990 to 2002. The main criterion for inclusion was the isolation of Mycobacterium tuberculosis from a bone marrow specimen. RESULTS: We identified 22 patients with culture-proven M. tuberculosis infection of the bone marrow. Underlying conditions found in this series include solid organ transplantation in 4 patients (18%), HIV infection in 2 (9%), and diabetes in 3 (14%). No risk factor was identified in 12 patients (55%). Histopathological findings were reported as granuloma in 19 patients (86%) and caseating granuloma in 3 (14%). The outcome was favourable for 11 patients (50%), while 10 (45%) died during hospitalisation and one was lost to follow-up. For patients who completed their regimens, the duration of treatment was 12 months. CONCLUSION: The isolation of M. tuberculosis from a bone marrow specimen is an indication of disseminated disease which carries a high mortality rate and requires prompt initiation of appropriate treatment. © 2006 The Union.

Institution

(Hakawi) Section of Infectious Diseases, Department of Medicine, King Fahad Medical City, Riyadh, Saudi Arabia (Alrajhi) Section of Infectious Diseases, Department of Medicine, King Faisal Specialist Hospital and Research Center, Riyadh, Saudi Arabia

(Hakawi) Section of Infectious Diseases, Department of Medicine, King Fahad Medical City, P O Box 59046, Riyadh 11525, Saudi Arabia

Publisher

International Union against Tubercul. and Lung Dis.

Emtree Heading

anamnesis; article; bacterium culture; *bone marrow; bone marrow examination; clinical article; controlled study; diabetes mellitus; *extrapulmonary tuberculosis/di [Diagnosis]; *extrapulmonary tuberculosis/dt [Drug Therapy]; female; granuloma/di [Diagnosis]; histopathology; human; Human immunodeficiency virus infection; human tissue; male; organ transplantation; outcome assessment; pathology; priority journal; risk factor; Saudi Arabia; tertiary health care; thorax radiography; *tuberculous osteomyelitis/di [Diagnosis]; *tuberculous osteomyelitis/dt [Drug Therapy]; corticosteroid/dt [Drug Therapy]; ethambutol/dt [Drug Therapy]; isoniazid/dt [Drug Therapy]; pyrazinamide/dt [Drug Therapy]; rifampicin/dt [Drug Therapy].

Link to the Ovid Full Text or citation:

Click here for full text options

268.

Issues in the usages of new anti-rheumatic drugs in Japan.

Miyasaka N.

Japan Medical Association Journal. 49 (5-6) (pp 208-211), 2006. Date of Publication: May/June 2006.

AN: 2006385523

Rheumatoid arthritis (RA) is an inflammatory disease with its principal lesions appearing in the synovial membranes of joints. Historically, the objectives of the treatment were improvement of activity of daily living (ADL) and quality of life (QOL). However, the objective has now shifted to prevention of joint destruction due to the following reasons: 1) early diagnosis of RA has become possible; 2) early intervention with disease-modifying antirheumatic agents (DMARDs) has exhibited high efficacy; and 3) biological agents have been introduced. However, in Japan, as compared with the U.S. and Europe, there are disparities in the use of DMARDs and biological agents, and in their adverse effects. Physicians should acknowledge these disparities when providing treatment.

Institution

(Miyasaka) Department of Medicine and Rheumatology, Graduate School, Tokyo Medical and Dental University, 1-5-45 Yushima, Bunkyo-ku, Tokyo 113-8519, Japan

Publisher

Japan Medical Association

Emtree Heading

aging; clinical protocol; daily life activity; diabetes mellitus; disease activity; disease exacerbation; dose response; drug efficacy; drug utilization; early diagnosis; early intervention; geographic distribution; human; hypoalbuminemia; incidence; infection risk; interstitial pneumonia/si [Side Effect]; Japan; joint destruction/dt [Drug Therapy]; joint destruction/pc [Prevention]; lung disease; mortality; opportunistic infection/si [Side Effect]; Pneumocystis pneumonia/si [Side Effect]; pneumonia/si [Side Effect]; postmarketing surveillance; quality of life; race difference; review; *rheumatoid arthritis/di [Diagnosis]; *rheumatoid arthritis/dt [Drug Therapy]; risk assessment; risk factor; treatment indication; treatment outcome; tuberculosis/si [Side Effect]; *antirheumatic agent/ae [Adverse Drug Reaction]; *antirheumatic agent/cb [Drug Combination]; *antirheumatic agent/dt [Drug Therapy]; corticosteroid derivative/dt [Drug Therapy]; disease modifying antirheumatic drug/ae [Adverse Drug Reaction]; disease modifying antirheumatic drug/cb [Drug Combination]; disease modifying antirheumatic drug/dt [Drug Therapy]; etanercept/dt [Drug Therapy]; infliximab/ae [Adverse Drug Reaction]; infliximab/cb [Drug Combination]; infliximab/dt [Drug Therapy]; leflunomide/ae [Adverse Drug Reaction]; leflunomide/dt [Drug Therapy]; methotrexate/cb [Drug Combination]; methotrexate/do [Drug Dose]; methotrexate/dt [Drug Therapy]; nonsteroid antiinflammatory agent/ae [Adverse Drug Reaction]; nonsteroid antiinflammatory agent/dt [Drug Therapy].

Link to the Ovid Full Text or citation:

Click here for full text options

269.

Evaluation of subcutaneous proleukin (interleukin-2) in a randomized international trial (ESPRIT): Geographical and gender differences in the baseline characteristics of participants.

Pett S.L., Wand H., Law M.G., Arduino R., Lopez J.C., Knysz B., Pereira L.C., Pollack S., Reiss P., Tambussi G., Abrams D.I., Cooper D.A., Darbyshire J.H., Duncan W.R., Emery S., Clifford Lane H., Lehrman S., Lundgren J.D., Neaton J.D., Aguilar L., Angel E.B., Aquilia S., Belloso W., Benetucci J., Bittar V., Cahn P., Casiro A., Contarelli J., Corral J., Daciuk L., David D., Ines F., Fridman D., Galache V., Guaragna G., Ivalo S., Laplume H., Lasala M.B., Lattes R., Lasovsky J., Lopardo G., Losso M., Lourtau L., Lupo S., Maranzana A., Marson C., Massera L., del Lujan Sanchez M., Somenzini C., Tocci M., Algar S., Anderson J., Austin D., Baker D., Blavius K., Bloch M., Boyle M., Bradford D., Carrall L., Carr A., Chuah J., Curry M., D'Arcy-Evans C., Dobson P., Doong N., Ferguson W., Finlayson R., French M., Gold J., Habel P., Hoy J., Hudson J., James R., Jeganathan S., Leung J., Lowe K., MacRae K., Mallal S., McMurchie M., Medland N., Mijch A., Miller S., Murray J., Orth D., Patching J., Primrose R., Quan D., Rawlinson M., Ree H., Richardson R., Rogers G., Roney J., Roth N., Shaw D., Soo T.M., Sowden D., Street A., Vale R.

HIV Clinical Trials. 7 (2) (pp 70-85), 2006. Date of Publication: March/April 2006.

AN: 2006345637

Background: ESPRIT, is a phase III, open-label, randomized, international clinical trial evaluating the effects of subcutaneous recombinant interleukin-2 (rIL-2) plus antiretroviral therapy (ART) versus ART alone on HIV-disease progression and death in HIV-1-infected individuals with CD4+ T-cells >300 cells/muL. Objectives: To describe the baseline characteristics of participants randomized to ESPRIT overall and by geographic location. Method: Baseli ne characteristics of randomized participants were summarized by region. Results: 4,150 patients were enrolled in ESPRIT from 254 sites in 25 countries. 41%, 27%, 16%, 11%, and 5% were enrolled in Europe, North America, South America, Asia, and Australia, respectively. The median age was 40 years, 81% were men, and 76%, 11%, and 9% were Caucasian, Asian, and African American or African, respectively. 44% of women enrolled (n = 769) were enrolled in Thailand and Argentina. Overall, 55% and 38% of the cohort acquired HIV through male homosexual and heterosexual contact, respectively. 25% had a prior history of AIDS-defining illness; Pneumocystis jirovecii pneumonia, M. tuberculosis, and esophageal candida were most commonly reported. Median nadir and baseline CD4+ T-cell counts were 199 and 458 cells/muL, respectively. 6% and 13% were hepatitis B or C virus coinfected, respectively. Median duration of antiretroviral therapy (ART) was 4.2 years; the longest median duration was in Australia (5.2 years) and the shortest was in Asia (2.3 years). 17%, 13%, and 69% of participants began ART before 1995, between 1996 and 1997, and from 1998 onward, respectively. 86% used ART from two or more ART classes, with 49% using a protease inhibitor-based regimen and 46% using a nonnucleoside reverse transcriptase inhibitor-based regimen. 78% had plasma HIV RNA below detection (<500 cp/mL). Conclusion: ESPRIT has enrolled a diverse population of HIV-infected individuals including large populations of women and patients of African-American/African and Asian ethnicity often underrepresented in HIV research. As a consequence, the results of the study may have wide global applicability. © 2006 Thomas Land Publishers, Inc.

Institution

(Pett, Wand, Law) National Centre in HIV Epidemiology and Clinical Research, University of New South Wales, 376 Victoria St., Sydney, NSW 2010, Australia (Arduino) University of Texas Medical School of Houston, Houston, TX, United States

(Lopez) Hospital Universitario Gergorio Maranon, Servicio de Micobiologica y Enf. Infecciosas, Madrid, Spain

(Knysz) Medical University of Wroclaw, Department of Infectiou Diseases, Wroclaw University School of Medicine, Wroclaw, Poland

(Pereira) Instituto de Infectologia Emillio Ribas, Sao Paulo, Brazil

(Pollack) Rambam Medical Center, Immunology Allergy and AIDS Institute, B. Rappaport Faculty of Medicine, Technion, Haifa, Israel

(Reiss) Academish Medisch Centrum - Amsterdam, Amsterdam, Netherlands

(Tambussi) Ospedale San Raffaele, Divisione Malattie Infettive - Centro San Luigi, Milano, Italy

(Aguilar, Angel, Aquilia, Belloso, Benetucci, Bittar, Cahn, Casiro, Contarelli, Corral, Daciuk, David, Ines, Fridman, Galache, Guaragna, Ivalo, Laplume, Lasala, Lattes, Lasovsky, Lopardo, Losso, Lourtau, Lupo, Maranzana, Marson, Massera, del Lujan Sanchez, Somenzini, Tocci) Argentina National Coordinating Center, Argentina

(Algar, Anderson, Austin, Baker, Blavius, Bloch, Boyle, Bradford, Carrall, Carr, Chuah, Curry, D'Arcy-Evans, Dobson, Doong, Ferguson, Finlayson, French, Gold, Habel, Hoy, Hudson, James, Jeganathan, Leung, Lowe, MacRae, Mallal, McMurchie, Medland, Mijch, Miller, Murray, Orth, Patching, Primrose, Quan, Rawlinson, Ree, Richardson, Rogers, Roney, Roth, Shaw, Soo, Sowden, Street, Street, Vale) Australia National Trial Coordinating Center, Australia

Publisher

Thomas Land Publishers Inc.

Emtree Heading

adult; African American; AIDS related complex; anamnesis; Argentina; article; Asia; Asian; Australia; bacterial pneumonia; candidiasis; CD4 lymphocyte count; clinical trial; cohort analysis; controlled clinical trial; controlled study; death; diabetes mellitus/dt [Drug Therapy]; diabetes mellitus/si [Side Effect]; disease course; disease duration; disease transmission; drug efficacy; drug safety; dyslipidemia/dt [Drug Therapy]; dyslipidemia/si [Side Effect]; ethnology; Europe; female; geographic distribution; heart infarction/si [Side Effect]; hepatitis B/dt [Drug Therapy]; hepatitis C/dt [Drug Therapy]; herpes simplex/dt [Drug Therapy]; herpes simplex/pc [Prevention]; herpes simplex/si [Side Effect]; heterosexuality; HIV associated lipodystrophy/si [Side Effect]; human; Human immunodeficiency virus; *Human immunodeficiency virus infection/dt [Drug Therapy]; hypertension/dt [Drug Therapy]; hypertension/si [Side Effect]; intravenous drug abuse; lipid storage; major clinical study; male; male homosexual; methodology; Negro; nonhuman; North America; open study; phase 3 clinical trial; priority journal; randomized controlled trial; sex difference; side effect/si [Side Effect]; South America; stroke/si [Side Effect]; superinfection; Thailand; time series analysis; treatment duration; treatment outcome; tuberculosis; virus load; antihypertensive agent/dt [Drug Therapy]; antilipemic agent/dt [Drug Therapy]; *antiretrovirus agent/ae [Adverse Drug Reaction]; *antiretrovirus agent/ct [Clinical Trial]; *antiretrovirus agent/cb [Drug Combination]; *antiretrovirus agent/cm [Drug Comparison]; *antiretrovirus agent/dt [Drug Therapy]; hepatitis B surface antigen/ec [Endogenous Compound]; hepatitis B vaccine/dt [Drug Therapy]; herpes vaccine/dt [Drug Therapy]; insulin/dt [Drug Therapy]; oral antidiabetic agent/dt [Drug Therapy]; oral antidiabetic agent/po [Oral Drug Administration]; proteinase inhibitor/ae [Adverse Drug Reaction]; proteinase inhibitor/ct [Clinical Trial]; proteinase inhibitor/cb [Drug Combination]; proteinase inhibitor/cm [Drug Comparison]; proteinase inhibitor/dt [Drug Therapy]; *recombinant interleukin 2/ad [Drug Administration]; *recombinant interleukin 2/cb [Drug Combination]; *recombinant interleukin 2/cm [Drug Comparison]; *recombinant interleukin 2/dt [Drug Therapy]; *recombinant interleukin 2/sc [Subcutaneous Drug Administration]; ritonavir/ae [Adverse Drug Reaction]; ritonavir/ct [Clinical Trial]; ritonavir/cb [Drug Combination]; ritonavir/cm [Drug Comparison]; ritonavir/dt [Drug Therapy]; RNA directed DNA polymerase inhibitor/ae [Adverse Drug Reaction]; RNA directed DNA polymerase inhibitor/ct [Clinical Trial]; RNA directed DNA polymerase inhibitor/cb [Drug Combination]; RNA directed DNA polymerase inhibitor/cm [Drug Comparison]; RNA directed DNA polymerase inhibitor/dt [Drug Therapy]; virus RNA/ec [Endogenous Compound].

Drug Trade Names and Manufacturers

proleukin

Link to the Ovid Full Text or citation:

Click here for full text options

270.

Development and validation of the medication-based disease burden index.

George J., Vuong T., Bailey M.J., Kong D.C.M., Marriott J.L., Stewart K.

Annals of Pharmacotherapy. 40 (4) (pp 645-650), 2006. Date of Publication: April 2006.

AN: 2006208149

BACKGROUND: Medication lists offer an alternative source of data on comorbidities and disease burden. OBJECTIVE: To develop and validate the Medication-Based Disease Burden Index (MDBI). METHODS: A list of medication corresponding to the leading causes of global death was pilot tested and finalized by an expert panel. The resulting index was tested on drug regimens of patients at risk of medication misadventure. Criterion validity of the index was established against Charlsons's Index and Chronic Disease Score (CDS). Sensitivity, specificity, predictive validity, convergent and discriminant validity, and interrater and test-retest reliabilities of the index were also assessed. RESULTS: The MDBI consisting of specific medication for 20 chronic medical conditions and corresponding disability weightings was developed. The MDBI was tested on 317 patients with mean +/- SD Charlson's index scores of 2.8 +/- 2.2 and CDS scores of 7.3 +/- 2.8. Mean MDBI scores (0.33 +/- 0.28) demonstrated significant correlations with Charlson's index scores (r = 0.30; p < 0.001) and CDS (r = 0.53; p < 0.001). MDBI had satisfactory sensitivity and high specificity. Age of the patients and number of medications had significant correlation with the MDBI scores, but the MDBI scores were not significantly different in males and females. MDBI scores could successfully predict death and planned or unplanned readmissions (OR = 4.7, 95% CI 1.4 to 15.5; p = 0.01). MDBI demonstrated high inter-rater (intraclass correlation coefficient [ICC] = 0.99) and test-retest reliabilities (ICC = 0.98). CONCLUSIONS: Initial testing suggests that MDBI could offer an alternative low-cost and convenient method for quantifying disease burden and predicting health outcomes.

Institution

(George, Vuong, Marriott, Stewart) Department of Pharmacy Practice, Victorian College of Pharmacy, Monash University, Parkville, Vic., Australia (Bailey) Department of Epidemiology and Preventive Medicine, Faculty of Medicine, Monash University, Parkville, Vic., Canada

(Kong) Pharmacy Department, Alfred Hospital, Commercial Road, Melbourne, Vic., Australia

(Stewart) Department of Pharmacy Practice, Victorian College of Pharmacy, Monash University, 381 Royal Parade, Parkville, Vic. 3052, Australia

Publisher

Harvey Whitney Books Company

Emtree Heading

acquired immune deficiency syndrome/dt [Drug Therapy]; adult; aged; Alzheimer disease/dt [Drug Therapy]; article; asthma/dt [Drug Therapy]; cancer/dt [Drug Therapy]; cerebrovascular disease/dt [Drug Therapy]; *chronic disease/dt [Drug Therapy]; chronic obstructive lung disease/dt [Drug Therapy]; *comorbidity; dementia/dt [Drug Therapy]; diabetes mellitus/dt [Drug Therapy]; disease severity; epilepsy/dt [Drug Therapy]; female; health service; hepatitis B/dt [Drug Therapy]; hepatitis C/dt [Drug Therapy]; human; Human immunodeficiency virus infection/dt [Drug Therapy]; hypertension/dt [Drug Therapy]; ischemic heart disease/dt [Drug Therapy]; liver cirrhosis/dt [Drug Therapy]; major clinical study; male; *Medication Based Disease Burden Index; nephritis/dt [Drug Therapy]; nephrosis/dt [Drug Therapy]; outcomes research; Parkinson disease/dt [Drug Therapy]; peptic ulcer/dt [Drug Therapy]; pharmacist; prediction; priority journal; prostate hypertrophy/dt [Drug Therapy]; rheumatic heart disease/dt [Drug Therapy]; risk factor; *scoring system; skin disease/dt [Drug Therapy]; treatment outcome; tuberculosis/dt [Drug Therapy]; abciximab/dt [Drug Therapy]; acetylsalicylic acid/cb [Drug Combination]; acetylsalicylic acid/dt [Drug Therapy]; alpha interferon/cb [Drug Combination]; alpha interferon/dt [Drug Therapy]; anticonvulsive agent/dt [Drug Therapy]; antineoplastic agent/dt [Drug Therapy]; antivirus agent/cb [Drug Combination]; antivirus agent/dt [Drug Therapy]; benzathine penicillin/dt [Drug Therapy]; cholinergic receptor blocking agent/cb [Drug Combination]; cholinergic receptor blocking agent/dt [Drug Therapy]; cholinergic receptor blocking agent/ih [Inhalational Drug Administration]; cholinesterase inhibitor/dt [Drug Therapy]; corticosteroid/cb [Drug Combination]; corticosteroid/dt [Drug Therapy]; corticosteroid/ih [Inhalational Drug Administration]; corticosteroid/tp [Topical Drug Administration]; dipeptidyl carboxypeptidase inhibitor/dt [Drug Therapy]; dipyridamole/cb [Drug Combination]; dipyridamole/dt [Drug Therapy]; donepezil/dt [Drug Therapy]; eptifibatide/dt [Drug Therapy]; finasteride/dt [Drug Therapy]; insulin/dt [Drug Therapy]; isoniazid/dt [Drug Therapy]; levodopa/dt [Drug Therapy]; nedocromil sodium/dt [Drug Therapy]; nitrate/dt [Drug Therapy]; novel erythropoiesis stimulating protein/dt [Drug Therapy]; oral antidiabetic agent/dt [Drug Therapy]; oral antidiabetic agent/po [Oral Drug Administration]; proteinase inhibitor/dt [Drug Therapy]; proton pump inhibitor/dt [Drug Therapy]; pyrazinamide/dt [Drug Therapy]; RNA directed DNA polymerase inhibitor/dt [Drug Therapy]; spironolactone/dt [Drug Therapy]; tirofiban/dt [Drug Therapy]; unindexed drug; xanthine derivative/dt [Drug Therapy].

Link to the Ovid Full Text or citation:

Click here for full text options

271.

Sinogenic intracranial empyema in children.

Adame N., Hedlund G., Byington C.L.

Pediatrics. 116 (3) (pp e461-e467), 2005. Date of Publication: September 2005.

AN: 2006431198

Background. Sinogenic intracranial empyema (SIE) is an uncommon complication of sinusitis that can lead to devastating neurologic sequelae. Early recognition of the clinical findings is critical so that proper management can be instituted. Objective. To describe the symptoms, signs, and laboratory and imaging findings from one of the largest pediatric SIE case series reported. Methods. Descriptive study of a retrospective cohort of all children admitted to Primary Children's Medical Center with SIE between June 2000 and February 2004. Children were identified by a computerized search of Primary Children's Medical Center medical records using the terms "sinusitis" and "brain/subdural/epidural empyema." Erythrocyte sedimentation rate (ESR) and C-reactive protein (CRP) values of children with SIE were compared with a group of children with uncomplicated sinusitis cared for in the same health care system as outpatients. The medical records of the uncomplicated sinusitis group were not reviewed for any clinical or radiographic data other than CRP and ESR values. Results. Twelve children with SIE were identified. The median age of children with SIE was 11.5 years. Symptoms were usually present 10 days (median) before diagnosis and included headache (10), fever (11), nausea/vomiting (7), mental-status changes (5), and seizures (3). Physical findings included abnormal neurologic examination (9), Pott's puffy tumor (4), and orbital cellulitis (3). Using the Intermountain Health Care system's computerized database, 142 children with uncomplicated sinusitis treated as outpatients were identified. Children with SIE had markedly higher CRP levels (median: 10.05 mg/dL) and ESRs (median: 87 mm/hour) than those with uncomplicated sinusitis (median CRP: 0.7 mg/dL; median ESR: 6 mm/hour). Four children had hyperglycemia. Four children had a lumbar puncture at presentation, and the findings were normal for all of them. Craniofacial imaging included computed tomography (CT) and magnetic resonance imaging (MRI). SIE was not detected in 4 patients who had nonenhanced CT. Axial imaging alone was unable to demonstrate SIE in 1 child with sphenoid and ethmoid sinusitis, and coronal images were needed to demonstrate its presence and extent. The initial facial/orbital imaging studies in 2 patients with physical signs of complicated sinusitis (orbital cellulitis and Pott's puffy tumor) were not adequate to detect SIE. Using contrast-enhanced head CT or MRI, SIE was diagnosed in all 12 children. Conclusions. Children with sinusitis and any neurologic finding, signs of complicated sinusitis such as Pott's puffy tumor or orbital cellulitis, or persistent headache, fever, or nausea and vomiting after antibiotic therapy should have additional evaluation for SIE. Children with hyperglycemia or diabetes may be at increased risk for SIE. The ESR and CRP levels are markedly elevated in children with SIE and may be useful screening tools. MRI with gadolinium is the preferred method to diagnose SIE. If MRI is unavailable, a contrast-enhanced head CT with axial and coronal planes should be obtained. Nonenhanced CT alone lacks sensitivity, and a normal study may be falsely reassuring. Copyright © 2005 by the American Academy of Pediatrics.

Institution

(Adame, Byington) Department of Pediatrics, Primary Children's Medical Center, University of Utah, Salt Lake City, UT, United States (Hedlund) Department of Medical Imaging, Primary Children's Medical Center, Salt Lake City, UT, United States

(Adame) Department of Pediatrics, Division of Critical Care, 7703 Floyd Curl Dr, San Antonio, TX 78229-3900, United States

Publisher

American Academy of Pediatrics

Emtree Heading

adult; article; clinical feature; clinical trial; controlled clinical trial; controlled study; diagnostic imaging; *empyema/co [Complication]; *empyema/di [Diagnosis]; erythrocyte sedimentation rate; female; glucose blood level; health care system; human; hyperglycemia; male; medical record; neurologic examination; orbit cellulitis/co [Complication]; preschool child; priority journal; school child; screening; sinusitis/dm [Disease Management]; symptomatology; temperature measurement; treatment outcome; tuberculous spondylitis/co [Complication]; C reactive protein/ec [Endogenous Compound]; glucose/ec [Endogenous Compound]; glycosylated hemoglobin/ec [Endogenous Compound].

Link to the Ovid Full Text or citation:

Click here for full text options

272.

Cohort analysis for treatment outcomes TB pulmonary cases bacteriologically confirmed in Poland. <Skutecznosc leczenia przeciwpratkowego chorych na gruzlice pluc potwierdzona bakteriologicznie w Polsce.>

Szczuka I.

Pneumonologia i Alergologia Polska. 73 (3) (pp 245-253), 2005. Date of Publication: 2005.

AN: 2006186978

In Poland since 1986 system of treatment outcomes monitoring (TOM) of all confirmed pulmonary TB (PTB) cases has been introduced. This system is based on the Central Tuberculosis Register TB detected and registered in given year from the whole network tuberculosis and lung diseases dispensaries in the country -/ 400 disp. in 2004 yr/ The aim of the study was to asses treatment results of PTB confirmed cases registered in 2003 and in the period 2001-2003. In 2003 5558 cases were registered, The treatment outcomes were assessed according to WHO /Euro TB - categories after 12 months after start of treatment as PTB: cured - 67,8%; completed - 10,4% (success ratio - 77,8%); failed - 1,5%, died - 5,1% (from TB - 2,1%); defaulted - 7,6%, still on treatment - 0,6%; transferred - 1,9% and unknown - 5,5% all registered PTB cases. In period 2001-2003 proportion patients with success of treatment was stable. In 2003 proportion of patients with success of treatment was significantly higher in a groups of new cases - 83,7% than in patients with relapses - 73,9%; among women - 85,4% than in men - 80,9%. Among PTB cases smear positive success ratio - 81,3% was lower than among PTB cases culture positive - 84,0%. Among patients with diabetes and alcohol abuse the success of treatment was significantly lower than more patients without these diseases /habit. Alcoholics three times more frequently defaulted treatment than patients without this habit. Conclusion: the improvement of treatment outcomes is impossible without generalize directly observed treatment in all patients in continuation phase of treatment.

Institution

(Szczuka) Z Zakladu Epidemiologii I Organizacji Walki Z Gruzlica Instytutu Gruzlicy I Chorob Puc, p.o. kierownik dr med. I. Szczuka

Publisher

Instytut Gruzlicy i Chorob Pluc

Emtree Heading

alcohol abuse; article; cohort analysis; controlled study; diabetes mellitus; disease registry; female; human; *lung tuberculosis/ep [Epidemiology]; major clinical study; male; mortality; Poland; relapse; treatment outcome; world health organization.

Link to the Ovid Full Text or citation:

Click here for full text options

273.

Breastfeeding and the use of human milk.

Anonymous

Pediatrics. 115 (2) (pp 496-506), 2005. Date of Publication: February 2005.

AN: 2006067823

Considerable advances have occurred in recent years in the scientific knowledge of the benefits of breastfeeding, the mechanisms underlying these benefits, and in the clinical management of breastfeeding. This policy statement on breastfeeding replaces the 1997 policy statement of the American Academy of Pediatrics and reflects this newer knowledge and the supporting publications. The benefits of breastfeeding for the infant, the mother, and the community are summarized, and recommendations to guide the pediatrician and other health care professionals in assisting mothers in the initiation and maintenance of breastfeeding for healthy term infants and high-risk infants are presented. The policy statement delineates various ways in which pediatricians can promote, protect, and support breastfeeding not only in their individual practices but also in the hospital, medical school, community, and nation. Copyright © 2005 by the American Academy of Pediatrics.

Publisher

American Academy of Pediatrics

Emtree Heading

Africa; ambulatory care; bacteremia/ep [Epidemiology]; bacteremia/pc [Prevention]; bacterial meningitis/ep [Epidemiology]; bacterial meningitis/pc [Prevention]; brain development; *breast feeding; *breast feeding education; breast lesion; breast milk; cancer radiotherapy; child care; child health; clinical practice; cognitive development; community care; cost benefit analysis; cost minimization analysis; developing country; diarrhea/ep [Epidemiology]; diarrhea/pc [Prevention]; disease severity; drug abuse; fever; galactose 1 phosphate uridyltransferase deficiency; health care cost; health care delivery; health care management; health care personnel; *health care policy; health care quality; health program; health promotion; hepatitis C; herpes simplex; high risk patient; high risk population; hospital care; human; human t cell leukemia virus 1; hyperbilirubinemia; infant disease; infection control; infection prevention; insulin dependent diabetes mellitus/ep [Epidemiology]; insulin dependent diabetes mellitus/pc [Prevention]; jaundice; lactation; maternal behavior; maternal care; maternal disease; maternal welfare; medical literature; medical practice; medical school; necrotizing enterocolitis/ep [Epidemiology]; necrotizing enterocolitis/pc [Prevention]; newborn mortality; newborn screening; non insulin dependent diabetes mellitus/ep [Epidemiology]; non insulin dependent diabetes mellitus/pc [Prevention]; nutritional deficiency; otitis media/ep [Epidemiology]; otitis media/pc [Prevention]; outcomes research; pediatrician; perinatal development; population research; postpartum hemorrhage/pc [Prevention]; practice guideline; prematurity; prenatal care; priority journal; public health service; radiodiagnosis; respiratory tract infection/ep [Epidemiology]; respiratory tract infection/pc [Prevention]; review; risk assessment; risk reduction; sepsis/ep [Epidemiology]; sepsis/pc [Prevention]; smoking; sudden infant death syndrome/ep [Epidemiology]; sudden infant death syndrome/pc [Prevention]; T cell leukemia; treatment contraindication; tuberculosis; United States; urinary tract infection/ep [Epidemiology]; urinary tract infection/pc [Prevention]; virus transmission; antiinfective agent; antimetabolite; antineoplastic agent.

Link to the Ovid Full Text or citation:

Click here for full text options

274.

Treatment results of multi drug resistant tuberculosis, a hospital based study.

Yoshiyama T., Ogata H., Wada M.

Kekkaku. 80 (11) (pp 687-693), 2005. Date of Publication: November 2005.

AN: 2005563930

[Settings] Fukujuji Hospital, Japan. [Purpose] To evaluate treatment results of multi drug resistant tuberculosis cases. [Method] Retrospective review of 100 multi drug resistant tuberculosis cases who started treatment in 1990-1999 at Fukujuji Hospital. Life table analysis and analysis of long-term treatment results were done. [Result] Observation for more than 3 years after starting treatment revealed cure in 62, chronic excreters in 2, and died cases with positive culture in 10, and treatment completed with shorter observation period in 8. The remaining 18 cases were either transfer-out, defaulted or died with other causes. The older age, cavitary lesions (bilateral gt; nilateral > no), resistance to more drugs, interruption of treatment due to side effects to second line drugs and the complication of diabetes mellitus were independent risk factors for unfavorable results. The treatment results improved with the increase of the number of effective drugs used for 6 months or more. The surgical intervention improved the proportion of favorable treatment results for those that were culture positive after 5 months of treatment. [Discussion] Treatment results of MDR TB was not satisfactory. Diabetics was the risk factor of unfavorable results. The conditions for the indication of surgical intervention among culture negative cases after 5 months of medical treatment needs to be further investigated.

Institution

(Yoshiyama, Ogata) Fukujuji Hospital, Japan Anti-Tuberculosis Association (JATA), Tokyo, Japan (Wada) Research Institute of Tuberculosis, JATA, Tokyo, Japan

(Yoshiyama) Fukujuji Hospital, JATA, 3-1-24, Matsuyama, Kiyose-shi, Tokyo 204-8522, Japan

Publisher

Japanese Society for Tuberculosis

Emtree Heading

adult; aged; article; bacterium culture; diabetes mellitus; drug efficacy; female; hospital patient; human; life table; major clinical study; male; *multidrug resistance; Mycobacterium tuberculosis; nonhuman; relapse; retrospective study; risk factor; sputum smear; treatment outcome; *tuberculosis/di [Diagnosis]; *tuberculosis/dr [Drug Resistance]; *tuberculosis/dt [Drug Therapy]; *tuberculosis/ep [Epidemiology]; *tuberculosis/su [Surgery]; ethambutol/cm [Drug Comparison]; ethambutol/dt [Drug Therapy]; ethionamide/cm [Drug Comparison]; ethionamide/dt [Drug Therapy]; kanamycin/cm [Drug Comparison]; kanamycin/dt [Drug Therapy]; streptomycin/cm [Drug Comparison]; streptomycin/dt [Drug Therapy]; tuberculostatic agent/cm [Drug Comparison]; tuberculostatic agent/dt [Drug Therapy].

Link to the Ovid Full Text or citation:

Click here for full text options

275.

Spinal infections.

Govender S.

Journal of Bone and Joint Surgery - Series B. 87 (11) (pp 1454-1458), 2005. Date of Publication: November 2005.

AN: 2005547383

Institution

(Govender) Department of Orthopaedic Surgery, University of Natal, Private Bag 7, Congella 4013 Durban, South Africa

Publisher

British Editorial Society of Bone and Joint Surgery

Emtree Heading

alpha hemolytic Streptococcus; antibiotic therapy; arterial circulation; aspiration biopsy; blood culture; clinical feature; comorbidity; computer assisted tomography; debridement; diabetes mellitus; diagnostic accuracy; disease predisposition; disease severity; drug contraindication; drug indication; early diagnosis; epidural abscess; erythrocyte sedimentation rate; human; Human immunodeficiency virus infection; immune deficiency; incidence; infection risk; kidney failure; laminectomy; morbidity; mortality; Mycobacterium tuberculosis; mycosis; myelography; neurologic disease/co [Complication]; nuclear magnetic resonance imaging; parenteral nutrition; pathophysiology; priority journal; protein malnutrition; review; risk factor; Salmonella; sensitivity and specificity; septicemia; side effect/si [Side Effect]; spinal pain; spine stabilization; *spondylitis/di [Diagnosis]; *spondylitis/dt [Drug Therapy]; *spondylitis/si [Side Effect]; *spondylitis/su [Surgery]; *spondylitis/th [Therapy]; Staphylococcus aureus; substance abuse; treatment outcome; tuberculosis/dt [Drug Therapy]; venous circulation; amphotericin B/ae [Adverse Drug Reaction]; amphotericin B/cb [Drug Combination]; amphotericin B/dt [Drug Therapy]; antibiotic agent/ae [Adverse Drug Reaction]; antibiotic agent/cb [Drug Combination]; antibiotic agent/dt [Drug Therapy]; corticosteroid derivative/dt [Drug Therapy]; flucytosine/ae [Adverse Drug Reaction]; flucytosine/cb [Drug Combination]; flucytosine/dt [Drug Therapy]; steroid/ae [Adverse Drug Reaction].

Link to the Ovid Full Text or citation:

Click here for full text options

276.

ANCA-associated renal vasculitis at the end of the twentieth century - A disease of older patients.

Harper L., Savage C.O.

Rheumatology. 44 (4) (pp 495-501), 2005. Date of Publication: April 2005.

AN: 2005480793

Objective. Antineutrophil cytoplasmic antibody (ANCA)-associated vasculitides are increasingly recognized in older patients. However, it is unknown whether disease presentation and response to treatment differs from younger patients. We aimed to examine the presentation, response to treatment and outcome of patients over 65 yr of age compared with a younger cohort. Methods. This retrospective, single centre, sequential cohort study reports presenting features and outcome of 233 consecutive new patients with ANCA-associated vasculitis between 1990 and 2000. Results. The median age of all patients was 65 yr (range 16-90 yr). Older patients (>65 yr) presented with more severe renal involvement at presentation (P<0.001). Older patients were as likely to respond to treatment or undergo relapse as the younger patients. Older patients receiving immunosuppression had an increased risk of infection (P = 0.0027). Survival was worse in the older group (P = 0.016) and death occurred early. Mortality was associated with poor renal function (creatinine >400 mumol/l), infection and low serum albumin. Leucopenia was associated with severe renal impairment (P = 0.0048) and increased risk of infection (P = 0.0006). Multivariate analysis determined that serum creatinine >400 mumol/l and age were independent risk factors for poor prognosis. Conclusion. ANCA-associated vasculitis occurs frequently in older patients and physicians should maintain a high index of suspicion. Older patients have a poorer prognosis due to more severe renal involvement and increased sensitivity to adverse effects of treatment. This study highlights the importance of careful dosing of cyclophosphamide: In those aged over 65 yr a 25% dose reduction is safe and reduces the risk of leucopenia. This study further highlights the importance of renal function on prognosis and the need for less toxic treatment regimens. © British Society for Rheumatology 2005; all rights reserved.

Institution

(Harper, Savage) Division of Immunology and Infection, The Medical School, University of Birmingham, Edgbaston, Birmingham B15 2TT, United Kingdom

Publisher

Oxford University Press

Emtree Heading

adolescent; adult; age distribution; aged; *ANCA associated vasculitis/di [Diagnosis]; *ANCA associated vasculitis/dt [Drug Therapy]; article; bladder cancer/si [Side Effect]; cerebrovascular disease/si [Side Effect]; clinical feature; clinical trial; cohort analysis; controlled clinical trial; controlled study; creatinine blood level; death; deep vein thrombosis/si [Side Effect]; diabetes mellitus/si [Side Effect]; disease association; disease severity; drug dose reduction; drug dose regimen; drug fatality/si [Side Effect]; female; fracture/si [Side Effect]; health care facility; heart infarction/si [Side Effect]; human; immunosuppressive treatment; infection/dt [Drug Therapy]; infection/pc [Prevention]; infection/si [Side Effect]; *kidney disease/di [Diagnosis]; *kidney disease/dt [Drug Therapy]; kidney dysfunction/si [Side Effect]; leukopenia/si [Side Effect]; lower respiratory tract infection/si [Side Effect]; lung embolism/si [Side Effect]; major clinical study; male; malignant neoplastic disease/si [Side Effect]; mortality; multivariate analysis; neutropenia/si [Side Effect]; physician; pneumonia/si [Side Effect]; priority journal; prognosis; relapse; retrospective study; risk assessment; sensitivity analysis; sepsis/si [Side Effect]; skin cancer/si [Side Effect]; survival rate; treatment outcome; tuberculosis/si [Side Effect]; azathioprine/dt [Drug Therapy]; corticosteroid/ae [Adverse Drug Reaction]; corticosteroid/dt [Drug Therapy]; cotrimoxazole/dt [Drug Therapy]; creatinine/ec [Endogenous Compound]; cyclophosphamide/ae [Adverse Drug Reaction]; cyclophosphamide/ct [Clinical Trial]; cyclophosphamide/do [Drug Dose]; cyclophosphamide/dt [Drug Therapy]; immunosuppressive agent/ae [Adverse Drug Reaction]; immunosuppressive agent/ct [Clinical Trial]; immunosuppressive agent/do [Drug Dose]; immunosuppressive agent/dt [Drug Therapy]; methylprednisolone/dt [Drug Therapy]; prednisolone/dt [Drug Therapy]; serum albumin/ec [Endogenous Compound].

Drug Trade Names and Manufacturers

septrin

Link to the Ovid Full Text or citation:

Click here for full text options

277.

Treating the patient in pain.

Katz W.A., Rothenberg R.

Journal of Clinical Rheumatology. 11 (2 SUPPL.) (pp S16-S28), 2005. Date of Publication: April 2005.

AN: 2005199081

Physicians may choose from a variety of pharmacologic and nonpharmacologic options to treat patients with painful rheumatic diseases. Osteoarthritis (OA) is the most common type of arthritis requiring pain management. New disease-modifying antirheumatic drugs and biologic response modifiers can improve disease states in patients with rheumatoid arthritis (RA). After the inflammatory component of RA is minimized with such agents, treatment goals shift to those similar to secondary OA and other degenerative joint diseases. Relief of pain and improvement in functional status are essential components of effective therapy. A pure analgesic such as acetaminophen and nonsteroidal antiinflammatory drugs, including the cyclooxygenase-2-selective inhibitors for those at risk for gastrointestinal side effects, may be used at the lowest effective doses. Combination therapy for acetaminophen and an opioid may maximize pain relief and provide greater speed and duration of action than the separate components. Use of the atypical opioid tramadol with acetaminophen often results in an improved side-effect profile compared with stronger opioids, with similar levels of pain relief. Adjunctive therapy with agents such as topical analgesics, intraarticular hyaluron, tricyclic antidepressants, anticonvulsants, muscle relaxants, and anxiolytics may also be helpful. Nonpharmacologic therapies such as exercise, physical therapy, and psychologic counseling may also diminish pain and improve outcome in patients with rheumatic diseases. One may also consider yoga, acupuncture, biofeedback, massage, relaxation techniques, and other alternative therapies. Copyright © 2005 by Lippincott Williams & Wilkins.

Institution

(Katz) Department of Medicine, Univ. of PA School of Medicine, Philadelphia, PA, United States (Rothenberg) Department of Medicine, George Washington Univ. Med. Center, Washington, DC, United States

Publisher

Lippincott Williams and Wilkins

Emtree Heading

acupuncture; adjuvant therapy; alopecia/si [Side Effect]; alternative medicine; analgesia; *arthralgia/co [Complication]; *arthralgia/dm [Disease Management]; *arthralgia/dt [Drug Therapy]; *arthralgia/th [Therapy]; article; bleeding/si [Side Effect]; bone marrow suppression/si [Side Effect]; cardiovascular disease/si [Side Effect]; clinical practice; clinical trial; comorbidity; comparative study; constipation/si [Side Effect]; corticosteroid therapy; cryotherapy; Cushingoid syndrome/si [Side Effect]; diabetes mellitus/si [Side Effect]; diagnostic test; diet; digestive system ulcer/si [Side Effect]; disease association; disease exacerbation/si [Side Effect]; disease severity; dizziness/si [Side Effect]; doctor patient relation; drowsiness/si [Side Effect]; drug contraindication; drug cross reactivity; drug dosage form; drug dose regimen; drug effect; drug efficacy; drug hypersensitivity/si [Side Effect]; drug indication; drug potency; drug potentiation; edema/si [Side Effect]; exercise; feedback system; fluid retention; fracture healing/si [Side Effect]; functional assessment; gastrointestinal hemorrhage/si [Side Effect]; gastrointestinal symptom/si [Side Effect]; habituation; human; hypertension/si [Side Effect]; hyperthermic therapy; infection risk; kidney disease/si [Side Effect]; liver dysfunction/si [Side Effect]; liver level; liver toxicity/si [Side Effect]; low drug dose; massage; nausea/si [Side Effect]; occupational therapy; osteoarthritis/dt [Drug Therapy]; osteoporosis/si [Side Effect]; *pain/co [Complication]; *pain/dm [Disease Management]; *pain/dt [Drug Therapy]; *pain/th [Therapy]; pain assessment; pathophysiology; patient care; patient compliance; patient counseling; patient education; physician attitude; physiotherapy; polypharmacy; postoperative pain/co [Complication]; postoperative pain/dt [Drug Therapy]; priority journal; psychotherapy; relaxation training; respiration depression/si [Side Effect]; *rheumatic disease/dt [Drug Therapy]; rheumatoid arthritis/dt [Drug Therapy]; risk assessment; sedation; seizure; sepsis/si [Side Effect]; side effect/si [Side Effect]; social support; somnolence/si [Side Effect]; tablet formulation; tachyphylaxis; treatment outcome; treatment planning; tuberculosis/si [Side Effect]; vomiting/si [Side Effect]; weight gain; xerostomia/si [Side Effect]; yoga; *analgesic agent/ae [Adverse Drug Reaction]; *analgesic agent/ct [Clinical Trial]; *analgesic agent/ad [Drug Administration]; *analgesic agent/cb [Drug Combination]; *analgesic agent/cm [Drug Comparison]; *analgesic agent/do [Drug Dose]; *analgesic agent/it [Drug Interaction]; *analgesic agent/dt [Drug Therapy]; *analgesic agent/po [Oral Drug Administration]; *analgesic agent/pa [Parenteral Drug Administration]; *analgesic agent/pr [Pharmaceutics]; *analgesic agent/pk [Pharmacokinetics]; *analgesic agent/pd [Pharmacology]; *analgesic agent/tp [Topical Drug Administration]; anticonvulsive agent/dt [Drug Therapy]; antiinflammatory agent/ae [Adverse Drug Reaction]; antiinflammatory agent/dt [Drug Therapy]; antiinflammatory agent/pd [Pharmacology]; antipyretic agent; antipyretic analgesic agent/ae [Adverse Drug Reaction]; antipyretic analgesic agent/dt [Drug Therapy]; antipyretic analgesic agent/pd [Pharmacology]; anxiolytic agent/dt [Drug Therapy]; celecoxib/ae [Adverse Drug Reaction]; celecoxib/dt [Drug Therapy]; celecoxib/pd [Pharmacology]; corticosteroid/cb [Drug Combination]; corticosteroid/dt [Drug Therapy]; corticosteroid/pd [Pharmacology]; cyclooxygenase 2 inhibitor/ae [Adverse Drug Reaction]; cyclooxygenase 2 inhibitor/cb [Drug Combination]; cyclooxygenase 2 inhibitor/cm [Drug Comparison]; cyclooxygenase 2 inhibitor/do [Drug Dose]; cyclooxygenase 2 inhibitor/dt [Drug Therapy]; cyclooxygenase 2 inhibitor/pk [Pharmacokinetics]; cyclooxygenase 2 inhibitor/pd [Pharmacology]; diclofenac/ae [Adverse Drug Reaction]; diclofenac/dt [Drug Therapy]; diclofenac/pd [Pharmacology]; disease modifying antirheumatic drug/ae [Adverse Drug Reaction]; disease modifying antirheumatic drug/cb [Drug Combination]; disease modifying antirheumatic drug/cm [Drug Comparison]; disease modifying antirheumatic drug/do [Drug Dose]; disease modifying antirheumatic drug/dt [Drug Therapy]; disease modifying antirheumatic drug/po [Oral Drug Administration]; disease modifying antirheumatic drug/pd [Pharmacology]; hyaluronic acid/ad [Drug Administration]; hyaluronic acid/cb [Drug Combination]; hyaluronic acid/cm [Drug Comparison]; hyaluronic acid/dt [Drug Therapy]; hyaluronic acid/pd [Pharmacology]; hydroxychloroquine/ae [Adverse Drug Reaction]; hydroxychloroquine/cb [Drug Combination]; hydroxychloroquine/dt [Drug Therapy]; hydroxychloroquine/pd [Pharmacology]; hypnotic agent/dt [Drug Therapy]; ibuprofen/ae [Adverse Drug Reaction]; ibuprofen/dt [Drug Therapy]; ibuprofen/pd [Pharmacology]; leflunomide/ae [Adverse Drug Reaction]; leflunomide/cb [Drug Combination]; leflunomide/dt [Drug Therapy]; leflunomide/pd [Pharmacology]; long acting drug/dt [Drug Therapy]; methotrexate/ae [Adverse Drug Reaction]; methotrexate/cb [Drug Combination]; methotrexate/dt [Drug Therapy]; methotrexate/pd [Pharmacology]; muscle relaxant agent/dt [Drug Therapy]; naproxen/ae [Adverse Drug Reaction]; naproxen/dt [Drug Therapy]; naproxen/pd [Pharmacology]; nonsteroid antiinflammatory agent/ae [Adverse Drug Reaction]; nonsteroid antiinflammatory agent/cb [Drug Combination]; nonsteroid antiinflammatory agent/cm [Drug Comparison]; nonsteroid antiinflammatory agent/do [Drug Dose]; nonsteroid antiinflammatory agent/dt [Drug Therapy]; nonsteroid antiinflammatory agent/po [Oral Drug Administration]; nonsteroid antiinflammatory agent/pk [Pharmacokinetics]; nonsteroid antiinflammatory agent/pd [Pharmacology]; opiate/ae [Adverse Drug Reaction]; opiate/cb [Drug Combination]; opiate/cm [Drug Comparison]; opiate/do [Drug Dose]; opiate/dt [Drug Therapy]; opiate/pd [Pharmacology]; opiate/tp [Topical Drug Administration]; paracetamol/ae [Adverse Drug Reaction]; paracetamol/ct [Clinical Trial]; paracetamol/cb [Drug Combination]; paracetamol/cm [Drug Comparison]; paracetamol/do [Drug Dose]; paracetamol/dt [Drug Therapy]; paracetamol/pr [Pharmaceutics]; paracetamol/pk [Pharmacokinetics]; paracetamol/pd [Pharmacology]; recombinant interleukin 1 receptor blocking agent/ae [Adverse Drug Reaction]; recombinant interleukin 1 receptor blocking agent/cb [Drug Combination]; recombinant interleukin 1 receptor blocking agent/dt [Drug Therapy]; recombinant interleukin 1 receptor blocking agent/pd [Pharmacology]; salazosulfapyridine/ae [Adverse Drug Reaction]; salazosulfapyridine/cb [Drug Combination]; salazosulfapyridine/dt [Drug Therapy]; salazosulfapyridine/pd [Pharmacology]; short acting drug/dt [Drug Therapy]; tramadol/ae [Adverse Drug Reaction]; tramadol/ct [Clinical Trial]; tramadol/cb [Drug Combination]; tramadol/cm [Drug Comparison]; tramadol/do [Drug Dose]; tramadol/dt [Drug Therapy]; tramadol/pr [Pharmaceutics]; tramadol/pd [Pharmacology]; tramadol/tp [Topical Drug Administration]; tricyclic antidepressant agent/dt [Drug Therapy]; tumor necrosis factor antibody/ae [Adverse Drug Reaction]; tumor necrosis factor antibody/cb [Drug Combination]; tumor necrosis factor antibody/dt [Drug Therapy]; tumor necrosis factor antibody/pd [Pharmacology]; unindexed drug.

Link to the Ovid Full Text or citation:

Click here for full text options

278.

Challenging scenarios in a travel clinic: Advising the complex traveler.

Suh K.N., Mileno M.D.

Infectious Disease Clinics of North America. 19 (1) (pp 15-47), 2005. Date of Publication: March 2005.

AN: 2005069716

With adequate preparation and in consultation with a travel medicine expert, most travelers today can travel safely regardless of their age and health status. The few instances when it is prudent to alter travel plans or postpone travel altogether are not to be taken lightly. For the most part, however, most complex travelers can enjoy a healthy and rewarding travel experience.

Institution

(Suh) Division of Infectious Diseases, Children's Hosp. of Eastern Ontario, 401 Smyth Road, Ottawa, Ont. K1H 8L1, Canada (Suh) Department of Paediatrics, University of Ottawa, Ottawa, Ont., Canada

(Mileno) Miriam Hospital, 164 Summit Avenue, Providence, RI 02906, United States

(Mileno) Department of Medicine, Brown Medical School, Providence, RI, United States

Publisher

W.B. Saunders

Emtree Heading

acquired immune deficiency syndrome; age distribution; bacterial infection/dt [Drug Therapy]; bacterial infection/pc [Prevention]; biosafety; breast feeding; consultation; diabetes mellitus; diphtheria/dt [Drug Therapy]; diphtheria/pc [Prevention]; dose response; elderly care; epidemic encephalitis/dt [Drug Therapy]; epidemic encephalitis/pc [Prevention]; epilepsy; gastrointestinal disease/si [Side Effect]; health status; hepatitis A/dt [Drug Therapy]; hepatitis A/pc [Prevention]; hepatitis B/dt [Drug Therapy]; hepatitis B/pc [Prevention]; herpes zoster/dt [Drug Therapy]; herpes zoster/pc [Prevention]; human; Human immunodeficiency virus infection; ileus/si [Side Effect]; immunization; *infection/dt [Drug Therapy]; *infection/pc [Prevention]; influenza/dt [Drug Therapy]; influenza/pc [Prevention]; malaria/dt [Drug Therapy]; malaria/pc [Prevention]; marital therapy; measles/dt [Drug Therapy]; measles/pc [Prevention]; meningococcosis/dt [Drug Therapy]; meningococcosis/pc [Prevention]; mental disease/si [Side Effect]; mumps/dt [Drug Therapy]; mumps/pc [Prevention]; outpatient department; patient counseling; pediatrics; pertussis/dt [Drug Therapy]; pertussis/pc [Prevention]; poliomyelitis/dt [Drug Therapy]; poliomyelitis/pc [Prevention]; pregnancy; rabies/dt [Drug Therapy]; rabies/pc [Prevention]; respiration depression/si [Side Effect]; review; risk factor; rubella/dt [Drug Therapy]; rubella/pc [Prevention]; seizure; side effect/si [Side Effect]; South America; Streptococcus infection/dt [Drug Therapy]; Streptococcus infection/pc [Prevention]; survival; tetanus/dt [Drug Therapy]; tetanus/pc [Prevention]; tick borne encephalitis/dt [Drug Therapy]; tick borne encephalitis/pc [Prevention]; toxic megacolon/si [Side Effect]; travel; traveller diarrhea/dt [Drug Therapy]; treatment contraindication; treatment outcome; tuberculosis/dt [Drug Therapy]; tuberculosis/pc [Prevention]; typhoid fever/dt [Drug Therapy]; typhoid fever/pc [Prevention]; yellow fever/dt [Drug Therapy]; yellow fever/pc [Prevention]; atovaquone plus proguanil/do [Drug Dose]; atovaquone plus proguanil/dt [Drug Therapy]; bacterial vaccine/dt [Drug Therapy]; BCG vaccine/dt [Drug Therapy]; chloroquine/ae [Adverse Drug Reaction]; chloroquine/do [Drug Dose]; chloroquine/dt [Drug Therapy]; diphtheria pertussis tetanus vaccine/do [Drug Dose]; diphtheria pertussis tetanus vaccine/dt [Drug Therapy]; diphtheria pertussis tetanus vaccine/im [Intramuscular Drug Administration]; doxycycline/do [Drug Dose]; doxycycline/dt [Drug Therapy]; Haemophilus influenzae type b vaccine/do [Drug Dose]; Haemophilus influenzae type b vaccine/dt [Drug Therapy]; Haemophilus influenzae type b vaccine/im [Intramuscular Drug Administration]; hepatitis A antibody/do [Drug Dose]; hepatitis A antibody/dt [Drug Therapy]; hepatitis A antibody/im [Intramuscular Drug Administration]; hepatitis A vaccine/do [Drug Dose]; hepatitis A vaccine/dt [Drug Therapy]; hepatitis A vaccine/im [Intramuscular Drug Administration]; inactivated vaccine/do [Drug Dose]; inactivated vaccine/dt [Drug Therapy]; inactivated vaccine/im [Intramuscular Drug Administration]; influenza vaccine/do [Drug Dose]; influenza vaccine/dt [Drug Therapy]; influenza vaccine/im [Intramuscular Drug Administration]; Japanese encephalitis vaccine/do [Drug Dose]; Japanese encephalitis vaccine/dt [Drug Therapy]; Japanese encephalitis vaccine/sc [Subcutaneous Drug Administration]; live vaccine/do [Drug Dose]; live vaccine/dt [Drug Therapy]; loperamide/ae [Adverse Drug Reaction]; loperamide/dt [Drug Therapy]; malaria vaccine/dt [Drug Therapy]; measles mumps rubella vaccine/do [Drug Dose]; measles mumps rubella vaccine/dt [Drug Therapy]; measles mumps rubella vaccine/sc [Subcutaneous Drug Administration]; Meningococcus vaccine/do [Drug Dose]; Meningococcus vaccine/dt [Drug Therapy]; Meningococcus vaccine/im [Intramuscular Drug Administration]; Meningococcus vaccine/sc [Subcutaneous Drug Administration]; pertussis vaccine/dt [Drug Therapy]; Pneumococcus vaccine/do [Drug Dose]; Pneumococcus vaccine/dt [Drug Therapy]; Pneumococcus vaccine/im [Intramuscular Drug Administration]; Pneumococcus vaccine/sc [Subcutaneous Drug Administration]; poliomyelitis vaccine/do [Drug Dose]; poliomyelitis vaccine/dt [Drug Therapy]; poliomyelitis vaccine/im [Intramuscular Drug Administration]; poliomyelitis vaccine/sc [Subcutaneous Drug Administration]; polysaccharide vaccine/do [Drug Dose]; polysaccharide vaccine/dt [Drug Therapy]; polysaccharide vaccine/sc [Subcutaneous Drug Administration]; rabies vaccine/do [Drug Dose]; rabies vaccine/dt [Drug Therapy]; rabies vaccine/im [Intramuscular Drug Administration]; recombinant hepatitis B vaccine/do [Drug Dose]; recombinant hepatitis B vaccine/dt [Drug Therapy]; tetanus toxoid/dt [Drug Therapy]; tick borne encephalitis vaccine/dt [Drug Therapy]; typhoid vaccine/do [Drug Dose]; typhoid vaccine/dt [Drug Therapy]; typhoid vaccine/im [Intramuscular Drug Administration]; unindexed drug; *vaccine/ae [Adverse Drug Reaction]; *vaccine/do [Drug Dose]; *vaccine/dt [Drug Therapy]; *vaccine/im [Intramuscular Drug Administration]; *vaccine/sc [Subcutaneous Drug Administration]; varicella zoster vaccine/do [Drug Dose]; varicella zoster vaccine/dt [Drug Therapy]; yellow fever vaccine/do [Drug Dose]; yellow fever vaccine/dt [Drug Therapy]; yellow fever vaccine/sc [Subcutaneous Drug Administration].

Link to the Ovid Full Text or citation:

Click here for full text options

279.

Development of an ELISA for the determination of the major haptoglobin phenotype: An interesting technical development and its potential consequences.

Delanghe J.R., De Buyzere M.L.

Clinical Chemistry. 50 (11) (pp 1972-1973), 2004. Date of Publication: November 2004.

AN: 2004460370

Institution

(Delanghe, De Buyzere) Department of Clinical Chemistry, University Hospital Ghent (2P8), De Pintelaan 185, B9000 Ghent, Belgium

Publisher

American Association for Clinical Chemistry Inc.

Emtree Heading

antibody specificity; antigen binding; cardiovascular disease; clinical laboratory; diabetes mellitus; editorial; *enzyme linked immunosorbent assay; genotype; hemolysis; human; Human immunodeficiency virus infection; malaria; outcomes research; phenotype; risk factor; tuberculosis; *haptoglobin.

Link to the Ovid Full Text or citation:

Click here for full text options

280.

Benefit/risk of therapies for rheumatoid arthritis: Underestimation of the "side effects" or risks of RA leads to underestimation of the benefit/risk of therapies.

Pincus T., Kavanaugh A., Sokka T.

Clinical and Experimental Rheumatology. 22 (5 SUPPL. 35) (pp S2-S11), 2004. Date of Publication: 2004.

AN: 2004428782

Most physicians are familiar with the side effects or risks of drugs used to treat rheumatoid arthritis (RA), but relatively less familiar with the "side effects" or risks associated with RA itself. RA is not thought to have the same potential severity as a cardiovascular or neoplastic disease by most physicians, the public, or even some rheumatologists, although relative rates of predicted mortality in some patients with RA are in the range of some people with coronary artery disease or Hodgkin's disease. Many reasons may be identified to explain why the risks of RA have been underestimated: RA does not lead to acute life-threatening situations; population-based data have suggested that most people who meet criteria for RA have a mild or self-limited process; acute attributed causes of death in people with RA are superficially similar to those in the general population; clinical trials have suggested many therapies that are efficacious over a period of 3-12 months; few long-term longitudinal studies were performed prior to the 1980s; medical recommendations made during the 1950s-1980s suggested that simple therapies were adequate for most patients; and quantitative information concerning patient status is generally not included in standard rheumatology care. As more information has emerged concerning severe long-term outcomes in the "natural history" of RA (as treated prior to the 1990s, new strategies of aggressive intervention have been developed. Furthermore, basic research has led to new therapies. It appears that the benefit/risk ratio of therapies for RA has increased substantially over the last two decades, and the outlook for patients with RA is much better at this time than in previous years. © Copyright Clinical and Experimental Rheumatology 2004.

Institution

(Pincus, Sokka) Division of Rheumatology/Immunology, Department of Medicine, Vanderbilt University Medical Center, 203 Oxford House, Nashville, TN 37232-4500, United States (Kavanaugh) The Center for Innovative Therapy, Div. Rheumatol. Allergy/Immunology, Univ. California San Diego Sch. Med., La Jolla, CA, United States

(Sokka) Jyvaskyla Central Hospital, Jyvaskyla, Finland

Publisher

Clinical and Experimental Rheumatology S.A.S.

Emtree Heading

bladder disease/si [Side Effect]; cardiovascular disease; cause of death; central nervous system disease/si [Side Effect]; clinical trial; diabetes mellitus/si [Side Effect]; disease course; disease severity; drug efficacy; drug research; drug use; gastrointestinal disease/si [Side Effect]; hematologic disease/si [Side Effect]; Hodgkin disease; human; hypertension/si [Side Effect]; ischemic heart disease; kidney disease/si [Side Effect]; liver disease/si [Side Effect]; longitudinal study; lymphoma/si [Side Effect]; mortality; neoplasm; osteopenia/si [Side Effect]; population research; prediction; priority journal; quantitative analysis; retinopathy/si [Side Effect]; review; *rheumatoid arthritis/dt [Drug Therapy]; risk assessment; *risk benefit analysis; teratogenicity; treatment outcome; tuberculosis/si [Side Effect]; adalimumab/ae [Adverse Drug Reaction]; adalimumab/cm [Drug Comparison]; adalimumab/dt [Drug Therapy]; *antirheumatic agent/ae [Adverse Drug Reaction]; *antirheumatic agent/ct [Clinical Trial]; *antirheumatic agent/cb [Drug Combination]; *antirheumatic agent/cm [Drug Comparison]; *antirheumatic agent/dt [Drug Therapy]; *antirheumatic agent/to [Drug Toxicity]; azathioprine/ae [Adverse Drug Reaction]; azathioprine/dt [Drug Therapy]; corticosteroid/ae [Adverse Drug Reaction]; corticosteroid/ct [Clinical Trial]; corticosteroid/cb [Drug Combination]; corticosteroid/cm [Drug Comparison]; corticosteroid/dt [Drug Therapy]; cyclophosphamide/ae [Adverse Drug Reaction]; cyclophosphamide/dt [Drug Therapy]; cyclosporin A/ae [Adverse Drug Reaction]; cyclosporin A/dt [Drug Therapy]; etanercept/ae [Adverse Drug Reaction]; etanercept/cm [Drug Comparison]; etanercept/dt [Drug Therapy]; gold salt/ae [Adverse Drug Reaction]; gold salt/cm [Drug Comparison]; gold salt/dt [Drug Therapy]; hydroxychloroquine/ae [Adverse Drug Reaction]; hydroxychloroquine/ct [Clinical Trial]; hydroxychloroquine/cb [Drug Combination]; hydroxychloroquine/cm [Drug Comparison]; hydroxychloroquine/dt [Drug Therapy]; infliximab/ae [Adverse Drug Reaction]; infliximab/cm [Drug Comparison]; infliximab/dt [Drug Therapy]; leflunomide/ae [Adverse Drug Reaction]; leflunomide/cm [Drug Comparison]; leflunomide/dt [Drug Therapy]; leflunomide/to [Drug Toxicity]; methotrexate/ae [Adverse Drug Reaction]; methotrexate/ct [Clinical Trial]; methotrexate/cb [Drug Combination]; methotrexate/cm [Drug Comparison]; methotrexate/dt [Drug Therapy]; nonsteroid antiinflammatory agent/ae [Adverse Drug Reaction]; nonsteroid antiinflammatory agent/dt [Drug Therapy]; penicillamine/ae [Adverse Drug Reaction]; penicillamine/cm [Drug Comparison]; penicillamine/dt [Drug Therapy]; prednisolone/ct [Clinical Trial]; prednisolone/cb [Drug Combination]; prednisolone/cm [Drug Comparison]; prednisolone/dt [Drug Therapy]; prednisone/ct [Clinical Trial]; prednisone/cb [Drug Combination]; prednisone/cm [Drug Comparison]; prednisone/dt [Drug Therapy]; recombinant interleukin 1 receptor blocking agent/ae [Adverse Drug Reaction]; recombinant interleukin 1 receptor blocking agent/cm [Drug Comparison]; recombinant interleukin 1 receptor blocking agent/dt [Drug Therapy]; salazosulfapyridine/ct [Clinical Trial]; salazosulfapyridine/cb [Drug Combination]; salazosulfapyridine/cm [Drug Comparison]; salazosulfapyridine/dt [Drug Therapy].

Link to the Ovid Full Text or citation:

Click here for full text options

281.

Single-center experience with cyclosporine therapy for kidney transplantation: Analysis of a twenty-year period in 1200 patients.

Snanoudj R., Kriaa F., Arzouk N., Beaudreuil S., Hiesse C., Durrbach A., Charpentier B.

Transplantation Proceedings. 36 (2 SUPPL.) (pp 83S-88S), 2004. Date of Publication: March 2004.

AN: 2004133099

Since the introduction of cyclosporine (CyA) in our center in February 1983, 1267 kidney transplant patients have received an immunosuppressive regimen based on CyA, usually in association with azathioprine and steroids and following an induction therapy in three quarters of patients. The aim of this study was to retrospectively analyze our 20-year experience with CyA and examine the evolution of therapy during this period. Induction treatment has been less commonly used during the past 5 years. Even in the early years of our experience, CyA doses were low (under 6 mg/kg per day at 3 months after transplantation). Acute tubular necrosis was observed in 39.4% of patients. The incidence of acute rejection episodes has dramatically decreased since 1984, but the frequency of steroid-resistant rejection has remained constant (around 20%). The first year of transplantation, 32.7% of patients had arterial hypertension. De novo diabetes mellitus occurred in 2.5% of patients. An incidence of 11.8% of malignancies was observed. Skin cancer and lymphomas accounted for 50% and 12% of neoplasms. Five-year graft and patient survivals were 70% and 87%, respectively. Renal function remained remarkably constant during the first 10 years of follow-up with a mean creatinine of 150 mumol/L. Chronic allograft nephropathy resulted in 43% graft losses. In conclusion, CyA has been well tolerated in our patients. However, the occurrence of chronic allograft nephropathy was a major concern in our cohort.

Institution

(Snanoudj, Kriaa, Arzouk, Beaudreuil, Hiesse, Durrbach, Charpentier) Nephrology and Transplant. Dept., Kremlin-Bicetre Hospital, Le Kremlin-Bicetre, France (Charpentier) Hopital du Kremlin Bicetre, Service de Nephrologie, 78 avenue du general Leclerc, 94275 Le Kremlin Bicetre, France

Publisher

Elsevier USA

Emtree Heading

acute kidney tubule necrosis/co [Complication]; acute pyelonephritis/co [Complication]; adult; aged; article; bacterial infection/co [Complication]; bacterial infection/dt [Drug Therapy]; basal cell carcinoma/co [Complication]; chronic allograft nephropathy/co [Complication]; controlled study; creatinine blood level; cystitis/co [Complication]; death; diabetes mellitus/co [Complication]; diabetes mellitus/dt [Drug Therapy]; dose response; drug tolerability; female; follow up; graft rejection/dt [Drug Therapy]; graft rejection/pc [Prevention]; graft survival; human; hypertension/co [Complication]; hypertrichosis/si [Side Effect]; immunoadsorption; immunosuppressive treatment; incidence; Kaposi sarcoma/co [Complication]; kidney disease/co [Complication]; *kidney transplantation; lymphoma/co [Complication]; lymphoproliferative disease/si [Side Effect]; major clinical study; male; nephrotoxicity/si [Side Effect]; plasmapheresis; priority journal; prostatitis/co [Complication]; retrospective study; side effect/si [Side Effect]; skin cancer/co [Complication]; squamous cell carcinoma/co [Complication]; treatment outcome; tremor/si [Side Effect]; tuberculosis/co [Complication]; urinary tract infection/co [Complication]; azathioprine/cb [Drug Combination]; azathioprine/dt [Drug Therapy]; basiliximab/cb [Drug Combination]; basiliximab/dt [Drug Therapy]; creatinine/ec [Endogenous Compound]; *cyclosporin A/ae [Adverse Drug Reaction]; *cyclosporin A/cb [Drug Combination]; *cyclosporin A/do [Drug Dose]; *cyclosporin A/dt [Drug Therapy]; *cyclosporin A/pd [Pharmacology]; ganciclovir/dt [Drug Therapy]; immunosuppressive agent/cb [Drug Combination]; immunosuppressive agent/dt [Drug Therapy]; insulin/dt [Drug Therapy]; methylprednisolone/cb [Drug Combination]; methylprednisolone/dt [Drug Therapy]; OKT 3/cb [Drug Combination]; OKT 3/dt [Drug Therapy]; prednisolone/cb [Drug Combination]; prednisolone/dt [Drug Therapy]; prednisolone/po [Oral Drug Administration]; steroid/cb [Drug Combination]; steroid/do [Drug Dose]; steroid/dt [Drug Therapy]; steroid/iv [Intravenous Drug Administration]; tacrolimus/dt [Drug Therapy]; thymocyte antibody/cb [Drug Combination]; thymocyte antibody/dt [Drug Therapy]; tumor necrosis factor antibody/cb [Drug Combination]; tumor necrosis factor antibody/dt [Drug Therapy]; unclassified drug.

Drug Trade Names and Manufacturers

neoral, sandimmune

Link to the Ovid Full Text or citation:

Click here for full text options

282.

Predictors of treatment failure among tuberculosis patients under DOTS strategy in Egypt.

Morsy A.M., Zaher H.H., Hassan M.H., Shouman A.

Eastern Mediterranean Health Journal. 9 (4) (pp 689-701), 2003. Date of Publication: July 2003.

AN: 2005160699

Treatment failure is a serious problem facing some national tuberculosis (TB) control programmes. Irregularity of treatment is a factor that can lead to treatment failure. A case-control study was carried out in TB centres in Egypt during April 2001 -December 2002 aimed at investigating the predictors of treatment failure. We interviewed 119 people with treatment failure and an equal number of cured cases (controls) and their families regarding sociodemographic characteristics, information about TB, information about drugs, treatment compliance, family support and patient-family interaction. Significant risk factors for treatment failure were non-compliance to treatment, deficient health education to the patient, poor patient knowledge regarding the disease and diabetes mellitus as co-morbid condition.

Institution

(Morsy, Zaher) Natl. Tuberculosis Control Programme, Ministry of Health and Population, Cairo, Egypt (Hassan) Public Health Department, Faculty of Medicine, University of Cairo, Cairo, Egypt

(Shouman) Public Health Department, Faculty of Medicine, Ain Shams University, Cairo, Egypt

Publisher

World Health Organization

Emtree Heading

adult; aged; article; case control study; comorbidity; controlled study; demography; diabetes mellitus; drug information; Egypt; family; female; human; interview; *lung tuberculosis/dt [Drug Therapy]; major clinical study; male; medical information; patient compliance; patient education; prediction; risk factor; *short course therapy; socioeconomics; treatment failure; treatment outcome; tuberculosis control; *tuberculostatic agent/dt [Drug Therapy].

Link to the Ovid Full Text or citation:

Click here for full text options

283.

Adverse events of standardized regimens of corticosteroids for prophylaxis and treatment of nerve function impairment in leprosy: Results from the 'TRIPOD' trials.

Richardus J.H., Withington S.G., Anderson A.M., Croft R.P., Nicholls P.G., Van Brakel W.H., Smith W.C.S.

Leprosy Review. 74 (4) (pp 319-327), 2003. Date of Publication: December 2003.

AN: 2004014112

Reactions in leprosy causing nerve function impairment (NFI) are increasingly treated with standardized regimens of corticosteroids, often under field conditions. Safety concerns led to an assessment of adverse events of corticosteroids, based on data of three trials studying prevention of NFI (the TRIPOD study). A multicentre, randomized, double-blind placebo-controlled trial was conducted in leprosy control programmes in Nepal and Bangladesh. Treatment was with prednisolone according to fixed schedules for 16 weeks, starting in one trial with 20 mg/day (prophylactic regimen: total dosage 1.96 g) and in the other two trials with 40 mg/day (therapeutic regimen: total dosage 2.52 g). Minor adverse events were defined as moon face, fungal infections, acne, and gastric pain requiring antacid. Major adverse events were defined as psychosis, peptic ulcer, glaucoma, cataract, diabetes and hypertension. Also, the occurrence of infected plantar, palmar, and corneal ulceration was monitored, together with occurrence of TB. Considering all three trials together, minor adverse events were observed in 130/815 patients (16%). Of these, 51/414 (12%) were in the placebo group and 79/401 (20%) in the prednisolone group. The relative risk for minor adverse events in the prednisolone group was 1.6 (P= 0.004). Adverse events with a significantly increased risk were acne, fungal infections and gastric pain. Major adverse events were observed in 15/815 patients (2%); 7/414 (2%) in the placebo group and 8/401 (2%) in the prednisolone group. No major adverse events had a significantly increased risk in the prednisolone arm of the trials. No cases of TB were observed in 300 patients who could be followed-up for 24 months. Standardized regimens of corticosteroids for both prophylaxis and treatment of reactions and NFI in leprosy under field conditions in developing countries are safe when a standard pre-treatment examination is performed, treatment for minor conditions can be carried out by field staff, referral for specialized medical care is possible, and sufficient follow-up is done during and after treatment.

Institution

(Richardus) Department of Public Health, Erasmus MC, University Medical Center Rotterdam, P.O. Box 1738, 3000 DR Rotterdam, Netherlands (Withington) Leprosy Mission Bangladesh, House 17A, Banani (Old) DOHS, Road 3, Dhaka 1206, Bangladesh

(Anderson) Intl. Nepal Fellowship-RELEASE, P.O. BOX 28, Pokhara, Nepal

(Croft) 56a St Peter's Road, Early, Reading RG6 1PH, United Kingdom

(Nicholls, Smith) Department of Public Health, University of Aberdeen, Polwarth Building, Foresterhill, Aberdeen, AB25 2ZD, United Kingdom

(Van Brakel) KIT Leprosy Unit, Wibautstraat 137 J, 1097 DN Amsterdam, Netherlands

Publisher

British Leprosy Relief Association

Emtree Heading

acne/si [Side Effect]; adolescent; adult; article; Bangladesh; cataract/si [Side Effect]; clinical trial; controlled clinical trial; controlled study; cornea ulcer/si [Side Effect]; corticosteroid therapy; diabetes mellitus/si [Side Effect]; disability; double blind procedure; glaucoma/si [Side Effect]; human; hypertension/si [Side Effect]; infection/co [Complication]; *leprosy/di [Diagnosis]; major clinical study; moon face/si [Side Effect]; multicenter study; mycosis/co [Complication]; Nepal; *nerve function impairment/co [Complication]; *nerve function impairment/dt [Drug Therapy]; *nerve function impairment/pc [Prevention]; *neuropathy/co [Complication]; *neuropathy/dt [Drug Therapy]; *neuropathy/pc [Prevention]; peptic ulcer/si [Side Effect]; psychosis/si [Side Effect]; randomized controlled trial; risk assessment; side effect/si [Side Effect]; stomach pain/si [Side Effect]; treatment outcome; tuberculosis/co [Complication]; *corticosteroid/ae [Adverse Drug Reaction]; *corticosteroid/ct [Clinical Trial]; *corticosteroid/do [Drug Dose]; *corticosteroid/dt [Drug Therapy]; placebo; *prednisolone/ae [Adverse Drug Reaction]; *prednisolone/ct [Clinical Trial]; *prednisolone/do [Drug Dose]; *prednisolone/dt [Drug Therapy].

Link to the Ovid Full Text or citation:

Click here for full text options

284.

Adherence to long-term therapies: Evidence for action.

De Geest S., Sabate E.

European Journal of Cardiovascular Nursing. 2 (4) (pp 323), 2003. Date of Publication: December 2003.

AN: 2003506561

Institution

(De Geest) Institute of Nursing Science, University of Basel, Bernoullistrasse 28, CH-4056 Basel, Switzerland (Sabate) WHO/OMS - NMH 4059, 20, Avenue Appia, CH 1211 Geneva 27, Switzerland

Publisher

Elsevier

Emtree Heading

asthma; cancer; cardiovascular disease; diabetes mellitus; health care; human; Human immunodeficiency virus; hypertension; letter; literature; *long term care; nurse; priority journal; research; risk factor; smoking; treatment outcome; tuberculosis; world health organization.

Link to the Ovid Full Text or citation:

Click here for full text options

285.

Problems about the management of active pulmonary tuberculosis patients undergoing haemodialysis - Our experiences and nation-wide questionnaire survey.

Tomioka H., Tada K., Ohyama A., Fujiyama R., Ohnishi H., Sakurai T., Sakamoto H., Nishiguchi H., Yamamoto T., Sakashita T., Shirai C., Iwasaki H.

Kekkaku. 78 (4) (pp 353-358), 2003. Date of Publication: 01 Apr 2003.

AN: 2003165766

End-stage renal failure patients on chronic dialysis are high risk groups of tuberculosis due to attenuated cellular immunity. Patients receiving haemodialysis stay prolonged time inside the health-care facilities, thereby increased risk of tuberculosis transmission if a patient has active disease. So management of active pulmonary tuberculosis undergoing haemodialysis is important, however, the number of hospitals which are capable of taking care of such patients is estimated to be few in Japan. Methods: From August 1994 through July 2002, 1059 active pulmonary tuberculosis patients (mean age; 57 +/- 19, male/female=773/286) were admitted to Nishi-Kobe Medical Center, a 500-bed teaching hospital. Out of them, patients undergoing haemodialysis were retrospectively studied to describe the clinical characteristics of such cases. Then we conducted a questionnaire survey regarding the management of active pulmonary tuberculosis patients undergoing haemodialysis for 86 self-governing bodies in Japan. Results: (1) Clinical characteristics of active pulmonary tuberculosis undergoing haemodialysis. We encountered 14 cases (mean age; 65+/-11, male/female= 7/7) of pulmonary tuberculosis undergoing haemodialysis during 8 years. In addition to pulmonary involvement, 3 pleural, one knee joint and one lymph node involvement was detected. Primary renal disease included diabetic nephropathy (n=3), chronic glomerulonephritis (n=3), congenital anomaly (n=1), and unknown (n=7). Nine cases were referred to our hospital from health-care facilities located out of city or prefecture. In five cases it took more than three months from the onset or detection of abnormal chest X-ray findings to the admission to our hospital. Five cases developed pulmonary tuberculosis within the first year after the initiation of dialysis. None of the patients had a past history of tuberculosis. Cavitary lesion on chest X-ray was observed in only one case. Triple antituberculosis therapy was used in 9 patients, and 4 antituberculosis drugs were used in 5 patients. Antituberculosis therapy was successfully done in all cases except two patients who died of apoplexy and cerebral infarction. (2) The nation-wide questionnaire survey. Of the 86 self-governing bodies we mailed, 66 self-govern ing bodies replied. Of them, 31% reported that they have experienced difficulties in the management of active pulmonary tuberculosis patients undergoing haemodialysis, and 25% reported the lack of health-care facilities to take care of such cases in their territory. They have referred such patients to hospitals located in the nearby prefectures or they have recommended antituberculosis therapy visiting a local haemodialysis facility. Conclusion: There are sometimes difficulties to manage active pulmonary tuberculosis patients undergoing haemodialysis in Japan. Health-care facilities to take care of such patients should be arranged and the formation of the network is necessarily.

Institution

(Tomioka, Tada, Fujiyama, Ohnishi, Sakurai, Sakamoto, Iwasaki) Department of Respiratory Medicine, Nishi-Kobe Medical Center, 5-7-1, Koujidai, Nishi-ku, Kobe-shi, Hyogo 651-2273, Japan (Ohyama) Department of Nephrology, Nishi-Kobe Medical Center, Hyogo, Japan

(Nishiguchi, Yamamoto, Sakashita) Clinical Laboratory, Nishi-Kobe Medical Center, Hyogo, Japan

Publisher

Japanese Society for Tuberculosis

Emtree Heading

adult; aged; antibiotic therapy; article; bacterial transmission; cause of death; cerebrovascular accident; chronic glomerulonephritis; congenital malformation/cn [Congenital Disorder]; controlled study; diabetic nephropathy; disease activity; female; health care facility; health survey; *hemodialysis; high risk patient; human; infection risk; kidney failure/th [Therapy]; *lung tuberculosis/dt [Drug Therapy]; lymph node; major clinical study; male; questionnaire; thorax radiography; treatment outcome; *tuberculostatic agent/dt [Drug Therapy].

Link to the Ovid Full Text or citation:

Click here for full text options

286.

Evaluation of tuberculosis treatment results in Italy, report 1999.

Centis R., Migliori G.B.

Monaldi Archives for Chest Disease - Pulmonary Series. 57 (5-6) (pp 297-305), 2002. Date of Publication: October/December 2002.

AN: 2003219421

In Italy no national data on tuberculosis (TB) treatment results was available. In 1995 the A.I.P.O. (Italian Association of Hospital Pneumologists) TB Study Group, in collaboration with the Istituto Superiore di Sanita (technical branch of the Ministry of Health) started a prospective monitoring activity based on the World Health Organisation and the International Union Against Tuberculosis and Lung Disease recommendations. Data was collected from a representative network of TB units nationwide, managing a significant proportion of all TB cases notified in Italy each year. Aim of this study was to analyse the casefinding and treatment results for 1999. 906 TB cases were reported (61.2% males; 36.2% immigrants), 714 (78.8%) being new cases. Of these cases 639 (70.5%) were pulmonary, 221 (24.4%) extrapulmonary cases and 46 (5.1%) both pulmonary and extrapulmonary. The main risk-factors for TB were history of recent contact, diabetes and alcohol abuse among native Italians, the human immunodeficiency virus-sieropositive status and history of recent contact among immigrants. The majority of immigrants came from Africa and Asia, and had stayed in Italy for more than 24 months before diagnosis of TB. Thirty-five per cent (320/906) of pulmonary patients had a positive direct sputum smear examination for alcohol acid-fast bacilli; 22.5% were resistant to any drug (from combined monoresistance to rifampicin: 1.6%; combined multi-drug resistance: 4.8%). In 97% of cases the duration of treatment was <12 months. The overall success rate (cured plus treatment completed excluding transferred out from the denominator) was 86%. A significantly higher percentage of deaths were recorded amongst native Italians (being age-related; p<0.001) while immigrants had a higher default rate (p<0.001).

Institution

(Migliori) WHO Collab. Ctr. for Tb./Lung Dis., Dept. of Pneumology, Fondazione S. Maugeri, Via Roncaccio 16, 21049 Tradate (VA), Italy

Publisher

PI-ME Tipografia Editrice S.r.l.

Emtree Heading

acid fast bacterium; adolescent; adult; Africa; alcohol abuse; article; Asia; data analysis; data base; death; diabetes mellitus; disease severity; evaluation; extrapulmonary tuberculosis/dr [Drug Resistance]; extrapulmonary tuberculosis/dt [Drug Therapy]; extrapulmonary tuberculosis/ep [Epidemiology]; female; human; Human immunodeficiency virus; immigrant; Italy; lung tuberculosis/dr [Drug Resistance]; lung tuberculosis/dt [Drug Therapy]; lung tuberculosis/ep [Epidemiology]; major clinical study; male; multidrug resistance; risk factor; side effect/si [Side Effect]; sputum smear; treatment failure; treatment outcome; *tuberculosis/dr [Drug Resistance]; *tuberculosis/dt [Drug Therapy]; *tuberculosis/ep [Epidemiology]; virus transmission; isoniazid/dt [Drug Therapy]; rifampicin/cb [Drug Combination]; rifampicin/dt [Drug Therapy]; streptomycin/dt [Drug Therapy]; *tuberculostatic agent/ae [Adverse Drug Reaction]; *tuberculostatic agent/cb [Drug Combination]; *tuberculostatic agent/dt [Drug Therapy].

Link to the Ovid Full Text or citation:

Click here for full text options

287.

Treatment for tuberculosis in difficult situations.

Rikimaru T., Kawashiro T.

Kekkaku. 77 (11) (pp 759-761), 2002. Date of Publication: 01 Nov 2002.

AN: 2002444020

Many patients with tuberculosis have complications and concern with underlying diseases. Those statuses make us difficult to treat the patients with tuberculosis, We presented the therapy of senile and infant tuberculosis and tuberculosis of patients with hemodialysis, diabetes mellitus, liver dysfunction, pregnancy, and puerperium in this symposium. We discussed about further issues with audiences. Symposium Topics and Presenters: 1. Treatment for senile tuberculosis and tuberculosis associated with hemodialysis: Toshinobu Yokoyama (Department of Internal Medicine, Kurume University School of Medicine) 2. Treatment for tuberculosis associated with diabetes mellitus: Fumio Yamagishi (Department of Respiratory, National Chiba-Higashi Hospital) 3. Clinical investigation of patients with pulmonary tuberculosis associated with liver disorder: Tetsuya Shiomi (Department of Internal Medicine, National Higashi-Saitama Hospital) 4. Treatment for tuberculosis associated with pregnancy and puerperium: Emiko Toyota1, Shigeki Minoura2, Hirofumi Miyazawa3 (Department of 1Respiratory, 2Gynecology, 3Pediatrics, International Medical Center) 5. Treating infants, especially 12 month-old or less, for tuberculosis: Shinya Kondo, Masaki Itoh (Division of Respiratory Disease, Tokyo Metropolitan Children's Hospital) Main issue of this symposium was treatment for tuberculosis in difficult situations. At first, we decided what were difficult situations. There were so many personal and social problems to treat patients for tuberculosis. We focused personal and popular issues, such as senile and infantile tuberculosis and tuberculosis associated with hemodialysis, diabetes mellitus, liver disorder, pregnancy and puerperium. Dr. Yokoyama reported as for tuberculous patients with dialysis and elder patients with pulmonary tuberculosis. He described that those patients were treated successfully although both states were immunosuppressive. Treatment for patients with hemodialysis was problem in care and management, not in therapeutic. Dr. Yamagishi strongly described that patients with diabetes mellitus were one of the high risk groups of developing pulmonary tuberculosis and they had to undergo chest X-ray examination periodically. He revealed that many physicians treating for diabetes mellitus had little concern with tuberculosis, thus the retraining of physicians dealing with diabetics on tuberculosis is considered to be necessary. We discussed with the duration of treating patients for tuberculosis associated with diabetes mellitus. Thus far, the duration was controversial. Dr. Shiomi presented about clinical investigation of pulmonary tuberculosis with liver disorder. He described that patients with liver disorder needed to discontinue the use of the medicine. Duration of smear positive in the patients, however, had no significant difference compared with patients without liver disorder. Dr. Toyota reported 22 cases with tuberculosis associated with pregnancy and puerperium. 10 cases out of all had extra pulmonary lesions and 9 cases were foreign women. It was difficult to diagnose the patient's illness as tuberculosis because pregnancy and puerperium hid symptoms of tuberculosis. A long time was needed to diagnose the pregnant patients as tuberculosis. WHO and BTS recommended standard four drugs regimen: INH, RFP, EB, and PZA, for these patients. On the other hand, ATS suggested three drugs regimen without PZA due to less data of adverse reaction of PZA in patients with pregnancy and puerperium. All guidelines recommended that SM was not suitable for patients with pregnancy and puerperium because of the adverse reaction as hearing loss. Dr. Kondo revealed that the number of tuberculosis infants less than one year were considered to be risky against tuberculosis. One of major reasons of difficulties in treatments is the rapid progress of the disease because of underdevelopment of cell-mediated immunity among them. Anatomical underdevelopment of cranial arteries and narrow cerebro-spinal passages easily cause cerebral infarction and hydrocephalus following meningeal inflammation of tuberculosis. A belated diagnosis also makes the treatment difficult in some infants whose tuberculin skin test shows false negative and radiographic manifestation of chest is unwarranted. During the treatment, systemic and enteric viral infections occur frequently among infants with tuberculosis, and liver functional disorders caused by these infections sometimes prevent the treatment. It is important to treat tuberculosis as soon as possible and to recognize both susceptibilities to tuberculosis and difficulties in diagnosis in some infants less than one year. We appreciate all of participants in this symposium.

Institution

(Rikimaru) Internal Medicine, Kurume University School of Medicine, 67 Asahi-machi, Kurume-shi, Fukuoka 830-0011, Japan

Publisher

Japanese Society for Tuberculosis

Emtree Heading

conference paper; diabetes mellitus; disease duration; hearing loss/si [Side Effect]; hemodialysis; human; infection control; infection risk; *lung tuberculosis/di [Diagnosis]; *lung tuberculosis/dt [Drug Therapy]; *lung tuberculosis/th [Therapy]; pregnancy; thorax radiography; treatment indication; treatment outcome; world health organization; ethambutol/dt [Drug Therapy]; isoniazid/dt [Drug Therapy]; pyrazinamide/dt [Drug Therapy]; rifampicin/dt [Drug Therapy]; streptomycin/ae [Adverse Drug Reaction]; streptomycin/dt [Drug Therapy].

Link to the Ovid Full Text or citation:

Click here for full text options

288.

Experience of surgical treatment of the main diseases of thyroid gland.

Shakhsuvarov O.M., Mirsalimov F.M., Huseynov R.G., Saidova F.Ch., Muradov N.F., Zamanov R.M.

Azerbaijan Medical Journal. (1) (pp 81-84), 2002. Date of Publication: 2002.

AN: 2002197916

The experience of operative treatment of 16000 patients with different thyroid pathology is summarised in the given article. The very large group consisted of patients with endemic goiter - 10916 patients, most of them had nodal and mixed form (94%) with prevalence of degree III-IV gland enlargement (80%), and degree V - 20% of patients. The patients underwent different interventions, such as enucleation with resection of the opposite lobe, and different kinds of resections. Excision of the gland isthmus was considered to be necessary. During postoperative period patients were administered iodine preparations for a period 3-6 months. After operation 17 (0,16%) of patients died of pulmonary artery thrombosis, insult, acute cardiopulmonary failure, and etc. Tracheotomy was performed in 11 and thyrotoxicosis in 4569 patients. A special attention was given to patients with concomitant diseases, such as hypertension, diabetes, bronchial asthma, pulmonary tuberculosis, anaemia, etc. Preparation of such patients with high risk of anaesthesia in operation should be carried out more carefully and directed at maximal control of manifestations of concomitant diseases. All the patients underwent subtotal gland resection. Good and satisfactory results were obtained in 84% of patients. The following methods of diagnosis were used: USI, aspiration and intraoperative biopsy, roentgenotomography of trachea and oesophagus, radioisotope, radionuclide investigations and computer tomography. Complex investigation, elaborated in the clinic with use of immunologic, radionuclide methods, USI, thickneedle biopsy and others allowed to make a correct preoperative diagnosis in 85% of patients.

Institution

(Shakhsuvarov, Mirsalimov, Huseynov, Saidova, Muradov, Zamanov) Azerbaijan SRI of Clinical Medicine, Department of Endocrine Surgery, Baku, Azerbaijan

Publisher

WHO Office in Azerbaijan

Emtree Heading

anemia; anesthesia; artery thrombosis/co [Complication]; article; aspiration biopsy; asthma; biopsy; comorbidity; computer assisted tomography; controlled study; diabetes mellitus; enucleation; goiter/su [Surgery]; heart failure/co [Complication]; high risk patient; human; hypertension; lung artery; lung insufficiency/co [Complication]; lung tuberculosis; major clinical study; pathogenesis; postoperative period; *thyroid disease/di [Diagnosis]; *thyroid disease/su [Surgery]; thyroid surgery; thyrotoxicosis; tomography; tracheotomy; treatment outcome; iodine; radioisotope.

Link to the Ovid Full Text or citation:

Click here for full text options

289.

Why evidence-based medicine?.

Jamrozik K.

Bailliere's Best Practice and Research in Clinical Anaesthesiology. 15 (4) (pp 505-518), 2001. Date of Publication: 2001.

AN: 2002066451

This chapter considers briefly what counts as 'evidence' and describes three historical strands, in medical science generally, in epidemiology and in clinical trials, that have converged to give rise to the concept of evidence-based medicine. It then returns to the randomized controlled trial as the fundamental building block of evidence-based practice. Finally, through considering whether all trials are equally useful, it demonstrates that discerning what is the best way to proceed in the clinical management of individual patients and problems is not necessarily a simple matter.

Institution

(Jamrozik) Department of Primary Health Care and General Practice, Imperial College of Science, Technology and Medicine, Reynolds Building, St Dunstan's Road, London, W6 8RP, United Kingdom

Publisher

Bailliere Tindall Ltd

Emtree Heading

ascites/dt [Drug Therapy]; cardiovascular disease/dt [Drug Therapy]; clinical trial; controlled clinical trial; controlled study; deep vein thrombosis/dt [Drug Therapy]; deep vein thrombosis/pc [Prevention]; drug efficacy; drug indication; epidemic; epidemiological data; *evidence based medicine; fever/dt [Drug Therapy]; health care quality; health promotion; history of medicine; human; insulin dependent diabetes mellitus/dt [Drug Therapy]; lung embolism/dt [Drug Therapy]; lung embolism/pc [Prevention]; lung tuberculosis/di [Diagnosis]; lung tuberculosis/dt [Drug Therapy]; medical decision making; medical research; medicine; meta analysis; outcomes research; patient care; pharyngitis/dt [Drug Therapy]; priority journal; public health; publication; randomized controlled trial; review; risk factor; screening test; scurvy/dt [Drug Therapy]; scurvy/pc [Prevention]; statistical analysis; stroke; syphilis/dt [Drug Therapy]; theory; thorax radiography; validation process; acetylsalicylic acid/ct [Clinical Trial]; acetylsalicylic acid/dt [Drug Therapy]; acetylsalicylic acid/pd [Pharmacology]; arsphenamine/dt [Drug Therapy]; citrus fruit extract/dt [Drug Therapy]; digitalis/dt [Drug Therapy]; hydroxymethylglutaryl coenzyme A reductase inhibitor/ct [Clinical Trial]; hydroxymethylglutaryl coenzyme A reductase inhibitor/dt [Drug Therapy]; hydroxymethylglutaryl coenzyme A reductase inhibitor/pd [Pharmacology]; insulin/ct [Clinical Trial]; insulin/dt [Drug Therapy]; insulin/pd [Pharmacology]; penicillin G/ct [Clinical Trial]; penicillin G/dt [Drug Therapy]; penicillin G/pd [Pharmacology]; streptomycin/ct [Clinical Trial]; streptomycin/dt [Drug Therapy]; streptomycin/pd [Pharmacology].

Link to the Ovid Full Text or citation:

Click here for full text options

290.

Increased incidence of multidrug-resistant tuberculosis in diabetic patients on the bellevue chest service, 1987 to 1997.

Bashar M., Alcabes P., Rom W.N., Condos R.

Chest. 120 (5) (pp 1514-1519), 2001. Date of Publication: 2001.

AN: 2001410574

Study objectives: To investigate the characteristics of tuberculosis infection in diabetic patients at Bellevue Hospital. Design: We conducted a case-control study retrospectively reviewing the records of patients at Bellevue Hospital Center from 1987 to 1997 with a discharge diagnosis of tuberculosis and diabetes mellitus. Setting: Bellevue Hospital Center is a 1,200-bed, inner-city municipal hospital located in the Lower East Side of New York City. Patients: Fifty-three identified patients had verified tuberculosis infection and diabetes; of these, 50 charts were available for review. One hundred five control cases were selected from nondiabetic patients with a discharge diagnosis of tuberculosis during the same time period. Measurements and results: Thirty-six percent (18 cases) of the patients with diabetes and tuberculosis had multidrug-resistant tuberculosis (MDR-TB) compared to only 10% (10 cases) in the control group (p < 0.01) Controlling for homelessness, HIV status, and directly observed therapy, the relative risk of MDR-TB was calculated to be 8.6 (confidence interval, 3.1 to 23.6) in the diabetic group compared to the control group. Conclusions: There was a significant association between diabetes and MDR-TB. Diabetes continues to be a risk factor for tuberculosis and was associated with MDR-TB in our patients.

Institution

(Bashar, Alcabes, Rom, Condos) Division of Pulmonary Medicine, Department of Medicine, New York Univ. School of Medicine, 550 First Ave, New York, NY 10016, United States

Publisher

American College of Chest Physicians

Emtree Heading

adolescent; adult; aged; article; calculation; child; controlled study; *diabetes mellitus/di [Diagnosis]; *diabetes mellitus/dt [Drug Therapy]; disease association; female; homelessness; human; Human immunodeficiency virus infection; infection risk; major clinical study; male; *multidrug resistance; priority journal; retrospective study; risk factor; short course therapy; treatment outcome; *tuberculosis/di [Diagnosis]; *tuberculosis/dr [Drug Resistance]; *tuberculosis/dt [Drug Therapy]; United States; ciprofloxacin/cb [Drug Combination]; ciprofloxacin/dt [Drug Therapy]; ciprofloxacin/pd [Pharmacology]; ethambutol/cb [Drug Combination]; ethambutol/dt [Drug Therapy]; ethambutol/pd [Pharmacology]; ethionamide/cb [Drug Combination]; ethionamide/dt [Drug Therapy]; ethionamide/pd [Pharmacology]; isoniazid/cb [Drug Combination]; isoniazid/dt [Drug Therapy]; isoniazid/pd [Pharmacology]; pyrazinamide/cb [Drug Combination]; pyrazinamide/dt [Drug Therapy]; pyrazinamide/pd [Pharmacology]; rifabutin/cb [Drug Combination]; rifabutin/dt [Drug Therapy]; rifabutin/pd [Pharmacology]; rifampicin/cb [Drug Combination]; rifampicin/dt [Drug Therapy]; rifampicin/pd [Pharmacology]; streptomycin/cb [Drug Combination]; streptomycin/dt [Drug Therapy]; streptomycin/pd [Pharmacology].

Link to the Ovid Full Text or citation:

Click here for full text options

291.

The effectiveness of pyrazinamide-containing six-month short course chemotherapy.

Wada M.

Kekkaku. 75 (11) (pp 665-673), 2000. Date of Publication: November 2000.

AN: 2000430671

Incidence of tuberculosis worldwide will increase progressively unless the effective program is implemented immediately. In Japan, the decreasing of tuberculosis incidence has been very slow since 1977 and this trend has not been improved till now. Six-month regimens for the treatment of tuberculosis were recommended by IUATLD, ATS, CDC, and WHO and have been adopted in most developed countries since late 1980s, but not adopted in Japan till April, 1996. We studied effectiveness oF 6-month regimen including pyrazinamide (2HRZS or E/4HRE) on newly diagnosed pulmonary tuberculosis who started the treatment in the Fukujuji Hospital, Japan Anti-Tuberculosis Association (JATA). From January 1991 to December 1997, 726 newly diagnosed pulmonary tuberculosis patients started treatment with 6-month regimen. Bacillary negative conversion rate among 424 patients whose bacilli were susceptible to both isoniazid and rifampicin, was 92.9 % after 2 months of treatment and who completed treatment without change of treatment regimen. Among 726 cases, 593 (81.7%) succeeded, 48 (6.6%) defaulted, 53 (7.3%) were referred to other doctors, and 32 (4.4%) died. The relapse rate after completion of the treatment was 3.2 percent among 345 patients whose bacilli were susceptible to both isoniazid and rifampicin and who completed the treatment without change of regimen. The relapse rate among the patients complicated with diabetes mellitus (DM) was higher than that among non-DM patients (6.31/100 person-years vs 0.90/100 person-years) (P<0.001). When drug-induced hepatitis was defined as the elevation of serum liver enzyme levels with the clinical symptoms of hepatitis or their elevation over 5 times of normal upper limit, the incidence of drug-induced hepatitis among the patients treated with pyrazinamide-containing 6-month regimen was not higher than that among the patients treated with 9-month regimen without pyrazinamide (6HRS or E/3HR) (7.9 % vs 7.3%). The risk factors for drug-induced hepatitis included elderly, history of gastrectomy, hypoalbuminemia, the higher dose of isoniazid over than 7.5mg/kg, higher than 30mg/kg of pyrazinamide and positive HCV antibody. The effectiveness of 6-month regimen on the patients whose organisms were resistant to isoniazid and susceptible to rifampicin was studied. The average duration of the treatment for the patients started 6-month regimen was 3.2 months shorter than for the patients started 9-month regimen (10.2 months vs 13.4 months). I concluded that 6-month regimen containing-pyrazinamide was effective for the patients with isoniazid-rifampicin susceptible tuberculosis patients except the patients complicated with diabetes mellitus. But the frequency of drug-induced hepatitis was higher than that of previous reports, and further studies are needed to elucidate the cause of high frequency of hepatitis among Japanese patients.

Institution

(Wada) Research Institute of Tuberculosis, Japan Anti-Tuberculosis Association, 3-1-24, Matsuyama, Kiyose-shi, Tokyo 204-8533, Japan

Publisher

Japanese Society for Tuberculosis

Emtree Heading

article; clinical protocol; comorbidity; controlled study; diabetes mellitus; drug efficacy; Hepatitis C virus; human; incidence; Japan; *lung tuberculosis/dr [Drug Resistance]; *lung tuberculosis/dt [Drug Therapy]; *lung tuberculosis/ep [Epidemiology]; major clinical study; recurrence risk; risk factor; seroconversion; toxic hepatitis/si [Side Effect]; treatment outcome; ethambutol/cb [Drug Combination]; ethambutol/do [Drug Dose]; ethambutol/dt [Drug Therapy]; isoniazid/cb [Drug Combination]; isoniazid/do [Drug Dose]; isoniazid/dt [Drug Therapy]; *pyrazinamide/ae [Adverse Drug Reaction]; *pyrazinamide/cb [Drug Combination]; *pyrazinamide/do [Drug Dose]; *pyrazinamide/dt [Drug Therapy]; rifampicin/cb [Drug Combination]; rifampicin/do [Drug Dose]; rifampicin/dt [Drug Therapy]; streptomycin/cb [Drug Combination]; streptomycin/do [Drug Dose]; streptomycin/dt [Drug Therapy].

Link to the Ovid Full Text or citation:

Click here for full text options

292.

Evaluation of tuberculosis treatment results in Italy, report 1998.

Centis R., Ianni A., Migliori G.B., Casali L., Agati G., Aiolfi S., Altieri A., Ambrosetti M., Arossa W., Bagnasco G., Bazzerla G., Berra A., Besozzi G., Bottrighi P., Bugiani M., Calabro S., Castiglioni G., Cavallero M., Cicchitto G., Codecasa L.R., Colorizio V., Confalonieri M., D'Ambrosio L., D'Ambrosio V., DellaTommasa S., Di Pisa G., Ermeti M., Faccini E., Falcone F., Farinelli G., Fatigante R., Farris B., Fatur Volante T., Felisatti G., Ferranti P., Fiorentini F., Foschi C., Garzone A., Giorgio V., Gozzellino F., Jacopino L., Lauriello G., Le Donne R., Longi R., Macor G., Mancini D., Marchesani M.F., Marcias S., Mariano V., Mennea G., Monaco A., Montesano G., Moretti G., Napolitano G., Nardini S., Nutini S., Orani G., Orsi A., Parpanesi M., Pasi A., Penza O., Perboni A., Petrozzi L., Pretto P., Rossi S., Sabato E., Saini L., Schiavi L., Tazza R., Trucco G., Vertuccio C., Viviani U., Volante M., Vinciguerra P., Viola S., Zaccara F., Angarano G., Barelle L., Bertoli G., Bisetti A., Bistoni F., Buono L., Carbonara S., Carosi G.P., Cascina A., Cassone A., Costa D., Crepaldi P., De Santis A., Diamare F., Fabbro L., Fadda G., Farris A.G., Fattorini L., Ferrara G., Gamba S., Iona E., Losi M.

Monaldi Archives for Chest Disease. 55 (4) (pp 293-298), 2000. Date of Publication: 2000.

AN: 2000369479

In Italy no national data on tuberculosis (TB) treatment results were available. In 1995 the AIPO (Italian Association of Hospital Pneumologists) TB Study Group, in collaboration with the Istituto Superiore di Sanita (technical branch of the Ministry of Health), started a prospective monitoring activity based on World Health Organization (WHO) and International Union against Tuberculosis and Lung Disease (IUATLD) recommendations. Data were collected from a representative network of TB units nationwide, managing a significant proportion of all TB cases notified in Italy each year. The aim of this study was to analyse the case findings and treatment results for the year 1998. The number of TB cases reported was 1162 (61.7% males; 28.2% immigrants), 888 (76.6%) being new cases. Of these cases, 1,001 (86.4%) were pulmonary, 132 (11.4%) extrapulmonary and 26 (2.2%) both pulmonary and extrapulmonary. The main risk factors for TB were a history of recent contact, diabetes and alcohol abuse among native Italians and human immunodeficiency virus-seropositive status and history of recent contact among immigrants. The majority of immigrants were from Africa and South America, and had been in Italy >24 months before diagnosis of TB. Forty-seven per cent (552 of 1,162) of patients had a positive direct sputum smear examination for alcohol acid-fast bacilli; 30% were resistant to any drug (combined monoresistance (the sum of primary and acquired drug resistance) to rifampicin 2.3%; combined multidrug resistance 11.4%). In 97% of cases, the duration of treatment was <12 months. The overall success rate (cured plus treatment completed excluding transferred out from the denominator) was 83%. A significantly higher percentage of deaths was found in native Italians (being age-related; p<0.001), whereas immigrants had a higher default rate.

Institution

(Centis, Ianni, Migliori, Casali, Agati, Aiolfi, Altieri, Ambrosetti, Angarano, Arossa, Bagnasco, Bazzerla, Berra, Besozzi, Bottrighi, Bugiani, Calabro, Castiglioni, Cavallero, Cicchitto, Codecasa, Colorizio, Confalonieri, D'Ambrosio, D'Ambrosio, DellaTommasa, Di Pisa, Ermeti, Faccini, Falcone, Farinelli, Fatigante, Farris, Fatur Volante, Felisatti, Ferranti, Fiorentini, Foschi, Garzone, Giorgio, Gozzellino, Jacopino, Lauriello, Le Donne, Longi, Macor, Mancini, Marchesani, Marcias, Mariano, Mennea, Monaco, Montesano, Moretti, Napolitano, Nardini, Nutini, Orani, Orsi, Parpanesi, Pasi, Penza, Perboni, Petrozzi, Pretto, Rossi, Sabato, Saini, Schiavi, Tazza, Trucco, Vertuccio, Viviani, Volante, Vinciguerra, Viola, Zaccara, Angarano, Barelle, Bertoli, Bisetti, Bistoni, Buono, Carbonara, Carosi, Cascina, Cassone, Costa, Crepaldi, De Santis, Diamare, Fabbro, Fadda, Farris, Fattorini, Ferrara, Gamba, Iona, Losi) Fondazione Salvatore Maugeri, Clinica del Lavoro e Riabilitazione, Care and Research Institute, via Roncaccio 16, 21049 Tradate (VA), Italy

Publisher

PI-ME Tipografia Editrice S.r.l.

Emtree Heading

adolescent; adult; aged; alcohol abuse; article; child; cohort analysis; diabetes mellitus; female; human; Human immunodeficiency virus infection; immigrant; infant; Italy; *lung tuberculosis/dr [Drug Resistance]; *lung tuberculosis/dt [Drug Therapy]; *lung tuberculosis/ep [Epidemiology]; *lung tuberculosis/et [Etiology]; major clinical study; male; mortality; multidrug resistance; newborn; risk factor; sputum smear; treatment outcome; world health organization; alcohol; isoniazid/dt [Drug Therapy]; rifampicin/dt [Drug Therapy]; streptomycin/dt [Drug Therapy]; *tuberculostatic agent/dt [Drug Therapy].

Link to the Ovid Full Text or citation:

Click here for full text options

293.

Use of structural allografts in spinal osteomyelitis: A review of 47 cases.

Schuster J.M., Avellino A.M., Mann F.A., Girouard A.A., Grady M.S., Newell D.W., Winn H.R., Chapman J.R., Mirza S.K.

Journal of Neurosurgery. 93 (1 SUPPL.) (pp 8-14), 2000. Date of Publication: July 2000.

AN: 2000251160

Object. The use of structural allografts in spinal osteomyelitis remains controversial because of the perceived risk of persistent infection related to a devitalized graft and spinal hardware. The authors have identified 47 patients over the last 3.5 years who underwent a surgical decompression and stabilization procedure in which fresh-frozen allografts were used after aggressive removal of infected and devitalized tissue. The patients subsequently underwent 6 weeks of postoperative antibiotic therapy (12 months for those with tuberculosis [TB]). Methods. Follow-up data included results of serial clinical examinations, radiography, laboratory analysis (erythrocyte sedimentation rate and white blood cell count), and clinical outcome questionnaires. Of the original 47 patients (14 women and 33 men, aged 14-83 years), 39 were available for follow up. The average follow-up period at the time this article was submitted was 17 +/- 9 months (median 14 months, range 6-45 months). In the majority of cases (57%), a Staphylococcus species was the infectious organism. Predisposing risk factors included intravenous drug abuse (IVDA), previous surgery, diabetes, TB, and concurrent infections. During the follow-up period only two patients suffered recurrent infection at a contiguous level; both had a history of IVDA and one also had a chronic excoriating skin condition. No other recurrent infections have been identified, and no patient has required reoperation for persistent infection or allograft/hardware failure. Conclusions. It is the authors' opinion that the use of structural allografts in combination with aggressive tissue debridement and adjuvant antibiotic therapy provide a safe and effective therapy in cases of spinal osteomyelitis requiring surgery.

Institution

(Schuster) Department of Neurological Surgery, University of Washington, Harborview Medical Center, 325 Ninth Avenue, Seattle, WA 98104, United States

Publisher

American Association of Neurological Surgeons

Emtree Heading

adolescent; adult; aged; allograft; antibiotic therapy; article; clinical article; decompression surgery; diabetes mellitus; disease predisposition; female; graft failure; graft infection; human; intravenous drug abuse; male; *osteomyelitis/dt [Drug Therapy]; *osteomyelitis/su [Surgery]; priority journal; risk factor; spine stabilization; Staphylococcus; treatment outcome; tuberculosis; *antibiotic agent/dt [Drug Therapy].

Link to the Ovid Full Text or citation:

Click here for full text options

294.

Long-term peritoneal dialysis outcome in a single center.

Cancarini G.C., Sandrini M., Vizzardi V., Scaini P., Mombelloni S., Pola A., Maiorca R.

Peritoneal Dialysis International. 20 (SUPPL. 2) (pp S121-S126), 2000. Date of Publication: 2000.

AN: 2000221310

Institution

(Cancarini, Sandrini, Vizzardi, Scaini, Mombelloni, Pola, Maiorca) Department of Nephrology, University of Brescia, Division of Nephrology, Brescia, Italy (Cancarini) Divisione di Nefrologia, Azienda Ospedaliera, Spedali Civili di Brescia, Piazza Ospedale 1, Brescia 25123, Italy

Publisher

Multimed Inc.

Emtree Heading

adult; age; aged; article; biocompatibility; cerebrovascular disease; clinical trial; cohort analysis; comorbidity; continuous ambulatory peritoneal dialysis; female; hemodialysis; host resistance; human; hypertension; insulin dependent diabetes mellitus; ischemic heart disease; kidney failure/th [Therapy]; kidney transplantation; major clinical study; male; non insulin dependent diabetes mellitus; peripheral vascular disease; *peritoneal dialysis; priority journal; respiratory tract infection; risk factor; survival rate; treatment indication; treatment outcome; tuberculosis; peritoneal dialysis fluid.

Link to the Ovid Full Text or citation:

Click here for full text options

295.

The Italian AIPO study on tuberculosis treatment results, report 1997.

Ambrosetti M., Besozzi G., Codecasa L.R., Farris B., Nutini S., Sainit L., Casali L., Nardini S., Migliori G.B.

Monaldi Archives for Chest Disease. 54 (5) (pp 407-412), 1999. Date of Publication: 1999.

AN: 2000017674

In Italy no national data have been available on tuberculosis (TB) treatment results. In 1995 the AIPO (Italian Association of Hospital Pneumologists) TB Study Group, in collaboration with the Istituto Superiore di Sanita (technical branch of the Ministry of Health) started a prospective monitoring activity based on the recommendations of the World Health Organization and the International Union Against Tuberculosis and Lung Disease. The aim of this study was to analyse the case findings and treatment results during 1997. Data were collected from a representative network of TB units nationwide, managing a significant proportion of all TB cases notified in Italy each year. A total of 715 TB cases were reported (56% males; 24% immigrants), of which 635 (89%) were new cases. Of these cases 493 (69%) were pulmonary, 187 (26%) extrapulmonary cases and 35 (5%) both pulmonary and extrapulmonary. The main risk factors for TB were history of recent contact and diabetes among native Italians, human immunodeficiency virus-seropositive status and a history of recent contact among immigrants. The majority of immigrants came from Africa and Central and South America, and stayed in Italy for more than 24 months before the diagnosis of TB was made. Thirty-six per cent of patients had a positive direct sputum smear examination for alcohol acid-fast bacilli and 27% were resistant to any drug (monoresistance to isoniazid: 2.4%; multi-drug resistance: 7.5%). In 97% of cases the duration of treatment was < 12 months. The overall success rate (cured plus treatment completed) was 78.1%. A significantly higher percentage of deaths, which was age related, was found in native Italians, while immigrants had a higher default rate. In conclusion, case finding and treatment results in Italy in 1997 are similar to those described in 1995 and 1996.

Institution

(Ambrosetti, Migliori) Fondazione Salvatore Maugeri, Clin. del Lavoro e della Riabilit., Care and Research Institute, Tradate, Italy (Ambrosetti) Dept. of Clin. and Biol. Sciences, Faculty of Medicine, University of Insubria, Italy

(Besozzi, Codecasa) Istititto Villa Marelli P.T., Milano, Italy

(Farris) Cagliari Hospital, Italy

(Nutini) Dept. of Pulmonary Diseases, Careggi Hospital, Firenze, Italy

(Sainit) Dept. of Pulmonary Diseases, Novara Hospital, Italy

(Casali) Department of Pulmonary Diseases, Pavia University, Italy

(Ambrosetti) National AIPO TB Study Group, Fondazione Salvatore Maugeri, Care and Research Institute, via Roncaccio 16, 21049 Tradate (VA), Italy

Publisher

PI-ME Tipografia Editrice S.r.l.

Emtree Heading

acid fast bacterium; adolescent; adult; Africa; age; aged; antibiotic resistance; article; case finding; Central America; child; death; diabetes mellitus; female; human; Human immunodeficiency virus; immigrant; infant; Italy; lung tuberculosis/ep [Epidemiology]; lung tuberculosis/et [Etiology]; major clinical study; male; multidrug resistance; outcomes research; risk factor; South America; sputum smear; *tuberculosis/ep [Epidemiology]; *tuberculosis/et [Etiology]; world health organization; alcohol; isoniazid.

Link to the Ovid Full Text or citation:

Click here for full text options

296.

The Italian AIPO study on tuberculosis treatment results, report 1996.

Ambrosetti M., Besozzi G., Farris B., Nutini S., Saini L., Casali L., Pretto P., Orani G., Calabro S., Migliori G.B.

Monaldi Archives for Chest Disease. 54 (3) (pp 237-241), 1999. Date of Publication: 1999.

AN: 1999273056

In Italy, no national data on tuberculosis (TB) treatment results were available. In 1995, the AIPO (Italian Association of Hospital Pneumologists) TB Study Group, in collaboration with the Istituto Superiore di Sanita (technical branch of the Ministry of Health), started a prospective monitoring activity based on World Health Organization (WHO) and International Union against Tuberculosis and Lung Disease (IUATLD) recommendations. Data were collected from a nationwide network of 41 TB units, managing a significant proportion of all the TB cases notified in Italy each year. The aim of this study was to analyse the case findings and treatment results for the year 1996. Eight hundred and thirty eight TB cases were reported (56% males; 26% immigrants), 708 (84%) being new cases. Of these cases, 565 (67%) were pulmonary, 235 (28%) extrapulmonary and 38 (5%) both pulmonary and extrapulmonary. The main risk factors for TB were a history of recent contact and diabetes among native Italians and recent contact and human immunodeficiency virus-seropositive status among immigrants. The majority of immigrants were from Africa and Asia, and had been in Italy > 24 months before diagnosis of TB. Thirty-six per cent of patients had a positive direct sputum smear examination for alcohol acid-fast bacilli; 37% were resistant to any drug (monoresistance to isoniazid 4.4%; multidrug resistance 10.4%). In 96% of cases, the duration of treatment was < 12 months. The overall success rate (cured plus treatment completed) was 84.1%. A significantly higher percentage of deaths was found in native Italians (being age-related), whereas immigrant's had a higher default rate.

Institution

(Ambrosetti, Migliori) Fondazione Salvatore Maugeri, Clin. del Lavoro e della Riabilit., Care and Research Institute, Tradate, Italy (Ambrosetti) Dept. of Clin. and Biol. Sciences, Faculty of Medicine, University of Insubria, Italy

(Besozzi) Ist. Villa Marelli per Patol. Torace, Milan, Italy

(Farris, Orani) Cagliari Dispensary, Italy

(Nutini) Dept. of Pulmonary Diseases, Careggi Hospital, Florence, Italy

(Saini) Dept. of Pulmonary Diseases, Novara Hospital, Italy

(Casali) Dept. of Pulmonary Diseases, Pavia University, Italy

(Pretto) Bolzano Dispensary, Italy

(Calabro) Feltre Hospital, Italy

(Migliori) Natl. AIPO Tuberculosis Study Group, Fondazione Salvatore Maugeri, Care and Research Institute, via Roncaccio 16, 21049, Tradate (VA), Italy

Publisher

PI-ME Tipografia Editrice S.r.l.

Emtree Heading

acid fast bacterium; adolescent; adult; Africa; age; aged; alcoholism; article; Asia; child; cohort analysis; diabetes mellitus; drug dependence; female; human; Human immunodeficiency virus infection; immigrant; infant; Italy; lung tuberculosis/dr [Drug Resistance]; lung tuberculosis/dt [Drug Therapy]; major clinical study; male; mortality; multidrug resistance; patient compliance; risk factor; sputum cytodiagnosis; steroid therapy; *treatment outcome; *tuberculosis/dr [Drug Resistance]; *tuberculosis/dt [Drug Therapy]; isoniazid/dt [Drug Therapy]; pyrazinamide/dt [Drug Therapy]; rifampicin/dt [Drug Therapy]; streptomycin/dt [Drug Therapy].

Link to the Ovid Full Text or citation:

Click here for full text options

297.

Assessment of a combined preparation of isoniazid, rifampicin and pyrazinamide (Rifater) in the initial phase of chemotherapy in three 6- month regimens for smear-positive pulmonary tuberculosis: A five-year follow- up report.

Teo S.K.

International Journal of Tuberculosis and Lung Disease. 3 (2) (pp 126-132), 1999. Date of Publication: February 1999.

AN: 1999116554

SETTING: Singapore Tuberculosis Service. OBJECTIVE: To assess the acceptability, efficacy and relapse rate of a combined formulation of three drugs-isoniazid, rifampicin and pyrazinamide (Rifater)-given in the initial phase of chemotherapy in three 6-month regimens (2SHRZ/4H3R3, 1SHRZ/5H3R3 and 2HRZ/4H3R3) under direct observation for all patients. DESIGN: A randomised, controlled, unblinded study comparing a group of patients treated with Rifater and another given the three component drugs as separate formulations. RESULTS: The 310 patients admitted to the study were divided into two groups of 155 patients. The frequency of side effects was similar in both groups. Of 271 patients with drug-sensitive strains who had completed treatment without interruption, sputum cultures converted in all patients. At the end of 5 years, there were 15 relapses: three (2.2%) in the separate drugs group and 12 (9.3%) in the Rifater group. Exclusion of two cases in the Rifater group, one with silicotuberculosis and another with no bacteriological confirmation of diagnosis, gave a relapse rate of 7.9% (P = 0.03 for the comparison of relapse rates in the two groups). CONCLUSION: A combined formulation of three drugs given daily in the initial phase of 6- month short-course therapy, followed by intermittent treatment with isoniazid and rifampicin given three times a week under direct observation for all patients, appears to be less effective than treatment with the component drugs given as separate formulations.

Institution

(Teo) Department of Medicine, Alexandra Hospital, Singapore, Singapore (Teo) 378 Alexandra Road, Singapore 159964, Singapore

Publisher

International Union against Tubercul. and Lung Dis.

Emtree Heading

article; clinical trial; combination chemotherapy; controlled clinical trial; controlled study; diabetes mellitus; drug efficacy; follow up; human; Human immunodeficiency virus infection; *lung tuberculosis/dr [Drug Resistance]; *lung tuberculosis/dt [Drug Therapy]; *lung tuberculosis/ep [Epidemiology]; major clinical study; Mycobacterium tuberculosis; priority journal; randomized controlled trial; recurrence risk; Singapore; sputum culture; treatment outcome; *isoniazid/ct [Clinical Trial]; *isoniazid/cb [Drug Combination]; *isoniazid/cm [Drug Comparison]; *isoniazid/do [Drug Dose]; *isoniazid/dt [Drug Therapy]; *pyrazinamide/ct [Clinical Trial]; *pyrazinamide/cb [Drug Combination]; *pyrazinamide/cm [Drug Comparison]; *pyrazinamide/do [Drug Dose]; *pyrazinamide/dt [Drug Therapy]; *rifampicin/ct [Clinical Trial]; *rifampicin/cb [Drug Combination]; *rifampicin/cm [Drug Comparison]; *rifampicin/do [Drug Dose]; *rifampicin/dt [Drug Therapy]; *rifater/ct [Clinical Trial]; *rifater/cb [Drug Combination]; *rifater/cm [Drug Comparison]; *rifater/do [Drug Dose]; *rifater/dt [Drug Therapy]; streptomycin/cb [Drug Combination]; streptomycin/dt [Drug Therapy].

Drug Trade Names and Manufacturers

Bracco [Italy], Lepetit [Italy], rifater: Lepetit [Italy]

Link to the Ovid Full Text or citation:

Click here for full text options

298.

The frequency, aetiology and outcome of severe hyponatraemia in adult hospitalised patients.

Erasmus R.T., Matsha T.E.

The Central African journal of medicine. 44 (6) (pp 154-158), 1998. Date of Publication: Jun 1998.

AN: 9810397

OBJECTIVE: To study the frequency, outcome and association with neurological signs and symptoms of severe hyponatraemia (< 120 mmol/l) in adult hospitalised patients. DESIGN: Retrospective study of severe hyponatraemia using laboratory data to identify patients. SETTING: 800 bed peri-urban hospital in the former homeland of Transkei. SUBJECTS: All adult inpatients from the medical and surgical wards with severe hyponatraemia during a 10 month period of which 18 cases records could not be traced and one was antefactual. MAIN OUTCOME MEASURES: Incidence of severe hyponatraemia, mortality, admission hyponatraemia, hospital acquired hyponatraemia, neurological symptoms and outcome. RESULTS: The overall incidence of severe hyponatraemia was 1.1%. Among the 58 patients (mean age 49.3 years), pulmonary tuberculosis (24%), Diabetes mellitus (19%) and renal failure (17.2%) were the principal aetiological conditions occurring in approximately 60% of hyponatraemic patients. A high mortality rate of 44.8% was observed which was probably associated with the severity of the underlying disease. 36.2% of patients had serum sodium levels of less than 115 mmol/l with a significantly (p < 0.05) higher mortality (62%) than those with values greater than 115 mmol/l (32%). Admission hyponatraemia (63.8%) was more frequent (p < 0.05) than hospital acquired hyponatraemia (36.2%); in the latter, hyponatraemia was more likely to normalise (p < 0.05). Neurological signs and symptoms developed in 17.2%, this being associated with a high mortality and lower mean serum sodium levels of 112 mmol/l respectively. Following therapy, serum sodium levels normalised in nearly half the patients. In three (5.2%) patients, the hyponatraemia worsened whilst another three became hypernatraemic. CONCLUSION: The incidence of severe hyponatraemia was lower than that observed in developed countries. It was associated with severe underlying disease and had a high mortality rate. Patients whose hyponatraemia did not normalise were more likely to have higher mortality.

Institution

(Erasmus, Matsha) Department of Chemical Pathology, Faculty of Medicine, University of Transkei, Umtata, E Cape, South Africa.

Emtree Heading

adolescent; adult; article; blood; female; *hospitalization; human; *hyponatremia/et [Etiology]; *hyponatremia/th [Therapy]; incidence; male; middle aged; mortality; prognosis; retrospective study; risk factor; South Africa; treatment outcome.

Link to the Ovid Full Text or citation:

Click here for full text options
