# Supplementary material for: Effect of glycemic control and type of diabetes treatment on TB treatment outcomes among people with TB-diabetes: A systematic review (updated August 2024)
Source: PLoS One. 2025 Jul 18;20(7):e0328619. doi: 10.1371/journal.pone.0328619 (PMC12273911; doi:10.1371/journal.pone.0328619)
Supplement: S1 Appendix — (ZIP) [file pone.0328619.s004.zip › S1 appendix_old/EMBASE/81_without risk search reuslt no 16.doc]

Database: Embase <1996 to 2016 September 16>

Search Strategy:

--------------------------------------------------------------------------------

1	tuberculosis.mp. or exp tuberculosis/ (151164)

2	exp treatment outcome/ or treatment outcome*.mp. (1189741)

3	diabetes mellitus.mp. or exp diabetes mellitus/ (627157)

4	diabetes mellitus/ (362441)

5	DM.mp. (48079)

6	diabetes.mp. (663193)

7	mellitus.mp. (588341)

8	3 or 4 or 5 or 6 or 7 (715025)

9	1 and 2 and 8 (724)

10	cohort studies.mp. or cohort analysis/ (263408)

11	exp risk factor/ or cohort.mp. or exp risk/ (2148599)

12	10 or 11 (2148599)

13	9 and 10 (81)

14	9 and 12 (304)

15	limit 14 to human (298)

16	limit 13 to human (81)

17	trial*.mp. or exp controlled clinical trial/ or exp "clinical trial (topic)"/ or exp "randomized controlled trial (topic)"/ or exp "controlled clinical trial (topic)"/ (1555047)

18	random.ab. (191735)

19	randomized controlled trial.pt. (0)

20	controlled clinical trial.pt. (0)

21	randomized.ab. (454621)

22	placebo.ab. (188623)

23	clinical trials as topic.sh. (15)

24	trial.ti. (176210)

25	17 or 18 or 19 or 20 or 21 or 22 or 23 or 24 (1831290)

26	9 and 25 (128)

27	limit 26 to human (128)

***************************

1.

Screening for diabetes mellitus in tuberculosis patients in a referral center in Iran.

Baghaei P., Tabarsi P., Marjani M., Moniri A., Masjedi M.R.

Infectious Diseases. 47 (7) (pp 472-476), 2015. Date of Publication: 2015.

AN: 20160253952

Background: A significant link between diabetes mellitus (DM) and tuberculosis (TB) has been widely demonstrated. DM increases the risk of TB in all aspects. The aims of this study were to assess the prevalence of DM among newly diagnosed TB patients, to screen these patients for DM, and to determine the number needed to screen (NNS) to diagnose new cases of DM. Methods: A prospective cohort descriptive study was carried out in Iranian adults admitted to the National Research Institute of Tuberculosis and Lung Disease from 2012 to 2013 with a new diagnosis of TB. Glycated hemoglobin (HbA1c) and fasting blood glucose (FBG) were measured for all patients. Results: Of the 293 patients included, 101 (34.5%) had DM. DM was newly diagnosed in 45 (19%) patients. The number needed to screen was 5 to identify one new DM case. Age > 40 years was associated with DM in this population (odds ratio (OR)=3.91, 95% confidence interval (CI)=1.47 - 10.38). Conclusion: Screening for DM should be performed routinely in patients with TB and may improve treatment outcomes.

Institution

(Baghaei, Tabarsi, Marjani, Moniri, Masjedi) Clinical Tuberculosis and Epidemiology Research Center, National Research Institute of Tuberculosis and Lung Diseases (NRITLD), Shahid Beheshti University of Medical Sciences, Tehran, Iran, Islamic Republic of

Publisher

Taylor and Francis Ltd.

Emtree Heading

adult; age; article; cohort analysis; descriptive research; *diabetes mellitus/di [Diagnosis]; diabetic patient; fasting blood glucose; female; gender; glucose blood level; human; impaired fasting glucose; impaired glucose tolerance; Iran; *lung tuberculosis/di [Diagnosis]; major clinical study; male; number needed to screen; prevalence; prospective study; screening test; sensitivity and specificity; glucose/ec [Endogenous Compound]; hemoglobin A1c/ec [Endogenous Compound].

Link to the Ovid Full Text or citation:

Click here for full text options

2.

Prevalence, risk factors, management, and treatment outcomes of first-line antituberculous drug-induced liver injury: a prospective cohort study.

Sun Q., Zhang Q., Gu J., Sun W.-W., Wang P., Bai C., Xiao H.-P., Sha W.

Pharmacoepidemiology and Drug Safety. 25 (8) (pp 908-917), 2016. Date of Publication: 01 Aug 2016.

AN: 20160572220

Purpose: Antituberculosis drug-induced liver injury (ATDILI) is one of the most deleterious side effects associated with chemotherapy against tuberculosis (TB). In this study, our objective was to determine the incidence, risk factors, and management of ATDILI and analyze its impact on the treatment outcome in patients receiving standard anti-TB chemotherapy. Methods: A prospective cohort study of ATDILI prevalence was conducted in 938 enrolled patients of the 1426 TB cases in Shanghai from March 2011 to September 2012. Patients were followed up until February 2014. Univariate and multivariate logistic regression analyses were used to determine the risk factors of ATDILI. Successful therapeutic outcome, rates of drug resistance conversion, sputum smear/culture conversion, and lung cavity closure were analyzed. Results: Hepatitis B surface antigen/hepatitis B e antigen-positive hepatitis B carriers, complicated with systemic lupus erythematosus, albumin < 25 g/L, and chronic alcoholism were independent risk factors for ATDILI. Of the 121 cases with ATDILI (incidence rate of 12.9%), 84 (69.4%) used modified anti-TB therapy after recovery of liver function. Compared with the non-ATDILI group, patients with ATDILI exhibited remarkably decreased lung cavity closure rate (84.6% vs. 93.0%, P < 0.001) along with significantly reduced sputum smear/culture conversion rate (85.4% vs. 94.0%, P < 0.001). Conclusions: Our findings indicated that 12.9% patients developed ATDILI during standard anti-TB therapy, resulting in poor therapeutic outcome. Hepatitis B carriers with systemic lupus erythematosus, albumin < 25 g/L, and chronic alcoholism manifested increased risks for ATDILI. Copyright © 2016 John Wiley & Sons, Ltd.

Institution

(Sun, Zhang, Gu, Sun, Wang, Xiao, Sha) Clinic and Research Center of Tuberculosis, Shanghai Key Laboratory of Tuberculosis, Shanghai Pulmonary Hospital, Tongji University School of Medicine, Shanghai, China (Sun, Bai) Department of Respiratory and Critical Care Medicine, Changhai Hospital, the Second Military Medical University, Shanghai, China

Publisher

John Wiley and Sons Ltd (Southern Gate, Chichester, West Sussex PO19 8SQ, United Kingdom)

Emtree Heading

adult; age; albumin blood level; alcoholism; article; body mass; cohort analysis; convalescence; diabetes mellitus; extrapulmonary tuberculosis; female; follow up; gender; hemoglobin blood level; hepatitis B; human; hypertension; incidence; liver function; lung cavity; major clinical study; male; prevalence; priority journal; prognosis; prospective study; risk factor; smoking; sputum culture; systemic lupus erythematosus; *toxic hepatitis/ep [Epidemiology]; *toxic hepatitis/et [Etiology]; *toxic hepatitis/si [Side Effect]; treatment outcome; albumin/ec [Endogenous Compound]; hemoglobin/ec [Endogenous Compound]; hepatitis B surface antigen; hepatitis B(e) antigen; *tuberculostatic agent/ae [Adverse Drug Reaction].

Link to the Ovid Full Text or citation:

Click here for full text options

3.

The influence of smoking on pulmonary tuberculosis in diabetic and non-diabetic patients.

Bai K.-J., Lee J.-J., Chien S.-T., Suk C.-W., Chiang C.-Y.

PLoS ONE. 11 (6) (no pagination), 2016. Article Number: e0156677. Date of Publication: 01 Jun 2016.

AN: 20160457069

Background: Both smoking and diabetes can increase the risk and influence the manifestations and outcomes of tuberculosis (TB). It is not clear whether the influence of smoking on pulmonary TB differs between non-diabetic and diabetic patients. Herein, we assessed the manifestations and outcomes of TB in relation to smoking in both diabetic and non-diabetic TB patients. Methodology/Principal Findings: All diabetic culture-positive pulmonary TB patients notified from 2005-2010 at three teaching hospitals in Taiwan were enrolled. A culture-positive pulmonary TB patient without DM who was notified to the health authority immediately prior to each diabetic TB patient was selected for comparison. The 972 patients in this study cohort included 365 (37.6%) nondiabetic non-smokers, 149 (15.3%) non-diabetic smokers, 284 (29.2%) diabetic non-smokers, and 174 (17.9%) diabetic smokers. The adjusted relative risk of a pretreatment positive smear for a smoker compared with a non-smoker was 2.19 (95% CI 1.38-3.47) in non-diabetic patients and 2.23 (95% CI 1.29-3.87) in diabetic culture-positive pulmonary TB patients. The adjusted relative risk for a positive smear among diabetic smokers was 5.61 (95% CI 3.35-9.41) compared with non-diabetic non-smokers. Smoking was significantly associated with an increased frequency of bilateral lung parenchyma involvement (AdjOR 1.84, 95% CI 1.16-2.93), far-advanced pulmonary TB (AdjOR 1.91, 95% CI 1.04-3.50), cavitary lesions (AdjOR 2.03, 95% CI 1.29-3.20), and unfavorable outcomes of TB (AdjOR 2.35, 95% CI 1.02-5.41) in non-diabetic patients. However, smoking was not associated with cavitary lung parenchyma lesions regarding the location, number or size of the cavity in diabetic TB patients. Conclusions/Significance: Smoking and diabetes have joint effects on a pretreatment positive smear. Diabetic smokers had more than a 5-fold increased risk of a pretreatment positive smear than did non-diabetic non-smokers, indicating remarkable joint effects of diabetes and smoking on the risk of TB transmission.

Institution

(Bai, Suk, Chiang) Division of Pulmonary Medicine, Department of Internal Medicine, Wan Fang Hospital, Taipei Medical University, Taipei, Taiwan (Republic of China) (Bai) School of Respiratory Therapy, College of Medicine, Taipei Medical University, Taipei, Taiwan (Republic of China)

(Lee) Department of Internal Medicine, Buddhist Tzu Chi General Hospital, Tzu Chi University, Hualien, Taiwan (Republic of China)

(Chien) Chest Hospital, Department of Health and Welfare, Tainan, Taiwan (Republic of China)

(Chiang) Department of Internal Medicine, School of Medicine, College of Medicine, Taipei Medical University, Taipei, Taiwan (Republic of China)

(Chiang) International Union Against Tuberculosis and Lung Disease, Paris, France

Publisher

Public Library of Science

Emtree Heading

adult; aged; article; cohort analysis; controlled study; *diabetic patient; disease association; disease course; disease severity; female; human; *lung tuberculosis/di [Diagnosis]; major clinical study; male; outcome assessment; *smoking; sputum culture; sputum smear; Taiwan.

Link to the Ovid Full Text or citation:

Click here for full text options

4.

Treatment effect analysis of the standard regimen and the optimized regimen for retreatment pulmonary tuberculosis complicated with diabetes.

Du J., Gao W., Ma Y., Zhong Q., Liang X., Fu Y., Ji B., Xie L., Huang X., Ge Q., Han X., Tian X., Shu W., Li L.

Zhonghua jie he he hu xi za zhi = Zhonghua jiehe he huxi zazhi = Chinese journal of tuberculosis and respiratory diseases. 38 (12) (pp 886-891), 2015. Date of Publication: 01 Dec 2015.

AN: 26850888

OBJECTIVE: To analyze the therapeutic effects of the standard regimen and the optimized regimen in retreatment pulmonary tuberculosis complicated with diabetes mellitus (DM). METHODS: In a multi-center cohort study, patients with smear positive retreatment pulmonary tuberculosis (TB) with DM and those without DM [excluding multi-drug resistance (MDR), extensively drug-resistant (XDR) and non-tuberculosis Mycobacterium pulmonary disease(NTM)] were enrolled. There were a total of 178 cases, including 60 smear positive retreatment TB patients with DM and 118 without DM, who were randomly divided into 4 groups: Optimized group 1 [individualized treatment in 30 DM cases, 29 males, age (48 +/- 11)], retreatment group 1 [standard retreatment regimen in 30 DM cases, 28 males, age(48 +/- 10)], Optimized group 2[individual regimen in 57 non-DM cases, 37 males, age (41 +/- 14)], and retreatment group 2 [standard retreatment regimen in 61 non-DM cases, 49 males, age (43 +/- 13)]. Patients in the optimized group were treated with optimized individualized regimen based on DST result, with 3-4 sensitive drugs in the regimen, while those in the retreatment group were treated with national standard retreatment regimen. The therapeutic effect of different groups were recorded and the related factors of treatment outcome were analyzed with Chi-square test and multi-factor analysis.

RESULTS: The treatment success rates of the optimized group 1 and the retreatment group 1 were 83.3%(25/30) and 60.0%(18/30), respectively, and the difference was statistically significant (chi(2)=4.02, P=0.045<0.05). The treatment failure rate of the optimized group 1 (6.7%, 2/30) and the retreatment group 1(30.0%, 9/30) was statistically different (chi(2)=5.46, P=0.02<0.05). The outcome difference between the optimized group 2 and the retreatment group 2 showed no statistical significance. Multi-factor analysis showed that treatment regimen, DM, gender and drug resistance were the significant factors related with treatment outcome. The probability of treatment success using the individualized treatment regimen was 2.7 times higher than that using the standard regimen (P=0.025). The risk of treatment failure of the drug resistance cases was 2.8 times higher than that of the drug sensitive cases (P=0.038). The probability of treatment success in DM cases was 0.4 times that in non-DM cases (P<0.05).

CONCLUSION: The outcome of the optimized regimen group was better than that of the standard regimen group, and retreatment TB patients complicated with DM faced a higher risk of treatment failure, which should receive more attention.

Institution

(Du, Gao, Ma, Zhong, Liang, Fu, Ji, Xie, Huang, Ge, Han, Tian, Shu) Beijing Chest Hospital, Capital Medical University, Beijing Tuberculosis and Thoracic Tumor Research Institute, Beijing 101149, China	(Li) Email:

Emtree Heading

adult; cohort analysis; controlled study; diabetes mellitus; female; human; *lung tuberculosis; male; middle aged; randomized controlled trial; retreatment; treatment outcome.

Link to the Ovid Full Text or citation:

Click here for full text options

5.

Global urine metabolomics in patients treated with first-line tuberculosis drugs and identification of a novel metabolite of ethambutol.

Das M.K., Arya R., Debnath S., Debnath R., Lodh A., Bishwal S.C., Das A., Nanda R.K.

Antimicrobial Agents and Chemotherapy. 60 (4) (pp 2257-2264), 2016. Date of Publication: April 2016.

AN: 20160308876

Population level variation of drug metabolism phenotype (DMP) has great implications in treatment outcome, drug-related side effects, and resistance development. In this study, we used a gas chromatography-time of flight-mass spectrometry (GC-TOF-MS)-based untargeted urine metabolomics approach to understand the DMP of a tuberculosis (TB) patient cohort (n = 20) from Tripura, a state in the northeastern part of India. Urine samples collected at different postdose time points (2 h, 6 h, 12 h, 24 h, 36 h, and 48 h) from these newly diagnosed TB patients receiving first-line anti-TB drugs were analyzed, and we have successfully detected three of the four first-line drugs, viz., isoniazid (INH), ethambutol (ETB), andpyrazinamide (PZA). The majority of their known metabolites, acetyl-isoniazid (AcINH), isonicotinic acid (INA), isonicotinuric acid (INTA), 2,2'-(ethylenediimino)-dibutyric acid (EDBA), 5-hydroxypyrazinamide (5OH-PZA), pyrazinoic acid (POA), and 5-hydroxypyrazinoic acid (5OH-POA), were also detected. Analyzing the variation in abundances of drugs and their known metabolites and calculating the metabolic ratios in these samples, we offer comprehensive DMP information on this small patient cohort that represents Tripura, India. The majority (75%) of these patients are found to be slow acetylators of INH. The average metabolic ratios of POA/PZA and 5OH-POA/POA are 3.16 +/- 3.03 and 6.09 +/- 6.15, respectively. Employing correlation analysis of the metabolomics metadata and a manual prediction of drug catabolism, we have proposed 2-aminobutyric acid (AABA) as a novel metabolite of ETB. These observations indicate the usefulness of GC-MS-based metabolomics to characterize the DMP at a population level and also to identify novel drug metabolites.

Institution

(Das, Arya, Bishwal, Nanda) Immunology Group, International Centre for Genetic Engineering and Biotechnology (ICGEB), New Delhi, India (Debnath, Debnath, Lodh, Das) Department of Tuberculosis and Respiratory Disease, Agartala Government Medical College, Agartala, Tripura, India

(Bishwal) School of Life Sciences, Sambalpur University, Sambalpur, Odisha, India

Publisher

American Society for Microbiology

Emtree Heading

adult; aged; article; aspartate aminotransferase blood level; body mass; chemical structure; chronic kidney disease; clinical article; cohort analysis; computer assisted tomography; controlled study; diabetes mellitus; drug megadose; drug metabolism; female; gas chromatography; human; *lung tuberculosis/dt [Drug Therapy]; male; metabolic ratio; *metabolomics; obesity; priority journal; sputum cytodiagnosis; time of flight mass spectrometry; underweight; *urinalysis; very elderly; 2 aminobutyric acid; 2,2' (ethylenediimino)dibutyric acid; 5 hydroxypyrazinamide; 5 hydroxypyrazinoic acid; acetylisoniazid; alanine aminotransferase/ec [Endogenous Compound]; alkaline phosphatase; *ethambutol/dt [Drug Therapy]; *isoniazid/dt [Drug Therapy]; isonicotinic acid; isonicotinuric acid; *pyrazinamide/dt [Drug Therapy]; pyrazinoic acid; unclassified drug.

Link to the Ovid Full Text or citation:

Click here for full text options

6.

Changing Trends in Complications and Mortality Rates among US Youth and Young Adults with HIV Infection in the Era of Combination Antiretroviral Therapy.

Mirani G., Williams P.L., Chernoff M., Abzug M.J., Levin M.J., Seage G.R., Oleske J.M., Purswani M.U., Hazra R., Traite S., Zimmer B., Van Dyke R.B.

Clinical Infectious Diseases. 61 (12) (pp 1850-1861), 2015. Date of Publication: 15 Dec 2015.

AN: 20160250451

Background. Combination antiretroviral therapy (cART) has resulted in a dramatic decrease in human immunodeficiency virus (HIV)-related opportunistic infections and deaths in US youth, but both continue to occur. Methods. We estimated the incidence of complications and deaths in IMPAACT P1074, a long-term US-based prospective multicenter cohort study conducted from April 2008 to June 2014. Incidence rates of selected diagnoses and trends over time were compared with those from a previous observational cohort study, P219C (2004-2007). Causes of death and relevant demographic and clinical features were reviewed. Results. Among 1201 HIV-infected youth in P1074 (87% perinatally infected; mean [standard deviation] age at last chart review, 20.9 [5.4] years), psychiatric and neurodevelopmental disorders, asthma, pneumonia, and genital tract infections were among the most common comorbid conditions. Compared with findings in P219C, conditions with significantly increased incidence included substance or alcohol abuse, latent tuberculosis, diabetes mellitus, atypical mycobacterial infections, vitamin D deficiency or metabolic bone disorders, anxiety disorders, and fractures; the incidence of pneumonia decreased significantly. Twenty-eight deaths occurred, yielding a standardized mortality rate 31.5 times that of the US population. Those who died were older, less likely to be receiving cART, and had lower CD4 cell counts and higher viral loads. Most deaths (86%) were due to HIV-related medical conditions. Conclusions. Opportunistic infections and deaths are less common among HIV-infected youth in the US in the cART era, but the mortality rate remains elevated. Deaths were associated with poor HIV control and older age. Emerging complications, such as psychiatric, inflammatory, metabolic, and genital tract diseases, need to be addressed.

Institution

(Mirani, Van Dyke) Tulane University School of Medicine, New Orleans, LA, United States (Williams, Chernoff, Traite) Center for Biostatistics in AIDS Research, United States

(Williams) Department of Biostatistics, Harvard T. H. Chan School of Public Health, 655 Huntington Ave, Boston, MA 02115, United States

(Williams, Seage) Department of Epidemiology, Harvard T. H. Chan School of Public Health, Boston, MA, United States

(Abzug, Levin) University of Colorado, School of Medicine and Children's Hospital Colorado, Aurora, United States

(Oleske) Rutgers New Jersey Medical School, Newark, United States

(Purswani) Albert Einstein College of Medicine, Bronx-Lebanon Hospital Center, Bronx, United States

(Zimmer) Frontier Science Technology and Research Foundation, Amherst, NY, United States

(Hazra) Eunice Kennedy Shriver National Institute of Child Health and Human Development, Bethesda, MD, United States

Publisher

Oxford University Press

Emtree Heading

adolescent; adult; age; alcohol abuse; anxiety disorder; appendicitis; article; asthma; atypical mycobacteriosis; Black person; body mass; cause of death; CD4 lymphocyte count; cellulitis; Chlamydia; chlamydiasis; clinical feature; cohort analysis; *combination chemotherapy; communication disorder; controlled study; diabetes mellitus; disease severity; dyslipidemia; eczema; esophagus candidiasis; ethnicity; failure to thrive; female; fetus death; follow up; fracture; gastroesophageal reflux; gender; genital tract infection; gonorrhea; hepatitis; herpes simplex; herpes zoster; *highly active antiretroviral therapy; Hispanic; human; Human immunodeficiency virus; *Human immunodeficiency virus infection/ep [Epidemiology]; *Human immunodeficiency virus infection/dt [Drug Therapy]; hypertension; incidence; induced abortion; intrauterine growth retardation; iron deficiency anemia; kidney disease; latent tuberculosis; learning disorder; lipodystrophy; major clinical study; male; medical record review; mental disease; metabolic bone disease; mood disorder; *mortality; mortality rate; neutropenia; observational study; opportunistic infection; outcome assessment; papillomavirus infection; perinatal infection; pneumonia; posttraumatic stress disorder; preeclampsia; premature labor; priority journal; prospective study; pulmonary candidiasis; spontaneous abortion; substance abuse; syphilis; trend study; trichomoniasis; virus load; vitamin deficiency; wasting syndrome; young adult; *antiretrovirus agent/dt [Drug Therapy]; virus RNA; vitamin D.

Link to the Ovid Full Text or citation:

Click here for full text options

7.

Screening for diabetes mellitus in tuberculosis patients in a referral center in Iran.

Baghaei P., Tabarsi P., Marjani M., Moniri A., Masjedi M.R.

Infectious Diseases. 47 (7) (pp 472-476), 2015. Date of Publication: 2015.

AN: 20160253952

Background: A significant link between diabetes mellitus (DM) and tuberculosis (TB) has been widely demonstrated. DM increases the risk of TB in all aspects. The aims of this study were to assess the prevalence of DM among newly diagnosed TB patients, to screen these patients for DM, and to determine the number needed to screen (NNS) to diagnose new cases of DM. Methods: A prospective cohort descriptive study was carried out in Iranian adults admitted to the National Research Institute of Tuberculosis and Lung Disease from 2012 to 2013 with a new diagnosis of TB. Glycated hemoglobin (HbA1c) and fasting blood glucose (FBG) were measured for all patients. Results: Of the 293 patients included, 101 (34.5%) had DM. DM was newly diagnosed in 45 (19%) patients. The number needed to screen was 5 to identify one new DM case. Age > 40 years was associated with DM in this population (odds ratio (OR)=3.91, 95% confidence interval (CI)=1.47 - 10.38). Conclusion: Screening for DM should be performed routinely in patients with TB and may improve treatment outcomes.

Institution

(Baghaei, Tabarsi, Marjani, Moniri, Masjedi) Clinical Tuberculosis and Epidemiology Research Center, National Research Institute of Tuberculosis and Lung Diseases (NRITLD), Shahid Beheshti University of Medical Sciences, Tehran, Iran, Islamic Republic of

Publisher

Taylor and Francis Ltd.

Emtree Heading

adult; age; article; cohort analysis; descriptive research; *diabetes mellitus/di [Diagnosis]; diabetic patient; fasting blood glucose; female; gender; glucose blood level; human; impaired fasting glucose; impaired glucose tolerance; Iran; *lung tuberculosis/di [Diagnosis]; major clinical study; male; number needed to screen; prevalence; prospective study; screening test; sensitivity and specificity; glucose/ec [Endogenous Compound]; hemoglobin A1c/ec [Endogenous Compound].

Link to the Ovid Full Text or citation:

Click here for full text options

8.

Does enhanced diabetes management reduce the risk and improve the outcome of tuberculosis?.

Lo H.-Y., Yang S.-L., Lin H.-H., Bai K.-J., Lee J.-J., Lee T.-I., Chiang C.-Y.

International Journal of Tuberculosis and Lung Disease. 20 (3) (pp 376-382), 2016. Date of Publication: 01 Mar 2016.

AN: 20160148052

SETTING: The Bureau of National Health Insurance (NHI) has implemented a pay-for-performance (p4p) programme for diabetes mellitus (DM) in Taiwan. OBJECTIVE : To investigate whether patients with DM enrolled in the p4p programme (DM-p4p) are less likely to develop tuberculosis (TB) and whether they have a better outcome than patients with DMnot enrolled in the p4p programme (DM-non-p4p) if they do develop TB. DESIGN: A random sample of 79 471 DM-p4p, 100 000 DM-non-p4p and 100 000 non-diabetic patients (non-DM) was obtained from the 2008-2009 NHI database, and the patients were matched with the National TB Registry to determine whether they had developed TB by the end of 2010. RESULTS : The average annual incidence of TB was respectively 259.9 (95%CI 230.2-293.4), 137.5 (95%CI 116.4-162.5) and 74.1 (95%CI 59.0-93.0) per 100 000 population among DM-non-p4p, DM-p4p and non-DM patients. The relative risk of death over treatment success was 1.79 (95%CI 1.05-3.04) among DM-non-p4p and 1.69 (95%CI 0.84-3.40) among non-DM patients, relative to DM-p4p patients. CONCLUSIONS : Enhanced case management of DM reduced risk and improved outcomes of TB among patients with DM.

Institution

(Lo, Yang) Taiwan Centers for Disease Control, Ministry of Health and Welfare, Taipei, Taiwan (Republic of China) (Lin) Institute of Epidemiology and Preventive Medicine, National Taiwan University, Taipei, Taiwan (Republic of China)

(Bai) School of Respiratory Therapy, College of Medicine, Taipei Medical University, Taipei, Taiwan (Republic of China)

(Bai, Chiang) Division of Pulmonary Medicine, Department of Internal Medicine, Wan Fang Hospital, Taipei Medical University, 111 Hsin-Long Road, Taipei, Taiwan (Republic of China)

(Lee) Department of Internal Medicine, Tzu Chi General Hospital and Tzu Chi University, Hualien, Taiwan (Republic of China)

(Lee) Division of Endocrinology and Metabolism, Department of Internal Medicine, Wan Fang Hospital, Taipei Medical University, Taipei, Taiwan (Republic of China)

(Lee) Department of General Medicine, School of Medicine, College of Medicine, Taipei Medical University, Taipei, Taiwan (Republic of China)

(Chiang) Department of Internal Medicine, School of Medicine, College of Medicine, Taipei Medical University, Taipei, Taiwan (Republic of China)

(Chiang) International Union Against Tuberculosis and Lung Disease, Paris, France

Publisher

International Union against Tubercul. and Lung Dis.

Emtree Heading

adult; aged; article; case management; cohort analysis; controlled study; *diabetes mellitus; female; *health insurance; human; incidence; major clinical study; male; mortality; *pay-for-performance; priority journal; *risk reduction; treatment outcome; *tuberculosis; very elderly.

Link to the Ovid Full Text or citation:

Click here for full text options

9.

Incidence of tuberculosis is high in chronic kidney disease patients in South East England and drug resistance common.

Ostermann M., Palchaudhuri P., Riding A., Begum P., Milburn H.J.

Renal Failure. 38 (2) (pp 256-261), 2016. Date of Publication: 07 Feb 2016.

AN: 20160022470

The risk of tuberculosis (TB) is significantly increased in chronic kidney disease (CKD). Data on TB in CKD in the UK are sparse; most information stems from countries with high background prevalence. The aim of this study was to estimate the incidence of TB in CKD patients in South East London and to describe the epidemiology, treatment, and outcome. CKD patients with TB between 1994 and 2010 were identified retrospectively. Data were collected on type of renal replacement therapy, the method of TB diagnosis, disease site, treatment regimens, and risk factors. Forty patients were identified of whom 67.5% had CKD stages IV-V. Sixty-five percent were from non-UK born ethnic minorities. Median time from diagnosis of CKD to TB development was 12 months (range 0-192 months). Cumulative incidence of TB was 1267/100,000 [95% confidence interval (CI): 630-1904; 85 x background UK rate] in hemodialysis patients; 398/100,000 (95% CI: 80-1160; 26 x background UK rate) in peritoneal dialysis; and 522/100,000 (CI: 137-909; 35 x background UK rate) in transplant recipients. Sixty-three percent of patients had pulmonary TB and 25% of patients with culture-positive TB had resistant isolates. Fifty percent of patients were immunosuppressed due to drugs, diabetes, and/or retroviral disease. Treatment regimens were according to recent national guidance in 73% of cases. Seventy-six percent of patients experienced side effects. Greater awareness of risk factors, drug resistance, treatment regimens, and potential side effects is needed.

Institution

(Ostermann) Department of Renal Medicine, Guys and St Thomas NHS Foundation Trust, London, United Kingdom (Ostermann, Milburn) Guys and St Thomas NHS Foundation Trust, Kings College London, Great Maze Pond, London SE1 9RT, United Kingdom

(Palchaudhuri, Riding, Begum, Milburn) Department of Respiratory Medicine, Guys and St Thomas NHS Foundation Trust, London, United Kingdom

Publisher

Taylor and Francis Ltd

Emtree Heading

adult; anorexia; article; atypical mycobacteriosis; *chronic kidney disease; clinical article; cohort analysis; female; fever; graft recipient; hemodialysis patient; hemoptysis; human; hyperparathyroidism/dt [Drug Therapy]; hyperphosphatasemia/dt [Drug Therapy]; immunocompromized patient; incidence; interferon gamma release assay; kidney transplantation; male; peritoneal dialysis; priority journal; renal replacement therapy; retrospective study; thorax radiography; treatment duration; treatment outcome; *tuberculosis/di [Diagnosis]; *tuberculosis/dr [Drug Resistance]; *tuberculosis/ep [Epidemiology]; United Kingdom; unspecified side effect/si [Side Effect]; vitamin blood level; vitamin supplementation; weight reduction; cycloserine; ethambutol; immunosuppressive agent; isoniazid; phosphate binding agent/dt [Drug Therapy]; pyrazinamide; streptomycin; *tuberculostatic agent/ae [Adverse Drug Reaction]; vitamin D.

Link to the Ovid Full Text or citation:

Click here for full text options

10.

Diabetes is associated with worse clinical presentation in tuberculosis patients from Brazil: A retrospective cohort study.

Gil-Santana L., Almeida J.L., Oliveira C.A.M., Hickson L.S., Daltro C., Castro S., Kornfeld H., Netto E.M., Andrade B.B.

PLoS ONE. 11 (1) (no pagination), 2016. Article Number: e0146876. Date of Publication: 11 Jan 2016.

AN: 20160054999

Background. The rising prevalence of diabetes mellitus (DM) worldwide, especially in developing countries, and the persistence of tuberculosis (TB) as a major public health issue in these same regions, emphasize the importance of investigating this association. Here, we compared the clinical profile and disease outcomes of TB patients with or without coincident DM in a TB reference center in Brazil. Methods. We performed a retrospective analysis of a TB patient cohort (treatment naive) of 408 individuals recruited at a TB primary care center in Brazil between 2004 and 2010. Data on diagnosis of TB and DM were used to define the groups. The study groups were compared with regard to TB disease presentation at diagnosis as well as to clinical outcomes such as cure and mortality rates upon anti-tuberculosis therapy (ATT) initiation. A composite score utilizing clinical, radiological and microbiological parameters was used to compare TB severity between the groups. Results. DM patients were older than non-diabetic TB patients. In addition, diabetic individuals more frequently presented with cough, night sweats, hemoptysis and malaise than those without DM. The overall pattern of lung lesions assessed by chest radiographic examination was similar between the groups. Compared to non-diabetic patients, those with TB-diabetes exhibited positive acid-fast bacilli in sputum samples more frequently at diagnosis and at 30 days after ATT initiation. Notably, higher values of the TB severity score were significantly associated with TB-diabetes comorbidity after adjustment for confounding factors. Moreover, during ATT, diabetic patients required more frequent transfers to TB reference hospitals for complex clinical management. Nevertheless, overall mortality and cure rates were indistinguishable between the study groups. Conclusions. These findings reinforce the idea that diabetes negatively impacts pulmonary TB severity. Our study argues for the systematic screening for DM in TB reference centers in endemic areas.

Institution

(Gil-Santana, Almeida, Andrade) Unidade de Medicina Investigativa, Laboratorio Integrado de Microbiologia e Imunorregulacao, Centro de Pesquisas Goncalo Moniz, Fundacao Oswaldo Cruz, Salvador, Bahia, Brazil (Gil-Santana, Almeida, Andrade) School of Medicine, Faculdades de Tecnologia e Ciencias, Salvador, Bahia, Brazil

(Gil-Santana, Almeida, Andrade) Multinational Organization Network Sponsoring Translational and Epidemiological Research (MONSTER), Fundacao Jose Silveira, Salvador, Brazil

(Oliveira, Hickson, Castro, Netto, Andrade) Research Center, Instituto Brasileiro para Investigacao da Tuberculose (IBIT), Fundacao Jose Silveira, Salvador, Bahia, Brazil

(Daltro, Netto) Hospital Universitario Pro. Edgard Santos, Universidade Federal da Bahia, Salvador, Bahia, Brazil

(Kornfeld) Department of Medicine, University of Massachusetts Medical School, Worcester, MA, United States

Publisher

Public Library of Science

Emtree Heading

acid fast bacterium; adult; alcoholism; article; body mass; Brazil; clinical feature; cohort analysis; comorbidity; controlled study; coughing; *diabetes mellitus; disease association; disease severity; extrapulmonary tuberculosis; female; hemoptysis; human; Human immunodeficiency virus infection; major clinical study; malaise; male; mortality; Mycobacterium tuberculosis; neoplasm; night sweat; outcome assessment; prevalence; primary medical care; retrospective study; scoring system; sex ratio; smoking; thorax radiography; tuberculin test; *tuberculosis; illicit drug.

Link to the Ovid Full Text or citation:

Click here for full text options

11.

Survival benefit associated with early cyclosporine treatment for dermatomyositis-associated interstitial lung disease.

Go D.J., Park J.K., Kang E.H., Kwon H.M., Lee Y.J., Song Y.W., Lee E.B.

Rheumatology International. 36 (1) (pp 125-131), 2016. Date of Publication: 01 Jan 2016.

AN: 2015246394

Interstitial lung disease (ILD) is the most common cause of death in dermatomyositis (DM). Cyclosporine A (CsA) has shown to be effective in DM-associated ILD (DM-ILD). This study aimed to define the optimal time of CsA administration. A total of 47 patients with DM-ILD, who were treated with CsA at Seoul National University Hospital between January 1998 and June 2013, were enrolled. ILD was diagnosed based on typical chest high-resolution computed tomography (HRCT) findings. Patients with early and delayed CsA treatment were compared in regard to the mortality and ILD progression on HRCT. The early (n = 16) and the delayed treatment group (n = 31) did not differ in regard to baseline clinical characteristics including HRCT scores and pulmonary function. Patients with clinically amyopathic DM (CADM) were more common in the early treatment group. The mortality rate was significantly lower in the early treatment group than in the delayed treatment group (p = 0.009). The survival benefit of early CsA treatment remained significant even after adjusting for age, degree of dyspnea, CADM status, and the year of CsA treatment (hazard ratio 0.057, 95 % confidence interval 0.007-0.472). CsA stabilized disease progression on HRCT in the early treatment group (p = 0.738). Delay in CsA treatment is associated with a worse survival in patients with DM-ILD. Early CsA treatment should be considered at DM-ILD diagnosis especially in patients at a higher risk of developing a rapidly progressive ILD.

Institution

(Go, Park, Kwon, Song, Lee) Division of Rheumatology, Department of Internal Medicine, Seoul National University College of Medicine, Seoul, South Korea (Go, Park, Song) Department of Molecular Medicine and Biopharmaceutical Sciences, Graduate School of Convergence Science and Technology, Medical Research Institute, Seoul National University, Seoul, South Korea

(Kang, Lee) Division of Rheumatology, Department of Internal Medicine, Seoul National University Bundang Hospital, Seongnam, South Korea

Publisher

Springer Verlag

Emtree Heading

adult; article; clinical article; cohort analysis; *dermatomyositis; disease association; disease course; drug withdrawal; dyspnea; female; hand tremor/si [Side Effect]; high resolution computer tomography; human; infection/si [Side Effect]; *interstitial lung disease/co [Complication]; *interstitial lung disease/dt [Drug Therapy]; leukopenia/si [Side Effect]; lung function; male; middle aged; mortality; nephrotoxicity/si [Side Effect]; priority journal; rash/si [Side Effect]; retrospective study; *survival; therapy delay; thorax pain/si [Side Effect]; *time to treatment; treatment outcome; tuberculosis/si [Side Effect]; upper abdominal pain/si [Side Effect]; azathioprine/cm [Drug Comparison]; azathioprine/dt [Drug Therapy]; corticosteroid/cm [Drug Comparison]; corticosteroid/dt [Drug Therapy]; cyclophosphamide/iv [Intravenous Drug Administration]; cyclophosphamide/cm [Drug Comparison]; cyclophosphamide/dt [Drug Therapy]; *cyclosporin A/ae [Adverse Drug Reaction]; *cyclosporin A/dt [Drug Therapy]; *cyclosporin A/iv [Intravenous Drug Administration]; *cyclosporin A/po [Oral Drug Administration]; methotrexate/cm [Drug Comparison]; methotrexate/dt [Drug Therapy].

Link to the Ovid Full Text or citation:

Click here for full text options

12.

Dose response protective effect of inhaled corticosteroid on osteoporosis in patients with chronic obstructive pulmonary disease.

Kuo H.-C.

Respirology. Conference: 20th Congress of the Asian Pacific Society of Respirology Kuala Lumpur Malaysia. Conference Start: 20151203 Conference End: 20151206. Conference Publication: (var.pagings). 20 (pp 32), 2015. Date of Publication: December 2015.

AN: 72115886

Importance: Whether the use of inhaled corticosteroid (ICS) in chronic obstructive pulmonary disease (COPD) patients can protect the incidence of osteoporosis remains undetermined. Objective: to assess the incidence of osteoporosis in COPD patients with ICS use and without. Design: Retrospective cohort and population-based study Participants: From January 1996 through December 2011, from 23 million Taiwan Health Insurance database, we got newly diagnosed female patients with COPD (n = 33125, ICD-9-CM 491, 492, 496). We analysed the one million beneficiaries randomly selected from all insurant from 1997 to 2009, which has been demonstrated to be representative of the entire population. Patients were excluded if they were younger than 40 years of age, osteoporosis diagnosed prior to the diagnosis of COPD and cases of Asthma (ICD-9 CM code 493.X) before the index date. In total, 10723 patients with COPD were included in the cohort study group. Interventions: Users (n =812) and nonusers (n =9911) of ICS. Main outcome measurement: We used Cox Proportional hazards regression models to estimate hazard ratios (HRS) for osteoporosis incidence for ICS users vs non-users. Results: From 1996 to 2011 trace, the incidence rate of osteoporosis per100,000 person years is 4,395 in non-users and 2,709 in ICS users (HR: 0.73, 95% CI:0.63-084) adjusted for age, income, and medical diseases (pulmonary tuberculosis, bacterial pneumonia, bronchiectasis, pulmonary fibrosis, hypertension, and diabetes mellitus). The ICS accumulative dose is associated with lower risk for osteoporosis (0 mg ~ < 20 mg, HR: 0.80, 95% CI 0.65~0.97; >20 mg ~ < 60 mg, HR: 0.70, 95% CI 0.52~0.92; >60 mg, HR: 0.66, 95% CI 0.50~0.86; p < 0.0001). Conclusion and relevance: COPD patients with inhaled corticosteroid treatment exhibit associated with dose response protective effect for osteoporosis.

Institution

(Kuo) Kaohsiung Chang Gung Memorial Hospital, South Korea

Publisher

Blackwell Publishing

Emtree Heading

*osteoporosis; *patient; *human; *chronic obstructive lung disease; *Asian; *society; *dose response; population; ICD-9-CM; cohort analysis; income; asthma; Taiwan; diseases; lung tuberculosis; bacterial pneumonia; hazard ratio; proportional hazards model; diagnosis; data base; incidence; health insurance; risk; outcome assessment; diabetes mellitus; hypertension; bronchiectasis; lung fibrosis; female; corticosteroid therapy; ICD-9; *corticosteroid.

Link to the Ovid Full Text or citation:

Click here for full text options

13.

Diabetes mellitus and pulmonary tuberculosis-Portuguese retrospective cohort.

Da Costa J.C., Gomes M., Duarte R.

European Respiratory Journal. Conference: European Respiratory Society Annual Congress 2015 Amsterdam Netherlands. Conference Start: 20150926 Conference End: 20150930. Conference Publication: ( var.pagings). 46 (no pagination), 2015. Date of Publication: 01 Sep 2015.

AN: 72108571

I: Diabetes mellitus (DM) is recognized as a risk factor to development and poor outcome on Tuberculosis (TB) patients. The goal of this study was to evaluate the association of DM and pulmonary TB in Portugal. M: Retrospective cohort study. National database was revised from 2008 to 2013. All patients with pulmonary TB reported were included. A global evaluation of the prevalence of DM on TB patients, presentation features and main TB treatment outcomes was performed. The trend of DM prevalence from 2008-13 was analysed. Statistical analysis was performed using IBM SPSS version 21; a significance level was defined to p<0.05. T student and Chi-square tests were used. R: Sample size: 11771 TB cases (10615 naive TB cases and 1156 retreatment cases). The prevalence of DM was 6.0%. There was a male predominance on DM group (DMg) and non-DM group (nDM) (71.0 vs 70.4%, p=0.725). The mean age was 60.5 (+/-15.2) on DMg and 46.1 (+/-17.3) years on nDM (p<0.001). There was no difference on the proportion of patients with retreatments on both groups (10.8% DMg vs 9.8% nDM, p=0.370), neither in the drug resistance profiles. There was a trend to severe treatment toxicity on DMg (4.6% vs 3.1%, p=0.06). Diabetic patients had more unsuccessful outcomes (treatment failure and death), p<0.001. From 2008 to 2013, DM prevalence on TB patients varied from 5.4% to 6.9%. D: DM patients had worse TB outcomes. This is of particular relevance since population is getting older and DM is increasing its prevalence.

Publisher

European Respiratory Society

Emtree Heading

*Portuguese (citizen); *human; *lung tuberculosis; *diabetes mellitus; *European; *society; patient; prevalence; treatment outcome; Portugal; statistical analysis; population; male; data base; risk factor; treatment failure; tuberculosis; retreatment; cohort analysis; chi square test; sample size; drug resistance; toxicity; diabetic patient; student; death; data analysis software.

Link to the Ovid Full Text or citation:

Click here for full text options

14.

Risk factors for multidrug-resistant tuberculosis among patients with pulmonary tuberculosis at the central chest institute of Thailand.

Chuchottaworn C., Thanachartwet V., Sangsayunh P., Than T.Z.M., Sahassananda D., Surabotsophon M., Desakorn V.

PLoS ONE. 10 (10) (no pagination), 2015. Article Number: e0139986. Date of Publication: 07 Oct 2015.

AN: 2015550285

There are limited data available on the risk factors for multidrug-resistant tuberculosis (MDR-TB). Therefore, we here conducted a retrospective matched case-control study among adults with pulmonary TB who received treatment at the Central Chest Institute of Thailand (CCIT) between January 2007 and December 2013, in order to determine the risk factors associated with MDR-TB among patients with pulmonary TB. We identified 145 patients with pulmonary MDR-TB (cases) and 145 patients with drug-sensitive pulmonary TB (controls). Multivariate analysis identified the independent risk factors for MDR-TB as follows: (1) > 2 episodes of prior pulmonary TB (odds ratio [OR] 39.72, 95% confidence interval (95% CI) 7.86-200.66), (2) duration of illness > 60 days (OR 3.08, 95% CI 1.52 -6.22), (3) sputum acid fast bacilli smear 3+ (OR 13.09, 95% CI 4.64-36.91), (4) presence of lung cavities (OR 3.82, 95% CI 1.89-7.73), and (5) presence of pleural effusion (OR 2.75, 95% CI 1.06-7.16). Prior pulmonary TB management with a non-category I regimen (P = 0.012) and having treatment failure or default as treatment outcomes (P = 0.036) were observed in a higher proportion among patients with MDR-TB. Particular characteristics of lung cavities, including the maximum diameter > 30 mm (P < 0.001), the number of cavities > 3 (P = 0.001), bilateral involvement (P < 0.001), and > 2 lung zones involved (P = 0.001) were more commonly observed in patients with MDR-TB. In conclusion, these clinical factors and chest radiographic findings associated with MDR-TB among patients with pulmonary TB may help physicians to provide proper management of cases for prevention of the development and spread of MDR-TB in future.

Institution

(Chuchottaworn, Sangsayunh) Division of Respiratory Medicine, Central Chest Institute of Thailand (CCIT), Nonthaburi, Thailand (Thanachartwet, Than, Desakorn) Department of Clinical Tropical Medicine, Faculty of Tropical Medicine, Mahidol University, Bangkok, Thailand

(Sahassananda) Information Technology Unit, Faculty of Tropical Medicine, Mahidol University, Bangkok, Thailand

(Surabotsophon) Division of Pulmonary and Critical Care Medicine, Ramkhamhaeng Hospital, Bangkok, Thailand

Publisher

Public Library of Science

Emtree Heading

acid fast bacterium; adult; article; atelectasis/di [Diagnosis]; cardiovascular disease; cohort analysis; controlled study; coughing; diabetes mellitus; disease duration; female; hemoptysis; human; Human immunodeficiency virus infection; lung disease; lung nodule; *lung tuberculosis/dr [Drug Resistance]; *lung tuberculosis/dt [Drug Therapy]; major clinical study; male; microbiological examination; mortality; *multidrug resistant tuberculosis/dr [Drug Resistance]; *multidrug resistant tuberculosis/dt [Drug Therapy]; Mycobacterium tuberculosis; pleura effusion; prevalence; retrospective study; risk factor; short course therapy; sputum smear; Thailand; thorax radiography; treatment failure; treatment outcome; ethambutol/dt [Drug Therapy]; isoniazid/dt [Drug Therapy]; pyrazinamide/dt [Drug Therapy]; rifampicin/dt [Drug Therapy].

Link to the Ovid Full Text or citation:

Click here for full text options

15.

Trends in the incidence and causes of severe visual impairment and blindness in children from Israel.

Mezer E., Chetrit A., Kalter-Leibovici O., Kinori M., Ben-Zion I., Wygnanski-Jaffe T.

Journal of AAPOS. 19 (3) (pp 260-265e1), 2015. Date of Publication: 01 Jun 2015.

AN: 2015504062

Purpose To describe trends in the incidence and causes of legal childhood blindness in Israel, one of the few countries worldwide that maintain a national registry of the blind. Methods We performed a historical cohort study of annual reports of the National Registry of the Blind (NRB) between 1999 and 2013. All data regarding demographic information, year of registration and cause of blindness of children 0-18 years of age registered for blind certification were obtained from the annual reports of the NRB. Causes of legal blindness analyzed were optic atrophy, retinitis pigmentosa, retinopathy of prematurity (ROP), albinism, other retinal disorders, cataract, and glaucoma. The main outcome measure was the incidence of new cases of certified legal blindness. Results The incidence of newly registered legally blind children in Israel almost halved from 7.7 per 100,000 in 1999 to 3.1 per 100,000 in 2013. The decline was mainly attributable to a decreased incidence of blindness resulting from retinitis pigmentosa and ROP. The incidence of registered cases due to cerebral visual impairment increased. Conclusions During the past decade the incidence of severe childhood visual impairment and blindness declined in Israel. A continuous decline in consanguineous marriages among the Jewish and Arab populations in Israel may have contributed to the decrease in the rate of vision loss due to retinitis pigmentosa in children.

Author NameID

Mezer, Eedy; ORCID: <http://orcid.org/0000-0002-2818-7538>

Institution

(Mezer) Department of Ophthalmology, Rambam Health Care Campus, Haifa, Israel (Mezer) Ruth and Bruce Rappaport Faculty of Medicine, Technion-Israel Institute of Technology, Haifa, Israel

(Chetrit, Kalter-Leibovici) Unit of Cardiovascular Epidemiology, Gertner Institute for Epidemiology and Health Policy Research, Tel Hashomer, Israel

(Kalter-Leibovici, Kinori, Ben-Zion, Wygnanski-Jaffe) Sackler Faculty of Medicine, Tel Aviv University, Tel Aviv, Israel

(Kinori, Ben-Zion, Wygnanski-Jaffe) Goldschleger Eye Institute, Sheba Medical Center, Tel Hashomer, Israel

Publisher

Mosby Inc.

Emtree Heading

adolescent; adult; age related macular degeneration; albinism; amblyopia; anophthalmia; article; *blindness/co [Complication]; *blindness/ep [Epidemiology]; *blindness/et [Etiology]; cataract; child; *childhood disease/co [Complication]; *childhood disease/ep [Epidemiology]; *childhood disease/et [Etiology]; cohort analysis; congenital cataract/cn [Congenital Disorder]; congenital glaucoma/cn [Congenital Disorder]; cornea opacity; demography; diabetic retinopathy; disease severity; female; glaucoma; human; incidence; infant; Israel; keratoconus; lung tuberculosis; major clinical study; male; microphthalmia; myopic maculopathy; newborn; nystagmus; optic nerve atrophy; outcome assessment; primary glaucoma; priority journal; retina detachment; retina disease; retinitis pigmentosa; retrolental fibroplasia; secondary glaucoma; senile cataract; uveitis; *visual impairment/co [Complication]; *visual impairment/ep [Epidemiology]; *visual impairment/et [Etiology]; vitreous hemorrhage.

Link to the Ovid Full Text or citation:

Click here for full text options

16.

Years lived with disability (YLDs) for 1160 sequelae of 289 diseases and injuries 1990-2010: A systematic analysis for the Global Burden of Disease Study 2010.

Vos T., Flaxman A.D., Naghavi M., Lozano R., Michaud C., Ezzati M., Shibuya K., Salomon J.A., Abdalla S., Aboyans V., Abraham J., Ackerman I., Aggarwal R., Ahn S.Y., Ali M.K., Alvarado M., Anderson H.R., Anderson L.M., Andrews K.G., Atkinson C., Baddour L.M., Bahalim A.N., Barker-Collo S., Barrero L.H., Bartels D.H., Basanez M.-G., Baxter A., Bell M.L., Benjamin E.J., Bennett D., Bernabe E., Bhalla K., Bhandari B., Bikbov B., Abdulhak A.B., Birbeck G., Black J.A., Blencowe H., Blore J.D., Blyth F., Bolliger I., Bonaventure A., Boufous S., Bourne R., Boussinesq M., Braithwaite T., Brayne C., Bridgett L., Brooker S., Brooks P., Brugha T.S., Bryan-Hancock C., Bucello C., Buchbinder R., Buckle G., Budke C.M., Burch M., Burney P., Burstein R., Calabria B., Campbell B., Canter C.E., Carabin H., Carapetis J., Carmona L., Cella C., Charlson F., Chen H., Cheng A.T.-A., Chou D., Chugh S.S., Coffeng L.E., Colan S.D., Colquhoun S., Colson K.E., Condon J., Connor M.D., Cooper L.T., Corriere M., Cortinovis M., De Vaccaro K.C., Couser W., Cowie B.C., Criqui M.H., Cross M., Dabhadkar K.C., Dahiya M., Dahodwala N., Damsere-Derry J., Danaei G., Davis A., De Leo D., Degenhardt L., Dellavalle R., Delossantos A., Denenberg J., Derrett S., Des Jarlais D.C., Dharmaratne S.D., Dherani M., Diaz-Torne C., Dolk H., Dorsey E.R., Driscoll T., Duber H., Ebel B., Edmond K., Elbaz A., Ali S.E., Erskine H., Erwin P.J., Espindola P., Ewoigbokhan S.E., Farzadfar F., Feigin V., Felson D.T., Ferrari A., Ferri C.P., Fevre E.M., Finucane M.M., Flaxman S., Flood L., Foreman K., Forouzanfar M.H., Fowkes F.G.R., Franklin R., Fransen M., Freeman M.K., Gabbe B.J., Gabriel S.E., Gakidou E., Ganatra H.A., Garcia B., Gaspari F., Gillum R.F., Gmel G., Gonzalez-Medina D., Gosselin R., Grainger R., Grant B., Groeger J., Guillemin F., Gunnell D., Gupta R., Haagsma J., Hagan H., Halasa Y.A., Hall W., Haring D., Haro J.M., Harrison J.E., Havmoeller R., Hay R.J., Higashi H., Hill C., Hoen B., Hoffman H., Hotez P.J., Hoy D., Huang J.J., Ibeanusi S.E., Jacobsen K.H., James S.L., Jarvis D., Jasrasaria R., Jayaraman S., Johns N., Jonas J.B., Karthikeyan G., Kassebaum N., Kawakami N., Keren A., Khoo J.-P., King C.H., Knowlton L.M., Kobusingye O., Koranteng A., Krishnamurthi R., Laden F., Lalloo R., Laslett L.L., Lathlean T., Leasher J.L., Lee Y.Y., Leigh J., Levinson D., Lim S.S., Limb E., Lin J.K., Lipnick M., Lipshultz S.E., Liu W., Loane M., Ohno S.L., Lyons R., Ma J., Mabweijano J., MacIntyre M.F., Malekzadeh R., Mallinger L., Manivannan S., Marcenes W., March L., Margolis D.J., Marks G.B., Marks R., Matsumori A., Matzopoulos R., Mayosi B.M., McAnulty J.H., McDermott M.M., McGill N., McGrath J., Medina-Mora M.E., Meltzer M., Mensah G.A., Merriman T.R., Meyer A.-C., Miglioli V., Miller M., Miller T.R., Mitchell P.B., Mock C., Mocumbi A.O., Moffitt T.E., Mokdad A.A., Monasta L., Montico M., Moran A., Morawska L., Mori R., Murdoch M.E., Mwaniki M.K., Naidoo K., Nair M.N., Naldi L., Narayan K.M.V., Nelson P.K., Nelson R.G., Nevitt M.C., Newton C.R., Nolte S., Norman P., Norman R., O'Donnell M., O'Hanlon S., Olives C., Omer S.B., Ortblad K., Osborne R., Ozgediz D., Page A., Pahari B., Pandian J.D., Rivero A.P., Patten S.B., Pearce N., Padilla R.P., Perez-Ruiz F., Perico N., Pesudovs K., Phillips D., Phillips M.R., Pierce K., Pion S., Polanczyk G.V., Polinder S., Pope III C.A., Popova S., Porrini E., Pourmalek F., Prince M., Pullan R.L., Ramaiah K.D., Ranganathan D., Razavi H., Regan M., Rehm J.T., Rein D.B., Remuzzi G., Richardson K., Rivara F.P., Roberts T., Robinson C., De Leon F.R., Ronfani L., Room R., Rosenfeld L.C., Rushton L., Sacco R.L., Saha S., Sampson U., Sanchez-Riera L., Sanman E., Schwebel D.C., Scott J.G., Segui-Gomez M., Shahraz S., Shepard D.S., Shin H., Shivakoti R., Singh D., Singh G.M., Singh J.A., Singleton J., Sleet D.A., Sliwa K., Smith E., Smith J.L., Stapelberg N.J.C., Steer A., Steiner T., Stolk W.A., Stovner L.J., Sudfeld C., Syed S., Tamburlini G., Tavakkoli M., Taylor H.R., Taylor J.A., Taylor W.J., Thomas B., Thomson W.M., Thurston G.D., Tleyjeh I.M., Tonelli M., Towbin J.A., Truelsen T., Tsilimbaris M.K., Ubeda C., Undurraga E.A., Van Der Werf M.J., Van Os J., Vavilala M.S., Venketasubramanian N., Wang M., Wang W., Watt K., Weatherall D.J., Weinstock M.A., Weintraub R., Weisskopf M.G., Weissman M.M., White R.A., Whiteford H., Wiebe N., Wiersma S.T., Wilkinson J.D., Williams H.C., Williams S.R.M., Witt E., Wolfe F., Woolf A.D., Wulf S., Yeh P.-H., Zaidi A.K.M., Zheng Z.-J., Zonies D., Lopez A.D., Murray C.J.L., Moradi-Lakeh M.

The Lancet. 380 (9859) (pp 2163-2196), 2012. Date of Publication: 2012.

AN: 2012732549

Background Non-fatal health outcomes from diseases and injuries are a crucial consideration in the promotion and monitoring of individual and population health. The Global Burden of Disease (GBD) studies done in 1990 and 2000 have been the only studies to quantify non-fatal health outcomes across an exhaustive set of disorders at the global and regional level. Neither effort quantified uncertainty in prevalence or years lived with disability (YLDs). Methods Of the 291 diseases and injuries in the GBD cause list, 289 cause disability. For 1160 sequelae of the 289 diseases and injuries, we undertook a systematic analysis of prevalence, incidence, remission, duration, and excess mortality. Sources included published studies, case notification, population-based cancer registries, other disease registries, antenatal clinic serosurveillance, hospital discharge data, ambulatory care data, household surveys, other surveys, and cohort studies. For most sequelae, we used a Bayesian meta-regression method, DisMod-MR, designed to address key limitations in descriptive epidemiological data, including missing data, inconsistency, and large methodological variation between data sources. For some disorders, we used natural history models, geospatial models, back-calculation models (models calculating incidence from population mortality rates and case fatality); or registration completeness models; (models adjusting for incomplete registration with health-system access and other covariates). Disability weights for 220 unique health states were used to capture the severity of health loss. YLDs by cause at age, sex, country, and year levels were adjusted for comorbidity with simulation methods. We included uncertainty estimates at all stages of the analysis. Findings Global prevalence for all ages combined in 2010 across the 1160 sequelae ranged from fewer than one case per 1 million people to 350 000 cases per 1 million people. Prevalence and severity of health loss were weakly correlated (correlation coefficient -0 37). In 2010, there were 777 million YLDs from all causes, up from 583 million in 1990. The main contributors to global YLDs were mental and behavioural disorders, musculoskeletal disorders, and diabetes or endocrine diseases. The leading specific causes of YLDs were much the same in 2010 as they were in 1990: low back pain, major depressive disorder, iron-deficiency anaemia, neck pain, chronic obstructive pulmonary disease, anxiety disorders, migraine, diabetes, and falls. Age-specific prevalence of YLDs increased with age in all regions and has decreased slightly from 1990 to 2010. Regional patterns of the leading causes of YLDs were more similar compared with years of life lost due to premature mortality. Neglected tropical diseases, HIV/AIDS, tuberculosis, malaria, and anaemia were important causes of YLDs in sub-Saharan Africa. Interpretation Rates of YLDs per 100 000 people have remained largely constant over time but rise steadily with age. Population growth and ageing have increased YLD numbers and crude rates over the past two decades. Prevalences of the most common causes of YLDs, such as mental and behavioural disorders and musculoskeletal disorders, have not decreased. Health systems will need to address the needs of the rising numbers of individuals with a range of disorders that largely cause disability but not mortality. Quantification of the burden of non-fatal health outcomes will be crucial to understand how well health systems are responding to these challenges. Effective and affordable strategies to deal with this rising burden are an urgent priority for health systems in most parts of the world.

Institution

(Baxter, Erskine, Ferrari, Keren, Saha, Whiteford) Queensland Centre for Mental Health Research, Australia (Vos, Blore, Charlson, Hay, Leasher, Norman, Page, Lopez) School Population of Healtho, Australia

(Ganatra, Halasa, Patten) Centre for Clinical Research, Australia

(McGill) Queensland Brain Institute, University of Queensland, Brisbane, QLD, Australia

(Flaxman, Naghavi, Lozano, Ahn, Alvarado, Andrews, Atkinson, Bolliger, Burstein, Campbell, Colson, Delossantos, Dharmaratne, Forouzanfar, Fransen, Gabriel, Gmel, Hall, Haro, Jarvis, Jayaraman, Levinson, Loane, Mabweijano, Malekzadeh, Moffitt, Nair, Ortblad, Phillips, Pierce, Ranganathan, Roberts, Rosenfeld, Sanman, Wang, Wulf, Murray) Institute for Health Metrics and Evaluation, University of Washington, 2301 Fifth Avenue, Seattle, WA 98121, United States

(Anderson) Department of Epidemiology, School of Public Health, United States

(Karthikeyan) Department of Anesthesiology and Pain Medicine, United States

(Couser, Duber, Ebel, Mitchell, Olives, Rivara, Vavilala) University of Washington, Seattle, WA, United States

(Michaud) China Medical Board, Boston, MA, United States

(Ezzati) MRCHPA Centre for Environment and Health, Department of Epidemiology and Biostatistics, School of Public Health, United Kingdom

(Basanez, Burney, Foreman, James, O'Hanlon, Rushton) Imperial College London, London, United Kingdom

(Shibuya) Department of Global Health Policy, Japan

(Kassebaum) University of Tokyo, Tokyo, Japan

(Finucane) Department of Biostatistics, United States

(Salomon) School of Public Health, United States

(Danaei, Krishnamurthi, Limb, Miglioli, Singh, Sudfeld, Tavakkoli, Weisskopf, White) 1Department of Epidemiology, United States

(Lipshultz) Harvard Humanitarian Initiative, United States

(King) Boston Childrenaes Hospital, United States

(Colan) Brigham and Womenaes Hospital, United States

(Jasrasaria) Harvard Medical School, Harvard University, Boston, MA, United States

(Bartels) Sudanese Public Health Consultancy Group, Sudan

(Abdalla) Department of Cardiology, Dupuytren University Hospital, Limoges, France

(Aboyans) University of Texas, San Antonio, TX, United States

(Abraham) Centre for International Child Health, Australia

(Steer) Department of Pediatrics, Australia

(Weintraub) Centre for Health Policy, Programs and Economics, Australia

(Degenhardt) School of Population Health, Australia

(Room) University of Melbourne, Melbourne, VIC, Australia

(Ackerman, Brooks, Marks, Taylor) Sanjay Gandhi Postgraduate Institute of Medical Sciences, Lucknow, India

(Aggarwal) Schools of Public Health and Medicine, United States

(Omer) Emory University, Atlanta, GA, United States

(Ali) St Georgeaes University of London, London, United Kingdom

(Dabhadkar, Gakidou, Narayan) Mayo Clinic, Rochester, MN, United States

(Anderson, Lim) Independent Consultant, Geneva, Switzerland

(Gunnell) University of Auckland, Auckland, New Zealand

(Baddour, Erwin, Gabbe) Department of Industrial Engineering, School of Engineering, Pontificia Universidad Javeriana, Bogota, Colombia

(Bahalim) Global Partners in Anesthesia and Surgery, United States

(Barker-Collo) Yale University, New Haven, CT, United States

(Barrero) School of Medicine, United States

(Ozgediz) Boston University, Boston, MA, United States

(Bell, Hoy) Clinical Trial Service Unit and Epidemiological Studies Unit, United Kingdom

(Felson) University of Oxford, Oxford, United Kingdom

(Benjamin) Dental Institute, United Kingdom

(Bennett) Hospital NHS Trust, United Kingdom

(Bernabe) Institute of Psychiatry, Kingaes College London, London, United Kingdom

(Havmoeller) Institute of Dentistry, United Kingdom

(Prince) Queen Mary University of London, London, United Kingdom

(Dahiya) Moscow State University of Medicine and Dentistry, Research Institute of Transplantology and Artificial Organs, Moscow, Russian Federation

(Bhandari) King Fahad Medical City, Riyadh, Saudi Arabia

(Manivannan) Michigan State University, East Lansing, MI, United States

(Bikbov) MRC Epidemiology Unit, Cambridge, United Kingdom

(Abdulhak) London School of Hygiene and Tropical Medicine, London, United Kingdom

(Tleyjeh) Department of Rheumatololgy, Northern Clinical School, Australia

(Birbeck) Faculty of Health Sciences, Australia

(Black) Institute of Bone and Joint Research, Australia

(Blencowe, Brooker, Edmond, Pearce, Pullan, Smith) Sydney School of Public Health, Australia

(Smith) University of Sydney, Sydney, NSW, Australia

(Marcenes, Sanchez-Riera) Inserm, Paris, France

(Driscoll, Lee) Transport and Road Safety Research, Australia

(Blyth, Bridgett, Cross, Margolis, McDermott) National Drug and Alcohol Research Centre, Australia

(Bonaventure, Elbaz) University of New South Wales, Sydney, NSW, Australia

(Boufous) Vision and Eye Research Unit, Anglia Ruskin University, Cambridge, United Kingdom

(Calabria, Degenhardt, Nelson, Singleton) Institut de Recherche pour le Developpement, Martinique

(Bucello, Miller) Moorfields Eye Hospital, London, United Kingdom

(Bourne) University of Cambridge, Cambridge, United Kingdom

(Boussinesq) University of Leicester, Leicester, United Kingdom

(Pion) Flinders University, Adelaide, SA, Australia

(Braithwaite) Cabrini Institute, Malvern, VIC, Australia

(Brayne, Richardson) Department of Epidemiology and Preventive Medicine, Australia

(Brugha) Monash University, Melbourne, VIC, Australia

(Bryan-Hancock) Bloomberg School of Public Health, United States

(Flood, Haro, Laslett, Pesudovs) Johns Hopkins University, Baltimore, MD, United States

(Buchbinder) Texas AandM University, College Station, TX, United States

(Freeman) Great Ormond Street Hospital, London, United Kingdom

(Buchbinder, Hotez) Washington University, St Louis, MO, United States

(Buckle, Mallinger) University of Oklahoma, Health Sciences Center, Oklahoma City, OK, United States

(Dorsey, Shivakoti) Telethon Institute for Child Health Research, Centre for Child Health Research, Australia

(Budke) University of Western Australia, Perth, WA, Australia

(Burch) Universidad Camilo Jose Cela, Villanueva de la Canada, Spain

(Canter) Mario Negri Institute for Pharmacological Research, Bergamo, Italy

(Carabin) National Institute of Environmental Health Sciences, Research Triangle Park, NC, United States

(Carapetis) Institute of Biomedical Sciences, Academia Sinica, Taipei, Taiwan (Republic of China)

(Norman) Heart Institute, United States

(Carmona) Cedars-Sinai Medical Center, Los Angeles, CA, United States

(Cella, Cortinovis, Garcia, Meyer, Perico, Remuzzi) Department of Public Health, United States

(Chen) Erasmus MC, University Medical Center Rotterdam, Rotterdam, Netherlands

(Cheng) Menzies School of Health Research, Darwin, NT, Australia

(Harrison) National Health Services, Fife, Edinburgh, United Kingdom

(Chugh) University of Edinburgh, Edinburgh, United Kingdom

(Polinder) University of the Witwatersrand, Johannesburg, South Africa

(Coffeng, Gupta, Stolk) Loyola University Medical School, Chicago, IL, United States

(Colquhoun, Condon) School of Public Health Sciences, Wake Forest University, Winston-Salem, NC, United States

(Connor, Fowkes) Hospital Dr Gustavo N Collado, Puerto Chitre, Panama

(Connor, Fevre) Victorian Infectious Diseases Reference Laboratory, Melbourne, VIC, Australia

(Cooper) University of California, San Diego, San Diego, CA, United States

(Corriere) University of Pennsylvania, Philadelphia, PA, United States

(De Vaccaro) Building and Road Research Institute, Kumasi, Ghana

(Cowie) MRC Hearing and Communication Group, Manchester, United Kingdom

(Criqui, Denenberg) School of Dentistry and Oral Health, Australia

(Dahodwala, March) Population and Social Health Research Program, Australia

(Damsere-Derry) Griffith University, Brisbane, QLD, Australia

(Davis, Laden) Denver VA Medical Center, Denver, CO, United States

(Laden) University of Otago, Dunedin, New Zealand

(De Leo, Stapelberg) Beth Israel Medical Center, New York City, NY, United States

(Dellavalle) University of Peradeniya, Peradeniya, Sri Lanka

(Derrett, Gosselin, Mensah, Taylor, Thomson) University of Liverpool, Liverpool, United Kingdom

(Des Jarlais) Hospital de la Santa Creu i Sant Pau, Barcelona, Spain

(Dharmaratne) University of Ulster, Ulster, United Kingdom

(Dherani) Federal Ministry of Health, Khartoum, Sudan

(Diaz-Torne) Hospital Maciel, Montevideo, Uruguay

(Dolk, Liu) Emerald Public Health Consulting Services Ltd, Abuja, Nigeria

(Moffitt) Digestive Disease Research Center, Iran, Islamic Republic of

(Espindola, Farzadfar, Moradi-Lakeh) Tehran University of Medical Sciences, Tehran, Iran, Islamic Republic of

(Ewoigbokhan) National Institute for Stroke and Applied Neurosciences, New Zealand

(MacIntyre) Auckland Technical University, Auckland, New Zealand

(Montico) Medical School, Brazil

(Feigin) Federal University of Sao Paulo, Sao Paulo, Brazil

(Koranteng, Witt) Carnegie Mellon University, Pittsburgh, PA, United States

(Polanczyk) James Cook University, Townsville, QLD, Australia

(Ferri) Howard University, College of Medicine, Washington, DC, United States

(Watt) Addiction Info Switzerland, Lausanne, Switzerland

(Gaspari) Department of Epidemiology and Biostatistics, United States

(Gillum) University of California, San Francisco, San Francisco, CA, United States

(Nevitt) College of Medicine, SUNY Downstate Medical Center, Brooklyn, NY, United States

(Gonzalez-Medina, Lin, Merriman, Robinson) National Center for Injury Prevention and Control, United States

(Grant) Centers for Disease Control and Prevention, Atlanta, GA, United States

(Sleet) Universite de Lorraine, Nancy, France

(Wiersma) University of Bristol, Bristol, United Kingdom

(Groeger) New York University, New York City, NY, United States

(Guillemin) Brandeis University, Waltham, MA, United States

(Haagsma) Parc Sanitari Sant Joan de Deu, CIBERSAM, Universitat de Barcelona, Sant Boi de Llobregat, Spain

(Thurston) Karolinska University Hospital, Stockholm, Sweden

(Hagan) Queen Elizabeth Hospital, Adelaide, SA, Australia

(Shahraz) Universite de Franche-Comte, Besancon, France

(Shepard) Centre Hospitalier Regional, Universitaire de Besancon, Besancon, France

(Undurraga) National Institute on Deafness and Other Communication Disorders, United States

(Haring) National Institute of Diabetes and Digestive and Kidney Diseases, National Institutes of Health, Bethesda, MD, United States

(Harrison) National School of Tropical Medicine, Baylor College of Medicine, Waco, TX, United States

(Higashi) University of Port Harcourt, Port Harcourt, Nigeria

(Hill) George Mason University, Fairfax, VA, United States

(Hill) Department of Ophthalmology, Medical Faculty Mannheim, Ruprecht Karls University, Heidelberg, Germany

(Hoen) All India Institute of Medical Sciences, New Delhi, India

(Nelson) Department of Cardiology, Hebrew University Hadassah Medical School, Jerusalem, Israel

(Hoffman) Case Western Reserve University, Cleveland, OH, United States

(Huang) School of Public Health, Makerere University, Kampala, Uganda

(Ibeanusi, Johns) University of South Africa, Johannesburg, South Africa

(Jonas) Kwame Nkrumah University of Science and Technology, Kumasi, Ghana

(Kawakami) University of Tasmania, TAS, Australia

(Khoo) Nova Southeastern University, Fort Lauderdale, FL, United States

(Knowlton) Ministry of Health, Jerusalem, Israel

(Knowlton) Miller School of Medicine, University of Miami, Miami, FL, United States

(Kobusingye) Swansea University, Swansea, United Kingdom

(Lalloo) Mulago Hospital, Kampala, Uganda

(Lathlean) Asian Pacific Society of Cardiology, Kyoto, Japan

(Leigh, Lipnick, Sacco, Wilkinson) Medical Research Council, Tygerberg, South Africa

(Ohno) Hatter Institute, South Africa

(Lyons) Department of Medicine, South Africa

(Marks) University of Cape Town, Cape Town, South Africa

(Matsumori) Legacy Health System, Portland, OR, United States

(Sliwa) Northwestern University Feinberg, School of Medicine, Evanston, IL, United States

(Meltzer) National Institute on Psychiatry Ramon de la Fuente, Mexico City, Mexico

(Matsumori, Matzopoulos) Thomas Jefferson University, Philadelphia, PA, United States

(Mayosi) Pacific Institute for Research and Evaluation, Calverton, MD, United States

(McAnulty) National Institute of Health, Maputo, Mozambique

(McGrath) University Eduardo Mondlane, Maputo, Mozambique

(Medina-Mora) Duke University, Durham, NC, United States

(Miller) Institute for Maternal and Child Health, IRCCS Burlo Garofolo, Trieste, Italy

(Mock) Columbia University, New York City, NY, United States

(Mock) Queensland University of Technology, Brisbane, QLD, Australia

(Mocumbi, Mokdad, Monasta, Ronfani, Tamburlini) National Center for Child Health and Development, Tokyo, Japan

(Moran, Weissman) Watford General Hospital, Watford, United Kingdom

(Morawska) Kemri-Wellcome Trust, Kilifi, Kenya

(Mori) AVRI, University of KwaZulu-Natal, Durban, South Africa

(Murdoch) Centro Studi GISED, Bergamo, Italy

(Mwaniki) Charite- Universitatsmedizin Berlin, Berlin, Germany

(Naidoo) HRB-Clinical Research Facility, National University of Ireland Galway, Galway, Ireland

(Naldi) Deakin University, Melbourne, VIC, Australia

(Newton) B P Koirala Institute of Health Sciences, Dharan, Nepal

(O'Donnell) Betty Cowan Research and Innovation Center, Ludhiana, India

(Osborne) Hospital Juan XXIII, La Paz, Bolivia

(Pahari) University of Calgary, Calgary, AB, Canada

(Pandian) Instituto Nacional de Enfermedades Respiratorias, Mexico City, Mexico

(Rivero) Hospital Universitario Cruces, Barakaldo, Spain

(Patten) Shanghai Mental Health Center, School of Medicine, China

(Padilla) School of Public Health, Shanghai Jiao Tong University, Shanghai, China

(Perez-Ruiz) Brigham Young University, Provo, UT, United States

(Phillips) Centre for Addiction and Mental Health, Toronto, ON, Canada

(Zheng) Hospital Universitario de Canarias, Tenerif, Spain

(Pope III) Faculty of Medicine, School of Population and Public Health, University of British Columbia, Vancouver, BC, Canada

(Popova) Vector Control Research Centre, Pondicherry, India

(Rehm) Center for Disease Analysis, Louisville, CO, United States

(Porrini) University of California, Berkeley, Berkeley, CA, United States

(Pourmalek) NORC, University of Chicago, Chicago, IL, United States

(Ramaiah) Complejo Hospitalario Caja De Seguro Social, Panama City, Panama

(Razavi) Centre for Alcohol Policy Research, Turning Point Alcohol and Drug Centre, Fitzroy, SA, Australia

(Regan, Sampson) Vanderbilt University, Nashville, TN, United States

(Rein, Schwebel) University of Alabama at Birmingham, Birmingham, AL, United States

(De Leon) Ministry of Interior, Madrid, Spain

(Room) Health Canada, Ottawa, ON, Canada

(Stovner) Queens Medical Center, Honolulu, HI, United States

(Singh) Department of Neuroscience, Norway

(Segui-Gomez, Taylor) Norwegian University of Science and Technology, Trondheim, Norway

(Shin) Aga Khan University, Karachi, Pakistan

(Tonelli) Drexel University, School of Public Health, Philadelphia, PA, United States

(Steiner, Tsilimbaris, Wiebe) Alberta Kidney Disease Network, Canada

(Towbin, Zaidi) University of Alberta, Edmonton, AB, Canada

(Truelsen) Cincinnati Childrenaes Hospital, Cincinnati, OH, United States

(Syed) Department of Neurology, Copenhagen University Hospital, Herlev, Denmark

(Ubeda) University of Crete Medical School, Crete, Greece

(Van Der Werf) Instituto Nacional de Epidemiologia, ANLIS, Malbran, Argentina

(Van Os) KNCV Tuberculosis Foundation, The Hague, Netherlands

(Thomas) Maastricht University Medical Centre, Maastricht, Netherlands

(Wang) National University of Singapore, Singapore

(Weinstock) Beijing Neurosurgical Institute, Capital Medical University, Beijing, China

(Grainger) Brown University, Providence, RI, United States

(Venketasubramanian, Williams) Royal Childrenaes Hospital, Critical Care and Neurosciences Theme, Murdoch Childrenaes Research Institute, Melbourne, VIC, Australia

(Williams, Woolf) University of Nottingham, Nottingham, United Kingdom

(Williams) Hollywood Orthopaedic Group, Perth, WA, Australia

(Wolfe) Arthritis Research, Wichita, KS, United States

(Weintraub) Royal Cornwall Hospital, Truro, United Kingdom

(Weintraub, Yeh) London School of Economics, London, United Kingdom

(Zonies) Landstuhl Regional Medical Center, Landstuhl, Germany

(Ma) National Center for Non-Communicable Disease Control and Prevention, China Centers for Disease Control, Beijing, China

Publisher

Lancet Publishing Group

Emtree Heading

acquired immune deficiency syndrome/co [Complication]; acquired immune deficiency syndrome/dt [Drug Therapy]; acquired immune deficiency syndrome/ep [Epidemiology]; age distribution; aging; anemia/co [Complication]; anemia/ep [Epidemiology]; antiviral therapy; anxiety disorder/co [Complication]; anxiety disorder/ep [Epidemiology]; article; behavior disorder/co [Complication]; behavior disorder/ep [Epidemiology]; chronic obstructive lung disease/co [Complication]; chronic obstructive lung disease/ep [Epidemiology]; comorbidity; diabetes mellitus/co [Complication]; diabetes mellitus/ep [Epidemiology]; *disability; disability severity; disease duration; endocrine disease/co [Complication]; endocrine disease/ep [Epidemiology]; falling; health care system; human; Human immunodeficiency virus infection/co [Complication]; Human immunodeficiency virus infection/ep [Epidemiology]; *iatrogenic disease/co [Complication]; *iatrogenic disease/ep [Epidemiology]; incidence; iron deficiency anemia/co [Complication]; iron deficiency anemia/ep [Epidemiology]; low back pain/co [Complication]; low back pain/ep [Epidemiology]; major depression/co [Complication]; major depression/ep [Epidemiology]; malaria/co [Complication]; malaria/ep [Epidemiology]; mental disease/co [Complication]; mental disease/ep [Epidemiology]; migraine/co [Complication]; migraine/ep [Epidemiology]; mortality; musculoskeletal disease/co [Complication]; musculoskeletal disease/ep [Epidemiology]; neck pain/co [Complication]; neck pain/ep [Epidemiology]; outcome assessment; population growth; prevalence; priority journal; remission; systematic review; tropical disease/co [Complication]; tropical disease/ep [Epidemiology]; tuberculosis/co [Complication]; tuberculosis/ep [Epidemiology]; *years lived with disability; anti human immunodeficiency virus agent/dt [Drug Therapy].

Link to the Ovid Full Text or citation:

Click here for full text options

17.

Outcomes of open repair of mycotic descending thoracic and thoracoabdominal aortic aneurysms.

Lau C., Gaudino M., De Biasi A.R., Munjal M., Girardi L.N.

Annals of Thoracic Surgery. 100 (5) (pp 1712-1717), 2015. Date of Publication: November 2015.

AN: 2015323381

Background The purpose of this study was to evaluate the short- and intermediate-term outcomes of open repair of mycotic thoracic and thoracoabdominal aneurysms. Contemporary surgical and perioperative techniques were utilized. Methods From November 1997 to May 2014, 14 consecutive patients underwent open repair of descending thoracic (n = 9, 64.3%) and thoracoabdominal (n = 5, 35.7%) mycotic aortic aneurysms. All procedures were performed through the left side of the chest. Infected tissue was completely debrided and excised. Aortic continuity was restored in situ with a Dacron prosthesis (Macquet Corp, Oakland, NJ). Soft tissue coverage of the prosthesis was performed when anatomy and patient condition permitted. Perioperative outcomes, intermediate-term survival, and reinfection rates were examined. Results All patients presented with either aneurysm-related symptoms or a clinical picture of sepsis. Diagnosis was confirmed utilizing computed tomography imaging. Mean age was 66 +/- 13 years, 8 patients (57.1%) were male, and mean aneurysm size was 5.9 +/- 1.3 cm. All patients were hypertensive, 3 (21.4%) had prior coronary revascularization, 7 (50%) had chronic pulmonary disease, 5 (35.7%) had diabetes mellitus, and 2 (14.3%) had end-stage renal disease requiring dialysis. Twelve patients (85.7%) had aneurysm-related pain, and 9 (64.3%) of them had contained rupture. Mean time from onset of illness to surgery was 36 days (range, 0 to 153). On preoperative blood cultures, 4 (28.6%) grew Staphylococcus aureus, 4 (28.6%) grew gram negative organisms, 2 (14.3%) grew mycobacterium, and 4 cultures (28.6%) had negative results. Empiric broad-spectrum antibiotics were initiated on all patients and adjusted based on final cultures. A majority of patients underwent repair utilizing a clamp-and-sew technique (n = 10, 71.4%); the remainder (n = 4, 28.6%) required repair under profound hypothermic circulatory arrest. After radical debridement of the infected tissue, grafts were placed in the normal anatomic position; 6 (42.9%) patients had additional soft tissue coverage, 5 (35.7%) utilizing an omental flap and 1 (7.1%), a serratus muscle flap. There was 1 in-hospital death (7.1%) secondary to ischemic bowel. Four patients (28.6%) required tracheostomy, and 1 (7.1%) had recurrent nerve injury. None of the patients incurred spinal cord injury, stroke, or new onset renal failure requiring dialysis. After surgery, all patients were given 6 weeks of intravenous antibiotics. Lifelong suppression therapy was maintained with oral antibiotics. There were no episodes of prosthetic graft infection on follow-up. Univariate analysis revealed that New York Heart Association functional class, diabetes, and preoperative renal dysfunction were preoperative risk factors for major adverse events. Mean follow-up time was 26.5 months (median 8.2; range, 1 to 142). Actuarial 5-year survival was 71%. Conclusions Open repair of mycotic descending thoracic and thoracoabdominal aortic aneurysms remains the gold standard of therapy. Aggressive intraoperative debridement with in situ prosthetic reconstruction permits a high rate of success in this very high risk cohort of patients. Lifelong antibiotic suppression therapy may prevent late prosthetic graft infection.

Institution

(Lau, Gaudino, De Biasi, Munjal, Girardi) Department of Cardiothoracic Surgery, Weill Cornell Medical College, New York, NY, United States

Publisher

Elsevier USA

Emtree Heading

adult; aged; article; blood culture; cardiac patient; cardiovascular risk; cerebrovascular accident; chronic lung disease; clinical article; cohort analysis; computer assisted tomography; deep hypothermic circulatory arrest; diabetes mellitus; end stage renal disease/th [Therapy]; female; follow up; gold standard; heart muscle revascularization; human; hypertension; male; mean arterial pressure; *mycotic aneurysm/su [Surgery]; *mycotic descending thoracic aortic aneurysm/su [Surgery]; New York Heart Association class; outcome assessment; peritoneal dialysis; positron emission tomography; priority journal; prospective study; salmonellosis; spinal cord injury; Staphylococcus aureus; sternotomy; surgical patient; *thoracoabdominal aorta aneurysm/su [Surgery]; tracheostomy; treatment duration; tuberculosis/dt [Drug Therapy]; nafcillin; oxacillin; piperacillin plus tazobactam; sultamicillin; vancomycin/dt [Drug Therapy].

Link to the Ovid Full Text or citation:

Click here for full text options

18.

Tuberculosis is frequently diagnosed within 12 months of diabetes mellitus.

Heo E.Y., Choi N.-K., Yang B.R., Koo B.K., Hwang S.-S., Lee C.-H., Kang Y.A.

International Journal of Tuberculosis and Lung Disease. 19 (9) (pp 1098-1101), 2015. Date of Publication: 01 Sep 2015.

AN: 2015299235

BACKGROUND: Evidence regarding the effects of tuberculosis (TB) screening among patients with diabetes mellitus (DM) in intermediate TB burden countries is insufficient, and the most appropriate time point for TB screening is unclear. OBJ ECT IVE : To investigate trends in TB incidence among newly diagnosed DM patients. DESIGN: A retrospective cohort study of the claims database of the Health Insurance Review and Assessment Service in Korea was performed. Participants were newly diagnosed with type 2 DM in 2009. The study outcome was TB incidence between 2009 and 2011 among participants according to duration of type 2 DM. RESULT S : A cohort of 331 601 patients with newly diagnosed type 2 DMin 2009 was identified. During the 3-year follow-up period, 1533 patients were diagnosed with TB. The estimated incidence of TB among newly diagnosed type 2 DM patients was 18/10 000 patientyears (py) (95%CI 17.5-19.4). TB incidence was 33/ 10 000 py (95%CI 30.0-35.6) in the first 6 months, and 19/10 000 py (95%CI 16.5-20.6) in the following 6-month period. CONCLUS IONS : The risk of developing TB was increased among DM patients, particularly during the first 12 months after DM diagnosis.

Institution

(Heo, Yang, Koo) Department of Internal Medicine, Boramae Medical Center, Seoul National University, Seoul Metropolitan Government, Seoul, South Korea (Choi) Medical Research Collaborating Center, College of Medicine, Seoul National University, Seoul National University Hospital, Seoul, South Korea

(Choi) Seoul National University Medical Research Center, Institute of Environmental Medicine, Seoul, South Korea

(Yang) Department of Preventive Medicine, Seoul National University College of Medicine, Seoul, South Korea

(Hwang) Department of Social and Preventive Medicine, Inha University School of Medicine, Incheon, South Korea

(Lee) Department of Internal Medicine, College of Medicine, Seoul National University, Seoul National University Hospital, Seoul, South Korea

(Kang) Department of Internal Medicine, College of Medicine, Yonsei University, Severance Hospital, 50-1 Yonsei-ro, Seodaemun-gu, Seoul 120-752, South Korea

Publisher

International Union against Tubercul. and Lung Dis.

Emtree Heading

adult; aged; article; cohort analysis; controlled study; data base; *diabetes mellitus; diabetic patient; disease duration; female; follow up; human; incidence; infection risk; major clinical study; male; non insulin dependent diabetes mellitus/dt [Drug Therapy]; outcome assessment; priority journal; retrospective study; screening; *tuberculosis/di [Diagnosis]; *tuberculosis/dt [Drug Therapy]; 2,4 thiazolidinedione derivative/dt [Drug Therapy]; alpha glucosidase inhibitor/dt [Drug Therapy]; aminosalicylic acid/dt [Drug Therapy]; cycloserine/dt [Drug Therapy]; ethambutol/dt [Drug Therapy]; insulin/dt [Drug Therapy]; isoniazid plus rifampicin/dt [Drug Therapy]; meglitinide/dt [Drug Therapy]; metformin/dt [Drug Therapy]; protionamide/dt [Drug Therapy]; pyrazinamide/dt [Drug Therapy]; sulfonylurea/dt [Drug Therapy].

Link to the Ovid Full Text or citation:

Click here for full text options

19.

Serum drug concentrations of INH and RMP predict 2-month sputum culture results in tuberculosis patients.

Mah A., Kharrat H., Ahmed R., Gao Z., Der E., Hansen E., Long R., Kunimoto D., Cooper R.

International Journal of Tuberculosis and Lung Disease. 19 (2) (pp 210-215), 2015. Date of Publication: 01 Feb 2015.

AN: 2015200884

BACKGROUND: In Alberta provincial tuberculosis (TB) clinics, serum drug concentrations (SDCs) are measured in patients with human immunodeficiency virus, diabetes mellitus or at extremes of weight, or showing slow clinical response to treatment, to guide treatment. DESIGN: A retrospective review was performed of TB cases in Northern Alberta with SDCs measured from 1998 to 2013. Adequacy of SDC was based on the maximum concentration (Cmax) achieved in serum, with rifampicin (RMP) values <8 mug/ml and isoniazid (INH) values <3 mug/ml for daily dosing and <9 mug/ml for intermittent dosing considered inadequate. Clinical variables and microbiological outcomes were then compared between the adequate and inadequate groups. RESULTS: Of 134 pulmonary TB cases with SDCs for INH and/or RMP, we found a significant increase in 2-month sputum culture positivity in the cohort with inadequate concentrations of INH compared to those with adequate INH concentrations (42.5% vs. 18.3%, P = 0.0084). A similar trend was seen in the cohort with inadequate concentrations of RMP (39% vs. 21%, P = 0.0725). CONCLUS IONS: Among our study population, low SDCs of INH and, to a lesser extent, RMP, appear to be associated with reduced sputum culture conversion after 2 months of treatment.

Institution

(Mah, Ahmed, Long, Kunimoto, Cooper) Department of Medicine, University of Alberta, Edmonton, AB, Canada (Kharrat) Faculty of Medicine and Dentistry, University of Alberta, Edmonton, AB, Canada

(Gao, Hansen) Alberta Health Services TB Program, Alberta Health Services, Edmonton, AB, Canada

(Der) Central TB Services, Alberta Health Services, Calgary, AB, Canada

Publisher

International Union against Tubercul. and Lung Dis.

Emtree Heading

adult; antibiotic sensitivity; Canada; cohort analysis; comorbidity; *drug blood level; drug intermittent therapy; drug treatment failure; female; human; Human immunodeficiency virus infection; insulin dependent diabetes mellitus; *lung tuberculosis/dt [Drug Therapy]; major clinical study; male; maximum plasma concentration; medical record review; non insulin dependent diabetes mellitus; patient compliance; prediction; priority journal; relapse; retrospective study; review; sputum culture; treatment duration; treatment outcome; ethambutol/dt [Drug Therapy]; *isoniazid/cr [Drug Concentration]; *isoniazid/dt [Drug Therapy]; pyrazinamide/dt [Drug Therapy]; *rifampicin/cr [Drug Concentration]; *rifampicin/dt [Drug Therapy].

Link to the Ovid Full Text or citation:

Click here for full text options

20.

Duration of treatment in pulmonary tuberculosis: Are international guidelines on the management of tuberculosis missing something?.

Atif M., Sulaiman S.A.S., Shafie A.A., Babar Z.U.

Public Health. 129 (6) (pp 777-782), 2015. Date of Publication: 01 Jun 2015.

AN: 2015054629

Background: Despite evidence of an association between tuberculosis (TB) treatment outcomes and the performance of national tuberculosis programmes (NTP), no study to date has rigorously documented the duration of treatment among TB patients. As such, this study was conducted to report the durations of the intensive and continuation phases of TB treatment and their predictors among new smear-positive pulmonary tuberculosis (PTB) patients in Malaysia. Study design: Descriptive, non-experimental, follow-up cohort study. Methods: This study was conducted at the Chest Clinic of Penang General Hospital between March 2010 and February 2011. The medical records and TB notification forms of all new smear-positive PTB patients, diagnosed during the study period, were reviewed to obtain sociodemographic and clinical data. Based on standard guidelines, the normal benchmarks for the durations of the intensive and continuation phases of PTB treatment were taken as two and four months, respectively. A patient in whom the clinicians decided to extend the intensive phase of treatment by >2 weeks was categorized as a case with a prolonged intensive phase. The same criterion applied for the continuation phase. Multiple logistic regression analysis was performed to find independent factors associated with the duration of TB treatment. Data were analyzed using Predictive Analysis Software Version 19.0. Results: Of the 336 patients included in this study, 261 completed the intensive phase of treatment, and 226 completed the continuation phase of treatment. The mean duration of TB treatment (n = 226) was 8.19 (standard deviation 1.65) months. Half (49.4%, 129/261) of the patients completed the intensive phase of treatment in two months, whereas only 37.6% (85/226) of the patients completed the continuation phase of treatment in four months. On multiple logistic regression analysis, being a smoker, being underweight and having a history of cough for >4 weeks at TB diagnosis were found to be predictive of a prolonged intensive phase of treatment. Diabetes mellitus and the presence of lung cavities at the start of treatment were the only predictors found for a prolonged continuation phase of treatment. Conclusions: The average durations of the intensive and continuation phases of treatment among PTB patients were longer than the targets recommended by the World Health Organization. As there are no internationally agreed criteria, it was not possible to judge how well the Malaysian NTP performed in terms of managing treatment duration among PTB patients.

Institution

(Atif) Department of Pharmacy, Islamia University of Bahawalpur, Punjab, Pakistan (Sulaiman) Discipline of Clinical Pharmacy, School of Pharmaceutical Sciences, Universiti Sains Malaysia, Penang, Malaysia

(Shafie) Discipline of Social and Administrative Pharmacy, School of Pharmaceutical Sciences, Universiti Sains Malaysia, Penang, Malaysia

(Babar) Division of Pharmacy Practice, School of Pharmacy, Faculty of Medical and Health Sciences, University of Auckland, Auckland, New Zealand

Publisher

Elsevier

Emtree Heading

adult; article; Chinese; cohort analysis; comorbidity; continuation phase; coughing; diabetes mellitus; female; human; hypertension; intensive phase; lung cavity; *lung tuberculosis/dt [Drug Therapy]; major clinical study; Malay (people); Malaysia; male; medical history; middle aged; *practice guideline; risk factor; smoking; Tamil (people); *treatment duration; treatment outcome; treatment planning; underweight; ethambutol/cb [Drug Combination]; ethambutol/dt [Drug Therapy]; isoniazid/cb [Drug Combination]; isoniazid/dt [Drug Therapy]; pyrazinamide/cb [Drug Combination]; pyrazinamide/dt [Drug Therapy]; rifampicin/cb [Drug Combination]; rifampicin/dt [Drug Therapy].

Link to the Ovid Full Text or citation:

Click here for full text options

21.

Diabetes mellitus is associated with cavities, smear grade, and multidrug-resistant tuberculosis in Georgia.

Magee M.J., Kempker R.R., Kipiani M., Gandhi N.R., Darchia L., Tukvadze N., Howards P.P., Narayan K.M.V., Blumberg H.M.

International Journal of Tuberculosis and Lung Disease. 19 (6) (pp 685-692), 2015. Date of Publication: 01 Jun 2015.

AN: 2015052742

SETTING: National tuberculosis (TB) treatment facility in the country of Georgia. OBJECTIVE: To determine the prevalence of diabetes mellitus (DM) and pre-DM among patients with TB using glycosylated-hemoglobin (HbA1c), and to estimate the association between DM and clinical characteristics and response to anti-tuberculosis treatment. DESIGN: A cohort study was conducted from 2011 to 2014 at the National Centre for TB and Lung Disease in Tbilisi. Patients aged >35 years with pulmonary TB were included. HbA1c was used to defineDM(>6.5%), pre-DM (>5.7-6.4%), and no DM (<5.7%). Interviews and medical chart abstraction were performed. Regression analyses estimated associations between DM and 1) baseline TB characteristics and 2) anti-tuberculosis treatment outcomes. RESULTS : A total of 318 newly diagnosed patients with TB were enrolled. The prevalence of DM and pre-DM was 11.6% and 16.4%, respectively. In multivariable analyses, patients with TB-DM had more cavitation (adjusted OR [aOR] 2.26), higher smear grade (aOR 2.37), and more multidrug-resistant TB (MDR-TB) (aOR 2.27) than patients without DM. The risk of poor anti-tuberculosis treatment outcomes was similar among patients with and those without DM(28.1%vs. 23.6%). CONCLUSION: DM and pre-DM were common among adults with newly diagnosed pulmonary TB in Tbilisi, Georgia, and DM was associated with more clinical symptoms, and MDR-TB, at presentation.

Institution

(Magee) Division of Epidemiology and Biostatistics, School of Public Health, Georgia State University, One Park Place NE, Atlanta, GA 30303, United States (Magee, Gandhi, Howards, Narayan, Blumberg) Department of Epidemiology and Global Health, Rollins School of Public Health, Emory University, Atlanta, GA, United States

(Kempker, Gandhi, Blumberg) Division of Infectious Diseases, Department of Medicine, Emory University School of Medicine, Atlanta, GA, United States

(Kipiani, Darchia, Tukvadze) National Center for Tuberculosis and Lung Diseases, Tbilisi, Georgia

Publisher

International Union against Tubercul. and Lung Dis.

Emtree Heading

acid fast bacterium; adult; aged; alcohol consumption; article; clinical feature; cohort analysis; controlled study; *diabetes mellitus/di [Diagnosis]; disease association; female; follow up; Georgia (republic); hemoptysis; human; impaired glucose tolerance/di [Diagnosis]; *lung cavitation; major clinical study; male; middle aged; *multidrug resistant tuberculosis/dt [Drug Therapy]; Mycobacterium tuberculosis; prevalence; priority journal; risk assessment; risk factor; smoking; sputum culture; *sputum smear; treatment outcome; hemoglobin A1c/ec [Endogenous Compound]; tuberculostatic agent/dt [Drug Therapy].

Link to the Ovid Full Text or citation:

Click here for full text options

22.

Diabetes and the risk of infections with immunomodulator therapy in inflammatory bowel diseases.

Ananthakrishnan A.N., Cagan A., Cai T., Gainer V.S., Shaw S.Y., Churchill S., Karlson E.W., Murphy S.N., Kohane I., Liao K.P.

Alimentary Pharmacology and Therapeutics. 41 (11) (pp 1141-1148), 2015. Date of Publication: 01 Jun 2015.

AN: 2015929997

Background Infections are an important concern in patients using immunosuppressive therapy for their inflammatory bowel disease (IBD). Diabetes affects nearly 10% of Americans. Whether it confers an additional risk with immunosuppression in IBD has not been examined previously. Aim To examine the association between diabetes and infections with immunomodulator use in IBD Methods Using a validated, multi-institutional IBD cohort, we identified all patients who received at least one prescription for immunomodulators (thiopurines, methotrexate). Our primary outcome was infection within 1 year of the prescription of the immunomodulator. Multivariable logistic regression adjusting for relevant confounders was used to estimate the independent association with diabetes. Results Our study included 2766 patients receiving at least one prescription for immunomodulators among whom 210 (8%) developed an infection within 1 year. Patients who developed an infection were likely to be older, have more comorbidities, more likely to have received a prescription for steroids but similar in initiation of anti-TNF therapy within that year. Only 8% of those without an infection had diabetes compared to 19% of those who developed an infection within 1 year [odds ratio (OR) 2.74, 95% confidence interval (CI) 1.88-3.98, P < 0.001]. On multivariate analysis, diabetes was independently associated with a nearly two-fold increase in risk of infections (OR: 1.80, 95% CI: 1.20-2.68). There was no increase in risk of infections with addition of anti-TNF therapy (OR: 1.14, 95% CI: 0.80-1.63). Conclusion Diabetes is an independent risk factor for infection in IBD patients using immunomodulator therapy.

Institution

(Ananthakrishnan) Division of Gastroenterology, Massachusetts General Hospital, Boston, MA, United States (Ananthakrishnan, Shaw, Karlson, Murphy, Kohane, Liao) Harvard Medical School, Boston, MA, United States

(Ananthakrishnan, Shaw, Murphy) Department of Medicine, Massachusetts General Hospital, Boston, MA, United States

(Cagan, Gainer) Research is and Computing, Partners HealthCare, Charlestown, MA, United States

(Cai) Department of Biostatistics, Harvard School of Public Health, Boston, MA, United States

(Shaw) Center for Systems Biology, Massachusetts General Hospital, Boston, MA, United States

(Churchill, Murphy, Kohane) I2b2 National Center for Biomedical Computing, Brigham and Women's Hospital, Boston, MA, United States

(Karlson, Liao) Division of Rheumatology, Allergy and Immunology, Brigham and Women's Hospital, Boston, MA, United States

(Murphy) Department of Neurology, Massachusetts General Hospital, Boston, MA, United States

(Kohane) Children's Hospital Boston, Boston, MA, United States

Publisher

Blackwell Publishing Ltd

Emtree Heading

adult; article; aspergillosis; bacteremia; cellulitis; cohort analysis; comorbidity; controlled study; Crohn disease; cytomegalovirus infection; *diabetes mellitus; disease association; Epstein Barr virus infection; female; follow up; herpes simplex; herpes zoster; human; *immunosuppressive treatment; infection/si [Side Effect]; *infection risk; *inflammatory bowel disease/dt [Drug Therapy]; influenza; lung abscess; major clinical study; male; meningitis; outcome assessment; pneumonia; population research; prescription; priority journal; sepsis; Streptococcus infection; tuberculosis; ulcerative colitis; urinary tract infection; azathioprine/ae [Adverse Drug Reaction]; azathioprine/dt [Drug Therapy]; *immunomodulating agent/ae [Adverse Drug Reaction]; *immunomodulating agent/dt [Drug Therapy]; mercaptopurine/ae [Adverse Drug Reaction]; mercaptopurine/dt [Drug Therapy]; methotrexate/ae [Adverse Drug Reaction]; methotrexate/dt [Drug Therapy].

Link to the Ovid Full Text or citation:

Click here for full text options

23.

Mortality among MDR-TB cases: Comparison with drug-susceptible tuberculosis and associated factors.

Chung-Delgado K., Guillen-Bravo S., Revilla-Montag A., Bernabe-Ortiz A.

PLoS ONE. 10 (3) (no pagination), 2015. Article Number: e0119332. Date of Publication: 19 Mar 2015.

AN: 2015861675

Background: An increase in multidrug-resistant tuberculosis (MDR-TB) cases is evident worldwide. Its management implies a complex treatment, high costs, more toxic anti-tuberculosis drug use, longer treatment time and increased treatment failure and mortality. The aims of this study were to compare mortality between MDR and drug-susceptible cases of tuberculosis, and to determine risk factors associated with mortality among MDR-TB cases. Methods and Results: A retrospective cohort study was performed using data from clinical records of the National Strategy for Prevention and Control of Tuberculosis in Lima, Peru. In the first objective, MDR-TB, compared to drug-susceptible cases, was themain exposure variable and time to death, censored at 180 days, the outcome of interest. For the second objective, different variables obtained from clinical records were assessed as potential risk factors for death among MDR-TB cases. Cox regression analysis was used to determine hazard ratios (HR) and 95%confidence intervals (95%CI). A total of 1,232 patients were analyzed: mean age 30.9 +/-14.0 years, 60.0%were males. 61 patients (5.0%) died during treatment, whereas the MDR-TB prevalence was 19.2%. MDR-TB increased the risk of death during treatment (HR = 7.5; IC95%: 4.1-13.4) when compared to presumed drug-susceptible cases after controlling for potential confounders. Education level (p = 0.01), previous TB episodes (p<0.001), diabetes history (p<0.001) and HIV infection (p = 0.04) were factors associated with mortality among MDR-TB cases. Conclusions: MDR-TB is associated with an increased risk of death during treatment. Lower education, greater number of previous TB episodes, diabetes history, and HIV infection were independently associated with mortality among MDR-TB cases. New strategies for appropriate MDR-TB detection and management should be implemented, including drug sensitivity tests, diabetes and HIV screening, as well as guarantee for a complete adherence to therapy.

Institution

(Chung-Delgado, Guillen-Bravo, Revilla-Montag, Bernabe-Ortiz) Escuela de Medicina, Universidad Peruana de Ciencias Aplicadas, Lima, Peru

Publisher

Public Library of Science

Emtree Heading

adult; article; cohort analysis; controlled study; diabetes mellitus; *drug susceptible tuberculosis; drug susceptible tuberculosis/dt [Drug Therapy]; educational status; female; high risk patient; human; Human immunodeficiency virus infection; major clinical study; male; medical history; mixed infection; *mortality; *multidrug resistant tuberculosis/dt [Drug Therapy]; outcome assessment; Peru; prevalence; retrospective study; risk assessment; *risk factor; *tuberculosis/dt [Drug Therapy]; tuberculostatic agent/dt [Drug Therapy].

Link to the Ovid Full Text or citation:

Click here for full text options

24.

Temporal trends in critical events complicating HIV infection: 1999-2010 multicentre cohort study in France.

Barbier F., Roux A., Canet E., Martel-Samb P., Aegerter P., Wolff M., Guidet B., Azoulay E.

Intensive Care Medicine. 40 (12) (pp 1906-1915), 2014. Date of Publication: 21 Nov 2014.

AN: 2014877889

Purpose: Multicentre data are limited to appraise the management and prognosis of critically ill human immunodeficiency virus (HIV)-infected patients. We sought to describe temporal trends in demographic and clinical characteristics, indications for intensive care and outcome in this patient population. Methods: We conducted a cohort study of unselected HIV-infected patients admitted between 1999 and 2010 to 34 French ICUs contributing to the CUB-Rea prospective database.

Results: We included 6,373 consecutive patients. Over the 12-year period, increases occurred in median age (39 years in 1999-2001; 47 years in 2008-2010, p < 0.0001) and prevalence of comorbidities (notably malignancies, from 6.7 to 16.4 %, p < 0.0001). Admissions for respiratory failure (39.8 % overall), shock (8.1 %) and coma (22.7 %) decreased (p < 0.0001), while those for sepsis (19.3 %) remained stable. The main final diagnoses were bacterial sepsis (24.6 %) and non-bacterial acquired immune deficiency syndrome (AIDS)-defining diseases (steady decline from 26.0 to 17.5 %, p < 0.0001). Patients increasingly received mechanical ventilation (from 42.9 to 54.0 %) and renal replacement therapy (from 9.6 to 16.8 %) (p < 0.0001), whereas vasopressor use remained stable (27.4 %). ICU readmissions increased after 2004 (p < 0.0001). ICU and hospital mortality (17.6 and 26.9 %, respectively) dropped markedly in the most severely ill patients requiring multiple life-sustaining therapies. Malignancies and chronic liver disease were heavily associated with hospital mortality by multivariate analysis, while the most common AIDS-defining complications (Pneumocystis jirovecii pneumonia, cerebral toxoplasmosis and tuberculosis) had no independent impact.

Conclusions: Progressive ageing, increasing prevalence of comorbidities (mainly malignancies), a steady decline in AIDS-related illnesses and improved benefits from life-sustaining therapies were the main temporal trends in HIV-infected patients requiring ICU admission.

Institution

(Barbier) Medical Intensive Care Unit (ICU), La Source Hospital, Orleans, France (Roux) Respiratory ICU, Georges Pompidou European Hospital, Paris, France

(Canet, Azoulay) Medical ICU, Saint-Louis Hospital, Paris, France

(Martel-Samb, Aegerter) Department of Public Health and Clinical Research, Ambroise Pare Hospital, Boulogne-Billancourt, France

(Wolff) Medical and Infectious Diseases ICU, Bichat-Claude Bernard Hospital, Paris, France

(Guidet) Medical ICU, Saint-Antoine Hospital, Paris, France

Publisher

Springer Verlag

Emtree Heading

*acquired immune deficiency syndrome/ep [Epidemiology]; acute respiratory failure/ep [Epidemiology]; adult; AIDS related complex/co [Complication]; AIDS related complex/ep [Epidemiology]; article; cardiovascular disease/ep [Epidemiology]; chronic kidney disease/ep [Epidemiology]; cohort analysis; colorectal cancer/ep [Epidemiology]; combination antiretroviral therapy; *comorbidity; *critically ill patient; diabetes mellitus/ep [Epidemiology]; female; France; hepatitis B/ep [Epidemiology]; hepatitis C/ep [Epidemiology]; highly active antiretroviral therapy; Hodgkin disease/ep [Epidemiology]; hospital admission; human; Human immunodeficiency virus infected patient; *Human immunodeficiency virus infection/ep [Epidemiology]; *Human immunodeficiency virus infection/dt [Drug Therapy]; intensive care unit; Kaposi sarcoma/ep [Epidemiology]; liver cancer/ep [Epidemiology]; liver cirrhosis/ep [Epidemiology]; lung cancer/ep [Epidemiology]; major clinical study; male; mixed infection/ep [Epidemiology]; mortality; multicenter study; nonhodgkin lymphoma/ep [Epidemiology]; outcome assessment; Pneumocystis pneumonia/co [Complication]; Pneumocystis pneumonia/ep [Epidemiology]; prevalence; respiratory tract disease/ep [Epidemiology]; toxoplasmosis/co [Complication]; toxoplasmosis/ep [Epidemiology]; tuberculosis/co [Complication]; tuberculosis/ep [Epidemiology]; antiretrovirus agent/dt [Drug Therapy].

Link to the Ovid Full Text or citation:

Click here for full text options

25.

Diabetes mellitus, smoking status, and rate of sputum culture conversion in patients with multidrug-resistant tuberculosis: a cohort study from the country of Georgia.

Magee M.J., Kempker R.R., Kipiani M., Tukvadze N., Howards P.P., Narayan K.M., Blumberg H.M.

PloS one. 9 (4) (pp e94890), 2014. Date of Publication: 2014.

AN: 24736471

INTRODUCTION: Diabetes mellitus (DM) is a risk factor for active tuberculosis (TB) but little is known about the effect of DM on culture conversion among patients with multidrug-resistant (MDR)-TB. The primary aim was to estimate the association between DM and rate of TB sputum culture conversion. A secondary objective was to estimate the association between DM and the risk of poor treatment outcomes among patients with MDR-TB. MATERIALS AND METHODS: A cohort of all adult patients starting MDR-TB treatment in the country of Georgia between 2009-2011 was followed during second-line TB therapy. Cox proportional models were used to estimate the adjusted hazard rate of sputum culture conversion. Log-binomial regression models were used to estimate the cumulative risk of poor TB treatment outcome.

RESULTS: Among 1,366 patients with sputum culture conversion information, 966 (70.7%) had culture conversion and the median time to conversion was 68 days (interquartile range 50-120). The rate of conversion was similar among patients with MDR-TB and DM (adjusted hazard ratio [aHR] 0.95, 95%CI 0.71-1.28) compared to patients with MDR-TB only. The rate of culture conversion was significantly less in patients that currently smoked (aHR 0.82, 95%CI 0.71-0.95), had low body mass index (aHR 0.71, 95%CI 0.59-0.84), second-line resistance (aHR 0.56, 95%CI 0.43-0.73), lung cavities (aHR 0.70, 95%CI 0.59-0.83) and with disseminated TB (aHR 0.75, 95%CI 0.62-0.90). The cumulative risk of poor treatment outcome was also similar among TB patients with and without DM (adjusted risk ratio [aRR] 1.03, 95%CI 0.93-1.14).

CONCLUSIONS: In adjusted analyses, DM did not impact culture conversion rates in a clinically meaningful way but smoking did.

Institution

(Magee) Department of Epidemiology, Rollins School of Public Health, Emory University, Atlanta, Georgia, United States of America; Hubert Department of Global Health, Rollins School of Public Health, Emory University, Atlanta, Georgia, United States of America (Kempker) Division of Infectious Diseases, Department of Medicine, Emory University School of Medicine, Atlanta, Georgia, United States of America

(Kipiani) National Center for Tuberculosis and Lung Diseases, Tbilisi, Georgia

(Tukvadze) National Center for Tuberculosis and Lung Diseases, Tbilisi, Georgia

(Howards) Department of Epidemiology, Rollins School of Public Health, Emory University, Atlanta, Georgia, United States of America

(Narayan) Department of Epidemiology, Rollins School of Public Health, Emory University, Atlanta, Georgia, United States of America; Hubert Department of Global Health, Rollins School of Public Health, Emory University, Atlanta, Georgia, United States of America

(Blumberg) Department of Epidemiology, Rollins School of Public Health, Emory University, Atlanta, Georgia, United States of America; Hubert Department of Global Health, Rollins School of Public Health, Emory University, Atlanta, Georgia, United States of America; Division of Infectious Diseases, Department of Medicine, Emory University School of Medicine, Atlanta, Georgia, United States of America

Emtree Heading

adolescent; adult; aged; cohort analysis; *diabetes mellitus; epidemiology; female; Georgia; human; male; *microbiology; middle aged; *smoking; sputum; treatment outcome; Tuberculosis, Multidrug-Resistant/dt [Drug Therapy]; Tuberculosis, Multidrug-Resistant/ep [Epidemiology]; Tuberculosis, Pulmonary/dt [Drug Therapy]; Tuberculosis, Pulmonary/ep [Epidemiology]; young adult; tuberculostatic agent/dt [Drug Therapy].

Link to the Ovid Full Text or citation:

Click here for full text options

26.

Predictors of pulmonary tuberculosis treatment outcomes in South Korea: a prospective cohort study, 2005-2012.

Choi H., Lee M., Chen R.Y., Kim Y., Yoon S., Joh J.S., Park S.K., Dodd L.E., Lee J., Song T., Cai Y., Goldfeder L.C., Via L.E., Carroll M.W., Barry C.E., Cho S.-N.

BMC infectious diseases. 14 (pp 360), 2014. Date of Publication: 2014.

AN: 24990578

BACKGROUND: Tuberculosis remains an important health concern in many countries. The aim of this study was to identify predictors of unfavorable outcomes at the end of treatment (EOT) and at the end of study (EOS; 40 months after EOT) in South Korea. METHODS: New or previously treated tuberculosis patients were recruited into a prospective observational cohort study at two hospitals in South Korea. To identify predictors of unfavorable outcomes at EOT and EOS, logistic regression analysis was performed.

RESULTS: The proportion of multidrug-resistant tuberculosis (MDR-TB) was 8.2% in new cases and 57.9% in previously treated cases. Of new cases, 68.6% were cured, as were 40.7% of previously treated cases. At EOT, diabetes, >3 previous TB episodes, >1 significant regimen change, and MDR-TB were significantly associated with treatment failure or death. At EOS, age >35, body-mass index (BMI) <18.5, diabetes, and MDR-TB were significantly associated with treatment failure, death, or relapse. Among cases that were cured at EOT, age >50 and a BMI <18.5 were associated with subsequent death or relapse during follow-up to EOS. Treatment interruption was associated with service sector employees or laborers, bilateral lesions on chest X-ray, and previous treatment failure or treatment interruption history.

CONCLUSIONS: Risk factors for poor treatment outcomes at EOT and EOS include both patient factors (diabetes status, age, BMI) and disease factors (history of multiple previous treatment episodes, MDR-TB). In this longitudinal, observational cohort study, diabetes mellitus and MDR-TB were risk factors for poor treatment outcomes and relapse. Measures to help ensure that the first tuberculosis treatment episode is also the last one may improve treatment outcomes.

TRIAL REGISTRATION: ClinicalTrials.gov ID: NCT00341601.

Institution

(Cho) International Tuberculosis Research Center, Changwon, Republic of Korea. raycho@yonsei.kr

Emtree Heading

adult; cohort analysis; female; human; male; middle aged; prospective study; risk factor; South Korea; treatment failure; treatment outcome; Tuberculosis, Multidrug-Resistant/dt [Drug Therapy]; Tuberculosis, Multidrug-Resistant/ep [Epidemiology]; Tuberculosis, Pulmonary/dt [Drug Therapy]; Tuberculosis, Pulmonary/ep [Epidemiology]; tuberculostatic agent/dt [Drug Therapy].

Link to the Ovid Full Text or citation:

Click here for full text options

27.

Screening for diabetes mellitus in patients diagnosed with pulmonary tuberculosis.

Pablo-Villamor M.P., Benedicto J.P., Benedicto M.T.J.U., Perez V.M.

Phillippine Journal of Internal Medicine. 52 (4) (pp 1-9), 2014. Date of Publication: 01 Oct 2014.

AN: 2015714619

Background: The epidemic of diabetes mellitus (DM) poses a threat for global tuberculosis (TB) control. Objective: This study attempts to assess the value of screening for diabetes in patients with pulmonary tuberculosis and reviews the disease burden, clinical and radiographic manifestations, rates of sputum smear positivity and time to conversion, treatment outcomes and fatality rates, in the local setting.

Methods: This is a prospective observational cohort study involving adults diagnosed with pulmonary tuberculosis at the PTSI TB DOTS out-patient clinic, regardless of sputum-smear status, from July 2011-November 2012. A diabetes screening tool was used and patients were screened for presence of DM. Treatment outcomes were also determined.

Results: Of the 38 patients enrolled, seven (18.4%, 95% confidence interval 7.7-34.3) were diagnosed with DM. This is higher than the estimated 12.9% in 2010 and 14.4% projected estimate in 2030 in our country by a report of WHO as well as in reported prevalence of DM among patients with PTB in large studies done in China (12.4%) and India (13%). There was no significant difference noted in the basic profile, clinical and radiographic presentation, sputum conversion and treatment outcomes among patients with DM and without DM who were being treated for PTB.

Conclusion: This study demonstrates the value and feasibility of screening for DM among patients with PTB. Although the findings of this study are consistent with most of previous similar studies, the estimate on the true prevalence of DM may not be very accurate because of the small sample size. Hence, a multi-center study with a larger sample size must be conducted to more accurately measure the true prevalence of DM among patients with TB and to determine associations of various clinical and radiographic presentations and clinical outcomes.

Institution

(Pablo-Villamor) Medical Specialist II, Vicente Sotto Memorial Medical Center, Philippines (Benedicto) Philippine Tuberculosis Society Inc, Philippines

(Benedicto) Diagnostic Section Department of Radiology, PGH, Philippines

(Perez) Quezon Institute and Central Chest Clinic and Dispensary Philippine Tuberculosis Society Inc, Philippines

Publisher

Philippine College of Physicians

Emtree Heading

adult; article; China; clinical article; clinical feature; cohort analysis; controlled study; *diabetes mellitus/di [Diagnosis]; diagnostic value; disease association; fatality; feasibility study; female; human; India; *lung tuberculosis; male; observational study; outpatient care; prevalence; prospective study; radiography; risk factor; sample size; *screening test; short course therapy; sputum smear; systematic review; treatment outcome.

Link to the Ovid Full Text or citation:

Click here for full text options

28.

Survival analysis of adult tuberculosis disease.

Ajagbe O.B., Kabair Z., O'Connor T.

PLoS ONE. 9 (11) (no pagination), 2014. Article Number: 112838. Date of Publication: 19 Nov 2014.

AN: 2014954075

Background: We conducted a survival analysis of all the confirmed cases of Adult Tuberculosis (TB) patients treated in Cork-City, Ireland. The aim of this study was to estimate Survival time (ST), including median time of survival and to assess the association and impact of covariates (TB risk factors) to event status and ST. The outcome of the survival analysis is reported in this paper. Methods: We used a retrospective cohort study research design to review data of 647 bacteriologically confirmed TB patients from the medical record of two teaching hospitals. Mean age 49 years (Range 18-112). We collected information on potential risk factors of all confirmed cases of TB treated between 2008-2012. For the survival analysis, the outcome of interest was 'treatment failure' or 'death' (whichever came first). A univariate descriptive statistics analysis was conducted using a non-parametric procedure, Kaplan-Meier (KM) method to estimate overall survival (OS), while the Cox proportional hazard model was used for the multivariate analysis to determine possible association of predictor variables and to obtain adjusted hazard ratio. P value was set at <0.05, log likelihood ratio test at >0.10. Data were analysed using SPSS version 15.0.

Results: There was no significant difference in the survival curves of male and female patients. (Log rank statistic = 0.194, df = 1, p = 0.66) and among different age group (Log rank statistic = 1.337, df = 3, p = 0.72). The mean overall survival (OS) was 209 days (95%CI: 92-346) while the median was 51 days (95% CI: 35.7-66). The mean ST for women was 385 days (95%CI: 76.6-694) and for men was 69 days (95%CI: 48.8-88.5). Multivariate Cox regression showed that patient who had history of drug misuse had 2.2 times hazard than those who do not have drug misuse. Smokers and alcohol drinkers had hazard of 1.8 while patients born in country of high endemicity (BICHE) had hazard of 6.3 and HIV co-infection hazard was 1.2.

Conclusion: There was no significant difference in survival curves of male and female and among age group. Women had a higher ST compared to men. But men had a higher hazard rate compared to women. Anti-TNF, immunosuppressive medication and diabetes were found to be associated with longer ST, while alcohol, smoking, RICHE, BICHE was associated with shorter ST.

Institution

(Ajagbe, Kabair) Department of Public Health and Epidemiology, University College Cork, Cork, Ireland (O'Connor) Mercy University Hospital, Cork, Ireland

Publisher

Public Library of Science

Emtree Heading

adult; age; aged; alcohol consumption; article; cohort analysis; diabetes mellitus; disease association; drug misuse; endemic disease; ethnicity; female; hazard ratio; human; Human immunodeficiency virus infection; Ireland; Kaplan Meier method; major clinical study; male; medical history; medical record review; middle aged; mixed infection; mortality; multivariate analysis; overall survival; prediction; proportional hazards model; retrospective study; risk factor; sex difference; smoking; *survival rate; survival time; teaching hospital; treatment failure; *tuberculosis/pc [Prevention]; *tuberculosis/dt [Drug Therapy]; very elderly; BCG vaccine/dt [Drug Therapy]; immunosuppressive agent; tuberculostatic agent/dt [Drug Therapy]; tumor necrosis factor inhibitor.

Link to the Ovid Full Text or citation:

Click here for full text options

29.

Pulmonary tuberculosis increases the risk of pulmonary thromboembolism: A nationwide population-based cohort study.

Chung W.-S., Lin C.-L., Chen Y.-F., Hsu W.-H., Kao C.-H.

Thrombosis and Haemostasis. 112 (6) (pp 1325-1327), 2014. Date of Publication: 2014.

AN: 2014948743

Institution

(Chung) Department of Internal Medicine, Taichung Hospital, Ministry of Health and Welfare, Executive Yuan, Taichung, Taiwan (Republic of China) (Chung, Hsu, Kao) Graduate Institute of Clinical Medical Science and School of Medicine, College of Medicine, China Medical University, Taichung, Taiwan (Republic of China)

(Chung, Chen) Department of Healthcare Administration, Central Taiwan University of Science and Technology, Taichung, Taiwan (Republic of China)

(Lin) Management Office for Health Data, China Medical University Hospital, Taichung, Taiwan (Republic of China)

(Kao) Department of Nuclear Medicine and PET Center, China Medical University Hospital, Taichung, Taiwan (Republic of China)

Publisher

Schattauer GmbH

Emtree Heading

adult; aged; cerebrovascular accident; clinical article; cohort analysis; comorbidity; controlled study; diabetes mellitus; female; hazard ratio; heart atrium fibrillation; heart failure; hormone substitution; human; hyperlipidemia; hypertension; incidence; leg fracture; letter; *lung embolism; *lung tuberculosis; male; Mycobacterium tuberculosis; national health insurance; neoplasm; outcome assessment; risk factor; venous thromboembolism; anticoagulant agent; antithrombocytic agent; contraceptive agent.

Link to the Ovid Full Text or citation:

Click here for full text options

30.

Results of the implementation of a pilot model for the bidirectional screening and joint management of patients with pulmonary Tuberculosis and Diabetes mellitus in Mexico.

Castellanos-Joya M., Delgado-Sanchez G., Ferreyra-Reyes L., Cruz-Hervert P., Ferreira-Guerrero E., Ortiz-Solis G., Jimenez M.I., Salazar L.L., Montero-Campos R., Mongua-Rodriguez N., Baez-Saldana R., Bobadilla-Del-Valle M., Gonza Lez-Roldan J.F., Ponce-De-Leon A., Sifuentes-Osornio J., Garcia-Garcia L.

PLoS ONE. 9 (9) (no pagination), 2014. Article Number: e106961. Date of Publication: 17 Sep 2014.

AN: 2014804910

Objective: To evaluate the feasibility and effectiveness of the CFTB/DM in Mexico. Design. Prospective observational cohort. Setting. 15 primary care units in 5 states in Mexico. Participants: Patients aged >20 years diagnosed with DM or pulmonary TB who sought care at participating clinics. Intervention: The WHO/Union CFTB/DM was adapted and implemented according to official Mexican guidelines. We recruited participants from July 2012 to April 2013 and followed up until March 2014. Bidirectional screening was performed. Patients diagnosed with TB and DM were invited to receive TB treatment under joint management. Main outcome measures. Diagnoses of TB among DM, of DM among TB, and treatment outcomes among patients with DM and TB. Background: Recently, the World Health Organisation and the International Union Against Tuberculosis and Lung Disease published a Collaborative Framework for the Care and Control of Tuberculosis (TB) and Diabetes (DM) (CFTB/DM) proposing bidirectional screening and joint management.

Results: Of 783 DM patients, 11 (1.4%) were unaware of their TB. Of 361 TB patients, 16 (4.4%) were unaware of their DM. 95 TB/DM patients accepted to be treated under joint management, of whom 85 (89.5%) successfully completed treatment. Multiple linear regression analysis with change in HbA1c and random capillary glucose as dependent variables revealed significant decrease with time (regression coefficients (beta) =20.660, (95% confidence interval (CI), 20.96 to 20.35); and beta =21.889 (95% CI, 22.77 to 21.01, respectively)) adjusting by sex, age and having been treated for a previous TB episode. Patients treated under joint management were more likely to experience treatment success than patients treated under routine DM and TB programs as compared to historical (adjusted OR (aOR), 2.8, 95%CI 1.28-6.13) and same period (aOR 2.37, 95% CI 1.13-4.96) comparison groups.

Conclusions: Joint management of TB and DM is feasible and appears to improve clinical outcomes.

Institution

(Castellanos-Joya, Jimenez, Salazar) Direccion Del Programa de Micobacteriosis, Centro Nacional de Programas Preventivos y Control de Enfermedades, Mexico, Distrito Federal, Mexico (Delgado-Sanchez, Ferreyra-Reyes, Cruz-Hervert, Ferreira-Guerrero, Montero-Campos, Mongua-Rodriguez, Baez-Saldana, Garcia-Garcia) Centro de Investigacion Sobre Enfermedades Infecciosas, Instituto Nacional de Salud Publica, Cuernavaca, Morelos, Mexico

(Ortiz-Solis) Direccion Del Programa de Salud en El Adulto y en El Anciano, Centro Nacional de Programas Preventivos y Control de Enfermedades, Mexico, Distrito Federal, Mexico

(Bobadilla-Del-Valle, Ponce-De-Leon) Laboratorio de Microbiologia, Instituto Nacional de Ciencias Medicas y de Nutricion Salvador Zubira N, Mexico, Distrito Federal, Mexico

(Gonza Lez-Roldan) Direccion General, Centro Nacional de Programas Preventivos y Control de Enfermedades, Mexico, Distrito Federal, Mexico

(Sifuentes-Osornio) Direccion Medica, Instituto Nacional de Ciencias Medicas y de Nutricion Salvador Zubiran, Mexico, Distrito Federal, Mexico

Publisher

Public Library of Science

Emtree Heading

adult; age; aged; article; *bidirectional screening; clinical effectiveness; cohort analysis; Collaborative Framework for the Care and Control of Tuberculosis and Diabetes; control strategy; controlled study; *diabetes mellitus/di [Diagnosis]; *diabetes mellitus/ep [Epidemiology]; *diabetes mellitus/pc [Prevention]; *diabetes mellitus/dt [Drug Therapy]; disease control; feasibility study; female; follow up; glucose blood level; *health care planning; hemoglobin blood level; human; human cell; *lung tuberculosis/di [Diagnosis]; *lung tuberculosis/ep [Epidemiology]; *lung tuberculosis/pc [Prevention]; *lung tuberculosis/dt [Drug Therapy]; major clinical study; male; Mexico; multiple linear regression analysis; observational study; *patient care; physical activity; pilot study; prospective study; screening; sex difference; glucose/ec [Endogenous Compound]; hemoglobin A1c/ec [Endogenous Compound]; insulin/dt [Drug Therapy]; isoniazid/dt [Drug Therapy]; oral antidiabetic agent/po [Oral Drug Administration]; oral antidiabetic agent/dt [Drug Therapy]; rifampicin/dt [Drug Therapy].

Link to the Ovid Full Text or citation:

Click here for full text options

31.

Predictors of pulmonary tuberculosis treatment outcomes in South Korea: A prospective cohort study, 2005-2012.

Choi H., Lee M., Chen R.Y., Kim Y., Yoon S., Joh J.S., Park S.K., Dodd L.E., Lee J., Song T., Cai Y., Goldfeder L.C., Via L.E., Carroll M.W., Barry III C.E., Cho S.-N.

BMC Infectious Diseases. 14 (1) (no pagination), 2014. Article Number: 360. Date of Publication: 02 Jul 2014.

AN: 2014465794

Background: Tuberculosis remains an important health concern in many countries. The aim of this study was to identify predictors of unfavorable outcomes at the end of treatment (EOT) and at the end of study (EOS; 40 months after EOT) in South Korea.Methods: New or previously treated tuberculosis patients were recruited into a prospective observational cohort study at two hospitals in South Korea. To identify predictors of unfavorable outcomes at EOT and EOS, logistic regression analysis was performed.Results: The proportion of multidrug-resistant tuberculosis (MDR-TB) was 8.2% in new cases and 57.9% in previously treated cases. Of new cases, 68.6% were cured, as were 40.7% of previously treated cases. At EOT, diabetes, >3 previous TB episodes, >1 significant regimen change, and MDR-TB were significantly associated with treatment failure or death. At EOS, age >35, body-mass index (BMI) <18.5, diabetes, and MDR-TB were significantly associated with treatment failure, death, or relapse. Among cases that were cured at EOT, age >50 and a BMI <18.5 were associated with subsequent death or relapse during follow-up to EOS. Treatment interruption was associated with service sector employees or laborers, bilateral lesions on chest X-ray, and previous treatment failure or treatment interruption history.Conclusions: Risk factors for poor treatment outcomes at EOT and EOS include both patient factors (diabetes status, age, BMI) and disease factors (history of multiple previous treatment episodes, MDR-TB). In this longitudinal, observational cohort study, diabetes mellitus and MDR-TB were risk factors for poor treatment outcomes and relapse. Measures to help ensure that the first tuberculosis treatment episode is also the last one may improve treatment outcomes.Trial registration: ClinicalTrials.gov ID: NCT00341601. © 2014 Choi et al.; licensee BioMed Central Ltd.

Institution

(Choi, Lee, Kim, Yoon, Lee, Song, Cho) International Tuberculosis Research Center, Changwon, South Korea (Choi) BK21PLUS Program in Public Health Sciences, Department of Health Sciences, Graduate School, Korea University, Seoul, South Korea

(Chen, Cai, Goldfeder, Via, Carroll, Barry III) Tuberculosis Research Section, Laboratory of Clinical Infectious Disease, National Institute of Allergy and Infectious Diseases (NIAID), National Institutes of Health (NIH), Bethesda, Maryland, United States

(Joh) Department of Internal Medicine, National Medical Center, Seoul, South Korea

(Park) National Masan Tuberculosis Hospital, Changwon, South Korea

(Dodd) Biostatistics Research Branch, NIAID, NIH, Bethesda, Maryland, United States

(Cho) Department of Microbiology and Institute for Immunology, Immunological Diseases, Yonsei University College of Medicine, Brain Korea 21 Project for Medical Science, Seoul, South Korea

Publisher

BioMed Central Ltd.

Emtree Heading

adult; article; body mass; cohort analysis; controlled study; death; diabetes mellitus; female; follow up; human; *lung tuberculosis; major clinical study; male; multidrug resistant tuberculosis; observational study; prediction; prognosis; prospective study; relapse; South Korea; thorax radiography; treatment failure; *treatment outcome; treatment withdrawal.

Link to the Ovid Full Text or citation:

Click here for full text options

32.

Does alcohol consumption during multidrug-resistant tuberculosis treatment affect outcome? A population-based study in Kerala, India.

Duraisamy K., Mrithyunjayan S., Ghosh S., Nair S.A., Balakrishnan S., Subramoniapillai J., Oeltmann J.E., Moonan P.K., Kumar A.M.V.

Annals of the American Thoracic Society. 11 (5) (pp 712-715), 2014. Date of Publication: June 2014.

AN: 2014444288

Rationale: India reports the largest number of multidrug-resistant tuberculosis cases in the world; yet, no longitudinal study has assessed factors related to treatment outcomes under programmatic conditions in the public sector. Objectives: To describe demographic, clinical, and risk characteristics associated with treatment outcomes for all patients with multidrug-resistant tuberculosis registered in the Revised National Tuberculosis Control Programme, Kerala State, India from January 1, 2009 to June 30, 2010. Methods: Cox regression methods were used to calculate adjusted hazard ratios with 95% confidence intervals (CIs) to assess factors associated with an unsuccessful treatment outcome. Measurements and Main Results: Of 179 patients with multidrug-resistant tuberculosis registered, 112 (63%) had successful treatment outcomes (77 bacteriologically cured, 35 treatment completed) and 67 (37%) had unsuccessful treatment outcomes (30 died, 26 defaulted, 9 failed treatment, 1 stopped treatment because of drug-related adverse events, and 1 developed extensively drug-resistant tuberculosis). The hazard for unsuccessful outcome was significantly higher among patients who consumed alcohol during treatment (adjusted hazard ratio, 4.3; 95% CI, 1.1-17.6) than those who did not. Persons who consumed alcohol during treatment, on average, missed 18 more intensive-phase doses (95% CI, 13-22) than those who did not. Although many patients had diabetes (33%), were ever smokers (39%), or had low body mass index (47%), these factors were not associated with outcome. Conclusion: Overall treatment success was greater than global and national averages; however, outcomes among patients consuming alcohol remained poor. Integration of care for multidrug-resistant tuberculosis and alcoholism should be considered to improve treatment adherence and outcomes. Copyright © 2014 by the American Thoracic Society.

Institution

(Duraisamy, Nair, Balakrishnan) Department of Tuberculosis, WHO Country Office for India, New Delhi 110029, India (Mrithyunjayan, Subramoniapillai) State TB Cell, Direct. of Health Services, Thiruvananthapuram, Kerala, India

(Ghosh, Oeltmann, Moonan) U.S. Centers for Disease Control and Prevention, Division of TB Elimination, Atlanta, GA, United States

(Kumar) International Union Against Tuberculosis and Lung Disease (The Union), South-East Asia Regional Office, New Delhi, India

Publisher

American Thoracic Society

Emtree Heading

adolescent; adult; *alcohol consumption; antibiotic sensitivity; arthralgia/si [Side Effect]; article; body mass; cohort analysis; controlled study; demography; diabetes mellitus; drinking behavior; drug treatment failure; extensively drug resistant tuberculosis/di [Diagnosis]; extensively drug resistant tuberculosis/dt [Drug Therapy]; female; follow up; gastrointestinal symptom/si [Side Effect]; health program; human; India; jaundice/si [Side Effect]; major clinical study; male; *multidrug resistant tuberculosis/di [Diagnosis]; *multidrug resistant tuberculosis/dt [Drug Therapy]; *multidrug resistant tuberculosis/ep [Epidemiology]; Mycobacterium tuberculosis; nephrotoxicity/si [Side Effect]; neuropathy/si [Side Effect]; obesity; ototoxicity/si [Side Effect]; outcome assessment; population research; retrospective study; smoking; suicidal ideation/si [Side Effect]; tuberculosis control; *alcohol; cycloserine/ae [Adverse Drug Reaction]; cycloserine/dt [Drug Therapy]; ethambutol/ae [Adverse Drug Reaction]; ethambutol/dt [Drug Therapy]; ethionamide/ae [Adverse Drug Reaction]; ethionamide/dt [Drug Therapy]; isoniazid/ae [Adverse Drug Reaction]; isoniazid/dt [Drug Therapy]; kanamycin/ae [Adverse Drug Reaction]; kanamycin/dt [Drug Therapy]; levofloxacin/ae [Adverse Drug Reaction]; levofloxacin/dt [Drug Therapy]; ofloxacin/ae [Adverse Drug Reaction]; ofloxacin/dt [Drug Therapy]; pyrazinamide/ae [Adverse Drug Reaction]; pyrazinamide/dt [Drug Therapy]; rifampicin/ae [Adverse Drug Reaction]; rifampicin/dt [Drug Therapy]; streptomycin/ae [Adverse Drug Reaction]; streptomycin/dt [Drug Therapy].

Link to the Ovid Full Text or citation:

Click here for full text options

33.

Cohort analysis of antenatal care and delivery outcomes in pregnancy: A basis for improving maternal health.

Harries A.D., Jahn A., Ben-Smith A., Gadabu O.J., Douglas G.P., Seita A., Khader A., Zachariah R.

Public Health Action. 4 (2) (pp 75-78), 2014. Date of Publication: 01 Jun 2014.

AN: 2014421981

Cohort analysis has been the cornerstone of tuberculosis (TB) monitoring and evaluation for nearly two decades; these principles have been adapted for patients with the human immunodeficiency virus/acquired immune-deficiency syndrome on antiretroviral treatment and patients with diabetes mellitus and hypertension. We now make the case for using cohort analyses for monitoring pregnant women during antenatal care, up to and including childbirth. We believe that this approach would strengthen the current monitoring and evaluation systems used in antenatal care by providing more precise information at regular time intervals. Accurate real-time data on how many pregnant women are enrolled in antenatal care, their characteristics, the interventions they are receiving and the outcomes for mother and child should provide a solid basis for action to reduce maternal mortality. © 2014 The Union.

Institution

(Harries) International Union Against Tuberculosis and Lung Disease, Paris, France (Harries) London School of Hygiene and Tropical Medicine, London, United Kingdom

(Jahn) Department for HIV and AIDS, Ministry of Health, Lilongwe, Malawi

(Jahn) International Training and Education Centre for Health, Malawi

(Jahn) University of Washington, Seattle, WA, United States

(Ben-Smith) Department of Biomedical Informatics, University of Pittsburgh, Pittsburgh, PA, United States

(Gadabu) Baobab Health Trust, Lilongwe, Malawi

(Douglas) Center for Health Informatics for the Underserved, University of Pittsburgh, Pittsburgh, PA, United States

(Seita, Khader) United Nations Relief and Works Agency for Palestine Refugees in the Near East, UNRWA Headquarters, Amman, Jordan

(Zachariah) Medecins Sans Frontieres, MSF, Medical Department, Brussels Operational Centre, Luxembourg, Luxembourg

Publisher

International Union Against Tuberculosis and Lung Disease (The Union) (68 boulevard Saint Michel, Paris 75006, France)

Emtree Heading

article; clinical evaluation; cohort analysis; diabetes mellitus; female; follow up; highly active antiretroviral therapy; human; Human immunodeficiency virus infection; hypertension; maternal mortality; *maternal welfare; patient care; *pregnancy outcome; *prenatal care; public health; risk factor; treatment outcome; tuberculosis.

Link to the Ovid Full Text or citation:

Click here for full text options

34.

Predictors of non-traumatic coma in a pediatric cohort from a South Indian tertiary care center: Results of a multivariate analysis.

Gowda V.K.N., Bannigidad N.B., Kumar P., Srikanteswara P.-K., Shivananda, Govindraj, Vykuntaraju S.H., Ramaswamy P.

Journal of Pediatric Neurology. 12 (1) (pp 35-43), 2014. Date of Publication: 2014.

AN: 2014381538

Objective: To study the etiology and clinical profile of non-traumatic coma in children at tertiary care center and to determine the predictors of outcome. Methods: One hundred and four consecutive children between 2 mo-12 yr were studied. The clinical signs at admission; vital signs, Glasgow coma scale, respiratory pattern, papillary reflex, extra-ocular movements, fundus picture and motor deficits were recorded. Etiology of coma was determined by clinical history, examination and relevant investigations. Their progress was monitored clinically, biochemically and with multi-system monitors. Outcome was recorded as survived or died. Results: Etiology of coma in 65% cases was intracranial infections; other causes were metabolic (20%). Sixty-seven percent recovered completely, 16% had residual neurodeficits, 16% died. Survival was better in children with intracranial infections (13%) as compared to metabolic coma (33%). On multivariate logistic regression, bradycardia, hypotension, abnormal respiratory pattern (especially, ataxic type), duration of coma more than 48 h, Glasgow coma scale < 7 at admission, unequal and non-reactive pupils, papilledema, abnormal extra-ocular movements, motor deficits, signs of meningitis correlated with mortality. Requirement of ventilatory support and abnormal computerized tomography findings correlated with mortality. Conclusions: Intracranial infections were the most common cause of non-traumatic coma in children; the most common cause of death being metabolic coma. Simple clinical signs and relevant investigations served as prognostic indicators of outcome. © 2014-IOS Press.

Institution

(Gowda) Department of Pediatric Neurology, Indira Gandhi Institute of Child Health, Bangalore, India (Bannigidad, Kumar, Shivananda, Govindraj, Ramaswamy) Department of Pediatrics, Indira Gandhi Institute of Child Health, Bangalore, India

(Srikanteswara) Department of Neurology, Bangalore Medical College and Research Institute, Bangalore, India

(Vykuntaraju) Department of Anatomy, Kempegowda Institute of Medical Sciences, Bangalore, India

(Gowda) Bangalore Child Neurology and Rehabilitation Center, HANS Complex, 8/A 1st Main 1st Cross, Vijayanagar, Bangalore 560040, India

Publisher

IOS Press (Nieuwe Hemweg 6B, Amsterdam 1013 BG, Netherlands)

Emtree Heading

abnormal respiratory sound; anterior fontanel; article; assisted ventilation; bacterial meningitis; bradycardia; cause of death; child; *childhood disease/dt [Drug Therapy]; *childhood disease/et [Etiology]; *childhood disease/th [Therapy]; childhood mortality; clinical examination; cohort analysis; *coma/et [Etiology]; *coma/th [Therapy]; computer assisted tomography; controlled study; convulsion; dehydration; diabetic ketoacidosis; epileptic state; extraocular muscle; eye movement; female; fever; Glasgow coma scale; glucose blood level; headache; hemiparesis; hemiplegia; hepatic encephalopathy; hospital admission; human; hyperventilation; hypotension; hypoxia; Indian; intensive care unit; jaundice; loose feces; major clinical study; male; malnutrition; meningitis; organophosphate poisoning/dt [Drug Therapy]; outcome assessment; pallor; papilledema; prognosis; quadriplegia; rash; retrospective study; Reye syndrome; snakebite/dt [Drug Therapy]; sodium blood level; teaching hospital; tendon reflex; tertiary care center; tuberculous meningitis; vital sign; vomiting; glucose/ec [Endogenous Compound]; organophosphorus compound/to [Drug Toxicity]; snake venom antiserum/dt [Drug Therapy]; sodium/ec [Endogenous Compound].

Link to the Ovid Full Text or citation:

Click here for full text options

35.

Diabetes mellitus, smoking status, and rate of sputum culture conversion in patients with multidrug-resistant tuberculosis: A cohort study from the country of Georgia.

Magee M.J., Kempker R.R., Kipiani M., Tukvadze N., Howards P.P., Venkat Narayan K.M., Blumberg H.M.

PLoS ONE. 9 (4) (no pagination), 2014. Article Number: e94890. Date of Publication: 15 Apr 2014.

AN: 2014302165

Introduction: Diabetes mellitus (DM) is a risk factor for active tuberculosis (TB) but little is known about the effect of DM on culture conversion among patients with multidrug-resistant (MDR)-TB. The primary aim was to estimate the association between DM and rate of TB sputum culture conversion. A secondary objective was to estimate the association between DM and the risk of poor treatment outcomes among patients with MDR-TB. Materials and Methods: A cohort of all adult patients starting MDR-TB treatment in the country of Georgia between 2009-2011 was followed during second-line TB therapy. Cox proportional models were used to estimate the adjusted hazard rate of sputum culture conversion. Log-binomial regression models were used to estimate the cumulative risk of poor TB treatment outcome. Results: Among 1,366 patients with sputum culture conversion information, 966 (70.7%) had culture conversion and the median time to conversion was 68 days (interquartile range 50-120). The rate of conversion was similar among patients with MDR-TB and DM (adjusted hazard ratio [aHR] 0.95, 95%CI 0.71-1.28) compared to patients with MDR-TB only. The rate of culture conversion was significantly less in patients that currently smoked (aHR 0.82, 95%CI 0.71-0.95), had low body mass index (aHR 0.71, 95%CI 0.59-0.84), second-line resistance (aHR 0.56, 95%CI 0.43-0.73), lung cavities (aHR 0.70, 95%CI 0.59-0.83) and with disseminated TB (aHR 0.75, 95%CI 0.62-0.90). The cumulative risk of poor treatment outcome was also similar among TB patients with and without DM (adjusted risk ratio [aRR] 1.03, 95%CI 0.93-1.14). Conclusions: In adjusted analyses, DM did not impact culture conversion rates in a clinically meaningful way but smoking did. © 2014 Magee et al.

Institution

(Magee, Howards, Venkat Narayan, Blumberg) Department of Epidemiology, Rollins School of Public Health, Emory University, Atlanta, GA, United States (Magee, Venkat Narayan, Blumberg) Hubert Department of Global Health, Rollins School of Public Health, Emory University, Atlanta, GA, United States

(Kempker, Blumberg) Division of Infectious Diseases, Department of Medicine, Emory University, Atlanta, GA, United States

(Kipiani, Tukvadze) National Center for Tuberculosis and Lung Diseases, Tbilisi, GA, Georgia

Publisher

Public Library of Science

Emtree Heading

adult; article; bacterium isolate; *biotransformation; body mass; cohort analysis; *diabetes mellitus; disease association; female; human; lung cavity; major clinical study; male; miliary tuberculosis; *multidrug resistant tuberculosis/dr [Drug Resistance]; *multidrug resistant tuberculosis/dt [Drug Therapy]; Mycobacterium tuberculosis; nonhuman; risk assessment; *smoking; *sputum culture; treatment duration; treatment outcome; United States; capreomycin/dt [Drug Therapy]; cycloserine/dt [Drug Therapy]; isoniazid/dt [Drug Therapy]; kanamycin/dt [Drug Therapy]; levofloxacin/dt [Drug Therapy]; protionamide/dt [Drug Therapy]; pyrazinamide/dt [Drug Therapy]; rifampicin/dt [Drug Therapy].

Link to the Ovid Full Text or citation:

Click here for full text options

36.

Infection-related mortality is higher for kidney allograft recipients with pretransplant diabetes mellitus.

Hayer M.K., Farrugia D., Begaj I., Ray D., Sharif A.

Diabetologia. 57 (3) (pp 554-561), 2014. Date of Publication: March 2014.

AN: 2014143513

Aims/hypothesis: The risk of infection-related mortality in kidney allograft recipients with pre-existing diabetes mellitus is unknown. We determined the risk of infection-related mortality after kidney transplantation in a population-based cohort stratified by diagnosis of pre-existing diabetes mellitus. Methods: We linked data between two national registries (Hospital Episode Statistics and the Office for National Statistics) to select all mortality events after kidney transplantation in England between April 2001 and March 2012. The primary outcome measure was infection-related mortality after transplantation comparing diabetic with non-diabetic recipients. Results: A total of 19,103 kidney allograft recipients were analysed; 2,968 (15.5%) were known to have diabetes before kidney transplantation. After transplantation, 2,085 deaths (10.9%) occurred (median follow-up 4.4 years [interquartile range 2.2-7.3]), with 434 classified as secondary to infection (20.8% of all deaths). Risk of overall (16.0% vs 10.0%, p < 0.001) and infection-related (3.3% vs 2.1%, p < 0.001) mortality after kidney transplantation was higher for diabetic than non-diabetic recipients, respectively. No cytomegalovirus-related deaths occurred in diabetic recipients compared with 5.7% in non-diabetic recipients (p < 0.007), with a trend towards more unspecified sepsis in diabetic recipients (30.6% vs 22.6%, respectively, p = 0.070). Diabetes at the time of transplantation was an independent risk factor predicting infection-related mortality in kidney allograft recipients after transplantation (HR 1.71 [95% CI 1.36, 2.15], p < 0.001). Conclusions/interpretation: Infection-related mortality is more common in kidney allograft recipients with pre-existing diabetes mellitus. Further work is required to determine whether attenuated immunosuppression is beneficial for diabetic kidney allograft recipients. © 2013 Springer-Verlag Berlin Heidelberg.

Institution

(Hayer, Farrugia, Sharif) Department of Nephrology and Transplantation, Renal Institute of Birmingham, Queen Elizabeth Hospital, Edgbaston, Birmingham B15 2WB, United Kingdom (Begaj, Ray) Department of Medical Informatics, Queen Elizabeth Hospital, Birmingham, United Kingdom

Publisher

Springer Verlag (Tiergartenstrasse 17, Heidelberg D-69121, Germany)

Emtree Heading

adult; aged; article; brain infection/co [Complication]; brain infection/ep [Epidemiology]; bronchopneumonia/co [Complication]; bronchopneumonia/ep [Epidemiology]; cellulitis/co [Complication]; cellulitis/ep [Epidemiology]; cholecystitis/co [Complication]; cholecystitis/ep [Epidemiology]; cohort analysis; colitis/co [Complication]; colitis/ep [Epidemiology]; controlled study; Cytomegalovirus; *cytomegalovirus infection/co [Complication]; *cytomegalovirus infection/ep [Epidemiology]; *diabetes mellitus; diabetic patient; disease registry; *end stage renal disease/su [Surgery]; endocarditis/co [Complication]; endocarditis/ep [Epidemiology]; female; *graft infection/co [Complication]; *graft infection/ep [Epidemiology]; graft recipient; hazard ratio; hepatitis/co [Complication]; hepatitis/ep [Epidemiology]; human; incidence; kidney allograft; *kidney transplantation; major clinical study; male; middle aged; multicenter study; mycosis/co [Complication]; mycosis/ep [Epidemiology]; osteomyelitis/co [Complication]; osteomyelitis/ep [Epidemiology]; outcome assessment; peritonitis/co [Complication]; peritonitis/ep [Epidemiology]; priority journal; risk factor; sepsis/co [Complication]; skin ulcer/co [Complication]; skin ulcer/ep [Epidemiology]; surgical mortality; surgical patient; surgical risk; trend study; tuberculosis/co [Complication]; tuberculosis/ep [Epidemiology]; United Kingdom; urinary tract infection/co [Complication]; urinary tract infection/ep [Epidemiology].

Link to the Ovid Full Text or citation:

Click here for full text options

37.

Impact of diabetes on treatment outcomes and long-term survival in multidrug-resistant tuberculosis.

Kang Y.A., Kim S.Y., Jo K.-W., Kim H.J., Park S.-K., Kim T.-H., Kim E.K., Lee K.M., Lee S.S., Park J.S., Koh W.-J., Kim D.Y., Shim T.S.

Respiration. 86 (6) (pp 472-478), 2014. Date of Publication: January 2014.

AN: 2014071108

Background: Few studies have investigated the impact of diabetes mellitus (DM), a globally increasing metabolic disease, on treatment outcomes and long-term survival in patients with multidrug-resistant forms of tuberculosis (MDR-TB). Objectives: We analyzed outcomes in a large cohort to assess the impact of DM on treatment outcomes of patients with MDR-TB. Methods: MDR-TB patients newly diagnosed or retreated between 2000 and 2002 and followed for 8-11 years were retrospectively analyzed with respect to the effect of DM as a comorbidity on their treatment outcome and long-term survival. Results: Of 1,407 patients with MDR-TB, 239 (17.0%) had coexisting DM. The mean age and body mass index were higher in MDR-TB patients with DM [MDR-TBDM(+)] than in those without DM [MDR-TBDM(-)]. Patients with MDR-TB and a comorbidity of DM had a significantly lower treatment success rate than those without a history of DM (36.0 vs. 47.2%, p = 0.002). In addition, DM was the negative predictor for MDR-TB treatment success in multivariate analyses [odds ratio 0.51, 95% confidence interval (CI) 0.26-0.99]. Mean survival times were also lower in MDR-TBDM(+) than in MDR-TBDM(-) patients (102 vs. 114 months, p = 0.001), with DM as a significant predictor of poor long-term survival in multivariate analyses (hazard ratio 1.59, 95% CI 1.01-2.50). Conclusions: Among MDR-TB patients, DM was a relatively common comorbidity. In patients undergoing treatment for MDR-TB and followed for 8-11 years, it was found to be independently associated with an increased risk of both treatment failure and death. Copyright © 2013 S. Karger AG, Basel.

Institution

(Kang, Kim) Department of Internal Medicine, Yonsei University College of Medicine, Seoul, South Korea (Jo, Shim) Division of Pulmonary and Critical Care Medicine, University of Ulsan College of Medicine, Asan Medical Center, 388-1 Pungnap-dong, Songpa-gu, Seoul, South Korea

(Kim) Department of Epidemiology, Korean Institute of Tuberculosis, Seoul, South Korea

(Kim) Department of Internal Medicine, Hanyang University College of Medicine, Seoul, South Korea

(Koh) Division of Pulmonary and Critical Care Medicine, Department of Medicine, Sungkyunkwan University School of Medicine, Seoul, South Korea

(Park) Clinical Research Center, National Masan Hospital, Masan, South Korea

(Kim) Division of Pulmonary and Critical Care Medicine, Department of Internal Medicine, Pochon CHA University, Seongnam, South Korea

(Lee) Department of Internal Medicine, Chungbuk National University College of Medicine, Chungbuk National University Hospital, Cheongju, South Korea

(Lee) Department of Internal Medicine, Inje University School of Medicine, Ilsan Paik Hospital, Goyang, South Korea

(Park) Department of Internal Medicine, Dankook University College of Medicine, Dankook University Hospital, Cheonan, South Korea

(Kim) Department of Thoracic Surgery, National Mokpo Hospital, Mokpo, South Korea

Publisher

S. Karger AG (Allschwilerstrasse 10, P.O. Box, Basel CH-4009, Switzerland)

Emtree Heading

adolescent; adult; aged; antibiotic resistance; article; body mass; child; cohort analysis; comorbidity; *diabetes mellitus; female; human; *long term survival; major clinical study; male; middle aged; *multidrug resistant tuberculosis/dt [Drug Therapy]; outcome assessment; predictor variable; priority journal; retrospective study; school child; short course therapy; survival time; treatment duration; treatment outcome; very elderly; young adult; capreomycin/dt [Drug Therapy]; enviomycin/dt [Drug Therapy]; kanamycin/dt [Drug Therapy].

Link to the Ovid Full Text or citation:

Click here for full text options

38.

Comorbidities and MDR-TB treatment outcomes in Georgia-2009-11 cohort.

Kikvidze M., Ikiashvili L.

European Respiratory Journal. Conference: European Respiratory Society Annual Congress 2014 Munich Germany. Conference Start: 20140906 Conference End: 20140906. Conference Publication: (var.pagings). 44 (no pagination), 2014. Date of Publication: 01 Sep 2014.

AN: 71851788

Background. Treatment of MDR-TB is complex and chemotherapy course's outcome depends on many factors. Comorbidities may complicate TB disease and effect treatment response. Aims and objectives. The aim of the study was comparison of chemotherapy response among patients with and those without comorbidities in a setting of individualized MDR-TB care and adequate management of all diseases. Methods. We conducted a retrospective cohort study of 1970 drug-resistant tuberculosis patients integrated in DOTS+ programme in 2009-2011 years in Georgia. Patients were routinely investigated for HIV, diabetes mellitus (DM) and viral hepatitis. Documentary approved diagnoses of other diseases were taken in account. Dispersive analysis using Mann-Whitney/Wilcoxon Two-Sample Test was provided to compare the treatment outcomes of patients with and those without comorbidity. Results. Of 1970 patients with drug-resistant tuberculosis 486(24,7%) had one or more comorbidity. 223 patient(11,3%) had liver diseases, DM-104(5,2%), HIV-72(3,7%), Psychiatric diseases - 53(2,7%), other-55(2,9%). Definition "success" received 966(49%) patient:44,0% with comorbidities vs 50,7%-without(p=0,01),"Default"-30,5% vs 28,4%, "Failure"- 4,1% vs 4,0(p>0,05, CI-95%),"Death"- 10,7% vs 5,7%(p<0,05, CI-95%). Mortality rate was highest during HIV-co-infection(RR-3, 73, 95% CI 2,38-5,86, p<0, 05). Definition "success" was lowest during HIV-co-infection(RR-0,67, 95%CI48-0,93, p<0,05) and chronic viral hepatitis "C"(RR-0,79, 95%CI 0,66-0,95, p<0,05). Conclusions. Our cohort study revealed that comorbidities had serious impact on treatment outcomes andwere associated with significant lower successful treatment outcomes and about twice higher death rate.

Institution

(Kikvidze, Ikiashvili) Department of MDR-TB, National Center for Tuberculosis and Lung Diseases (NCTBLD), Tbilisi, Georgia

Publisher

European Respiratory Society

Emtree Heading

*treatment outcome; *European; *society; patient; human; drug resistant tuberculosis; cohort analysis; mixed infection; virus hepatitis; chemotherapy; mortality; diseases; Human immunodeficiency virus; comorbidity; death; mental disease; liver disease; diagnosis; diabetes mellitus; treatment response; short course therapy.

Link to the Ovid Full Text or citation:

Click here for full text options

39.

Gender differences and TB treatment outcome among anti tuberculosis drug induced hepatitis patients.

Nadarajah K., Khan A.H., Hadi M.A., Aftab R.A., Ahmad N.

Respirology. Conference: 19th Congress of the Asian Pacific Society of Respirology Bali Indonesia. Conference Start: 20141113 Conference End: 20141116. Conference Publication: (var.pagings). 19 (pp 246), 2014. Date of Publication: November 2014.

AN: 71678367

Background: Anti-tubercle drug-induced hepatotoxicity (ATDH) causes substantial morbidity and mortality and diminishes treatment effectiveness. Aims And Objective: The aim of current study was to note the association between gender differences among anti-tuberculosis drug induced hepatotoxity patients and to evaluate TB treatment outcomes. Method: A retrospective cohort study was conducted at Hospital Pulau Pinang Malaysia. All TB patients presented between January 2007 and September 2013 were included for the current study. Patients with inadequate medical records and patients receiving concurrent potential hepatotoxic medications along with anti-TB treatment were excluded from current study. A valid data collection form was used for collecting patient demographic and clinical data. Data was analyzed by using SPSS version 20. Results: A total of 3093 TB patients were included for the final analysis. One hundred and ninety patients suffered from drug induced hepatitis, of which 146 (76.8%) were male and 44 (23.3%) were female patients. The mean age of male patients suffering from drug induced hepatitits was 47.49 (SD +/- 14.28) years whereas the mean age for female patients was 45.0 (SD +/- 14.96). Area of residence (p 0.001), smoking habit (p < 0.001), alcohol consumption (p < 0.001) and intravenous drug users (p < 0.001) had statistical significant association with drug induced hepatitis patient gender. Anti-tuberculosis induced drug hepatitis patients with diabetes mellitus (p 0.046) and HIV/AIDS 107 (p 0.014) also had statistical significant association with gender. A total of 82 (72.6%) male and 31(27.4%) female patients treatment outcome were successful, 21(87.5%) male and 3 (12.5%) female patients were defaulter, 12 (85.7%) male and 2 (14.3%) female patients were transferred out, 22 (81.5) male and 5 (18.5) female patients expired whereas 9 (75%) male and 3 (25%) female patients treatment was continued. Conclusion: Multiple factors are associated with patient gender in the development of anti- tubercle drug induced hepatitis. Therefore careful monitoring and regular patient assessment can optimize TB treatment outcomes in such patients.

Institution

(Nadarajah, Khan, Aftab, Ahmad) Department of Clinical Pharmacy, School of Pharmaceutical Sciences Universiti Sains Malaysia, Penang 11800, Malaysia (Hadi) Monash University Malaysia, Bandar Sunway 47500, Malaysia

Publisher

Blackwell Publishing

Emtree Heading

*treatment outcome; *tuberculosis; *toxic hepatitis; *patient; *human; *Asian; *society; *sex difference; female; male; implantable cardioverter defibrillator; gender; liver toxicity; hepatitis; intravenous drug abuse; alcohol consumption; smoking habit; clinical study; therapy; mortality; information processing; drug therapy; medical record; Malaysia; betel nut; cohort analysis; morbidity; patient assessment; diabetes mellitus; monitoring; hospital; data analysis software.

Link to the Ovid Full Text or citation:

Click here for full text options

40.

Impact of diabetes on treatment outcomes and long-term survival in multidrug-resistant tuberculosis.

Kang Y.A., Kim S.Y., Jo K.-W., Kim H.J., Park S.-K., Kim T.-H., Kim E.K., Lee K.M., Lee S.S., Park J.S., Koh W.-J., Kim D.Y., Shim T.S.

Respiration. 86 (6) (pp 472-478), 2013. Date of Publication: 2013.

AN: 2015947462

Background: Few studies have investigated the impact of diabetes mellitus (DM), a globally increasing metabolic disease, on treatment outcomes and long-term survival in patients with multidrug-resistant forms of tuberculosis (MDR-TB). Objectives: We analyzed outcomes in a large cohort to assess the impact of DM on treatment outcomes of patients with MDR-TB. Methods: MDR-TB patients newly diagnosed or retreated between 2000 and 2002 and followed for 8-11 years were retrospectively analyzed with respect to the effect of DM as a comorbidity on their treatment outcome and long-term survival. Results: Of 1,407 patients with MDR-TB, 239 (17.0%) had coexisting DM. The mean age and body mass index were higher in MDR-TB patients with DM [MDR-TBDM(+)] than in those without DM [MDR-TBDM(-)]. Patients with MDR-TB and a comorbidity of DM had a significantly lower treatment success rate than those without a history of DM (36.0 vs. 47.2%, p = 0.002). In addition, DM was the negative predictor for MDR-TB treatment success in multivariate analyses [odds ratio 0.51, 95% confidence interval (CI) 0.26-0.99]. Mean survival times were also lower in MDR-TBDM(+) than in MDR-TBDM(-) patients (102 vs. 114 months, p = 0.001), with DM as a significant predictor of poor long-term survival in multivariate analyses (hazard ratio 1.59, 95% CI 1.01-2.50). Conclusions: Among MDR-TB patients, DM was a relatively common comorbidity. In patients undergoing treatment for MDR-TB and followed for 8-11 years, it was found to be independently associated with an increased risk of both treatment failure and death.

Institution

(Kang, Kim) Department of Internal Medicine, Yonsei University College of Medicine, Asan Medical Center, Seoul, South Korea (Jo, Shim) Division of Pulmonary and Critical Care Medicine, University of Ulsan College of Medicine, Asan Medical Center, 388-1 Pungnap-dong, Songpa-gu, Seoul, South Korea

(Kim) Department of Epidemiology, Korean Institute of Tuberculosis, Sungkyunkwan University School of Medicine, Seoul, South Korea

(Kim) Department of Internal Medicine, Hanyang University College of Medicine, Sungkyunkwan University School of Medicine, Seoul, South Korea

(Koh) Department of Medicine, Samsung Medical Center, Sungkyunkwan University School of Medicine, Seoul, South Korea

(Park) Clinical Research Center, National Masan Hospital, Masan, South Korea

(Kim) Department of Internal Medicine, College of Medicine, Pochon CHA University, Seongnam, South Korea

(Lee) Department of Internal Medicine, Chungbuk National University College of Medicine, Chungbuk National University Hospital, Cheongju, South Korea

(Lee) Department of Internal Medicine, Inje University School of Medicine, Ilsan Paik Hospital, Goyang, South Korea

(Park) Department of Internal Medicine, Dankook University College of Medicine, Dankook University Hospital, Cheonan, South Korea

(Kim) Department of Thoracic Surgery, National Mokpo Hospital, Mokpo, South Korea

Publisher

S. Karger AG

Emtree Heading

acid fast bacterium; adult; aged; article; bacterium culture; body mass; chronic kidney disease; chronic lung disease; cohort analysis; comorbidity; connective tissue disease; controlled study; *diabetes mellitus; diabetic patient; drug sensitivity; extensively drug resistant tuberculosis; female; human; hyperthyroidism; hypothyroidism; Korean (people); long term survival; major clinical study; male; malnutrition; mental disease; *multidrug resistant tuberculosis; outcome assessment; postgastrectomy syndrome; priority journal; randomized controlled trial; retrospective study; short course therapy; survival time; thorax radiography; treatment duration.

Link to the Ovid Full Text or citation:

Click here for full text options

41.

Tuberculosis and diabetes mellitus: Time for an integrated approach.

Babu S., Salve H., Krishnan A.

National Medical Journal of India. 26 (6) (pp 342-343), 2013. Date of Publication: November/December 2013.

AN: 2014479332

Institution

(Babu, Salve, Krishnan) Centre for Community Medicine, All India Institute of Medical Sciences, New Delhi, India

Publisher

All India Institute of Medical Sciences

Emtree Heading

Australia; cohort analysis; comorbidity; *diabetes mellitus; disease association; disease predisposition; disease registry; disease transmission; dose response; health center; human; impaired glucose tolerance; *integrated health care system; mass screening; note; opportunistic infection; prevalence; relapse; sputum culture; treatment outcome; *tuberculosis; hemoglobin A1c/ec [Endogenous Compound].

Link to the Ovid Full Text or citation:

Click here for full text options

42.

Prevalence of type 2 diabetes among newly detected pulmonary tuberculosis patients in china: A community based cohort study.

Wang Q., Ma A., Han X., Zhao S., Cai J., Ma Y., Zhao J., Wang Y., Dong H., Zhao Z., Wei L., Yu T., Chen P., Schouten E.G., Kok F.J., Kapur A.

PLoS ONE. 8 (12) (no pagination), 2013. Article Number: e82660. Date of Publication: 18 Dec 2013.

AN: 2014080630

Background: Patients with type 2 diabetes (DM) have a higher risk of developing pulmonary tuberculosis (PTB); moreover, DM co-morbidity in PTB is associated with poor PTB treatment outcomes. Community based prevalence data on DM and prediabetes (pre-DM) among TB patients is lacking, particularly from the developing world. Therefore we conducted a prospective study to investigate the prevalence of DM and pre-DM and evaluated the risk factors for the presence of DM among newly detected PTB patients in rural areas of China. Methods and Findings: In a prospective community based study carried out from 2010 to 2012, a representative sample of 6382 newly detected PTB patients from 7 TB clinics in Linyi were tested for DM. A population of 6674 non-TB controls from the same community was similarly tested as well. The prevalence of DM in TB patients (6.3%) was higher than that in non-TB controls (4.7%, p<0.05). PTB patients had a higher odds of DM than non-TB controls (adjusted OR 3.17, 95% CI 1.14-8.84). The prevalence of DM increased with age and was significantly higher in TB patients in the age categories above 30 years (p<0.05). Among TB patients, those with normal weight (BMI 18.5-23.9) had the lowest prevalence of DM (5.8%). Increasing age, family history of DM, positive sputum smear, cavity on chest X-ray and higher yearly income (>10000 RMB yuan) were positively associated and frequent outdoor activity was negatively associated with DM in PTB patients. Conclusions: The prevalence of DM in PTB patients was higher than in non-TB controls with a 3 fold higher adjusted odds ratio of having DM. Given the increasing DM prevalence and still high burden of TB in China, this association may represent a new public health challenge concerning the prevention and treatment of both diseases. © 2013 Wang et al.

Institution

(Wang, Ma, Han, Cai) Nutrition Institute, Qingdao University, Qingdao, China (Zhao, Ma) Linyi Chest Hospital, Linyi, China

(Zhao) Yishui Tuberculosis Clinic, Linyi, China

(Wang) Tancheng Tuberculosis Clinic, Linyi, China

(Dong) Yinan Tuberculosis Clinic, Linyi, China

(Zhao) Lanshan Tuberculosis Clinic, Linyi, China

(Wei) Feixian Tuberculosis Clinic, Linyi, China

(Yu) Pingyi Tuberculosis Clinic, Linyi, China

(Chen) Cangshan Tuberculosis Clinic, Linyi, China

(Schouten, Kok) Division of Human Nutrition, Wageningen University, Wageningen, Netherlands

(Kapur) World Diabetes Foundation, Gentofte, Denmark

Publisher

Public Library of Science (185 Berry Street, Suite 1300, San Francisco CA 94107, United States)

Emtree Heading

adult; age distribution; article; body mass; China; cholesterol blood level; cohort analysis; controlled study; family history; female; glucose blood level; human; income; lung cavity; *lung tuberculosis; major clinical study; male; *non insulin dependent diabetes mellitus/ep [Epidemiology]; obesity; prevalence; prospective study; risk assessment; risk factor; rural area; sputum smear; triacylglycerol blood level; underweight; cholesterol/ec [Endogenous Compound]; glucose/ec [Endogenous Compound]; triacylglycerol/ec [Endogenous Compound].

Link to the Ovid Full Text or citation:

Click here for full text options

43.

Poor performance status is a strong predictor for death in patients with smear-positive pulmonary TB admitted to two Japanese hospitals.

Horita N., Miyazawa N., Yoshiyama T., Kojima R., Omori N., Kaneko T., Ishigatsubo Y.

Transactions of the Royal Society of Tropical Medicine and Hygiene. 107 (7) (pp 451-456), 2013. Article Number: trt037. Date of Publication: July 2013.

AN: 2013767794

Background: Estimation of performance status (PS) has been assessed as a tool to determine which patients with newly diagnosed pulmonary TB (PTB) are most at risk of dying. This simple prediction rule has not been validated in patients with PTB with different background characteristics and from different geographic areas. Methods: A retrospective cohort study was conducted in two Japanese hospitals in different regions and included 432 inpatients with newly diagnosed smear-positive non-multidrug-resistant lung TB without HIV infection. The patients had a mean +/- SD age of 64.9 +/- 19.7 years and 135 were female (31.3%). Detailed nursing charts were reviewed to estimate PS, which was graded 0 (best condition), 1, 2, 3 or 4 (worst condition), for each patient. Results: Single variable and multivariable Cox regression analyses models revealed that a one-point increase in PS was associated with a 2.8-fold (95% CI 2.2-3.6) and 2.3-fold (95% CI 1.8-3.0), adjusted for age, gender, comorbidities and treatment regimen, increase in the HR for death (p < 0.001 for both models). Kaplan-Meier curves also showed a significant difference in mortality among different PS groups (p < 0.001). Conclusion: PS was strongly associated with mortality from PTB in the study cohort. Estimation of PS at the start of treatment for newly diagnosed PTB patients could be a useful tool in case management in resource-limited countries. © Royal Society of Tropical Medicine and Hygiene 2013. All rights reserved.

Institution

(Horita, Ishigatsubo) Department of Internal Medicine and Clinical Immunology, Yokohama City University Graduate School of Medicine, Yokohama, Japan (Horita, Miyazawa, Kojima, Omori) Department of Respiratory Medicine, Saiseikai Yokohamashi Nanbu Hospital, Yokohama, Japan

(Yoshiyama) Division of Respiratory Medicine, Japan Antituberculosis Association Fukujuji Hospital, Tokyo, Japan

(Kaneko) Respiratory Disease Center, Yokohama City University Medical Center, Yokohama, Japan

Publisher

Oxford University Press (Great Clarendon Street, Oxford OX2 6DP, United Kingdom)

Emtree Heading

adult; aged; article; cause of death; chronic kidney disease; chronic liver disease; chronic respiratory tract disease; clinical observation; cohort analysis; comorbidity; daily life activity; diabetes mellitus; dying; female; follow up; *health status; heart disease; human; Japanese; *lung tuberculosis; major clinical study; male; *mortality; *performance status; prognosis; retrospective study; treatment outcome; X ray; aspartate aminotransferase/ec [Endogenous Compound]; creatinine/ec [Endogenous Compound]; hemoglobin/ec [Endogenous Compound].

Link to the Ovid Full Text or citation:

Click here for full text options

44.

Profile of HIV-infected patients receiving second-line antiretroviral therapy in a resource-limited setting in Nigeria.

Onyedum C.C., Iroezindu M.O., Chukwuka C.J., Anyaene C.A., Obi F.I., Young E.E.

Transactions of the Royal Society of Tropical Medicine and Hygiene. 107 (10) (pp 608-614), 2013. Article Number: trt071. Date of Publication: October 2013.

AN: 2013767756

Background: Second-line antiretroviral therapy (ART) accounts for lessthan5%of totalARTin resource-limited settings. We described the baseline characteristics, reasons for switch and treatment outcomes of Nigerian patients receiving second-line ART. Methods: In this retrospective cohort study we recorded the baseline characteristics of HIV-infected adults whose treatment regimen was switched from a non-nucleoside reverse transcriptase inhibitor, a first-line agent, to a protease inhibitor-based second-line regimen. The duration of follow-up was 12 months. Results: Of 4229 patients who started first-line therapy, 186 (4.4%) were switched to second-line therapy after a mean duration of 16.6+/-7.6 months. Their mean age was 41.8+/-9.6 years and 59.1% were women. The median (range) viral load andCD4 cell counts at switch were 4.7 (4.1-6.3) log10 copies/ml and 71 (6-610) cells/mul, respectively. The predominantreason for switchwas virological failure (79.0%). Only55.4%and36.6%of patientshadCD4 cell count and viral load at 12 months. About 82%, 79% and 82% of patients with available data achieved virological suppression at 3 months, 6 months and 12 months respectively (p 1/4 0.81). The proportion of patients who achieved >50%rise in CD4 cell count increased from55.8% at 3 months to 78.6% at 12 months (p 1/4 0.0002). Conclusion: The rate of switch to second-line therapy was low but there were good treatment outcomes among patients with available data. Attrition rate was high. Regular viral load monitoring, improved availability/affordability of second-line regimens and retention in care should become priorities in resource-limited settings. © The Author 2013. Published by Oxford University Press on behalf of Royal Society of Tropical Medicine and Hygiene. All rights reserved.

Institution

(Onyedum, Iroezindu, Chukwuka, Young) Department of Medicine, College of Medicine, University of Nigeria Teaching Hospital (UNTH), Enugu, Nigeria (Anyaene) APIN/CDC Centre, UNTH, Nigeria

(Obi) Department of Community Medicine, UNTH, Nigeria

Publisher

Oxford University Press (Great Clarendon Street, Oxford OX2 6DP, United Kingdom)

Emtree Heading

adolescent; adult; alcohol consumption; antibiotic prophylaxis; antiviral resistance; *antiviral therapy; article; blood transfusion; CD4 lymphocyte count; cohort analysis; comorbidity; diabetes mellitus; drug substitution; drug treatment failure; educational status; female; follow up; heterosexuality; *highly active antiretroviral therapy; human; Human immunodeficiency virus 1; *Human immunodeficiency virus 1 infection/dt [Drug Therapy]; Human immunodeficiency virus 2; *Human immunodeficiency virus 2 infection/dt [Drug Therapy]; Human immunodeficiency virus infected patient; hypertension; immune response; limit of detection; major clinical study; male; marriage; medication compliance; Nigeria; nonhuman; patient compliance; pneumonia; retrospective study; *second line antiretroviral therapy; treatment duration; treatment outcome; tuberculosis; virus load; virus transmission; world health organization; cotrimoxazole; efavirenz plus emtricitabine plus tenofovir disoproxil/dt [Drug Therapy]; emtricitabine plus tenofovir disoproxil/cb [Drug Combination]; emtricitabine plus tenofovir disoproxil/dt [Drug Therapy]; *Human immunodeficiency virus proteinase inhibitor/dt [Drug Therapy]; lamivudine plus nevirapine plus stavudine/dt [Drug Therapy]; lamivudine plus nevirapine plus zidovudine/dt [Drug Therapy]; lopinavir plus ritonavir/cb [Drug Combination]; lopinavir plus ritonavir/dt [Drug Therapy]; *nonnucleoside reverse transcriptase inhibitor/dt [Drug Therapy]; tenofovir plus emtricitabine plus nevirapine/dt [Drug Therapy]; unclassified drug; virus RNA/ec [Endogenous Compound]; zidovudine/cb [Drug Combination]; zidovudine/dt [Drug Therapy].

Link to the Ovid Full Text or citation:

Click here for full text options

45.

Clinical and laboratory characteristics of penicilliosis marneffei among patients with and without HIV infection in Northern Thailand: A retrospective study.

Kawila R., Chaiwarith R., Supparatpinyo K.

BMC Infectious Diseases. 13 (1) (no pagination), 2013. Article Number: 464. Date of Publication: 05 Oct 2013.

AN: 2013718369

Background: Penicilliosis marneffei is increasingly observed in individuals without HIV infection. This study aimed to compare the clinical and laboratory features among HIV infected and uninfected individuals with penicilliosis marneffei.Methods: A retrospective cohort study was conducted between January 1, 2007 and December 31, 2011 at Chiang Mai University Hospital. We included individuals who were >15 years of age and presented with culture-proven P. marneffei infection.Results: 116 HIV-infected and 34 HIV-uninfected patients were enrolled. Comparing to HIV-infected patients, HIV-uninfected patients were older; less likely to have fever, splenomegaly, and umbilicated skin lesions; more likely to have Sweet's syndrome and bone and joint infections; had higher white blood cell count, platelet count, and CD4 cell count; had lower alanine transaminase (ALT); and less likely to have positive fungal blood cultures. The mortality rates were 20.7% and 29.4% among HIV infected and uninfected patients, respectively.Conclusions: Clinical manifestations of penicilliosis marneffei are different between patients with and without HIV infection. Physician's awareness of this disease in HIV-uninfected patients may prompt the diagnosis and timely treatment, and can lead to a better outcome. © 2013 Kawila et al.; licensee BioMed Central Ltd.

Institution

(Kawila, Chaiwarith, Supparatpinyo) Department of Medicine, Faculty of Medicine, Chiang Mai University, Chiang Mai 50200, Thailand (Supparatpinyo) Research Institute for Health Sciences, Faculty of Medicine, Chiang Mai University, Chiang Mai, Thailand

Publisher

BioMed Central Ltd. (Floor 6, 236 Gray's Inn Road, London WC1X 8HB, United Kingdom)

Emtree Heading

acute febrile neutrophilic dermatosis; adolescent; adult; age; article; bone infection; candidiasis/co [Complication]; CD4 lymphocyte count; clinical feature; cohort analysis; colon cancer; comorbidity; convalescence; cryptococcosis/co [Complication]; cytomegalovirus infection/co [Complication]; diabetes mellitus; female; fever; fungus culture; herpes zoster/co [Complication]; histoplasmosis/co [Complication]; human; *Human immunodeficiency virus infection; infectious arthritis; isosporiasis/co [Complication]; leukocytosis; lymphoma; major clinical study; male; mixed connective tissue disease/dt [Drug Therapy]; mortality; myasthenia gravis; nocardiosis/co [Complication]; opportunistic infection/co [Complication]; *penicilliosis/di [Diagnosis]; *penicilliosis/dt [Drug Therapy]; *penicilliosis/et [Etiology]; Penicillium marneffei; retrospective study; salmonellosis/co [Complication]; skin defect; splenomegaly; systemic lupus erythematosus/dt [Drug Therapy]; Thailand; thrombocytosis; treatment duration; treatment outcome; tuberculosis/co [Complication]; university hospital; alanine aminotransferase/ec [Endogenous Compound]; amphotericin B deoxycholate/dt [Drug Therapy]; amphotericin B deoxycholate/iv [Intravenous Drug Administration]; azathioprine/dt [Drug Therapy]; CD4 antigen/ec [Endogenous Compound]; itraconazole/dt [Drug Therapy]; itraconazole/po [Oral Drug Administration]; prednisolone/dt [Drug Therapy].

Link to the Ovid Full Text or citation:

Click here for full text options

46.

Nutritional Status of Adult Patients with Pulmonary Tuberculosis in Rural Central India and Its Association with Mortality.

Bhargava A., Chatterjee M., Jain Y., Chatterjee B., Kataria A., Bhargava M., Kataria R., D'Souza R., Jain R., Benedetti A., Pai M., Menzies D.

PLoS ONE. 8 (10) (no pagination), 2013. Article Number: e77979. Date of Publication: 24 Oct 2013.

AN: 2013674017

Under-nutrition is a known risk factor for TB and can adversely affect treatment outcomes. However, data from India are sparse, despite the high burden of TB as well as malnutrition in India. We assessed the nutritional status at the time of diagnosis and completion of therapy, and its association with deaths during TB treatment, in a consecutive cohort of 1695 adult patients with pulmonary tuberculosis in rural India during 2004 - 2009.Multivariable logistic regression was used to obtain adjusted estimates of the association of nutritional status with deaths during treatment. At the time of diagnosis, median BMI and body weights were 16.0 kg/m2and 42.1 kg in men, and 15.0 kg/m2and 34.1 kg in women, indicating that 80% of women and 67% of men had moderate to severe under-nutrition (BMI<17.0 kg/m2). Fifty two percent of the patients (57% of men and 48% of women) had stunting indicating chronic under-nutrition. Half of women and one third of men remained moderately to severely underweight at the end of treatment. 60 deaths occurred in 1179 patients (5%) in whom treatment was initiated. Severe under-nutrition at diagnosis was associated with a 2 fold higher risk of death. Overall, a majority of patients had evidence of chronic severe under-nutrition at diagnosis, which persisted even after successful treatment in a significant proportion of them. These findings suggest the need for nutritional support during treatment of pulmonary TB in this rural population. © 2013 Bhargava et al.

Institution

(Bhargava) Department of Internal Medicine, Himalayan Institute of Medical Sciences, Jolly Grant, Uttarakhand, India (Chatterjee) Krishak Maitri Hospital, Chengail, West Bengal, India

(Jain, Kataria, Kataria, Jain) Jan Swasthya Sahyog, Village and P.O.Ganiyari, Chhattisgarh, India

(Chatterjee) Department of Microbiology, Himalayan Institute of Medical Sciences, Jolly Grant, Uttarakhand, India

(Bhargava) Department of Community Medicine, Himalayan Institute of Medical Sciences, Jolly Grant, Uttarakhand, India

(D'Souza) Centre for Public Health and Equity, Bhopal, Madhya Pradesh, India

(Benedetti, Menzies) Respiratory Epidemiology and Clinical Research Unit, Montreal Chest Institute, McGill University Health Centre, Montreal, QC, Canada

(Pai) Department of Epidemiology, Biostatistics and Occupational Health, McGill University, Montreal, QC, Canada

Publisher

Public Library of Science (185 Berry Street, Suite 1300, San Francisco CA 94107, United States)

Emtree Heading

adult; age distribution; anemia; article; body height; body mass; body weight; cohort analysis; comorbidity; controlled study; diabetes mellitus; disease association; female; human; Human immunodeficiency virus infection; India; *lung tuberculosis/ep [Epidemiology]; major clinical study; male; malnutrition; *mortality; *nutritional status; nutritional support; prevalence; retrospective study; rural area; sex difference; treatment outcome; underweight.

Link to the Ovid Full Text or citation:

Click here for full text options

47.

Adalimumab for the treatment of psoriasis in real life: A retrospective cohort of 119 patients at a single Spanish centre.

Lopez-Ferrer A., Vilarrasa E., Gich I.J., Puig L.

British Journal of Dermatology. 169 (5) (pp 1141-1147), 2013. Date of Publication: November 2013.

AN: 2013696878

Background Patients with moderate-to-severe psoriasis treated with adalimumab in daily clinical practice are different from those in clinical trials, and outcomes may differ in different geographical settings. Objectives To analyse the efficacy, retention of treatment and adverse events in a cohort of such patients at a referral centre in Barcelona, Spain. Methods Data from a cohort of 119 consecutive patients treated between January 2008 and March 2013 were retrospectively collected. Drug survival was analysed by the Kaplan-Meier method with log-rank test and Cox regression. Results The mean duration of treatment was 25 months (median 22, range 2-60). The 75% improvement in Psoriasis Area and Severity Index (PASI 75) response rates at 16 weeks, 6 months and 1 year of treatment were 64%, 58% and 53%, respectively (intention-to-treat analysis). The corresponding PASI 90 values were 49%, 52% and 50%. Biologic-naive patients (41%) had significantly higher PASI 75 and PASI 90 response rates at 6 months and 1 year. On multivariate analysis, only PASI 90 response at 6 months was significantly associated with treatment retention (P = 0.0009), with a hazard ratio of 7.3 (95% confidence interval 2.3-23.6). Forty-eight adverse events (AEs) occurred in 29 patients, and were serious in eight (0.032 events per patient-year). Paradoxical flares of psoriasis or arthritis were seen in five patients. Infections accounted for seven serious AEs, and were the reason for discontinuation in two patients. Conclusions PASI 90 response at 6 months was the only independent variable predicting drug survival on multivariate analysis. Infections, including de novo infection by Mycobacterium tuberculosis, accounted for seven serious AEs. What's already known about this topic? There are few reports on the use of adalimumab for the treatment of moderate-to-severe psoriasis in clinical practice according to the European Medicines Agency. Psoriasis Area and Severity Index (PASI) 75% response rates at 16 weeks and 6 months were approximately 60% in a previously published U.K. series. Male sex and the presence of arthritis have been associated with decreased drug survival in one study. What does this study add? Biologic-naive status and efficacy parameters denoting a good or excellent response appear to be associated with a higher probability of drug survival. Combination treatment increased PASI response rates at 6 months and might provide an explanation for the relatively high rate of PASI 90 responders in our cohort. Infections, including de novo infection by Mycobacterium tuberculosis, accounted for most serious adverse events, and paradoxical flares of psoriasis and psoriatic arthritis were relatively frequent. © 2013 British Association of Dermatologists.

Institution

(Lopez-Ferrer, Vilarrasa, Puig) Department of Dermatology, Hospital de la Santa Creu i Sant Pau, Universitat Autonoma de Barcelona, Sant Antoni Maria Claret 167, 08025 Barcelona, Catalonia, Spain (Gich) Department of Clinical Epidemiology and Public Health, Hospital de la Santa Creu i Sant Pau, Universitat Autonoma de Barcelona, Sant Antoni Maria Claret 167, 08025 Barcelona, Catalonia, Spain

Publisher

Blackwell Publishing Ltd (9600 Garsington Road, Oxford OX4 2XG, United Kingdom)

Emtree Heading

abscess/si [Side Effect]; adult; aged; alcoholism; article; bronchitis/si [Side Effect]; cohort analysis; comparative study; controlled study; diabetes mellitus; disease course; drug efficacy; drug safety; drug withdrawal; dyslipidemia; female; furunculosis/si [Side Effect]; gastroenteritis/si [Side Effect]; heart infarction/si [Side Effect]; herpes simplex/si [Side Effect]; human; hyperglycemia/si [Side Effect]; hypertension/si [Side Effect]; hypertriglyceridemia/si [Side Effect]; injection site reaction/si [Side Effect]; intention to treat analysis; latent tuberculosis; leukopenia/si [Side Effect]; major clinical study; male; medical record review; miliary tuberculosis/si [Side Effect]; muscle cramp/si [Side Effect]; nonalcoholic fatty liver; obesity; open study; paronychia/si [Side Effect]; patient monitoring; patient referral; pneumonia/si [Side Effect]; priority journal; prostatitis/si [Side Effect]; pruritus/si [Side Effect]; *psoriasis/dt [Drug Therapy]; Psoriasis Area and Severity Index; psoriasis vulgaris; psoriatic arthritis/di [Diagnosis]; psoriatic arthritis/dt [Drug Therapy]; retrospective study; scabies/si [Side Effect]; Spain; survival; thrombocytopenia/si [Side Effect]; thrush/si [Side Effect]; treatment duration; treatment outcome; treatment response; tuberculosis/dt [Drug Therapy]; urinary tract infection/si [Side Effect]; vasculitis/si [Side Effect]; *adalimumab/ae [Adverse Drug Reaction]; *adalimumab/ct [Clinical Trial]; *adalimumab/cb [Drug Combination]; *adalimumab/dt [Drug Therapy]; *adalimumab/pd [Pharmacology]; cyclosporin A/cb [Drug Combination]; cyclosporin A/dt [Drug Therapy]; etretin/cb [Drug Combination]; etretin/dt [Drug Therapy]; infliximab/dt [Drug Therapy]; leflunomide/dt [Drug Therapy]; methotrexate/cb [Drug Combination]; methotrexate/dt [Drug Therapy]; tuberculostatic agent/dt [Drug Therapy].

Link to the Ovid Full Text or citation:

Click here for full text options

48.

Diabetes mellitus and tuberculosis: Pattern of tuberculosis, two-month smear conversion and treatment outcomes in Guangzhou, China.

Mi F., Tan S., Liang L., Harries A.D., Hinderaker S.G., Lin Y., Yue W., Chen X., Liang B., Gong F., Du J.

Tropical Medicine and International Health. 18 (11) (pp 1379-1385), 2013. Date of Publication: November 2013.

AN: 2013674768

Objective: There is a high burden of both diabetes (DM) and tuberculosis (TB) in China. We evaluated the association between DM and the pattern of disease, 2-month sputum smear conversion and treatment outcomes of patients with TB in Guangzhou, China. Method: All patients registered with TB from September 2011 to June 2012 were screened for DM and assessed for treatment outcomes in relation to presence or absence of DM and quality of DM control using patient registers, treatment cards and electronic record systems. Results: There were 1589 patients with TB of whom 189 (12%) had DM. Among those with DM, there was a significantly higher proportion of men, persons aged 35 years and older and persons with smear-positive pulmonary tuberculosis (PTB) (P < 0.01). In patients with DM and new smear-positive PTB, there was a higher proportion who had positive sputum smears at 2 months (21.7% vs. 5.6%, RR 3.85, 95%CI 2.24-6.63), who were lost-to-follow-up (5.2% vs. 1.7%, RR 3.23, 95%CI 1.08-9.63) and who failed treatment (10.3% vs. 2.3%, RR 4.46, 95%CI 1.96-10.18) compared with patients who had no DM. There was no significant association between these adverse outcomes and DM control as measured by 2 and 6-month fasting blood glucose. Conclusion: Diabetes mellitus in new smear-positive patients with PTB was associated with failure to sputum smear convert at 2 months and adverse treatment outcomes of loss-to-follow-up and failure. Further research is needed to understand the reasons for these findings and to determine whether the current length of treatment of 6 months is adequate. © 2013 John Wiley & Sons Ltd.

Institution

(Mi, Liang, Yue, Chen, Du) Beijing Chest Hospital, Capital Medical University, Beijing, China (Mi, Liang, Yue, Chen, Du) Beijing Tuberculosis and Thoracic Tumor Research Institute, Beijing, China

(Tan, Liang, Gong) Guangzhou Chest Hospital, Guangzhou, China

(Tan, Liang, Gong) State Key Laboratory of Respiratory Diseases, Guangzhou, China

(Harries) International Union Against Tuberculosis and Lung Disease, Paris, France

(Harries) London School of Hygiene and Tropical Medicine, London, United Kingdom

(Hinderaker) University of Bergen, Bergen, Norway

(Lin) The China Union Office, Beijing, China

Publisher

Blackwell Publishing Ltd (9600 Garsington Road, Oxford OX4 2XG, United Kingdom)

Emtree Heading

adult; antibiotic therapy; article; China; clinical feature; cohort analysis; cross-sectional study; demography; diabetes control; *diabetes mellitus/ep [Epidemiology]; disease association; female; follow up; glucose blood level; human; *lung tuberculosis/dt [Drug Therapy]; *lung tuberculosis/ep [Epidemiology]; major clinical study; male; prevalence; retrospective study; sputum smear; treatment outcome; ethambutol/cb [Drug Combination]; ethambutol/dt [Drug Therapy]; glucose/ec [Endogenous Compound]; isoniazid/cb [Drug Combination]; isoniazid/dt [Drug Therapy]; pyrazinamide/cb [Drug Combination]; pyrazinamide/dt [Drug Therapy]; rifampicin/cb [Drug Combination]; rifampicin/dt [Drug Therapy].

Link to the Ovid Full Text or citation:

Click here for full text options

49.

The impact of diabetes mellitus and its control on the development of tuberculosis: A nationwide longitudinal study in Taiwan.

Lee M.-C., Lee C.-H., Shu C.-C., Pong W.-B., Lan C.-C., Wang J.-Y., Lee L.-N., Chao K.-M.

Pharmacoepidemiology and Drug Safety. 22 (9) (pp 995-1003), 2013. Date of Publication: September 2013.

AN: 2013527414

Purpose: Diabetic mellitus (DM) is a well-known risk factor of tuberculosis (TB). However, there is paucity of reports on the impact of diabetic control and adherence to anti-diabetic treatment on the risk of TB. This nationwide cohort study aimed to address these issues. Methods: In the Longitudinal Health Insurance Database 2005 of Taiwan, DM cases and matched control subjects were selected. Potential risk factors for developing TB were investigated using the Cox proportional hazards regression analysis. Time-dependent variables were used to measure drug prescription, adherence, and number of DM-associated admissions from 360 to 90days prior to each outcome event. Results: The 49903 DM patients identified (52.5% males) had a mean age of 61.9+/-14.2years. Among DM patients and control subjects, the independent risk factors of TB were age, male sex, chronic obstructive pulmonary disease (COPD), autoimmune disease, and DM (hazard ratio [HR] 1.293 [1.154-1.449]). Among DM patients, time-dependent Cox analysis revealed that age, male sex, COPD, end-stage renal disease, maximum average daily dose of oral hypoglycemic agent (HR 1.13 [1.071-1.193]), insulin use during admission (HR 1.462 [1.087-1.966]), adherence to anti-diabetic medication (0.577 [0.429-0.776]), and number of DM-associated admissions (1.789 [1.231-2.601]) were independent factors associated with the development of TB. Conclusions: The risk of TB parallels DM severity. Some cases of TB can be prevented by fostering adherence to anti-diabetic medication. © 2013 John Wiley & Sons, Ltd.

Institution

(Lee) Department of Pharmacy, Buddhist Tzu Chi General Hospital, Taipei Branch, Taiwan (Republic of China) (Lee, Chao) Graduate Institute of Biomedical Electronics and Bioinformatics, National Taiwan University, Taipei, Taiwan (Republic of China)

(Lee, Pong, Lan) Department of Internal Medicine, Buddhist Tzu Chi General Hospital, Taipei Branch, Taiwan (Republic of China)

(Lee, Lan) School of Medicine, Tzu Chi University, Hualien, Taiwan (Republic of China)

(Shu) Department of Traumatology, National Taiwan University Hospital, Taipei, Taiwan (Republic of China)

(Wang) Department of Internal Medicine, National Taiwan University Hospital, Taipei, Taiwan (Republic of China)

(Lee) Department of Laboratory Medicine, National Taiwan University Hospital, Taipei, Taiwan (Republic of China)

Publisher

John Wiley and Sons Ltd (Southern Gate, Chichester, West Sussex PO19 8SQ, United Kingdom)

Emtree Heading

adult; age; aged; article; autoimmune disease; chronic obstructive lung disease; cohort analysis; comorbidity; controlled study; diabetes control; *diabetes mellitus/dt [Drug Therapy]; diabetic patient; drug megadose; female; follow up; gender; hospital admission; human; insulin dependent diabetes mellitus; kidney failure; liver cirrhosis; longitudinal study; lowest income group; major clinical study; male; medication compliance; organ transplantation; outcome assessment; prescription; priority journal; risk factor; Taiwan; *tuberculosis/dt [Drug Therapy]; *tuberculosis control; corticosteroid; insulin/dt [Drug Therapy]; oral antidiabetic agent/dt [Drug Therapy]; prescription drug; tuberculostatic agent/dt [Drug Therapy].

Link to the Ovid Full Text or citation:

Click here for full text options

50.

Selective uptake of influenza vaccine and pregnancy outcomes.

Cantu J., Biggio J., Jauk V., Wetta L., Andrews W., Tita A.

Journal of Maternal-Fetal and Neonatal Medicine. 26 (12) (pp 1207-1211), 2013. Date of Publication: August 2013.

AN: 2013447781

Objective: To describe the characteristics of pregnant women who accept the influenza vaccine and evaluate the relationship between vaccination and adverse pregnancy outcomes. Methods: Retrospective cohort study of women receiving prenatal care during the 2009-2011 influenza seasons. Vaccination status was ascertained through our perinatal record system and clinic vaccination logs. Pregnancy outcomes included a primary composite of miscarriage, fetal demise, preterm birth (PTB) <37 weeks and neonatal demise. Stratification and logistic regression were used to adjust for potential confounders. Results: Of 3104 eligible pregnant women, 1094 (35%) received the influenza vaccine. Women vaccinated were more likely to be older, obese, primiparae, and have medical complications or a prior PTB. In univariable analyses, flu vaccination was associated with increased adverse composite outcome and PTB. After multivariable adjustments, vaccination was no longer associated with adverse outcomes in women with medical complications but remained associated with adverse outcomes among those without known co-morbidity. Conclusions: Vaccination was associated with an increased adverse composite outcome in pregnant women without identified co-morbidity but not those with co-morbidities. This association is likely due to selection bias, which should be considered in planning of observational studies of the impact of vaccination on pregnancy outcomes. © 2013 Informa UK Ltd. All rights reserved: reproduction in whole or part not permitted.

Institution

(Cantu, Biggio, Jauk, Wetta, Andrews, Tita) Center for Women's Reproductive Health, University of Alabama at Birmingham, Birmingham, AL, United States

Publisher

Informa Healthcare (69-77 Paul Street, London EC2A 4LQ, United Kingdom)

Emtree Heading

adult; age distribution; antiviral therapy; article; clinical feature; cohort analysis; *drug uptake; female; fetus death; gestational age; hepatitis C/co [Complication]; human; Human immunodeficiency virus infection; influenza/dt [Drug Therapy]; influenza/pc [Prevention]; influenza vaccination; low birth weight; lung tuberculosis; lupus vulgaris/co [Complication]; maternal hypertension/co [Complication]; medical record; newborn death; obesity; outcome assessment; preeclampsia; pregnancy complication; pregnancy diabetes mellitus/co [Complication]; *pregnancy outcome; pregnant woman; premature labor; prenatal care; primipara; priority journal; retrospective study; sickle cell anemia/co [Complication]; small for date infant; spontaneous abortion; stillbirth; *influenza vaccine/dt [Drug Therapy]; oseltamivir/dt [Drug Therapy].

Link to the Ovid Full Text or citation:

Click here for full text options

51.

Beyond pregnancy - The neglected burden of mortality in young women of reproductive age in Bangladesh: A prospective cohort study.

Labrique A.B., Sikder S.S., Wu L., Rashid M., Ali H., Ullah B., Shamim A.A., Mehra S., Klemm R., Banu H., West Jr. K.P., Christian P.

BJOG: An International Journal of Obstetrics and Gynaecology. 120 (9) (pp 1085-1089), 2013. Date of Publication: August 2013.

AN: 2013441665

Objective To describe proportionate mortality and causes of death unrelated to pregnancy. Design Prospective cohort study. Setting Rural northwest Bangladesh. Population A cohort of 133 617 married women of reproductive age. Methods Verbal autopsies were conducted for women who died whilst under surveillance in the cohort trial. Physician-assigned causes of death based on verbal autopsies were used to categorise deaths. Main outcome measures The proportion of deaths due to non-communicable diseases, infectious diseases, injury or pregnancy. Results Of the 1107 deaths occurring among women between 2001 and 2007, 48% were attributed to non-communicable diseases, 22% to pregnancy, 17% to infections, 9% to injury and 4% to other causes. Conclusions Although focus on pregnancy-related mortality remains important, more attention is warranted on non-communicable diseases among women of reproductive age. © 2013 The Authors BJOG An International Journal of Obstetrics and Gynaecology © 2013 RCOG.

Institution

(Labrique, Sikder, Wu, Mehra, Klemm, West Jr., Christian) Department of International Health, Johns Hopkins Bloomberg School of Public Health, 615 N. Wolfe St., Baltimore, MD 21205, United States (Rashid, Ali, Shamim) JiVitA Maternal and Child Health Research Project Gaibandha, Poschim Para, Bangladesh

(Ullah) Maternal and Neonatal Health Initiative, United Nations Population Fund Narail, Aladatpur, Bangladesh

(Banu) Department of Gynecology, Institute of Child and Mother Health Matuail, Dhaka, Bangladesh

Publisher

Blackwell Publishing Ltd (9600 Garsington Road, Oxford OX4 2XG, United Kingdom)

Emtree Heading

adult; anaphylactic shock; anemia; anorexia nervosa; asthma; autopsy; Bangladesh; bleeding; cause of death; cerebrovascular accident; cohort analysis; dehydration; diabetes mellitus; diarrhea; diphtheria; epilepsy; female; gastrointestinal disease; heart failure; heart infarction; homicide; human; infection; injury; ischemic heart disease; kidney disease; lung embolism; major clinical study; malaria; malnutrition; married woman; meningitis; *mortality; neoplasm; non communicable disease; outcome assessment; pericardial effusion; *pregnancy; priority journal; prospective study; rabies; respiratory tract infection; review; rheumatic disease; sepsis; suicide; tetanus; tonsillitis; tuberculosis; typhoid fever.

Link to the Ovid Full Text or citation:

Click here for full text options

52.

Diabetes is a strong predictor of mortality during tuberculosis treatment: A prospective cohort study among tuberculosis patients from Mwanza, Tanzania.

Faurholt-Jepsen D., Range N., Praygod G., Jeremiah K., Faurholt-Jepsen M., Aabye M.G., Changalucha J., Christensen D.L., Grewal H.M.S., Martinussen T., Krarup H., Witte D.R., Andersen A.B., Friis H.

Tropical Medicine and International Health. 18 (7) (pp 822-829), 2013. Date of Publication: July 2013.

AN: 2013391100

Objective: Strong evidence suggests diabetes may be associated with tuberculosis (TB) and could influence TB treatment outcomes. We assessed the role of diabetes on sputum culture conversion and mortality among patients undergoing TB treatment. Methods: A total of 1250 Tanzanian TB patients were followed prospectively during TB treatment with sputum culture after 2 and 5 months. Survival status was assessed at least 1 year after initiation of treatment. At baseline, all participants underwent testing for diabetes and HIV, and the serum concentration of the acute phase reactant alpha-1 glycoprotein (AGP) was determined. Results: There were no differences between participants with and without diabetes regarding the proportion of positive cultures at 2 (3.8% vs. 5.8%) and 5 (1.3% vs. 0.9%) months (P > 0.46). However, among patients with a positive TB culture, relatively more patients with diabetes died before the 5-month follow-up. Within the initial 100 days of TB treatment, diabetes was associated with a fivefold increased risk of mortality (RR 5.09, 95% CI 2.36; 11.02, P < 0.001) among HIV uninfected, and a twofold increase among HIV co-infected patient (RR 2.33 95% CI 1.20; 4.53, P = 0.012), while diabetes was not associated with long-term mortality. Further adjustment with AGP did not change the estimates. Conclusion: Diabetes considerably increases risk of early mortality during TB treatment. The effect may not be explained by increased severity of TB, but could be due to impaired TB treatment response. Research is needed to clarify the mechanism and to assess whether glycaemic control improves survival. © 2013 Blackwell Publishing Ltd.

Institution

(Faurholt-Jepsen, Faurholt-Jepsen, Friis) Department of Nutrition, Exercise and Sports, University of Copenhagen, Frederiksberg, Denmark (Range) Muhimbili Research Centre, National Institute for Medical Research, Dar Es Salaam, Tanzania

(Praygod, Jeremiah, Changalucha) Mwanza Research Centre, National Institute for Medical Research, Mwanza, Tanzania

(Aabye) Clinical Research Centre, University of Copenhagen, Hvidovre Hospital, Hvidovre, Denmark

(Christensen) Department of International Health, University of Copenhagen, Copenhagen, Denmark

(Grewal) The Gade Institute, University of Bergen, Bergen, Norway

(Martinussen) Biostatistics and Mathematics, University of Copenhagen, Copenhagen, Denmark

(Krarup) Department of Clinical Biochemistry, Aalborg University Hospital, Aalborg, Denmark

(Witte) Steno Diabetes Center, Gentofte, Denmark

(Andersen) Department of Infectious Diseases, Odense University Hospital, Odense, Denmark

Publisher

Blackwell Publishing Ltd (9600 Garsington Road, Oxford OX4 2XG, United Kingdom)

Emtree Heading

adult; article; cohort analysis; comorbidity; *diabetes mellitus; female; human; Human immunodeficiency virus infection; lung tuberculosis/dt [Drug Therapy]; major clinical study; male; mixed infection; mortality; risk factor; sex difference; sputum culture; survival rate; Tanzania; *tuberculosis/dt [Drug Therapy]; alpha1 glycoprotein/ec [Endogenous Compound]; tuberculostatic agent/dt [Drug Therapy].

Link to the Ovid Full Text or citation:

Click here for full text options

53.

The biological and clinical activity of anti-malarial drugs in autoimmune disorders.

Taherian E., Rao A., Malemud C.J., Askari A.D.

Current Rheumatology Reviews. 9 (1) (pp 45-62), 2013. Date of Publication: 2013.

AN: 2013358663

Chloroquine and hydroxychloroquine are 4-aminoquinoline compounds commonly employed as anti-malarial drugs. Chloroquine and its synthetic analogue, hydroxychloroquine also belong to the disease-modifying anti-rheumatic drug class because these drugs are immunosuppressive. The immunosuppressive activity of chloroquine and hydroxychloroquine is likely to account for their capacity to reduce T-cell and B-cell hyperactivity as well as pro-inflammatory cytokine gene expression. This review evaluated experimental and clinical trials results as well as clinical response data relative to the use of chloroquine and/or hydroxychloroquine as first-line medical therapies in systemic lupus erythematosus, rheumatoid arthritis, primary Sjogren's syndrome, the anti-phospholipid syndrome and in the treatment of sarcoidosis. A primary outcomes measure in these clinical trials was the extent to which chloroquine and/or hydroxychloroquine reduced disease progression or exacerbations and/or the use and dosage of corticosteroids. The relative efficacy of chloroquine and hydroxychloroquine in modifying the clinical course of these autoimmune disorders is balanced against evidence that these drugs induce adverse effects which may reduce their use and effectiveness in the therapy of autoimmune disorders. © 2013 Bentham Science Publishers.

Institution

(Taherian, Rao, Malemud, Askari) Department of Medicine, Division of Rheumatic Diseases, Case Western Reserve University School of Medicine and University Hospitals Case Medical Center, Cleveland, OH 44106, United States (Malemud) Department of Anatomy, Case Western Reserve University School of Medicine, University Hospitals Case Medical Center, Cleveland, OH 44106, United States

Publisher

Bentham Science Publishers B.V. (P.O. Box 294, Bussum 1400 AG, Netherlands)

Emtree Heading

anticoagulation; antigen antibody complex; *antimalarial activity; *antiphospholipid syndrome; Apgar score; arterial stiffness; artery thrombosis/co [Complication]; artery thrombosis/pc [Prevention]; arthralgia; atherosclerosis; atomic force microscopy; autologous mixed lymphocyte reaction; birth weight; body mass; bone density; bone erosion; burning sensation; cardiomyopathy; cardiovascular risk; cell surface; cholesterol blood level; clinical practice; cohort analysis; coronary artery disease; cross-sectional study; deep vein thrombosis/pc [Prevention]; diabetes mellitus; disease activity; disease course; disease exacerbation; drug dose titration; drug efficacy; dyslipidemia; echography; ex vivo study; fat intake; follow up; glucose tolerance; heart left ventricle filling pressure; heart muscle biopsy; human; hypercalcemia; hypertension; in vitro study; in vivo study; insulin release; insulin sensitivity; joint function; kidney failure; kidney injury; lean body weight; lung embolism/pc [Prevention]; lung function; lung infiltrate; lung lesion; lupus erythematosus nephritis/dt [Drug Therapy]; lupus erythematosus nephritis/pc [Prevention]; lymphadenopathy; morning stiffness; mouth mucosa; myalgia; Mycobacterium tuberculosis; myopathy; North America; open study; outcome assessment; overall survival; phospholipid bilayer; pigment epithelium; pneumonia; postmenopause; postoperative period; pregnancy; pregnancy outcome; priority journal; propensity score; prospective study; pulse wave; randomized controlled trial (topic); remission; retinopathy/dt [Drug Therapy]; retrospective study; review; *rheumatoid arthritis/dt [Drug Therapy]; rheumatology; risk factor; sarcoidosis/dt [Drug Therapy]; sarcoidosis/et [Etiology]; *Sjoegren syndrome; skin defect; skin pigmentation; SLEDAI; *systemic lupus erythematosus/di [Diagnosis]; *systemic lupus erythematosus/dt [Drug Therapy]; *systemic lupus erythematosus/et [Etiology]; thorax radiography; thrombocyte aggregation; tissue injury; transthoracic echocardiography; world health organization; xerostomia; azathioprine; CD8 antigen/ec [Endogenous Compound]; chlorambucil; *chloroquine/dt [Drug Therapy]; cholesterol/ec [Endogenous Compound]; colchicine; cyclosporin; *hydroxychloroquine/cb [Drug Combination]; *hydroxychloroquine/dt [Drug Therapy]; *hydroxychloroquine/po [Oral Drug Administration]; interleukin 1/ec [Endogenous Compound]; interleukin 15/ec [Endogenous Compound]; interleukin 18/ec [Endogenous Compound]; interleukin 2/ec [Endogenous Compound]; interleukin 6/ec [Endogenous Compound]; interleukin 8/ec [Endogenous Compound]; leflunomide; lipocortin 5; low density lipoprotein/ec [Endogenous Compound]; methotrexate/cb [Drug Combination]; mycophenolic acid 2 morpholinoethyl ester/dt [Drug Therapy]; placebo; prednisone; quinoprox; salazosulfapyridine/cb [Drug Combination]; triacylglycerol/ec [Endogenous Compound]; tumor necrosis factor alpha/ec [Endogenous Compound]; unclassified drug.

Drug Trade Names and Manufacturers

plaquenil, quinoprox

Link to the Ovid Full Text or citation:

Click here for full text options

54.

Clinical characteristics, drug resistance, and treatment outcomes among tuberculosis patients with diabetes in Peru.

Magee M.J., Bloss E., Shin S.S., Contreras C., Huaman H.A., Ticona J.C., Bayona J., Bonilla C., Yagui M., Jave O., Cegielski J.P.

International Journal of Infectious Diseases. 17 (6) (pp e404-e412), 2013. Date of Publication: June 2013.

AN: 2013289598

Objectives: Diabetes is a risk factor for active tuberculosis (TB). Data are limited regarding the association between diabetes and TB drug resistance and treatment outcomes. We examined characteristics of TB patients with and without diabetes in a Peruvian cohort at high risk for drug-resistant TB. Among TB patients with diabetes (TB-DM), we studied the association between diabetes clinical/management characteristics and TB drug resistance and treatment outcomes. Methods: During 2005-2008, adults with suspected TB with respiratory symptoms in Lima, Peru, who received rapid drug susceptibility testing (DST), were prospectively enrolled and followed during treatment. Bivariate and Kaplan-Meier analyses were used to examine the relationships of diabetes characteristics with drug-resistant TB and TB outcomes. Results: Of 1671 adult TB patients enrolled, 186 (11.1%) had diabetes. TB-DM patients were significantly more likely than TB patients without diabetes to be older, have had no previous TB treatment, and to have a body mass index (BMI) >18.5kg/m2 (p<0.05). In patients without and with previous TB treatment, the prevalence of multidrug-resistant TB was 23% and 26%, respectively, among patients without diabetes, and 12% and 28%, respectively, among TB-DM patients. Among 149 TB-DM patients with DST results, 104 (69.8%) had drug-susceptible TB and 45 (30.2%) had drug-resistant TB, of whom 29 had multidrug-resistant TB. There was no association between diabetes characteristics and drug-resistant TB. Of 136 TB-DM patients with outcome information, 107 (78.7%) had a favorable TB outcome; active diabetes management was associated with a favorable outcome. Conclusions: Diabetes was common in a cohort of TB patients at high risk for drug-resistant TB. Despite prevalent multidrug-resistant TB among TB-DM patients, the majority had a favorable TB treatment outcome. © 2013 International Society for Infectious Diseases.

Institution

(Magee) Rollins School of Public Health, Emory University, Atlanta, United States (Bloss, Cegielski) US Centers for Disease Control and Prevention, 1600 Clifton Rd, MS E-10, Atlanta, GA 30333, United States

(Shin, Bayona) Partners in Health, Boston, United States

(Shin, Contreras, Bayona) Socios En Salud, Lima, Peru

(Huaman, Ticona, Bonilla, Jave) Ministerio de Salud del Peru, Lima, Peru

(Yagui) Instituto Nacional de Salud, Lima, Peru

(Yagui) Universidad Nacional Mayor de San Marcos, Lima, Peru

Publisher

Elsevier (P.O. Box 211, Amsterdam 1000 AE, Netherlands)

Emtree Heading

adolescent; adult; article; body mass; cohort analysis; diabetes control; *diabetes mellitus/dt [Drug Therapy]; diabetic patient; disease association; *drug resistant tuberculosis; drug sensitivity; female; high risk patient; human; insulin treatment; laboratory; major clinical study; male; *multidrug resistant tuberculosis; outcome assessment; Peru; prospective study; sputum culture; insulin/dt [Drug Therapy]; oral antidiabetic agent/dt [Drug Therapy].

Link to the Ovid Full Text or citation:

Click here for full text options

55.

Impact of Diabetes and Smoking on Mortality in Tuberculosis.

Reed G.W., Choi H., Lee S.Y., Lee M., Kim Y., Park H., Lee J., Zhan X., Kang H., Hwang S.H., Carroll M., Cai Y., Cho S.-N., Barry III C.E., Via L.E., Kornfeld H.

PLoS ONE. 8 (2) (no pagination), 2013. Article Number: e58044. Date of Publication: 28 Feb 2013.

AN: 2013139362

Background: Diabetes mellitus is a risk factor for tuberculosis (TB) disease. There is evidence that diabetes also influences TB severity and treatment outcomes but information is incomplete and some published results have been inconsistent. Methods: A longitudinal cohort study was conducted at the National Masan Tuberculosis Hospital in the Republic of Korea. Subjects presenting with a first episode of TB or for retreatment of TB were followed from enrollment through completion of treatment. Demographic, clinical, and microbiological variables were recorded, along with assessment of outcomes. Results were compared in TB patients with and without diabetes or smoking history. Data were adjusted for gender, age, cohort, educational level and alcohol consumption. Results: The combined cohorts comprised 657 subjects. Diabetes was present in 25% and was associated with greater radiographic severity and with recurrent or relapsed TB. Diabetes and cigarette smoking independently increased the risk of death in the first 12 months after enrollment. Estimating the combined impact of diabetes and smoking yielded a hazard ratio of 5.78. Only 20% of diabetic subjects were non-smokers; 54% smoked >1 pack daily. In this cohort, the impact of diabetes on mortality was greater in patients younger than 50 years, compared to older patients. Conclusions: In this cohort of Korean patients, diabetes exacerbated the severity of TB disease. Diabetic subjects who smoked >1 pack of cigarettes daily were at particularly high risk of death from TB. Strategies to improve TB outcomes could productively focus resources for patient education and TB prevention on the vulnerable population of younger diabetics, particularly those who also smoke.

Institution

(Reed) Department of Preventive and Behavioral Medicine, University of Massachusetts Medical School, Worcester, MA, United States (Choi, Lee, Lee, Kim, Park, Lee, Cho) International Tuberculosis Research Center, Changwon, South Korea

(Cho) Department of Microbiology, Yonsei University College of Medicine, Seoul, South Korea

(Zhan) Information Services Department, University of Massachusetts Medical School, Worcester, MA, United States

(Kang, Hwang) National Masan Tuberculosis Hospital, Changwon, South Korea

(Carroll, Cai, Barry III, Via) Tuberculosis Research Section, Laboratory of Clinical Infectious Disease, National Institute of Allergy and Infectious Diseases, Bethesda, MD, United States

(Kornfeld) Department of Medicine, University of Massachusetts Medical School, Worcester, MA, United States

Publisher

Public Library of Science (185 Berry Street, Suite 1300, San Francisco CA 94107, United States)

Emtree Heading

adult; adverse outcome; article; cohort analysis; comorbidity; controlled study; *diabetes mellitus; disease exacerbation; disease severity; female; hazard ratio; human; longitudinal study; *lung tuberculosis/dt [Drug Therapy]; major clinical study; male; mortality; multidrug resistant tuberculosis; recurrent infection; relapse; retreatment; *smoking; South Korea; survival rate; thorax radiography; ethambutol/cb [Drug Combination]; ethambutol/dt [Drug Therapy]; isoniazid/cb [Drug Combination]; isoniazid/dt [Drug Therapy]; pyrazinamide/cb [Drug Combination]; pyrazinamide/dt [Drug Therapy]; rifampicin/cb [Drug Combination]; rifampicin/dt [Drug Therapy].

Link to the Ovid Full Text or citation:

Click here for full text options

56.

Impact of diabetes mellitus on treatment outcomes of patients with active tuberculosis in an endemic area.

Socaci A., Nini G., Marica C.

European Respiratory Journal. Conference: European Respiratory Society Annual Congress 2013 Barcelona Spain. Conference Start: 20130907 Conference End: 20130911. Conference Publication: (var.pagings). 42 (no pagination), 2013. Date of Publication: 01 Sep 2013.

AN: 71843977

Introduction. Diabetes mellitus (DM) is an emerging chronic health condition of developed and developing countries. Due to the association between diabetes and pulmonary tuberculosis (TB), diabetes may threaten the control of TB. Aims. We conducted a study to determine the prevalence of DM among TB patients, and to determine and compare treatment outcomes among TB patients with versus without DM. Methods. A retrospective cohort study included all patients with culture-confirmed TB diagnosed in 2010 and 2011, in west of the country. Results. Of 228 TB patients, 42 (18.4%) had DM. Patients with diabetes had 2.0 times higher odds of death than patients without diabetes (p = 0.18). Of the diabetic TB patients, 9.5% died within one month after starting TB treatment, compared with 3.5% of patients without diabetes (p = 0.09) Time to sputum culture conversion was longer in patients with diabetes than patients without diabetes (median 49 versus 39 days, p = 0.09). Two-month culture conversion proportions were similar (69% and 70%). There was no difference in the proportion of patients with baseline drug resistance, comparing patients with diabetes to patients without diabetes (14.3% versus 15.3%, p = 0.87). Treatment failure occurred in 4.1% of patients without diabetes and 6.7% of patients with diabetes (p = 0.51). Conclusions. In our TB patient population, DM was a risk factor for death, and it had a negative impact on treatment outcome. Enhanced medical vigilance, especially during the early part of TB treatment, is warranted.

Publisher

European Respiratory Society

Emtree Heading

*tuberculosis; *chronic disease; *treatment outcome; *diabetes mellitus; *patient; *human; *European; *society; death; lung tuberculosis; developing country; cohort analysis; sputum culture; health; prevalence; risk factor; population; drug resistance; treatment failure; alertness.

Link to the Ovid Full Text or citation:

Click here for full text options

57.

Impact of diabetes mellitus on drug-resistant tuberculosis treatment outcomes in Georgia-cohort study.

Kikvidze M., Mikiashvili L.

European Respiratory Journal. Conference: European Respiratory Society Annual Congress 2013 Barcelona Spain. Conference Start: 20130907 Conference End: 20130911. Conference Publication: (var.pagings). 42 (no pagination), 2013. Date of Publication: 01 Sep 2013.

AN: 71843894

Background. Diabetes mellitus (DM) is associated with an increased risk of tuberculosis (TB). There is growing evidence that this comorbidity might affect TB-disease presentation and treatment response. Aims and objectives. We investigated the level of impact of diabetes mellitus on drug-resistant tuberculosis (DR-TB) treatment outcomes in condition of adequate management of both diseases. Methods. Cohort study was provided among 1154 drug-resistant tuberculosis patients integrated in DOTS+ programme in 2009-2010 years in Georgia. Every patient was routinely investigated for DM. To compare the treatment outcomes of patients with and those without diabetes comorbidity dispersive analysis using Kruskal-Wallis - H criteria was provided. Results. Among 1154 DR-TB patients only 69 (5, 98%) had diabetes mellitus. All of them developed pulmonary form of TB and were HIV-negative. 61 (88, 4%) patient was male, mean age was 46, 8 (Standard deviation-12,67), mean BMI-23,44 (St.dev.- 3,78). In 3 (4,3%) patient was revealed extensively-DR-TB, in 58 (84,1%) - multi-DR-TB, and in 7 (10, 1%) - poly-DR-TB. 91,3% patients had II type of DM and 86,9% received insulin therapy. Definition: "successful treatment" received 55% diabetic patient vs 56 % non-diabetic, "Default"-36,2% vs 27,6%; "Failure" - 5,8 % vs 4,7 %; "Death" - 4,3% vs 9,6 % (p>0,05). Conclusions. We found no significant difference in proportion of successful treatment outcomes, though default rate was rather higher in patients with diabetes than non-diabetes in the setting of adequate treatment options and management in Georgia.

Institution

(Kikvidze, Mikiashvili) MDR-TB Department, National Center for Tuberculosis and Lung Diseases (NCTBLD), Tbilisi 0101, Georgia

Publisher

European Respiratory Society

Emtree Heading

*diabetes mellitus; *drug resistant tuberculosis; *treatment outcome; *cohort analysis; *European; *society; *tuberculosis; human; patient; comorbidity; treatment response; risk; death; diabetic patient; insulin treatment; spinal spacer; male; diseases; Human immunodeficiency virus; short course therapy.

Link to the Ovid Full Text or citation:

Click here for full text options

58.

Clinical profile of MDR-TB patients and their early response to DOTS PLUS: An Indian perspective.

Javia A., Mehta D., Ganchi F., Rathod B., Chopada K., Bhanderi M.

European Respiratory Journal. Conference: European Respiratory Society Annual Congress 2013 Barcelona Spain. Conference Start: 20130907 Conference End: 20130911. Conference Publication: (var.pagings). 42 (no pagination), 2013. Date of Publication: 01 Sep 2013.

AN: 71843868

Background:The emergence MDR-TB is an obstacle to effective TB control in India Aim:to analyze the MDR TB patients(Pt) & to evaluate their response to DOTS PLUS Methods: A prospective cohort study of 58 MDR-TB Pts. Pt's detailed demographic data recorded & put on DOTS PLUS.Clinical evaluation, sputum smear, culture & DST at the end of 3,4,5,6 and 9 month were done to monitor progress during treatment. Treatment outcomes were noted at the end of initiation phase (IP) Results:MDR-TB were common in males (M:F=3:2),in age group 18-35 yrs(64%),in lower socioeconomic class(88%),in Pts with BMI <18.5(89% M,96% F),in rural areas(48%),with bilateral(85%) & cavitory(67%) lesion on CXR.Commonest symptoms were cough(100%),fever(95%) & weight loss(60%). Associated with DM(8.6%) & HIV(5.2%).High grade sputum & culture positivity (+2 & +3) were more prevalent(60% & 69% respectively). 85% had resistance to both H & R. End IP smear & culture conversion rate was 72.4% and 56.9% respectively while 15.5% showed smear conversion but culture non-conversion. Mean smear & culture conversion time was 3.4 & 4.12 months respectively. Low smear & culture conversion rate was seen in Pts with H& R resistance,bilateral & cavitory lesions on CXR and initial high grade smear & culture positivity. 6 Pts(10.3%) died and 4 Pts(6.9%) defaulted during IP. 33% Pts had ADR, the commonest being GI disturbance(42%),hearing loss(32%) & psychiatric disturbance(16%) Conclusion:Early diagnosis & treatment of MDR-TB is essential. Resistance to both H & R,bilateral & cavitory lesion in CXR,high grade initial smear & culture positivity,low BMI and DM is associated with poor outcome. We will continue our study till completion of treatment.

Institution

(Javia, Mehta, Ganchi, Rathod, Chopada, Bhanderi) Pulmonary Medicine, M P Shah Medical College, Jamnagar, Gujarat 361008, India

Publisher

European Respiratory Society

Emtree Heading

*tuberculosis; *human; *diagnosis; *patient; *Indian; *European; *society; *short course therapy; smear; sputum smear; cohort analysis; weight reduction; gastrointestinal symptom; male; social class; groups by age; rural area; coughing; fever; treatment outcome; sputum; India; hearing impairment; early diagnosis; Human immunodeficiency virus.

Link to the Ovid Full Text or citation:

Click here for full text options

59.

Disease severity score could not predict the outcomes in peritoneal dialysis-associated peritonitis.

Dong J., Li Z., Xu R., Chen Y., Luo S., Li Y.

Nephrology Dialysis Transplantation. 27 (6) (pp 2496-2501), 2012. Date of Publication: June 2012.

AN: 2012322939

Background. We aim to explore if disease severity score (DSS) at onset is associated with dialysate white cell counts, the severity of causative organisms and the risk for treatment failure of peritoneal dialysis (PD)-associated peritonitis in an adult PD cohort. Methods .Our prospective cohort study recorded all peritonitis episodes between 2008 and 2010. The DSS, demographic data and clinical characteristics were recorded at the onset of peritonitis. The dialysate cells were counted at regular intervals and organism culture were examined too. Treatment failure of peritonitis was defined as peritonitis-associated death and transfer to haemodialysis. Results. A total of 219 episodes of peritonitis in 146 PD patients were recorded, 21.9% of which resulted in treatment failure. There were no significant differences in dialysate white cell counts on the fifth and seventh day and the distribution of causative organism between groups with varied DSS level. DSS could not predict treatment failure including peritonitis-related death and transfer to haemodialysis after adjusting for age, gender, diabetes, dialysis duration, dialysate white cell count on the third day, the presence of Staphylococcus aureus, gram-negative organisms and polymicrobial organisms. Conclusion. Our study demonstrated that DSS at onset was not associated with prolonged elevation of dialysate white cell counts, severity of causative organisms and outcome of peritonitis episodes in adult PD patients. © 2012 The Author.

Institution

(Dong, Li, Xu, Chen, Luo, Li) Department of Medicine, Peking University, First Medical Hospital, Peking, China (Li) Department of Nephrology, West China Hospital of Sichuan University, Sichuan, China

Publisher

Oxford University Press (Great Clarendon Street, Oxford OX2 6DP, United Kingdom)

Emtree Heading

abdominal pain; Acinetobacter; adult; age; aged; article; cohort analysis; controlled study; Corynebacterium; diabetes mellitus; dialysate; dialysate white cell count; *disease severity; *disease severity score; Enterococcus; Escherichia coli; female; fever; fungus; gender; Gram negative bacterium; Gram positive bacterium; hemodialysis; human; Klebsiella; leukocyte count; major clinical study; male; mortality; *peritoneal dialysis; *peritonitis/co [Complication]; priority journal; prospective study; Pseudomonas; *scoring system; Staphylococcus; Staphylococcus aureus; Streptococcus; treatment duration; treatment failure; treatment outcome; tuberculosis.

Link to the Ovid Full Text or citation:

Click here for full text options

60.

Pulmonary Tuberculosis and delay in anti-tuberculous treatment are important risk factors for chronic obstructive pulmonary disease.

Lee C.-H., Lee M.-C., Lin H.-H., Shu C.-C., Wang J.-Y., Lee L.-N., Chao K.-M.

PLoS ONE. 7 (5) (no pagination), 2012. Article Number: e37978. Date of Publication: 25 May 2012.

AN: 2012302785

Objective: Tuberculosis (TB) remains the leading cause of death among infectious diseases worldwide. It has been suggested as an important risk factor of chronic obstructive pulmonary disease (COPD), which is also a major cause of morbidity and mortality. This study investigated the impact of pulmonary TB and anti-TB treatment on the risk of developing COPD. Design, Setting, and Participants: This cohort study used the National Health Insurance Database of Taiwan, particularly the Longitudinal Health Insurance Database 2005 to obtain 3,176 pulmonary TB cases and 15,880 control subjects matched in age, sex, and timing of entering the database. Main Outcome Measures: Hazard ratios of potential risk factors of COPD, especially pulmonary TB and anti-TB treatment. Results: The mean age of pulmonary TB cases was 51.9+/-19.2. The interval between the initial study date and commencement of anti-TB treatment (delay in anti-TB treatment) was 75.8+/-65.4 days. Independent risk factors for developing COPD were age, male, low income, and history of pulmonary TB (hazard ratio 2.054 [1.768-2.387]), while diabetes mellitus was protective. The impact of TB persisted for six years after TB diagnosis and was significant in women and subjects aged >70 years. Among TB patients, delay in anti-TB treatment had a dose-response relationship with the risk of developing COPD. Conclusions: Some cases of COPD may be preventable by controlling the TB epidemic, early TB diagnosis, and prompt initiation of appropriate anti-TB treatment. Follow-up care and early intervention for COPD may be necessary for treated TB patients. © 2012 Lee et al.

Institution

(Lee, Chao) Graduate Institute of Biomedical Electronics and Bioinformatics, National Taiwan University, Taipei, Taiwan (Republic of China) (Lee) Department of Internal Medicine, Buddhist Tzu Chi General Hospital, Taipei Branch, New Taipei, Taiwan (Republic of China)

(Lee) School of Medicine, Tzu Chi University, Hualien, Taiwan (Republic of China)

(Lee) Department of Pharmacy, Buddhist Tzu Chi General Hospital, Taipei Branch, New Taipei, Taiwan (Republic of China)

(Lin) Graduate Institute of Epidemiology and Preventive Medicine, National Taiwan University, Taipei, Taiwan (Republic of China)

(Shu) Department of Traumatology, National Taiwan University Hospital, Taipei, Taiwan (Republic of China)

(Shu, Wang) Department of Internal Medicine, National Taiwan University Hospital, Taipei, Taiwan (Republic of China)

(Lee) Department of Laboratory Medicine, National Taiwan University Hospital, Taipei, Taiwan (Republic of China)

Publisher

Public Library of Science (185 Berry Street, Suite 1300, San Francisco CA 94107, United States)

Emtree Heading

adult; age; aged; article; *chronic obstructive lung disease/di [Diagnosis]; cohort analysis; controlled study; data base; diabetes mellitus; disease association; female; gender; human; lowest income group; *lung tuberculosis/di [Diagnosis]; major clinical study; male; medical history; national health insurance; outcome assessment; patient attitude; patient compliance; patient participation; *risk assessment; risk factor; Taiwan; *therapy delay; tuberculosis/dt [Drug Therapy]; *tuberculostatic agent/dt [Drug Therapy].

Link to the Ovid Full Text or citation:

Click here for full text options

61.

Role of diabetes in the prognosis and therapeutic outcome of tuberculosis.

Syed Suleiman S.A., Ishaq Aweis D.M., Mohamed A.J., Razakmuttalif A., Moussa M.A.A.

International Journal of Endocrinology. 2012 (no pagination), 2012. Article Number: 645362. Date of Publication: 2012.

AN: 2012278888

Background. Increased susceptibility of diabetic mellitus (DM) patients to infection, including tuberculosis (TB), is well documented. The prevalence of DM in Malaysia is reaching epidemic proportions. In this study, we sought to assess risk factors for TB and the impact of DM on the outcome of TB treatment. Methods. TB patients, diabetic patients, and diabetic patients with TB were divided into three groups of 200 subjects each. Data were obtained from patients' medical files at the beginning and end of the study period. Prevalence rates of DM and HIV among TB patients were assessed. Prognosis, TB-related complications, anatomical site of infection, and duration of infection and diabetes were also examined. Results. The prevalence rates of HIV and DM amongst TB patients were 7.7 and 30%, respectively. The diabetic TB patient group contained more males (72%) and smokers (45.5%) compared to the nondiabetic group (58.3% and 33.5%, resp.). Approximately 74% of diabetic patients were Mycobacterium sputum positive compared to only 51% of nondiabetic patients. Diabetic patients were also more likely to develop pulmonary TB (87%) compared to nondiabetic TB patients (59%). Diabetic TB patients had a higher mortality rate (7.5%) compared to the TB only and DM only groups (1 and 2%, resp.). The duration of TB symptoms was longer in nondiabetic TB patients compared to diabetic TB patients (4.5 versus 2.6 months, resp.). Diabetes antedated TB by a mean time of 4 years. Conclusions. We found a higher number of sputum-smear-positive cases and pulmonary TB cases as well as a greater number of males and higher mortality rate in diabetic patients compared to nondiabetic patients. Copyright © 2012 Syed Azhar Syed Suleiman et al.

Institution

(Syed Suleiman) School of Pharmaceutical Sciences, USM, 11800 Penang, Malaysia (Ishaq Aweis) Department of Clinical Pharmacy, School of Pharmaceutical Sciences, USM, 11800 Penang, Malaysia

(Ishaq Aweis) Ahmadi Hospital, Kuwait Oil Company, Kuwait

(Mohamed) Department of Pharmacology, School of Pharmaceutical Sciences, USM, 11800 Penang, Malaysia

(Mohamed) Faculty of Medicine, Benadir University, Mogadishu, Somalia

(Razakmuttalif) Respiratory Clinic, Hospital Pulau Pinang (HPP), Malaysia

(Moussa) Department of Community Medicine and Behavioural Sciences, Faculty of Medicine, Kuwait University, Kuwait

Publisher

Hindawi Publishing Corporation (410 Park Avenue, 15th Floor, 287 pmb, New York NY 10022, United States)

Emtree Heading

adult; article; cohort analysis; controlled study; *diabetes mellitus/et [Etiology]; disease duration; female; high risk population; human; Human immunodeficiency virus infection/ep [Epidemiology]; lung tuberculosis; major clinical study; male; mortality; Mycobacterium; prevalence; priority journal; *prognosis; risk factor; sex difference; smoking; *treatment outcome; *tuberculosis.

Link to the Ovid Full Text or citation:

Click here for full text options

62.

Predictors of mortality in staphylococcus aureus bacteremia.

van Hal S.J., Jensen S.O., Vaska V.L., Espedido B.A., Paterson D.L., Gosbell I.B.

Clinical Microbiology Reviews. 25 (2) (pp 362-386), 2012. Date of Publication: April 2012.

AN: 2012205805

Staphylococcus aureus bacteremia (SAB) is an important infection with an incidence rate ranging from 20 to 50 cases/100,000 population per year. Between 10% and 30% of these patients will die from SAB. Comparatively, this accounts for a greater number of deaths than for AIDS, tuberculosis, and viral hepatitis combined. Multiple factors influence outcomes for SAB patients. The most consistent predictor of mortality is age, with older patients being twice as likely to die. Except for the presence of comorbidities, the impacts of other host factors, including gender, ethnicity, socioeconomic status, and immune status, are unclear. Pathogen-host interactions, especially the presence of shock and the source of SAB, are strong predictors of outcomes. Although antibiotic resistance may be associated with increased mortality, questions remain as to whether this reflects pathogen-specific factors or poorer responses to antibiotic therapy, namely, vancomycin. Optimal management relies on starting appropriate antibiotics in a timely fashion, resulting in improved outcomes for certain patient subgroups. The roles of surgery and infectious disease consultations require further study. Although the rate of mortality from SAB is declining, it remains high. Future international collaborative studies are required to tease out the relative contributions of various factors to mortality, which would enable the optimization of SAB management and patient outcomes. © 2012, American Society for Microbiology. All Rights Reserved.

Institution

(van Hal, Gosbell) Department of Microbiology and Infectious Diseases, Sydney South West Pathology Service-Liverpool, South Western Sydney Local Health Network, Sydney, NSW, Australia (van Hal, Jensen, Espedido, Gosbell) Antibiotic Resistance and Mobile Elements Group, Microbiology and Infectious Diseases Unit, School of Medicine, University of Western Sydney, Sydney, NSW, Australia

(Jensen, Espedido, Gosbell) Ingham Institute of Applied Medical Research, Sydney, NSW, Australia

(Vaska, Paterson) The University of Queensland, UQ Centre for Clinical Research (UQCCR), Herston, QLD, Australia

Publisher

American Society for Microbiology (1752 N Street N.W., Washington DC 20036-2904, United States)

Emtree Heading

acquired immune deficiency syndrome; alcoholism; bacterial endocarditis; bactericidal activity; bone and joint infections/ep [Epidemiology]; Caucasian; cellular immunity; central nervous system infection/ep [Epidemiology]; chronic kidney failure; cohort analysis; confidence interval; congestive heart failure; crowding; device infection/co [Complication]; diabetes mellitus; drug fatality/si [Side Effect]; ethnicity; gender; hematologic malignancy; host pathogen interaction; human; humoral immunity; immune deficiency/cn [Congenital Disorder]; immune status; in vitro study; innate immunity; intensive care unit; liver cirrhosis; meta analysis (topic); methicillin resistant Staphylococcus aureus; methicillin resistant Staphylococcus aureus infection/dt [Drug Therapy]; mortality; multidrug resistance; multivariate analysis; neutropenia; newborn intensive care; penicillin resistance; phagocyte; postoperative complication/co [Complication]; prospective study; prostate cancer; prosthetic valve endocarditis/co [Complication]; prosthetic valve endocarditis/et [Etiology]; randomized controlled trial (topic); retrospective study; review; risk; sepsis; shock; social status; socioeconomics; soft tissue infection/co [Complication]; *staphylococcal bacteremia/dr [Drug Resistance]; *staphylococcal bacteremia/ep [Epidemiology]; *staphylococcal bacteremia/et [Etiology]; Staphylococcus infection/dt [Drug Therapy]; surgical infection/co [Complication]; survival prediction; systematic review (topic); treatment failure; tuberculosis; United States; unspecified side effect/si [Side Effect]; virus hepatitis; cefazolin/dt [Drug Therapy]; cloxacillin/dt [Drug Therapy]; cotrimoxazole/cm [Drug Comparison]; cotrimoxazole/dt [Drug Therapy]; exotoxin; flucloxacillin; gentamicin/ae [Adverse Drug Reaction]; levofloxacin; linezolid/ae [Adverse Drug Reaction]; linezolid/cm [Drug Comparison]; meticillin; rifampicin/pk [Pharmacokinetics]; vancomycin/cm [Drug Comparison]; vancomycin/dt [Drug Therapy].

Link to the Ovid Full Text or citation:

Click here for full text options

63.

Tuberculosis treatment outcomes in patients with and without diabetes mellitus.

Khan A.H., Sulaiman S.A.S., Abdul Muttalif A.R., Hassali M.A., Ahmad N.

Value in Health. Conference: ISPOR 15th Annual European Congress Berlin Germany. Conference Start: 20121103 Conference End: 20121107. Conference Publication: (var.pagings). 15 (7) (pp A387), 2012. Date of Publication: November 2012.

AN: 70916571

OBJECTIVES: To evaluate treatment outcomes in tuberculosis patients with and without diabetes mellitus. METHODS: This was a retrospective cohort study conducted at respiratory clinic of Hospital Pulau Pinang (HPP), Malaysia. All Tuberculosis (TB) patients who were presented to the clinic from January 2006 to December 2007 were included in the study. A purpose developed valid data collection form was used for collecting demographic and clinical data. Treatment outcomes initially recorded as cured, treatment completed, defaulted, transferred out, expired and treatment continued were then classified into two categories; successful and unsuccessful treatment. Cured and treatment completed patients were placed in treatment successful category while the rest were placed in the category of unsuccessful treatment. Data was analyzed by using SPSS 16. RESULTS: Final analysis included 1266 patients.Three hundred and thirty eight patients (26.7%) had DM along with TB. In multivariate analysis TB-DM was more likely to be present in Chinese (OR=1.470, p-value=0.003), married patients (OR=1.408, p-value=0.011) and patients having age 46-60 years (OR=2.002, p-value<0.001), and>60 years (OR = 1.594, p-value = 0.010). Nine hundred and eighty five (78.8%) patients were successfully treated. Successful Treatment was observed in patients having age of 46-60 years (OR=1.567, p-value=0.001), whereas male gender (OR=0.721, p-value = 0.049) and patients with relapse TB (OR = 0.494, p-value = 0.002) were less likely to have successful treatment. CONCLUSIONS: In the present study Chinese ethnicity, age (> 46 years) and being married were the predictors of prevalence of TB-DM. Majority patients were successfully treated. Male gender and patients with relapse TB were the predictors of treatment failure. No statistically significant difference in treatment outcomes was observed between TB patients with and without diabetes mellitus.

Institution

(Khan, Sulaiman) Universiti Sains Malaysia, Penang, Pulau Pinang, Malaysia (Abdul Muttalif) Respiratory Institute of Malaysia, Kula Lumpur, Malaysia

(Hassali) Universiti Sains Malaysia, Minden, Penang, Malaysia

(Ahmad) Universiti Sains Malaysia, Pinang, Penang, Malaysia

Publisher

Elsevier Ltd

Emtree Heading

*treatment outcome; *patient; *human; *diabetes mellitus; *tuberculosis; statistical significance; hospital; gender; male; relapse; betel nut; multivariate analysis; cohort analysis; clinical study; information processing; prevalence; Malaysia; ethnicity; treatment failure.

Link to the Ovid Full Text or citation:

Click here for full text options

64.

Peritoneal dialysis as the first-line renal replacement therapy in patients with autosomal dominant polycystic kidney disease.

Li L., Szeto C.-C., Kwan B.C.-H., Chow K.-M., Leung C.-B., Kam-Tao Li P.

American Journal of Kidney Diseases. 57 (6) (pp 903-907), 2011. Date of Publication: June 2011.

AN: 2011285196

Background: Autosomal dominant polycystic kidney disease (ADPKD) is the most common hereditary cause of kidney failure. Peritoneal dialysis (PD) often is avoided because of concerns about hernias and peritonitis. Study Design: Retrospective matched-cohort study. Setting & Participants: 42 consecutive patients with ADPKD and 84 nondiabetic patients matched by time of PD therapy initiation. Predictors: ADPKD and comorbid conditions. Outcomes: Patient survival, technique survival, peritonitis-free survival; peritonitis rate; abdominal hernias. Measurements: Dialysis adequacy and nutritional indexes; rate of decrease in residual renal function; bacteriologic cause of peritonitis. Results: 5-year actuarial survival of the ADPKD and control groups was 71.0% and 69.7% (P = 0.4), whereas technique survival was 51.6% and 37.3%, respectively (P = 0.2). There was no difference in overall rates of peritonitis between the ADPKD and control groups (0.51 vs 0.53 episodes/patient-year; P = 0.3), and the incidence of Gram-negative peritonitis also was similar (0.16 vs 0.14 episodes/patient year; P = 0.5). Abdominal wall hernia was significantly more common in the ADPKD than control group (14 vs 6 cases; P < 0.001), but all patients were able to resume PD therapy after surgical repair. Limitations: Retrospective study with limited sample size. Conclusion: PD is a feasible treatment option for most patients with ADPKD with end-stage renal disease. Although patients with ADPKD have a higher risk of abdominal wall hernia, their overall survival rate and risk of peritonitis are similar to those of other nondiabetic PD patients. © 2011 National Kidney Foundation, Inc.

Institution

(Li) Department of Nephrology, Shanghai Changzheng Hospital, Second Military Medical University, Shanghai, China (Szeto, Kwan, Chow, Leung, Kam-Tao Li) Department of Medicine and Therapeutics, Prince of Wales Hospital, Chinese University of Hong Kong, Shatin, Hong Kong

Publisher

W.B. Saunders (Independence Square West, Philadelphia PA 19106-3399, United States)

Emtree Heading

abdominal wall hernia; Acinetobacter; adult; aged; article; *autosomal dominant polycystic kidney disease/cn [Congenital Disorder]; *autosomal dominant polycystic kidney disease/th [Therapy]; blood stain; cerebrovascular disease; clinical article; cohort analysis; comorbidity; controlled study; diabetes mellitus; disease free survival; Enterococcus; Escherichia coli; female; follow up; human; hypertension; incidence; ischemic heart disease; *kidney polycystic disease/cn [Congenital Disorder]; *kidney polycystic disease/th [Therapy]; Klebsiella; male; Mycobacterium tuberculosis; nonhuman; nutritional status; overall survival; *peritoneal dialysis; peritonitis; Pseudomonas; *renal replacement therapy; retrospective study; sample size; Staphylococcus aureus; Streptococcus; therapy effect; treatment outcome.

Link to the Ovid Full Text or citation:

Click here for full text options

65.

Clinical characteristics and treatment outcomes of tuberculosis in hospitals of two large cities in Thailand.

Manosuthi W., Kawkitinarong K., Suwanpimolkul G., Chokbumrungsuk C., Jirawattanapisal T., Ruxrungtham K., Akksilp S.

Clinical Microbiology and Infection. Conference: 21st ECCMID/27th ICC Milan Italy. Conference Start: 20110507 Conference End: 20110510. Conference Publication: (var.pagings). 17 (pp S50), 2011. Date of Publication: May 2011.

AN: 70599050

Background: Tuberculosis (TB) is a major public health threat of large cities in many countries. The current clinical situation of TB in large cities of Thailand has not been well established. Methods: A retrospective cohort study was conducted among patients diagnosed active TB in 6 hospitals in Bangkok and Nonthaburi between January 2008 and December 2008. Patients were classified into HIV sero-positive, sero-negative, and undetermined sero-status; and were followed until December 2009. Results: A total of 813 patients were identified. Mean+/-SD age was 41+/-14 years, 62% were male, and mean+/-SD body weight was 53+/-11 kilograms. The first three co-morbidities included 38% HIV infection, 6% diabetes, and 2% chronic liver disease. Of all, 66% were diagnosed isolated pulmonary TB. Median (IQR) duration from first presentation to TB diagnosis was 3 (0-10) days. Of all, 43% of patients performed TB culture and 16% did anti-TB drug susceptibility test. Isoniazid, rifampicin, and multi-drug resistance were found in 11%, 6% and 5%, respectively. For 1-year TB treatment outcomes, 52.1%, 19.4%, 11.9%, 8.7%, 6.8%, 1.0% were cured/completed treated, transferred out, defaulted, on-going treated, dead and failed, respectively. Survival rates at 2, 6, 12 and 24 months were 93%, 85%, 81%, and 81% in HIV sero-positve group; 96%, 94%, 92%, and 92% in HIV sero-negative group; 98%, 97%, 97%, and 97% in undetermined sero-status (log rank test, P<0.001). Cox's proportional hazard model showed that death was associated with TB/HIV co-infection (P<0.001, HR= 2.801), low body weight (P = 0.005, HR= 1.637), old age (P = 0.005, HR= 1.439), and extrapulmonary/disseminated TB (P = 0.021, HR= 2.184). Conclusions: HIV infection and diabetes are the common co-morbidities in TB patients. Unfavorable outcome of TB treatment is relatively high, particularly in patients with HIV.

Institution

(Manosuthi, Kawkitinarong, Suwanpimolkul, Chokbumrungsuk, Jirawattanapisal, Ruxrungtham, Akksilp) Nonthaburi, BangkokThailand

Publisher

Blackwell Publishing Ltd

Emtree Heading

*Thailand; *city; *treatment outcome; *hospital; *tuberculosis; human; Human immunodeficiency virus; patient; Human immunodeficiency virus infection; morbidity; body weight; diabetes mellitus; survival rate; log rank test; proportional hazards model; death; mixed infection; senescence; public health; cohort analysis; male; chronic liver disease; diagnosis; drug sensitivity; drug resistance; isoniazid; rifampicin.

Link to the Ovid Full Text or citation:

Click here for full text options

66.

The effect of smoking on the treatment outcome in patients with pulmonary tuberculosis: A prospective cohort study.

Castillo M.

Respirology. Conference: 16th Congress of the Asian Pacific Society of Respirology Shanghai China. Conference Start: 20111103 Conference End: 20111106. Conference Publication: (var.pagings). 16 (pp 73), 2011. Date of Publication: November 2011.

AN: 70575950

Smoking and tuberculosis have great impact on health. Smoking is prevalent worldwide occurring in 20% of adults while Tuberculosis is a universal problem. Studies have shown that although smoking has no etiological role in TB, higher prevalence of TB has been observed in smokers. However, only few studies have evaluated the impact of smoking on TB treatment. General Objective To determine the effect of smoking on the treatment outcome among patients with pulmonary tuberculosis. Specific Objectives To determine the prevalence of smoking among patients with PTB, incidence of treatment success and failure among patients treated for PTB and to determine the association between smoking and treatment outcomes. Methodology A prospective cohort study done in Dr. Jose Rodriguez Memorial Hospital, DOTS Centre included all patients diagnosed with PTB, ages 18 years and above. Demographics and smoking history were obtained by standardized interview. CXR and sputum AFB were done. Sputum examination was repeated on 2nd, 4th month and on completion of treatment. Result A total of 197 pulmonary tuberculosis patients were enrolled in the DOTs Center during the study period. Of the 111 patients available for interview, only 105 patients were included in the assessment. There were 38 non smoker (36.2%) and 65 with smoking history (39 current smoker and 28 previous smoker). Patient's mean age was 42.6 +/- 15.4 (mean +/-SD). Female patients predominate among non smokers (73.7%) while male gender among current and previous smokers (84.6% and 82.1% respectively). Co-existing illnesses are hypertension, diabetes, asthma and COPD. Current smokers have more progressive CXR findings as manifested by either far advanced/ cavitary lesion (33.3%). Only 67 out of 197 patients had 2 or more sputum positive smear on baseline examination. The rest had either doubtful or negative smear but was assessed by the TB Diagnostic Committee. Among the 67 smear positive patients, 40 patients are +3 (38.1%), and 27 patient are +2 (25.7%). The remaining 38 subjects are either sputum +1 (14.3%) or sputum smear negative (21.9%). Of the 105 patients, 5 patients were classified treatment failure, 1 non-smoker, 2 previous smokers and 2 current smokers. Conclusion: Patients under DOTS program have high success rate. Although the study did not reach statistical significance, there was a strong trend on the association of smoking with cavitary or far advanced PTB and treatment failure.

Institution

(Castillo) Division of Pulmonary, Critical Care Medicine, Philippine Heart Center, Quezon, Philippines

Publisher

Blackwell Publishing

Emtree Heading

*smoking; *lung tuberculosis; *human; *patient; *society; *treatment outcome; *Asian; *cohort analysis; sputum; smear; interview; prevalence; tuberculosis; treatment failure; short course therapy; health; adult; methodology; hospital; sputum examination; male; gender; general aspects of disease; hypertension; diabetes mellitus; asthma; examination; diagnosis; sputum smear; statistical significance; female.

Link to the Ovid Full Text or citation:

Click here for full text options

67.

Years lived with disability (YLDs) for 1160 sequelae of 289 diseases and injuries 1990-2010: A systematic analysis for the Global Burden of Disease Study 2010.

Vos T., Flaxman A.D., Naghavi M., Lozano R., Michaud C., Ezzati M., Shibuya K., Salomon J.A., Abdalla S., Aboyans V., Abraham J., Ackerman I., Aggarwal R., Ahn S.Y., Ali M.K., Alvarado M., Anderson H.R., Anderson L.M., Andrews K.G., Atkinson C., Baddour L.M., Bahalim A.N., Barker-Collo S., Barrero L.H., Bartels D.H., Basanez M.-G., Baxter A., Bell M.L., Benjamin E.J., Bennett D., Bernabe E., Bhalla K., Bhandari B., Bikbov B., Abdulhak A.B., Birbeck G., Black J.A., Blencowe H., Blore J.D., Blyth F., Bolliger I., Bonaventure A., Boufous S., Bourne R., Boussinesq M., Braithwaite T., Brayne C., Bridgett L., Brooker S., Brooks P., Brugha T.S., Bryan-Hancock C., Bucello C., Buchbinder R., Buckle G., Budke C.M., Burch M., Burney P., Burstein R., Calabria B., Campbell B., Canter C.E., Carabin H., Carapetis J., Carmona L., Cella C., Charlson F., Chen H., Cheng A.T.-A., Chou D., Chugh S.S., Coffeng L.E., Colan S.D., Colquhoun S., Colson K.E., Condon J., Connor M.D., Cooper L.T., Corriere M., Cortinovis M., De Vaccaro K.C., Couser W., Cowie B.C., Criqui M.H., Cross M., Dabhadkar K.C., Dahiya M., Dahodwala N., Damsere-Derry J., Danaei G., Davis A., De Leo D., Degenhardt L., Dellavalle R., Delossantos A., Denenberg J., Derrett S., Des Jarlais D.C., Dharmaratne S.D., Dherani M., Diaz-Torne C., Dolk H., Dorsey E.R., Driscoll T., Duber H., Ebel B., Edmond K., Elbaz A., Ali S.E., Erskine H., Erwin P.J., Espindola P., Ewoigbokhan S.E., Farzadfar F., Feigin V., Felson D.T., Ferrari A., Ferri C.P., Fevre E.M., Finucane M.M., Flaxman S., Flood L., Foreman K., Forouzanfar M.H., Fowkes F.G.R., Franklin R., Fransen M., Freeman M.K., Gabbe B.J., Gabriel S.E., Gakidou E., Ganatra H.A., Garcia B., Gaspari F., Gillum R.F., Gmel G., Gonzalez-Medina D., Gosselin R., Grainger R., Grant B., Groeger J., Guillemin F., Gunnell D., Gupta R., Haagsma J., Hagan H., Halasa Y.A., Hall W., Haring D., Haro J.M., Harrison J.E., Havmoeller R., Hay R.J., Higashi H., Hill C., Hoen B., Hoffman H., Hotez P.J., Hoy D., Huang J.J., Ibeanusi S.E., Jacobsen K.H., James S.L., Jarvis D., Jasrasaria R., Jayaraman S., Johns N., Jonas J.B., Karthikeyan G., Kassebaum N., Kawakami N., Keren A., Khoo J.-P., King C.H., Knowlton L.M., Kobusingye O., Koranteng A., Krishnamurthi R., Laden F., Lalloo R., Laslett L.L., Lathlean T., Leasher J.L., Lee Y.Y., Leigh J., Levinson D., Lim S.S., Limb E., Lin J.K., Lipnick M., Lipshultz S.E., Liu W., Loane M., Ohno S.L., Lyons R., Ma J., Mabweijano J., MacIntyre M.F., Malekzadeh R., Mallinger L., Manivannan S., Marcenes W., March L., Margolis D.J., Marks G.B., Marks R., Matsumori A., Matzopoulos R., Mayosi B.M., McAnulty J.H., McDermott M.M., McGill N., McGrath J., Medina-Mora M.E., Meltzer M., Mensah G.A., Merriman T.R., Meyer A.-C., Miglioli V., Miller M., Miller T.R., Mitchell P.B., Mock C., Mocumbi A.O., Moffitt T.E., Mokdad A.A., Monasta L., Montico M., Moran A., Morawska L., Mori R., Murdoch M.E., Mwaniki M.K., Naidoo K., Nair M.N., Naldi L., Narayan K.M.V., Nelson P.K., Nelson R.G., Nevitt M.C., Newton C.R., Nolte S., Norman P., Norman R., O'Donnell M., O'Hanlon S., Olives C., Omer S.B., Ortblad K., Osborne R., Ozgediz D., Page A., Pahari B., Pandian J.D., Rivero A.P., Patten S.B., Pearce N., Padilla R.P., Perez-Ruiz F., Perico N., Pesudovs K., Phillips D., Phillips M.R., Pierce K., Pion S., Polanczyk G.V., Polinder S., Pope III C.A., Popova S., Porrini E., Pourmalek F., Prince M., Pullan R.L., Ramaiah K.D., Ranganathan D., Razavi H., Regan M., Rehm J.T., Rein D.B., Remuzzi G., Richardson K., Rivara F.P., Roberts T., Robinson C., De Leon F.R., Ronfani L., Room R., Rosenfeld L.C., Rushton L., Sacco R.L., Saha S., Sampson U., Sanchez-Riera L., Sanman E., Schwebel D.C., Scott J.G., Segui-Gomez M., Shahraz S., Shepard D.S., Shin H., Shivakoti R., Singh D., Singh G.M., Singh J.A., Singleton J., Sleet D.A., Sliwa K., Smith E., Smith J.L., Stapelberg N.J.C., Steer A., Steiner T., Stolk W.A., Stovner L.J., Sudfeld C., Syed S., Tamburlini G., Tavakkoli M., Taylor H.R., Taylor J.A., Taylor W.J., Thomas B., Thomson W.M., Thurston G.D., Tleyjeh I.M., Tonelli M., Towbin J.A., Truelsen T., Tsilimbaris M.K., Ubeda C., Undurraga E.A., Van Der Werf M.J., Van Os J., Vavilala M.S., Venketasubramanian N., Wang M., Wang W., Watt K., Weatherall D.J., Weinstock M.A., Weintraub R., Weisskopf M.G., Weissman M.M., White R.A., Whiteford H., Wiebe N., Wiersma S.T., Wilkinson J.D., Williams H.C., Williams S.R.M., Witt E., Wolfe F., Woolf A.D., Wulf S., Yeh P.-H., Zaidi A.K.M., Zheng Z.-J., Zonies D., Lopez A.D., Murray C.J.L., Moradi-Lakeh M.

The Lancet. 380 (9859) (pp 2163-2196), 2012. Date of Publication: December 2012.

AN: 2012732549

Background Non-fatal health outcomes from diseases and injuries are a crucial consideration in the promotion and monitoring of individual and population health. The Global Burden of Disease (GBD) studies done in 1990 and 2000 have been the only studies to quantify non-fatal health outcomes across an exhaustive set of disorders at the global and regional level. Neither effort quantified uncertainty in prevalence or years lived with disability (YLDs). Methods Of the 291 diseases and injuries in the GBD cause list, 289 cause disability. For 1160 sequelae of the 289 diseases and injuries, we undertook a systematic analysis of prevalence, incidence, remission, duration, and excess mortality. Sources included published studies, case notification, population-based cancer registries, other disease registries, antenatal clinic serosurveillance, hospital discharge data, ambulatory care data, household surveys, other surveys, and cohort studies. For most sequelae, we used a Bayesian meta-regression method, DisMod-MR, designed to address key limitations in descriptive epidemiological data, including missing data, inconsistency, and large methodological variation between data sources. For some disorders, we used natural history models, geospatial models, back-calculation models (models calculating incidence from population mortality rates and case fatality); or registration completeness models; (models adjusting for incomplete registration with health-system access and other covariates). Disability weights for 220 unique health states were used to capture the severity of health loss. YLDs by cause at age, sex, country, and year levels were adjusted for comorbidity with simulation methods. We included uncertainty estimates at all stages of the analysis. Findings Global prevalence for all ages combined in 2010 across the 1160 sequelae ranged from fewer than one case per 1 million people to 350 000 cases per 1 million people. Prevalence and severity of health loss were weakly correlated (correlation coefficient -0 37). In 2010, there were 777 million YLDs from all causes, up from 583 million in 1990. The main contributors to global YLDs were mental and behavioural disorders, musculoskeletal disorders, and diabetes or endocrine diseases. The leading specific causes of YLDs were much the same in 2010 as they were in 1990: low back pain, major depressive disorder, iron-deficiency anaemia, neck pain, chronic obstructive pulmonary disease, anxiety disorders, migraine, diabetes, and falls. Age-specific prevalence of YLDs increased with age in all regions and has decreased slightly from 1990 to 2010. Regional patterns of the leading causes of YLDs were more similar compared with years of life lost due to premature mortality. Neglected tropical diseases, HIV/AIDS, tuberculosis, malaria, and anaemia were important causes of YLDs in sub-Saharan Africa. Interpretation Rates of YLDs per 100 000 people have remained largely constant over time but rise steadily with age. Population growth and ageing have increased YLD numbers and crude rates over the past two decades. Prevalences of the most common causes of YLDs, such as mental and behavioural disorders and musculoskeletal disorders, have not decreased. Health systems will need to address the needs of the rising numbers of individuals with a range of disorders that largely cause disability but not mortality. Quantification of the burden of non-fatal health outcomes will be crucial to understand how well health systems are responding to these challenges. Effective and affordable strategies to deal with this rising burden are an urgent priority for health systems in most parts of the world.

Institution

(Baxter, Erskine, Ferrari, Keren, Saha, Whiteford) Queensland Centre for Mental Health Research, Australia (Vos, Blore, Charlson, Hay, Leasher, Norman, Page, Lopez) School Population of Healtho, Australia

(Ganatra, Halasa, Patten) Centre for Clinical Research, Australia

(McGill) Queensland Brain Institute, University of Queensland, Brisbane, QLD, Australia

(Flaxman, Naghavi, Lozano, Ahn, Alvarado, Andrews, Atkinson, Bolliger, Burstein, Campbell, Colson, Delossantos, Dharmaratne, Forouzanfar, Fransen, Gabriel, Gmel, Hall, Haro, Jarvis, Jayaraman, Levinson, Loane, Mabweijano, Malekzadeh, Moffitt, Nair, Ortblad, Phillips, Pierce, Ranganathan, Roberts, Rosenfeld, Sanman, Wang, Wulf, Murray) Institute for Health Metrics and Evaluation, University of Washington, 2301 Fifth Avenue, Seattle, WA 98121, United States

(Anderson) Department of Epidemiology, School of Public Health, United States

(Karthikeyan) Department of Anesthesiology and Pain Medicine, United States

(Couser, Duber, Ebel, Mitchell, Olives, Rivara, Vavilala) University of Washington, Seattle, WA, United States

(Michaud) China Medical Board, Boston, MA, United States

(Ezzati) MRCHPA Centre for Environment and Health, Department of Epidemiology and Biostatistics, School of Public Health, United Kingdom

(Basanez, Burney, Foreman, James, O'Hanlon, Rushton) Imperial College London, London, United Kingdom

(Shibuya) Department of Global Health Policy, Japan

(Kassebaum) University of Tokyo, Tokyo, Japan

(Finucane) Department of Biostatistics, United States

(Salomon) School of Public Health, United States

(Danaei, Krishnamurthi, Limb, Miglioli, Singh, Sudfeld, Tavakkoli, Weisskopf, White) 1Department of Epidemiology, United States

(Lipshultz) Harvard Humanitarian Initiative, United States

(King) Boston Childrenaes Hospital, United States

(Colan) Brigham and Womenaes Hospital, United States

(Jasrasaria) Harvard Medical School, Harvard University, Boston, MA, United States

(Bartels) Sudanese Public Health Consultancy Group, Sudan

(Abdalla) Department of Cardiology, Dupuytren University Hospital, Limoges, France

(Aboyans) University of Texas, San Antonio, TX, United States

(Abraham) Centre for International Child Health, Australia

(Steer) Department of Pediatrics, Australia

(Weintraub) Centre for Health Policy, Programs and Economics, Australia

(Degenhardt) School of Population Health, Australia

(Room) University of Melbourne, Melbourne, VIC, Australia

(Ackerman, Brooks, Marks, Taylor) Sanjay Gandhi Postgraduate Institute of Medical Sciences, Lucknow, India

(Aggarwal) Schools of Public Health and Medicine, United States

(Omer) Emory University, Atlanta, GA, United States

(Ali) St Georgeaes University of London, London, United Kingdom

(Dabhadkar, Gakidou, Narayan) Mayo Clinic, Rochester, MN, United States

(Anderson, Lim) Independent Consultant, Geneva, Switzerland

(Gunnell) University of Auckland, Auckland, New Zealand

(Baddour, Erwin, Gabbe) Department of Industrial Engineering, School of Engineering, Pontificia Universidad Javeriana, Bogota, Colombia

(Bahalim) Global Partners in Anesthesia and Surgery, United States

(Barker-Collo) Yale University, New Haven, CT, United States

(Barrero) School of Medicine, United States

(Ozgediz) Boston University, Boston, MA, United States

(Bell, Hoy) Clinical Trial Service Unit and Epidemiological Studies Unit, United Kingdom

(Felson) University of Oxford, Oxford, United Kingdom

(Benjamin) Dental Institute, United Kingdom

(Bennett) Hospital NHS Trust, United Kingdom

(Bernabe) Institute of Psychiatry, Kingaes College London, London, United Kingdom

(Havmoeller) Institute of Dentistry, United Kingdom

(Prince) Queen Mary University of London, London, United Kingdom

(Dahiya) Moscow State University of Medicine and Dentistry, Research Institute of Transplantology and Artificial Organs, Moscow, Russian Federation

(Bhandari) King Fahad Medical City, Riyadh, Saudi Arabia

(Manivannan) Michigan State University, East Lansing, MI, United States

(Bikbov) MRC Epidemiology Unit, Cambridge, United Kingdom

(Abdulhak) London School of Hygiene and Tropical Medicine, London, United Kingdom

(Tleyjeh) Department of Rheumatololgy, Northern Clinical School, Australia

(Birbeck) Faculty of Health Sciences, Australia

(Black) Institute of Bone and Joint Research, Australia

(Blencowe, Brooker, Edmond, Pearce, Pullan, Smith) Sydney School of Public Health, Australia

(Smith) University of Sydney, Sydney, NSW, Australia

(Marcenes, Sanchez-Riera) Inserm, Paris, France

(Driscoll, Lee) Transport and Road Safety Research, Australia

(Blyth, Bridgett, Cross, Margolis, McDermott) National Drug and Alcohol Research Centre, Australia

(Bonaventure, Elbaz) University of New South Wales, Sydney, NSW, Australia

(Boufous) Vision and Eye Research Unit, Anglia Ruskin University, Cambridge, United Kingdom

(Calabria, Degenhardt, Nelson, Singleton) Institut de Recherche pour le Developpement, Martinique

(Bucello, Miller) Moorfields Eye Hospital, London, United Kingdom

(Bourne) University of Cambridge, Cambridge, United Kingdom

(Boussinesq) University of Leicester, Leicester, United Kingdom

(Pion) Flinders University, Adelaide, SA, Australia

(Braithwaite) Cabrini Institute, Malvern, VIC, Australia

(Brayne, Richardson) Department of Epidemiology and Preventive Medicine, Australia

(Brugha) Monash University, Melbourne, VIC, Australia

(Bryan-Hancock) Bloomberg School of Public Health, United States

(Flood, Haro, Laslett, Pesudovs) Johns Hopkins University, Baltimore, MD, United States

(Buchbinder) Texas AandM University, College Station, TX, United States

(Freeman) Great Ormond Street Hospital, London, United Kingdom

(Buchbinder, Hotez) Washington University, St Louis, MO, United States

(Buckle, Mallinger) University of Oklahoma, Health Sciences Center, Oklahoma City, OK, United States

(Dorsey, Shivakoti) Telethon Institute for Child Health Research, Centre for Child Health Research, Australia

(Budke) University of Western Australia, Perth, WA, Australia

(Burch) Universidad Camilo Jose Cela, Villanueva de la Canada, Spain

(Canter) Mario Negri Institute for Pharmacological Research, Bergamo, Italy

(Carabin) National Institute of Environmental Health Sciences, Research Triangle Park, NC, United States

(Carapetis) Institute of Biomedical Sciences, Academia Sinica, Taipei, Taiwan (Republic of China)

(Norman) Heart Institute, United States

(Carmona) Cedars-Sinai Medical Center, Los Angeles, CA, United States

(Cella, Cortinovis, Garcia, Meyer, Perico, Remuzzi) Department of Public Health, United States

(Chen) Erasmus MC, University Medical Center Rotterdam, Rotterdam, Netherlands

(Cheng) Menzies School of Health Research, Darwin, NT, Australia

(Harrison) National Health Services, Fife, Edinburgh, United Kingdom

(Chugh) University of Edinburgh, Edinburgh, United Kingdom

(Polinder) University of the Witwatersrand, Johannesburg, South Africa

(Coffeng, Gupta, Stolk) Loyola University Medical School, Chicago, IL, United States

(Colquhoun, Condon) School of Public Health Sciences, Wake Forest University, Winston-Salem, NC, United States

(Connor, Fowkes) Hospital Dr Gustavo N Collado, Puerto Chitre, Panama

(Connor, Fevre) Victorian Infectious Diseases Reference Laboratory, Melbourne, VIC, Australia

(Cooper) University of California, San Diego, San Diego, CA, United States

(Corriere) University of Pennsylvania, Philadelphia, PA, United States

(De Vaccaro) Building and Road Research Institute, Kumasi, Ghana

(Cowie) MRC Hearing and Communication Group, Manchester, United Kingdom

(Criqui, Denenberg) School of Dentistry and Oral Health, Australia

(Dahodwala, March) Population and Social Health Research Program, Australia

(Damsere-Derry) Griffith University, Brisbane, QLD, Australia

(Davis, Laden) Denver VA Medical Center, Denver, CO, United States

(Laden) University of Otago, Dunedin, New Zealand

(De Leo, Stapelberg) Beth Israel Medical Center, New York City, NY, United States

(Dellavalle) University of Peradeniya, Peradeniya, Sri Lanka

(Derrett, Gosselin, Mensah, Taylor, Thomson) University of Liverpool, Liverpool, United Kingdom

(Des Jarlais) Hospital de la Santa Creu i Sant Pau, Barcelona, Spain

(Dharmaratne) University of Ulster, Ulster, United Kingdom

(Dherani) Federal Ministry of Health, Khartoum, Sudan

(Diaz-Torne) Hospital Maciel, Montevideo, Uruguay

(Dolk, Liu) Emerald Public Health Consulting Services Ltd, Abuja, Nigeria

(Moffitt) Digestive Disease Research Center, Iran, Islamic Republic of

(Espindola, Moradi-Lakeh) Tehran University of Medical Sciences, Tehran, Iran, Islamic Republic of

(Ewoigbokhan) National Institute for Stroke and Applied Neurosciences, New Zealand

(MacIntyre) Auckland Technical University, Auckland, New Zealand

(Farzadfar, Montico) Medical School, Brazil

(Feigin) Federal University of Sao Paulo, Sao Paulo, Brazil

(Koranteng, Witt) Carnegie Mellon University, Pittsburgh, PA, United States

(Polanczyk) James Cook University, Townsville, QLD, Australia

(Ferri) Howard University, College of Medicine, Washington, DC, United States

(Watt) Addiction Info Switzerland, Lausanne, Switzerland

(Gaspari) Department of Epidemiology and Biostatistics, United States

(Gillum) University of California, San Francisco, San Francisco, CA, United States

(Nevitt) College of Medicine, SUNY Downstate Medical Center, Brooklyn, NY, United States

(Gonzalez-Medina, Lin, Merriman, Robinson) National Center for Injury Prevention and Control, United States

(Grant) Centers for Disease Control and Prevention, Atlanta, GA, United States

(Sleet) Universite de Lorraine, Nancy, France

(Wiersma) University of Bristol, Bristol, United Kingdom

(Groeger) New York University, New York City, NY, United States

(Guillemin) Brandeis University, Waltham, MA, United States

(Haagsma) Parc Sanitari Sant Joan de Deu, CIBERSAM, Universitat de Barcelona, Sant Boi de Llobregat, Spain

(Thurston) Karolinska University Hospital, Stockholm, Sweden

(Hagan) Queen Elizabeth Hospital, Adelaide, SA, Australia

(Shahraz) Universite de Franche-Comte, Besancon, France

(Shepard) Centre Hospitalier Regional, Universitaire de Besancon, Besancon, France

(Undurraga) National Institute on Deafness and Other Communication Disorders, United States

(Haring) National Institute of Diabetes and Digestive and Kidney Diseases, National Institutes of Health, Bethesda, MD, United States

(Harrison) National School of Tropical Medicine, Baylor College of Medicine, Waco, TX, United States

(Higashi) University of Port Harcourt, Port Harcourt, Nigeria

(Hill) George Mason University, Fairfax, VA, United States

(Hill) Department of Ophthalmology, Medical Faculty Mannheim, Ruprecht Karls University, Heidelberg, Germany

(Hoen) All India Institute of Medical Sciences, New Delhi, India

(Nelson) Department of Cardiology, Hebrew University Hadassah Medical School, Jerusalem, Israel

(Hoffman) Case Western Reserve University, Cleveland, OH, United States

(Huang) School of Public Health, Makerere University, Kampala, Uganda

(Ibeanusi, Johns) University of South Africa, Johannesburg, South Africa

(Jonas) Kwame Nkrumah University of Science and Technology, Kumasi, Ghana

(Kawakami) University of Tasmania, TAS, Australia

(Khoo) Nova Southeastern University, Fort Lauderdale, FL, United States

(Knowlton) Ministry of Health, Jerusalem, Israel

(Knowlton) Miller School of Medicine, University of Miami, Miami, FL, United States

(Kobusingye) Swansea University, Swansea, United Kingdom

(Lalloo) Mulago Hospital, Kampala, Uganda

(Lathlean) Asian Pacific Society of Cardiology, Kyoto, Japan

(Leigh, Lipnick, Sacco, Wilkinson) Medical Research Council, Tygerberg, South Africa

(Ohno) Hatter Institute, South Africa

(Lyons) Department of Medicine, South Africa

(Marks) University of Cape Town, Cape Town, South Africa

(Matsumori) Legacy Health System, Portland, OR, United States

(Sliwa) Northwestern University Feinberg, School of Medicine, Evanston, IL, United States

(Meltzer) National Institute on Psychiatry Ramon de la Fuente, Mexico City, Mexico

(Matsumori, Matzopoulos) Thomas Jefferson University, Philadelphia, PA, United States

(Mayosi) Pacific Institute for Research and Evaluation, Calverton, MD, United States

(McAnulty) National Institute of Health, Maputo, Mozambique

(McGrath) University Eduardo Mondlane, Maputo, Mozambique

(Medina-Mora) Duke University, Durham, NC, United States

(Miller) Institute for Maternal and Child Health, IRCCS Burlo Garofolo, Trieste, Italy

(Mock) Columbia University, New York City, NY, United States

(Mock) Queensland University of Technology, Brisbane, QLD, Australia

(Mocumbi, Mokdad, Monasta, Ronfani, Tamburlini) National Center for Child Health and Development, Tokyo, Japan

(Moran, Weissman) Watford General Hospital, Watford, United Kingdom

(Morawska) Kemri-Wellcome Trust, Kilifi, Kenya

(Mori) AVRI, University of KwaZulu-Natal, Durban, South Africa

(Murdoch) Centro Studi GISED, Bergamo, Italy

(Mwaniki) Charite- Universitatsmedizin Berlin, Berlin, Germany

(Naidoo) HRB-Clinical Research Facility, National University of Ireland Galway, Galway, Ireland

(Naldi) Deakin University, Melbourne, VIC, Australia

(Newton) B P Koirala Institute of Health Sciences, Dharan, Nepal

(O'Donnell) Betty Cowan Research and Innovation Center, Ludhiana, India

(Osborne) Hospital Juan XXIII, La Paz, Bolivia

(Pahari) University of Calgary, Calgary, AB, Canada

(Pandian) Instituto Nacional de Enfermedades Respiratorias, Mexico City, Mexico

(Rivero) Hospital Universitario Cruces, Barakaldo, Spain

(Patten) Shanghai Mental Health Center, School of Medicine, China

(Padilla) School of Public Health, Shanghai Jiao Tong University, Shanghai, China

(Perez-Ruiz) Brigham Young University, Provo, UT, United States

(Phillips) Centre for Addiction and Mental Health, Toronto, ON, Canada

(Zheng) Hospital Universitario de Canarias, Tenerif, Spain

(Pope III) Faculty of Medicine, School of Population and Public Health, University of British Columbia, Vancouver, BC, Canada

(Popova) Vector Control Research Centre, Pondicherry, India

(Rehm) Center for Disease Analysis, Louisville, CO, United States

(Porrini) University of California, Berkeley, Berkeley, CA, United States

(Pourmalek) NORC, University of Chicago, Chicago, IL, United States

(Ramaiah) Complejo Hospitalario Caja De Seguro Social, Panama City, Panama

(Razavi) Centre for Alcohol Policy Research, Turning Point Alcohol and Drug Centre, Fitzroy, SA, Australia

(Regan, Sampson) Vanderbilt University, Nashville, TN, United States

(Rein, Schwebel) University of Alabama at Birmingham, Birmingham, AL, United States

(De Leon) Ministry of Interior, Madrid, Spain

(Room) Health Canada, Ottawa, ON, Canada

(Stovner) Queens Medical Center, Honolulu, HI, United States

(Singh) Department of Neuroscience, Norway

(Segui-Gomez, Taylor) Norwegian University of Science and Technology, Trondheim, Norway

(Shin) Aga Khan University, Karachi, Pakistan

(Tonelli) Drexel University, School of Public Health, Philadelphia, PA, United States

(Steiner, Tsilimbaris, Wiebe) Alberta Kidney Disease Network, Canada

(Towbin, Zaidi) University of Alberta, Edmonton, AB, Canada

(Truelsen) Cincinnati Childrenaes Hospital, Cincinnati, OH, United States

(Syed) Department of Neurology, Copenhagen University Hospital, Herlev, Denmark

(Ubeda) University of Crete Medical School, Crete, Greece

(Van Der Werf) Instituto Nacional de Epidemiologia, ANLIS, Malbran, Argentina

(Van Os) KNCV Tuberculosis Foundation, The Hague, Netherlands

(Thomas) Maastricht University Medical Centre, Maastricht, Netherlands

(Wang) National University of Singapore, Singapore

(Weinstock) Beijing Neurosurgical Institute, Capital Medical University, Beijing, China

(Grainger) Brown University, Providence, RI, United States

(Venketasubramanian, Williams) Royal Childrenaes Hospital, Critical Care and Neurosciences Theme, Murdoch Childrenaes Research Institute, Melbourne, VIC, Australia

(Williams, Woolf) University of Nottingham, Nottingham, United Kingdom

(Williams) Hollywood Orthopaedic Group, Perth, WA, Australia

(Wolfe) Arthritis Research, Wichita, KS, United States

(Weintraub) Royal Cornwall Hospital, Truro, United Kingdom

(Weintraub, Yeh) London School of Economics, London, United Kingdom

(Zonies) Landstuhl Regional Medical Center, Landstuhl, Germany

(Ma) National Center for Non-Communicable Disease Control and Prevention, China Centers for Disease Control, Beijing, China

Publisher

Lancet Publishing Group (Langford Lane, Kidlington, Oxford OX5 1GB, United Kingdom)

Emtree Heading

acquired immune deficiency syndrome/co [Complication]; acquired immune deficiency syndrome/dt [Drug Therapy]; acquired immune deficiency syndrome/ep [Epidemiology]; age distribution; aging; anemia/co [Complication]; anemia/ep [Epidemiology]; antiviral therapy; anxiety disorder/co [Complication]; anxiety disorder/ep [Epidemiology]; article; behavior disorder/co [Complication]; behavior disorder/ep [Epidemiology]; chronic obstructive lung disease/co [Complication]; chronic obstructive lung disease/ep [Epidemiology]; comorbidity; diabetes mellitus/co [Complication]; diabetes mellitus/ep [Epidemiology]; *disability; disability severity; disease duration; endocrine disease/co [Complication]; endocrine disease/ep [Epidemiology]; falling; health care system; human; Human immunodeficiency virus infection/co [Complication]; Human immunodeficiency virus infection/ep [Epidemiology]; *iatrogenic disease/co [Complication]; *iatrogenic disease/ep [Epidemiology]; incidence; iron deficiency anemia/co [Complication]; iron deficiency anemia/ep [Epidemiology]; low back pain/co [Complication]; low back pain/ep [Epidemiology]; major depression/co [Complication]; major depression/ep [Epidemiology]; malaria/co [Complication]; malaria/ep [Epidemiology]; mental disease/co [Complication]; mental disease/ep [Epidemiology]; migraine/co [Complication]; migraine/ep [Epidemiology]; mortality; musculoskeletal disease/co [Complication]; musculoskeletal disease/ep [Epidemiology]; neck pain/co [Complication]; neck pain/ep [Epidemiology]; outcome assessment; population growth; prevalence; priority journal; remission; systematic review; tropical disease/co [Complication]; tropical disease/ep [Epidemiology]; tuberculosis/co [Complication]; tuberculosis/ep [Epidemiology]; *years lived with disability; anti human immunodeficiency virus agent/dt [Drug Therapy].

Link to the Ovid Full Text or citation:

Click here for full text options

68.

The role of diabetes co-morbidity for tuberculosis treatment outcomes: a prospective cohort study from Mwanza, Tanzania.

Faurholt-Jepsen D., Range N., Praygod G., Kidola J., Faurholt-Jepsen M., Aabye M.G., Changalucha J., Christensen D.L., Martinussen T., Krarup H., Witte D.R., Andersen T.B., Friis H.

BMC Infectious Diseases. 12 (no pagination), 2012. Article Number: 165. Date of Publication: 27 Jul 2012.

AN: 2012579890

Background: Due to the association between diabetes and pulmonary tuberculosis (TB), diabetes may threaten the control of TB. In a prospective cohort study nested in a nutrition trial, we investigated the role of diabetes on changes in anthropometry, grip strength, and clinical parameters over a five months follow-up period.Methods: Among pulmonary TB patients with known diabetes status, we assessed anthropometry and clinical parameters (e.g. haemoglobin) at baseline and after two and five months of TB treatment. A linear mixed-effects model (repeated measurements) was used to investigate the role of diabetes during recovery.Results: Of 1205 TB patients, the mean (standard deviation) age was 36.6 (13.0) years, 40.9% were females, 48.9% were HIV co-infected, and 16.3% had diabetes. TB patients with diabetes co-morbidity experienced a lower weight gain at two (1.3 kg, CI95% 0.5; 2.0, p = 0.001) and five months (1.0 kg, CI95% 0.3; 1.7, p = 0.007). Similarly, the increase in the level of haemoglobin was lower among TB patients with diabetes co-morbidity after two (DELTA 0.6 g/dL, CI95% 0.3; 0.9 p < 0.001) and five months (DELTA 0.5 g/dL, CI95% 0.2; 0.9 p = 0.004) of TB treatment, respectively.Conclusion: TB patients initiating TB treatment with diabetes co-morbidity experience delayed recovery of body mass and haemoglobin, which are important for the functional recovery from disease. © 2012 Faurholt-Jepsen et al.; licensee BioMed Central Ltd.

Institution

(Faurholt-Jepsen, Faurholt-Jepsen, Friis) Department of Human Nutrition, University of Copenhagen, Frederiksberg, Denmark (Range) Muhimbili Medical Centre, National Institute of Medical Research, Dar Es Salaam, Tanzania

(Praygod, Kidola, Changalucha) Mwanza Medical Centre, National Institute of Medical Research, Mwanza, Tanzania

(Aabye) Clinical Research Centre, University of Copenhagen, Hvidovre Hospital, Hvidovre, Denmark

(Christensen) Department of International Health, University of Copenhagen, Copenhagen, Denmark

(Christensen, Witte) Steno Diabetes Center, Gentofte, Denmark

(Martinussen) Department of Basic Sciences and Environment, University of Copenhagen, Frederiksberg, Denmark

(Krarup) Department of Clinical Biochemistry, Aalborg University Hospital, Aalborg, Denmark

(Andersen) Department of Infectious Diseases, Odense University Hospital, Odense, Denmark

Publisher

BioMed Central Ltd. (Floor 6, 236 Gray's Inn Road, London WC1X 8HB, United Kingdom)

Emtree Heading

adult; arm circumference; article; body mass; CD4 lymphocyte count; cohort analysis; comorbidity; controlled study; *diabetes mellitus; female; follow up; grip strength; human; leukocyte count; *lung tuberculosis/dt [Drug Therapy]; major clinical study; male; mixed infection; prospective study; skinfold thickness; Tanzania; treatment outcome; waist circumference; weight gain; hemoglobin/ec [Endogenous Compound]; tuberculostatic agent/dt [Drug Therapy].

Link to the Ovid Full Text or citation:

Click here for full text options

69.

Cohort monitoring of persons with hypertension: An illustrated example from a primary healthcare clinic for Palestine refugees in Jordan.

Khader A., Farajallah L., Shahin Y., Hababeh M., Abu-Zayed I., Kochi A., Harries A.D., Zachariah R., Kapur A., Venter W., Seita A.

Tropical Medicine and International Health. 17 (9) (pp 1163-1170), 2012. Date of Publication: September 2012.

AN: 2012510847

Objective Recording and reporting systems borrowed from the DOTS framework for tuberculosis control can be used to record, monitor and report on chronic disease. In a primary healthcare clinic run by UNRWA in Amman, Jordan, serving Palestine refugees with hypertension, we set out to illustrate the method of cohort reporting for persons with hypertension by presenting on quarterly and cumulative case finding, cumulative and 12-month analysis of cohort outcomes and to assess how these data may inform and improve the quality of hypertension care services. Method This was a descriptive study using routine programme data collected through E-Health. Results There were 97 newly registered patients with hypertension in quarter 1, 2012, and a total of 4130 patients with hypertension ever registered since E-Health started in October 2009. By 31 March 2012, 3119 (76%) of 4130 patients were retained in care, 878 (21%) had failed to present to a healthcare worker in the last 3months and the remainder had died, transferred out or were lost to follow-up. Cumulative and 12-month cohort outcome analysis indicated deficiencies in several components of clinical performance related to blood pressure measurements and fasting blood glucose tests to screen simultaneously for diabetes. Between 8% and 15% of patients with HT had serious complications such as cardiovascular disease and stroke. Conclusion Cohort analysis is a valuable tool for the monitoring and management of non-communicable chronic diseases such as HT. © 2012 Blackwell Publishing Ltd.

Institution

(Khader, Farajallah, Shahin, Hababeh, Venter, Seita) United Nations Relief and Works Agency for Palestine Refugees in the Near East, UNRWA HQ (A), Amman, Jordan (Abu-Zayed) Field Health Programme, UNRWA, Amman, Jordan

(Kochi) Tokyo University Medical School, Tokyo, Japan

(Harries) International Union Against Tuberculosis and Lung Disease, Paris, France

(Harries) London School of Hygiene and Tropical Medicine, London, United Kingdom

(Zachariah) Medecins sans Frontieres, Medical Department, Operational Research Unit, Brussels Operational Center, Luxembourg, Luxembourg

(Kapur) World Diabetes Foundation, Gentofte, Denmark

Publisher

Blackwell Publishing Ltd (9600 Garsington Road, Oxford OX4 2XG, United Kingdom)

Emtree Heading

adult; article; blood pressure measurement; cardiovascular disease/co [Complication]; cohort analysis; descriptive research; diabetes mellitus; female; follow up; glucose blood level; health care quality; human; *hypertension/dt [Drug Therapy]; Jordan; major clinical study; male; mortality; outcomes research; Palestine; patient monitoring; primary health care; refugee; retrospective study; stroke/co [Complication]; amiloride/dt [Drug Therapy]; atenolol/dt [Drug Therapy]; diltiazem/dt [Drug Therapy]; enalapril/dt [Drug Therapy]; furosemide/dt [Drug Therapy]; glucose/ec [Endogenous Compound]; hydrochlorothiazide/dt [Drug Therapy]; methyldopa/dt [Drug Therapy]; nifedipine/dt [Drug Therapy]; propranolol/dt [Drug Therapy].

Link to the Ovid Full Text or citation:

Click here for full text options

70.

Therapeutic drug monitoring for slow response to tuberculosis treatment in a state control program, Virginia, USA.

Heysell S.K., Moore J.L., Keller S.J., Houpt E.R.

Emerging Infectious Diseases. 16 (10) (pp 1546-1553), 2010. Date of Publication: October 2010.

AN: 2010592688

Therapeutic drug monitoring may be useful in tuberculosis management, but programmatic implementation is understudied. We performed a retrospective cohort study to determine prevalence of lower than expected levels of isoniazid, rifampin, ethambutol, and pyrazinamide measured at time of estimated peak serum concentration. Patients were tested for serum concentration at 2 hours after medication administration. When patients were tested, 22 had concen- trations lower than expected range for rifampin, 23 of 39 patients had low levels of isoniazid, and 8 of 26 patients had low levels of ethambutol; all 20 patients tested for pyrazinamide were within expected range. Over 26 months, 42 patients met criteria for slow response. Diabetes was associated with slow response (p<0.001), and persons with diabetes were more likely than persons without diabetes to have low rifampin levels (p = 0.03). Dosage adjustment of rifampin was more likely to elevate serum concentration to the target range than adjustment of isoniazid given in daily doses (p = 0.01).

Institution

(Heysell, Houpt) University of Virginia, Charlottesville, VA, United States (Moore, Keller) Virginia Department of Health, Richmond, VA, United States

Publisher

Centers for Disease Control and Prevention (CDC) (1600 Clifton Road, Mailstop C-12, Atlanta GA 30333, United States)

Emtree Heading

adult; aged; article; cohort analysis; controlled study; diabetes mellitus; drug blood level; drug dose increase; *drug monitoring; drug program; drug sensitivity; female; human; *lung tuberculosis/dt [Drug Therapy]; major clinical study; male; maximum plasma concentration; outcome assessment; retrospective study; *treatment response; United States; *ethambutol/an [Drug Analysis]; *ethambutol/cb [Drug Combination]; *ethambutol/cr [Drug Concentration]; *ethambutol/dt [Drug Therapy]; *isoniazid/an [Drug Analysis]; *isoniazid/cb [Drug Combination]; *isoniazid/cr [Drug Concentration]; *isoniazid/do [Drug Dose]; *isoniazid/dt [Drug Therapy]; *pyrazinamide/an [Drug Analysis]; *pyrazinamide/cb [Drug Combination]; *pyrazinamide/cr [Drug Concentration]; *pyrazinamide/dt [Drug Therapy]; *rifampicin/an [Drug Analysis]; *rifampicin/cb [Drug Combination]; *rifampicin/cr [Drug Concentration]; *rifampicin/dt [Drug Therapy].

Link to the Ovid Full Text or citation:

Click here for full text options

71.

Tuberculosis treatment outcomes: A retrospective cohort analysis of smoking versus non-smoking patients in the state of Penang, Malaysia.

Dujaili J.A., Sulaiman S.A.S., Awaisu A., Muttalif A.R., Jaafar A.Q.

Pharmacotherapy. Conference: 2010 Annual Meeting of the American College of Clinical Pharmacy, ACCP 2010 Austin, TX United States. Conference Start: 20101017 Conference End: 20101020. Conference Publication: (var.pagings). 30 (10) (pp 402e-403e), 2010. Date of Publication: October 2010.

AN: 71764599

Purpose: The association between tobacco smoking and tuberculosis (TB) is increasingly coming to light and the literature is laden with the evidence of this association. However, only a few observational studies specifically investigated the association between smoking and TB treatment outcomes. The present study aims to compare the treatment outcomes between smoking and non-smoking TB patients. Methods: A retrospective cohort study comparing smoking and nonsmoking TB patients with regards to treatment outcomes was conducted. The data were extracted from medical records of newly diagnosed TB patients of both sexes, above 15 years of age whose smoking status was documented and who registered at a chest clinic of a tertiary care hospital in Penang, Malaysia from January 1 2006 to June 30 2008. Category II (TB relapse, treatment failure and treatment default), Category III (chronic cases) and HIV co-infected TB patients were excluded from the study. Furthermore, other Category I cases comprising of pregnant women, patients with diabetes and those with incomplete data were also excluded. Results: Smoking was significantly associated with male gender, alcohol use, and intravenous drug use. Ever smokers had increased likelihood of treatment failure (OR=7.48; CI 0.93, 60.30), default (OR=7.17; CI 2.76, 18.62) and were less likely to be cured (OR=0.34; CI 0.21, 0.49). After controlling for the effects of confounders using multivariate logistic regression, ever smokers were still less likely to be cured (aOR=0.31; CI 0.17, 0.57) and more likely to default treatment (aOR=3.24; CI 1.01, 10.45). Dying from TB did not reach statistical significance. Conclusion: This study further reaffirms that smoking is an independent predictor of poor TB treatment outcomes and prognosis. Therefore, smoking cessation interventions should be offered to all TB patients who are smokers when they are undergoing TB treatment.

Institution

(Dujaili, Sulaiman, Awaisu, Jaafar) School of Pharmaceutical Science, Universiti Sains Malaysia, Minden, Malaysia (Muttalif) Department of Respiratory Medicine, Penang Hospital, Jalan Residensi, Malaysia

Publisher

Pharmacotherapy Publications Inc.

Emtree Heading

*smoking; *treatment outcome; *cohort analysis; *patient; *human; *Malaysia; *American; *college; *clinical pharmacy; *tuberculosis; treatment failure; pregnant woman; tertiary care center; relapse; male; female; smoking cessation; prognosis; hospital; logistic regression analysis; thorax; medical record; drug use; alcohol consumption; gender; statistical significance; diabetes mellitus; observational study; Human immunodeficiency virus.

Link to the Ovid Full Text or citation:

Click here for full text options

72.

Impact of diabetes mellitus on treatment outcomes of patients with active tuberculosis.

Dooley K.E., Tang T., Golub J.E., Dorman S.E., Cronin W.

American Journal of Tropical Medicine and Hygiene. 80 (4) (pp 634-639), 2009. Date of Publication: April 2009.

AN: 2009288272

Diabetes mellitus (DM) is an emerging chronic health condition of developed and developing countries. We conducted a retrospective cohort study of patients with active, culture-confirmed tuberculosis (TB) in Maryland to determine the impact of DM on TB treatment outcomes. Of 297 TB patients, 42 (14%) had DM. Patients with diabetes had 2.0 times higher odds of death than patients without diabetes (95% confidence interval [CI] 0.74-5.2, P - 0.18). Adjusting for human immunodeficiency virus (HIV), age, weight, and foreign birth, the odds of death were 6.5 times higher in patients with diabetes than patients without diabetes (95% CI 1.1-38.0, P = 0.039). In pulmonary TB patients, time to sputum culture conversion was longer in patients with diabetes than patients without diabetes (median 49 versus 39 days, P = 0.09). Two-month culture conversion proportions were similar (70% and 69%). Treatment failure occurred in 4.1% of patients without diabetes and 6.7% of patients with diabetes (P = 0.51). In conclusion, DM was a risk factor for death in Maryland TB patients. There was a trend toward increased time to culture conversion; two-month culture conversion proportions, however, were similar. Copyright © 2009 by The American Society of Tropical Medicine and Hygiene.

Institution

(Dooley) Division of TB Control, Refugee and Migrant Health, Maryland Department of Health and Mental Hygiene, 201 W. Preston Street, Baltimore, MD 21201 (Tang) Division of Clinical Pharmacology and Infectious Diseases, Johns Hopkins University, School of Medicine, 600 N. Wolfe Street Osier 527, Baltimore, MD 21287

(Golub) Johns Hopkins University Bloomberg, School of Medicine, 615 N.Wolfe Street, Baltimore, MD 21205

(Dorman) Center for Tuberculosis Research, Johns Hopkins University, School of Medicine, 1550 Orleans Street, Baltimore, MD 21231

(Cronin) Center for Tuberculosis Research, Johns Hopkins University, School of Medicine

Publisher

American Society of Tropical Medicine and Hygiene (111 Deer Lake Road, Suite 100,Deerfield, Illinois 60015, United States)

Emtree Heading

adult; article; bacterium culture; cohort analysis; confidence interval; controlled study; *diabetes mellitus; disease association; drug sensitivity; ethnicity; female; human; Human immunodeficiency virus; lung tuberculosis/dt [Drug Therapy]; major clinical study; male; miliary tuberculosis; mortality; multidrug resistance; Mycobacterium tuberculosis; retrospective study; risk assessment; risk factor; sputum analysis; treatment failure; treatment outcome; treatment response; *tuberculosis/dt [Drug Therapy]; United States; tuberculostatic agent/dt [Drug Therapy].

Link to the Ovid Full Text or citation:

Click here for full text options

73.

Global epidemiology of tuberculosis: Prospects for control.

Lonnroth K., Raviglione M.

Seminars in Respiratory and Critical Care Medicine. 29 (5) (pp 481-491), 2008. Date of Publication: October 2008.

AN: 2008474656

The burden of tuberculosis (TB) is now very slowly decreasing globally. However, the rate of decline is too slow to reach all the epidemiological impact targets set for 2015. The prospects for reaching the TB elimination target set for 2050 are even bleaker. Implementation of the World Health Organization's Stop TB Strategy is currently lagging behind the envisioned scale-up pace, particularly with regard to TB/HIV collaborative activities and management of drug resistant TB. To ensure long-term TB control there is, first, a need to ensure that all components of the Stop TB Strategy are scaled up according to plans, with special attention to improved access for the poor. However, this may not be enough. Recent analyses suggest that the impact of current efforts to reduce TB incidence is less than expected and that improved diagnostic and curative efforts need to be combined with additional preventive efforts. New and more effective vaccines and drugs for preventive treatment would revolutionize TB control. A stronger focus on prevention would also entail more concerted actions to limit the impact of TB risk factors, including HIV/AIDS, smoking, malnutrition, alcoholism, diabetes, crowded living conditions, and indoor air pollution, which may all contribute a considerable proportion of the global TB burden. Copyright © 2008 by Thieme Medical Publishers, Inc.

Institution

(Lonnroth, Raviglione) Stop TB Department, World Health Organization, Geneva, Switzerland (Lonnroth) Stop TB Department, World Health Organization, 20 Avenue Appia, CH-1211 Geneva, Switzerland

Publisher

Thieme Medical Publishers, Inc.

Emtree Heading

acquired immune deficiency syndrome; alcoholism; antibiotic resistance; Chile; China; cohort analysis; Cuba; diabetes mellitus; drug sensitivity; health program; health service; highly active antiretroviral therapy; human; Human immunodeficiency virus; Human immunodeficiency virus infection/dt [Drug Therapy]; incidence; India; indoor air pollution; infection risk; lifestyle; malnutrition; mixed infection; mortality; multidrug resistance; Peru; prevalence; prevention and control; priority journal; public health; review; risk factor; risk reduction; screening test; smoking; socioeconomics; treatment outcome; *tuberculosis/di [Diagnosis]; *tuberculosis/dt [Drug Therapy]; *tuberculosis/ep [Epidemiology]; *tuberculosis/pc [Prevention]; *tuberculosis control; Uruguay; world health organization; antiretrovirus agent/dt [Drug Therapy]; BCG vaccine/dt [Drug Therapy]; cotrimoxazole/dt [Drug Therapy]; isoniazid/dt [Drug Therapy]; tuberculostatic agent/dt [Drug Therapy].

Link to the Ovid Full Text or citation:

Click here for full text options

74.

Chronic heart failure in Japan: Implications of the CHART studies.

Shiba N., Shimokawa H.

Vascular Health and Risk Management. 4 (1) (pp 103-113), 2008. Date of Publication: 2008.

AN: 2008158492

The prognosis of patients with chronic heart failure (CHF) still remains poor, despite the recent advances in medical and surgical treatment. Furthermore, CHF is a major public health problem in most industrialized countries where the elderly population is rapidly increasing. Although the prevalence and mortality of CHF used to be relatively low in Japan, the disorder has been markedly increasing due to the rapid aging of the society and the Westernization of lifestyle that facilitates the development of coronary artery disease. The Chronic Heart Failure Analysis and Registry in the Tohoku District (CHART)-1 study was one of the largest cohorts in Japan. The study has clarified the characteristics and prognosis of Japanese patients with CHF, demonstrating that their prognosis was similarly poor compared with those in Western countries. However, we still need evidence for the prevention and treatment of CHF based on the large cohort studies or randomized treatment trials in the Japanese population. Since the strategy for CHF management is now changing from treatment to prevention, a larger-size prospective cohort, called the CHART-2 study, has been initiated to evaluate the risk factors of CHF in Japan. This review summarizes the current status of CHF studies in Japan and discusses their future perspectives. © 2008 Shiba and Shimokawa, publisher and licensee Dove Medical Press Ltd.

Institution

(Shiba, Shimokawa) Department of Cardiovascular Medicine, Department of Evidence-Based Cardiovascular Medicine, Tohoku University Graduate School of Medicine, 1-1 Seiryo-machi, Aobaku, Sendai City 980-8574, Japan

Publisher

DOVE Medical Press Ltd.

Emtree Heading

aging; cardiovascular disease; cause of death; clinical trial; cost effectiveness analysis; diabetes mellitus; drug dose comparison; drug efficacy; echocardiography; gastroenteritis; health insurance; *heart failure/di [Diagnosis]; *heart failure/dm [Disease Management]; *heart failure/dt [Drug Therapy]; *heart failure/et [Etiology]; *heart failure/pc [Prevention]; *heart failure/su [Surgery]; heart left ventricle ejection fraction; heart left ventricle enddiastolic volume; human; hypercholesterolemia/dt [Drug Therapy]; hypertension/dt [Drug Therapy]; Japan; medical care; optimal drug dose; pneumonia; prognosis; proportional hazards model; quality of life; race difference; review; social status; treatment outcome; tuberculosis; angiotensin 2 receptor antagonist/dt [Drug Therapy]; azosemide/ct [Clinical Trial]; azosemide/cm [Drug Comparison]; azosemide/dt [Drug Therapy]; beta adrenergic receptor blocking agent/dt [Drug Therapy]; candesartan/ct [Clinical Trial]; candesartan/dt [Drug Therapy]; carvedilol/ct [Clinical Trial]; carvedilol/do [Drug Dose]; carvedilol/dt [Drug Therapy]; dipeptidyl carboxypeptidase inhibitor/dt [Drug Therapy]; furosemide/ct [Clinical Trial]; furosemide/cm [Drug Comparison]; furosemide/dt [Drug Therapy]; olmesartan/ct [Clinical Trial]; olmesartan/dt [Drug Therapy]; pitavastatin/ct [Clinical Trial]; pitavastatin/dt [Drug Therapy]; placebo; valsartan/ct [Clinical Trial]; valsartan/dt [Drug Therapy].

Link to the Ovid Full Text or citation:

Click here for full text options

75.

Fluoroquinolones for the treatment of pulmonary tuberculosis.

Moadebi S., Harder C.K., Fitzgerald M.J., Elwood K.R., Marra F.

Drugs. 67 (14) (pp 2077-2099), 2007. Date of Publication: 2007.

AN: 2007487057

Tuberculosis (TB) continues to be a significant problem globally. Treatment includes a multiple drug regimen with isoniazid, rifampicin (rifampin), pyrazinamide and ethambutol. Often, one of these medications needs to be replaced as a result of adverse events or because Mycobacterium tuberculosis develops resistance against one these first-line agents. Fluoroquinolones, particularly the newer ones, possess good in vitro (levofloxacin, gatifloxacin, moxifloxacin) and in vivo (gatifloxacin and moxifloxacin) bactericidal activity against M. tuberculosis, making them attractive agents for the treatment of pulmonary TB.All relevant clinical trials, cohort studies and case reports investigating the clinical efficacy and tolerability of fluoroquinolones when used for the treatment of pulmonary TB were evaluated for this review. Specifically, efficacy and safety in the following indications were investigated: (i) first-line treatment of drug-sensitive pulmonary TB; (ii) first-line treatment for multi-drug resistant (MDR) TB; and (iii) treatment of patients with drug intolerance. Twenty-seven articles met our inclusion criteria; nine articles presented data from randomised, controlled or cohort studies. Seven studies used fluoroquinolones as first-line agents in drug-sensitive TB (1469 patients), 15 studies used fluoroquinolones to treat MDR-TB (1025 patients) and six studies (951 patients) investigated the use of fluoroquinolones in patients intolerant to other TB medications.In patients with susceptible M. tuberculosis strains, substitution with a fluoroquinolone did not have an effect on cure or radiological improvement at 8 weeks or failure at 12 months. Substitution of older fluoroquinolones into a regimen, especially ciprofloxacin, resulted in a higher rate of relapse and a longer time to sputum-culture conversions. The use of fluoroquinolones in patients with MDR-TB is supported by some trials where others show a lack of improvement in efficacy of a regimen.Our review of the literature does not support the use of older fluoroquinolones, especially ciprofloxacin, as substitute agents for drug-sensitive or drug-resistant TB. However, newer fluoroquinolones, such as moxifloxacin, may be a reasonable alternative based on results from one large clinical trial. Fluoroquinolones have an important role as substitute agents for those who are intolerant of first-line TB agents. © 2007 Adis Data Information BV. All rights reserved.

Institution

(Moadebi, Fitzgerald, Elwood, Marra) BC Centre for Disease Control, Vancouver, BC, Canada (Harder, Elwood, Marra) University of British Columbia, Vancouver, BC, Canada

(Fitzgerald) Centre for Clinical Epidemiology and Evaluation, Vancouver, BC, Canada

(Marra) BC Centre for Disease Control, Vaccine and Pharmacy Services, 655 West 12th Avenue, Vancouver, BC V5Z 1L8, Canada

Publisher

Adis International Ltd

Emtree Heading

absence of side effects/si [Side Effect]; acute kidney failure/si [Side Effect]; add on therapy; ageusia/si [Side Effect]; case study; central nervous system disease/si [Side Effect]; clinical study; clinical trial; cohort analysis; disease severity; drug dose comparison; drug effect; drug efficacy; drug hypersensitivity; drug induced headache/si [Side Effect]; drug megadose; drug safety; drug substitution; drug tolerability; drug treatment failure; drug withdrawal; gastrointestinal disease/si [Side Effect]; gastrointestinal symptom/si [Side Effect]; heart arrhythmia/si [Side Effect]; human; insulin dependent diabetes mellitus/si [Side Effect]; low drug dose; *lung tuberculosis/dt [Drug Therapy]; Medline; monotherapy; myalgia/si [Side Effect]; nausea/si [Side Effect]; outcome assessment; photosensitivity/si [Side Effect]; rash/si [Side Effect]; review; single drug dose; sputum culture; sputum smear; statistical analysis; treatment duration; treatment planning; treatment response; unspecified side effect/si [Side Effect]; vertigo/si [Side Effect]; vomiting/si [Side Effect]; aminosalicylic acid/ae [Adverse Drug Reaction]; aminosalicylic acid/ct [Clinical Trial]; aminosalicylic acid/dt [Drug Therapy]; capreomycin/ae [Adverse Drug Reaction]; capreomycin/ct [Clinical Trial]; capreomycin/dt [Drug Therapy]; capreomycin/im [Intramuscular Drug Administration]; *ciprofloxacin/ae [Adverse Drug Reaction]; *ciprofloxacin/ct [Clinical Trial]; *ciprofloxacin/cm [Drug Comparison]; *ciprofloxacin/do [Drug Dose]; *ciprofloxacin/dt [Drug Therapy]; clofazimine/ae [Adverse Drug Reaction]; clofazimine/ct [Clinical Trial]; clofazimine/dt [Drug Therapy]; clofazimine/im [Intramuscular Drug Administration]; cycloserine/ae [Adverse Drug Reaction]; cycloserine/ct [Clinical Trial]; cycloserine/dt [Drug Therapy]; ethambutol/ae [Adverse Drug Reaction]; ethambutol/ct [Clinical Trial]; ethambutol/cm [Drug Comparison]; ethambutol/dt [Drug Therapy]; ethionamide/ae [Adverse Drug Reaction]; ethionamide/ct [Clinical Trial]; ethionamide/cb [Drug Combination]; ethionamide/dt [Drug Therapy]; isoniazid/ae [Adverse Drug Reaction]; isoniazid/ct [Clinical Trial]; isoniazid/cb [Drug Combination]; isoniazid/cm [Drug Comparison]; isoniazid/dt [Drug Therapy]; kanamycin/ae [Adverse Drug Reaction]; kanamycin/ct [Clinical Trial]; kanamycin/cb [Drug Combination]; kanamycin/dt [Drug Therapy]; kanamycin/im [Intramuscular Drug Administration]; levofloxacin/ae [Adverse Drug Reaction]; levofloxacin/ct [Clinical Trial]; levofloxacin/cm [Drug Comparison]; levofloxacin/dt [Drug Therapy]; *moxifloxacin/ae [Adverse Drug Reaction]; *moxifloxacin/ct [Clinical Trial]; *moxifloxacin/cb [Drug Combination]; *moxifloxacin/dt [Drug Therapy]; *ofloxacin/ae [Adverse Drug Reaction]; *ofloxacin/ct [Clinical Trial]; *ofloxacin/cm [Drug Comparison]; *ofloxacin/do [Drug Dose]; *ofloxacin/dt [Drug Therapy]; pefloxacin/ae [Adverse Drug Reaction]; pefloxacin/ct [Clinical Trial]; pefloxacin/cb [Drug Combination]; pefloxacin/dt [Drug Therapy]; protionamide/ae [Adverse Drug Reaction]; protionamide/ct [Clinical Trial]; protionamide/dt [Drug Therapy]; pyrazinamide/ae [Adverse Drug Reaction]; pyrazinamide/ct [Clinical Trial]; pyrazinamide/cb [Drug Combination]; pyrazinamide/cm [Drug Comparison]; pyrazinamide/dt [Drug Therapy]; quinoline derived antiinfective agent/ae [Adverse Drug Reaction]; quinoline derived antiinfective agent/ct [Clinical Trial]; quinoline derived antiinfective agent/dt [Drug Therapy]; rifabutin/ae [Adverse Drug Reaction]; rifabutin/ct [Clinical Trial]; rifabutin/dt [Drug Therapy]; rifampicin/ae [Adverse Drug Reaction]; rifampicin/ct [Clinical Trial]; rifampicin/cb [Drug Combination]; rifampicin/cm [Drug Comparison]; rifampicin/dt [Drug Therapy]; sparfloxacin/ae [Adverse Drug Reaction]; sparfloxacin/ct [Clinical Trial]; sparfloxacin/cb [Drug Combination]; sparfloxacin/dt [Drug Therapy]; streptomycin/ae [Adverse Drug Reaction]; streptomycin/ct [Clinical Trial]; streptomycin/dt [Drug Therapy]; streptomycin/im [Intramuscular Drug Administration]; thioacetazone/ae [Adverse Drug Reaction]; thioacetazone/ct [Clinical Trial]; thioacetazone/cb [Drug Combination]; thioacetazone/dt [Drug Therapy].

Link to the Ovid Full Text or citation:

Click here for full text options

76.

Evaluation of subcutaneous proleukin (interleukin-2) in a randomized international trial (ESPRIT): Geographical and gender differences in the baseline characteristics of participants.

Pett S.L., Wand H., Law M.G., Arduino R., Lopez J.C., Knysz B., Pereira L.C., Pollack S., Reiss P., Tambussi G., Abrams D.I., Cooper D.A., Darbyshire J.H., Duncan W.R., Emery S., Clifford Lane H., Lehrman S., Lundgren J.D., Neaton J.D., Aguilar L., Angel E.B., Aquilia S., Belloso W., Benetucci J., Bittar V., Cahn P., Casiro A., Contarelli J., Corral J., Daciuk L., David D., Ines F., Fridman D., Galache V., Guaragna G., Ivalo S., Laplume H., Lasala M.B., Lattes R., Lasovsky J., Lopardo G., Losso M., Lourtau L., Lupo S., Maranzana A., Marson C., Massera L., del Lujan Sanchez M., Somenzini C., Tocci M., Algar S., Anderson J., Austin D., Baker D., Blavius K., Bloch M., Boyle M., Bradford D., Carrall L., Carr A., Chuah J., Curry M., D'Arcy-Evans C., Dobson P., Doong N., Ferguson W., Finlayson R., French M., Gold J., Habel P., Hoy J., Hudson J., James R., Jeganathan S., Leung J., Lowe K., MacRae K., Mallal S., McMurchie M., Medland N., Mijch A., Miller S., Murray J., Orth D., Patching J., Primrose R., Quan D., Rawlinson M., Ree H., Richardson R., Rogers G., Roney J., Roth N., Shaw D., Soo T.M., Sowden D., Street A., Vale R.

HIV Clinical Trials. 7 (2) (pp 70-85), 2006. Date of Publication: March/April 2006.

AN: 2006345637

Background: ESPRIT, is a phase III, open-label, randomized, international clinical trial evaluating the effects of subcutaneous recombinant interleukin-2 (rIL-2) plus antiretroviral therapy (ART) versus ART alone on HIV-disease progression and death in HIV-1-infected individuals with CD4+ T-cells >300 cells/muL. Objectives: To describe the baseline characteristics of participants randomized to ESPRIT overall and by geographic location. Method: Baseli ne characteristics of randomized participants were summarized by region. Results: 4,150 patients were enrolled in ESPRIT from 254 sites in 25 countries. 41%, 27%, 16%, 11%, and 5% were enrolled in Europe, North America, South America, Asia, and Australia, respectively. The median age was 40 years, 81% were men, and 76%, 11%, and 9% were Caucasian, Asian, and African American or African, respectively. 44% of women enrolled (n = 769) were enrolled in Thailand and Argentina. Overall, 55% and 38% of the cohort acquired HIV through male homosexual and heterosexual contact, respectively. 25% had a prior history of AIDS-defining illness; Pneumocystis jirovecii pneumonia, M. tuberculosis, and esophageal candida were most commonly reported. Median nadir and baseline CD4+ T-cell counts were 199 and 458 cells/muL, respectively. 6% and 13% were hepatitis B or C virus coinfected, respectively. Median duration of antiretroviral therapy (ART) was 4.2 years; the longest median duration was in Australia (5.2 years) and the shortest was in Asia (2.3 years). 17%, 13%, and 69% of participants began ART before 1995, between 1996 and 1997, and from 1998 onward, respectively. 86% used ART from two or more ART classes, with 49% using a protease inhibitor-based regimen and 46% using a nonnucleoside reverse transcriptase inhibitor-based regimen. 78% had plasma HIV RNA below detection (<500 cp/mL). Conclusion: ESPRIT has enrolled a diverse population of HIV-infected individuals including large populations of women and patients of African-American/African and Asian ethnicity often underrepresented in HIV research. As a consequence, the results of the study may have wide global applicability. © 2006 Thomas Land Publishers, Inc.

Institution

(Pett, Wand, Law) National Centre in HIV Epidemiology and Clinical Research, University of New South Wales, 376 Victoria St., Sydney, NSW 2010, Australia (Arduino) University of Texas Medical School of Houston, Houston, TX, United States

(Lopez) Hospital Universitario Gergorio Maranon, Servicio de Micobiologica y Enf. Infecciosas, Madrid, Spain

(Knysz) Medical University of Wroclaw, Department of Infectiou Diseases, Wroclaw University School of Medicine, Wroclaw, Poland

(Pereira) Instituto de Infectologia Emillio Ribas, Sao Paulo, Brazil

(Pollack) Rambam Medical Center, Immunology Allergy and AIDS Institute, B. Rappaport Faculty of Medicine, Technion, Haifa, Israel

(Reiss) Academish Medisch Centrum - Amsterdam, Amsterdam, Netherlands

(Tambussi) Ospedale San Raffaele, Divisione Malattie Infettive - Centro San Luigi, Milano, Italy

(Aguilar, Angel, Aquilia, Belloso, Benetucci, Bittar, Cahn, Casiro, Contarelli, Corral, Daciuk, David, Ines, Fridman, Galache, Guaragna, Ivalo, Laplume, Lasala, Lattes, Lasovsky, Lopardo, Losso, Lourtau, Lupo, Maranzana, Marson, Massera, del Lujan Sanchez, Somenzini, Tocci) Argentina National Coordinating Center, Argentina

(Algar, Anderson, Austin, Baker, Blavius, Bloch, Boyle, Bradford, Carrall, Carr, Chuah, Curry, D'Arcy-Evans, Dobson, Doong, Ferguson, Finlayson, French, Gold, Habel, Hoy, Hudson, James, Jeganathan, Leung, Lowe, MacRae, Mallal, McMurchie, Medland, Mijch, Miller, Murray, Orth, Patching, Primrose, Quan, Rawlinson, Ree, Richardson, Rogers, Roney, Roth, Shaw, Soo, Sowden, Street, Street, Vale) Australia National Trial Coordinating Center, Australia

Publisher

Thomas Land Publishers Inc.

Emtree Heading

adult; African American; AIDS related complex; anamnesis; Argentina; article; Asia; Asian; Australia; bacterial pneumonia; candidiasis; CD4 lymphocyte count; clinical trial; cohort analysis; controlled clinical trial; controlled study; death; diabetes mellitus/dt [Drug Therapy]; diabetes mellitus/si [Side Effect]; disease course; disease duration; disease transmission; drug efficacy; drug safety; dyslipidemia/dt [Drug Therapy]; dyslipidemia/si [Side Effect]; ethnology; Europe; female; geographic distribution; heart infarction/si [Side Effect]; hepatitis B/dt [Drug Therapy]; hepatitis C/dt [Drug Therapy]; herpes simplex/dt [Drug Therapy]; herpes simplex/pc [Prevention]; herpes simplex/si [Side Effect]; heterosexuality; HIV associated lipodystrophy/si [Side Effect]; human; Human immunodeficiency virus; *Human immunodeficiency virus infection/dt [Drug Therapy]; hypertension/dt [Drug Therapy]; hypertension/si [Side Effect]; intravenous drug abuse; lipid storage; major clinical study; male; male homosexual; methodology; Negro; nonhuman; North America; open study; phase 3 clinical trial; priority journal; randomized controlled trial; sex difference; side effect/si [Side Effect]; South America; stroke/si [Side Effect]; superinfection; Thailand; time series analysis; treatment duration; treatment outcome; tuberculosis; virus load; antihypertensive agent/dt [Drug Therapy]; antilipemic agent/dt [Drug Therapy]; *antiretrovirus agent/ae [Adverse Drug Reaction]; *antiretrovirus agent/ct [Clinical Trial]; *antiretrovirus agent/cb [Drug Combination]; *antiretrovirus agent/cm [Drug Comparison]; *antiretrovirus agent/dt [Drug Therapy]; hepatitis B surface antigen/ec [Endogenous Compound]; hepatitis B vaccine/dt [Drug Therapy]; herpes vaccine/dt [Drug Therapy]; insulin/dt [Drug Therapy]; oral antidiabetic agent/dt [Drug Therapy]; oral antidiabetic agent/po [Oral Drug Administration]; proteinase inhibitor/ae [Adverse Drug Reaction]; proteinase inhibitor/ct [Clinical Trial]; proteinase inhibitor/cb [Drug Combination]; proteinase inhibitor/cm [Drug Comparison]; proteinase inhibitor/dt [Drug Therapy]; *recombinant interleukin 2/ad [Drug Administration]; *recombinant interleukin 2/cb [Drug Combination]; *recombinant interleukin 2/cm [Drug Comparison]; *recombinant interleukin 2/dt [Drug Therapy]; *recombinant interleukin 2/sc [Subcutaneous Drug Administration]; ritonavir/ae [Adverse Drug Reaction]; ritonavir/ct [Clinical Trial]; ritonavir/cb [Drug Combination]; ritonavir/cm [Drug Comparison]; ritonavir/dt [Drug Therapy]; RNA directed DNA polymerase inhibitor/ae [Adverse Drug Reaction]; RNA directed DNA polymerase inhibitor/ct [Clinical Trial]; RNA directed DNA polymerase inhibitor/cb [Drug Combination]; RNA directed DNA polymerase inhibitor/cm [Drug Comparison]; RNA directed DNA polymerase inhibitor/dt [Drug Therapy]; virus RNA/ec [Endogenous Compound].

Drug Trade Names and Manufacturers

proleukin

Link to the Ovid Full Text or citation:

Click here for full text options

77.

Cohort analysis for treatment outcomes TB pulmonary cases bacteriologically confirmed in Poland. <Skutecznosc leczenia przeciwpratkowego chorych na gruzlice pluc potwierdzona bakteriologicznie w Polsce.>

Szczuka I.

Pneumonologia i Alergologia Polska. 73 (3) (pp 245-253), 2005. Date of Publication: 2005.

AN: 2006186978

In Poland since 1986 system of treatment outcomes monitoring (TOM) of all confirmed pulmonary TB (PTB) cases has been introduced. This system is based on the Central Tuberculosis Register TB detected and registered in given year from the whole network tuberculosis and lung diseases dispensaries in the country -/ 400 disp. in 2004 yr/ The aim of the study was to asses treatment results of PTB confirmed cases registered in 2003 and in the period 2001-2003. In 2003 5558 cases were registered, The treatment outcomes were assessed according to WHO /Euro TB - categories after 12 months after start of treatment as PTB: cured - 67,8%; completed - 10,4% (success ratio - 77,8%); failed - 1,5%, died - 5,1% (from TB - 2,1%); defaulted - 7,6%, still on treatment - 0,6%; transferred - 1,9% and unknown - 5,5% all registered PTB cases. In period 2001-2003 proportion patients with success of treatment was stable. In 2003 proportion of patients with success of treatment was significantly higher in a groups of new cases - 83,7% than in patients with relapses - 73,9%; among women - 85,4% than in men - 80,9%. Among PTB cases smear positive success ratio - 81,3% was lower than among PTB cases culture positive - 84,0%. Among patients with diabetes and alcohol abuse the success of treatment was significantly lower than more patients without these diseases /habit. Alcoholics three times more frequently defaulted treatment than patients without this habit. Conclusion: the improvement of treatment outcomes is impossible without generalize directly observed treatment in all patients in continuation phase of treatment.

Institution

(Szczuka) Z Zakladu Epidemiologii I Organizacji Walki Z Gruzlica Instytutu Gruzlicy I Chorob Puc, p.o. kierownik dr med. I. Szczuka

Publisher

Instytut Gruzlicy i Chorob Pluc

Emtree Heading

alcohol abuse; article; cohort analysis; controlled study; diabetes mellitus; disease registry; female; human; *lung tuberculosis/ep [Epidemiology]; major clinical study; male; mortality; Poland; relapse; treatment outcome; world health organization.

Link to the Ovid Full Text or citation:

Click here for full text options

78.

ANCA-associated renal vasculitis at the end of the twentieth century - A disease of older patients.

Harper L., Savage C.O.

Rheumatology. 44 (4) (pp 495-501), 2005. Date of Publication: April 2005.

AN: 2005480793

Objective. Antineutrophil cytoplasmic antibody (ANCA)-associated vasculitides are increasingly recognized in older patients. However, it is unknown whether disease presentation and response to treatment differs from younger patients. We aimed to examine the presentation, response to treatment and outcome of patients over 65 yr of age compared with a younger cohort. Methods. This retrospective, single centre, sequential cohort study reports presenting features and outcome of 233 consecutive new patients with ANCA-associated vasculitis between 1990 and 2000. Results. The median age of all patients was 65 yr (range 16-90 yr). Older patients (>65 yr) presented with more severe renal involvement at presentation (P<0.001). Older patients were as likely to respond to treatment or undergo relapse as the younger patients. Older patients receiving immunosuppression had an increased risk of infection (P = 0.0027). Survival was worse in the older group (P = 0.016) and death occurred early. Mortality was associated with poor renal function (creatinine >400 mumol/l), infection and low serum albumin. Leucopenia was associated with severe renal impairment (P = 0.0048) and increased risk of infection (P = 0.0006). Multivariate analysis determined that serum creatinine >400 mumol/l and age were independent risk factors for poor prognosis. Conclusion. ANCA-associated vasculitis occurs frequently in older patients and physicians should maintain a high index of suspicion. Older patients have a poorer prognosis due to more severe renal involvement and increased sensitivity to adverse effects of treatment. This study highlights the importance of careful dosing of cyclophosphamide: In those aged over 65 yr a 25% dose reduction is safe and reduces the risk of leucopenia. This study further highlights the importance of renal function on prognosis and the need for less toxic treatment regimens. © British Society for Rheumatology 2005; all rights reserved.

Institution

(Harper, Savage) Division of Immunology and Infection, The Medical School, University of Birmingham, Edgbaston, Birmingham B15 2TT, United Kingdom

Publisher

Oxford University Press

Emtree Heading

adolescent; adult; age distribution; aged; *ANCA associated vasculitis/di [Diagnosis]; *ANCA associated vasculitis/dt [Drug Therapy]; article; bladder cancer/si [Side Effect]; cerebrovascular disease/si [Side Effect]; clinical feature; clinical trial; cohort analysis; controlled clinical trial; controlled study; creatinine blood level; death; deep vein thrombosis/si [Side Effect]; diabetes mellitus/si [Side Effect]; disease association; disease severity; drug dose reduction; drug dose regimen; drug fatality/si [Side Effect]; female; fracture/si [Side Effect]; health care facility; heart infarction/si [Side Effect]; human; immunosuppressive treatment; infection/dt [Drug Therapy]; infection/pc [Prevention]; infection/si [Side Effect]; *kidney disease/di [Diagnosis]; *kidney disease/dt [Drug Therapy]; kidney dysfunction/si [Side Effect]; leukopenia/si [Side Effect]; lower respiratory tract infection/si [Side Effect]; lung embolism/si [Side Effect]; major clinical study; male; malignant neoplastic disease/si [Side Effect]; mortality; multivariate analysis; neutropenia/si [Side Effect]; physician; pneumonia/si [Side Effect]; priority journal; prognosis; relapse; retrospective study; risk assessment; sensitivity analysis; sepsis/si [Side Effect]; skin cancer/si [Side Effect]; survival rate; treatment outcome; tuberculosis/si [Side Effect]; azathioprine/dt [Drug Therapy]; corticosteroid/ae [Adverse Drug Reaction]; corticosteroid/dt [Drug Therapy]; cotrimoxazole/dt [Drug Therapy]; creatinine/ec [Endogenous Compound]; cyclophosphamide/ae [Adverse Drug Reaction]; cyclophosphamide/ct [Clinical Trial]; cyclophosphamide/do [Drug Dose]; cyclophosphamide/dt [Drug Therapy]; immunosuppressive agent/ae [Adverse Drug Reaction]; immunosuppressive agent/ct [Clinical Trial]; immunosuppressive agent/do [Drug Dose]; immunosuppressive agent/dt [Drug Therapy]; methylprednisolone/dt [Drug Therapy]; prednisolone/dt [Drug Therapy]; serum albumin/ec [Endogenous Compound].

Drug Trade Names and Manufacturers

septrin

Link to the Ovid Full Text or citation:

Click here for full text options

79.

Evaluation of tuberculosis treatment results in Italy, report 1998.

Centis R., Ianni A., Migliori G.B., Casali L., Agati G., Aiolfi S., Altieri A., Ambrosetti M., Arossa W., Bagnasco G., Bazzerla G., Berra A., Besozzi G., Bottrighi P., Bugiani M., Calabro S., Castiglioni G., Cavallero M., Cicchitto G., Codecasa L.R., Colorizio V., Confalonieri M., D'Ambrosio L., D'Ambrosio V., DellaTommasa S., Di Pisa G., Ermeti M., Faccini E., Falcone F., Farinelli G., Fatigante R., Farris B., Fatur Volante T., Felisatti G., Ferranti P., Fiorentini F., Foschi C., Garzone A., Giorgio V., Gozzellino F., Jacopino L., Lauriello G., Le Donne R., Longi R., Macor G., Mancini D., Marchesani M.F., Marcias S., Mariano V., Mennea G., Monaco A., Montesano G., Moretti G., Napolitano G., Nardini S., Nutini S., Orani G., Orsi A., Parpanesi M., Pasi A., Penza O., Perboni A., Petrozzi L., Pretto P., Rossi S., Sabato E., Saini L., Schiavi L., Tazza R., Trucco G., Vertuccio C., Viviani U., Volante M., Vinciguerra P., Viola S., Zaccara F., Angarano G., Barelle L., Bertoli G., Bisetti A., Bistoni F., Buono L., Carbonara S., Carosi G.P., Cascina A., Cassone A., Costa D., Crepaldi P., De Santis A., Diamare F., Fabbro L., Fadda G., Farris A.G., Fattorini L., Ferrara G., Gamba S., Iona E., Losi M.

Monaldi Archives for Chest Disease. 55 (4) (pp 293-298), 2000. Date of Publication: 2000.

AN: 2000369479

In Italy no national data on tuberculosis (TB) treatment results were available. In 1995 the AIPO (Italian Association of Hospital Pneumologists) TB Study Group, in collaboration with the Istituto Superiore di Sanita (technical branch of the Ministry of Health), started a prospective monitoring activity based on World Health Organization (WHO) and International Union against Tuberculosis and Lung Disease (IUATLD) recommendations. Data were collected from a representative network of TB units nationwide, managing a significant proportion of all TB cases notified in Italy each year. The aim of this study was to analyse the case findings and treatment results for the year 1998. The number of TB cases reported was 1162 (61.7% males; 28.2% immigrants), 888 (76.6%) being new cases. Of these cases, 1,001 (86.4%) were pulmonary, 132 (11.4%) extrapulmonary and 26 (2.2%) both pulmonary and extrapulmonary. The main risk factors for TB were a history of recent contact, diabetes and alcohol abuse among native Italians and human immunodeficiency virus-seropositive status and history of recent contact among immigrants. The majority of immigrants were from Africa and South America, and had been in Italy >24 months before diagnosis of TB. Forty-seven per cent (552 of 1,162) of patients had a positive direct sputum smear examination for alcohol acid-fast bacilli; 30% were resistant to any drug (combined monoresistance (the sum of primary and acquired drug resistance) to rifampicin 2.3%; combined multidrug resistance 11.4%). In 97% of cases, the duration of treatment was <12 months. The overall success rate (cured plus treatment completed excluding transferred out from the denominator) was 83%. A significantly higher percentage of deaths was found in native Italians (being age-related; p<0.001), whereas immigrants had a higher default rate.

Institution

(Centis, Ianni, Migliori, Casali, Agati, Aiolfi, Altieri, Ambrosetti, Angarano, Arossa, Bagnasco, Bazzerla, Berra, Besozzi, Bottrighi, Bugiani, Calabro, Castiglioni, Cavallero, Cicchitto, Codecasa, Colorizio, Confalonieri, D'Ambrosio, D'Ambrosio, DellaTommasa, Di Pisa, Ermeti, Faccini, Falcone, Farinelli, Fatigante, Farris, Fatur Volante, Felisatti, Ferranti, Fiorentini, Foschi, Garzone, Giorgio, Gozzellino, Jacopino, Lauriello, Le Donne, Longi, Macor, Mancini, Marchesani, Marcias, Mariano, Mennea, Monaco, Montesano, Moretti, Napolitano, Nardini, Nutini, Orani, Orsi, Parpanesi, Pasi, Penza, Perboni, Petrozzi, Pretto, Rossi, Sabato, Saini, Schiavi, Tazza, Trucco, Vertuccio, Viviani, Volante, Vinciguerra, Viola, Zaccara, Angarano, Barelle, Bertoli, Bisetti, Bistoni, Buono, Carbonara, Carosi, Cascina, Cassone, Costa, Crepaldi, De Santis, Diamare, Fabbro, Fadda, Farris, Fattorini, Ferrara, Gamba, Iona, Losi) Fondazione Salvatore Maugeri, Clinica del Lavoro e Riabilitazione, Care and Research Institute, via Roncaccio 16, 21049 Tradate (VA), Italy

Publisher

PI-ME Tipografia Editrice S.r.l.

Emtree Heading

adolescent; adult; aged; alcohol abuse; article; child; cohort analysis; diabetes mellitus; female; human; Human immunodeficiency virus infection; immigrant; infant; Italy; *lung tuberculosis/dr [Drug Resistance]; *lung tuberculosis/dt [Drug Therapy]; *lung tuberculosis/ep [Epidemiology]; *lung tuberculosis/et [Etiology]; major clinical study; male; mortality; multidrug resistance; newborn; risk factor; sputum smear; treatment outcome; world health organization; alcohol; isoniazid/dt [Drug Therapy]; rifampicin/dt [Drug Therapy]; streptomycin/dt [Drug Therapy]; *tuberculostatic agent/dt [Drug Therapy].

Link to the Ovid Full Text or citation:

Click here for full text options

80.

Long-term peritoneal dialysis outcome in a single center.

Cancarini G.C., Sandrini M., Vizzardi V., Scaini P., Mombelloni S., Pola A., Maiorca R.

Peritoneal Dialysis International. 20 (SUPPL. 2) (pp S121-S126), 2000. Date of Publication: 2000.

AN: 2000221310

Institution

(Cancarini, Sandrini, Vizzardi, Scaini, Mombelloni, Pola, Maiorca) Department of Nephrology, University of Brescia, Division of Nephrology, Brescia, Italy (Cancarini) Divisione di Nefrologia, Azienda Ospedaliera, Spedali Civili di Brescia, Piazza Ospedale 1, Brescia 25123, Italy

Publisher

Multimed Inc.

Emtree Heading

adult; age; aged; article; biocompatibility; cerebrovascular disease; clinical trial; cohort analysis; comorbidity; continuous ambulatory peritoneal dialysis; female; hemodialysis; host resistance; human; hypertension; insulin dependent diabetes mellitus; ischemic heart disease; kidney failure/th [Therapy]; kidney transplantation; major clinical study; male; non insulin dependent diabetes mellitus; peripheral vascular disease; *peritoneal dialysis; priority journal; respiratory tract infection; risk factor; survival rate; treatment indication; treatment outcome; tuberculosis; peritoneal dialysis fluid.

Link to the Ovid Full Text or citation:

Click here for full text options

81.

The Italian AIPO study on tuberculosis treatment results, report 1996.

Ambrosetti M., Besozzi G., Farris B., Nutini S., Saini L., Casali L., Pretto P., Orani G., Calabro S., Migliori G.B.

Monaldi Archives for Chest Disease. 54 (3) (pp 237-241), 1999. Date of Publication: 1999.

AN: 1999273056

In Italy, no national data on tuberculosis (TB) treatment results were available. In 1995, the AIPO (Italian Association of Hospital Pneumologists) TB Study Group, in collaboration with the Istituto Superiore di Sanita (technical branch of the Ministry of Health), started a prospective monitoring activity based on World Health Organization (WHO) and International Union against Tuberculosis and Lung Disease (IUATLD) recommendations. Data were collected from a nationwide network of 41 TB units, managing a significant proportion of all the TB cases notified in Italy each year. The aim of this study was to analyse the case findings and treatment results for the year 1996. Eight hundred and thirty eight TB cases were reported (56% males; 26% immigrants), 708 (84%) being new cases. Of these cases, 565 (67%) were pulmonary, 235 (28%) extrapulmonary and 38 (5%) both pulmonary and extrapulmonary. The main risk factors for TB were a history of recent contact and diabetes among native Italians and recent contact and human immunodeficiency virus-seropositive status among immigrants. The majority of immigrants were from Africa and Asia, and had been in Italy > 24 months before diagnosis of TB. Thirty-six per cent of patients had a positive direct sputum smear examination for alcohol acid-fast bacilli; 37% were resistant to any drug (monoresistance to isoniazid 4.4%; multidrug resistance 10.4%). In 96% of cases, the duration of treatment was < 12 months. The overall success rate (cured plus treatment completed) was 84.1%. A significantly higher percentage of deaths was found in native Italians (being age-related), whereas immigrant's had a higher default rate.

Institution

(Ambrosetti, Migliori) Fondazione Salvatore Maugeri, Clin. del Lavoro e della Riabilit., Care and Research Institute, Tradate, Italy (Ambrosetti) Dept. of Clin. and Biol. Sciences, Faculty of Medicine, University of Insubria, Italy

(Besozzi) Ist. Villa Marelli per Patol. Torace, Milan, Italy

(Farris, Orani) Cagliari Dispensary, Italy

(Nutini) Dept. of Pulmonary Diseases, Careggi Hospital, Florence, Italy

(Saini) Dept. of Pulmonary Diseases, Novara Hospital, Italy

(Casali) Dept. of Pulmonary Diseases, Pavia University, Italy

(Pretto) Bolzano Dispensary, Italy

(Calabro) Feltre Hospital, Italy

(Migliori) Natl. AIPO Tuberculosis Study Group, Fondazione Salvatore Maugeri, Care and Research Institute, via Roncaccio 16, 21049, Tradate (VA), Italy

Publisher

PI-ME Tipografia Editrice S.r.l.

Emtree Heading

acid fast bacterium; adolescent; adult; Africa; age; aged; alcoholism; article; Asia; child; cohort analysis; diabetes mellitus; drug dependence; female; human; Human immunodeficiency virus infection; immigrant; infant; Italy; lung tuberculosis/dr [Drug Resistance]; lung tuberculosis/dt [Drug Therapy]; major clinical study; male; mortality; multidrug resistance; patient compliance; risk factor; sputum cytodiagnosis; steroid therapy; *treatment outcome; *tuberculosis/dr [Drug Resistance]; *tuberculosis/dt [Drug Therapy]; isoniazid/dt [Drug Therapy]; pyrazinamide/dt [Drug Therapy]; rifampicin/dt [Drug Therapy]; streptomycin/dt [Drug Therapy].

Link to the Ovid Full Text or citation:

Click here for full text options
