# Supplementary material for: Effect of glycemic control and type of diabetes treatment on TB treatment outcomes among people with TB-diabetes: A systematic review (updated August 2024)
Source: PLoS One. 2025 Jul 18;20(7):e0328619. doi: 10.1371/journal.pone.0328619 (PMC12273911; doi:10.1371/journal.pone.0328619)
Supplement: S1 Appendix — (ZIP) [file pone.0328619.s004.zip › S1 appendix_old/EMBASE/update Sept 2016 to current 24_04_2017/128_with RCT search results no 27_2016 to current.doc]

Database: EMBASE <1947-Present>

Search Strategy:

--------------------------------------------------------------------------------

1	tuberculosis.mp. or exp tuberculosis/ (291433)

2	exp treatment outcome/ or treatment outcome*.mp. (1289437)

3	diabetes mellitus.mp. or exp diabetes mellitus/ (880018)

4	diabetes mellitus/ (599265)

5	DM.mp. (234038)

6	diabetes.mp. (891272)

7	mellitus.mp. (827628)

8	3 or 4 or 5 or 6 or 7 (1125394)

9	1 and 2 and 8 (1560)

10	cohort studies.mp. or cohort analysis/ (334934)

11	exp risk factor/ or cohort.mp. or exp risk/ (2843815)

12	10 or 11 (2843815)

13	9 and 10 (161)

14	9 and 12 (652)

15	limit 14 to human (642)

16	limit 13 to human (160)

17	trial*.mp. or exp controlled clinical trial/ or exp "clinical trial (topic)"/ or exp "randomized controlled trial (topic)"/ or exp "controlled clinical trial (topic)"/ (2044040)

18	random.ab. (253017)

19	randomized controlled trial.pt. (0)

20	controlled clinical trial.pt. (0)

21	randomized.ab. (540645)

22	placebo.ab. (252710)

23	clinical trials as topic.sh. (2)

24	trial.ti. (233523)

25	17 or 18 or 19 or 20 or 21 or 22 or 23 or 24 (2406494)

26	9 and 25 (443)

27	limit 26 to human (442)

28	limit 16 to yr="2016 - 2017" (19)

29	limit 15 to yr="2016 -Current" (61)

30	limit 16 to yr="2016 -Current" (19)

31	limit 27 to yr="2016 -Current" (50)

***************************

1.

It takes more than a sensitive test to find more tuberculosis cases.

Trajman A., Cobelens F.

The Lancet Infectious Diseases. 17 (4) (pp 357-358), 2017. Date of Publication: 01 Apr 2017.

AN: 614011778

Institution

(Trajman) Social Medicine Institute, State University of Rio de Janeiro, Brazil (Trajman) McGill University, Montreal, QC, Canada

(Cobelens) Amsterdam Institute for Global Health and development, Academic Medical Center, Amsterdam, Netherlands

(Cobelens) KNCV Tuberculosis Foundation, The Hague, Netherlands

Publisher

Lancet Publishing Group (E-mail: cususerv@lancet.com)

Link to the Ovid Full Text or citation:

[Click here for full text options](http://ovidsp.ovid.com/ovidweb.cgi?T=JS&CSC=Y&NEWS=N&PAGE=fulltext&D=emed18b&AN=614011778)

Link to the External Link Resolver:

[SFX](http://whel-primo.hosted.exlibrisgroup.com/openurl/44WHELF_CAR/44WHELF_CAR_services_page?sid=OVID:embase&id=pmid:&id=doi:10.1016%2FS1473-3099%252816%252930458-3&issn=1473-3099&isbn=&volume=17&issue=4&spage=357&pages=357-358&date=2017&title=The+Lancet+Infectious+Diseases&atitle=It+takes+more+than+a+sensitive+test+to+find+more+tuberculosis+cases&aulast=Trajman&pid=<author>Trajman+A.%3BCobelens+F.<%2Fauthor><AN>614011778<%2FAN><DT>Journal%3A+Note<%2FDT>)

2.

Efficacy and safety of sirukumab in patients with active rheumatoid arthritis refractory to anti-TNF therapy (SIRROUND-T): a randomised, double-blind, placebo-controlled, parallel-group, multinational, phase 3 study.

Aletaha D., Bingham C.O., Tanaka Y., Agarwal P., Kurrasch R., Tak P.P., Popik S.

The Lancet. 389 (10075) (pp 1206-1217), 2017. Date of Publication: 25 Mar 2017.

AN: 614463178

Background Sirukumab, a human monoclonal antibody that selectively binds to the interleukin-6 cytokine with high affinity, is under development for the treatment of rheumatoid arthritis and other diseases. We aimed to assess the efficacy and safety of sirukumab for rheumatoid arthritis in a phase 3 study (SIRROUND-T). Methods We did a randomised, double-blind, placebo-controlled, parallel-group, multicentre study at 183 hospitals and private rheumatology clinics in 20 countries (Argentina, Australia, Austria, Belgium, Canada, France, Germany, Italy, Japan, Lithuania, Mexico, Netherlands, Poland, Portugal, Russia, South Korea, Spain, Taiwan, UK, and USA). Eligible participants were patients with active rheumatoid arthritis aged at least 18 years, with four or more of 68 tender joints and four or more of 66 swollen joints, who were refractory or intolerant to previous treatment with at least one anti-TNF drug. We randomly assigned patients (1:1:1) via a central interactive voice or web response system to either placebo every 2 weeks, 50 mg sirukumab every 4 weeks, or 100 mg sirukumab every 2 weeks, all given for 52 weeks or less. We allowed participants to continue using any concomitant disease-modifying antirheumatic drugs (DMARDs). We based the randomisation on a computer-generated, permuted-block schedule stratified by use of methotrexate at baseline (0, >0 to <12.5 mg/week, or >12.5 mg/week). Masking was achieved with the use of multipart labels on the study drug containers which contained directions for use and other information, but not the drug's identity. Treatments were administered by subcutaneous injection; patients assigned to 50 mg sirukumab given every 4 weeks also received a placebo injection every 2 weeks to maintain masking. At week 18, placebo-treated patients meeting early escape criteria (<20% improvement in swollen and tender joint counts) were randomly reassigned to either 50 mg or 100 mg of sirukumab. All remaining placebo-treated patients were subsequently randomly reassigned at week 24 to either sirukumab dose (crossover). The primary outcome was the proportion of patients who achieved a response of at least 20% improvement at week 16 according to American College of Rheumatology criteria (ACR20) in the intention-to-treat population (all randomly assigned participants). Safety analyses included all participants who received at least one dose (partial or complete) of study drug. This study is registered at EudraCT (number: 2010-022243-38) and ClinicalTrials.gov (number: NCT01606761). Findings Between July 25, 2012, and Jan 12, 2016, we randomly assigned 878 patients to treatment: 294 to placebo, 292 to 50 mg sirukumab every 4 weeks, and 292 to 100 mg sirukumab every 2 weeks. 523 (60%) of 878 patients had previously received two or more biological treatments including non-TNF drugs, and 166 (19%) of 878 were not taking a DMARD at baseline. The proportions of patients who achieved an ACR20 response at week 16 were 117 (40%) of 292 with 50 mg sirukumab every 4 weeks, and 132 (45%) of 292 with 100 mg sirukumab every 2 weeks versus 71 (24%) of 294 with placebo; differences compared with placebo were 0.16 (95% CI 0.09-0.23) for 50 mg sirukumab every 4 weeks and 0.21 (0.14-0.29) for 100 mg sirukumab every 2 weeks (both p<0.0001). Adverse event incidences in the 24-week placebo-controlled period were similar across groups (at least one event occurred for 182 patients assigned to placebo [62%, including early escape patients switched to sirukumab at week 18] of 294; 194 [66%] of 292 with 50 mg sirukumab every 4 weeks; and 207 [71%] of 292 with 100 mg sirukumab every 2 weeks). The most common adverse events in this period were injection-site erythema (four [1%] with placebo, 22 [8%] with 50 mg sirukumab every 4 weeks, and 41 [14%] with 100 mg sirukumab every 2 weeks). At week 52, of all patients receiving sirukumab including those reassigned from placebo, the most common adverse events were again injection-site erythema (33 [8%] of 416 with 50 mg sirukumab every 4 weeks and 66 [16%] of 418 with 100 mg sirukumab every 2 weeks). Interpretation In patients with active rheumatoid arthritis who were refractory or intolerant to anti-TNF drugs and other biological treatments, both dosing regimens of sirukumab were well tolerated and significantly improved signs and symptoms of the disease, compared with placebo, in this difficult-to-treat population. Funding Janssen Research & Development, LLC, and GlaxoSmithKline. Copyright © 2017 Elsevier Ltd

Institution

(Aletaha) Division of Rheumatology, Department of Internal Medicine 3, Medical University of Vienna, Vienna, Austria (Bingham) Division of Rheumatology, Johns Hopkins University, Baltimore, MD, United States

(Tanaka) Department of Internal Medicine, University of Occupational and Environmental Health, Kitakyushu, Japan

(Agarwal, Popik) Janssen Research & Development, LLC, Spring House, PA, United States

(Kurrasch) GlaxoSmithKline, Collegeville, PA, United States

(Tak) GlaxoSmithKline, Stevenage, Hertfordshire, United Kingdom

Publisher

Lancet Publishing Group (E-mail: cususerv@lancet.com)

Link to the Ovid Full Text or citation:

[Click here for full text options](http://ovidsp.ovid.com/ovidweb.cgi?T=JS&CSC=Y&NEWS=N&PAGE=fulltext&D=emed18b&AN=614463178)

Link to the External Link Resolver:

[SFX](http://whel-primo.hosted.exlibrisgroup.com/openurl/44WHELF_CAR/44WHELF_CAR_services_page?sid=OVID:embase&id=pmid:&id=doi:10.1016%2FS0140-6736%252817%252930401-4&issn=0140-6736&isbn=&volume=389&issue=10075&spage=1206&pages=1206-1217&date=2017&title=The+Lancet&atitle=Efficacy+and+safety+of+sirukumab+in+patients+with+active+rheumatoid+arthritis+refractory+to+anti-TNF+therapy+(SIRROUND-T)%3A+a+randomised%2C+double-blind%2C+placebo-controlled%2C+parallel-group%2C+multinational%2C+phase+3+study&aulast=Aletaha&pid=<author>Aletaha+D.%3BBingham+C.O.%3BTanaka+Y.%3BAgarwal+P.%3BKurrasch+R.%3BTak+P.P.%3BPopik+S.<%2Fauthor><AN>614463178<%2FAN><DT>Journal%3A+Article<%2FDT>)

3.

Contemporary management of pericardial effusion: practical aspects for clinical practice.

Imazio M., Gaido L., Battaglia A., Gaita F.

Postgraduate Medicine. 129 (2) (pp 178-186), 2017. Date of Publication: 17 Feb 2017.

AN: 614367614

A pericardial effusion (PE) is a relatively common finding in clinical practice. It may be either isolated or associated with pericarditis with or without an underlying disease. The aetiology is varied and may be either infectious (especially tuberculosis as the most common cause in developing countries) or non-infectious (cancer, systemic inflammatory diseases). The management is essentially guided by the hemodynamic effect (presence or absence of cardiac tamponade), the presence of concomitant pericarditis or underlying disease, and its size and duration. The present paper reviews the current knowledge on the aetiology, classification, diagnosis, management, therapy, and prognosis of PE in clinical practice. Copyright © 2017 Informa UK Limited, trading as Taylor & Francis Group.

PMID

28135875 [<http://www.ncbi.nlm.nih.gov/pubmed/?term=28135875>]

Institution

(Imazio, Gaido, Battaglia, Gaita) Department of Medical Sciences, University Cardiology, AOU Citta della Salute e della Scienza di Torino, and University of Torino, Torino, Italy

Publisher

Taylor and Francis Inc. (325 Chestnut St, Suite 800, Philadelphia PA 19106, United States)

Link to the Ovid Full Text or citation:

[Click here for full text options](http://ovidsp.ovid.com/ovidweb.cgi?T=JS&CSC=Y&NEWS=N&PAGE=fulltext&D=emed18b&AN=614367614)

Link to the External Link Resolver:

[SFX](http://whel-primo.hosted.exlibrisgroup.com/openurl/44WHELF_CAR/44WHELF_CAR_services_page?sid=OVID:embase&id=pmid:28135875&id=doi:10.1080%2F00325481.2017.1285676&issn=0032-5481&isbn=&volume=129&issue=2&spage=178&pages=178-186&date=2017&title=Postgraduate+Medicine&atitle=Contemporary+management+of+pericardial+effusion%3A+practical+aspects+for+clinical+practice&aulast=Imazio&pid=<author>Imazio+M.%3BGaido+L.%3BBattaglia+A.%3BGaita+F.<%2Fauthor><AN>614367614<%2FAN><DT>Journal%3A+Review<%2FDT>)

4.

The nature and duration of symptoms and time to starting treatment comparing older with younger pulmonary tuberculosis patients.

Abbara A., Hardman E., Collin S.M., Kon O.M., Mahomed Z., Sullivan J.A.L., Buell K., Hansel T., Corrah T., Davidson R.N.

Thorax. Conference: British Thoracic Society Winter Meeting 2016. United Kingdom. Conference Start: 20161207. Conference End: 20161209. 71 (pp A51-A52), 2016. Date of Publication: December 2016.

AN: 615030876

Introduction and objectives In 2013, Public Health England (PHE) reported that 39% of pulmonary tuberculosis (TB) patients >65 years old started treatment >4 months after symptom onset compared to 25% of patients aged 15-44 years. A longer symptomatic period, particular if smear positive, may result in increased transmission and poorer treatment outcomes. We investigated age-related differences in symptom duration and time to starting TB treatment in a large UK cohort of TB patients. Methods The cohort comprised patients on the London TB register (LTBR) who were treated at Northwick Park Hospital between 2002-2015. Patients aged >65 years were compared with a random sample of patients aged 18-40 years, with respect to symptoms, symptom duration at presenting to secondary care and time to starting treatment after presenting to secondary care. Results 357 patients over 65 and 517 younger patients were identified. Demographics for the total number of patients are included in Table 1. Data were available for 489 of the younger group and 73 of the older group. 26 of the >65s (35.6%) and 9 (1.8%) of the 65s were diabetic. Conclusions This study has identified that a potential cause for a delay in diagnosis in the elderly may be related to the decreased frequency of 'classical' TB symptoms in those >65 years of age. Clinicians need to be vigilant despite the lack of these symptoms. Median duration of symptoms was twice that in older patients versus younger patients (90 days versus 45) with a longer time to starting treatment (7 days versus 2). The total duration (in months) from symptom onset was also higher at 3 months versus 2 months. A higher proportion of older patients (32%) started treatment greater than 4 months from symptom onset compared to 13.1% in younger patients. Biases in this work including recording bias due to the retrospective nature of symptom recording, the effect of selecting younger patients from more recent years (to improve data availability) and the low proportion of patients over 65 which had symptom and treatment data available. (Table presented).

Institution

(Abbara, Mahomed, Corrah, Davidson) London North West Healthcare NHS Trust, London, United Kingdom (Hardman) University College Hospital, London, United Kingdom

(Collin) University of Bristol, Bristol, United Kingdom

(Kon) Imperial Healthcare NHS Trust, London, United Kingdom

(Sullivan, Buell) Imperial College, London, United Kingdom

(Hansel) NHLI, Imperial College, London, United Kingdom

Publisher

BMJ Publishing Group

Link to the Ovid Full Text or citation:

[Click here for full text options](http://ovidsp.ovid.com/ovidweb.cgi?T=JS&CSC=Y&NEWS=N&PAGE=fulltext&D=emed18b&AN=615030876)

Link to the External Link Resolver:

[SFX](http://whel-primo.hosted.exlibrisgroup.com/openurl/44WHELF_CAR/44WHELF_CAR_services_page?sid=OVID:embase&id=pmid:&id=doi:10.1136%2Fthoraxjnl-2016-209333.96&issn=1468-3296&isbn=&volume=71&issue=3&spage=A51&pages=A51-A52&date=2016&title=Thorax&atitle=The+nature+and+duration+of+symptoms+and+time+to+starting+treatment+comparing+older+with+younger+pulmonary+tuberculosis+patients&aulast=Abbara&pid=<author>Abbara+A.%3BHardman+E.%3BCollin+S.M.%3BKon+O.M.%3BMahomed+Z.%3BSullivan+J.A.L.%3BBuell+K.%3BHansel+T.%3BCorrah+T.%3BDavidson+R.N.<%2Fauthor><AN>615030876<%2FAN><DT>Journal%3A+Conference+Abstract<%2FDT>)

5.

The effect of diabetes control status on treatment response in pulmonary tuberculosis: A prospective study.

Yoon Y.S., Jung J.-W., Jeon E.J., Seo H., Ryu Y.J., Yim J.-J., Kim Y.H., Lee B.-H., Park Y.B., Lee B.J., Kang H., Choi J.C.

Thorax. 72 (3) (pp 263-270), 2017. Date of Publication: 01 Mar 2017.

AN: 611939205

Background: Uncontrolled diabetes, unlike controlled diabetes, is associated with an impaired immune response. However, little is known about the impact of the status of diabetes control on clinical features and treatment outcomes in patients with pulmonary TB (PTB). We conducted this study to evaluate whether the status of diabetes control influences clinical manifestations and treatment responses in PTB. Methods: A multicentre prospective study was performed between September 2012 and September 2014. The patients were categorised into three groups according to the glycated haemoglobin (HbA1C) level: PTB without diabetes mellitus (non-DM), PTB with controlled diabetes (controlled-DM) and PTB with uncontrolled diabetes (uncontrolled-DM). The primary outcome was the sputum culture conversion rate after 2 months of intensive treatment. Results: Among 661 patients with PTB, 157 (23.8%) had diabetes and 108 (68.8%) had uncontrolled diabetes (HbA1C>7.0%). The uncontrolled-DM group exhibited more symptoms, positive sputum smears (p<0.001) and presence of cavities (p<0.001) than the non-DM group. Regarding treatment responses, patients with uncontrolled-DM were more likely to have a positive culture after 2 months (p=0.009) and either treatment failure (p=0.015) or death (p=0.027) compared with the non-DM group. In contrast, those with controlled-DM showed similar treatment responses to the non-DM group. In multivariable analysis, uncontrolled diabetes was an independent risk factor for a positive sputum culture after 2 months of treatment (adjusted OR, 2.11; p=0.042) and either treatment failure or death (adjusted OR, 4.11; p=0.022). Conclusions: Uncontrolled diabetes is an independent risk factor for poor treatment response in PTB. Copyright © BMJ Publishing Group Limited 2017.

Institution

(Yoon) Division of Pulmonology and Critical Care Medicine, Department of Internal Medicine, Dongguk University Ilsan Hospital, Dongguk University College of Medicine, Goyang, South Korea (Jung, Choi) Division of Pulmonary Medicine, Department of Internal Medicine, Chung-Ang University, School of Medicine, HeukSeok Ro 102, Dong Jak Gu, Seoul 06973, South Korea

(Jeon) Department of Internal Medicine, Hallym Hospital, Incheon, South Korea

(Seo) Department of Tuberculosis, Seobuk Hospital, Seoul Metropolitan Government, Seoul, South Korea

(Ryu) Division of Pulmonary and Critical Care Medicine, Department of Internal Medicine, Ewha Womans University, School of Medicine, Seoul, South Korea

(Yim) Division of Pulmonary and Critical Care Medicine, Department of Internal Medicine, Seoul National University College of Medicine, Seoul, South Korea

(Kim) Division of Pulmonary and Critical Care Medicine, Department of Internal Medicine, Kyung Hee University Hospital at Gangdong, School of Medicine, Kyung Hee University, Seoul, South Korea

(Lee) Department of Pulmonology and Allergy, Eulji Hospital, College of Medicine, Eulji University, Seoul, South Korea

(Park) Department of Internal Medicine, Hallym University Medical Center, Kangdong Sacred Heart Hospital, Seoul, South Korea

(Lee) Division of Pulmonary and Critical Care Medicine, Department of Internal Medicine, Veterans Health Service Medical Center, Seoul, South Korea

(Kang) Department of Anesthesiology and Pain Medicine, Chung-Ang University, School of Medicine, Seoul, South Korea

Publisher

BMJ Publishing Group (E-mail: subscriptions@bmjgroup.com)

Link to the Ovid Full Text or citation:

[Click here for full text options](http://ovidsp.ovid.com/ovidweb.cgi?T=JS&CSC=Y&NEWS=N&PAGE=fulltext&D=emed18b&AN=611939205)

Link to the External Link Resolver:

[SFX](http://whel-primo.hosted.exlibrisgroup.com/openurl/44WHELF_CAR/44WHELF_CAR_services_page?sid=OVID:embase&id=pmid:&id=doi:10.1136%2Fthoraxjnl-2015-207686&issn=0040-6376&isbn=&volume=72&issue=3&spage=263&pages=263-270&date=2017&title=Thorax&atitle=The+effect+of+diabetes+control+status+on+treatment+response+in+pulmonary+tuberculosis%3A+A+prospective+study&aulast=Yoon&pid=<author>Yoon+Y.S.%3BJung+J.-W.%3BJeon+E.J.%3BSeo+H.%3BRyu+Y.J.%3BYim+J.-J.%3BKim+Y.H.%3BLee+B.-H.%3BPark+Y.B.%3BLee+B.J.%3BKang+H.%3BChoi+J.C.<%2Fauthor><AN>611939205<%2FAN><DT>Journal%3A+Article<%2FDT>)

6.

Thoracic tuberculosis (TB) in 75 to 100 year-old patients.

Garrido I.S., Huguet E.T., Garay J.G., Alana P.G., Ayuso E.C., Aranaga I.L., Basanez R.A.

European Respiratory Journal. Conference: European Respiratory Society Annual Congress 2016. United Kingdom. Conference Start: 20160903. Conference End: 20160907. 48 (no pagination), 2016. Date of Publication: 01 Sep 2016.

AN: 614780228

In developed countries, the incidence of TB is decreasing and the age of onset is delayed relying more on the reactivation of old infection. In 2014, the age-group >64 had the highest incidence in our area (21.4/100,000). The immunosenescence and comorbidities appear to play an important role. Aim: To analyse the clinical aspects and treatment outcome of TB in our elderly patients. Methods: Retrospective study. All patients with TB >75 years in the urban area of Bilbao between 2009- 2013 were included. Controls were randomly selected from patients <65 years with TB from the same metropolitan area and period. VIH-positive patients were excluded. Variables collected were sociodemographic data, comorbidities, clinico-radiological and microbiologic features, toxicity and mortality. Results: 50 patients with TB >75 years, mean age 82+/-5, with a Charlson index 2.9: COPD (38%), heart failure (36%), diabetes (24%), dementia (24%), neoplasms (20%). 19% were institutionalized. Previous history of TB was present in 44% of patients. Main results are shown in the table below. CONCLUSIONS: TB in very elderly patients represents a major challenge for the future: -Miliary presentations are more frequent -Mortality and liver toxicity are higher -Difficult and delayed diagnosis due to unespecific clinico-radiological features -Institutionalized patients present a great epidemiological risk. -More hospital admissions.

Publisher

European Respiratory Society

Link to the Ovid Full Text or citation:

[Click here for full text options](http://ovidsp.ovid.com/ovidweb.cgi?T=JS&CSC=Y&NEWS=N&PAGE=fulltext&D=emed18b&AN=614780228)

Link to the External Link Resolver:

[SFX](http://whel-primo.hosted.exlibrisgroup.com/openurl/44WHELF_CAR/44WHELF_CAR_services_page?sid=OVID:embase&id=pmid:&id=doi:10.1183%2F13993003.congress-2016.PA2654&issn=1399-3003&isbn=&volume=48&issue=60&spage=no+pagination&pages=no+pagination&date=2016&title=European+Respiratory+Journal&atitle=Thoracic+tuberculosis+(TB)+in+75+to+100+year-old+patients&aulast=Garrido&pid=<author>Garrido+I.S.%3BHuguet+E.T.%3BGaray+J.G.%3BAlana+P.G.%3BAyuso+E.C.%3BAranaga+I.L.%3BBasanez+R.A.<%2Fauthor><AN>614780228<%2FAN><DT>Journal%3A+Conference+Abstract<%2FDT>)

7.

Risk factors and treatment outcomes in patients with chronic pulmonary aspergillosis.

Agarwal K., Chowdhary A., Rahman M., Chaudhry A., Gaur S.N.

European Respiratory Journal. Conference: European Respiratory Society Annual Congress 2016. United Kingdom. Conference Start: 20160903. Conference End: 20160907. 48 (no pagination), 2016. Date of Publication: 01 Sep 2016.

AN: 614779718

Background: Chronic pulmonary aspergillosis (CPA) is still under-diagnosed in high tuberculosis (TB) burden areas like India where similar clinical presentation and disregard for underlying risk factors lead to its misdiagnosis as the latter. Aim: To evaluate patient characteristics and treatment outcomes in CPA patients Methods: Consecutive patients with a differential diagnosis of CPA enrolled from Jan. 2012-Dec. 2013 were administered oral itraconazole (ITZ) or voriconazole. Weight, BMI, haemoglobin (Hb), total leucocyte count (TLC), specific IgG, precipitins, CRP, sputum fungus and radiology were followed till 2 years after the end of treatment. Negative specific IgG was taken as end-point of therapy. Results: CPA was diagnosed in 24(70.5%) patients including 10 CCPA, 9 CNPA, 3 CFPA and 2 simple aspergilloma. Mean age was 47.2+/-10.9years. Average 2.1 risk factors were present per patient; TB was the commonest risk factor (29.3%) but ABPA, sarcoidosis and diabetes mellitus were significantly associated with development of CPA. Cavitation was noted in 29% and aspergilloma in 12%. Specific IgG and serum precipitins were raised in 79% and CRP 92%. A. fumigatus was isolated in 80%. Both drugs exerted similar but significant improvements in weight (51.8+/-7 vs 56.3+/-8 Kg), BMI (18.7+/-3.1 vs 20.3+/-3.0 Kg/m2), Hb (11.3+/-1.9 vs 12.3+/-1.7 g/dL), TLC (10.2+/-3.1 vs 7.9+/-2.6x109/muL), precipitins and specific IgG (69.1; 26.5-84.2 vs 29.6; 0-39.3 MgA/L). Two cases on ITZ relapsed at 12 months; no deaths were reported. 2 3 Conclusions: CPA should be investigated in patients with multiple risk factors. Triazole antifungals are effective in treatment. Specific IgG titers can be used to assess disease activity and response to treatment.

Publisher

European Respiratory Society

Link to the Ovid Full Text or citation:

[Click here for full text options](http://ovidsp.ovid.com/ovidweb.cgi?T=JS&CSC=Y&NEWS=N&PAGE=fulltext&D=emed18b&AN=614779718)

Link to the External Link Resolver:

[SFX](http://whel-primo.hosted.exlibrisgroup.com/openurl/44WHELF_CAR/44WHELF_CAR_services_page?sid=OVID:embase&id=pmid:&id=doi:10.1183%2F13993003.congress-2016.PA2647&issn=1399-3003&isbn=&volume=48&issue=60&spage=no+pagination&pages=no+pagination&date=2016&title=European+Respiratory+Journal&atitle=Risk+factors+and+treatment+outcomes+in+patients+with+chronic+pulmonary+aspergillosis&aulast=Agarwal&pid=<author>Agarwal+K.%3BChowdhary+A.%3BRahman+M.%3BChaudhry+A.%3BGaur+S.N.<%2Fauthor><AN>614779718<%2FAN><DT>Journal%3A+Conference+Abstract<%2FDT>)

8.

Impact of type of calcineurin inhibitor on post-transplant tuberculosis: Single-center study from India.

Agarwal S.K., Bhowmik D., Mahajan S., Bagchi S.

Transplant Infectious Disease. 19 (1) (no pagination), 2017. Article Number: e12626. Date of Publication: 01 Feb 2017.

AN: 613850784

Introduction: Tuberculosis (TB) is an important cause of morbidity and mortality in renal transplant recipients. Immunosuppressive drugs are one of the most important risk factor for post-transplant tuberculosis (PTTB). A paucity of data exists about the impact of the type of calcineurin inhibitor on PTTB. Methods: In this retrospective study, all adult patients on calcineurin inhibitor-based immunosuppression were included. Patients receiving TB chemoprophylaxis were excluded. Diabetes, duration of dialysis, hepatitis B and C, past treated TB, induction therapy, type of antimetabolite, acute rejection, new onset of diabetes after renal transplantation (RT) (NODAT) and cytomegalovirus (CMV) were analyzed in tacrolimus (Tac) and cyclosporine (CsA) groups. Primary outcome was incidence of TB and secondary outcomes were timeline of development of TB after RT and pattern of TB in the two groups. Results: Of the 1664 patients included, 582 patients received CsA-based immunosuppression while 1082 received Tac-based immunosuppression. Duration of dialysis, positive tuberculin skin test, use of induction, mycophenolate mofetil use, CMV infection, and NODAT were significantly more, and hepatitis B infection, past treated TB, and acute rejection episodes were significantly less in the Tac group. At the end of follow-up, incidence of TB in the Tac group was significantly less than in the CsA group (6.1% vs 19.9%, P<.001). Mean time for development of TB after RT was similar in both the groups and nodal and disseminated TB were more common in the Tac group. Conclusion: In conclusion, our study shows that use of Tac as compared to CsA significantly decreases incidence of PTTB. Time of infection since transplant was similar in both the groups. However, nodal and disseminated TB were more common in the Tac group. Copyright © 2016 John Wiley & Sons A/S. Published by John Wiley & Sons Ltd

Institution

(Agarwal, Bhowmik, Mahajan, Bagchi) Department of Nephrology, All India Institute of Medical Sciences, New Delhi, India

Publisher

Blackwell Publishing Inc. (E-mail: subscrip@blackwellpub.com)

Link to the Ovid Full Text or citation:

[Click here for full text options](http://ovidsp.ovid.com/ovidweb.cgi?T=JS&CSC=Y&NEWS=N&PAGE=fulltext&D=emed18b&AN=613850784)

Link to the External Link Resolver:

[SFX](http://whel-primo.hosted.exlibrisgroup.com/openurl/44WHELF_CAR/44WHELF_CAR_services_page?sid=OVID:embase&id=pmid:&id=doi:10.1111%2Ftid.12626&issn=1398-2273&isbn=&volume=19&issue=1&spage=no+pagination&pages=no+pagination&date=2017&title=Transplant+Infectious+Disease&atitle=Impact+of+type+of+calcineurin+inhibitor+on+post-transplant+tuberculosis%3A+Single-center+study+from+India&aulast=Agarwal&pid=<author>Agarwal+S.K.%3BBhowmik+D.%3BMahajan+S.%3BBagchi+S.<%2Fauthor><AN>613850784<%2FAN><DT>Journal%3A+Article<%2FDT>)

9.

Early interventions for diabetes related tuberculosis associate with hastened sputum microbiological clearance in Virginia, USA.

Alkabab Y., Keller S., Dodge D., Houpt E., Staley D., Heysell S.

BMC Infectious Diseases. 17 (1) (no pagination), 2017. Article Number: 125. Date of Publication: 06 Feb 2017.

AN: 614295261

Background: Diabetes complicates tuberculosis (TB) treatment including a prolonged time of sputum culture conversion to negative growth. Since 2013 in Virginia, interventions early in the treatment course have used therapeutic drug monitoring and dose correction for isoniazid and rifampin after 2 weeks of TB treatment in patients with diabetes along with nurse manager initiated diabetes education and linkage to care. Methods: A retrospective cohort study of the state TB registry was performed for patients initiating drug-susceptible pulmonary TB treatment that were matched for age, gender, chest imaging and sputum smear status to compare time to sputum culture conversion and other clinical outcomes in the pre-and post-intervention groups. Results: Three hundred sixty-three patients had documented time to sputum culture conversion in the pre-and post-intervention periods, including 56 (15%) with diabetes. Seventy-four (57%) of all patients with diabetes were >60 years of age at treatment initiation. Twenty-six patients with diabetes were matched in each group. Mean time to sputum culture conversion in the post-intervention group was 42 +/- 22 days compared to the pre-intervention group of 62 +/- 31 days (p = 0.01). In the post-intervention group 21 (80%) of patients with diabetes had culture conversion by 2 months compared to 13 (50%) in the pre-intervention group (p = 0.04). Conclusions: Early interventions for diabetes related TB in the programmatic setting may hasten sputum culture conversion. Copyright © 2017 The Author(s).

Institution

(Alkabab, Houpt, Heysell) University of Virginia School of Medicine, Division of Infectious Diseases and International Health, P.O. Box 801340, Charlottesville, VA 22908, United States (Keller) TB Control, Virginia Department of Health, Division of Disease Prevention, 109 Governor Street, Richmond, VA 23219, United States

(Dodge, Staley) Virginia Department of Health, TB Control and Newcomer Health, 109 Governor Street, Room 326, PO Box 2448, Richmond, VA 23218, United States

Publisher

BioMed Central Ltd. (E-mail: info@biomedcentral.com)

Link to the Ovid Full Text or citation:

[Click here for full text options](http://ovidsp.ovid.com/ovidweb.cgi?T=JS&CSC=Y&NEWS=N&PAGE=fulltext&D=emed18b&AN=614295261)

Link to the External Link Resolver:

[SFX](http://whel-primo.hosted.exlibrisgroup.com/openurl/44WHELF_CAR/44WHELF_CAR_services_page?sid=OVID:embase&id=pmid:&id=doi:10.1186%2Fs12879-017-2226-y&issn=1471-2334&isbn=&volume=17&issue=1&spage=no+pagination&pages=no+pagination&date=2017&title=BMC+Infectious+Diseases&atitle=Early+interventions+for+diabetes+related+tuberculosis+associate+with+hastened+sputum+microbiological+clearance+in+Virginia%2C+USA&aulast=Alkabab&pid=<author>Alkabab+Y.%3BKeller+S.%3BDodge+D.%3BHoupt+E.%3BStaley+D.%3BHeysell+S.<%2Fauthor><AN>614295261<%2FAN><DT>Journal%3A+Article<%2FDT>)

10.

Cost-effectiveness of isoniazid preventive therapy for HIVinfected pregnant women in India.

Kapoor S., Gupta A., Shah M.

International Journal of Tuberculosis and Lung Disease. 20 (1) (pp 85-92), 2016. Date of Publication: 01 Jan 2016.

AN: 607442788

Background: India has a high burden of active tuberculosis (TB) and human immunodeficiency virus (HIV) infection. Pregnancy increases the risks of developing TB in HIV-infected women. Isoniazid preventive therapy (IPT) reduces progression to TB, but may increase costs and hepatotoxicity. The costeffectiveness of IPT for HIV-infected pregnant women in India is unknown. DESIGN: We evaluated the cost-effectiveness of antepartum IPT among HIV-infected women in India using a decision-analytic model. We compared current practice (no IPT) with: Intervention 1 (IPT regardless of CD4 count) and Intervention 2 (IPT for those with CD4 count < 200 cells/mul). We modeled IPT irrespective of tuberculin skin test (TST) status and TST-driven strategies. Primary outcomes were anticipated costs, disability-adjusted life-years (DALYs) and TB cases. R ESULT S: Both IPT interventions are highly costeffective compared to no IPT at current willingness-topay thresholds (respectively US$178.00 and US$201.00 per DALY averted for Interventions 1 and 2). However, providing IPT irrespective of CD4 count results in the greatest health benefits (21 TB cases averted/1000 patients) compared to current practice. IPT irrespective of TSTstatus was also highly cost-effective compared to TST-driven IPT (respectively US$1027.00 and US$1154.00/DALY averted for Interventions 1 and 2). CONCLUS ION: Antepartum IPT for HIV-infected women is highly cost-effective for TB prevention compared to current practices in India. Copyright © 2016 The Union.

PMID

26688533 [<http://www.ncbi.nlm.nih.gov/pubmed/?term=26688533>]

Institution

(Kapoor, Gupta, Shah) Johns Hopkins University, School of Medicine, PCTB Building-224, 725 N Wolfe St., Baltimore, MD 21205, United States

Publisher

International Union against Tubercul. and Lung Dis.

Link to the Ovid Full Text or citation:

[Click here for full text options](http://ovidsp.ovid.com/ovidweb.cgi?T=JS&CSC=Y&NEWS=N&PAGE=fulltext&D=emed18b&AN=607442788)

Link to the External Link Resolver:

[SFX](http://whel-primo.hosted.exlibrisgroup.com/openurl/44WHELF_CAR/44WHELF_CAR_services_page?sid=OVID:embase&id=pmid:26688533&id=doi:10.5588%2Fijtld.15.0391&issn=1027-3719&isbn=&volume=20&issue=1&spage=85&pages=85-92&date=2016&title=International+Journal+of+Tuberculosis+and+Lung+Disease&atitle=Cost-effectiveness+of+isoniazid+preventive+therapy+for+HIVinfected+pregnant+women+in+India&aulast=Kapoor&pid=<author>Kapoor+S.%3BGupta+A.%3BShah+M.<%2Fauthor><AN>607442788<%2FAN><DT>Journal%3A+Article<%2FDT>)

11.

Optimizing treatment outcome of first-line anti-tuberculosis drugs: the role of therapeutic drug monitoring.

Verbeeck R.K., Gunther G., Kibuule D., Hunter C., Rennie T.W.

European Journal of Clinical Pharmacology. 72 (8) (pp 905-916), 2016. Date of Publication: 01 Aug 2016.

AN: 610850501

Introduction: Tuberculosis (TB) remains one of the world's deadliest communicable diseases. Although cure rates of the standard four-drug (rifampicin, isoniazid, pyrazinamide, ethambutol) treatment schedule can be as high as 95-98 % under clinical trial conditions, success rates may be much lower in less well resourced countries. Unsuccessful treatment with these first-line anti-TB drugs may lead to the development of multidrug resistant and extensively drug resistant TB. The intrinsic interindividual variability in the pharmacokinetics (PK) of the first-line anti-TB drugs is further exacerbated by co-morbidities such as HIV infection and diabetes. Methods: Therapeutic drug monitoring has been proposed in an attempt to optimize treatment outcome and reduce the development of drug resistance. Several studies have shown that maximum plasma concentrations (Cmax), especially of rifampicin and isoniazid, are well below the proposed target Cmax concentrations in a substantial fraction of patients being treated with the standard four-drug treatment schedule, even though treatment's success rate in these studies was typically at least 85 %. Discussion: The proposed target Cmax concentrations are based on the concentrations of these agents achieved in healthy volunteers and patients receiving the standard doses. Estimation of Cmax based on one or two sampling times may not have the necessary accuracy since absorption rate, especially for rifampicin, may be highly variable. In addition, minimum inhibitory concentration (MIC) variability should be taken into account to set clinically meaningful susceptibility breakpoints. Clearly, there is a need to better define the key target PK and pharmacodynamic (PD) parameters for therapeutic drug monitoring (TDM) of the first-line anti-TB drugs to be efficacious, Cmax (or area under the curve (AUC)) and Cmax/MIC (or AUC/MIC). Conclusion: Although TDM of first-line anti-TB drugs has been successfully used in a limited number of specialized centers to improve treatment outcome in slow responders, a better characterization of the target PK and/or PK/PD parameters is in our opinion necessary to make it cost-effective. Copyright © 2016, Springer-Verlag Berlin Heidelberg.

PMID

27305904 [<http://www.ncbi.nlm.nih.gov/pubmed/?term=27305904>]

Institution

(Verbeeck, Kibuule, Hunter, Rennie) Faculty of Health Sciences, University of Namibia, Windhoek, Namibia (Gunther) Katutura State Hospital, Windhoek, Namibia

(Gunther) Leibniz Center for Medicine and Biosciences, Borstel, Germany

Publisher

Springer Verlag (E-mail: service@springer.de)

Link to the Ovid Full Text or citation:

[Click here for full text options](http://ovidsp.ovid.com/ovidweb.cgi?T=JS&CSC=Y&NEWS=N&PAGE=fulltext&D=emed18b&AN=610850501)

Link to the External Link Resolver:

[SFX](http://whel-primo.hosted.exlibrisgroup.com/openurl/44WHELF_CAR/44WHELF_CAR_services_page?sid=OVID:embase&id=pmid:27305904&id=doi:10.1007%2Fs00228-016-2083-4&issn=0031-6970&isbn=&volume=72&issue=8&spage=905&pages=905-916&date=2016&title=European+Journal+of+Clinical+Pharmacology&atitle=Optimizing+treatment+outcome+of+first-line+anti-tuberculosis+drugs%3A+the+role+of+therapeutic+drug+monitoring&aulast=Verbeeck&pid=<author>Verbeeck+R.K.%3BGunther+G.%3BKibuule+D.%3BHunter+C.%3BRennie+T.W.<%2Fauthor><AN>610850501<%2FAN><DT>Journal%3A+Review<%2FDT>)

12.

Treatment outcomes of fixed-dose combination versus separate tablet regimens in pulmonary tuberculosis patients with or without diabetes in Qatar.

Al-Shaer M.H., Mansour H., Elewa H., Salameh P., Iqbal F.

BMC Infectious Diseases. 17 (1) (no pagination), 2017. Article Number: 118. Date of Publication: 02 Feb 2017.

AN: 614259878

Background: Tuberculosis is considered the second most common cause of death due to infectious agent. The currently preferred regimen for treatment of pulmonary tuberculosis (PTB) is isoniazid, rifampin, pyrazinamide, and ethambutol, which has been used either as separate tablets (ST) or as fixed-dose combination (FDC). To date, no studies have compared both regimens in Qatar. We aim to evaluate the safety and effectiveness of FDC and ST regimen for treating PTB, in addition to comparing safety and efficacy of FDC and ST regimens in patients with diabetes treated for TB. Methods: A retrospective observational study was conducted in two general hospitals in Qatar. Patients diagnosed with PTB received anti-tuberculosis medications (either as FDC or ST) administered by the nurse. Sputum smears were tested weekly. We assessed the time to negative sputum smear and incidence of adverse events among FDC and ST groups. Results: The study included 148 patients. FDC was used in 90 patients (61%). Effectiveness was not different between FDC and ST regimens as shown by mean time to sputum conversion (29.9+/-18.3 vs. 35.6+/-23days, p=0.12). Similarly, there was no difference in the incidence of adverse events, except for visual one that was higher in ST group. Among the 33 diabetic patients, 19 received the FDC and had faster sputum conversion compared to those who received ST (31+/-12 vs. 49.4+/-30.9days, p=0.05). Overall, diabetic patients needed longer time for sputum conversion and had more hepatotoxic and gastric adverse events compared to non-diabetics. Conclusion: ST group had higher visual side effects compared to FDC. FDC may be more effective in diabetic patients; however, further studies are required to confirm such finding. Copyright © 2017 The Author(s).

Institution

(Al-Shaer) Al Wakra Hospital - Hamad Medical Corporation, Department of Pharmacy, Doha, Qatar (Al-Shaer) University of Florida, Department of Pharmacotherapy and Translational Research, Infectious Disease Pharmacokinetics Laboratory, College of Pharmacy and Emerging Pathogens Institute, Gainesville, FL 32610, United States

(Mansour, Salameh) Lebanese American University, School of Pharmacy, P.O Box 36-S 23, Byblos, Lebanon

(Elewa) Qatar University, College of Pharmacy, Doha, Qatar

(Salameh) Lebanese University, Faculty of Medical Sciences, Beirut, Lebanon

(Iqbal) Rumailah Hospital - Hamad Medical Corporation, Department of Pharmacy, Doha, Qatar

Publisher

BioMed Central Ltd. (E-mail: info@biomedcentral.com)

Link to the Ovid Full Text or citation:

[Click here for full text options](http://ovidsp.ovid.com/ovidweb.cgi?T=JS&CSC=Y&NEWS=N&PAGE=fulltext&D=emed18b&AN=614259878)

Link to the External Link Resolver:

[SFX](http://whel-primo.hosted.exlibrisgroup.com/openurl/44WHELF_CAR/44WHELF_CAR_services_page?sid=OVID:embase&id=pmid:&id=doi:10.1186%2Fs12879-017-2231-1&issn=1471-2334&isbn=&volume=17&issue=1&spage=no+pagination&pages=no+pagination&date=2017&title=BMC+Infectious+Diseases&atitle=Treatment+outcomes+of+fixed-dose+combination+versus+separate+tablet+regimens+in+pulmonary+tuberculosis+patients+with+or+without+diabetes+in+Qatar&aulast=Al-Shaer&pid=<author>Al-Shaer+M.H.%3BMansour+H.%3BElewa+H.%3BSalameh+P.%3BIqbal+F.<%2Fauthor><AN>614259878<%2FAN><DT>Journal%3A+Article<%2FDT>)

13.

Personalized medicine for patients with MDR-TB.

Olaru I.D., Lange C., Heyckendorf J.

Journal of Antimicrobial Chemotherapy. 71 (4) (pp 852-855), 2016. Article Number: dkv354. Date of Publication: 01 Apr 2016.

AN: 610060891

The emergence of MDR-TB is a cause of great concern due to difficulties in patient management and poor treatment outcomes. Currently the duration of treatment and the choice of drugs for patients with MDR-TB are standardized in many countries. This might not be the best approach since the optimal therapy may depend on different pathogen- and host-related features. Combining the introduction of technological innovations such as whole bacillary genome sequencing for the identification of drug-resistance-associated mutations, therapeutic drug monitoring and host-directed therapies with an individualized approach to MDR-TB management will likely lead to more tolerable, shorter and more efficient treatment regimens and an increase in the quality of life of those affected by MDR-TB. Copyright © The Author 2015. Published by Oxford University Press on behalf of the British Society for Antimicrobial Chemotherapy. All rights reserved.

PMID

26507429 [<http://www.ncbi.nlm.nih.gov/pubmed/?term=26507429>]

Institution

(Olaru, Lange, Heyckendorf) Division of Clinical Infectious Diseases, Research Center Borstel, Borstel, Germany (Olaru, Lange, Heyckendorf) Clinical Tuberculosis Center, German Center for Infection Research (DZIF), Borstel, Germany

(Olaru, Lange, Heyckendorf) International Health/Infectious Diseases, University of Lubeck, Lubeck, Germany

(Lange) Department of Medicine, Karolinska Institute, Stockholm, Sweden

(Lange) Department of Internal Medicine, University of Namibia, School of Medicine, Windhoek, Namibia

Publisher

Oxford University Press (E-mail: jnl.info@oup.co.uk)

Link to the Ovid Full Text or citation:

[Click here for full text options](http://ovidsp.ovid.com/ovidweb.cgi?T=JS&CSC=Y&NEWS=N&PAGE=fulltext&D=emed18b&AN=610060891)

Link to the External Link Resolver:

[SFX](http://whel-primo.hosted.exlibrisgroup.com/openurl/44WHELF_CAR/44WHELF_CAR_services_page?sid=OVID:embase&id=pmid:26507429&id=doi:10.1093%2Fjac%2Fdkv354&issn=0305-7453&isbn=&volume=71&issue=4&spage=852&pages=852-855&date=2016&title=Journal+of+Antimicrobial+Chemotherapy&atitle=Personalized+medicine+for+patients+with+MDR-TB&aulast=Olaru&pid=<author>Olaru+I.D.%3BLange+C.%3BHeyckendorf+J.<%2Fauthor><AN>610060891<%2FAN><DT>Journal%3A+Article<%2FDT>)

14.

Tolerability and healthcare utilization in maintenance hemodialysis patients undergoing treatment for tuberculosis-related conditions.

Hamadah A.M., Beaulieu L.M., Wilson J.W., Aksamit T.R., Gregoire J.R., Williams A.W., Dillon J.J., Albright R.C., Onuigbo M., Iyer V.K., Hickson L.J.

Nephron. 132 (3) (pp 198-206), 2016. Date of Publication: 01 Mar 2016.

AN: 608327383

Background: The incidence of tuberculosis (TB) in end-stage renal disease is significantly higher than that in the general population. Among those with kidney dysfunction, anti-TB treatment is associated with increased side effects, but the effect on healthcare utilization is unknown. Methods/Aim: To assess patient-reported symptoms, adverse effects and describe changes in healthcare utilization patterns during treatment for TB, we conducted a case series (n = 12) of patients receiving maintenance hemodialysis (HD) from Mayo Clinic Dialysis Services and concurrent drug therapy for TB from January 2002 through May 2014. Healthcare utilization (hospitalizations and emergency department (ED) visits independent of hospital admission) was compared before and during treatment. Results: Patients were treated for latent (n = 7) or active (n = 5) TB. The majority of patients with latent disease were treated with isoniazid (n = 5, 71%), while active-disease patients received a 4-drug regimen. Adverse effects were reported in 83% of patients. Compared to measurements prior to drug initiation, serum albumin and dialysis weights were similar at 3 months. Commonly reported anti-TB drug toxicities were described. More than half (58%) of the patients were hospitalized at least once. No ED or hospital admissions occurred in the period prior to drug therapy, but healthcare utilization increased during treatment in the latent disease group (hospitalization rate per person-month: pre 0 vs. post 1). Conclusions: Among HD patients, anti-TB therapy is associated with frequently reported symptoms and increased healthcare utilization. Among this subset, patients receiving treatment for latent disease may be those with greatest increase in healthcare use. Careful monitoring and early complication detection may help optimize medication adherence and minimize hospitalizations. Copyright © 2016 S. Karger AG, Basel.

PMID

26859893 [<http://www.ncbi.nlm.nih.gov/pubmed/?term=26859893>]

Institution

(Hamadah, Beaulieu, Gregoire, Williams, Dillon, Albright, Onuigbo, Iyer, Hickson) Division of Nephrology and Hypertension, Mayo Clinic, Rochester, MN, United States (Wilson) Division of Infectious Diseases, Mayo Clinic, Rochester, MN, United States

(Aksamit) Division of Pulmonary and Critical Care Medicine, Department of Internal Medicine, Mayo Clinic, Rochester, MN, United States

(Hickson) Robert D. and Patricia E. Kern Center for the Science of Health Care Delivery, Mayo Clinic, Rochester, MN, United States

(Hickson) Division of Nephrology and Hypertension, Department of Medicine, Mayo Clinic, 200 First Street SW, Rochester, MN 55905, United States

Publisher

S. Karger AG

Link to the Ovid Full Text or citation:

[Click here for full text options](http://ovidsp.ovid.com/ovidweb.cgi?T=JS&CSC=Y&NEWS=N&PAGE=fulltext&D=emed18b&AN=608327383)

Link to the External Link Resolver:

[SFX](http://whel-primo.hosted.exlibrisgroup.com/openurl/44WHELF_CAR/44WHELF_CAR_services_page?sid=OVID:embase&id=pmid:26859893&id=doi:10.1159%2F000444148&issn=1660-8151&isbn=&volume=132&issue=3&spage=198&pages=198-206&date=2016&title=Nephron&atitle=Tolerability+and+healthcare+utilization+in+maintenance+hemodialysis+patients+undergoing+treatment+for+tuberculosis-related+conditions&aulast=Hamadah&pid=<author>Hamadah+A.M.%3BBeaulieu+L.M.%3BWilson+J.W.%3BAksamit+T.R.%3BGregoire+J.R.%3BWilliams+A.W.%3BDillon+J.J.%3BAlbright+R.C.%3BOnuigbo+M.%3BIyer+V.K.%3BHickson+L.J.<%2Fauthor><AN>608327383<%2FAN><DT>Journal%3A+Conference+Paper<%2FDT>)

15.

The effect size of type 2 diabetes mellitus on tuberculosis drug resistance and adverse treatment outcomes.

Perez-Navarro L.M., Restrepo B.I., Fuentes-Dominguez F.J., Duggirala R., Morales-Romero J., Lopez-Alvarenga J.C., Comas I., Zenteno-Cuevas R.

Tuberculosis. 103 (pp 83-91), 2017. Date of Publication: 01 Mar 2017.

AN: 614301639

Objective To evaluate the effect size of type 2 diabetes mellitus (T2DM) on tuberculosis (TB) treatment outcomes and multi drug resistance (MDR). Methods A cohort with 507 individuals with diagnosed TB included 183 with coexistence of T2DM and TB (TB-T2DM). Participants were identified at the time of TB diagnosis and followed during the course of TB treatment. Then we computed relative risks and adjustments by Cox proportional hazards for outcome variables (drug resistance, death, relapse, treatment failure), and the size of their effect as Cohen's-d. Results Patients with TB-T2DM were more likely to remain positive for acid-fast bacilli after two months of anti-TB treatment RR = [2.01 (95% CI: 1.3, 3.1)], to have drug resistant (DR) [OR 3.5 (95% CI: 1.8, 6.7)] and multi-drug resistant (MDR) TB [OR 3.5 (95% CI: 1.8, 7.1)]. The Cohen's-d for DR or MDR in T2DM was 0.69 when compared with non-DM subjects. The T2DM patients had higher odds of resistance to isoniazid (OR 3.9, 95% CI: 2.01, 7.9), rifampicin (OR 3.4, 95% CI: 1.6, 7.2) and pyrazinamide (OR 9.4, 95% CI: 2.8, 25.6), and their effect sizes were >0.67. Patients with TB-T2DM (versus no DM) were more likely to present with MDR TB (HR 3.1; 95% CI: 1.7, 5.8; p < 0.001), treatment failure (HR 2.04; 95% CI: 1.07, 3.8; p = 0.02) and relapse (HR 1.86; 95% CI: 1.09, 3.1; p = 0.02), with effect size >0.34. Conclusion T2DM showed a substantial contribution to the presence of DR or MDR-TB and to adverse clinical outcomes during and after TB treatment. Our findings support the importance for routine screening of T2DM among newly-diagnosed TB patients in order to stratify them for immediate DR assessment, and highlight the need for clinical trials to evaluate variations to the standard TB treatment in TB-T2DM to prevent adverse treatment outcomes. Copyright © 2017 Elsevier Ltd

Author NameID

Perez-Navarro, Lucia Monserrat; ORCID: <http://orcid.org/0000-0002-9281-9202> Restrepo, Blanca I.; ORCID: <http://orcid.org/0000-0002-3743-9098>

Zenteno-Cuevas, Roberto; ORCID: <http://orcid.org/0000-0001-9597-127X>

Institution

(Perez-Navarro) Nephrology Service, Research Division, Hospital General de Mexico "Dr. Eduardo Liceaga", Mexico (Perez-Navarro, Morales-Romero, Zenteno-Cuevas) Public Health Institute, University of Veracruz, Veracruz, Mexico

(Restrepo) University of Texas Health Science Center Houston, Brownsville Campus, Brownsville, TX, United States

(Fuentes-Dominguez) Mycobacteriosis State Program, Veracruz Health Services, Mexico

(Duggirala, Lopez-Alvarenga) South Texas Diabetes and Obesity Institute, University of Texas Rio Grande Valley, Edinburg, TX, United States

(Comas) Unidad de Genomica de la Tuberculosis, Instituto de Biomedicina de Valencia, Consejo Superior de Investigaciones Cientificas, Valencia, Spain

(Zenteno-Cuevas) Red multidisciplinaria de Investigacion en tuberculosis, Mexico

Publisher

Churchill Livingstone

Link to the Ovid Full Text or citation:

[Click here for full text options](http://ovidsp.ovid.com/ovidweb.cgi?T=JS&CSC=Y&NEWS=N&PAGE=fulltext&D=emed18b&AN=614301639)

Link to the External Link Resolver:

[SFX](http://whel-primo.hosted.exlibrisgroup.com/openurl/44WHELF_CAR/44WHELF_CAR_services_page?sid=OVID:embase&id=pmid:&id=doi:10.1016%2Fj.tube.2017.01.006&issn=1472-9792&isbn=&volume=103&issue=&spage=83&pages=83-91&date=2017&title=Tuberculosis&atitle=The+effect+size+of+type+2+diabetes+mellitus+on+tuberculosis+drug+resistance+and+adverse+treatment+outcomes&aulast=Perez-Navarro&pid=<author>Perez-Navarro+L.M.%3BRestrepo+B.I.%3BFuentes-Dominguez+F.J.%3BDuggirala+R.%3BMorales-Romero+J.%3BLopez-Alvarenga+J.C.%3BComas+I.%3BZenteno-Cuevas+R.<%2Fauthor><AN>614301639<%2FAN><DT>Journal%3A+Article<%2FDT>)

16.

Association between diabetes mellitus and multi-drug-resistant tuberculosis: A protocol for a systematic review and meta-analysis.

Tegegne B.S., Habtewold T.D., Mengesha M.M., Burgerhof J.G.M.

Systematic Reviews. 6 (1) (no pagination), 2017. Article Number: 6. Date of Publication: 14 Jan 2017.

AN: 614054547

Introduction: Multi-drug-resistant tuberculosis (MDR-TB) has emerged as a challenge to global tuberculosis (TB) control and remains a major public health concern in many countries. Diabetes mellitus (DM) is an increasingly recognized comorbidity that can both accelerate TB disease and complicate its treatment. The aim of this study is to summarize available evidence on the association of DM and MDR-TB among TB patients and to provide a pooled estimate of risks. Methods: All studies published in English before October 2016 will be searched using comprehensive search strings through PubMed, EMBASE, Web of Science, and WHO Global Health Library databases which have reported the association of DM and MDR-TB in adults with TB (age > =15). Two authors will independently collect detailed information using structured data abstraction form. The quality of studies will be checked using Newcastle-Ottawa Scale for cohort and case-control studies and the Agency for Healthcare Research and Quality tool for cross-sectional studies. Heterogeneity between included studies will be assessed using the I2 statistic. We will check potential publication bias by visual inspection of the funnel plot and Egger's regression test statistic. We will use the random effects model to compute a pooled estimate. Discussion: Increases in the burden of non-communicable diseases and aging populations are changing the importance of different risk factors for TB, and the profile of comorbidities and clinical challenges for people with TB. Although classic risk factors and comorbidities such as overcrowding, under-nutrition, silicosis, and HIV infection are crucial to address, chronic conditions like diabetes are important factors that impair host defenses against TB. Thus, undertaking integrated multifaceted approach is remarkably necessary for reducing the burden of DM and successful TB treatment outcome. Systematic review registration: PROSPERO CRD42016045692. Copyright © 2017 The Author(s).

Institution

(Tegegne, Mengesha) Haramaya University, Department of Public Health, College of Health and Medical Sciences, Harar, Ethiopia (Tegegne, Habtewold, Burgerhof) University of Groningen, University Medical Center Groningen, Department of Epidemiology, Groningen, Netherlands

Publisher

BioMed Central Ltd. (E-mail: info@biomedcentral.com)

Link to the Ovid Full Text or citation:

[Click here for full text options](http://ovidsp.ovid.com/ovidweb.cgi?T=JS&CSC=Y&NEWS=N&PAGE=fulltext&D=emed18b&AN=614054547)

Link to the External Link Resolver:

[SFX](http://whel-primo.hosted.exlibrisgroup.com/openurl/44WHELF_CAR/44WHELF_CAR_services_page?sid=OVID:embase&id=pmid:&id=doi:10.1186%2Fs13643-017-0407-9&issn=2046-4053&isbn=&volume=6&issue=1&spage=no+pagination&pages=no+pagination&date=2017&title=Systematic+Reviews&atitle=Association+between+diabetes+mellitus+and+multi-drug-resistant+tuberculosis%3A+A+protocol+for+a+systematic+review+and+meta-analysis&aulast=Tegegne&pid=<author>Tegegne+B.S.%3BHabtewold+T.D.%3BMengesha+M.M.%3BBurgerhof+J.G.M.<%2Fauthor><AN>614054547<%2FAN><DT>Journal%3A+Article<%2FDT>)

17.

Host-directed therapies for multidrug resistant tuberculosis.

Zumla A., Maeurer M.

International Journal of Mycobacteriology. 5 (pp S21-S22), 2016. Date of Publication: 01 Dec 2016.

AN: 613939627

Tuberculosis (TB) causes 1.3 million deaths annually. There are 0.5 million cases of multidrug resistant TB (MDR-TB) and the number of cases is rising globally. The current status quo of the lengthy treatment duration and poor treatment outcomes associated with MDR/extensively drug-resistant TB, and those with comorbidity of TB with human immunodeficiency virus and noncommunicable diseases in sub-Saharan Africa is unacceptable. The TB drug pipeline remains sparse. New innovations for shortening the duration of therapy and improving treatment outcomes (cure and long-term functional disability due to lung damage) are urgently required. A wide range of host-directed therapies (HDT) are now available which require evaluation as adjuncts to current TB drug treatment. Examples are: (1) Repurposed drugs: * Analgesics/nonsteroidal anti-inflammatory drugs (cyclooxygenase-2 inhibitors, e.g., ibupofen).* Cholesterol-lowering drugs (3-hydroxy-3-methylglutaryl-coenzyme A reductase inhibitors, e.g., simvastatin).* Asthma drugs (leukotriene synthesis inhibitors, e.g., zileuton).* Diabetes drugs (reactive oxygen species removal and increased CD8+ T-cell responses (e.g., metformin).* Anticonvulsants (inhibition of histone deacylation, e.g., valproic acid).(2) Cellular therapy: using the patient's own bone marrow-derived stromal cells.(3) Immune therapies: for example, anti-interleukin-6/interleulin-6 receptor monoclonal antibody.(4) Therapeutic vaccines: protein vaccines (e.g., granulysin), DNA vaccines, environmental mycobacteria vaccines (e.g., Mycobacterium vaccae, Mycobacterium indicus pranii).(5) Micronutrients: for example, Vitamin D, zinc, probiotics, and so forth.The Host-directed Therapies Network consortium of 64 partners was launched in Cape Town after a meeting hosted by the South African Medical Research Council in April 2015. This network (which is open to anyone interested) plans to take forward a wide range of HDTs in randomized, placebo-controlled clinical trials as adjuncts to current TB treatment regimens with the aims of: (1) Shortening the duration of treatment for drug-sensitive TB and MDR-TB.(2) Improving treatment outcomes (mortality/morbidity) for MDR/extensively drug-resistant TB patients.(3) Improving lung function and preventing lung damage so that the patient can return to gainful employment after treatment.(4) Improving treatment outcomes for clinical presentations associated with tissue injury: (i) Miliary TB (including TB meningitis and TB pericarditis).(ii) Immune reconstitution inflammatory syndrome.(5) Improving treatment outcome of TB and/or human immunodeficiency virus-positive individuals with comorbidities such as noncommunicable diseases (e.g., diabetes, liver disease, and cardiac disease), and cancers. Copyright © 2016

Institution

(Zumla) Division of Infection and Immunity, University College London, NIHR Biomedical Research Center, UCL Hospitals NHS, Foundation Trust, London, United Kingdom (Maeurer) Centre for Allogeneic Stem Cell Transplantation, Karolinska University Hospital Huddinge, Stockholm, Sweden

Publisher

Elsevier Ltd

Link to the Ovid Full Text or citation:

[Click here for full text options](http://ovidsp.ovid.com/ovidweb.cgi?T=JS&CSC=Y&NEWS=N&PAGE=fulltext&D=emed18b&AN=613939627)

Link to the External Link Resolver:

[SFX](http://whel-primo.hosted.exlibrisgroup.com/openurl/44WHELF_CAR/44WHELF_CAR_services_page?sid=OVID:embase&id=pmid:&id=doi:10.1016%2Fj.ijmyco.2016.09.044&issn=2212-5531&isbn=&volume=5&issue=&spage=S21&pages=S21-S22&date=2016&title=International+Journal+of+Mycobacteriology&atitle=Host-directed+therapies+for+multidrug+resistant+tuberculosis&aulast=Zumla&pid=<author>Zumla+A.%3BMaeurer+M.<%2Fauthor><AN>613939627<%2FAN><DT>Journal%3A+Conference+Paper<%2FDT>)

18.

Assessment of diabetes among tuberculosis patients presenting at a tertiary care facility in Pakistan.

Irfan M., Salahuddin N., Masood Q., Ahmed O., Moosajee U.S., Hasan Z.

International Journal of Mycobacteriology. 5 (pp S248), 2016. Date of Publication: 01 Dec 2016.

AN: 613939477

Objective/background Pakistan ranks sixth among high-tuberculosis (TB)-burden countries worldwide and fifth worldwide for diabetes incidence. Although both these factors are independently known, the rate of diabetes among TB patients is not well known. We aimed to determine the prevalence of diabetes among patients with TB presenting at tertiary care health centers in Karachi, Pakistan. Methods A total of 216 patients with TB were recruited and their blood samples were taken for testing of glycosylated hemoglobin (HbA1c) and random blood sugar. Diabetes was defined as HbA1c > 6.5% and random blood sugar > 180 mg/dL, pre-diabetes as HbA1c 5.7-6.4%, and normoglycemia as HbA1c < 5.7%. Results Data for 211 patients were available and showed that 24 (11.4%) patients had diabetes. Of these, 17 were newly diagnosed, while seven were known diabetics. Prediabetes was identified in 45 (21.3%) cases. Of the TB patients, 165 were newly diagnosed, while 46 were retreatment cases. The majority of patients (60%) were underweight with a body mass index of <18.5. Conclusion This study identified 11.4% diabetics among TB patients presenting to a tertiary care facility. Despite the high diabetes incidence in Pakistan, 71% of the diabetics in the group studied did not know their status. Given the negative impact of diabetes on treatment outcomes of TB, it is important that screening for diabetes be included as initial workup for TB patients. Identification and management of diabetes would result in improved outcomes for TB treatment. Copyright © 2016

Institution

(Irfan, Masood, Ahmed, Hasan) The Aga Khan University, Karachi, Pakistan (Salahuddin, Moosajee) Indus Hospital, Karachi, Pakistan

Publisher

Elsevier Ltd

Link to the Ovid Full Text or citation:

[Click here for full text options](http://ovidsp.ovid.com/ovidweb.cgi?T=JS&CSC=Y&NEWS=N&PAGE=fulltext&D=emed18b&AN=613939477)

Link to the External Link Resolver:

[SFX](http://whel-primo.hosted.exlibrisgroup.com/openurl/44WHELF_CAR/44WHELF_CAR_services_page?sid=OVID:embase&id=pmid:&id=doi:10.1016%2Fj.ijmyco.2016.09.072&issn=2212-5531&isbn=&volume=5&issue=&spage=S248&pages=S248&date=2016&title=International+Journal+of+Mycobacteriology&atitle=Assessment+of+diabetes+among+tuberculosis+patients+presenting+at+a+tertiary+care+facility+in+Pakistan&aulast=Irfan&pid=<author>Irfan+M.%3BSalahuddin+N.%3BMasood+Q.%3BAhmed+O.%3BMoosajee+U.S.%3BHasan+Z.<%2Fauthor><AN>613939477<%2FAN><DT>Journal%3A+Conference+Paper<%2FDT>)

19.

Trends of comorbidities in taiwanese patients infected with multi-drug resistant tuberculosis in seeking favorable treatment outcomes.

Cheng S., Lin A., Chien L.C.

Annals of Global Health. Conference: 7th Annual CUGH Conference: Bridging to a Sustainable Future in Global Health. United States. Conference Start: 20160409. Conference End: 20160411. 82 (3) (pp 334-335), 2016. Date of Publication: 2016.

AN: 614045107

Background: Multi-drug resistant tuberculosis (MDRTB) accounts for 3.5% of new tuberculosis (TB) cases globally and is a major public health problem with potential global threats. Patients with comorbidities further complicate the complex treatment of MDRTB. Studies have shown that MDRTB patients with comorbidities have poorer treatment outcomes. The aim of this study is to evaluate clinical characteristics in MDRTB patients at TIHTC Taipei Hospital, Taiwan and raise awareness to help establish effective treatment regimens among comorbid patients infected with MDRTB. Methods: As a retrospective study, data for 26 patients with MDRTB from the years 2009 to 2014 was gathered from the TIHTC Taipei Hospital. The independent variables in the data included age, drug treatment regimen, drug sensitivities, and type as well as number of comorbidities. Trends were observed on the variables of age and the various comorbidities with theMDRTB patients. Findings: The sample (N=26) consisted of 16 males (61.5%) and 10 females (38.5%) and the mean age (+/-SD) of the patients with MDRTB was 58.3 +/- 19.4 years. Patients with at least one comorbidity was 50.0% (N=13) and at least two comorbidities was 25.9% (N=7). 68.8% (N=11) of the males and 20.0% (N=2) of the females had at least one comorbidity in addition to the MDRTB infection. There were higher percentages of MDRTB patients presenting with hypertension, 23.1% (N=6), and cancer, 15.4% (N=4). Other comorbidities included diabetes mellitus, 11.5% (N=3), hepatitis B, 7.7% (N=2), anemia, 7.7% (N=2), and miscellaneous- grouped diseases, 38.5% (N=10). Out of the 13 patients with comorbidities, hypertension counts 46.2% (N=6). Interpretation: This study suggests that patients infected with MDRTB at Taipei Hospital tend to be male (68.8%) over female (20.0%) and altogether have a higher probability of having comorbidities (38.5%). Among the MDRTB patients with comorbidities, hypertension counts 46.2%, suggesting that hypertension is the most likely comorbidity in MDRTB patients received at Taipei Hospital. Because many anti-hypertension drugs are associated with serious toxicities, which can complicate the management of MDRTB, it is thus necessary to establish favorable treatment regimens for MDRTB patients with hypertension to improve patient outcomes.

Institution

(Cheng, Lin) Touro University California, College of Osteopathic Medicine, Vallejo, CA, United States (Chien) TIHTC Taipei Hospital, Ministry of Health and Welfare, Taipei, Taiwan (Republic of China)

Publisher

Elsevier USA

Link to the Ovid Full Text or citation:

[Click here for full text options](http://ovidsp.ovid.com/ovidweb.cgi?T=JS&CSC=Y&NEWS=N&PAGE=fulltext&D=emed18b&AN=614045107)

Link to the External Link Resolver:

[SFX](http://whel-primo.hosted.exlibrisgroup.com/openurl/44WHELF_CAR/44WHELF_CAR_services_page?sid=OVID:embase&id=pmid:&id=doi:&issn=2214-9996&isbn=&volume=82&issue=3&spage=334&pages=334-335&date=2016&title=Annals+of+Global+Health&atitle=Trends+of+comorbidities+in+taiwanese+patients+infected+with+multi-drug+resistant+tuberculosis+in+seeking+favorable+treatment+outcomes&aulast=Cheng&pid=<author>Cheng+S.%3BLin+A.%3BChien+L.C.<%2Fauthor><AN>614045107<%2FAN><DT>Journal%3A+Conference+Abstract<%2FDT>)

20.

Cost and cost-effectiveness of tuberculosis treatment shortening: A model-based analysis.

Gomez G.B., Dowdy D.W., Bastos M.L., Zwerling A., Sweeney S., Foster N., Trajman A., Islam M.A., Kapiga S., Sinanovic E., Knight G.M., White R.G., Wells W.A., Cobelens F.G., Vassall A.

BMC Infectious Diseases. 16 (1) (no pagination), 2016. Article Number: 726. Date of Publication: 01 Dec 2016.

AN: 613465606

Background: Despite improvements in treatment success rates for tuberculosis (TB), current six-month regimen duration remains a challenge for many National TB Programmes, health systems, and patients. There is increasing investment in the development of shortened regimens with a number of candidates in phase 3 trials. Methods: We developed an individual-based decision analytic model to assess the cost-effectiveness of a hypothetical four-month regimen for first-line treatment of TB, assuming non-inferiority to current regimens of six-month duration. The model was populated using extensive, empirically-collected data to estimate the economic impact on both health systems and patients of regimen shortening for first-line TB treatment in South Africa, Brazil, Bangladesh, and Tanzania. We explicitly considered 'real world' constraints such as sub-optimal guideline adherence. Results: From a societal perspective, a shortened regimen, priced at USD1 per day, could be a cost-saving option in South Africa, Brazil, and Tanzania, but would not be cost-effective in Bangladesh when compared to one gross domestic product (GDP) per capita. Incorporating 'real world' constraints reduces cost-effectiveness. Patient-incurred costs could be reduced in all settings. From a health service perspective, increased drug costs need to be balanced against decreased delivery costs. The new regimen would remain a cost-effective option, when compared to each countries' GDP per capita, even if new drugs cost up to USD7.5 and USD53.8 per day in South Africa and Brazil; this threshold was above USD1 in Tanzania and under USD1 in Bangladesh. Conclusion: Reducing the duration of first-line TB treatment has the potential for substantial economic gains from a patient perspective. The potential economic gains for health services may also be important, but will be context-specific and dependent on the appropriate pricing of any new regimen. Copyright © 2016 The Author(s).

Institution

(Gomez, Cobelens) Academic Medical Center, University of Amsterdam, Amsterdam Institute for Global Health and Development and Department of Global Health, Trinity Building C, Pietersbergweg 17, Amsterdam 1105 BM, Netherlands (Gomez, Sweeney, Vassall) London School of Hygiene and Tropical Medicine, Department of Global Health and Development, London, United Kingdom

(Dowdy, Zwerling) Johns Hopkins Bloomberg School of Public Health, Department of Epidemiology, Baltimore, United States

(Bastos, Trajman) Federal University of Rio de Janeiro, Rio de Janeiro, Brazil

(Bastos, Trajman) Tuberculosis Scientific League, Rio de Janeiro, Brazil

(Foster, Sinanovic) Health Economics Unit, School of Public Health and Family Medicine, University of Cape Town, Cape Town, South Africa

(Trajman) McGill University, Montreal, Canada

(Islam) BRAC Health Nutrition and Population Programme, BRAC Centre, Dhaka, Bangladesh

(Kapiga) Mwanza Intervention Trials Unit, National Institute for Medical Research, Mwanza, Tanzania

(Knight, White) London School of Hygiene and Tropical Medicine, TB Modelling Group, Department of Infectious Disease Epidemiology, London, United Kingdom

(Wells) Global Alliance for TB Drug Development, NY, United States

(Cobelens) KNCV Tuberculosis Foundation, The Hague, Netherlands

(Wells) United States Agency for International Development, Washington, DC, United States

Publisher

BioMed Central Ltd. (E-mail: info@biomedcentral.com)

Link to the Ovid Full Text or citation:

[Click here for full text options](http://ovidsp.ovid.com/ovidweb.cgi?T=JS&CSC=Y&NEWS=N&PAGE=fulltext&D=emed18b&AN=613465606)

Link to the External Link Resolver:

[SFX](http://whel-primo.hosted.exlibrisgroup.com/openurl/44WHELF_CAR/44WHELF_CAR_services_page?sid=OVID:embase&id=pmid:&id=doi:10.1186%2Fs12879-016-2064-3&issn=1471-2334&isbn=&volume=16&issue=1&spage=no+pagination&pages=no+pagination&date=2016&title=BMC+Infectious+Diseases&atitle=Cost+and+cost-effectiveness+of+tuberculosis+treatment+shortening%3A+A+model-based+analysis&aulast=Gomez&pid=<author>Gomez+G.B.%3BDowdy+D.W.%3BBastos+M.L.%3BZwerling+A.%3BSweeney+S.%3BFoster+N.%3BTrajman+A.%3BIslam+M.A.%3BKapiga+S.%3BSinanovic+E.%3BKnight+G.M.%3BWhite+R.G.%3BWells+W.A.%3BCobelens+F.G.%3BVassall+A.<%2Fauthor><AN>613465606<%2FAN><DT>Journal%3A+Article<%2FDT>)

21.

Clinical effectiveness and cost-effectiveness of use of therapeutic monitoring of tumour necrosis factor alpha (TNF-alpha) inhibitors [LISA-TRACKER enzyme-linked immunosorbent assay (ELISA) kits, TNF-alpha-Blocker ELISA kits and Promonitor ELISA kits] versus standard care in patients with Crohn's disease: Systematic reviews and economic modelling.

Freeman K., Connock M., Auguste P., Taylor-Phillips S., Mistry H., Shyangdan D., Court R., Arasaradnam R., Sutcliffe P., Clarke A.

Health Technology Assessment. 20 (83) (no pagination), 2016. Date of Publication: November 2016.

AN: 613569956

Background and objectives: Systematic reviews and economic modelling of clinical effectiveness and cost-effectiveness of therapeutic monitoring of tumour necrosis factor alpha (TNF-alpha) inhibitors [using LISA-TRACKER enzyme-linked immunosorbent assay (ELISA) kits (Theradiag, Marne La Vallee France, or Alpha Laboratories, Heriot, UK), TNF-alpha-Blocker ELISA kits (Immundiagnostik AG, Bensheim, Germany) and Promonitor ELISA kits (Proteomika, Progenika Biopharma, Bizkaia, Spain)] versus standard care for Crohn's disease (CD). Methods: Multiple electronic databases were searched from inception to December 2014 in order to identify primary studies and meta-analyses. Population: Patients with moderate to severe active CD treated with infliximab (IFX) (Remicade,Merck Sharp & Dohme Ltd, Kenilworth, NJ, USA) or adalimumab (ADA) (Humira, AbbVie Inc., North Chicago, IL, USA). Intervention: Monitoring of serum anti-TNF-alpha (IFX or ADA) and/or of anti-drug antibody levels using test assays with a test-treatment algorithm. Comparator: Standard care. Outcomes: Any patient-related outcome, test agreement and cost-effectiveness estimates. The quality assessments used recognised checklists (Quality Assessment of Diagnostic Accuracy Studies-2, Cochrane, Philips and Consolidated Health Economic Evaluation Reporting Standards). Evidence was synthesised using narrative review and meta-analysis. A Markov model was built in TreeAge Pro 2013 (TreeA Software, Inc., Williamstown, MA, USA). The model had a 4-week cycle and a 10-year time horizon, adopted a NHS and Personal Social Services perspective and used a linked evidence approach. Costs were adjusted to 2013/14 prices and discounted at 3.5%. Results: We included 68 out of 2434 and 4 out of 2466 studies for the clinical effectiveness and cost-effectiveness reviews, respectively. Twenty-three studies comparing test methods were identified. Evidence on test concordance was sparse and contradictory, offering scant data for a linked evidence approach. Three studies [two randomised controlled trials (RCTs) and one retrospective observational study] investigated outcomes following implementation of a test algorithm. None used the specified commercial ELISA immunoassay test kits. Neither of the two RCTs demonstrated clinical benefit of a test-treatment regimen. A meta-analysis of 31 studies to estimate test accuracy for predicting clinical status indicated that 20-30% of test results are likely to be inaccurate. The four cost-effectiveness studies suggested that testing results in small cost reductions. In the economic analysis the base-case analysis showed that standard practice (no testing/therapeutic monitoring with the intervention tests) was more costly and more effective than testing for IFX. Sensitivity and scenario analyses gave similar results. The probabilistic sensitivity analysis indicated a 92% likelihood that the 'no-testing' strategy was cost-effective at a willingness to pay of 20,000 per quality-adjusted life-year. Strengths and limitations: Rigorous systematic reviews were undertaken; however, the underlying evidence base was poor or lacking. There was uncertainty about a linked evidence approach and a lack of gold standard for assay comparison. The only comparative evidence available for economic evaluation was for assays other than the intervention assays. Conclusions: Our finding that testing is not cost-effective for IFX should be viewed cautiously in view of the limited evidence. Clinicians should be mindful of variation in performance of different assays and of the absence of standardised approaches to patient assessment and treatment algorithms. Copyright © Queen's Printer and Controller of HMSO 2016.

Institution

(Freeman, Connock, Auguste, Taylor-Phillips, Mistry, Shyangdan, Court, Sutcliffe, Clarke) Warwick Evidence, Division of Health Sciences, Warwick Medical School, University of Warwick, Coventry, United Kingdom (Arasaradnam) Clinical Sciences Research Institute, University of Warwick, University Hospitals Coventry and Warwickshire NHS Trust, Coventry, United Kingdom

Publisher

NIHR Journals Library

Link to the Ovid Full Text or citation:

[Click here for full text options](http://ovidsp.ovid.com/ovidweb.cgi?T=JS&CSC=Y&NEWS=N&PAGE=fulltext&D=emed18b&AN=613569956)

Link to the External Link Resolver:

[SFX](http://whel-primo.hosted.exlibrisgroup.com/openurl/44WHELF_CAR/44WHELF_CAR_services_page?sid=OVID:embase&id=pmid:&id=doi:10.3310%2Fhta20830&issn=1366-5278&isbn=&volume=20&issue=83&spage=no+pagination&pages=no+pagination&date=2016&title=Health+Technology+Assessment&atitle=Clinical+effectiveness+and+cost-effectiveness+of+use+of+therapeutic+monitoring+of+tumour+necrosis+factor+alpha+(TNF-alpha)+inhibitors+%5BLISA-TRACKER+enzyme-linked+immunosorbent+assay+(ELISA)+kits%2C+TNF-alpha-Blocker+ELISA+kits+and+Promonitor+ELISA+kits%5D+versus+standard+care+in+patients+with+Crohn's+disease%3A+Systematic+reviews+and+economic+modelling&aulast=Freeman&pid=<author>Freeman+K.%3BConnock+M.%3BAuguste+P.%3BTaylor-Phillips+S.%3BMistry+H.%3BShyangdan+D.%3BCourt+R.%3BArasaradnam+R.%3BSutcliffe+P.%3BClarke+A.<%2Fauthor><AN>613569956<%2FAN><DT>Journal%3A+Article<%2FDT>)

22.

Cohort profile: The diabetes-tuberculosis treatment outcome (DITTO) study in Pakistan.

Mukhtar F., Butt Z.A.

BMJ Open. 6 (12) (no pagination), 2016. Article Number: e012970. Date of Publication: 01 Dec 2016.

AN: 613514830

Purpose: Pakistan is faced with an increasing prevalence of diabetes in addition to its existing high burden of tuberculosis (TB). Diabetes has a detrimental effect on treatment outcomes of patients with TB, which may hinder achieving the goals of the End-TB strategy by 2030. We conducted a prospective cohort study to determine difference between treatment outcomes among patients with diabetes and new pulmonary tuberculosis (PTB) and patients without diabetes and new PTB. This would help generate contextual and valid scientific evidence from a developing country like Pakistan with its unique interplay of sociocultural, economic and health system factors to inform policy and practice. Participants: This paper outlines the baseline characteristics of 614 new cases of PTB, aged 15 years and older, which were followed up prospectively at 2nd, 5th and 6th months while on antituberculosis treatment and at 6 months after treatment completion. Findings to date: We ascertained patients' diabetic status by conducting random and fasting blood glucose tests and their glycaemic control by determining glycosylated haemoglobin. Treatment outcomes were established using standardised definitions provided by WHO. The assessment of 614 respondents' diabetic status revealed that 113 (18%) were diabetic and 501 (82%) were nondiabetic. A greater proportion of patients with diabetes and PTB were illiterate (n=74/113, 65.5%) as compared to patients without diabetes and PTB (n=249/501, 50%) ( p=0.035). More patients with diabetes and PTB gave a history of heart disease (n=14/113, 12%) and hypertension (n=26/113, 23%) as compared to patients without diabetes and PTB (n=2/501, 0.4% (heart disease) and n=13 501, 3% (hypertension)) ( p<0.001). Unfavourable treatment outcome was more likely among patients with diabetes and PTB (n=23/93, 25%) as opposed to patients without diabetes and PTB (n=46/410, 11%) (p=0.001). Future plans: We are negotiating with the government regarding funding for a further 2-year follow-up of the cohort to ascertain death and relapse in the post-treatment period and also differentiate between re-infection and recurrence among these patients with respect to their diabetic status.

Institution

(Mukhtar) Health Services Academy, Islamabad, Pakistan (Mukhtar) Department of Community Medicine, Lahore Medical and Dental College, Lahore, Pakistan

(Butt) Department of Epidemiology and Biostatistics, Health Services Academy, Islamabad, Pakistan

(Butt) School of Population and Public Health, University of British Columbia, Vancouver, BC, Canada

Publisher

BMJ Publishing Group (E-mail: subscriptions@bmjgroup.com)

Link to the Ovid Full Text or citation:

[Click here for full text options](http://ovidsp.ovid.com/ovidweb.cgi?T=JS&CSC=Y&NEWS=N&PAGE=fulltext&D=emed18b&AN=613514830)

Link to the External Link Resolver:

[SFX](http://whel-primo.hosted.exlibrisgroup.com/openurl/44WHELF_CAR/44WHELF_CAR_services_page?sid=OVID:embase&id=pmid:&id=doi:10.1136%2Fbmjopen-2016-012970&issn=2044-6055&isbn=&volume=6&issue=12&spage=no+pagination&pages=no+pagination&date=2016&title=BMJ+Open&atitle=Cohort+profile%3A+The+diabetes-tuberculosis+treatment+outcome+(DITTO)+study+in+Pakistan&aulast=Mukhtar&pid=<author>Mukhtar+F.%3BButt+Z.A.<%2Fauthor><AN>613514830<%2FAN><DT>Journal%3A+Article<%2FDT>)

23.

Effectiveness and safety of imipenem/clavulanate and linezolid to treat multidrug and extensively drug-resistant tuberculosis at a referral hospital in brazil.

Arbex M.A., Bonini E.H., Kawakame Pirolla G., D'Ambrosio L., Centis R., Migliori G.B.

Revista Portuguesa de Pneumologia. 22 (6) (pp 337-341), 2016. Date of Publication: 01 Nov 2016.

AN: 613727328

Evidence on effectiveness, safety, and tolerability of imipenem/clavulanate (IC) and linezolid containing regimens to treat multidrug-resistant (MDR-) and extensively drug-resistant tuberculosis (XDR-TB) is scarce. The aim of this observational study is to evaluate the therapeutic contribution of IC and linezolid to manage MDR/XDR-TB cases at the reference centre of Sao Paulo state, Brazil. Twelve patients (9 males, 1 HIV positive in antiretroviral treatment, 4 MDR, 8 XDR) were treated with IC, 11 of them within linezolid-containing regimens. They all were previously treated with treatment failure, for a median (IQR, interquartile range) of 4.5 (2-6.5) times, having a severe resistance pattern (median number of resistances: 7 (5-8)) and being sputum smear and culture positive. IC and linezolid were prescribed at the dose of 1000 mg/day and 600 mg/day, respectively. The overall exposure was (median (IQR)) 419 (375.5-658) days for IC and 678 (392-720) days for linezolid. All of them converted their sputum (time to sputum conversion; 60 (37.5-90) days) and culture (75 (60-135) days), and 7 were cured while 5 are still on treatment with a gradually improving clinical picture. While no adverse events were reported for IC, 2 minor side effects, only, were attributed to linezolid (17%); in both cases the drug was re-started without further problems. Our study suggests that IC and linezolid-containing regimens can be used safely and with satisfactory outcomes in reference centres to treat MDR/XDR-TB patients. Copyright © 2016 Sociedade Portuguesa de Pneumologia.

Institution

(Arbex, Bonini) University Center of Araraquara, Sao Paulo, Brazil (Arbex, Bonini, Kawakame Pirolla) Hospital Nestor Goulart Reis, Sao Paulo State Secretary of Health, Sao Paulo, Brazil

(D'Ambrosio, Centis, Migliori) WHO Collaborating Centre for TB and Lung Diseases, Fondazione S. Maugeri, Tradate, Italy

(D'Ambrosio) Public Health Consulting Group, Lugano, Switzerland

Publisher

Elsevier Doyma (E-mail: editorial@elsevier.com)

Link to the Ovid Full Text or citation:

[Click here for full text options](http://ovidsp.ovid.com/ovidweb.cgi?T=JS&CSC=Y&NEWS=N&PAGE=fulltext&D=emed18b&AN=613727328)

Link to the External Link Resolver:

[SFX](http://whel-primo.hosted.exlibrisgroup.com/openurl/44WHELF_CAR/44WHELF_CAR_services_page?sid=OVID:embase&id=pmid:&id=doi:10.1016%2Fj.rppnen.2016.06.006&issn=2173-5115&isbn=&volume=22&issue=6&spage=337&pages=337-341&date=2016&title=Revista+Portuguesa+de+Pneumologia&atitle=Effectiveness+and+safety+of+imipenem%2Fclavulanate+and+linezolid+to+treat+multidrug+and+extensively+drug-resistant+tuberculosis+at+a+referral+hospital+in+brazil&aulast=Arbex&pid=<author>Arbex+M.A.%3BBonini+E.H.%3BKawakame+Pirolla+G.%3BD'Ambrosio+L.%3BCentis+R.%3BMigliori+G.B.<%2Fauthor><AN>613727328<%2FAN><DT>Journal%3A+Article<%2FDT>)

24.

Clinical predictors of aminoglycoside-induced ototoxicity in drug-resistant Tuberculosis patients on intensive therapy.

Sogebi O.A., Adefuye B.O., Adebola S.O., Oladeji S.M., Adedeji T.O.

Auris Nasus Larynx. (no pagination), 2016. Date of Publication: July 21, 2016.

AN: 613767631

Objective: The study objectives were to determine the incidence of aminoglycoside-induced ototoxicity in institutionalized patients on intensive phase of therapy for drug-resistant Tuberculosis (DR Tb) and also to assess clinical factors which could predict the ototoxicity. Methods: The study was a prospective analytical study among consecutive DR Tb patients who were admitted for intensive phase of therapy (of 4 months) at the DR-Tb center over a 12-month period. Patients were diagnosed as DR Tb using the Gene Xpert machine to confirm Rifampicin resistance. All eligible 70 out of 87 consenting patients were consecutively recruited into the study. Patients had baseline (admission) and serial pure tone audiometries (PTAs) performed at 4 weekly intervals until discharge after 4 months of admission. Audiometric confirmation of aminoglycoside-induced ototoxicity was done by comparing serial with baseline PTA. Results: Among the 70 patients the male:female ratio was 1.7:1. Nine patients (12.9%) were retroviral-positive, and 16 patients (22.9%) were confirmed to have ototoxicity by audiometric criteria. The duration of treatment when ototoxicity was detected in the patients ranged 4-17 (Mean. +/-. SD; 9.4. +/-. 3.4) weeks. Ototoxicity was detected in the audiometric low frequency ranges in 7 (43.8%) and at the high frequencies in 4 (25.0%) of the patients. Univariate analyses of clinical parameters found that age, underlying diabetes mellitus, deranged baseline PTAv >25. dB HL, BMI on admission and retroviral status were significantly associated, while sex and previous drug regimen failure were not associated with ototoxicity. Multivariate adjusted logistic regression analyses, controlling for sex, revealed age (OR. = 1.068, . p = . 0.018), BMI on admission (OR. = 0.673, . p = . 0.012) and retroviral positivity (OR. = 8.822, . p = . 0.014) of patients could significantly predict aminoglycoside-induced ototoxicity. Conclusion: Incidence of aminoglycoside-induced ototoxicity in DR Tb patients was 22.9%. The clinical predictors for ototoxicity were age, BMI on admission, and co-existing retroviral infection in the patients. Clinicians should consider these factors in making choices of aminoglycosides to be used during intensive phase of treatment with second line anti-Tuberculous therapy. Copyright © 2016 Elsevier Ireland Ltd.

Institution

(Sogebi) ENT Unit, Department of Surgery, Faculty of Clinical Sciences, Olabisi Onabanjo University, Sagamu, Nigeria (Adefuye) Department of Medicine, Faculty of Clinical Sciences, Olabisi Onabanjo University, Sagamu, Nigeria

(Adebola) Department of Otorhinolaryngology, Ladoke Akintola University of Technology (LAUTECH) Teaching Hospital, Ogbomoso, Nigeria

(Oladeji) ENT Unit, Department of Surgery, Babcock University Teaching Hospital, Ilishan, Nigeria

(Adedeji) Department of Otorhinolaryngology, Ladoke Akintola University of Technology (LAUTECH) Teaching Hospital, Osogbo, Nigeria

Publisher

Elsevier Ireland Ltd

Link to the Ovid Full Text or citation:

[Click here for full text options](http://ovidsp.ovid.com/ovidweb.cgi?T=JS&CSC=Y&NEWS=N&PAGE=fulltext&D=emed18b&AN=613767631)

Link to the External Link Resolver:

[SFX](http://whel-primo.hosted.exlibrisgroup.com/openurl/44WHELF_CAR/44WHELF_CAR_services_page?sid=OVID:embase&id=pmid:&id=doi:10.1016%2Fj.anl.2016.10.005&issn=0385-8146&isbn=&volume=&issue=&spage=no+pagination&pages=no+pagination&date=2016&title=Auris+Nasus+Larynx&atitle=Clinical+predictors+of+aminoglycoside-induced+ototoxicity+in+drug-resistant+Tuberculosis+patients+on+intensive+therapy&aulast=Sogebi&pid=<author>Sogebi+O.A.%3BAdefuye+B.O.%3BAdebola+S.O.%3BOladeji+S.M.%3BAdedeji+T.O.<%2Fauthor><AN>613767631<%2FAN><DT>Journal%3A+Article+In+Press<%2FDT>)

25.

Educational Outreach with an Integrated Clinical Tool for Nurse-Led Non-communicable Chronic Disease Management in Primary Care in South Africa: A Pragmatic Cluster Randomised Controlled Trial.

Fairall L.R., Folb N., Timmerman V., Lombard C., Steyn K., Bachmann M.O., Bateman E.D., Lund C., Cornick R., Faris G., Gaziano T., Georgeu-Pepper D., Zwarenstein M., Levitt N.S.

PLoS Medicine. 13 (11) (no pagination), 2016. Article Number: e1002178. Date of Publication: November 2016.

AN: 613530964

Background: In many low-income countries, care for patients with non-communicable diseases (NCDs) and mental health conditions is provided by nurses. The benefits of nurse substitution and supplementation in NCD care in high-income settings are well recognised, but evidence from low- and middle-income countries is limited. Primary Care 101 (PC101) is a programme designed to support and expand nurses' role in NCD care, comprising educational outreach to nurses and a clinical management tool with enhanced prescribing provisions. We evaluated the effect of the programme on primary care nurses' capacity to manage NCDs. Methods and Findings: In a cluster randomised controlled trial design, 38 public sector primary care clinics in the Western Cape Province, South Africa, were randomised. Nurses in the intervention clinics were trained to use the PC101 management tool during educational outreach sessions delivered by health department trainers and were authorised to prescribe an expanded range of drugs for several NCDs. Control clinics continued use of the Practical Approach to Lung Health and HIV/AIDS in South Africa (PALSA PLUS) management tool and usual training. Patients attending these clinics with one or more of hypertension (3,227), diabetes (1,842), chronic respiratory disease (1,157) or who screened positive for depression (2,466), totalling 4,393 patients, were enrolled between 28 March 2011 and 10 November 2011. Primary outcomes were treatment intensification in the hypertension, diabetes, and chronic respiratory disease cohorts, defined as the proportion of patients in whom treatment was escalated during follow-up over 14 mo, and case detection in the depression cohort. Primary outcome data were analysed for 2,110 (97%) intervention and 2,170 (97%) control group patients. Treatment intensification rates in intervention clinics were not superior to those in the control clinics (hypertension: 44% in the intervention group versus 40% in the control group, risk ratio [RR] 1.08 [95% CI 0.94 to 1.24; p = 0.252]; diabetes: 57% versus 50%, RR 1.10 [0.97 to 1.24; p = 0.126]; chronic respiratory disease: 14% versus 12%, RR 1.08 [0.75 to 1.55; p = 0.674]), nor was case detection of depression (18% versus 24%, RR 0.76 [0.53 to 1.10; p = 0.142]). No adverse effects of the nurses' expanded scope of practice were observed. Limitations of the study include dependence on self-reported diagnoses for inclusion in the patient cohorts, limited data on uptake of PC101 by users, reliance on process outcomes, and insufficient resources to measure important health outcomes, such as HbA1c, at follow-up. Conclusions: Educational outreach to primary care nurses to train them in the use of a management tool involving an expanded role in managing NCDs was feasible and safe but was not associated with treatment intensification or improved case detection for index diseases. This notwithstanding, the intervention, with adjustments to improve its effectiveness, has been adopted for implementation in primary care clinics throughout South Africa. Trial Registration: The trial is registered with Current Controlled Trials (ISRCTN20283604) Copyright © 2016 Fairall et al.

Author NameID

Timmerman, Venessa; ORCID: <http://orcid.org/0000-0002-6172-9386> Cornick, Ruth; ORCID: <http://orcid.org/0000-0002-7391-3383>

Folb, Naomi; ORCID: <http://orcid.org/0000-0002-2667-0956>

Institution

(Fairall, Folb, Timmerman, Bateman, Cornick, Faris, Georgeu-Pepper) Knowledge Translation Unit, University of Cape Town Lung Institute, Cape Town, South Africa (Fairall, Folb, Steyn, Bateman, Levitt) Department of Medicine, University of Cape Town, Cape Town, South Africa

(Fairall, Folb, Steyn, Gaziano, Levitt) Chronic Disease Initiative for Africa, Department of Medicine, University of Cape Town, Cape Town, South Africa

(Lombard) Biostatistics Unit, Medical Research Council, Cape Town, South Africa

(Bachmann) Norwich Medical School, University of East Anglia, Norwich, United Kingdom

(Lund) Alan J Flisher Centre for Public Mental Health, Department of Psychiatry and Mental Health, University of Cape Town, Cape Town, South Africa

(Gaziano) Division of Cardiovascular Medicine, Brigham & Women's Hospital, Boston, MA, United States

(Zwarenstein) Centre for Studies in Family Medicine, Department of Family Medicine, Schulich School of Medicine and Dentistry, Western University, London, ON, Canada

Publisher

Public Library of Science (E-mail: plos@plos.org)

Link to the Ovid Full Text or citation:

[Click here for full text options](http://ovidsp.ovid.com/ovidweb.cgi?T=JS&CSC=Y&NEWS=N&PAGE=fulltext&D=emed18b&AN=613530964)

Link to the External Link Resolver:

[SFX](http://whel-primo.hosted.exlibrisgroup.com/openurl/44WHELF_CAR/44WHELF_CAR_services_page?sid=OVID:embase&id=pmid:&id=doi:10.1371%2Fjournal.pmed.1002178&issn=1549-1277&isbn=&volume=13&issue=11&spage=no+pagination&pages=no+pagination&date=2016&title=PLoS+Medicine&atitle=Educational+Outreach+with+an+Integrated+Clinical+Tool+for+Nurse-Led+Non-communicable+Chronic+Disease+Management+in+Primary+Care+in+South+Africa%3A+A+Pragmatic+Cluster+Randomised+Controlled+Trial&aulast=Fairall&pid=<author>Fairall+L.R.%3BFolb+N.%3BTimmerman+V.%3BLombard+C.%3BSteyn+K.%3BBachmann+M.O.%3BBateman+E.D.%3BLund+C.%3BCornick+R.%3BFaris+G.%3BGaziano+T.%3BGeorgeu-Pepper+D.%3BZwarenstein+M.%3BLevitt+N.S.<%2Fauthor><AN>613530964<%2FAN><DT>Journal%3A+Article<%2FDT>)

26.

Preventing acquired resistance to bedaquiline and delamanid in multidrug-resistant tuberculosis treatment requires optimal management.

Guglielmetti L., Du D.L., Frechet-Jachym M., Mitnick C.

American Journal of Respiratory and Critical Care Medicine. 194 (9) (pp 1170-1171), 2016. Date of Publication: 01 Nov 2016.

AN: 613234032

Author NameID

Guglielmetti, Lorenzo; ORCID: <http://orcid.org/0000-0003-0886-9635>

Institution

(Guglielmetti, Du, Frechet-Jachym) Centre Hospitalier de Bligny Briis-sous-Forges, France (Guglielmetti) Pierre and Marie Curie University, Paris, France

(Guglielmetti) INSERM U1135, Paris, France

(Mitnick) Harvard Medical School, Boston, MA, United States

(Mitnick) Partners in Health, Boston, MA, United States

(Mitnick) Brigham and Women's Hospital, Boston, MA, United States

Publisher

American Thoracic Society (E-mail: malexander@thoracic.org)

Link to the Ovid Full Text or citation:

[Click here for full text options](http://ovidsp.ovid.com/ovidweb.cgi?T=JS&CSC=Y&NEWS=N&PAGE=fulltext&D=emed18b&AN=613234032)

Link to the External Link Resolver:

[SFX](http://whel-primo.hosted.exlibrisgroup.com/openurl/44WHELF_CAR/44WHELF_CAR_services_page?sid=OVID:embase&id=pmid:&id=doi:10.1164%2Frccm.201604-0841LE&issn=1073-449X&isbn=&volume=194&issue=9&spage=1170&pages=1170-1171&date=2016&title=American+Journal+of+Respiratory+and+Critical+Care+Medicine&atitle=Preventing+acquired+resistance+to+bedaquiline+and+delamanid+in+multidrug-resistant+tuberculosis+treatment+requires+optimal+management&aulast=Guglielmetti&pid=<author>Guglielmetti+L.%3BDu+D.L.%3BFrechet-Jachym+M.%3BMitnick+C.<%2Fauthor><AN>613234032<%2FAN><DT>Journal%3A+Letter<%2FDT>)

27.

Longitudinal assessment of health related quality of life of HIV infected patients treated for tuberculosis and HIV in a high burden setting.

Mthiyane T., Pym A., Dheda K., Rustomjee R., Reddy T., Manie S.

Quality of Life Research. 25 (12) (pp 3067-3076), 2016. Date of Publication: 01 Dec 2016.

AN: 610774932

Introduction: Assessment of patients receiving treatment for human immunodeficiency virus (HIV) and tuberculosis (TB) using a Health Related Quality of Life (HRQoL) instrument is important to get the subjective view of the patients' wellbeing. Methods: We used the Functional Assessment of HIV Infection (FAHI) HRQoL instrument to collect perceived wellness information at baseline, month 3, 6 and 12 from patients enrolled in a pharmacokinetic study between March 2007 and April 2008. Composite domain scores at each time point and their relationship with the rate of adverse events (AEs) and serious adverse events were compared between treatment arms. Results: Out of the 82 patients enrolled, 76 were analysed. There was a significant increase in total score in all groups between baseline, month 3, 6 and 12 (all p values < 0.0001), and over time (p < 0.001). Adjusting for baseline total score, baseline CD4 count had a significant effect on the total score over time (p = 0.002) and the rate of change in total score over time, that is; interaction effect (p < 0.001). There was no difference in each domain scores between participants that received ART with TB treatment and those that received TB treatment only. Respiratory AEs had a significant effect on HRQoL. Conclusion: We found that assessment of HRQoL of participants in TB-HIV treatment using the FAHI instrument was useful in evaluating treatment responses. It showed improvement consistent with decrease in adverse events and signs and symptoms of TB. Number and type of AEs was related to lower HRQoL in spite of TB cure. Copyright © 2016, Springer International Publishing Switzerland.

Author NameID

Mthiyane, Thuli; ORCID: <http://orcid.org/0000-0002-1782-5485>

Institution

(Mthiyane, Rustomjee) South African Medical Research Council, Durban, South Africa (Reddy) Biostatistics Unit, South African Medical Research Council, Durban, South Africa

(Dheda) Division of Pulmonology, Department of Medicine, University of Cape Town, Cape Town, South Africa

(Pym) KZN Research Institute for Tuberculosis and HIV, Durban, South Africa

(Manie) Division of Physiotherapy, Department of Health and Rehabilitation Sciences, University of Cape Town, Cape Town, South Africa

Publisher

Springer International Publishing

Link to the Ovid Full Text or citation:

[Click here for full text options](http://ovidsp.ovid.com/ovidweb.cgi?T=JS&CSC=Y&NEWS=N&PAGE=fulltext&D=emed18b&AN=610774932)

Link to the External Link Resolver:

[SFX](http://whel-primo.hosted.exlibrisgroup.com/openurl/44WHELF_CAR/44WHELF_CAR_services_page?sid=OVID:embase&id=pmid:&id=doi:10.1007%2Fs11136-016-1332-4&issn=0962-9343&isbn=&volume=25&issue=12&spage=3067&pages=3067-3076&date=2016&title=Quality+of+Life+Research&atitle=Longitudinal+assessment+of+health+related+quality+of+life+of+HIV+infected+patients+treated+for+tuberculosis+and+HIV+in+a+high+burden+setting&aulast=Mthiyane&pid=<author>Mthiyane+T.%3BPym+A.%3BDheda+K.%3BRustomjee+R.%3BReddy+T.%3BManie+S.<%2Fauthor><AN>610774932<%2FAN><DT>Journal%3A+Article<%2FDT>)

28.

Outcomes of radical debridement versus no debridement for the treatment of thoracic and lumbar spinal tuberculosis.

Qian J., Rijiepu A., Zhu B., Tian D., Chen L., Jing J.

International Orthopaedics. 40 (10) (pp 2081-2088), 2016. Date of Publication: 01 Oct 2016.

AN: 610987115

Purpose: Whether radical debridement is necessary for the treatment of thoracic and lumbar tuberculosis is still questionable. The objective of this prospective randomized study was to compare the outcomes of radical debridement versus no debridement for the treatment of thoracic and lumbar tuberculosis. Methods: Seventy-four thoracic and lumbar tuberculosis patients with a neurological function of grade D and E underwent surgery and received the same chemotherapy regiment from January 2009 to October 2014. All patients were divided into group A and B by taking the drawing of lots. In group A, radical debridement, bone graft, and instrumentation were performed. Isolated posterior instrumentation without debridement were performed in group B. The operative time, blood loss, visual analogue score (VAS), erythrocyte sedimentation rate (ESR), kyphotic angle, Frankel grading, fusion rate, and complications were evaluated. Results: Group B had a better clinical outcome with regard to the operative time, blood loss, VAS score first week post-operatively, and the ESR value in the third and sixth month post-operatively than group A, and the differences between the two groups about those values all presented a significant difference (P < 0.05). However, no difference was observed between the two groups for the kyphotic angle (P = 0.088) and fusion rate (P = 0.164) at the final follow-up. Neurological function of all cases exhibited normal neurological function in the two groups at the final follow-up. Two cases of pulmonary infection and four cases of wound infection in group A. No serious complications were observed in group B. Conclusions: Isolated posterior instrumentation without debridement is a suitable treatment for selected patients because of minor surgical trauma, fewer complications, and spontaneous fusion. Copyright © 2016, SICOT aisbl.

Institution

(Qian, Zhu, Tian, Chen, Jing) Department of Orthopaedics, the Second Hospital of AnHui Medical University, Hefei, Anhui 230601, China (Rijiepu) Department of Orthopaedics, Hetian Area Hospital, Hetian, Xinjiang, China

Publisher

Springer Verlag (E-mail: service@springer.de)

Link to the Ovid Full Text or citation:

[Click here for full text options](http://ovidsp.ovid.com/ovidweb.cgi?T=JS&CSC=Y&NEWS=N&PAGE=fulltext&D=emed18b&AN=610987115)

Link to the External Link Resolver:

[SFX](http://whel-primo.hosted.exlibrisgroup.com/openurl/44WHELF_CAR/44WHELF_CAR_services_page?sid=OVID:embase&id=pmid:&id=doi:10.1007%2Fs00264-016-3234-z&issn=0341-2695&isbn=&volume=40&issue=10&spage=2081&pages=2081-2088&date=2016&title=International+Orthopaedics&atitle=Outcomes+of+radical+debridement+versus+no+debridement+for+the+treatment+of+thoracic+and+lumbar+spinal+tuberculosis&aulast=Qian&pid=<author>Qian+J.%3BRijiepu+A.%3BZhu+B.%3BTian+D.%3BChen+L.%3BJing+J.<%2Fauthor><AN>610987115<%2FAN><DT>Journal%3A+Article<%2FDT>)

29.

Statin non-adherence and residual cardiovascular risk: There is need for substantial improvement.

Banach M., Stulc T., Dent R., Toth P.P.

International Journal of Cardiology. 225 (pp 184-196), 2016. Date of Publication: 15 Dec 2016.

AN: 612522694

Although statin therapy has proven to be the cornerstone for prevention and treatment of cardiovascular disease (CVD), there are many patients for whom long-term therapy remains suboptimal. The aims of this article are to review the current complex issues associated with statin use and to explore when novel treatment approaches should be considered. Statin discontinuation as well as adherence to statin therapy remain two of the greatest challenges for lipidologists. Evidence suggests that between 40 and 75% of patients discontinue their statin therapy within one year after initiation. Furthermore, whilst the reasons for persistence with statin therapy are complex, evidence shows that low-adherence to statins negatively impacts clinical outcomes and residual CV risk remains a major concern. Non-adherence or lack of persistence with long-term statin therapy in real-life may be the main cause of inadequate low density lipoprotein cholesterol lowering with statins. There is a large need for the improvement of the use of statins, which have good safety profiles and are inexpensive. On the other hand, in a non-cost-constrained environment, proprotein convertase subtilisin/kexin type 9 inhibitors should arguably be used more often in those patients in whom treatment with statins remains unsatisfactory. Copyright © 2016 Elsevier Ireland Ltd

Author NameID

Banach, Maciej; ORCID: <http://orcid.org/0000-0001-6690-6874>

Institution

(Banach) Department of Hypertension, Chair of Nephrology and Hypertension, Medical University of Lodz, Lodz, Poland (Banach) Healthy Aging Research Centre, Medical University of Lodz, Lodz, Poland

(Banach) Polish Mother's Memorial Hospital Research Institute, Lodz, Poland

(Stulc) 3rd Department of Internal Medicine, 1st University of Medicine, Prague, Czech Republic

(Dent) Amgen (Europe) GmbH, Zug, Switzerland

(Toth) CGH Medical Center, Sterling, IL, United States

(Toth) Ciccarone Center for the Prevention of Cardiovascular Disease, Johns Hopkins University School of Medicine, Baltimore, MD, United States

Publisher

Elsevier Ireland Ltd

Link to the Ovid Full Text or citation:

[Click here for full text options](http://ovidsp.ovid.com/ovidweb.cgi?T=JS&CSC=Y&NEWS=N&PAGE=fulltext&D=emed18b&AN=612522694)

Link to the External Link Resolver:

[SFX](http://whel-primo.hosted.exlibrisgroup.com/openurl/44WHELF_CAR/44WHELF_CAR_services_page?sid=OVID:embase&id=pmid:&id=doi:10.1016%2Fj.ijcard.2016.09.075&issn=0167-5273&isbn=&volume=225&issue=&spage=184&pages=184-196&date=2016&title=International+Journal+of+Cardiology&atitle=Statin+non-adherence+and+residual+cardiovascular+risk%3A+There+is+need+for+substantial+improvement&aulast=Banach&pid=<author>Banach+M.%3BStulc+T.%3BDent+R.%3BToth+P.P.<%2Fauthor><AN>612522694<%2FAN><DT>Journal%3A+Review<%2FDT>)

30.

Current trends and intricacies in the management of HIV-associated pulmonary tuberculosis.

Gopalan N., Chandrasekaran P., Swaminathan S., Tripathy S.

AIDS Research and Therapy. 13 (1) (no pagination), 2016. Article Number: 34. Date of Publication: 26 Sep 2016.

AN: 612339406

Human immunodeficiency virus (HIV) epidemic has undoubtedly increased the incidence of tuberculosis (TB) globally, posing a formidable global health challenge affecting 1.2 million cases. Pulmonary TB assumes utmost significance in the programmatic perspective as it is readily transmissible as well as easily diagnosable. HIV complicates every aspect of pulmonary tuberculosis from diagnosis to treatment, demanding a different approach to effectively tackle both the diseases. In order to control these converging epidemics, it is important to diagnose early, initiate appropriate therapy for both infections, prevent transmission and administer preventive therapy. Liquid culture methods and nucleic acid amplification tests for TB confirmation have replaced conventional solid media, enabling quicker and simultaneous detection of mycobacterium and its drug sensitivity profile Unique problems posed by the syndemic include Acquired rifampicin resistance, drug-drug interactions, malabsorption of drugs and immune reconstitution inflammatory syndrome or paradoxical reaction that complicate dual and concomitant therapy. While the antiretroviral therapy armamentarium is constantly reinforced by discovery of newer and safer drugs every year, only a few drugs for anti tuberculosis treatment have successfully emerged. These include bedaquiline, delamanid and pretomanid which have entered phase III B trials and are also available through conditional access national programmes. The current guidelines by WHO to start Antiretroviral therapy irrespective of CD4+ cell count based on benefits cited by recent trials could go a long way in preventing various complications caused by the deadly duo. This review provides a consolidated gist of the advancements, concepts and updates that have emerged in the management of HIV-associated pulmonary TB for maximizing efficacy, offering latest solutions for tackling drug-drug interactions and remedial measures for immune reconstitution inflammatory syndrome. Copyright © 2016 The Author(s).

Institution

(Gopalan, Chandrasekaran, Swaminathan, Tripathy) National Institute for Research in Tuberculosis (Formerly Tuberculosis Research Centre), Division of HIV, No. 1, Mayor Sathyamoorthy Road, Chetpet, Chennai 600 031, India

Publisher

BioMed Central Ltd. (E-mail: info@biomedcentral.com)

Link to the Ovid Full Text or citation:

[Click here for full text options](http://ovidsp.ovid.com/ovidweb.cgi?T=JS&CSC=Y&NEWS=N&PAGE=fulltext&D=emed18b&AN=612339406)

Link to the External Link Resolver:

[SFX](http://whel-primo.hosted.exlibrisgroup.com/openurl/44WHELF_CAR/44WHELF_CAR_services_page?sid=OVID:embase&id=pmid:&id=doi:10.1186%2Fs12981-016-0118-7&issn=1742-6405&isbn=&volume=13&issue=1&spage=no+pagination&pages=no+pagination&date=2016&title=AIDS+Research+and+Therapy&atitle=Current+trends+and+intricacies+in+the+management+of+HIV-associated+pulmonary+tuberculosis&aulast=Gopalan&pid=<author>Gopalan+N.%3BChandrasekaran+P.%3BSwaminathan+S.%3BTripathy+S.<%2Fauthor><AN>612339406<%2FAN><DT>Journal%3A+Review<%2FDT>)

31.

Extensively Drug-Resistant Tuberculosis: Principles of Resistance, Diagnosis, and Management.

Wilson J.W., Tsukayama D.T.

Mayo Clinic Proceedings. 91 (4) (pp 482-495), 2016. Date of Publication: 01 Apr 2016.

AN: 608510141

Extensively drug-resistant (XDR) tuberculosis (TB) is an unfortunate by-product of mankind's medical and pharmaceutical ingenuity during the past 60 years. Although new drug developments have enabled TB to be more readily curable, inappropriate TB management has led to the emergence of drug-resistant disease. Extensively drug-resistant TB describes Mycobacterium tuberculosis that is collectively resistant to isoniazid, rifampin, a fluoroquinolone, and an injectable agent. It proliferates when established case management and infection control procedures are not followed. Optimized treatment outcomes necessitate time-sensitive diagnoses, along with expanded combinations and prolonged durations of antimicrobial drug therapy. The challenges to public health institutions are immense and most noteworthy in underresourced communities and in patients coinfected with human immunodeficiency virus. A comprehensive and multidisciplinary case management approach is required to optimize outcomes. We review the principles of TB drug resistance and the risk factors, diagnosis, and managerial approaches for extensively drug-resistant TB. Treatment outcomes, cost, and unresolved medical issues are also discussed. Copyright © 2016 Mayo Foundation for Medical Education and Research.

PMID

26906649 [<http://www.ncbi.nlm.nih.gov/pubmed/?term=26906649>]

Institution

(Wilson) Department of Medicine, Division of Infectious Diseases, Mayo Clinic, Rochester, MN, United States (Tsukayama) Division of Infectious Diseases and Internal Medicine, University of Minnesota, Hennepin County Medical Center, Minneapolis, MN, United States

Publisher

Elsevier Ltd

Link to the Ovid Full Text or citation:

[Click here for full text options](http://ovidsp.ovid.com/ovidweb.cgi?T=JS&CSC=Y&NEWS=N&PAGE=fulltext&D=emed18b&AN=608510141)

Link to the External Link Resolver:

[SFX](http://whel-primo.hosted.exlibrisgroup.com/openurl/44WHELF_CAR/44WHELF_CAR_services_page?sid=OVID:embase&id=pmid:26906649&id=doi:10.1016%2Fj.mayocp.2016.01.014&issn=0025-6196&isbn=&volume=91&issue=4&spage=482&pages=482-495&date=2016&title=Mayo+Clinic+Proceedings&atitle=Extensively+Drug-Resistant+Tuberculosis%3A+Principles+of+Resistance%2C+Diagnosis%2C+and+Management&aulast=Wilson&pid=<author>Wilson+J.W.%3BTsukayama+D.T.<%2Fauthor><AN>608510141<%2FAN><DT>Journal%3A+Article<%2FDT>)

32.

An integrated community TB-HIV adherence model provides an alternative to DOT for tuberculosis patients in Cape Town.

Kaplan R., Caldwell J., Hermans S., Adriaanse S., Mtwisha L., Bekker L.-G., Jennings K., Wood R.

International Journal of Tuberculosis and Lung Disease. 20 (9) (pp 1185-1191 and i), 2016. Date of Publication: 01 Sep 2016.

AN: 611736437

SETTING: Cape Town, South Africa. OBJECTIVE: To evaluate anti-tuberculosis treatment outcomes and rate of antiretroviral therapy (ART) initiation using weekly community-based adherence support compared to daily directly observed therapy (DOT). METHODS: This was a retrospective analysis comparing two cohorts treated for tuberculosis (TB) in 70 TB clinics during 6-month periods before and after the introduction of a new adherence model comprising treatment literacy sessions during 2 weeks of DOT, followed by weekly home visits by community care workers to eligible patients managing their own treatment. Odds ratios (ORs) of treatment success and ART initiation were calculated using multivariable random effects logistic regression models. Hazard ratios (HRs) of default and death were calculated using multivariable random effects Cox regression models. RESULTS: The pre-intervention cohort comprised 11 896 patients with TB and the post-intervention cohort 11 314. There was no difference in pre- and post-intervention anti-tuberculosis treatment success rates (respectively 82.8% and 82.5%, adjusted OR [aOR] 1.02, 95%CI 0.89-1.17, P = 0.76) nor an increased hazard of death (adjusted HR [aHR] 0.98, 95%CI 0.80-1.21, P = 0.87) or default (aHR 0.97, 95%CI 0.81-1.15, P = 0.69). The ART initiation rate increased from 67% to 74% (aOR 1.43; 95%CI 1.01-1.85, P < 0.01). CONCLUSION: Weekly community-based adherence support was a viable alternative to daily DOT, with no deterioration in anti-tuberculosis treatment outcomes and an increase in ART initiation. Copyright © 2016 The Union.

Institution

(Kaplan, Hermans, Mtwisha, Bekker, Wood) Desmond Tutu HIV Centre, Institute for Infectious Disease and Molecular Medicine, Faculty of Health Sciences, University of Cape Town, Anzio Road, Observatory 7925, South Africa (Caldwell, Adriaanse, Jennings) City Health, City of Cape Town, Cape Town, South Africa

(Hermans) Department of Global Health, Academic Medical Center, University of Amsterdam, Amsterdam Institute for Global Health and Development, Netherlands

(Hermans) Department of Internal Medicine, School of Medicine, Makerere University College of Health Sciences, Kampala, Uganda

(Bekker, Wood) Department of Medicine, University of Cape Town, Cape Town, South Africa

Publisher

International Union against Tubercul. and Lung Dis.

Link to the Ovid Full Text or citation:

[Click here for full text options](http://ovidsp.ovid.com/ovidweb.cgi?T=JS&CSC=Y&NEWS=N&PAGE=fulltext&D=emed18b&AN=611736437)

Link to the External Link Resolver:

[SFX](http://whel-primo.hosted.exlibrisgroup.com/openurl/44WHELF_CAR/44WHELF_CAR_services_page?sid=OVID:embase&id=pmid:&id=doi:10.5588%2Fijtld.15.0855&issn=1027-3719&isbn=&volume=20&issue=9&spage=1185&pages=1185-1191+and+i&date=2016&title=International+Journal+of+Tuberculosis+and+Lung+Disease&atitle=An+integrated+community+TB-HIV+adherence+model+provides+an+alternative+to+DOT+for+tuberculosis+patients+in+Cape+Town&aulast=Kaplan&pid=<author>Kaplan+R.%3BCaldwell+J.%3BHermans+S.%3BAdriaanse+S.%3BMtwisha+L.%3BBekker+L.-G.%3BJennings+K.%3BWood+R.<%2Fauthor><AN>611736437<%2FAN><DT>Journal%3A+Article<%2FDT>)

33.

Effects of multidrug resistant tuberculosis treatment on patients' health related quality of life: Results from a follow up study.

Ahmad N., Javaid A., Sulaiman S.A.S., Basit A., Afridi A.K., Jaber A.A.S., Khan A.H.

PLoS ONE. 11 (7) (no pagination), 2016. Article Number: e0159560. Date of Publication: July 2016.

AN: 611503209

Background At present, within the management of multidrug resistant tuberculosis (MDR-TB) much attention is being paid to the traditional microbiological and clinical indicators. Evaluation of the impact of MDR-TB treatment on patients' Health Related Quality of Life (HRQoL) has remained a neglected area. Objective To evaluate the impact of MDR-TB treatment on patients HRQoL, and determine the predictors of variability in HRQoL along the course of treatment Methods A prospective follow up study was conducted at the programmatic management unit for drug resistant TB of Lady Reading Hospital Peshawar. Culture confirmed eligible MDR-TB patients were asked to self complete SF-36v2 at the baseline visit, and subsequently after the completion of 12 months of treatment and at the end of treatment. A score of <47 normbased scoring (NBS) points on component summary measures and health domain scales was considered indicative of function impairment. General linear model repeated measures ANOVA was used examine the change and predictors of change in physical component summary (PCS) and mental component summary (MCS) scores over the time. Results A total of 68 out of enrolled 81 eligible MDR-TB patients completed SF-36v2 questionnaire at the three time points. Patients' mean PCS scores at the three time points were, 38.2+/-4.7, 38.6+/-4.4 and 42.2+/-5.2 respectively, and mean MCS were 33.7+/-7.0, 35.5+/-6.9 and 40.0+/-6.9 respectively. Length of sickness prior to the diagnosis of MDR-TB was predictive of difference in PCS scores (F = 4.988, Df = 1, 66), whereas patients' gender (F = 5.638, Df = 1, 66) and length of sickness prior to the diagnosis of MDR-TB (F = 4.400, Df = 1, 66) were predictive of difference in MCS scores. Conclusion Despite the positive impact of MDR-TB treatment on patients' HRQoL, the scores on component summary measures suggested compromised physical and mental health even at the end of treatment. A large multicenter study is suggested to confirm the present findings. Copyright © 2016 Ahmad et al. This is an open access article distributed under the terms of the Creative Commons Attribution License, which permits unrestricted use, distribution, and reproduction in any medium, provided the original author and source are credited.

Institution

(Ahmad, Sulaiman, Jaber, Khan) Discipline of Clinical Pharmacy, School of Pharmaceutical Sciences, Universiti Sains Malaysia, Pulau Pinang, Malaysia (Javaid, Basit) Department of Pulmonology, Postgraduate Medical Institute Peshawar, Peshawar, Pakistan

(Afridi) PMDT, Lady Reading Hospital Peshawar, Peshawar, Pakistan

Publisher

Public Library of Science (E-mail: plos@plos.org)

Link to the Ovid Full Text or citation:

[Click here for full text options](http://ovidsp.ovid.com/ovidweb.cgi?T=JS&CSC=Y&NEWS=N&PAGE=fulltext&D=emed18b&AN=611503209)

Link to the External Link Resolver:

[SFX](http://whel-primo.hosted.exlibrisgroup.com/openurl/44WHELF_CAR/44WHELF_CAR_services_page?sid=OVID:embase&id=pmid:&id=doi:10.1371%2Fjournal.pone.0159560&issn=1932-6203&isbn=&volume=11&issue=7&spage=no+pagination&pages=no+pagination&date=2016&title=PLoS+ONE&atitle=Effects+of+multidrug+resistant+tuberculosis+treatment+on+patients'+health+related+quality+of+life%3A+Results+from+a+follow+up+study&aulast=Ahmad&pid=<author>Ahmad+N.%3BJavaid+A.%3BSulaiman+S.A.S.%3BBasit+A.%3BAfridi+A.K.%3BJaber+A.A.S.%3BKhan+A.H.<%2Fauthor><AN>611503209<%2FAN><DT>Journal%3A+Article<%2FDT>)

34.

Infliximab, adalimumab and golimumab for treating moderately to severely active ulcerative colitis after the failure of conventional therapy (Including a review of TA140 and TA262): Clinical effectiveness systematic review and economic model.

Archer R., Tappenden P., Ren S., James M.M.-S., Harvey R., Basarir H., Stevens J., Carroll C., Cantrel A., Lobo A., Hoque S.

Health Technology Assessment. 20 (39) (no pagination), 2016. Date of Publication: May 2016.

AN: 610521840

Background: Ulcerative colitis (UC) is the most common form of inflammatory bowel disease in the UK. UC can have a considerable impact on patients' quality of life. The burden for the NHS is substantial. Objectives: To evaluate the clinical effectiveness and safety of interventions, to evaluate the incremental cost-effectiveness of all interventions and comparators (including medical and surgical options), to estimate the expected net budget impact of each intervention, and to identify key research priorities. Data sources: Peer-reviewed publications, European Public Assessment Reports and manufacturers' submissions. The following databases were searched from inception to December 2013 for clinical effectiveness searches and from inception to January 2014 for cost-effectiveness searches for published and unpublished research evidence: MEDLINE, EMBASE, Cumulative Index to Nursing and Allied Health Literature, The Cochrane Library including the Cochrane Systematic Reviews Database, Cochrane Controlled Trials Register, Database of Abstracts of Reviews of Effects, the Health Technology Assessment database and NHS Economic Evaluation Database; ISI Web of Science, including Science Citation Index, and the Conference Proceedings Citation Index-Science and Bioscience Information Service Previews. The US Food and Drug Administration website and the European Medicines Agency website were also searched, as were research registers, conference proceedings and key journals. Review methods: A systematic review [including network meta-analysis (NMA)] was conducted to evaluate the clinical effectiveness and safety of named interventions. The health economic analysis included a review of published economic evaluations and the development of a de novo model. Results: Ten randomised controlled trials were included in the systematic review. The trials suggest that adult patients receiving infliximab (IFX) [Remicade, Merck Sharp Dohme Ltd (MSD)], adalimumab (ADA) (Humira, AbbVie) or golimumab (GOL) (Simponi, MSD) were more likely to achieve clinical response and remission than those receiving placebo (PBO). Hospitalisation data were limited, but suggested more favourable outcomes for ADA- and IFX-treated patients. Data on the use of surgical intervention were sparse, with a potential benefit for intervention-treated patients. Data were available from one trial to support the use of IFX in paediatric patients. Safety issues identified included serious infections, malignancies and administration site reactions. Based on the NMA, in the induction phase, all biological treatments were associated with statistically significant beneficial effects relative to PBO, with the greatest effect associated with IFX. For patients in response following induction, all treatments except ADA and GOL 100 mg at 32-52 weeks were associated with beneficial effects when compared with PBO, although these were not significant. The greatest effects at 8-32 and 32-52 weeks were associated with 100 mg of GOL and 5 mg/kg of IFX, respectively. For patients in remission following induction, all treatments except ADA at 8-32 weeks and GOL 50 mg at 32-52 weeks were associated with beneficial effects when compared with PBO, although only the effect of ADA at 32-52 weeks was significant. The greatest effects were associated with GOL (at 8-32 weeks) and ADA (at 32-52 weeks). The economic analysis suggests that colectomy is expected to dominate drug therapies, but for some patients, colectomy may not be considered acceptable. In circumstances in which only drug options are considered, IFX and GOL are expected to be ruled out because of dominance, while the incremental cost-effectiveness ratio for ADA versus conventional treatment is approximately 50,300 per QALY gained. Limitations: The health economic model is subject to several limitations: uncertainty associated with extrapolating trial data over a lifetime horizon, the model does not consider explicit sequential pathways of non-biological treatments, and evidence relating to complications of colectomy was identified through consideration of approaches used within previous models rather than a full systematic review. Conclusions: Adult patients receiving IFX, ADA or GOL were more likely to achieve clinical response and remission than those receiving PBO. Further data are required to conclusively demonstrate the effect of interventions on hospitalisation and surgical outcomes. The economic analysis indicates that colectomy is expected to dominate medical treatments for moderate to severe UC.Study registration:This study is registered as PROSPERO CRD42013006883. Funding:The National Institute for Health Research Health Technology Assessment programme. Copyright © Queen's Printer and Controller of HMSO 2016.

Institution

(Archer, Tappenden, Ren, James, Harvey, Basarir, Stevens, Carroll, Cantrel) Health Economics and Decision Science, School of Health and Related Research (ScHARR), University of Sheffield, Sheffield, United Kingdom (Lobo) Sheffield Teaching Hospitals NHS Foundation Trust, Sheffield, United Kingdom

(Hoque) Barts Health NHS Trust, London, United Kingdom

Publisher

NIHR Journals Library

Link to the Ovid Full Text or citation:

[Click here for full text options](http://ovidsp.ovid.com/ovidweb.cgi?T=JS&CSC=Y&NEWS=N&PAGE=fulltext&D=emed18a&AN=610521840)

Link to the External Link Resolver:

[SFX](http://whel-primo.hosted.exlibrisgroup.com/openurl/44WHELF_CAR/44WHELF_CAR_services_page?sid=OVID:embase&id=pmid:&id=doi:10.3310%2Fhta20390&issn=1366-5278&isbn=&volume=20&issue=39&spage=no+pagination&pages=no+pagination&date=2016&title=Health+Technology+Assessment&atitle=Infliximab%2C+adalimumab+and+golimumab+for+treating+moderately+to+severely+active+ulcerative+colitis+after+the+failure+of+conventional+therapy+(Including+a+review+of+TA140+and+TA262)%3A+Clinical+effectiveness+systematic+review+and+economic+model&aulast=Archer&pid=<author>Archer+R.%3BTappenden+P.%3BRen+S.%3BJames+M.M.-S.%3BHarvey+R.%3BBasarir+H.%3BStevens+J.%3BCarroll+C.%3BCantrel+A.%3BLobo+A.%3BHoque+S.<%2Fauthor><AN>610521840<%2FAN><DT>Journal%3A+Article<%2FDT>)

35.

Health and ethical consequences of outsourcing pivotal clinical trials to Latin America: A cross-sectional, descriptive study.

Homedes N., Ugalde A.

PLoS ONE. 11 (6) (no pagination), 2016. Article Number: e0157756. Date of Publication: June 2016.

AN: 610977356

Introduction: The implications of conducting clinical trials in low and middle income countries on the financial accessibility and safety of the pharmaceutical products available in those markets have not been studied. Regulatory practices and ethical declarations lead to the commercialization of the new products, referred to as New Molecular Entities (NMEs), in the countries where tested as soon as they are approved in high surveillance countries. Patients and patients' associations use the Latin American courts to access new and expensive treatments, regardless of their safety profile and therapeutic value. Design and Objectives: Cross-sectional, descriptive study. To determine the therapeutic value and safety profile of the NMEs approved by the Food and Drug Administration (FDA) in 2011 and 2012 that had been tested in Latin America, and the implications of their market approval for the pharmaceutical budgets in the countries where tested. Setting: Latin America. Measures: To assess the therapeutic value and safety of the NMEs commercialized in the different countries we used f independent drug bulletins. The prices of the NMEs for the consumers were obtained from the pharmaceutical price observatories of the countries were the medicines had been tested. If the price was not available in the observatories, it was obtained from pharmaceutical distributors. We used the countries' minimum wage and per capita income to calculate the financial accessibility of a course of treatment with the NMEs. Results: We found that 33 NMEs approved by the FDA in 2011 and 2012 have been tested in Latin America. Of these, 26 had been evaluated by independent drug bulletins and only five were found to add some value to a subset of patients and had significant side-effects. The pharmaceutical prices were very high, varied widely across countries and were unrelated to the countries' income per capita or minimum wage. Conclusion: The implementation of clinical trials in Latin America results in the commercialization of medicines with questionable safety profiles and limited therapeutic value, putting patients at risk and causing budgetary strains in pharmaceutical budgets. Copyright © 2016 Homedes, Ugalde. This is an open access article distributed under the terms of the Creative Commons Attribution License, which permits unrestricted use, distribution, and reproduction in any medium, provided the original author and source are credited.

Institution

(Homedes) Management Policy and Community Health, School of Public Health, University of Texas, El Paso, TX, United States (Homedes) Department of International Health, Georgetown University, Washington, DC, United States

(Ugalde) Department of Sociology, University of Texas, Austin, TX, United States

Publisher

Public Library of Science (E-mail: plos@plos.org)

Link to the Ovid Full Text or citation:

[Click here for full text options](http://ovidsp.ovid.com/ovidweb.cgi?T=JS&CSC=Y&NEWS=N&PAGE=fulltext&D=emed18a&AN=610977356)

Link to the External Link Resolver:

[SFX](http://whel-primo.hosted.exlibrisgroup.com/openurl/44WHELF_CAR/44WHELF_CAR_services_page?sid=OVID:embase&id=pmid:&id=doi:10.1371%2Fjournal.pone.0157756&issn=1932-6203&isbn=&volume=11&issue=6&spage=no+pagination&pages=no+pagination&date=2016&title=PLoS+ONE&atitle=Health+and+ethical+consequences+of+outsourcing+pivotal+clinical+trials+to+Latin+America%3A+A+cross-sectional%2C+descriptive+study&aulast=Homedes&pid=<author>Homedes+N.%3BUgalde+A.<%2Fauthor><AN>610977356<%2FAN><DT>Journal%3A+Article<%2FDT>)

36.

Management of Pemphigus Vulgaris.

Cholera M., Chainani-Wu N.

Advances in Therapy. 33 (6) (pp 910-958), 2016. Date of Publication: 01 Jun 2016.

AN: 610774927

Introduction: Pemphigus vulgaris (PV) is a chronic, autoimmune, vesiculobullous disease. As a result of the relative rarity of PV, published randomized controlled trials (RCTs) are limited, which makes it difficult to evaluate the efficacy of different treatment regimens in this disease. This also precludes conduct of a meta-analysis. Methods: English-language publications describing treatment outcomes of patients with PV were identified by searches of electronic databases through May 2015, and additionally by review of the bibliography of these publications. A total of 89 papers, which included 21 case reports, 47 case series, 8 RCTs, and 13 observational studies, were identified. The findings from these publications, including information on disease course and prognosis, medications used, treatment responses, and side effects, are summarized in the tables and text of this review. Results: Prior to availability of corticosteroid therapy, PV had a high fatality rate. Early publications from the 1970s reported high-dose, prolonged corticosteroid use and significant associated side effects. Later reports described use of corticosteroids along with steroid-sparing adjuvants, which allows a reduction in the total dose of corticosteroids and a reduction in observed mortality and morbidity. For the majority of patients in these reports, a long-term course on medications lasting about 5-10 years was observed; however, subgroups of patients requiring shorter courses or needing longer-term therapy have also been described. Early diagnosis of PV and early initiation of treatment were prognostic factors. In recent publications, commonly used initial regimens include corticosteroids in combination with mycophenolate or azathioprine; whereas, for patients with inadequate response to these regimens, adjuvants such as intravenous immunoglobulin (IVIg) or rituximab are used. Conclusion: The review findings emphasize the importance of early diagnosis, early initiation of treatment, and use of steroid-sparing adjuvants to allow a reduced total dose and duration on corticosteroids. Also highlighted is the need for more RCTs. Copyright © 2016, The Author(s).

Institution

(Cholera, Chainani-Wu) Private Practice in Oral Medicine Clinic, 100 West El Camino Real Suite 76, Mountain View, CA 94040, United States

Publisher

Springer Healthcare (E-mail: kathleen.burke@springer.com)

Link to the Ovid Full Text or citation:

[Click here for full text options](http://ovidsp.ovid.com/ovidweb.cgi?T=JS&CSC=Y&NEWS=N&PAGE=fulltext&D=emed18a&AN=610774927)

Link to the External Link Resolver:

[SFX](http://whel-primo.hosted.exlibrisgroup.com/openurl/44WHELF_CAR/44WHELF_CAR_services_page?sid=OVID:embase&id=pmid:&id=doi:10.1007%2Fs12325-016-0343-4&issn=0741-238X&isbn=&volume=33&issue=6&spage=910&pages=910-958&date=2016&title=Advances+in+Therapy&atitle=Management+of+Pemphigus+Vulgaris&aulast=Cholera&pid=<author>Cholera+M.%3BChainani-Wu+N.<%2Fauthor><AN>610774927<%2FAN><DT>Journal%3A+Review<%2FDT>)

37.

The effectiveness of mobile phone text messaging in improving medication adherence for patients with chronic diseases: A systematic review.

Sarabi R.E., Sadoughi F., Orak R.J., Bahaadinbeigy K.

Iranian Red Crescent Medical Journal. 18 (5) (no pagination), 2016. Article Number: e25183. Date of Publication: May 2016.

AN: 610446062

Context: Medication non-adherence is a commonly observed problem in the self-administration of treatment, regardless of the disease type. Text messaging reminders, as electronic reminders, provide an opportunity to improve medication adherence. In this study, we aimed to provide evidence addressing the question of whether text message reminders were effective in improving patients' adherence to medication. Evidence Acquisition: We carried out a systematic literature search, using the five electronic bibliographic databases: PubMed, Embase, PsycINFO, CINAHL, and the Cochrane central register of controlled trials. Studies were included on the basis of whether they examined the benefits and effects of short-message service (SMS) interventions on medication adherence. Results: The results of this systematic review indicated that text messaging interventions have improved patients' medication adherence rate (85%, 29.34). Included in the review, those who had problems with adherence, or those whom text messaging was most helpful had HIV, asthma, diabetes, schizophrenia and heart disease (73.5%). The period of intervention varied from 1 week to 14 months. The most common study design was randomized controlled trials (RCTs) (66%) carried out in the developed countries. Conclusions: This study demonstrated the potential of mobile phone text messaging for medication non-adherence problem solving. Copyright © 2016, Iranian Red Crescent Medical Journal.

Institution

(Sarabi, Sadoughi, Orak) Health Information Management Department, School of Management and Medical Information Sciences, Iran University of Medical Sciences, Tehran, Iran, Islamic Republic of (Bahaadinbeigy) Medical Informatics Research Center, Institute of Futures Studies in Health, Kerman University of Medical Sciences, Kerman, Iran, Islamic Republic of

Publisher

Kowsar Medical Publishing Company (E-mail: ircmj@irmj.ir)

Link to the Ovid Full Text or citation:

[Click here for full text options](http://ovidsp.ovid.com/ovidweb.cgi?T=JS&CSC=Y&NEWS=N&PAGE=fulltext&D=emed18a&AN=610446062)

Link to the External Link Resolver:

[SFX](http://whel-primo.hosted.exlibrisgroup.com/openurl/44WHELF_CAR/44WHELF_CAR_services_page?sid=OVID:embase&id=pmid:&id=doi:10.5812%2Fircmj.25183&issn=2074-1804&isbn=&volume=18&issue=5&spage=no+pagination&pages=no+pagination&date=2016&title=Iranian+Red+Crescent+Medical+Journal&atitle=The+effectiveness+of+mobile+phone+text+messaging+in+improving+medication+adherence+for+patients+with+chronic+diseases%3A+A+systematic+review&aulast=Sarabi&pid=<author>Sarabi+R.E.%3BSadoughi+F.%3BOrak+R.J.%3BBahaadinbeigy+K.<%2Fauthor><AN>610446062<%2FAN><DT>Journal%3A+Review<%2FDT>)

38.

Endovascular stent graft repair of aorto-iliac pseudoaneurysms - Hospital Kuala Lumpur experience.

Khang N.C., bin Azizi Z.A.B.

Medical Journal of Malaysia. 71 (1) (pp 17-22), 2016. Date of Publication: February 2016.

AN: 610216318

Background: This is a single institutional review of aortoiliac pseudoaneurysm of various aetiologies managed with endovascular stent graft repair. Methods: From 2009 to 2014, 16 patients had endovascular stent graft inserted for pseudoaneurysm of the thoracic aorta, abdominal aorta and iliac arteries in Hospital Kuala Lumpur. Co-morbidity, causative agents, in-hospital mortality, complications and outcomes were examined. Results: The average age was 59.1 years (range 36-77). Comorbidities include hypertension, diabetes mellitus, tuberculosis, prior infection and previous open aneurysmal repair. All patients had raised WBC (>10.0x109 /L), ESR or Creactive protein on admission while 50% of patients had fever. Blood cultures were positive in 4 patients. All patients were given antibiotics. Only one in-hospital mortality was noted at day-47 post-procedure. Two patients died of aortoenteric fistula at district hospital eight and 16 months later. One patient died of chronic graft infection two years later. One died of unrelated cause. One patient developed type IB endoleak from internal iliac artery two years later and surgical ligation was performed. The average follow up was 15.8 months. Conclusion: Endovascular stent graft repair for pseudoaneurysm is a viable option compared to open surgery. It is less invasive, has lower operative morbidity and fair outcomes. However, some cases may be due to inflammatory aortitis instead of infective pseudoaneurysm, given the frequent culture-negative results. In order to obtain high yield of bacteria culture for infected pseudoaneurysm, open repair with tissue culture is still the main mode of treatment especially for patients with low comorbidity. Copyright © 2016, Malaysian Medical Association. All rights reserved.

Institution

(Khang, bin Azizi) Department of Surgery, Hospital Kuala Lumpur, Kuala Lumpur, Malaysia

Publisher

Malaysian Medical Association (E-mail: mma@tm.net.my)

Link to the Ovid Full Text or citation:

[Click here for full text options](http://ovidsp.ovid.com/ovidweb.cgi?T=JS&CSC=Y&NEWS=N&PAGE=fulltext&D=emed18a&AN=610216318)

Link to the External Link Resolver:

[SFX](http://whel-primo.hosted.exlibrisgroup.com/openurl/44WHELF_CAR/44WHELF_CAR_services_page?sid=OVID:embase&id=pmid:&id=doi:&issn=0300-5283&isbn=&volume=71&issue=1&spage=17&pages=17-22&date=2016&title=Medical+Journal+of+Malaysia&atitle=Endovascular+stent+graft+repair+of+aorto-iliac+pseudoaneurysms+-+Hospital+Kuala+Lumpur+experience&aulast=Khang&pid=<author>Khang+N.C.%3Bbin+Azizi+Z.A.B.<%2Fauthor><AN>610216318<%2FAN><DT>Journal%3A+Article<%2FDT>)

39.

Personalized medicine for patients with MDR-TB.

Olaru I.D., Lange C., Heyckendorf J.

Journal of Antimicrobial Chemotherapy. 71 (4) (pp 852-855), 2016. Article Number: dkv354. Date of Publication: 01 Apr 2016.

AN: 610060891

The emergence of MDR-TB is a cause of great concern due to difficulties in patient management and poor treatment outcomes. Currently the duration of treatment and the choice of drugs for patients with MDR-TB are standardized in many countries. This might not be the best approach since the optimal therapy may depend on different pathogen- and host-related features. Combining the introduction of technological innovations such as whole bacillary genome sequencing for the identification of drug-resistance-associated mutations, therapeutic drug monitoring and host-directed therapies with an individualized approach to MDR-TB management will likely lead to more tolerable, shorter and more efficient treatment regimens and an increase in the quality of life of those affected by MDR-TB. Copyright © The Author 2015. Published by Oxford University Press on behalf of the British Society for Antimicrobial Chemotherapy. All rights reserved.

Institution

(Olaru, Lange, Heyckendorf) Division of Clinical Infectious Diseases, Research Center Borstel, Borstel, Germany (Olaru, Lange, Heyckendorf) Clinical Tuberculosis Center, German Center for Infection Research (DZIF), Borstel, Germany

(Olaru, Lange, Heyckendorf) International Health/Infectious Diseases, University of Lubeck, Lubeck, Germany

(Lange) Department of Medicine, Karolinska Institute, Stockholm, Sweden

(Lange) Department of Internal Medicine, University of Namibia, School of Medicine, Windhoek, Namibia

Publisher

Oxford University Press (E-mail: jnl.info@oup.co.uk)

Link to the Ovid Full Text or citation:

[Click here for full text options](http://ovidsp.ovid.com/ovidweb.cgi?T=JS&CSC=Y&NEWS=N&PAGE=fulltext&D=emed18a&AN=610060891)

Link to the External Link Resolver:

[SFX](http://whel-primo.hosted.exlibrisgroup.com/openurl/44WHELF_CAR/44WHELF_CAR_services_page?sid=OVID:embase&id=pmid:&id=doi:10.1093%2Fjac%2Fdkv354&issn=0305-7453&isbn=&volume=71&issue=4&spage=852&pages=852-855&date=2016&title=Journal+of+Antimicrobial+Chemotherapy&atitle=Personalized+medicine+for+patients+with+MDR-TB&aulast=Olaru&pid=<author>Olaru+I.D.%3BLange+C.%3BHeyckendorf+J.<%2Fauthor><AN>610060891<%2FAN><DT>Journal%3A+Article<%2FDT>)

40.

Pemphigus in North-Western Yemen: A therapeutic study of 75 cases.

Khatri M.L.

Indian Journal of Dermatology, Venereology and Leprology. 82 (3) (pp 359), 2016. Date of Publication: May-June 2016.

AN: 610199424

Background: The incidence of pemphigus, though not documented, seems to be quite high in Yemen. There is no universal consensus on the treatment of this disease. Aims: The aim was to evaluate the efficacy and side effects of different therapeutic regimens used in patients of pemphigus in North-Western Yemen. Patients and Methods: Seventy-five Yemeni patients (39 males and 36 females) were included. Diagnosis was based on clinical features, histopathology and the Tzanck test. Results of treatment with these different therapeutic regimens were compared: (1) dexamethasone-cyclophosphamide pulse (DCP), (2) dexamethasone pulse with oral azathioprine, (3) oral prednisolone with azathioprine, (4) oral prednisolone with oral cyclophosphamide, and (5) prednisolone monotherapy. Results: Pemphigus vulgaris (PV) was diagnosed in 46 patients, pemphigus foliaceus (PF) in 23, pemphigus vegetans (PVEG) in 5 and pemphigus herpetiformis (PH) in one. Among the 16 patients who received regular DCP therapy, 13 were in remission for 6 months to 11 years without medications (phase 4). Remission without pharmacotherapy could not be achieved with the other regimens and steroid-induced side-effects appeared to be more than with DCP. Limitations: Immunofluorescence was not available to confirm the diagnosis of pemphigus. Randomization was not done. Conclusion: The DCP regimen seemed to be superior to the other regimens used. Copyright © 2016 Indian Journal of Dermatology, Venereology, and Leprology.

Institution

(Khatri) Department of Dermatology, Saudi Hospital, Hajjah, Yemen (Khatri) Saudi Hospital, Hajjah, P.O. Box: 2757, Sana'a, Yemen

Publisher

Medknow Publications (B9, Kanara Business Centre, off Link Road, Ghatkopar (E), Mumbai 400 075, India)

Link to the Ovid Full Text or citation:

[Click here for full text options](http://ovidsp.ovid.com/ovidweb.cgi?T=JS&CSC=Y&NEWS=N&PAGE=fulltext&D=emed18a&AN=610199424)

Link to the External Link Resolver:

[SFX](http://whel-primo.hosted.exlibrisgroup.com/openurl/44WHELF_CAR/44WHELF_CAR_services_page?sid=OVID:embase&id=pmid:&id=doi:10.4103%2F0378-6323.175917&issn=0378-6323&isbn=&volume=82&issue=3&spage=359&pages=359&date=2016&title=Indian+Journal+of+Dermatology%2C+Venereology+and+Leprology&atitle=Pemphigus+in+North-Western+Yemen%3A+A+therapeutic+study+of+75+cases&aulast=Khatri&pid=<author>Khatri+M.L.<%2Fauthor><AN>610199424<%2FAN><DT>Journal%3A+Article<%2FDT>)

41.

Global urine metabolomics in patients treated with first-line tuberculosis drugs and identification of a novel metabolite of ethambutol.

Das M.K., Arya R., Debnath S., Debnath R., Lodh A., Bishwal S.C., Das A., Nanda R.K.

Antimicrobial Agents and Chemotherapy. 60 (4) (pp 2257-2264), 2016. Date of Publication: April 2016.

AN: 609689782

Population level variation of drug metabolism phenotype (DMP) has great implications in treatment outcome, drug-related side effects, and resistance development. In this study, we used a gas chromatography-time of flight-mass spectrometry (GC-TOF-MS)-based untargeted urine metabolomics approach to understand the DMP of a tuberculosis (TB) patient cohort (n = 20) from Tripura, a state in the northeastern part of India. Urine samples collected at different postdose time points (2 h, 6 h, 12 h, 24 h, 36 h, and 48 h) from these newly diagnosed TB patients receiving first-line anti-TB drugs were analyzed, and we have successfully detected three of the four first-line drugs, viz., isoniazid (INH), ethambutol (ETB), andpyrazinamide (PZA). The majority of their known metabolites, acetyl-isoniazid (AcINH), isonicotinic acid (INA), isonicotinuric acid (INTA), 2,2'-(ethylenediimino)-dibutyric acid (EDBA), 5-hydroxypyrazinamide (5OH-PZA), pyrazinoic acid (POA), and 5-hydroxypyrazinoic acid (5OH-POA), were also detected. Analyzing the variation in abundances of drugs and their known metabolites and calculating the metabolic ratios in these samples, we offer comprehensive DMP information on this small patient cohort that represents Tripura, India. The majority (75%) of these patients are found to be slow acetylators of INH. The average metabolic ratios of POA/PZA and 5OH-POA/POA are 3.16 +/- 3.03 and 6.09 +/- 6.15, respectively. Employing correlation analysis of the metabolomics metadata and a manual prediction of drug catabolism, we have proposed 2-aminobutyric acid (AABA) as a novel metabolite of ETB. These observations indicate the usefulness of GC-MS-based metabolomics to characterize the DMP at a population level and also to identify novel drug metabolites. Copyright © 2016, American Society for Microbiology. All Rights Reserved.

Institution

(Das, Arya, Bishwal, Nanda) Immunology Group, International Centre for Genetic Engineering and Biotechnology (ICGEB), New Delhi, India (Debnath, Debnath, Lodh, Das) Department of Tuberculosis and Respiratory Disease, Agartala Government Medical College, Agartala, Tripura, India

(Bishwal) School of Life Sciences, Sambalpur University, Sambalpur, Odisha, India

Publisher

American Society for Microbiology (E-mail: Journals@asmusa.org)

Link to the Ovid Full Text or citation:

[Click here for full text options](http://ovidsp.ovid.com/ovidweb.cgi?T=JS&CSC=Y&NEWS=N&PAGE=fulltext&D=emed18a&AN=609689782)

Link to the External Link Resolver:

[SFX](http://whel-primo.hosted.exlibrisgroup.com/openurl/44WHELF_CAR/44WHELF_CAR_services_page?sid=OVID:embase&id=pmid:&id=doi:10.1128%2FAAC.02586-15&issn=0066-4804&isbn=&volume=60&issue=4&spage=2257&pages=2257-2264&date=2016&title=Antimicrobial+Agents+and+Chemotherapy&atitle=Global+urine+metabolomics+in+patients+treated+with+first-line+tuberculosis+drugs+and+identification+of+a+novel+metabolite+of+ethambutol&aulast=Das&pid=<author>Das+M.K.%3BArya+R.%3BDebnath+S.%3BDebnath+R.%3BLodh+A.%3BBishwal+S.C.%3BDas+A.%3BNanda+R.K.<%2Fauthor><AN>609689782<%2FAN><DT>Journal%3A+Article<%2FDT>)

42.

Diabetes mellitus is associated with increased mortality during tuberculosis treatment: A prospective cohort study among tuberculosis patients in South-Eastern Amahra Region, Ethiopia.

Workneh M.H., Bjune G.A., Yimer S.A.

Infectious Diseases of Poverty. 5 (1) (no pagination), 2016. Article Number: 22. Date of Publication: March 21, 2016.

AN: 609673076

Background: There is growing evidence suggesting that diabetes mellitus (DM) affects disease presentation and treatment outcome in tuberculosis (TB) patients. This study aimed at investigating the role of DM on clinical presentations and treatment outcomes among newly diagnosed TB patients. Methods: A prospective cohort study was conducted in South-Eastern Amhara Region, Ethiopia from September 2013 till March 2015. Study subjects were consecutively recruited from 44 randomly selected health facilities in the study area. Participants were categorized into two patient groups, namely, patients with TB and DM (TBDM) and TB patients without DM (TBNDM). Findings on clinical presentations and treatment outcomes were compared between the two patient groups. Cox proportional hazard regression analysis was applied to identify factors associated with death. Results: Out of 1314 TB patients enrolled in the study, 109 (8.3 %) had coexisting DM. TBDM comorbidity [adjusted hazard ratio (AHR) 3.96; 95 % confidence interval (C.I.) (1.76-8.89)], and TB coinfection with human immunodeficiency virus (HIV) [AHR 2.59; 95 % C.I. (1.21-5.59)] were associated with increased death. TBDM and TBNDM patients did not show significant difference in clinical symptoms at baseline and during anti-TB treatment period. However, at the 2nd month of treatment, TBDM patients were more symptomatic compared to patients in the TBNDM group. Conclusions: The study showed that DM is associated with increased death during TB treatment. DM has no association with clinical presentation of TB except at the end of the intensive phase treatment. Routine screening of TB patients for DM is recommended for early diagnosis and treatment of patients with TBDM comorbidity. Copyright © 2016 Workneh et al.

Institution

(Workneh, Bjune, Yimer) University of Oslo, Institute of Health and Society, Faculty of Medicine, Oslo, Norway (Workneh, Yimer) Amhara Regional State Health Bureau, Bahir-Dar, Ethiopia

(Yimer) Oslo University Hospital, Department of Microbiology, Oslo, Norway

(Yimer) Norwegian Institute of Public Health, Department of Bacteriology and Immunology, Oslo, Norway

Publisher

BioMed Central Ltd. (E-mail: info@biomedcentral.com)

Link to the Ovid Full Text or citation:

[Click here for full text options](http://ovidsp.ovid.com/ovidweb.cgi?T=JS&CSC=Y&NEWS=N&PAGE=fulltext&D=emed18a&AN=609673076)

Link to the External Link Resolver:

[SFX](http://whel-primo.hosted.exlibrisgroup.com/openurl/44WHELF_CAR/44WHELF_CAR_services_page?sid=OVID:embase&id=pmid:&id=doi:10.1186%2Fs40249-016-0115-z&issn=2049-9957&isbn=&volume=5&issue=1&spage=no+pagination&pages=no+pagination&date=2016&title=Infectious+Diseases+of+Poverty&atitle=Diabetes+mellitus+is+associated+with+increased+mortality+during+tuberculosis+treatment%3A+A+prospective+cohort+study+among+tuberculosis+patients+in+South-Eastern+Amahra+Region%2C+Ethiopia&aulast=Workneh&pid=<author>Workneh+M.H.%3BBjune+G.A.%3BYimer+S.A.<%2Fauthor><AN>609673076<%2FAN><DT>Journal%3A+Article<%2FDT>)

43.

Tolerability and healthcare utilization in maintenance hemodialysis patients undergoing treatment for tuberculosis-related conditions.

Hamadah A.M., Beaulieu L.M., Wilson J.W., Aksamit T.R., Gregoire J.R., Williams A.W., Dillon J.J., Albright R.C., Onuigbo M., Iyer V.K., Hickson L.J.

Nephron. 132 (3) (pp 198-206), 2016. Date of Publication: 01 Mar 2016.

AN: 608327383

Background: The incidence of tuberculosis (TB) in end-stage renal disease is significantly higher than that in the general population. Among those with kidney dysfunction, anti-TB treatment is associated with increased side effects, but the effect on healthcare utilization is unknown. Methods/Aim: To assess patient-reported symptoms, adverse effects and describe changes in healthcare utilization patterns during treatment for TB, we conducted a case series (n = 12) of patients receiving maintenance hemodialysis (HD) from Mayo Clinic Dialysis Services and concurrent drug therapy for TB from January 2002 through May 2014. Healthcare utilization (hospitalizations and emergency department (ED) visits independent of hospital admission) was compared before and during treatment. Results: Patients were treated for latent (n = 7) or active (n = 5) TB. The majority of patients with latent disease were treated with isoniazid (n = 5, 71%), while active-disease patients received a 4-drug regimen. Adverse effects were reported in 83% of patients. Compared to measurements prior to drug initiation, serum albumin and dialysis weights were similar at 3 months. Commonly reported anti-TB drug toxicities were described. More than half (58%) of the patients were hospitalized at least once. No ED or hospital admissions occurred in the period prior to drug therapy, but healthcare utilization increased during treatment in the latent disease group (hospitalization rate per person-month: pre 0 vs. post 1). Conclusions: Among HD patients, anti-TB therapy is associated with frequently reported symptoms and increased healthcare utilization. Among this subset, patients receiving treatment for latent disease may be those with greatest increase in healthcare use. Careful monitoring and early complication detection may help optimize medication adherence and minimize hospitalizations. Copyright © 2016 S. Karger AG, Basel.

Institution

(Hamadah, Beaulieu, Gregoire, Williams, Dillon, Albright, Onuigbo, Iyer, Hickson) Division of Nephrology and Hypertension, Mayo Clinic, Rochester, MN, United States (Wilson) Division of Infectious Diseases, Mayo Clinic, Rochester, MN, United States

(Aksamit) Division of Pulmonary and Critical Care Medicine, Department of Internal Medicine, Mayo Clinic, Rochester, MN, United States

(Hickson) Robert D. and Patricia E. Kern Center for the Science of Health Care Delivery, Mayo Clinic, Rochester, MN, United States

(Hickson) Division of Nephrology and Hypertension, Department of Medicine, Mayo Clinic, 200 First Street SW, Rochester, MN 55905, United States

Publisher

S. Karger AG

Link to the Ovid Full Text or citation:

[Click here for full text options](http://ovidsp.ovid.com/ovidweb.cgi?T=JS&CSC=Y&NEWS=N&PAGE=fulltext&D=emed18a&AN=608327383)

Link to the External Link Resolver:

[SFX](http://whel-primo.hosted.exlibrisgroup.com/openurl/44WHELF_CAR/44WHELF_CAR_services_page?sid=OVID:embase&id=pmid:&id=doi:10.1159%2F000444148&issn=1660-8151&isbn=&volume=132&issue=3&spage=198&pages=198-206&date=2016&title=Nephron&atitle=Tolerability+and+healthcare+utilization+in+maintenance+hemodialysis+patients+undergoing+treatment+for+tuberculosis-related+conditions&aulast=Hamadah&pid=<author>Hamadah+A.M.%3BBeaulieu+L.M.%3BWilson+J.W.%3BAksamit+T.R.%3BGregoire+J.R.%3BWilliams+A.W.%3BDillon+J.J.%3BAlbright+R.C.%3BOnuigbo+M.%3BIyer+V.K.%3BHickson+L.J.<%2Fauthor><AN>608327383<%2FAN><DT>Journal%3A+Conference+Paper<%2FDT>)

44.

The clinical outcomes of oldest old patients with tuberculosis treated by regimens containing rifampicin, isoniazid, and pyrazinamide.

Huang-Shen L., Cheng C.-W., Lin M.-S., Chou Y.-L., Chang P.-J., Lin J.-C., Ye J.-J.

Clinical Interventions in Aging. 11 (pp 299-306), 2016. Date of Publication: 11 Mar 2016.

AN: 609001677

Objectives: To investigate the clinical characteristics, adverse drug reactions, and outcomes of the oldest old patients (aged >80 years) with tuberculosis (TB) treated with rifampicin, isoniazid, and pyrazinamide (RIP)-containing regimens. Design: A retrospective chart review study. Setting: A 1,200-bed tertiary teaching hospital in southwest Taiwan. Participants: We conducted a retrospective observational study between January 1, 2005 and December 31, 2011. Seven hundred adult patients (aged >18 years) with TB treated with RIP-containing anti-TB regimens were reviewed, including 161 oldest old patients. Outcome measures: Clinical outcomes included clinical responsiveness and microbiological eradication. Adverse outcomes included drug-induced hepatitis, and other symptoms included gastrointestinal upset (eg, abdominal pain, vomiting, diarrhea, or dyspepsia), skin rash, joint pain, and hyperuricemia. Results: Compared with the non-oldest old adult patients, the oldest old patients more frequently had hepatitis (P=0.014), gastrointestinal upset (P=0.029), and unfavorable outcomes (P<0.001). In a multivariate analysis, hepatitis during treatment (adjusted odds ratio: 3.482, 95% confidence interval: 1.537-7.885; P<0.003) and oldest old age (adjusted odds ratio: 5.161, 95% confidence interval: 2.294-11.613; P<0.010) were independent risk factors for unfavorable outcomes. In the oldest old patients with hepatitis, rifampicin use was more common in the favorable outcome group than in the unfavorable outcome group (100% vs 37.5%; P=0.001). Conclusion: The oldest old age and hepatitis during RIP treatment were associated with unfavorable outcomes. For the oldest old patients with TB having hepatitis during treatment, rifampicin rechallenge and use might benefit the treatment outcome. Copyright © 2016 Lin et al.

Institution

(Huang-Shen) Division of Infectious Diseases, Department of Internal Medicine, Chang gung Memorial Hospital, Chia-Yi, Taiwan (Republic of China) (Huang-Shen, Chang) Graduate Institute of Clinical Medical Sciences, College of Medicine, Chang gung University, Taoyuan, Taiwan (Republic of China)

(Cheng, Ye) Division of Infectious Diseases, Department of Internal Medicine, Chang gung Memorial Hospital at linkou, Chang gung University College of Medicine, Taoyuan, Taiwan (Republic of China)

(Lin) Division of Cardiology, Chang gung Memorial Hospital, Yunlin, Taiwan (Republic of China)

(Chou) Division of Pulmonary and Critical Care Medicine, Department of Internal Medicine, Chang gung Memorial Hospital, Chia-Yi, Taiwan (Republic of China)

(Lin) Division of Allergy and Immunology and rheumatology, Department of Internal Medicine, Chang gung Memorial Hospital, Chia-Yi, Taiwan (Republic of China)

Publisher

Dove Medical Press Ltd. (PO Box 300-008, Albany, Auckland, New Zealand)

Link to the Ovid Full Text or citation:

[Click here for full text options](http://ovidsp.ovid.com/ovidweb.cgi?T=JS&CSC=Y&NEWS=N&PAGE=fulltext&D=emed18a&AN=609001677)

Link to the External Link Resolver:

[SFX](http://whel-primo.hosted.exlibrisgroup.com/openurl/44WHELF_CAR/44WHELF_CAR_services_page?sid=OVID:embase&id=pmid:&id=doi:10.2147%2FCIA.S95411&issn=1176-9092&isbn=&volume=11&issue=&spage=299&pages=299-306&date=2016&title=Clinical+Interventions+in+Aging&atitle=The+clinical+outcomes+of+oldest+old+patients+with+tuberculosis+treated+by+regimens+containing+rifampicin%2C+isoniazid%2C+and+pyrazinamide&aulast=Huang-Shen&pid=<author>Huang-Shen+L.%3BCheng+C.-W.%3BLin+M.-S.%3BChou+Y.-L.%3BChang+P.-J.%3BLin+J.-C.%3BYe+J.-J.<%2Fauthor><AN>609001677<%2FAN><DT>Journal%3A+Review<%2FDT>)

45.

Impact of point-of-care diagnostics on maternal outcomes in HIV-infected women: Systematic review and meta-analysis protocol.

Mashamba-Thompson T.P., Sartorius B., Thabane L., Shi C.X., Drain P.K.

BMJ Open. 6 (1) (no pagination), 2016. Article Number: e008002. Date of Publication: 2016.

AN: 608798197

Introduction: Studies indicate substandard diagnostic care, delayed and missed diagnosis as some of the contributing factors to maternal mortality. The clinical impact of point-of-care (POC) diagnostics has been shown in the monitoring and treatment of a variety of infectious diseases, including HIV/AIDS and tuberculosis. The objective of this systematic review is to investigate the impact of POC diagnostics on maternal outcomes for HIV-infected women. Methods: We will conduct a systematic review to evaluate the impact of POC diagnostics for improving desired healthcare outcomes for HIV-infected women. The search strategy will involve electronic databases including: Cochrane Infectious Disease Group Specialised Register; Cochrane Central Register of Control Trials, published in The Cochrane Library; PubMed; EBSCOhost and LILACS. The studies will be mapped in 2 stages: stage 1 will map studies descriptively by focus and method; stage 2 will involve additional inclusion criteria, quality assessment and data extraction undertaken by 2 reviewers in parallel. Evidence will be synthesised using relevant systematic research tools: meta-analysis and subgroup analysis will be conducted using RevMan and Stata 13 will be used for meta-regressions. We will follow recommendations described in the Preferred Reporting Items for Systematic Reviews and Meta-Analyses (PRISMA) statement and the Cochrane Handbook for Intervention Reviews. Ethics and dissemination: We anticipate finding a large number of studies on POC diagnostic interventions on maternal outcomes in HIV-infected women, which, once summarised, will be useful to guide future diagnostic interventions. The protocol for the systematic review has been registered in PROSPERO. The study will be disseminated electronically and in print. It will also be presented to conferences related to HIV/AIDS, POC diagnostics and maternal health. Trial registration number: PROSPERO CRD42014015439.

Institution

(Mashamba-Thompson, Sartorius) Discipline of Public Health, School of Nursing and Public Health, University of KwaZulu Natal, Durban, South Africa (Thabane) Biostatistics Unit/FSORC, St Joseph's Healthcare, Hamilton, ON, Canada

(Thabane) Department of Clinical Epidemiology and Biostatistics, McMaster University, Hamilton, ON, Canada

(Shi) Department of Epidemiology of Microbial Diseases, Yale School of Public Health, New Haven, CT, United States

(Drain) International Clinical Research Center, Department of Global Health, University of Washington, Seattle, WA, United States

(Drain) Division of Infectious Diseases, Department of Medicine, University of Washington, Seattle, WA, United States

(Drain) Department of Surgery, Massachusetts General Hospital, Boston, MA, United States

Publisher

BMJ Publishing Group (E-mail: subscriptions@bmjgroup.com)

Link to the Ovid Full Text or citation:

[Click here for full text options](http://ovidsp.ovid.com/ovidweb.cgi?T=JS&CSC=Y&NEWS=N&PAGE=fulltext&D=emed18a&AN=608798197)

Link to the External Link Resolver:

[SFX](http://whel-primo.hosted.exlibrisgroup.com/openurl/44WHELF_CAR/44WHELF_CAR_services_page?sid=OVID:embase&id=pmid:&id=doi:10.1136%2Fbmjopen-2015-008002&issn=2044-6055&isbn=&volume=6&issue=1&spage=no+pagination&pages=no+pagination&date=2016&title=BMJ+Open&atitle=Impact+of+point-of-care+diagnostics+on+maternal+outcomes+in+HIV-infected+women%3A+Systematic+review+and+meta-analysis+protocol&aulast=Mashamba-Thompson&pid=<author>Mashamba-Thompson+T.P.%3BSartorius+B.%3BThabane+L.%3BShi+C.X.%3BDrain+P.K.<%2Fauthor><AN>608798197<%2FAN><DT>Journal%3A+Review<%2FDT>)

46.

Comparison of Efficacy and Safety of Ciclosporin to Prednisolone in the Treatment of Erythema Nodosum Leprosum: Two Randomised, Double Blind, Controlled Pilot Studies in Ethiopia.

Lambert S.M., Nigusse S.D., Alembo D.T., Walker S.L., Nicholls P.G., Idriss M.H., Yamuah L.K., Lockwood D.N.J.

PLoS Neglected Tropical Diseases. 10 (2) (no pagination), 2016. Article Number: e0004149. Date of Publication: 26 Feb 2016.

AN: 608697928

Background: Erythema Nodosum Leprosum (ENL) is a serious complication of leprosy. It is normally treated with high dose steroids, but its recurrent nature leads to prolonged steroid usage and associated side effects. There is little evidence on the efficacy of alternative treatments for ENL, especially for patients who have become steroid resistant or have steroid side effects. These two pilot studies compare the efficacy and side effect profile of ciclosporin plus prednisolone against prednisolone alone in the treatment of patients with either new ENL or chronic and recurrent ENL. Methods and Results: Thirteen patients with new ENL and twenty patients with chronic ENL were recruited into two double-blinded randomised controlled trials. Patients were randomised to receive ciclosporin and prednisolone or prednisolone treatment only. Patients with acute ENL had a delay of 16 weeks in the occurrence of ENL flare-up episode, with less severe flare-ups and decreased requirements for additional prednisolone. Patients with chronic ENL on ciclosporin had the first episode of ENL flare-up 4 weeks earlier than those on prednisolone, as well as more severe ENL flare-ups requiring 2.5 times more additional prednisolone. Adverse events attributable to prednisolone were more common that those attributable to ciclosporin. Conclusions: This is the first clinical trial on ENL management set in the African context, and also the first trial in leprosy to use patients' assessment of outcomes. Patients on ciclosporin showed promising results in the management of acute ENL in this small pilot study. But ciclosporin, did not appear to have a significant steroid-sparing effects in patients with chronic ENL, which may have been due to the prolonged use of steroids in these patients in combination with a too rapid decrease of steroids in patients given ciclosporin. Further research is needed to determine whether the promising results of ciclosporin in acute ENL can be reproduced on a larger scale. Copyright © 2016 Lambert et al.

Institution

(Lambert, Walker, Lockwood) Department of Clinical Research, London School of Hygiene and Tropical Medicine, London, United Kingdom (Nigusse, Alembo, Idriss) All Africa Leprosy Rehabilitation and Training (ALERT) Center, Addis Ababa, Ethiopia

(Nicholls) School of Health Sciences, University of Southampton, Southampton, United Kingdom

(Yamuah) Data Management, Armauer Hansen Research Institute (AHRI), Addis Ababa, Ethiopia

Publisher

Public Library of Science (E-mail: plos@plos.org)

Link to the Ovid Full Text or citation:

[Click here for full text options](http://ovidsp.ovid.com/ovidweb.cgi?T=JS&CSC=Y&NEWS=N&PAGE=fulltext&D=emed18a&AN=608697928)

Link to the External Link Resolver:

[SFX](http://whel-primo.hosted.exlibrisgroup.com/openurl/44WHELF_CAR/44WHELF_CAR_services_page?sid=OVID:embase&id=pmid:&id=doi:10.1371%2Fjournal.pntd.0004149&issn=1935-2727&isbn=&volume=10&issue=2&spage=no+pagination&pages=no+pagination&date=2016&title=PLoS+Neglected+Tropical+Diseases&atitle=Comparison+of+Efficacy+and+Safety+of+Ciclosporin+to+Prednisolone+in+the+Treatment+of+Erythema+Nodosum+Leprosum%3A+Two+Randomised%2C+Double+Blind%2C+Controlled+Pilot+Studies+in+Ethiopia&aulast=Lambert&pid=<author>Lambert+S.M.%3BNigusse+S.D.%3BAlembo+D.T.%3BWalker+S.L.%3BNicholls+P.G.%3BIdriss+M.H.%3BYamuah+L.K.%3BLockwood+D.N.J.<%2Fauthor><AN>608697928<%2FAN><DT>Journal%3A+Article<%2FDT>)

47.

Does enhanced diabetes management reduce the risk and improve the outcome of tuberculosis?.

Lo H.-Y., Yang S.-L., Lin H.-H., Bai K.-J., Lee J.-J., Lee T.-I., Chiang C.-Y.

International Journal of Tuberculosis and Lung Disease. 20 (3) (pp 376-382), 2016. Date of Publication: 01 Mar 2016.

AN: 608513164

SETTING: The Bureau of National Health Insurance (NHI) has implemented a pay-for-performance (p4p) programme for diabetes mellitus (DM) in Taiwan. OBJECTIVE : To investigate whether patients with DM enrolled in the p4p programme (DM-p4p) are less likely to develop tuberculosis (TB) and whether they have a better outcome than patients with DMnot enrolled in the p4p programme (DM-non-p4p) if they do develop TB. DESIGN: A random sample of 79 471 DM-p4p, 100 000 DM-non-p4p and 100 000 non-diabetic patients (non-DM) was obtained from the 2008-2009 NHI database, and the patients were matched with the National TB Registry to determine whether they had developed TB by the end of 2010. RESULTS : The average annual incidence of TB was respectively 259.9 (95%CI 230.2-293.4), 137.5 (95%CI 116.4-162.5) and 74.1 (95%CI 59.0-93.0) per 100 000 population among DM-non-p4p, DM-p4p and non-DM patients. The relative risk of death over treatment success was 1.79 (95%CI 1.05-3.04) among DM-non-p4p and 1.69 (95%CI 0.84-3.40) among non-DM patients, relative to DM-p4p patients. CONCLUSIONS : Enhanced case management of DM reduced risk and improved outcomes of TB among patients with DM. Copyright © 2016 The Union.

Institution

(Lo, Yang) Taiwan Centers for Disease Control, Ministry of Health and Welfare, Taipei, Taiwan (Republic of China) (Lin) Institute of Epidemiology and Preventive Medicine, National Taiwan University, Taipei, Taiwan (Republic of China)

(Bai) School of Respiratory Therapy, College of Medicine, Taipei Medical University, Taipei, Taiwan (Republic of China)

(Bai, Chiang) Division of Pulmonary Medicine, Department of Internal Medicine, Wan Fang Hospital, Taipei Medical University, 111 Hsin-Long Road, Taipei, Taiwan (Republic of China)

(Lee) Department of Internal Medicine, Tzu Chi General Hospital and Tzu Chi University, Hualien, Taiwan (Republic of China)

(Lee) Division of Endocrinology and Metabolism, Department of Internal Medicine, Wan Fang Hospital, Taipei Medical University, Taipei, Taiwan (Republic of China)

(Lee) Department of General Medicine, School of Medicine, College of Medicine, Taipei Medical University, Taipei, Taiwan (Republic of China)

(Chiang) Department of Internal Medicine, School of Medicine, College of Medicine, Taipei Medical University, Taipei, Taiwan (Republic of China)

(Chiang) International Union Against Tuberculosis and Lung Disease, Paris, France

Publisher

International Union against Tubercul. and Lung Dis.

Link to the Ovid Full Text or citation:

[Click here for full text options](http://ovidsp.ovid.com/ovidweb.cgi?T=JS&CSC=Y&NEWS=N&PAGE=fulltext&D=emed18a&AN=608513164)

Link to the External Link Resolver:

[SFX](http://whel-primo.hosted.exlibrisgroup.com/openurl/44WHELF_CAR/44WHELF_CAR_services_page?sid=OVID:embase&id=pmid:&id=doi:10.5588%2Fijtld.15.0654&issn=1027-3719&isbn=&volume=20&issue=3&spage=376&pages=376-382&date=2016&title=International+Journal+of+Tuberculosis+and+Lung+Disease&atitle=Does+enhanced+diabetes+management+reduce+the+risk+and+improve+the+outcome+of+tuberculosis%3F&aulast=Lo&pid=<author>Lo+H.-Y.%3BYang+S.-L.%3BLin+H.-H.%3BBai+K.-J.%3BLee+J.-J.%3BLee+T.-I.%3BChiang+C.-Y.<%2Fauthor><AN>608513164<%2FAN><DT>Journal%3A+Article<%2FDT>)

48.

Direct observation of treatment provided by a family member as compared to non-family member among children with new tuberculosis: A pragmatic, non-inferiority, cluster-randomized trial in Gujarat, India.

Dave P.V., Shah A.N., Nimavat P.B., Modi B.B., Pujara K.R., Patel P., Mehariya K., Rade K.V., Shekar S., Sachdeva K.S., Oeltmann J.E., Kumar A.M.V.

PLoS ONE. 11 (2) (no pagination), 2016. Article Number: e0148488. Date of Publication: 01 Feb 2016.

AN: 608548264

Background TheWorld Health Organization recommends direct observation of treatment (DOT) to support patients with tuberculosis (TB) and to ensure treatment completion. As per national programme guidelines in India, a DOT provider can be anyone who is acceptable and accessible to the patient and accountable to the health system, except a family member. This poses challenges among children with TB who may be more comfortable receiving medicines from their parents or family members than from unfamiliar DOT providers. We conducted a non-inferiority trial to assess the effect of family DOT on treatment success rates among children with newly diagnosed TB registered for treatment during June- September 2012. Methods We randomly assigned all districts (n = 30) in Gujarat to the intervention (n = 15) or usualpractice group (n = 15). Adult family members in the intervention districts were given the choice to become their child's DOT provider. DOT was provided by a non-family member in the usual-practice districts. Using routinely collected clinic-based TB treatment cards, we compared treatment success rates (cured and treatment completed) between the two groups and the non-inferiority limit was kept at 5%. Results Of 624 children with newly diagnosed TB, 359 (58%) were from intervention districts and 265 (42%) were from usual-practice districts. The two groups were similar with respect to baseline characteristics including age, sex, type of TB, and initial body weight. The treatment success rates were 344 (95.8%) and 247 (93.2%) (p = 0.11) among the intervention and usual-practice groups respectively. Conclusion DOT provided by a family member is not inferior to DOT provided by a non-family member among new TB cases in children and can attain international targets for treatment success. Copyright This is an open access article, free of all copyright, and may be freely reproduced, distributed, transmitted, modified, built upon, or otherwise used by anyone for any lawful purpose. The work is made available under the Creative Commons CC0 public domain dedication.

Institution

(Dave, Nimavat, Modi, Pujara, Patel, Mehariya) Department of Health and Family Welfare, Government of Gujarat, Gandhinagar, Gujarat, India (Shah, Rade) WHO Country Office for India, New Delhi, India

(Shekar) National Tuberculosis Institute, Government of India, Bangalore, India

(Sachdeva) Central TB Division, Ministry of Health and Family Welfare, Government of India, New Delhi, India

(Oeltmann) U.S. Centers for Disease Control and Prevention, Atlanta, GA, United States

(Kumar) International Union Against Tuberculosis and Lung Disease, South-East Asia Regional Office, New Delhi, India

Publisher

Public Library of Science (E-mail: plos@plos.org)

Link to the Ovid Full Text or citation:

[Click here for full text options](http://ovidsp.ovid.com/ovidweb.cgi?T=JS&CSC=Y&NEWS=N&PAGE=fulltext&D=emed18a&AN=608548264)

Link to the External Link Resolver:

[SFX](http://whel-primo.hosted.exlibrisgroup.com/openurl/44WHELF_CAR/44WHELF_CAR_services_page?sid=OVID:embase&id=pmid:&id=doi:10.1371%2Fjournal.pone.0148488&issn=1932-6203&isbn=&volume=11&issue=2&spage=no+pagination&pages=no+pagination&date=2016&title=PLoS+ONE&atitle=Direct+observation+of+treatment+provided+by+a+family+member+as+compared+to+non-family+member+among+children+with+new+tuberculosis%3A+A+pragmatic%2C+non-inferiority%2C+cluster-randomized+trial+in+Gujarat%2C+India&aulast=Dave&pid=<author>Dave+P.V.%3BShah+A.N.%3BNimavat+P.B.%3BModi+B.B.%3BPujara+K.R.%3BPatel+P.%3BMehariya+K.%3BRade+K.V.%3BShekar+S.%3BSachdeva+K.S.%3BOeltmann+J.E.%3BKumar+A.M.V.<%2Fauthor><AN>608548264<%2FAN><DT>Journal%3A+Article<%2FDT>)

49.

Review on diverse issues in improvising drugs for treating leprosy.

Shanthi V.

International Journal of Pharmaceutical Sciences Review and Research. 36 (2) (pp 157-162), 2016. Article Number: 27. Date of Publication: January-February 2016.

AN: 608745523

Leprosy control depends exclusively on case recognition and management with multi-drug therapy. This approach is based on recognizing and treating chronic infectious diseases with effective drug combinations limits the materialization and spread of existing drug resistant pathogens. Besides, tuberculosis educated the relapse cases that are under risk for drug resistance which can destabilize the control measures on hand. Consequently, the construction of multi-drug therapy like approach for controlling leprosy involves systematic evaluation of treatment failures. Several studies have recognized relapses after MDT and drug-resistant strains of Mycobacterium leprae have been identified. In this review, we describe the drugs used to treat leprosy and the need for addition of novel drugs in MDT to improve treatment of leprosy patients. Copyright © protected.

Institution

(Shanthi) Department of Biotechnology, School of Bio Sciences and Technology, VIT University, Vellore, Tamil Nadu, India

Publisher

Global Research Online (Plot No: 6, R. K. Lake view, Hebbagudi, Anekal Taluk, Bangalore, India)

Link to the Ovid Full Text or citation:

[Click here for full text options](http://ovidsp.ovid.com/ovidweb.cgi?T=JS&CSC=Y&NEWS=N&PAGE=fulltext&D=emed18a&AN=608745523)

Link to the External Link Resolver:

[SFX](http://whel-primo.hosted.exlibrisgroup.com/openurl/44WHELF_CAR/44WHELF_CAR_services_page?sid=OVID:embase&id=pmid:&id=doi:&issn=0976-044X&isbn=&volume=36&issue=2&spage=157&pages=157-162&date=2016&title=International+Journal+of+Pharmaceutical+Sciences+Review+and+Research&atitle=Review+on+diverse+issues+in+improvising+drugs+for+treating+leprosy&aulast=Shanthi&pid=<author>Shanthi+V.<%2Fauthor><AN>608745523<%2FAN><DT>Journal%3A+Review<%2FDT>)

50.

Cost-effectiveness of isoniazid preventive therapy for HIVinfected pregnant women in India.

Kapoor S., Gupta A., Shah M.

International Journal of Tuberculosis and Lung Disease. 20 (1) (pp 85-92), 2016. Date of Publication: 01 Jan 2016.

AN: 607442788

Background: India has a high burden of active tuberculosis (TB) and human immunodeficiency virus (HIV) infection. Pregnancy increases the risks of developing TB in HIV-infected women. Isoniazid preventive therapy (IPT) reduces progression to TB, but may increase costs and hepatotoxicity. The costeffectiveness of IPT for HIV-infected pregnant women in India is unknown. DESIGN: We evaluated the cost-effectiveness of antepartum IPT among HIV-infected women in India using a decision-analytic model. We compared current practice (no IPT) with: Intervention 1 (IPT regardless of CD4 count) and Intervention 2 (IPT for those with CD4 count < 200 cells/mul). We modeled IPT irrespective of tuberculin skin test (TST) status and TST-driven strategies. Primary outcomes were anticipated costs, disability-adjusted life-years (DALYs) and TB cases. R ESULT S: Both IPT interventions are highly costeffective compared to no IPT at current willingness-topay thresholds (respectively US$178.00 and US$201.00 per DALY averted for Interventions 1 and 2). However, providing IPT irrespective of CD4 count results in the greatest health benefits (21 TB cases averted/1000 patients) compared to current practice. IPT irrespective of TSTstatus was also highly cost-effective compared to TST-driven IPT (respectively US$1027.00 and US$1154.00/DALY averted for Interventions 1 and 2). CONCLUS ION: Antepartum IPT for HIV-infected women is highly cost-effective for TB prevention compared to current practices in India. Copyright © 2016 The Union.

Institution

(Kapoor, Gupta, Shah) Johns Hopkins University, School of Medicine, PCTB Building-224, 725 N Wolfe St., Baltimore, MD 21205, United States

Publisher

International Union against Tubercul. and Lung Dis.

Link to the Ovid Full Text or citation:

[Click here for full text options](http://ovidsp.ovid.com/ovidweb.cgi?T=JS&CSC=Y&NEWS=N&PAGE=fulltext&D=emed18a&AN=607442788)

Link to the External Link Resolver:

[SFX](http://whel-primo.hosted.exlibrisgroup.com/openurl/44WHELF_CAR/44WHELF_CAR_services_page?sid=OVID:embase&id=pmid:&id=doi:10.5588%2Fijtld.15.0391&issn=1027-3719&isbn=&volume=20&issue=1&spage=85&pages=85-92&date=2016&title=International+Journal+of+Tuberculosis+and+Lung+Disease&atitle=Cost-effectiveness+of+isoniazid+preventive+therapy+for+HIVinfected+pregnant+women+in+India&aulast=Kapoor&pid=<author>Kapoor+S.%3BGupta+A.%3BShah+M.<%2Fauthor><AN>607442788<%2FAN><DT>Journal%3A+Article<%2FDT>)
