# Supplementary material for: Effect of glycemic control and type of diabetes treatment on TB treatment outcomes among people with TB-diabetes: A systematic review (updated August 2024)
Source: PLoS One. 2025 Jul 18;20(7):e0328619. doi: 10.1371/journal.pone.0328619 (PMC12273911; doi:10.1371/journal.pone.0328619)
Supplement: S1 Appendix — (ZIP) [file pone.0328619.s004.zip › S1 appendix_old/EMBASE/update Sept 2016 to current 24_04_2017/298-1_with RISK search reuslts no 15_2016 to current.doc]

Database: EMBASE <1947-Present>

Search Strategy:

--------------------------------------------------------------------------------

1	tuberculosis.mp. or exp tuberculosis/ (291433)

2	exp treatment outcome/ or treatment outcome*.mp. (1289437)

3	diabetes mellitus.mp. or exp diabetes mellitus/ (880018)

4	diabetes mellitus/ (599265)

5	DM.mp. (234038)

6	diabetes.mp. (891272)

7	mellitus.mp. (827628)

8	3 or 4 or 5 or 6 or 7 (1125394)

9	1 and 2 and 8 (1560)

10	cohort studies.mp. or cohort analysis/ (334934)

11	exp risk factor/ or cohort.mp. or exp risk/ (2843815)

12	10 or 11 (2843815)

13	9 and 10 (161)

14	9 and 12 (652)

15	limit 14 to human (642)

16	limit 13 to human (160)

17	trial*.mp. or exp controlled clinical trial/ or exp "clinical trial (topic)"/ or exp "randomized controlled trial (topic)"/ or exp "controlled clinical trial (topic)"/ (2044040)

18	random.ab. (253017)

19	randomized controlled trial.pt. (0)

20	controlled clinical trial.pt. (0)

21	randomized.ab. (540645)

22	placebo.ab. (252710)

23	clinical trials as topic.sh. (2)

24	trial.ti. (233523)

25	17 or 18 or 19 or 20 or 21 or 22 or 23 or 24 (2406494)

26	9 and 25 (443)

27	limit 26 to human (442)

28	limit 16 to yr="2016 - 2017" (19)

29	limit 15 to yr="2016 -Current" (61)

30	limit 16 to yr="2016 -Current" (19)

31	limit 27 to yr="2016 -Current" (50)

***************************

1.

Prevalence and Impact of Diabetes Mellitus Among Patients with Active Pulmonary Tuberculosis in South Korea.

Lee E.H., Lee J.M., Kang Y.A., Leem A.Y., Kim E.Y., Jung J.Y., Park M.S., Kim Y.S., Kim S.K., Chang J., Kim S.Y.

Lung. 195 (2) (pp 209-215), 2017. Date of Publication: 01 Apr 2017.

AN: 615272346

Objective: South Korea has an increasing prevalence of diabetes and a relatively high burden of tuberculosis. We aimed to determine the prevalence of diabetes in patients with active pulmonary tuberculosis (PTB) and examine the effect of diabetes on tuberculosis treatment outcomes. Setting and Design: Data from patients >30 years diagnosed with and treated for PTB between January 2010 and December 2012 at Severance Hospital, a 2000-bed tertiary referral hospital in Seoul, South Korea, were analyzed and compared with data from a contemporaneous general population sample extracted from KNHANES V. Results: Diabetes prevalence was 24.2% (252/1044) among patients with PTB and 11.6% (1700/14,655) among controls. Diabetes [odds ratios (OR) 2.56, 95% confidence interval (CI) 1.56-4.21, P < 0.001], male sex (OR 1.93, 95% CI 1.08-3.44, P = 0.027), and cavitary disease (OR 2.08, 95% CI 1.29-3.35, P = 0.003) were significant risk factors for 2-month culture positivity. Diabetes was the only factor associated with unsuccessful treatment outcomes (OR 1.67, 95% CI 1.03-2.70, P = 0.039). Conclusion: The prevalence of diabetes was markedly higher in patients with PTB than in a sample of the general South Korean population. Diabetes may delay sputum conversion and adversely affect treatment outcomes; detection and management of diabetes in patients with PTB is crucial. Copyright © 2017, Springer Science+Business Media New York.

Institution

(Lee, Lee, Kang, Leem, Kim, Jung, Park, Kim, Kim, Chang, Kim) Division of Pulmonology, Department of Internal Medicine, Severance Hospital, Institute of Chest Diseases, Yonsei University College of Medicine, 50-1 Yonsei-ro, Seodaemun-gu, Seoul 120-752, South Korea

Publisher

Springer New York LLC (E-mail: barbara.b.bertram@gsk.com)

Link to the Ovid Full Text or citation:

[Click here for full text options](http://ovidsp.ovid.com/ovidweb.cgi?T=JS&CSC=Y&NEWS=N&PAGE=fulltext&D=emed18b&AN=615272346)

Link to the External Link Resolver:

[SFX](http://whel-primo.hosted.exlibrisgroup.com/openurl/44WHELF_CAR/44WHELF_CAR_services_page?sid=OVID:embase&id=pmid:&id=doi:10.1007%2Fs00408-017-9978-4&issn=0341-2040&isbn=&volume=195&issue=2&spage=209&pages=209-215&date=2017&title=Lung&atitle=Prevalence+and+Impact+of+Diabetes+Mellitus+Among+Patients+with+Active+Pulmonary+Tuberculosis+in+South+Korea&aulast=Lee&pid=<author>Lee+E.H.%3BLee+J.M.%3BKang+Y.A.%3BLeem+A.Y.%3BKim+E.Y.%3BJung+J.Y.%3BPark+M.S.%3BKim+Y.S.%3BKim+S.K.%3BChang+J.%3BKim+S.Y.<%2Fauthor><AN>615272346<%2FAN><DT>Journal%3A+Article<%2FDT>)

2.

Diabetes mellitus in patients with pulmonary tuberculosis in an aging population in Shanghai, China: Prevalence, clinical characteristics and outcomes.

Wu Z., Guo J., Huang Y., Cai E., Zhang X., Pan Q., Yuan Z., Shen X.

Journal of Diabetes and its Complications. 30 (2) (pp 237-241), 2016. Date of Publication: 01 Mar 2016.

AN: 609073866

Aims To determine the prevalence of diabetes mellitus among pulmonary tuberculosis patients and the difference of clinical characteristics and outcomes between pulmonary tuberculosis patients with and without diabetes mellitus in an aging population in Shanghai, China. Methods This is a retrospective population-based study. 201 newly diagnosed pulmonary tuberculosis patients in Changning District, Shanghai during 2007-2008 were included. Clinical characteristics and outcomes were collected. Determination of diabetes mellitus was based on the medical records before pulmonary tuberculosis was diagnosed. Results The prevalence of diabetes mellitus among pulmonary tuberculosis patients was 19.9% (40/201). Pulmonary tuberculosis patients with diabetes mellitus were more likely to be old (> 50, OR = 5.23, 95% CI = 2.07-13.25), to have pulmonary cavities (OR = 3.02, 95% CI = 1.31-6.98), to be sputum smear positive (OR = 2.90, 95% CI = 1.12-7.51), and to have extension of anti-tuberculosis treatment duration (OR = 2.68, 95% CI 1.17-6.14). Besides, they had a higher 2nd month sputum smear positive proportion (OR = 2.97, 95% CI 1.22-7.22) and a higher 5-year recurrence rate (OR = 5.87, 95% CI 1.26-27.40). Conclusions High prevalence, severe clinical characteristics and poor outcomes of pulmonary tuberculosis patients with diabetes mellitus highlight the necessity of early bi-directional screening and co-management of these two diseases in Shanghai, China. Copyright © 2016 Elsevier Inc. All rights reserved.

PMID

26684166 [<http://www.ncbi.nlm.nih.gov/pubmed/?term=26684166>]

Institution

(Wu, Guo, Pan, Yuan, Shen) Department of Tuberculosis Control, Shanghai Municipal Center for Disease Control and Prevention, Shanghai, China (Huang, Cai, Zhang) Shanghai Changning Center for Disease Control and Prevention, Shanghai, China

Publisher

Elsevier Inc. (E-mail: usjcs@elsevier.com)

Link to the Ovid Full Text or citation:

[Click here for full text options](http://ovidsp.ovid.com/ovidweb.cgi?T=JS&CSC=Y&NEWS=N&PAGE=fulltext&D=emed18b&AN=609073866)

Link to the External Link Resolver:

[SFX](http://whel-primo.hosted.exlibrisgroup.com/openurl/44WHELF_CAR/44WHELF_CAR_services_page?sid=OVID:embase&id=pmid:26684166&id=doi:10.1016%2Fj.jdiacomp.2015.11.014&issn=1056-8727&isbn=&volume=30&issue=2&spage=237&pages=237-241&date=2016&title=Journal+of+Diabetes+and+its+Complications&atitle=Diabetes+mellitus+in+patients+with+pulmonary+tuberculosis+in+an+aging+population+in+Shanghai%2C+China%3A+Prevalence%2C+clinical+characteristics+and+outcomes&aulast=Wu&pid=<author>Wu+Z.%3BGuo+J.%3BHuang+Y.%3BCai+E.%3BZhang+X.%3BPan+Q.%3BYuan+Z.%3BShen+X.<%2Fauthor><AN>609073866<%2FAN><DT>Journal%3A+Article<%2FDT>)

3.

Incidence of tuberculosis is high in chronic kidney disease patients in South East England and drug resistance common.

Ostermann M., Palchaudhuri P., Riding A., Begum P., Milburn H.J.

Renal Failure. 38 (2) (pp 256-261), 2016. Date of Publication: 07 Feb 2016.

AN: 607623274

The risk of tuberculosis (TB) is significantly increased in chronic kidney disease (CKD). Data on TB in CKD in the UK are sparse; most information stems from countries with high background prevalence. The aim of this study was to estimate the incidence of TB in CKD patients in South East London and to describe the epidemiology, treatment, and outcome. CKD patients with TB between 1994 and 2010 were identified retrospectively. Data were collected on type of renal replacement therapy, the method of TB diagnosis, disease site, treatment regimens, and risk factors. Forty patients were identified of whom 67.5% had CKD stages IV-V. Sixty-five percent were from non-UK born ethnic minorities. Median time from diagnosis of CKD to TB development was 12 months (range 0-192 months). Cumulative incidence of TB was 1267/100,000 [95% confidence interval (CI): 630-1904; 85 x background UK rate] in hemodialysis patients; 398/100,000 (95% CI: 80-1160; 26 x background UK rate) in peritoneal dialysis; and 522/100,000 (CI: 137-909; 35 x background UK rate) in transplant recipients. Sixty-three percent of patients had pulmonary TB and 25% of patients with culture-positive TB had resistant isolates. Fifty percent of patients were immunosuppressed due to drugs, diabetes, and/or retroviral disease. Treatment regimens were according to recent national guidance in 73% of cases. Seventy-six percent of patients experienced side effects. Greater awareness of risk factors, drug resistance, treatment regimens, and potential side effects is needed. Copyright © 2015 Taylor & Francis.

PMID

26726960 [<http://www.ncbi.nlm.nih.gov/pubmed/?term=26726960>]

Institution

(Ostermann) Department of Renal Medicine, Guys and St Thomas NHS Foundation Trust, London, United Kingdom (Ostermann, Milburn) Guys and St Thomas NHS Foundation Trust, Kings College London, Great Maze Pond, London SE1 9RT, United Kingdom

(Palchaudhuri, Riding, Begum, Milburn) Department of Respiratory Medicine, Guys and St Thomas NHS Foundation Trust, London, United Kingdom

Publisher

Taylor and Francis Ltd (E-mail: healthcare.enquiries@informa.com)

Link to the Ovid Full Text or citation:

[Click here for full text options](http://ovidsp.ovid.com/ovidweb.cgi?T=JS&CSC=Y&NEWS=N&PAGE=fulltext&D=emed18b&AN=607623274)

Link to the External Link Resolver:

[SFX](http://whel-primo.hosted.exlibrisgroup.com/openurl/44WHELF_CAR/44WHELF_CAR_services_page?sid=OVID:embase&id=pmid:26726960&id=doi:10.3109%2F0886022X.2015.1128290&issn=0886-022X&isbn=&volume=38&issue=2&spage=256&pages=256-261&date=2016&title=Renal+Failure&atitle=Incidence+of+tuberculosis+is+high+in+chronic+kidney+disease+patients+in+South+East+England+and+drug+resistance+common&aulast=Ostermann&pid=<author>Ostermann+M.%3BPalchaudhuri+P.%3BRiding+A.%3BBegum+P.%3BMilburn+H.J.<%2Fauthor><AN>607623274<%2FAN><DT>Journal%3A+Article<%2FDT>)

4.

Diabetes reduces the rate of sputum culture conversion in patients with newly diagnosed multidrug-resistant tuberculosis.

Salindri A.D., Kipiani M., Kempker R.R., Gandhi N.R., Darchia L., Tukvadze N., Blumberg H.M., Magee M.J.

Open Forum Infectious Diseases. 3 (3) (no pagination), 2016. Article Number: ofw126. Date of Publication: 2016.

AN: 614929507

Background. Diabetes is a risk factor for active tuberculosis (TB), but little is known about the relationship between diabetes and multidrug-resistant (MDR) TB. We aimed to assess risk factors for primary MDR TB, including diabetes, and determine whether diabetes reduced the rate of sputum culture conversion among patients with MDR TB. Methods. From 2011 to 2014, we conducted a cohort study at the National Center for Tuberculosis and Lung Diseases in Tbilisi, Georgia. Adult (>35 years) patients with primary TB were eligible. Multidrug-resistant TB was defined as resistance to at least rifampicin and isoniazid. Patients with capillary glycosylated hemoglobin (HbA1c) > 6.5% or previous diagnosis were defined to have diabetes. Polytomous regression was used to estimate the association of patient characteristics with drug resistance. Cox regression was used to compare rates of sputum culture conversion in patients with and without diabetes. Results. Among 318 patients with TB, 268 had drug-susceptibility test (DST) results. Among patients with DST results, 19.4% (52 of 268) had primary MDR TB and 13.4% (36 of 268) had diabetes. In multivariable analyses, diabetes (adjusted odds ratio [aOR], 2.51; 95% confidence interval [CI], 1.00-6.31) and lower socioeconomic status (aOR, 3.51; 95% CI, 1.56-8.20) were associated with primary MDR TB. Among patients with primaryMDR TB, 44 (84.6%) converted sputum cultures to negative. The rate of sputum culture conversion was lower among patients with diabetes (adjusted hazard ratio [aHR], 0.34; 95% CI,.13-.87) and among smokers (aHR, 0.16; 95% CI,.04-.61). Conclusions. We found diabetes was associated with an increased risk of primary MDR TB; both diabetes and smoking were associated with a longer time to sputum culture conversion. Copyright © The Author 2016.

Institution

(Salindri, Magee) Division of Epidemiology and Biostatistics, School of Public Health, Georgia State University, GA, United States (Kipiani, Darchia, Tukvadze) National Center for Tuberculosis and Lung Disease, Tbilisi, Georgia

(Kempker, Gandhi, Blumberg) Division of Infectious Diseases, Department of Medicine, Emory University School of Medicine, GA, United States

(Gandhi, Blumberg, Magee) Departments of Epidemiology and Global Health, Rollins School of Public Health, Emory University, Atlanta, GA, United States

Publisher

Oxford University Press (E-mail: info@idsociety.org)

Link to the Ovid Full Text or citation:

[Click here for full text options](http://ovidsp.ovid.com/ovidweb.cgi?T=JS&CSC=Y&NEWS=N&PAGE=fulltext&D=emed18b&AN=614929507)

Link to the External Link Resolver:

[SFX](http://whel-primo.hosted.exlibrisgroup.com/openurl/44WHELF_CAR/44WHELF_CAR_services_page?sid=OVID:embase&id=pmid:&id=doi:10.1093%2Fofid%2Fofw126&issn=2328-8957&isbn=&volume=3&issue=3&spage=no+pagination&pages=no+pagination&date=2016&title=Open+Forum+Infectious+Diseases&atitle=Diabetes+reduces+the+rate+of+sputum+culture+conversion+in+patients+with+newly+diagnosed+multidrug-resistant+tuberculosis&aulast=Salindri&pid=<author>Salindri+A.D.%3BKipiani+M.%3BKempker+R.R.%3BGandhi+N.R.%3BDarchia+L.%3BTukvadze+N.%3BBlumberg+H.M.%3BMagee+M.J.<%2Fauthor><AN>614929507<%2FAN><DT>Journal%3A+Article<%2FDT>)

5.

The nature and duration of symptoms and time to starting treatment comparing older with younger pulmonary tuberculosis patients.

Abbara A., Hardman E., Collin S.M., Kon O.M., Mahomed Z., Sullivan J.A.L., Buell K., Hansel T., Corrah T., Davidson R.N.

Thorax. Conference: British Thoracic Society Winter Meeting 2016. United Kingdom. Conference Start: 20161207. Conference End: 20161209. 71 (pp A51-A52), 2016. Date of Publication: December 2016.

AN: 615030876

Introduction and objectives In 2013, Public Health England (PHE) reported that 39% of pulmonary tuberculosis (TB) patients >65 years old started treatment >4 months after symptom onset compared to 25% of patients aged 15-44 years. A longer symptomatic period, particular if smear positive, may result in increased transmission and poorer treatment outcomes. We investigated age-related differences in symptom duration and time to starting TB treatment in a large UK cohort of TB patients. Methods The cohort comprised patients on the London TB register (LTBR) who were treated at Northwick Park Hospital between 2002-2015. Patients aged >65 years were compared with a random sample of patients aged 18-40 years, with respect to symptoms, symptom duration at presenting to secondary care and time to starting treatment after presenting to secondary care. Results 357 patients over 65 and 517 younger patients were identified. Demographics for the total number of patients are included in Table 1. Data were available for 489 of the younger group and 73 of the older group. 26 of the >65s (35.6%) and 9 (1.8%) of the 65s were diabetic. Conclusions This study has identified that a potential cause for a delay in diagnosis in the elderly may be related to the decreased frequency of 'classical' TB symptoms in those >65 years of age. Clinicians need to be vigilant despite the lack of these symptoms. Median duration of symptoms was twice that in older patients versus younger patients (90 days versus 45) with a longer time to starting treatment (7 days versus 2). The total duration (in months) from symptom onset was also higher at 3 months versus 2 months. A higher proportion of older patients (32%) started treatment greater than 4 months from symptom onset compared to 13.1% in younger patients. Biases in this work including recording bias due to the retrospective nature of symptom recording, the effect of selecting younger patients from more recent years (to improve data availability) and the low proportion of patients over 65 which had symptom and treatment data available. (Table presented).

Institution

(Abbara, Mahomed, Corrah, Davidson) London North West Healthcare NHS Trust, London, United Kingdom (Hardman) University College Hospital, London, United Kingdom

(Collin) University of Bristol, Bristol, United Kingdom

(Kon) Imperial Healthcare NHS Trust, London, United Kingdom

(Sullivan, Buell) Imperial College, London, United Kingdom

(Hansel) NHLI, Imperial College, London, United Kingdom

Publisher

BMJ Publishing Group

Link to the Ovid Full Text or citation:

[Click here for full text options](http://ovidsp.ovid.com/ovidweb.cgi?T=JS&CSC=Y&NEWS=N&PAGE=fulltext&D=emed18b&AN=615030876)

Link to the External Link Resolver:

[SFX](http://whel-primo.hosted.exlibrisgroup.com/openurl/44WHELF_CAR/44WHELF_CAR_services_page?sid=OVID:embase&id=pmid:&id=doi:10.1136%2Fthoraxjnl-2016-209333.96&issn=1468-3296&isbn=&volume=71&issue=3&spage=A51&pages=A51-A52&date=2016&title=Thorax&atitle=The+nature+and+duration+of+symptoms+and+time+to+starting+treatment+comparing+older+with+younger+pulmonary+tuberculosis+patients&aulast=Abbara&pid=<author>Abbara+A.%3BHardman+E.%3BCollin+S.M.%3BKon+O.M.%3BMahomed+Z.%3BSullivan+J.A.L.%3BBuell+K.%3BHansel+T.%3BCorrah+T.%3BDavidson+R.N.<%2Fauthor><AN>615030876<%2FAN><DT>Journal%3A+Conference+Abstract<%2FDT>)

6.

Effect of poor glycemic control in newly diagnosed patients with smear-positive pulmonary tuberculosis and type-2 diabetes mellitus.

Mahishale V., Avuthu S., Patil B., Lolly M., Eti A., Khan S.

Iranian Journal of Medical Sciences. 42 (2) (pp 144-151), 2017. Date of Publication: March 2017.

AN: 614701548

Background: There is growing evidence that diabetes mellitus (DM) is an important risk factor for tuberculosis (TB). A significant number of DM patients have poor glycemic control. This study was carried out to find the impact of poor glycemic control on newly diagnosed smear-positive pulmonary tuberculosis patients with type-2 diabetes mellitus in a tertiary care hospital. Methods: In a hospital-based prospective study, newly diagnosed smear-positive pulmonary TB with DM patients were classified as poorly controlled diabetes (HBA1C>7%) and optimal control diabetics (HbA1c<7%). Patients were started on anti-TB treatment and followed for 2 years for severity and treatment outcome. ANOVA was used for numerical variables in the univariable analysis. Logistic regression analysis was used for multivariable analysis of treatment outcome. The significance level was kept at a P<0.05. Results: A total of 630 individuals who met the inclusion criteria were analyzed; of which 423 patients had poor glycemic control (PGC) and 207 patients had optimal glycemic control (OGC). The average HbA1c was 10+/-2.6 and 5+/-1.50 in the PGC and OGC groups, respectively. The mean symptom score was significantly higher in the PGC group compared with patients in the OGC group (4.55+/-0.80 vs. 2.70+/-0.82, P<0.001). PGC was associated with more extensive lung disease, lung cavitation, and positive sputum smear at the baseline. In PGC, sputum smears were significantly more likely to remain positive after 2 months of treatment. PGC patients had significantly higher rates of treatment failure (adj. OR 0.72, 95% CI 0.58-0.74, P<0.001) and relapse (adj. OR 2.83, 95% CI 2.60-2.92, P<0.001). Conclusion: Poor glycemic control is associated with an increased risk of advanced and more severe TB disease in the form of lung cavitations, positive sputum smear, and slower smear conversion. It has a profound negative effect on treatment completion, cure, and relapse rates in patients with pulmonary tuberculosis. Copyright © 2017, Shiraz University of Medical Sciences. All rights reserved.

Institution

(Mahishale, Avuthu, Patil, Lolly, Eti, Khan) Department of Pulmonary Medicine, Karnataka Lingayat Education University's J. N. Medical College, Belgaum, Karnataka, India

Publisher

Shiraz University of Medical Sciences (E-mail: ijms@sums.ac.ir)

Link to the Ovid Full Text or citation:

[Click here for full text options](http://ovidsp.ovid.com/ovidweb.cgi?T=JS&CSC=Y&NEWS=N&PAGE=fulltext&D=emed18b&AN=614701548)

Link to the External Link Resolver:

[SFX](http://whel-primo.hosted.exlibrisgroup.com/openurl/44WHELF_CAR/44WHELF_CAR_services_page?sid=OVID:embase&id=pmid:&id=doi:&issn=0253-0716&isbn=&volume=42&issue=2&spage=144&pages=144-151&date=2017&title=Iranian+Journal+of+Medical+Sciences&atitle=Effect+of+poor+glycemic+control+in+newly+diagnosed+patients+with+smear-positive+pulmonary+tuberculosis+and+type-2+diabetes+mellitus&aulast=Mahishale&pid=<author>Mahishale+V.%3BAvuthu+S.%3BPatil+B.%3BLolly+M.%3BEti+A.%3BKhan+S.<%2Fauthor><AN>614701548<%2FAN><DT>Journal%3A+Article<%2FDT>)

7.

The effect of diabetes control status on treatment response in pulmonary tuberculosis: A prospective study.

Yoon Y.S., Jung J.-W., Jeon E.J., Seo H., Ryu Y.J., Yim J.-J., Kim Y.H., Lee B.-H., Park Y.B., Lee B.J., Kang H., Choi J.C.

Thorax. 72 (3) (pp 263-270), 2017. Date of Publication: 01 Mar 2017.

AN: 611939205

Background: Uncontrolled diabetes, unlike controlled diabetes, is associated with an impaired immune response. However, little is known about the impact of the status of diabetes control on clinical features and treatment outcomes in patients with pulmonary TB (PTB). We conducted this study to evaluate whether the status of diabetes control influences clinical manifestations and treatment responses in PTB. Methods: A multicentre prospective study was performed between September 2012 and September 2014. The patients were categorised into three groups according to the glycated haemoglobin (HbA1C) level: PTB without diabetes mellitus (non-DM), PTB with controlled diabetes (controlled-DM) and PTB with uncontrolled diabetes (uncontrolled-DM). The primary outcome was the sputum culture conversion rate after 2 months of intensive treatment. Results: Among 661 patients with PTB, 157 (23.8%) had diabetes and 108 (68.8%) had uncontrolled diabetes (HbA1C>7.0%). The uncontrolled-DM group exhibited more symptoms, positive sputum smears (p<0.001) and presence of cavities (p<0.001) than the non-DM group. Regarding treatment responses, patients with uncontrolled-DM were more likely to have a positive culture after 2 months (p=0.009) and either treatment failure (p=0.015) or death (p=0.027) compared with the non-DM group. In contrast, those with controlled-DM showed similar treatment responses to the non-DM group. In multivariable analysis, uncontrolled diabetes was an independent risk factor for a positive sputum culture after 2 months of treatment (adjusted OR, 2.11; p=0.042) and either treatment failure or death (adjusted OR, 4.11; p=0.022). Conclusions: Uncontrolled diabetes is an independent risk factor for poor treatment response in PTB. Copyright © BMJ Publishing Group Limited 2017.

Institution

(Yoon) Division of Pulmonology and Critical Care Medicine, Department of Internal Medicine, Dongguk University Ilsan Hospital, Dongguk University College of Medicine, Goyang, South Korea (Jung, Choi) Division of Pulmonary Medicine, Department of Internal Medicine, Chung-Ang University, School of Medicine, HeukSeok Ro 102, Dong Jak Gu, Seoul 06973, South Korea

(Jeon) Department of Internal Medicine, Hallym Hospital, Incheon, South Korea

(Seo) Department of Tuberculosis, Seobuk Hospital, Seoul Metropolitan Government, Seoul, South Korea

(Ryu) Division of Pulmonary and Critical Care Medicine, Department of Internal Medicine, Ewha Womans University, School of Medicine, Seoul, South Korea

(Yim) Division of Pulmonary and Critical Care Medicine, Department of Internal Medicine, Seoul National University College of Medicine, Seoul, South Korea

(Kim) Division of Pulmonary and Critical Care Medicine, Department of Internal Medicine, Kyung Hee University Hospital at Gangdong, School of Medicine, Kyung Hee University, Seoul, South Korea

(Lee) Department of Pulmonology and Allergy, Eulji Hospital, College of Medicine, Eulji University, Seoul, South Korea

(Park) Department of Internal Medicine, Hallym University Medical Center, Kangdong Sacred Heart Hospital, Seoul, South Korea

(Lee) Division of Pulmonary and Critical Care Medicine, Department of Internal Medicine, Veterans Health Service Medical Center, Seoul, South Korea

(Kang) Department of Anesthesiology and Pain Medicine, Chung-Ang University, School of Medicine, Seoul, South Korea

Publisher

BMJ Publishing Group (E-mail: subscriptions@bmjgroup.com)

Link to the Ovid Full Text or citation:

[Click here for full text options](http://ovidsp.ovid.com/ovidweb.cgi?T=JS&CSC=Y&NEWS=N&PAGE=fulltext&D=emed18b&AN=611939205)

Link to the External Link Resolver:

[SFX](http://whel-primo.hosted.exlibrisgroup.com/openurl/44WHELF_CAR/44WHELF_CAR_services_page?sid=OVID:embase&id=pmid:&id=doi:10.1136%2Fthoraxjnl-2015-207686&issn=0040-6376&isbn=&volume=72&issue=3&spage=263&pages=263-270&date=2017&title=Thorax&atitle=The+effect+of+diabetes+control+status+on+treatment+response+in+pulmonary+tuberculosis%3A+A+prospective+study&aulast=Yoon&pid=<author>Yoon+Y.S.%3BJung+J.-W.%3BJeon+E.J.%3BSeo+H.%3BRyu+Y.J.%3BYim+J.-J.%3BKim+Y.H.%3BLee+B.-H.%3BPark+Y.B.%3BLee+B.J.%3BKang+H.%3BChoi+J.C.<%2Fauthor><AN>611939205<%2FAN><DT>Journal%3A+Article<%2FDT>)

8.

The impact of patient demographics and comorbidities upon burns admitted to Tygerberg Hospital Burns Unit, Western Cape, South Africa.

Cloake T., Haigh T., Cheshire J., Walker D.

Burns. 43 (2) (pp 411-416), 2017. Date of Publication: 01 Mar 2017.

AN: 614262868

In South Africa, burns are a major public health problem responsible for significant morbidity and long-term physical disability. This is, in part, due to a significant proportion of the urban population living in poorly constructed, combustible accommodation. The presence of co-morbid diseases such as diabetes and malignancy in patients with burns has been associated with a poorer outcome. The impact of other diseases such as HIV has yet to be defined. A retrospective data collection study analysed the 221 patients admitted to Tygerberg Hospital Burns Unit in 2011 and the first six months of 2013. Using hospital records, patient demographic data was collected alongside burn agent, ICU admission, complications, and patient outcome in terms of length of stay and mortality. The most common burn agent was hot liquid (45.7%). A significant proportion of patients were subject to intentional attacks (34.3%). Shack fires and flame accounted cumulatively for 85% of total inhalational burns, the highest rates of admission to ICU (85.5%), the highest rate of complications, as well as 92.3% of all total fatalities. HIV+ patients had a higher mortality (13.3% vs 5%, p = 0.22) and a higher complication rate (46.7% vs 30%, p = 0.21). There was no difference in length of stay between the HIV+ and HIV- cohort (12 days vs. 15.5 days, p = 0.916). Burns are a significant yet preventable cause of mortality and morbidity. The rising number of shack fires, responsible for extensive burns and resultant mortality is concerning and indicates urgent attention and action. HIV complicates the recovery from burn and is responsible for an increased rate of in hospital mortality. Copyright © 2016 Elsevier Ltd and ISBI

PMID

27658998 [<http://www.ncbi.nlm.nih.gov/pubmed/?term=27658998>]

Institution

(Cloake, Haigh, Cheshire, Walker) University of Birmingham Medical School, University of Birmingham, United Kingdom

Publisher

Elsevier Ltd

Link to the Ovid Full Text or citation:

[Click here for full text options](http://ovidsp.ovid.com/ovidweb.cgi?T=JS&CSC=Y&NEWS=N&PAGE=fulltext&D=emed18b&AN=614262868)

Link to the External Link Resolver:

[SFX](http://whel-primo.hosted.exlibrisgroup.com/openurl/44WHELF_CAR/44WHELF_CAR_services_page?sid=OVID:embase&id=pmid:27658998&id=doi:10.1016%2Fj.burns.2016.08.031&issn=0305-4179&isbn=&volume=43&issue=2&spage=411&pages=411-416&date=2017&title=Burns&atitle=The+impact+of+patient+demographics+and+comorbidities+upon+burns+admitted+to+Tygerberg+Hospital+Burns+Unit%2C+Western+Cape%2C+South+Africa&aulast=Cloake&pid=<author>Cloake+T.%3BHaigh+T.%3BCheshire+J.%3BWalker+D.<%2Fauthor><AN>614262868<%2FAN><DT>Journal%3A+Article<%2FDT>)

9.

Particularities of a cohort of preXDR / XDR TB patients treated with group 5 medication, hospitalized in MDR TB Department Institute of Pneumophtisiology Bucharest, Romania.

Popescu G., Popa C., Spinu V., Cristian P.

European Respiratory Journal. Conference: European Respiratory Society Annual Congress 2016. United Kingdom. Conference Start: 20160903. Conference End: 20160907. 48 (no pagination), 2016. Date of Publication: 01 Sep 2016.

AN: 614780926

PreXDR and XDRTB (second line injectables SLI+/-fluoroquinolone FQ added to HR resistance) is a crucial Romanian issue since TB group 5 medicines are accessible only through donor-funded programs. 35 patients were hospitalized in 6 months (July 2015-Feb 2016). Most were middle age males (68.5%, 45.7 ys), from rural area (75%). 34 patients had pulmonary TB-1 new case,4 relapses,29 failures to individualized treatment;1 had new extrapulmonary XDRTB (hepatic and splenic abscess). Previous period of treatment was 3.4 ys in average. All pts had a drug sensitivity test, registering preXDR SLI(5.7%), preXDR FQ(11.4%) and XDR (82.9%). 4 patients had previous surgical treatment (lobectomy, pneumonectomy). Comorbidities associated with decrease imunity were HIV, diabetes, neoplasia. Treatment regimen associated moxifloxacine(100%), SLI(capreomicine 88.5%, kanamicine 5.7%, amikacine 2.8%), second line TBdrugs (cycloserine 82.9%, prothionamide 65.7%, PAS 5.7%) and group 5 TB drugs (linezolid 97,1%, clofazimine 100%, imipenem+cilastatin 85.7%, amoxicilina+clavulanat 85.7%, bedaquiline 22.4%). We notified some adverse events such as digestive problems, liver cytolysis, peripheral neuropathy. No QTc prolongation was registered at bedaquiline treated group. If we considere only the contagious patients at hospital admission,78.2% became smear negative, after 2 months of treatment. At 3 months of treatment, 48,4% were culture negative. These first stage encouraging results may become evidence to support the stakeholders to insure availability of group 5 medicines as XDR TB is a Romanian recognized milestone public health problem.

Publisher

European Respiratory Society

Link to the Ovid Full Text or citation:

[Click here for full text options](http://ovidsp.ovid.com/ovidweb.cgi?T=JS&CSC=Y&NEWS=N&PAGE=fulltext&D=emed18b&AN=614780926)

Link to the External Link Resolver:

[SFX](http://whel-primo.hosted.exlibrisgroup.com/openurl/44WHELF_CAR/44WHELF_CAR_services_page?sid=OVID:embase&id=pmid:&id=doi:10.1183%2F13993003.congress-2016.PA2680&issn=1399-3003&isbn=&volume=48&issue=60&spage=no+pagination&pages=no+pagination&date=2016&title=European+Respiratory+Journal&atitle=Particularities+of+a+cohort+of+preXDR+%2F+XDR+TB+patients+treated+with+group+5+medication%2C+hospitalized+in+MDR+TB+Department+Institute+of+Pneumophtisiology+Bucharest%2C+Romania&aulast=Popescu&pid=<author>Popescu+G.%3BPopa+C.%3BSpinu+V.%3BCristian+P.<%2Fauthor><AN>614780926<%2FAN><DT>Journal%3A+Conference+Abstract<%2FDT>)

10.

Risk factors and treatment outcomes in patients with chronic pulmonary aspergillosis.

Agarwal K., Chowdhary A., Rahman M., Chaudhry A., Gaur S.N.

European Respiratory Journal. Conference: European Respiratory Society Annual Congress 2016. United Kingdom. Conference Start: 20160903. Conference End: 20160907. 48 (no pagination), 2016. Date of Publication: 01 Sep 2016.

AN: 614779718

Background: Chronic pulmonary aspergillosis (CPA) is still under-diagnosed in high tuberculosis (TB) burden areas like India where similar clinical presentation and disregard for underlying risk factors lead to its misdiagnosis as the latter. Aim: To evaluate patient characteristics and treatment outcomes in CPA patients Methods: Consecutive patients with a differential diagnosis of CPA enrolled from Jan. 2012-Dec. 2013 were administered oral itraconazole (ITZ) or voriconazole. Weight, BMI, haemoglobin (Hb), total leucocyte count (TLC), specific IgG, precipitins, CRP, sputum fungus and radiology were followed till 2 years after the end of treatment. Negative specific IgG was taken as end-point of therapy. Results: CPA was diagnosed in 24(70.5%) patients including 10 CCPA, 9 CNPA, 3 CFPA and 2 simple aspergilloma. Mean age was 47.2+/-10.9years. Average 2.1 risk factors were present per patient; TB was the commonest risk factor (29.3%) but ABPA, sarcoidosis and diabetes mellitus were significantly associated with development of CPA. Cavitation was noted in 29% and aspergilloma in 12%. Specific IgG and serum precipitins were raised in 79% and CRP 92%. A. fumigatus was isolated in 80%. Both drugs exerted similar but significant improvements in weight (51.8+/-7 vs 56.3+/-8 Kg), BMI (18.7+/-3.1 vs 20.3+/-3.0 Kg/m2), Hb (11.3+/-1.9 vs 12.3+/-1.7 g/dL), TLC (10.2+/-3.1 vs 7.9+/-2.6x109/muL), precipitins and specific IgG (69.1; 26.5-84.2 vs 29.6; 0-39.3 MgA/L). Two cases on ITZ relapsed at 12 months; no deaths were reported. 2 3 Conclusions: CPA should be investigated in patients with multiple risk factors. Triazole antifungals are effective in treatment. Specific IgG titers can be used to assess disease activity and response to treatment.

Publisher

European Respiratory Society

Link to the Ovid Full Text or citation:

[Click here for full text options](http://ovidsp.ovid.com/ovidweb.cgi?T=JS&CSC=Y&NEWS=N&PAGE=fulltext&D=emed18b&AN=614779718)

Link to the External Link Resolver:

[SFX](http://whel-primo.hosted.exlibrisgroup.com/openurl/44WHELF_CAR/44WHELF_CAR_services_page?sid=OVID:embase&id=pmid:&id=doi:10.1183%2F13993003.congress-2016.PA2647&issn=1399-3003&isbn=&volume=48&issue=60&spage=no+pagination&pages=no+pagination&date=2016&title=European+Respiratory+Journal&atitle=Risk+factors+and+treatment+outcomes+in+patients+with+chronic+pulmonary+aspergillosis&aulast=Agarwal&pid=<author>Agarwal+K.%3BChowdhary+A.%3BRahman+M.%3BChaudhry+A.%3BGaur+S.N.<%2Fauthor><AN>614779718<%2FAN><DT>Journal%3A+Conference+Abstract<%2FDT>)

11.

Successful TB treatment outcome and its associated factors among TB/HIV co-infected patients attending Gondar University Referral Hospital, Northwest Ethiopia: An institution based cross-sectional study.

Sinshaw Y., Alemu S., Fekadu A., Gizachew M.

BMC Infectious Diseases. 17 (1) (no pagination), 2017. Article Number: 132. Date of Publication: 08 Feb 2017.

AN: 614343137

Background: Tuberculosis/Human immunodeficiency virus (TB/HIV) co-infection is bidirectional and synergistic which mainly affects interventions that have been taken on the area. Tb patients co-infected with HIV have poorer treatment outcome as compared to non-co-infected patients. There is limited information regarding successful TB treatment outcomes and its associated factors; a reason that this study was planned to investigate. Methods: An institution based cross sectional study was carried out from July 2010 to January 2016. Data were abstracted from patients' medical chart using data abstraction format. The completeness of the data was checked and cleaned manually. Then, it was entered and analyzed by using SPSS version 20.0. Bi-variable and Multi-variable logistic regression model was fitted to identify factors associated with successful Tb treatment outcome. Significance was obtained through adjusted odds ratio with its 95% CI and a p < 0.05. Results: Successful TB treatment outcome among TB/HIV co-infected patients in Gondar University Hospital was 77.3% [95%CI 72.6-81.9]. Being residing in outside the Gondar town [AOR = 0.44, 95%CI: 0.25-0.80], having less than the mean baseline weight (<43.7 kg) at initiation of TB treatment [AOR = 0.51, 95% CI: 0.29-0.89], being in the bedridden condition [AOR = 0.23, 95% CI: 0.1-0.23], and experiencing anti-TB treatment side effect [AOR = 0.35, 95% CI: 0.12-0.98] were the factors that resulted the patient in treatment failure. Conclusion: Successful Tb treatment outcome among TB/HIV co-infected patients was lower than the target set by Global Plan to Stop TB 2011-2015. Strengthening collaborative TB/HIV management activities that would trace the identified factors shall be recommended to increase successful treatment outcome of TB. Copyright © 2017 The Author(s).

Institution

(Sinshaw, Alemu) University of Gondar referral Hospital, Gondar, Ethiopia (Fekadu) University of Gondar, Institute of Public Health, Department of Epidemiology and Biostatistics, Gondar, Ethiopia

(Gizachew) University of Gondar, College of Medicine and Health Science, School of Biomedical Sciences, Gondar, Ethiopia

Publisher

BioMed Central Ltd. (E-mail: info@biomedcentral.com)

Link to the Ovid Full Text or citation:

[Click here for full text options](http://ovidsp.ovid.com/ovidweb.cgi?T=JS&CSC=Y&NEWS=N&PAGE=fulltext&D=emed18b&AN=614343137)

Link to the External Link Resolver:

[SFX](http://whel-primo.hosted.exlibrisgroup.com/openurl/44WHELF_CAR/44WHELF_CAR_services_page?sid=OVID:embase&id=pmid:&id=doi:10.1186%2Fs12879-017-2238-7&issn=1471-2334&isbn=&volume=17&issue=1&spage=no+pagination&pages=no+pagination&date=2017&title=BMC+Infectious+Diseases&atitle=Successful+TB+treatment+outcome+and+its+associated+factors+among+TB%2FHIV+co-infected+patients+attending+Gondar+University+Referral+Hospital%2C+Northwest+Ethiopia%3A+An+institution+based+cross-sectional+study&aulast=Sinshaw&pid=<author>Sinshaw+Y.%3BAlemu+S.%3BFekadu+A.%3BGizachew+M.<%2Fauthor><AN>614343137<%2FAN><DT>Journal%3A+Article<%2FDT>)

12.

Tuberculosis-diabetes epidemiology in the border and non-border regions of Tamaulipas, Mexico.

Abdelbary B.E., Garcia-Viveros M., Ramirez-Oropesa H., Rahbar M.H., Restrepo B.I.

Tuberculosis. 101 (pp S124-S134), 2016. Date of Publication: 01 Dec 2016.

AN: 612650444

Type 2 diabetes mellitus (DM) is a re-emerging risk factor for TB development and adverse TB outcomes. As a follow-up of our previous study in 1998-2004, we reassessed prevalence of DM and its associated factors among 8431 TB patients using surveillance data from 2006 to 2013 for the Mexican state of Tamaulipas, across the border with Texas. Prevalence of DM was 25.2%, with an increase of at least 2.8% over the study period. Newly discovered factors associated with TB-DM (versus no DM) were lower education and higher unemployment (p < 0.001), which are reportedly associated with poorer DM management. At least 15% of the DM patients were newly-diagnosed and younger than those previously diagnosed, showing the importance of early DM diagnosis at TB clinics. TB-DM patients were more likely to have smear-positive, pulmonary (versus extra-pulmonary) and drug-resistant TB (1.9-, 3.8- and 1.4-fold, respectively). During treatment, TB-DM patients were more likely to be smear-positive, and less likely to die or abandon TB treatment. Thus, the increasing prevalence of DM among TB, and its association with low education, features of a more contagious TB, and drug resistance, highlight the need for design of TB management programs in DM patients, blood testing of all new TB patients for DM, and if positive for DM, testing for drug resistance. Copyright © 2016 Elsevier Ltd

Institution

(Abdelbary, Restrepo) Department of Epidemiology, Human Genetics and Environmental Sciences, School of Public Health in Brownsville, University of Texas Health Science Center at Houston, Brownsville, TX, United States (Garcia-Viveros, Ramirez-Oropesa) Secretaria de Salud de Tamaulipas, Ciudad Victoria and Matamoros, Mexico

(Rahbar) Department of Epidemiology, Human Genetics and Environmental Sciences, School of Public Health at Houston, University of Texas Health Science Center at Houston, TX, United States

(Rahbar) Division of Clinical and Translational Sciences, Department of Internal Medicine, McGovern Medical School, The Center for Clinical and Translational Sciences, University of Texas Health Science Center at Houston, TX, United States

Publisher

Churchill Livingstone

Link to the Ovid Full Text or citation:

[Click here for full text options](http://ovidsp.ovid.com/ovidweb.cgi?T=JS&CSC=Y&NEWS=N&PAGE=fulltext&D=emed18b&AN=612650444)

Link to the External Link Resolver:

[SFX](http://whel-primo.hosted.exlibrisgroup.com/openurl/44WHELF_CAR/44WHELF_CAR_services_page?sid=OVID:embase&id=pmid:&id=doi:10.1016%2Fj.tube.2016.09.024&issn=1472-9792&isbn=&volume=101&issue=&spage=S124&pages=S124-S134&date=2016&title=Tuberculosis&atitle=Tuberculosis-diabetes+epidemiology+in+the+border+and+non-border+regions+of+Tamaulipas%2C+Mexico&aulast=Abdelbary&pid=<author>Abdelbary+B.E.%3BGarcia-Viveros+M.%3BRamirez-Oropesa+H.%3BRahbar+M.H.%3BRestrepo+B.I.<%2Fauthor><AN>612650444<%2FAN><DT>Journal%3A+Article<%2FDT>)

13.

Impact of type of calcineurin inhibitor on post-transplant tuberculosis: Single-center study from India.

Agarwal S.K., Bhowmik D., Mahajan S., Bagchi S.

Transplant Infectious Disease. 19 (1) (no pagination), 2017. Article Number: e12626. Date of Publication: 01 Feb 2017.

AN: 613850784

Introduction: Tuberculosis (TB) is an important cause of morbidity and mortality in renal transplant recipients. Immunosuppressive drugs are one of the most important risk factor for post-transplant tuberculosis (PTTB). A paucity of data exists about the impact of the type of calcineurin inhibitor on PTTB. Methods: In this retrospective study, all adult patients on calcineurin inhibitor-based immunosuppression were included. Patients receiving TB chemoprophylaxis were excluded. Diabetes, duration of dialysis, hepatitis B and C, past treated TB, induction therapy, type of antimetabolite, acute rejection, new onset of diabetes after renal transplantation (RT) (NODAT) and cytomegalovirus (CMV) were analyzed in tacrolimus (Tac) and cyclosporine (CsA) groups. Primary outcome was incidence of TB and secondary outcomes were timeline of development of TB after RT and pattern of TB in the two groups. Results: Of the 1664 patients included, 582 patients received CsA-based immunosuppression while 1082 received Tac-based immunosuppression. Duration of dialysis, positive tuberculin skin test, use of induction, mycophenolate mofetil use, CMV infection, and NODAT were significantly more, and hepatitis B infection, past treated TB, and acute rejection episodes were significantly less in the Tac group. At the end of follow-up, incidence of TB in the Tac group was significantly less than in the CsA group (6.1% vs 19.9%, P<.001). Mean time for development of TB after RT was similar in both the groups and nodal and disseminated TB were more common in the Tac group. Conclusion: In conclusion, our study shows that use of Tac as compared to CsA significantly decreases incidence of PTTB. Time of infection since transplant was similar in both the groups. However, nodal and disseminated TB were more common in the Tac group. Copyright © 2016 John Wiley & Sons A/S. Published by John Wiley & Sons Ltd

Institution

(Agarwal, Bhowmik, Mahajan, Bagchi) Department of Nephrology, All India Institute of Medical Sciences, New Delhi, India

Publisher

Blackwell Publishing Inc. (E-mail: subscrip@blackwellpub.com)

Link to the Ovid Full Text or citation:

[Click here for full text options](http://ovidsp.ovid.com/ovidweb.cgi?T=JS&CSC=Y&NEWS=N&PAGE=fulltext&D=emed18b&AN=613850784)

Link to the External Link Resolver:

[SFX](http://whel-primo.hosted.exlibrisgroup.com/openurl/44WHELF_CAR/44WHELF_CAR_services_page?sid=OVID:embase&id=pmid:&id=doi:10.1111%2Ftid.12626&issn=1398-2273&isbn=&volume=19&issue=1&spage=no+pagination&pages=no+pagination&date=2017&title=Transplant+Infectious+Disease&atitle=Impact+of+type+of+calcineurin+inhibitor+on+post-transplant+tuberculosis%3A+Single-center+study+from+India&aulast=Agarwal&pid=<author>Agarwal+S.K.%3BBhowmik+D.%3BMahajan+S.%3BBagchi+S.<%2Fauthor><AN>613850784<%2FAN><DT>Journal%3A+Article<%2FDT>)

14.

Early interventions for diabetes related tuberculosis associate with hastened sputum microbiological clearance in Virginia, USA.

Alkabab Y., Keller S., Dodge D., Houpt E., Staley D., Heysell S.

BMC Infectious Diseases. 17 (1) (no pagination), 2017. Article Number: 125. Date of Publication: 06 Feb 2017.

AN: 614295261

Background: Diabetes complicates tuberculosis (TB) treatment including a prolonged time of sputum culture conversion to negative growth. Since 2013 in Virginia, interventions early in the treatment course have used therapeutic drug monitoring and dose correction for isoniazid and rifampin after 2 weeks of TB treatment in patients with diabetes along with nurse manager initiated diabetes education and linkage to care. Methods: A retrospective cohort study of the state TB registry was performed for patients initiating drug-susceptible pulmonary TB treatment that were matched for age, gender, chest imaging and sputum smear status to compare time to sputum culture conversion and other clinical outcomes in the pre-and post-intervention groups. Results: Three hundred sixty-three patients had documented time to sputum culture conversion in the pre-and post-intervention periods, including 56 (15%) with diabetes. Seventy-four (57%) of all patients with diabetes were >60 years of age at treatment initiation. Twenty-six patients with diabetes were matched in each group. Mean time to sputum culture conversion in the post-intervention group was 42 +/- 22 days compared to the pre-intervention group of 62 +/- 31 days (p = 0.01). In the post-intervention group 21 (80%) of patients with diabetes had culture conversion by 2 months compared to 13 (50%) in the pre-intervention group (p = 0.04). Conclusions: Early interventions for diabetes related TB in the programmatic setting may hasten sputum culture conversion. Copyright © 2017 The Author(s).

Institution

(Alkabab, Houpt, Heysell) University of Virginia School of Medicine, Division of Infectious Diseases and International Health, P.O. Box 801340, Charlottesville, VA 22908, United States (Keller) TB Control, Virginia Department of Health, Division of Disease Prevention, 109 Governor Street, Richmond, VA 23219, United States

(Dodge, Staley) Virginia Department of Health, TB Control and Newcomer Health, 109 Governor Street, Room 326, PO Box 2448, Richmond, VA 23218, United States

Publisher

BioMed Central Ltd. (E-mail: info@biomedcentral.com)

Link to the Ovid Full Text or citation:

[Click here for full text options](http://ovidsp.ovid.com/ovidweb.cgi?T=JS&CSC=Y&NEWS=N&PAGE=fulltext&D=emed18b&AN=614295261)

Link to the External Link Resolver:

[SFX](http://whel-primo.hosted.exlibrisgroup.com/openurl/44WHELF_CAR/44WHELF_CAR_services_page?sid=OVID:embase&id=pmid:&id=doi:10.1186%2Fs12879-017-2226-y&issn=1471-2334&isbn=&volume=17&issue=1&spage=no+pagination&pages=no+pagination&date=2017&title=BMC+Infectious+Diseases&atitle=Early+interventions+for+diabetes+related+tuberculosis+associate+with+hastened+sputum+microbiological+clearance+in+Virginia%2C+USA&aulast=Alkabab&pid=<author>Alkabab+Y.%3BKeller+S.%3BDodge+D.%3BHoupt+E.%3BStaley+D.%3BHeysell+S.<%2Fauthor><AN>614295261<%2FAN><DT>Journal%3A+Article<%2FDT>)

15.

Clinical outcomes of kidney transplants on patients with end-stage renal disease secondary to lupus nephritis, polycystic kidney disease and diabetic nephropathy. <Resultados clinicos de trasplantes de rinon en pacientes con enfermedad renal en etapa terminal secundaria a nefritis lupus, enfermedad poliquistica renal y nefropatia diabetica.>

Nieto-Rios J.F., Serna-Higuita L.M., Builes-Rodriguez S.A., Restrepo-Correa R.C., Aristizabal-Alzate A., Ocampo-Kohn C., Serna-Campuzano A., Cardona-Diaz N., Giraldo-Ramirez N.D., Zuluaga-Valencia G.A.

Colombia Medica. 47 (1) (pp 51-58), 2016. Date of Publication: January-March 2016.

AN: 609639332

Background: Patients with lupus nephritis could progress to endstage renal disease (10-22%); hence, kidney transplants should be considered as the treatment of choice for these patients. Objective: To evaluate the clinical outcomes after kidney transplants in patients with chronic kidney diseases secondary to lupus nephritis, polycystic kidney disease and diabetes nephropathy at Pablo Tobon Uribe Hospital. Methods: A descriptive and retrospective study performed at one kidney transplant center between 2005 and 2013. Results: A total of 136 patients, 27 with lupus nephritis (19.9%), 31 with polycystic kidney disease (22.8%) and 78 with diabetes nephropathy (57.4%), were included in the study. The graft survivals after one, three and five years were 96.3%, 82.5% and 82.5% for lupus nephritis; 90%, 86% and 76.5% for polycystic kidney disease and 91.7%, 80.3% and 67.9% for diabetes nephropathy, respectively, with no significant differences (p= 0.488); the rate of lupus nephritis recurrence was 0.94%/person-year. The etiology of lupus vs diabetes vs polycystic disease was not a risk factor for a decreased time of graft survival (Hazard ratio: 1.43; 95% CI: 0.52-3.93). Conclusion: Kidney transplant patients with end stage renal disease secondary to lupus nephritis has similar graft and patient survival success rates to patients with other kidney diseases. The complication rate and risk of recurrence for lupus nephritis are low. Kidney transplants should be considered as the treatment of choice for patients with end stage renal disease secondary to lupus nephritis. Copyright © 2016. Universidad del Valle.

PMID

27226665 [<http://www.ncbi.nlm.nih.gov/pubmed/?term=27226665>]

Institution

(Nieto-Rios, Serna-Higuita, Aristizabal-Alzate, Ocampo-Kohn, Cardona-Diaz, Giraldo-Ramirez, Zuluaga-Valencia) Seccion Nefrologia, Hospital Pablo Tobon Uribe, Medellin, Colombia (Nieto-Rios, Aristizabal-Alzate, Ocampo-Kohn, Zuluaga-Valencia) Seccion de Nefrologia, Universidad de Antioquia, Medellin, Colombia

(Serna-Higuita) Seccion de Pediatria y Puericultura, Universidad de Antioquia, Medellin, Colombia

(Builes-Rodriguez, Serna-Campuzano) Seccion de Nefrologia, Universidad Pontificia Bolivariana, Medellin, Colombia

(Restrepo-Correa) Seccion de Medicina Interna, Hospital Pablo Tobon Uribe, Medellin, Colombia

Publisher

Facultad de Salud de la Universidad del Valle (E-mail: comedica@univalle.edu.co)

Link to the Ovid Full Text or citation:

[Click here for full text options](http://ovidsp.ovid.com/ovidweb.cgi?T=JS&CSC=Y&NEWS=N&PAGE=fulltext&D=emed18b&AN=609639332)

Link to the External Link Resolver:

[SFX](http://whel-primo.hosted.exlibrisgroup.com/openurl/44WHELF_CAR/44WHELF_CAR_services_page?sid=OVID:embase&id=pmid:27226665&id=doi:&issn=0120-8322&isbn=&volume=47&issue=1&spage=51&pages=51-58&date=2016&title=Colombia+Medica&atitle=Resultados+clinicos+de+trasplantes+de+rinon+en+pacientes+con+enfermedad+renal+en+etapa+terminal+secundaria+a+nefritis+lupus%2C+enfermedad+poliquistica+renal+y+nefropatia+diabetica&aulast=Nieto-Rios&pid=<author>Nieto-Rios+J.F.%3BSerna-Higuita+L.M.%3BBuiles-Rodriguez+S.A.%3BRestrepo-Correa+R.C.%3BAristizabal-Alzate+A.%3BOcampo-Kohn+C.%3BSerna-Campuzano+A.%3BCardona-Diaz+N.%3BGiraldo-Ramirez+N.D.%3BZuluaga-Valencia+G.A.<%2Fauthor><AN>609639332<%2FAN><DT>Journal%3A+Article<%2FDT>)

16.

Factors affecting patient outcome in primary cutaneous aspergillosis.

Tatara A.M., Mikos A.G., Kontoyiannis D.P.

Medicine (United States). 95 (26) (no pagination), 2016. Article Number: e3747. Date of Publication: 01 Jun 2016.

AN: 611303108

Primary cutaneous aspergillosis (PCA) is an uncommon infection of the skin. There is a paucity of organized literature regarding this entity in regard to patient characteristics, associated Aspergillus species, and treatment modalities on outcome (disease recurrence, disease dissemination, and mortality). We reviewed all published reports of PCA from 1967 to 2015. Cases were deemed eligible if they included the following: patient baseline characteristics (age, sex, underlying condition), evidence of proven or probable PCA, primary treatment strategy, and outcome. We identified 130 eligible cases reported from 1967 to 2015. The patients were predominantly male (63.8%) with a mean age of 30.4 +/- 22.1 years. Rates of PCA recurrence, dissemination, and mortality were 10.8%, 18.5%, and 31.5%, respectively. In half of the cases, there was an association with a foreign body. Seven different Aspergillus species were reported to cause PCA. Systemic antifungal therapy without surgery was the most common form of therapy (60% of cases). Disease dissemination was more common in patients with underlying systemic conditions and occurred on average 41.4 days after PCA diagnosis (range of 3-120 days). In a multivariate linear regression model of mortality including only patients with immunosuppressive conditions, dissemination and human immunodeficiency virus/acquired immune deficiency syndrome were statistically significantly associated with increased mortality. Nearly one-third of patients with PCA die with the disease. Dissemination and host status are critical in patient outcome. Copyright © 2016 Wolters Kluwer Health, Inc. All rights reserved.

PMID

27367980 [<http://www.ncbi.nlm.nih.gov/pubmed/?term=27367980>]

Institution

(Tatara, Mikos) Department of Bioengineering, Rice University, United States (Tatara, Kontoyiannis) Department of Infectious Diseases Infection Control and Employee Health, Unit 1416, University of Texas MD Anderson Cancer Center, 1515 Holcombe Boulevard, Houston, TX 77030, United States

Publisher

Lippincott Williams and Wilkins (E-mail: kathiest.clai@apta.org)

Link to the Ovid Full Text or citation:

[Click here for full text options](http://ovidsp.ovid.com/ovidweb.cgi?T=JS&CSC=Y&NEWS=N&PAGE=fulltext&D=emed18b&AN=611303108)

Link to the External Link Resolver:

[SFX](http://whel-primo.hosted.exlibrisgroup.com/openurl/44WHELF_CAR/44WHELF_CAR_services_page?sid=OVID:embase&id=pmid:27367980&id=doi:10.1097%2FMD.0000000000003747&issn=0025-7974&isbn=&volume=95&issue=26&spage=no+pagination&pages=no+pagination&date=2016&title=Medicine+(United+States)&atitle=Factors+affecting+patient+outcome+in+primary+cutaneous+aspergillosis&aulast=Tatara&pid=<author>Tatara+A.M.%3BMikos+A.G.%3BKontoyiannis+D.P.<%2Fauthor><AN>611303108<%2FAN><DT>Journal%3A+Article<%2FDT>)

17.

Treatment outcomes of fixed-dose combination versus separate tablet regimens in pulmonary tuberculosis patients with or without diabetes in Qatar.

Al-Shaer M.H., Mansour H., Elewa H., Salameh P., Iqbal F.

BMC Infectious Diseases. 17 (1) (no pagination), 2017. Article Number: 118. Date of Publication: 02 Feb 2017.

AN: 614259878

Background: Tuberculosis is considered the second most common cause of death due to infectious agent. The currently preferred regimen for treatment of pulmonary tuberculosis (PTB) is isoniazid, rifampin, pyrazinamide, and ethambutol, which has been used either as separate tablets (ST) or as fixed-dose combination (FDC). To date, no studies have compared both regimens in Qatar. We aim to evaluate the safety and effectiveness of FDC and ST regimen for treating PTB, in addition to comparing safety and efficacy of FDC and ST regimens in patients with diabetes treated for TB. Methods: A retrospective observational study was conducted in two general hospitals in Qatar. Patients diagnosed with PTB received anti-tuberculosis medications (either as FDC or ST) administered by the nurse. Sputum smears were tested weekly. We assessed the time to negative sputum smear and incidence of adverse events among FDC and ST groups. Results: The study included 148 patients. FDC was used in 90 patients (61%). Effectiveness was not different between FDC and ST regimens as shown by mean time to sputum conversion (29.9+/-18.3 vs. 35.6+/-23days, p=0.12). Similarly, there was no difference in the incidence of adverse events, except for visual one that was higher in ST group. Among the 33 diabetic patients, 19 received the FDC and had faster sputum conversion compared to those who received ST (31+/-12 vs. 49.4+/-30.9days, p=0.05). Overall, diabetic patients needed longer time for sputum conversion and had more hepatotoxic and gastric adverse events compared to non-diabetics. Conclusion: ST group had higher visual side effects compared to FDC. FDC may be more effective in diabetic patients; however, further studies are required to confirm such finding. Copyright © 2017 The Author(s).

Institution

(Al-Shaer) Al Wakra Hospital - Hamad Medical Corporation, Department of Pharmacy, Doha, Qatar (Al-Shaer) University of Florida, Department of Pharmacotherapy and Translational Research, Infectious Disease Pharmacokinetics Laboratory, College of Pharmacy and Emerging Pathogens Institute, Gainesville, FL 32610, United States

(Mansour, Salameh) Lebanese American University, School of Pharmacy, P.O Box 36-S 23, Byblos, Lebanon

(Elewa) Qatar University, College of Pharmacy, Doha, Qatar

(Salameh) Lebanese University, Faculty of Medical Sciences, Beirut, Lebanon

(Iqbal) Rumailah Hospital - Hamad Medical Corporation, Department of Pharmacy, Doha, Qatar

Publisher

BioMed Central Ltd. (E-mail: info@biomedcentral.com)

Link to the Ovid Full Text or citation:

[Click here for full text options](http://ovidsp.ovid.com/ovidweb.cgi?T=JS&CSC=Y&NEWS=N&PAGE=fulltext&D=emed18b&AN=614259878)

Link to the External Link Resolver:

[SFX](http://whel-primo.hosted.exlibrisgroup.com/openurl/44WHELF_CAR/44WHELF_CAR_services_page?sid=OVID:embase&id=pmid:&id=doi:10.1186%2Fs12879-017-2231-1&issn=1471-2334&isbn=&volume=17&issue=1&spage=no+pagination&pages=no+pagination&date=2017&title=BMC+Infectious+Diseases&atitle=Treatment+outcomes+of+fixed-dose+combination+versus+separate+tablet+regimens+in+pulmonary+tuberculosis+patients+with+or+without+diabetes+in+Qatar&aulast=Al-Shaer&pid=<author>Al-Shaer+M.H.%3BMansour+H.%3BElewa+H.%3BSalameh+P.%3BIqbal+F.<%2Fauthor><AN>614259878<%2FAN><DT>Journal%3A+Article<%2FDT>)

18.

Tuberculosis management continues to utilize a large amount of hospital resources in the United States.

Rampa S., Allareddy V., Nalliah R.P.

Clinical Respiratory Journal. 11 (1) (pp 21-27), 2017. Date of Publication: 01 Jan 2017.

AN: 604120759

Background and Aims: The objective of this study is to examine longitudinal trends in hospital admissions attributed to tuberculosis and resulting hospitalization outcomes in the United States for the years 2000-2010. Methods: We used the Nationwide Inpatient Sample, which is the largest all-payer and nationally representative in-hospital dataset in the United States. All hospitalizations that had a primary diagnosis for tuberculosis were selected for analysis. Patient characteristics and outcomes including discharge status following hospitalization, length of stay in hospital and hospitalization charges were examined. Results: During the study period, a total of 96 431 hospitalizations occurred due to tuberculosis. The mean age of hospitalizations was 48.6 years. Males comprised 64.2% of all hospitalizations; 24.8% were Whites, 25.5% Blacks, 26.5% Hispanics, 14.3% Asians/Pacific Islanders, 1% Native Americans, and 7.9% other/mixed races. Following hospitalization, 72.1% were discharged routinely, 3.4% were transferred to another acute-care hospital, 10.7% to long-term care facilities including skilled nursing facilities, 7.6% to home health care, and 2.1% were discharged against medical advice. There were 3815 patients who died (4% of hospitalizations). The total hospitalization charge for this cohort of patients admitted due to tuberculosis across the United States was $6.96 billion and the total hospitalization days over study period was 1 419 605 days. Conclusion: High-risk cohorts who are likely to be hospitalized due to tuberculosis included Blacks and Hispanics. Majority of hospitalization comprised of males. Even though the annual number of hospitalizations reduced over the study period, substantial amounts of resources are used in hospital settings to manage tuberculosis. Copyright © 2015 John Wiley & Sons Ltd

Institution

(Allareddy) College of Dentistry, The University of Iowa, Iowa City, IA, United States (Rampa) College of Public Health, University of Nebraska Medical Center, Omaha, NE, United States

(Allareddy) Department of Pediatric Critical Care and Pharmacology, Rainbow Babies and Children's Hospital, University Hospitals, Case Medical Center, Cleveland, OH, United States

(Nalliah) Office of Global Health, Harvard School of Dental Medicine, Boston, MA, United States

Publisher

Blackwell Publishing Ltd (E-mail: customerservices@oxonblackwellpublishing.com)

Link to the Ovid Full Text or citation:

[Click here for full text options](http://ovidsp.ovid.com/ovidweb.cgi?T=JS&CSC=Y&NEWS=N&PAGE=fulltext&D=emed18b&AN=604120759)

Link to the External Link Resolver:

[SFX](http://whel-primo.hosted.exlibrisgroup.com/openurl/44WHELF_CAR/44WHELF_CAR_services_page?sid=OVID:embase&id=pmid:&id=doi:10.1111%2Fcrj.12296&issn=1752-6981&isbn=&volume=11&issue=1&spage=21&pages=21-27&date=2017&title=Clinical+Respiratory+Journal&atitle=Tuberculosis+management+continues+to+utilize+a+large+amount+of+hospital+resources+in+the+United+States&aulast=Rampa&pid=<author>Rampa+S.%3BAllareddy+V.%3BNalliah+R.P.<%2Fauthor><AN>604120759<%2FAN><DT>Journal%3A+Article<%2FDT>)

19.

The spectrum and outcome of surgical sepsis in pietermaritzburg, South Africa.

Green S., Kong V.Y., Clarke D.L., Sartorius B., Odendaal J., Bruce J.L., Laing G.L., Brysiewicz P., Bekker W., Harknett E.

South African Medical Journal. 107 (2) (pp 134-136), 2017. Date of Publication: February 2017.

AN: 614389395

Background. Sepsis is a leading cause of morbidity and mortality worldwide, and the incidence appears to be increasing. In the resource-limited environment in low- and middle-income countries, the management of surgical sepsis (SS) continues to represent a significant portion of the workload for most general surgeons. Objective. To describe the spectrum of SS seen at a busy emergency department, and categorise the outcomes. Methods. The Pietermaritzburg Metropolitan Trauma Service (PMTS) and Pietermaritzburg Metropolitan Surgical Service (PMSS) in KwaZulu-Natal Province, South Africa (SA), maintain a prospective electronic registry. All patients with features of sepsis among emergency general surgical patients >15 years of age admitted to the PMSS over the period January 2012 - January 2015 were identified. From this cohort, all patients with sepsis that required surgical source control or who had a documented surgical source of sepsis (i.e. had SS) were selected for analysis. Results. Of a total of 6 020 adult surgical patients on the database, a cohort of 1 240 acute surgical patients with features of sepsis were identified, and 675 with SS were then analysed further. Of the 675 patients, 49.2% were male, and the mean age was 46 years (standard deviation (SD) 19); 47.0% presented to the PMSS directly from within the metropolitan area, while the remaining 53.0% were referred from hospitals outside the area. Physiological parameters (mean values) on presentation were as follows: systolic blood pressure 123 mmHg (standard deviation (SD) 23), respiratory rate 22 breaths/min (SD 5.2), heart rate 107 bpm (SD 19), temperature 37degreeC (SD 2) and white cell count 20 x 109/L (SD 8). Of the patients, 21.6% were known to be HIV-positive, 13.5% (91/675) were negative and 64.9% were of unknown status; 57.6% had intra-abdominal sepsis, 26.1% diabetes-related limb sepsis and the remaining 16.3% soft-tissue infections; 17.5% required intensive care unit admission, with a mean length of stay of 4 days (SD 4), and 30.7% developed complications. In this last group (n=207), a total of 313 morbidities were identified. The overall mortality rate was 12.7% (86/675). The mortality rate for intra-abdominal sepsis was 13.1%, for diabetic foot sepsis 14.2% and for necrotising fasciitis 27.3%. Conclusions. The spectrum of SS in SA is different to that seen in the developed world. Intra-abdominal sepsis is the most common SS and is overwhelmingly caused by acute appendicitis. Diabetic foot infection is a major cause of SS, reflecting the increasing burden of non-communicable chronic diseases in SA. Copyright © 2017, South African Medical Association. All rights reserved.

Institution

(Green, Kong, Clarke, Odendaal, Bruce, Laing, Bekker, Harknett) Department of Surgery, Pietermaritzburg Hospital Complex and School of Clinical Medicine, College of Health Sciences, Nelson R Mandela School of Medicine, University of KwaZulu-Natal, Durban, South Africa (Clarke) Department of Surgery, Faculty of Health Sciences, University of the Witwatersrand, Johannesburg, South Africa

(Sartorius, Brysiewicz) School of Nursing and Public Health, College of Health Sciences, Howard College Campus, University of KwaZulu-Natal, Durban, South Africa

Publisher

South African Medical Association (E-mail: publishing@samedical.org)

Link to the Ovid Full Text or citation:

[Click here for full text options](http://ovidsp.ovid.com/ovidweb.cgi?T=JS&CSC=Y&NEWS=N&PAGE=fulltext&D=emed18b&AN=614389395)

Link to the External Link Resolver:

[SFX](http://whel-primo.hosted.exlibrisgroup.com/openurl/44WHELF_CAR/44WHELF_CAR_services_page?sid=OVID:embase&id=pmid:&id=doi:10.7196%2FSAMJ.2017.v107i2.11339&issn=0256-9574&isbn=&volume=107&issue=2&spage=134&pages=134-136&date=2017&title=South+African+Medical+Journal&atitle=The+spectrum+and+outcome+of+surgical+sepsis+in+pietermaritzburg%2C+South+Africa&aulast=Green&pid=<author>Green+S.%3BKong+V.Y.%3BClarke+D.L.%3BSartorius+B.%3BOdendaal+J.%3BBruce+J.L.%3BLaing+G.L.%3BBrysiewicz+P.%3BBekker+W.%3BHarknett+E.<%2Fauthor><AN>614389395<%2FAN><DT>Journal%3A+Article<%2FDT>)

20.

Advances in immune characteristics of tuberculosis patients complicated with diabetes mellitus.

Li L.

Chinese Journal of Microbiology and Immunology (China). 36 (7) (pp 549-554), 2016. Date of Publication: 31 Jul 2016.

AN: 614370149

Diabetes mellitus(DM) rates remain highly and is increasing dramatically worldwide. Meanwhile, the incidence of tuberculosis (TB) decreased slowly. Moreover, DM is an important risk factor for TB incidence. Therefore, TB-DM comorbidity is becoming a new challenge in the field of prevention and control of TB. TB patients complicated with DM displayed distinct pathological manifestations, severity, course of disease, treatment complexity, failure rate of anti-TB treatment, death and recurrence compared with ordinary TB patients. Infection and incidence of TB is closely related to the immune function of the host. For patients with DM or hyperglycemia, the immune function is changed, which leads to the increase of the risk of TB and also affects the condition and prognosis of the disease. The present review summarize the current advances in the study of the immunological characteristics of TB-DM comorbidity, including the changes of both innate and adaptive immune responses, aiming to provide reference for the basic research and prevention and control of TB-DM comorbidity. Copyright © 2016 by the Chinese Medical Association.

Institution

(Li) Central Laboratory, Wuhan Pulmonary Hospital (Wuhan Institute for Tuberculosis Control), Wuhan 430030, China

Publisher

Society of Microbiology and Immunology

Link to the Ovid Full Text or citation:

[Click here for full text options](http://ovidsp.ovid.com/ovidweb.cgi?T=JS&CSC=Y&NEWS=N&PAGE=fulltext&D=emed18b&AN=614370149)

Link to the External Link Resolver:

[SFX](http://whel-primo.hosted.exlibrisgroup.com/openurl/44WHELF_CAR/44WHELF_CAR_services_page?sid=OVID:embase&id=pmid:&id=doi:10.3760%2Fcma.j.issn.0254-5101.2016.07.014&issn=0254-5101&isbn=&volume=36&issue=7&spage=549&pages=549-554&date=2016&title=Chinese+Journal+of+Microbiology+and+Immunology+(China)&atitle=Advances+in+immune+characteristics+of+tuberculosis+patients+complicated+with+diabetes+mellitus&aulast=Li&pid=<author>Li+L.<%2Fauthor><AN>614370149<%2FAN><DT>Journal%3A+Review<%2FDT>)

21.

Comparison of bacteriological conversion and treatment outcomes among MDR-TB patients with and without diabetes in Mexico: Preliminary data.

Munnoz-Torrico M., Luna J.C., Migliori G.B., D'Ambrosio L., Carrillo-Alduenda J.L., Villareal-Velarde H., Torres-Cruz A., Flores-Ergara H., Martinez-Mendoza D., Garcia-Sancho C., Centis R., Salazar-Lezama M.A., Perez-Padilla R.

Revista Portuguesa de Pneumologia. 23 (1) (pp 27-30), 2017. Date of Publication: 01 Jan 2017.

AN: 614203106

Diabetes mellitus (DM) is a well-known risk factor for tuberculosis (TB). However, it is not known to what extent DM affects the outcome in patients with multidrug-resistant (MDR-TB) and extensively drug-resistant TB (XDR-TB) treated with second-line anti-TB drugs. The objective of this study was to compare the microbiological evolution (sputum smear and culture conversion) and final outcomes of MDR/XDR-TB patients with and without DM, managed at the national TB reference centre in Mexico City. Results: Ninety patients were enrolled between 2010 and 2015: 73 with MDR-TB (81.1%), 11 with pre-XDR-TB (e.g. MDR-TB with additional resistance to one injectable drug or a fluoroquinolone, 12.2%) and 6 (6.7%) with XDR-TB. Out of these, 49 (54.4%) had DM and 42 (86%) were undergoing insulin treatment. No statistically significant differences were found in treatment outcomes comparing DM vs. non-DM MDR-TB cases: 18/32 (56.3%) of DM cases and 19/24 (79.2%) non DM patients achieved treatment success (p = 0.07). The time to sputum smear and culture conversion was longer (although not statistically) in patients without DM, as follows: the mean (+/-SD) time to sputum smear conversion was 53.9 (+/-31.4) days in DM patients and 65.2 (+/-34.8) days in non-DM ones (p = 0.15), while the time to culture conversion was 66.2 (+/-27.6) days for DM and 81.4 (+/-37.7) days for non-DM MDR-TB cases (p = 0.06). Conclusions: The study results support the Mexican National TB programme to strengthen itscollaboration with the DM programme, as an entry point for TB (and latent TB infection)screening and management. Copyright © 2016 Sociedade Portuguesa de Pneumologia. Published by Elsevier Espanna, S.L.U.

Institution

(Munnoz-Torrico, Villareal-Velarde, Torres-Cruz, Flores-Ergara, Martinez-Mendoza, Salazar-Lezama) Tuberculosis Clinic, National Institute of Respiratory Diseases of Mexico (INER), Mexico City, Mexico (Luna) Pneumology Department, University Hospital of Gran Canaria "Dr. Negrin", Las Palmas, Spain

(Migliori, D'Ambrosio, Centis) WHO Collaborating Centre for TB and Lug Diseases, Maugeri Institute, IRCCS, Tradate, Italy

(D'Ambrosio) Public Health Consulting Group, Lugano, Switzerland

(Carrillo-Alduenda, Perez-Padilla) Sleep Clinic, National Institute of Respiratory Diseases of Mexico (INER), Mexico City, Mexico

(Garcia-Sancho) Department of Epidemiology, National Institute of Respiratory Diseases of Mexico (INER), Mexico City, Mexico

Publisher

Elsevier Doyma (E-mail: editorial@elsevier.com)

Link to the Ovid Full Text or citation:

[Click here for full text options](http://ovidsp.ovid.com/ovidweb.cgi?T=JS&CSC=Y&NEWS=N&PAGE=fulltext&D=emed18b&AN=614203106)

Link to the External Link Resolver:

[SFX](http://whel-primo.hosted.exlibrisgroup.com/openurl/44WHELF_CAR/44WHELF_CAR_services_page?sid=OVID:embase&id=pmid:&id=doi:10.1016%2Fj.rppnen.2016.11.009&issn=2173-5115&isbn=&volume=23&issue=1&spage=27&pages=27-30&date=2017&title=Revista+Portuguesa+de+Pneumologia&atitle=Comparison+of+bacteriological+conversion+and+treatment+outcomes+among+MDR-TB+patients+with+and+without+diabetes+in+Mexico%3A+Preliminary+data&aulast=Munnoz-Torrico&pid=<author>Munnoz-Torrico+M.%3BLuna+J.C.%3BMigliori+G.B.%3BD'Ambrosio+L.%3BCarrillo-Alduenda+J.L.%3BVillareal-Velarde+H.%3BTorres-Cruz+A.%3BFlores-Ergara+H.%3BMartinez-Mendoza+D.%3BGarcia-Sancho+C.%3BCentis+R.%3BSalazar-Lezama+M.A.%3BPerez-Padilla+R.<%2Fauthor><AN>614203106<%2FAN><DT>Journal%3A+Article<%2FDT>)

22.

The effect size of type 2 diabetes mellitus on tuberculosis drug resistance and adverse treatment outcomes.

Perez-Navarro L.M., Restrepo B.I., Fuentes-Dominguez F.J., Duggirala R., Morales-Romero J., Lopez-Alvarenga J.C., Comas I., Zenteno-Cuevas R.

Tuberculosis. 103 (pp 83-91), 2017. Date of Publication: 01 Mar 2017.

AN: 614301639

Objective To evaluate the effect size of type 2 diabetes mellitus (T2DM) on tuberculosis (TB) treatment outcomes and multi drug resistance (MDR). Methods A cohort with 507 individuals with diagnosed TB included 183 with coexistence of T2DM and TB (TB-T2DM). Participants were identified at the time of TB diagnosis and followed during the course of TB treatment. Then we computed relative risks and adjustments by Cox proportional hazards for outcome variables (drug resistance, death, relapse, treatment failure), and the size of their effect as Cohen's-d. Results Patients with TB-T2DM were more likely to remain positive for acid-fast bacilli after two months of anti-TB treatment RR = [2.01 (95% CI: 1.3, 3.1)], to have drug resistant (DR) [OR 3.5 (95% CI: 1.8, 6.7)] and multi-drug resistant (MDR) TB [OR 3.5 (95% CI: 1.8, 7.1)]. The Cohen's-d for DR or MDR in T2DM was 0.69 when compared with non-DM subjects. The T2DM patients had higher odds of resistance to isoniazid (OR 3.9, 95% CI: 2.01, 7.9), rifampicin (OR 3.4, 95% CI: 1.6, 7.2) and pyrazinamide (OR 9.4, 95% CI: 2.8, 25.6), and their effect sizes were >0.67. Patients with TB-T2DM (versus no DM) were more likely to present with MDR TB (HR 3.1; 95% CI: 1.7, 5.8; p < 0.001), treatment failure (HR 2.04; 95% CI: 1.07, 3.8; p = 0.02) and relapse (HR 1.86; 95% CI: 1.09, 3.1; p = 0.02), with effect size >0.34. Conclusion T2DM showed a substantial contribution to the presence of DR or MDR-TB and to adverse clinical outcomes during and after TB treatment. Our findings support the importance for routine screening of T2DM among newly-diagnosed TB patients in order to stratify them for immediate DR assessment, and highlight the need for clinical trials to evaluate variations to the standard TB treatment in TB-T2DM to prevent adverse treatment outcomes. Copyright © 2017 Elsevier Ltd

Author NameID

Perez-Navarro, Lucia Monserrat; ORCID: <http://orcid.org/0000-0002-9281-9202> Restrepo, Blanca I.; ORCID: <http://orcid.org/0000-0002-3743-9098>

Zenteno-Cuevas, Roberto; ORCID: <http://orcid.org/0000-0001-9597-127X>

Institution

(Perez-Navarro) Nephrology Service, Research Division, Hospital General de Mexico "Dr. Eduardo Liceaga", Mexico (Perez-Navarro, Morales-Romero, Zenteno-Cuevas) Public Health Institute, University of Veracruz, Veracruz, Mexico

(Restrepo) University of Texas Health Science Center Houston, Brownsville Campus, Brownsville, TX, United States

(Fuentes-Dominguez) Mycobacteriosis State Program, Veracruz Health Services, Mexico

(Duggirala, Lopez-Alvarenga) South Texas Diabetes and Obesity Institute, University of Texas Rio Grande Valley, Edinburg, TX, United States

(Comas) Unidad de Genomica de la Tuberculosis, Instituto de Biomedicina de Valencia, Consejo Superior de Investigaciones Cientificas, Valencia, Spain

(Zenteno-Cuevas) Red multidisciplinaria de Investigacion en tuberculosis, Mexico

Publisher

Churchill Livingstone

Link to the Ovid Full Text or citation:

[Click here for full text options](http://ovidsp.ovid.com/ovidweb.cgi?T=JS&CSC=Y&NEWS=N&PAGE=fulltext&D=emed18b&AN=614301639)

Link to the External Link Resolver:

[SFX](http://whel-primo.hosted.exlibrisgroup.com/openurl/44WHELF_CAR/44WHELF_CAR_services_page?sid=OVID:embase&id=pmid:&id=doi:10.1016%2Fj.tube.2017.01.006&issn=1472-9792&isbn=&volume=103&issue=&spage=83&pages=83-91&date=2017&title=Tuberculosis&atitle=The+effect+size+of+type+2+diabetes+mellitus+on+tuberculosis+drug+resistance+and+adverse+treatment+outcomes&aulast=Perez-Navarro&pid=<author>Perez-Navarro+L.M.%3BRestrepo+B.I.%3BFuentes-Dominguez+F.J.%3BDuggirala+R.%3BMorales-Romero+J.%3BLopez-Alvarenga+J.C.%3BComas+I.%3BZenteno-Cuevas+R.<%2Fauthor><AN>614301639<%2FAN><DT>Journal%3A+Article<%2FDT>)

23.

Association between diabetes mellitus and multi-drug-resistant tuberculosis: A protocol for a systematic review and meta-analysis.

Tegegne B.S., Habtewold T.D., Mengesha M.M., Burgerhof J.G.M.

Systematic Reviews. 6 (1) (no pagination), 2017. Article Number: 6. Date of Publication: 14 Jan 2017.

AN: 614054547

Introduction: Multi-drug-resistant tuberculosis (MDR-TB) has emerged as a challenge to global tuberculosis (TB) control and remains a major public health concern in many countries. Diabetes mellitus (DM) is an increasingly recognized comorbidity that can both accelerate TB disease and complicate its treatment. The aim of this study is to summarize available evidence on the association of DM and MDR-TB among TB patients and to provide a pooled estimate of risks. Methods: All studies published in English before October 2016 will be searched using comprehensive search strings through PubMed, EMBASE, Web of Science, and WHO Global Health Library databases which have reported the association of DM and MDR-TB in adults with TB (age > =15). Two authors will independently collect detailed information using structured data abstraction form. The quality of studies will be checked using Newcastle-Ottawa Scale for cohort and case-control studies and the Agency for Healthcare Research and Quality tool for cross-sectional studies. Heterogeneity between included studies will be assessed using the I2 statistic. We will check potential publication bias by visual inspection of the funnel plot and Egger's regression test statistic. We will use the random effects model to compute a pooled estimate. Discussion: Increases in the burden of non-communicable diseases and aging populations are changing the importance of different risk factors for TB, and the profile of comorbidities and clinical challenges for people with TB. Although classic risk factors and comorbidities such as overcrowding, under-nutrition, silicosis, and HIV infection are crucial to address, chronic conditions like diabetes are important factors that impair host defenses against TB. Thus, undertaking integrated multifaceted approach is remarkably necessary for reducing the burden of DM and successful TB treatment outcome. Systematic review registration: PROSPERO CRD42016045692. Copyright © 2017 The Author(s).

Institution

(Tegegne, Mengesha) Haramaya University, Department of Public Health, College of Health and Medical Sciences, Harar, Ethiopia (Tegegne, Habtewold, Burgerhof) University of Groningen, University Medical Center Groningen, Department of Epidemiology, Groningen, Netherlands

Publisher

BioMed Central Ltd. (E-mail: info@biomedcentral.com)

Link to the Ovid Full Text or citation:

[Click here for full text options](http://ovidsp.ovid.com/ovidweb.cgi?T=JS&CSC=Y&NEWS=N&PAGE=fulltext&D=emed18b&AN=614054547)

Link to the External Link Resolver:

[SFX](http://whel-primo.hosted.exlibrisgroup.com/openurl/44WHELF_CAR/44WHELF_CAR_services_page?sid=OVID:embase&id=pmid:&id=doi:10.1186%2Fs13643-017-0407-9&issn=2046-4053&isbn=&volume=6&issue=1&spage=no+pagination&pages=no+pagination&date=2017&title=Systematic+Reviews&atitle=Association+between+diabetes+mellitus+and+multi-drug-resistant+tuberculosis%3A+A+protocol+for+a+systematic+review+and+meta-analysis&aulast=Tegegne&pid=<author>Tegegne+B.S.%3BHabtewold+T.D.%3BMengesha+M.M.%3BBurgerhof+J.G.M.<%2Fauthor><AN>614054547<%2FAN><DT>Journal%3A+Article<%2FDT>)

24.

Prevalence and Impact of Diabetes Mellitus Among Patients with Active Pulmonary Tuberculosis in South Korea.

Lee E.H., Lee J.M., Kang Y.A., Leem A.Y., Kim E.Y., Jung J.Y., Park M.S., Kim Y.S., Kim S.K., Chang J., Kim S.Y.

Lung. (pp 1-7), 2017. Date of Publication: 09 Feb 2017.

AN: 614367862

Abstract: Objective: South Korea has an increasing prevalence of diabetes and a relatively high burden of tuberculosis. We aimed to determine the prevalence of diabetes in patients with active pulmonary tuberculosis (PTB) and examine the effect of diabetes on tuberculosis treatment outcomes. Abstract: Setting and Design: Data from patients >30 years diagnosed with and treated for PTB between January 2010 and December 2012 at Severance Hospital, a 2000-bed tertiary referral hospital in Seoul, South Korea, were analyzed and compared with data from a contemporaneous general population sample extracted from KNHANES V. Abstract: Results: Diabetes prevalence was 24.2% (252/1044) among patients with PTB and 11.6% (1700/14,655) among controls. Diabetes [odds ratios (OR) 2.56, 95% confidence interval (CI) 1.56-4.21, P < 0.001], male sex (OR 1.93, 95% CI 1.08-3.44, P = 0.027), and cavitary disease (OR 2.08, 95% CI 1.29-3.35, P = 0.003) were significant risk factors for 2-month culture positivity. Diabetes was the only factor associated with unsuccessful treatment outcomes (OR 1.67, 95% CI 1.03-2.70, P = 0.039). Abstract: Conclusion: The prevalence of diabetes was markedly higher in patients with PTB than in a sample of the general South Korean population. Diabetes may delay sputum conversion and adversely affect treatment outcomes; detection and management of diabetes in patients with PTB is crucial. Copyright © 2017 Springer Science+Business Media New York

Institution

(Lee, Lee, Kang, Leem, Kim, Jung, Park, Kim, Kim, Chang, Kim) Division of Pulmonology, Department of Internal Medicine, Severance Hospital, Institute of Chest Diseases, Yonsei University College of Medicine, 50-1 Yonsei-ro, Seodaemun-gu, Seoul 120-752, South Korea

Publisher

Springer New York LLC (E-mail: barbara.b.bertram@gsk.com)

Link to the Ovid Full Text or citation:

[Click here for full text options](http://ovidsp.ovid.com/ovidweb.cgi?T=JS&CSC=Y&NEWS=N&PAGE=fulltext&D=emed18b&AN=614367862)

Link to the External Link Resolver:

[SFX](http://whel-primo.hosted.exlibrisgroup.com/openurl/44WHELF_CAR/44WHELF_CAR_services_page?sid=OVID:embase&id=pmid:&id=doi:10.1007%2Fs00408-017-9978-4&issn=0341-2040&isbn=&volume=195&issue=2&spage=1&pages=1-7&date=2017&title=Lung&atitle=Prevalence+and+Impact+of+Diabetes+Mellitus+Among+Patients+with+Active+Pulmonary+Tuberculosis+in+South+Korea&aulast=Lee&pid=<author>Lee+E.H.%3BLee+J.M.%3BKang+Y.A.%3BLeem+A.Y.%3BKim+E.Y.%3BJung+J.Y.%3BPark+M.S.%3BKim+Y.S.%3BKim+S.K.%3BChang+J.%3BKim+S.Y.<%2Fauthor><AN>614367862<%2FAN><DT>Journal%3A+Article+In+Press<%2FDT>)

25.

Identifying patients at high risk of tuberculosis recurrence.

Sadikot R.T.

International Journal of Mycobacteriology. 5 (pp S66), 2016. Date of Publication: 01 Dec 2016.

AN: 613555332

Several studies have been done in relation to recurrence of tuberculosis (TB) following completion of treatment. However, recurrence of TB is still a major problem from a public health perspective in high-burden countries, where no special attention is being given to this issue. Disease recurrence is an important indicator of the efficacy of antituberculosis treatment. The rate of recurrence is highly variable and has been estimated to range from 4.9% to 25%. This variability is not only a reflection of regional epidemiology of recurrence but differences in the definitions used by the TB control programs. In addition to treatment failure related to medication adherence, there are several key host factors that are associated with high rates of recurrence. The widely recognized host factors independent of treatment program that predispose to TB recurrence include: malnutrition; human immunodeficiency virus; substance abuse including tobacco use; comorbidity such as diabetes, renal failure and systemic diseases, especially immunosuppressive states; and environmental exposure such as silicosis. With improved understanding of the human genome, proteome, and metabolome, additional host-specific factors that predispose to recurrence are being discovered. Information on temporal and geographical trends of TB cases as well as genotyping might provide further information to enable us to fully understand TB recurrence and discriminate between reactivation and new infection. The recently launched World Health Organization End TB Strategy emphasizes the importance of integrated, patient-centered TB care. Continued improvement in diagnosis, treatment approaches, and defining host-specific factors are needed to fully understand the clinical epidemiological and social determinants of TB recurrence. Copyright © 2016

Institution

(Sadikot) Pulmonary and Critical Care Medicine, Emory University, 1670 Clairmont Rd, Atlanta, GA 30033, United States

Publisher

Elsevier Ltd

Link to the Ovid Full Text or citation:

[Click here for full text options](http://ovidsp.ovid.com/ovidweb.cgi?T=JS&CSC=Y&NEWS=N&PAGE=fulltext&D=emed18b&AN=613555332)

Link to the External Link Resolver:

[SFX](http://whel-primo.hosted.exlibrisgroup.com/openurl/44WHELF_CAR/44WHELF_CAR_services_page?sid=OVID:embase&id=pmid:&id=doi:10.1016%2Fj.ijmyco.2016.08.017&issn=2212-5531&isbn=&volume=5&issue=&spage=S66&pages=S66&date=2016&title=International+Journal+of+Mycobacteriology&atitle=Identifying+patients+at+high+risk+of+tuberculosis+recurrence&aulast=Sadikot&pid=<author>Sadikot+R.T.<%2Fauthor><AN>613555332<%2FAN><DT>Journal%3A+Conference+Paper<%2FDT>)

26.

<Diabetes is Associated with Severe Adverse Events in Multidrug-Resistant Tuberculosis.>

Munoz-Torrico M., Caminero-Luna J., Migliori G.B., D'Ambrosio L., Carrillo-Alduenda J.L., Villareal-Velarde H., Torres-Cruz A., Flores-Vergara H., Martinez-Mendoza D., Garcia-Sancho C., Centis R., Salazar-Lezama M.A., Perez-Padilla R.

Archivos de Bronconeumologia. (no pagination), 2016. Date of Publication: August 03, 2016.

AN: 614101337

Introduction: Diabetes mellitus (DM), a very common disease in Mexico, is a well-known risk factor for tuberculosis (TB). However, it is not known by which extent DM predisposes to adverse events (AE) to anti-TB drugs and/or to worse outcomes in patients with multidrug-resistant (MDR-TB) and extensively drug-resistant TB (XDR-TB). The main objective of this study was to describe the outcomes of TB treatment, the impact of DM and the prevalence of AE in a cohort of patients with MDR-/XDR pulmonary TB treated at the national TB referral centre in Mexico City. Results: Ninety patients were enrolled between 2010 and 2015: 73 with MDR-TB (81.1%), 11 with pre-XDR-TB (12.2%) and 6 (6.7%) with XDR-TB, including 49 (54.4%) with DM, and 3 with Human Immunodeficiency Virus (HIV) co-infection (3.3%). In 98% of patients, diagnosis was made by culture and drug susceptibility testing, while in a single case the diagnosis was made by a molecular test. The presence of DM was associated with an increased risk of serious drug-related AEs, such as nephrotoxicity (Odds Ratio [OR] = 6.5; 95% Confidence Interval [95% CI]: 1.9-21.8) and hypothyroidism (OR = 8.8; 95% CI: 1.8-54.2), but not for a worse outcome. Conclusions: Our data suggest that DM does not impact second-line TB treatment outcomes, but patients with DM have a higher risk of developing serious AEs to drug-resistant TB treatment, such as nephrotoxicity and hypothyroidism. Copyright © 2016 SEPAR.

Institution

(Munoz-Torrico, Villareal-Velarde, Torres-Cruz, Flores-Vergara, Martinez-Mendoza, Salazar-Lezama) Clinica de Tuberculosis, Instituto Nacional de Enfermedades Respiratorias de Mexico (INER), Ciudad de Mexico, Mexico (Caminero-Luna) Departamento de Neumologia, Hospital Universitario de Gran Canaria Dr. Negrin, Las Palmas, Canarias, Espana

(Migliori, D'Ambrosio, Centis) WHO Collaborating Centre for TB and Lung Diseases, Maugeri Institute, IRCCS, Tradate, Italia

(D'Ambrosio) Public Health Consulting Group, Lugano, Suiza

(Carrillo-Alduenda, Perez-Padilla) Clinica del Sueno, Instituto Nacional de Enfermedades Respiratorias de Mexico (INER), Ciudad de Mexico, Mexico

(Garcia-Sancho) Departamento de Epidemiologia, Instituto Nacional de Enfermedades Respiratorias de Mexico (INER), Ciudad de Mexico, Mexico

Publisher

Elsevier Doyma (E-mail: editorial@elsevier.com)

Link to the Ovid Full Text or citation:

[Click here for full text options](http://ovidsp.ovid.com/ovidweb.cgi?T=JS&CSC=Y&NEWS=N&PAGE=fulltext&D=emed18b&AN=614101337)

Link to the External Link Resolver:

[SFX](http://whel-primo.hosted.exlibrisgroup.com/openurl/44WHELF_CAR/44WHELF_CAR_services_page?sid=OVID:embase&id=pmid:&id=doi:10.1016%2Fj.arbres.2016.10.021&issn=0300-2896&isbn=&volume=&issue=&spage=no+pagination&pages=no+pagination&date=2016&title=Archivos+de+Bronconeumologia&atitle=Diabetes+is+Associated+with+Severe+Adverse+Events+in+Multidrug-Resistant+Tuberculosis&aulast=Munoz-Torrico&pid=<author>Munoz-Torrico+M.%3BCaminero-Luna+J.%3BMigliori+G.B.%3BD'Ambrosio+L.%3BCarrillo-Alduenda+J.L.%3BVillareal-Velarde+H.%3BTorres-Cruz+A.%3BFlores-Vergara+H.%3BMartinez-Mendoza+D.%3BGarcia-Sancho+C.%3BCentis+R.%3BSalazar-Lezama+M.A.%3BPerez-Padilla+R.<%2Fauthor><AN>614101337<%2FAN><DT>Journal%3A+Article+In+Press<%2FDT>)

27.

Diabetes and tuberculosis interaction in Kazakhstan.

Aifah A., El-Bassel N.

Annals of Global Health. Conference: 7th Annual CUGH Conference: Bridging to a Sustainable Future in Global Health. United States. Conference Start: 20160409. Conference End: 20160411. 82 (3) (pp 398), 2016. Date of Publication: 2016.

AN: 614044936

Background: The global increase in diabetes prevalence highlights an urgent need to address diabetes interaction with tuberculosis (TB) prevention and treatment. In Kazakhstan where multi-drug resistant TB (MDR-TB) is serious, an estimated 26% of new TB cases have MDR-TB. Studies show that for individuals with TB, co-occurring diabetes is often associated with TB treatment failure which may lead to MDR-TB. The factors associated with the synergistic relationship between TB and diabetes is understudied in Kazakhstan. The aim of this paper is to examine factors associated with the co-occurrence of diabetes and TB in Kazakhstan using data from a population based study of the Social, Environmental and Genetic Factors Determining Susceptibility to Tuberculosis project. Methods: Using a cross sectional study design, we collected data from 1600 participants consisting of TB positive individuals, household controls and community controls recruited from June 2012 to May 2014 from four regions in Kazakhstan: Almaty city, Almatinskaya oblast (province), Kyzylordinskaya oblast, and Kostanayskaya oblast. For this analysis, we focused on the TB positive individuals and used multivariate analysis to test the prevalence of co-occurring TB and diabetes as well as the associated multilevel risk factors. Findings: Of the 562 TB positive individuals 7.1% report having concomitant diabetes. A significant proportion of new TB positive cases were associated with both diabetes and smoking - accounting for 28%. We found that major risk factors associated with co-occurring TB and diabetes include: age, education and living in a rural area. Interpretation: To our knowledge, this is the first study on the cooccurrence of TB and diabetes in Kazakhstan. The high prevalence of co-occurring TB and diabetes has implications on screening index or TB positive cases as well as individuals with diabetes. National TB programs in countries like Kazakhstan should move towards integrated screenings for both diseases given the increasing prevalence of diabetes and its negative effects on TB treatment and management. Integrated screenings for both TB and diabetes in Kazakhstan is not only urgent but must address risk factors such as age, education and rural living. More evidence based research on co-occurring TB and diabetes is needed in high TB burdened settings.

Institution

(Aifah, El-Bassel) Columbia School of Social Work, New York, NY, United States

Publisher

Elsevier USA

Link to the Ovid Full Text or citation:

[Click here for full text options](http://ovidsp.ovid.com/ovidweb.cgi?T=JS&CSC=Y&NEWS=N&PAGE=fulltext&D=emed18b&AN=614044936)

Link to the External Link Resolver:

[SFX](http://whel-primo.hosted.exlibrisgroup.com/openurl/44WHELF_CAR/44WHELF_CAR_services_page?sid=OVID:embase&id=pmid:&id=doi:&issn=2214-9996&isbn=&volume=82&issue=3&spage=398&pages=398&date=2016&title=Annals+of+Global+Health&atitle=Diabetes+and+tuberculosis+interaction+in+Kazakhstan&aulast=Aifah&pid=<author>Aifah+A.%3BEl-Bassel+N.<%2Fauthor><AN>614044936<%2FAN><DT>Journal%3A+Conference+Abstract<%2FDT>)

28.

Clinical manifestations and outcomes of pulmonary aspergillosis: Experience from pakistan.

Iqbal N., Irfan M., Zubairi A.B.S., Jabeen K., Awan S., Khan J.A.

BMJ Open Respiratory Research. 3 (1) (no pagination), 2016. Article Number: e000155. Date of Publication: 01 Dec 2016.

AN: 613771828

Introduction: Pulmonary aspergillosis has variable course of illness, severity and outcomes depending on underlying conditions. There is limited data available on the clinical manifestations and outcome of pulmonary aspergillosis from Pakistan. Methods: To determine the clinical manifestations and outcome of pulmonary aspergillosis in a tertiary care hospital a retrospective study was conducted from 2004 to 2014 in patients admitted with pulmonary aspergillosis at the Aga Khan University Hospital Karachi, Pakistan. Results: Of the 280 cases with provisional diagnosis of aspergillosis 69 met the inclusion criteria. The mean age was 45+/-15.7 years, 48 (69.6%) were men and 21 (30.4%) had diabetes mellitus (DM). The average length of hospital stay (LOS) was 10.61+/-9.08 days. Aspergillus fumigatus was the most common (42.0%), followed by Aspergillus flavus (28.9%). More than onethird of patients previously had tuberculosis (TB) (39.13%). The commonest pulmonary manifestation was chronic pulmonary aspergillosis (CPA) 47 (68.1%) followed by invasive pulmonary aspergillosis (IPA) 12 (17.4%) and subacute invasive aspergillosis (SAIA) 8 (11.6%). Surgical excision was performed in 28 patients (40.57%). Intensive care unit admission was required for 18 patients (26.08%). Case fatality rate was 14/69 (20.3%). DM, mean LOS and hypoxic respiratory failure were identified as independent risk factors of mortality on multivariate analysis. Conclusion: A. fumigatus was the most frequent species found especially in patients with prior TB. CPA was the commonest pulmonary manifestation seen as post TB sequel. Diabetes, hypoxic respiratory failure and increased LOS were independent predictors of poor outcomes. Overall patients had good outcome with CPA compared with SAIA and IPA. Copyright © 2016, British Thoracic Society. All rights reserved.

Institution

(Iqbal, Irfan, Zubairi, Khan) Department of Medicine, Section of Pulmonary and Critical Care, Aga Khan University Hospital, Karachi, Pakistan (Jabeen) Department of Pathology and Laboratory Medicine, Aga Khan University, Karachi, Pakistan

(Awan) Department of Medicine, Aga Khan University, Karachi, Pakistan

Publisher

BMJ Publishing Group (E-mail: subscriptions@bmjgroup.com)

Link to the Ovid Full Text or citation:

[Click here for full text options](http://ovidsp.ovid.com/ovidweb.cgi?T=JS&CSC=Y&NEWS=N&PAGE=fulltext&D=emed18b&AN=613771828)

Link to the External Link Resolver:

[SFX](http://whel-primo.hosted.exlibrisgroup.com/openurl/44WHELF_CAR/44WHELF_CAR_services_page?sid=OVID:embase&id=pmid:&id=doi:10.1136%2Fbmjresp-2016-000155&issn=2052-4439&isbn=&volume=3&issue=1&spage=no+pagination&pages=no+pagination&date=2016&title=BMJ+Open+Respiratory+Research&atitle=Clinical+manifestations+and+outcomes+of+pulmonary+aspergillosis%3A+Experience+from+pakistan&aulast=Iqbal&pid=<author>Iqbal+N.%3BIrfan+M.%3BZubairi+A.B.S.%3BJabeen+K.%3BAwan+S.%3BKhan+J.A.<%2Fauthor><AN>613771828<%2FAN><DT>Journal%3A+Article<%2FDT>)

29.

The 2016 International Society for Heart Lung Transplantation listing criteria for heart transplantation: A 10-year update.

Mehra M.R., Canter C.E., Hannan M.M., Semigran M.J., Uber P.A., Baran D.A., Danziger-Isakov L., Kirklin J.K., Kirk R., Kushwaha S.S., Lund L.H., Potena L., Ross H.J., Taylor D.O., Verschuuren E.A.M., Zuckermann A.

Journal of Heart and Lung Transplantation. 35 (1) (pp 1-23), 2016. Date of Publication: 01 Jan 2016.

AN: 613931674

Publisher

Elsevier USA

Link to the Ovid Full Text or citation:

[Click here for full text options](http://ovidsp.ovid.com/ovidweb.cgi?T=JS&CSC=Y&NEWS=N&PAGE=fulltext&D=emed18b&AN=613931674)

Link to the External Link Resolver:

[SFX](http://whel-primo.hosted.exlibrisgroup.com/openurl/44WHELF_CAR/44WHELF_CAR_services_page?sid=OVID:embase&id=pmid:&id=doi:10.1016%2Fj.healun.2015.10.023&issn=1053-2498&isbn=&volume=35&issue=1&spage=1&pages=1-23&date=2016&title=Journal+of+Heart+and+Lung+Transplantation&atitle=The+2016+International+Society+for+Heart+Lung+Transplantation+listing+criteria+for+heart+transplantation%3A+A+10-year+update&aulast=Mehra&pid=<author>Mehra+M.R.%3BCanter+C.E.%3BHannan+M.M.%3BSemigran+M.J.%3BUber+P.A.%3BBaran+D.A.%3BDanziger-Isakov+L.%3BKirklin+J.K.%3BKirk+R.%3BKushwaha+S.S.%3BLund+L.H.%3BPotena+L.%3BRoss+H.J.%3BTaylor+D.O.%3BVerschuuren+E.A.M.%3BZuckermann+A.<%2Fauthor><AN>613931674<%2FAN><DT>Journal%3A+Article<%2FDT>)

30.

Cohort profile: The diabetes-tuberculosis treatment outcome (DITTO) study in Pakistan.

Mukhtar F., Butt Z.A.

BMJ Open. 6 (12) (no pagination), 2016. Article Number: e012970. Date of Publication: 01 Dec 2016.

AN: 613514830

Purpose: Pakistan is faced with an increasing prevalence of diabetes in addition to its existing high burden of tuberculosis (TB). Diabetes has a detrimental effect on treatment outcomes of patients with TB, which may hinder achieving the goals of the End-TB strategy by 2030. We conducted a prospective cohort study to determine difference between treatment outcomes among patients with diabetes and new pulmonary tuberculosis (PTB) and patients without diabetes and new PTB. This would help generate contextual and valid scientific evidence from a developing country like Pakistan with its unique interplay of sociocultural, economic and health system factors to inform policy and practice. Participants: This paper outlines the baseline characteristics of 614 new cases of PTB, aged 15 years and older, which were followed up prospectively at 2nd, 5th and 6th months while on antituberculosis treatment and at 6 months after treatment completion. Findings to date: We ascertained patients' diabetic status by conducting random and fasting blood glucose tests and their glycaemic control by determining glycosylated haemoglobin. Treatment outcomes were established using standardised definitions provided by WHO. The assessment of 614 respondents' diabetic status revealed that 113 (18%) were diabetic and 501 (82%) were nondiabetic. A greater proportion of patients with diabetes and PTB were illiterate (n=74/113, 65.5%) as compared to patients without diabetes and PTB (n=249/501, 50%) ( p=0.035). More patients with diabetes and PTB gave a history of heart disease (n=14/113, 12%) and hypertension (n=26/113, 23%) as compared to patients without diabetes and PTB (n=2/501, 0.4% (heart disease) and n=13 501, 3% (hypertension)) ( p<0.001). Unfavourable treatment outcome was more likely among patients with diabetes and PTB (n=23/93, 25%) as opposed to patients without diabetes and PTB (n=46/410, 11%) (p=0.001). Future plans: We are negotiating with the government regarding funding for a further 2-year follow-up of the cohort to ascertain death and relapse in the post-treatment period and also differentiate between re-infection and recurrence among these patients with respect to their diabetic status.

Institution

(Mukhtar) Health Services Academy, Islamabad, Pakistan (Mukhtar) Department of Community Medicine, Lahore Medical and Dental College, Lahore, Pakistan

(Butt) Department of Epidemiology and Biostatistics, Health Services Academy, Islamabad, Pakistan

(Butt) School of Population and Public Health, University of British Columbia, Vancouver, BC, Canada

Publisher

BMJ Publishing Group (E-mail: subscriptions@bmjgroup.com)

Link to the Ovid Full Text or citation:

[Click here for full text options](http://ovidsp.ovid.com/ovidweb.cgi?T=JS&CSC=Y&NEWS=N&PAGE=fulltext&D=emed18b&AN=613514830)

Link to the External Link Resolver:

[SFX](http://whel-primo.hosted.exlibrisgroup.com/openurl/44WHELF_CAR/44WHELF_CAR_services_page?sid=OVID:embase&id=pmid:&id=doi:10.1136%2Fbmjopen-2016-012970&issn=2044-6055&isbn=&volume=6&issue=12&spage=no+pagination&pages=no+pagination&date=2016&title=BMJ+Open&atitle=Cohort+profile%3A+The+diabetes-tuberculosis+treatment+outcome+(DITTO)+study+in+Pakistan&aulast=Mukhtar&pid=<author>Mukhtar+F.%3BButt+Z.A.<%2Fauthor><AN>613514830<%2FAN><DT>Journal%3A+Article<%2FDT>)

31.

Prognostic factors of treatment among patients with multidrug-resistant tuberculosis in Egypt.

Gadallah M.A., Mokhtar A., Rady M., El-Moghazy E., Fawzy M., Kandil S.K.

Journal of the Formosan Medical Association. 115 (11) (pp 997-1003), 2016. Date of Publication: 01 Nov 2016.

AN: 607258282

Background/purpose Multidrug-resistant tuberculosis (MDR-TB) represents 5% of TB cases globally. In Egypt, it represents 11.4% of TB cases (2.2% of new and 38.2% of previously treated). Our objectives were to evaluate the treatment outcomes and determine the associated prognostic factors among the first national treatment cohort of MDR-TB from 2006 to 2010. Methods All patients diagnosed with MDR-TB from July 2006 to December 2010 who were admitted to Abbassia Chest Hospital, the first Egyptian national center established for MDR-TB treatment, were included. They were followed up clinically, radiologically, and bacteriologically by sputum smear, culture, and drug-susceptibility testing at regular intervals. Individualized treatment regimens were prescribed according to each patient's drug-susceptibility testing and the drug treatment history. Patients received at least five effective drugs. Outcome rates, and crude and adjusted odds ratios of unsuccessful outcomes were calculated. Results The number of bacteriologically proven MDR-TB patients was 228, of which 225 were pulmonary cases. Half of the cases showed moderate or extensive lung lesions, and 15.8% were diabetics. A total of 158 (119 cured and 39 completed treatment) patients achieved successful outcome (69.3%), 16 (7.1%) failed treatment, 27 (11.8%) were lost to follow up, and 27 (11.8%) died. Predictors of unsuccessful outcome were delay in sputum culture conversion to 2 months or more, moderate or extensive lung lesions, and a history of diabetes. Conclusion A treatment success rate of approximately 69% was achieved with the first national treatment cohort of MDR-TB under the Egyptian program. Predictors of unsuccessful treatment were delayed culture conversion, moderate or extensive lung affection, and diabetes. Copyright © 2015

Institution

(Gadallah, Rady, Kandil) Department of Community, Environmental and Occupational Medicine, Faculty of Medicine, Ain Shams University, Cairo, Egypt (Mokhtar, El-Moghazy, Fawzy) Ministry of Health and Population, Chest Directorate, Abbassia Chest Hospital, Cairo, Egypt

Publisher

Elsevier B.V.

Link to the Ovid Full Text or citation:

[Click here for full text options](http://ovidsp.ovid.com/ovidweb.cgi?T=JS&CSC=Y&NEWS=N&PAGE=fulltext&D=emed18b&AN=607258282)

Link to the External Link Resolver:

[SFX](http://whel-primo.hosted.exlibrisgroup.com/openurl/44WHELF_CAR/44WHELF_CAR_services_page?sid=OVID:embase&id=pmid:&id=doi:10.1016%2Fj.jfma.2015.10.002&issn=0929-6646&isbn=&volume=115&issue=11&spage=997&pages=997-1003&date=2016&title=Journal+of+the+Formosan+Medical+Association&atitle=Prognostic+factors+of+treatment+among+patients+with+multidrug-resistant+tuberculosis+in+Egypt&aulast=Gadallah&pid=<author>Gadallah+M.A.%3BMokhtar+A.%3BRady+M.%3BEl-Moghazy+E.%3BFawzy+M.%3BKandil+S.K.<%2Fauthor><AN>607258282<%2FAN><DT>Journal%3A+Article<%2FDT>)

32.

Educational Outreach with an Integrated Clinical Tool for Nurse-Led Non-communicable Chronic Disease Management in Primary Care in South Africa: A Pragmatic Cluster Randomised Controlled Trial.

Fairall L.R., Folb N., Timmerman V., Lombard C., Steyn K., Bachmann M.O., Bateman E.D., Lund C., Cornick R., Faris G., Gaziano T., Georgeu-Pepper D., Zwarenstein M., Levitt N.S.

PLoS Medicine. 13 (11) (no pagination), 2016. Article Number: e1002178. Date of Publication: November 2016.

AN: 613530964

Background: In many low-income countries, care for patients with non-communicable diseases (NCDs) and mental health conditions is provided by nurses. The benefits of nurse substitution and supplementation in NCD care in high-income settings are well recognised, but evidence from low- and middle-income countries is limited. Primary Care 101 (PC101) is a programme designed to support and expand nurses' role in NCD care, comprising educational outreach to nurses and a clinical management tool with enhanced prescribing provisions. We evaluated the effect of the programme on primary care nurses' capacity to manage NCDs. Methods and Findings: In a cluster randomised controlled trial design, 38 public sector primary care clinics in the Western Cape Province, South Africa, were randomised. Nurses in the intervention clinics were trained to use the PC101 management tool during educational outreach sessions delivered by health department trainers and were authorised to prescribe an expanded range of drugs for several NCDs. Control clinics continued use of the Practical Approach to Lung Health and HIV/AIDS in South Africa (PALSA PLUS) management tool and usual training. Patients attending these clinics with one or more of hypertension (3,227), diabetes (1,842), chronic respiratory disease (1,157) or who screened positive for depression (2,466), totalling 4,393 patients, were enrolled between 28 March 2011 and 10 November 2011. Primary outcomes were treatment intensification in the hypertension, diabetes, and chronic respiratory disease cohorts, defined as the proportion of patients in whom treatment was escalated during follow-up over 14 mo, and case detection in the depression cohort. Primary outcome data were analysed for 2,110 (97%) intervention and 2,170 (97%) control group patients. Treatment intensification rates in intervention clinics were not superior to those in the control clinics (hypertension: 44% in the intervention group versus 40% in the control group, risk ratio [RR] 1.08 [95% CI 0.94 to 1.24; p = 0.252]; diabetes: 57% versus 50%, RR 1.10 [0.97 to 1.24; p = 0.126]; chronic respiratory disease: 14% versus 12%, RR 1.08 [0.75 to 1.55; p = 0.674]), nor was case detection of depression (18% versus 24%, RR 0.76 [0.53 to 1.10; p = 0.142]). No adverse effects of the nurses' expanded scope of practice were observed. Limitations of the study include dependence on self-reported diagnoses for inclusion in the patient cohorts, limited data on uptake of PC101 by users, reliance on process outcomes, and insufficient resources to measure important health outcomes, such as HbA1c, at follow-up. Conclusions: Educational outreach to primary care nurses to train them in the use of a management tool involving an expanded role in managing NCDs was feasible and safe but was not associated with treatment intensification or improved case detection for index diseases. This notwithstanding, the intervention, with adjustments to improve its effectiveness, has been adopted for implementation in primary care clinics throughout South Africa. Trial Registration: The trial is registered with Current Controlled Trials (ISRCTN20283604) Copyright © 2016 Fairall et al.

Author NameID

Timmerman, Venessa; ORCID: <http://orcid.org/0000-0002-6172-9386> Cornick, Ruth; ORCID: <http://orcid.org/0000-0002-7391-3383>

Folb, Naomi; ORCID: <http://orcid.org/0000-0002-2667-0956>

Institution

(Fairall, Folb, Timmerman, Bateman, Cornick, Faris, Georgeu-Pepper) Knowledge Translation Unit, University of Cape Town Lung Institute, Cape Town, South Africa (Fairall, Folb, Steyn, Bateman, Levitt) Department of Medicine, University of Cape Town, Cape Town, South Africa

(Fairall, Folb, Steyn, Gaziano, Levitt) Chronic Disease Initiative for Africa, Department of Medicine, University of Cape Town, Cape Town, South Africa

(Lombard) Biostatistics Unit, Medical Research Council, Cape Town, South Africa

(Bachmann) Norwich Medical School, University of East Anglia, Norwich, United Kingdom

(Lund) Alan J Flisher Centre for Public Mental Health, Department of Psychiatry and Mental Health, University of Cape Town, Cape Town, South Africa

(Gaziano) Division of Cardiovascular Medicine, Brigham & Women's Hospital, Boston, MA, United States

(Zwarenstein) Centre for Studies in Family Medicine, Department of Family Medicine, Schulich School of Medicine and Dentistry, Western University, London, ON, Canada

Publisher

Public Library of Science (E-mail: plos@plos.org)

Link to the Ovid Full Text or citation:

[Click here for full text options](http://ovidsp.ovid.com/ovidweb.cgi?T=JS&CSC=Y&NEWS=N&PAGE=fulltext&D=emed18b&AN=613530964)

Link to the External Link Resolver:

[SFX](http://whel-primo.hosted.exlibrisgroup.com/openurl/44WHELF_CAR/44WHELF_CAR_services_page?sid=OVID:embase&id=pmid:&id=doi:10.1371%2Fjournal.pmed.1002178&issn=1549-1277&isbn=&volume=13&issue=11&spage=no+pagination&pages=no+pagination&date=2016&title=PLoS+Medicine&atitle=Educational+Outreach+with+an+Integrated+Clinical+Tool+for+Nurse-Led+Non-communicable+Chronic+Disease+Management+in+Primary+Care+in+South+Africa%3A+A+Pragmatic+Cluster+Randomised+Controlled+Trial&aulast=Fairall&pid=<author>Fairall+L.R.%3BFolb+N.%3BTimmerman+V.%3BLombard+C.%3BSteyn+K.%3BBachmann+M.O.%3BBateman+E.D.%3BLund+C.%3BCornick+R.%3BFaris+G.%3BGaziano+T.%3BGeorgeu-Pepper+D.%3BZwarenstein+M.%3BLevitt+N.S.<%2Fauthor><AN>613530964<%2FAN><DT>Journal%3A+Article<%2FDT>)

33.

Clinical implications of diabetes mellitus in adults with TB: Risk for poor outcomes and mortality.

Barss L., Orlikow E., Phang S., Sabur N., Arget M., Field S., Ainslie M., Fisher D.

Chest. Conference: CHEST 2016. United States. Conference Start: 20161022. Conference End: 20161026. 150 (4 Supplement 1) (pp 206A), 2016. Date of Publication: October 2016.

AN: 613468925

PURPOSE: To evaluate the association between incident and prevalent diabetes mellitus (DM) and clinical presentation, treatment failure, and mortality in adults with active tuberculosis (TB) in Canada. METHODS: A retrospective chart review of all adult patients treated for TB in two large Canadian cities (Calgary, AB and Winnipeg, MB) between 2007-2012 was performed. The primary outcome was all cause mortality during TB treatment. Patients aged >18 years with a clinical or microbiologic diagnosis of mycobacterium tuberculosis (pulmonary and/or extrapulmonary) with adequate chart data were included. Determination of DM prevalence at time of TB diagnosis was based on chart diagnosis of DM or documented use of insulin or oral hypoglycemics. An incident diagnosis of DM was defined as new diagnosis within six months of TB diagnosis. Secondary outcomes included: treatment failure (death, relapse, or culture positivity at 4 months), presence of cavitary disease, smear and culture positivity, and presence of drug resistance. Unadjusted odds ratios were used to estimate the association between DM and mortality and treatment failure. RESULTS: 788 patients treated for TB based on a clinical or microbiologic diagnosis were identified. Pre-existing DM was found in 131 patients (17%) and an incident diagnosis of DM was made in 19 patients (2%). The average hemoglobin A1c was 8.7% and 9.4% in patients with pre-existing and new DM, respectively. HIV co-infection was found in zero patients with DM and 41 patients without DM (7%). Compared to patients without a diagnosis of DM, mortality during TB treatment was higher in patients with pre-existing DM (OR 2.62, 95% CI 1.37-5.02), but not in patients with an incident diagnosis of DM (OR 1.13, 95% CI 0.14-8.72). Patients with pre-existing DM were also more likely to have treatment failure (OR 3.02, 95% CI 1.64- 5.56) compared to those without DM. Both death and relapse contributed to treatment failure in pre-exisitng DM (relapse OR 6.49, 95% CI 1.07-39.19). There were no documented culture positive cases at four months in either group. Patients with DM (incident or prevalent) were more likely to have smear positive disease (44% vs. 34%) and cavitary disease (37% vs. 20%) compared to patients without DM. Drug resistance rates between DM and non DM patients were similar (11% versus 10%, respectively). Ethnicity varied by geographic region, with 326 (91%) of TB cases in Calgary, AB occurring in foreign born patients, versus 229 (53%) of TB cases in Winnipeg, MB occurring in Canadian born Aboriginals. Patterns in clinical presentation, treatment failure, and mortality in patients with DM versus without DM were similar between geographic sites. CONCLUSIONS: In our cohort of active TB cases, patients with pre-existing DM had an increased risk of mortality and treatment failure. Consistent with the finding of worse treatment outcomes, patients with pre-existing DM (and incident DM) had more extensive disease at presentation.

Institution

(Barss, Orlikow, Phang, Sabur, Arget, Field, Ainslie, Fisher) University of Calgary, Calgary, AB, Canada

Publisher

Elsevier B.V.

Link to the Ovid Full Text or citation:

[Click here for full text options](http://ovidsp.ovid.com/ovidweb.cgi?T=JS&CSC=Y&NEWS=N&PAGE=fulltext&D=emed18b&AN=613468925)

Link to the External Link Resolver:

[SFX](http://whel-primo.hosted.exlibrisgroup.com/openurl/44WHELF_CAR/44WHELF_CAR_services_page?sid=OVID:embase&id=pmid:&id=doi:10.1016%2Fj.chest.2016.08.215&issn=1931-3543&isbn=&volume=150&issue=4+Supplement+1&spage=206A&pages=206A&date=2016&title=Chest&atitle=Clinical+implications+of+diabetes+mellitus+in+adults+with+TB%3A+Risk+for+poor+outcomes+and+mortality&aulast=Barss&pid=<author>Barss+L.%3BOrlikow+E.%3BPhang+S.%3BSabur+N.%3BArget+M.%3BField+S.%3BAinslie+M.%3BFisher+D.<%2Fauthor><AN>613468925<%2FAN><DT>Journal%3A+Conference+Abstract<%2FDT>)

34.

Preventing acquired resistance to bedaquiline and delamanid in multidrug-resistant tuberculosis treatment requires optimal management.

Guglielmetti L., Du D.L., Frechet-Jachym M., Mitnick C.

American Journal of Respiratory and Critical Care Medicine. 194 (9) (pp 1170-1171), 2016. Date of Publication: 01 Nov 2016.

AN: 613234032

Author NameID

Guglielmetti, Lorenzo; ORCID: <http://orcid.org/0000-0003-0886-9635>

Institution

(Guglielmetti, Du, Frechet-Jachym) Centre Hospitalier de Bligny Briis-sous-Forges, France (Guglielmetti) Pierre and Marie Curie University, Paris, France

(Guglielmetti) INSERM U1135, Paris, France

(Mitnick) Harvard Medical School, Boston, MA, United States

(Mitnick) Partners in Health, Boston, MA, United States

(Mitnick) Brigham and Women's Hospital, Boston, MA, United States

Publisher

American Thoracic Society (E-mail: malexander@thoracic.org)

Link to the Ovid Full Text or citation:

[Click here for full text options](http://ovidsp.ovid.com/ovidweb.cgi?T=JS&CSC=Y&NEWS=N&PAGE=fulltext&D=emed18b&AN=613234032)

Link to the External Link Resolver:

[SFX](http://whel-primo.hosted.exlibrisgroup.com/openurl/44WHELF_CAR/44WHELF_CAR_services_page?sid=OVID:embase&id=pmid:&id=doi:10.1164%2Frccm.201604-0841LE&issn=1073-449X&isbn=&volume=194&issue=9&spage=1170&pages=1170-1171&date=2016&title=American+Journal+of+Respiratory+and+Critical+Care+Medicine&atitle=Preventing+acquired+resistance+to+bedaquiline+and+delamanid+in+multidrug-resistant+tuberculosis+treatment+requires+optimal+management&aulast=Guglielmetti&pid=<author>Guglielmetti+L.%3BDu+D.L.%3BFrechet-Jachym+M.%3BMitnick+C.<%2Fauthor><AN>613234032<%2FAN><DT>Journal%3A+Letter<%2FDT>)

35.

A prediction rule to stratify mortality risk of patients with pulmonary tuberculosis.

Bastos H.N., Osorio N.S., Castro A.G., Ramos A., Carvalho T., Meira L., Araujo D., Almeida L., Boaventura R., Fragata P., Chaves C., Costa P., Portela M., Ferreira I., Magalhaes S.P., Rodrigues F., Sarmento-Castro R., Duarte R., Guimaraes J.T., Saraiva M.

PLoS ONE. 11 (9) (no pagination), 2016. Article Number: e0162797. Date of Publication: September 2016.

AN: 612857061

Tuberculosis imposes high human and economic tolls, including in Europe. This study was conducted to develop a severity assessment tool for stratifying mortality risk in pulmonary tuberculosis (PTB) patients. A derivation cohort of 681 PTB cases was retrospectively reviewed to generate a model based on multiple logistic regression analysis of prognostic variables with 6-month mortality as the outcome measure. A clinical scoring system was developed and tested against a validation cohort of 103 patients. Five risk features were selected for the prediction model: hypoxemic respiratory failure (OR 4.7, 95% CI 2.8-7.9), age <50 years (OR 2.9, 95% CI 1.7-4.8), bilateral lung involvement (OR 2.5, 95% CI 1.4-4.4), <1 significant comorbidity-HIV infection, diabetes mellitus, liver failure or cirrhosis, congestive heart failure and chronic respiratory disease-(OR 2.3, 95% CI 1.3-3.8), and hemoglobin <12 g/dL (OR 1.8, 95% CI 1.1-3.1). A tuberculosis risk assessment tool (TReAT) was developed, stratifying patients with low (score >2), moderate (score 3-5) and high (score >6) mortality risk. The mortality associated with each group was 2.9%, 22.9% and 53.9%, respectively. The model performed equally well in the validation cohort. We provide a new, easy-To-use clinical scoring system to identify PTB patients with high-mortality risk in settings with good healthcare access, helping clinicians to decide which patients are in need of closer medical care during treatment. Copyright © 2016 Bastos et al. This is an open access article distributed under the terms of the Creative Commons Attribution License, which permits unrestricted use, distribution, and reproduction in any medium, provided the original author and source are credited.

Institution

(Bastos, Meira, Araujo, Almeida, Boaventura) Department of Pneumology, Centro Hospitalar Sao Joao, Porto, Portugal (Bastos, Osorio, Castro, Chaves, Costa, Rodrigues, Sarmento-Castro, Saraiva) Life and Health Sciences Research Institute (ICVS), School of Health Sciences, University of Minho, Braga, Portugal

(Bastos, Osorio, Castro, Costa, Rodrigues, Sarmento-Castro, Saraiva) ICVS/3B's-PT Government Associ. Laboratory, Braga/Guimaraes, Portugal

(Ramos, Carvalho, Guimaraes) Department of Clinical Pathology, Centro Hospitalar Sao Joao, Porto, Portugal

(Ramos, Carvalho, Duarte, Guimaraes) Institute of Public Health, University of Porto, Porto, Portugal

(Fragata) Department of Internal Medicine, Hospital Do Santo Espirito da Ilha Terceira, Angra do Heroismo, Portugal

(Costa) Faculty of Psychology and Educational Sciences, University of Porto, Porto, Portugal

(Portela) School of Economics and Management, University of Minho, Braga, Portugal

(Ferreira, Magalhaes) Department of Radiology, Centro Hospitalar Do Porto, Porto, Portugal

(Sarmento-Castro) Department of Infectious Diseases, Centro Hospitalar Do Porto, Hospital Joaquim Urbano Unit, Porto, Portugal

(Duarte) Department of Pneumology, Centro Hospitalar de Vila Nova de Gaia/Espinho, Vila Nova de Gaia, Portugal

(Duarte) Referral Centre for Multidrug-resistant Tuberculosis in the Northern Region of Portugal, Chest Disease Centre of Vila Nova de Gaia, Vila Nova de Gaia, Portugal

(Duarte) General Direct. of Health, Lisboa, Portugal

(Guimaraes) Department of Biochemistry, Faculty of Medicine, University of Porto, Porto, Portugal

(Saraiva) S-Instituto de Investigacao e Inovacao em Saude, University of Porto, Portugal

(Saraiva) IBMC-Instituto de Biologia Molecular e Celular, University of Porto, Porto, Portugal

Publisher

Public Library of Science (E-mail: plos@plos.org)

Link to the Ovid Full Text or citation:

[Click here for full text options](http://ovidsp.ovid.com/ovidweb.cgi?T=JS&CSC=Y&NEWS=N&PAGE=fulltext&D=emed18b&AN=612857061)

Link to the External Link Resolver:

[SFX](http://whel-primo.hosted.exlibrisgroup.com/openurl/44WHELF_CAR/44WHELF_CAR_services_page?sid=OVID:embase&id=pmid:&id=doi:10.1371%2Fjournal.pone.0162797&issn=1932-6203&isbn=&volume=11&issue=9&spage=no+pagination&pages=no+pagination&date=2016&title=PLoS+ONE&atitle=A+prediction+rule+to+stratify+mortality+risk+of+patients+with+pulmonary+tuberculosis&aulast=Bastos&pid=<author>Bastos+H.N.%3BOsorio+N.S.%3BCastro+A.G.%3BRamos+A.%3BCarvalho+T.%3BMeira+L.%3BAraujo+D.%3BAlmeida+L.%3BBoaventura+R.%3BFragata+P.%3BChaves+C.%3BCosta+P.%3BPortela+M.%3BFerreira+I.%3BMagalhaes+S.P.%3BRodrigues+F.%3BSarmento-Castro+R.%3BDuarte+R.%3BGuimaraes+J.T.%3BSaraiva+M.<%2Fauthor><AN>612857061<%2FAN><DT>Journal%3A+Article<%2FDT>)

36.

Statin non-adherence and residual cardiovascular risk: There is need for substantial improvement.

Banach M., Stulc T., Dent R., Toth P.P.

International Journal of Cardiology. 225 (pp 184-196), 2016. Date of Publication: 15 Dec 2016.

AN: 612522694

Although statin therapy has proven to be the cornerstone for prevention and treatment of cardiovascular disease (CVD), there are many patients for whom long-term therapy remains suboptimal. The aims of this article are to review the current complex issues associated with statin use and to explore when novel treatment approaches should be considered. Statin discontinuation as well as adherence to statin therapy remain two of the greatest challenges for lipidologists. Evidence suggests that between 40 and 75% of patients discontinue their statin therapy within one year after initiation. Furthermore, whilst the reasons for persistence with statin therapy are complex, evidence shows that low-adherence to statins negatively impacts clinical outcomes and residual CV risk remains a major concern. Non-adherence or lack of persistence with long-term statin therapy in real-life may be the main cause of inadequate low density lipoprotein cholesterol lowering with statins. There is a large need for the improvement of the use of statins, which have good safety profiles and are inexpensive. On the other hand, in a non-cost-constrained environment, proprotein convertase subtilisin/kexin type 9 inhibitors should arguably be used more often in those patients in whom treatment with statins remains unsatisfactory. Copyright © 2016 Elsevier Ireland Ltd

Author NameID

Banach, Maciej; ORCID: <http://orcid.org/0000-0001-6690-6874>

Institution

(Banach) Department of Hypertension, Chair of Nephrology and Hypertension, Medical University of Lodz, Lodz, Poland (Banach) Healthy Aging Research Centre, Medical University of Lodz, Lodz, Poland

(Banach) Polish Mother's Memorial Hospital Research Institute, Lodz, Poland

(Stulc) 3rd Department of Internal Medicine, 1st University of Medicine, Prague, Czech Republic

(Dent) Amgen (Europe) GmbH, Zug, Switzerland

(Toth) CGH Medical Center, Sterling, IL, United States

(Toth) Ciccarone Center for the Prevention of Cardiovascular Disease, Johns Hopkins University School of Medicine, Baltimore, MD, United States

Publisher

Elsevier Ireland Ltd

Link to the Ovid Full Text or citation:

[Click here for full text options](http://ovidsp.ovid.com/ovidweb.cgi?T=JS&CSC=Y&NEWS=N&PAGE=fulltext&D=emed18b&AN=612522694)

Link to the External Link Resolver:

[SFX](http://whel-primo.hosted.exlibrisgroup.com/openurl/44WHELF_CAR/44WHELF_CAR_services_page?sid=OVID:embase&id=pmid:&id=doi:10.1016%2Fj.ijcard.2016.09.075&issn=0167-5273&isbn=&volume=225&issue=&spage=184&pages=184-196&date=2016&title=International+Journal+of+Cardiology&atitle=Statin+non-adherence+and+residual+cardiovascular+risk%3A+There+is+need+for+substantial+improvement&aulast=Banach&pid=<author>Banach+M.%3BStulc+T.%3BDent+R.%3BToth+P.P.<%2Fauthor><AN>612522694<%2FAN><DT>Journal%3A+Review<%2FDT>)

37.

Current trends and intricacies in the management of HIV-associated pulmonary tuberculosis.

Gopalan N., Chandrasekaran P., Swaminathan S., Tripathy S.

AIDS Research and Therapy. 13 (1) (no pagination), 2016. Article Number: 34. Date of Publication: 26 Sep 2016.

AN: 612339406

Human immunodeficiency virus (HIV) epidemic has undoubtedly increased the incidence of tuberculosis (TB) globally, posing a formidable global health challenge affecting 1.2 million cases. Pulmonary TB assumes utmost significance in the programmatic perspective as it is readily transmissible as well as easily diagnosable. HIV complicates every aspect of pulmonary tuberculosis from diagnosis to treatment, demanding a different approach to effectively tackle both the diseases. In order to control these converging epidemics, it is important to diagnose early, initiate appropriate therapy for both infections, prevent transmission and administer preventive therapy. Liquid culture methods and nucleic acid amplification tests for TB confirmation have replaced conventional solid media, enabling quicker and simultaneous detection of mycobacterium and its drug sensitivity profile Unique problems posed by the syndemic include Acquired rifampicin resistance, drug-drug interactions, malabsorption of drugs and immune reconstitution inflammatory syndrome or paradoxical reaction that complicate dual and concomitant therapy. While the antiretroviral therapy armamentarium is constantly reinforced by discovery of newer and safer drugs every year, only a few drugs for anti tuberculosis treatment have successfully emerged. These include bedaquiline, delamanid and pretomanid which have entered phase III B trials and are also available through conditional access national programmes. The current guidelines by WHO to start Antiretroviral therapy irrespective of CD4+ cell count based on benefits cited by recent trials could go a long way in preventing various complications caused by the deadly duo. This review provides a consolidated gist of the advancements, concepts and updates that have emerged in the management of HIV-associated pulmonary TB for maximizing efficacy, offering latest solutions for tackling drug-drug interactions and remedial measures for immune reconstitution inflammatory syndrome. Copyright © 2016 The Author(s).

Institution

(Gopalan, Chandrasekaran, Swaminathan, Tripathy) National Institute for Research in Tuberculosis (Formerly Tuberculosis Research Centre), Division of HIV, No. 1, Mayor Sathyamoorthy Road, Chetpet, Chennai 600 031, India

Publisher

BioMed Central Ltd. (E-mail: info@biomedcentral.com)

Link to the Ovid Full Text or citation:

[Click here for full text options](http://ovidsp.ovid.com/ovidweb.cgi?T=JS&CSC=Y&NEWS=N&PAGE=fulltext&D=emed18b&AN=612339406)

Link to the External Link Resolver:

[SFX](http://whel-primo.hosted.exlibrisgroup.com/openurl/44WHELF_CAR/44WHELF_CAR_services_page?sid=OVID:embase&id=pmid:&id=doi:10.1186%2Fs12981-016-0118-7&issn=1742-6405&isbn=&volume=13&issue=1&spage=no+pagination&pages=no+pagination&date=2016&title=AIDS+Research+and+Therapy&atitle=Current+trends+and+intricacies+in+the+management+of+HIV-associated+pulmonary+tuberculosis&aulast=Gopalan&pid=<author>Gopalan+N.%3BChandrasekaran+P.%3BSwaminathan+S.%3BTripathy+S.<%2Fauthor><AN>612339406<%2FAN><DT>Journal%3A+Review<%2FDT>)

38.

Tuberculosis.

Dheda K., Barry C.E., Maartens G.

The Lancet. 387 (10024) (pp 1211-1226), 2016. Date of Publication: 19 Mar 2016.

AN: 606036294

Summary Although the worldwide incidence of tuberculosis has been slowly decreasing, the global disease burden remains substantial (~9 million cases and ~1.5 million deaths in 2013), and tuberculosis incidence and drug resistance are rising in some parts of the world such as Africa. The modest gains achieved thus far are threatened by high prevalence of HIV, persisting global poverty, and emergence of highly drug-resistant forms of tuberculosis. Tuberculosis is also a major problem in health-care workers in both low-burden and high-burden settings. Although the ideal preventive agent, an effective vaccine, is still some time away, several new diagnostic technologies have emerged, and two new tuberculosis drugs have been licensed after almost 50 years of no tuberculosis drugs being registered. Efforts towards an effective vaccine have been thwarted by poor understanding of what constitutes protective immunity. Although new interventions and investment in control programmes will enable control, eradication will only be possible through substantial reductions in poverty and overcrowding, political will and stability, and containing co-drivers of tuberculosis, such as HIV, smoking, and diabetes. Copyright © 2016 Elsevier Ltd.

PMID

26377143 [<http://www.ncbi.nlm.nih.gov/pubmed/?term=26377143>]

Institution

(Dheda) Lung Infection and Immunity Unit, Division of Pulmonology and UCT Lung Institute, University of Cape Town, Cape Town, South Africa (Maartens) Division of Clinical Pharmacology, University of Cape Town, Cape Town, South Africa

(Barry) Department of Medicine, Institute of Infectious Disease and Molecular Medicine, Faculty of Health Sciences, University of Cape Town, Cape Town, South Africa

(Dheda, Barry, Maartens) Tuberculosis Research Section, Laboratory of Clinical Infectious Diseases, National Institute of Allergy and Infectious Diseases (NIAID), National Institutes of Health (NIH), Bethesda, MD, United States

Publisher

Lancet Publishing Group (E-mail: cususerv@lancet.com)

Link to the Ovid Full Text or citation:

[Click here for full text options](http://ovidsp.ovid.com/ovidweb.cgi?T=JS&CSC=Y&NEWS=N&PAGE=fulltext&D=emed18b&AN=606036294)

Link to the External Link Resolver:

[SFX](http://whel-primo.hosted.exlibrisgroup.com/openurl/44WHELF_CAR/44WHELF_CAR_services_page?sid=OVID:embase&id=pmid:26377143&id=doi:10.1016%2FS0140-6736%252815%252900151-8&issn=0140-6736&isbn=&volume=387&issue=10024&spage=1211&pages=1211-1226&date=2016&title=The+Lancet&atitle=Tuberculosis&aulast=Dheda&pid=<author>Dheda+K.%3BBarry+C.E.%3BMaartens+G.<%2Fauthor><AN>606036294<%2FAN><DT>Journal%3A+Article<%2FDT>)

39.

Tuberculosis prevention and control: We need to pay heed to diabetes care.

Kapur A.

International Journal of Tuberculosis and Lung Disease. 20 (3) (pp 287), 2016. Date of Publication: 01 Mar 2016.

AN: 608513099

PMID

27046704 [<http://www.ncbi.nlm.nih.gov/pubmed/?term=27046704>]

Institution

(Kapur) World Diabetes Foundation, Gentofte, Denmark

Publisher

International Union against Tubercul. and Lung Dis.

Link to the Ovid Full Text or citation:

[Click here for full text options](http://ovidsp.ovid.com/ovidweb.cgi?T=JS&CSC=Y&NEWS=N&PAGE=fulltext&D=emed18b&AN=608513099)

Link to the External Link Resolver:

[SFX](http://whel-primo.hosted.exlibrisgroup.com/openurl/44WHELF_CAR/44WHELF_CAR_services_page?sid=OVID:embase&id=pmid:27046704&id=doi:10.5588%2Fijtld.15.0917&issn=1027-3719&isbn=&volume=20&issue=3&spage=287&pages=287&date=2016&title=International+Journal+of+Tuberculosis+and+Lung+Disease&atitle=Tuberculosis+prevention+and+control%3A+We+need+to+pay+heed+to+diabetes+care&aulast=Kapur&pid=<author>Kapur+A.<%2Fauthor><AN>608513099<%2FAN><DT>Journal%3A+Editorial<%2FDT>)

40.

Extensively Drug-Resistant Tuberculosis: Principles of Resistance, Diagnosis, and Management.

Wilson J.W., Tsukayama D.T.

Mayo Clinic Proceedings. 91 (4) (pp 482-495), 2016. Date of Publication: 01 Apr 2016.

AN: 608510141

Extensively drug-resistant (XDR) tuberculosis (TB) is an unfortunate by-product of mankind's medical and pharmaceutical ingenuity during the past 60 years. Although new drug developments have enabled TB to be more readily curable, inappropriate TB management has led to the emergence of drug-resistant disease. Extensively drug-resistant TB describes Mycobacterium tuberculosis that is collectively resistant to isoniazid, rifampin, a fluoroquinolone, and an injectable agent. It proliferates when established case management and infection control procedures are not followed. Optimized treatment outcomes necessitate time-sensitive diagnoses, along with expanded combinations and prolonged durations of antimicrobial drug therapy. The challenges to public health institutions are immense and most noteworthy in underresourced communities and in patients coinfected with human immunodeficiency virus. A comprehensive and multidisciplinary case management approach is required to optimize outcomes. We review the principles of TB drug resistance and the risk factors, diagnosis, and managerial approaches for extensively drug-resistant TB. Treatment outcomes, cost, and unresolved medical issues are also discussed. Copyright © 2016 Mayo Foundation for Medical Education and Research.

PMID

26906649 [<http://www.ncbi.nlm.nih.gov/pubmed/?term=26906649>]

Institution

(Wilson) Department of Medicine, Division of Infectious Diseases, Mayo Clinic, Rochester, MN, United States (Tsukayama) Division of Infectious Diseases and Internal Medicine, University of Minnesota, Hennepin County Medical Center, Minneapolis, MN, United States

Publisher

Elsevier Ltd

Link to the Ovid Full Text or citation:

[Click here for full text options](http://ovidsp.ovid.com/ovidweb.cgi?T=JS&CSC=Y&NEWS=N&PAGE=fulltext&D=emed18b&AN=608510141)

Link to the External Link Resolver:

[SFX](http://whel-primo.hosted.exlibrisgroup.com/openurl/44WHELF_CAR/44WHELF_CAR_services_page?sid=OVID:embase&id=pmid:26906649&id=doi:10.1016%2Fj.mayocp.2016.01.014&issn=0025-6196&isbn=&volume=91&issue=4&spage=482&pages=482-495&date=2016&title=Mayo+Clinic+Proceedings&atitle=Extensively+Drug-Resistant+Tuberculosis%3A+Principles+of+Resistance%2C+Diagnosis%2C+and+Management&aulast=Wilson&pid=<author>Wilson+J.W.%3BTsukayama+D.T.<%2Fauthor><AN>608510141<%2FAN><DT>Journal%3A+Article<%2FDT>)

41.

An integrated community TB-HIV adherence model provides an alternative to DOT for tuberculosis patients in Cape Town.

Kaplan R., Caldwell J., Hermans S., Adriaanse S., Mtwisha L., Bekker L.-G., Jennings K., Wood R.

International Journal of Tuberculosis and Lung Disease. 20 (9) (pp 1185-1191 and i), 2016. Date of Publication: 01 Sep 2016.

AN: 611736437

SETTING: Cape Town, South Africa. OBJECTIVE: To evaluate anti-tuberculosis treatment outcomes and rate of antiretroviral therapy (ART) initiation using weekly community-based adherence support compared to daily directly observed therapy (DOT). METHODS: This was a retrospective analysis comparing two cohorts treated for tuberculosis (TB) in 70 TB clinics during 6-month periods before and after the introduction of a new adherence model comprising treatment literacy sessions during 2 weeks of DOT, followed by weekly home visits by community care workers to eligible patients managing their own treatment. Odds ratios (ORs) of treatment success and ART initiation were calculated using multivariable random effects logistic regression models. Hazard ratios (HRs) of default and death were calculated using multivariable random effects Cox regression models. RESULTS: The pre-intervention cohort comprised 11 896 patients with TB and the post-intervention cohort 11 314. There was no difference in pre- and post-intervention anti-tuberculosis treatment success rates (respectively 82.8% and 82.5%, adjusted OR [aOR] 1.02, 95%CI 0.89-1.17, P = 0.76) nor an increased hazard of death (adjusted HR [aHR] 0.98, 95%CI 0.80-1.21, P = 0.87) or default (aHR 0.97, 95%CI 0.81-1.15, P = 0.69). The ART initiation rate increased from 67% to 74% (aOR 1.43; 95%CI 1.01-1.85, P < 0.01). CONCLUSION: Weekly community-based adherence support was a viable alternative to daily DOT, with no deterioration in anti-tuberculosis treatment outcomes and an increase in ART initiation. Copyright © 2016 The Union.

Institution

(Kaplan, Hermans, Mtwisha, Bekker, Wood) Desmond Tutu HIV Centre, Institute for Infectious Disease and Molecular Medicine, Faculty of Health Sciences, University of Cape Town, Anzio Road, Observatory 7925, South Africa (Caldwell, Adriaanse, Jennings) City Health, City of Cape Town, Cape Town, South Africa

(Hermans) Department of Global Health, Academic Medical Center, University of Amsterdam, Amsterdam Institute for Global Health and Development, Netherlands

(Hermans) Department of Internal Medicine, School of Medicine, Makerere University College of Health Sciences, Kampala, Uganda

(Bekker, Wood) Department of Medicine, University of Cape Town, Cape Town, South Africa

Publisher

International Union against Tubercul. and Lung Dis.

Link to the Ovid Full Text or citation:

[Click here for full text options](http://ovidsp.ovid.com/ovidweb.cgi?T=JS&CSC=Y&NEWS=N&PAGE=fulltext&D=emed18b&AN=611736437)

Link to the External Link Resolver:

[SFX](http://whel-primo.hosted.exlibrisgroup.com/openurl/44WHELF_CAR/44WHELF_CAR_services_page?sid=OVID:embase&id=pmid:&id=doi:10.5588%2Fijtld.15.0855&issn=1027-3719&isbn=&volume=20&issue=9&spage=1185&pages=1185-1191+and+i&date=2016&title=International+Journal+of+Tuberculosis+and+Lung+Disease&atitle=An+integrated+community+TB-HIV+adherence+model+provides+an+alternative+to+DOT+for+tuberculosis+patients+in+Cape+Town&aulast=Kaplan&pid=<author>Kaplan+R.%3BCaldwell+J.%3BHermans+S.%3BAdriaanse+S.%3BMtwisha+L.%3BBekker+L.-G.%3BJennings+K.%3BWood+R.<%2Fauthor><AN>611736437<%2FAN><DT>Journal%3A+Article<%2FDT>)

42.

Diabetic tuberculosis.

Kalra S., Atreja A.

Journal of the Pakistan Medical Association. 66 (9) (pp 1194-1195), 2016. Date of Publication: September 2016.

AN: 611662775

Diabetes and tuberculosis coexist together, and influence each other's natural history and treatment outcomes significantly. This assumes clinical as well as public health significance. This article describes these associations, and discusses action that can be taken at the primary care level to tackle this challenge. Copyright © 2016, Pakistan Medical Association. All rights reserved.

Institution

(Kalra) Department of Endocrinology, Bharti Hospital, Karnal, India (Atreja) Department of Pulmonary Medicine, MMIMSR, Mullana, India

Publisher

Pakistan Medical Association

Link to the Ovid Full Text or citation:

[Click here for full text options](http://ovidsp.ovid.com/ovidweb.cgi?T=JS&CSC=Y&NEWS=N&PAGE=fulltext&D=emed18b&AN=611662775)

Link to the External Link Resolver:

[SFX](http://whel-primo.hosted.exlibrisgroup.com/openurl/44WHELF_CAR/44WHELF_CAR_services_page?sid=OVID:embase&id=pmid:&id=doi:&issn=0030-9982&isbn=&volume=66&issue=9&spage=1194&pages=1194-1195&date=2016&title=Journal+of+the+Pakistan+Medical+Association&atitle=Diabetic+tuberculosis&aulast=Kalra&pid=<author>Kalra+S.%3BAtreja+A.<%2Fauthor><AN>611662775<%2FAN><DT>Journal%3A+Article<%2FDT>)

43.

Prevalence, risk factors, management, and treatment outcomes of first-line antituberculous drug-induced liver injury: a prospective cohort study.

Sun Q., Zhang Q., Gu J., Sun W.-W., Wang P., Bai C., Xiao H.-P., Sha W.

Pharmacoepidemiology and Drug Safety. 25 (8) (pp 908-917), 2016. Date of Publication: 01 Aug 2016.

AN: 611526560

Purpose: Antituberculosis drug-induced liver injury (ATDILI) is one of the most deleterious side effects associated with chemotherapy against tuberculosis (TB). In this study, our objective was to determine the incidence, risk factors, and management of ATDILI and analyze its impact on the treatment outcome in patients receiving standard anti-TB chemotherapy. Methods: A prospective cohort study of ATDILI prevalence was conducted in 938 enrolled patients of the 1426 TB cases in Shanghai from March 2011 to September 2012. Patients were followed up until February 2014. Univariate and multivariate logistic regression analyses were used to determine the risk factors of ATDILI. Successful therapeutic outcome, rates of drug resistance conversion, sputum smear/culture conversion, and lung cavity closure were analyzed. Results: Hepatitis B surface antigen/hepatitis B e antigen-positive hepatitis B carriers, complicated with systemic lupus erythematosus, albumin < 25 g/L, and chronic alcoholism were independent risk factors for ATDILI. Of the 121 cases with ATDILI (incidence rate of 12.9%), 84 (69.4%) used modified anti-TB therapy after recovery of liver function. Compared with the non-ATDILI group, patients with ATDILI exhibited remarkably decreased lung cavity closure rate (84.6% vs. 93.0%, P < 0.001) along with significantly reduced sputum smear/culture conversion rate (85.4% vs. 94.0%, P < 0.001). Conclusions: Our findings indicated that 12.9% patients developed ATDILI during standard anti-TB therapy, resulting in poor therapeutic outcome. Hepatitis B carriers with systemic lupus erythematosus, albumin < 25 g/L, and chronic alcoholism manifested increased risks for ATDILI. Copyright © 2016 John Wiley & Sons, Ltd. Copyright © 2016 John Wiley & Sons, Ltd.

PMID

26935778 [<http://www.ncbi.nlm.nih.gov/pubmed/?term=26935778>]

Institution

(Sun, Zhang, Gu, Sun, Wang, Xiao, Sha) Clinic and Research Center of Tuberculosis, Shanghai Key Laboratory of Tuberculosis, Shanghai Pulmonary Hospital, Tongji University School of Medicine, Shanghai, China (Sun, Bai) Department of Respiratory and Critical Care Medicine, Changhai Hospital, the Second Military Medical University, Shanghai, China

Publisher

John Wiley and Sons Ltd (Southern Gate, Chichester, West Sussex PO19 8SQ, United Kingdom)

Link to the Ovid Full Text or citation:

[Click here for full text options](http://ovidsp.ovid.com/ovidweb.cgi?T=JS&CSC=Y&NEWS=N&PAGE=fulltext&D=emed18b&AN=611526560)

Link to the External Link Resolver:

[SFX](http://whel-primo.hosted.exlibrisgroup.com/openurl/44WHELF_CAR/44WHELF_CAR_services_page?sid=OVID:embase&id=pmid:26935778&id=doi:10.1002%2Fpds.3988&issn=1053-8569&isbn=&volume=25&issue=8&spage=908&pages=908-917&date=2016&title=Pharmacoepidemiology+and+Drug+Safety&atitle=Prevalence%2C+risk+factors%2C+management%2C+and+treatment+outcomes+of+first-line+antituberculous+drug-induced+liver+injury%3A+a+prospective+cohort+study&aulast=Sun&pid=<author>Sun+Q.%3BZhang+Q.%3BGu+J.%3BSun+W.-W.%3BWang+P.%3BBai+C.%3BXiao+H.-P.%3BSha+W.<%2Fauthor><AN>611526560<%2FAN><DT>Journal%3A+Article<%2FDT>)

44.

Effects of multidrug resistant tuberculosis treatment on patients' health related quality of life: Results from a follow up study.

Ahmad N., Javaid A., Sulaiman S.A.S., Basit A., Afridi A.K., Jaber A.A.S., Khan A.H.

PLoS ONE. 11 (7) (no pagination), 2016. Article Number: e0159560. Date of Publication: July 2016.

AN: 611503209

Background At present, within the management of multidrug resistant tuberculosis (MDR-TB) much attention is being paid to the traditional microbiological and clinical indicators. Evaluation of the impact of MDR-TB treatment on patients' Health Related Quality of Life (HRQoL) has remained a neglected area. Objective To evaluate the impact of MDR-TB treatment on patients HRQoL, and determine the predictors of variability in HRQoL along the course of treatment Methods A prospective follow up study was conducted at the programmatic management unit for drug resistant TB of Lady Reading Hospital Peshawar. Culture confirmed eligible MDR-TB patients were asked to self complete SF-36v2 at the baseline visit, and subsequently after the completion of 12 months of treatment and at the end of treatment. A score of <47 normbased scoring (NBS) points on component summary measures and health domain scales was considered indicative of function impairment. General linear model repeated measures ANOVA was used examine the change and predictors of change in physical component summary (PCS) and mental component summary (MCS) scores over the time. Results A total of 68 out of enrolled 81 eligible MDR-TB patients completed SF-36v2 questionnaire at the three time points. Patients' mean PCS scores at the three time points were, 38.2+/-4.7, 38.6+/-4.4 and 42.2+/-5.2 respectively, and mean MCS were 33.7+/-7.0, 35.5+/-6.9 and 40.0+/-6.9 respectively. Length of sickness prior to the diagnosis of MDR-TB was predictive of difference in PCS scores (F = 4.988, Df = 1, 66), whereas patients' gender (F = 5.638, Df = 1, 66) and length of sickness prior to the diagnosis of MDR-TB (F = 4.400, Df = 1, 66) were predictive of difference in MCS scores. Conclusion Despite the positive impact of MDR-TB treatment on patients' HRQoL, the scores on component summary measures suggested compromised physical and mental health even at the end of treatment. A large multicenter study is suggested to confirm the present findings. Copyright © 2016 Ahmad et al. This is an open access article distributed under the terms of the Creative Commons Attribution License, which permits unrestricted use, distribution, and reproduction in any medium, provided the original author and source are credited.

Institution

(Ahmad, Sulaiman, Jaber, Khan) Discipline of Clinical Pharmacy, School of Pharmaceutical Sciences, Universiti Sains Malaysia, Pulau Pinang, Malaysia (Javaid, Basit) Department of Pulmonology, Postgraduate Medical Institute Peshawar, Peshawar, Pakistan

(Afridi) PMDT, Lady Reading Hospital Peshawar, Peshawar, Pakistan

Publisher

Public Library of Science (E-mail: plos@plos.org)

Link to the Ovid Full Text or citation:

[Click here for full text options](http://ovidsp.ovid.com/ovidweb.cgi?T=JS&CSC=Y&NEWS=N&PAGE=fulltext&D=emed18b&AN=611503209)

Link to the External Link Resolver:

[SFX](http://whel-primo.hosted.exlibrisgroup.com/openurl/44WHELF_CAR/44WHELF_CAR_services_page?sid=OVID:embase&id=pmid:&id=doi:10.1371%2Fjournal.pone.0159560&issn=1932-6203&isbn=&volume=11&issue=7&spage=no+pagination&pages=no+pagination&date=2016&title=PLoS+ONE&atitle=Effects+of+multidrug+resistant+tuberculosis+treatment+on+patients'+health+related+quality+of+life%3A+Results+from+a+follow+up+study&aulast=Ahmad&pid=<author>Ahmad+N.%3BJavaid+A.%3BSulaiman+S.A.S.%3BBasit+A.%3BAfridi+A.K.%3BJaber+A.A.S.%3BKhan+A.H.<%2Fauthor><AN>611503209<%2FAN><DT>Journal%3A+Article<%2FDT>)

45.

Transient hyperglycemia in patients with tuberculosis in Tanzania: Implications for diabetes screening algorithms.

Boillat-Blanco N., Ramaiya K.L., Mganga M., Minja L.T., Bovet P., Schindler C., Von Eckardstein A., Gagneux S., Daubenberger C., Reither K., Probst-Hensch N.

Journal of Infectious Diseases. 213 (7) (pp 1163-1172), 2016. Date of Publication: 2016.

AN: 609202350

Background. Diabetes mellitus (DM) increases tuberculosis risk while tuberculosis, as an infectious disease, leads to hyperglycemia. We compared hyperglycemia screening strategies in controls and patients with tuberculosis in Dar es Salaam, Tanzania. Methods. Consecutive adults with tuberculosis and sex- and age-matched volunteers were included in a case-control study between July 2012 and June 2014. All underwent DM screening tests (fasting capillary glucose [FCG] level, 2-hour CG [2-hCG] level, and glycated hemoglobin A1c [HbA1c] level) at enrollment, and cases were tested again after receipt of tuberculosis treatment. Association of tuberculosis and its outcome with hyperglycemia was assessed using logistic regression analysis adjusted for sex, age, body mass index, human immunodeficiency virus infection status, and socioeconomic status. Patients with tuberculosis and newly diagnosed DM were not treated for hyperglycemia. Results. At enrollment, DM prevalence was significantly higher among patients with tuberculosis (n = 539; FCG level > 7 mmol/ L, 4.5% of patients, 2-hCG level > 11 mmol/L, 6.8%; and HbA1c level > 6.5%, 9.3%), compared with controls (n = 496; 1.2%, 3.1%, and 2.2%, respectively). The association between hyperglycemia and tuberculosis disappeared after tuberculosis treatment (adjusted odds ratio [aOR] for the FCG level: 9.6 [95% confidence interval {CI}, 3.7-24.7] at enrollment vs 2.4 [95% CI, .7-8.7] at follow-up; aOR for the 2-hCG level: 6.6 [95% CI, 4.0-11.1] vs 1.6 [95% CI, .8-2.9]; and aOR for the HbA1c level, 4.2 [95% CI, 2.9-6.0] vs 1.4 [95% CI, .9-2.0]). Hyperglycemia, based on the FCG level, at enrollment was associated with tuberculosis treatment failure or death (aOR, 3.3; 95% CI, 1.2-9.3). Conclusions. Transient hyperglycemia is frequent during tuberculosis, and DM needs confirmation after tuberculosis treatment. Performance of DM screening at tuberculosis diagnosis gives the opportunity to detect patients at risk of adverse outcome. Copyright © The Author 2015.

PMID

26609005 [<http://www.ncbi.nlm.nih.gov/pubmed/?term=26609005>]

Institution

(Boillat-Blanco, Minja, Reither) Ifakara Health Institute, Tanzania (Ramaiya) Shree Hindu Mandal Hospital, Tanzania

(Ramaiya) Muhimbili University of Health Sciences, Tanzania

(Mganga) Kinondoni Municipal Council, National Tuberculosis Program, Dar es Salaam, Tanzania

(Boillat-Blanco, Schindler, Gagneux, Daubenberger, Reither, Probst-Hensch) Swiss Tropical and Public Health Institute, Tanzania

(Boillat-Blanco, Schindler, Gagneux, Daubenberger, Reither, Probst-Hensch) Department of Sciences, University of Basel, Switzerland

(Boillat-Blanco) Infectious Diseases Service, Lausanne University Hospital, Rue du Bugnon 46, Lausanne 1011, Switzerland

(Bovet) Institute of Social and Preventive Medicine, Lausanne University Hospital, Switzerland

(Von Eckardstein) Institute of Clinical Chemistry, University of Zurich, University Hospital of Zurich, Switzerland

Publisher

Oxford University Press (E-mail: jnl.info@oup.co.uk)

Link to the Ovid Full Text or citation:

[Click here for full text options](http://ovidsp.ovid.com/ovidweb.cgi?T=JS&CSC=Y&NEWS=N&PAGE=fulltext&D=emed18b&AN=609202350)

Link to the External Link Resolver:

[SFX](http://whel-primo.hosted.exlibrisgroup.com/openurl/44WHELF_CAR/44WHELF_CAR_services_page?sid=OVID:embase&id=pmid:26609005&id=doi:10.1093%2Finfdis%2Fjiv568&issn=0022-1899&isbn=&volume=213&issue=7&spage=1163&pages=1163-1172&date=2016&title=Journal+of+Infectious+Diseases&atitle=Transient+hyperglycemia+in+patients+with+tuberculosis+in+Tanzania%3A+Implications+for+diabetes+screening+algorithms&aulast=Boillat-Blanco&pid=<author>Boillat-Blanco+N.%3BRamaiya+K.L.%3BMganga+M.%3BMinja+L.T.%3BBovet+P.%3BSchindler+C.%3BVon+Eckardstein+A.%3BGagneux+S.%3BDaubenberger+C.%3BReither+K.%3BProbst-Hensch+N.<%2Fauthor><AN>609202350<%2FAN><DT>Journal%3A+Article<%2FDT>)

46.

Social, economic, and immunological impacts of TB treatment in Eastern rural China.

Xiao X., Yuan L., Qiu L., Lu W., Jiang W., Zhao Q.

International Journal of Infectious Diseases. Conference: 17th International Congress on Infectious Diseases. Hyderabad India. Conference Start: 20160302. Conference End: 20160305. Conference Publication: (var.pagings). 45 (pp 250), 2016. Date of Publication: April 2016.

AN: 72245512

Background: Drug resistant TB in China has been rapidly growing and becoming a cause for critical public health concern. It was reported that the acquired drug-resistant TB mainly results from incompliance to treatment, uncompleted treatment, irregular treatment and unqualified health services. Methods & Materials: Case-cohort was established in four counties in eastern rural China. All registered active TB patients from April 2013 to March 2014 were investigated using structure questionnaire to collect demographic, geographic, socioeconomic information and disease profile. Enzyme-linked immunosorbent assay (ELISA) was used to assess the level of serum cytokines (IFN-gamma, IL - 4) before anti-TB treatment. All the participants were followed up 6 or 8 months until treatment was completed. Results: In total, 1404 patients were recruited, in which 1270 (94.5%) were newly diagnosed TB patients, 447 (33.8%) were smearpositive (SS+). The average age was 49.5+/-18.8, and 76.2% was male. The serum IFN-gamma and IL-4 before anti-TB treatment was 7.936 and 12.292 pg/ml respectively. 80.9% of newly diagnosed TB patients received standard anti-TB treatment, while 87.7% retreatment TB patients received standard anti-TB treatment. The successfully treated rate was 92.5% and 78.6% of new TB patients and retreatment TB patients respectively. The sputum conversion rate of SS+ TB patients after 2 month anti-TB treatment was 96.5%, while the cure rate and successful rate was 80.5% and 90.0% respectively. 92.6% smear-negative patients completed anti-TB treatment. The successful rate in Jiangsu (97.3%), the more developed area, was higher than in Jiangxi (87.6%) (OR = 6.276, 95% CI: 2.528 15.579). Patients with BMI<18.5 may be more risk to fail the treatment compared with the health BMI (18.5-23.9) patients (OR = 3.196, 95% CI: 1.592 6.418). There was no relationship between serum cytokines level and treatment outcomes. The cure rate of TB comorbidity with diabetes was same as the one without diabetes. Conclusion: The sputum conversion rate, cure rate and successful rate of TB patients were high in eastern rural China. Improving patients' nutrition, keeping a healthy BMI could be the key point of increasing successfully treated rate of tuberculosis. More research should be done in future to evaluate the impact of the serum cytokines during anti-TB treatment.

Institution

(Xiao, Yuan, Jiang, Zhao) School of Public Health, Fudan University, Shanghai, China (Qiu) Jiangxi Provincial Center For Disease Control And Prevention, Jiangxi, China

(Lu) Jiangsu Provincial Center For Disease Control And Prevention, Jiangsu, China

Publisher

Elsevier

Link to the Ovid Full Text or citation:

[Click here for full text options](http://ovidsp.ovid.com/ovidweb.cgi?T=JS&CSC=Y&NEWS=N&PAGE=fulltext&D=emed18a&AN=72245512)

Link to the External Link Resolver:

[SFX](http://whel-primo.hosted.exlibrisgroup.com/openurl/44WHELF_CAR/44WHELF_CAR_services_page?sid=OVID:embase&id=pmid:&id=doi:10.1016%2Fj.ijid.2016.02.559&issn=1201-9712&isbn=&volume=45&issue=&spage=250&pages=250&date=2016&title=International+Journal+of+Infectious+Diseases&atitle=Social%2C+economic%2C+and+immunological+impacts+of+TB+treatment+in+Eastern+rural+China&aulast=Xiao&pid=<author>Xiao+X.%3BYuan+L.%3BQiu+L.%3BLu+W.%3BJiang+W.%3BZhao+Q.<%2Fauthor><AN>72245512<%2FAN><DT>Journal%3A+Conference+Abstract<%2FDT>)

47.

Clinical spectrum and outcome of pulmonary nocardiosis: 5-year experience.

Singh A., Chhina D., Soni R.K., Kakkar C., Sidhu U.S.

Lung India. 33 (4) (pp 398-403), 2016. Date of Publication: July-August 2016.

AN: 611202282

Background: Pulmonary nocardiosis is a rare but a life-threatening infection caused by Nocardia spp. The diagnosis is often missed and delayed resulting in delay in appropriate treatment and thus higher mortality. Aim: In this study, we aim to evaluate the clinical spectrum and outcome of patients with pulmonary nocardiosis. Methods: A retrospective, 5-year (2009-2014) review of demographic profile, risk factors, clinical manifestations, imaging findings, treatment, and outcome of patients with pulmonary nocardiosis admitted to a tertiary care hospital. Results: The median age of the study subjects was 54 years (range, 16-76) and majority of them (75%) were males. The risk factors for pulmonary nocardiosis identified in our study were long-term steroid use (55.6%), chronic lung disease (52.8%), diabetes (27.8%), and solid-organ transplantation (22.2%). All the patients were symptomatic, and the most common symptoms were cough (91.7%), fever (78%), and expectoration (72%). Almost two-third of the patients were initially misdiagnosed and the alternative diagnosis included pulmonary tuberculosis (n = 7), community-acquired pneumonia (n = 5), lung abscess (n = 4), invasive fungal infection (n = 3), lung cancer (n = 2), and Wegener's granulomatosis (n = 2). The most common radiographic features were consolidation (77.8%) and nodules (56%). The mortality rate for indoor patients was 33% despite treatment. Higher mortality rate was observed among those who had brain abscess (100.0%), HIV positivity (100%), need for mechanical ventilation (87.5%), solid-organ transplantation (50%), and elderly (age > 60 years) patients (43%). Conclusion: The diagnosis of pulmonary nocardiosis is often missed and delayed resulting in delay in appropriate treatment and thus high mortality. A lower threshold for diagnosing pulmonary nocardiosis needs to be exercised, in chest symptomatic patients with underlying chronic lung diseases or systemic immunosuppression, for the early diagnosis, and treatment of this uncommon but potentially lethal disease. Despite treatment mortality remains high, especially in those with brain abscess, HIV positivity, need for mechanical ventilation, solid-organ transplantation, and elderly. Copyright © 2016 Indian Chest Society Published by Wolters Kluwer - Medknow.

Institution

(Singh, Sidhu) Department of Pulmonary Medicine, Dayanand Medical College and Hospital, Ludhiana, Punjab, India (Chhina) Department of Microbiology, Dayanand Medical College and Hospital, Ludhiana, Punjab, India

(Soni) Department of Social and Preventive Medicine, Dayanand Medical College and Hospital, Ludhiana, Punjab, India

(Kakkar) Department of Radiology, Dayanand Medical College and Hospital, Ludhiana, Punjab, India

Publisher

Medknow Publications (B9, Kanara Business Centre, off Link Road, Ghatkopar (E), Mumbai 400 075, India)

Link to the Ovid Full Text or citation:

[Click here for full text options](http://ovidsp.ovid.com/ovidweb.cgi?T=JS&CSC=Y&NEWS=N&PAGE=fulltext&D=emed18a&AN=611202282)

Link to the External Link Resolver:

[SFX](http://whel-primo.hosted.exlibrisgroup.com/openurl/44WHELF_CAR/44WHELF_CAR_services_page?sid=OVID:embase&id=pmid:&id=doi:10.4103%2F0970-2113.184873&issn=0970-2113&isbn=&volume=33&issue=4&spage=398&pages=398-403&date=2016&title=Lung+India&atitle=Clinical+spectrum+and+outcome+of+pulmonary+nocardiosis%3A+5-year+experience&aulast=Singh&pid=<author>Singh+A.%3BChhina+D.%3BSoni+R.K.%3BKakkar+C.%3BSidhu+U.S.<%2Fauthor><AN>611202282<%2FAN><DT>Journal%3A+Article<%2FDT>)

48.

Health and ethical consequences of outsourcing pivotal clinical trials to Latin America: A cross-sectional, descriptive study.

Homedes N., Ugalde A.

PLoS ONE. 11 (6) (no pagination), 2016. Article Number: e0157756. Date of Publication: June 2016.

AN: 610977356

Introduction: The implications of conducting clinical trials in low and middle income countries on the financial accessibility and safety of the pharmaceutical products available in those markets have not been studied. Regulatory practices and ethical declarations lead to the commercialization of the new products, referred to as New Molecular Entities (NMEs), in the countries where tested as soon as they are approved in high surveillance countries. Patients and patients' associations use the Latin American courts to access new and expensive treatments, regardless of their safety profile and therapeutic value. Design and Objectives: Cross-sectional, descriptive study. To determine the therapeutic value and safety profile of the NMEs approved by the Food and Drug Administration (FDA) in 2011 and 2012 that had been tested in Latin America, and the implications of their market approval for the pharmaceutical budgets in the countries where tested. Setting: Latin America. Measures: To assess the therapeutic value and safety of the NMEs commercialized in the different countries we used f independent drug bulletins. The prices of the NMEs for the consumers were obtained from the pharmaceutical price observatories of the countries were the medicines had been tested. If the price was not available in the observatories, it was obtained from pharmaceutical distributors. We used the countries' minimum wage and per capita income to calculate the financial accessibility of a course of treatment with the NMEs. Results: We found that 33 NMEs approved by the FDA in 2011 and 2012 have been tested in Latin America. Of these, 26 had been evaluated by independent drug bulletins and only five were found to add some value to a subset of patients and had significant side-effects. The pharmaceutical prices were very high, varied widely across countries and were unrelated to the countries' income per capita or minimum wage. Conclusion: The implementation of clinical trials in Latin America results in the commercialization of medicines with questionable safety profiles and limited therapeutic value, putting patients at risk and causing budgetary strains in pharmaceutical budgets. Copyright © 2016 Homedes, Ugalde. This is an open access article distributed under the terms of the Creative Commons Attribution License, which permits unrestricted use, distribution, and reproduction in any medium, provided the original author and source are credited.

Institution

(Homedes) Management Policy and Community Health, School of Public Health, University of Texas, El Paso, TX, United States (Homedes) Department of International Health, Georgetown University, Washington, DC, United States

(Ugalde) Department of Sociology, University of Texas, Austin, TX, United States

Publisher

Public Library of Science (E-mail: plos@plos.org)

Link to the Ovid Full Text or citation:

[Click here for full text options](http://ovidsp.ovid.com/ovidweb.cgi?T=JS&CSC=Y&NEWS=N&PAGE=fulltext&D=emed18a&AN=610977356)

Link to the External Link Resolver:

[SFX](http://whel-primo.hosted.exlibrisgroup.com/openurl/44WHELF_CAR/44WHELF_CAR_services_page?sid=OVID:embase&id=pmid:&id=doi:10.1371%2Fjournal.pone.0157756&issn=1932-6203&isbn=&volume=11&issue=6&spage=no+pagination&pages=no+pagination&date=2016&title=PLoS+ONE&atitle=Health+and+ethical+consequences+of+outsourcing+pivotal+clinical+trials+to+Latin+America%3A+A+cross-sectional%2C+descriptive+study&aulast=Homedes&pid=<author>Homedes+N.%3BUgalde+A.<%2Fauthor><AN>610977356<%2FAN><DT>Journal%3A+Article<%2FDT>)

49.

Diabetes mellitus in patients with pulmonary tuberculosis in an aging population in Shanghai, China: Prevalence, clinical characteristics and outcomes.

Wu Z., Guo J., Huang Y., Cai E., Zhang X., Pan Q., Yuan Z., Shen X.

Journal of Diabetes and its Complications. 30 (2) (pp 237-241), 2016. Date of Publication: 01 Mar 2016.

AN: 609073866

Aims To determine the prevalence of diabetes mellitus among pulmonary tuberculosis patients and the difference of clinical characteristics and outcomes between pulmonary tuberculosis patients with and without diabetes mellitus in an aging population in Shanghai, China. Methods This is a retrospective population-based study. 201 newly diagnosed pulmonary tuberculosis patients in Changning District, Shanghai during 2007-2008 were included. Clinical characteristics and outcomes were collected. Determination of diabetes mellitus was based on the medical records before pulmonary tuberculosis was diagnosed. Results The prevalence of diabetes mellitus among pulmonary tuberculosis patients was 19.9% (40/201). Pulmonary tuberculosis patients with diabetes mellitus were more likely to be old (> 50, OR = 5.23, 95% CI = 2.07-13.25), to have pulmonary cavities (OR = 3.02, 95% CI = 1.31-6.98), to be sputum smear positive (OR = 2.90, 95% CI = 1.12-7.51), and to have extension of anti-tuberculosis treatment duration (OR = 2.68, 95% CI 1.17-6.14). Besides, they had a higher 2nd month sputum smear positive proportion (OR = 2.97, 95% CI 1.22-7.22) and a higher 5-year recurrence rate (OR = 5.87, 95% CI 1.26-27.40). Conclusions High prevalence, severe clinical characteristics and poor outcomes of pulmonary tuberculosis patients with diabetes mellitus highlight the necessity of early bi-directional screening and co-management of these two diseases in Shanghai, China. Copyright © 2016 Elsevier Inc. All rights reserved.

Institution

(Wu, Guo, Pan, Yuan, Shen) Department of Tuberculosis Control, Shanghai Municipal Center for Disease Control and Prevention, Shanghai, China (Huang, Cai, Zhang) Shanghai Changning Center for Disease Control and Prevention, Shanghai, China

Publisher

Elsevier Inc. (E-mail: usjcs@elsevier.com)

Link to the Ovid Full Text or citation:

[Click here for full text options](http://ovidsp.ovid.com/ovidweb.cgi?T=JS&CSC=Y&NEWS=N&PAGE=fulltext&D=emed18a&AN=609073866)

Link to the External Link Resolver:

[SFX](http://whel-primo.hosted.exlibrisgroup.com/openurl/44WHELF_CAR/44WHELF_CAR_services_page?sid=OVID:embase&id=pmid:&id=doi:10.1016%2Fj.jdiacomp.2015.11.014&issn=1056-8727&isbn=&volume=30&issue=2&spage=237&pages=237-241&date=2016&title=Journal+of+Diabetes+and+its+Complications&atitle=Diabetes+mellitus+in+patients+with+pulmonary+tuberculosis+in+an+aging+population+in+Shanghai%2C+China%3A+Prevalence%2C+clinical+characteristics+and+outcomes&aulast=Wu&pid=<author>Wu+Z.%3BGuo+J.%3BHuang+Y.%3BCai+E.%3BZhang+X.%3BPan+Q.%3BYuan+Z.%3BShen+X.<%2Fauthor><AN>609073866<%2FAN><DT>Journal%3A+Article<%2FDT>)

50.

The influence of smoking on pulmonary tuberculosis in diabetic and non-diabetic patients.

Bai K.-J., Lee J.-J., Chien S.-T., Suk C.-W., Chiang C.-Y.

PLoS ONE. 11 (6) (no pagination), 2016. Article Number: e0156677. Date of Publication: 01 Jun 2016.

AN: 610786648

Background: Both smoking and diabetes can increase the risk and influence the manifestations and outcomes of tuberculosis (TB). It is not clear whether the influence of smoking on pulmonary TB differs between non-diabetic and diabetic patients. Herein, we assessed the manifestations and outcomes of TB in relation to smoking in both diabetic and non-diabetic TB patients. Methodology/Principal Findings: All diabetic culture-positive pulmonary TB patients notified from 2005-2010 at three teaching hospitals in Taiwan were enrolled. A culture-positive pulmonary TB patient without DM who was notified to the health authority immediately prior to each diabetic TB patient was selected for comparison. The 972 patients in this study cohort included 365 (37.6%) nondiabetic non-smokers, 149 (15.3%) non-diabetic smokers, 284 (29.2%) diabetic non-smokers, and 174 (17.9%) diabetic smokers. The adjusted relative risk of a pretreatment positive smear for a smoker compared with a non-smoker was 2.19 (95% CI 1.38-3.47) in non-diabetic patients and 2.23 (95% CI 1.29-3.87) in diabetic culture-positive pulmonary TB patients. The adjusted relative risk for a positive smear among diabetic smokers was 5.61 (95% CI 3.35-9.41) compared with non-diabetic non-smokers. Smoking was significantly associated with an increased frequency of bilateral lung parenchyma involvement (AdjOR 1.84, 95% CI 1.16-2.93), far-advanced pulmonary TB (AdjOR 1.91, 95% CI 1.04-3.50), cavitary lesions (AdjOR 2.03, 95% CI 1.29-3.20), and unfavorable outcomes of TB (AdjOR 2.35, 95% CI 1.02-5.41) in non-diabetic patients. However, smoking was not associated with cavitary lung parenchyma lesions regarding the location, number or size of the cavity in diabetic TB patients. Conclusions/Significance: Smoking and diabetes have joint effects on a pretreatment positive smear. Diabetic smokers had more than a 5-fold increased risk of a pretreatment positive smear than did non-diabetic non-smokers, indicating remarkable joint effects of diabetes and smoking on the risk of TB transmission. Copyright © 2016 Bai et al. This is an open access article distributed under the terms of the Creative Commons Attribution License, which permits unrestricted use, distribution, and reproduction in any medium, provided the original author and source are credited.

Institution

(Bai, Suk, Chiang) Division of Pulmonary Medicine, Department of Internal Medicine, Wan Fang Hospital, Taipei Medical University, Taipei, Taiwan (Republic of China) (Bai) School of Respiratory Therapy, College of Medicine, Taipei Medical University, Taipei, Taiwan (Republic of China)

(Lee) Department of Internal Medicine, Buddhist Tzu Chi General Hospital, Tzu Chi University, Hualien, Taiwan (Republic of China)

(Chien) Chest Hospital, Department of Health and Welfare, Tainan, Taiwan (Republic of China)

(Chiang) Department of Internal Medicine, School of Medicine, College of Medicine, Taipei Medical University, Taipei, Taiwan (Republic of China)

(Chiang) International Union Against Tuberculosis and Lung Disease, Paris, France

Publisher

Public Library of Science (E-mail: plos@plos.org)

Link to the Ovid Full Text or citation:

[Click here for full text options](http://ovidsp.ovid.com/ovidweb.cgi?T=JS&CSC=Y&NEWS=N&PAGE=fulltext&D=emed18a&AN=610786648)

Link to the External Link Resolver:

[SFX](http://whel-primo.hosted.exlibrisgroup.com/openurl/44WHELF_CAR/44WHELF_CAR_services_page?sid=OVID:embase&id=pmid:&id=doi:10.1371%2Fjournal.pone.0156677&issn=1932-6203&isbn=&volume=11&issue=6&spage=no+pagination&pages=no+pagination&date=2016&title=PLoS+ONE&atitle=The+influence+of+smoking+on+pulmonary+tuberculosis+in+diabetic+and+non-diabetic+patients&aulast=Bai&pid=<author>Bai+K.-J.%3BLee+J.-J.%3BChien+S.-T.%3BSuk+C.-W.%3BChiang+C.-Y.<%2Fauthor><AN>610786648<%2FAN><DT>Journal%3A+Article<%2FDT>)

51.

Glycated hemoglobin screening identifies patients admitted for retreatment of tuberculosis at risk for diabetes in Tanzania.

Sariko M.L., Mpagama S.G., Gratz J., Kisonga R., Saidi Q., Kibiki G.S., Heysell S.K.

Journal of Infection in Developing Countries. 10 (4) (pp 423-426), 2016. Date of Publication: April 2016.

AN: 610201261

Introduction: World Health Organization recommendations of bidirectional screening for tuberculosis (TB) and diabetes have been met with varying levels of uptake by national TB programs in resource-limited settings. Methodology: Kibong'oto Infectious Diseases Hospital (KIDH) is a referral hospital for TB from northern Tanzania, and the national referral hospital for multidrug-resistant (MDR)-TB. Glycated hemoglobin (HgbA1c) testing was done on patients admitted to KIDH for newly diagnosed TB, retreatment TB, and MDR-TB, to determine the point prevalence of diabetes (HgbA1c > 6.5%) and prediabetes (HgbA1c 5.7%-6.4%). Results: Of 148 patients hospitalized at KIDH over a single week, 59 (38%) had no prior TB treatment, 22 (15%) were retreatment cases, and 69 (47%) had MDR-TB. Only 3 (2%) had a known history of diabetes. A total of 144 (97%) had successful screening, of which 110 (77%) had an HgbA1c < 5.6%, 28 (19%) had > 5.7 < 6.5, and 6 (4%) had > 6.5. Comparing subjects with prediabetes or diabetes to those with normal A1c levels, retreatment patients were significantly more likely to have a A1c > 5.7% (odds ratio: 3.2, 95% CI: 1.2-9.0; p = 0.02) compared to those without prior TB treatment. No retreatment case was a known diabetic, thus the number needed to screen to diagnose one new case of diabetes among retreatment cases was 11. Conclusions: Diabetes prevalence by HgbA1c was less common than expected, but higher HgA1c values were significantly more frequent among retreatment cases, allowing for a rational, resource-conscious screening approach. Copyright © 2016 Sariko et al.

Institution

(Sariko, Saidi, Kibiki) Kilimanjaro Clinical Research Institute, Moshi, Tanzania (Mpagama, Kisonga) Kibong'oto Infectious Diseases Hospital, Kilimanjaro, Sanya Juu, Tanzania

(Gratz, Heysell) Division of Infectious Diseases and International Health, University of Virginia, Charlottesville, VA, United States

Publisher

Journal of Infection in Developing Countries (E-mail: info@jidc.org )

Link to the Ovid Full Text or citation:

[Click here for full text options](http://ovidsp.ovid.com/ovidweb.cgi?T=JS&CSC=Y&NEWS=N&PAGE=fulltext&D=emed18a&AN=610201261)

Link to the External Link Resolver:

[SFX](http://whel-primo.hosted.exlibrisgroup.com/openurl/44WHELF_CAR/44WHELF_CAR_services_page?sid=OVID:embase&id=pmid:&id=doi:10.3855%2Fjidc.7324&issn=2036-6590&isbn=&volume=10&issue=4&spage=423&pages=423-426&date=2016&title=Journal+of+Infection+in+Developing+Countries&atitle=Glycated+hemoglobin+screening+identifies+patients+admitted+for+retreatment+of+tuberculosis+at+risk+for+diabetes+in+Tanzania&aulast=Sariko&pid=<author>Sariko+M.L.%3BMpagama+S.G.%3BGratz+J.%3BKisonga+R.%3BSaidi+Q.%3BKibiki+G.S.%3BHeysell+S.K.<%2Fauthor><AN>610201261<%2FAN><DT>Journal%3A+Article<%2FDT>)

52.

Global urine metabolomics in patients treated with first-line tuberculosis drugs and identification of a novel metabolite of ethambutol.

Das M.K., Arya R., Debnath S., Debnath R., Lodh A., Bishwal S.C., Das A., Nanda R.K.

Antimicrobial Agents and Chemotherapy. 60 (4) (pp 2257-2264), 2016. Date of Publication: April 2016.

AN: 609689782

Population level variation of drug metabolism phenotype (DMP) has great implications in treatment outcome, drug-related side effects, and resistance development. In this study, we used a gas chromatography-time of flight-mass spectrometry (GC-TOF-MS)-based untargeted urine metabolomics approach to understand the DMP of a tuberculosis (TB) patient cohort (n = 20) from Tripura, a state in the northeastern part of India. Urine samples collected at different postdose time points (2 h, 6 h, 12 h, 24 h, 36 h, and 48 h) from these newly diagnosed TB patients receiving first-line anti-TB drugs were analyzed, and we have successfully detected three of the four first-line drugs, viz., isoniazid (INH), ethambutol (ETB), andpyrazinamide (PZA). The majority of their known metabolites, acetyl-isoniazid (AcINH), isonicotinic acid (INA), isonicotinuric acid (INTA), 2,2'-(ethylenediimino)-dibutyric acid (EDBA), 5-hydroxypyrazinamide (5OH-PZA), pyrazinoic acid (POA), and 5-hydroxypyrazinoic acid (5OH-POA), were also detected. Analyzing the variation in abundances of drugs and their known metabolites and calculating the metabolic ratios in these samples, we offer comprehensive DMP information on this small patient cohort that represents Tripura, India. The majority (75%) of these patients are found to be slow acetylators of INH. The average metabolic ratios of POA/PZA and 5OH-POA/POA are 3.16 +/- 3.03 and 6.09 +/- 6.15, respectively. Employing correlation analysis of the metabolomics metadata and a manual prediction of drug catabolism, we have proposed 2-aminobutyric acid (AABA) as a novel metabolite of ETB. These observations indicate the usefulness of GC-MS-based metabolomics to characterize the DMP at a population level and also to identify novel drug metabolites. Copyright © 2016, American Society for Microbiology. All Rights Reserved.

Institution

(Das, Arya, Bishwal, Nanda) Immunology Group, International Centre for Genetic Engineering and Biotechnology (ICGEB), New Delhi, India (Debnath, Debnath, Lodh, Das) Department of Tuberculosis and Respiratory Disease, Agartala Government Medical College, Agartala, Tripura, India

(Bishwal) School of Life Sciences, Sambalpur University, Sambalpur, Odisha, India

Publisher

American Society for Microbiology (E-mail: Journals@asmusa.org)

Link to the Ovid Full Text or citation:

[Click here for full text options](http://ovidsp.ovid.com/ovidweb.cgi?T=JS&CSC=Y&NEWS=N&PAGE=fulltext&D=emed18a&AN=609689782)

Link to the External Link Resolver:

[SFX](http://whel-primo.hosted.exlibrisgroup.com/openurl/44WHELF_CAR/44WHELF_CAR_services_page?sid=OVID:embase&id=pmid:&id=doi:10.1128%2FAAC.02586-15&issn=0066-4804&isbn=&volume=60&issue=4&spage=2257&pages=2257-2264&date=2016&title=Antimicrobial+Agents+and+Chemotherapy&atitle=Global+urine+metabolomics+in+patients+treated+with+first-line+tuberculosis+drugs+and+identification+of+a+novel+metabolite+of+ethambutol&aulast=Das&pid=<author>Das+M.K.%3BArya+R.%3BDebnath+S.%3BDebnath+R.%3BLodh+A.%3BBishwal+S.C.%3BDas+A.%3BNanda+R.K.<%2Fauthor><AN>609689782<%2FAN><DT>Journal%3A+Article<%2FDT>)

53.

Diabetes mellitus is associated with increased mortality during tuberculosis treatment: A prospective cohort study among tuberculosis patients in South-Eastern Amahra Region, Ethiopia.

Workneh M.H., Bjune G.A., Yimer S.A.

Infectious Diseases of Poverty. 5 (1) (no pagination), 2016. Article Number: 22. Date of Publication: March 21, 2016.

AN: 609673076

Background: There is growing evidence suggesting that diabetes mellitus (DM) affects disease presentation and treatment outcome in tuberculosis (TB) patients. This study aimed at investigating the role of DM on clinical presentations and treatment outcomes among newly diagnosed TB patients. Methods: A prospective cohort study was conducted in South-Eastern Amhara Region, Ethiopia from September 2013 till March 2015. Study subjects were consecutively recruited from 44 randomly selected health facilities in the study area. Participants were categorized into two patient groups, namely, patients with TB and DM (TBDM) and TB patients without DM (TBNDM). Findings on clinical presentations and treatment outcomes were compared between the two patient groups. Cox proportional hazard regression analysis was applied to identify factors associated with death. Results: Out of 1314 TB patients enrolled in the study, 109 (8.3 %) had coexisting DM. TBDM comorbidity [adjusted hazard ratio (AHR) 3.96; 95 % confidence interval (C.I.) (1.76-8.89)], and TB coinfection with human immunodeficiency virus (HIV) [AHR 2.59; 95 % C.I. (1.21-5.59)] were associated with increased death. TBDM and TBNDM patients did not show significant difference in clinical symptoms at baseline and during anti-TB treatment period. However, at the 2nd month of treatment, TBDM patients were more symptomatic compared to patients in the TBNDM group. Conclusions: The study showed that DM is associated with increased death during TB treatment. DM has no association with clinical presentation of TB except at the end of the intensive phase treatment. Routine screening of TB patients for DM is recommended for early diagnosis and treatment of patients with TBDM comorbidity. Copyright © 2016 Workneh et al.

Institution

(Workneh, Bjune, Yimer) University of Oslo, Institute of Health and Society, Faculty of Medicine, Oslo, Norway (Workneh, Yimer) Amhara Regional State Health Bureau, Bahir-Dar, Ethiopia

(Yimer) Oslo University Hospital, Department of Microbiology, Oslo, Norway

(Yimer) Norwegian Institute of Public Health, Department of Bacteriology and Immunology, Oslo, Norway

Publisher

BioMed Central Ltd. (E-mail: info@biomedcentral.com)

Link to the Ovid Full Text or citation:

[Click here for full text options](http://ovidsp.ovid.com/ovidweb.cgi?T=JS&CSC=Y&NEWS=N&PAGE=fulltext&D=emed18a&AN=609673076)

Link to the External Link Resolver:

[SFX](http://whel-primo.hosted.exlibrisgroup.com/openurl/44WHELF_CAR/44WHELF_CAR_services_page?sid=OVID:embase&id=pmid:&id=doi:10.1186%2Fs40249-016-0115-z&issn=2049-9957&isbn=&volume=5&issue=1&spage=no+pagination&pages=no+pagination&date=2016&title=Infectious+Diseases+of+Poverty&atitle=Diabetes+mellitus+is+associated+with+increased+mortality+during+tuberculosis+treatment%3A+A+prospective+cohort+study+among+tuberculosis+patients+in+South-Eastern+Amahra+Region%2C+Ethiopia&aulast=Workneh&pid=<author>Workneh+M.H.%3BBjune+G.A.%3BYimer+S.A.<%2Fauthor><AN>609673076<%2FAN><DT>Journal%3A+Article<%2FDT>)

54.

Clinical outcomes of kidney transplants on patients with end-stage renal disease secondary to lupus nephritis, polycystic kidney disease and diabetic nephropathy. <Resultados clinicos de trasplantes de rinon en pacientes con enfermedad renal en etapa terminal secundaria a nefritis lupus, enfermedad poliquistica renal y nefropatia diabetica.>

Nieto-Rios J.F., Serna-Higuita L.M., Builes-Rodriguez S.A., Restrepo-Correa R.C., Aristizabal-Alzate A., Ocampo-Kohn C., Serna-Campuzano A., Cardona-Diaz N., Giraldo-Ramirez N.D., Zuluaga-Valencia G.A.

Colombia Medica. 47 (1) (pp 51-58), 2016. Date of Publication: January-March 2016.

AN: 609639332

Background: Patients with lupus nephritis could progress to endstage renal disease (10-22%); hence, kidney transplants should be considered as the treatment of choice for these patients. Objective: To evaluate the clinical outcomes after kidney transplants in patients with chronic kidney diseases secondary to lupus nephritis, polycystic kidney disease and diabetes nephropathy at Pablo Tobon Uribe Hospital. Methods: A descriptive and retrospective study performed at one kidney transplant center between 2005 and 2013. Results: A total of 136 patients, 27 with lupus nephritis (19.9%), 31 with polycystic kidney disease (22.8%) and 78 with diabetes nephropathy (57.4%), were included in the study. The graft survivals after one, three and five years were 96.3%, 82.5% and 82.5% for lupus nephritis; 90%, 86% and 76.5% for polycystic kidney disease and 91.7%, 80.3% and 67.9% for diabetes nephropathy, respectively, with no significant differences (p= 0.488); the rate of lupus nephritis recurrence was 0.94%/person-year. The etiology of lupus vs diabetes vs polycystic disease was not a risk factor for a decreased time of graft survival (Hazard ratio: 1.43; 95% CI: 0.52-3.93). Conclusion: Kidney transplant patients with end stage renal disease secondary to lupus nephritis has similar graft and patient survival success rates to patients with other kidney diseases. The complication rate and risk of recurrence for lupus nephritis are low. Kidney transplants should be considered as the treatment of choice for patients with end stage renal disease secondary to lupus nephritis. Copyright © 2016. Universidad del Valle.

Institution

(Nieto-Rios, Serna-Higuita, Aristizabal-Alzate, Ocampo-Kohn, Cardona-Diaz, Giraldo-Ramirez, Zuluaga-Valencia) Seccion Nefrologia, Hospital Pablo Tobon Uribe, Medellin, Colombia (Nieto-Rios, Aristizabal-Alzate, Ocampo-Kohn, Zuluaga-Valencia) Seccion de Nefrologia, Universidad de Antioquia, Medellin, Colombia

(Serna-Higuita) Seccion de Pediatria y Puericultura, Universidad de Antioquia, Medellin, Colombia

(Builes-Rodriguez, Serna-Campuzano) Seccion de Nefrologia, Universidad Pontificia Bolivariana, Medellin, Colombia

(Restrepo-Correa) Seccion de Medicina Interna, Hospital Pablo Tobon Uribe, Medellin, Colombia

Publisher

Facultad de Salud de la Universidad del Valle (E-mail: comedica@univalle.edu.co)

Link to the Ovid Full Text or citation:

[Click here for full text options](http://ovidsp.ovid.com/ovidweb.cgi?T=JS&CSC=Y&NEWS=N&PAGE=fulltext&D=emed18a&AN=609639332)

Link to the External Link Resolver:

[SFX](http://whel-primo.hosted.exlibrisgroup.com/openurl/44WHELF_CAR/44WHELF_CAR_services_page?sid=OVID:embase&id=pmid:&id=doi:&issn=0120-8322&isbn=&volume=47&issue=1&spage=51&pages=51-58&date=2016&title=Colombia+Medica&atitle=Resultados+clinicos+de+trasplantes+de+rinon+en+pacientes+con+enfermedad+renal+en+etapa+terminal+secundaria+a+nefritis+lupus%2C+enfermedad+poliquistica+renal+y+nefropatia+diabetica&aulast=Nieto-Rios&pid=<author>Nieto-Rios+J.F.%3BSerna-Higuita+L.M.%3BBuiles-Rodriguez+S.A.%3BRestrepo-Correa+R.C.%3BAristizabal-Alzate+A.%3BOcampo-Kohn+C.%3BSerna-Campuzano+A.%3BCardona-Diaz+N.%3BGiraldo-Ramirez+N.D.%3BZuluaga-Valencia+G.A.<%2Fauthor><AN>609639332<%2FAN><DT>Journal%3A+Article<%2FDT>)

55.

The clinical outcomes of oldest old patients with tuberculosis treated by regimens containing rifampicin, isoniazid, and pyrazinamide.

Huang-Shen L., Cheng C.-W., Lin M.-S., Chou Y.-L., Chang P.-J., Lin J.-C., Ye J.-J.

Clinical Interventions in Aging. 11 (pp 299-306), 2016. Date of Publication: 11 Mar 2016.

AN: 609001677

Objectives: To investigate the clinical characteristics, adverse drug reactions, and outcomes of the oldest old patients (aged >80 years) with tuberculosis (TB) treated with rifampicin, isoniazid, and pyrazinamide (RIP)-containing regimens. Design: A retrospective chart review study. Setting: A 1,200-bed tertiary teaching hospital in southwest Taiwan. Participants: We conducted a retrospective observational study between January 1, 2005 and December 31, 2011. Seven hundred adult patients (aged >18 years) with TB treated with RIP-containing anti-TB regimens were reviewed, including 161 oldest old patients. Outcome measures: Clinical outcomes included clinical responsiveness and microbiological eradication. Adverse outcomes included drug-induced hepatitis, and other symptoms included gastrointestinal upset (eg, abdominal pain, vomiting, diarrhea, or dyspepsia), skin rash, joint pain, and hyperuricemia. Results: Compared with the non-oldest old adult patients, the oldest old patients more frequently had hepatitis (P=0.014), gastrointestinal upset (P=0.029), and unfavorable outcomes (P<0.001). In a multivariate analysis, hepatitis during treatment (adjusted odds ratio: 3.482, 95% confidence interval: 1.537-7.885; P<0.003) and oldest old age (adjusted odds ratio: 5.161, 95% confidence interval: 2.294-11.613; P<0.010) were independent risk factors for unfavorable outcomes. In the oldest old patients with hepatitis, rifampicin use was more common in the favorable outcome group than in the unfavorable outcome group (100% vs 37.5%; P=0.001). Conclusion: The oldest old age and hepatitis during RIP treatment were associated with unfavorable outcomes. For the oldest old patients with TB having hepatitis during treatment, rifampicin rechallenge and use might benefit the treatment outcome. Copyright © 2016 Lin et al.

Institution

(Huang-Shen) Division of Infectious Diseases, Department of Internal Medicine, Chang gung Memorial Hospital, Chia-Yi, Taiwan (Republic of China) (Huang-Shen, Chang) Graduate Institute of Clinical Medical Sciences, College of Medicine, Chang gung University, Taoyuan, Taiwan (Republic of China)

(Cheng, Ye) Division of Infectious Diseases, Department of Internal Medicine, Chang gung Memorial Hospital at linkou, Chang gung University College of Medicine, Taoyuan, Taiwan (Republic of China)

(Lin) Division of Cardiology, Chang gung Memorial Hospital, Yunlin, Taiwan (Republic of China)

(Chou) Division of Pulmonary and Critical Care Medicine, Department of Internal Medicine, Chang gung Memorial Hospital, Chia-Yi, Taiwan (Republic of China)

(Lin) Division of Allergy and Immunology and rheumatology, Department of Internal Medicine, Chang gung Memorial Hospital, Chia-Yi, Taiwan (Republic of China)

Publisher

Dove Medical Press Ltd. (PO Box 300-008, Albany, Auckland, New Zealand)

Link to the Ovid Full Text or citation:

[Click here for full text options](http://ovidsp.ovid.com/ovidweb.cgi?T=JS&CSC=Y&NEWS=N&PAGE=fulltext&D=emed18a&AN=609001677)

Link to the External Link Resolver:

[SFX](http://whel-primo.hosted.exlibrisgroup.com/openurl/44WHELF_CAR/44WHELF_CAR_services_page?sid=OVID:embase&id=pmid:&id=doi:10.2147%2FCIA.S95411&issn=1176-9092&isbn=&volume=11&issue=&spage=299&pages=299-306&date=2016&title=Clinical+Interventions+in+Aging&atitle=The+clinical+outcomes+of+oldest+old+patients+with+tuberculosis+treated+by+regimens+containing+rifampicin%2C+isoniazid%2C+and+pyrazinamide&aulast=Huang-Shen&pid=<author>Huang-Shen+L.%3BCheng+C.-W.%3BLin+M.-S.%3BChou+Y.-L.%3BChang+P.-J.%3BLin+J.-C.%3BYe+J.-J.<%2Fauthor><AN>609001677<%2FAN><DT>Journal%3A+Review<%2FDT>)

56.

Does enhanced diabetes management reduce the risk and improve the outcome of tuberculosis?.

Lo H.-Y., Yang S.-L., Lin H.-H., Bai K.-J., Lee J.-J., Lee T.-I., Chiang C.-Y.

International Journal of Tuberculosis and Lung Disease. 20 (3) (pp 376-382), 2016. Date of Publication: 01 Mar 2016.

AN: 608513164

SETTING: The Bureau of National Health Insurance (NHI) has implemented a pay-for-performance (p4p) programme for diabetes mellitus (DM) in Taiwan. OBJECTIVE : To investigate whether patients with DM enrolled in the p4p programme (DM-p4p) are less likely to develop tuberculosis (TB) and whether they have a better outcome than patients with DMnot enrolled in the p4p programme (DM-non-p4p) if they do develop TB. DESIGN: A random sample of 79 471 DM-p4p, 100 000 DM-non-p4p and 100 000 non-diabetic patients (non-DM) was obtained from the 2008-2009 NHI database, and the patients were matched with the National TB Registry to determine whether they had developed TB by the end of 2010. RESULTS : The average annual incidence of TB was respectively 259.9 (95%CI 230.2-293.4), 137.5 (95%CI 116.4-162.5) and 74.1 (95%CI 59.0-93.0) per 100 000 population among DM-non-p4p, DM-p4p and non-DM patients. The relative risk of death over treatment success was 1.79 (95%CI 1.05-3.04) among DM-non-p4p and 1.69 (95%CI 0.84-3.40) among non-DM patients, relative to DM-p4p patients. CONCLUSIONS : Enhanced case management of DM reduced risk and improved outcomes of TB among patients with DM. Copyright © 2016 The Union.

Institution

(Lo, Yang) Taiwan Centers for Disease Control, Ministry of Health and Welfare, Taipei, Taiwan (Republic of China) (Lin) Institute of Epidemiology and Preventive Medicine, National Taiwan University, Taipei, Taiwan (Republic of China)

(Bai) School of Respiratory Therapy, College of Medicine, Taipei Medical University, Taipei, Taiwan (Republic of China)

(Bai, Chiang) Division of Pulmonary Medicine, Department of Internal Medicine, Wan Fang Hospital, Taipei Medical University, 111 Hsin-Long Road, Taipei, Taiwan (Republic of China)

(Lee) Department of Internal Medicine, Tzu Chi General Hospital and Tzu Chi University, Hualien, Taiwan (Republic of China)

(Lee) Division of Endocrinology and Metabolism, Department of Internal Medicine, Wan Fang Hospital, Taipei Medical University, Taipei, Taiwan (Republic of China)

(Lee) Department of General Medicine, School of Medicine, College of Medicine, Taipei Medical University, Taipei, Taiwan (Republic of China)

(Chiang) Department of Internal Medicine, School of Medicine, College of Medicine, Taipei Medical University, Taipei, Taiwan (Republic of China)

(Chiang) International Union Against Tuberculosis and Lung Disease, Paris, France

Publisher

International Union against Tubercul. and Lung Dis.

Link to the Ovid Full Text or citation:

[Click here for full text options](http://ovidsp.ovid.com/ovidweb.cgi?T=JS&CSC=Y&NEWS=N&PAGE=fulltext&D=emed18a&AN=608513164)

Link to the External Link Resolver:

[SFX](http://whel-primo.hosted.exlibrisgroup.com/openurl/44WHELF_CAR/44WHELF_CAR_services_page?sid=OVID:embase&id=pmid:&id=doi:10.5588%2Fijtld.15.0654&issn=1027-3719&isbn=&volume=20&issue=3&spage=376&pages=376-382&date=2016&title=International+Journal+of+Tuberculosis+and+Lung+Disease&atitle=Does+enhanced+diabetes+management+reduce+the+risk+and+improve+the+outcome+of+tuberculosis%3F&aulast=Lo&pid=<author>Lo+H.-Y.%3BYang+S.-L.%3BLin+H.-H.%3BBai+K.-J.%3BLee+J.-J.%3BLee+T.-I.%3BChiang+C.-Y.<%2Fauthor><AN>608513164<%2FAN><DT>Journal%3A+Article<%2FDT>)

57.

Incidence of tuberculosis is high in chronic kidney disease patients in South East England and drug resistance common.

Ostermann M., Palchaudhuri P., Riding A., Begum P., Milburn H.J.

Renal Failure. 38 (2) (pp 256-261), 2016. Date of Publication: 07 Feb 2016.

AN: 607623274

The risk of tuberculosis (TB) is significantly increased in chronic kidney disease (CKD). Data on TB in CKD in the UK are sparse; most information stems from countries with high background prevalence. The aim of this study was to estimate the incidence of TB in CKD patients in South East London and to describe the epidemiology, treatment, and outcome. CKD patients with TB between 1994 and 2010 were identified retrospectively. Data were collected on type of renal replacement therapy, the method of TB diagnosis, disease site, treatment regimens, and risk factors. Forty patients were identified of whom 67.5% had CKD stages IV-V. Sixty-five percent were from non-UK born ethnic minorities. Median time from diagnosis of CKD to TB development was 12 months (range 0-192 months). Cumulative incidence of TB was 1267/100,000 [95% confidence interval (CI): 630-1904; 85 x background UK rate] in hemodialysis patients; 398/100,000 (95% CI: 80-1160; 26 x background UK rate) in peritoneal dialysis; and 522/100,000 (CI: 137-909; 35 x background UK rate) in transplant recipients. Sixty-three percent of patients had pulmonary TB and 25% of patients with culture-positive TB had resistant isolates. Fifty percent of patients were immunosuppressed due to drugs, diabetes, and/or retroviral disease. Treatment regimens were according to recent national guidance in 73% of cases. Seventy-six percent of patients experienced side effects. Greater awareness of risk factors, drug resistance, treatment regimens, and potential side effects is needed. Copyright © 2015 Taylor & Francis.

Institution

(Ostermann) Department of Renal Medicine, Guys and St Thomas NHS Foundation Trust, London, United Kingdom (Ostermann, Milburn) Guys and St Thomas NHS Foundation Trust, Kings College London, Great Maze Pond, London SE1 9RT, United Kingdom

(Palchaudhuri, Riding, Begum, Milburn) Department of Respiratory Medicine, Guys and St Thomas NHS Foundation Trust, London, United Kingdom

Publisher

Taylor and Francis Ltd (E-mail: healthcare.enquiries@informa.com)

Link to the Ovid Full Text or citation:

[Click here for full text options](http://ovidsp.ovid.com/ovidweb.cgi?T=JS&CSC=Y&NEWS=N&PAGE=fulltext&D=emed18a&AN=607623274)

Link to the External Link Resolver:

[SFX](http://whel-primo.hosted.exlibrisgroup.com/openurl/44WHELF_CAR/44WHELF_CAR_services_page?sid=OVID:embase&id=pmid:&id=doi:10.3109%2F0886022X.2015.1128290&issn=0886-022X&isbn=&volume=38&issue=2&spage=256&pages=256-261&date=2016&title=Renal+Failure&atitle=Incidence+of+tuberculosis+is+high+in+chronic+kidney+disease+patients+in+South+East+England+and+drug+resistance+common&aulast=Ostermann&pid=<author>Ostermann+M.%3BPalchaudhuri+P.%3BRiding+A.%3BBegum+P.%3BMilburn+H.J.<%2Fauthor><AN>607623274<%2FAN><DT>Journal%3A+Article<%2FDT>)

58.

Efficacy and effect of free treatment on multidrug-resistant tuberculosis.

Zhang Q., Wu Z., Zhang Z., Sha W., Shen X., Xiao H.

Experimental and Therapeutic Medicine. 11 (3) (pp 777-782), 2016. Date of Publication: March 2016.

AN: 607998358

Multidrug-resistant tuberculosis (MDR-TB) is a serious public health and social issue. It pertains to the type of tuberculosis that is resistant simultaneously to isoniazid and rifampicin. MDR-TB has a high mortality and is expensive to treat. The aim of the present study was to examine the therapeutic effects of individualized free treatment and the relevant influencing factors on the treatment outcome for MDR-TB. A prospective study module was used to analyze the therapeutic outcome of MDR-TB with individualized free treatment for 160 patients between 2011 and 2014. Statistical analysis was performed using the Chi-square or Fisher's exact test, and the odds ratio was calculated using a logistic regression analysis model. In total, 160 patients were enrolled in the study for treatment of MDR-TB. From these, 88 cases completed the course of treatment, and 70 cases were successfully treated. Of the remaining 72 cases, 37 cases exhibited treatment failure, 18 cases were suspended during treatment and 17 patients succumbed to the disease. The results showed that the confounding factors were: i) retreatment (p<0.05); ii) occurrence of diabetes (p<0.001); iii) lesion without improvement in radiography during treatment (p=0.001); iv) positive sputum culture for Mycobacterium tuberculosis after 3-month treatment (p<0.05); and v) termination of treatment due to adverse reaction (p<0.05). These factors were associated with poor treatment outcomes by logistic regression analysis. Adverse drug reaction was observed in 33 cases and treatment was terminated or changed permanently in 29 of these cases. The most common adverse reaction was liver function damage caused by pyrazinamide and leucopenia caused by rifabutin. One patient suffered from serious liver failure. In conclusion, the success rate of long treatment course for MDR-TB is not high due to many adverse reactions. Occurrence of diabetes is the main factor that caused poor efficacy. Copyright © 2016, Spandidos Publications. All Rights reserved.

Institution

(Zhang, Sha, Xiao) Clinic and Research Center of Tuberculosis, Shanghai Key Laboratory of Tuberculosis, Shanghai Pulmonary Hospital, Tongji University School of Medicine, Shanghai 200433, China (Wu, Zhang, Shen) Shanghai Center for Disease Control and Prevention, Shanghai 200065, China

Publisher

Spandidos Publications (10 Vriaxidos Street, Athens 116 10, Greece)

Link to the Ovid Full Text or citation:

[Click here for full text options](http://ovidsp.ovid.com/ovidweb.cgi?T=JS&CSC=Y&NEWS=N&PAGE=fulltext&D=emed18a&AN=607998358)

Link to the External Link Resolver:

[SFX](http://whel-primo.hosted.exlibrisgroup.com/openurl/44WHELF_CAR/44WHELF_CAR_services_page?sid=OVID:embase&id=pmid:&id=doi:10.3892%2Fetm.2015.2966&issn=1792-0981&isbn=&volume=11&issue=3&spage=777&pages=777-782&date=2016&title=Experimental+and+Therapeutic+Medicine&atitle=Efficacy+and+effect+of+free+treatment+on+multidrug-resistant+tuberculosis&aulast=Zhang&pid=<author>Zhang+Q.%3BWu+Z.%3BZhang+Z.%3BSha+W.%3BShen+X.%3BXiao+H.<%2Fauthor><AN>607998358<%2FAN><DT>Journal%3A+Article<%2FDT>)

59.

Diabetes is associated with worse clinical presentation in tuberculosis patients from Brazil: A retrospective cohort study.

Gil-Santana L., Almeida J.L., Oliveira C.A.M., Hickson L.S., Daltro C., Castro S., Kornfeld H., Netto E.M., Andrade B.B.

PLoS ONE. 11 (1) (no pagination), 2016. Article Number: e0146876. Date of Publication: 11 Jan 2016.

AN: 607727123

Background. The rising prevalence of diabetes mellitus (DM) worldwide, especially in developing countries, and the persistence of tuberculosis (TB) as a major public health issue in these same regions, emphasize the importance of investigating this association. Here, we compared the clinical profile and disease outcomes of TB patients with or without coincident DM in a TB reference center in Brazil. Methods. We performed a retrospective analysis of a TB patient cohort (treatment naive) of 408 individuals recruited at a TB primary care center in Brazil between 2004 and 2010. Data on diagnosis of TB and DM were used to define the groups. The study groups were compared with regard to TB disease presentation at diagnosis as well as to clinical outcomes such as cure and mortality rates upon anti-tuberculosis therapy (ATT) initiation. A composite score utilizing clinical, radiological and microbiological parameters was used to compare TB severity between the groups. Results. DM patients were older than non-diabetic TB patients. In addition, diabetic individuals more frequently presented with cough, night sweats, hemoptysis and malaise than those without DM. The overall pattern of lung lesions assessed by chest radiographic examination was similar between the groups. Compared to non-diabetic patients, those with TB-diabetes exhibited positive acid-fast bacilli in sputum samples more frequently at diagnosis and at 30 days after ATT initiation. Notably, higher values of the TB severity score were significantly associated with TB-diabetes comorbidity after adjustment for confounding factors. Moreover, during ATT, diabetic patients required more frequent transfers to TB reference hospitals for complex clinical management. Nevertheless, overall mortality and cure rates were indistinguishable between the study groups. Conclusions. These findings reinforce the idea that diabetes negatively impacts pulmonary TB severity. Our study argues for the systematic screening for DM in TB reference centers in endemic areas. Copyright © 2016 Gil-Santana et al. This is an open access article distributed under the terms of the Creative Commons Attribution License, which permits unrestricted use, distribution, and reproduction in any medium, provided the original author and source are credited.

Institution

(Gil-Santana, Almeida, Andrade) Unidade de Medicina Investigativa, Laboratorio Integrado de Microbiologia e Imunorregulacao, Centro de Pesquisas Goncalo Moniz, Fundacao Oswaldo Cruz, Salvador, Bahia, Brazil (Gil-Santana, Almeida, Andrade) School of Medicine, Faculdades de Tecnologia e Ciencias, Salvador, Bahia, Brazil

(Gil-Santana, Almeida, Andrade) Multinational Organization Network Sponsoring Translational and Epidemiological Research (MONSTER), Fundacao Jose Silveira, Salvador, Brazil

(Oliveira, Hickson, Castro, Netto, Andrade) Research Center, Instituto Brasileiro para Investigacao da Tuberculose (IBIT), Fundacao Jose Silveira, Salvador, Bahia, Brazil

(Daltro, Netto) Hospital Universitario Pro. Edgard Santos, Universidade Federal da Bahia, Salvador, Bahia, Brazil

(Kornfeld) Department of Medicine, University of Massachusetts Medical School, Worcester, MA, United States

Publisher

Public Library of Science (E-mail: plos@plos.org)

Link to the Ovid Full Text or citation:

[Click here for full text options](http://ovidsp.ovid.com/ovidweb.cgi?T=JS&CSC=Y&NEWS=N&PAGE=fulltext&D=emed18a&AN=607727123)

Link to the External Link Resolver:

[SFX](http://whel-primo.hosted.exlibrisgroup.com/openurl/44WHELF_CAR/44WHELF_CAR_services_page?sid=OVID:embase&id=pmid:&id=doi:10.1371%2Fjournal.pone.0146876&issn=1932-6203&isbn=&volume=11&issue=1&spage=no+pagination&pages=no+pagination&date=2016&title=PLoS+ONE&atitle=Diabetes+is+associated+with+worse+clinical+presentation+in+tuberculosis+patients+from+Brazil%3A+A+retrospective+cohort+study&aulast=Gil-Santana&pid=<author>Gil-Santana+L.%3BAlmeida+J.L.%3BOliveira+C.A.M.%3BHickson+L.S.%3BDaltro+C.%3BCastro+S.%3BKornfeld+H.%3BNetto+E.M.%3BAndrade+B.B.<%2Fauthor><AN>607727123<%2FAN><DT>Journal%3A+Article<%2FDT>)

60.

Diabetes and tuberculosis co-epidemic: The Bali Declaration.

Kapur A., Harries A.D., Lonnroth K., Wilson P., Sulistyowati L.S.

The Lancet Diabetes and Endocrinology. 4 (1) (pp 8-10), 2016. Date of Publication: 01 Jan 2016.

AN: 607245913

Institution

(Kapur) World Diabetes Foundation, Gentofte, Denmark (Harries) International Union Against Tuberculosis and Lung Disease, Paris 75006, France

(Harries) London School of Hygiene and Tropical Medicine, Keppel Street, London, United Kingdom

(Lonnroth) World Health Organization, Geneva, Switzerland

(Lonnroth) Department of Public Health Sciences, Karolinska Institutet, Stockholm, Sweden

(Wilson) International Diabetes Federation, Brussels, Belgium

(Sulistyowati) Directorate of Non-Communicable Diseases Control, Directorate of Diseases Control and Environmental Health, Ministry of Health, Jakarta, Indonesia

Publisher

Lancet Publishing Group (E-mail: cususerv@lancet.com)

Link to the Ovid Full Text or citation:

[Click here for full text options](http://ovidsp.ovid.com/ovidweb.cgi?T=JS&CSC=Y&NEWS=N&PAGE=fulltext&D=emed18a&AN=607245913)

Link to the External Link Resolver:

[SFX](http://whel-primo.hosted.exlibrisgroup.com/openurl/44WHELF_CAR/44WHELF_CAR_services_page?sid=OVID:embase&id=pmid:&id=doi:10.1016%2FS2213-8587%252815%252900461-1&issn=2213-8587&isbn=&volume=4&issue=1&spage=8&pages=8-10&date=2016&title=The+Lancet+Diabetes+and+Endocrinology&atitle=Diabetes+and+tuberculosis+co-epidemic%3A+The+Bali+Declaration&aulast=Kapur&pid=<author>Kapur+A.%3BHarries+A.D.%3BLonnroth+K.%3BWilson+P.%3BSulistyowati+L.S.<%2Fauthor><AN>607245913<%2FAN><DT>Journal%3A+Note<%2FDT>)

61.

Survival benefit associated with early cyclosporine treatment for dermatomyositis-associated interstitial lung disease.

Go D.J., Park J.K., Kang E.H., Kwon H.M., Lee Y.J., Song Y.W., Lee E.B.

Rheumatology International. 36 (1) (pp 125-131), 2016. Date of Publication: 01 Jan 2016.

AN: 605475146

Interstitial lung disease (ILD) is the most common cause of death in dermatomyositis (DM). Cyclosporine A (CsA) has shown to be effective in DM-associated ILD (DM-ILD). This study aimed to define the optimal time of CsA administration. A total of 47 patients with DM-ILD, who were treated with CsA at Seoul National University Hospital between January 1998 and June 2013, were enrolled. ILD was diagnosed based on typical chest high-resolution computed tomography (HRCT) findings. Patients with early and delayed CsA treatment were compared in regard to the mortality and ILD progression on HRCT. The early (n = 16) and the delayed treatment group (n = 31) did not differ in regard to baseline clinical characteristics including HRCT scores and pulmonary function. Patients with clinically amyopathic DM (CADM) were more common in the early treatment group. The mortality rate was significantly lower in the early treatment group than in the delayed treatment group (p = 0.009). The survival benefit of early CsA treatment remained significant even after adjusting for age, degree of dyspnea, CADM status, and the year of CsA treatment (hazard ratio 0.057, 95 % confidence interval 0.007-0.472). CsA stabilized disease progression on HRCT in the early treatment group (p = 0.738). Delay in CsA treatment is associated with a worse survival in patients with DM-ILD. Early CsA treatment should be considered at DM-ILD diagnosis especially in patients at a higher risk of developing a rapidly progressive ILD. Copyright © 2015, Springer-Verlag Berlin Heidelberg.

Institution

(Go, Park, Kwon, Song, Lee) Division of Rheumatology, Department of Internal Medicine, Seoul National University College of Medicine, Seoul, South Korea (Go, Park, Song) Department of Molecular Medicine and Biopharmaceutical Sciences, Graduate School of Convergence Science and Technology, Medical Research Institute, Seoul National University, Seoul, South Korea

(Kang, Lee) Division of Rheumatology, Department of Internal Medicine, Seoul National University Bundang Hospital, Seongnam, South Korea

Publisher

Springer Verlag (E-mail: service@springer.de)

Link to the Ovid Full Text or citation:

[Click here for full text options](http://ovidsp.ovid.com/ovidweb.cgi?T=JS&CSC=Y&NEWS=N&PAGE=fulltext&D=emed18a&AN=605475146)

Link to the External Link Resolver:

[SFX](http://whel-primo.hosted.exlibrisgroup.com/openurl/44WHELF_CAR/44WHELF_CAR_services_page?sid=OVID:embase&id=pmid:&id=doi:10.1007%2Fs00296-015-3328-8&issn=0172-8172&isbn=&volume=36&issue=1&spage=125&pages=125-131&date=2016&title=Rheumatology+International&atitle=Survival+benefit+associated+with+early+cyclosporine+treatment+for+dermatomyositis-associated+interstitial+lung+disease&aulast=Go&pid=<author>Go+D.J.%3BPark+J.K.%3BKang+E.H.%3BKwon+H.M.%3BLee+Y.J.%3BSong+Y.W.%3BLee+E.B.<%2Fauthor><AN>605475146<%2FAN><DT>Journal%3A+Article<%2FDT>)
