# Supplementary material for: Effect of glycemic control and type of diabetes treatment on TB treatment outcomes among people with TB-diabetes: A systematic review (updated August 2024)
Source: PLoS One. 2025 Jul 18;20(7):e0328619. doi: 10.1371/journal.pone.0328619 (PMC12273911; doi:10.1371/journal.pone.0328619)
Supplement: S1 Appendix — (ZIP) [file pone.0328619.s004.zip › S1 appendix_old/EMBASE/update Sept 2016 to current 24_04_2017/81_without risk search reuslt no 16_2016 to current.doc]

Database: EMBASE <1947-Present>

Search Strategy:

--------------------------------------------------------------------------------

1	tuberculosis.mp. or exp tuberculosis/ (291433)

2	exp treatment outcome/ or treatment outcome*.mp. (1289437)

3	diabetes mellitus.mp. or exp diabetes mellitus/ (880018)

4	diabetes mellitus/ (599265)

5	DM.mp. (234038)

6	diabetes.mp. (891272)

7	mellitus.mp. (827628)

8	3 or 4 or 5 or 6 or 7 (1125394)

9	1 and 2 and 8 (1560)

10	cohort studies.mp. or cohort analysis/ (334934)

11	exp risk factor/ or cohort.mp. or exp risk/ (2843815)

12	10 or 11 (2843815)

13	9 and 10 (161)

14	9 and 12 (652)

15	limit 14 to human (642)

16	limit 13 to human (160)

17	trial*.mp. or exp controlled clinical trial/ or exp "clinical trial (topic)"/ or exp "randomized controlled trial (topic)"/ or exp "controlled clinical trial (topic)"/ (2044040)

18	random.ab. (253017)

19	randomized controlled trial.pt. (0)

20	controlled clinical trial.pt. (0)

21	randomized.ab. (540645)

22	placebo.ab. (252710)

23	clinical trials as topic.sh. (2)

24	trial.ti. (233523)

25	17 or 18 or 19 or 20 or 21 or 22 or 23 or 24 (2406494)

26	9 and 25 (443)

27	limit 26 to human (442)

28	limit 16 to yr="2016 - 2017" (19)

29	limit 15 to yr="2016 -Current" (61)

30	limit 16 to yr="2016 -Current" (19)

31	limit 27 to yr="2016 -Current" (50)

***************************

1.

Incidence of tuberculosis is high in chronic kidney disease patients in South East England and drug resistance common.

Ostermann M., Palchaudhuri P., Riding A., Begum P., Milburn H.J.

Renal Failure. 38 (2) (pp 256-261), 2016. Date of Publication: 07 Feb 2016.

AN: 607623274

The risk of tuberculosis (TB) is significantly increased in chronic kidney disease (CKD). Data on TB in CKD in the UK are sparse; most information stems from countries with high background prevalence. The aim of this study was to estimate the incidence of TB in CKD patients in South East London and to describe the epidemiology, treatment, and outcome. CKD patients with TB between 1994 and 2010 were identified retrospectively. Data were collected on type of renal replacement therapy, the method of TB diagnosis, disease site, treatment regimens, and risk factors. Forty patients were identified of whom 67.5% had CKD stages IV-V. Sixty-five percent were from non-UK born ethnic minorities. Median time from diagnosis of CKD to TB development was 12 months (range 0-192 months). Cumulative incidence of TB was 1267/100,000 [95% confidence interval (CI): 630-1904; 85 x background UK rate] in hemodialysis patients; 398/100,000 (95% CI: 80-1160; 26 x background UK rate) in peritoneal dialysis; and 522/100,000 (CI: 137-909; 35 x background UK rate) in transplant recipients. Sixty-three percent of patients had pulmonary TB and 25% of patients with culture-positive TB had resistant isolates. Fifty percent of patients were immunosuppressed due to drugs, diabetes, and/or retroviral disease. Treatment regimens were according to recent national guidance in 73% of cases. Seventy-six percent of patients experienced side effects. Greater awareness of risk factors, drug resistance, treatment regimens, and potential side effects is needed. Copyright © 2015 Taylor & Francis.

PMID

26726960 [<http://www.ncbi.nlm.nih.gov/pubmed/?term=26726960>]

Institution

(Ostermann) Department of Renal Medicine, Guys and St Thomas NHS Foundation Trust, London, United Kingdom (Ostermann, Milburn) Guys and St Thomas NHS Foundation Trust, Kings College London, Great Maze Pond, London SE1 9RT, United Kingdom

(Palchaudhuri, Riding, Begum, Milburn) Department of Respiratory Medicine, Guys and St Thomas NHS Foundation Trust, London, United Kingdom

Publisher

Taylor and Francis Ltd (E-mail: healthcare.enquiries@informa.com)

Link to the Ovid Full Text or citation:

[Click here for full text options](http://ovidsp.ovid.com/ovidweb.cgi?T=JS&CSC=Y&NEWS=N&PAGE=fulltext&D=emed18b&AN=607623274)

Link to the External Link Resolver:

[SFX](http://whel-primo.hosted.exlibrisgroup.com/openurl/44WHELF_CAR/44WHELF_CAR_services_page?sid=OVID:embase&id=pmid:26726960&id=doi:10.3109%2F0886022X.2015.1128290&issn=0886-022X&isbn=&volume=38&issue=2&spage=256&pages=256-261&date=2016&title=Renal+Failure&atitle=Incidence+of+tuberculosis+is+high+in+chronic+kidney+disease+patients+in+South+East+England+and+drug+resistance+common&aulast=Ostermann&pid=<author>Ostermann+M.%3BPalchaudhuri+P.%3BRiding+A.%3BBegum+P.%3BMilburn+H.J.<%2Fauthor><AN>607623274<%2FAN><DT>Journal%3A+Article<%2FDT>)

2.

Diabetes reduces the rate of sputum culture conversion in patients with newly diagnosed multidrug-resistant tuberculosis.

Salindri A.D., Kipiani M., Kempker R.R., Gandhi N.R., Darchia L., Tukvadze N., Blumberg H.M., Magee M.J.

Open Forum Infectious Diseases. 3 (3) (no pagination), 2016. Article Number: ofw126. Date of Publication: 2016.

AN: 614929507

Background. Diabetes is a risk factor for active tuberculosis (TB), but little is known about the relationship between diabetes and multidrug-resistant (MDR) TB. We aimed to assess risk factors for primary MDR TB, including diabetes, and determine whether diabetes reduced the rate of sputum culture conversion among patients with MDR TB. Methods. From 2011 to 2014, we conducted a cohort study at the National Center for Tuberculosis and Lung Diseases in Tbilisi, Georgia. Adult (>35 years) patients with primary TB were eligible. Multidrug-resistant TB was defined as resistance to at least rifampicin and isoniazid. Patients with capillary glycosylated hemoglobin (HbA1c) > 6.5% or previous diagnosis were defined to have diabetes. Polytomous regression was used to estimate the association of patient characteristics with drug resistance. Cox regression was used to compare rates of sputum culture conversion in patients with and without diabetes. Results. Among 318 patients with TB, 268 had drug-susceptibility test (DST) results. Among patients with DST results, 19.4% (52 of 268) had primary MDR TB and 13.4% (36 of 268) had diabetes. In multivariable analyses, diabetes (adjusted odds ratio [aOR], 2.51; 95% confidence interval [CI], 1.00-6.31) and lower socioeconomic status (aOR, 3.51; 95% CI, 1.56-8.20) were associated with primary MDR TB. Among patients with primaryMDR TB, 44 (84.6%) converted sputum cultures to negative. The rate of sputum culture conversion was lower among patients with diabetes (adjusted hazard ratio [aHR], 0.34; 95% CI,.13-.87) and among smokers (aHR, 0.16; 95% CI,.04-.61). Conclusions. We found diabetes was associated with an increased risk of primary MDR TB; both diabetes and smoking were associated with a longer time to sputum culture conversion. Copyright © The Author 2016.

Institution

(Salindri, Magee) Division of Epidemiology and Biostatistics, School of Public Health, Georgia State University, GA, United States (Kipiani, Darchia, Tukvadze) National Center for Tuberculosis and Lung Disease, Tbilisi, Georgia

(Kempker, Gandhi, Blumberg) Division of Infectious Diseases, Department of Medicine, Emory University School of Medicine, GA, United States

(Gandhi, Blumberg, Magee) Departments of Epidemiology and Global Health, Rollins School of Public Health, Emory University, Atlanta, GA, United States

Publisher

Oxford University Press (E-mail: info@idsociety.org)

Link to the Ovid Full Text or citation:

[Click here for full text options](http://ovidsp.ovid.com/ovidweb.cgi?T=JS&CSC=Y&NEWS=N&PAGE=fulltext&D=emed18b&AN=614929507)

Link to the External Link Resolver:

[SFX](http://whel-primo.hosted.exlibrisgroup.com/openurl/44WHELF_CAR/44WHELF_CAR_services_page?sid=OVID:embase&id=pmid:&id=doi:10.1093%2Fofid%2Fofw126&issn=2328-8957&isbn=&volume=3&issue=3&spage=no+pagination&pages=no+pagination&date=2016&title=Open+Forum+Infectious+Diseases&atitle=Diabetes+reduces+the+rate+of+sputum+culture+conversion+in+patients+with+newly+diagnosed+multidrug-resistant+tuberculosis&aulast=Salindri&pid=<author>Salindri+A.D.%3BKipiani+M.%3BKempker+R.R.%3BGandhi+N.R.%3BDarchia+L.%3BTukvadze+N.%3BBlumberg+H.M.%3BMagee+M.J.<%2Fauthor><AN>614929507<%2FAN><DT>Journal%3A+Article<%2FDT>)

3.

The effect of diabetes control status on treatment response in pulmonary tuberculosis: A prospective study.

Yoon Y.S., Jung J.-W., Jeon E.J., Seo H., Ryu Y.J., Yim J.-J., Kim Y.H., Lee B.-H., Park Y.B., Lee B.J., Kang H., Choi J.C.

Thorax. 72 (3) (pp 263-270), 2017. Date of Publication: 01 Mar 2017.

AN: 611939205

Background: Uncontrolled diabetes, unlike controlled diabetes, is associated with an impaired immune response. However, little is known about the impact of the status of diabetes control on clinical features and treatment outcomes in patients with pulmonary TB (PTB). We conducted this study to evaluate whether the status of diabetes control influences clinical manifestations and treatment responses in PTB. Methods: A multicentre prospective study was performed between September 2012 and September 2014. The patients were categorised into three groups according to the glycated haemoglobin (HbA1C) level: PTB without diabetes mellitus (non-DM), PTB with controlled diabetes (controlled-DM) and PTB with uncontrolled diabetes (uncontrolled-DM). The primary outcome was the sputum culture conversion rate after 2 months of intensive treatment. Results: Among 661 patients with PTB, 157 (23.8%) had diabetes and 108 (68.8%) had uncontrolled diabetes (HbA1C>7.0%). The uncontrolled-DM group exhibited more symptoms, positive sputum smears (p<0.001) and presence of cavities (p<0.001) than the non-DM group. Regarding treatment responses, patients with uncontrolled-DM were more likely to have a positive culture after 2 months (p=0.009) and either treatment failure (p=0.015) or death (p=0.027) compared with the non-DM group. In contrast, those with controlled-DM showed similar treatment responses to the non-DM group. In multivariable analysis, uncontrolled diabetes was an independent risk factor for a positive sputum culture after 2 months of treatment (adjusted OR, 2.11; p=0.042) and either treatment failure or death (adjusted OR, 4.11; p=0.022). Conclusions: Uncontrolled diabetes is an independent risk factor for poor treatment response in PTB. Copyright © BMJ Publishing Group Limited 2017.

Institution

(Yoon) Division of Pulmonology and Critical Care Medicine, Department of Internal Medicine, Dongguk University Ilsan Hospital, Dongguk University College of Medicine, Goyang, South Korea (Jung, Choi) Division of Pulmonary Medicine, Department of Internal Medicine, Chung-Ang University, School of Medicine, HeukSeok Ro 102, Dong Jak Gu, Seoul 06973, South Korea

(Jeon) Department of Internal Medicine, Hallym Hospital, Incheon, South Korea

(Seo) Department of Tuberculosis, Seobuk Hospital, Seoul Metropolitan Government, Seoul, South Korea

(Ryu) Division of Pulmonary and Critical Care Medicine, Department of Internal Medicine, Ewha Womans University, School of Medicine, Seoul, South Korea

(Yim) Division of Pulmonary and Critical Care Medicine, Department of Internal Medicine, Seoul National University College of Medicine, Seoul, South Korea

(Kim) Division of Pulmonary and Critical Care Medicine, Department of Internal Medicine, Kyung Hee University Hospital at Gangdong, School of Medicine, Kyung Hee University, Seoul, South Korea

(Lee) Department of Pulmonology and Allergy, Eulji Hospital, College of Medicine, Eulji University, Seoul, South Korea

(Park) Department of Internal Medicine, Hallym University Medical Center, Kangdong Sacred Heart Hospital, Seoul, South Korea

(Lee) Division of Pulmonary and Critical Care Medicine, Department of Internal Medicine, Veterans Health Service Medical Center, Seoul, South Korea

(Kang) Department of Anesthesiology and Pain Medicine, Chung-Ang University, School of Medicine, Seoul, South Korea

Publisher

BMJ Publishing Group (E-mail: subscriptions@bmjgroup.com)

Link to the Ovid Full Text or citation:

[Click here for full text options](http://ovidsp.ovid.com/ovidweb.cgi?T=JS&CSC=Y&NEWS=N&PAGE=fulltext&D=emed18b&AN=611939205)

Link to the External Link Resolver:

[SFX](http://whel-primo.hosted.exlibrisgroup.com/openurl/44WHELF_CAR/44WHELF_CAR_services_page?sid=OVID:embase&id=pmid:&id=doi:10.1136%2Fthoraxjnl-2015-207686&issn=0040-6376&isbn=&volume=72&issue=3&spage=263&pages=263-270&date=2017&title=Thorax&atitle=The+effect+of+diabetes+control+status+on+treatment+response+in+pulmonary+tuberculosis%3A+A+prospective+study&aulast=Yoon&pid=<author>Yoon+Y.S.%3BJung+J.-W.%3BJeon+E.J.%3BSeo+H.%3BRyu+Y.J.%3BYim+J.-J.%3BKim+Y.H.%3BLee+B.-H.%3BPark+Y.B.%3BLee+B.J.%3BKang+H.%3BChoi+J.C.<%2Fauthor><AN>611939205<%2FAN><DT>Journal%3A+Article<%2FDT>)

4.

The impact of patient demographics and comorbidities upon burns admitted to Tygerberg Hospital Burns Unit, Western Cape, South Africa.

Cloake T., Haigh T., Cheshire J., Walker D.

Burns. 43 (2) (pp 411-416), 2017. Date of Publication: 01 Mar 2017.

AN: 614262868

In South Africa, burns are a major public health problem responsible for significant morbidity and long-term physical disability. This is, in part, due to a significant proportion of the urban population living in poorly constructed, combustible accommodation. The presence of co-morbid diseases such as diabetes and malignancy in patients with burns has been associated with a poorer outcome. The impact of other diseases such as HIV has yet to be defined. A retrospective data collection study analysed the 221 patients admitted to Tygerberg Hospital Burns Unit in 2011 and the first six months of 2013. Using hospital records, patient demographic data was collected alongside burn agent, ICU admission, complications, and patient outcome in terms of length of stay and mortality. The most common burn agent was hot liquid (45.7%). A significant proportion of patients were subject to intentional attacks (34.3%). Shack fires and flame accounted cumulatively for 85% of total inhalational burns, the highest rates of admission to ICU (85.5%), the highest rate of complications, as well as 92.3% of all total fatalities. HIV+ patients had a higher mortality (13.3% vs 5%, p = 0.22) and a higher complication rate (46.7% vs 30%, p = 0.21). There was no difference in length of stay between the HIV+ and HIV- cohort (12 days vs. 15.5 days, p = 0.916). Burns are a significant yet preventable cause of mortality and morbidity. The rising number of shack fires, responsible for extensive burns and resultant mortality is concerning and indicates urgent attention and action. HIV complicates the recovery from burn and is responsible for an increased rate of in hospital mortality. Copyright © 2016 Elsevier Ltd and ISBI

PMID

27658998 [<http://www.ncbi.nlm.nih.gov/pubmed/?term=27658998>]

Institution

(Cloake, Haigh, Cheshire, Walker) University of Birmingham Medical School, University of Birmingham, United Kingdom

Publisher

Elsevier Ltd

Link to the Ovid Full Text or citation:

[Click here for full text options](http://ovidsp.ovid.com/ovidweb.cgi?T=JS&CSC=Y&NEWS=N&PAGE=fulltext&D=emed18b&AN=614262868)

Link to the External Link Resolver:

[SFX](http://whel-primo.hosted.exlibrisgroup.com/openurl/44WHELF_CAR/44WHELF_CAR_services_page?sid=OVID:embase&id=pmid:27658998&id=doi:10.1016%2Fj.burns.2016.08.031&issn=0305-4179&isbn=&volume=43&issue=2&spage=411&pages=411-416&date=2017&title=Burns&atitle=The+impact+of+patient+demographics+and+comorbidities+upon+burns+admitted+to+Tygerberg+Hospital+Burns+Unit%2C+Western+Cape%2C+South+Africa&aulast=Cloake&pid=<author>Cloake+T.%3BHaigh+T.%3BCheshire+J.%3BWalker+D.<%2Fauthor><AN>614262868<%2FAN><DT>Journal%3A+Article<%2FDT>)

5.

Impact of type of calcineurin inhibitor on post-transplant tuberculosis: Single-center study from India.

Agarwal S.K., Bhowmik D., Mahajan S., Bagchi S.

Transplant Infectious Disease. 19 (1) (no pagination), 2017. Article Number: e12626. Date of Publication: 01 Feb 2017.

AN: 613850784

Introduction: Tuberculosis (TB) is an important cause of morbidity and mortality in renal transplant recipients. Immunosuppressive drugs are one of the most important risk factor for post-transplant tuberculosis (PTTB). A paucity of data exists about the impact of the type of calcineurin inhibitor on PTTB. Methods: In this retrospective study, all adult patients on calcineurin inhibitor-based immunosuppression were included. Patients receiving TB chemoprophylaxis were excluded. Diabetes, duration of dialysis, hepatitis B and C, past treated TB, induction therapy, type of antimetabolite, acute rejection, new onset of diabetes after renal transplantation (RT) (NODAT) and cytomegalovirus (CMV) were analyzed in tacrolimus (Tac) and cyclosporine (CsA) groups. Primary outcome was incidence of TB and secondary outcomes were timeline of development of TB after RT and pattern of TB in the two groups. Results: Of the 1664 patients included, 582 patients received CsA-based immunosuppression while 1082 received Tac-based immunosuppression. Duration of dialysis, positive tuberculin skin test, use of induction, mycophenolate mofetil use, CMV infection, and NODAT were significantly more, and hepatitis B infection, past treated TB, and acute rejection episodes were significantly less in the Tac group. At the end of follow-up, incidence of TB in the Tac group was significantly less than in the CsA group (6.1% vs 19.9%, P<.001). Mean time for development of TB after RT was similar in both the groups and nodal and disseminated TB were more common in the Tac group. Conclusion: In conclusion, our study shows that use of Tac as compared to CsA significantly decreases incidence of PTTB. Time of infection since transplant was similar in both the groups. However, nodal and disseminated TB were more common in the Tac group. Copyright © 2016 John Wiley & Sons A/S. Published by John Wiley & Sons Ltd

Institution

(Agarwal, Bhowmik, Mahajan, Bagchi) Department of Nephrology, All India Institute of Medical Sciences, New Delhi, India

Publisher

Blackwell Publishing Inc. (E-mail: subscrip@blackwellpub.com)

Link to the Ovid Full Text or citation:

[Click here for full text options](http://ovidsp.ovid.com/ovidweb.cgi?T=JS&CSC=Y&NEWS=N&PAGE=fulltext&D=emed18b&AN=613850784)

Link to the External Link Resolver:

[SFX](http://whel-primo.hosted.exlibrisgroup.com/openurl/44WHELF_CAR/44WHELF_CAR_services_page?sid=OVID:embase&id=pmid:&id=doi:10.1111%2Ftid.12626&issn=1398-2273&isbn=&volume=19&issue=1&spage=no+pagination&pages=no+pagination&date=2017&title=Transplant+Infectious+Disease&atitle=Impact+of+type+of+calcineurin+inhibitor+on+post-transplant+tuberculosis%3A+Single-center+study+from+India&aulast=Agarwal&pid=<author>Agarwal+S.K.%3BBhowmik+D.%3BMahajan+S.%3BBagchi+S.<%2Fauthor><AN>613850784<%2FAN><DT>Journal%3A+Article<%2FDT>)

6.

Early interventions for diabetes related tuberculosis associate with hastened sputum microbiological clearance in Virginia, USA.

Alkabab Y., Keller S., Dodge D., Houpt E., Staley D., Heysell S.

BMC Infectious Diseases. 17 (1) (no pagination), 2017. Article Number: 125. Date of Publication: 06 Feb 2017.

AN: 614295261

Background: Diabetes complicates tuberculosis (TB) treatment including a prolonged time of sputum culture conversion to negative growth. Since 2013 in Virginia, interventions early in the treatment course have used therapeutic drug monitoring and dose correction for isoniazid and rifampin after 2 weeks of TB treatment in patients with diabetes along with nurse manager initiated diabetes education and linkage to care. Methods: A retrospective cohort study of the state TB registry was performed for patients initiating drug-susceptible pulmonary TB treatment that were matched for age, gender, chest imaging and sputum smear status to compare time to sputum culture conversion and other clinical outcomes in the pre-and post-intervention groups. Results: Three hundred sixty-three patients had documented time to sputum culture conversion in the pre-and post-intervention periods, including 56 (15%) with diabetes. Seventy-four (57%) of all patients with diabetes were >60 years of age at treatment initiation. Twenty-six patients with diabetes were matched in each group. Mean time to sputum culture conversion in the post-intervention group was 42 +/- 22 days compared to the pre-intervention group of 62 +/- 31 days (p = 0.01). In the post-intervention group 21 (80%) of patients with diabetes had culture conversion by 2 months compared to 13 (50%) in the pre-intervention group (p = 0.04). Conclusions: Early interventions for diabetes related TB in the programmatic setting may hasten sputum culture conversion. Copyright © 2017 The Author(s).

Institution

(Alkabab, Houpt, Heysell) University of Virginia School of Medicine, Division of Infectious Diseases and International Health, P.O. Box 801340, Charlottesville, VA 22908, United States (Keller) TB Control, Virginia Department of Health, Division of Disease Prevention, 109 Governor Street, Richmond, VA 23219, United States

(Dodge, Staley) Virginia Department of Health, TB Control and Newcomer Health, 109 Governor Street, Room 326, PO Box 2448, Richmond, VA 23218, United States

Publisher

BioMed Central Ltd. (E-mail: info@biomedcentral.com)

Link to the Ovid Full Text or citation:

[Click here for full text options](http://ovidsp.ovid.com/ovidweb.cgi?T=JS&CSC=Y&NEWS=N&PAGE=fulltext&D=emed18b&AN=614295261)

Link to the External Link Resolver:

[SFX](http://whel-primo.hosted.exlibrisgroup.com/openurl/44WHELF_CAR/44WHELF_CAR_services_page?sid=OVID:embase&id=pmid:&id=doi:10.1186%2Fs12879-017-2226-y&issn=1471-2334&isbn=&volume=17&issue=1&spage=no+pagination&pages=no+pagination&date=2017&title=BMC+Infectious+Diseases&atitle=Early+interventions+for+diabetes+related+tuberculosis+associate+with+hastened+sputum+microbiological+clearance+in+Virginia%2C+USA&aulast=Alkabab&pid=<author>Alkabab+Y.%3BKeller+S.%3BDodge+D.%3BHoupt+E.%3BStaley+D.%3BHeysell+S.<%2Fauthor><AN>614295261<%2FAN><DT>Journal%3A+Article<%2FDT>)

7.

Treatment outcomes of fixed-dose combination versus separate tablet regimens in pulmonary tuberculosis patients with or without diabetes in Qatar.

Al-Shaer M.H., Mansour H., Elewa H., Salameh P., Iqbal F.

BMC Infectious Diseases. 17 (1) (no pagination), 2017. Article Number: 118. Date of Publication: 02 Feb 2017.

AN: 614259878

Background: Tuberculosis is considered the second most common cause of death due to infectious agent. The currently preferred regimen for treatment of pulmonary tuberculosis (PTB) is isoniazid, rifampin, pyrazinamide, and ethambutol, which has been used either as separate tablets (ST) or as fixed-dose combination (FDC). To date, no studies have compared both regimens in Qatar. We aim to evaluate the safety and effectiveness of FDC and ST regimen for treating PTB, in addition to comparing safety and efficacy of FDC and ST regimens in patients with diabetes treated for TB. Methods: A retrospective observational study was conducted in two general hospitals in Qatar. Patients diagnosed with PTB received anti-tuberculosis medications (either as FDC or ST) administered by the nurse. Sputum smears were tested weekly. We assessed the time to negative sputum smear and incidence of adverse events among FDC and ST groups. Results: The study included 148 patients. FDC was used in 90 patients (61%). Effectiveness was not different between FDC and ST regimens as shown by mean time to sputum conversion (29.9+/-18.3 vs. 35.6+/-23days, p=0.12). Similarly, there was no difference in the incidence of adverse events, except for visual one that was higher in ST group. Among the 33 diabetic patients, 19 received the FDC and had faster sputum conversion compared to those who received ST (31+/-12 vs. 49.4+/-30.9days, p=0.05). Overall, diabetic patients needed longer time for sputum conversion and had more hepatotoxic and gastric adverse events compared to non-diabetics. Conclusion: ST group had higher visual side effects compared to FDC. FDC may be more effective in diabetic patients; however, further studies are required to confirm such finding. Copyright © 2017 The Author(s).

Institution

(Al-Shaer) Al Wakra Hospital - Hamad Medical Corporation, Department of Pharmacy, Doha, Qatar (Al-Shaer) University of Florida, Department of Pharmacotherapy and Translational Research, Infectious Disease Pharmacokinetics Laboratory, College of Pharmacy and Emerging Pathogens Institute, Gainesville, FL 32610, United States

(Mansour, Salameh) Lebanese American University, School of Pharmacy, P.O Box 36-S 23, Byblos, Lebanon

(Elewa) Qatar University, College of Pharmacy, Doha, Qatar

(Salameh) Lebanese University, Faculty of Medical Sciences, Beirut, Lebanon

(Iqbal) Rumailah Hospital - Hamad Medical Corporation, Department of Pharmacy, Doha, Qatar

Publisher

BioMed Central Ltd. (E-mail: info@biomedcentral.com)

Link to the Ovid Full Text or citation:

[Click here for full text options](http://ovidsp.ovid.com/ovidweb.cgi?T=JS&CSC=Y&NEWS=N&PAGE=fulltext&D=emed18b&AN=614259878)

Link to the External Link Resolver:

[SFX](http://whel-primo.hosted.exlibrisgroup.com/openurl/44WHELF_CAR/44WHELF_CAR_services_page?sid=OVID:embase&id=pmid:&id=doi:10.1186%2Fs12879-017-2231-1&issn=1471-2334&isbn=&volume=17&issue=1&spage=no+pagination&pages=no+pagination&date=2017&title=BMC+Infectious+Diseases&atitle=Treatment+outcomes+of+fixed-dose+combination+versus+separate+tablet+regimens+in+pulmonary+tuberculosis+patients+with+or+without+diabetes+in+Qatar&aulast=Al-Shaer&pid=<author>Al-Shaer+M.H.%3BMansour+H.%3BElewa+H.%3BSalameh+P.%3BIqbal+F.<%2Fauthor><AN>614259878<%2FAN><DT>Journal%3A+Article<%2FDT>)

8.

The effect size of type 2 diabetes mellitus on tuberculosis drug resistance and adverse treatment outcomes.

Perez-Navarro L.M., Restrepo B.I., Fuentes-Dominguez F.J., Duggirala R., Morales-Romero J., Lopez-Alvarenga J.C., Comas I., Zenteno-Cuevas R.

Tuberculosis. 103 (pp 83-91), 2017. Date of Publication: 01 Mar 2017.

AN: 614301639

Objective To evaluate the effect size of type 2 diabetes mellitus (T2DM) on tuberculosis (TB) treatment outcomes and multi drug resistance (MDR). Methods A cohort with 507 individuals with diagnosed TB included 183 with coexistence of T2DM and TB (TB-T2DM). Participants were identified at the time of TB diagnosis and followed during the course of TB treatment. Then we computed relative risks and adjustments by Cox proportional hazards for outcome variables (drug resistance, death, relapse, treatment failure), and the size of their effect as Cohen's-d. Results Patients with TB-T2DM were more likely to remain positive for acid-fast bacilli after two months of anti-TB treatment RR = [2.01 (95% CI: 1.3, 3.1)], to have drug resistant (DR) [OR 3.5 (95% CI: 1.8, 6.7)] and multi-drug resistant (MDR) TB [OR 3.5 (95% CI: 1.8, 7.1)]. The Cohen's-d for DR or MDR in T2DM was 0.69 when compared with non-DM subjects. The T2DM patients had higher odds of resistance to isoniazid (OR 3.9, 95% CI: 2.01, 7.9), rifampicin (OR 3.4, 95% CI: 1.6, 7.2) and pyrazinamide (OR 9.4, 95% CI: 2.8, 25.6), and their effect sizes were >0.67. Patients with TB-T2DM (versus no DM) were more likely to present with MDR TB (HR 3.1; 95% CI: 1.7, 5.8; p < 0.001), treatment failure (HR 2.04; 95% CI: 1.07, 3.8; p = 0.02) and relapse (HR 1.86; 95% CI: 1.09, 3.1; p = 0.02), with effect size >0.34. Conclusion T2DM showed a substantial contribution to the presence of DR or MDR-TB and to adverse clinical outcomes during and after TB treatment. Our findings support the importance for routine screening of T2DM among newly-diagnosed TB patients in order to stratify them for immediate DR assessment, and highlight the need for clinical trials to evaluate variations to the standard TB treatment in TB-T2DM to prevent adverse treatment outcomes. Copyright © 2017 Elsevier Ltd

Author NameID

Perez-Navarro, Lucia Monserrat; ORCID: <http://orcid.org/0000-0002-9281-9202> Restrepo, Blanca I.; ORCID: <http://orcid.org/0000-0002-3743-9098>

Zenteno-Cuevas, Roberto; ORCID: <http://orcid.org/0000-0001-9597-127X>

Institution

(Perez-Navarro) Nephrology Service, Research Division, Hospital General de Mexico "Dr. Eduardo Liceaga", Mexico (Perez-Navarro, Morales-Romero, Zenteno-Cuevas) Public Health Institute, University of Veracruz, Veracruz, Mexico

(Restrepo) University of Texas Health Science Center Houston, Brownsville Campus, Brownsville, TX, United States

(Fuentes-Dominguez) Mycobacteriosis State Program, Veracruz Health Services, Mexico

(Duggirala, Lopez-Alvarenga) South Texas Diabetes and Obesity Institute, University of Texas Rio Grande Valley, Edinburg, TX, United States

(Comas) Unidad de Genomica de la Tuberculosis, Instituto de Biomedicina de Valencia, Consejo Superior de Investigaciones Cientificas, Valencia, Spain

(Zenteno-Cuevas) Red multidisciplinaria de Investigacion en tuberculosis, Mexico

Publisher

Churchill Livingstone

Link to the Ovid Full Text or citation:

[Click here for full text options](http://ovidsp.ovid.com/ovidweb.cgi?T=JS&CSC=Y&NEWS=N&PAGE=fulltext&D=emed18b&AN=614301639)

Link to the External Link Resolver:

[SFX](http://whel-primo.hosted.exlibrisgroup.com/openurl/44WHELF_CAR/44WHELF_CAR_services_page?sid=OVID:embase&id=pmid:&id=doi:10.1016%2Fj.tube.2017.01.006&issn=1472-9792&isbn=&volume=103&issue=&spage=83&pages=83-91&date=2017&title=Tuberculosis&atitle=The+effect+size+of+type+2+diabetes+mellitus+on+tuberculosis+drug+resistance+and+adverse+treatment+outcomes&aulast=Perez-Navarro&pid=<author>Perez-Navarro+L.M.%3BRestrepo+B.I.%3BFuentes-Dominguez+F.J.%3BDuggirala+R.%3BMorales-Romero+J.%3BLopez-Alvarenga+J.C.%3BComas+I.%3BZenteno-Cuevas+R.<%2Fauthor><AN>614301639<%2FAN><DT>Journal%3A+Article<%2FDT>)

9.

Cohort profile: The diabetes-tuberculosis treatment outcome (DITTO) study in Pakistan.

Mukhtar F., Butt Z.A.

BMJ Open. 6 (12) (no pagination), 2016. Article Number: e012970. Date of Publication: 01 Dec 2016.

AN: 613514830

Purpose: Pakistan is faced with an increasing prevalence of diabetes in addition to its existing high burden of tuberculosis (TB). Diabetes has a detrimental effect on treatment outcomes of patients with TB, which may hinder achieving the goals of the End-TB strategy by 2030. We conducted a prospective cohort study to determine difference between treatment outcomes among patients with diabetes and new pulmonary tuberculosis (PTB) and patients without diabetes and new PTB. This would help generate contextual and valid scientific evidence from a developing country like Pakistan with its unique interplay of sociocultural, economic and health system factors to inform policy and practice. Participants: This paper outlines the baseline characteristics of 614 new cases of PTB, aged 15 years and older, which were followed up prospectively at 2nd, 5th and 6th months while on antituberculosis treatment and at 6 months after treatment completion. Findings to date: We ascertained patients' diabetic status by conducting random and fasting blood glucose tests and their glycaemic control by determining glycosylated haemoglobin. Treatment outcomes were established using standardised definitions provided by WHO. The assessment of 614 respondents' diabetic status revealed that 113 (18%) were diabetic and 501 (82%) were nondiabetic. A greater proportion of patients with diabetes and PTB were illiterate (n=74/113, 65.5%) as compared to patients without diabetes and PTB (n=249/501, 50%) ( p=0.035). More patients with diabetes and PTB gave a history of heart disease (n=14/113, 12%) and hypertension (n=26/113, 23%) as compared to patients without diabetes and PTB (n=2/501, 0.4% (heart disease) and n=13 501, 3% (hypertension)) ( p<0.001). Unfavourable treatment outcome was more likely among patients with diabetes and PTB (n=23/93, 25%) as opposed to patients without diabetes and PTB (n=46/410, 11%) (p=0.001). Future plans: We are negotiating with the government regarding funding for a further 2-year follow-up of the cohort to ascertain death and relapse in the post-treatment period and also differentiate between re-infection and recurrence among these patients with respect to their diabetic status.

Institution

(Mukhtar) Health Services Academy, Islamabad, Pakistan (Mukhtar) Department of Community Medicine, Lahore Medical and Dental College, Lahore, Pakistan

(Butt) Department of Epidemiology and Biostatistics, Health Services Academy, Islamabad, Pakistan

(Butt) School of Population and Public Health, University of British Columbia, Vancouver, BC, Canada

Publisher

BMJ Publishing Group (E-mail: subscriptions@bmjgroup.com)

Link to the Ovid Full Text or citation:

[Click here for full text options](http://ovidsp.ovid.com/ovidweb.cgi?T=JS&CSC=Y&NEWS=N&PAGE=fulltext&D=emed18b&AN=613514830)

Link to the External Link Resolver:

[SFX](http://whel-primo.hosted.exlibrisgroup.com/openurl/44WHELF_CAR/44WHELF_CAR_services_page?sid=OVID:embase&id=pmid:&id=doi:10.1136%2Fbmjopen-2016-012970&issn=2044-6055&isbn=&volume=6&issue=12&spage=no+pagination&pages=no+pagination&date=2016&title=BMJ+Open&atitle=Cohort+profile%3A+The+diabetes-tuberculosis+treatment+outcome+(DITTO)+study+in+Pakistan&aulast=Mukhtar&pid=<author>Mukhtar+F.%3BButt+Z.A.<%2Fauthor><AN>613514830<%2FAN><DT>Journal%3A+Article<%2FDT>)

10.

Educational Outreach with an Integrated Clinical Tool for Nurse-Led Non-communicable Chronic Disease Management in Primary Care in South Africa: A Pragmatic Cluster Randomised Controlled Trial.

Fairall L.R., Folb N., Timmerman V., Lombard C., Steyn K., Bachmann M.O., Bateman E.D., Lund C., Cornick R., Faris G., Gaziano T., Georgeu-Pepper D., Zwarenstein M., Levitt N.S.

PLoS Medicine. 13 (11) (no pagination), 2016. Article Number: e1002178. Date of Publication: November 2016.

AN: 613530964

Background: In many low-income countries, care for patients with non-communicable diseases (NCDs) and mental health conditions is provided by nurses. The benefits of nurse substitution and supplementation in NCD care in high-income settings are well recognised, but evidence from low- and middle-income countries is limited. Primary Care 101 (PC101) is a programme designed to support and expand nurses' role in NCD care, comprising educational outreach to nurses and a clinical management tool with enhanced prescribing provisions. We evaluated the effect of the programme on primary care nurses' capacity to manage NCDs. Methods and Findings: In a cluster randomised controlled trial design, 38 public sector primary care clinics in the Western Cape Province, South Africa, were randomised. Nurses in the intervention clinics were trained to use the PC101 management tool during educational outreach sessions delivered by health department trainers and were authorised to prescribe an expanded range of drugs for several NCDs. Control clinics continued use of the Practical Approach to Lung Health and HIV/AIDS in South Africa (PALSA PLUS) management tool and usual training. Patients attending these clinics with one or more of hypertension (3,227), diabetes (1,842), chronic respiratory disease (1,157) or who screened positive for depression (2,466), totalling 4,393 patients, were enrolled between 28 March 2011 and 10 November 2011. Primary outcomes were treatment intensification in the hypertension, diabetes, and chronic respiratory disease cohorts, defined as the proportion of patients in whom treatment was escalated during follow-up over 14 mo, and case detection in the depression cohort. Primary outcome data were analysed for 2,110 (97%) intervention and 2,170 (97%) control group patients. Treatment intensification rates in intervention clinics were not superior to those in the control clinics (hypertension: 44% in the intervention group versus 40% in the control group, risk ratio [RR] 1.08 [95% CI 0.94 to 1.24; p = 0.252]; diabetes: 57% versus 50%, RR 1.10 [0.97 to 1.24; p = 0.126]; chronic respiratory disease: 14% versus 12%, RR 1.08 [0.75 to 1.55; p = 0.674]), nor was case detection of depression (18% versus 24%, RR 0.76 [0.53 to 1.10; p = 0.142]). No adverse effects of the nurses' expanded scope of practice were observed. Limitations of the study include dependence on self-reported diagnoses for inclusion in the patient cohorts, limited data on uptake of PC101 by users, reliance on process outcomes, and insufficient resources to measure important health outcomes, such as HbA1c, at follow-up. Conclusions: Educational outreach to primary care nurses to train them in the use of a management tool involving an expanded role in managing NCDs was feasible and safe but was not associated with treatment intensification or improved case detection for index diseases. This notwithstanding, the intervention, with adjustments to improve its effectiveness, has been adopted for implementation in primary care clinics throughout South Africa. Trial Registration: The trial is registered with Current Controlled Trials (ISRCTN20283604) Copyright © 2016 Fairall et al.

Author NameID

Timmerman, Venessa; ORCID: <http://orcid.org/0000-0002-6172-9386> Cornick, Ruth; ORCID: <http://orcid.org/0000-0002-7391-3383>

Folb, Naomi; ORCID: <http://orcid.org/0000-0002-2667-0956>

Institution

(Fairall, Folb, Timmerman, Bateman, Cornick, Faris, Georgeu-Pepper) Knowledge Translation Unit, University of Cape Town Lung Institute, Cape Town, South Africa (Fairall, Folb, Steyn, Bateman, Levitt) Department of Medicine, University of Cape Town, Cape Town, South Africa

(Fairall, Folb, Steyn, Gaziano, Levitt) Chronic Disease Initiative for Africa, Department of Medicine, University of Cape Town, Cape Town, South Africa

(Lombard) Biostatistics Unit, Medical Research Council, Cape Town, South Africa

(Bachmann) Norwich Medical School, University of East Anglia, Norwich, United Kingdom

(Lund) Alan J Flisher Centre for Public Mental Health, Department of Psychiatry and Mental Health, University of Cape Town, Cape Town, South Africa

(Gaziano) Division of Cardiovascular Medicine, Brigham & Women's Hospital, Boston, MA, United States

(Zwarenstein) Centre for Studies in Family Medicine, Department of Family Medicine, Schulich School of Medicine and Dentistry, Western University, London, ON, Canada

Publisher

Public Library of Science (E-mail: plos@plos.org)

Link to the Ovid Full Text or citation:

[Click here for full text options](http://ovidsp.ovid.com/ovidweb.cgi?T=JS&CSC=Y&NEWS=N&PAGE=fulltext&D=emed18b&AN=613530964)

Link to the External Link Resolver:

[SFX](http://whel-primo.hosted.exlibrisgroup.com/openurl/44WHELF_CAR/44WHELF_CAR_services_page?sid=OVID:embase&id=pmid:&id=doi:10.1371%2Fjournal.pmed.1002178&issn=1549-1277&isbn=&volume=13&issue=11&spage=no+pagination&pages=no+pagination&date=2016&title=PLoS+Medicine&atitle=Educational+Outreach+with+an+Integrated+Clinical+Tool+for+Nurse-Led+Non-communicable+Chronic+Disease+Management+in+Primary+Care+in+South+Africa%3A+A+Pragmatic+Cluster+Randomised+Controlled+Trial&aulast=Fairall&pid=<author>Fairall+L.R.%3BFolb+N.%3BTimmerman+V.%3BLombard+C.%3BSteyn+K.%3BBachmann+M.O.%3BBateman+E.D.%3BLund+C.%3BCornick+R.%3BFaris+G.%3BGaziano+T.%3BGeorgeu-Pepper+D.%3BZwarenstein+M.%3BLevitt+N.S.<%2Fauthor><AN>613530964<%2FAN><DT>Journal%3A+Article<%2FDT>)

11.

A prediction rule to stratify mortality risk of patients with pulmonary tuberculosis.

Bastos H.N., Osorio N.S., Castro A.G., Ramos A., Carvalho T., Meira L., Araujo D., Almeida L., Boaventura R., Fragata P., Chaves C., Costa P., Portela M., Ferreira I., Magalhaes S.P., Rodrigues F., Sarmento-Castro R., Duarte R., Guimaraes J.T., Saraiva M.

PLoS ONE. 11 (9) (no pagination), 2016. Article Number: e0162797. Date of Publication: September 2016.

AN: 612857061

Tuberculosis imposes high human and economic tolls, including in Europe. This study was conducted to develop a severity assessment tool for stratifying mortality risk in pulmonary tuberculosis (PTB) patients. A derivation cohort of 681 PTB cases was retrospectively reviewed to generate a model based on multiple logistic regression analysis of prognostic variables with 6-month mortality as the outcome measure. A clinical scoring system was developed and tested against a validation cohort of 103 patients. Five risk features were selected for the prediction model: hypoxemic respiratory failure (OR 4.7, 95% CI 2.8-7.9), age <50 years (OR 2.9, 95% CI 1.7-4.8), bilateral lung involvement (OR 2.5, 95% CI 1.4-4.4), <1 significant comorbidity-HIV infection, diabetes mellitus, liver failure or cirrhosis, congestive heart failure and chronic respiratory disease-(OR 2.3, 95% CI 1.3-3.8), and hemoglobin <12 g/dL (OR 1.8, 95% CI 1.1-3.1). A tuberculosis risk assessment tool (TReAT) was developed, stratifying patients with low (score >2), moderate (score 3-5) and high (score >6) mortality risk. The mortality associated with each group was 2.9%, 22.9% and 53.9%, respectively. The model performed equally well in the validation cohort. We provide a new, easy-To-use clinical scoring system to identify PTB patients with high-mortality risk in settings with good healthcare access, helping clinicians to decide which patients are in need of closer medical care during treatment. Copyright © 2016 Bastos et al. This is an open access article distributed under the terms of the Creative Commons Attribution License, which permits unrestricted use, distribution, and reproduction in any medium, provided the original author and source are credited.

Institution

(Bastos, Meira, Araujo, Almeida, Boaventura) Department of Pneumology, Centro Hospitalar Sao Joao, Porto, Portugal (Bastos, Osorio, Castro, Chaves, Costa, Rodrigues, Sarmento-Castro, Saraiva) Life and Health Sciences Research Institute (ICVS), School of Health Sciences, University of Minho, Braga, Portugal

(Bastos, Osorio, Castro, Costa, Rodrigues, Sarmento-Castro, Saraiva) ICVS/3B's-PT Government Associ. Laboratory, Braga/Guimaraes, Portugal

(Ramos, Carvalho, Guimaraes) Department of Clinical Pathology, Centro Hospitalar Sao Joao, Porto, Portugal

(Ramos, Carvalho, Duarte, Guimaraes) Institute of Public Health, University of Porto, Porto, Portugal

(Fragata) Department of Internal Medicine, Hospital Do Santo Espirito da Ilha Terceira, Angra do Heroismo, Portugal

(Costa) Faculty of Psychology and Educational Sciences, University of Porto, Porto, Portugal

(Portela) School of Economics and Management, University of Minho, Braga, Portugal

(Ferreira, Magalhaes) Department of Radiology, Centro Hospitalar Do Porto, Porto, Portugal

(Sarmento-Castro) Department of Infectious Diseases, Centro Hospitalar Do Porto, Hospital Joaquim Urbano Unit, Porto, Portugal

(Duarte) Department of Pneumology, Centro Hospitalar de Vila Nova de Gaia/Espinho, Vila Nova de Gaia, Portugal

(Duarte) Referral Centre for Multidrug-resistant Tuberculosis in the Northern Region of Portugal, Chest Disease Centre of Vila Nova de Gaia, Vila Nova de Gaia, Portugal

(Duarte) General Direct. of Health, Lisboa, Portugal

(Guimaraes) Department of Biochemistry, Faculty of Medicine, University of Porto, Porto, Portugal

(Saraiva) S-Instituto de Investigacao e Inovacao em Saude, University of Porto, Portugal

(Saraiva) IBMC-Instituto de Biologia Molecular e Celular, University of Porto, Porto, Portugal

Publisher

Public Library of Science (E-mail: plos@plos.org)

Link to the Ovid Full Text or citation:

[Click here for full text options](http://ovidsp.ovid.com/ovidweb.cgi?T=JS&CSC=Y&NEWS=N&PAGE=fulltext&D=emed18b&AN=612857061)

Link to the External Link Resolver:

[SFX](http://whel-primo.hosted.exlibrisgroup.com/openurl/44WHELF_CAR/44WHELF_CAR_services_page?sid=OVID:embase&id=pmid:&id=doi:10.1371%2Fjournal.pone.0162797&issn=1932-6203&isbn=&volume=11&issue=9&spage=no+pagination&pages=no+pagination&date=2016&title=PLoS+ONE&atitle=A+prediction+rule+to+stratify+mortality+risk+of+patients+with+pulmonary+tuberculosis&aulast=Bastos&pid=<author>Bastos+H.N.%3BOsorio+N.S.%3BCastro+A.G.%3BRamos+A.%3BCarvalho+T.%3BMeira+L.%3BAraujo+D.%3BAlmeida+L.%3BBoaventura+R.%3BFragata+P.%3BChaves+C.%3BCosta+P.%3BPortela+M.%3BFerreira+I.%3BMagalhaes+S.P.%3BRodrigues+F.%3BSarmento-Castro+R.%3BDuarte+R.%3BGuimaraes+J.T.%3BSaraiva+M.<%2Fauthor><AN>612857061<%2FAN><DT>Journal%3A+Article<%2FDT>)

12.

Prevalence, risk factors, management, and treatment outcomes of first-line antituberculous drug-induced liver injury: a prospective cohort study.

Sun Q., Zhang Q., Gu J., Sun W.-W., Wang P., Bai C., Xiao H.-P., Sha W.

Pharmacoepidemiology and Drug Safety. 25 (8) (pp 908-917), 2016. Date of Publication: 01 Aug 2016.

AN: 611526560

Purpose: Antituberculosis drug-induced liver injury (ATDILI) is one of the most deleterious side effects associated with chemotherapy against tuberculosis (TB). In this study, our objective was to determine the incidence, risk factors, and management of ATDILI and analyze its impact on the treatment outcome in patients receiving standard anti-TB chemotherapy. Methods: A prospective cohort study of ATDILI prevalence was conducted in 938 enrolled patients of the 1426 TB cases in Shanghai from March 2011 to September 2012. Patients were followed up until February 2014. Univariate and multivariate logistic regression analyses were used to determine the risk factors of ATDILI. Successful therapeutic outcome, rates of drug resistance conversion, sputum smear/culture conversion, and lung cavity closure were analyzed. Results: Hepatitis B surface antigen/hepatitis B e antigen-positive hepatitis B carriers, complicated with systemic lupus erythematosus, albumin < 25 g/L, and chronic alcoholism were independent risk factors for ATDILI. Of the 121 cases with ATDILI (incidence rate of 12.9%), 84 (69.4%) used modified anti-TB therapy after recovery of liver function. Compared with the non-ATDILI group, patients with ATDILI exhibited remarkably decreased lung cavity closure rate (84.6% vs. 93.0%, P < 0.001) along with significantly reduced sputum smear/culture conversion rate (85.4% vs. 94.0%, P < 0.001). Conclusions: Our findings indicated that 12.9% patients developed ATDILI during standard anti-TB therapy, resulting in poor therapeutic outcome. Hepatitis B carriers with systemic lupus erythematosus, albumin < 25 g/L, and chronic alcoholism manifested increased risks for ATDILI. Copyright © 2016 John Wiley & Sons, Ltd. Copyright © 2016 John Wiley & Sons, Ltd.

PMID

26935778 [<http://www.ncbi.nlm.nih.gov/pubmed/?term=26935778>]

Institution

(Sun, Zhang, Gu, Sun, Wang, Xiao, Sha) Clinic and Research Center of Tuberculosis, Shanghai Key Laboratory of Tuberculosis, Shanghai Pulmonary Hospital, Tongji University School of Medicine, Shanghai, China (Sun, Bai) Department of Respiratory and Critical Care Medicine, Changhai Hospital, the Second Military Medical University, Shanghai, China

Publisher

John Wiley and Sons Ltd (Southern Gate, Chichester, West Sussex PO19 8SQ, United Kingdom)

Link to the Ovid Full Text or citation:

[Click here for full text options](http://ovidsp.ovid.com/ovidweb.cgi?T=JS&CSC=Y&NEWS=N&PAGE=fulltext&D=emed18b&AN=611526560)

Link to the External Link Resolver:

[SFX](http://whel-primo.hosted.exlibrisgroup.com/openurl/44WHELF_CAR/44WHELF_CAR_services_page?sid=OVID:embase&id=pmid:26935778&id=doi:10.1002%2Fpds.3988&issn=1053-8569&isbn=&volume=25&issue=8&spage=908&pages=908-917&date=2016&title=Pharmacoepidemiology+and+Drug+Safety&atitle=Prevalence%2C+risk+factors%2C+management%2C+and+treatment+outcomes+of+first-line+antituberculous+drug-induced+liver+injury%3A+a+prospective+cohort+study&aulast=Sun&pid=<author>Sun+Q.%3BZhang+Q.%3BGu+J.%3BSun+W.-W.%3BWang+P.%3BBai+C.%3BXiao+H.-P.%3BSha+W.<%2Fauthor><AN>611526560<%2FAN><DT>Journal%3A+Article<%2FDT>)

13.

The influence of smoking on pulmonary tuberculosis in diabetic and non-diabetic patients.

Bai K.-J., Lee J.-J., Chien S.-T., Suk C.-W., Chiang C.-Y.

PLoS ONE. 11 (6) (no pagination), 2016. Article Number: e0156677. Date of Publication: 01 Jun 2016.

AN: 610786648

Background: Both smoking and diabetes can increase the risk and influence the manifestations and outcomes of tuberculosis (TB). It is not clear whether the influence of smoking on pulmonary TB differs between non-diabetic and diabetic patients. Herein, we assessed the manifestations and outcomes of TB in relation to smoking in both diabetic and non-diabetic TB patients. Methodology/Principal Findings: All diabetic culture-positive pulmonary TB patients notified from 2005-2010 at three teaching hospitals in Taiwan were enrolled. A culture-positive pulmonary TB patient without DM who was notified to the health authority immediately prior to each diabetic TB patient was selected for comparison. The 972 patients in this study cohort included 365 (37.6%) nondiabetic non-smokers, 149 (15.3%) non-diabetic smokers, 284 (29.2%) diabetic non-smokers, and 174 (17.9%) diabetic smokers. The adjusted relative risk of a pretreatment positive smear for a smoker compared with a non-smoker was 2.19 (95% CI 1.38-3.47) in non-diabetic patients and 2.23 (95% CI 1.29-3.87) in diabetic culture-positive pulmonary TB patients. The adjusted relative risk for a positive smear among diabetic smokers was 5.61 (95% CI 3.35-9.41) compared with non-diabetic non-smokers. Smoking was significantly associated with an increased frequency of bilateral lung parenchyma involvement (AdjOR 1.84, 95% CI 1.16-2.93), far-advanced pulmonary TB (AdjOR 1.91, 95% CI 1.04-3.50), cavitary lesions (AdjOR 2.03, 95% CI 1.29-3.20), and unfavorable outcomes of TB (AdjOR 2.35, 95% CI 1.02-5.41) in non-diabetic patients. However, smoking was not associated with cavitary lung parenchyma lesions regarding the location, number or size of the cavity in diabetic TB patients. Conclusions/Significance: Smoking and diabetes have joint effects on a pretreatment positive smear. Diabetic smokers had more than a 5-fold increased risk of a pretreatment positive smear than did non-diabetic non-smokers, indicating remarkable joint effects of diabetes and smoking on the risk of TB transmission. Copyright © 2016 Bai et al. This is an open access article distributed under the terms of the Creative Commons Attribution License, which permits unrestricted use, distribution, and reproduction in any medium, provided the original author and source are credited.

Institution

(Bai, Suk, Chiang) Division of Pulmonary Medicine, Department of Internal Medicine, Wan Fang Hospital, Taipei Medical University, Taipei, Taiwan (Republic of China) (Bai) School of Respiratory Therapy, College of Medicine, Taipei Medical University, Taipei, Taiwan (Republic of China)

(Lee) Department of Internal Medicine, Buddhist Tzu Chi General Hospital, Tzu Chi University, Hualien, Taiwan (Republic of China)

(Chien) Chest Hospital, Department of Health and Welfare, Tainan, Taiwan (Republic of China)

(Chiang) Department of Internal Medicine, School of Medicine, College of Medicine, Taipei Medical University, Taipei, Taiwan (Republic of China)

(Chiang) International Union Against Tuberculosis and Lung Disease, Paris, France

Publisher

Public Library of Science (E-mail: plos@plos.org)

Link to the Ovid Full Text or citation:

[Click here for full text options](http://ovidsp.ovid.com/ovidweb.cgi?T=JS&CSC=Y&NEWS=N&PAGE=fulltext&D=emed18a&AN=610786648)

Link to the External Link Resolver:

[SFX](http://whel-primo.hosted.exlibrisgroup.com/openurl/44WHELF_CAR/44WHELF_CAR_services_page?sid=OVID:embase&id=pmid:&id=doi:10.1371%2Fjournal.pone.0156677&issn=1932-6203&isbn=&volume=11&issue=6&spage=no+pagination&pages=no+pagination&date=2016&title=PLoS+ONE&atitle=The+influence+of+smoking+on+pulmonary+tuberculosis+in+diabetic+and+non-diabetic+patients&aulast=Bai&pid=<author>Bai+K.-J.%3BLee+J.-J.%3BChien+S.-T.%3BSuk+C.-W.%3BChiang+C.-Y.<%2Fauthor><AN>610786648<%2FAN><DT>Journal%3A+Article<%2FDT>)

14.

Global urine metabolomics in patients treated with first-line tuberculosis drugs and identification of a novel metabolite of ethambutol.

Das M.K., Arya R., Debnath S., Debnath R., Lodh A., Bishwal S.C., Das A., Nanda R.K.

Antimicrobial Agents and Chemotherapy. 60 (4) (pp 2257-2264), 2016. Date of Publication: April 2016.

AN: 609689782

Population level variation of drug metabolism phenotype (DMP) has great implications in treatment outcome, drug-related side effects, and resistance development. In this study, we used a gas chromatography-time of flight-mass spectrometry (GC-TOF-MS)-based untargeted urine metabolomics approach to understand the DMP of a tuberculosis (TB) patient cohort (n = 20) from Tripura, a state in the northeastern part of India. Urine samples collected at different postdose time points (2 h, 6 h, 12 h, 24 h, 36 h, and 48 h) from these newly diagnosed TB patients receiving first-line anti-TB drugs were analyzed, and we have successfully detected three of the four first-line drugs, viz., isoniazid (INH), ethambutol (ETB), andpyrazinamide (PZA). The majority of their known metabolites, acetyl-isoniazid (AcINH), isonicotinic acid (INA), isonicotinuric acid (INTA), 2,2'-(ethylenediimino)-dibutyric acid (EDBA), 5-hydroxypyrazinamide (5OH-PZA), pyrazinoic acid (POA), and 5-hydroxypyrazinoic acid (5OH-POA), were also detected. Analyzing the variation in abundances of drugs and their known metabolites and calculating the metabolic ratios in these samples, we offer comprehensive DMP information on this small patient cohort that represents Tripura, India. The majority (75%) of these patients are found to be slow acetylators of INH. The average metabolic ratios of POA/PZA and 5OH-POA/POA are 3.16 +/- 3.03 and 6.09 +/- 6.15, respectively. Employing correlation analysis of the metabolomics metadata and a manual prediction of drug catabolism, we have proposed 2-aminobutyric acid (AABA) as a novel metabolite of ETB. These observations indicate the usefulness of GC-MS-based metabolomics to characterize the DMP at a population level and also to identify novel drug metabolites. Copyright © 2016, American Society for Microbiology. All Rights Reserved.

Institution

(Das, Arya, Bishwal, Nanda) Immunology Group, International Centre for Genetic Engineering and Biotechnology (ICGEB), New Delhi, India (Debnath, Debnath, Lodh, Das) Department of Tuberculosis and Respiratory Disease, Agartala Government Medical College, Agartala, Tripura, India

(Bishwal) School of Life Sciences, Sambalpur University, Sambalpur, Odisha, India

Publisher

American Society for Microbiology (E-mail: Journals@asmusa.org)

Link to the Ovid Full Text or citation:

[Click here for full text options](http://ovidsp.ovid.com/ovidweb.cgi?T=JS&CSC=Y&NEWS=N&PAGE=fulltext&D=emed18a&AN=609689782)

Link to the External Link Resolver:

[SFX](http://whel-primo.hosted.exlibrisgroup.com/openurl/44WHELF_CAR/44WHELF_CAR_services_page?sid=OVID:embase&id=pmid:&id=doi:10.1128%2FAAC.02586-15&issn=0066-4804&isbn=&volume=60&issue=4&spage=2257&pages=2257-2264&date=2016&title=Antimicrobial+Agents+and+Chemotherapy&atitle=Global+urine+metabolomics+in+patients+treated+with+first-line+tuberculosis+drugs+and+identification+of+a+novel+metabolite+of+ethambutol&aulast=Das&pid=<author>Das+M.K.%3BArya+R.%3BDebnath+S.%3BDebnath+R.%3BLodh+A.%3BBishwal+S.C.%3BDas+A.%3BNanda+R.K.<%2Fauthor><AN>609689782<%2FAN><DT>Journal%3A+Article<%2FDT>)

15.

Diabetes mellitus is associated with increased mortality during tuberculosis treatment: A prospective cohort study among tuberculosis patients in South-Eastern Amahra Region, Ethiopia.

Workneh M.H., Bjune G.A., Yimer S.A.

Infectious Diseases of Poverty. 5 (1) (no pagination), 2016. Article Number: 22. Date of Publication: March 21, 2016.

AN: 609673076

Background: There is growing evidence suggesting that diabetes mellitus (DM) affects disease presentation and treatment outcome in tuberculosis (TB) patients. This study aimed at investigating the role of DM on clinical presentations and treatment outcomes among newly diagnosed TB patients. Methods: A prospective cohort study was conducted in South-Eastern Amhara Region, Ethiopia from September 2013 till March 2015. Study subjects were consecutively recruited from 44 randomly selected health facilities in the study area. Participants were categorized into two patient groups, namely, patients with TB and DM (TBDM) and TB patients without DM (TBNDM). Findings on clinical presentations and treatment outcomes were compared between the two patient groups. Cox proportional hazard regression analysis was applied to identify factors associated with death. Results: Out of 1314 TB patients enrolled in the study, 109 (8.3 %) had coexisting DM. TBDM comorbidity [adjusted hazard ratio (AHR) 3.96; 95 % confidence interval (C.I.) (1.76-8.89)], and TB coinfection with human immunodeficiency virus (HIV) [AHR 2.59; 95 % C.I. (1.21-5.59)] were associated with increased death. TBDM and TBNDM patients did not show significant difference in clinical symptoms at baseline and during anti-TB treatment period. However, at the 2nd month of treatment, TBDM patients were more symptomatic compared to patients in the TBNDM group. Conclusions: The study showed that DM is associated with increased death during TB treatment. DM has no association with clinical presentation of TB except at the end of the intensive phase treatment. Routine screening of TB patients for DM is recommended for early diagnosis and treatment of patients with TBDM comorbidity. Copyright © 2016 Workneh et al.

Institution

(Workneh, Bjune, Yimer) University of Oslo, Institute of Health and Society, Faculty of Medicine, Oslo, Norway (Workneh, Yimer) Amhara Regional State Health Bureau, Bahir-Dar, Ethiopia

(Yimer) Oslo University Hospital, Department of Microbiology, Oslo, Norway

(Yimer) Norwegian Institute of Public Health, Department of Bacteriology and Immunology, Oslo, Norway

Publisher

BioMed Central Ltd. (E-mail: info@biomedcentral.com)

Link to the Ovid Full Text or citation:

[Click here for full text options](http://ovidsp.ovid.com/ovidweb.cgi?T=JS&CSC=Y&NEWS=N&PAGE=fulltext&D=emed18a&AN=609673076)

Link to the External Link Resolver:

[SFX](http://whel-primo.hosted.exlibrisgroup.com/openurl/44WHELF_CAR/44WHELF_CAR_services_page?sid=OVID:embase&id=pmid:&id=doi:10.1186%2Fs40249-016-0115-z&issn=2049-9957&isbn=&volume=5&issue=1&spage=no+pagination&pages=no+pagination&date=2016&title=Infectious+Diseases+of+Poverty&atitle=Diabetes+mellitus+is+associated+with+increased+mortality+during+tuberculosis+treatment%3A+A+prospective+cohort+study+among+tuberculosis+patients+in+South-Eastern+Amahra+Region%2C+Ethiopia&aulast=Workneh&pid=<author>Workneh+M.H.%3BBjune+G.A.%3BYimer+S.A.<%2Fauthor><AN>609673076<%2FAN><DT>Journal%3A+Article<%2FDT>)

16.

Does enhanced diabetes management reduce the risk and improve the outcome of tuberculosis?.

Lo H.-Y., Yang S.-L., Lin H.-H., Bai K.-J., Lee J.-J., Lee T.-I., Chiang C.-Y.

International Journal of Tuberculosis and Lung Disease. 20 (3) (pp 376-382), 2016. Date of Publication: 01 Mar 2016.

AN: 608513164

SETTING: The Bureau of National Health Insurance (NHI) has implemented a pay-for-performance (p4p) programme for diabetes mellitus (DM) in Taiwan. OBJECTIVE : To investigate whether patients with DM enrolled in the p4p programme (DM-p4p) are less likely to develop tuberculosis (TB) and whether they have a better outcome than patients with DMnot enrolled in the p4p programme (DM-non-p4p) if they do develop TB. DESIGN: A random sample of 79 471 DM-p4p, 100 000 DM-non-p4p and 100 000 non-diabetic patients (non-DM) was obtained from the 2008-2009 NHI database, and the patients were matched with the National TB Registry to determine whether they had developed TB by the end of 2010. RESULTS : The average annual incidence of TB was respectively 259.9 (95%CI 230.2-293.4), 137.5 (95%CI 116.4-162.5) and 74.1 (95%CI 59.0-93.0) per 100 000 population among DM-non-p4p, DM-p4p and non-DM patients. The relative risk of death over treatment success was 1.79 (95%CI 1.05-3.04) among DM-non-p4p and 1.69 (95%CI 0.84-3.40) among non-DM patients, relative to DM-p4p patients. CONCLUSIONS : Enhanced case management of DM reduced risk and improved outcomes of TB among patients with DM. Copyright © 2016 The Union.

Institution

(Lo, Yang) Taiwan Centers for Disease Control, Ministry of Health and Welfare, Taipei, Taiwan (Republic of China) (Lin) Institute of Epidemiology and Preventive Medicine, National Taiwan University, Taipei, Taiwan (Republic of China)

(Bai) School of Respiratory Therapy, College of Medicine, Taipei Medical University, Taipei, Taiwan (Republic of China)

(Bai, Chiang) Division of Pulmonary Medicine, Department of Internal Medicine, Wan Fang Hospital, Taipei Medical University, 111 Hsin-Long Road, Taipei, Taiwan (Republic of China)

(Lee) Department of Internal Medicine, Tzu Chi General Hospital and Tzu Chi University, Hualien, Taiwan (Republic of China)

(Lee) Division of Endocrinology and Metabolism, Department of Internal Medicine, Wan Fang Hospital, Taipei Medical University, Taipei, Taiwan (Republic of China)

(Lee) Department of General Medicine, School of Medicine, College of Medicine, Taipei Medical University, Taipei, Taiwan (Republic of China)

(Chiang) Department of Internal Medicine, School of Medicine, College of Medicine, Taipei Medical University, Taipei, Taiwan (Republic of China)

(Chiang) International Union Against Tuberculosis and Lung Disease, Paris, France

Publisher

International Union against Tubercul. and Lung Dis.

Link to the Ovid Full Text or citation:

[Click here for full text options](http://ovidsp.ovid.com/ovidweb.cgi?T=JS&CSC=Y&NEWS=N&PAGE=fulltext&D=emed18a&AN=608513164)

Link to the External Link Resolver:

[SFX](http://whel-primo.hosted.exlibrisgroup.com/openurl/44WHELF_CAR/44WHELF_CAR_services_page?sid=OVID:embase&id=pmid:&id=doi:10.5588%2Fijtld.15.0654&issn=1027-3719&isbn=&volume=20&issue=3&spage=376&pages=376-382&date=2016&title=International+Journal+of+Tuberculosis+and+Lung+Disease&atitle=Does+enhanced+diabetes+management+reduce+the+risk+and+improve+the+outcome+of+tuberculosis%3F&aulast=Lo&pid=<author>Lo+H.-Y.%3BYang+S.-L.%3BLin+H.-H.%3BBai+K.-J.%3BLee+J.-J.%3BLee+T.-I.%3BChiang+C.-Y.<%2Fauthor><AN>608513164<%2FAN><DT>Journal%3A+Article<%2FDT>)

17.

Incidence of tuberculosis is high in chronic kidney disease patients in South East England and drug resistance common.

Ostermann M., Palchaudhuri P., Riding A., Begum P., Milburn H.J.

Renal Failure. 38 (2) (pp 256-261), 2016. Date of Publication: 07 Feb 2016.

AN: 607623274

The risk of tuberculosis (TB) is significantly increased in chronic kidney disease (CKD). Data on TB in CKD in the UK are sparse; most information stems from countries with high background prevalence. The aim of this study was to estimate the incidence of TB in CKD patients in South East London and to describe the epidemiology, treatment, and outcome. CKD patients with TB between 1994 and 2010 were identified retrospectively. Data were collected on type of renal replacement therapy, the method of TB diagnosis, disease site, treatment regimens, and risk factors. Forty patients were identified of whom 67.5% had CKD stages IV-V. Sixty-five percent were from non-UK born ethnic minorities. Median time from diagnosis of CKD to TB development was 12 months (range 0-192 months). Cumulative incidence of TB was 1267/100,000 [95% confidence interval (CI): 630-1904; 85 x background UK rate] in hemodialysis patients; 398/100,000 (95% CI: 80-1160; 26 x background UK rate) in peritoneal dialysis; and 522/100,000 (CI: 137-909; 35 x background UK rate) in transplant recipients. Sixty-three percent of patients had pulmonary TB and 25% of patients with culture-positive TB had resistant isolates. Fifty percent of patients were immunosuppressed due to drugs, diabetes, and/or retroviral disease. Treatment regimens were according to recent national guidance in 73% of cases. Seventy-six percent of patients experienced side effects. Greater awareness of risk factors, drug resistance, treatment regimens, and potential side effects is needed. Copyright © 2015 Taylor & Francis.

Institution

(Ostermann) Department of Renal Medicine, Guys and St Thomas NHS Foundation Trust, London, United Kingdom (Ostermann, Milburn) Guys and St Thomas NHS Foundation Trust, Kings College London, Great Maze Pond, London SE1 9RT, United Kingdom

(Palchaudhuri, Riding, Begum, Milburn) Department of Respiratory Medicine, Guys and St Thomas NHS Foundation Trust, London, United Kingdom

Publisher

Taylor and Francis Ltd (E-mail: healthcare.enquiries@informa.com)

Link to the Ovid Full Text or citation:

[Click here for full text options](http://ovidsp.ovid.com/ovidweb.cgi?T=JS&CSC=Y&NEWS=N&PAGE=fulltext&D=emed18a&AN=607623274)

Link to the External Link Resolver:

[SFX](http://whel-primo.hosted.exlibrisgroup.com/openurl/44WHELF_CAR/44WHELF_CAR_services_page?sid=OVID:embase&id=pmid:&id=doi:10.3109%2F0886022X.2015.1128290&issn=0886-022X&isbn=&volume=38&issue=2&spage=256&pages=256-261&date=2016&title=Renal+Failure&atitle=Incidence+of+tuberculosis+is+high+in+chronic+kidney+disease+patients+in+South+East+England+and+drug+resistance+common&aulast=Ostermann&pid=<author>Ostermann+M.%3BPalchaudhuri+P.%3BRiding+A.%3BBegum+P.%3BMilburn+H.J.<%2Fauthor><AN>607623274<%2FAN><DT>Journal%3A+Article<%2FDT>)

18.

Diabetes is associated with worse clinical presentation in tuberculosis patients from Brazil: A retrospective cohort study.

Gil-Santana L., Almeida J.L., Oliveira C.A.M., Hickson L.S., Daltro C., Castro S., Kornfeld H., Netto E.M., Andrade B.B.

PLoS ONE. 11 (1) (no pagination), 2016. Article Number: e0146876. Date of Publication: 11 Jan 2016.

AN: 607727123

Background. The rising prevalence of diabetes mellitus (DM) worldwide, especially in developing countries, and the persistence of tuberculosis (TB) as a major public health issue in these same regions, emphasize the importance of investigating this association. Here, we compared the clinical profile and disease outcomes of TB patients with or without coincident DM in a TB reference center in Brazil. Methods. We performed a retrospective analysis of a TB patient cohort (treatment naive) of 408 individuals recruited at a TB primary care center in Brazil between 2004 and 2010. Data on diagnosis of TB and DM were used to define the groups. The study groups were compared with regard to TB disease presentation at diagnosis as well as to clinical outcomes such as cure and mortality rates upon anti-tuberculosis therapy (ATT) initiation. A composite score utilizing clinical, radiological and microbiological parameters was used to compare TB severity between the groups. Results. DM patients were older than non-diabetic TB patients. In addition, diabetic individuals more frequently presented with cough, night sweats, hemoptysis and malaise than those without DM. The overall pattern of lung lesions assessed by chest radiographic examination was similar between the groups. Compared to non-diabetic patients, those with TB-diabetes exhibited positive acid-fast bacilli in sputum samples more frequently at diagnosis and at 30 days after ATT initiation. Notably, higher values of the TB severity score were significantly associated with TB-diabetes comorbidity after adjustment for confounding factors. Moreover, during ATT, diabetic patients required more frequent transfers to TB reference hospitals for complex clinical management. Nevertheless, overall mortality and cure rates were indistinguishable between the study groups. Conclusions. These findings reinforce the idea that diabetes negatively impacts pulmonary TB severity. Our study argues for the systematic screening for DM in TB reference centers in endemic areas. Copyright © 2016 Gil-Santana et al. This is an open access article distributed under the terms of the Creative Commons Attribution License, which permits unrestricted use, distribution, and reproduction in any medium, provided the original author and source are credited.

Institution

(Gil-Santana, Almeida, Andrade) Unidade de Medicina Investigativa, Laboratorio Integrado de Microbiologia e Imunorregulacao, Centro de Pesquisas Goncalo Moniz, Fundacao Oswaldo Cruz, Salvador, Bahia, Brazil (Gil-Santana, Almeida, Andrade) School of Medicine, Faculdades de Tecnologia e Ciencias, Salvador, Bahia, Brazil

(Gil-Santana, Almeida, Andrade) Multinational Organization Network Sponsoring Translational and Epidemiological Research (MONSTER), Fundacao Jose Silveira, Salvador, Brazil

(Oliveira, Hickson, Castro, Netto, Andrade) Research Center, Instituto Brasileiro para Investigacao da Tuberculose (IBIT), Fundacao Jose Silveira, Salvador, Bahia, Brazil

(Daltro, Netto) Hospital Universitario Pro. Edgard Santos, Universidade Federal da Bahia, Salvador, Bahia, Brazil

(Kornfeld) Department of Medicine, University of Massachusetts Medical School, Worcester, MA, United States

Publisher

Public Library of Science (E-mail: plos@plos.org)

Link to the Ovid Full Text or citation:

[Click here for full text options](http://ovidsp.ovid.com/ovidweb.cgi?T=JS&CSC=Y&NEWS=N&PAGE=fulltext&D=emed18a&AN=607727123)

Link to the External Link Resolver:

[SFX](http://whel-primo.hosted.exlibrisgroup.com/openurl/44WHELF_CAR/44WHELF_CAR_services_page?sid=OVID:embase&id=pmid:&id=doi:10.1371%2Fjournal.pone.0146876&issn=1932-6203&isbn=&volume=11&issue=1&spage=no+pagination&pages=no+pagination&date=2016&title=PLoS+ONE&atitle=Diabetes+is+associated+with+worse+clinical+presentation+in+tuberculosis+patients+from+Brazil%3A+A+retrospective+cohort+study&aulast=Gil-Santana&pid=<author>Gil-Santana+L.%3BAlmeida+J.L.%3BOliveira+C.A.M.%3BHickson+L.S.%3BDaltro+C.%3BCastro+S.%3BKornfeld+H.%3BNetto+E.M.%3BAndrade+B.B.<%2Fauthor><AN>607727123<%2FAN><DT>Journal%3A+Article<%2FDT>)

19.

Survival benefit associated with early cyclosporine treatment for dermatomyositis-associated interstitial lung disease.

Go D.J., Park J.K., Kang E.H., Kwon H.M., Lee Y.J., Song Y.W., Lee E.B.

Rheumatology International. 36 (1) (pp 125-131), 2016. Date of Publication: 01 Jan 2016.

AN: 605475146

Interstitial lung disease (ILD) is the most common cause of death in dermatomyositis (DM). Cyclosporine A (CsA) has shown to be effective in DM-associated ILD (DM-ILD). This study aimed to define the optimal time of CsA administration. A total of 47 patients with DM-ILD, who were treated with CsA at Seoul National University Hospital between January 1998 and June 2013, were enrolled. ILD was diagnosed based on typical chest high-resolution computed tomography (HRCT) findings. Patients with early and delayed CsA treatment were compared in regard to the mortality and ILD progression on HRCT. The early (n = 16) and the delayed treatment group (n = 31) did not differ in regard to baseline clinical characteristics including HRCT scores and pulmonary function. Patients with clinically amyopathic DM (CADM) were more common in the early treatment group. The mortality rate was significantly lower in the early treatment group than in the delayed treatment group (p = 0.009). The survival benefit of early CsA treatment remained significant even after adjusting for age, degree of dyspnea, CADM status, and the year of CsA treatment (hazard ratio 0.057, 95 % confidence interval 0.007-0.472). CsA stabilized disease progression on HRCT in the early treatment group (p = 0.738). Delay in CsA treatment is associated with a worse survival in patients with DM-ILD. Early CsA treatment should be considered at DM-ILD diagnosis especially in patients at a higher risk of developing a rapidly progressive ILD. Copyright © 2015, Springer-Verlag Berlin Heidelberg.

Institution

(Go, Park, Kwon, Song, Lee) Division of Rheumatology, Department of Internal Medicine, Seoul National University College of Medicine, Seoul, South Korea (Go, Park, Song) Department of Molecular Medicine and Biopharmaceutical Sciences, Graduate School of Convergence Science and Technology, Medical Research Institute, Seoul National University, Seoul, South Korea

(Kang, Lee) Division of Rheumatology, Department of Internal Medicine, Seoul National University Bundang Hospital, Seongnam, South Korea

Publisher

Springer Verlag (E-mail: service@springer.de)

Link to the Ovid Full Text or citation:

[Click here for full text options](http://ovidsp.ovid.com/ovidweb.cgi?T=JS&CSC=Y&NEWS=N&PAGE=fulltext&D=emed18a&AN=605475146)

Link to the External Link Resolver:

[SFX](http://whel-primo.hosted.exlibrisgroup.com/openurl/44WHELF_CAR/44WHELF_CAR_services_page?sid=OVID:embase&id=pmid:&id=doi:10.1007%2Fs00296-015-3328-8&issn=0172-8172&isbn=&volume=36&issue=1&spage=125&pages=125-131&date=2016&title=Rheumatology+International&atitle=Survival+benefit+associated+with+early+cyclosporine+treatment+for+dermatomyositis-associated+interstitial+lung+disease&aulast=Go&pid=<author>Go+D.J.%3BPark+J.K.%3BKang+E.H.%3BKwon+H.M.%3BLee+Y.J.%3BSong+Y.W.%3BLee+E.B.<%2Fauthor><AN>605475146<%2FAN><DT>Journal%3A+Article<%2FDT>)
